# The burden of neurological conditions in north Africa and the Middle East, 1990–2019: a systematic analysis of the Global Burden of Disease Study 2019

**DOI:** 10.1016/S2214-109X(24)00093-7

**Published:** 2024-04-08

**Authors:** GBD 2019 North Africa and the Middle East Neurology Collaborators, Abolfazl Avan, Valery L Feigin, Derrick A. Bennett, Jaimie D Steinmetz, Vladimir Hachinski, Saverio Stranges, Mayowa O Owolabi, Amirali Aali, Mohsen Abbasi-Kangevari, Zeinab Abbasi-Kangevari, Foad Abd-Allah, Sina Abdollahzade, Hassan Abidi, Hassan Abolhassani, Ahmed Abualhasan, Eman Abu-Gharbieh, Niveen ME Abu-Rmeileh, Ahmed Abu-Zaid, Aqeel Ahmad, Sepideh Ahmadi, Luai A Ahmed, Marjan Ajami, Hanadi Al Hamad, Fahad Mashhour Alanezi, Turki M Alanzi, Yousef Alimohamadi, Syed Mohamed Aljunid, Rajaa M Al-Raddadi, Sohrab Amiri, Jalal Arabloo, Judie Arulappan, Ashokan Arumugam, Ali A Asadi-Pooya, Mohammad Athar, Seyyed Shamsadin Athari, Maha Moh'd Wahbi Atout, Sina Azadnajafabad, Mohammadreza Azangou-Khyavy, Amirhossein Azari Jafari, Ahmed Y Azzam, Nayereh Baghcheghi, Sara Bagherieh, Ovidiu Constantin Baltatu, Gholamreza Bazmandegan, Vijayalakshmi S Bhojaraja, Ali Bijani, Saeid Bitaraf, Daniela Calina, Amira Hamed Darwish, Shirin Djalalinia, Mohamed Fahmy Doheim, Fariba Dorostkar, Ebrahim Eini, Nevine El Nahas, Iman El Sayed, Muhammed Elhadi, Mohamed A Elmonem, Sharareh Eskandarieh, Shahriar Faghani, Aida Fallahzadeh, Mohammad Farahmand, Mansour Ghafourifard, Seyyed-Hadi Ghamari, Ali Gholami, Sherief Ghozy, Pouya Goleij, Mostafa Hadei, Nima Hafezi-Nejad, Arvin Haj-Mirzaian, Rabih Halwani, Samer Hamidi, Ahmed I Hasaballah, Amr Hassan, Khedidja Hedna, Mohamed I Hegazy, Reza Heidari-Soureshjani, Mohammad-Salar Hosseini, Soodabeh Hoveidamanesh, Haitham Jahrami, Elham Jamshidi, Tahereh Javaheri, Sathish Kumar Jayapal, Laleh R Kalankesh, Rohollah Kalhor, Zahra Kamiab, Mohammad Keykhaei, Yousef Saleh Khader, Maseer Khan, Moien AB Khan, Khatatbeh, Hamid Reza Khayat Kashani, Ahmad Khosravi, Farzad Kompani, Hamid Reza Koohestani, Bagher Larijani, Savita Lasrado, Mohammed Magdy Abd El Razek, Mohammad-Reza Malekpour, Ahmad Azam Malik, Mohammad Ali Mansournia, Parham Mardi, Seyed Farzad Maroufi, Sahar Masoudi, Mahsa Mayeli, Entezar Mehrabi Nasab, Ritesh G Menezes, Seyyedmohammadsadeq Mirmoeeni, Mohammad Mirza-Aghazadeh-Attari, Maryam Mobarakabadi, Esmaeil Mohammadi, Soheil Mohammadi, Syam Mohan, Ali H Mokdad, Sara Momtazmanesh, Fateme Montazeri, Mostafa Moradi Sarabi, Paula Moraga, Negar Morovatdar, Majid Motaghinejad, Mohsen Naghavi, Zuhair S Natto, Seyed Aria Nejadghaderi, Nafise Noroozi, Hassan Okati-Aliabad, Hamidreza Pazoki Toroudi, Simone Perna, Michael A Piradov, Mohammadreza Pourahmadi, Alireza Rafiei, Vafa Rahimi-Movaghar, Amir Masoud Rahmani, Shayan Rahmani, Vahid Rahmanian, Ali Rajabpour-Sanati, Chythra R Rao, Mohammad-Mahdi Rashidi, Reza Rawassizadeh, Iman Razeghian-Jahromi, Elrashdy Moustafa Mohamed Redwan, Malihe Rezaee, Nazila Rezaei, Negar Rezaei, Nima Rezaei, Mohsen Rezaeian, Reza Rikhtegar, Aly M A Saad, Basema Saddik, Masoumeh Sadeghi, Saeid Sadeghian, Sahar Saeedi Moghaddam, Amirhossein Sahebkar, Saina Salahi, Sarvenaz Salahi, Abdallah M Samy, Nima Sanadgol, Arash Sarveazad, Brijesh Sathian, Mete Saylan, Ataollah Shahbandi, Shayan Shahrokhi, Mehran Shams-Beyranvand, Mohd Shanawaz, Javad Sharifi-Rad, Rahim Ali Sheikhi, Jeevan K Shetty, Parnian Shobeiri, Seyed Afshin Shorofi, Soraya Siabani, Seyyed Mohammad Tabatabaei, Yasaman Taheri Abkenar, Moslem Taheri Soodejani, Mohamad-Hani Temsah, Alireza Vakilian, Sahel Valadan Tahbaz, Rohollah Valizadeh, Siavash Vaziri, Bay Vo, Seyed Hossein Yahyazadeh Jabbari, Metin Yesiltepe, Nazar Zaki, Iman Zare, Ali Zare Dehnavi, Mohammad Zoladl

## Abstract

**Background:**

The burden of neurological conditions in north Africa and the Middle East is increasing. We aimed to assess the changes in the burden of neurological conditions in this super-region to aid with future decision making.

**Methods:**

In this analysis of the Global Burden of Diseases, Injuries, and Risk Factors Study 2019 data, we examined temporal trends of disability-adjusted life-years (DALYs; deaths and disabilities combined), deaths, incident cases, and prevalent cases of 14 major neurological conditions and eight subtypes in 21 countries in the north Africa and the Middle East super-region. Additionally, we assessed neurological DALYs due to 22 potentially modifiable risk factors, within four levels of classification, during the period 1990–2019. We used a Bayesian modelling estimation approach, and generated 95% uncertainty intervals (UIs) for final estimates on the basis of the 2·5th and 97·5th percentiles of 1000 draws from the posterior distribution.

**Findings:**

In 2019, there were 441·1 thousand (95% UI 347·2–598·4) deaths and 17·6 million (12·5–24·7) neurological DALYs in north Africa and the Middle East. The leading causes of neurological DALYs were stroke, migraine, and Alzheimer's disease and other dementias (hereafter dementias). In north Africa and the Middle East in 2019, 85·8% (82·6–89·1) of stroke and 39·9% (26·4–54·7) of dementia age-standardised DALYs were attributable to modifiable risk factors. North Africa and the Middle East had the highest age-standardised DALY rates per 100 000 population due to dementia (387·0 [172·0–848·5]), Parkinson's disease (84·4 [74·7–103·2]), and migraine (601·4 [107·0–1371·8]) among the global super-regions. Between 1990 and 2019, there was a decrease in the age-standardised DALY rates related to meningitis (–75·8% [–81·1 to –69·5]), tetanus (–88·2% [–93·9 to –76·1]), stroke (–32·0% [–39·1 to –23·3]), intracerebral haemorrhage (–51·7% [–58·2 to –43·8]), idiopathic epilepsy (–26·2% [–43·6 to –1·1]), and subarachnoid haemorrhage (–62·8% [–71·6 to –41·0]), but for all other neurological conditions there was no change. During 1990–2019, the number of DALYs due to dementias, Parkinson's disease, multiple sclerosis, ischaemic stroke, and headache disorder (ie, migraine and tension-type headache) more than doubled in the super-region, and the burden of years lived with disability (YLDs), incidence, and prevalence of multiple sclerosis, motor neuron disease, Parkinson's disease, and ischaemic stroke increased both in age-standardised rate and count. During this period, the absolute burden of YLDs due to head and spinal injuries almost doubled.

**Interpretation:**

The increasing burden of neurological conditions in north Africa and the Middle East accompanies the increasing ageing population. Stroke and dementia are the primary causes of neurological disability and death, primarily attributable to common modifiable risk factors. Synergistic, systematic, lifetime, and multi-sectoral interventions aimed at preventing or mitigating the burden are needed.

**Funding:**

Bill & Melinda Gates Foundation.

**Translations:**

For the Persian, Arabic and Turkish translations of the abstract see Supplementary Materials section.

## Introduction

Increased life expectancy is associated with an increased risk of neurological conditions,^1^, ^2^ despite improvements in quality of life, overall health, and improved public awareness.^3^ Due to rapid sociodemographic and epidemiological changes and inequalities in low-income and middle-income countries (LMICs),^4^, ^5^, ^6^ most of the global neurological burden is in these nations and is expected to persist.^6^, ^7^, ^8^

The Global Burden of Diseases, Injuries, and Risk Factors Study (GBD) defined super-region of north Africa and the Middle East, which slightly differs from the World Bank classification, encompasses 15 LMICs and six high-income countries (HICs). Collectively, north Africa and the Middle East comprises 7·9% (609 million) of the global population, with an estimated mean life expectancy of 73·8 years, and an estimated 3·1 million deaths occurring in 2019.^9^ Countries in north Africa and the Middle East are mostly Muslim societies with cultural similarities and comparable lifestyle habits, but with widespread disparities in resources, and many involved in regional and international conflicts and wars during the past three decades.

**Research in context**

**Evidence before this study**

The Global Burden of Diseases, Injuries, and Risk Factors Study (GBD) 2019 has provided, to our knowledge, the most comprehensive systematic and analytical evidence, based on Bayesian estimation, of the burden of neurological conditions globally using published and unpublished data to estimate measures in areas with a paucity of data of sufficient quality on the basis of geographical, geopolitical, and socioeconomic statuses. To evaluate the availability of evidence, we did a review of the published scientific literature in PubMed for relevant reports published in any language up to Jan 1, 2024, with no start date restriction, using search terms that included “encephalitis”, “meningitis”, “tetanus”, “brain (central nervous system) cancer”, “head injury”, “spinal injury”, “stroke”, “subarachnoid h(a)emorrhage”, “Alzheimer(‘s)”, “dementia(s)”, “Parkinson(‘s)”, “epilepsy”, “multiple sclerosis”, “headache”, “migraine”, “motor neuron disease”, “Amyloid lateral sclerosis”, “neurological disorder”, OR “neurological disease”, AND “Middle East”, “North Africa”, “Afghanistan”, “Algeria”, “Bahrain”, “Egypt”, “Iran”, “Iraq”, “Jordan”, “Kuwait”, “Lebanon”, “Libya”, “Morocco”, “Palestine”, “Oman”, “Qatar”, “Saudi Arabia”, “Sudan”, “Syria”, “Tunisia”, “Turkey”, “Türkiye”, “Emirates”, OR “Yemen”, AND “population-based”, “community-based”, “community-dwelling(s)”, OR “population-wide”, AND “burden”, “disability”, “mortality”, “death”, “incidence”, “prevalence”, “daly(s)”, “yll(s)”, “yld(s)”, “population attributable fraction”, OR “risk factor(s)”. We identified 74 publications reporting on the epidemiological features of neurological conditions in countries in north Africa and the Middle East. GBD 2015 estimated stroke, Alzheimer's disease and other dementias, migraine, epilepsy, and meningitis as the leading causes of neurological disabilities and deaths combined in north Africa and the Middle East. Moreover, this super-region had the highest burden of Alzheimer's disease and other dementias, and Afghanistan had the highest burdens of neurological conditions worldwide. One paper published in 2023 (based on GBD 2019 estimates) reported that the incidence and prevalence rates of seven neurological disorders (mostly neurodegenerative and headache disorders) have increased, while their mortality and disability-adjusted life-year (DALY) rates decreased in north Africa and the Middle East. However, that study comprised less than 41% of the neurological burden in north Africa and the Middle East. That study did not include estimates for the burden of stroke, meningitis, encephalitis, tetanus, and brain and central nervous system cancer at the national level. Previous evidence has shown an increasing number of people with neurological conditions over the past few decades, despite an overall reduction in deaths due to neurological conditions, such that neurological conditions are the leading cause of disabilities and deaths combined globally.

**Added value of this study**

This study is based on analysis of data from almost a thousand published and unpublished data sources (such as vital registries) reporting on the status of 14 major neurological conditions and eight subtypes and their changes from 1990 to 2019 in 21 countries in north Africa and the Middle East by age, sex, and risk factors. The findings highlight priority areas in these countries regarding the neurological burden. We also report the proportion of the burden of each neurological condition attributable to 22 potentially modifiable risk factors, within four levels of classification, and the changes in this burden from 1990 to 2019. Furthermore, we compared the incidence, prevalence, mortality, and burden of neurological conditions in north Africa and the Middle East with global estimates, the six other GBD super-regions, and four country income levels classified by the World Bank. We found that the north Africa and the Middle East super-region had the highest rates of age-standardised DALYs related to Alzheimer's disease and other dementias, Parkinson's disease, and migraine, and the second highest rate for ischaemic stroke and brain and CNS cancer compared with all other super-regions. Additionally, age-standardised metabolic risk-attributed DALYs increased for dementia by 50·2% (95% uncertainty interval 35·7–76·7) in north Africa and the Middle East from 1990 to 2019, offering potential for prevention. Data from GBD 2019 showed a 39·0% higher potential preventable proportion of Alzheimer's disease and other dementias in this region than global estimates (154·7 *vs* 111·3 per 100 000 people).

**Implications of all the available evidence**

Our findings, drawn from extensive data resources and a rigorous methodology, offer comprehensive and systematic evidence for health-care planning, resource allocation, prioritisation, research strategies, and interventions aimed at treating and rehabilitating individuals with neurological conditions at both regional and national levels. Analysing the risk-attributable proportion of neurological conditions can guide targeted preventive measures in diverse regions and countries, allowing us to understand the contribution of each modifiable risk factor to the overall burden.

Between 1990 and 2016, north Africa and the Middle East had a 38% increase in the burden of neurological conditions, increasing from an estimated 12·5 million (95% uncertainty interval [UI] 10·8–14·4) to 17·3 million (14·8–20·1) disability-adjusted life-years (DALYs).^10^ This increase was twice the global estimated increase for the same period (15%). Additionally, north Africa and the Middle East had a smaller decrease in age-standardised neurological DALY rates (–20%) than the global estimate (–27%).^10^ Furthermore, an analysis of GBD 2016 found that cases of stroke—a top contributor to neurological burden—significantly increased in north Africa and the Middle East between 1990 and 2016.^11^ Given previous evidence up to 2016 that the neurological burden in north Africa and the Middle East exceeded global estimates, and that stroke, a major driver of neurological health loss, has increased in prevalence, up-to-date estimates of temporal trends in north Africa and the Middle East are essential to highlight the conditions that contribute the most to the burden. We conducted a systematic analysis of temporal trends of the burdens of neurological conditions in countries in the north Africa and the Middle East super-region compared with other super-regions and globally from 1990 to 2019.

## Methods

### Data sources

We used data from various sources, including census and population registries or surveys, vital statistics, demographic surveillance data, verbal autopsies, hospital and Ministry of Health and WHO data, health insurance data, morbidity notification data, and published and grey literature (eg, dissertations and hospital reports) from online databases (eg, PubMed and Scopus), books, and other resources. Data sources are available on the GBD 2019 Data Resources webpage.^12^

### Data analysis

We selected 14 major neurological conditions and eight subtypes among the GBD causes of mortality and morbidity for analysis among the 21 countries of the north Africa and the Middle East super-region. Selected causes were encephalitis, meningitis, tetanus, brain and central nervous system (CNS) cancer, head injuries, spinal injuries, stroke (including ischaemic stroke, intracerebral haemorrhage, and subarachnoid haemorrhage), Alzheimer's disease and other dementias (hereafter referred to as dementias), Parkinson's disease, idiopathic epilepsy, multiple sclerosis, headache disorders (including migraine and tension-type headache), motor neuron disease, and other neurological disorders. For head injury and spinal injury, we also considered the subcategories of minor traumatic brain injury (TBI) and major or severe TBI for head injury, and spinal cord lesion at neck level and spinal cord lesion below neck level for spinal injury. We accounted for potential overlap in health states; for example, for dementia, we assessed dementia relative risk due to other known conditions (ie, stroke, Parkinson's disease, traumatic brain injury, and Down syndrome) and subtracted these cases from total dementia.

We calculated estimates of years lived with disability (YLDs; 1 full year of healthy life lost due to disability or ill health), years of life lost (YLLs; a measure of premature mortality), DALYs (the sum of YLDs and YLLs; a measure of overall disease burden), mortality (number of deaths per year), incidence (number of new cases per year), and prevalence (number of existing cases by the end of a year). YLD=prevalence × disability weights, and disability weights range from 0 to 1, with 0 indicating no health loss and 1 indicating death. YLL=life expectancy of someone at a given age – actual age of death, with actual age at death standardised across all populations, and not based on different life expectancies in different nations. Headache disorders (including migraine and tension-type headache), head injuries, and spinal injuries do not have a fatal component per GBD classification, and so YLLs were not calculated. Hence, any presentation of DALYs for these conditions are equivalent to YLDs.

We summarised age-specific, sex-specific, risk-specific, and age-standardised estimates for neurological DALYs attributable to risk factors. We selected 22 potentially modifiable risk factors at the most granular level (high systolic blood pressure, high LDL cholesterol, high fasting plasma glucose, high BMI, and kidney dysfunction, which are all metabolic risk factors; low birthweight, short gestation, smoke, secondhand smoke, diet high in red meat, diet high in sodium, diet low in fibre, diet low in fruits, diet low in vegetables, diet low in whole grain, low physical activity, and alcohol use, which are all behavioural risk factors; and ambient particular matter pollution, household air pollution from solid fuels, low temperature, high temperature, and lead exposure, which are all environmental or occupational risk factors), categorised into four levels (appendix 4 pp 16–18). We present data for these 22 risk factors and as aggregates of the risk factors in the Levels above them in the risk factor hierarchy. We presented data on risk factors in the format of population-attributable fraction (PAF) of a neurological condition due to a risk factor (appendix 4 pp 26–35). We presented data in all-age absolute numbers, age-standardised rates per 100 000 population, proportions, sex ratios (female to male), and their changes between 1990 and 2019.

### Statistical analysis

Modelling methods varied by condition. We used a Bayesian approach to estimate the model parameters and to generate 95% UIs for final estimates as the 2·5th and 97·5th percentiles of 1000 posterior draws and cross-validated the model performances for out-of-sample predictive validity. We included data for a given disease that met our gold standard (reference) case definition (appendix 4 pp 15–16) and alternative case definitions (eg, for epilepsy, our gold standard is active epilepsy, but we considered studies of lifetime recall). We used meta-regression to adjust non-reference case definition data to the reference, enabling us to include data that were collected using heterogeneous methods. We used a tool called MR-BRT (meta-regression—Bayesian, Regularized, Trimmed) to determine adjustments for each alternative case definition.^13^ This tool combines functionality for linear regressions, mixed-effects models, meta-analyses, Bayesian priors, and flexible model fits with splines. It also allows for outlier trimming using a likelihood estimator and for automated covariate selection. We used log ratio, logit difference network, or intercept-only meta-analyses to systematically adjust alternative case definitions to reference for the different conditions included in our analysis. Input data to the model consisted of the logit difference between matched pairs of data with different case definitions from similar geographies and collection periods for a given measure (eg, prevalence).

To estimate incidence, prevalence, and deaths, we used vetted GBD tools, such as Disease-Model-Bayesian Meta-Regression (DisMod-MR) and the Cause Of Death Ensemble model (CODEm) frameworks, with cross-validation approaches (eg, leaving out 20% of data and root mean square error [RMSE]) to establish standard model settings.

For non-fatal estimates, we input all data into a DisMod-MR model, obtained an initial global fit across all data regardless of collection year or geography, and estimated coefficients for predictive covariates. The global fit was passed down as a prior to the next level of the geographical cascade, which comprised the seven super-regions, and models were run for each super-region with input data only from the respective super-region. This process was repeated for regions and countries. Random effects on locations informed whether the prior passed down from the previous level of the geographical cascade was higher or lower than the original fit. Data from all locations informed the model fitted for north Africa and the Middle East in combination with data specific to countries in north Africa and the Middle East, and the data were harmonised before model input to account for differing case definitions. We ran 5000 samples and took the 1000 posterior samples after model convergence to get stable estimates with estimated uncertainty.

For modelling of mortality, deaths due to neurological conditions were estimated using CODEm. Input data consisted primarily of International Classification of Diseases (ICD) 9th or 10th revision coded vital registration data, supplemented in some cases by verbal autopsy data, police records, or registry data. Before input into cause of death ensemble models, death data that had insufficiently defined ICD coding were redistributed to other underlying causes, and all data went through noise-reduction to account for variation in temporal trends due to small numbers. Linear mixed-effect component models were run with all input data and used to select covariates associated with the disease outcome. Subsequent models ran through location-time, smoothing by borrowing information over location, time, and age. Subsequent models were run through location-time smoothing and Gaussian process regression. Location-time smoothing borrows information over location, time, and age. Gaussian process regression was used in location-age smoothing with at least one datapoint to improve predictions. Component models were assessed for performance (eg, RMSE and percentage of correct predictions of time trend from adjacent datapoints) and were weighted in a final ensemble model that maximised out-of-sample predictive validity. Final model uncertainty was determined from regression parameters, variance around input datapoints, and heterogeneity of component models.

Deaths, YLDs, YLLs, and DALYs for each condition are additive in our results (eg, DALYs for stroke and DALYs for dementia can be added together for a combined DALY burden), whereas for prevalence we accounted for independent comorbidity using a comorbidity correction, but since we only did this correction for the original neurological grouping, we opted not to emphasise total case numbers across all conditions.

Detailed methods, including eligibility criteria, literature search strategy, case definitions for each disease, data selection and extraction, and fatal and non-fatal disease modelling for generating estimates, predictive covariates, and geographical proximity are described in appendix 4 (pp 10–89). Analyses were done using Python (version 3.6.2), Stata (version 13), and R (version 3.5.0). The statistical code used for GBD estimation is available online.

### Role of the funding source

The funder of the study had no role in study design, data collection, data analysis, data interpretation, or writing of the report.

## Results

In 2019, neurological conditions resulted in 286·3 million (95% UI 224·8–369·3) DALYs globally, with 17·6 million (12·5–24·7) occurring in the 21 countries comprising the north Africa and the Middle East super-region (10·7% of 164·8 million all-cause DALYs in north Africa and the Middle East). Overall, burden of DALYs due to neurological conditions in this super-region ranked second after cardiovascular diseases, excluding stroke (with approximately 22·9 million DALYs). In particular, stroke was ranked the second highest contributor to all-cause DALYs in this super-region (with 7·9 million DALYs [7·1–8·9]) in 2019, after ischaemic heart disease (with 18·0 million DALYs [15·6–20·8]). In north Africa and the Middle East in 2019, an estimated 441·1 thousand (347·2–598·4) neurological deaths occurred (14·2% of 3·1 million all-cause deaths in north Africa and the Middle East), as the second leading cause of deaths after cardiovascular diseases with approximately 984·5 million (872·6–1085·4) deaths (31·7% of 3·1 million all-cause deaths).

**Figure 1:**
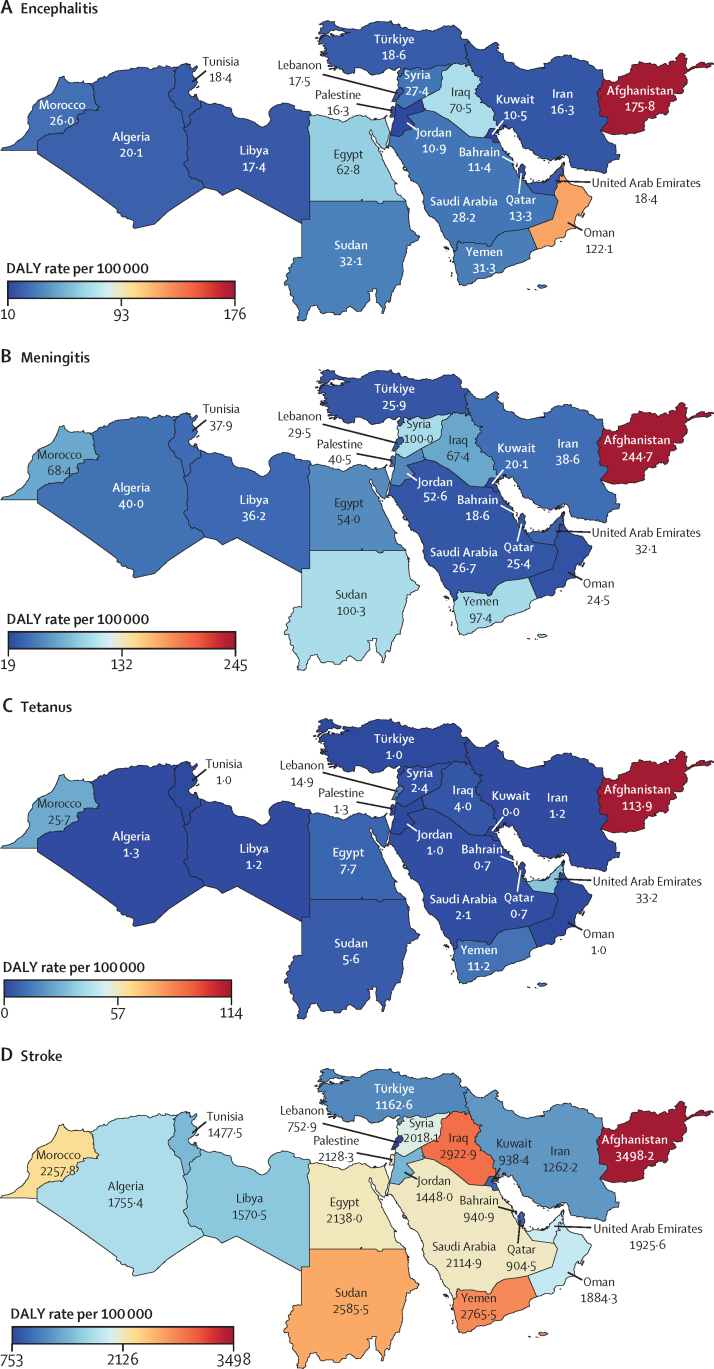

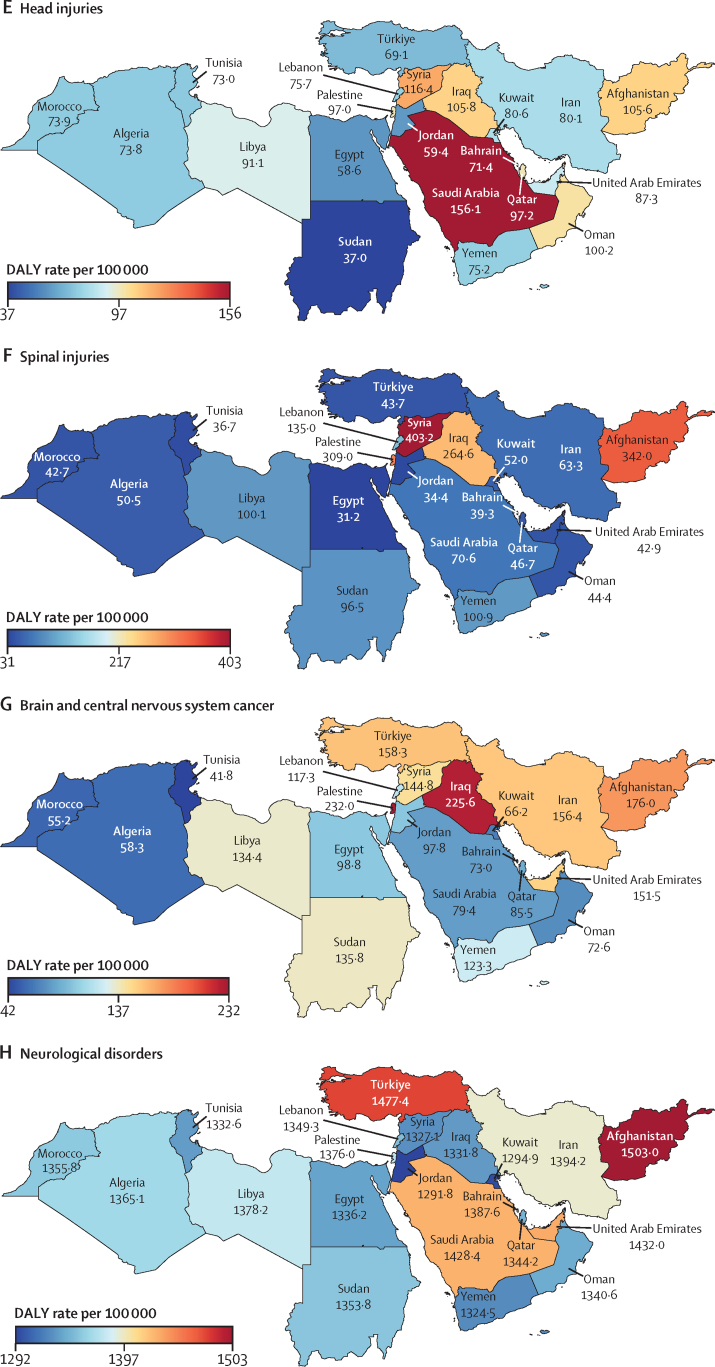

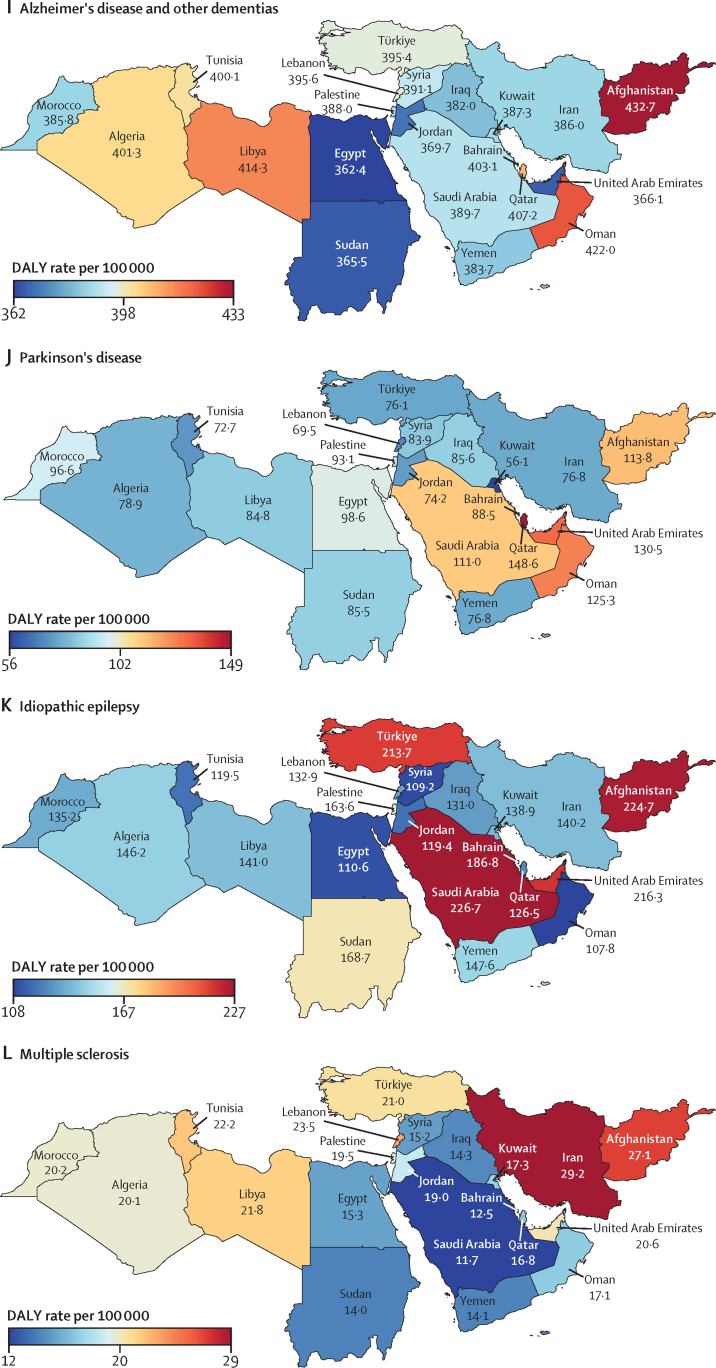

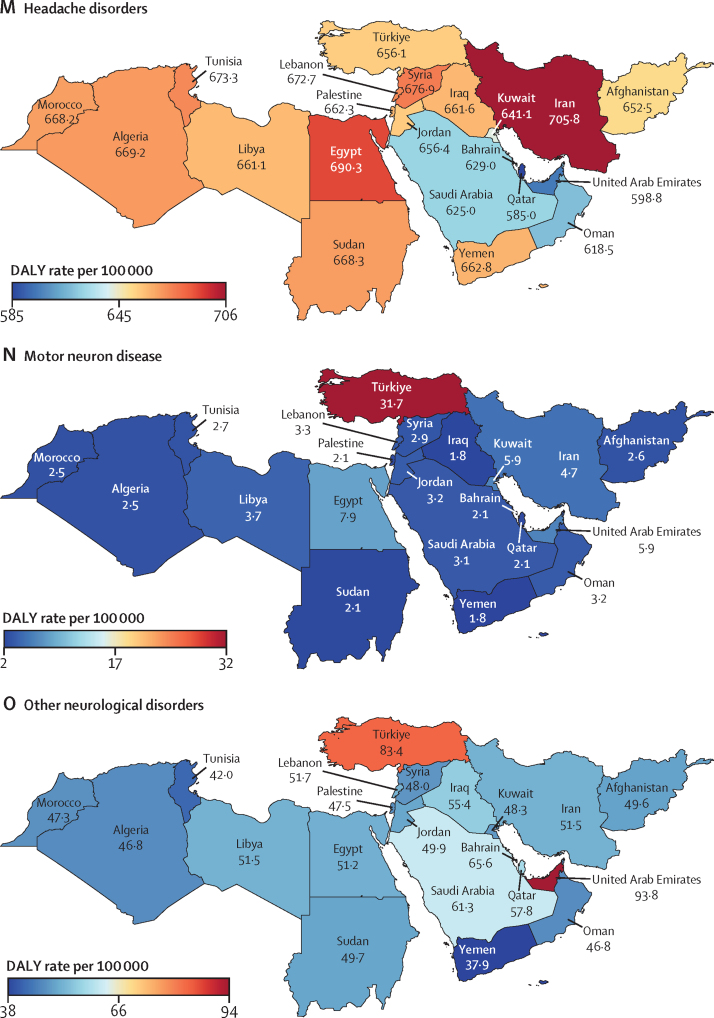
Heat maps of the burden of neurological conditions in north Africa and the Middle East, 2019 For head injuries and spinal injuries, since there is no fatal compoenet, DALYs are equivalent to years lived with disability (YLDs). DALYs=disability-adjusted life-years.

In 2019, the age-standardised rate of DALYs due to stroke in north Africa and the Middle East (1826·2 [95% UI 1635·3–2026·2] per 100 000; table 1) was higher than in the Latin America and Caribbean, south Asia, and high-income super-regions (appendix 4 pp 99, 105–42). Among the global super-regions, north Africa and the Middle East had the highest rates of DALYs per 100 000 population due to dementias (387·0 [172·0–848·5]), Parkinson's disease (84·4 [74·7–103·2]), and migraine (601·4 [107·0–1371·8]) and the second highest, after central Europe, eastern Europe, and central Asia, for ischaemic stroke (1183·6 [1060·8–1307·0]) and brain and CNS cancer (128·3 [87·8–151·3] table 1; appendix 4 pp 105–42). The country-specific burdens of 15 neurological conditions (measured by age-standardised rates of DALY or YLD) in the region are shown in figure 1.

**Table 1:** Estimated DALYs, deaths, incidence, prevalence, YLDs, and YLLs of neurological conditions in north Africa and the Middle East, from 1990 to 2019

	**All ages**		**Age-standardised estimates**		
	Counts, 2019 (thousands)	Percentage change in counts, 1990 to 2019	Rate per 100 000, 2019	Female to male ratio, 2019	Percentage change in rate, 1990 to 2019
**Meningitis**					
DALYs	412·8 (345·2 to 495)	−70·5% (−77·5 to −62·3)	70·0 (58·7 to 83·7)	0·91	−75·8% (−81·1 to −69·5)
Deaths	6·3 (5·3 to 7·4)	−64·1% (−72·2 to −54·5)	1·2 (1·0 to 1·4)	0·88	−71·6% (−77·1 to −64·9)
Incidence	128·5 (106·7 to 152·7)	−9·9% (−16·3 to −1·9)	22·5 (18·9 to 26·4)	1·12	−37·0 (−39·9 to −33·9)
Prevalence	356·2 (306·7 to 419·9)	5·7% (−1·6 to 14·0)	60·5 (52·2 to 71·1)	1·12	−44·7% (−48·4 to −40·7)
YLDs	36·2 (25·1 to 48·7)	17·1% (9·2 to 26·8)	6·0 (4·2 to 8·0)	1·16	−34·6% (−39·0 to −29·4)
YLLs	376·5 (310·2 to 457·5)	−72·5% (−79·3 to −64·6)	64·1 (53 to 77·6)	0·89	−77·2% (−82·4 to −70·9)
**Encephalitis**					
DALYs	251·0 (200·2 to 332·9)	13·5% (−21·0 to 59·6)	42·0 (33·6 to 55·6)	1·09	−20·7% (−43·3 to 8·7)
Deaths	3·9 (3·1 to 5·1)	26·2% (−9·4 to 71·2)	0·7 (0·6 to 0·9)	1·03	−24·9% (−42·7 to −1·9)
Incidence	58·1 (49·8 to 67)	46·2% (40·2 to 53·3)	9·9 (8·5 to 11·3)	1·21	−4·4% (−5·8 to −3·0)
Prevalence	167·5 (127·8 to 205·9)	79·3% (73·4 to 86·9)	28·0 (21·2 to 34·6)	1·30	−10·4% (−13·5 to −5·9)
YLDs	19·8 (13·8 to 26·8)	84·9% (76·6 to 93·9)	3·2 (2·2 to 4·3)	1·35	−4·1% (−8·1 to 0·4)
YLLs	231·3 (179·8 to 314·1)	9·8% (−25·4 to 58·2)	38·8 (30·3 to 52·5)	1·07	−21·8% (−45·9 to 9·5)
**Tetanus**					
DALYs	87·4 (53·6 to 139·5)	−85·8% (−92·9 to −68·7)	15·0 (9·2 to 24·1)	0·85	−88·2% (−93·9 to −76·1)
Deaths	1·3 (0·8 to 2)	−84·1% (−91·3 to −69·0)	0·2 (0·2 to 0·4)	0·84	−88·9% (−93·3 to −80·6)
Incidence	1·9 (1·2 to 2·9)	−83·0% (−90·6 to −68·0)	0·3 (0·2 to 0·5)	0·82	−88·4% (−92·9 to −79)
Prevalence	0·7 (0·5 to 1·1)	−35·7% (−53·8 to −16·2)	0·1 (0·1 to 0·2)	0·63	−61·7% (−70·5 to −51·9)
YLDs	<0·1 (<0·1 to <0·1)*	−70·2% (−81·9 to −50·5)	<0·1 (<0·1 to <0·1)†	0·71	−80·9% (−87·4 to −70·7)
YLLs	87·4 (53·6 to 139·5)	−85·8% (−92·9 to −68·7)	15·0 (9·2 to 24·1)	0·85	−88·2% (−93·9 to −76·1)
**Brain and central nervous system cancer**					
DALYs	716·3 (493·9 to 848·2)	71·0% (0·9 to 131·7)	128·3 (87·8 to 151·3)	0·86	−5·0% (−40·0 to 23·4)
Deaths	17·8 (12·1 to 20·9)	111·5% (30·3 to 173·4)	3·7 (2·5 to 4·3)	0·87	3·0% (−33·4 to 30·2)
Incidence	27·5 (18·6 to 32·6)	152·5% (49·6 to 233·1)	5·2 (3·5 to 6·1)	0·84	28·0% (−19·6 to 63·3)
Prevalence	97·2 (64·2 to 115·6)	280·5% (113·9 to 424·7)	16·5 (10·8 to 19·5)	0·81	119·1% (30·2 to 189·1)
YLDs	10·6 (6·2 to 15·0)	203·1% (78·6 to 311·3)	1·9 (1·1 to 2·7)	0·78	55·6% (−2·6 to 105·3)
YLLs	705·6 (486 to 836·9)	69·9% (0·4 to 130·4)	126·4 (86·6 to 149·3)	0·86	−5·6% (−40·2 to 22·6)
**Stroke**‡					
DALYs	7946·0 (7060·2 to 8870·8)	43·3% (27·2 to 61·4)	1826·2 (1635·3 to 2026·2)	1·04	−32·0% (−39·1 to −23·3)
Deaths	312·2 (278·4 to 349·7)	75·5% (56·2 to 98·8)	87·7 (78·2 to 97·6)	1·07	−27·8% (−35·4 to −16·0)
Incidence	829·8 (758·4 to 912·8)	130·7% (124·4 to 137·7)	183·0 (166·7 to 201·7)	1·13	−5·4% (−7·4 to −3·3)
Prevalence	7323·4 (6794·7 to 7863·1)	142·1% (137·8 to 146·3)	1537·5 (1421·9 to 1659·9)	1·24	−0·5% (−2·3 to 1·1)
YLDs	1113·7 (812·1 to 1400·8)	140·2% (135·3 to 145·3)	239·4 (176·1 to 301·4)	1·43	−0·7% (−2·6 to 1·2)
YLLs	6832·3 (6014·8 to 7787·8)	34·5% (18·0 to 53·6)	1586·8 (1407 to 1782·8)	0·99	−35·1% (−42·5 to −25·9)
**Ischaemic stroke**					
DALYs	4751·4 (4227·7 to 5271·9)	120·0% (89·0 to 148·1)	1183·6 (1060·8 to 1307·0)	1·09	−8·8% (−19·6 to 2·2)
Deaths	210·1 (187·1 to 234·0)	141·8% (110·1 to 175·0)	62·9 (56·3 to 69·9)	1·10	−9·1% (−20·7 to 4·0)
Incidence	602·5 (531·3 to 682·5)	166·9% (157·2 to 177·2)	135·5 (119·7 to 153·6)	1·16	8·8% (6·3 to 11·4)
Prevalence	5998·8 (5474·0 to 6566·7)	157·6% (152·8 to 162·7)	1303·6 (1183·2 to 1435·4)	1·25	5·9% (3·6 to 8)
YLDs	877·3 (639·8 to 1114·1)	157·6% (151·5 to 164·4)	195·6 (144·4 to 249·1)	1·45	5·2% (2·8 to 7·6)
YLLs	3874·1 (3427·8 to 4371·1)	112·9% (77·1 to 146·1)	987·9 (880·9 to 1105·9)	1·03	−11·1% (−23·3 to 1·3)
**Intracerebral haemorrhage**					
DALYs	2702·2 (2343·7 to 3120·3)	3·4% (−12·3 to 21·4)	548·4 (479·5 to 623·1)	0·93	−51·7% (−58·2 to −43·8)
Deaths	88·5 (77·9 to 101·1)	18·0% (1·5 to 37·8)	21·6 (19·0 to 24·3)	0·99	−51·4% (−58·1 to −41·3)
Incidence	163·2 (149·2 to 179·8)	65·1% (60·7 to 69·7)	35·0 (31·8 to 38·6)	1·00	−32·6% (−34·5 to −30·6)
Prevalence	1301·3 (1169·2 to 1441·4)	89·8% (86·0 to 93·8)	241·6 (217·5 to 265·7)	1·07	−21·1% (−23·1 to −19·2)
YLDs	182·2 (129·1 to 229·6)	88·9% (82·6 to 95·8)	34·4 (24·4 to 43·5)	1·26	−21·5% (−24·2 to −18·4)
YLLs	2520·0 (2171·8 to 2943·1)	0·1% (−15·7 to 18·7)	514·0 (447·9 to 593·0)	0·91	−52·9% (−59·7 to −44·9)
**Subarachnoid haemorrhage**					
DALYs	492·4 (408·3 to 617·2)	−36·0% (−50·6 to 6·8)	94·2 (79·0 to 116·8)	1·08	−62·8% (−71·6 to −41·0)
Deaths	13·6 (11·2 to 16·8)	−15·0% (−35·7 to 38·8)	3·2 (2·6 to 3·9)	1·05	−59·0% (−70·7 to −31·6)
Incidence	64·1 (54·6 to 75·6)	82·9% (75·3 to 92·5)	12·5 (10·7 to 14·7)	1·24	−26·4% (−29·2 to −23·3)
Prevalence	376·9 (320·2 to 444·6)	103·4% (85·0 to 112·9)	64·0 (54·5 to 75·3)	1·58	−18·1% (−26·3 to −14·1)
YLDs	54·2 (37·9 to 72·3)	103·1% (83·8 to 116·5)	9·3 (6·6 to 12·4)	1·77	−17·9% (−26·2 to −12·2)
YLLs	438·2 (355·9 to 559·6)	−41·0% (−55·3 to 0·5)	84·8 (69·4 to 107·1)	1·03	−64·9% (−73·3 to −43·4)
**Neurological disorders**§					
DALYs	7156·0 (3769·7 to 12 184·4)	91·1% (72·0 to 109·7)	1382·1 (776·6 to 2273·8)	1·25	−3·9% (−10·3 to 3·5)
Deaths	99·6 (47·3 to 213·2)	153·3% (111·0 to 207·8)	33·2 (14·3 to 73·9)	1·01	−2·8% (−9·6 to 13·9)
Incidence	61 542·7 (54 639·6 to 68 418·4)	89·1% (82·4 to 95·8)	10 090·8 (9024·1 to 11 159·3)	1·07	0·8% (0·0 to 1·6)
Prevalence	208 460·5 (190 465·7 to 226 945·6)	101·2% (95·9 to 106·6)	34 170·6 (31 389·5 to 37 068·5)	1·19	1·4% (0·6 to 2·3)
YLDs	5447·8 (2227·5 to 10 439·2)	99·6% (83·8 to 121·6)	935·0 (419·6 to 1737·7)	1·42	0·2% (−6·5 to 8·3)
YLLs	1708·2 (1049·4 to 3130·8)	68·3% (24·8 to 124·2)	447·2 (241·7 to 897·3)	0·97	−11·5% (−23·1 to 7·1)
**Alzheimer's disease and other dementias**					
DALYs	1208·1 (532·0 to 2672·8)	177·3% (161·1 to 215·8)	387·0 (172·0 to 848·5)	1·12	−1·1% (−6·6 to 12·1)
Deaths	70·5 (17·2 to 185·8)	191·6% (169·8 to 253·2)	25·5 (6·3 to 67·1)	1·12	−2·3% (−9·0 to 17·3)
Incidence	361·2 (309·7 to 413·1)	177·5% (171·2 to 184·3)	110·2 (93·9 to 125·6)	1·09	0·8% (−0·7 to 2·6)
Prevalence	2485·1 (2117·2 to 2865·0)	184·5% (178·1 to 190·7)	777·6 (660·8 to 896)	1·11	3·0% (1·5 to 4·6)
YLDs	352·2 (248·1 to 474·3)	187·7% (180·0 to 195·6)	112·8 (79·1 to 151·9)	1·10	2·8% (0·9 to 4·6)
YLLs	855·9 (207·4 to 2304·9)	173·2% (151·8 to 232·1)	274·2 (67·3 to 727·7)	1·13	−2·6% (−9·7 to 17·2)
**Parkinson's disease**					
DALYs	300·7 (266·3 to 365·4)	163·8% (134·8 to 202·1)	84·4 (74·7 to 103·2)	0·68	0·9% (−10·2 to 15·7)
Deaths	16·8 (14·6 to 21·6)	179·4% (145·9 to 225·5)	5·3 (4·6 to 6·9)	0·66	2·3% (−10·0 to 19·0)
Incidence	42·8 (38·3 to 47·3)	192·5% (184·3 to 201·9)	11·4 (10·3 to 12·5)	0·68	12·7% (9·9 to 15·7)
Prevalence	309·9 (265·0 to 362·8)	199·5% (188·8 to 211·2)	82·6 (70·2 to 95·6)	0·74	15·4% (11·5 to 20·0)
YLDs	44·5 (30·7 to 60·5)	197·3% (182·1 to 214·4)	11·7 (8·0 to 15·7)	0·73	14·8% (9·2 to 21·2)
YLLs	256·2 (225·1 to 319·5)	158·7% (125·3 to 202·8)	72·7 (63·9 to 91·5)	0·67	−1·0% (−13·1 to 15·8)
**Idiopathic epilepsy**					
DALYs	955·3 (682·8 to 1293·2)	13·8% (−13·8 to 56·0)	158·3 (112·6 to 213·2)	0·87	−26·2% (−43·6 to −1·1)
Deaths	6·6 (5·4 to 7·6)	5·3% (−16·7 to 70·4)	1·2 (1·0 to 1·4)	0·88	−33·4% (−46·2 to 2·1)
Incidence	295·5 (195·0 to 407·7)	67·4% (20·3 to 135·5)	48·2 (32·2 to 66·4)	0·85	8·3% (−21·5 to 51·4)
Prevalence	1990·9 (1360·2 to 2600·0)	77·6% (26·2 to 150·6)	336·9 (231·8 to 437·4)	0·88	8·6% (−23·0 to 52·4)
YLDs	603·1 (342·3 to 938·7)	47·5% (−1·0 to 119·0)	101·0 (57·3 to 156·5)	0·87	−9·3% (−39·8 to 33·8)
YLLs	352·2 (279·1 to 412·5)	−18·1% (−37·2 to 39·0)	57·3 (45·6 to 67·2)	0·89	−44·4% (−55·6 to −11·7)
**Multiple sclerosis**					
DALYs	115·9 (93·1 to 144·8)	146·0% (98·9 to 205·5)	19·9 (16·1 to 24·7)	1·58	6·2% (−11·6 to 30·5)
Deaths	1·4 (1·2 to 1·8)	147·3% (83·1 to 248·0)	0·3 (0·2 to 0·3)	1·21	5·1% (−25·0 to 47·6)
Incidence	9·2 (7·9 to 10·5)	120·0% (110·0 to 129·4)	1·4 (1·2 to 1·6)	1·75	5·5% (4·0 to 6·8)
Prevalence	222·7 (190·7 to 256·8)	171·3% (165·8 to 176·5)	39·0 (33·6 to 44·7)	1·98	11·5% (10·0 to 12·8)
YLDs	57·7 (40·8 to 76·8)	169·3% (156·5 to 183·1)	10·0 (7·1 to 13·3)	1·88	10·9% (6·1 to 16·3)
YLLs	58·2 (45·2 to 80·4)	126·5% (55·9 to 238·0)	9·9 (7·8 to 13·6)	1·34	1·8% (−27·9 to 52·7)
**Headache disorders**¶					
DALYs	4209·8 (990·1 to 9068·3)	103·3% (94·5 to 123·7)	669·6 (159·1 to 1431·3)	1·65	0·1% (−2·4 to 2·4)
Incidence	60 830·7 (53 891·3 to 67 676·1)	88·8% (82·0 to 95·6)	9919·0 (8853·1 to 10 989·5)	1·07	0·8% (−0·1 to 1·5)
Prevalence	205 280·4 (186 994·5 to 223 750·1)	100·9% (95·4 to 106·3)	33 389·7 (30 530·9 to 36 328·1)	1·19	1·3% (0·5 to 2·2)
YLDs	4209·8 (990·1 to 9068·3)	103·3% (94·5 to 123·7)	669·6 (159·1 to 1431·3)	1·65	0·1% (−2·4 to 2·4)
**Migraine**					
DALYs	3793·2 (645·3 to 8665·8)	102·1% (93·2 to 125·1)	601·4 (107·0 to 1371·8)	1·70	0·0 % (−1·6 to 1·6)
Incidence	7950·9 (6837·6 to 9083·6)	70·6% (62·7 to 79·1)	1238·9 (1063·5 to 1415·6)	1·69	0·4% (−0·7 to 1·6)
Prevalence	96 931·5 (83 756·7 to 112 609·4)	100·9% (92·7 to 108·8)	15 355·0 (13 305·5 to 17 806·0)	1·76	−0·1% (−1·5 to 1·4)
YLDs	3793·2 (645·3 to 8665·8)	102·1% (93·2 to 125·1)	601·4 (107·0 to 1371·8)	1·70	0·0 % (−1·6 to 1·6)
**Tension-type headache**					
DALYs	416·6 (138·3 to 1196·8)	115·5% (84·9 to 132·3)	68·1 (22·8 to 195·5)	1·28	1·0 % (−9·5 to 8·7)
Incidence	52 879·8 (46 137·0 to 59485·2)	91·9% (84·0 to 99·8)	8680·1 (7631·6 to 9732·5)	1·01	0·9% (−0·1 to 1·7)
Prevalence	149 061·7 (128 455·9 to 170 990·9)	102·2% (93·5 to 111·0)	24 504·5 (21 304·8 to 27 987·5)	1·02	2·0 % (0·7 to 3·4)
YLDs	416·6 (138·3 to 1196·8)	115·5% (84·9 to 132·3)	68·1 (22·8 to 195·5)	1·28	1·0 % (−9·5 to 8·7)
**Motor neuron disease**					
DALYs	41·6 (33·8 to 50·6)	22·5% (−28·9 to 88·7)	7·8 (6·4 to 9·5)	0·73	−16·6% (−44·8 to 17·9)
Deaths	1·1 (0·9 to 1·3)	90·4% (23·9 to 177·6)	0·2 (0·2 to 0·3)	0·73	9·3% (−26·7 to 51·1)
Incidence	3·4 (2·9 to 4·1)	83·1% (70·8 to 94·9)	0·6 (0·5 to 0·7)	0·82	1·9% (0·3 to 3·6)
Prevalence	15·6 (12·7 to 19)	103·3% (92·1 to 113·8)	2·6 (2·1 to 3·1)	0·86	3·5% (2·0 to 5·1)
YLDs	3·3 (2·3 to 4·6)	103·3% (92·1 to 113·8)	0·5 (0·4 to 0·8)	0·86	3·6% (2·0 to 5·1)
YLLs	38·3 (30·6 to 47·1)	18·4% (−32·8 to 87·7)	7·3 (5·8 to 9)	0·72	−17·8% (−47·1 to 19·1)
**Other neurological disorders**					
DALYs	324·7 (250·4 to 417·3)	59·4% (26·5 to 98·9)	55·2 (43 to 70·4)	0·76	6·3% (−13·5 to 30·6)
Deaths	3·3 (2·9 to 3·8)	85·9% (42·8 to 136·2)	0·7 (0·6 to 0·8)	0·67	17·4% (−7·9 to 49·9)
Incidence	NA	NA	NA	NA	NA
Prevalence	3·9 (2·6 to 5·5)	96·6% (81·0 to 113·8)	0·7 (0·5 to 1·0)	1·00	0·3% (−0·9 to 1·5)
YLDs	177·2 (106·1 to 265·1)	98·6% (41·9 to 177·9)	29·4 (18·0 to 43·9)	0·92	25·5% (−8·9 to 71·0)
YLLs	147·4 (127·9 to 171·8)	28·8% (−3·1 to 67·3)	25·7 (22·4 to 29·9)	0·61	−9·5% (−30·0 to 15·6)
**Head injuries**‖					
Incidence	1911·5 (1624·9 to 2305·2)	49·0% (31·3 to 67·4)	318·6 (271·9 to 382·4)	0·58	−14·5% (−24·8 to −4·0)
Prevalence	3096·9 (2843·7 to 3481·3)	127·5% (113·2 to 135·7)	552·2 (507·4 to 619·2)	0·54	2·4% (−3·3 to 5·7)
YLDs	453·0 (331·6 to 604·0)	124·9% (111·3 to 133·7)	80·1 (58·6 to 106·9)	0·53	1·9% (−3·5 to 5·4)
**Minor TBI**					
Incidence	1008·8 (766·2 to 1364·3)	67·1% (46·0 to 87·7)	161·5 (122·4 to 220·8)	0·58	−1·1% (−14·1 to 13·2)
Prevalence	720·4 (591·3 to 955·6)	119·7% (98·8 to 132·8)	129·0 (106·2 to 170·2)	0·58	0·2% (−9·6 to 6·3)
YLDs	86·6 (60·6 to 118·7)	120·2% (98·7 to 134·7)	15·4 (10·9 to 21·0)	0·57	0·7% (−9·4 to 7·1)
**Moderate or severe TBI**					
Incidence	902·7 (790·6 to 1024·6)	33·0 % (10·3 to 57·0)	157·1 (137·9 to 179·1)	0·58	−25% (−37·3 to −12·1)
Prevalence	2376·5 (2188·4 to 2700·6)	130·0% (116·8 to 138·2)	423·2 (390·4 to 479·2)	0·53	3·1% (−2·0 to 6·1)
YLDs	366·4 (262·9 to 489·6)	126·0% (113·5 to 134·9)	64·7 (46·6 to 86·2)	0·52	2·1% (−2·6 to 5·7)
Spinal injuries**					
Incidence	52·8 (34·8 to 97·7)	23·6% (−24·7 to 91·9)	9·2 (6·0 to 17·0)	0·76	−32·7% (−60·7 to 8·9)
Prevalence	1597·7 (989·3 to 3176·1)	109·7% (55·4 to 149·4)	264·3 (165·5 to 520·5)	0·71	5·0 % (−19·4 to 21·4)
YLDs	564·2 (289·6 to 1229·1)	85·5% (36·6 to 124·6)	92·2 (48·0 to 198·8)	0·70	−5·4% (−27·6 to 12·3)
**Spinal cord lesion at neck level**					
Incidence	37·7 (20·8 to 81·1)	55·3% (−12·6 to 145·9)	6·6 (3·6 to 14·3)	0·77	−13·3% (−54·0 to 45·7)
Prevalence	1179·7 (578·7 to 2753·3)	98·8% (42·8 to 150·3)	192·6 (95·9 to 445·7)	0·70	2·3% (−24·7 to 24·9)
YLDs	478·8 (220 to 1142·1)	85·0% (33·5 to 134·8)	77·7 (36·2 to 184·0)	0·69	−3·6% (−28·8 to 18·6)
**Spinal cord lesion below neck level**					
Incidence	15·1 (11·5 to 19·6)	−18·2% (−57·7 to 42·1)	2·6 (2·0 to 3·4)	0·76	−57·2% (−78·4 to −24·2)
Prevalence	418·0 (387·0 to 462·5)	148·2% (141·0 to 157·3)	71·7 (66·4 to 79·4)	0·72	13·0 % (9·8 to 17·6)
YLDs	85·4 (59·7 to 113·8)	88·1% (74·3 to 101·8)	14·5 (10·2 to 19·3)	0·71	−14·1% (−20·4 to −7·9)

The 14 major neurological conditions and eight subtypes conditions are in bold. Data in parentheses are 95% UIs. All data are given to one decimal place, expect for female to male ratio, which is provided to two decimal places. Percentages and numbers are not mutually exclusive: the sum of percentages and numbers in the columns exceeds the totals for all causes combined because of overlap between various causes. Measures with insufficient data are not reported here. DALYs=disability-adjusted life-years. NA=no data available. TBI=traumatic brain injury. UI=uncertainty interval. YLDs=years lived with disability. YLLs=years of life lost.

* Crude YLD count for tetanus in 2019 was 27·3 (95% UI 15·4–42·5).

† Age-standardised rate of tetanus per 100 000 was 0·005 (95% UI 0·003–0·008).

‡ Stroke includes ischaemic stroke, intracerebral haemorrhage, and subarachnoid haemorrhage.

§ Neurological disorders include Alzheimer's disease and other dementias, Parkinson's disease, idiopathic epilepsy, multiple sclerosis, headache disorders (including migraine and tension-type headache), motor neuron disease, and other neurological disorders.

¶ Headache disorders include migraine and tension-type headache.

‖ Head injury includes minor TBI and moderate or severe TBI.

** Spinal injuries include spinal cord lesion at neck level and below neck level.

In 2019, among all neurological conditions in north Africa and the Middle East, stroke, migraine, and dementias had the highest absolute numbers of neurological DALYs (overall 73·6% of total neurological DALYs; table 1; appendix 4 p 92). Stroke and dementias were the leading causes of neurological deaths (86·8%). Egypt, Türkiye, Iran, Morocco, Iraq, and Algeria had the highest numbers of stroke DALYs, deaths, incidence, and prevalence; and Türkiye, Iran, Egypt, Algeria, Morocco, and Iraq had the highest numbers of dementia DALYs (appendix 4 pp 143–94). For neuroinfectious diseases (ie, meningitis, encephalitis, and tetanus), Afghanistan, Egypt, Iraq, Sudan, and Iran had the highest DALYs. After excluding stroke, dementia, and neuroinfectious diseases, Türkiye, Egypt, Iran, and Iraq were the countries with the highest DALYs related to all other neurological conditions.

Between 1990 and 2019, the absolute number of DALYs and deaths related to meningitis, tetanus, and subarachnoid haemorrhage decreased in north Africa and the Middle East, but for all other neurological conditions they increased (appendix 4 pp 143–253). The highest increases in the number of DALYs were related to dementias (177·3% [95% UI 161·1 to 215·8]), Parkinson's disease (163·8% [134·8 to 202·1]), multiple sclerosis (146·0% [98·9 to 205·5]), and ischaemic stroke (120·0% [89·0 to 148·1]; table 1). Furthermore, the highest increases in incidence during this period were related to Parkinson's disease (192·5% [184·3 to 201·9]), dementia (177·5% [171·2 to 184·3]), ischaemic stroke (166·9% [157·2 to 177·2]), and brain and CNS cancer (152·5% [49·6 to 233·1]). Notably, from 2010 to 2019, the incidence counts for dementias more than doubled in the United Arab Emirates (UAE; 168·4% [153·3 to 184·4]), Qatar (125·6% [112·7 to 140·5]), Jordan (105·0% [96·6 to 113·6]), and Bahrain (102·1% [91·2 to 114·3]; appendix 4 pp 540–52). The UAE and Qatar had the most substantial increases in absolute numbers of DALYs, deaths, incidence, and prevalence related to almost all neurological conditions (appendix 4 pp 540–52).

**Figure 2:**
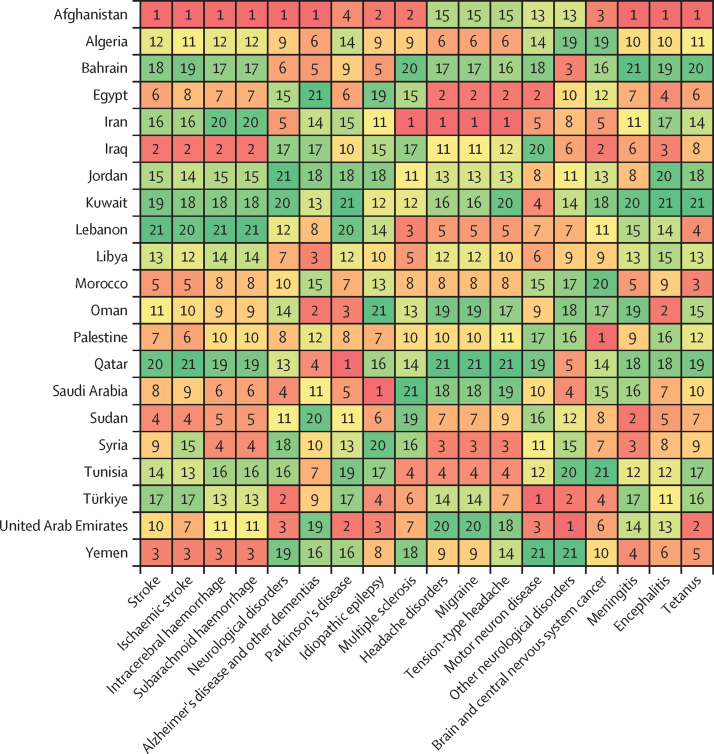
Ranks of age-standardised DALY rates related to neurological conditions in countries in north Africa and the Middle East, 2019 Ranks range from 1 (dark red) with the highest rate to 21 (dark green) with the lowest rate. Stroke includes ischaemic stroke, intracerebral haemorrhage, and subarachnoid haemorrhage. Neurological disorders include Alzheimer's disease and other dementias, Parkinson's disease, idiopathic epilepsy, multiple sclerosis, headache disorders, motor neuron disease, and other neurological disorders. Headache disorders include migraine and tension-type headache. DALY=disability-adjusted life-year.

On the basis of age-standardised rates in 2019, we observed the highest incidence and prevalence of neuroinfectious diseases in Afghanistan, of brain and CNS cancer in Palestine (with the highest incidence) and in Lebanon (with the highest prevalence), of stroke in the UAE, and of dementias in Türkiye (appendix 4 pp 143–253). Afghanistan had the highest rates of deaths and DALYs related to stroke, neuroinfectious diseases, and dementia; figure 2). Afghanistan, Oman, and Libya had the highest dementia-related deaths and DALY rates. Palestine and Iraq had the highest rates of deaths and DALYs related to brain and CNS cancer. In contrast, Lebanon, Kuwait, Qatar, and Bahrain had the lowest rates of stroke deaths and DALYs. Kuwait, Bahrain, Qatar, and Türkiye had the lowest rates of deaths and DALYs related to neuroinfectious diseases. We observed the lowest incidence rates or prevalence rates, or both, in Türkiye for meningitis, in Morocco for brain and CNS cancer, in Bahrain for stroke, and in the UAE for dementias.

During 1990–2019, there was a decline or flattening in trends for age-standardised DALYs, deaths, incidence, and prevalence rates of almost all neurological conditions in north Africa and the Middle East (appendix 4 pp 100, 195–253). The highest significant increases in DALY rates were in Morocco for Parkinson's disease (39·6%) and in Kuwait for multiple sclerosis (38·2%). We observed the greatest significant decreases in DALY rates for each of the neurological conditions in Türkiye for meningitis (–89·9%) and tetanus (–98·3%), in Jordan for encephalitis (–60·2%) and ischaemic stroke (–46·3%), in Algeria for intracerebral haemorrhage (–67·9%), in Egypt for subarachnoid haemorrhage (–77·4%), in Bahrain for stroke (–52·5%), and in Kuwait for Parkinson's disease (–26·8%; appendix 4 pp 195–253). For idiopathic epilepsy only Iran (–34·8%), and for motor neuron disease only Kuwait (–61·7%) and Bahrain (–54·1%) had significant decreases in DALY rates. None of the countries showed significant changes in age-standardised DALY rates for brain and CNS cancer, dementia, multiple sclerosis, migraine, tension-type headache, and other neurological disorders between 1990 and 2019. However, there was a significant increase in age-standardised YLD rates of Parkinson's disease, multiple sclerosis, ischaemic stroke, and dementias (table 1). Additionally, from 2010 to 2019, there was a significant increase in age-standardised YLD rates of brain and CNS cancer (22·3% [2·5 to 37·9]), motor neuron disease (5·4% [3·8 to 7·1]), and multiple sclerosis (4·7% [0·1 to 9·4]). And age-standardised incidence and prevalence rates of head injuries, spinal injuries, Parkinson's disease, motor neuron disease, multiple sclerosis, headache disorder, and tension-type headache; and prevalence of brain and CNS cancer increased significantly in north Africa and the Middle East (appendix 4 pp 553–56). During this period, there was significant reduction in incidence and prevalence rates of meningitis, tetanus, and intracerebral haemorrhage, and in incidence for subarachnoid haemorrhage (appendix 4 pp 553–65).

In 2019, 453·0 thousand (95% UI 331·6 to 604·0; 6·4%) of 7076·9 thousand (4997·9 to 9588·1) YLDs globally due to head injuries, and 564·2 thousand (289·6 to 1229·1; 9·1%) of 6200·1 thousand (4465·3 to 1856·2) YLDs globally due to spinal injuries happened in north Africa and the Middle East (appendix 4 pp 254–75). In the super-region, the highest age-standardised YLD rates per 100 000 population due to head injuries were in Saudi Arabia (156·1 [109·5 to 212·2]), Syria (116·4 [75·7 to 170·0]), Iraq 105·8 [76·4 to 143·3]), Afghanistan (105·6 [66·4 to 176·9]), and Oman (100·2 [70·5 to 136·3]). For spinal injuries, the highest age-standardised YLD rates per 100 000 population were in Syria (403·2 [112·6 to 1150·3]), Afghanistan (342·0 [87·2 to 1016·4]), Palestine (309·0 [100·2 to 843·4]), Iraq (264·6 [89·6–729·2]), and Lebanon (135·0 [45·1 to 386·4]). The greatest significant increases in age-standardised YLD rates for head injuries (107·7% [51·8 to 211·9]) and spinal injuries (579·2% [218·2 to 1263·6]) were in Syria, and the greatest significant decreases were in Lebanon for head injuries (–30·4% [–42·8 to –18·0]) and for spinal injuries (–58·1% [–63·8 to –41·5]) from 1990 to 2019 (appendix 4 pp 254–75).

In our assessment of risk factor specific estimates and trends, the neurological conditions potentially most affected by modifiable risk factors (ie, with the highest PAF and DALY rates in 2019) in north Africa and the Middle East included stroke (PAF of 85·8% [95% UI 82·6–89·1]; 1566·9 [1393·2–1757·1] DALYs per 100 000) and dementia (PAF 39·9% [26·4–54·7]; 154·7 [58·8–375·1] DALYs per 100 000; table 2). In 2019, PAFs of DALYs due to intracerebral haemorrhage (84·5% [81·1–87·7] in north Africa and the Middle East *vs* 88·1% [85·1–90·5] globally), subarachnoid haemorrhage (77·2% [72·5–81·8] *vs* 83·6% [80·3–86·7]), idiopathic epilepsy (2·1% [1·4–3·1] *vs* 10·1% [7·3–13·0]), stroke (85·8% [82·6–89·1] *vs* 86·4% [83·4–89·2]), and multiple sclerosis (10·8% [7·8–14·5] *vs* 13·7% [10·4–17·2]) were lower in north Africa and the Middle East than the overall global estimates (appendix 4 pp 276–320). In contrast, PAFs of ischaemic stroke (87·1% [82·8–92·0] *vs* 85·0% [80·2–89·9]), dementia (39·9% [26·4–54·7] *vs* 32·8% [21·8–45·8]), meningitis (7·1% [5·4–9·4] *vs* 6·8% [6·1–7·7]), and encephalitis (3·8% [2·2–6·1] *vs* 2·5% [1·9–2·9]) were higher in north Africa and the Middle East than overall globally.

**Table 2:** Risk-specific DALYs of neurological conditions in north Africa and the Middle East, from 1990 to 2019

	**Population attributable fraction, 2019**	**Female to male ratio of population attributable fraction, 2019**	**Change in population attributable fraction, from 1990 to 2019**	**Risk-attributed DALY rate per 100 000, 2019**	**Percentage change in DALY rate, from 1990 to 2019**
**All risk factors**					
Meningitis	7·1% (5·4 to 9·4)	0·75	−11·4% (−36·7 to 21·3)	5·0 (3·5 to 7·0)	−78·5% (−85·6 to −67·8)
Encephalitis	3·8% (2·2 to 6·1)	0·81	7·7% (−32·2 to 72·6)	1·6 (0·9 to 2·6)	−15·4% (−52·1 to 49·5)
Stroke	85·8% (82·6 to 89·1)	1·01	9·1% (4·8 to 14·4)	1566·9 (1393·2 to 1757·1)	−25·8% (−34·1 to −14·0)
Ischaemic stroke	87·1% (82·8 to 92·0)	1·00	5·0% (2·8 to 8·2)	1031·0 (912·5 to 1161·6)	−4·2% (−16·2 to 9·0)
Intracerebral haemorrhage	84·5% (81·1 to 87·7)	1·02	8·9% (3·6 to 18·4)	463·3 (401·0 to 535·2)	−47·4% (−55·4 to −35·9)
Subarachnoid haemorrhage	77·2% (72·5 to 81·8)	1·07	26·4% (10·7 to 56·8)	72·7 (60·5 to 89·5)	−53·1% (−66·3 to −27·2)
Neurological disorders*	11·2% (4·5 to 22·3)	0·69	25·9% (13·4 to 41·8)	152·6 (56·5 to 371·9)	20·2% (7·5 to 39·9)
Alzheimer's disease and other dementias	39·9% (26·4 to 54·7)	0·71	20·3% (9·2 to 30·5)	154·7 (58·8 to 375·1)	19·0% (6·7 to 37·7)
Parkinson's disease	−9·1% (−13·4 to −4·8)	0·10	−19·7% (−30·0 to −9·3)	−7·6 (−11·5 to −4·0)	−19·0% (−31·8 to −1·7)
Idiopathic epilepsy	2·1% (1·4 to 3·1)	0·28	11·8% (−16·0 to 45·8)	3·4 (1·9 to 5·5)	−17·0% (−44·5 to 21·7)
Multiple sclerosis	10·8% (7·8 to 14·5)	0·18	−12·0% (−28·2 to 5·0)	2·2 (1·5 to 3·0)	−6·7% (−28·5 to 8·8)
**Metabolic risk factors**†					
Stroke	73·9% (67·6 to 80·1)	1·04	18·3% (12·1 to 26)	1349·7 (1156 to 1552·3)	−19·6% (−29·1 to −6·3)
Ischaemic stroke	75·1% (66·7 to 84·3)	1·03	12·7% (8·0 to 18·8)	888·8 (753·9 to 1040·5)	2·8% (−11·2 to 18)
Intracerebral haemorrhage	72·9% (66·0 to 78·6)	1·05	19·0% (11·2 to 31·9)	399·9 (339·2 to 470·1)	−42·6% (−51·5 to −29·4)
Subarachnoid haemorrhage	64·7% (57·3 to 71·4)	1·11	39·2% (19·4 to 78·0)	60·9 (49·0 to 76·6)	−48·4% (−63·2 to −19·4)
Neurological disorders*	8·2% (2·8 to 17·4)	1·07	55·3% (38·6 to 85)	111·7 (34·2 to 279·7)	48·4% (31·4 to 80·4)
Alzheimer's disease and other dementias	28·8% (14·7 to 45·8)	1·15	50·2% (35·7 to 76·7)	111·7 (34·2 to 279·7)	48·4% (31·4 to 80·4)
**High systolic blood pressure**					
Stroke	52·8% (45·7 to 59·4)	1·05	10·5% (4·2 to 18·4)	965·0 (800·6 to 1143·2)	−24·9% (−34·3 to −12·0)
Ischaemic stroke	51·3% (41·6 to 60·2)	1·05	8·4% (4·2 to 13·7)	607·8 (477·7 to 734·5)	−1·1% (−13·7 to 13·3)
Intracerebral haemorrhage	56·4% (47·4 to 64·3)	1·06	12·1% (5·3 to 23·8)	309·1 (247·8 to 378)	−45·9% (−54·5 to −33·5)
Subarachnoid haemorrhage	51·1% (42·6 to 59·1)	1·11	30·7% (11·8 to 67·3)	48·1 (37·3 to 61·4)	−51·5% (−65·5 to −23·8)
**High BMI**					
Stroke	33·8% (24·3 to 42·8)	1·11	37·9% (22·8 to 61·1)	618·0 (440·1 to 822·7)	−6·2% (−21·8 to 16·6)
Ischaemic stroke	27·3% (18·8 to 36·0)	1·14	50·1% (34·0 to 77·5)	323·4 (218·8 to 443·9)	37·1% (13·1 to 68·5)
Intracerebral haemorrhage	46·0% (34·5 to 56·7)	1·11	47·3% (29·8 to 76·7)	252·2 (182·5 to 329·8)	−28·8% (−42·6 to −7·3)
Subarachnoid haemorrhage	45·1% (34·0 to 55·2)	1·24	65·6% (37·8 to 115·7)	42·4 (30·6 to 56·2)	−38·1% (−55·5 to −5·0)
Neurological disorders*	5·3% (1·6 to 11·9)	1·17	55·7% (35·3 to 100·0)	72·6 (21·0 to 181·2)	48·7% (28·5 to 94·4)
Alzheimer's disease and other dementias	18·7% (8·2 to 33·1)	1·27	50·5% (31·6 to 90·9)	72·6 (21·0 to 181·2)	48·7% (28·5 to 94·4)
**High fasting plasma glucose**					
Stroke	27·6% (17·7 to 42·8)	1·04	98·2% (68·5 to 132·0)	504·9 (318·8 to 793·9)	34·8% (7·9 to 64·8)
Ischaemic stroke	29·9% (15·7 to 53·0)	1·01	83·6% (61·2 to 111·5)	353·8 (190·4 to 627·5)	67·7% (38·9 to 103·2)
Intracerebral haemorrhage	24·2% (15·6 to 33·9)	1·08	94·8% (68·2 to 126·8)	132·5 (81·9 to 191·0)	−5·9% (−23·4 to 18·3)
Subarachnoid haemorrhage	19·8% (12·9 to 28·3)	1·08	121·7% (80·7 to 199·7)	18·6 (11·4 to 27·2)	−18·1% (−41·7 to 32·6)
Neurological disorders*	3·5% (0·6 to 9·7)	0·94	66·6% (54·4 to 89·3)	48·3 (8·7 to 151·5)	59·1% (47·1 to 88·7)
Alzheimer's disease and other dementias	12·4% (3·0 to 25·9)	1·02	61·0% (52·2 to 77·0)	48·3 (8·7 to 151·5)	59·1% (47·1 to 88·7)
**High LDL cholesterol**					
Stroke	15·0% (9·1 to 24·8)	1·13	45·8% (34·3 to 56·5)	274·7 (160·6 to 448·1)	−0·9% (−14 to 13·7)
Ischaemic stroke	23·2% (13·9 to 38·2)	1·08	8·5% (5·0 to 14·1)	274·7 (160·6 to 448·1)	−0·9% (−14 to 13·7)
**Kidney dysfunction**					
Stroke	11·0% (8·7 to 13·3)	1·12	51·9% (38·8 to 65·2)	200·8 (155·6 to 247·9)	3·2% (−12·2 to 21·1)
Ischaemic stroke	12·5% (9·2 to 15·7)	1·08	38·8% (28·4 to 47·9)	148·5 (107·9 to 190·0)	26·7% (6·0 to 45·3)
Intracerebral haemorrhage	9·5% (7·8 to 11·5)	1·21	40·2% (28·7 to 58·8)	52·3 (41·0 to 64·9)	−32·4% (−42·9 to −15·8)
**Behavioural risk factors**‡					
Meningitis	6·8% (5·2 to 9·0)	0·75	−10·3% (−35·5 to 22·9)	4·8 (3·4 to 6·7)	−78·3% (−85·5 to −67·4)
Encephalitis	3·7% (2·1 to 5·9)	0·81	8·8% (−31·6 to 74·9)	1·5 (0·9 to 2·5)	−14·5% (−51·5 to 51·4)
Stroke	34·0% (29·6 to 38·9)	0·70	1·3% (−4·8 to 7·5)	621·8 (518·0 to 746·8)	−31·1% (−39·8 to −19·7)
Ischaemic stroke	35·3% (29·2 to 42·0)	0·72	−2·5% (−6·4 to 0·7)	418·1 (335·0 to 521·4)	−11·0% (−23·6 to 1·1)
Intracerebral haemorrhage	32·1% (27·9 to 37·1)	0·65	−1·4% (−7·4 to 6·5)	176·3 (144·0 to 214·4)	−52·4% (−59·6 to −42·2)
Subarachnoid haemorrhage	29·1% (24·7 to 33·9)	0·75	17·3% (1·5 to 44)	27·4 (21·5 to 36·3)	−56·5% (−68·5 to −33·2)
Neurological disorders*	4·2% (1·6 to 8·7)	0·16	−5·5% (−15·0 to 5·0)	57·4 (21·1 to 137·7)	−9·8% (−18·9 to 4·0)
Alzheimer's disease and other dementias	15·4% (9·9 to 20·7)	0·15	−9·8% (−16·6 to −3·1)	59·5 (23·6 to 138·6)	−10·8% (−19·1 to 0·9)
Parkinson's disease	−9·1% (−13·4 to −4·8)	0·10	−19·7% (−30·0 to −9·3)	−7·6 (−11·5 to −4·0)	−19·0% (−31·8 to −1·7)
Idiopathic epilepsy	2·1% (1·4 to 3·1)	0·28	11·8% (−16·0 to 45·8)	3·4 (1·9 to 5·5)	−17·0% (−44·5 to 21·7)
Multiple sclerosis	10·8% (7·8 to 14·5)	0·18	−12·0% (−28·2 to 5·0)	2·2 (1·5 to 3·0)	−6·7% (−28·5 to 8·8)
**Tobacco**					
Stroke	16·9% (15·6 to 18·1)	0·38	−4·1% (−10·2 to 2·0)	307·8 (267·1 to 351)	−34·8% (−44·0 to −23·9)
Ischaemic stroke	15·6% (14·4 to 16·8)	0·36	−7·0% (−12·4 to −1·8)	184·4 (161·0 to 210·0)	−15·1% (−31·8 to −2·3)
Intracerebral haemorrhage	19·4% (17·9 to 21·0)	0·42	1·5% (−6·1 to 9·6)	106·5 (90·3 to 125·1)	−51·0% (−58·9 to −40·7)
Subarachnoid haemorrhage	18·0 % (16·1 to 20·1)	0·50	21·3% (0·3 to 50·2)	16·9 (14·1 to 21·4)	−55·0% (−67·9 to −29·2)
Neurological disorders*	3·9% (1·4 to 8·5)	0·16	−4·9% (−15·0 to 7·7)	54·0 (17·8 to 133·9)	−9·3% (−18·6 to 5·1)
Alzheimer's disease and other dementias	15·4% (9·9 to 20·7)	0·15	−9·8% (−16·6 to −3·1)	59·5 (23·6 to 138·6)	−10·8% (−19·1 to 0·9)
Parkinson's disease	−9·1% (−13·4 to −4·8)	0·10	−19·7% (−30·0 to −9·3)	−7·6 (−11·5 to −4·0)	−19·0% (−31·8 to −1·7)
Multiple sclerosis	10·8% (7·8 to 14·5)	0·18	−12·0% (−28·2 to 5·0)	2·2 (1·5 to 3·0)	−6·7% (−28·5 to 8·8)
**Smoking**					
Stroke	13·5% (12·5 to 14·5)	0·22	−4·3% (−11·5 to 2·9)	246·0 (214·2 to 280·1)	−34·9% (−44·3 to −23·8)
Ischaemic stroke	12·4% (11·5 to 13·4)	0·21	−8·0% (−14·8 to −1·1)	147·0 (128·6 to 166·4)	−16·0% (−32·2 to −2·9)
Intracerebral haemorrhage	15·6% (14·3 to 16·9)	0·24	2·0% (−6·8 to 10·9)	85·5 (72·4 to 100·0)	−50·7% (−59·1 to −40·5)
Subarachnoid haemorrhage	14·2% (12·7 to 16·0)	0·31	23·9% (−1·7 to 60·1)	13·4 (11·2 to 17·2)	−54·1% (−67·6 to −26·6)
Neurological disorders*	3·9% (1·4 to 8·5)	0·16	−4·9% (−15·0 to 7·7)	54·0 (17·8 to 133·9)	−9·3% (−18·6 to 5·1)
Alzheimer's disease and other dementias	15·4% (9·9 to 20·7)	0·15	−9·8% (−16·6 to −3·1)	59·5 (23·6 to 138·6)	−10·8% (−19·1 to 0·9)
Parkinson's disease	−9·1% (−13·4 to −4·8)	0·10	−19·7% (−30·0 to −9·3)	−7·6 (−11·5 to −4·0)	−19·0% (−31·8 to −1·7)
Multiple sclerosis	10·8% (7·8 to 14·5)	0·18	−12·0% (−28·2 to 5·0)	2·2 (1·5 to 3·0)	−6·7% (−28·5 to 8·8)
**Secondhand smoke**					
Stroke	3·9% (3·0 to 4·9)	1·49	−5·5% (−10·2 to 0·3)	71·9 (53·5 to 91·9)	−35·7% (−43·7 to −24·8)
Ischaemic stroke	3·6% (2·7 to 4·5)	1·38	−4·9% (−8·9 to −0·2)	42·9 (31·6 to 54·7)	−13·2% (−26·0 to −0·1)
Intracerebral haemorrhage	4·5% (3·4 to 5·7)	1·65	−1·9% (−8·4 to 8·2)	24·9 (18·1 to 32·3)	−52·6% (−60·2 to −41·9)
Subarachnoid haemorrhage	4·4% (3·3 to 5·6)	1·90	11·3% (−6·0 to 42·8)	4·1 (2·9 to 5·6)	−58·5% (−70·5 to −35·3)
**Child and maternal malnutrition**					
Meningitis	6·8% (5·2 to 9·0)	0·75	−10·3% (−35·5 to 22·9)	4·8 (3·4 to 6·7)	−78·3% (−85·5 to −67·4)
Encephalitis	3·7% (2·1 to 5·9)	0·81	8·8% (−31·6 to 74·9)	1·5 (0·9 to 2·5)	−14·5% (−51·5 to 51·4)
**Low birthweight and short gestation**					
Meningitis	6·8% (5·2 to 9·0)	0·75	−10·3% (−35·5 to 22·9)	4·8 (3·4 to 6·7)	−78·3% (−85·6 to −67·5)
Encephalitis	3·7% (2·1 to 5·9)	0·81	8·8% (−31·6 to 74·9)	1·5 (0·9 to 2·5)	−15·1% (−52·3 to 50·9)
**Short gestation**					
Meningitis	4·8% (3·6 to 6·3)	0·78	1·8% (−26·9 to 40·4)	3·4 (2·4 to 4·7)	−75·3% (−83·4 to −62·9)
Encephalitis	2·7% (1·5 to 4·4)	0·84	23·6% (−22 to 100·5)	1·1 (0·6 to 1·8)	−2·9% (−45·9 to 69·7)
**Low birthweight**					
Meningitis	6·5% (4·9 to 8·6)	0·74	−10·7% (−36·1 to 22·6)	4·5 (3·2 to 6·4)	−78·3% (−85·6 to −67·5)
Encephalitis	3·5% (2·0 to 5·6)	0·80	8·1% (−32·0 to 75·9)	1·4 (0·9 to 2·4)	−15·1% (−52·3 to 50·9)
**Dietary risk factors**					
Stroke	16·7% (12·6 to 21·5)	0·98	−1·7% (−10·2 to 6·8)	304·6 (223·3 to 400·8)	−33·2% (−42·5 to −21·5)
Ischaemic stroke	17·4% (12·1 to 22·4)	0·96	−4·1% (−8·8 to 0·0)	206·1 (140·3 to 270·9)	−12·5% (−23·8 to −0·8)
Intracerebral haemorrhage	15·5% (10·1 to 21·3)	1·00	−6·1% (−14·1 to 3·6)	84·9 (54·4 to 121·0)	−54·6% (−62·1 to −44·9)
Subarachnoid haemorrhage	14·3% (9·2 to 19·9)	1·12	11·1% (−5·3 to 39·6)	13·5 (8·3 to 20·3)	−58·8% (−70·3 to −36·6)
**Diet high in red meat**					
Stroke	3·6% (1·4 to 5·5)	0·99	1·4% (−21·0 to 28·5)	66·0 (25·9 to 102·1)	−30·9% (−48·0 to −10·0)
Ischaemic stroke	3·2% (0·6 to 4·9)	0·98	4·4% (−0·5 to 8·9)	38·3 (6·5 to 61·1)	−4·6% (−18·1 to 9·4)
Intracerebral haemorrhage	4·3% (0·7 to 6·8)	1·02	4·1% (−5·9 to 14·4)	23·5 (3·7 to 39·4)	−49·6% (−58 to −38·1)
Subarachnoid haemorrhage	4·4% (0·7 to 7·0)	1·10	28·5% (11·0 to 58·4)	4·1 (0·6 to 6·9)	−52·3% (−65·9 to −26·6)
**Diet high in sodium**					
Stroke	1·8% (0·3 to 7·2)	0·51	4·4% (−7·4 to 30·0)	33·6 (5·8 to 133·3)	−28·8% (−40·4 to −8·7)
Ischaemic stroke	1·7% (0·3 to 6·7)	0·52	2·9% (−11·5 to 25·2)	20·3 (3·8 to 80·5)	−5·9% (−25·7 to 18·8)
Intracerebral haemorrhage	2·1% (0·3 to 8·4)	0·51	8·6% (−5·9 to 39·0)	11·6 (1·7 to 46·7)	−47·4% (−57·9 to −29·6)
Subarachnoid haemorrhage	1·9% (0·3 to 7·4)	0·55	29·2% (−0·6 to 75·1)	1·8 (0·3 to 6·9)	−52·0% (−66·6 to −18·5)
**Diet low in fibre**					
Stroke	2·0% (0·5 to 3·6)	1·06	−5·9% (−13·1 to 6·6)	36·2 (9·2 to 66·7)	−36·1% (−44·0 to −23·8)
Ischaemic stroke	1·8% (0·5 to 3·3)	1·04	−10·2% (−16·2 to 0·3)	21·2 (5·6 to 39·0)	−18·2% (−28·1 to −4·8)
Intracerebral haemorrhage	2·4% (0·6 to 4·3)	1·10	2·7% (−7·4 to 19·7)	13·1 (3·1 to 23·9)	−50·5% (−58·7 to −37·9)
Subarachnoid haemorrhage	2·1% (0·5 to 4·0)	1·28	21·5% (3·3 to 52·2)	2·0 (0·5 to 4·2)	−54·9% (−65·7 to −32·7)
**Diet low in fruits**					
Stroke	3·9% (2·0 to 5·9)	1·08	−22·0% (−35·9 to −8·3)	70·5 (35·4 to 112·5)	−47·0% (−58·1 to −34·3)
Ischaemic stroke	2·9% (0·8 to 5·3)	1·08	−15·6% (−21·4 to −6·2)	34·1 (9·6 to 63·6)	−23·1% (−34·1 to −9·1)
Intracerebral haemorrhage	5·7% (2·9 to 9·5)	1·11	−13·3% (−20·5 to −3·7)	31·5 (15·9 to 54·1)	−58·1% (−64·9 to −48·3)
Subarachnoid haemorrhage	5·2% (2·8 to 8·4)	1·31	−1·9% (−16·8 to 23·2)	4·9 (2·4 to 8·7)	−63·5% (−73·3 to −44·2)
**Diet low in vegetables**					
Stroke	1·7% (0·7 to 2·7)	1·07	−28·8% (−41·7 to −7·6)	31·1 (13·4 to 50·9)	−51·6% (−61·6 to −36·8)
Ischaemic stroke	1·3% (0·3 to 2·3)	1·00	−25·1% (−32·2 to −4·4)	15·4 (4·0 to 27·6)	−31·5% (−41·6 to −11·6)
Intracerebral haemorrhage	2·5% (0·7 to 4·8)	1·17	−21·1% (−31·9 to −2·3)	13·6 (3·5 to 26·9)	−61·9% (−69·4 to −50·7)
Subarachnoid haemorrhage	2·2% (0·6 to 4·3)	1·31	−4·5% (−23·1 to 33·0)	2·1 (0·5 to 4·4)	−64·8% (−73·9 to −44·4)
**Diet low in whole grains**					
Stroke	5·3% (2·6 to 7·0)	1·06	35·5% (23·1 to 42·8)	96·7 (46·5 to 131·4)	−8·0% (−19·2 to 3·7)
Ischaemic stroke	8·2% (4·0 to 10·7)	1·01	0·9% (−2·9 to 3·4)	96·7 (46·5 to 131·4)	−8·0% (−19·2 to 3·7)
**Low physical activity**					
Stroke	5·0% (1·1 to 11·2)	1·12	48·9% (35·5 to 63)	91·7 (18·8 to 203·4)	1·2% (−11·8 to 16·0)
Ischaemic stroke	7·7% (1·7 to 17·2)	1·07	10·7% (4·5 to 20·3)	91·7 (18·8 to 203·4)	1·2% (−11·8 to 16·0)
**Alcohol use**					
Stroke	0·4% (0·2 to 0·7)	0·00	−30·8% (−55·2 to −8·8)	7·7 (3·2 to 12·3)	−52·9% (−69·5 to −37·4)
Ischaemic stroke	0·0% (−0·2 to 0·3)	−0·72	−48·2% (−399·7 to 533·7)	0·4 (−2·4 to 3·3)	−52·7% (−370 to 468·5)
Intracerebral haemorrhage	1·3% (0·8 to 1·9)	0·20	−2·5% (−21·3 to 16·6)	7·3 (4·3 to 10·9)	−52·9% (−63·2 to −41·1)
Neurological disorders*	0·3% (0·1 to 0·5)	0·20	−13·2% (−39·9 to 23·5)	3·4 (1·9 to 5·5)	−17·0 % (−44·5 to 21·7)
Idiopathic epilepsy	2·1% (1·4 to 3·1)	0·28	11·8% (−16·0 to 45·8)	3·4 (1·9 to 5·5)	−17·0 % (−44·5 to 21·7)
**Environmental and occupational risk factors**§					
Meningitis	1·6% (1·2 to 2·2)	0·78	−10·2% (−39·3 to 27·6)	1·1 (0·8 to 1·6)	−78·3% (−86·3 to −65·8)
Encephalitis	0·8% (0·5 to 1·4)	0·82	8·3% (−36·9 to 85·9)	0·4 (0·2 to 0·6)	−14·9% (−54·3 to 56·2)
Stroke	37·5% (34·5 to 40·7)	0·97	−2·2% (−6·9 to 3·9)	685·0 (590·6 to 792·8)	−33·5% (−41·7 to −22·2)
Ischaemic stroke	36·4% (33·4 to 39·7)	0·95	−2·3% (−6·4 to 1·9)	430·7 (370·7 to 494·6)	−10·9% (−24·0 to 2·5)
Intracerebral haemorrhage	40·0% (36·9 to 43·3)	1·00	−2·0% (−8·2 to 7·1)	219·4 (184·6 to 260·2)	−52·7% (−60·4 to −41·9)
Subarachnoid haemorrhage	37·1% (33·5 to 40·8)	1·07	12·8% (0·2 to 36·4)	35·0 (27·8 to 44·8)	−58·2% (−69·6 to −33·8)
**Air pollution**					
Meningitis	1·6% (1·2 to 2·2)	0·78	−10·2% (−39·3 to 27·6)	1·1 (0·8 to 1·6)	−78·3% (−86·3 to −65·8)
Encephalitis	0·8% (0·5 to 1·4)	0·82	8·3% (−36·9 to 85·9)	0·4 (0·2 to 0·6)	−14·9% (−54·3 to 56·2)
Stroke	29·2% (26·5 to 32·1)	1·00	−2·5% (−8·3 to 5·1)	533·2 (450·2 to 622·2)	−33·5% (−41·7 to −22·2)
Ischaemic stroke	28·1% (25·3 to 31)	0·98	−2·7% (−8·3 to 2·3)	332·5 (281·9 to 387·2)	−10·9% (−24·0 to 2·5)
Intracerebral haemorrhage	31·6% (28·7 to 34·6)	1·04	−1·9% (−9·8 to 9·6)	173·3 (142·8 to 209·6)	−52·7% (−60·4 to −41·9)
Subarachnoid haemorrhage	29·1% (25·8 to 32·3)	1·14	16·4% (−0·1 to 47·7)	27·4 (21·4 to 35·1)	−58·2% (−69·6 to −33·8)
**Particulate matter pollution**					
Meningitis	1·6% (1·2 to 2·2)	0·78	−10·2% (−39·3 to 27·6)	1·1 (0·8 to 1·6)	−78·3% (−86·3 to −65·8)
Encephalitis	0·8% (0·5 to 1·4)	0·82	8·3% (−36·9 to 85·9)	0·4 (0·2 to 0·6)	−14·9% (−54·3 to 56·2)
Stroke	29·2% (26·5 to 32·1)	1·00	−2·5% (−8·3 to 5·1)	533·2 (450·2 to 622·2)	−33·7% (−42·6 to −21·4)
Ischaemic stroke	28·1% (25·3 to 31)	0·98	−2·7% (−8·3 to 2·3)	332·5 (281·9 to 387·2)	−11·3% (−24·9 to 2·1)
Intracerebral haemorrhage	31·6% (28·7 to 34·6)	1·04	−1·9% (−9·8 to 9·6)	173·3 (142·8 to 209·6)	−52·6% (−60·5 to −40·9)
Subarachnoid haemorrhage	29·1% (25·8 to 32·3)	1·14	16·4% (−0·1 to 47·7)	27·4 (21·4 to 35·1)	−56·9% (−69·5 to −31·9)
**Ambient particulate matter pollution**					
Meningitis	0·9% (0·6 to 1·3)	0·79	17·5% (−20·5 to 75·9)	0·6 (0·4 to 0·9)	−71·5% (−81·8 to −54·2)
Encephalitis	0·7% (0·2 to 1·3)	0·80	32·2% (−30·3 to 152·9)	0·3 (0·1 to 0·5)	3·5% (−48·6 to 109·4)
Stroke	25·5% (22·5 to 28·5)	0·94	51·1% (32·0 to 79·4)	466·4 (391·1 to 553·9)	2·8% (−14·7 to 26·1)
Ischaemic stroke	25·0% (22·1 to 27·9)	0·94	43·0% (26·5 to 68·6)	296·5 (249·2 to 349·1)	30·6% (8·7 to 58·7)
Intracerebral haemorrhage	26·8% (23·6 to 30·0)	0·94	58·5% (35·5 to 88·9)	147·0 (120·3 to 178·4)	−23·4% (−38·7 to −3·4)
Subarachnoid haemorrhage	24·4% (20·8 to 27·7)	1·05	77·7% (47 to 132·4)	23·0 (18·5 to 28·8)	−33·7% (−52·9 to 6·2)
**Household air pollution from solid fuels**					
Meningitis	0·7% (0·4 to 1·1)	0·75	−31·9% (−63·3 to 19·9)	0·5 (0·3 to 0·8)	−83·5% (−91·5 to −68·6)
Encephalitis	0·2% (0·1 to 0·3)	0·93	−39·2% (−71·4 to 29·4)	0·1 (0·0 to 0·2)	−51·1% (−78·0 to 26·9)
Stroke	3·7% (2·5 to 5·0)	1·57	−71·9% (−76·9 to −66·1)	66·8 (44·8 to 93·1)	−80·9% (−84·8 to −76·1)
Ischaemic stroke	3·0% (2·1 to 4·3)	1·45	−73·2% (−77·9 to −67·4)	36·1 (24·1 to 52·9)	−75·6% (−80·2 to −69·8)
Intracerebral haemorrhage	4·8% (3·3 to 6·5)	1·76	−68·6% (−74·3 to −62·6)	26·4 (17·6 to 37·1)	−84·9% (−88·3 to −80·3)
Subarachnoid haemorrhage	4·6% (2·5 to 7·1)	1·83	−58·7% (−73·6 to −46·3)	4·4 (2·1 to 7·5)	−84·7% (−88·8 to −77·9)
**Non-optimal temperature**					
Stroke	7·1% (5·0 to 9·1)	0·97	0·1% (−12·5 to 18·5)	129·1 (90·0 to 168·5)	−31·9% (−44·4 to −17·3)
Ischaemic stroke	6·8% (4·8 to 8·8)	0·96	−0·4% (−12·5 to 15·6)	80·3 (56·2 to 104·7)	−9·2% (−28·0 to 9·5)
Intracerebral haemorrhage	7·6% (5·3 to 9·8)	1·00	4·0% (−10·4 to 26·2)	41·7 (28·7 to 55·6)	−49·8% (−59·0 to −36·9)
Subarachnoid haemorrhage	7·5% (5·4 to 9·6)	0·96	4·9% (−10·2 to 29·3)	7·0 (4·9 to 9·7)	−61·0% (−71·5 to −33·3)
**High temperature**					
Stroke	1·6% (0·5 to 3·1)	0·88	15·9% (−40·6 to 99·9)	29·4 (10·2 to 59·3)	−21·2% (−59·9 to 33·6)
Ischaemic stroke	1·5% (0·5 to 3·0)	0·87	21·8% (−30·5 to 127·1)	17·9 (6·1 to 36·2)	11·1% (−39·8 to 103·6)
Intracerebral haemorrhage	1·8% (0·6 to 3·6)	0·90	17·5% (−43·1 to 89·1)	10·0 (3·5 to 20·3)	−43·2% (−71·3 to −8·4)
Subarachnoid haemorrhage	1·5% (0·6 to 3·0)	0·93	11·0% (−53·8 to 89·5)	1·5 (0·5 to 3·1)	−58·5% (−83·3 to −17·7)
**Low temperature**					
Stroke	5·5% (3·5 to 7·7)	0·99	−3·5% (−12·9 to 5·4)	100·9 (62·0 to 140·7)	−34·4% (−43·8 to −23·6)
Ischaemic stroke	5·3% (3·4 to 7·5)	0·98	−5·1% (−15·6 to 2·4)	63·1 (39·4 to 87·8)	−13·4% (−28·1 to 0·3)
Intracerebral haemorrhage	5·9% (3·6 to 8·3)	1·03	0·5% (−9·4 to 12·3)	32·1 (19·1 to 45·9)	−51·5% (−59·1 to −41·7)
Subarachnoid haemorrhage	6·0% (4·0 to 8·3)	0·96	3·5% (−9·8 to 29·6)	5·6 (3·5 to 8·0)	−61·6% (−72·0 to −35·0)
**Other environmental risks**					
Stroke	5·2% (3·3 to 7·3)	0·72	−12·9% (−22·2 to −5·2)	95·5 (57·8 to 137·7)	−40·8% (−50·5 to −30·4)
Ischaemic stroke	5·2% (3·2 to 7·4)	0·69	−7·1% (−13·5 to −2·2)	61·3 (36·2 to 89·0)	−15·2% (−28·5 to −3·3)
Intracerebral haemorrhage	5·4% (3·2 to 8·0)	0·77	−19·1% (−29·6 to −9·5)	29·9 (17·2 to 44·8)	−60·9% (−68·6 to −52·2)
Subarachnoid haemorrhage	4·6% (2·5 to 7·1)	0·80	−4·7% (−20·6 to 19·1)	4·4 (2·1 to 7·3)	−64·8% (−74·1 to −46·8)
**Lead exposure**					
Stroke	5·2% (3·3 to 7·3)	0·72	−12·9% (−22·2 to −5·2)	95·5 (57·8 to 137·7)	−40·8% (−50·5 to −30·4)
Ischaemic stroke	5·2% (3·2 to 7·4)	0·69	−7·1% (−13·5 to −2·2)	61·3 (36·2 to 89·0)	−15·2% (−28·5 to −3·3)
Intracerebral haemorrhage	5·4% (3·2 to 8·0)	0·77	−19·1% (−29·6 to −9·5)	29·9 (17·2 to 44·8)	−60·9% (−68·6 to −52·2)
Subarachnoid haemorrhage	4·6% (2·5 to 7·1)	0·80	−4·7% (−20·6 to 19·1)	4·4 (2·1 to 7·3)	−64·8% (−74·1 to −46·8)

All 22 risk factors within four Levels of classification are listed in this table, as well as aggregates of these risk factors within the Levels above them in the risk factors hierarchy. Level 1 and Level 2 risk factors in bold. Data in parentheses are 95% uncertainty intervals. All data are provided to one decimal place, except female to male ratio, which is provided to two decimal places. Percentages of DALYs are not mutually exclusive: the sum of percentages of DALYs in the columns exceeds the totals for all risk factors combined because of overlap between various risk factors. Risk factors with insufficient data and neurological conditions with insufficient data for each risk factor are not reported here. The crude sum of population attributable fraction of the risk factors might exceed 100% because the effects of many of these risk factors are mediated partly or wholly through another risk factor or risk factors. DALY=disability-adjusted life-year. PM_2·5_=particulate matter with a diameter of <25 μm.

* Neurological disorders in the Global Burden of Disease Study 2019 include Alzheimer's disease and other dementias, Parkinson's disease, idiopathic epilepsy, multiple sclerosis, headache disorders (including migraine and tension-type headache), motor neuron disease, and other neurological disorders.

† Metabolic risk factors cluster includes high BMI, high fasting plasma glucose, high LDL cholesterol, high systolic blood pressure, and kidney dysfunction.

‡ Behavioural risk factors cluster includes tobacco (including tobacco smoke and secondhand smoke), dietary risks (diet high in sodium, diet low in fruits, diet low in vegetables, diet high in red meat, and diet low in whole grains), low physical activity, alcohol consumption, and child and maternal malnutrition (including low birthweight and short gestation).

§ Environmental risk factors cluster includes air pollution (including ambient PM_2·5_ pollution and household air pollution from solid fuels), non-optimal temperature (including low ambient temperature and high ambient temperature), and other environmental risks (including lead exposure).

In north Africa and the Middle East in 2019, the risk factors that attributed the highest proportion of DALYs for stroke were high systolic blood pressure (52·8% [95% UI 45·7 to 59·4] of total stroke DALYs), high BMI (33·8% [24·3 to 42·8]), air pollution (29·2% [26·5 to 32·1]), high fasting plasma glucose (27·6% [17·7 to 42·8]), tobacco (16·9% [15·6 to 18·1]), and dietary risk factors (16·7% [12·6 to 21·5]; table 2; appendix 4 pp 321–439). For dementias, risk factors that attributed the highest proportion of DALYs were metabolic risk factors (28·8% [14·7 to 45·8]), including high BMI (18·7% [8·3 to 33·1]) and high fasting plasma glucose (12·4% [3·0 to 25·9]), and behavioural risk factors (15·4% [9·9 to 20·7]), including tobacco (15·4% [9·9 to 20·7]). These proportions were lower for other neurological conditions. Behavioural risk factors overall, and tobacco use and smoking, were associated with a decreased risk of Parkinson's disease (–9·1% [–13·4 to –4·8]).

**Figure 3:**
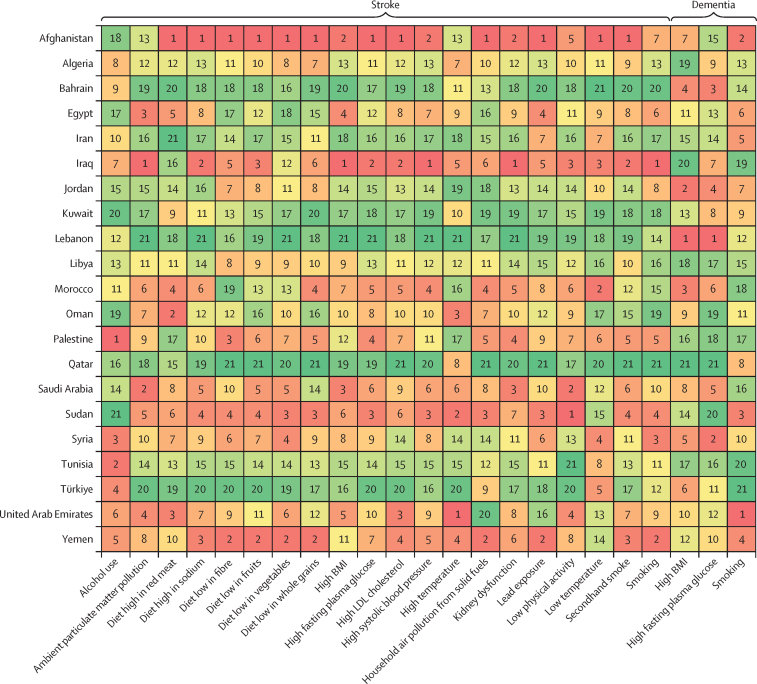
Ranks of age-standardised DALY rates for stroke and dementia, per 100 000 population, attributable to modifiable risk factors in countries in north Africa and the Middle East, 2019 Ranks range from 1 (dark red) with the highest rate to 21 (dark green) with the lowest rate. Stroke includes ischaemic stroke, intracerebral haemorrhage, and subarachnoid haemorrhage. Dementia includes Alzheimer's disease and other dementias. DALYs=disability-adjusted life-years.

Among the 21 countries in north Africa and the Middle East, Iraq (90·3% [95% UI 87·5–93·0]), Afghanistan (89·6% [86·8–92·3]), Saudi Arabia (89·6% [86·4–92·5]), Sudan (89·3% [86·2–92·2]), and the UAE (89·3% [85·6–92·7]) had the highest proportion of stroke DALYs attributable to modifiable risk factors in 2019 (figure 3; appendix 4 pp 276–320). Similarly, Qatar (53·5% [34·4–71·6]), Lebanon (51·1% [36·7–64·9]), Bahrain (50·2% [33·0–68·0]), Kuwait (49·7% [33·6–65·4]), and the UAE (49·4% [32·3–66·4]) had the highest proportion of dementia DALYs attributable to modifiable risk factors (figure 3). The highest proportion of neurological DALYs was attributable to high systolic blood pressure (>60% of their individual risks) in Iraq, Sudan, and Morocco; high BMI (>46%) in Saudi Arabia, the UAE, and Kuwait; air pollution (>36%) in Afghanistan, Egypt, and Sudan; high fasting plasma glucose (>37%) in Palestine, Bahrain, and Qatar; and tobacco (>25%) in Lebanon, Jordan, Yemen, Iraq, and Türkiye (appendix 4 pp 276–439).

**Figure 4:**
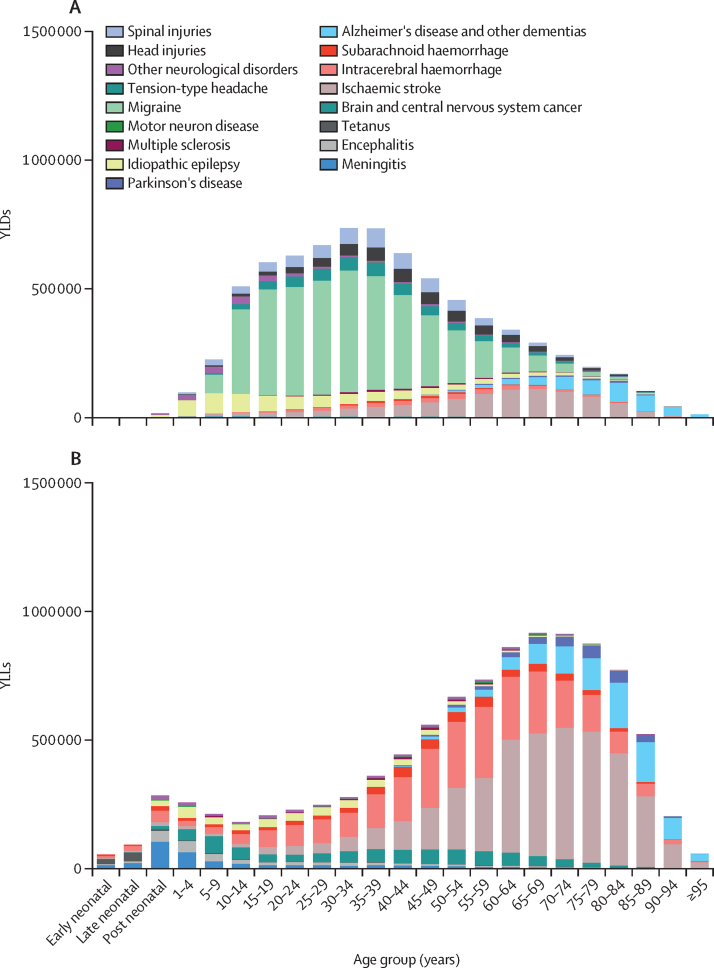
Burden of neurological diseases in north Africa and the Middle East, in YLDs (A) and YLLs (B), in 2019 We accounted for potential overlaps in health status and the causes depicted in this figure are distinct from each other. YLDs=years lived with disability. YLLs=years of life lost.

In 2019, most neurological YLDs happened before age 50 years and most neurological YLLs happened after age 50 years (figure 4; appendix 4 pp 440–46). 5·3 million (95% UI 4·6–6·0; 66·1%) of 7·9 million (7·1–8·9) DALYs due to stroke happened in people younger than 70 years (appendix 4 pp 447–53). In contrast, 950·2 thousand (425·0–2122·9; 78·7%) of 1208·1 thousand DALYS due to dementias (appendix 4 pp 454–60) and 195·9 thousand (172·8–246·3; 65·2%) of 300·7 thousand DALYs due to Parkinson's disease (appendix 4 pp 461–47) occurred in individuals aged 70 years and older. Approximately 205·6 thousand (49·8%) of 412·8 thousand DALYs due to meningitis (appendix 4 pp 468–74), 95·6 thousand (38·1%) of 251·0 thousand DALYs due to encephalitis (appendix 4 pp 475–81), and 62·7 thousand (71·7%) of 87·4 thousand DALYs due to tetanus (appendix 4 pp 482–88) happened within the first 5 years of life. 288·6 thousand (40·3%) of 706·3 thousand DALYs due to brain and CNS cancer occurred among people aged 15–49 years (appendix 4 pp 489–95). Most other neurological DALYs occurred in individuals younger than 50 years (appendix 4 pp 496–539), of which migraine was the main contributor (3·2 million [39·8%] of 8·1 million neurological DALYs), given its early onset, leading to a large burden.

In north Africa and the Middle East, stroke age-standardised DALY rates were slightly higher in females than males in 2019 (female to male ratio of 1·04 *vs* 0·76 globally; table 1; appendix 4 pp 105–42); the lowest ratio was observed in Kuwait (0·68). Among neurological conditions, those with a female to male ratio of more than 1 for their DALY rates included migraine (1·70), multiple sclerosis (1·58), tension-type headache (1·28), dementias (1·12), ischaemic stroke (1·09), encephalitis (1·09), and subarachnoid haemorrhage (1·08; appendix 4 pp 143–94). The burden of all other neurological conditions was higher in males than in females. Female to male ratios for DALY rates of dementias were lower in north Africa and the Middle East than globally (1·12 *vs* 1·19). The highest female to male ratios for dementia DALY rates were in Türkiye (1·17), Lebanon (1·16), Egypt (1·14), and Syria (1·14) . The burden of DALY rates due to Parkinson's disease was higher in males than in females overall in north Africa and the Middle East (female to male ratio 0·68), except in Egypt (1·05) and Qatar (1·03) where the DALY rate burden was higher in females. For multiple sclerosis, the female to male ratio of DALYs was much higher in Qatar than for the super-region overall (2·31 *vs* 1·58).

The incidence of head and spinal injuries was higher in males than females (female to male ratio of 0·58 for head injuries and 0·76 for spinal injuries; table 1). This ratio reversed among individuals aged 70 years and older (0·48 and 0·64 at <70 years for head and spinal injuries, respectively, and 1·09 and 1·17 at ≥70 years, respectively; appendix 4 pp 534–39).

## Discussion

In 2019, neurological conditions were the second leading cause of DALYs and deaths after cardiovascular diseases in north Africa and the Middle East, with 17·6 million DALYs and 441·1 thousand deaths, and had almost doubled since 1990 (12·4 million DALYs and 254·3 thousand deaths). Our findings show the ongoing transitions in ageing, increase in metabolic risk factors, and worsening of environmental risks, with high heterogeneity within and across countries (the wide 95% UIs in these countries reflect the heterogeneity of data sources). The burden of disease for stroke, dementia, brain and CNS cancer, and other neurological disorders are all increasing with time. The reduction of the burden of neuroinfectious conditions between 1990 and 2019 might mainly be due to vaccination, improved health and hygiene, and effective therapeutics, while the increase in the burden of some other neurological conditions might mainly be related to population growth and ageing,^10^, ^14^ and improved survival of fatal diseases such as brain and CNS cancer. Compared with other global super-regions, north Africa and the Middle East had the highest age-standardised DALY rates for dementias, Parkinson's disease, migraine, and the second highest rate for ischaemic stroke and brain and CNS cancer.

Neurological conditions affect a considerable number of individuals across a wide age range. With increasing life expectancy, the risk of developing a neurological pathology increases. Prevention and integrated management of these conditions are essential for optimal management of health care and are effective to reduce the burden of neurological conditions. Culture-specific interventions are required to improve general awareness and reduce the burden of modifiable risk factors at the population level. For instance, specific parks designated for women in Muslim countries could help facilitate their participation in sports activities and socialisation. The media can assist with national programmes to educate people and restaurants to reduce salt and sugar intake as well as red and processed meat and to prioritise life-course prevention strategies. Hypertension is highly prevalent and treatable with lifestyle changes and widely available drugs. Globally, only half of people with hypertension are aware they have the condition and less than half of them have their condition under control,^15^ providing substantial prevention potential.

The number of new patients with dementia and the related burden in north Africa and the Middle East increase by approximately 38% between 2010 and 2019, particularly in the UAE, Qatar, Jordan, and Bahrain, where DALY counts more than doubled, with all but Jordan being HICs. Most of this increase is due to population growth, ageing, and the contribution of increased diagnoses—more prominently in LMICs.^10^, ^16^ The age-standardised burden of DALYs of dementia in north Africa and the Middle East was higher than in all other super-regions. A deeper regional analysis of health status and lifestyle might shed light on the cause and underlying risk factors of dementia. The increasing dementia DALYs might be associated with higher PAFs and increasing trends of high fasting plasma glucose (PAF change of 61·0% [95% UI 52·2–77·0]) and high BMI (PAF change of 50·5% [31·6–90·9]) over the period 1990–2019 (table 2) and the increasing burden of stroke in north Africa and the Middle East, which is a condition that doubles the risk of dementia.^17^ On the basis of our findings, 39·9% (26·4–54·7) of dementias were potentially preventable. This finding implies an approximately 39% higher potential preventable proportion of dementias in north Africa and the Middle East than for the global population overall (154·7 *vs* 111·3 per 100 000 population). Additionally, the proportion of dementia DALYs attributed to metabolic risk factors increased by 50·2% (35·7–76·7) between 1990 and 2019, indicating substantial prevention potential. The GBD Dementia Forecasting study estimated a 166% increase in the prevalence of dementia, from 57·4 million patients globally in 2019 to 152·8 million in 2050.^18^ North Africa and the Middle East is expected to have the highest increase in prevalence (367%).^18^ These projections necessitate urgent, synergistic, and lifelong action plans for dementia prevention through new approaches.^19^, ^20^

A study based on GBD 2019 data^21^ analysed the burden of seven neurological conditions, comprising approximately 41% of the neurological burden covered in our study—eg, stroke, as the leading cause of neurological DALYs (45%) and deaths (71%) in north Africa and the Middle East with almost 8 million DALYs and 312 thousand deaths in 2019, was not included, but was analysed in our study in addition to nine more conditions.^21^ We found a decreasing trend in risk-attributed stroke DALYs from 1990 to 2019, suggesting effective primary prevention strategies and therapeutics are in place.^14^, ^19^ However, ischaemic stroke DALYs attributable to high fasting plasma glucose (68%) and high BMI (37%) increased, compared with a non-significant reduction globally (approximately –5% for both risks).

Increasing trends in age-standardised YLD rates from 2010 to 2019 due to brain and CNS cancer, Parkinson's disease, multiple sclerosis, ischaemic stroke, motor neuron disease, and dementia, as well as increasing incidence and prevalence of head and spinal injuries, motor neuron disease, Parkinson's disease, multiple sclerosis, and tension-type headache during 2010 to 2019, require individual health policies. Therefore, urgent implementation of population-wide primary prevention strategies and public education campaigns are warranted.^22^ Nevertheless, the decreasing trends in age-standardised rates of meningitis, tetanus, stroke, and idiopathic epilepsy DALYs, as well as incidence rates of head and spinal injuries, in most countries in north Africa and the Middle East during the past decade are encouraging.

While the burden of the majority of neurological conditions has decreased or showed no change in age-standardised DALY rates between 1990 and 2019, some conditions have had a significant increase in DALY count (eg, dementia, Parkinson's disease, headache disorders, multiple sclerosis, and ischaemic stroke). The increase in DALY count can mostly be attributed to population ageing and growth, but the decrease in rate is most likely because of improved health practices, better diagnostics, and effective treatments. However, the burden of YLDs, incidence, and prevalence of some conditions (eg, multiple sclerosis, motor neuron disease, Parkinson's disease, dementia, and ischaemic stroke) has increased both in rate and count, particularly during 2010 to 2019. This finding suggests that the risk of these sequelae has also increased and the risks are not solely related to population growth and ageing. To address this, further exploration of causal factors associated with these disorders is necessary. Implementing population-wide and individual-level interventions to reduce exposure to these risks is crucial. Our findings highlight a significant increase in the risk attributed DALY rate for metabolic risk factors (ie, high fasting plasma glucose and high BMI) for ischaemic stroke and dementia, as well as an increase in ambient particulate matter pollution associated with ischaemic stroke. Advances in medical technology and increased awareness have substantially improved the diagnosis and reporting of neurological disorders, such as multiple sclerosis, Parkinson's disease, and motor neuron disease. Additionally, improved management and care have extended the lifespan of individuals living with these conditions. Lifestyle modifications, environmental factors, and genetic predisposition might also influence the risk of developing these neurodegenerative conditions. As populations age, either due to improved survival rates, or enhanced treatment options, the number of YLDs related to these conditions has increased. Notably, diseases that typically have older age of onset (eg, dementia) had a larger increase in prevalence and YLDs counts. By contrast, diseases with younger age of onset (eg, multiple sclerosis, motor neuron disease, and Parkinson's disease) had a decrease in age-standardised rates. This decrease could be attributed to improved disease management, early interventions, or changes in risk factors.

Heterogeneity of data across north Africa and the Middle East poses challenges in estimating the burden and trends of medical conditions, and thus in developing and implementing effective prevention and intervention strategies. Some of the contributing factors to this heterogeneity could be that few population-based studies have been done and few standardised registries for neurological conditions are present in many countries in the region; different diagnostic criteria and classifications for neurological conditions; and sociocultural, socioeconomic, and environmental differences among countries in the super-region. Such societal and environmental differences between countries could lead to uneven distribution of resources, such as access to neurologists, required facilities, and access to health care in rural and urban regions, uneven representation of countries in north Africa and the Middle East, with some having more data and research on particular neurological conditions, and stigma around some neurological diagnoses associated with acknowledging and reporting neurological conditions.^23^, ^24^

Additional studies of missing risk factors using WHO STEPS guidance might provide more reliable information on the burden of neurological conditions attributable to modifiable risk factors and inform action plans at both high-risk and population levels throughout the region.^6^, ^25^ We partly addressed the missing data for dementia in the present study and in a previous a study^26^ by analysing the risk-attributed burden of stroke (and ischaemic heart disease) because dementia, stroke, and ischaemic heart disease share common risk factors. Despite efforts in collecting data and accounting for the biases in the analysis, some estimates depended on predictive covariates and geographical proximity, and thus interpretation of our findings should be done with caution. Still, this study might provide the best quality descriptive epidemiological evidence from this region to date, since it is under-represented in the literature.

The role of social (eg, income and social protection, education, food insecurity, and housing), historical (eg, wars and sanctions), and environmental factors (eg, polluted air and water) on the burden of neurological conditions should be considered in future studies. Geopolitical characteristics of countries in north Africa and the Middle East have resulted in many regional wars and conflicts, which might have harmed mental health and increased the incidence of new dementias, head and spinal injuries, and direct and indirect mortality and morbidity.^27^ Disparities in race and race-associated risk factors should also be incorporated into future analyses. As has been found for the COVID-19-related neurological burden, harmonised universal health records and data would improve accuracy of estimates and foster timely measures against unfavourable trends in countries in the region.

Advances in medical science can have contrasting effects on disease burden. While improved diagnostics and effective therapies increase DALYs, affordable therapeutics and preventive strategies decrease them. Vigilant monitoring of trends is essential to promptly implement evidence-based preventive, protective, and therapeutic measures. Across nations, peace, democracy, and eradication of poverty have a pivotal role in preventing neurological conditions and enhancing health-care systems. Nation-specific brain health programmes should prioritise promoting lifelong brain health and disease prevention, providing patient-centred care and support, adopting holistic multidisciplinary approaches, and ensuring robust support for therapeutic research and innovation.^19^

Our study has some limitations, including a scarcity of good-quality studies for most of the countries included;^6^, ^28^ missing data on rural and urban disparities; missing data for some neurological conditions (eg, secondary epilepsy and the long-term neurological consequences of neonatal disorders), the severity of neurological conditions, and risk factors; missing data on early childhood neurological conditions, such as cerebral palsy and autism disorders, that have been analysed by others;^29^, ^30^ an absence of data on refugees, armed conflicts, economic sanctions, poverty, and social unrest resulting in poor health-care access and data; a scarcity of data to address subnational variations; missing risk factors such as head injuries, physical inactivity, atrial fibrillation, depression, sleep disturbance, hypertension (for both dementia and Parkinson's disease), air pollution, alcohol use, hearing loss, social isolation, substance abuse, and diastolic hypertension; heterogeneity of data both within and across countries in north Africa and the Middle East for many neurological conditions; heterogeneity of data on alcohol use because of prohibition in many countries in the super-region; and changes in diagnostic guidelines during the past 30 years.^31^, ^32^ Furthermore, we were unable to pool numbers of new or prevalent cases, except for the aggregates reported.

### Conclusions

In 2019, stroke, migraine, and dementia were the primary contributors to the burden of neurological conditions in north Africa and the Middle East, and stroke and dementia were the leading causes of neurological deaths. The heightened potential for joint prevention of these two major conditions in north Africa and the Middle East, compared with global trends, underscores the need for coordinated, systematic, lifelong, multi-sectoral, and governmental interventions at local, national, and international levels. These efforts aim to pre-emptively mitigate or reduce the burden posed by these conditions by addressing them collectively.

Our research findings should prompt vigilance to temporal trends of neurological conditions in most countries of north Africa and the Middle East super-region, enabling us to anticipate and implement timely interventions. Based on our estimates, the challenge of neurological conditions in north Africa and the Middle East seems to be increasing more rapidly than the global average, while also presenting an opportunity to prioritise and implement essential preventive measures.

## Data sharing

To download the data used in the present study, please visit the Global Health Data Exchange GBD 2019 website.

## Declaration of interests

We declare no competing interests.

## Supplementary Materials

Persian translation of the abstract

Arabic translation of the abstract

Turkish translation of the abstract
