# Correction to *The burden of neurological conditions in north Africa and the Middle East, 1990–2019: a systematic analysis of the Global Burden of Disease Study 2019.* Lancet Glob Health 2024; 12: e960–82

**DOI:** 10.1016/j.langlo.2026.104086

**Published:** 2026-08-26

*GBD 2019 North Africa and the Middle East Neurology Collaborators. The burden of neurological conditions in north Africa and the Middle East, 1990–2019: a systematic analysis of the Global Burden of Disease Study 2019.* Lancet Glob Health *2024;* ***12:*** *e960–82*—In the first paragraph of the Results on page e967, the last sentence should read: “In north Africa and the Middle East in 2019, an estimated 441·1 thousand (347·2–598·4) neurological deaths occurred (14·2% of 3·1 million all-cause deaths in north Africa and the Middle East), as the second leading cause of deaths after cardiovascular diseases with approximately 984·5 thousand (872·6–1085·4) deaths (31·7% of 3·1 million all-cause deaths).” The first sentence of the second paragraph on page e969 should have read: “In 2019, 453·0 thousand (95% UI 331·6 to 604·0; 6·4%) of 7076·9 thousand (4997·9 to 9588·1) YLDs globally due to head injuries, and 564·2 thousand (289·6 to 1229·1; 9·1%) of 6200·1 thousand (4465·3 to 8156·2) YLDs globally due to spinal injuries happened in north Africa and the Middle East (appendix 4 pp 254–75).” These corrections have been made as of Aug 26, 2026.

