# Supplementary material for: The burden of neurological conditions in north Africa and the Middle East, 1990–2019: a systematic analysis of the Global Burden of Disease Study 2019
Source: Lancet Glob Health. 2024 Apr 8;12(6):e960–82. doi: 10.1016/S2214-109X(24)00093-7 (PMC11099299; doi:10.1016/S2214-109X(24)00093-7)
Supplement: Persian translation of the abstract [file mmc1.pdf]

THE LANCET
Global Health

Supplementary appendix 1

This translation in Persian was submitted by the authors and we reproduce it as supplied. It has not been peer reviewed. The Lancet's editorial processes have only been applied to the original in English, which should serve as reference for this manuscript.

این ترجمه به زبان فارسی توسط نویسندگان ارسال شده است و ما آن را همانطور که تهیه می شود بازتولید می کنیم .مورد
فقط در اصل به زبان انگلیسی اعمال شده است ، که باید به عنوان Lancet بررسی قرار نگرفته است .مراحل تحریریه
مرجع برای این نسخه خطی باشد.

Supplement to: GBD 2019 North Africa and the Middle East Neurology Collaborators. The burden of neurological conditions in north Africa and the Middle East, 1990–2019: a systematic analysis of the Global Burden of Disease Study 2019. *Lancet Glob Health* 2024; published online April 8. https://doi.org/10.1016/S2214-109X(24)00093-7.

Persian summary

بار بیماری‌های مغز و اعصاب در شمال آفریقا و خاورمیانه، 1990-2019: تجزیه و تحلیل نظام‌مند از مطالعه بار جهانی بیماری 2019

همکاران مطالعه بار جهانی بیماری 2019 در ناحیه شمال آفریقا و خاورمیانه در زمینه علم مغز و اعصاب*

مقدمه: بار بیماری‌های مغز و اعصاب در شمال آفریقا و خاورمیانه در حال افزایش است. هدف ما ارزیابی تغییرات در بار بیماری‌های مغز و اعصاب در این منطقه جهت کمک به تصمیم گیری‌های آینده است.

روش مطالعه: در این تجزیه و تحلیل داده های مطالعه 2019 بار جهانی بیماری‌ها، آسیب ها و عوامل خطر (GBD 2019)، ما روند سال‌های از دست رفته به دلیل ناتوانی جسمی یا مرگ زودرس (DALYs)، موارد مرگ، موارد جدید بیماری و موارد شایع 14 بیماری مغز و اعصاب عمده و هشت زیر گروه را در 21 کشور در شمال آفریقا و منطقه خاورمیانه بررسی کردیم. همچنین، ما DALYs مغز و اعصاب را در زمینه 22 عامل خطر بالقوه قابل اصلاح در چهار سطح دسته بندی در طول دوره 1990–2019 ارزیابی کردیم. ما از یک رویکرد مدل‌سازی بیزی (Bayesian) استفاده کردیم و فواصل عدم قطعیت 95٪ (UIs) را برای تخمین‌های نهایی بر اساس صدک‌های 2 و 97 ام از 1000 توزیع پسین تولید کردیم.

یافته‌ها: در سال 2019، 441·1 هزار (UI 347·2–598·4) مرگ و 17·6 میلیون (12·5-24·7) DALYs به علت بیماری‌های مغز و اعصاب در شمال آفریقا و خاورمیانه وجود داشت. علت‌های اصلی DALYs بیماری‌های مغز و اعصاب، سکته مغزی، میگرن و بیماری آلزایمر و سایر علل زوال عقل (به صورت خلاصه، زوال عقل) بود. در شمال آفریقا و خاورمیانه در سال 2019، بر اساس نرخ DALYs استاندارد سنی، 85·8% (89·1–82·6) سکته مغزی و 39·9% (54·7%–26·4) زوال عقل مربوط به عوامل خطر قابل اصلاح بود. شمال آفریقا و خاورمیانه بالاترین نرخ DALYs استاندارد سنی را، در میان مناطق جهانی، در زمینه زوال عقل (387·0 [172·0–848·5])، بیماری پارکینسون (84.4 [74·7–103·2]) و میگرن (601·4 [107·0–1371·8]) داشتند. بین سال‌های 1990 تا 2019، کاهش نرخ DALYs استاندارد سنی در زمینه بیماری‌های منژیت (75·8%- [81·1- تا 69·5-])، کزاز (88·2%- [93·9- تا 76·1-]) و سکته مغزی (32·0%- [39·1- تا 23·3-])، خونریزی داخل مغزی (51·7%- [58·2- تا 43·8-])، صرع ایدیوپاتیک (26·2%- [43·6- تا 1·1-])، و خونریزی زیر عنکبوتیه (62·8%- [71·6- تا 41·0-]) وجود داشت، اما برای سایر بیماری‌های مغز و اعصاب تغییری مشاهده نشد. در طول سال‌های 1990–2019، تعداد DALYs به دلیل زوال عقل، بیماری پارکینسون، مولتیپل اسکلروز، سکته ایسکمیک مغزی و اختلالات سردرد (شامل میگرن و سردرد تنشی) در این منطقه بیش از دو برابر شده است و بار سال‌های زندگی همراه با ناتوانی (YLDs)، بروز و شیوع مولتیپل اسکلروز، بیماری نورون حرکتی، بیماری پارکینسون و سکته ایسکمیک مغزی، هم در تعداد و هم در نرخ استاندارد سنی، افزایش یافته است. در طول این دوره، بار مطلق YLDs به دلیل آسیب‌های سر و ستون فقرات تقریبا دو برابر شده است.

تفسیر: بار فزاینده بیماری‌های مغز و اعصاب در شمال آفریقا و خاورمیانه با افزایش جمعیت سالخورده همراه بوده است. سکته مغزی و زوال عقل علت‌های اصلی مرگ و میر بوده که عمده آن‌ها به عوامل خطر قابل اصلاح مربوط می‌شود. در نتیجه، برای پیشگیری یا کاهش این بار، مداخلات همسو، نظام‌مند، مادام العمر و چند بخشی نیاز است.

حمایت مالی: موسسه بیل و ملیندا گیتس

حق چاپ © 2024 نویسنده (ها). منتشر شده توسط Elsevier Ltd. این یک مقاله با دسترسی آزاد تحت مجوز CC BY 4.0 است.
