# Supplementary material for: The burden of neurological conditions in north Africa and the Middle East, 1990–2019: a systematic analysis of the Global Burden of Disease Study 2019
Source: Lancet Glob Health. 2024 Apr 8;12(6):e960–82. doi: 10.1016/S2214-109X(24)00093-7 (PMC11099299; doi:10.1016/S2214-109X(24)00093-7)
Supplement: Arabic translation of the abstract [file mmc2.pdf]

THE LANCET
Global Health

Supplementary appendix 2

This translation in Arabic was submitted by the authors and we reproduce it as supplied. It has not been peer reviewed. The Lancet's editorial processes have only been applied to the original in English, which should serve as reference for this manuscript.

تم تقديم هذه الترجمة باللغة العربية من قبل المؤلفين ونعيد إنتاجها كما هو مُقدم. إنها لم تخضع لاستعراض الأقران. تم تطبيق عمليات تحرير لانسيت فقط على النص الأصلي باللغة الإنجليزية، والذي يجب أن يكون بمثابة مرجع لهذه المخطوطة.

Supplement to: GBD 2019 North Africa and the Middle East Neurology Collaborators. The burden of neurological conditions in north Africa and the Middle East, 1990–2019: a systematic analysis of the Global Burden of Disease Study 2019. *Lancet Glob Health* 2024; published online April 8. https://doi.org/10.1016/S2214-109X(24)00093-7.

Arabic summary

عبء الأمراض العصبية في شمال أفريقيا والشرق الأوسط من 1990 إلى 2019: تحليل منهجي لبيانات دراسة العبء العالمي للأمراض لعام 2019

المتعاونون في طب الأعصاب في دائرة الموازنة العالمية للأمراض لعام 2019 في شمال أفريقيا والشرق الأوسط*

خلفية: إن عبء الامراض العصبية في شمال أفريقيا والشرق الأوسط في إزدياد مستمر. نحن نهدف إلى تقييم التغيرات في عبء الحالات العصبية في هذه المنطقة للمساعدة في اتخاذ القرارات في المستقبل.

المنهاجية :في هذا التحليل لبيانات دراسة العبء العالمي للأمراض والإصابات وعوامل الخطر لعام 2019، درسنا الاتجاهات الزمنية لسنوات العمر المصححة باحتساب مدة العجز (سنوات العمر المعدلة حسب العجز والوفيات، والعجز مجتمعة)، والوفيات، والحالات الحديثة، والحالات السائدة ل 14 حالة ومرض عصبي و ثمانية أنواع فرعية في 21 دولة في الشرق الأوسط وشمال افريقيا. بالإضافة إلى ذلك، قمنا بتقييم سنوات العمر المعدلة حسب العجز الناتج عن الامراض العصبية بسبب 34 عامل خطر قابل للتعديل تنتمي الى اربع مستويات للتصنيف خلال الفترة 1990-2019. استخدم نهج التقدير الخاص بنموذج بايزي، واحدثنا فاصل الشك بنسبة 95٪ (UIs) للتقديرات النهائية بناء على القيم المرتبة ال25 و ال975 من 1000 سحب من التوزيع الخلفي.

النتائج: في عام 2019، كان هناك 441·1 ألف حالة وفاة (95% فاصل الشك 347·2 الى 598.4) و 17·6 مليون سنة (95% فاصل الشك 12·5 الى 24·7%) من سنوات العمر المعدلة حسب العجز في شمال إفريقيا والشرق الأوسط. وكانت الأسباب الرئيسية لسنوات العمر المصححة للعجز الناتج عن الامراض العصبية هي السكتة الدماغية والصداع النصفي ومرض الزهايمر والانواع الاخرى من الخرف (المشار إليه فيما يلي بالخرف). في شمال إفريقيا والشرق الأوسط، يعزى 85·8% (95% فاصل الشك 82·6 الى89·1%) من سنوات العمر المصححة باحتساب مدة العجز للسكتات الدماغية و 39·9% (95% فاصل الشك 26·4 الى54·7%) من سنوات العمر المصححة باحتساب مدة العجز للخرف كانت تُعزى إلى عوامل خطر قابلة للتعديل. سجلت شمال إفريقيا والشرق الأوسط أعلى معدلات سنوات العمر المعدلة باحتساب مدة العجز للخرف 387.0 (95% فاصل الشك 107.0 الى 1371.8) و مرض باركنسون 84.4 (95% فاصل الشك 74.7 الى103.2) والصداع النصفي 601.4 (95% فاصل الشك 107.0 الى 1371.8) بين مناطق العالم. خلال الفترة 1990-2019، انخفضت سنوات العمر المصححة باحتساب العجز المطلقة المتعلقة بالتهاب السحايا -75.8 (95% فاصل الشك -81.1 الى -81.1) والكزاز -88.2% (95% فاصل الشك -93.9 الى -76.1) ونزيف تحت العنكبوتية -62.8 (95% فاصل الشك -71.6 الى -41.0)، ولكن بالنسبة لجميع الحالات العصبية الأخرى لم يكن هناك تغيير. خلال الفترة من 1990 إلى 2019. خلال الفترة 1990-2019، زاد عدد سنوات العمر المعدلة حسب العجز الناجم عن الخرف ومرض باركنسون والتصلب اللويحي والسكتة الدماغية الإقفارية واضطرابات الصداع (أي الصداع النصفي والصداع من نوع التوتري) بأكثر من الضعف في المنطقة. وزاد عبء سنوات العيش مع الإعاقة (YLDs)، وحدوث وانتشار التصلب المتعدد، ومرض الخلايا العصبية الحركية ، ومرض باركنسون ، والسكتة الدماغية الإقفارية في كل من المعدل المعياري للعمر وعدده. خلال هذه الفترة، تقريبا تضاعف العبء المطلق ل YLDs بسبب إصابات الرأس والعمود الفقري.

تفسير: يصاحب العبء العصبي المتزايد في شمال أفريقيا والشرق الأوسط ارتفاع نسبة الشيخوخة للسكان. السكتة الدماغية والخرف هي الأسباب الرئيسية للإعاقة العصبية ومعدلات الوفيات، والتي تعزى في المقام الأول إلى عوامل الخطر الشائعة القابلة للتعديل. وهي تتطلب تدخلات متآزرة ومنهجية ومستمرة ومتعددة القطاعات تهدف إلى كبح العبء أو التخفيف منه.

التمويل: مؤسسة بيل و ميليندا غيتس

حقوق النشر © 2024 المؤلف (المؤلفون). نشرت من قبل إلسفير المحدودة. هذه مقالة مفتوحة الوصول بموجب ترخيص CC BY 4.0.
