# Supplementary material for: The burden of neurological conditions in north Africa and the Middle East, 1990–2019: a systematic analysis of the Global Burden of Disease Study 2019
Source: Lancet Glob Health. 2024 Apr 8;12(6):e960–82. doi: 10.1016/S2214-109X(24)00093-7 (PMC11099299; doi:10.1016/S2214-109X(24)00093-7)
Supplement: Turkish translation of the abstract [file mmc3.pdf]

THE LANCET
Global Health

Supplementary appendix 3

This translation in Turkish was submitted by the authors and we reproduce it as supplied. It has not been peer reviewed. The Lancet's editorial processes have only been applied to the original in English, which should serve as reference for this manuscript.

Bu Türkçe çeviri yazarlar tarafindan gönderilmiştir ve biz bunu sunulduğu haliyle çoğaltıyoruz. Emsal değerlendirilmesi gerçekleştirilmemiştir. Lancet'in editoryal süreçleri, yalnızca bu el yazması için referans vazifesi gören İngilizce orijinaline uygulanmıştır.

Supplement to: GBD 2019 North Africa and the Middle East Neurology Collaborators. The burden of neurological conditions in north Africa and the Middle East, 1990–2019: a systematic analysis of the Global Burden of Disease Study 2019. *Lancet Glob Health* 2024; published online April 8. https://doi.org/10.1016/S2214-109X(24)00093-7.

Kuzey Afrika ve Ortadoğu'daki nörolojik durumların yükü, 1990-2019: Global Hastalık Yükü Çalışması 2019 verilerinin sistematik analizi

GBD 2019 Kuzey Afrika ve Orta Doğu Nöroloji İşbirliği Grubu*

Arka plan: Arka Plan Kuzey Afrika ve Orta Doğu'da nörolojik durumların yükü artmaktadır. Bu geniş bölgede nörolojik durumların yükündeki değişiklikleri değerlendirmeyi ve gelecekteki karar verme süreçlerine yardımcı olmayı amaçladık.

Yöntemler Yöntemler Global Hastalık, Yaralanmalar ve Risk Faktörleri Yükü Çalışması (GBD) 2019 verilerinin bu analizinde, Kuzey Afrika ve Orta Doğu süper bölgesindeki 21 ülkede, 14 önemli nörolojik durum ve sekiz alt tipinin engellilik ayarlı yaşam yılları (disability adjusted life years-DALYs, ölümler ve engellilikler birleşimi), ölümler, insidans ve prevelans vakalarının zamansal eğilimlerini inceledik. Ayrıca, 1990-2019 arasında dört sınıflandırma seviyesi içinde 22 potansiyel olarak modifiye edilebilir risk faktörü nedeniyle nörolojik DALY'leri değerlendirdik. Tahmin yaklaşımımızda Bayesian modelleme kullandık ve 1000 önceki dağılımdan çekilişin 2,5 ve 97,5'inci yüzdelik değerlerine dayanarak nihai tahminler için %95 belirsizlik aralıkları (UI) oluşturduk.

Bulgular Bulgulara göre 2019 yılında, Kuzey Afrika ve Orta Doğu'da 441,1 bin (%95 UI 347,2–598,4) ölüm ve 17,6 milyon (12,5–24,7) nörolojik DALY meydana gelmiştir. Nörolojik DALY'lerin başlıca nedenleri inme, migren ve Alzheimer hastalığı ile diğer demans türleri (bundan böyle demanslar olarak anılacaktır) olmuştur. Kuzey Afrika ve Orta Doğu'da 2019 yılında inme ile ilgili yaşa göre standardize edilmiş DALY'lerin %85,8'i (82,6–89,1) ve demanslarla ilgili DALY'lerin %39,9'u (26,4–54,7) modifiye edilebilir risk faktörlerine atfedilebilir bulunmuştur. Kuzey Afrika ve Orta Doğu, dünya genelindeki küresel bölgeler arasında demans (387,0 [172,0–848,5]), Parkinson hastalığı (84,4 [74,7–103,2]) ve migren (601,4 [107,0–1371,8]) nedeniyle 100.000 nüfus başına düşen yaşa göre standardize edilmiş DALY oranlarının en yüksek olduğu bölge olarak kaydedilmiştir. 1990 ile 2019 yılları arasında, menenjit (-%75,8 [-81,1 to -69,5]), tetanos (-%88,2 [-93,9 to -76,1]), inme (-%32,0 [-39,1 to -23,3]), intraserebral kanama (-%51,7 [-58,2 to -43,8]), idiopatik epilepsi (-%26,2 [-43,6 to -1,1]) ve subaraknoid kanama (-%62,8 [-71,6 to -41,0]) ile ilgili yaşa göre standardize edilmiş DALY oranlarında azalma görülürken, diğer nörolojik durumlarda bir değişiklik olmamıştır. 1990–2019 yılları arasında, bu geniş bölgede demanslar, Parkinson hastalığı, multipl skleroz, iskemik inme ve baş ağrısı bozuklukları (örneğin migren ve gerginlik tipi baş ağrısı) nedeniyle DALY sayısı iki katından fazla artmış ve multipl skleroz, motor nöron hastalığı, Parkinson hastalığı ve iskemik inme ile ilgili yaşanılan engelli yılların (years lived with disability-YLDs) yükü, insidans ve prevalansı hem yaşa göre standardize oranda hem de sayıda artış göstermiştir. Bu dönemde, baş ve omurilik yaralanmaları nedeniyle YLD'lerin mutlak yükü neredeyse iki katına çıkmıştır.

Yorum Kuzey Afrika ve Ortadoğu'da, nörolojik durumların yükündeki artış yaşlanan nüfusun artışıyla birlikte gözlemlenmektedir. İnme ve demans, nörolojik sakatlık ve ölüm oranlarının başlıca nedenleri olup, bu durumların çoğu modifiye edilebilir risk faktörlerine bağlıdır. Bu yükü önlemek veya en aza indirmek için sinerjik, sistematik, ömür boyu süren ve çok yönlü müdahaleler gerekmektedir.

Finansman Bill ve Melinda Gates Vakfı.

Telif Hakkı © 2024 Yazar(lar). Elsevier Ltd. tarafından yayımlanmıştır. Bu makale, CC BY 4.0 lisansı altında açık erişimli bir makaledir.
