# Supplementary material for: The burden of neurological conditions in north Africa and the Middle East, 1990–2019: a systematic analysis of the Global Burden of Disease Study 2019
Source: Lancet Glob Health. 2024 Apr 8;12(6):e960–82. doi: 10.1016/S2214-109X(24)00093-7 (PMC11099299; doi:10.1016/S2214-109X(24)00093-7)
Supplement: Supplementary appendix 4 [file mmc4.pdf]

THE LANCET
Global Health

Supplementary appendix 4

This appendix formed part of the original submission and has been peer reviewed. We post it as supplied by the authors.

Supplement to: GBD 2019 North Africa and the Middle East Neurology Collaborators. The burden of neurological conditions in north Africa and the Middle East, 1990–2019: a systematic analysis of the Global Burden of Disease Study 2019. *Lancet Glob Health* 2024; published online April 8. https://doi.org/10.1016/S2214-109X(24)00093-7.

Table of contents

Table of contents **1**
Authors' contributions **6**
Supplementary Methods **8**
Table S1. Guidelines for Accurate and Transparent Health Estimates Reporting (GATHER) checklist of information for reports of global health estimates 8
1. Data 10
1.1. Data sources and outcome measures 10
Table S2. Global Burden of Disease Study 2019 (GBD 2019) data input sources counts for neurological conditions in North Africa and Middle East 10
1.2. Regions and nations 13
Table S3. Global Burden of Disease Study 2019 (GBD 2019) classification of countries and regions 13
1.3. GBD 2019 neurological data changes 14
1.4. Literature search 14
2. Methodological summary of neurological conditions and definition of risk factors 15
Table S4. Neurological conditions included in the present study and their definitions based on the Global Burden of Disease Study 2019 (GBD 2019) 15
Table S5. Risk factor exposure definitions in the Global Burden of Disease Study 2019 16
Table S6. Classification of risk factors in the Global Burden of Disease Study 2019 (GBD 2019) 17
3. Modelling 19
3.1. Fatal disease modelling 19
3.1.1 Modelling steps for idiopathic epilepsy death estimation (as an example of fatal modelling processes) 20
Table S7. Predictive covariates used in the epilepsy fatal modelling process 20
Figure S1. Plot of the relative influence of covariates for idiopathic epilepsy 21
Figure S2. Flowchart of modelling steps to epilepsy-related death and years of life lost (YLLs) 21
3.2. Non-fatal disease modelling 22
3.2.1. Modelling steps for non-fatal dementia estimation (as an example of non-fatal modelling processes) 22
Figure S3. Flowchart of modelling steps for non-fatal dementia estimation 22
Table S8. Results of non-reference adjusted meta-regression analysis for dementia estimations 23
Table S9. Dementia severity definitions 24
3.3. Choosing the appropriate model and imprecision of the estimates 24
3.4. Heterogeneities 25
3.5. GBD global standard population 26
3.6. Risk factor estimation 26
3.6.1. Determine relative risks 27
3.6.2 Search terms and data preparation 28
3.6.3. Data analysis 29
3.6.4. Estimates exposure 30
Theoretical minimum-risk exposure level (TMREL) 31
Estimate population-attributable fractions 31
Estimate summary exposure values 32
Mediation 32
Calculating the burden of multiple risk factors 33
Adjusting for mediation 33
Calculating mediation factor 33
Uncertainty of aggregated and mediated PAFs 34
Important assumptions in aggregating risk factors and including mediation 34

Estimate attributable burden 34
Decomposition analysis of deaths and DALYs 34
4. Detailed methodology for each condition 36
4.1. Alzheimer's disease and other dementias (fatal modelling) 36
Input data 36
Modelling strategy 37
Overview 37
Modelling steps 37
4.2. Alzheimer's disease and other dementias (non-fatal modelling) 40
Flowchart 40
Input data and methodological summary 40
Case definition 40
Model inputs 40
Item Response Theory for prevalence prediction 41
Excluding incidence 41
Severity splits 41
Prisma diagram of dementia severity split systematic review 42
Figure. Severity ratios for each 5-year age bin, by sex. 43
Relative risk due to other causes 43
Modelling strategy 44
4.3. Parkinson's Disease (fatal modelling) 46
Input Data 46
Modelling Strategy 46
Overview 46
Modelling steps 47
4.4. Parkinson's Disease (non-fatal modelling) 51
Flowchart 51
Case definition 51
Model inputs 51
Modelling strategy 52
MR-BRT Crosswalk Adjustment Factors for Parkinson's Disease 52
Severity splits 53
4.5. Idiopathic Epilepsy (fatal modelling) 54
Flowchart 54
Input Data and Methodological Summary for Idiopathic Epilepsy 54
Input data 54
Modelling strategy 54
4.6. Epilepsy impairment envelope (non-fatal modelling) 56
Flowchart 56
Case definition 56
Input data and processing 56
Modelling strategy 58
Severity splits & disability weights 59
4.7. Multiple Sclerosis (fatal modelling) 60
Flowchart 60
Input Data and Methodological Summary for Multiple Sclerosis 60
Input data 60
Modelling strategy 60
4.8. Multiple sclerosis (non-fatal modelling) 62

Flowchart 62
Input data and methodological summary 62
Case definition 62
Input data and processing 62
Modelling strategy 63
Compartmental model 63
Severity splits 63
4.9. Headaches (non-fatal modelling) 65
Flowchart 65
Input Data and Methodological Summary for Headaches 65
Case definition 65
Migraine 65
Tension-type headache 66
Medication overuse headache 66
Input data 66
Migraine 66
Tension-type headache 67
Medication overuse headache 67
Age and sex splitting 67
Data adjustment (Bias adjustments) 67
Modelling strategy 69
Migraine 69
Tension-type headache 70
Medication overuse headache 70
Medication overuse headache split 71
4.10. Motor Neuron Disease (fatal modelling) 72
Flowchart 72
Input Data and Methodological Summary for Motor Neuron Disease 72
Input data 72
Modelling strategy 72
4.11. Motor neuron diseases (non-fatal modelling) 74
Flowchart 74
Case definition 74
Input data and data processing 74
Modelling strategy 75
Severity splits 76
4.12. Other Neurological Disorders (fatal modelling) 80
Flowchart 80
Input Data and Methodological Summary for Other Neurological Disorders 80
Input data 80
Modelling strategy 80
4.13. Other neurological disorders (non-fatal modelling) 82
4.14. Stroke (fatal modelling) 83
4.14.1. Ischaemic stroke 83
Input data 83
Modelling strategy 83
4.14.2. Intracerebral haemorrhage 84
Input data 84
Modelling strategy 85

4.14.3. Subarachnoid haemorrhage....85
Input data....85
Modelling strategy....85
Selected covariates for CODEm models, subarachnoid haemorrhage....86
4.14.4. Selected covariates for CODEm models, overall stroke and subtypes....86
DisMod covariates....87
ICD Codes used in fatal and non-fatal analysis....89
5. Supplementary limitations....90
6. Confounding factors in context....91
Supplementary Results....92
1. All ages values....92
Figure S4. The proportions of disability-adjusted life years related to neurological conditions in A) North Africa and Middle East and B) the globe....92
Figure S5. Heat maps of the burden of neurological conditions in all ages among 21 countries of North Africa and Middle East in 2019....93
Figure S6. Trends of the absolute number of disability-adjusted life years (DALYs) related to neurological conditions in North Africa and Middle East....97
2. Age-standardised values....99
Figure S7. Ranks of age-standardised neurological disability-adjusted life years (DALYs) rates in seven GBD super regions and four World Bank income levels in 2019....99
Figure S8. Trends of the age-standardised rate of disability-adjusted life years (DALYs) related to neurological conditions in North Africa and Middle East....99
Figure S9. Ranks of age-standardised neurological disability-adjusted life years (DALYs) rates in North Africa and Middle East countries compared with other regions in the world in 2019....101
3. Modifiable risk factors....102
Figure S10. Ranks of age-standardised rates of (A) stroke and (B) dementia disability-adjusted life years (DALYs) attributable to risk factors in North Africa and Middle East countries compared with other regions in the world in 2019....102
4. Age- and sex-specific proportions....103
Figure S11. Age-specific burden of neurological conditions in North Africa and Middle East measured by disability-adjusted life years (DALYs) among (A) males and (B) females....104
5. Supplementary results tables....105
Table S10 Global and regional burden, mortality, incidence, and prevalence of neurological conditions in different regions of the world....105
Table S11 Cause- and sex-specific burden, mortality, incidence, and prevalence of neurological conditions in North Africa and Middle East countries....143
Table S12 Burden, mortality, incidence, and prevalence of neurological conditions in North Africa and Middle East countries....195
Table S13 Disability, prevalence, and incidence of head and spinal injuries in North Africa and Middle East countries....254
Table S14 Regional incidence, prevalence, and disability of head and spinal injuries in the world and in North Africa and Middle East countries....266
Table S15 Burden of neurological conditions attributable to risk factors in North Africa and Middle East countries....276
Table S16 Regional and national disabilities and deaths related to neurological conditions attributable to risk factors in North Africa and Middle East countries....321
Table S17 Age-specific disability and death related to neurological conditions (NCs) in North Africa and Middle East countries in 2019....440

Table S18 Age-specific disability and death related to stroke in North Africa and Middle East countries in 2019 447
Table S19 Age-specific disability and death related to Alzheimer’s disease in North Africa and Middle East countries in 2019 454
Table S20 Age-specific disability and death related to Parkinson’s disease in North Africa and Middle East countries in 2019 461
Table S21 Age-specific disability and death related to meningitis in North Africa and Middle East countries in 2019 468
Table S22 Age-specific disability and death related to encephalitis in North Africa and Middle East countries in 2019 475
Table S23 Age-specific disability and death related to tetanus in North Africa and Middle East countries in 2019 482
Table S24 Age-specific disability and death related to brain and central nervous system cancer in North Africa and Middle East countries in 2019 489
Table S25 Age-specific disability and death related to idiopathic epilepsy in North Africa and Middle East countries in 2019 496
Table S26 Age-specific disability and death related to multiple sclerosis in North Africa and Middle East countries in 2019 503
Table S27 Age-specific disability related to headache disorders in North Africa and Middle East countries in 2019 510
Table S28 Age-specific disability and death related to motor neuron disease in North Africa and Middle East countries in 2019 513
Table S29 Age-specific disability and death related to other rare neurological conditions in North Africa and Middle East countries in 2019 520
Table S30 Age-specific disability and death related to neurological disorders in North Africa and Middle East countries in 2019 527
Table S31 Age- and sex-specific disability, prevalent cases, and new cases of head and spinal injuries in North Africa and Middle East countries in 2019 534
Table S32 Temporal changes in the number of DALY, mortality, incidence, prevalence, and YLD of neurological conditions in North Africa and Middle East and the world from 2010 to 2019 540
Table S33 Temporal changes in the age-standardised rate of DALY, mortality, incidence, prevalence, and YLD of neurological conditions in North Africa and Middle East and the world from 2010 to 2019 553
Table S34 Systematic literature search for the epidemiological measures of neurological conditions in North Africa and Middle East countries 566
References 569

Authors' contributions

Providing data or critical feedback on data sources

Sina Abdollahzade, Hassan Abidi, Hassan Abolhassani, Ahmed Abualhasan, Niveen ME Abu-Rmeileh, Ahmed Abu-Zaid, Sepideh Ahmadi, Hanadi Al Hamad, Fahad Mashhour Alanezi, Turki M Alanzi, Syed Mohamed Aljunid, Jalal Arabloo, Judie Arulappan, Mohammad Athar, Seyyed Shamsadin Athari, Abolfazl Avan, Ahmed Y Azzam, Sara Bagherieh, Ovidiu Constantin Baltatu, Derrick A. Bennett, Vijayalakshmi S Bhojaraja, Mohamed Fahmy Doheim, Fariba Dorostkar, Ebrahim Eini, Nevine El Nahas, Iman El Sayed, Sharareh Eskandarieh, Mohammad Farahmand, Valery L Feigin, Mansour Ghafourifard, Sherief Ghozy, Pouya Goleij, Vladimir Hachinski, Nima Hafezi-Nejad, Arvin Haj-Mirzaian, Ahmed I Hasaballah, Reza Heidari-Soureshjani, Haitham Jahrami, Tahereh Javaheri, Sathish Kumar Jayapal, Rohollah Kalhor, Yousef Saleh Khader, Maseer Khan, Moien AB Khan, Savita Lasrado, Mohammed Magdy Abd El Razek, Mohammad Ali Mansournia, Parham Mardi, Entezar Mehrabi Nasab, Ritesh G Menezes, Soheil Mohammadi, Syam Mohan, Ali H Mokdad, Sara Momtazmanesh, Majid Motaghinejad, Mohsen Naghavi, Zuhair S Natto, Mayowa O Owolabi, Simone Perna, Michael A Piradov, Vafa Rahimi-Movaghar, Amir Masoud Rahmani, Ali Rajabpour-Sanati, Chythra R Rao, Reza Rawassizadeh, Negar Rezaei, Nima Rezaei, Aly M A Saad, Basema Saddik, Sahar Saeedi Moghaddam, Abdallah M Samy, Brijesh Sathian, Mete Saylan, Mehran Shams-Beyranvand, Javad Sharifi-Rad, Jeevan K Shetty, Parnian Shobeiri, Soraya Siabani, Yasaman Taheri Abkenar, Sahel Valadan Tahbaz, Bay Vo, Seyed Hossein Yahyazadeh Jabbari, Iman Zare, and Mohammad Zoladl.

Developing methods or computational machinery

Abolfazl Avan, Valery L Feigin, Sherief Ghozy, Tahereh Javaheri, Sathish Kumar Jayapal, Ali H Mokdad, Mohsen Naghavi, Amir Masoud Rahmani, Reza Rawassizadeh, Javad Sharifi-Rad, and Bay Vo.

Providing critical feedback on methods or results

Amirali Aali, Sina Abdollahzade, Hassan Abidi, Hassan Abolhassani, Ahmed Abualhasan, Eman Abu-Gharbieh, Niveen ME Abu-Rmeileh, Ahmed Abu-Zaid, Aqeel Ahmad, Luai A Ahmed, Hanadi Al Hamad, Fahad Mashhour Alanezi, Turki M Alanzi, Yousef Alimohamadi, Syed Mohamed Aljunid, Rajaa M Al-Raddadi, Sohrab Amiri, Jalal Arabloo, Judie Arulappan, Ashokan Arumugam, Ali A Asadi-Pooya, Mohammad Athar, Seyyed Shamsadin Athari, Maha Moh'd Wahbi Atout, Abolfazl Avan, Sina Azadnajafabad, Mohammadreza Azangou-Khyavy, Amirhossein Azari Jafari, Ahmed Y Azzam, Nayereh Baghcheghi, Sara Bagherieh, Ovidiu Constantin Baltatu, Derrick A. Bennett, Vijayalakshmi S Bhojaraja, Ali Bijani, Amira Hamed Darwish, Shirin Djalalinia, Mohamed Fahmy Doheim, Fariba Dorostkar, Ebrahim Eini, Nevine El Nahas, Iman El Sayed, Muhammed Elhadi, Mohamed A Elmonem, Sharareh Eskandarieh, Shahriar Faghani, Aida Fallahzadeh, Valery L Feigin, Mansour Ghafourifard, Ali Gholami, Vladimir Hachinski, Mostafa Hadei, Nima Hafezi-Nejad, Arvin Haj-Mirzaian, Rabih Halwani, Samer Hamidi, Ahmed I Hasaballah, Khedidja Hedna, Reza Heidari-Soureshjani, Mohammad-Salar Hosseini, Soodabeh Hoveidamanesh, Haitham Jahrami, Elham Jamshidi, Tahereh Javaheri, Sathish Kumar Jayapal, Laleh R Kalankesh, Rohollah Kalhor, Zahra Kamiab, Mohammad Keykhaei, Yousef Saleh Khader, Maseer Khan, Moien AB Khan, Khatatbeh, Ahmad Khosravi, Farzad Kompani, Hamid Reza Koohestani, Bagher Larijani, Savita Lasrado, Mohammed Magdy Abd El Razek, Mohammad-Reza Malekpour, Ahmad Azam Malik, Mohammad Ali Mansournia, Parham Mardi, Seyed Farzad Maroufi, Sahar Masoudi, Mahsa Mayeli, Entezar Mehrabi Nasab, Ritesh G Menezes, Seyyedmohammadsadeq Mirmoeeni, Mohammad Mirza-Aghazadeh-Attari, Maryam Mobarakabadi, Esmaeil Mohammadi, Soheil Mohammadi, Syam Mohan, Ali H Mokdad, Sara Momtazmanesh, Fateme Montazeri, Mostafa Moradi Sarabi, Paula Moraga, Negar Morovatdar, Majid Motaghinejad, Mohsen Naghavi, Zuhair S Natto, Seyed Aria Nejadghaderi, Nafise Noroozi, Hassan Okati-Aliabad, Mayowa O Owolabi, Simone Perna, Michael A Piradov, Mohammadreza Pourahmadi, Alireza Rafiei, Vafa Rahimi-Movaghar, Amir Masoud Rahmani, Shayan Rahmani, Vahid Rahmanian, Ali Rajabpour-Sanati, Chythra R Rao, Mohammad-Mahdi Rashidi, Reza Rawassizadeh, Iman Razeghian-Jahromi, Elrashdy Moustafa Mohamed Redwan, Malihe Rezaee, Nazila Rezaei, Nima Rezaei, Mohsen Rezaeian, Reza Rikhtegar, Aly M A Saad, Basema Saddik, Saeid Sadeghian, Sahar Saeedi Moghaddam, Abdallah M Samy, Nima Sanadgol, Arash Sarveazad, Brijesh Sathian, Mete Saylan, Ataollah

Shahbandi, Shayan Shahrokhi, Mehran Shams-Beyranvand, Mohd Shanawaz, Javad Sharifi-Rad, Rahim Ali Sheikhi, Jeevan K Shetty, Parnian Shobeiri, Seyed Afshin Shorofi, Soraya Siabani, Jaimie D Steinmetz, Saverio Stranges, Seyyed Mohammad Tabatabaei, Yasaman Taheri Abkenar, Moslem Taheri Soodejani, Mohamad-Hani Temsah, Sahel Valadan Tahbaz, Rohollah Valizadeh, Siavash Vaziri, Bay Vo, Seyed Hossein Yahyazadeh Jabbari, Metin Yesiltepe, Nazar Zaki, Ali Zare Dehnavi, and Mohammad Zoladl.

Drafting the work or revising it critically for important intellectual content
Amirali Aali, Mohsen Abbasi-Kangevari, Zeinab Abbasi-Kangevari, Foad Abd-Allah, Sina Abdollahzade, Hassan Abidi, Hassan Abolhassani, Ahmed Abualhasan, Eman Abu-Gharbieh, Ahmed Abu-Zaid, Sepideh Ahmadi, Luai A Ahmed, Marjan Ajami, Fahad Mashhour Alanezi, Turki M Alanzi, Sohrab Amiri, Jalal Arabloo, Judie Arulappan, Ashokan Arumugam, Ali A Asadi-Pooya, Seyyed Shamsadin Athari, Maha Moh'd Wahbi Atout, Abolfazl Avan, Sina Azadnajafabad, Mohammadreza Azangou-Khyavy, Amirhossein Azari Jafari, Ahmed Y Azzam, Sara Bagherieh, Ovidiu Constantin Baltatu, Gholamreza Bazmandegan, Derrick A. Bennett, Vijayalakshmi S Bhojaraja, Saeid Bitaraf, Daniela Calina, Mohamed Fahmy Doheim, Ebrahim Eini, Iman El Sayed, Muhammed Elhadi, Mohamed A Elmonem, Sharareh Eskandarieh, Shahriar Faghani, Aida Fallahzadeh, Valery L Feigin, Seyyed-Hadi Ghamari, Sherief Ghozy, Vladimir Hachinski, Mostafa Hadei, Nima Hafezi-Nejad, Arvin Haj-Mirzaian, Rabih Halwani, Ahmed I Hasaballah, Amr Hassan, Khedidja Hedna, Mohamed I Hegazy, Mohammad-Salar Hosseini, Haitham Jahrami, Sathish Kumar Jayapal, Laleh R Kalankesh, Yousef Saleh Khader, Maseer Khan, Moien AB Khan, Khatatbeh, Hamid Reza Khayat Kashani, Farzad Kompani, Bagher Larijani, Savita Lasrado, Mohammed Magdy Abd El Razek, Mohammad-Reza Malekpour, Ahmad Azam Malik, Parham Mardi, Mahsa Mayeli, Entezar Mehrabi Nasab, Ritesh G Menezes, Seyyedmohammadsadeq Mirmoeeni, Esmaeil Mohammadi, Soheil Mohammadi, Ali H Mokdad, Sara Momtazmanesh, Fateme Montazeri, Mostafa Moradi Sarabi, Paula Moraga, Majid Motaghinejad, Mohsen Naghavi, Zuhair S Natto, Seyed Aria Nejadghaderi, Mayowa O Owolabi, Hamidreza Pazoki Toroudi, Michael A Piradov, Vafa Rahimi-Movaghar, Shayan Rahmani, Chythra R Rao, Iman Razeghian-Jahromi, Elrashdy Moustafa Mohamed Redwan, Nazila Rezaei, Nima Rezaei, Aly M A Saad, Basema Saddik, Masoumeh Sadeghi, Amirhossein Sahebkar, Saina Salahi, Sarvenaz Salahi, Abdallah M Samy, Arash Sarveazad, Mete Saylan, Mehran Shams-Beyranvand, Mohd Shanawaz, Javad Sharifi-Rad, Jeevan K Shetty, Parnian Shobeiri, Seyed Afshin Shorofi, Jaimie D Steinmetz, Saverio Stranges, Yasaman Taheri Abkenar, Mohamad-Hani Temsah, Alireza Vakilian, Sahel Valadan Tahbaz, Seyed Hossein Yahyazadeh Jabbari, Iman Zare, Ali Zare Dehnavi, and Mohammad Zoladl.

Managing the estimation or publications process
Abolfazl Avan, Ali H Mokdad, and Mohsen Naghavi.

Supplementary Methods

The proposal for the present study (1523-GBD2019-032020) has been approved by the Global Burden of Diseases, Injuries, and Risk Factors Study (GBD) Scientific Publications team. The manuscript is written according to the Guidelines for Accurate and Transparent Health Estimates Reporting (GATHER) statement (Table S1) and approved by the University of Washington institutional review board committee. The GBD protocol is also available online.

Table S1. Guidelines for Accurate and Transparent Health Estimates Reporting (GATHER) checklist of information for reports of global health estimates

Item	Checklist item	Reference
**Objectives and funding**		
1	Define the indicator(s), populations (including age, sex, and geographic entities), and time period(s) for which estimates were made	Methods, paragraphs 2, 3, and 6
2	List the funding sources for the work	Methods, section "role of the funding source"
**Data Inputs**		
For all data inputs from multiple sources that are synthesised as part of the study		
3	Describe how the data were identified and how the data were accessed	Supplementary methods, sections 1, 3.6, and 4
4	Specify the inclusion and exclusion criteria. Identify all ad-hoc exclusions	Supplementary Methods, sections 1.1 and 4
5	Provide information on all included data sources and their main characteristics. For each data source used, report reference information or contact name/institution, population represented, data collection method, year(s) of data collection, sex and age range, diagnostic criteria or measurement method, and sample size, as relevant	Methods; Supplementary Methods, sections 1, 3.6, and 4
6	Identify and describe any categories of input data that have potentially important biases (eg, based on characteristics listed in item 5)	Discussion, paragraphs 6 and 7; Supplementary Methods, sections 5 and 6
For data inputs that contribute to the analysis but were not synthesised as part of the study		
7	Describe and give sources for any other data inputs	Supplementary Methods, sections 1.1, 3.2, and 4
For all data inputs		
8	Provide all data inputs in a file format from which data can be efficiently extracted (eg, a spreadsheet rather than a PDF), including all relevant meta-data listed in item 5. For any data inputs that cannot be shared because of ethical or legal reasons, such as third-party ownership, provide a contact name or the name of the institution that retains the right to the data.	Supplementary Methods, section 1.1

Table S1. Guidelines for Accurate and Transparent Health Estimates Reporting (GATHER) checklist of information for reports of global health estimates		
Item	Checklist item	Reference
**Data analysis**		
9	Provide a conceptual overview of the data analysis method. A diagram may be helpful	Supplementary Methods, sections 2, 3.1, and 3.2
10	Provide a detailed description of all steps of the analysis, including mathematical formulae. This description should cover, as relevant, data cleaning, data pre-processing, data adjustments and weighting of data sources, and mathematical or statistical model(s)	Supplementary Methods, sections 2, 3, and 4
11	Describe how candidate models were evaluated and how the final model(s) were selected	Supplementary Methods, sections 3 and 4
12	Provide the results of an evaluation of model performance, if done, as well as the results of any relevant sensitivity analysis	Supplementary Methods, sections 3.3 and 4
13	Describe methods for calculating uncertainty of the estimates. State which sources of uncertainty were, and were not, accounted for in the uncertainty analysis	Supplementary Methods, sections 3.3, 3.4, and 4
14	State how analytic or statistical source code used to generate estimates can be accessed	Methods, the last paragraph
**Results and Discussion**		
15	Provide published estimates in a file format from which data can be efficiently extracted	Supplementary Methods, section 1.1
16	Report a quantitative measure of the uncertainty of the estimates (e.g. uncertainty intervals)	Results, estimates are provided with 95% uncertainty intervals
17	Interpret results in light of existing evidence. If updating a previous set of estimates, describe the reasons for changes in estimates	Discussion, paragraphs 2–4
18	Discuss limitations of the estimates. Include a discussion of any modelling assumptions or data limitations that affect interpretation of the estimates	Discussion, paragraphs 6–8<br>Supplementary Methods, sections 5 and 6

This appendix is supplemented to: "Age-, sex-, and risk-specific burden of neurological conditions in North Africa and Middle East from 1990 to 2019: A systematic analysis of the Global Burden of Disease Study 2019 data."

Portions of this Supplementary Methods section are reproduced or adapted from appendices of methodological detail for "Global burden of 369 diseases and injuries in 204 countries and territories, 1990–2019: a systematic analysis for the Global Burden of Disease Study 2019",[1] "Global burden of 87 risk factors in 204 countries and territories, 1990-2019: a systematic analysis for the Global Burden of Disease Study 2019,"[2] and Feigin et al.[3] Relevant references are provided separately at the end of the Supplementary Appendix.

1. Data

1.1. Data sources and outcome measures

The Global Burden of Diseases, Injuries, and Risk Factors Study 2019 (GBD 2019) gathered numerous data sources for each disease or injury to synthesise input sources to estimate different measures, including censuses, household surveys, civil registration and vital statistics, disease registries, health service use, air pollution monitors, satellite imaging, disease notifications, and other sources. These data sources were identified through a systematic literature review, data requests to international organisations and national agencies, and collaborations with GBD researchers and experts. The data sources were then harmonised and adjusted to account for different case definitions, diagnostic criteria, and reporting biases before being input into a statistical model.

For GBD 2019 estimates, data from 3686 vital registration sources, 147 verbal autopsy sources, 368 incidence sources, 117 prevalence sources, 229 excess mortality sources, 7753 risk factor exposure sources, and 2733 relative risk sources were used. Among these, 4474 sources belonged to all GBD measures in NAME. Among 2469 sources related to deaths and disabilities in NAME, those related to neurological conditions are summarised in Table S2 below. Additionally, there were 782 sources related to risk factors and 2067 sources related to covariates in NAME. GBD 2019 results are freely available via an interactive data downloading tool on the Global Health Data Exchange (GHDx) GBD 2019 website and VizHub - GBD Results.

Table S2. Global Burden of Disease Study 2019 (GBD 2019) data input sources counts for neurological conditions in North Africa and Middle East

	**Causes of death**		**Non-fatal health outcomes**	
	Total NAME citations	Total source metadata rows	Total NAME citations	Total source metadata rows
Meningitis	166	346,261	34	1583
Encephalitis	140	171,678	6	318
Tetanus	146	346,086	5	41
Brain and central nervous system cancer	217	302,124	70	19,680
Stroke	140	569,984	31	935
Ischaemic stroke	110	134,136	27	387
Intracerebral haemorrhage	103	132,032	20	313
Subarachnoid haemorrhage	103	132,032	12	235
Neurological disorders*	126	727,916	101	3778
Alzheimer's disease and other dementias	N/A†	N/A†	10	91
Parkinson's disease	123	119,408	8	76
Idiopathic epilepsy	126	154,306	34	783
Multiple sclerosis	123	140,406	30	2323
Motor neuron disease	116	163,790	2	34
Headache disorders‡	0	0	18	471
Migraine	0	0	18	394
Tension-type headache	0	0	9	77
Other neurological disorders	110	150,006	0	0

Table S2. Global Burden of Disease Study 2019 (GBD 2019) data input sources counts for neurological conditions in North Africa and Middle East

	Causes of death		Non-fatal health outcomes	
	Total NAME citations	Total source metadata rows	Total NAME citations	Total source metadata rows
Head injuries§	N/A	N/A	N/A	N/A
Spinal injuries§	N/A	N/A	N/A	N/A

Note: The numbers indicate data specific to the North Africa and Middle-East (NAME) region. All data are retrievable via the [Global Burden of Disease Study 2019 (GBD 2019) Data Input Sources Tool | GHDx (healthdata.org)](healthdata.org).
*Neurological disorders included Alzheimer's disease and other dementias, Parkinson's disease, idiopathic epilepsy, multiple sclerosis, headache disorders (including migraine and tension-type headache), motor neuron disease, and other neurological disorders. †N/A, not available; we did not use any of the vital registration or verbal autopsy data for traditional CODEm analyses for dementia fatal modelling. ‡Headaches are not regarded as cause of deaths, and we do not describe deaths to headaches. §N/A, not available; Head/spinal injuries are N-coded injuries and not E-coded injuries. E-coded injuries were split amongst the 47 N-coded injuries using an E-N matrix. Therefore, the total non-fatal citations would have included all citations used for E-coded injuries estimation. There were no cause of death citations because deaths were attributed to E-coded injuries and not estimated for N-coded injuries.

The steps to identify and access the data include:

- Searching for published and unpublished data on neurological conditions from various sources, such as PubMed, Embase, Scopus, Web of Science, Google Scholar, and national and international databases and reports.
- Contacting experts and collaborators in the field of neurology and public health to obtain additional data and information.
- Approved access and intake by IHME data librarians of larger-scale surveys, censuses, registries, and vital registration data
- Screening the data for eligibility based on predefined inclusion and exclusion criteria, such as study design, population, case definition, and quality assessment.
- Extracting relevant data from the selected sources and entering them into a standardised data collection tool.
- Harmonising the data to account for different case definitions and methods of data collection, using a meta-regression approach.
- Inputting the data into a Bayesian hierarchical model called Disease-Model-Bayesian Meta-Regression (DisMod-MR) 2.1, which estimates the prevalence, incidence, mortality, and disability-adjusted life years (DALYs) of neurological conditions for each country, region, and super-region.

The inclusion and exclusion criteria in GBD 2019 vary depending on the type of data, the disease or injury, and the risk factor. In general, data has to be population representative, at least 150-person sample size, and meet the reference or an alternative case definition accepted by the GBD (eg, some conditions will use self-report data, some will not; some conditions will use hospital data or insurance claims data and some will not).

In order to accept non-reference case definition data to bolster the number of studies and diversities of geographies included in our analyses, we used meta-regression analyses to determine whether we can systematically adjust non-reference data to reference. This is described in more detail in section 2. For example, our case definition for epilepsy specifies active epilepsy, but we accepted studies that look at lifetime recall of epilepsy diagnosis, and then systematically adjusted these data down. For dementia, we accepted non-reference criteria such as the 10/66 algorithm, NIA-AA criteria, or clinical records, and systematically adjusted these as described in section 3.2.

The Bayesian meta-regression methods are integral for modelling cause-specific mortality and morbidity, addressing data heterogeneity among different data sources and between countries, and handling uncertainties and the dynamic nature of global health.[4] These methods have allowed the integration of diverse data sources, the inclusion of expert priors such as age restrictions or remission rates, and the inclusion of predictive covariates and random effects (generally by geography). Please see the appendix sections 3.1 and 3.2 on fatal and non-fatal methods overviews. We have also included examples for dementia prevalence estimation and epilepsy death estimation in these sections.

The GBD study utilised a combination of expert opinion, literature reviews, and available data to inform the priors in their Bayesian models. These priors served as the initial assumptions about the distribution of parameters before incorporating the observed data. The specifics varied across different risk factors and diseases. The priors used in the GBD 2019 study were based on a variety of sources, including published literature, publicly available data, and contributed data. The rationale for using these priors was to incorporate as much relevant information as possible into the models, while also ensuring that the models are robust and reliable. The priors helped to guide the models, especially in situations where the data may be sparse or uncertain. In the Bayesian meta-regression tool DisMod-MR 2.1 used in GBD 2019, some prior distributions were revised based on simulation studies showing that less informative priors helped to improve the coverage of uncertainty intervals. This indicates that the choice of priors was guided by a desire to improve the accuracy and reliability of the estimates. The priors used in the GBD 2019 study were not explicitly elicited in the way that priors were sometimes elicited in other contexts, such as through expert elicitation methods. Instead, the priors were derived from a variety of data sources, including censuses, household surveys, civil registration and vital statistics, disease registries, health service use, air pollution monitors, satellite imaging, disease notifications, and other sources. This approach allowed for a comprehensive and systematic assessment of the available data, which is crucial for a study of this scale and complexity. This framework inherently involved updating priors with new data to generate posterior distributions.

For input data sources, GBD 2019 included studies that met the following criteria:

- Reported primary data on mortality, prevalence, incidence, or severity of a disease, injury, or risk factor
- Used a representative sample of the population of interest (including people of both sexes and all ages living in countries of NAME)
- Used a standardised and validated measurement tool or diagnostic criteria
- Reported age- and sex-specific data or provided enough information to disaggregate by age and sex
- Reported uncertainty estimates or provided enough information to calculate them

For risk-outcome pairs, GBD 2019 included those that met the following criteria:

- Had convincing or probable evidence of a causal relationship based on specific criteria such as the criteria of the World Cancer Research Fund or the Bradford Hill criteria
- Had at least one meta-analysis of epidemiological studies that estimated the relative risk of the outcome associated with the risk factor
- Had sufficient data on the exposure distribution and the outcome burden to estimate the population attributable fraction

For ad-hoc exclusions, GBD 2019 excluded some data sources or risk-outcome pairs that did not meet the quality standards or had major limitations. For example, GBD 2019 excluded data sources that had implausible values, high heterogeneity, or inconsistent trends. We vetted data and results with geographical expert collaborators to confirm study quality, and that many conditions employed systematic outlier methods (eg, for clinical data, outliers identified if age-standardised incidence or prevalence is more than two median absolute deviations from the median age-standardised incidence or prevalence across location-years).

Core summary results include deaths, years of life lost (YLLs), years lived with disability (YLDs), disability-adjusted life years (DALYs), prevalence, incidence, life expectancy, healthy life expectancy (HALE), causes of death and illness, risk factor-attributed DALYs and deaths, and some other measures. Data are categorised according to age (including numerous age groups), sex (including males, females, and both sexes), risk factor (including 87 modifiable risk factors), year (including annual results for all measures from 1990 to 2019), and location (including 21 regions and 204 nations) with different metrics (including absolute number, rate per 100,000, and percent). We have compared results for neurological diseases in North Africa and Middle East with the rest of the world.

1.2. Regions and nations

We have compared results for neurological conditions in North Africa and Middle East with other regions in the world. GBD produces estimates for 204 countries and territories grouped into 21 regions and seven super-regions (Table S3). The seven GBD super-regions are central Europe, eastern Europe, and central Asia; high income; Latin America and the Caribbean; North Africa and Middle East; South Asia; southeast Asia, east Asia, and Oceania; and sub-Saharan Africa. The list of countries in each region is as follows:

Table S3. Global Burden of Disease Study 2019 (GBD 2019) classification of countries and regions

GBD super regions	GBD classified sub-regions and nations
The North Africa and Middle East	● The Middle-East: Afghanistan, Bahrain, Iran (Islamic Republic of), Iraq, Jordan, Kuwait, Lebanon, Libya, Palestine, Oman, Qatar, Saudi Arabia, Syrian Arab Republic, Türkiye (Turkey), United Arab Emirates, Yemen;<br>● North Africa: Algeria, Egypt, Morocco, Sudan, Tunisia;
Southeast Asia, East Asia, and Oceania	● East Asia: China, the Democratic People's Republic of Korea, Taiwan (Province of China);<br>● Southeast Asia: Cambodia, Indonesia, Lao People's Democratic Republic, Malaysia, Maldives, Myanmar, Philippines, Sri Lanka, Thailand, Timor-Leste, Viet Nam;<br>● Oceania: American Samoa, Cook Islands, Fiji, Guam, Kiribati, Marshall Islands, Micronesia (Federated States of), Nauru, Niue, Northern Mariana Islands, Palau, Papua New Guinea, Samoa, Solomon Islands, Tokelau, Tonga, Tuvalu, Vanuatu;
South Asia	● South Asia comprises Bangladesh, Bhutan, India, Nepal, and Pakistan;
Sub-Saharan Africa	● Central Sub-Saharan Africa: Angola, Central African Republic, Congo, Democratic Republic of the Congo, Equatorial Guinea, and Gabon;<br>● Eastern Sub-Saharan Africa: Burundi, Comoros, Djibouti, Eritrea, Ethiopia, Kenya, Madagascar, Malawi, Mauritius, Mozambique, Rwanda, Seychelles, Somalia, South Sudan, United Republic of Tanzania, Uganda, Zambia;<br>● Southern Sub-Saharan Africa: Botswana, Eswatini, Lesotho, Namibia, South Africa, Zimbabwe;<br>● Western Sub-Saharan Africa: Benin, Burkina Faso, Cameroon, Cabo Verde, Chad, Côte d'Ivoire, Gambia, Ghana, Guinea, Guinea-Bissau, Liberia, Mali, Mauritania, Niger, Nigeria, Sao Tome and Principe, Senegal, Sierra Leone, Togo).
Latin America and Caribbean	● Caribbean: Antigua and Barbuda, Bahamas, Barbados, Belize, Bermuda, Cuba, Dominica, Dominican Republic, Grenada, Guyana, Haiti, Jamaica, Puerto Rico, Saint Kitts and Nevis, Saint Lucia, Saint Vincent and the Grenadines, Suriname, Trinidad and Tobago, United States Virgin Islands;

Table S3. Global Burden of Disease Study 2019 (GBD 2019) classification of countries and regions	
**GBD super regions**	**GBD classified sub-regions and nations**
	● Andean Latin America: Bolivia (Plurinational State of), Ecuador, Peru;<br>● Central Latin America: Colombia, Costa Rica, El Salvador, Guatemala, Honduras, Mexico, Nicaragua, Panama, Venezuela (Bolivarian Republic of);<br>● Tropical Latin America: Brazil, Paraguay;
Central Europe, Eastern Europe, and Central Asia	● Central Asia: Armenia, Azerbaijan, Georgia, Kazakhstan, Kyrgyzstan, Mongolia, Tajikistan, Turkmenistan, and Uzbekistan;<br>● Central Europe: Albania, Bosnia and Herzegovina, Bulgaria, Croatia, Czechia, Hungary, North Macedonia, Montenegro, Poland, Romania, Serbia, Slovakia, Slovenia;<br>● Eastern Europe: Belarus, Estonia, Latvia, Lithuania, Republic of Moldova, Russian Federation, Ukraine;
High-income	● High-income Asia Pacific: Brunei Darussalam, Japan, the Republic of Korea, and Singapore;<br>● Australasia: Australia, and New Zealand;<br>● Western Europe: Andorra, Austria, Belgium, Cyprus, Denmark, Finland, France, Germany, Greece, Iceland, Ireland, Israel, Italy, Luxembourg, Malta, Monaco, Netherlands, Norway, Portugal, San Marino, Spain, Sweden, Switzerland, United Kingdom;<br>● Southern Latin America: Argentina, Chile, Uruguay;<br>● High-income North America: Canada, Greenland, United States of America;

There are some variations between the GBD 2019 and the World Bank in classifying countries of North Africa and Middle Eastern area. The GBD 2019 classified Israel among high-income Western European nations; and Sudan among Eastern Sub-Saharan African nations. The World Bank classifies Türkiye among European and Central Asian nations; Afghanistan among South Asian nations; and Djibouti and Malta among Middle Eastern and North African nations. It is worth noting that the GBD classification differed from the World Health Organization or the World Bank classification of countries in this region, which is usually abbreviated to MENA.

1.3. GBD 2019 neurological data changes

The GBD 2019 categorisation did not change compared to the GBD 2017 in terms of neurological disorders, which both include Alzheimer's disease and other dementias, Parkinson's disease, idiopathic epilepsy, multiple sclerosis, headache disorders (including migraine and tension-type headache), motor neuron disease, and other rare neurological disorders. However, as with the previous GBD study of neurological disorders,[5] we added data on stroke (including ischaemic stroke, haemorrhagic stroke, and subarachnoid haemorrhage), meningitis, encephalitis, tetanus, head injuries and spinal injuries.

1.4. Literature search

To evaluate the availability of evidence on the burden of neurological conditions among 21 countries of NAME, we did a review of the published scientific literature in PubMed for relevant reports published in any language up to January 1, 2024, using the following search terms:

"(encephalitis[ti] OR meningitis[ti] OR tetanus[ti] OR ((brain[ti] OR central nervous system[ti]) cancer[ti]) OR head injur[ti] OR spinal injur*[ti] OR stroke[ti] OR subarachnoid haemorrhage[ti] OR subarachnoid hemorrhage[ti] OR Alzheimer*[ti] OR dementias[ti] OR Parkinson*[ti] OR epilepsy[ti] OR multiple sclerosis[ti] OR headache[ti] OR migraine[ti] OR motor neuron disease[ti] OR Amyloid lateral sclerosis[ti] OR neurological*

disorder[tw] OR neurological disease*[tw]) AND (Middle East[ti] OR north Africa[ti] OR Afghanistan[ti] OR Algeria[ti] OR Bahrain[ti] OR Egypt[ti] OR Iran[ti] OR Iraq[ti] OR Jordan[ti] OR Kuwait[ti] OR Lebanon[ti] OR Libya[ti] OR Morocco[ti] OR Palestine[ti] OR Oman[ti] OR Qatar[ti] OR Saudi Arabia[ti] OR Sudan[ti] OR Syria*[ti] OR Tunisia[ti] OR Turkey[ti] OR Türkiye[ti] OR Emirates[ti] OR Yemen[ti]) AND (Population based[tw] OR community based[tw] OR community dwelling*[tw] OR population wide[tw]) AND (burden[tw] OR disability[tw] OR mortality[tw] OR death*[tw] OR inciden*[tw] OR prevalen*[tw] OR dalys[tw] OR ylls[tw] OR ylds[tw] OR "population attributable fraction"[tw] OR risk factor*[tw])."*

2. Methodological summary of neurological conditions and definition of risk factors

GBD reference definitions for all conditions included in this analysis are listed in Table S4 and risk factor exposure in Table S5 (using definitions from GBD 2019 capstone appendices). Twenty risk factors were categorised into four levels (Table S6).

Table S4. Neurological conditions included in the present study and their definitions based on the Global Burden of Disease Study 2019 (GBD 2019)

Condition	GBD case definition
Brain and central nervous system cancer	Malignant neoplasms of the brain and central nervous system using the ICD-10 classification system.
Dementia	A progressive, degenerative, and chronic neurological disorder typified by memory impairment and other neurological dysfunctions. The case definition is either the DSM-III, IV, or V, or ICD classification.
Encephalitis	A disease caused by acute inflammation of the brain. The case definition follows ICD-10 diagnostic criteria.
Epilepsy (idiopathic)	A condition characterised by recurrent (2+) epileptic seizures, unprovoked by any immediate identified cause. The case definition follows the International League Against Epilepsy (ILAE) definition for active epilepsy, which stipulates one epileptic seizure in the previous five years, regardless of antiepileptic drug treatment. Idiopathic epilepsy refers to epilepsy of unknown origin (as opposed to secondary epilepsy due to conditions such as stroke, brain injury, etc).
Meningitis	A disease caused by inflammation of the meninges, which is the protective membrane surrounding the brain and spinal cord. It is typically caused by an infection in the cerebrospinal fluid. Meningitis includes viral meningitis and bacterial meningitis, and the case definition follows ICD-10 diagnostic criteria.
Migraine	A disabling primary headache disorder characterised by recurrent moderate or severe unilateral pulsatile headaches. The case definition follows the International Classification of Headache Disorders (ICHD)-3.
Motor neuron disease	A set of chronic, degenerate, and progressive neurological conditions typified by the destruction of motor neurons and subsequent deterioration of voluntary muscle activity, the most common form of which is amyotrophic lateral sclerosis (ALS). The case definition follows the El Escorial criteria.
Multiple sclerosis	A chronic, degenerative, and progressive neurological condition typified by damage to the myelin sheaths. The case definition follows McDonald's criteria, though other criteria such as Poser or Schumacher are also treated as reference, as is clinical neurological exam diagnosis.

Table S4. Neurological conditions included in the present study and their definitions based on the Global Burden of Disease Study 2019 (GBD 2019)

Condition	GBD case definition
Other neurological disorders	A residual category of neurological conditions not explicitly estimated in the GBD 2019 studies. These broadly include movement disorders, peripheral nerve disorders, myopathies, degenerative disorders, and others. These conditions are identified via ICD-9 and ICD-10 coding (in death data).
Parkinson's disease	A chronic, degenerative, and progressive neurological condition typified by loss of motor mobility and control - most notably tremors. The case definition follows the Gelb Criteria and requires the presence of at least two of the four primary symptoms (tremors, bradykinesia, stiffness of limbs and torso, posture instability).
Spinal cord injury	This is an N-coded injury (nature of injury) that includes spinal cord lesion at or below the neck level.
Stroke	Defined according to World Health Organization (WHO) criteria.<br>First-ever ischaemic stroke, intracerebral haemorrhage, and subarachnoid haemorrhage were defined from the day of incidence through 28 days and separately modelled survival beyond 28 days.[3]
Tension-type headache	A headache condition characterised by dull, non-pulsatile, diffuse, band-like pain of mild to moderate intensity in the head or neck. Classification follows ICHD-3.
Traumatic brain injury	This is an N-coded injury (nature of injury) that involves injury to the head that can lead to short-term and in some cases long-term damage to the brain.

Note: Details of input data and methods for estimation of epidemiological figures of neurological conditions are available in Appendix 1 of the article: Global burden of 369 diseases and injuries in 204 countries and territories;[1] on pages 305–307 for idiopathic epilepsy, pages 693–703 for meningitis, pages 704–711 for encephalitis, pages 718–720 for tetanus, pages 835–843 for strokes and its subtypes (including ischaemic stroke, intracerebral haemorrhage, and subarachnoid haemorrhage), pages 295–299 and 963–971 for Alzheimer's disease and other dementias, pages 300–304 and 972–978 for Parkinson's disease, pages 308–301 and 979–986 for multiple sclerosis, pages 309–313 and 987–993 for motor neuron disease, pages 994–1002 for headache disorders (including migraine and tension-type headache), pages 304–316 and 1003 for other neurological disorders, and pages 1350–1361 for head/spinal injuries.[1]

Table S5. Risk factor exposure definitions in the Global Burden of Disease Study 2019

Risk factors	Exposure definition
Ambient particulate matter pollution	population-weighted annual average mass concentration of particulate matter <2·5 mm in diameter ($PM_{2\cdot5}$) in a cubic metre of air >2·4-5·9 mg/m^3.
Dietary risks	a composite risk factor consisting of suboptimal exposure to dietary factors including fruits, vegetables, whole grains, nuts and seeds, fibre, omega-3 fatty acids, polyunsaturated fatty acids, calcium, milk, legumes, red meat, processed meat, sugar-sweetened beverages, trans fatty acids, and sodium.
High alcohol use	grams per day of pure alcohol consumed among current drinkers greater than the age-, sex-, and region-specific TMREL.
High body mass index	body mass index >20-23 kg/m2 in adults >20 years of age.

Table S5. Risk factor exposure definitions in the Global Burden of Disease Study 2019	
**Risk factors**	**Exposure definition**
High fasting plasma glucose	serum fasting plasma glucose >4·9-5·3 mmol/L in adults >25 years of age.
High low-density lipoprotein (LDL) cholesterol	LDL-cholesterol >0·9-1·4 mmol/L in adults >25 years of age.
High systolic blood pressure	brachial systolic blood pressure (SBP) >105-115 mm Hg in adults >25 years of age.
High temperature	exposure to temperatures warmer than the temperature associated with the lowest overall mortality attributable to the risk, in a given location and year.
Household air pollution from solid fuels	proportion of individuals exposed to >2·4-5·9 mg/m^3 of $PM_{2\cdot5}$ due to the use of solid fuels for cooking, including coal, charcoal, wood, agricultural residue, and animal dung.
Kidney dysfunction	estimated glomerular filtration rate <60 mL/min/1·73 m2 and/or albumin-to-creatinine ratio ≤30 mg/g.
Lead exposure	micrograms of lead per gram of bone greater than the age-specific theoretical minimum risk exposure level (TMREL).
Low physical activity	physical activity performed by adults >25 years of age, for at least 10 minutes at a time, across all domains of life (leisure/recreation, work/household, and transport) <3000-4500 metabolic equivalent minutes per week.
Low temperature	exposure to temperatures colder than the temperature associated with the lowest overall mortality attributable to the risk, in a given location and year.
Secondhand smoke	current exposure of nonsmokers to secondhand tobacco smoke at home, at work, or in other public places.
Smoking	current or former users of any smoked tobacco product on a daily or occasional basis.

Table S6. Classification of risk factors in the Global Burden of Disease Study 2019 (GBD 2019)			
**Level 1**	**Level 2**	**Level 3**	**Level 4**
Metabolic risk factors	High systolic blood pressure		
	High LDL cholesterol		
	High fasting plasma glucose		
	High body-mass index		
	Kidney dysfunction		
Behavioural risk factors	Child and maternal malnutrition	Low birth weight and short gestation	Low birth weight
			Short gestation
	Tobacco	Smoke	
		Secondhand smoke	
	Dietary risks	Diet high in red meat	
		Diet high in sodium	
		Diet low in fibre	

Table S6. Classification of risk factors in the Global Burden of Disease Study 2019 (GBD 2019)			
**Level 1**	**Level 2**	**Level 3**	**Level 4**
		Diet low in fruits	
		Diet low in vegetables	
		Diet low in whole grains	
	Low physical activity		
	Alcohol use		
Environmental/ Occupational risk factors	Air pollution	Particulate matter pollution	Ambient particulate matter pollution
			Household air pollution from solid fuels
	Non-optimal temperature	Low temperature	
		High temperature	
	Other environmental risks	Lead exposure	
Global Burden of Disease Study 2019 categorises 87 risk factors into three level 1 risk factors, 20 level 2 risk factors, 41 level 3 risk factors, and 22 level 4 risk factors. Risk factors lacking sufficient or quality data related to any neurological condition are not listed here.			

Although details of modelling methods can vary by condition, standard methods are as follows:
We accepted data for a given disease that meets our gold standard (reference) case definition as well as alternate case definitions (eg, for epilepsy, our gold standard is active epilepsy, but we accepted studies that look at lifetime recall). Then, we used a meta-regression to systematically adjust non-reference case definition data to the reference. In that way, we could include data that were collected using heterogeneous methods. To determine how much each alternative case definition should be adjusted, we used a tool called MR-BRT (meta-regression: Bayesian, Regularized, Trimmed).6 A meta-regression is like a meta-analysis, but includes covariates (fixed and random effects), and accounts for dependent variable uncertainty (measurement error). MR-BRT combines functionality for linear regressions, mixed effects models, meta-analyses, Bayesian priors, and flexible model fits with splines. It also allows for outlier trimming using a likelihood estimator and for automated covariate selection. We used log ratio or logit difference network or intercept-only meta-analyses. Input data consisted of the logit difference between matched pairs of data with different case definitions from similar geographies and collection periods for a given measure (eg, prevalence). Model results were used to adjust non-reference case definition data to the reference.

Data that are not sex-specific or provided in greater than 25-year age bins are sex- and/or age split. To split “both sex” data into male and female, we borrowed information from other studies in the dataset that provided male and female prevalence or incidence. We used the ratio of within-study male to female prevalence or incidence from these other studies as input to a meta-regression model to calculate the average proportion male and female, and applied this to “both sex” data after accounting for population structure (eg, some countries have higher proportion of males or females in their respective populations for different age groups). The following equations were used:

Male prevalence:

$$Prevalence_{male} = prevalence_{both} * (population_{both} / (population_{male} + ratio * population_{female}))$$

Female prevalence:

$$Prevalence_{female} = ratio * prevalence_{male}$$

We then splitted data with greater than 25-year age bins into 5-year age bins using the global age pattern from an initial DisMod-MR model where only granular age data were used as inputs.

Then, we input all data into a DisMod-MR model and got an initial global fit across all data regardless of collection year or geography. At this stage, we also got coefficients for predictive covariates. The global fit was passed down to super-region models as a prior, and models were run for each super-region with input data only from the respective super-region – then the same for regions and for countries. Random effects on location inform whether the prior passed down from the previous level of the geographic cascade should be higher or lower than the original fit. In this way, data from all locations informed the model fitted for North Africa and Middle East, in combination with data specific to North Africa and Middle East countries, and the data was harmonised prior to model input to account for differing case definitions.

3. Modelling

3.1. Fatal disease modelling

The Cause Of Death Ensemble modelling (CODEm) framework was used to model most cause-specific death rates and Years of Life Lost (YLLs) owing to premature mortality, which relies on four key components:

- Data identification and gathering to have some signal of the true epidemiological process.
- Development of plausible models to predict well-documented associations in the estimates.
- Assessment of the out-of-sample predictive validity for all individual models, which are ranked for use in the ensemble modelling stage.
- Evaluation and selection of the best model with the highest out-of-sample predictive validity.

To develop pool models in the CODEm framework, four statistical models were used including linear mixed effects regression (LMER) models of the natural log of the cause-specific death rate, LMER models of the logit of the cause fraction, spatiotemporal Gaussian process regression (ST-GPR) models of the natural logarithm of the cause-specific death rate, and ST-GPR models of the logit of the cause fraction. Implausible signs on coefficients or unstable coefficients may strengthen multi-collinearity between covariates. Therefore, each combination is tested for statistical significance (covariate coefficients with a p-value < 0·05) and plausibility (the coefficients with expected directions as to the literature). We used Markov Chain Monte Carlo (MCMC) for DisMod, Logit Model Regression (LMR) for MR-BRT, and ensemble approach for CODEm where component models were a combination of mixed-effect models and space-time Gaussian process regression models. For most of the conditions in the current study, we used DisMod to get prevalence and incidence. We ran 5000 samples in DisMod and used the final 1000 for estimation.

For fatal modelling, deaths for most conditions included in our analysis were estimated using CODEm. Input data consists primarily of ICD-9 or ICD-10 coded vital registration data, supplemented in some cases by verbal autopsy data, police records, or registry data. Insufficiently defined ICD-coded data were redistributed to other underlying causes, and data went through noise-reduction to account for variation in temporal trends due to small numbers. Linear mixed-effect component models were run with all input data and used to select covariates significantly associated with the disease outcome. A subset of subsequent models run space-time smoothing, which leads to additional smoothing by borrowing information over space, time, and age, and Gaussian Process Regression that improves predictions for locations where input data were available. Component models were assessed for performance (root mean squared error and percent of correct predictions of time trend from adjacent points) and were weighted in a final ensemble model that maximises out-of-sample predictive validity. Final model uncertainty comes from regression parameters, variance around input data appoints, and heterogeneity of component models.

3.1.1 Modelling steps for idiopathic epilepsy death estimation (as an example of fatal modelling processes)
ICD-9 and ICD-10 coded vital registration and verbal autopsy data as well as China mortality surveillance data were input into CODem models. Separate models were run for male and female. Predictive covariates added to the model included (Table S7):

Table S7. Predictive covariates used in the epilepsy fatal modelling process

Level	Covariate	Direction
1	Pigs (per capita)	+
	SEV scalar: epilepsy	+
	Mean systolic blood pressure (mmHg)	+
2	Health access and quality index	-
	Mean body mass index	+
	Mean serum total cholesterol (mmol/L)	+
3	Cumulative cigarettes (10 years)	+
	Cumulative cigarettes (5 years)	+
	Education (years per capita)	-
	Log LDI (per capita)	-
	Socio-demographic Index	-

Note: The level signifies strength of the evidence of association, where covariates with the strongest link to epilepsy are level 1, etc. The direction provides prior knowledge for the expected relationship, for example higher systolic blood pressure is expected to be predictive of more epilepsy deaths.

Covariates were added to mixed effect models sequentially and retained if they were significantly associated with the outcome (eg, epilepsy deaths). The final set of covariates selected by the model and their respective influence included systolic blood pressure, socio-demographic index, pigs (per capita), mean body mass index, the log-transformed SEV scalar for idiopathic epilepsy, healthcare access and quality index, education (years per capita), lag-distributed income (per capita), and cumulative cigarettes (5 and 10 years). An example plot of the relative influence of these covariates in one of the final ensemble models for idiopathic epilepsy is displayed below:

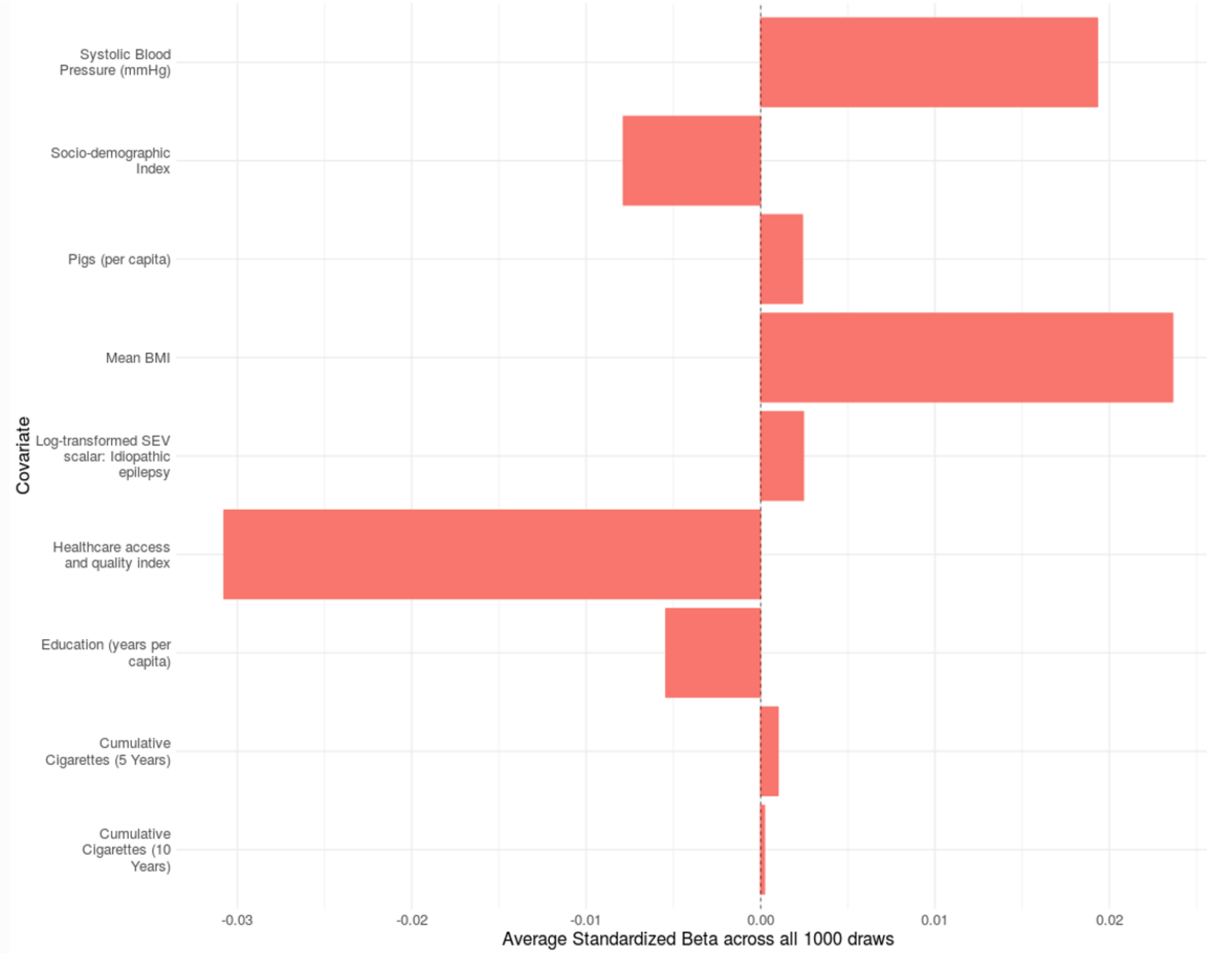

Figure S1. Plot of the relative influence of covariates for idiopathic epilepsy

The flowchart for epilepsy death and YLL modelling is shown below:

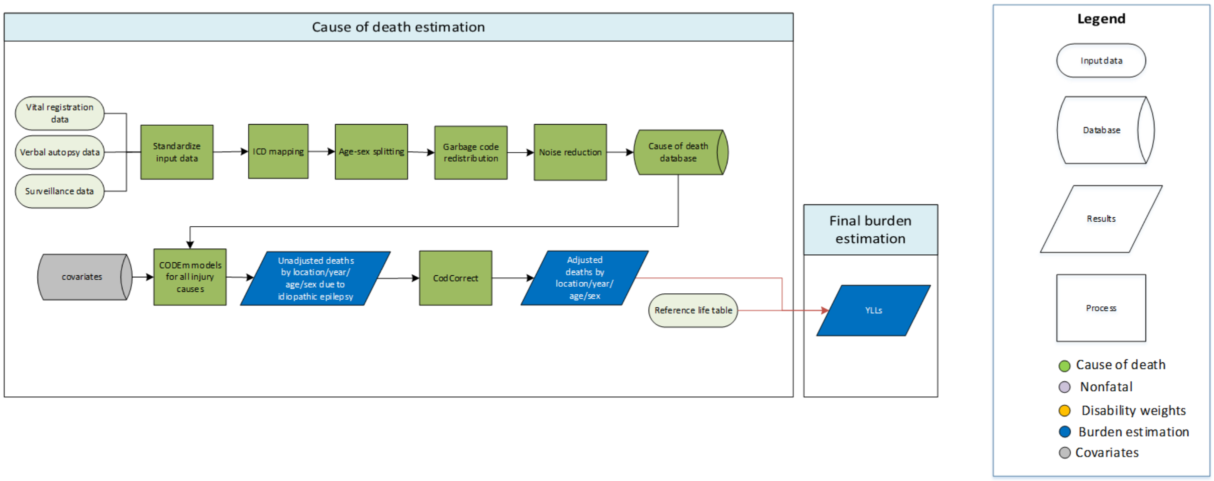

Figure S2. Flowchart of modelling steps to epilepsy-related death and years of life lost (YLLs)

3.2. Non-fatal disease modelling

The best available data on incidence, prevalence, and mortality were gained to estimate the burden of non-fatal neurological conditions. Estimates of the incidence, prevalence, and Years Lived with Disability (YLDs) of neurological conditions were generated with the DisMod-MR modelling tool version 2.1. DisMod-MR is a Bayesian geospatial disease modelling software that uses data on diverse parameters associated with each disease, the epidemiological relationships between the parameters, and geospatial relationships to produce estimates. We input data into either a single-parameter or compartmental Dismod-MR model and get an initial global fit across all data regardless of collection year or geography. At this stage, we also get coefficients for predictive covariates. Then, the global fit is passed down to super-region models as a prior, and models were run for each super-region, such as NAME, with input data only from the respective super-region – then the same for regions and for countries. Random effects on location inform whether the prior passed down from the previous level of the geographic cascade should be higher or lower than the original fit. Once models were fit for the lowest level of geography (national or subnational), final models were produced by aggregating - for example, the final prevalence model for the NAME region is the aggregate across all countries within NAME.

Of note, DisMod-MR does not produce YLDs, but the prevalence generated from DisMod is used to split total prevalence by disease into different severities, for instance, dementia is split into mild, moderate, and severe dementia, and then respective disability weights for each severity-specific health state is multiplied by prevalence by year-age-sex-location to get YLDs. Disability weights were produced using population-based surveys asking participants to make pairwise comparisons of the severity of different health states.[7]

3.2.1. Modelling steps for non-fatal dementia estimation (as an example of non-fatal modelling processes)

Dementia is an example that includes the key standard techniques of data collection, adjustment of non-reference case definition data, sex splitting, age splitting, severity splitting, and multiplying by DWs to get YLDs (flow diagram).

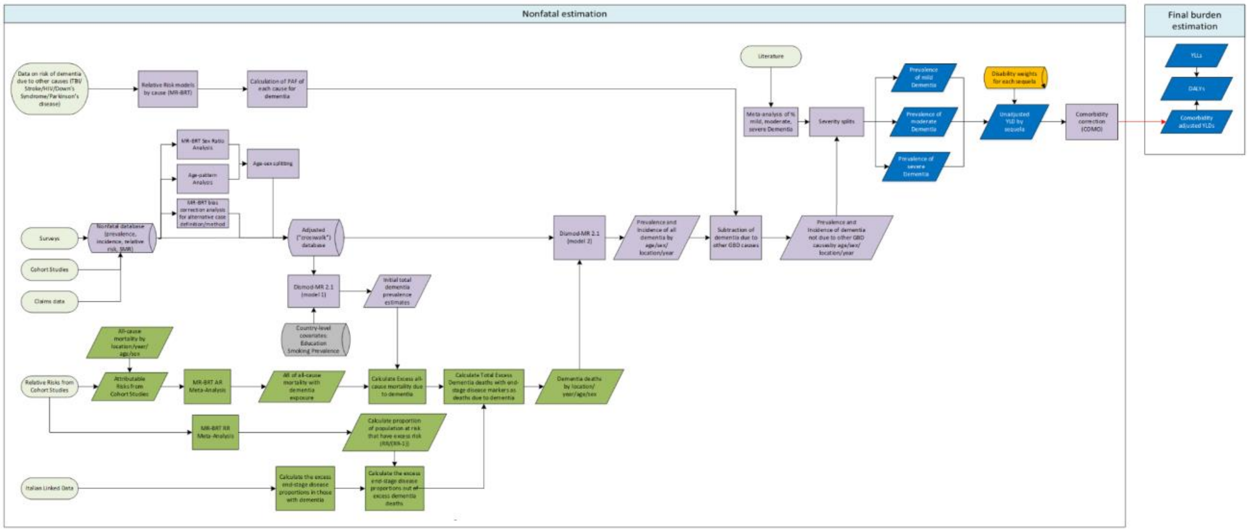

Figure S3. Flowchart of modelling steps for non-fatal dementia estimation

We estimated total dementia prevalence and incidence using DisMod-MR. Data on prevalence or incidence from population-based studies were identified via systematic review or expert collaborator advice. We used the Diagnostic and Statistical Manual of Mental Disorders (DSM) III, IV or V or ICD case definitions as the reference.

The DSM definition includes a combination of cognitive and functional deficits. Screening instruments were heterogeneous and included the Mini Mental State Examination (MMSE), Clinical Dementia Rating scale (CDR), and the Geriatric Mental State (GMS). Incidence data were included in dementia modelling for the first time in GBD 2019; previously only prevalence data were used. "Both" sex data and wide age-bin data were sex split and age spit as described in section 2. Non-reference case definitions were adjusted to the gold standard. These included:

- Diagnosis based on the result of an algorithm
- Diagnosis from general practitioner data
- Diagnosis based on clinical records
- Diagnosis with the 10/66 algorithm
- Diagnosis with the NIA-AA criteria

To adjust non-reference data, we performed a logit difference network crosswalk using meta-regression-Bayesian, Regularized, Trimmed (MR-BRT). Model inputs were the logit difference between data using different case definitions matched by sex, measure, location, and approximately the same age and years of collection. The results of this analysis are displayed in Table S8 below:

Table S8. Results of non-reference adjusted meta-regression analysis for dementia estimations

**Data input**	**Reference or alternative case definition**	**Gamma**	**Beta coefficient, logit (95% UI)***	**Adjustment factor†**
DSM or ICD case definition	Ref	0·34	---	---
Clinical records diagnosis criteria	Alt		-0·05 (-0·72 – 0·61)	0·95
Algorithm diagnosis criteria (AGECAT)	Alt		0·08 (-0·59 – 0·74)	1·08
NIA-AA diagnosis criteria	Alt		0·51 (-0·16 – 1·17)	1·67
10/66 algorithm diagnosis criteria	Alt		0·97 (0·30 – 1·64)	2·64
GP records used for diagnosis	Alt		-1·21 (-1·88 – -0·54)	0·30

UI, uncertainty interval.
*MR-BRT crosswalk adjustments can be interpreted as the factor the alternative case definition is adjusted by to reflect what it would have been had it been measured using the reference case definition. If the log/logit beta coefficient is negative, then the alternative is adjusted up to the reference. If the log/logit beta coefficient is positive, then the alternative is adjusted down to the reference.
†The adjustment factor column is the exponentiated beta coefficient. For log beta coefficients, this is the relative rate between the two case definitions. For logit beta coefficients, this is the relative odds between the two case definitions.

Adjusted data were input into a DisMod-MR model to estimate dementia incidence and prevalence by year, age, sex and location. Smoking prevalence and education were included as predictive covariates in the model, with random effects on location.

Dementia due to other GBD conditions (stroke, traumatic brain injury, Parkinson's disease, Down syndrome) were subtracted from total dementia to get final dementia cases. We do this to avoid double counting health loss (e.g., health loss from dementia due to stroke is captured under stroke health states). To determine the proportion of total

dementia attributed to stroke, Parkinson's disease, traumatic brain injury, and Down syndrome, we conducted systematic reviews to identify studies on the relative risk of dementia due to these conditions and used these data as input into meta-analyses to determine relative risk of dementia due to each condition over age.[8] Results of each meta-analysis were used to calculate population attributable fractions (PAFs) with the following equation:

$$PAF = (exposure * (RR-1)) / (exposure * (RR-1) + 1)$$

PAFs were multiplied by total dementia prevalence to calculate cases of dementia due to each respective condition and subtracted from total dementia to get final dementia estimates. Final dementia prevalence was split into mild, moderate, and severe dementia based on studies that looked at the proportion of dementia in each respective severity using the Clinical Dementia Rating (CDR) scale, or other severity scales such as the Geriatric Mental State Examination (GMS), CAMDEX, Blessed test of information, memory, and concentration (BIMC), Karasawa's, DISM-III-R, the Global Deterioration Scale (GDS), or Clinical Dementia Rating Sum-of-Boxes (CSR-SB). A total of 74 sources were identified for input to a meta-regression using MR-BRT to get severity by age.

To calculate YLDs, prevalence of dementia by severity was multiplied by its respective disability weight based on the following health state definitions (Table S9):

Table S9. Dementia severity definitions

**Severity level**	**Lay description**
Mild dementia	The person has some trouble remembering recent events and finds it hard to concentrate and make decisions and plans. They may have slight to moderate difficulty engaging in community affairs, complicated hobbies, and intellectual interests.
Moderate dementia	The person retains highly learned material, but has severe memory problems, is disoriented with respect to time and sometimes place. They are severely impaired in their ability to handle problems and make social judgments. They require assistance with daily activities, and only retain simple chores and hobbies.
Severe dementia	The person has complete memory loss, no longer recognises close family members, and requires help with all daily activities, including personal care.

3.3. Choosing the appropriate model and imprecision of the estimates

We used vetted tools such as DisMod-MR and CODEm for the majority of conditions-cross-validation approaches (eg, leaving out 20% of data, RMSE, etc) were used to assess standard model settings for DisMod. For nonfatal modelling using DisMod, we took the 1000 posterior draws after model convergence to get stable estimates with estimated uncertainty; 95% uncertainty interval (UI) is defined as the 0·025 and 0·975 quantiles of the draws. Choosing 1000 posterior samples is a common practice for balance and usually strikes a good trade-off between accuracy and efficiency, which is enough to capture the distribution without being computationally overwhelming. We have computed 1000 draws of the posterior for all elements that feed into GBD estimates. We did this to retain correlation. We have tested dropping this down to 500 draws for GBD 2021 and found this produced near identical results to those computed from 1000 draws.

For CODEm, candidate models were evaluated using out-of-sample predictive validity and cross-validation. Out-of-sample predictive validity measures how well the model predicts data that were not used in the model fitting, while cross-validation splits the data into training and testing sets and compares the model predictions with the testing data. Then, the final model(s) were selected based on the lowest RMSE and the lowest complexity penalty. The complexity penalty is a measure of how complex the model is, based on the number of parameters and random effects. The model with the lowest RMSE and the lowest complexity penalty is considered the best fit for the data.

In GBD analysis, the use of "uncertainty intervals" is a terminology choice. While Bayesian analysis often employs "credibility intervals," the concept is similar. Both intervals express the uncertainty around estimates. The term "uncertainty interval" may be preferred in this context for clarity across a broader audience, as it is not limited to the Bayesian framework. It is essentially addressing the same idea—acknowledging the range within which the true values might lie.

The GBD study accounts for the imprecision of estimates through several methods:

- Uncertainty intervals: GBD provides uncertainty intervals around its estimates, indicating the range within which the true values are likely to fall. This accounts for the imprecision in the data and modelling.
- Sensitivity analysis: GBD conducts sensitivity analyses to assess how changes in input parameters impact the estimates. This helps in understanding the robustness of the results and considering potential variations.
- Data quality metrics: GBD incorporates data quality metrics to assess the reliability of input data. Lower-quality data may lead to more imprecise estimates.

These approaches collectively address the inherent imprecision in the data and modelling processes, enhancing the transparency and reliability of the GBD estimates.

GBD estimates were assessed for stability through rigorous methods and ongoing validation. Stability was ensured by: a) data quality assurance thorough evaluation and validation of data sources to ensure accuracy; b) consistency checks with regular checks to maintain consistency across different sources and time periods; c) sensitivity analysis by examining how variations in input parameters affect the estimates, providing insights into stability; and d) peer review through inclusion of external experts for critical review ensures robustness and stability of the estimates. This continual process of validation and refinement contributed to the stability and reliability of GBD estimates.

In GBD analysis, for space time smoothing in CODEm, the temporal component relied on a tricubic function or exponential decay function. We run individual models per estimation year in DisMod. A common assumption regarding the time effect was that the relationships between risk factors, diseases, and demographic factors remain relatively stable over time. This assumption allowed for the extrapolation of trends and estimates into the future. However, this assumption might not always hold, and we did periodic reassessment and adjustments to account for any significant shifts or changes.

Further details are discussed in section 3 of Appendix 1 of the article: Global burden of 369 diseases and injuries in 204 countries and territories (on pages 48 to 56),[1] and Foreman et al.[9]

3.4. Heterogeneities

The inconsistency of global data for different conditions in different regions and nations has always been challenging. Seven issues create unique challenges: sparse or missing data for an outcome in a location; conflicting data (with non-overlapping confidence intervals) for the same outcome in the same place; heterogeneous case definitions, assays or instruments; non-sampling error; administrative data that exclude certain groups; limited predictive power of available covariates for an outcome; and the need to deal with anomalous studies or data points.[10] The wide uncertainty intervals for some of the findings reflect the heterogeneity of data sources in the North Africa and Middle East region and its countries and also incorporates uncertainty from modelling processes.

In the GBD study, handling heterogeneity of data in North Africa and Middle East for neurological conditions involved several key strategies:

- Data sources: GBD aggregates data from various sources, including national health surveys, vital registration systems, scientific literature, and other relevant databases. This helps capture the diversity of data available in the region.
- Adjustment for data quality: GBD assesses the quality of data sources and adjusts estimates accordingly using meta-regressions to test for and systematically adjust up or down the data collected using non-gold

standard case definitions (see the section describing MR-BRT "crosswalking" methodology). This is crucial for regions like North Africa and the Middle East where data quality may vary.
- Bayesian modelling: GBD often employs Bayesian statistical models to account for uncertainty and variability in data. This helps in providing more robust estimates, especially when dealing with heterogeneity. In particular, the DisMod-MR tool allows for multiple data points from different studies for the same demographics (eg, location, age, sex, year), and accounts for within-study variance to weight the relative importance of each data point (eg, larger sample size lends data more weight in the model).
- Subnational analysis: GBD recognises the diversity within regions and countries. Subnational analysis allows for a more granular understanding of the burden of disease, considering variations at a more localised level. This has been done for some of the countries in the region, such as Iran, and is in development for many others.
- Collaboration with experts: GBD involves collaboration with regional experts to ensure that local knowledge and nuances are considered in the estimation process.

These strategies collectively contribute to handling the heterogeneity of data in North Africa and the Middle East.

During the last three decades, GBD methodology evolved to build stronger tools to address these challenges. For instance, concerning data heterogeneity, mixed effects models were used for a linear random effects component and new analyses in place of classic approximations, such as log-linear models were used for nonlinear observation models.[6,11] GBD estimates are now internationally recognised as one of the most reliable health burden estimates and are officially used by the WHO for top-level decision-making.[10,12]

3.5. GBD global standard population

For GBD 2019, the non-weighted mean of age-specific proportional distributions from the population estimates for all national locations with a population greater than five million in 2019 was used to generate a standard population age structure.[13] Age-standardised rates in the GBD 2019 were calculated by using the GBD world standard population.[14]

In the GBD 2019 study, age-standardised rates were estimated using a method called direct standardisation:
- Age groups: The population is divided into specific age groups.
- Standard population: A standard population structure is chosen. This is a hypothetical population with known age distribution.
- Calculation: The age-specific rates in the studied population are applied to the standard population. This standardised the rates, making them comparable across different populations.
- Weighting: To account for variations in age distribution between the standard and studied populations, weights are applied to each age group.

This method allows for a meaningful comparison of rates across populations with different age structures.

3.6. Risk factor estimation

In GBD 2019, Meta-Regression— Bayesian, Regularised, Trimmed (MR-BRT) and Population Attributable Fractions (PAFs) were used to model relative risks.[2] In MR-BRT, a network random effects meta-regression was used. PAF is defined as "the proportion of disease cases that would not occur in a population if an individual risk factor were to be eliminated."[15] The attributable burden is calculated as the PAF multiplied by a total burden that is dementia incidence to dementia prevalence ratio.[2,9,15] Details of the method of risk estimates are available in Appendix 1 of the article "Global burden of 87 risk factors in 204 countries and territories, 1990–2019."[2]

Risk factors with a continuous exposure distribution with determined effect size include lead, high fasting plasma glucose, high LDL cholesterol, high systolic blood pressure, and high body-mass index, for which RRs were modelled with log-linear assumption using MR-BRT. For categorical or binary risk factors, such as all dietary risk

factors, low physical activity, and air pollution, non-linear functions using cubic splines were used to allow for monotonically increasing or decreasing.

The six analytical steps in the GBD 2019 method for estimating PAF included: (1) nineteen risk–outcome pairs meeting criteria for convincing or probable evidence on the basis of research studies were included; (2) based on published systematic reviews, GBD reviews, and meta-regressions, relative risks were estimated as a function of exposure; (3) based on all available data sources levels of exposure in each age/sex/location/year included in the study were estimated using spatiotemporal Gaussian process regression, DisMod-MR 2.1, a Bayesian meta-regression method, or alternative methods; (4) the level of exposure associated with minimum risk, called the theoretical minimum risk exposure level, was determined from published trials or cohort studies; (5) attributable DALYs were computed by multiplying PAFs by the relevant outcome quantity for each age, sex, location, and year. (6) PAFs and attributable burden for combinations of risk factors were estimated considering the mediation of different risk factors through other risk factors.[3] The PAF for each condition is calculated with the following equation, where exposure is defined as the prevalence of the condition.[2] The mediation analysis is reported by individual-level data from prospective cohort studies on the joint effects of combinations of risk factors."[3]

$$Population\ Attributable\ Fractions\ (PAF) = exposure * (RR - 1)\ /\ ([exposure * (RR - 1)] + 1)$$

3.6.1. Determine relative risks

The relative risk (RR) by level of exposure or by cause for mortality or morbidity can be found in published and unpublished primary studies or in secondary studies that summarise RRs. We collated information from RCTs, cohort, pooled cohort, and case- control studies, and used these data to determine the RR for the risk-outcome pairs included in GBD 2019. For most risks, data from pooled cohorts or meta-analyses of cohorts were used; in the case of the risk of cataracts from household air pollution (HAP), cohort data were not available, and instead we used case-control data. We estimated RRs of mortality and morbidity for all risk factors for which we determined attributable burden by using RR and exposure. We incorporated RRs from studies that controlled for confounding but not for factors along the causal pathway between exposure and outcome. For risk-outcome pairs with evidence available for only one element of mortality or morbidity, we generally assumed that the estimated RRs applied equally to both. Given evidence of statistically different RRs for mortality and morbidity, we incorporated different RRs for each. We did not find that RRs were consistently higher or lower for mortality compared with morbidity. Details and citation information for the data sources used for RRs are provided in searchable form through a web-tool (http://ghdx.healthdata.org/). Available data sources for determining RRs varied across risks.

For the following risks estimated from a continuous exposure distribution in which the effect size was reported by categories in pooled or meta-analysis studies, we converted those categories to RR per unit increase in exposure and assumed a linear increase in the log of the RR and exposure: ambient ozone pollution, lead, high fasting plasma glucose, high LDL cholesterol, high systolic blood pressure, and high body-mass index. Many meta-analyses convert RRs to per unit increase for convenience, particularly when studies choose different categories that could not otherwise be compared. If samples in the primary studies at high levels of exposure were sufficient to inform the shape of the tail of the distribution, we applied a cap to the maximum RR by using the midpoint of the last category for which an RR was reported.

In GBD 2019, for a selected set of continuous risk factors, we modelled RRs using meta-regression— Bayesian, regularised, trimmed (MR-BRT), relaxing the log-linear assumption to allow for monotonically increasing or decreasing but non-linear functions using cubic splines. The MR-BRT program is a set of wrappers customised for global health problems that use the open source mixed effects package (https://github.com/zhengp0/limetr). Risk factors for which we undertook this re-analysis include: all dietary risk factors, low physical activity, kidney dysfunction, and air pollution. Because knot placement can affect the shape of the risk function when modelling with a cubic spline, we generated a wide range of knot placements and created an ensemble across these different knot placements. We also included in the final estimation 10% trimming of the data to avoid the results being

sensitive to outliers.

For GBD 2019, we conducted systematic literature reviews for 18 risks. For other risk factors, only a small fraction of the existing data appears in the published literature, and other sources predominate, such as survey data and satellite data. Data were systematically screened from household surveys archived in the GHDx (http://ghdx.healthdata.org), including Demographic and Health Surveys, Multiple Indicator Cluster Surveys, Living Standards Measurement Surveys, and Reproductive Health Surveys. Other national health surveys were identified based on survey series that had yielded usable data for past rounds of GBD, sources suggested to us by in-country collaborators, and surveys identified in major multinational survey data catalogues, such as the International Household Survey Network and the WHO Central Data Catalog, as well as through country Ministry of Health and Central Statistical Office websites. Citations for all data sources used for risk factor estimation in GBD 2019 are provided in searchable form through a web-tool (http://ghdx.healthdata.org).

Information on systematic reviews were managed by using Research Electronic Data Capture (REDCap) electronic data capture tools hosted at the University of Washington.[16] REDCap is a secure, web-based application designed to support data capture for research studies that provides 1) an intuitive interface for validated data entry; 2) audit trails for tracking data manipulation and export procedures; 3) automated export procedures for seamless data downloads to common statistical packages; and 4) procedures for importing data from external sources.

3.6.2 Search terms and data preparation

Search terms for updates of systematic reviews for GBD 2019 are shown by risk factor in appendix section 4 Supplementary appendix 1 of Supplement to: “GBD 2019 Risk Factors Collaborators. Global burden of 87 risk factors in 204 countries and territories, 1990-2019: a systematic analysis for the Global Burden of Disease Study 2019”.[2]

Survey data constitutes a substantial part of the underlying data used in the estimation process. During extraction, we concentrated on demographic variables (such as location, gender, age), survey design variables (such as sampling strategy and sampling weights), and the variables used to define the population estimate (such a prevalence or a proportion) and a measure of uncertainty (standard error, confidence interval or sample size and number of cases).

Several adjustments were applied to extracted exposure sources to make the data more consistent and suitable for modelling. In GBD 2019, we implemented adjustments of risk exposure data to deal with alternative case definitions or study methods prior to entering data into our main analytical tools of DisMod-MR 2.1 and ST-GPR. This decision also included the adjustment of data presented for both sexes to a male and female equivalent. The starting point was to explicitly state the reference case definition and study method and identify alternative definitions and study characteristics that fall within our inclusion criteria. We compiled data from both within-study comparisons (ie, data that used alternative and reference definitions in the same population) and between-study comparisons (ie, data that used an alternative definition in one population and a reference definition in another population that overlap in location, time, age, and sex) of different case definitions. For between-study comparisons, we allowed a maximum calendar year difference between studies of five years. Where validation studies (ie, those carried out at the introduction of a new set of diagnostic criteria comparing to previous criteria) were available, we extracted data on the comparison of alternative to reference. For quantities of interest with multiple alternative definitions/methods we also look for pairs comparing two alternatives. In a network analysis, if A is the reference and B and C are two alternatives, a comparison of A vs B and B vs C provides an indirect comparison of the alternative C against the reference A.

We pooled either the logit difference between alternative and reference or the natural log of the ratio of alternative to reference. From simulations we found that the two methods provide almost identical results for quantities that after adjustment do not exceed a value of 0·5 (eg, prevalence or proportion). The logit difference method much better dealt with higher values and avoided prevalence or proportions to exceed one. If the values of either the reference or

alternative were zero, we aggregated values across age groups until both values had non-zero observations. We used the delta method to compute the standard error of the reference and alternative measures in logit space. The standard error of the logit difference was computed as the square root of the sum of the variances of each data point in a pair.

3.6.3. Data analysis

We used a network random effects meta-regression in MR-BRT. In a network analysis, if A is the reference and B and C are two alternatives, a comparison of A vs B and B vs C provides an indirect comparison of the alternative C against the reference A. To implement the network, we included dummy variables with a particular structure. This was implemented as follows, where A is the reference definition/method:

- Create *k* dummy variables where *k* are all definitions/methods other than A (eg, *k* = B, C)
- Code dummy *k* as
  - 1 if the first term of the logit difference is *k*;
  - -1 if k is second term of the logit difference;
  - 0 otherwise

For example:

Study	Comparison	DummyB	DummyC
1	logit(B)-logit(A)	1	0
2	logit(B)-logit(A)	1	0
3	logit(C)-logit(A)	0	1
4	logit(C)-logit(A)	0	1
5	logit(C)-logit(B)	-1	1
6	logit(C)-logit(B)	-1	1

The coding structure outlined above in step 1 assumes that all case definitions are mutually exclusive. In some cases, however, individual case definitions are a function of different components or dimensions. For example, case definitions may vary by the type of symptoms that a respondent experiences as well as the recall period over which those symptoms are experienced. In the presence of sparse data, it may be difficult to find both direct and indirect comparisons of all individual case definitions. In this case, an alternative approach is to assume different dimensions of case definitions have a multiplicative effect. In other words, the effect of recall period has the same relative effect across different categories of symptoms reported by respondents. To implement this coding scheme:

- Create *k* dummy variable columns for each case definition dimension
- For each dummy variable k:
  - Add 1 if *k* is a component of the first term in the logit difference
  - Subtract 1 if *k* is a component of the second term in the logit difference

In MR-BRT, we ran random effects meta-regression of the logit difference (or log ratio) with all the *k* dummy variables as covariates, omitting the intercept in the meta-regression. We used a study_id variable for be the unique combination of the NIDs of the reference and alternative studies (or alternative1 to alternative2). The coefficients on the *k* dummy variables represent the pooled logit difference of the *k* alternative definition to the reference taking into account evidence from both direct and indirect comparisons. In the example above, the coefficient on DummyA is the pooled logit difference of B minus A; the coefficient on DummyB is the pooled logit difference of C minus A. The standard error of the pooled logit difference incorporating the between study variance was calculated as:

$$se(\text{logit}(difference_k)) = \sqrt{var_k + \gamma^2}$$

Where: $se(\text{logit}(difference_k)$= standard error of the pooled logit difference of alternative k to the reference

var_k=variance of the coefficient on dummy variable *k*

γ^2 = between-study variance

If both between and within study pairs were available, we examine whether there was a systematic difference between these. If there was a significant difference, we made judgement call as to whether within-study or between study data comparisons were most appropriate. In general, this was the within-study data, however, there were important measurement or conceptual reasons for choosing between-study data. For example, for crosswalks between self-reported height and weight compared to measured height and weight, between-study comparisons may be preferable if respondents knew they would be measured and, therefore, were less likely to misreport their height and weight.

We also examined whether there were systematic differences in the adjustments by key demographics (age, sex, geographic location, year) and other potential factors that may lead to variation in crosswalks. This could only be done at present in a direct comparison model and not in a network. We did this when there was a strong rationale, eg, biological plausibility, for variation by such characteristics. After obtaining the pooled logit difference or log ratio estimates, we predicted adjustments based on the statistical model, including uncertainty in the adjustment and sampling error of each data point. For non-significant logit differences or log ratios we still applied the adjustments if there was a conceptual reason to believe that the alternative definition is biased. This expands the variance of these alternative definition data points.

3.6.4. Estimates exposure

We used systematic literature reviews to identify risk factor exposure studies published or identified since GBD 2017 and combined these with existing data from household and health examination surveys and census, morbidity, or satellite imagery and ground sensor data (used for estimation of particulate matter <2·5 μm in diameter [$PM_{2·5}$]). Certain risks, such as poor diet and excessive alcohol consumption, also incorporated administrative record systems. Data sources used in estimating risk factor exposure can be accessed through the data source tool at http://ghdx.healthdata.org/.

Once data were collected and compiled, the analytical flowchart describes the adjustments applied, where necessary, to correct for bias. Examples of these adjustments include use of urban studies for lead; crosswalks between different measurements, methods, and definitions, such as for self-report of obesity and glycated haemoglobin (HbA_{1C}) for diabetes; and age-sex splitting of data, such as for fasting plasma glucose (FPG) level, cholesterol level, and systolic blood pressure that may be reported from broad age-groups.

For the GBD, we developed two modelling approaches, a Bayesian meta-regression model (DisMod-MR 2.1) and a spatiotemporal Gaussian process regression model (ST-GPR), to pool data from different sources, control and adjust for bias in data, and incorporate other types of information such as country- level covariates. DisMod-MR 2.1 and ST-GPR are mixed effect models that borrow information across age, time, and locations to synthesise multiple data sources into unified estimates of levels and trends. A detailed description of the likelihood used for estimation and a full description of improvements made for DisMod-MR 2.1 were detailed by Vos and colleagues,[1] The ST-GPR model has three main hyper-parameters that control for smoothing across time, age, and location. Values for these hyper-parameters were selected on the basis of cross-validation. Cross-validation tests were conducted for different combinations of the hyper-parameters for three types of models: one data-sparse model, one data-moderate model, and one data-dense model. In each test, 20% of the data were held out, and the performance of each combination of hyper-parameters was evaluated on the held-out data. For each hyper-parameter combination, 10 cross-validation tests were conducted. The performance of each model in predicting the withheld 20% of the data was evaluated by using a combined measure based on root mean square error (RMSE) and uncertainty interval (UI) coverage. A detailed description of the ST-GPR process regression can be found below.

The main difference between these methods is their power to include unstructured types of data by sex and age group and their degree of flexibility. DisMod-MR 2.1 is the preferred tool in these cases because of its ability to integrate over age and adjust for different exposure definitions in the data; however, the use of Bayesian Markov Chain Monte Carlo (MCMC) simulations with large volumes of data renders the analysis computationally intensive and reduces the number of iterations that are possible. If standard age-group data are available – as is generally the

case for metabolic risks – using ST-GPR becomes the preferred approach.

In some cases, we adapted our methods of modelling exposure to risks where necessary to account for complexities in the risk-outcome relationship or the need for particular handling of data, for example, dietary risks and ambient air pollution (see appendix section 4 to: "GBD 2019 Risk Factors Collaborators. Global burden of 87 risk factors in 204 countries and territories, 1990-2019: a systematic analysis for the Global Burden of Disease Study 2019"[2] for more detail). A complete list of risks is reported in table S5, and additional details for adjustments or adaptations to particular risk models are provided in appendix section 4 ("GBD 2019 Risk Factors Collaborators. Global burden of 87 risk factors in 204 countries and territories, 1990-2019: a systematic analysis for the Global Burden of Disease Study 2019").[2]

Theoretical minimum-risk exposure level (TMREL)

In this and all previous GBD studies, the counterfactual level of risk exposure used is the risk exposure that is both theoretically possible and minimizes risk in the exposed population that consequently captures the maximum population-attributable burden.[17] For each risk evaluated in GBD 2019, Step 4 of the analytical process describes the use of the best available epidemiological evidence from published and unpublished RRs by level of exposure and the lowest observed level of exposure from cohorts, used to select a single level of risk exposure that minimises risk from all causes of deaths combined to establish the TMREL. In principle, the TMREL for a given risk may vary by age, sex, and location if supported by clear evidence. Based on the available evidence, the TMREL itself can be uncertain, which is reflected in the 95% UIs. In GBD 2019, we updated the process of estimating TMREL for dietary risks. We set the TMREL to zero for all harmful dietary risk factors with monotonically increasing risk functions (eg, processed meat intake); this excludes sodium. For protective risks with monotonically declining risk functions with exposure (eg, fruit intake), we first determined the 85th percentile of exposure in the cohorts or trials used in the meta-regression of each outcome that was associated with the risk. Then, we determined the TMREL by weighting each risk-outcome pair by the relative global magnitude of each outcome.

Estimate population-attributable fractions

Risks are categorised on the basis of how exposure was measured: dichotomous, polytomous, and continuous. High low-density lipoprotein (LDL) cholesterol level is an example of a risk measured on a continuous scale. The PAF, which represents the proportion of risk that would be reduced in a given year if the exposure to a risk factor in the past were reduced to an ideal exposure scenario, is defined for a continuous risk factor as:[18]

$$PAF_{joasgt} = \frac{\int_{x=l}^{u} RR_{joasg}(x)P_{jasgt}(x)dx - RR_{joasg}(TMREL_{jas})}{\int_{x=l}^{u} RR_{joasg}(x)P_{jasgt}(x)dx}$$

Where PAF_{joasgt} is the PAF for cause o due to risk factor j for age group a, sex s, location g, and year t.

$RR_{joasg}(x)$ is the RR as a function of exposure level x for risk factor j for cause o, age group a, sex s, and location g with the lowest level of observed exposure as l and the highest as u; $P_{jasgt}(x)$ is the distribution of exposure at x for age group a, sex s, location g, and year t; and $TMREL_{jas}$ is the TMREL for risk factor j, age group a, and sex s.

The PAF_{joasgt} for dichotomous and polytomous risk factors for every country is defined as:

$$PAF_{joasgt} = \frac{\sum_{x=1}^{u} RR_{joast}(x)P_{jasgt}(x) - RR_{joasg}(TMRE_{jas})}{\sum_{x=1}^{u} RR_{joas}(x)P_{jasgt}(x)}$$

where PAF_{joasgt} is the PAF for cause o due to risk factor j for age group a, sex s, location g, and year t.

$RR_{joasg}(x)$ is the RR as a function of exposure level x for risk factor j for cause o, age group a, sex s, and location g on a plausible range of exposure levels from l to u; $P_{jasgt}(x)$ is the proportion of the population in risk group (prevalence) for age group a, sex s, location g, and year t; and $TMREL_{jas}$ is the TMREL for risk factor j, age group a, and sex s.

Estimate summary exposure values

We first calculate risk, r, and cause, c, for specific SEVs by using the following equation, for each most-detailed age, sex, location, year, and outcome. PAF is the YLL (expect for occupational noise, bullying victimization, and occupational ergonomic factors, which are YLD only and thus use the YLD) PAF. RR_{max} for categorical risks is the RR at the highest category of exposure. For continuous risks, this is otherwise, and for custom modelled risks like ambient particulate matter pollution, HAP from solid fuels, alcohol, smoking, bullying, and activity, the modeller provides draws of RR_{max}. Generally, RRs do not vary across time and space. However, exceptions exist, such as risks from second-hand smoke (SHS) or HAP for which the RR is based on the integrated exposure response (IER) curve. In these cases, the RR is averaged across location and year to ensure no time or space variation. If the PAF is negative, which signifies a protective effect for that outcome, the PAF is set to 0 and the SEV is then also 0 because the SEV is univariate and constrained to be a value between 0 and 1. Once we obtained a set of risk-cause specific SEVs at the most-detailed risk, cause, age, sex, and location for all years, we averaged across causes to produce the final risk specific SEV_r, where $N(c)$ is the total number of outcomes for a risk.

$$SEV_{rc} = \frac{\frac{PAF_{rc}}{1 - PAF_{rc}}}{RR_{max} - 1}$$

$$RR_{max} = RR^{\frac{TMREL - 1^{st} exposure}{RR_{scalar}}} \text{ if protective, or}$$

$$= RR^{\frac{99^{th} exposure - TMREL}{RR_{scalar}}}$$

$$SEV_r = \frac{1}{N(c)} \sum_c SEV_{rc}$$

Mediation

The portion of the burden of disease that is attributable to various combinations of risk factors or to all risk factors combined has been a topic of broad interest.[17] In GBD 2010, we only aggregated the burden of risk factors for some clusters of risks, including access to improved water and sanitation, child and maternal malnutrition, tobacco smoking, alcohol use, dietary risk factors, occupational risk factors, and sexual abuse and violence. We did not aggregate air pollution and metabolic risk factors. For GBD 2013 onwards, we aggregated all risk factors into three large categories—behavioural, environmental, and occupational, and metabolic risks—and aggregated all GBD risk factors into a single attributable fraction for each disease and eventually for all causes of burden.

Aggregating risk factors at different levels shares three essential challenges:

1. Risk factor coexistence or aggregation: for example, metabolic risk factors often occur together, or high-risk behaviours such as drug abuse and unsafe sex are related.
2. Mediation: a risk factor may affect another risk factor that lies in the physiological pathway to a disease outcome. It can be inside a cluster of risk factors, such as the effect of obesity through an increase in FPG level and later cardiovascular disease (CVD) outcomes, or between clusters of risk factors, such as the effect of fibre on cholesterol.
3. The formula used to calculate the aggregated PAF.

The aggregation method is conceptually applicable to other aggregations such as socioeconomic factors, education, homelessness, and refugee status that are being considered for inclusion in future GBD iterations. In the next section, we explain our approach to dealing with these challenges.

There are three patterns of associations between risk factors to consider (figure below). The first concerns confounding; risk B affects risk A and outcome C (Pattern 1 in *Patterns of associations between risk factors*). In these cases, the RR for A should be adjusted for B; for example, the fruit RR is adjusted for smoking. If part of the effect of A is through B, a mediator, we do not adjust the effect of A for B. For example, we do not adjust the RR of body-mass index (BMI) for cholesterol because cholesterol lies in the biological pathway between BMI and cardiovascular outcomes (Pattern 2 in in *Patterns of associations between risk factors*). The third pattern occurs when risks A and B are proxies of a third variable Z and aggregation aims to estimate the total effect of a latent variable Z on C. An example is child growth failure, which is measured by stunting, wasting, and underweight as

proxies.

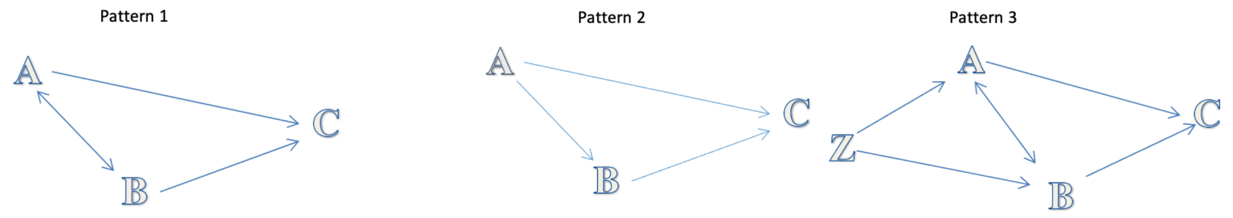

Calculating the burden of multiple risk factors

Validation studies have reported congruency between the true risk associated with multiple risk factors affecting the same outcome and a multiplicative aggregation of the PAFs of the individual risk factors (formula below),[18] where *PAF* is the population attributable fraction and *i* is each individual risk factor. The same validation studies also found that the overestimation from ignoring the covariance between risk factors is small. This small overestimation was important to note because few data sources exist from which we can draw information on covariance. We endeavoured to evaluate RRs that were controlled for confounders. However, because we had to rely on the literature for many RRs, we did not always have full control over the choice of confounders controlled for in each study.

$$PAF_{1..i} = 1 - \prod_{i=1}^{n}(1 - PAF_i)$$

Adjusting for mediation

When aggregating the effects of multiple risk factors, we included an MF if a part of the effect of one risk factor was included in the effect estimated for in the mediator. First, we prepared a list of possible mediations, and especially between behavioural risks and metabolic risk factors with cardiometabolic outcomes. We did not assume any mediation effect between risk factors for cancers. Danaei and colleagues assumed that part of the effect of BMI on ischaemic heart disease (IHD) is through high systolic blood pressure (SBP), cholesterol level, and FPG.[19] The proportion of the BMI effect that can be explained by other metabolic risk factors is the amount of mediation. The difference between the crude RR of BMI on IHD with the RR adjusted for SBP, FPG, and cholesterol level reflects the amount of BMI effect on IHD that is mediated and already included in SBP, FPG, and cholesterol level:

$$MF = \frac{RR_{crude} - RR_{adjusted}}{RR_{crude} - 1}$$

So, to aggregate the PAF of multiple risk factors, we first calculated the part of the excess risk (RR-1) of every risk factor that is not mediated, re-compute the PAF so that it only includes the non-mediated risk then aggregated PAFs by assuming they are independent. Therefore, if MF is the mediation factor of R2 through R1, the adjusted RR for R2 including only the non- mediated component of risk is The PAF accounting for mediation is then computed using the adjusted RR and the joint PAF computed. For every paired risk factor and outcome, the matrix of possible mediations was calculated and used.

$$RR_{1.2} = MF_{2/1}(RR_2 - 1) + 1$$

Calculating mediation factor

The best example is the mediation of BMI through SBP, FPG, and cholesterol level reported by Danaei et al. [19] In their meta-analysis, they report the adjusted and unadjusted RR of BMI on IHD and stroke based on combined data from individual cohorts. They calculated the MF by using the following equation, and we used it directly as the MF in risk factor aggregation. Using individual-level data from cohort studies, we estimated the MF for other metabolic risk factors and some dietary risks. For many other risk factors, no data are available to enable the use of the first method. Instead, we searched studies to estimate the effect of the risk factor on the mediator and, finally, the expected increase in IHD risk. We pooled available studies to calculate the unit increase in the mediator per unit increase in the risk factor to calculate the size of the IHD RR.

$$MF = \frac{RR_{crude} - RR_{adjusted}}{RR_{crude} - 1}$$

Uncertainty of aggregated and mediated PAFs
We generated 1000 draws of the posterior distribution of the MF calculated by different methods to use beside draws of other inputs to the PAF aggregation.

Important assumptions in aggregating risk factors and including mediation
1 – The MFs or PAF adjustments are similar across countries, age, sex, and years. Although the size of mediation is probably different in different populations, little data is available to inform the covariance between different risk factors or the MF amount by age and country. For example, in some countries, the size of the mediated BMI-IHD PAF exerted through cholesterol level, as calculated by the MF, was even bigger than the total burden of cholesterol level. This finding indicated that less of the effect of BMI is mediated through cholesterol level and MFs are not similar across countries.
2 – For many risk-mediator-outcome pairs, no data are available, so we assumed the mediation is zero.
3 – Because the covariance between undernutrition indicators differs by location (and across time, but results were not reported), and an interaction exists between these indicators, the total burden might be underestimated.
4 – We assumed no significant covariance between PAFs, which might not be true between some risk factors, such as metabolic risk factors. Although this overestimation can be controlled by using adjusted RRs, using crude RRs for BMI and other metabolic risk factors may cause significant overestimation of the aggregated metabolic risks burden.

Estimate attributable burden
Four key components are included in the estimation of the burden attributable to a given risk factor: the metric of burden being assessed (the number of deaths, YLLs, YLDs, or DALYs [the sum of YLLs and YLDs]); the exposure levels for a risk factor; the RR of a given outcome due to exposure; and the counterfactual level of risk factor exposure. Estimates of attributable burden as DALYs for risk-outcome pairs were generated by using the following model:

$$AB_{jasgt} = \sum_{o=1}^{w} DALY_{joasgt} PAF_{joasgt}$$

where AB_{jasgt} is the attributable burden for risk factor j for age group a, sex s, location g, and year t;

$DALY_{joasgt}$ is total DALYs for cause o (of w relevant outcomes for risk factor j) for age group a, sex s, location g, and year t; and PAF_{joasgt} is the PAF for cause o due to risk factor j for age group a, sex s, location g, and year t. The proportions of deaths, YLLs, or YLDs attributable to a given risk factor or risk factor cluster were analogously computed by sequentially substituting each metric in place of DALYs in the equation provided.

Decomposition analysis of deaths and DALYs
We conducted a decomposition analysis of changes in DALYs from 2010 to 2019, decomposing changes in all-age cause-specific DALYs attributable to all risk factors and individual risk factors due to changes in population growth, population age structure, exposure to the given risk for a disease, and risk-deleted death and DALY rates. In this case, risk-deleted rates are the rates obtained after removing the effect of a risk factor or combination of risk factors — in other words, observed DALY rates multiplied by one minus the PAF for the risk or set of risks. Our decomposition analyses draw from methods developed by Das Gupta[20] to provide a computationally tractable solution for isolating drivers of burden changes whereby all combinations of possible pathways are averaged across factors. Attributable burden was determined, following the methods of Das Gupta,[20] as a product of three factors such that: where T_{asgt} represents the attributable burden at year t; A_{sgt} is the age-specific population size for a given age group a, sex s, and location g at year t; B_{asgt} is the underlying rate of the outcome unrelated to the risk factor or observed rate, multiplied by $1 - PAF$ for a given age group a, sex s, and location g at year t; and C_{asgt} is the ratio of the attributable burden to the underlying rate, which reflects the risk exposure effect for a given age group a, sex s, and location g at

$$T_{asgt} = (A_{asgt} B_{asgt} C_{asgt})$$

$$E_A = (A_{19} - A_{10}) \left(\frac{B_{10}C_{10} + B_{19}C_{19}}{3} + \frac{B_{10}C_{19} + B_{19}C_{10}}{6} \right)$$

year t defined as $PAF/(1 - PAF)$ when decomposing attributable burden to a risk. Risk exposure effects for individual risk factors are scaled such that they sum to the all-risk exposure effect by location, age, sex, and cause accounting for mediation. This process allows for aggregation of risks; the exposure for all risks for a disease can be split into exposure to metabolic, behavioural, and environmental risks. The contribution of each factor to total change in attributable burden was determined by changing the level of one factor from time t_0 to t_1 – here 2010 to 2019 – with all other factors held constant. Thus, the effect of any of the three factors, for example A_{asgt} on the change of the attributable burden between 2010 (A_{10}) and 2019 (A_{17}) is calculated as: where E_A is the proportion of change due to factor A, and the subscripts for each factor in the equation denote the year for each estimate. Because the effect depends on the order of entry of the factor, we calculated the average of all combinations of the three factors. The proportion of change due to factorA_{sgt}, the age-specific population size for a given age group a, sex s, and location g at year t, is then further split, setting change in population growth equal to the percentage change in the all-age population from time t_0 to t_1 and change in population age structure to the residual, giving four factors.

4. Detailed methodology for each condition

This section is a verbatim from GBD 2019 Capstones with detailed methodology for each condition.[1,2,3]

4.1. Alzheimer's disease and other dementias (fatal modelling)

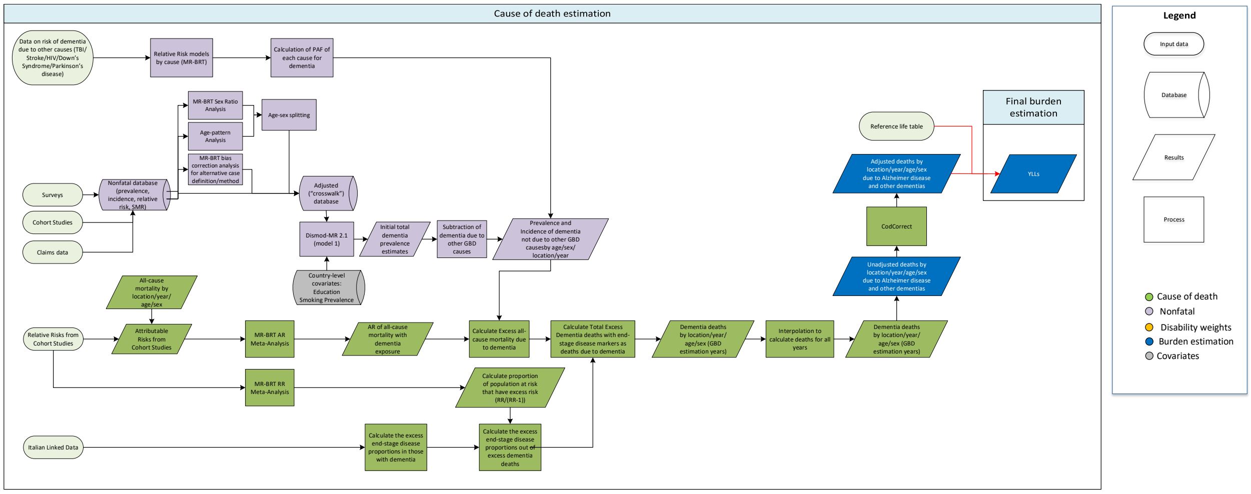

Input data

In GBD 2019, fatal modelling was redesigned to remove reliance on vital registration data (described in more detail in the "Modelling strategy" section). Instead, two new source types were extracted:

(1) Literature on the relative risk of all-cause mortality given the exposure of dementia. Relative risk sources were identified through a systematic review using search terms[2] in PubMed. This yielded 4470 total hits, of which 34 studies were marked for extraction. Overall, the data were heterogeneous and varied in the exposure category measured (all dementia, Alzheimer's disease, cognitive impairment) and in the different factors controlled for in analyses.

(2) Linked vital registration and hospitalisation data. We used mortality records linked to inpatient records, covering all deaths from 2003 to 2017 in the Emilia-Romagna region of Italy.

Table: Results of systematic review on all-cause excess mortality with dementia

N		60
Region name (%)	East Asia	4 (6.7)
	Eastern sub-Saharan Africa	2 (3.3)
	High-income Asia Pacific	4 (6.7)
	High-income North America	22 (36.7)
	North Africa and Middle East	1 (1.7)
	Tropical Latin America	1 (1.7)
	Western Europe	26 (43.3)
Exposure (%)	Alzheimer's disease	11 (18.3)
	cognitive impairment	10 (16.7)
	other dementia	35 (58.3)
	vascular dementia	4 (6.7)
Conducted in clinical setting (%)	Clinical setting	10 (16.7)
	Population representative	50 (83.3)
Controlled for education (%)	Controlled	32 (53.3)
	No control	28 (46.7)

Table: Results of systematic review on all-cause excess mortality with dementia

Controlled for basic CVD info (%)	Controlled	33 (55.0)
	No control	27 (45.0)
Extensive CVD control (%)	Controlled	15 (25.0)
	No control	45 (75.0)
Controlled for smoking and alcohol (%)	Controlled	11 (18.3)
	No control	49 (81.7)
Controlled for factors in causal pathway (%)	Controlled	13 (21.7)
	No control	47 (78.3)

Modelling strategy

Overview

Dementia mortality rates have increased more than five-fold since 1980 in high-quality vital registration systems such as in the USA and Scandinavia. We have not seen an equivalent increase in prevalence and incidence data sources. If at all, there has been a modest decline in incidence and prevalence of dementia in studies in the UK and the USA.[21,22] Also, the greater than 20-fold variation in mortality rates of dementia between countries is much greater than the four-fold difference in prevalence and incidence between countries. As it is unlikely that case fatality from dementia has dramatically increased over the time period and that it would differ by a very large margin between countries, the hypothesis is that certifying and coding practices have changed over time and at a different pace between countries. To avoid spurious large trends over time in the fatal component of the burden of dementia, we decided for GBD 2013 to make dementia mortality rates consistent with the most recent rates relative to prevalence of countries that are most likely to certify or code dementia as an underlying cause of death. This approach was applied again for GBD 2017 with some modifications. For GBD 2019, the fatal modelling process was redesigned to avoid the need for using estimates only from the highest dementia mortality locations. This was accomplished with an attributable risk model based on a systematic review of cohort studies and relative risk data, and end-stage disease proportions from linked hospital and death records. The modelling process is described below.

Modelling steps

Relative risk data

First, using relative risk data extracted from studies identified by systematic review, we calculated attributable risk and the GBD estimate of all-cause mortality rate for a given study location and time, using the following formula:

$$Attributable\ Risk = (Relative\ Risk - 1) * All\text{-}Cause\ Mortality$$

We then conducted a meta-analysis on the attributable risk data, using covariates for age, sex, exposure category (all dementia, Alzheimer's disease, cognitive impairment), whether the study was conducted in a clinical sample, and categories indicating different types of variables that were controlled for in the component studies (educational attainment, cardiovascular disease comorbidities, smoking and alcohol consumption, and daily activities or residence in a nursing home). Relative risks were estimated using a second Bayesian bias-reduction meta-regression model and the same studies identified through systematic review. Regression results for relative risk and attributable risk analyses are displayed below.

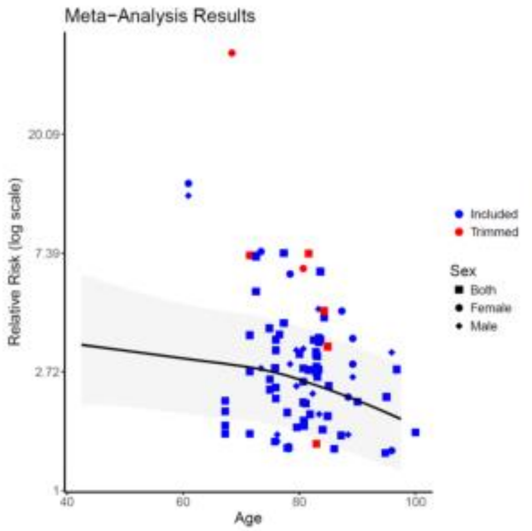

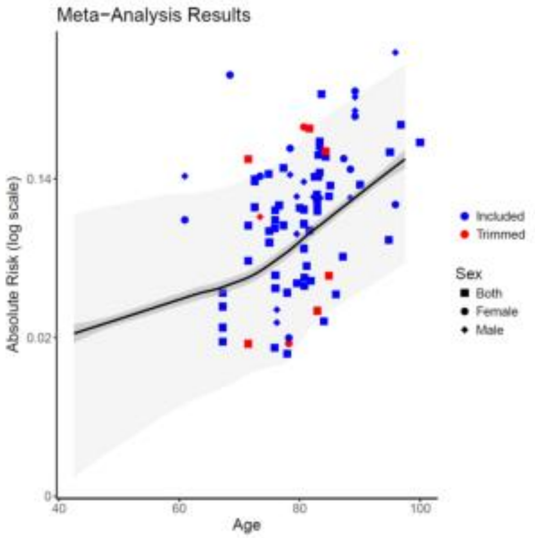

Meta-regression results were used to calculate the total number of excess deaths due to dementia as the product of our prevalence estimates (post-adjustment for dementia caused by other GBD diseases) and our estimates of attributable risk. See the non-fatal write-up on dementia for details on prevalence calculations.

Linked data
The excess deaths calculated through the multiplication of attributable risk and prevalence represent the total number of excess deaths due to having dementia, which likely includes deaths due to other conditions, such as cardiovascular diseases, that are more common in those with dementia as compared to the general population due to common underlying risk factors such as blood pressure, smoking, and lower educational attainment. In order to subset this total number of excess dementia deaths to calculate the number of deaths that were caused by dementia, we completed an analysis of linked clinical and mortality data. We used mortality records linked to inpatient records, covering all deaths from 2003 to 2017 in the Emilia-Romagna region of Italy. Using these data, we looked for markers of severe, end-stage disease in the clinical records up to one year before death.

To select these markers, for each ICD code that appeared in the data we calculated the difference in the proportion of individuals who died with dementia and had a record of each code in the year before death and the proportion of individuals who died without dementia and had a record of the same code in the year before death. We reviewed the 150 codes with the highest difference and selected codes that indicated end-stage disease, excluding codes for conditions such as cardiovascular disease. Codes for decubitus ulcer, malnutrition, sepsis, pneumonia, urinary tract infections, falling from bed, senility, dehydration, sodium imbalance, muscular wasting, bronchitis, dysphagia, hip fracture, and bedridden status were used as indicators of severe disease.

In order to determine the proportion of excess deaths that were caused by dementia, we calculated the proportion of dementia deaths that had clinical markers of end-stage disease in the year before death, above and beyond the occurrence of end-stage disease markers in those who died without dementia. The subtraction of the proportions with end-stage disease markers in those without dementia from the proportions in those with dementia represents the proportion of individuals who are assumed to have died with severe, end-stage dementia out of total deaths in those with dementia.

Figure 2: Percent of individuals who died with and without dementia with end stage conditions in the last year before death

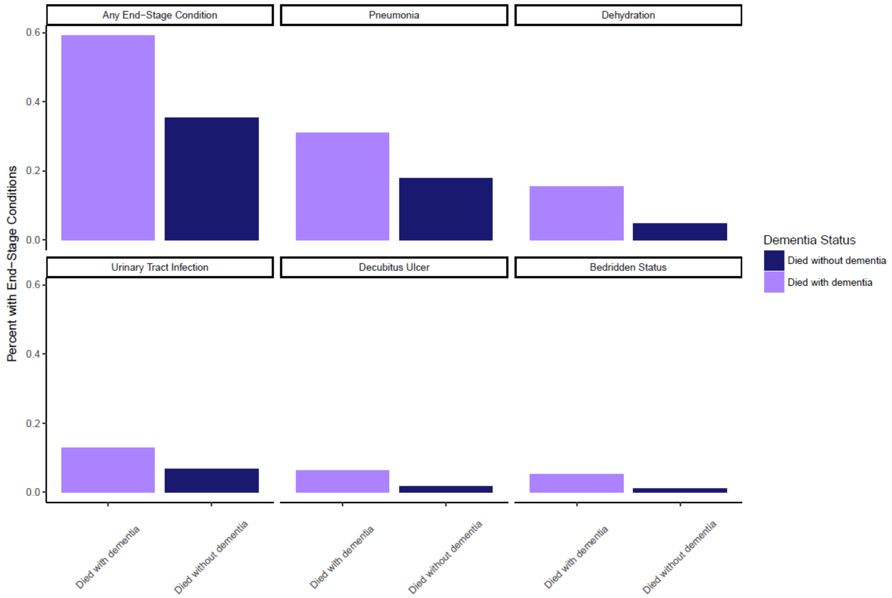

Calculation of deaths due to dementia

In order to apply these estimates to the total excess deaths we then adjusted these proportions to calculate the proportion of individuals who died with severe, end-stage dementia out of excess dementia deaths using the formula:

$$\frac{\textit{Died with Severe Disease}}{\textit{Excess Dementia Deaths}} = \frac{\textit{Died with Severe Disease}}{\textit{Total Dementia Deaths}} * \frac{\textit{Relative Risk}}{\textit{Relative Risk} - 1}$$

We then calculated the number of deaths due to dementia as the product of total excess dementia deaths and the proportion of those who died with severe disease out of excess dementia deaths. These final estimates of deaths due to dementia were then used to adjust data on causes of death from all other causes in vital registration systems.

Interpolation for all years

Finally, we used log-linear interpolation to interpolate these results (limited to 1990, 1995, 2000, 2005, 2010, 2015, 2017, 2019) to create estimates for the entire time series from 1980 to 2019. Socio-demographic Index was used as a covariate to extrapolate back to the year 1980.

4.2. Alzheimer's disease and other dementias (non-fatal modelling)

Flowchart

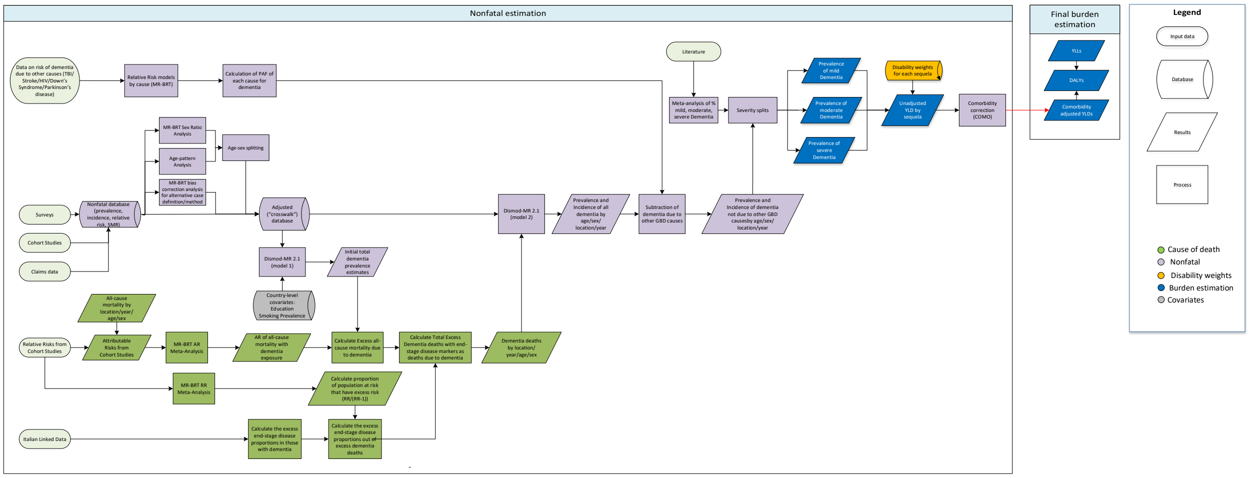

Input data and methodological summary

Case definition

Dementia is a progressive, degenerative, and chronic neurological disorder typified by memory impairment and other neurological dysfunctions. For the purposes of GBD 2019, we use the Diagnostic and Statistical Manual of Mental Disorders III, IV or V, or ICD case definitions as the reference. The DSM-IV definition is:

- Multiple cognitive deficits manifested by both memory impairment and one of the following: aphasia, apraxia, agnosia, disturbance in executive functioning
- Must cause significant impairment in occupational functioning and represent a significant decline.
- Course is characterized by gradual onset and continuing cognitive decline
- Cognitive deficits are not due to other psychiatric conditions
- Deficits do not occur exclusively during the course of a delirium

A wide array of diagnostic and screening instruments exists, including Clinical Dementia Rating scale (CDR), Mini Mental State Examination (MMSE), and the Geriatric Mental State (GMS). For severity rating purposes we use the CDR as the reference. The relevant ICD-10 codes for dementia are F00, F01, F02, F03, G30, and G31. The ICD-9 codes are 290, 291.2, 291.8, 294 and 331.

Unlike most causes in the Global Burden of Disease project, dementia mortality and morbidity estimates are modelled jointly. This is because of marked discrepancies between prevalence data and cause of death data. Specifically, prevalence data suggest little to no variation over time (eg, 1990–2019), whereas age-standardised mortality rates in vital registrations in high-income countries have increased multiple times over this same period. Additionally, prevalence variation between countries is much smaller than the variation in death rates assigned to dementia in vital registration. We attribute these discrepancies to changing coding practices rather than epidemiological change.

Because of this joint procedure, descriptions of the mortality estimation process are included where relevant.

Model inputs

To inform our estimates of burden due to dementia, we use mortality data from relative risk studies and linked hospital to mortality data, as well as prevalence data from surveys and administrative data such as claims sources.

Item Response Theory for prevalence prediction
The prevalence models for dementia are data sparse, and there are not many surveys done in low-income settings. However, there are a larger body of surveys that collect data on cognitive tests and functional limitations which are the two main components of a DSM or ICD diagnosis. Predictions of dementia prevalence using information from these questions would allow for expanded data coverage and additional information in locations where there are currently no data guiding estimates.

Generating these predictions requires calibrating a model to samples that have information about both functional limitations, cognition, and adjudicated dementia diagnoses. However, making comparisons across surveys can be difficult, as each survey asks a different set of questions about cognition and limitations, although there is some overlap. This overlap allows for the use of item response theory methods for the harmonization of these scales. Once the scales are harmonized the subsamples can be utilized to create a model for the prediction of prevalence.

In GBD 2019, data from the ADAMS and HRS surveys were extracted and used for Item Response Theory modelling to estimate prevalence. HRS is a nationally representative survey in the US, which has data on cognition and functional limitations. ADAMS is a subsample of HRS that includes much more detailed neuropsychological testing and adjudicated dementia diagnoses. ADAMS includes almost all questions in HRS plus additional questions as well.

Excluding incidence
Since 2016, we have made the decision to exclude incidence data, because in locations with high quality cohort data on prevalence and incidence, the two are not compatible (incidence data implies a higher prevalence than what is reported). Because dementia has a slow, insidious onset and prevalence is easier to measure, we trust prevalence data more and rely on this, excluding incidence data from DisMod.

Severity splits
Methods to determine severity splits for dementia were redesigned in GBD 2019. A new systematic review was conducted to collect information on the proportion of individuals in each dementia severity class out of the population of all individuals with dementia. There are a variety of commonly used methods for severity rating; for the purposes of GBD 2019, we took the Clinical Dementia Rating (CDR) scale as our reference definition for severity classification, along with a doctor-given diagnosis according to DSM III, IV, V or ICD case definitions as our reference definition for dementia.
However, as a neurodegenerative disorder with a wide range of categories in which symptoms manifest, there are an abundance of classification tools which discern between severity levels along different criteria. We accepted severities classified by:

- Clinical dementia rating sum-of-boxes (CSR-SB)
- Blessed test of information, memory, and concentration (BIMC)
- Global deterioration scale (GDS)
- Geriatric Mental State Examination (GMS)
- CAMDEX
- DSM-III-R
- Karasawa's

We excluded any studies which classified dementia severity according to scales that only evaluated cognitive function and memory, excluding activities of daily living (ADLs). The most prominent such scale is MMSE.

The following search string was used:
((dementia[MeSH Terms] OR dementia[Title] OR Alzheimer disease[Title]) AND (severity[Title/Abstract] OR CDR[Title/Abstract] OR Clinical Dementia Rating Scale[Title/Abstract]) AND (Severity of illness index[MeSH] OR

diagnosis[sh] OR Cross-Sectional Studies[MeSH])) AND ("1950/01/01"[Date - Publication] : "2100/02/25"[Date - Publication]) NOT (animals[MeSH] NOT humans[MeSH]))

Prisma diagram of dementia severity split systematic review

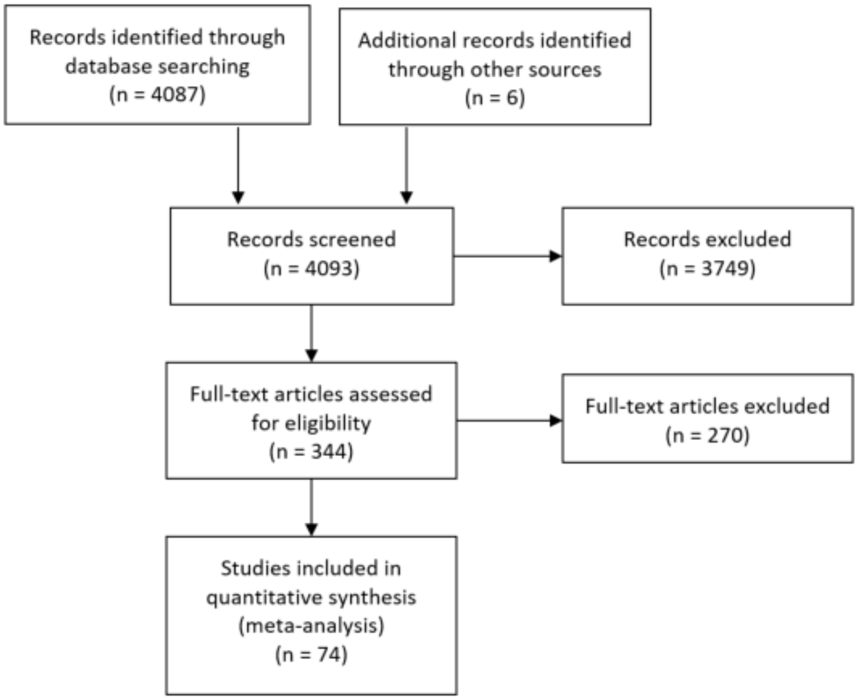

This yielded 4087 total hits, of which 338 passed initial title/abstract screening. After full-text screening, 68 sources met screening criteria and were extracted, along with one source identified through the bibliographies of other sources, and five additional sources used in GBD 2017 for other purposes. A total of 74 sources were extracted and informed the severity split, as compared to the 11 sources used in GBD 2017.

The severity split analysis was conducted using a MR-BRT meta-regression instead of being analysed as binned meta-analyses as in GBD 2017.

We multiplied estimations of prevalence (country-year-sex-age-specific) by the fractions of mild, moderate, and severe dementia and estimated 95% uncertainty intervals at the 1000-draw level. The severity distributions over age for each sex are visualized below, followed by a table describing each severity.

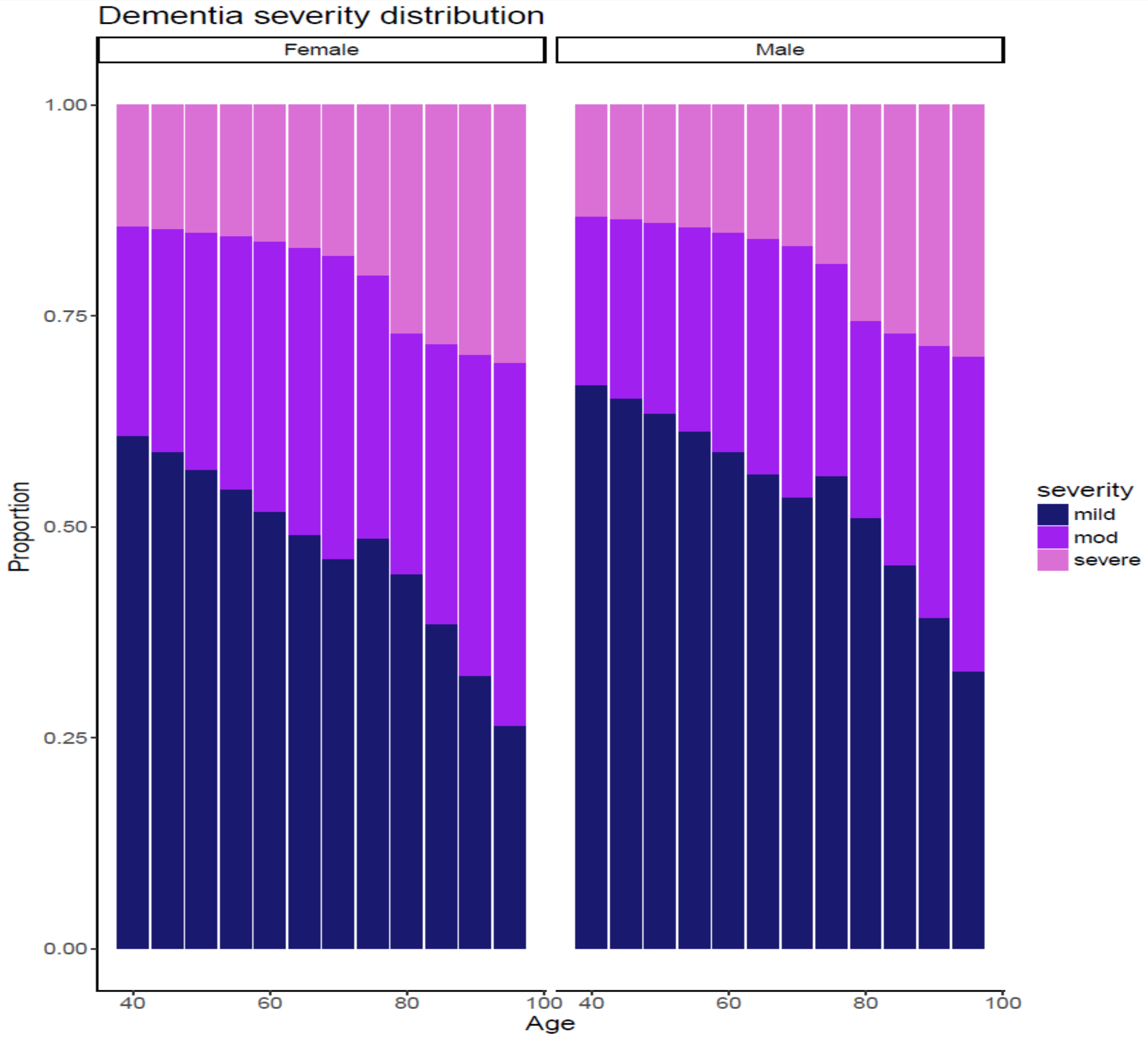

Figure. Severity ratios for each 5-year age bin, by sex.

Table of dementia severity levels.

Severity level	Lay description
Mild	The person has some trouble remembering recent events and finds it hard to concentrate and make decisions and plans. They may have slight to moderate difficulty engaging in community affairs, complicated hobbies, and intellectual interests.
Moderate	The person retains highly learned material, but has severe memory problems, is disoriented with respect to time and sometimes place. They are severely impaired in their ability to handle problems and make social judgements. They require assistance with daily activities, and only retain simple chores and hobbies.
Severe	The person has complete memory loss, no longer recognizes close family members, and requires help with all daily activities, including personal care.

Relative risk due to other causes

While the DSM definition excludes dementia cases, where the syndrome is caused by other psychiatric disorders, it does not exclude dementia cases caused by other diseases, not included in DSM. This includes, stroke, Parkinson’s

disease, Down's syndrome and traumatic brain injury (TBI), which are found elsewhere in the GBD cause list. To prevent double counting of prevalent cases, both under dementia and each of these other causes, we adjusted our dementia prevalence to exclude cases caused by these other conditions. To do so, in GBD 2019 we used data from the Aging, Demographics and Memory study (ADAMS), to estimate the relative risk of getting dementia for each condition included in the ADAMS dataset (stroke, Parkinson's disease, TBI). We then conducted more extensive systematic reviews on all five of these conditions to model each separately. Relative risk models were run using MR-BRT, and population attributable fractions (PAF) for each condition were calculated with the following equation, where exposure is defined as the prevalence of condition:

$$PAF = \frac{exposure * (RR - 1)}{[exposure * (RR - 1)] + 1}$$

Finally, attributable burden was calculated as the PAF multiplied by total burden (i.e. dementia incidence/prevalence).

Modelling strategy

First, prevalence data was sex split, crosswalked and age split. Studies with age and sex detail separately were split into age- and sex-specific data points. Data specified as "both" sex data were split into male- and female-specific data points using MR-BRT to get a model ratio of female/male prevalence and then using the following equations:

Male prevalence:

$$prev_{male} = prev_{both} * \frac{pop_{both}}{(pop_{male} + ratio * pop_{female})}$$

Female prevalence:

$$prev_{female} = ratio * prev_{male}$$

We also split data points where the age range was greater than 25 years using the global age pattern.

Dementia studies are heterogeneous. Even with a smaller number of definitions (DSM/ICD), there are a large number of different ways to diagnose dementia. Most use a two-step procedure, where you screen using a cognitive test and then only fully evaluate those that fall below a certain pre-defined threshold. We controlled for methods differences by crosswalking alternative case definitions to reference. Study covariates are based on broad categories determined after going through the diagnostic heterogeneity and there are some added for specific criteria that we know are biased. The same study-level covariates were used in 2019 as in 2017 with the addition of Item Response Theory HRS predictions.

MR-BRT Crosswalk Adjustment Factors for Dementia (Network Analysis)

Data input	Reference or alternative case definition	Gamma	Beta Coefficient, Logit (95% CI)	Adjustment factor*
DSM or ICD case definition	Ref	0·34	---	---
Clinical records diagnosis criteria	Alt		-0·05 (-0·72 – 0·61)	0·51
Algorithm diagnosis criteria (AGECAT)	Alt		0·08 (-0·59 – 0·74)	0·50
NIA-AA diagnosis criteria	Alt		0·51 (-0·16 – 1·17)	0·53
10/66 algorithm diagnosis criteria	Alt		0·97 (0·30 – 1·64)	0·50
GP records used for diagnosis	Alt		-1·21 (-1·88 – -0·54)	

Two country-level covariates were included in the initial DisMod model. Age-standardised education was used as a proxy for general brain health/use that may be protective of dementia – specifically Alzheimer's disease. Smoking prevalence (age-standardised, both sexes) was also used as a covariate to guide estimates, as the literature has shown a positive relationship between smoking and dementia.

Note that two DisMod models were run with prevalence inputs – the first uses adjusted prevalence data (DisMod Model 1 in flowchart), which accounts for dementia caused by other diseases. The second uses unadjusted dementia (DisMod Model 2 in flowchart) which accounts for all dementia regardless of cause (this is the dementia impairment envelope). The tables below summarize country-level covariates used in each of these DisMod model.

Covariates. Summary of covariates used in the Parkinson's Disease DisMod-MR meta-regression model (adjusted prevalence, Model 1)

Covariate	Type	Parameter	Exponentiated beta (95% Uncertainty Interval)
Smoking prevalence (age-standardized)	Prevalence	TBD – asking Emma	
Healthcare access and quality index	Excess mortality rate		

Covariates. Summary of covariates used in the Parkinson's Disease DisMod-MR meta-regression model (unadjusted prevalence, Model 2)

Covariate	Type	Parameter	Exponentiated beta (95% Uncertainty Interval)
Smoking prevalence (age-standardized)	Prevalence	0·005	1·00 (1·00-1·01)
Healthcare access and quality index	Excess mortality rate	-0·08	0·92 (0·92 – 0·92)

As mentioned previously, the estimation of morbidity due to dementia occurs in conjunction with the mortality estimation. Additional details on this process can be found in the COD capstone appendix.

We pull the cause-specific mortality results from final fatal estimates into a final DisMod model (Model 2), with the same settings as the models previous. To prevent double counting of prevalent cases, both under dementia and under other causes that can lead to dementia, we adjusted our dementia prevalence to exclude cases caused by these other conditions, which include stroke, Parkinson's disease, traumatic brain injury and Down's Syndrome. To do so, we used data from the Aging, Demographics and Memory study (ADAMS) and new systematic reviews, to estimate the relative risk of getting dementia for each condition included in the ADAMS dataset (stroke, Parkinson's disease, TBI). We first fit logistic regression models predicting the outcome of dementia given each exposure, with an additional covariate on age.

We then used these models to predict the probability of dementia given each exposure at various ages and divided the probability of having dementia by the probability of not having dementia at each age to calculate relative risks. After calculating age specific relative risks, we used these data and estimates of dementia prevalence from our DisMod-MR 2.1 model to calculate the population attributable fractions (PAFs) for each cause and age using the formula:

$$PAF = \frac{prevalence * (RR - 1)}{prevalence * (RR - 1) + 1}$$

Finally, we multiplied the PAF by the total prevalence to get the amount of dementia prevalence that can be attributed to each cause and subtracted this from the total prevalence to get the prevalence of dementia that is not due to other GBD causes.

4.3. Parkinson's Disease (fatal modelling)

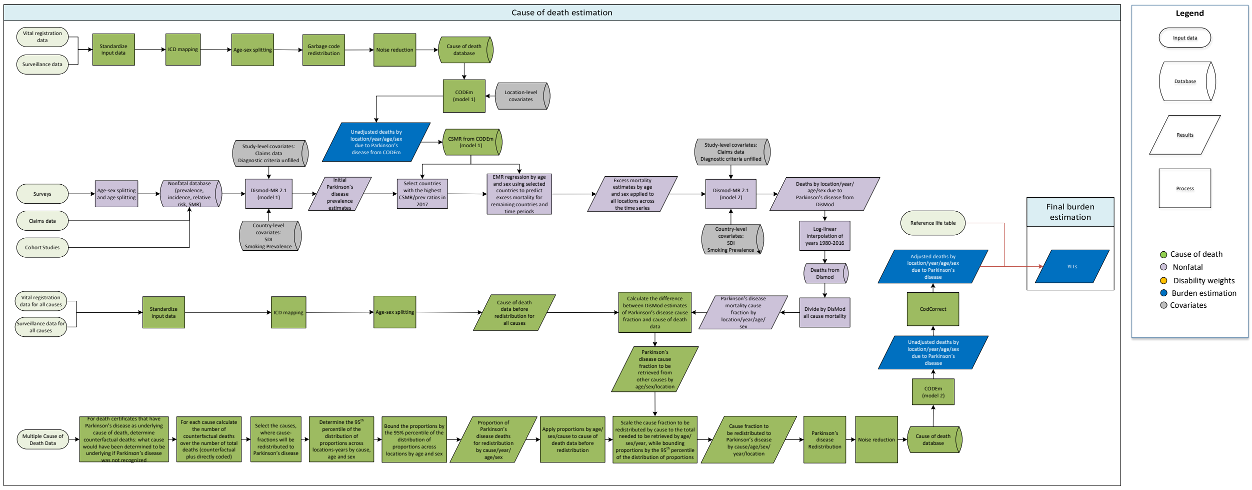

Input Data

In GBD 2017, data used to estimate deaths due to Parkinson's disease included mortality data from vital registration systems and prevalence data from surveys and claims sources.

An updated systematic review was conducted from September 2015 to August 2017, and search terms (below) were set to capture studies for Parkinson's disease.

Search terms: *(Parkinson disease[Title/Abstract] OR Parkinson's disease[Title/Abstract]) AND (epidemiology[Title/Abstract] OR prevalence[Title/Abstract] OR incidence[Title/Abstract]) AND ("2015/09/31"[PDAT] : "2017/08/23"[PDAT])*

Inclusion criteria comprised studies that reported prevalence, incidence, remission rate, excess mortality rate, relative risk of mortality, standardized mortality ratio, or with-condition mortality rate. Studies with no clearly defined sample or that drew from specific clinic/patient organizations were excluded. We also added US claims data for 2011 and 2012-2015. No further prevalence or incidence data were added in GBD 2019.

Modelling Strategy

Overview

Parkinson's disease mortality rates have more than doubled since 1980 in high-quality vital registration systems. We have not seen an equivalent increase in prevalence and incidence data sources. Additionally, the greater than 15-fold variation in mortality rates of Parkinson's disease between countries is much greater the three-fold difference in prevalence and incidence between high-income countries. As it is unlikely that case fatality from Parkinson's disease has dramatically increased over the time period and that it would differ by a very large margin between countries, the hypothesis is that certifying and coding practices have changed over time and at a different pace between countries. For GBD 2016, we decided to employ a modelling strategy which we have previously used to model mortality from Alzheimer disease and other dementias, which avoids spurious large trends over time in the fatal component of the burden of Parkinson's disease by making Parkinson's mortality rates consistent with the rates observed in 2016, relative to prevalence in countries that are most likely to certify or code Parkinson's disease as an underlying cause of death. For GBD 2017, we again employed this strategy.

Modelling steps
Fatal modelling for Parkinson's Disease is described in the following steps. The initial steps were not re-run in GBD 2019, and so the Multiple Cause of Death (MCOD) Parkinson's disease inputs were identical to those used in the GBD 2017 capstone.

First, we ran a CODEm model for Parkinson's disease and extracted the mortality rates by age, sex, and geography. The covariates used in this intermediary model are displayed below; some have a direction of 0 because this model was run early in the GBD 2019 cycle. The final Parkinson's model has a negative or positive direction specified for all covariates (see final table).

Level	Covariate	Direction
1	Cumulative cigarette consumption (10 years)	-
2	Absolute latitude	+
	Cholesterol (total, mean per capita)	+
	Sanitation (proportion with access)	0
	Improved water source (proportion with access)	0
	Fruit consumption adjusted (g)	-
	Healthcare access and quality index	-
3	Education (years per capita)	-
	Socio-demographic index	+
	Lag distributed income	0

Second, we ran a DisMod-MR 2.1 model with all data on incidence, prevalence, and mortality risk (RR, SMR, or with-condition mortality rates) and a setting of zero remission and extracted prevalence by age, sex, and geography. Studies where the case definition of two of the four cardinal symptoms of Parkinson's disease was not filled were crosswalked to studies using the reference case definition. No random effects were used in the model in order to prevent spurious inflation of regional differences due to differences in measurement and measurement error.

Third, we selected the seven countries (France, England, the United States, the Netherlands, Finland, Scotland, and Wales) with the highest cause-specific mortality rate (from step 1) to prevalence (from step 2) ratio in 2017, which also had an age-standardised prevalence rate greater than 0·0005, and a population greater than 1 million.

Fourth, we used a linear effects regression with dummies on age group and sex to predict excess mortality (i.e., the ratio of cause-specific mortality rate and prevalence) by age and sex, the results of which are found in the tables below.

Table: Fixed effect coefficients of EMR regression. Outcome: ln(EMR)

Independent variables	Coef	Std. error	P value	95% Confidence Interval	
Male	0·288	0·036	0·000	0·218	0·358
Age 40-59	-3·25	0·076	0·000	-3·399	-3·101
Age 60-64	-2·557	0·076	0·000	-2·706	-2·407
Age 65-69	-2·021	0·076	0·000	-2·17	-1·871
Age 70-74	-1·42	0·076	0·000	-1·57	-1·271
Age 75- 80	-0·898	0·076	0·000	-1·047	-0·749
Age 80-84	-0·502	0·076	0·000	-0·651	-0·352
Age 85-89	-0·248	0·076	0·001	-0·397	-0·099
Age 90-94	-0·047	0·076	0·537	-0·196	0·102
Constant	-2·357	0·057	0·000	-2·469	-2·246

Table: Predicted EMR values by age and sex (95% CI)

	Male	Female
Age 40-59	0·005 (0·004 – 0·005)	0·004 (0·003 – 0·004)
Age 60-64	0·01 (0·009 – 0·011)	0·007 (0·007 – 0·008)
Age 65-69	0·017 (0·015 – 0·019)	0·013 (0·011 – 0·014)
Age 70-74	0·031 (0·027 – 0·034)	0·023 (0·02 – 0·025)
Age 75-80	0·051 (0·046 – 0·057)	0·039 (0·035 – 0·043)
Age 80-84	0·076 (0·068 – 0·085)	0·058 (0·052 – 0·064)
Age 85-89	0·099 (0·089 – 0·111)	0·074 (0·066 – 0·083)
Age 90-94	0·12 (0·108 – 0·135)	0·09 (0·081 – 0·1)
Age 95+	0·126 (0·113 – 0·142)	0·095 (0·085 – 0·106)

Fifth, these estimates were added to a second DisMod-MR 2.1 model as pertaining to the full 1990–2017 estimation period. For the countries included in the regression, we allowed them to retain their original EMR values when the age-standardized EMR for a country was higher than the age-standardized EMR prediction generated from the regression. These countries retained their age- and sex-specific ratios and entered those also as pertaining to the full 1990–2017 estimation period. Smoking prevalence was used as a country-level covariate. We excluded data for standardized mortality ratio, with-condition mortality rate, and relative risk as we wanted to estimate cause-specific mortality rates that were consistent with the level of excess mortality from the seven chosen countries in 2017.

Sixth, we took the predictions of cause-specific mortality by age, sex, geography, and year that DisMod-MR 2.1 calculated as being consistent with the data on incidence, prevalence, and the priors on excess mortality from step five. Because DisMod-MR 2.1 produces estimates in five-year intervals only, we expanded the time series by log-linear interpolation; values for 1980-1990 were generated using a regression on the entire time series with Socio-demographic index included as a predictor. We divided this cause-specific mortality by the all-cause mortality used in DisMod to calculate the Parkinson’s disease cause-fraction based on prevalence data and the excess mortality derived from countries most likely to code to Parkinson’s disease as a cause of death.

Seventh, we calculated the difference between this cause-fraction derived from DisMod and the cause-fraction derived from the cause of death data prep process before redistribution in order to get the amount of cause fraction that needed to be retrieved from other causes through the Parkinson’s disease redistribution process.

Eighth, in order to calculate where these Parkinson’s disease deaths should be retrieved from, we analysed multiple cause of death (MCOD) data.

Ninth, for deaths where Parkinson’s disease is the underlying cause of death in the years 2010-2015, we calculated what the underlying cause of death would have been in the counterfactual scenario in which Parkinson’s disease had not been recognized. In order to calculate this counterfactual, we examined the causes listed in part one of the chain of the death certificate. For each death certificate chain we looked across the entire dataset from 1980-2015 and determine what the distribution of underlying causes of death was in individuals with that particular death certificate chain. Then, we assigned the counterfactual deaths proportionally to the causes that are listed as underlying in these death certificates. If, over the time period, there were less than 1000 death certificates that had exactly the same death certificate chain, then we included all death certificate chains that had those same causes, but which could additionally include other causes in the chain as well. To assign counterfactual deaths for these chains, we further subsetted the data to death certificate chains where any of the causes in the original death certificate chain were listed as underlying, determined the distribution of underlying causes of death among just this subset, and then assigned counterfactual deaths proportionally in the same manner.

Tenth, once we determined the counterfactual causes of death stemming from all Parkinson's disease deaths from 2010-2015, we calculated the proportion of deaths by cause that should be Parkinson's disease deaths according to the reference data by taking the counterfactual deaths for each cause and dividing by the sum of the counterfactual deaths for that cause plus the directly coded deaths for that cause.

Eleventh, we applied the proportions to cause of death data in cause fraction space and scaled the cause fractions to the total mortality cause fraction to be retrieved based on the DisMod model. We set caps on the percent of deaths that were moved by age, sex, and cause. The caps were determined by finding the 95th percentile of the percentages of deaths moved in each age-sex-cause category across all 5-star VR locations. The COD data is then processed using general redistribution strategies and noise reduction.

Finally, the data derived from this process was used in a final CODEm model, using the same covariates as the original CODEm model. These covariates were adjusted for this model in GBD 2019 so that every covariate had a specified directionality (see table below), and with some adjustments for level. These results were then adjusted through CodCorrect and become the final cause of death estimates for Parkinson's disease.

Level	Covariate	Direction
1	Cumulative cigarette consumption (10 years)	-
	Fruit consumption adjusted (g)	-
2	Absolute latitude	+
	Cholesterol (total, mean per capita)	+
	Sanitation (proportion with access)	+
	Improved water source (proportion with access)	+
	Healthcare access and quality index	-
3	Education (years per capita)	-
	Socio-demographic index	+
	Lag distributed income	+

The following plots show the influence of each covariate on the four CODEm models (male global, male data rich, female global, and female data rich). A positive standardized beta (to the right) means that the covariate was associated with increased death. A negative standardized beta (to the left) means the covariate was associated with decreased death.

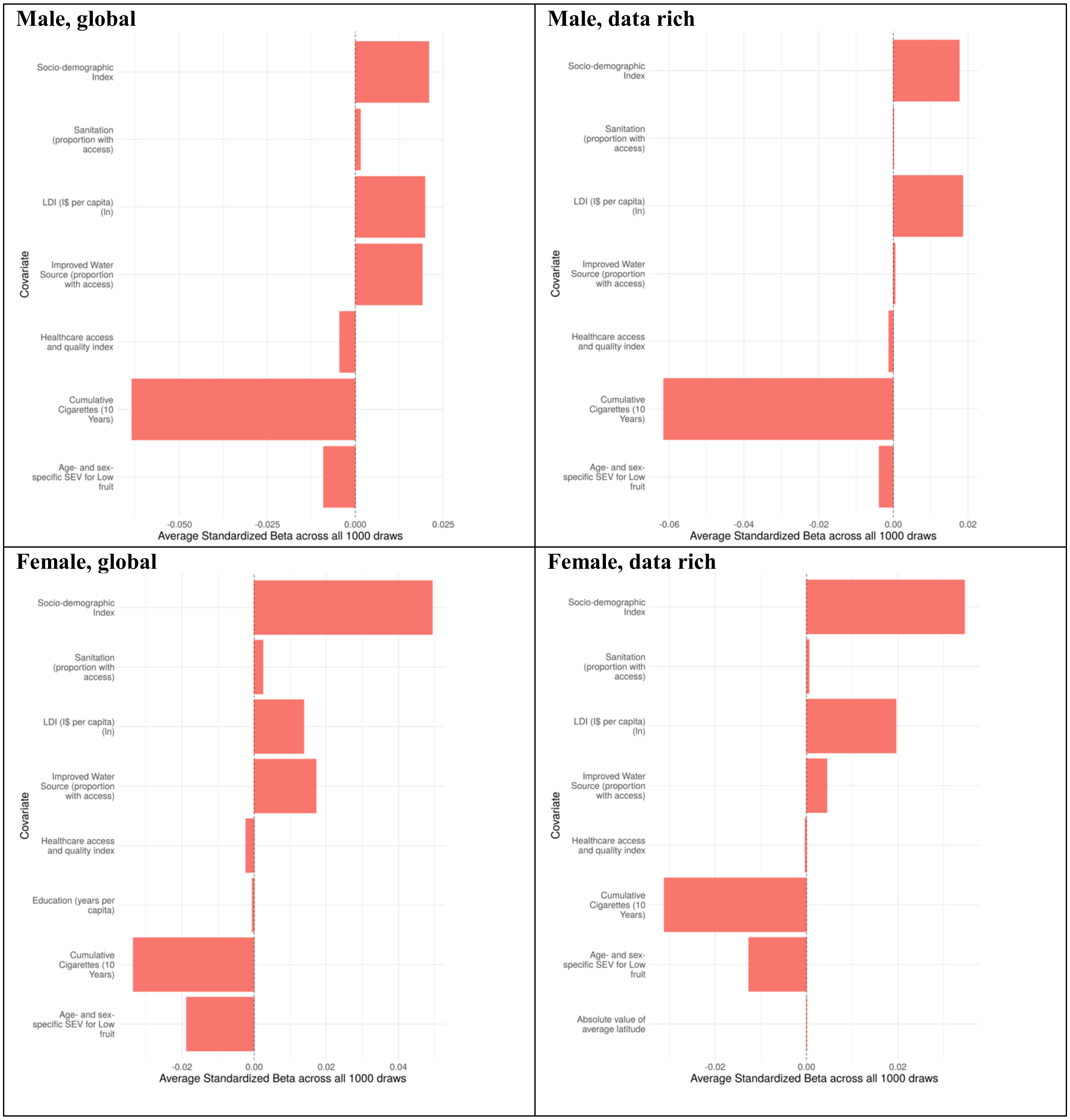
Male, global
Socio-demographic Index
Sanitation (proportion with access)
LDI (I$ per capita) (ln)
Improved Water Source (proportion with access)
Healthcare access and quality index
Cumulative Cigarettes (10 Years)
Age- and sex-specific SEV for Low fruit
Covariate
-0.050
-0.025
0.000
0.025
Average Standardized Beta across all 1000 draws
Male, data rich
Socio-demographic Index
Sanitation (proportion with access)
LDI (I$ per capita) (ln)
Improved Water Source (proportion with access)
Healthcare access and quality index
Cumulative Cigarettes (10 Years)
Age- and sex-specific SEV for Low fruit
Covariate
-0.06
-0.04
-0.02
0.00
0.02
Average Standardized Beta across all 1000 draws
Female, global
Socio-demographic Index
Sanitation (proportion with access)
LDI (I$ per capita) (ln)
Improved Water Source (proportion with access)
Healthcare access and quality index
Education (years per capita)
Cumulative Cigarettes (10 Years)
Age- and sex-specific SEV for Low fruit
Covariate
-0.02
0.00
0.02
0.04
Average Standardized Beta across all 1000 draws
Female, data rich
Socio-demographic Index
Sanitation (proportion with access)
LDI (I$ per capita) (ln)
Improved Water Source (proportion with access)
Healthcare access and quality index
Cumulative Cigarettes (10 Years)
Age- and sex-specific SEV for Low fruit
Absolute value of average latitude
Covariate
-0.02
0.00
0.02
Average Standardized Beta across all 1000 draws

4.4. Parkinson's Disease (non-fatal modelling)

Flowchart

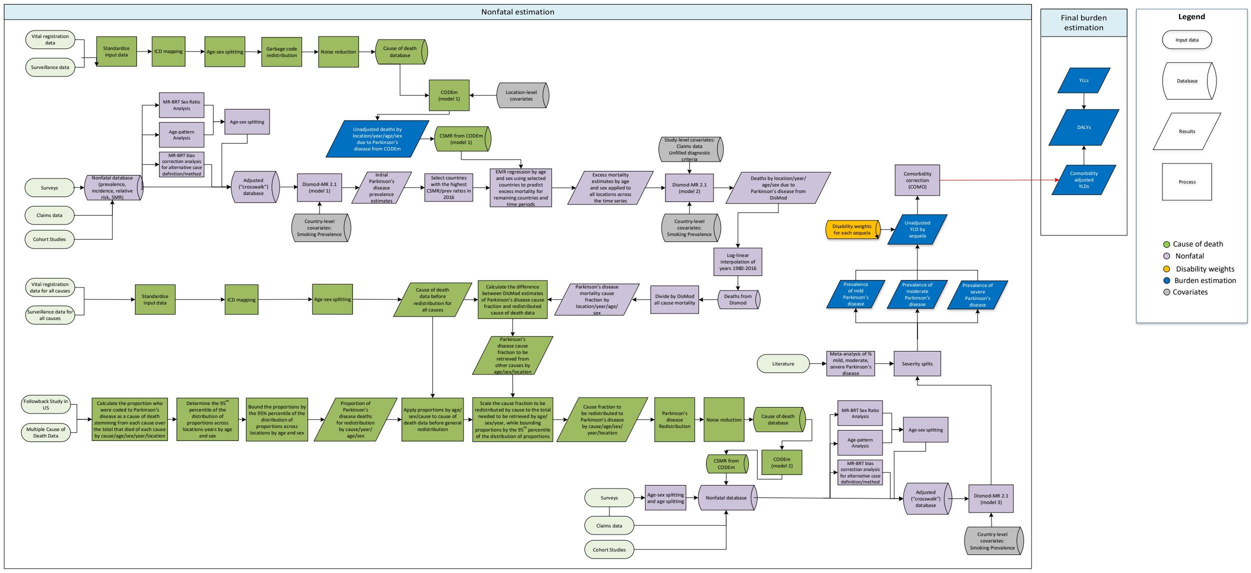

Case definition

Parkinson's disease is a chronic, degenerative, and progressive neurological condition typified by the loss of motor mobility and control – most notably tremors. The corresponding ICD-10 codes are G20, G21, and G22. Our case definition for GBD is the presence of at least two of the four primary symptoms: (1) tremors/trembling, (2) bradykinesia, (3) stiffness of limbs and torso, and (4) posture instability.

Unlike most causes in the Global Burden of Disease project, Parkinson's disease mortality and morbidity estimates are modelled jointly. This is because of marked discrepancies between prevalence data and cause of death data. Specifically, prevalence data suggest little to no variation over time (eg, 1990–2017) whereas age-standardised mortality rates in vital registrations in high-income countries have increased multiple times over this same period. Additionally, prevalence variation between countries is much smaller than the variation in death rates assigned to Parkinson's disease in vital registration. We attribute these discrepancies to changing coding practices rather than epidemiological change.

Because of this joint procedure, descriptions of the mortality estimation process are included where relevant, but see the Parkinson's disease fatal write up for more details.

Model inputs

To inform our estimates of burden due to Parkinson's disease, we use mortality data from vital registration systems, as well as prevalence data from surveys and administrative data such as claims sources.

An updated systematic review was conducted from September 2015 to August 2017, and the search terms (below) were set to capture studies for Parkinson's disease. This search term resulted in 660 initial hits with 20 sources marked for extraction. Studies with no clearly defined sample or that drew from specific clinic/patient organizations were excluded.

Search terms: *(Parkinson disease[Title/Abstract] OR Parkinson's disease[Title/Abstract]) AND (epidemiology[Title/Abstract] OR prevalence[Title/Abstract] OR incidence[Title/Abstract]) AND ("2015/09/31"[PDAT] : "2017/08/23"[PDAT])*

Studies using non-representative populations are excluded from modelling. Certain studies have been outliered on a case-by-case basis due to subsequent review and exclusion due to inappropriateness of the study design, or case ascertainment that conflict with existing gold-standard data – where possible. We exclude claims data from the year 2000 because these data are systematically lower than other years. As of GBD 2017, a prevalent case is identified from claims data where an individual has one inpatient visit, two outpatient visits, or one outpatient and one inpatient visit (arguing that a single mention of a code for PD in an individual could be a provisional diagnosis prior to confirmation).

Modelling strategy

Studies with age and sex detail separately were split into age- and sex-specific data points. Standard GBD sex splitting methods were used for studies with only "both" sex data points: we modeled the ratio of female/male prevalence in MR-BRT and then calculated male prevalence:

$$prev_{male} = prev_{both} * \frac{pop_{both}}{(pop_{male} + ratio * pop_{female})}$$

And then calculated female prevalence:

$$prev_{female} = ratio * prev_{male}$$

We also split data points where the age range was greater than 25 years. In GBD 2017, age splitting was based on the age pattern from the United States, where we had the most detail by age. In GBD 2019, age splitting was based on the global age pattern from a Dismod model that only used input data with less than a 25-year age range. Data are location split if they are at country level and cover a number of subnationals (or are UK data).

For GBD 2019, adjustment factors for all study-level covariates were determined using matched data (by year, age, sex, location) for reference and alternative case definitions in a logit difference network meta-regression. Study-level covariates included studies that were not population representative, excluded nursing homes from their estimates, followed UKPD Brain Bank diagnosis criteria, followed MDS diagnosis criteria, or did not explicitly define diagnosis criteria. Country covariates are used to inform global patterns. Cause-specific mortality results from the final fatal Parkinson's disease model is pulled into the final non-fatal DisMod model. The following tables provide an overview of the study-level and country covariates used in the Parkinson's disease DisMod MR-2.1 model.

MR-BRT Crosswalk Adjustment Factors for Parkinson's Disease

Data input	Reference or alternative case definition	Gamma	Beta Coefficient, Logit (95% CI)	Adjustment factor*
2 of 4 diagnostic criteria	Ref	0·48	---	---
Not population representative	Alt		0·03 (-0·95 – 1·04)	0·51
Excluded nursing homes	Alt		0·01 (-0·95 – 0·95)	0·50
UKPD Brain Bank criteria	Alt		0·01 (-1·46 – 0·47)	0·50
MDS criteria	Alt		0·14 (-0·83 – 1·54)	0·53
No explicit criteria	Alt		0·01 (-0·56 – 1·37)	0·50

Covariates. Summary of covariates used in the Parkinson's Disease DisMod-MR meta-regression model

**Covariate**	**Type**	**Parameter**	**Exponentiated beta (95% Uncertainty Interval)**
Smoking prevalence (age-standardized)	Prevalence	-1·15	0·32 (0·28 – 0·36)
Healthcare access and quality index	Excess mortality rate	-0·025	0·98 (0·97 – 0·98)

Severity splits

As in GBD 2013, we use Hoehn and Yahr stages to determine severity. However, for GBD 2017 onward, the cutpoints were updated in order to more accurately correspond with the lay descriptions of severities. Specifically, a Hoehn and Yahr stage 4 now corresponds to a designation of severe, where before it was classified as moderate.

Severity	Stage
Mild	≤2·0
Moderate	2·5-3·5
Severe	≥4

Severity estimates were generated by multiplying estimates of prevalence (country-year-sex-age-specific) by the fractions of mild, moderate, and severe PD, and 95% confidence intervals were estimated by taking 1000 draws. The following table provides the lay description and disability weights associated with Parkinson's disease.

Severity level	Lay description	DW (95% CI)
Mild	Has mild tremors and moves a little slowly, but is able to walk and do daily activities without assistance.	0·010 (0·005–0·019)
Moderate	Has moderate tremors and moves slowly, which causes some difficulty in walking and daily activities. The person has some trouble swallowing, talking, sleeping, and remembering things.	0·267 (0·181–0·372)
Severe	Has severe tremors and moves very slowly, which causes great difficulty in walking and daily activities. The person falls easily and has a lot of difficulty talking, swallowing, sleeping, and remembering things.	0·575 (0·396–0·730)

4.5. Idiopathic Epilepsy (fatal modelling)

Flowchart

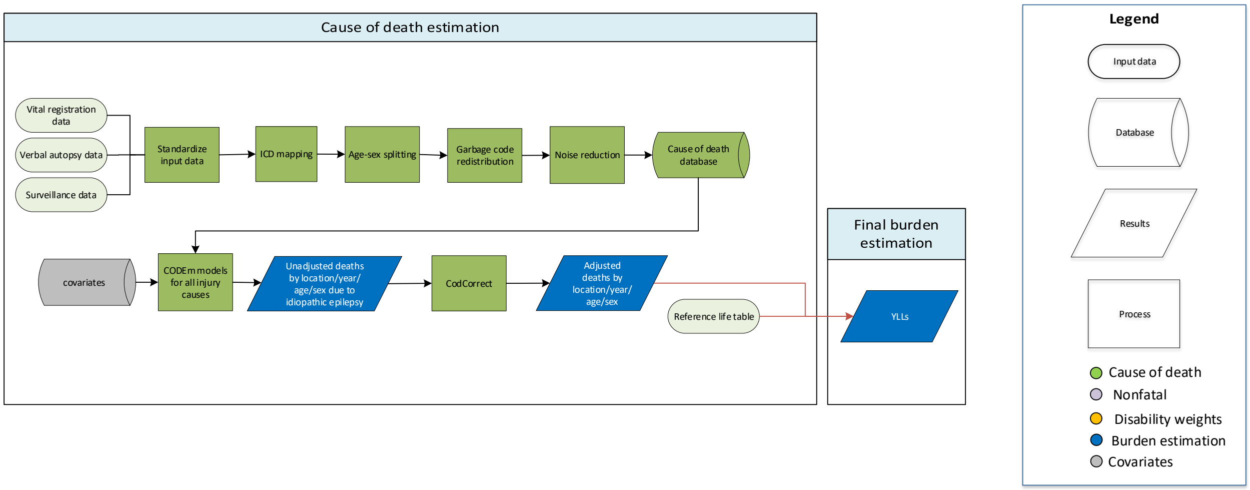

Input Data and Methodological Summary for Idiopathic Epilepsy

Input data
Data used to estimate epilepsy mortality included vital registration (VR), verbal autopsy, and China mortality surveillance data from the cause of death (COD) database. Our outlier criteria were to exclude data points that were (1) implausibly high or low relative to global or regional patterns, (2) substantially conflicted with established age or temporal patterns, or (3) substantially conflicted with other data sources based from the same locations or locations with similar characteristics (i.e., socio-demographic index).

Modelling strategy
The standard CODEm modelling approach (detailed in a appendix section 3.1) was used to estimate deaths due to idiopathic epilepsy. Separate models were conducted for male and female mortality, and the age range for both models was 28 days – 95+ years. Changes to these models relative to GBD 2017, and the complete list of covariates used in GBD 2019 are displayed below. Unadjusted death estimates were adjusted using CoD Correct to produce final estimates of YLLs.

Table 1. Covariates used in Idiopathic Epilepsy mortality modelling

Level	Covariate	Direction
1	Pigs (per capita)	+
	SEV scalar: epilepsy	+
	Mean systolic blood pressure (mmHg)	+
2	Health access and quality index	-
	Mean body mass index	+
	Mean serum total cholesterol (mmol/L)	+
3	Cumulative cigarettes (10 years)	+
	Cumulative cigarettes (5 years)	+
	Education (years per capita)	-
	Log LDI (per capita)	-
	Socio-demographic Index	-

The following plots show the influence of each covariate on the four CODEm models (male global, male data rich, female global, and female data rich). A positive standardized beta (to the right) means that the covariate was

associated with increased death. A negative standardized beta (to the left) means the covariate was associated with decreased death.

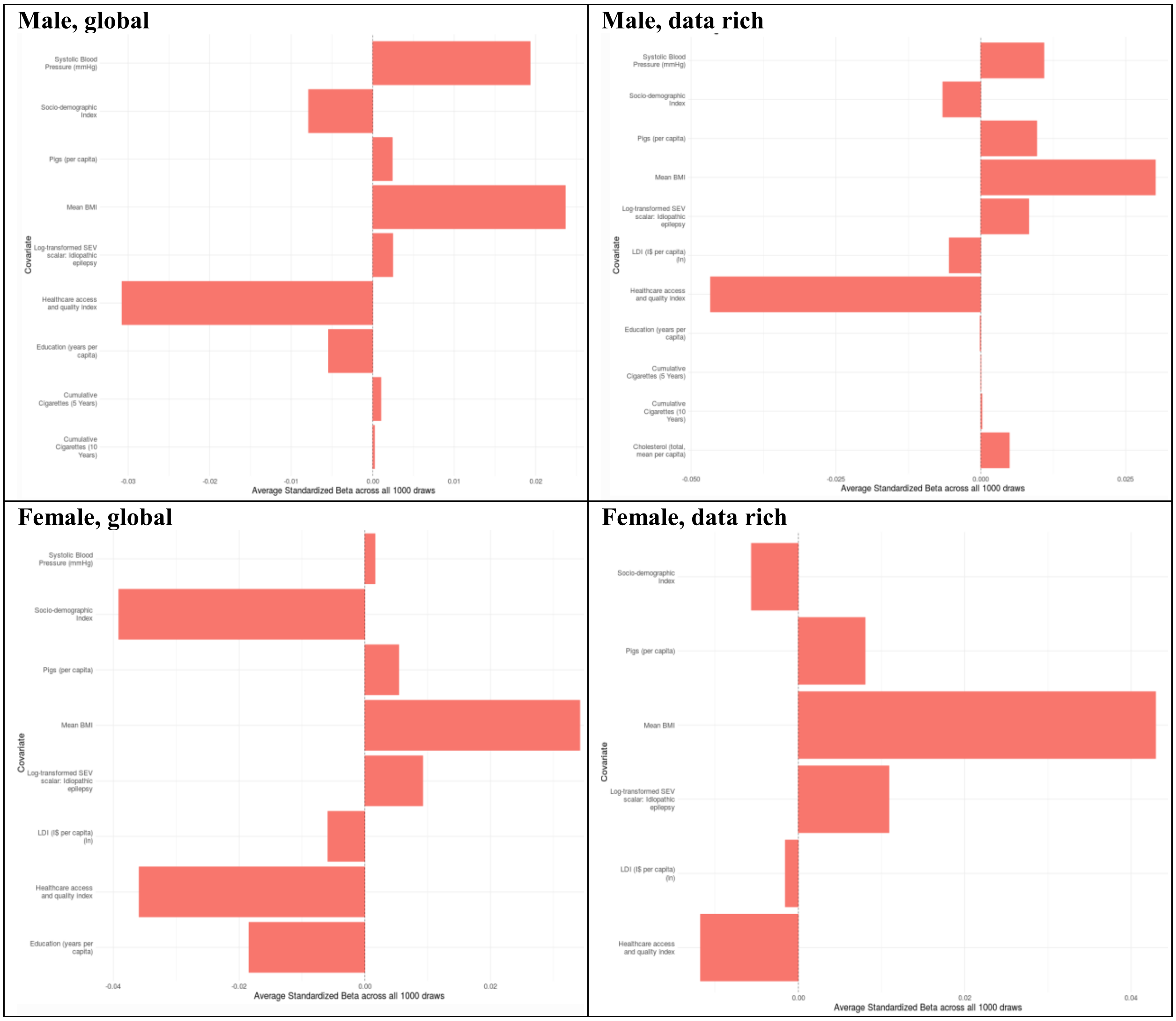

4.6. Epilepsy impairment envelope (non-fatal modelling)

Flowchart

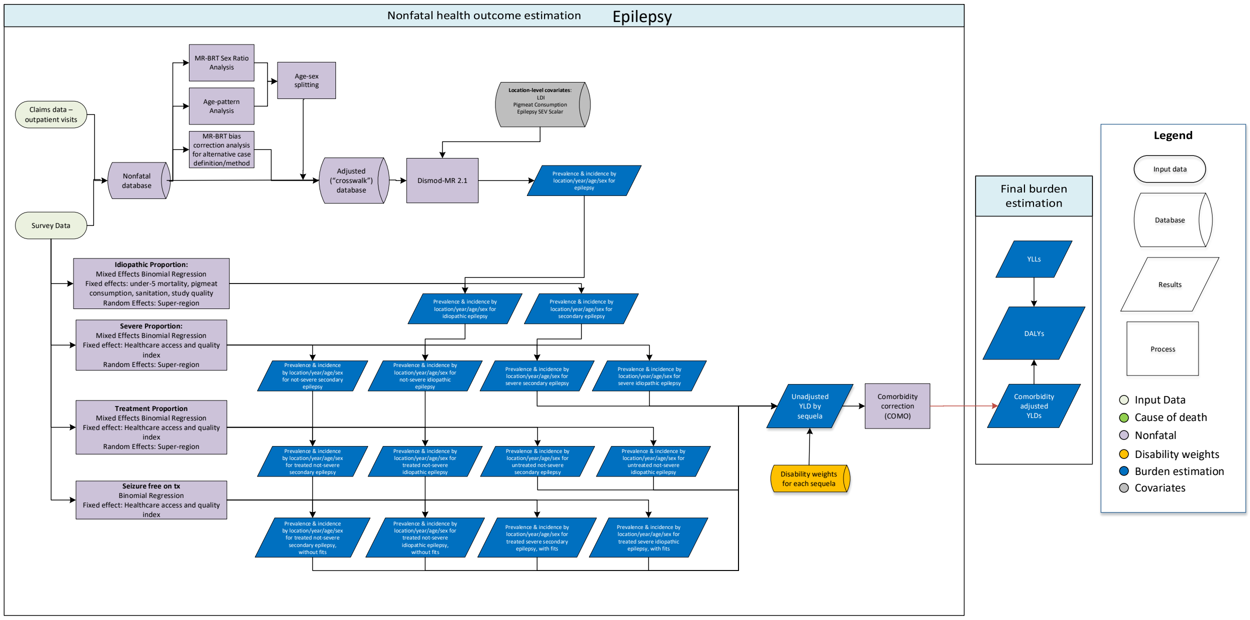

Case definition

Since GBD 2013, we have used the following definitions from the "Guidelines for Epidemiologic Studies on Epilepsy": 1) Epilepsy: a condition characterised by recurrent (two or more) epileptic seizures, unprovoked by any immediate identified cause, and 2) "Active" epilepsy: a prevalent case of active epilepsy is defined as a person with epilepsy who has had at least one epileptic seizure in the previous five years, regardless of antiepileptic drug (AED) treatment. We also use the following ICD-10 codes for epilepsy: G40 (Neuro, epilepsy, total) and G41 (Neuro, epilepsy, status epilepticus). We define severe epilepsy as having seizures one or more times per month.

Input data and processing

Data inputs

The primary data inputs for the epilepsy modelling strategy were measurements of prevalence, incidence, remission rate, excess mortality rate, relative risk of mortality, standardised mortality ratio, or with-condition mortality rate for all epilepsy, regardless of cause, severity, or treatment status.

For GBD 2016, we conducted a systematic review covering 10/1/2014 to 10/7/2016 using the following search string:

("2014/10/01"[PDAT] : "2016"[PDAT]) AND ("epilepsy"[MeSH Terms] OR "epilepsy, partial, motor"[MeSH Terms] OR "epilepsy, benign neonatal"[MeSH Terms] OR "epilepsy, reflex"[MeSH Terms] OR "myoclonic epilepsy, juvenile"[MeSH Terms] OR "epilepsy, frontal lobe"[MeSH Terms] OR "epilepsy, complex partial"[MeSH Terms] OR "epilepsy, post-traumatic"[MeSH Terms] OR "epilepsy, temporal lobe"[MeSH Terms] OR "epilepsy, absence"[MeSH Terms] OR "epilepsy, tonic-clonic"[MeSH Terms] OR "epilepsies, myoclonic"[MeSH Terms] OR "epilepsies, partial"[MeSH Terms] OR epilepsy[Title/Abstract]) AND (incidence[Title/Abstract] OR prevalence[Title/Abstract]) NOT(animals[MeSH] NOT humans[MeSH]).

We included representative, population-based surveys that reported on prevalence, incidence, remission rate, excess mortality rate, relative risk of mortality, standardised mortality ratio, or with-condition mortality rate. We excluded

studies with no clearly defined sample (eg, among clinic attenders or patient organisation members with non-specific or non-representative catchment area).

Additional data inputs include data on the proportion of epilepsy that is primary or idiopathic, the proportion of epilepsy that is severe (one or more fits per month), the proportion of epilepsy that is untreated (the treatment gap), and the proportion of treated epilepsy that is treated without fits (no fits reported in the preceding year).

Data processing
For GBD 2019, we started with the final age split dataset used in GBD 2017 - raw data with large age ranges were split into 5 year age bins using the age pattern generated from a Dismod model with input data of only less than 25 years age range. Standard GBD sex splitting methods were used for studies with only "both" sex data points. We modeled the ratio of female/male prevalence in MR-BRT and calculated male prevalence:

$$prev_{male} = prev_{both} * \frac{pop_{both}}{(pop_{male} + ratio * pop_{female})}$$

And then calculated female prevalence:

$$prev_{female} = ratio * prev_{male}$$

For epilepsy, the modelled female/male ratio demonstrated a higher prevalence in males, and was used to proportionally split "both" sex data points into male and female data points (as seen in the figure below).

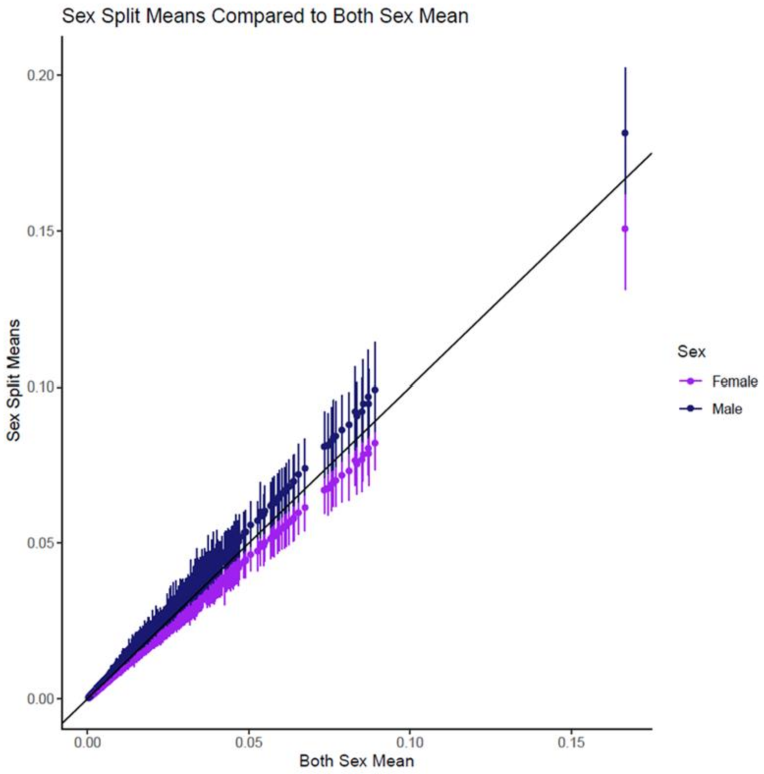

For GBD 2019, adjustment factors for all study-level covariates were determined using matched data (by year, age, sex, location) for reference and alternative case definitions in a log ratio network meta-regression. Studies that asked for lifetime recall were crosswalked to the reference definition for epilepsy.

The table below shows adjustment factors estimated using MR-BRT.

MR-BRT Crosswalk Adjustment Factors for Epilepsy Impairment Envelope

Data input	Reference or alternative case definition	Gamma	Beta Coefficient, Log (95% CI)	Adjustment factor*
	Ref	N/A	N/A	N/A
Marketscan	Alt	0·26	-0·86 (-1·40 to -0·33)	0·42
Marketscan 2000	Alt	0·37	-1·15 (-1·90 to -0·40)	0·32
Recall lifetime	Alt	0·25	0·20 (-0·29 to 0·70)	1·22
*Adjustment factor is the transformed Beta coefficient in normal space, and can be interpreted as the factor by which the alternative case definition is adjusted to reflect what it would have been if measured as the reference. Note that all of these crosswalks were run separately as opposed to in a network analysis, but all were adjusted to the same reference definition.				

Modelling strategy

We modelled the prevalence of epilepsy in two steps: first, we created an epilepsy impairment envelope. Second, we split the envelope into primary (or idiopathic) and secondary epilepsies. Each of these were subdivided into "severe" (on average one or more fits per month) and "non-severe." Non-severe cases were subdivided into "treated" and "un-treated." Finally, "treated" cases were divided into "treated cases with fits" (between one and 11 fits on average in the preceding year) and "treated cases without fits" (no fits reported in the preceding year).

In the first step, we used DisMod-MR 2.1 for the epilepsy impairment envelope to model a consistent fit between incidence, prevalence, remission, and standardised mortality ratio data.

We also included the SEV epilepsy scalar, which summarises the epilepsy risk exposure level for each country, as a predictive covariate on prevalence. We included cause-specific mortality rate (CSMR) results from the epilepsy mortality model as input data to the DisMod model. Where age-specific prevalence data were available, we calculated excess mortality rate (EMR) from prevalence and CSMR. We included the log of the lag-distributed income (LDI) as a covariate on EMR to account for lower mortality in developed countries. We included Bayesian priors on remission to account for the scarcity of remission data. We set bounds on remission from 0 to 0.25 from age 0–60 and 0 to 0·05 from age 61–100. The table below indicates the covariates used in the estimation process, as well as parameters, betas, and exponentiated betas.

Covariates. Summary of covariates used in the epilepsy impairment envelope DisMod-MR meta-regression model

Covariate	Type	Parameter	Exponentiated beta (95% Uncertainty Interval)
Log-transformed age-standardized SEV scalar: Idiopathic epilepsy	Prevalence	0·76	2·14 (2·12-2·18)
LDI ($ per capita)	Excess mortality rate	-0·55	0·58 (0·38-0·87)

In the second step, we used mixed effects generalised linear models (binomial family) run in GBD 2017 to predict the proportion of idiopathic epilepsy, the proportion of severe epilepsy, the proportion of treated epilepsy and the proportion of epilepsy that is treated without fits.

Because not all of the data on the proportion of idiopathic epilepsy use optimal case finding methods (using CT scans or MRIs in addition to EEGs in order to diagnose secondary epilepsy), we first run an initial linear regression model with a covariate on study quality. We then use the beta from this model to crosswalk studies with non-optimal case finding methods to those with adequate methods. The adjusted data are then used in the regression for the proportion of epilepsy that is idiopathic, with a fixed effect on SDI as well as a random effect on super-region.

We used similar models to predict the proportion of severe epilepsy and treatment gap based on the reported proportions extracted from the systematic review. To predict the proportion of severe epilepsy and the treatment gap, we used mixed-effects models with a fixed effect on the log of HAQ Index and a random effect on super-region.

For the regression to determine the proportion of treated epilepsy cases that have not had a fit in the last year, there is a much smaller dataset, and therefore we cannot use a random effect in the model. Therefore, we use generalised linear model (binomial family) to generate predictions for the proportion of treated epilepsy that is seizure-free with a fixed effect on the log of HAQ Index.

We tested a fixed effect on epilepsy cause-specific mortality, under-5 mortality rate, sanitation, and pig meat consumption as well as random effects on region and country in different models, but they did not improve the models. We generated 1000 draws of country-specific estimates for each year between 1980 and 2017 for each of the models. The table below shows the betas from these regressions.

Regression	covariate	beta	SE
Idiopathic	Study quality	0·75	0·59
Idiopathic	SDI	1·39	1·12
Severe	HAQ Index	-1·23	1·05
Treatment gap	HAQ Index	-3·54	1·37
Treated without fits	HAQ Index	2·49	1·87

Severity splits & disability weights

The table below illustrates the severity levels, descriptions, and disability weights associated with epilepsy. These are calculated using regressions from literature (ie, frequency of seizures).

Severity level	Lay description	Disability weights (95% CI)
severe (seizures ≥ once per month)	This person has sudden seizures one or more times each month, with violent muscle contractions and stiffness, loss of consciousness, and loss of urine or bowel control. Between seizures the person has memory loss and difficulty concentrating.	0·552 (0·375–0·71)
less severe (seizures < once per month)	This person has sudden seizures two to five times a year, with violent muscle contractions and stiffness, loss of consciousness, and loss of urine or bowel control.	0·263 (0·173–0·367)
Treated without fits	This person has a chronic disease that requires medication every day and causes some worry but minimal interference with daily activities.	0·049 (0·031–0·072)

4.7. Multiple Sclerosis (fatal modelling)

Flowchart

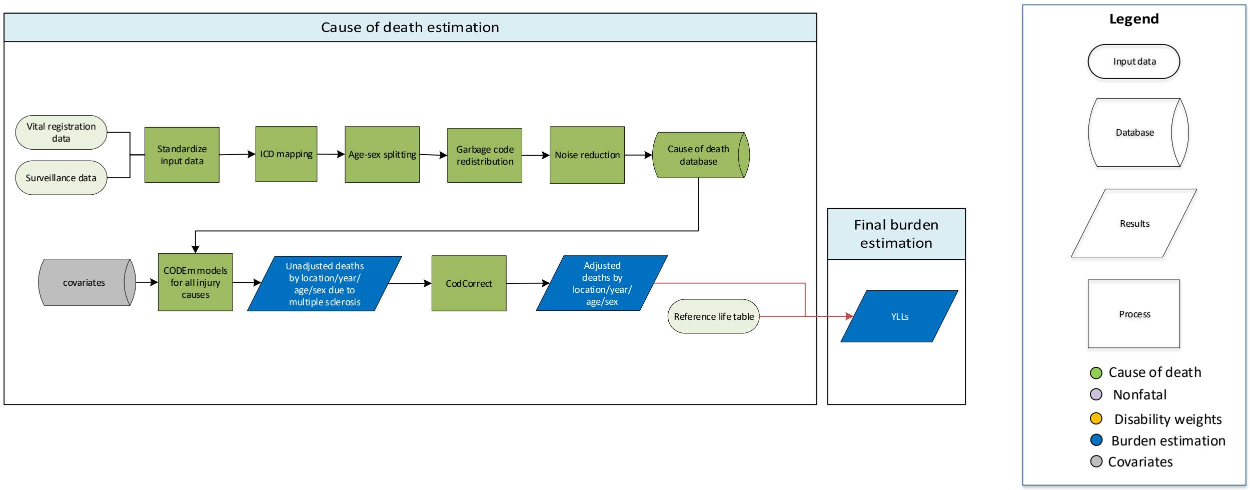

Input Data and Methodological Summary for Multiple Sclerosis

Input data

Data used to estimate multiple sclerosis included vital registration and surveillance data from the cause of death (COD) database. Our outlier criteria were to exclude data points that (1) were implausibly high or low, (2) substantially conflicted with established age or temporal patterns, or (3) substantially conflicted with other data sources conducted from the same locations or locations with similar characteristics (ie, Socio-demographic Index). In particular, where data-processing could not resolve discrepancies between different coding systems for the same location over time, one system was selected as more reliable and the other was excluded. In particular, this affected Kazakhstan, where the conversion from ICD9-BTL tabulated vital registration data (for years1981-2003) to ICD10-coded data (for year 2013 onwards) led to an implausible 5-fold increase between 1980 and 2017 and 2017 estimates more than two-fold greater than anywhere else in the world. The ICD10-coded data were excluded.

Modelling strategy

The standard CODEm modelling approach was used to estimate deaths due to multiple sclerosis. Separate models were conducted for male and female mortality, and the age range for both models was 5-95+ years (differing from previous years where the age range was 20-95+ years). The linear floor was set to 0·0001. Key changes from GBD 2017 and the full list of covariates used in GBD 2019 are displayed below. Unadjusted death estimates were adjusted using CoDCorrect to produce final estimates of YLLs.

Table 1. Covariates used in Multiple Sclerosis mortality modelling

Level	Covariate	Direction
1	Absolute value of average latitude	+
2	Mean serum total cholesterol (mmol/L)	+
	Health care access and quality index	-
3	Cumulative cigarettes (10 years)	+
	Cumulative cigarettes (5 years)	+
	Education (years per capita)	-
	Log-transformed LDI (per capita)	-
	Smoking prevalence	+
	Socio-demographic Index	+

The following plots show the influence of each covariate on the four CODEm models (male global, male data rich, female global, and female data rich). A positive standardized beta (to the right) means that the covariate was associated with increased death. A negative standardized beta (to the left) means the covariate was associated with decreased death.

Male, global

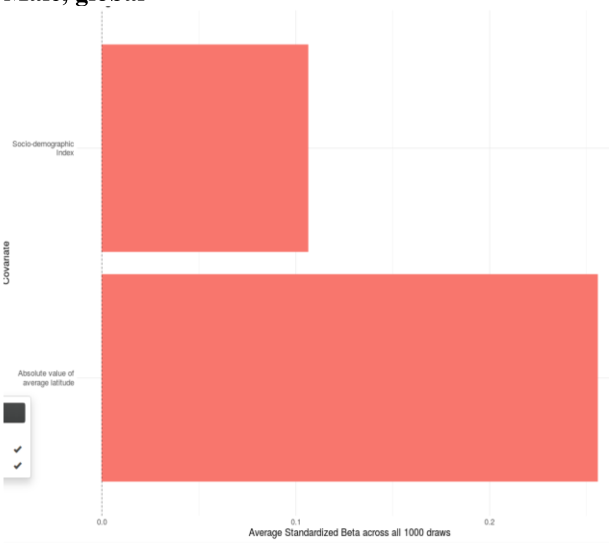

Male, data rich

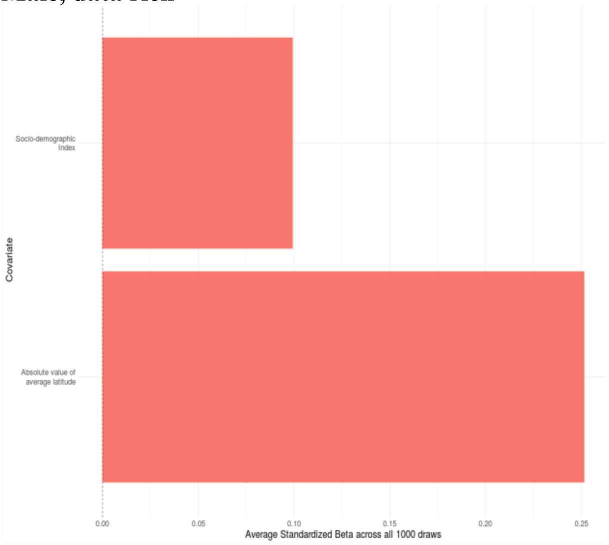

Female, global

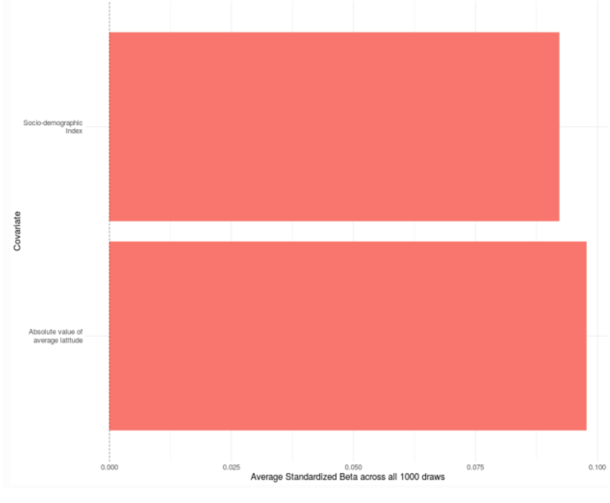

Female, data rich

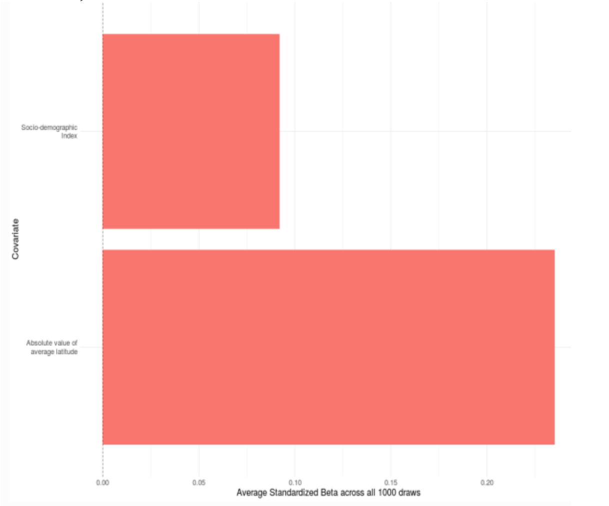

4.8. Multiple sclerosis (non-fatal modelling)

Flowchart

Multiple sclerosis

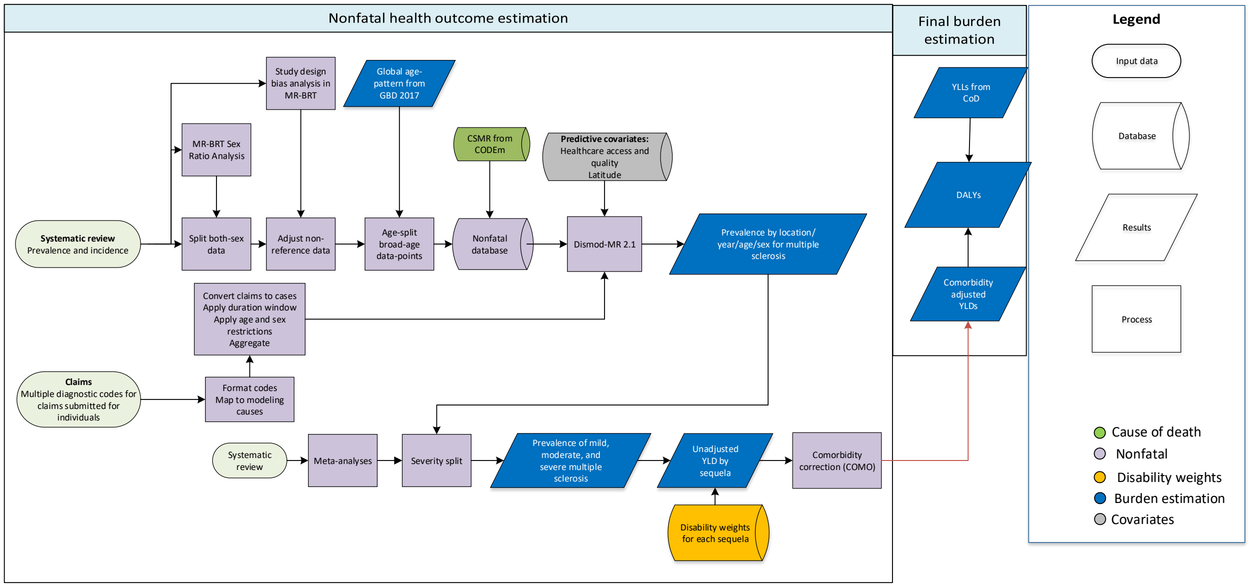

Input data and methodological summary
Case definition

Multiple sclerosis (MS) is a chronic, degenerative, and progressive neurological condition typified by the damaging of the myelin sheaths. McDonald's criteria for diagnosis are considered the contemporary gold standard. For GBD 2019, as for previous rounds, diagnosis by McDonald's criteria, other published criteria (such as Poser, Schumacher, or McAllen criteria), and clinical neurological exam are all treated as reference. The ICD-10 code for MS is G35.

Input data and processing

The data underpinning estimates of burden due to MS are generally of two types. The first are representative, population-based, cross-sectional or longitudinal studies reported in peer-reviewed journals and identified via a search-string-based review, last updated for GBD 2017 and described in previous reports. Estimates of epidemiologic measures (prevalence, incidence, *etc.*) were manually extracted from these publications. The second type are claims data as obtained and processed by the GBD Clinical Informatics team. An individual was extracted from claims data as a prevalent case if they had any peptic ulcer disease code as any diagnosis in one or more inpatient encounters or two or more outpatient encounters.

For studies that reported epidemiologic measures (generally prevalence or incidence) by age for both sexes combined, and also by sex for all ages combined, we calculated the sex-ratio of cases in that study and applied it to the age-specific measures to estimate age-sex-specific measures.

To estimate sex-specific measures from studies that reported only for both sexes combined, we modelled the log sex ratio in MR-BRT using all sex-specific measurements from all other studies in the database and combined these with the GBD sex-specific population estimates for the relevant age-group. For prevalence, this estimate was 0·63 (0·069 to 1·2); for incidence this estimate was 0·86 (0·53 to 1·2). These were applied by calculating male prevalence:

$$prev_{male} = prev_{both} * \frac{pop_{both}}{(pop_{male} + ratio * pop_{female})}$$

and then calculating female prevalence:

$$prev_{female} = ratio * prev_{male}$$

(Equivalent equations were used for incidence.)

After extraction and processing, some studies were marked as outliers and excluded on a case-by-case basis if they were inconsistent with established regional or temporal trends or if concerns about study quality were identified during extraction and processing.

Modelling strategy

Compartmental model
We used DisMod 2.1 as the main analytical tool for the MS estimation process. Inputs included prevalence and incidence data, as described above, as well as the cause-specific mortality rate (CSMR) estimated in the GBD causes of death analysis, and excess mortality rate (EMR) obtained by dividing CSMR by prevalence data-points. Prior settings included zero remission for all ages, no incidence or excess mortality for persons under 5 years old, and incidence limited to less than 0·000005 after the age of 60 years. We also constrained the super-region random effects for prevalence, incidence, and excess mortality to -1 and 1 for all locations except Greenland, United States, and Canada, where location random effects for incidence were constrained to -4, 2 and 2, respectively.
We employed the following covariates to improve model predictions:

Covariate	Measure	Beta coeff (95% CI)	Exponentiated
Absolute value of average latitude	prevalence	0·041 (0·037 to 0·042)	1·04 (1·04 to 1·04)
Absolute value of average latitude	incidence	0·041 (0·036 to 0·045)	1·04 (1·04 to 1·05)
Healthcare Access and Quality index	excess mortality rate	-0·027 (-0·037 to -0·022)	0·97 (0·96 to 0·98)

As described in the literature, extreme latitude is associated with higher prevalence and incidence of MS, although the pathway to explain the association is not understood. Our operationalisation of latitude is created by a population-weighted average of latitude by country and taking the absolute value. The underlying population distribution rasters are part of the Gridded Population of the World dataset.

Although there are no known cures for MS, we expect disease management to differ globally – largely as a function of available resources. To capture this, we use the Healthcare Access and Quality index covariate to capture this relationship in the estimation of excess mortality.

Severity splits
As we have done since GBD 2013, we used Kurtzke's Expanded Disability Status Scale (EDSS) to determine severity splits for MS. The EDSS scores corresponding to each severity are as follows:
Asymptomatic: EDSS = 0
Mild: 0 < EDSS ≤ 3·5
Moderate: 3·5 < EDSS ≤ 6·5
Severe: 6·5 < EDSS ≤ 9·5

The table below illustrates severity levels, lay descriptions, and DWs.

Severity level	Lay description	DW (95% CI)
Asymptomatic	-	0<br>(0-0)

Severity level	Lay description	DW (95% CI)
Mild	Has mild loss of feeling in one hand, is a little unsteady while walking, has slight loss of vision in one eye, and often needs to urinate urgently.	0·183 (0·124–0·253)
Moderate	Needs help walking, has difficulty with writing and arm coordination, has loss of vision in one eye and cannot control urinating.	0·463 (0·313–0·613)
Severe	Has slurred speech and difficulty swallowing. The person has weak arms and hands, very limited and stiff leg movement, has loss of vision in both eyes and cannot control urinating.	0·719 (0·534–0·858)

Because not all sources had information on the number of cases with EDSS stage 0, instead reporting on a mild category, we implemented a two-step meta-analysis strategy. First, we subsetted the studies to those that reported on the number of cases with EDSS stage 0, and did meta-analyses on the proportion of asymptomatic and mild cases. Then, we conducted meta-analyses on the full dataset to get the proportion mild, moderate, and severe, and we squeezed the asymptomatic and mild categories from the previous meta-analyses into the mild category established by the meta-analysis on the full dataset.

4.9. Headaches (non-fatal modelling)

Flowchart

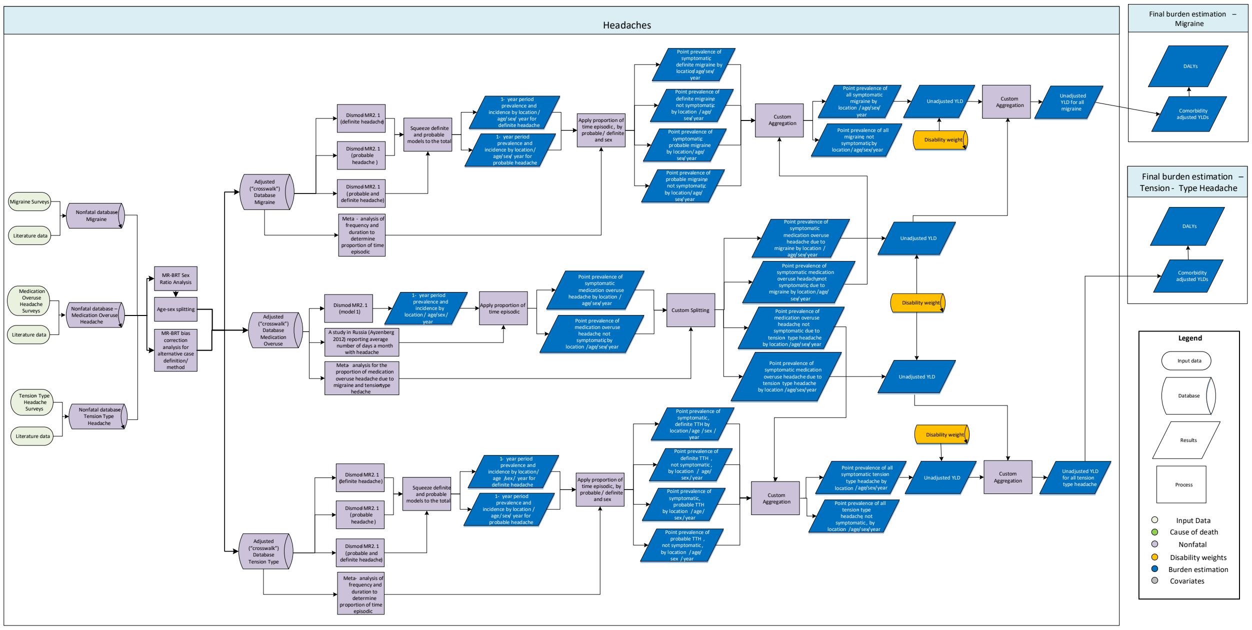

Input Data and Methodological Summary for Headaches

Case definition

Migraine

Migraine is a disabling primary headache disorder, typically characterised by recurrent moderate or severe unilateral pulsatile headaches. The two major types are migraine without aura and migraine with aura (transient neurological symptoms). In GBD, we do not distinguish between migraine with and without aura as most epidemiological studies report on overall migraine only. The reference diagnostic criteria for migraine are from the International Classification of Headache Disorders (ICHD)-3, which describe five criteria:

1. At least five attacks fulfilling criteria 2-5
2. Headache attacks lasting 4-72 hr (untreated or unsuccessfully treated)
3. Headache has at least two of the following four characteristics:
    a. Unilateral location
    b. Pulsating quality
    c. Moderate or severe pain intensity
    d. Aggravation by or causing avoidance of routine physical activity
4. During headache at least one of the following:
    a. Nausea and/or vomiting
    b. Photophobia and phonophobia
5. Not better accounted for by another ICHD-3 diagnosis

Definite migraine is headache that satisfies all the criteria outlined above, while probable migraine satisfies all of the above criteria except one. Studies that have looked at the reasons for cases with probable headache not fulfilling criteria definite diagnosis have suggested that most often it is the duration criterion that is left unfilled.[23–27] Before GBD 2017 we did not distinguish between probable and definite migraine. Since GBD 2017 we accounted for the varying case definitions used by different sources.

Tension-type headache
Tension-type headache (TTH) is characterised by a dull, non-pulsatile, diffuse, band-like (or vice-like) pain of mild to moderate intensity in the head or neck. The reference diagnostic criteria for tension-type headache are from the ICHD-3, which describe five criteria:

1. At least 10 attacks fulfilling criteria 2-5
2. Lasing from 30 minutes to 7 days
3. At least two of the following four characteristics:
    a. Bilateral location
    b. Pressing or tightening (non-pulsating) quality
    c. Mild or moderate intensity
    d. Not aggravated by routine physical activity such as walking or climbing stairs
4. Both of the following:
    a. No nausea or vomiting
    b. No more than one of photophobia or phonophobia
5. Not better accounted for by another ICHD-3 diagnosis

Definite tension-type headache is headache that satisfies all criteria outlined above, while probable tension-type headache satisfies all of the above criteria except one. Before GBD 2017 we did not distinguish between probable and definite tension-type headache. Since GBD 2017 we have accounted for varying case definitions used by different sources.

Medication overuse headache
Both migraine and tension-type headache can give rise to medication overuse headache (MOH), with the following International Classification of Headache Disorders (ICHD-3) diagnostic criteria:

1. Headache occurring ≥15 days/month in a patient with a pre-existing headache disorder
2. Regular overuse for >3 months of one or more drugs that can be taken for acute and/or symptomatic treatment of headache
3. Not better accounted for by another ICHD-3 diagnosis.

ICHD-3 explicitly states that, when a person fulfils criteria for both migraine and MOH, both diagnoses should be given. However, our GBD headache collaborators, Steiner and Stovner, say that in survey practice, a screening question on chronic headache is used first, followed by questions to determine if medication overuse is present. This means the diagnoses of migraine and MOH become mutually exclusive (obviating any potential problem of double-counting).

Input data
Migraine
We last conducted a systematic review of migraine for GBD 2017, which covered papers published through September 2017. The search string for this review was *((((("migraine disorders"[MeSH Terms] OR migraine[All Fields]) AND ((prevalence[Title/Abstract] OR incidence[Title/Abstract] OR remission[Title/Abstract] OR epidemiology[Title/Abstract]))))))).*

Inclusion criteria of the systematic reviews were:

- Representative, population-based surveys
- Reporting of prevalence of migraine headache

In GBD 2017 we decided to exclude medical claims data as the adjustment needed make the claims data comparable to population representative surveys was unstable.

Tension-type headache
We last conducted a systematic review of TTH for GBD 2017, which covered papers published through September 2017. The search string for this review was *((((("headache"[MeSH Terms]) OR ("headache"[Title/Abstract] AND "tension"[Title/Abstract])) AND ("epidemiology"[Title/Abstract] OR "prevalence"[Title/Abstract] OR "incidence"[Title/Abstract] OR "remission"[Title/Abstract])))).*

Inclusion criteria of the systematic reviews were:
- Representative, population-based surveys
- Reporting of prevalence of TTH headache

In GDB 2017 we decided to exclude medical claims data, as the adjustment needed make the claims data comparable to population representative surveys was unstable.

Medication overuse headache
We last conducted a systematic review of MOH for GBD 2017, which covered papers published through September 2017. The search string for this review was *(("headache"[MeSH Terms] OR "headache"[Title/Abstract]) AND ("pharmaceutical preparations"[MeSH Terms] OR "pharmaceutical preparations"[Title/Abstract] OR "medication"[Title/Abstract]) AND ("epidemiology"[Title/Abstract] OR "prevalence"[Title/Abstract] OR "incidence"[Title/Abstract] OR "remission"[Title/Abstract])).*

Inclusion criteria of the systematic reviews were:
- Representative, population-based surveys
- Reporting of prevalence of MOH headache

Age and sex splitting
Reported estimates of prevalence were split by age and sex where possible. First, if studies reported prevalence for broad age groups by sex (eg, prevalence in 15- to 65-year-old males and females separately), and also by specific age groups but for both sexes combined (eg, prevalence in 15- to 30-year-olds, then in 31- to 65-year-olds, for males and females combined), age-specific estimates were split by sex using the reported sex ratio and bounds of uncertainty. Second, prevalence data for both sexes that could not be split using a within-study ratio were split using a sex ratio derived from a meta-analysis of existing sex-specific data using MR-BRT. The female to male ratio was 1·90 (1·85 to 1·96). Finally, after the application of bias adjustments, where studies reported estimates across age groups spanning 25 years or more, these were split into five-year age groups using the prevalence age pattern estimated by the best DisMod-MR 2.1 for each headache type from GBD 2017.

Data adjustment (Bias adjustments)
We used a list of binary adjustment criteria which are a modified version of quality indicators of epidemiological studies on headache[28] and shown in the table below.

Study Covariates

Study covariate	Notation	
	**Less desirable (1)**	**Reference (zero)**
**Other than one-year recall period**	Point prevalence	One-year prevalence
**Not representative**	Selected population	General population or community-based sample from whole country OR general population or community-based sample from defined region within a country, or school-based (for children)

**Low-quality sampling method**	Not stated OR no (or failed) attempt to secure representativeness	Total defined population, or random sample corrected for population demographics OR random sample uncorrected for population demographics
**Poor response**	Not stated, or <70%	70–100%
**Low-quality survey method and type of interviewer**	Not stated OR self-administered (unsupervised) questionnaire OR telephone or face-to-face interview by untrained or unspecified interviewer(s)	Face-to-face interview with headache expert or trained interviewer
**Low-quality validation of diagnostic instrument**	Instrument not specified or not validated OR validated, but sensitivity and/or specificity <70% OR validated only in screen-positive sub-sample, or in clinic or unspecified sample, but sensitivity and specificity ≥70%	Validated in target population or similar, and sensitivity and specificity ≥70%, or all diagnoses made in face-to-face or telephone interviews by headache expert
**Low-quality diagnostic criteria**	Not stated OR stated, other than ICHD OR ICHD (or reasonable modification)	ICHD (or reasonable modification)

We also adjusted data reported in studies that were conducted in a school setting. Studies based on lifetime recall of headaches were not included because of the concern of recall bias. For migraine and tension-type headache, we additionally marked studies where the type of headache (probable/definite) was not explicitly mentioned in the report but was determined based on the diagnostic criteria stated.

The mean and standard error for the coefficients were calculated using the MR-BRT adjustment method. All study covariates were initially evaluated independently for each of the three types of headaches. However, covariate values varied not only in magnitude but in direction across the three headache types. Because we assume that the same study covariate should adjust data at least in the same direction for all headache types, the final study covariates were evaluated taking all migraine, tension-type, and medication overuse headache data into account. Studies conducting in a school setting remained in the models but were no longer adjusted in this round of the GBD, as we were unable to find matches to inform a reliable crosswalk. The school setting covariate should be re-tested in a future round of the GBD in which new data has been added to better inform the adjustment factor. These studies were not excluded because the headache models are relatively data sparse. Betas and inverse-logit values for these covariates are shown in the table below:

MR-BRT Crosswalk Adjustment Factors for Headaches

**Data input**	**Reference or alternative case definition**	**Gamma**	**Beta Coefficient, Logit (95% CI)**	**Adjustment factor***
Other than one-year recall	Alt	1·20	-0·89 (-0·97 to -0·80)	0·30 (0·28 to 0·31)
Not representative	Alt		-0·39 (-0·45 to -0·33)	0·40 (0·39 to 0·42)
Low-quality sampling method	Alt		0·73 (0·66 to 0·79)	0·67 (0·66 to 0·69)
Poor response	Alt		-0·45 (-0·53 to -0·36)	0·40 (0·37 to 0·41)
Low-quality survey method	Alt		-0·22 (-0·31 to -0·13)	0·45 (0·42 to 0·47)
Low-quality diagnostic instrument	Alt		0·15 (0·13 to 0·19)	0·54 (0·53 to 0·55)
Low-quality diagnostic criteria	Alt		-0·37 (-0·43 to -0·32)	0·41 (0·39 to 0·42)
Headache type assumed	Alt		0·37 (0·33 to 0·42)	0·59 (0·58 to 0·60)

**Adjustment factor is the transformed Beta coefficient in normal space, and can be interpreted as the factor by which the alternative case definition is adjusted to reflect what it would have been if measured as the reference.*

Modelling strategy
As in GBD 2017, standard DisMod settings across all headache models include setting excess mortality to 0, and assuming that there was no incidence or prevalence before the age of 5 years.

Migraine
We made no substantive changes in the modelling strategy of migraine from GBD 2017. As in the last round, we ran separate DisMod models for definite migraine, probable migraine, and the total migraine category and set an upper bound on remission of 0.1 across all models. After running the separate models, we then scaled the results of probable and definite headache to the total headache envelope to ensure consistency.

Because some data sources, especially earlier data from before ICHD became the standard (the initial criteria were published in 1988), largely report on definite migraine, we also adjusted studies that reported only on definite migraine to the total migraine category in order to better inform that model. All data that reported on both definite and total migraine were used in regression models by sex in order to derive an age- and sex-specific adjustment. The adjustment is shown in the graphs below.

Male **Female**

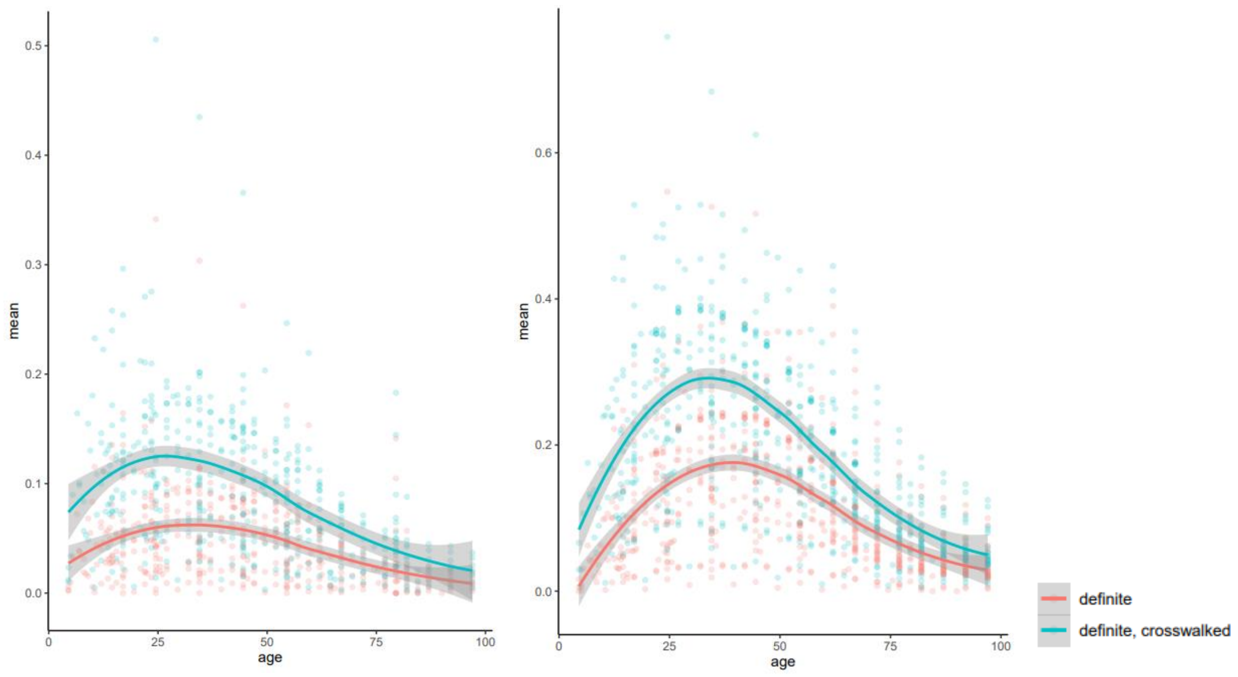

In GBD 2017, to determine the proportion of time over a year spent with migraine headache ("time symptomatic"), we performed a meta-analysis on the frequency and duration of definite headache and total headache combined using the "metafor" package in R. There were not enough data available to obtain reliable estimates on the frequency and duration of probable headache from the literature. As the proportion of time symptomatic for the total migraine category is the weighted average of time symptomatic for definite and probable migraine, weighted by the prevalence of each headache type, the proportion of time symptomatic for probable migraine was calculated as shown below.

$$Time\ Symp_{Probable} = \frac{Time\ Symp_{Total} - Prevalence_{Definite} * Time\ Symp_{Definite}}{Prevalence_{Probable}}$$

For GBD 2019, we used new multi-country survey unit-record data from 19 countries in the Lift the Burden survey series provided by our collaborators on the time symptomatic of various headache types. This source provided greater granularity of time symptomatic data, as we had used summary measures from survey reports instead of microdata in the past. This source also provided data on probable, definite, and total migraine, eliminating the need to back calculate time symptomatic for probable migraine. Using the MR-BRT regression method, we calculated the proportion of time symptomatic is 0·093 for definite migraine and 0·066 for probable migraine.

Tension-type headache
In GBD 2017, we ran a single model for total tension-type headache. For this round of the GBD, we replicated the modelling process for migraine headache and ran separate DisMod models for definite TTH, probable TTH, and the total TTH category, setting an upper bound on remission of 0.5 across all models. After running the separate models, we then scaled the results of probable and definite headache to the total headache envelope to ensure consistency. Because some data sources, especially earlier data from before ICHD became the standard (the initial criteria were published in 1988), largely report on definite TTH, we also adjusted studies that reported only on definite TTH to the total TTH category in order to better inform that model. Initially, all data that reported on both definite and total TTH were used in regression models by sex in order to derive an age- and sex-specific adjustment. These sex-specific models resulted in an implausible age pattern for females such that the age-pattern of the age-split data points was the inverse of the original data. Consequently, we ran a regression model to derive an age-specific adjustment that was applied to both sexes. The adjustment is shown in the graphs below.

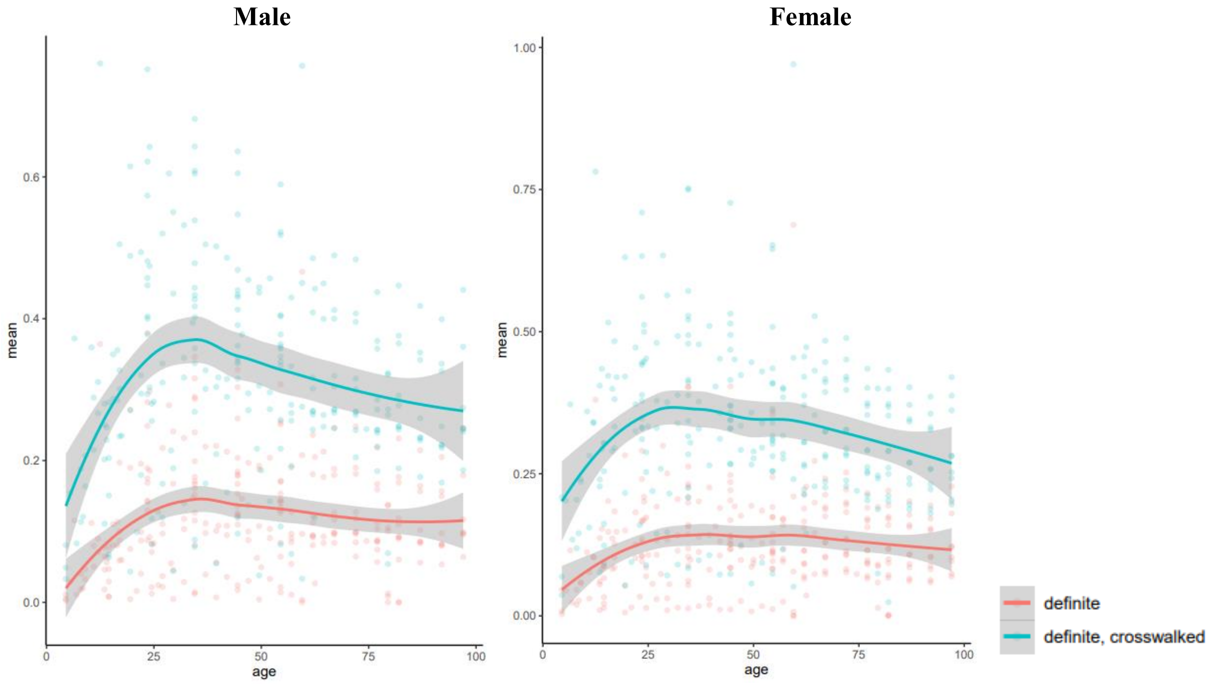

In GBD 2017, a single value derived from a meta-analysis of seven studies on the frequency of the total tension-type headache category was applied to the total TTH model. For GBD 2019 we used the results from the same meta-analysis of Lift the Burden unit-record data on the time symptomatic of headache, which also reported estimates for probable, definite, and total TTH. Using MR-BRT, we calculated the proportion of time symptomatic is 0·029 for definite TTH and 0·021 for probable TTH.

Medication overuse headache
Prior settings in the DisMod model included an upper bound on remission of 0·4. In GDB 2017, to determine the proportion of time over a year spent with medication overuse headache, we meta-analysed the two available studies

on frequency and used the one available study on duration. The result of the meta-analysis on frequency gave an estimate of 250·83 attacks per year, and the available source on duration estimated an average duration of 18·59 hours. From this data we estimated that the proportion of time symptomatic for medication overuse headache was 0·532. We made no substantive changes in the modelling strategy from GBD 2017.

Medication overuse headache split

As medication overuse headache can develop from migraine or tension-type headache, we split medication overuse into sequelae of both primary headache disorders. Based on a 2017 meta-analysis of three sources, 73·2% (63·7–81·0) of medication-overuse headache is assigned to medication overuse headache due to tension-type headache. The forest plot is shown below.

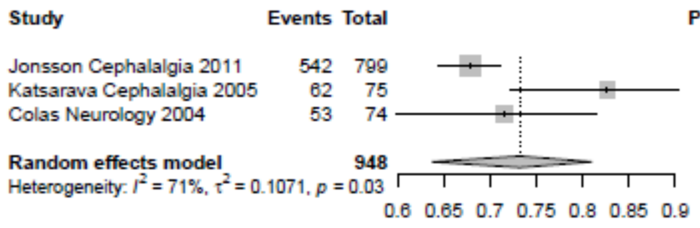

4.10. Motor Neuron Disease (fatal modelling)

Flowchart

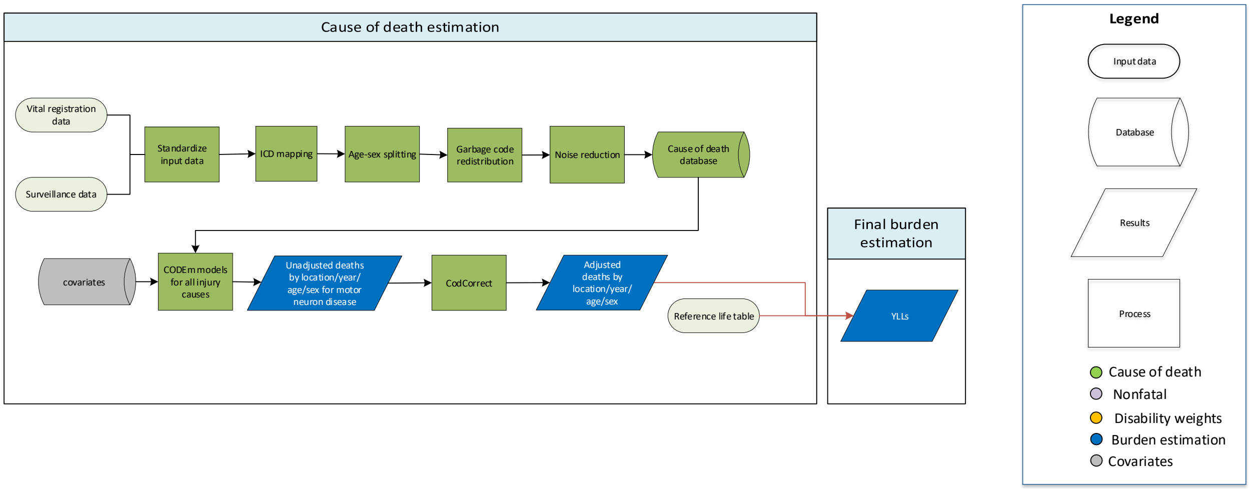

Input Data and Methodological Summary for Motor Neuron Disease

Input data

Data used to estimate Motor Neuron Disease included vital registration and surveillance data from the cause of death (COD) database. Our outlier criteria were to exclude data points that (1) were implausibly high or low, (2) substantially conflicted with established age or temporal patterns, or (3) substantially conflicted with other data sources from the same locations or locations with similar characteristics (ie, Socio-demographic Index). In GBD 2019, this affected Kazakhstan where ICD9-BTL tabulated vital registration data were available for 1991-2003 and ICD10-coded vital registration were available for 2013 onwards. The raw ICD9-BTL data for 1991 were 14-fold higher than raw ICD9-BTL (1992-2003) and ICD-10 (2013 onwards) causing an implausible time pattern via noise reduction data processing methods for ICD9-BTL data. For that reason, the ICD9-BTL data were excluded and the ICD-10 data retained.

Modelling strategy

The standard CODEm modelling approach (described appendix section 3.1) was used to estimate deaths due to multiple sclerosis. Separate models were conducted for male and female mortality, and the age range for both models was 0-days to 95+ years. Unadjusted death estimates were adjusted using CoDCorrect to produce final estimates of YLLs.

Table 1. Covariates used in Motor Neuron Disease mortality modelling

Level	Covariate	Direction
	Mean total body mass index (kg/m^2)	-
	Mean serum total cholesterol (mmol/L)	-
	Absolute value of average latitude	+
	Mean diabetes fasting plasma glucose (mmol/L)	+
	Fruit consumption (grams per day adjusted)	-
	Socio-demographic Index	+
	Health care access and quality index	-
2	Population-weighted mean temperature	-
	Sanitation (proportion with access)	+
	Improved water source (proportion with access)	-
3	Education (years per capita)	+
	Log-transformed LDI (per capita)	+

"The following plots show the influence of each covariate on the four CODEm models (male global, male data rich, female global, and female data rich). A positive standardized beta (to the right) means that the covariate was associated with increased death. A negative standardized beta (to the left) means the covariate was associated with decreased death.

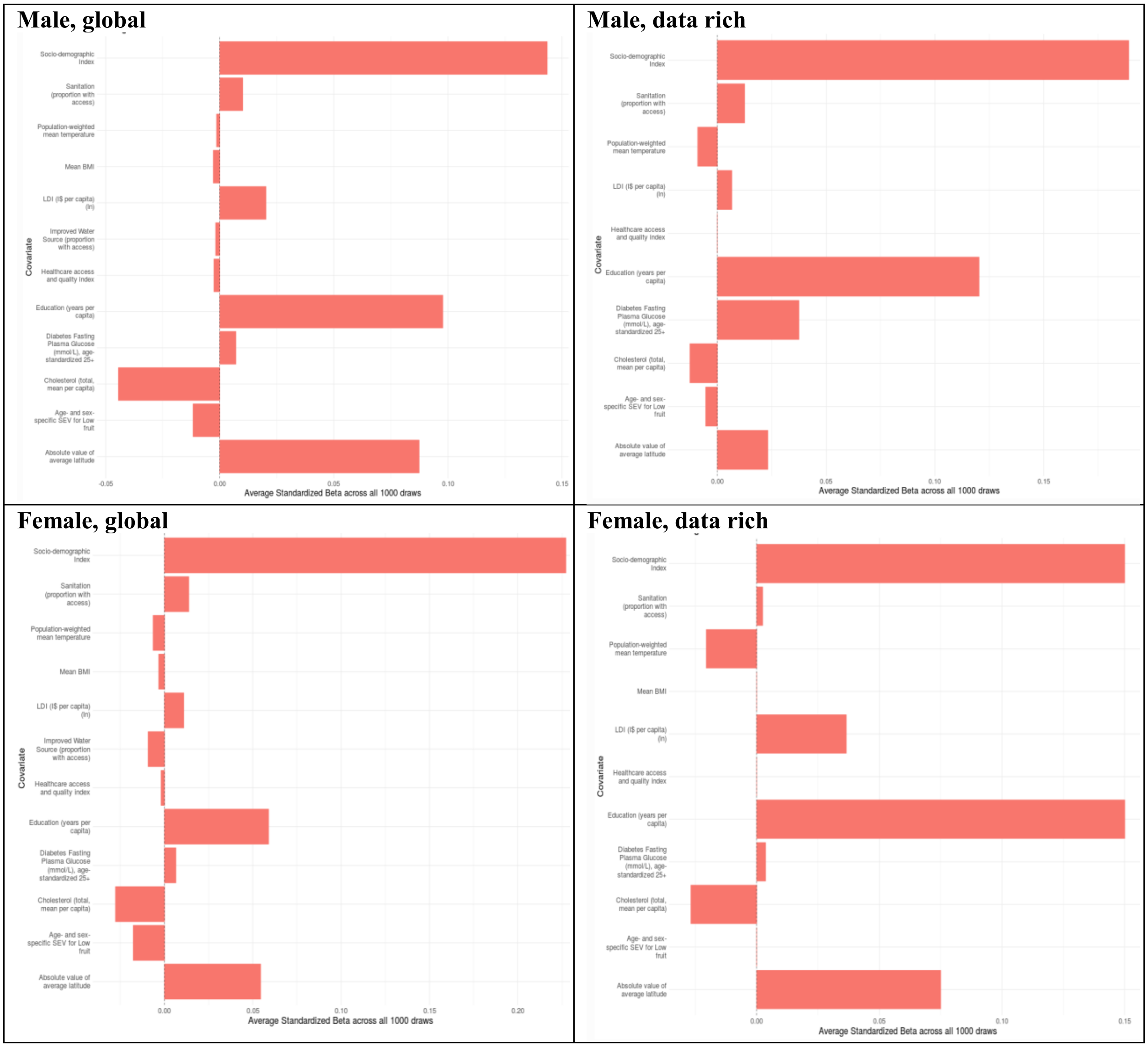

4.11. Motor neuron diseases (non-fatal modelling)

Flowchart

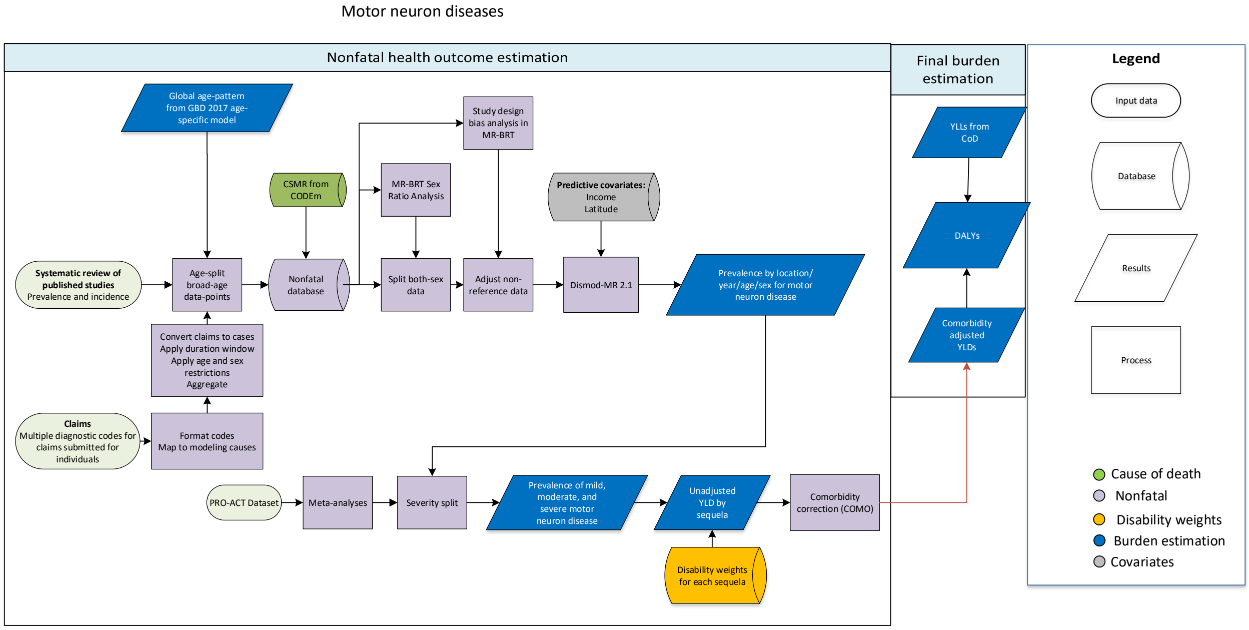

Case definition

Motor neuron diseases (MND) are a set of chronic, degenerative, and progressive neurological conditions typified by the destruction of motor neurons and the subsequent deterioration of voluntary muscle activity. The most common MND is amyotrophic lateral sclerosis (ALS). The El Escorial Criteria are the gold standard diagnostic criteria. The ICD-10 code corresponding to motor neuron diseases is G12.

Input data and data processing

A full systematic review was last conducted for GBD 2015 and will be updated in a future round of GBD. Selection criteria included: (1) the study is a representative population-based study with well-defined sample; and (2) reports on prevalence, incidence, remission, excess mortality, relative risk of mortality, standardised mortality ratio, or with-condition mortality rate for motor neuron diseases in aggregate or a specified motor neuron disease.

(('motor neuron disease'[MeSH Terms] OR ('motor'[All Fields] AND 'neuron'[All Fields] AND 'disease'[All Fields]) OR 'motor neuron disease'[All Fields] OR ('motor'[All Fields] AND 'neuron'[All Fields] AND 'diseases'[All Fields]) OR 'motor neuron diseases'[All Fields]) OR ('amyotrophic lateral sclerosis'[MeSH Terms] OR ('amyotrophic'[All Fields] AND 'lateral'[All Fields] AND 'sclerosis'[All Fields]) OR 'amyotrophic lateral sclerosis'[All Fields]) OR ALS[All Fields] OR ('motor neuron disease'[MeSH Terms] OR ('motor'[All Fields] AND 'neuron'[All Fields] AND 'disease'[All Fields]) OR 'motor neuron disease'[All Fields] OR ('primary'[All Fields] AND 'lateral'[All Fields] AND 'sclerosis'[All Fields]) OR 'primary lateral sclerosis'[All Fields]) OR ('Politics Life Sci'[Journal] OR 'pls'[All Fields]) OR ('muscular atrophy, spinal'[MeSH Terms] OR ('muscular'[All Fields] AND 'atrophy'[All Fields] AND 'spinal'[All Fields]) OR 'spinal muscular atrophy'[All Fields] OR ('progressive'[All Fields] AND 'muscular'[All Fields] AND 'atrophy'[All Fields]) OR 'progressive muscular atrophy'[All Fields]) OR PBP[All Fields] OR ('pseudobulbar palsy'[MeSH Terms] OR ('pseudobulbar'[All Fields] AND 'palsy'[All Fields]) OR 'pseudobulbar palsy'[All Fields])) AND (('epidemiology'[Subheading] OR 'epidemiology'[All Fields] OR 'epidemiology'[MeSH Terms]) OR population-based[All Fields])

Data from the systematic review were manually extracted for GBD 2015. For GBD 2017, data-points referring to broad age-groups were split according to the age-pattern estimated for that datum's location in a preliminary model

that used only age-specific data. For GBD 2019, all previously extracted studies were reviewed and assigned a design variable to indicate if the case definition was limited to ALS only or encompassed all MND.

Beyond data from the systematic review, as in previous rounds of GBD, we made use of claims data as obtained and processed by the GBD Clinical Informatics team and described in a separate section of this Appendix. These data link claims for all inpatient and outpatient encounters for a single individual, and provide primary and secondary diagnoses for all encounters. An individual was extracted from claims data as a prevalent case if they had any MND code as any diagnosis in one or more inpatient encounters or two or more outpatient encounters.

In GBD 2019, all sex-specific data were used to estimate a pooled sex-ratio using MR-BRT. This ratio was combined with sex-specific population estimates for the year-age-location combinations corresponding to each data point reported for both sexes combined, to estimate sex-specific data-points prior to modelling. These were applied by calculating male prevalence:

$$prev_{male} = prev_{both} * \frac{pop_{both}}{(pop_{male} + ratio * pop_{female})}$$

and then calculating female prevalence:

$$prev_{female} = ratio * prev_{male}$$

(Or the equivalent equations for incidence or other epidemiologic measure.)

Two pre-modelling adjustments were then made adjust for systematic biases in some data sources: data reporting on ALS only and data from USA claims in the year 2000 (a database that only covers a small commercially insured sub-population). Two studies of ALS only were found to be closely matched in year, age, sex and time with three studies of MND more broadly, and the log-ratios for all matched pairs were entered into an MR-BRT meta-analysis. Commercial claims data from the USA in 2000 were matched to USA claims data from later years with more complete coverage of the population, and these log-ratios were entered into a separate MR-BRT model.

MR-BRT Crosswalk Adjustment Factors

Data input	Reference or alternative case definition	Beta Coefficient, Log (95% CI)	Adjustment factor*
Surveys of all MND using combined clinical, imaging, electrophysiology and imaging criteria<br>OR<br>Claims data from location-years other than USA 2000	Ref	---	---
USA claims from year 2000	Alt	-0·026 (-1·2 to 1·1)	0·97 (0·31 to 3·1)
Surveys limited to ALS only	Alt	-0·13 (-0·23 to -0·029)	0·88 (0·79 to 0·97)
*Adjustment factor is the transformed Beta coefficient in normal space, and can be interpreted as the factor by which the alternative case definition is adjusted to reflect what it would have been if measured as the reference.			

After extraction and processing, some studies were marked as outliers and excluded on a case-by-case basis if they were inconsistent with established regional or temporal trends or if concerns about study quality were identified during extraction and processing.

Modelling strategy

We use DisMod 2.1 as the main analytical tool for MND estimation. Inputs included prevalence and incidence data, as described above, as well as the cause-specific mortality rate (CSMR) estimated in the GBD causes of death analysis, and excess mortality rate (EMR) obtained by dividing CSMR by prevalence data-points. Prior settings are limited to 0 remission at all ages and maximum incidence of 0·0004. We also constrain the super-region random effects for prevalence and incidence to -0·5 and 0·5 to account for spurious inflation of regional differences.

We employed the following covariates to improve model predictions:

Covariate	Measure	Beta coeff (95% CI)	Exponentiated
Absolute value of average latitude	Prevalence	0·032 (0·031 to 0·033)	1·03 (1·03 to 1·03)
LDI (I$ per capita)	Excess mortality rate	-0·5 (-0·5 to -0·5)	0·61 (0·61 to 0·61)

Although there are no known cures for MND, we expect disease management to differ globally – largely as a function of available resources. To capture this, we use the natural log of lagged distributed income per capita as a proxy to capture this relationship in the estimation of excess mortality.

As described in the literature, extreme latitude may be associated with higher prevalence and incidence of motor neuron disease, although the pathway to explain the association is not understood. Our operationalisation of latitude is created by a population-weighted average of latitude by country and taking the absolute value. The underlying population distribution rasters are part of the Gridded Population of the World dataset.

Severity splits

To calculate severity and disability due to MND we analysed a dataset from Pooled Resource Open-access ALS Clinical Trials (PRO-ACT). The ALS Function Rating Score (ALSFRS) is an instrument for evaluating the functional status of patients with amyotrophic lateral sclerosis. It can be used to monitor functional changes in a patient over time. It measures (1) speech, (2) salivation, (3) swallowing, (4) handwriting, (5) cutting food and handling utensils (with or without gastrostomy), (6) dressing and hygiene, (7) turning in bed and adjusting bed clothes, (8) walking, (9) climbing stairs, and (10) breathing. Each task is rated on a 5-point scale from 0 = can't do, to 4 = normal ability. Individual item scores are summed to produce a reported total score of between 0 and 40 (worst to best). ALSFRS has been revised to ALSFRS-R, which includes 12 questions (ALSFRS Q10 changes to (10) Dyspnea, (11) Orthopnea, and (12) Respiratory insufficiency), with individual item scores summed to a score between 0 and 48.

In order to eliminate any bias from the treatment effects on the ALSFRS, only the first observation at the time of trial is selected. If the first observation is missing at the time of trial (or prior), the next non-missing observation is selected to be included in the final analysis.

We subsequently mapped ALSFRS scores into GBD severities, and sequelae into different combinations of speech problems, chronic obstructive pulmonary disease, and motor impairment using the following logic:

Motor impairment

The ALSFRS assess motor function of the legs through questions on walking (Q8) and stair climbing (Q9).

Combined score	Severity level
8	None
5-7	Mild
2-4	Moderate
0-1	Severe

The ALSFRS also assesses motor impairment through questions on handwriting (Q4), cutting food and handling utensils (Q5), and dressing and hygiene (Q6).

Combined score	Severity level
12	None
9-11	Mild
3-8	Moderate
0-2	Severe

After determining case severity on these two separate metrics, we aggregate by taking the most severe ranking (eg, severe + mild = a severe case).

Respiratory problems:
Question 10 of the ALSFRS describes breathing difficulty as a function of MND.

ALSFRS score	Description	Severity level
4	Normal	None
3	Shortness of breath with minimal exertion	Mild
2	Shortness of breath at rest	Moderate
0-1	Intermittent ventilator assistance required/ventilator-dependent	Severe

Speech problems
Speech impairment due to MND is derived from ALSFRS question 1, which describes speech impediments. A score of 4 on this question denotes no impairment, while all other values suggest some impairment.

Creating sequelae
After determining the severity status of each case for the three symptom umbrellas, we subsequently estimated the relative proportion of each combination of symptom class and their respective severities. Those without any symptoms (eg, no severity) were categorised as having worry about the diagnosis for disability estimation. The following table displays the various sequelae and their associated proportions.

Sequela	Proportion (Mean)	Proportion (Lower)	Proportion (Upper)
Mild motor impairment, mild respiratory problems and speech problems due to motor neuron disease	0·01779	0·01658	0·01909
Mild motor impairment, moderate respiratory problems and speech problems due to motor neuron disease	0·00270	0·00225	0·00324
Mild motor impairment, severe respiratory problems and speech problems due to motor neuron disease	0·00082	0·00059	0·00113
Mild motor impairment, and speech problems due to motor neuron disease	0·02052	0·01922	0·02190
Moderate motor impairment, mild respiratory problems and speech problems due to motor neuron disease	0·03377	0·03210	0·03552
Moderate motor impairment, moderate respiratory problems and speech problems due to motor neuron disease	0·00715	0·00640	0·00799
Moderate motor impairment, severe respiratory problems and speech problems due to motor neuron disease	0·00286	0·00240	0·00342
Moderate motor impairment, and speech problems due to motor neuron disease	0·03041	0·02883	0·03208
Severe motor impairment, mild respiratory problems and speech problems due to motor neuron disease	0·05242	0·05035	0·05457
Severe motor impairment, moderate respiratory problems and speech problems due to motor neuron disease	0·02247	0·02111	0·02392
Severe motor impairment, severe respiratory problems and speech problems due to motor neuron disease	0·01365	0·01259	0·01479
Severe motor impairment and speech problems due to motor neuron disease	0·04765	0·04567	0·04970
Mild respiratory problems and speech problems due to motor neuron disease	0·01157	0·01060	0·01263

Sequela	Proportion (Mean)	Proportion (Lower)	Proportion (Upper)
Moderate respiratory problems and speech problems due to motor neuron disease	0·00142	0·00111	0·00182
Severe respiratory problems and speech problems due to motor neuron disease	0·00023	0·00013	0·00043
Speech problems due to motor neuron disease	0·02457	0·02315	0·02608
Mild motor impairment and mild respiratory problems due to motor neuron disease	0·02245	0·02109	0·02389
Mild motor impairment and moderate respiratory problems due to motor neuron disease	0·00275	0·00230	0·00329
Mild motor impairment and severe respiratory problems due to motor neuron disease	0·00068	0·00047	0·00097
Mild motor impairment due to motor neuron disease	0·10388	0·10103	0·10681
Moderate motor impairment and mild respiratory problems due to motor neuron disease	0·06744	0·06511	0·06985
Moderate motor impairment and moderate respiratory problems due to motor neuron disease	0·01302	0·01199	0·01413
Moderate motor impairment and severe respiratory problems due to motor neuron disease	0·00412	0·00356	0·00477
Moderate motor impairment due to motor neuron disease	0·20136	0·19760	0·20518
Severe motor impairment and mild respiratory problems due to motor neuron disease	0·06902	0·06666	0·07146
Severe motor impairment and moderate respiratory problems due to motor neuron disease	0·02000	0·01872	0·02137
Severe motor impairment and severe respiratory problems due to motor neuron disease	0·01062	0·00969	0·01163
Severe motor impairment due to motor neuron disease	0·15037	0·14702	0·15378
Mild respiratory problems due to motor neuron disease	0·00643	0·00571	0·00723
Moderate respiratory problems due to motor neuron disease	0·00044	0·00028	0·00069
Severe respiratory problems due to motor neuron disease	0·00005	0·00001	0·00017
Asymptomatic, but worry about diagnosis due to motor neuron disease	0·03738	0·03562	0·03921

To determine disability due to these sequelae, we use the standard multiplicative aggregation formula as described in the main text. The following table provides description and disability weight assigned to the sequelae as appropriate.

Symptom group	Severity level	Lay description	DW (95%)
Respiratory problems	Asymptomatic		
Respiratory problems	Mild	Has cough and shortness of breath after heavy physical activity, but is able to walk long distances and climb stairs.	0·019 (0·011–0·033)
Respiratory problems	Moderate	Has cough, wheezing, and shortness of breath, even after light physical activity. The person feels tired and can walk only short distances or climb only a few stairs.	0·225 (0·153–0·31)
Respiratory problems	Severe	Has cough, wheezing, and shortness of breath all the time. The person has great difficulty walking even short distances or climbing any stairs, feels tired when at rest, and is anxious.	0·408 (0·273–0·556)
Motor impairment	Asymptomatic		

**Symptom group**	**Severity level**	**Lay description**	**DW (95%)**
Motor impairment	Mild	Has some difficulty in moving around but is able to walk without help.	0·01 (0·005–0·019)
Motor impairment	Moderate	Has some difficulty in moving around and difficulty in lifting and holding objects, dressing, and sitting upright, but is able to walk without help.	0·061 (0·04–0·089)
Motor impairment	Severe	Is unable to move around without help, and is not able to lift or hold objects, get dressed, or sit upright.	0·402 (0·268–0·545)
Speech problems	No		
Speech problems	Yes	Has difficulty speaking, and others find it difficult to understand.	0·051 (0·032–0·078)
Asymptomatic, but worry	Yes	Has a disease diagnosis that causes some worry but minimal interference with daily activities.	0·012 (0·006–0·023)

4.12. Other Neurological Disorders (fatal modelling)

Flowchart

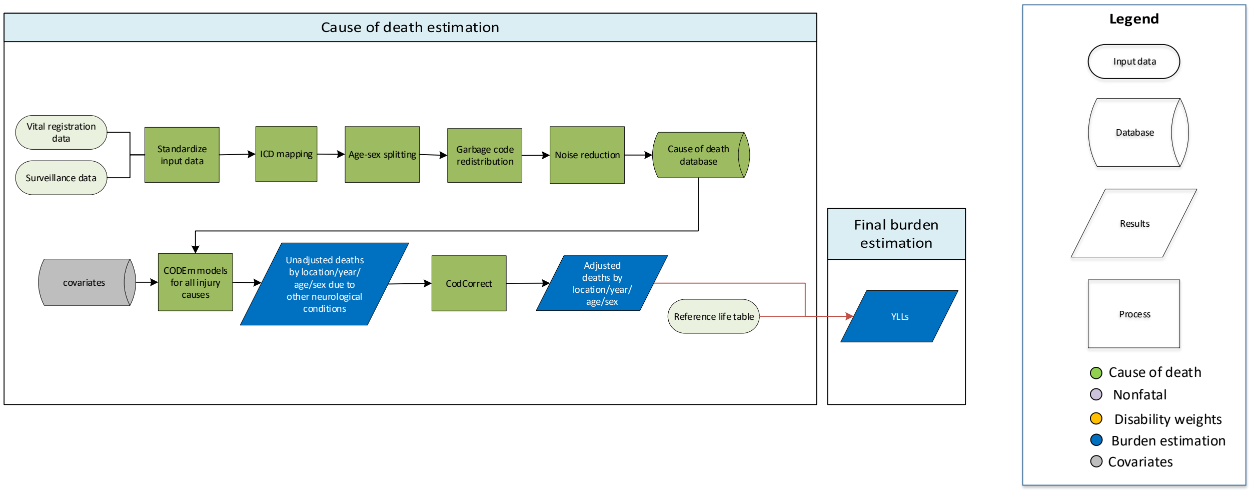

Input Data and Methodological Summary for Other Neurological Disorders

Input data

Data used to estimate other neurological disorders included vital registration and surveillance data from the cause of death (COD) database. Our outlier criteria were to exclude data points that (1) were implausibly high or low, (2) substantially conflicted with established age or temporal patterns, or (3) significantly conflicted with other data sources conducted from the same locations or locations with similar characteristics. Data excluded as outliers in GBD 2017 continued to be excluded in GBD 2019

Modelling strategy

The standard CODEm modelling approach (as described in appendix section 3.1) was used to estimate deaths due to multiple sclerosis. Separate models were conducted for male and female mortality, and the age range for both models was 28-days to 95+ years. Changes from GBD 2017 and the full list of covariates used in GBD 2019 are displayed below. Unadjusted death estimates were adjusted using CoD Correct to produce final estimates of YLLs.

Covariates used in Other Neurological Disorders mortality modelling

Level	Covariate	Direction
1	Mean total body mass index	+
	Mean serum total cholesterol (mmol/L)	+
	Mean systolic blood pressure (mm/Hg)	+
	Pigs per capita	+
	Underweight proportion under 2 standard deviations	+
	Red meat consumption adjusted	+
2	Population density over 1000 per square kilometer pct	+
	Health care access and quality index	-
	Fruit consumption (grams per day adjusted)	-
3	Cumulative cigarettes (10 years)	+
	Cumulative cigarettes (5 years)	+
	Education (years per capita)	-
	Log-transformed LDI (per capita)	-
	Smoking prevalence	+
	Socio-demographic Index	+

The following plots show the influence of each covariate on the four CODEm models (male global, male data rich, female global, and female data rich). A positive standardized beta (to the right) means that the covariate was associated with increased death. A negative standardized beta (to the left) means the covariate was associated with decreased death.

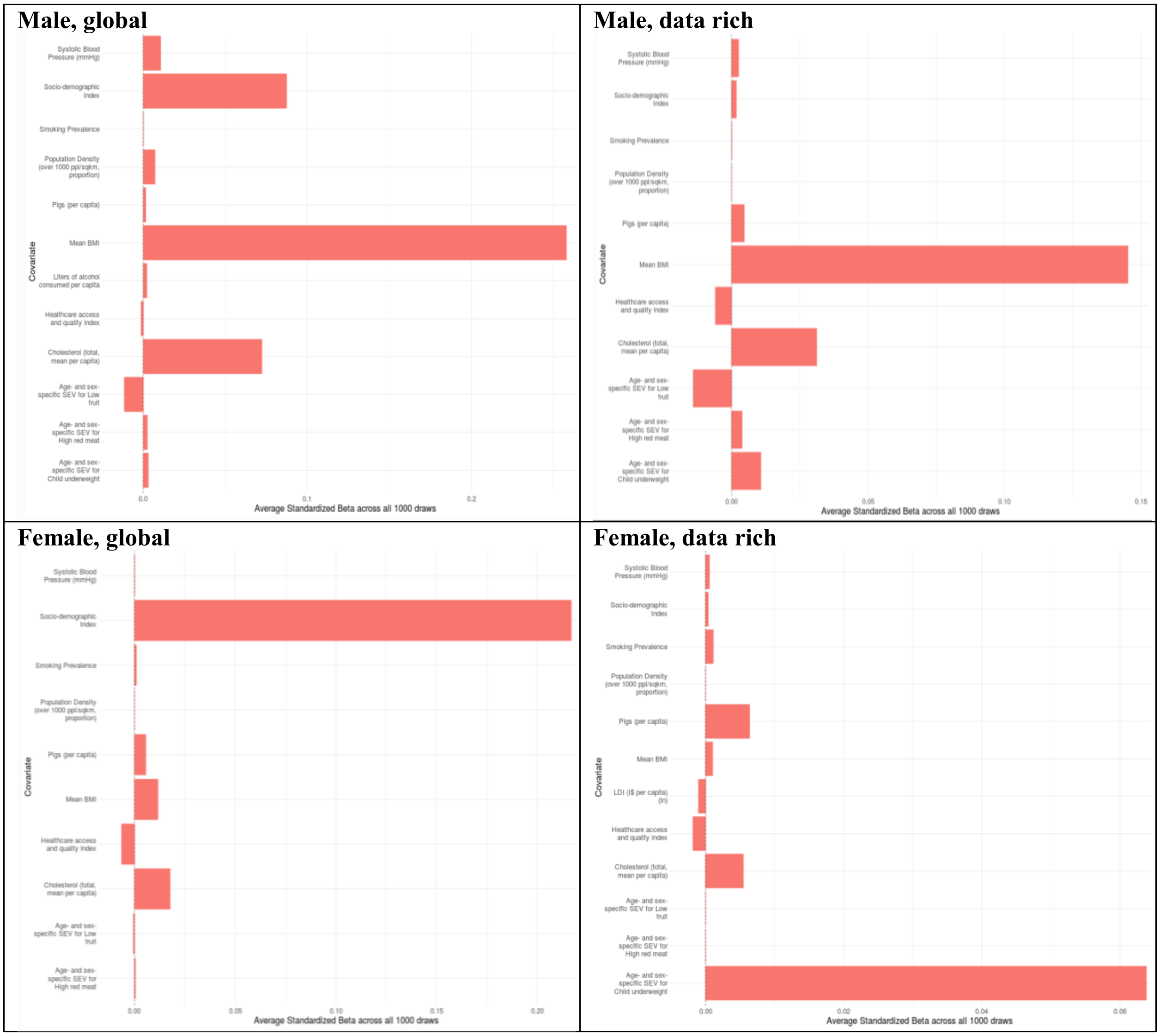

4.13. Other neurological disorders (non-fatal modelling)

In addition to the neurological disorders described above, there are many diverse types of neurological disorders with a range of severities and associated sequelae. Because these neurological disorders are diverse in their underlying causes and risk factors as well as in their associated health outcomes, modelling them together in a DisMod-MR model would not produce reliable estimates of prevalence or excess mortality. Instead, we calculated the YLDs caused by neurological disorders directly using a YLD/YLL ratio.

We calculated the ratio of YLDs to YLLs across the specified neurological disorders for which non-fatal outcomes were modelled, using YLL estimates from the GBD 2019 cause of death (CoD) analysis. We then multiplied this YLD/YLL ratio by the YLL estimates for other neurological disorders from the GBD 2019 CoD analysis, providing us with an estimate of the YLDs associated with other neurological disorders.

4.14. Stroke (fatal modelling)

4.14.1. Ischaemic stroke

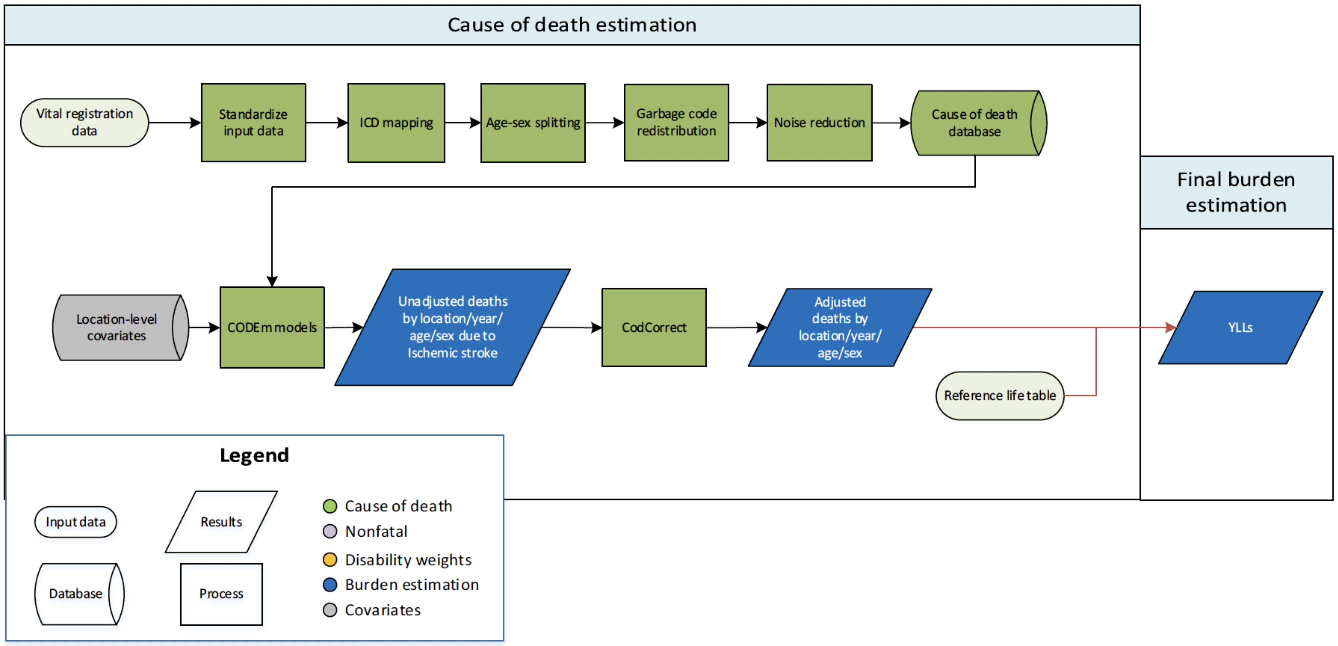

Input data
Vital registration data were used to model deaths from ischaemic stroke. We outliered ICD8 data points which were inconsistent with the rest of the data and created implausible time trends. We also outliered ICD10 data points in The Republic of Tajikistan due to unstable and implausible estimates in similar age groups.

Modelling strategy
We used a standard CODEm approach to model deaths from ischemic stroke. For GBD 2019, adjusted dietary covariates for consumption of fruits, omega-3 fatty acids, vegetables, nuts and seeds, and polyunsaturated fatty acids were replaced with the summary exposure value scalars for diet low in each of these factors. The direction for each dietary covariate was changed from -1 to 1 to as our *a priori* assumption is that low levels of intake of these dietary factors are associated with increasing mortality risk from ischaemic stroke. In addition, the dietary covariate for whole grains (kcal/capita, adjusted) and the socio-demographic index covariate were dropped as exploratory analyses indicated that the covariates were not predictive of the outcome. In addition, we changed the direction of the alcohol variable from 0 to 1 to reflect our *a priori* hypothesis about the expected direction of the association between this risk factor and mortality risk of ischaemic stroke. We also changed the level of the trans fatty acid covariate from 1 to 3. Besides these covariate changes, there are no other substantive changes from the approach used in GBD 2017.

Selected covariates for CODEm models, ischaemic stroke

Covariate	Transformation	Level	Direction
Summary exposure value, ischaemic stroke	None	1	1
Cholesterol (total, mean per capita)	None	1	1
Smoking prevalence	None	1	1

Systolic blood pressure (mmHg)	None	1	1
Mean BMI	None	2	1
Elevation over 1500m (proportion)	None	2	-1
Fasting plasma glucose	None	2	1
Outdoor pollution ($PM_{2.5}$)	None	2	1
Indoor air pollution	None	2	1
Healthcare access and quality index	None	2	-1
Lag distributed income per capita (I$)	Log	3	-1
Summary exposure value, omega-3	None	3	1
Summary exposure value, fruits	None	3	1
Summary exposure value, vegetables	None	3	1
Summary exposure value, nuts and seeds	None	3	1
Pulses/legumes (kcal/capita, unadjusted)	None	3	-1
Summary exposure value PUFA adjusted	None	3	1
Alcohol (litres per capita)	None	3	1
Trans fatty acid	None	3	1

4.14.2. Intracerebral haemorrhage

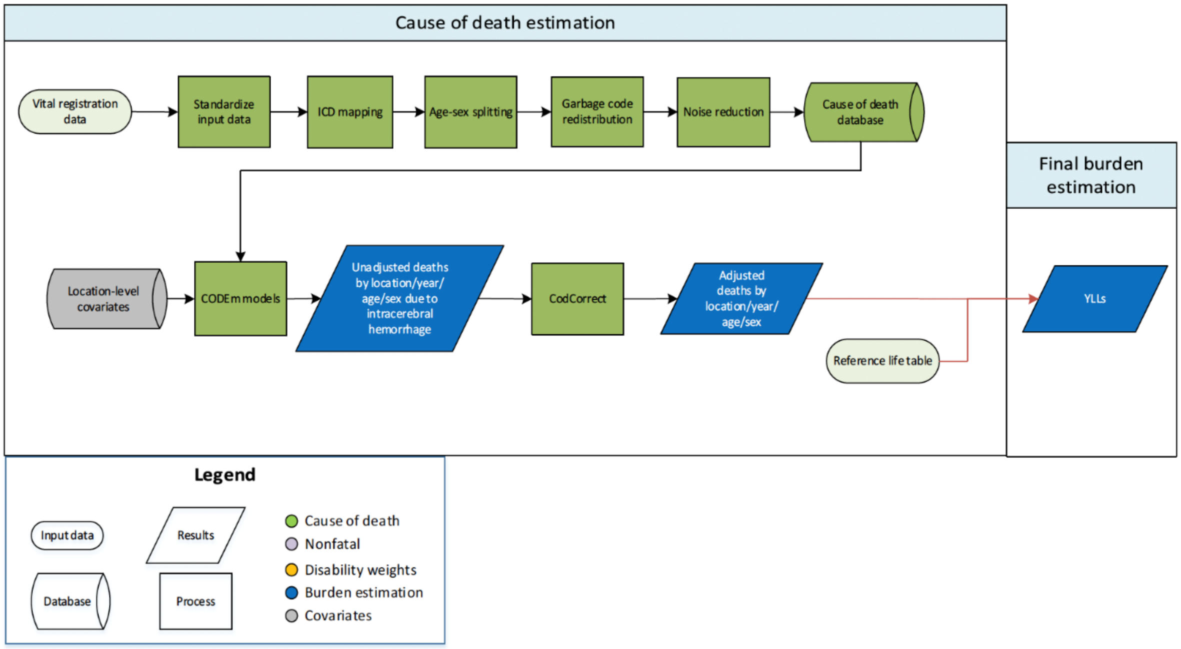

Input data

Vital registration data were used to model intracerebral haemorrhage. We outliered ICD8 data points which were inconsistent with the rest of the data and created implausible time trends. In addition, we outliered vital registration data points in certain countries in Latin American countries due to implausibly high values at the oldest age groups resulting in inconsistencies in time trends.

Modelling strategy
We used a standard CODEm approach to model deaths from intracerebral haemorrhage. For GBD 2019, adjusted dietary covariates for consumption of fruits, omega-3 fatty acids, vegetables, nuts and seeds, and polyunsaturated fatty acids were replaced with the summary exposure value scalars for diet low in each of these factors. The direction for each dietary covariate was changed from -1 to 1 to as our *a priori* assumption is that low levels of intake of these dietary factors are associated with increasing mortality risk from intracerebral haemorrhage. In addition, the dietary covariate for whole grains (kcal/capita, adjusted) and the social demographic index covariate were dropped as exploratory analyses indicated that these covariates were not predictive of the mortality risk from intracerebral haemorrhage. We changed the direction of the covariate for alcohol from 0 to 1 due to our *a priori* hypothesis about the direction of the association for this covariate. We also changed the level of the cholesterol covariate from 1 to 3 and the direction from 0 to -1 to reflect the mixed and inconclusive evidence regarding cholesterol levels and risk of intracerebral haemorrhage. In addition, we changed the level of the trans fatty acid from covariate from 1 to 3 in accordance with the expected importance of this risk factor on mortality from intracerebral haemorrhage. Besides these covariate changes, there are no other substantive changes from the approach used in GBD 2017.

4.14.3. Subarachnoid haemorrhage

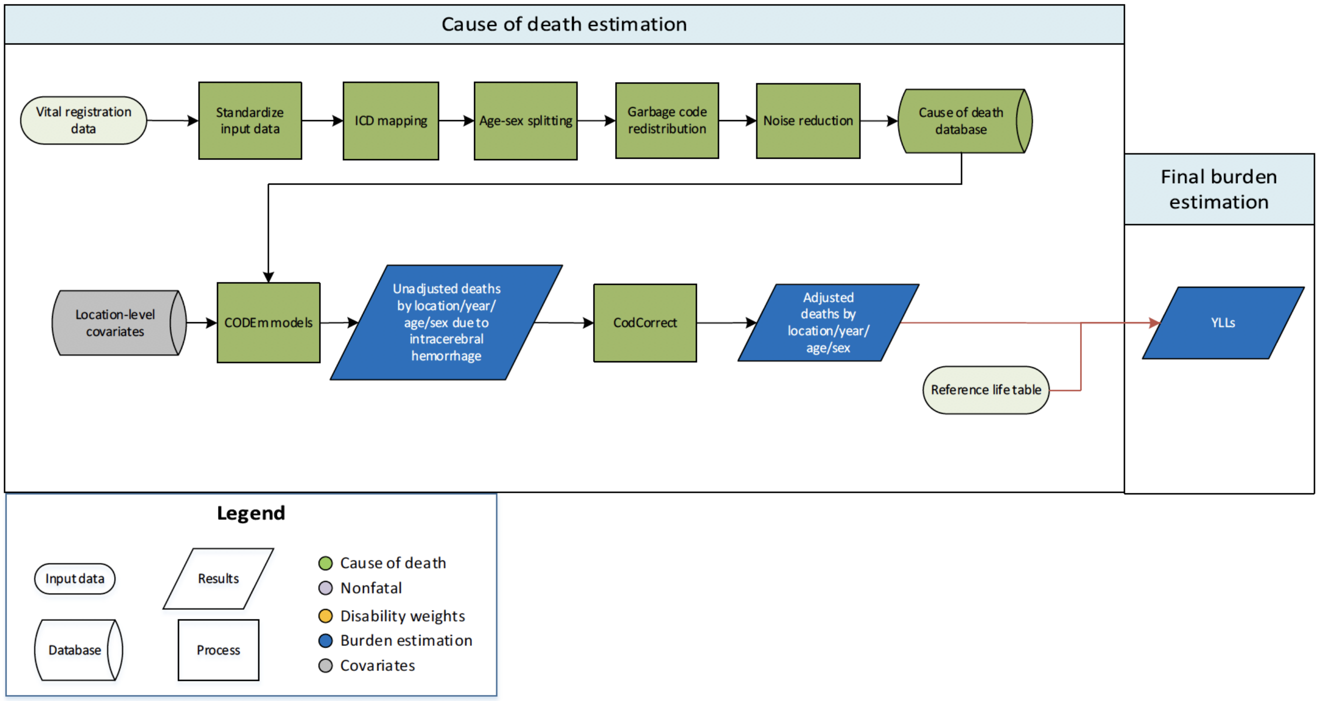

Input data
Vital registration data were used to model subarachnoid haemorrhage. We outliered ICD8 datapoints which were inconsistent with the rest of the data and created implausible time trends. In addition, we outliered vital registration data in Tibet that was implausibly high for all years and age groups.

Modelling strategy
We used a standard CODEm approach to model deaths from subarachnoid haemorrhage. The covariates chosen for inclusion in the ensemble modelling process are listed in the table below. For GBD 2019, we dropped the Socio-demographic Index covariate as exploratory analyses indicated that it was not predictive of the outcome. We also changed the direction of the alcohol covariate from 0 to 1 to reflect the expected direction of the association of this

risk factor with mortality risk. Apart from these changes to the covariates, there are no substantive changes from the approach used in GBD 2017.

Selected covariates for CODEm models, subarachnoid haemorrhage

Level	Covariate	Transformation	Direction
1	Smoking prevalence	None	1
1	Systolic blood pressure (mmHg)	None	1
2	Healthcare access and quality index	None	-1
3	Lag distributed income per capita (I$)	Log	-1
3	Alcohol (litres per capita)	None	1

4.14.4. Selected covariates for CODEm models, overall stroke and subtypes

CODEm is an analytical tool which explores a large variety of possible models to estimates trends in causes of death. This tool explores a large variety of possible models to estimate trends in causes of death. Possible models are identified using a covariate selection algorithm that yields many plausible combinations of covariates, which are then run through four model classes. The model classes include mixed effects linear models and spatial-temporal Gaussian Process Regression models for cause fractions and death rates. All models for each cause of death are then assessed using out-of-sample predictive validity and combined into an ensemble with optimal out-of-sample predictive performance. Ensemble models for cause of death estimation outperform any single component model in tests of root mean square error, frequency of predicting correct temporal trends, and achieving 95% coverage of the prediction interval.[9]

**Covariate**	**Level**	**Direction, Stroke**	**Direction, Ischaemic stroke**	**Direction, Haemorrhagic stroke**
Summary exposure variable	1	+	+	+
Cholesterol (total, mean per capita)	1	+	+	0
Smoking prevalence	1	+	+	+
Systolic blood pressure (mmHg)	1	+	+	+
Trans fatty acid	1	+	+	+
Mean BMI	2	+	+	+
Elevation over 1500m (proportion)	2	-	-	-
Fasting plasma glucose	2	+	+	+
Outdoor pollution ($PM_{2.5}$)	2	+	+	+
Indoor air pollution	2	+	+	+
Healthcare access and quality index	2	-	-	-
Lag distributed income per capita (I$)*	3	-	-	-
Socio-demographic Index	3	0	0	0
Omega-3 (kcal/capita, adjusted) *	3	-	-	-
Fruits (kcal/capita, adjusted)	3	-	-	-
Vegetables (kcal/capita, adjusted)	3	-	-	-
Nuts and seeds (kcal/capita, adjusted)	3	-	-	-
Whole grains (kcal/capita, adjusted)	3	-	-	-
Pulses/legumes (kcal/capita, adjusted)	3	-	-	-
PUFA adjusted (percent)	3	-	-	-

Covariate	Level	Direction, Stroke	Direction, Ischaemic stroke	Direction, Haemorrhagic stroke
Alcohol (litres per capita)	3	0	0	0

*Variables were log-transformed

DisMod covariates

Step 1:

Cause	Variable name	Measure	beta	Exponentiated beta
Chronic ischaemic stroke	Log-transformed SEV scalar: Isch Stroke	Prevalence	0·83 (0·75 – 1·03)	2·29 (2·12 – 2·80)
Chronic ischaemic stroke	LDI (I$ per capita)	Excess mortality rate	-0·16 (-0·29 – -0·1)	0·85 (0·75 – 0·90)
Chronic haemorrhagic stroke	Log-transformed SEV scalar: Hem Stroke	Prevalence	0·79 (0·75 – 0·92)	2·21 (2·12 – 2·50)
Chronic haemorrhagic stroke	LDI (I$ per capita)	Excess mortality rate	-0·12 (-0·16 – -0·1)	0·89 (0·85 – 0·90)
First ever acute haemorrhagic stroke	Hospital data	Incidence	0·54 (0·54 – 0·54)	1·71 (1·71 – 1·72)
First ever acute haemorrhagic stroke	Any stroke	Incidence	1·27 (1·27 – 1·28)	3·57 (3·56 – 3·59)
First ever acute haemorrhagic stroke	First-ever acute stroke, ischaemic or haemorrhagic	Incidence	0·52 (0·52 – 0·53)	1·69 (1·68 – 1·71)
First ever acute haemorrhagic stroke	Log-transformed age-standardized SEV scalar: haemorrhagic stroke	Incidence	0·77 (0·75 – 0·82)	2·17 (2·12 – 2·27)
First ever acute haemorrhagic stroke	Any stroke	Excess mortality rate	-0·48 (-0·66 – -0·32)	0·62 (0·52 – 0·73)
First ever acute haemorrhagic stroke	First-ever acute stroke, ischaemic or haemorrhagic	Excess mortality rate	-0·081 (-0·3 – 0·16)	0·62 (0·52 – 0·73)
First ever acute ischaemic stroke	Hospital data	Incidence	0·38 (0·37 – 0·38)	1·46 (1·45 – 1·46)
First ever acute ischaemic stroke	Any stroke	Incidence	0·31 (0·29 – 0·33)	1·37 (1·34 – 1·39)
First ever acute ischaemic stroke	First-ever acute stroke, ischaemic or haemorrhagic	Incidence	0·37 (0·36 – 0·38)	1·44 (1·43 – 1·46)
First ever acute ischaemic stroke	Log-transformed age-standardized SEV scalar: ischaemic stroke	Incidence	1·16 (1·09 – 1·22)	3·21 (2·99 – 3·39)

Step 2:

Cause	Variable name	Measure	beta	Exponentiated beta
Chronic ischaemic stroke with CSMR	Log-transformed SEV scalar: Ischaemic stroke	Prevalence	0·89 (0·75 – 1·19)	2·44 (2·13 – 3·27)
Chronic ischaemic stroke with CSMR	LDI (I$ per capita)	Excess mortality rate	-0·49 (-0·5 – -0·46)	0·61 (0·61 – 0·63)
Chronic haemorrhagic stroke with CSMR	Log-transformed SEV scalar: Haemorrhagic stroke	Prevalence	0·88 (0·75 – 1·15)	2·40 (2·13 – 3·17)

Cause	Variable name	Measure	beta	Exponentiated beta
Chronic haemorrhagic stroke with CSMR	LDI (I$ per capita)	Excess mortality rate	-0·48 (-0·5 – -0·44)	0·62 (0·61 – 0·64)
First-ever acute haemorrhagic stroke with CSMR	Any stroke	Incidence	1·27 (1·27 – 1·29)	3·58 (3·56 – 3·62)
First-ever acute haemorrhagic stroke with CSMR	First-ever acute stroke, ischaemic or haemorrhagic	Incidence	0·52 (0·52 – 0·54)	1·69 (1·68 – 1·71)
First-ever acute haemorrhagic stroke with CSMR	Log-transformed SEV scalar: Hem stroke	Incidence	1·11 (1·01 – 1·20)	3·03 (2·74 – 3·33)
First-ever acute haemorrhagic stroke with CSMR	Any stroke	Excess mortality rate	-0·37 (-0·49 – -0·27)	0·69 (0·62 – 0·77)
First-ever acute haemorrhagic stroke with CSMR	First-ever acute stroke, ischaemic or haemorrhagic	Excess mortality rate	0·023 (-0·2 – 0·23)	1·02 (0·82 – 1·25)
First-ever acute ischaemic stroke with CSMR	Any stroke	Incidence	0·32 (0·30 – 0·33)	1·38 (1·35 – 1·39)
First-ever acute ischaemic stroke with CSMR	First-ever acute stroke, ischaemic or haemorrhagic	Incidence	0·37 (0·36 – 0·38)	1·44 (1·43 – 1·46)
First-ever acute ischaemic stroke with CSMR	Log-transformed age-standardized SEV scalar: Ischaemic stroke	Incidence	1·11 (1·05 – 1·18)	3·04 (2·86 – 3·26)
First-ever acute ischaemic stroke with CSMR	Any stroke	Excess mortality rate	-0·34 (-0·45 – -0·24)	0·71 (0·64 – 0·79)
First-ever acute ischaemic stroke with CSMR	First-ever acute stroke, ischaemic or haemorrhagic	Excess mortality rate	-0·69 (-0·82 – -0·56)	0·51 (0·44 – 0·57)

Sequelae and disability weights for ischaemic stroke, intracerebral haemorrhage and subarachnoid haemorrhage

Sequela	Health state lay description	Disability weight
Asymptomatic chronic stroke	-	N/A
Acute and chronic stroke, severity level 1	Has some difficulty in moving around and some weakness in one hand, but is able to walk without help	0·019 (0·01 – 0·032)
Acute and chronic stroke, severity level 2	Has some difficulty in moving around and in using the hands for lifting and holding things, dressing and grooming	0·07 (0·046 – 0·099)
Acute and chronic stroke, severity level 3	Has some difficulty in moving around, in using the hands for lifting and holding things, dressing and grooming, and in speaking· The person is often forgetful and confused	0·316 (0·205 – 0·438)
Acute and chronic stroke, severity level 4	Is confined to bed or a wheelchair, has difficulty speaking and depends on others for feeding, toileting and dressing	0·552 (0·376 – 0·707)

Sequela	Health state lay description	Disability weight
Acute and chronic stroke, severity level 5	Is confined to bed or a wheelchair, depends on others for feeding, toileting and dressing, and has difficulty speaking, thinking clearly and remembering things	0·588 (0·411 – 0·745)

ICD Codes used in fatal and non-fatal analysis

Fatal analysis

	ICD10	ICD9
Cerebrovascular disease	G45-G46.8, I60-I63.9, I65-I66.9, I67.0-I67.3, I67.5-I67.6, I68.1-I68.2, I69.0-I69.3	430-435.9, 437.0-437.2, 437.5-437.8
Ischaemic stroke	G45-G46.8, I63-I63.9, I65-I66.9, I67.2-I67.3, I67.5-I67.6, I69.3	433-435.9, 437.0-437.1, 437.5-437.8
Haemorrhagic stroke	I60-I62.9, I67.0-I67.1, I68.1- I68.2, I69.0-I69.2	430-432.9, 437.2

Nonfatal analysis

	ICD10	ICD9
Cerebrovascular disease	I60-I63.9, I65-I66.9, I67.0-I67.3, I67.5-I67.6, I68.1-I68.2, I69.0- I69.3	430-434.9, 437.0-437.2, 437.5-437.8
Ischaemic stroke	I63-I63.9, I65-I66.9, I67.2-I67.3, I67.5-I67.6, I69.3	433-434.9, 437.0-437.1, 437.5-437.8
Haemorrhagic stroke	I60-I62.9, I67.0-I67.1, I68.1- I68.2, I69.0-I69.2	430-432.9, 437.2

5. Supplementary limitations

The GBD Study 2019, like any large-scale epidemiological study, is subject to several potential sources of bias. All possible biases related to data analyses were dealt with in the same way as in other GBD estimates. While the specific biases can vary depending on the disease, region, and data sources, here are some common types of biases that could potentially affect the estimates:

- Selection bias: This can occur if the data sources used in the study are not representative of the entire population. For example, if data is primarily collected from urban hospitals, this may not accurately reflect the disease burden in rural areas.
- Information bias: This can occur if there are inaccuracies in the data that is collected. This could be due to misdiagnosis, underreporting, or errors in data entry.
- Confounding bias: This occurs when the effect of one factor (such as a risk factor) on an outcome (such as disease incidence) is mixed with the effect of another factor.
- Bias in disease modelling: The GBD study uses complex statistical models to estimate disease burden. The assumptions made in these models can introduce bias.
- Bias due to missing data: Not all countries have comprehensive health data, and the methods used to estimate disease burden in these cases can introduce bias.
- Temporal bias: This can occur when the data used does not accurately reflect the current disease burden due to changes over time.
- Geographical bias: This can occur if the data sources used in the study do not equally represent all geographical areas.
- Bias in risk factor attribution: The GBD study estimates the disease burden attributable to various risk factors. Bias can be introduced in the process of attributing disease burden to these risk factors.
- Publication bias: This occurs when the published studies that are included in the analysis do not represent all relevant studies.
- Bias in disability weights: The GBD study uses disability weights to calculate years lived with disability (YLDs). Bias can be introduced in the process of assigning these weights.

It is important to note that the GBD study uses rigorous methods and multiple data sources to minimise these biases and provide the most accurate estimates possible. However, all epidemiological studies are subject to some degree of bias, and these should be considered when interpreting the results.

6. Confounding factors in context

- Selection bias: This occurs when the data used in the study is not representative of the entire population. For instance, if a study primarily uses data from urban hospitals, it may not accurately reflect the disease burden in rural areas. To mitigate this, GBD team strived to use a diverse range of data sources that cover different demographics and regions.
- Information bias: This happens when there are inaccuracies in the data collected due to factors like misdiagnosis, underreporting, or data entry errors. For all levels, GBD ensures rigorous data collection and validation processes to minimise this bias.
- Confounding bias: This arises when the effect of one factor on an outcome is mixed with the effect of another factor. GBD used different statistical methods to adjust for potential confounders to address this bias.
- Bias in disease modelling: The assumptions made in statistical models can introduce bias. GBD has assessed the impact of this bias through cross-validation or using different models and comparing the results.
- Bias due to missing data: In cases where comprehensive health data were not available for all countries, the methods used to estimate disease burden can introduce bias. Multiple imputation or other statistical techniques were used to handle missing data and to involve accurate and comprehensive assessments. GBD has used imputation techniques to consider missing data; conducted sensitivity analyses to assess how different approaches to handling missing data have impacted the results to assess robustness of the estimates; has considered the quality of available data sources for each country; and collaborated with countries to improve data reporting and fill gaps. This ongoing collaboration helps enhance the accuracy of estimates over time. By incorporating these elements, the GBD study aims to transparently communicate the level of missing data for each country, enabling users to interpret the results with a clear understanding of the data limitations.
- Temporal bias: This can occur when the data used does not accurately reflect the current disease burden due to changes over time. GBD could mitigate this bias through using the most recent data and accounting for trends over time.
- Geographical bias: If the data sources used in the study do not equally represent all geographical areas, this can introduce bias. Using a diverse range of data sources could help address this issue.
- Bias in risk factor attribution: Bias can be introduced in the process of attributing disease burden to various risk factors. Sensitivity analyses were used to assess the robustness of these attributions.
- Publication bias: This occurs when the published studies included in the analysis do not represent all relevant studies. A comprehensive literature search and the use of unpublished data in GBD 2019 study could help mitigate this bias.
- Bias in disability weights: Bias can be introduced in the process of assigning disability weights. GBD has used a wide range of resources for data and assigned these weights to all analyses.

It is important to note that while these biases can affect the results, we were aware of them and did our best to use rigorous methods and multiple data sources to provide the most accurate estimates possible. However, all epidemiological studies are subject to some degree of bias, and these should be considered when interpreting the results.

Supplementary Results

1. All ages values

In North Africa and Middle East, the proportion of neurological DALYs (6.1% of global neurological DALYs; absolute numbers; Figure S4) was lower than in Southeast Asia, East Asia, and Oceania (35·4% of global neurological DALYs), Central Europe, Eastern Europe, and Central Asia (8·1%), high-income (13·6%), South Asia (19·1%), and in Sub-Saharan Africa (12·2%) regions and slightly higher than in Latin America and the Caribbean region (5·6%). In NAME, total neurological YLDs were 7·6 million (3·7–13·8; 12·2% of global neurological YLDs) and neurological YLLs were 9·9 million (8·1–12·7; 9·8% of global neurological YLLs) in 2019. Total deaths related to NCs were 4·7% of global neurological deaths, the number of new NCs was 7·6% of global neurological new events per year, and the number of prevalent cases with NCs was 7·8% of global NCs. Lebanon, Qatar, Kuwait, and Bahrain had the lowest rates of stroke DALYs (Figure 2).

Figure S4. The proportions of disability-adjusted life years related to neurological conditions in A) North Africa and Middle East and B) the globe.

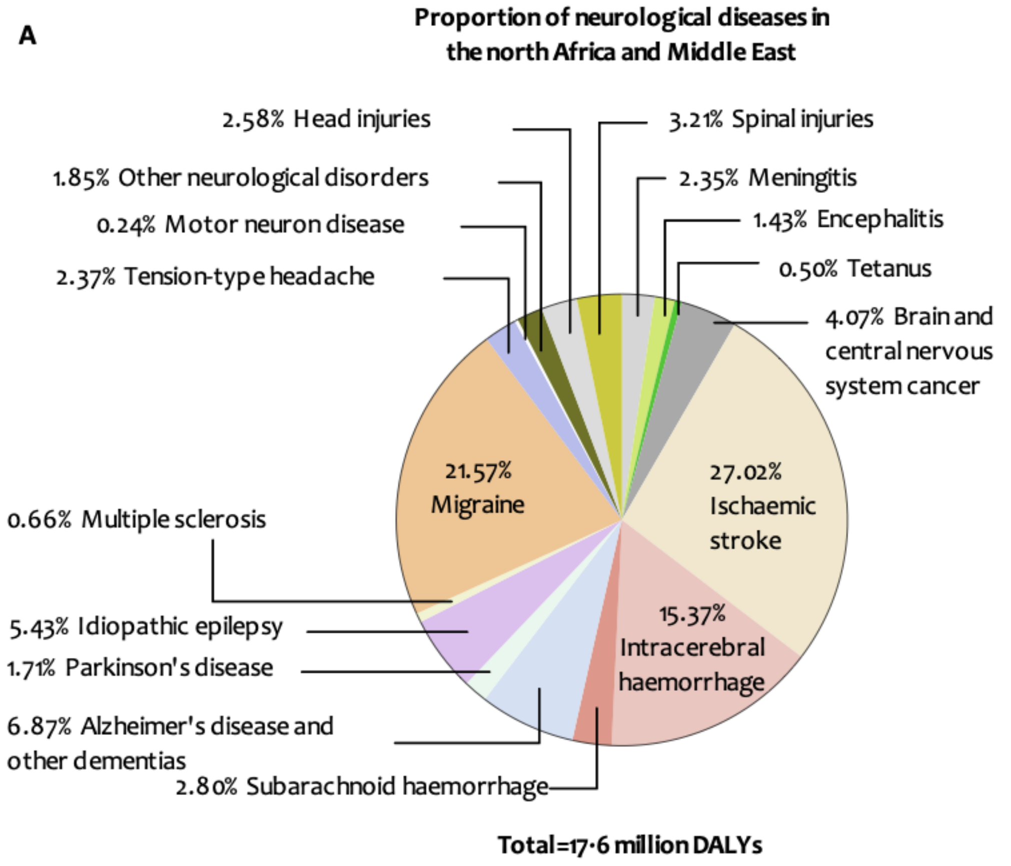

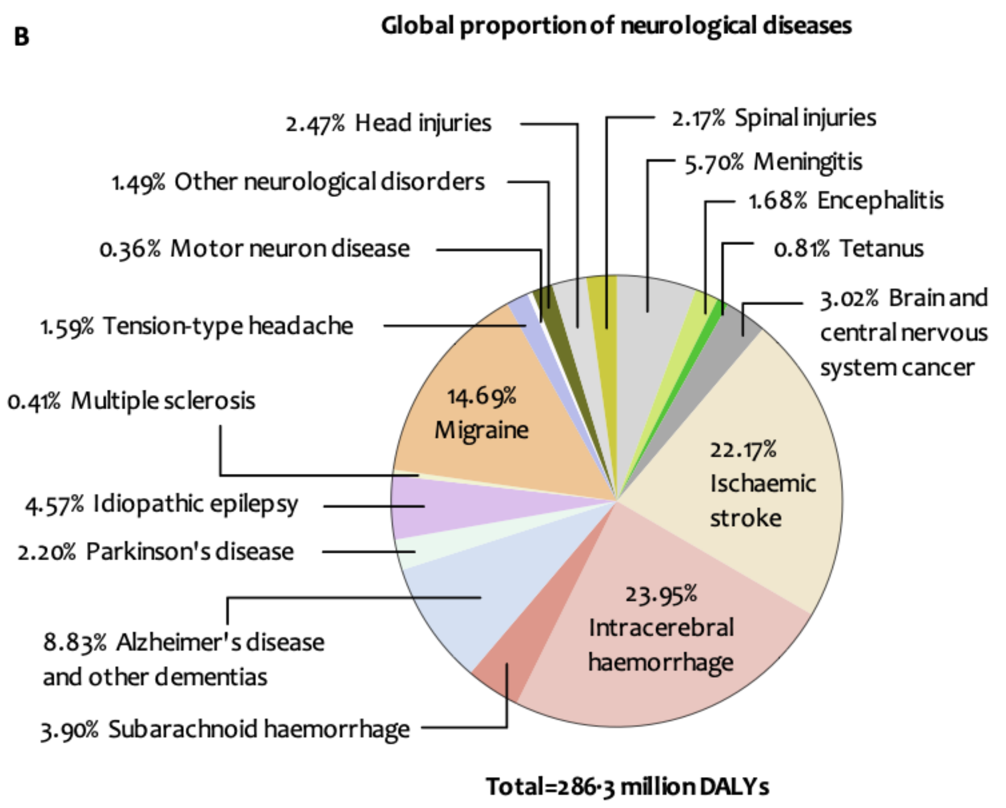

In 2019, among all NCs in NAME, stroke (7·9, 95% UI 7·1–8·9 million DALYs; 45·2% of all neurological DALYs), migraine (3·8, 0·6–8·7 million; 21·6%), and dementia (1·2, 0·5–2·7 million; 6·9%) were the leading causes of neurological DALYs with the highest absolute number. Likewise, stroke (312·2, 278·4–349·7 thousand deaths; 70·8%) and dementia (70·5, 17·2–185·8 thousand; 16·0%) were the leading causes of neurological deaths (overall, 86·8% of all neurological deaths). Heat maps in Figure S5 are showing the status of all-age DALYs of neurological conditions in the 21 countries of North Africa and Middle East in 2019.

Figure S5. Heat maps of the burden of neurological conditions in all ages among 21 countries of North Africa and Middle East in 2019

(A) stroke disability-adjusted life years (DALYs); (B) neurological disorders DALYs; (C) Alzheimer's disease and other dementias DALYs; (D) headache disorders DALYs; (E) brain and central nervous system DALYs; (F) meningitis DALYs; (G) encephalitis DALYs; (H) tetanus DALYs; (I) head injuries years lived with disability (YLDs); and (J) spinal injuries YLDs per 100,000 people. Abbreviations: DALYs, disability-adjusted life years; YLDs, years lived with disability; Neurological disorders include Alzheimer's disease and other dementias, Parkinson's disease, idiopathic epilepsy, multiple sclerosis, headache disorders, motor neuron disease, and other neurological disorders; Headache disorders include migraine and tension-type headache.

A. Stroke

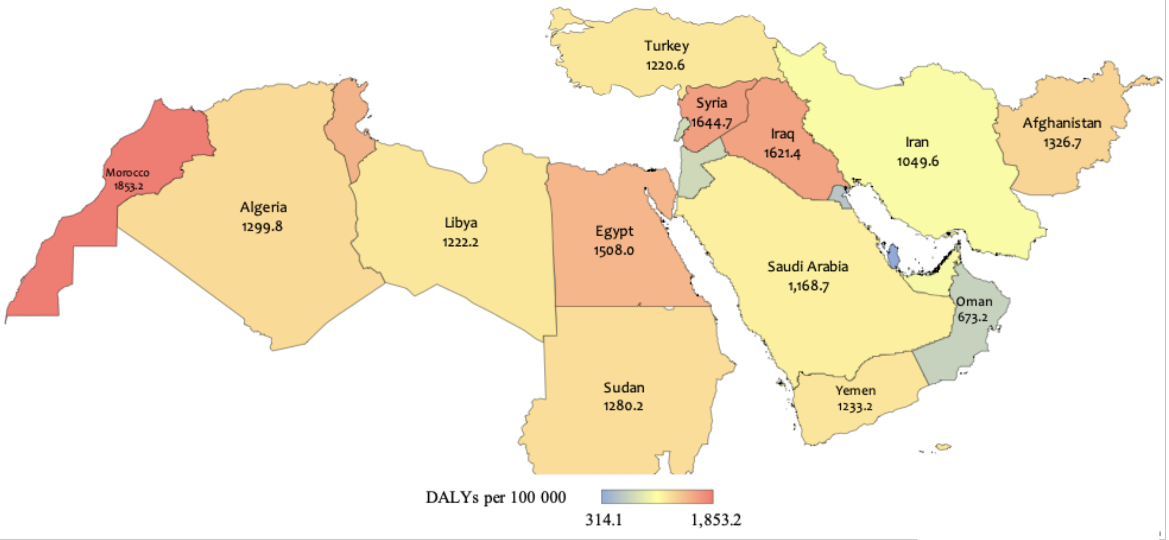

B. Neurological disorders

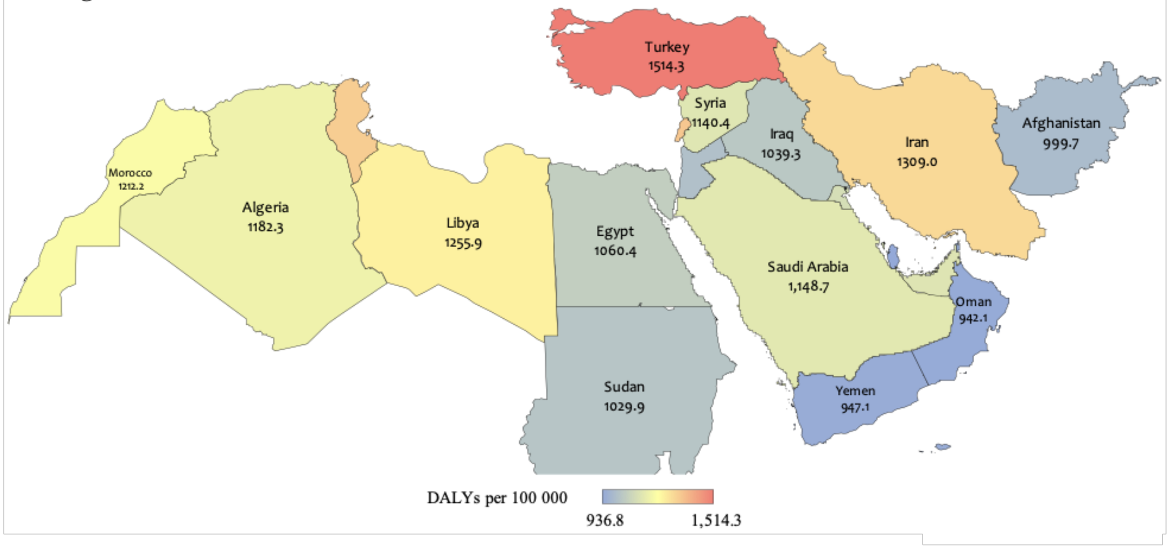

C. Alzheimer's disease and other dementias

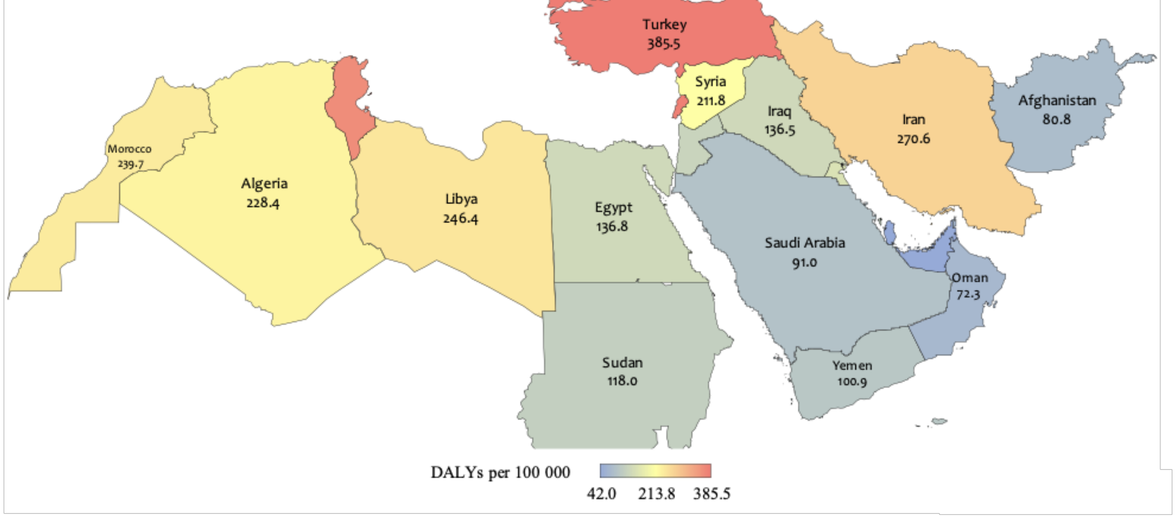

D. Headache disorders

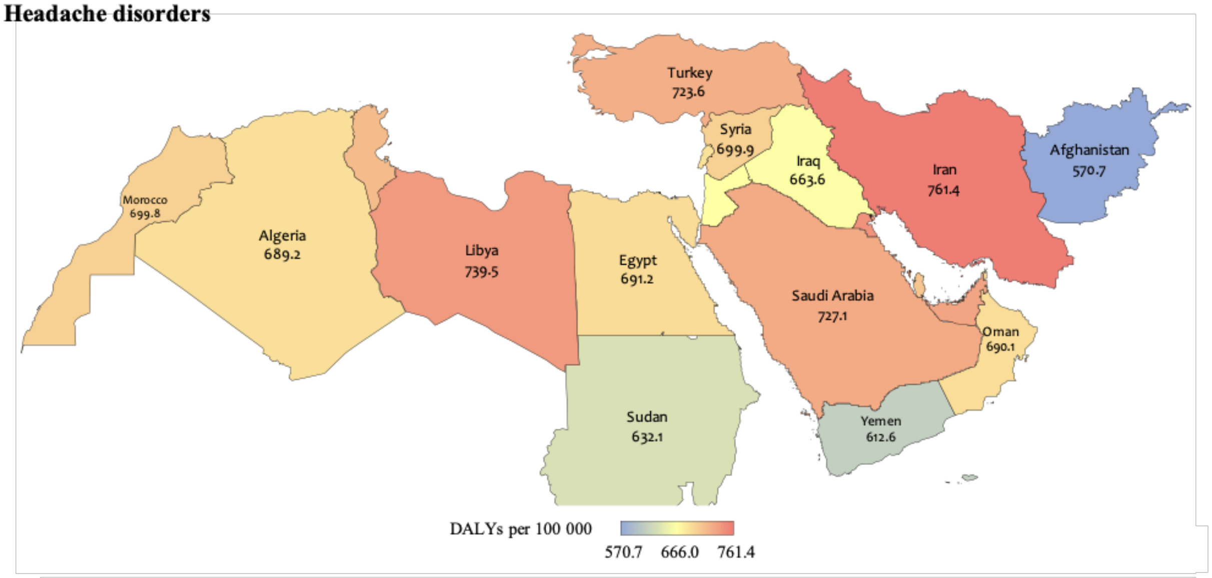

E. Brain and central nervous system

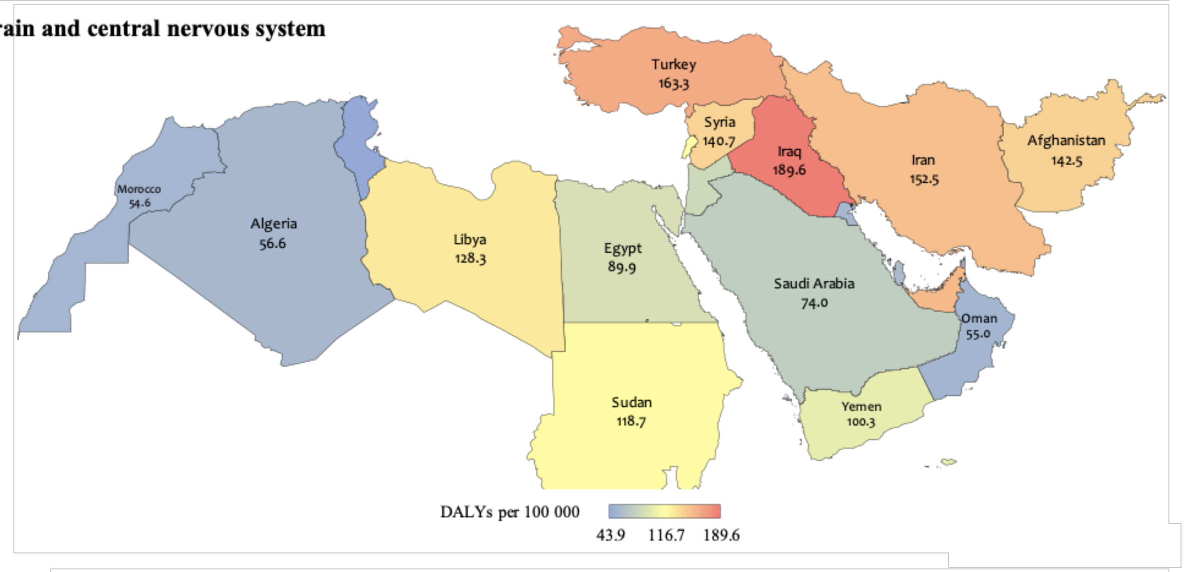

F. Meningitis

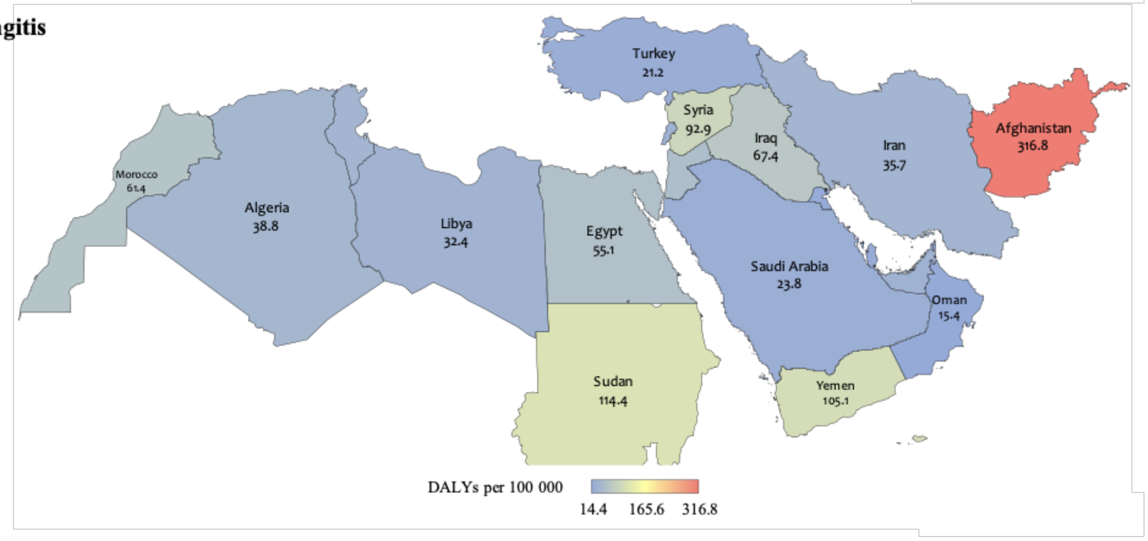

G. Encephalitis

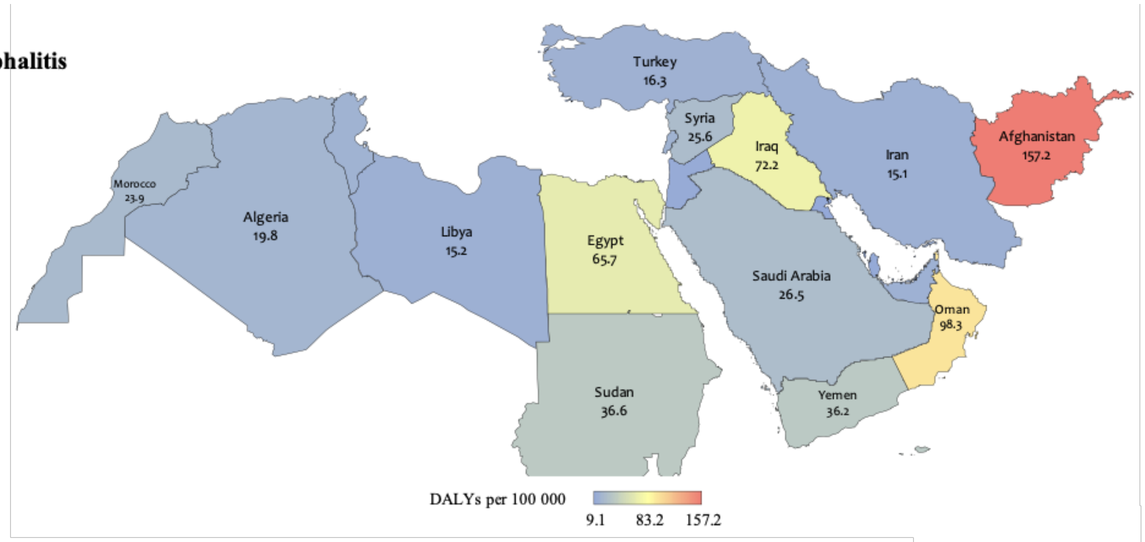

H. Tetanus

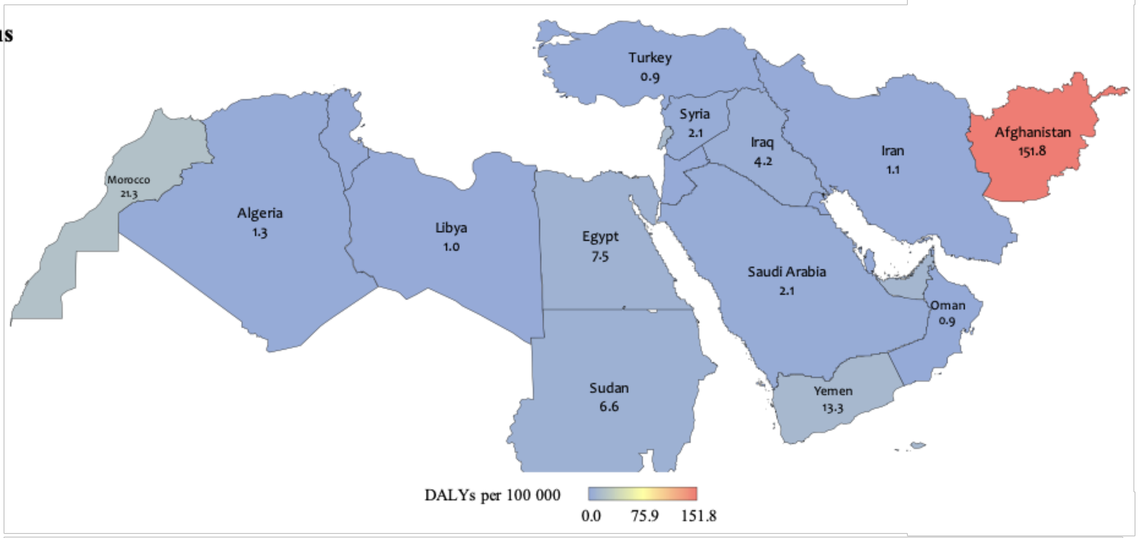

I. Head injuries

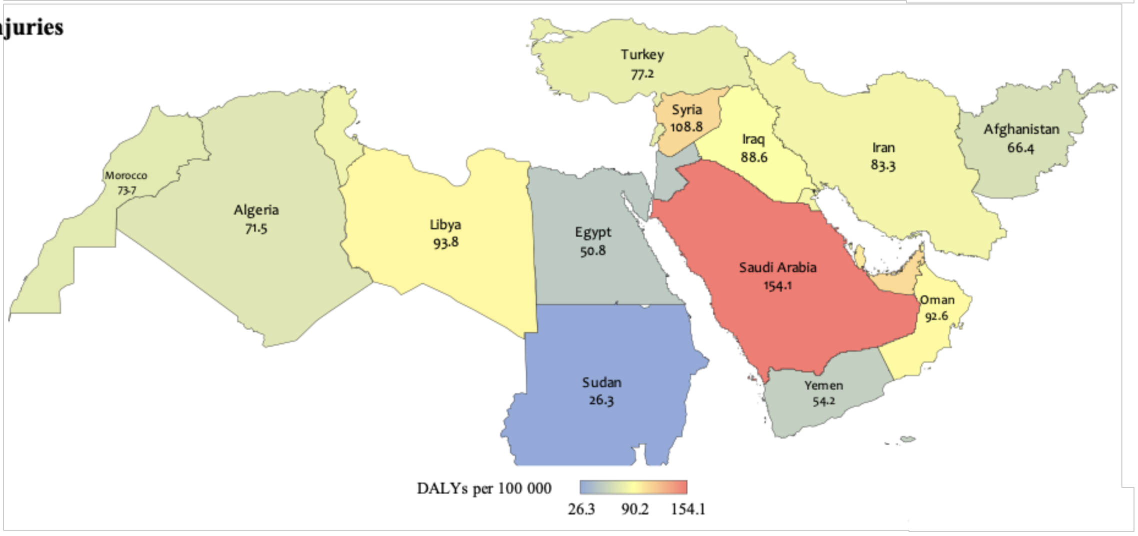

J. Spinal injuries

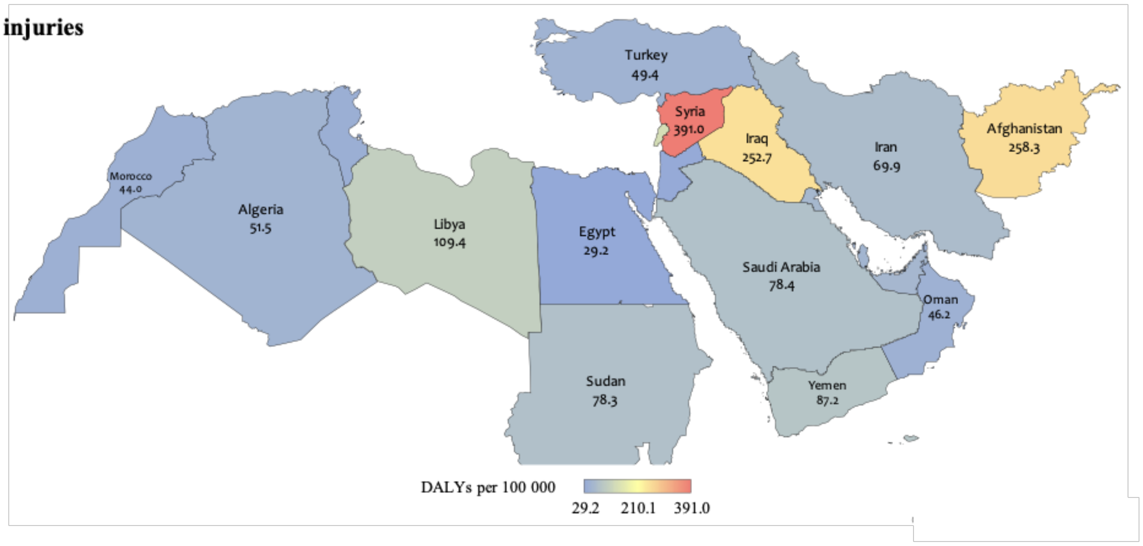

From 1990 to 2019, the absolute number of DALYs and deaths related to meningitis, tetanus, and subarachnoid haemorrhage decreased in NAME, but all other NCs almost doubled (Table S3; Figure S6). The highest increases in the number of deaths were related to dementias (1·9-time, 95% UI 1·7 to 2·5), Parkinson's disease (1·8-time, 1·5 to 2·3), multiple sclerosis (1·5-time, 0·8 to 2·5), and ischaemic stroke (1·4-time, 1·1 to 1·7; Table 1). The highest increases in the number of new cases during this period were related to Parkinson's disease, dementia, ischaemic stroke, and CNS cancer; on average with a 1·5 to 1·9 times increase. From 2010 to 2019 in particular, the number of new dementias and their burden doubled in the UAE, Qatar, Jordan, and Bahrain. The UAE and Qatar had the highest rises in the absolute number of DALYs, death, incidence, and prevalence related to almost all NCs (supplementary Table S3).

Figure S6. Trends of the absolute number of disability-adjusted life years (DALYs) related to neurological conditions in North Africa and Middle East

(A) meningitis; (B) encephalitis; (C) tetanus; (D) ischaemic stroke; (E) intracerebral haemorrhage; (F) subarachnoid haemorrhage; (G) Alzheimer's disease and other dementias; (H) migraine; (I) tension-type headache; (J) Parkinson's disease; (K) idiopathic epilepsy; (L) multiple sclerosis; (N) motor neuron disease; (O) other neurological disorders; and (P) brain and central nervous system cancer.

Figure S6 Trends of the absolute number of disability-adjusted life years lost (DALYs) related to neurological conditions in North Africa and Middle East

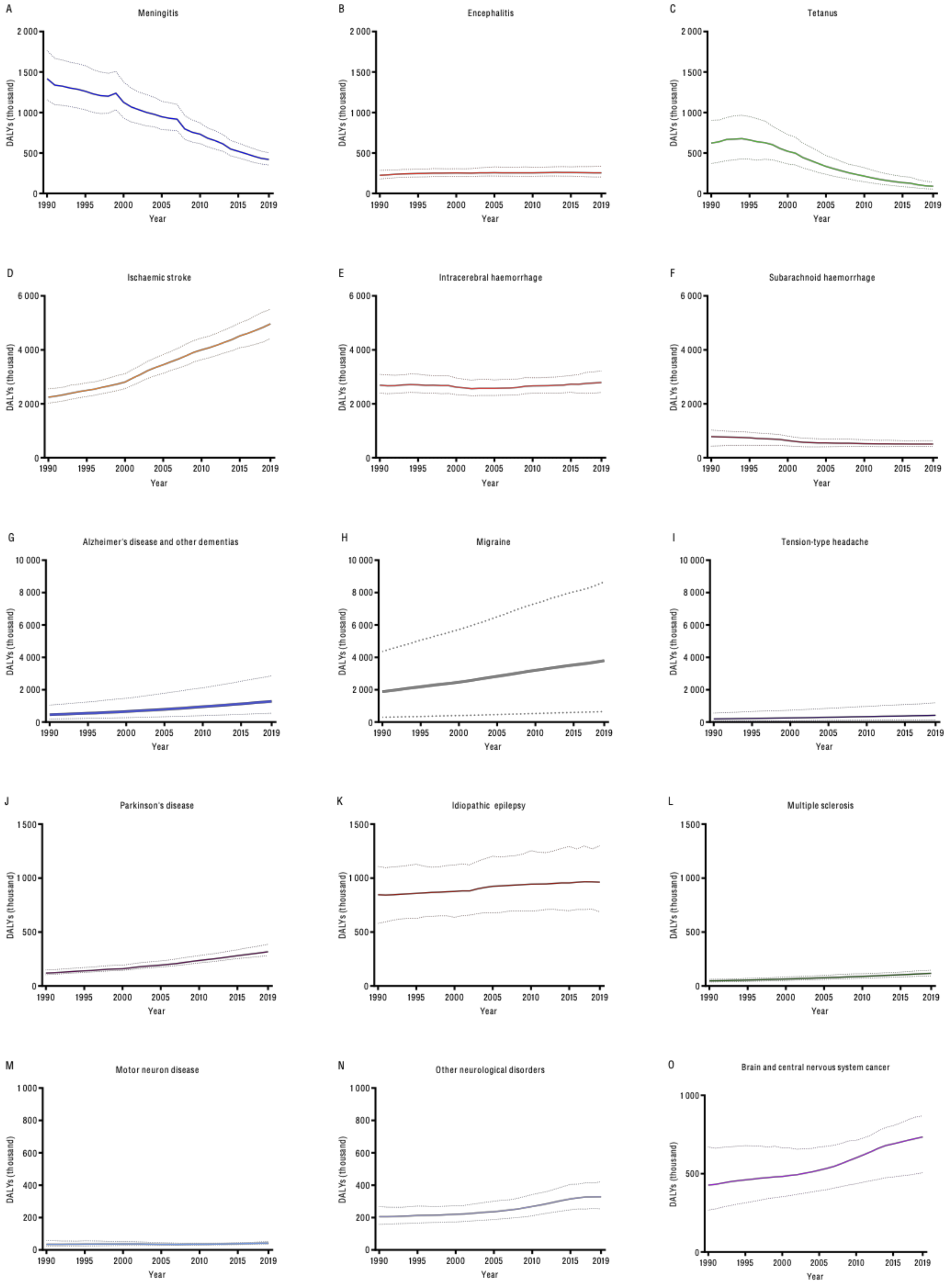

2. Age-standardised values

In NAME, the age-standardised rates of stroke DALYs per 100,000 (1826·2, 95% UI 1635·3–2026·2) were higher than HICs (653·2, 596·1–704·9), Latin America and Caribbean (1060·0, 976·7–1144·7), and South Asia (1702·6, 1510·9–1891·0 (Figure S7).

Figure S7. Ranks of age-standardised neurological disability-adjusted life years (DALYs) rates in seven GBD super regions and four World Bank income levels in 2019.

Ranks range from 1 (dark green) with the lowest rate to 12 (dark red) with the highest rate. Stroke includes ischaemic stroke, intracerebral haemorrhage, and subarachnoid haemorrhage; Neurological disorders include Alzheimer's disease and other dementias, Parkinson's disease, idiopathic epilepsy, multiple sclerosis, headache disorders, motor neuron disease, and other neurological disorders; Headache disorders include migraine and tension-type headache.

	Stroke	Ischaemic stroke	Intracerebral haemorrhage	Subarachnoid haemorrhage	Neurological disorders	Alzheimer's disease and other dementias	Parkinson's disease	Idiopathic epilepsy	Multiple sclerosis	Headache disorders	Migraine	Tension-type headache	Motor neuron disease	Other neurological disorders	Brain and central nervous system cancer	Meningitis	Encephalitis	Tetanus
World Bank Low Income	1	6	1	8	9	5	7	2	10	12	12	11	11	6	11	2	5	2
Southeast Asia, East Asia, and Oceania	2	3	3	5	12	2	2	12	12	10	10	12	8	12	7	7	6	7
Central Europe, Eastern Europe, and Central Asia	3	1	8	1	5	8	12	7	3	4	6	1	5	5	1	10	4	11
Sub-Saharan Africa	4	8	2	12	6	6	10	1	11	11	11	6	12	2	12	1	9	1
World Bank Lower Middle Income	5	5	4	4	8	11	3	5	7	6	5	7	10	9	9	3	2	4
World Bank Upper Middle Income	6	4	6	7	10	3	6	9	9	9	9	9	6	10	3	9	10	9
North Africa and Middle East	7	2	9	11	1	1	1	8	4	1	1	4	7	8	2	6	7	6
Global	8	7	7	6	7	7	5	6	5	7	8	5	4	7	8	4	3	5
South Asia	9	9	5	2	11	12	4	4	8	8	7	10	9	11	10	5	1	3
Latin America and Caribbean	10	10	10	3	2	4	11	3	6	5	4	8	3	1	6	8	8	8
World Bank High Income	11	11	11	10	3	9	8	10	2	3	3	3	2	4	4	12	11	10
High-income	12	12	12	9	4	10	9	11	1	2	2	2	1	3	5	11	12	12

Trends of neurological DALYs are reported in the main text and illustrated in Figure S8.

Figure S8. Trends of the age-standardised rate of disability-adjusted life years (DALYs) related to neurological conditions in North Africa and Middle East.

(A) meningitis; (B) encephalitis; (C) tetanus; (D) ischaemic stroke; (E) intracerebral haemorrhage; (F) subarachnoid haemorrhage; (G) Alzheimer's disease and other dementias; (H) migraine; (I) tension-type headache; (J) Parkinson's disease; (K) idiopathic epilepsy; (L) multiple sclerosis; (N) motor neuron disease; (O) other neurological disorders; and (P) brain and central nervous system cancer.

Figure S8 Trends of the age-standardised rate of disability-adjusted life years (DALYs) related to neurological conditions in North Africa and Middle East

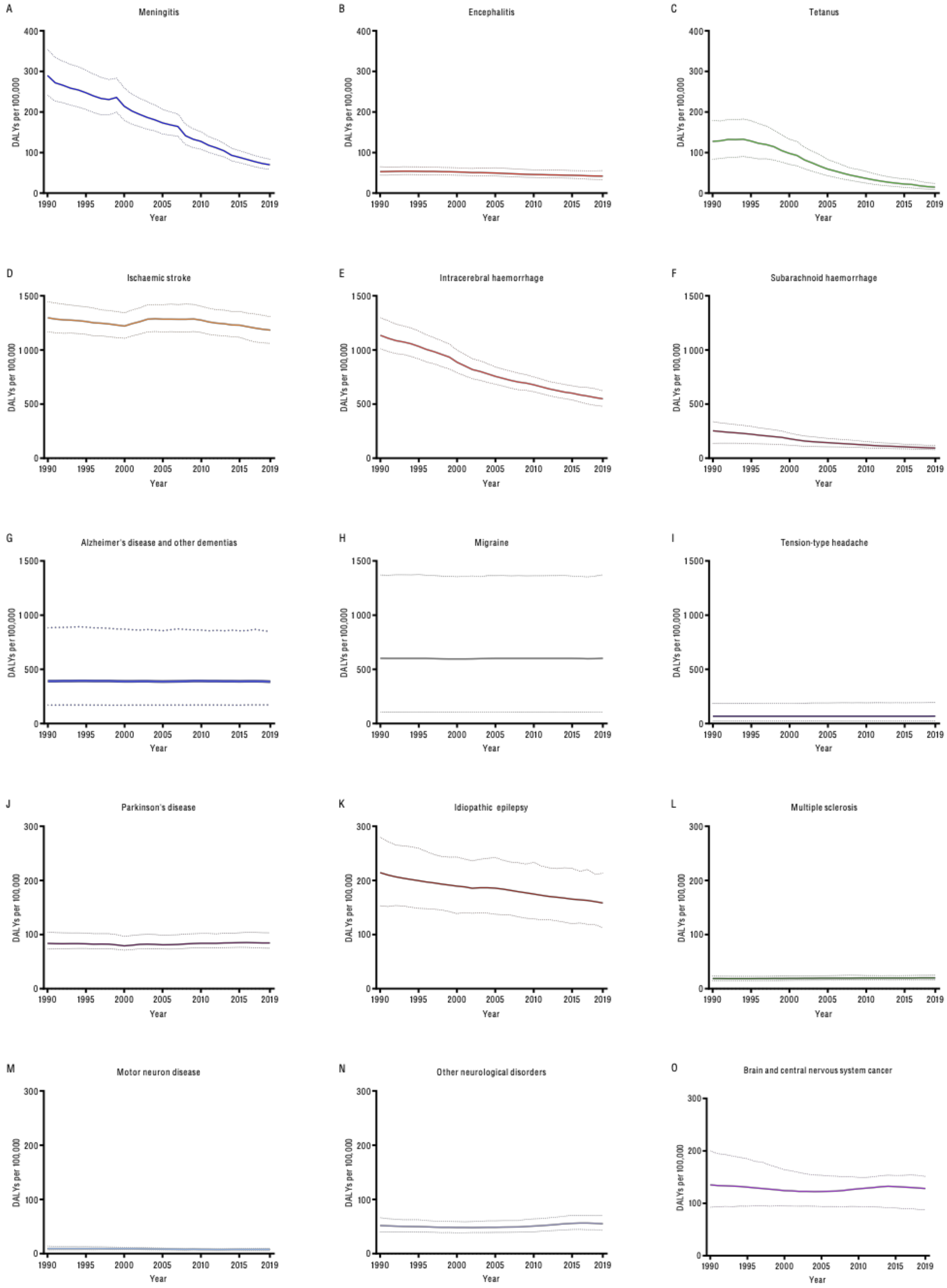

From 1990 to 2019, in NAME, the highest declines in the age-standardised death rate were related to tetanus (-88·9%, -93·3% to -80·6%), meningitis (-71·6%, -77·1% to -64·9%), subarachnoid haemorrhage (-59·0%, -70·7% to -31·6%), and idiopathic epilepsy (-33·4%, -46·2% to 2·1%; Table 1). Parkinson's disease (12·7%, 9·9% to 15·7%), ischaemic stroke (8·8%, 6·3% to 11·4%), and multiple sclerosis (5·5%, 4·0% to 6·8%) had the most substantial rises in age-standardised incidence rate from 1990 to 2019. We observed statistically significant rises in the age-standardised incidence rates in a) Saudi Arabia for CNS cancer; b) Saudi Arabia, Egypt, and Libya for all-type stroke; c) Egypt, Libya, Sudan, Saudi Arabia, Afghanistan, Yemen, Tunisia, Morocco, and Lebanon for ischaemic stroke; d) Oman, Saudi Arabia, Qatar, Morocco, Türkiye, the UAE, Iran, Bahrain, Tunisia, Egypt, Libya, the UAE, and the Syrian Arab Republic for Parkinson's disease; e) all the countries of the region except for Iran, the UAE, Jordan, and Türkiye for multiple sclerosis; f) Iran for headache disorders; and g) the UAE, Türkiye, Bahrain, Iraq, and Iran for motor neuron disease (supplementary Figure S9 and Table S4).

Figure S9. Ranks of age-standardised neurological disability-adjusted life years (DALYs) rates in North Africa and Middle East countries compared with other regions in the world in 2019.

Ranks range from 1 (dark red) with the highest rate to 18 (dark green) with the lowest rate. Stroke includes ischaemic stroke, haemorrhagic stroke, and subarachnoid haemorrhage; Neurological disorders include Alzheimer's disease and other dementias, Parkinson's disease, idiopathic epilepsy, multiple sclerosis, headache disorders, motor neuron disease, and other neurological disorders; Headache disorders include migraine and tension-type headache.

	Global	Southeast Asia, East Asia, and Oceania	Central Europe, Eastern Europe, and Central Asia	High-income	Latin America and Caribbean	South Asia	Sub-Saharan Africa	World Bank High Income	World Bank Upper Middle Income	World Bank Lower Middle Income	World Bank Low Income	**North Africa and Middle East**
Stroke	1	1	1	3	2	1	1	2	1	1	1	**1**
Neurological disorders	2	4	3	1	1	2	3	1	2	2	3	**2**
Intracerebral haemorrhage	3	2	4	7	6	3	2	7	4	3	2	**6**
Ischaemic stroke	4	3	2	5	5	4	5	5	3	4	4	**3**
Headache disorders	5	5	5	2	3	5	6	3	5	5	6	**4**
Migraine	6	6	6	4	4	6	7	4	6	6	7	**5**
Alzheimer's disease and other dementias	7	7	7	6	7	7	8	6	7	7	8	**7**
Meningitis	8	12	15	16	13	8	4	16	14	8	5	**12**
Idiopathic epilepsy	9	10	9	9	8	9	9	9	8	9	9	**8**
Subarachnoid haemorrhage	10	8	8	10	9	11	11	10	9	10	10	**10**
Brain and central nervous system cancer	11	9	10	8	10	13	14	8	10	12	13	**9**
Parkinson's disease	12	11	12	11	11	12	12	11	11	13	12	**11**
Encephalitis	13	14	14	17	15	10	16	17	15	11	16	**15**
Tension-type headache	14	13	11	12	14	14	15	12	12	14	15	**13**
Other neurological disorders	15	15	13	13	12	16	13	13	13	15	14	**14**
Tetanus	16	16	18	18	18	15	10	18	18	16	11	**17**
Multiple sclerosis	17	18	16	15	17	17	17	14	17	17	17	**16**
Motor neuron disease	18	17	17	14	16	18	18	15	16	18	18	**18**

	Algeria	Bahrain	Egypt	Iran	Iraq	Jordan	Kuwait	Lebanon	Libya	Morocco	Palestine	Oman	Qatar	Saudi Arabia	Syrian Arab Republic	Tunisia	Turkey	United Arab Emirates	Yemen	Afghanistan	Sudan
Stroke	1	2	1	2	1	1	2	2	1	1	1	1	2	1	1	1	2	1	1	1	1
Neurological disorders	2	1	3	1	3	2	1	1	2	3	3	2	1	2	2	2	1	2	3	3	3
Intracerebral haemorrhage	6	7	5	7	4	7	7	8	7	6	6	6	7	4	4	7	6	6	4	4	4
Ischaemic stroke	3	4	2	3	2	3	3	5	3	2	2	3	4	3	3	3	3	3	2	2	2
Headache disorders	4	3	4	4	5	4	4	3	4	4	4	4	3	5	5	4	4	4	5	5	5
Migraine	5	5	6	5	6	5	5	4	5	5	5	5	5	6	6	5	5	5	6	6	6
Alzheimer's disease and other dementias	7	6	7	6	7	6	6	6	6	7	7	7	6	7	7	6	7	7	7	7	7
Meningitis	14	14	14	14	13	12	14	13	14	11	14	15	14	15	11	14	15	15	11	8	11
Idiopathic epilepsy	8	8	9	9	9	8	8	7	8	8	9	10	9	8	10	8	8	8	9	10	8
Subarachnoid haemorrhage	10	13	8	12	11	14	13	14	11	9	12	11	11	13	9	11	10	13	8	9	10
Brain and central nervous system cancer	12	10	10	8	8	9	9	9	9	13	8	12	10	10	8	13	9	9	10	11	9
Parkinson's disease	9	9	11	11	10	10	11	10	10	10	10	8	8	9	12	9	12	10	12	14	12
Encephalitis	16	16	13	16	12	16	16	16	16	15	16	9	16	14	15	16	17	17	15	12	15
Tension-type headache	11	12	12	10	14	11	10	11	12	12	11	13	12	11	13	10	13	12	13	15	13
Other neurological disorders	13	11	15	13	15	13	12	12	13	14	13	14	13	12	14	12	11	11	14	16	14
Tetanus	18	18	18	18	17	18	18	17	18	16	18	18	18	18	18	18	18	14	17	13	17
Multiple sclerosis	15	15	16	15	16	15	15	15	15	17	15	16	15	16	16	15	16	16	16	17	16
Motor neuron disease	17	17	17	17	18	17	17	18	17	18	17	17	17	17	17	17	14	18	18	18	18

From 1990 to 2019, in NAME, the age-standardised rates of YLDs related to head injuries increased (1·9%, -3·5% to 5·4%) and spinal injuries decreased (-5·4%, -27·6% to 12·3%), both non-statistically significantly. Countries with a statistically significant rise in the rate of YLDs related to head injuries were the Syrian Arab Republic (107·7%, 51·8% to 211·9%), Saudi Arabia, Türkiye, Yemen, Palestine, and Libya (9·9%, 3·2% to 18·6%). Likewise, those with a significant rise in the rate of YLDs related to spinal injuries were the Syrian Arab Republic (579·2%, 218·2% to 1263·6%), Yemen, Libya, Türkiye, and Sudan (22·2%, 5·5% to 42·6%). In contrast, Lebanon, Iran, Iraq,

Afghanistan, Kuwait, Algeria, Qatar, Oman, and UAE showed statistically significant declines in age-standardised rates of YLDs related to head or spinal injuries.

3. Modifiable risk factors

Of total risk-attributed stroke DALYs in NAME, on average 73·3% (1287·3, 1028·1–1590·0) were related to metabolic risk factors, 34·1% (598·2, 464·3–757·4) related to behavioural risk factors, and 30·8% (540·9, 400·9–699·4) related to environmental/occupational risk factors (Table S7). In the region, 7·1% (5·0, 3·5–7·0) of meningitis, 3·8% (1·6, 0·9–2·6) of encephalitis, 2·1% (3·4, 1·9–5·5) of idiopathic epilepsy, and 10·8% (2·2, 1·5–3·0) of multiple sclerosis were attributable to modifiable risk factors. Ranks of neurological age-standardised DALYs rates attributable to modifiable risk factors in 21 North Africa and Middle East countries and seven GBD super regions are depicted in Figure S10.

Figure S10. Ranks of age-standardised rates of (A) stroke and (B) dementia disability-adjusted life years (DALYs) attributable to risk factors in North Africa and Middle East countries compared with other regions in the world in 2019.

Ranks range from 1 (dark red) with the highest rate to 20 (dark green) with the lowest rate. Dementia includes Alzheimer's disease and other dementias; Stroke includes ischaemic stroke, haemorrhagic stroke, and subarachnoid haemorrhage.

A Stroke

	Global	Southeast Asia, East Asia, and Oceania	Central Europe, Eastern Europe, and Central Asia	High-income	Latin America and Caribbean	South Asia	Sub-Saharan Africa	World Bank High Income	World Bank Upper Middle Income	World Bank Lower Middle Income	World Bank Low Income	**North Africa and Middle East**
High systolic blood pressure	1	1	1	1	1	1	1	1	1	1	1	**1**
High body-mass index	2	4	2	2	2	4	3	2	3	3	3	**2**
High fasting plasma glucose	3	6	4	3	3	2	4	3	5	2	4	**3**
Ambient particulate matter pollution	4	2	6	7	5	3	5	6	2	4	7	**4**
Smoking	5	3	3	4	4	6	7	4	4	6	5	**6**
Diet high in sodium	6	5	9	10	9	10	8	10	6	9	8	**17**
Household air pollution from solid fuels	7	8	17	19	10	5	2	20	13	5	2	**14**
High LDL cholesterol	8	7	5	5	7	11	12	5	7	10	11	**5**
Kidney dysfunction	9	10	10	11	8	9	9	11	10	8	9	**7**
Diet low in fruits	10	13	12	12	13	7	6	12	12	7	6	**13**
Diet high in red meat	11	9	8	6	6	19	14	7	8	16	15	**15**
Low temperature	12	12	7	8	18	17	15	8	9	17	14	**8**
Alcohol use	13	11	11	9	11	15	11	9	11	14	13	**20**
Lead exposure	14	14	19	18	14	8	13	18	14	11	12	**10**
Diet low in fiber	15	16	15	13	15	13	17	13	17	12	16	**16**
Secondhand smoke	16	15	14	17	17	14	16	16	15	15	17	**12**
Diet low in vegetables	17	18	18	16	12	12	10	17	19	13	10	**18**
Diet low in whole grains	18	17	13	14	19	18	18	14	16	18	18	**9**
Low physical activity	19	19	16	15	16	20	20	15	18	20	20	**11**
High temperature	20	20	20	20	20	16	19	19	20	19	19	**19**

	Algeria	Bahrain	Egypt	Iran	Iraq	Jordan	Kuwait	Lebanon	Libya	Morocco	Palestine	Oman	Qatar	Saudi Arabia	Syrian Arab Republic	Tunisia	Turkey	United Arab Emirates	Yemen	Afghanistan	Sudan
High systolic blood pressure	1	1	1	1	1	1	1	1	1	1	1	1	1	1	1	1	1	1	1	1	1
High body-mass index	2	2	2	2	2	2	2	2	2	3	3	3	2	2	2	3	2	2	3	4	3
High fasting plasma glucose	3	4	4	3	4	3	4	3	3	2	2	2	4	4	3	2	3	3	2	2	2
Ambient particulate matter pollution	4	3	3	4	3	4	3	5	4	4	4	4	3	3	4	4	4	4	4	7	4
Smoking	6	7	6	6	5	6	6	4	7	7	6	7	7	7	5	6	5	6	5	11	7
Diet high in sodium	17	16	16	14	16	17	12	15	16	15	17	16	14	17	17	15	14	15	19	18	19
Household air pollution from solid fuels	20	20	20	20	20	20	19	19	20	16	20	19	20	20	20	20	19	20	6	3	5
High LDL cholesterol	5	5	5	5	6	5	5	6	5	5	5	5	5	5	6	5	6	5	7	5	6
Kidney dysfunction	7	6	7	7	7	7	7	7	6	6	7	6	6	6	7	7	7	7	9	8	8
Diet low in fruits	12	14	14	15	8	11	10	14	11	14	11	15	16	9	10	12	15	13	10	10	12
Diet high in red meat	13	9	10	13	17	13	8	11	12	11	15	9	9	14	13	13	11	10	17	15	17
Low temperature	11	13	12	8	11	10	15	10	13	9	12	13	12	15	8	9	8	14	18	9	18
Alcohol use	19	18	19	19	19	19	20	17	19	20	18	20	18	19	18	16	16	19	20	20	20
Lead exposure	14	15	8	10	13	15	16	13	15	12	14	12	15	11	9	11	13	17	8	6	9
Diet low in fiber	18	17	17	16	14	14	17	16	14	19	10	18	17	18	15	18	17	18	12	13	13
Secondhand smoke	10	12	11	12	10	12	13	12	10	13	13	14	13	10	14	10	12	12	14	16	15
Diet low in vegetables	16	19	18	18	18	16	18	18	17	17	16	17	19	16	16	19	18	16	13	12	16
Diet low in whole grains	9	10	13	9	12	8	14	8	8	8	8	11	11	13	11	8	9	11	11	14	11
Low physical activity	8	8	9	11	9	9	9	9	9	10	9	8	8	8	12	14	10	8	15	17	10
High temperature	15	11	15	17	15	18	11	20	18	18	19	10	10	12	19	17	20	9	16	19	14

B **Dementias**

	Global	Southeast Asia, East Asia, and Oceania	Central Europe, Eastern Europe, and Central Asia	High-income	Latin America and Caribbean	South Asia	Sub-Saharan Africa	World Bank High Income	World Bank Upper Middle Income	World Bank Lower Middle Income	World Bank Low Income	**North Africa and Middle East**	Algeria	Bahrain	Egypt	Iran
Smoking	1	1	2	1	2	1	3	1	1	1	2	**2**	2	3	1	3
High body-mass index	2	3	1	2	1	3	1	2	2	3	3	**1**	1	2	2	1
High fasting plasma glucose	3	2	3	3	3	2	2	3	3	2	1	**3**	3	1	3	2

	Iraq	Jordan	Kuwait	Lebanon	Libya	Morocco	Palestine	Oman	Qatar	Saudi Arabia	Syrian Arab Republic	Tunisia	Turkey	United Arab Emirates	Yemen	Afghanistan	Sudan
Smoking	2	2	2	1	3	3	1	3	3	3	1	2	2	3	1	3	2
High body-mass index	1	1	1	2	1	1	3	1	1	1	2	1	1	1	3	2	3
High fasting plasma glucose	3	3	3	3	2	2	2	2	2	2	3	3	3	2	2	1	1

From 1990 to 2019, the highest rises in stroke-related DALYs attributable to risk factors were observed for high fasting plasma glucose (98·2%, 68·5% to 132·0%), kidney dysfunction (51·9%, 38·8% to 65·2%), ambient particulate matter pollution (51·1%, 32·0% to 79·4%), low physical activity (48·9%, 35·5% to 63·0%), and high LDL-cholesterol (45·8%, 34·3% to 56·5%; Tables S7 and S8). These increases were higher than the global average (except for ambient particulate matter pollution). Likewise, from 1990 to 2019, dementia-related DALYs attributable to high fasting plasma glucose and high body-mass index increased by 61·0% (52·2% to 77·0%) and 50·5% (31·6% to 90·9%), respectively, which were 2-times the corresponding global PAFs.

From 1990 to 2019, stroke DALYs rate attributable to high fasting plasma glucose decreased globally (-9·8%, 95% UI -17·6% to -0·5%), while it increased in North Africa and Middle East (34·8%, 7·9% to 64·8%) with its highest increases in Egypt and Morocco (supplementary Tables S7 and S8). These changes were even more substantial for ischaemic strokes. Dementia DALY rates attributable to high fasting plasma glucose in the region (59·1%, 47·1% to 88·7%) were almost twice the global estimates (34·0%, 28·0% to 47·7%). There was a statistically significant reduction in the risk-attributed age-standardised DALYs rate related to stroke (-25·8%, -34·1% to -14·0%), Parkinson's disease (-19·0%, -31·8% to -1·7%), and meningitis (-78·5%, -85·6% to -67·8%), while it increased for dementia (19·0%, 6·7% to 37·7%). The highest reduction in the risk was attributable to household air pollution from solid fuels (with almost 50% to 85% reduction) and the two most marked increases were related to high fasting plasma glucose (on average 67·7% increase for ischaemic stroke and 59·1% increase for dementia) and high body-mass index (on average 37·1% increase for ischaemic stroke and 48·7% increase for dementia).

During the same period, among the countries of the North Africa and Middle East region, stroke DALYs rate attributable to ambient particulate matter pollution increased in Sudan (206·9%, 95% UI 59·0% to 596·3%), Yemen (204·1%, 60·2% to 642·4%), Afghanistan (112·6%, 15·3% to 436·2%), and Morocco (93·7%, 24·3% to 264·0%). There were also increasing trends of ischaemic stroke DALYs rates attributable to tobacco in Afghanistan and Egypt; of dementia in Qatar (42·2%, 4·6% to 85·2%) and Egypt (36·1%, 12·0% to 65·5%); of Parkinson's disease in Egypt (36·2%, 0·6% to 80·7%); and of multiple sclerosis in Afghanistan (81·4%, 26·4% to 153·6%), Saudi Arabia (56·6%, 9·9% to 129·6%), and Lebanon (47%, 13·9% to 92·5%). Iran was the only country in the region where stroke DALYs (197·8%, 20·0% to 2297·6%) and idiopathic epilepsy DALYs (204·6%, 90·2% to 478·2%) attributable to alcohol significantly increased.

4. Age- and sex-specific proportions

For neurological deaths in 2019, 60·1% (187·6, 95% UI 167·5–207·4 thousand) of all-type stroke deaths happened in the elderly (aged 70 years and older; Table S10). It was 70·2% (147·4, 130·7–163·4 thousand) for ischaemic stroke, 39·7% (35·2, 30·9–39·6 thousand) for intracerebral haemorrhage, and 36·8% (5·0, 4·0–6·1 thousand) for subarachnoid haemorrhage. Haemorrhagic strokes were almost equally distributed among the age groups 50–69 and

≥70 years. Of all deaths, 90·5% of dementia (63·8, 15·8–169·6 thousand; Table S11) and 82·9% of Parkinson's disease (13·9, 11·9–18·3 thousand; Table S12) was estimated at the elderly.

In 2019, 2058 thousand head injuries and 143 thousand spinal injuries happened among females, 1351 thousand head injuries and 77 thousand spinal injuries among males in NAME. Age- and cause-specific neurological DALYs in North Africa and Middle East among women and men are illustrated in Figure S11.

Figure S11. Age-specific burden of neurological conditions in North Africa and Middle East measured by disability-adjusted life years (DALYs) among (A) males and (B) females.

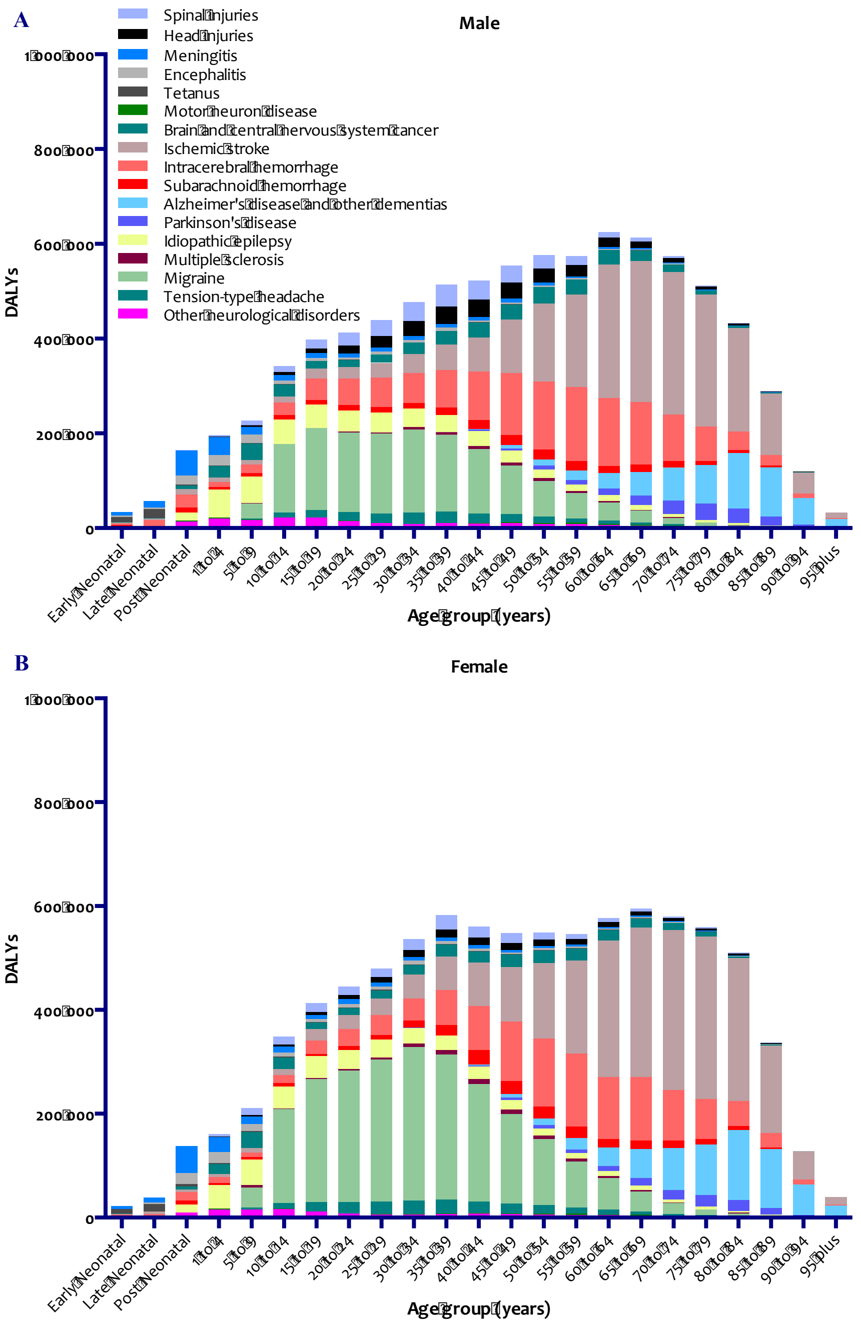

5. Supplementary results tables

Table S10 Global and regional burden, mortality, incidence, and prevalence of neurological conditions in different regions of the world
(NEXT PAGE)

Disability-Adjusted Life Years 106
Deaths 112
Incidence 117
Prevalence 123
Years Lived with Disability 130
Years of Life Lost 136

Table S10 Global and regional burden, mortality, incidence, and prevalence of neurological conditions in different regions of the world

		All Ages		Age-standardised		
		Number (thousand)	Percent change	Rate per 100 000	Percent change	Sex ratio
**Measure**		2019	From 1990 to 2019	2019	From 1990 to 2019	2019
Cause	Region	Mean (95% UI)	Mean (95% UI)	Mean (95% UI)	Mean (95% UI)	F:M
**DALYs (Disability-Adjusted Life Years)**						
Meningitis	Global	16333·2 (13775·1–19609·8)	-51·3% (-59·4 to -42%)	234 (195·8–282·5)	-57·2% (-64·4 to -48·6%)	0·86
	Southeast Asia, East Asia, and Oceania	1117·6 (985·8–1270·5)	-81·9% (-85·2 to -78·3%)	69·5 (60·4–80)	-79·9% (-83·6 to -75·7%)	0·71
	Central Europe, Eastern Europe, and Central Asia	149·1 (134·2–165·2)	-74·8% (-77·4 to -71·9%)	40·1 (35·8–45·1)	-75·2% (-78 to -72%)	0·68
	High-income	157·7 (146·4–169·1)	-64·4% (-66·2 to -62·5%)	16·2 (14·9–17·5)	-72·2% (-74·2 to -70·3%)	0·80
	Latin America and Caribbean	355·6 (295·1–421·5)	-75·2% (-80·2 to -69·8%)	67·5 (55·4–80·6)	-77·7% (-82·2 to -72·5%)	0·73
	North Africa and Middle East	412·8 (345·2–495)	-70·5% (-77·5 to -62·3%)	70 (58·7–83·7)	-75·8% (-81·1 to -69·5%)	0·91
	South Asia	3712·1 (3190·9–4384·3)	-62·8% (-70 to -54·4%)	217·8 (186·5–258·7)	-69·2% (-75·1 to -62·7%)	1·01
	Sub-Saharan Africa	10428·3 (8282·7–12983·1)	-22·8% (-37·9 to -2·2%)	781·5 (640·4–945·5)	-56·6% (-64·2 to -46·3%)	0·80
	World Bank High Income	167·6 (155·1–180·4)	-65·6% (-67·5 to -63·8%)	15·2 (14–16·4)	-73·6% (-75·5 to -71·8%)	0·80
	World Bank Upper Middle Income	941·8 (851·2–1042·1)	-82·2% (-84·9 to -79·2%)	42·3 (37·9–47·3)	-82·6% (-85·4 to -79·6%)	0·74
	World Bank Lower Middle Income	9369·5 (7877·6–11295·6)	-50·1% (-58·6 to -39·2%)	296·9 (250–357·4)	-60% (-66·5 to -51·8%)	0·90
	World Bank Low Income	5844·3 (4689·2–7297·5)	-34·8% (-47·8 to -18·4%)	670·1 (553·9–805·1)	-61·3% (-68·3 to -52·9%)	0·79
Encephalitis	Global	4797·4 (4059·5–6418·1)	-43·4% (-55·8 to -15·8%)	65·3 (55·1–87·3)	-54·3% (-63·9 to -32·9%)	0·87
	Southeast Asia, East Asia, and Oceania	756·8 (611·2–863·9)	-51·4% (-61·9 to -27·4%)	43·9 (34·1–50·7)	-50% (-61 to -30·1%)	0·76
	Central Europe, Eastern Europe, and Central Asia	198·8 (172·4–232)	-27·7% (-36·2 to -8·1%)	55·4 (47·2–66)	-22·8% (-33·1 to -0·2%)	0·79
	High-income	111·5 (95·8–121)	22·2% (5·1 to 31·8%)	10·1 (8·7–11·1)	-4% (-16·2 to 4·5%)	0·87
	Latin America and Caribbean	187·8 (155·6–227·1)	-17·2% (-32·2 to 4%)	34·8 (28·6–42·2)	-29·6% (-42·7 to -12·4%)	0·83
	North Africa and Middle East	251 (200·2–332·9)	13·5% (-21 to 59·6%)	42 (33·6–55·6)	-20·7% (-43·3 to 8·7%)	1·09
	South Asia	2924 (2349·5–4371·6)	-50·4% (-63·8 to -21%)	174·3 (140·4–258·4)	-61·2% (-70·7 to -39·2%)	0·95
	Sub-Saharan Africa	367·5 (294·9–480·1)	77·7% (32·8 to 123·6%)	32·3 (26·6–40·9)	-7·2% (-25·9 to 10·2%)	0·74
	World Bank High Income	129·8 (115·4–141·4)	3·8% (-5·7 to 11·1%)	10·9 (9·8–12·1)	-20·3% (-27·1 to -13·9%)	0·87
	World Bank Upper Middle Income	688·2 (604·3–803·5)	-50·6% (-59·6 to -27·5%)	30·7 (26·8–36·6)	-52·2% (-61·1 to -29·4%)	0·84
	World Bank Lower Middle Income	3641 (3003·3–5085·3)	-45·5% (-58·9 to -16·5%)	120·5 (100·2–167·9)	-58% (-67·2 to -38·1%)	0·89
	World Bank Low Income	336·5 (277·7–419·4)	23% (-13·3 to 104·7%)	46·9 (39·2–55·1)	-29·5% (-46·7 to 6%)	0·87
Tetanus	Global	2316·4 (1770–3279·4)	-89·4% (-92·1 to -83·8%)	33·7 (25·6–47·9)	-90·2% (-92·7 to -85·1%)	0·86
	Southeast Asia, East Asia, and Oceania	222·5 (134·8–275·7)	-93·9% (-96·6 to -91·2%)	11·6 (7·6–14·4)	-94·5% (-96·5 to -92·1%)	0·64
	Central Europe, Eastern Europe, and Central Asia	0·9 (0·6–1·9)	-84·3% (-88 to -68·6%)	0·2 (0·1–0·4)	-84·8% (-88·2 to -70·2%)	0·63
	High-income	1·4 (1·1–3)	-83·8% (-87·1 to -66·5%)	0·1 (0·1–0·2)	-88·9% (-91·1 to -77·3%)	0·67
	Latin America and Caribbean	23·4 (14·7–44·1)	-89·7% (-93·8 to -80·4%)	4·5 (2·7–8·7)	-91·5% (-94·9 to -83·5%)	1·07
	North Africa and Middle East	87·4 (53·6–139·5)	-85·8% (-92·9 to -68·7%)	15 (9·2–24·1)	-88·2% (-93·9 to -76·1%)	0·85
	South Asia	828·7 (567–1157·6)	-94·2% (-96 to -91·2%)	50·7 (34·6–71·1)	-94·3% (-96·2 to -91·5%)	1·00
	Sub-Saharan Africa	1152·1 (809·6–1865·2)	-62·8% (-76·5 to -33·7%)	84·5 (57·7–133·2)	-78·9% (-85·9 to -67·7%)	0·75
	World Bank High Income	3·2 (2–5·2)	-85·9% (-95·3 to -69·1%)	0·2 (0·2–0·4)	-91·3% (-97·3 to -79·1%)	0·60
	World Bank Upper Middle Income	40 (30–62·5)	-97·7% (-98·3 to -96·1%)	1·6 (1·3–2·4)	-98% (-98·5 to -96·7%)	0·50
	World Bank Lower Middle Income	1514·2 (1111·5–2028·3)	-91·1% (-93·8 to -87·3%)	49 (35·9–65·5)	-92·2% (-94·5 to -89·2%)	0·85

Table S10 Global and regional burden, mortality, incidence, and prevalence of neurological conditions in different regions of the world

		All Ages		Age-standardised		
		Number (thousand)	Percent change	Rate per 100 000	Percent change	Sex ratio
**Measure**		2019	From 1990 to 2019	2019	From 1990 to 2019	2019
Cause	Region	Mean (95% UI)	Mean (95% UI)	Mean (95% UI)	Mean (95% UI)	F:M
	World Bank Low Income	757·7 (508·7–1318)	-74·5% (-83·9 to -46·8%)	82·4 (53·6–138·9)	-84·4% (-89·6 to -71·7%)	0·77
Brain and central nervous system cancer	Global	8659·9 (6718–9574·5)	40·5% (-13·2 to 66·9%)	109 (84·6–120·9)	-10·4% (-43·5 to 5·3%)	0·71
	Southeast Asia, East Asia, and Oceania	2672·7 (2000·7–3135·9)	22·9% (-20·6 to 65·7%)	112·3 (84·7–131·7)	-18·5% (-47·3 to 8·2%)	0·77
	Central Europe, Eastern Europe, and Central Asia	823·5 (611·1–936·8)	18·6% (-21 to 36·4%)	168·5 (126·7–191·9)	5·8% (-29·8 to 21·7%)	0·65
	High-income	1738·8 (1250·5–1883·5)	30·9% (-16·5 to 42·2%)	122·6 (90·9–132)	-8·5% (-41 to -1%)	0·66
	Latin America and Caribbean	711·7 (480·3–832·7)	110·6% (3·3 to 175·7%)	121·3 (81·6–142·3)	28·7% (-36·7 to 68·1%)	0·76
	North Africa and Middle East	716·3 (493·9–848·2)	71% (0·9 to 131·7%)	128·3 (87·8–151·3)	-5% (-40 to 23·4%)	0·86
	South Asia	1383·2 (1081·7–1662·4)	47·8% (-13·8 to 119·4%)	81·1 (63·4–97·8)	-1·2% (-38·2 to 34·9%)	0·67
	Sub-Saharan Africa	613·7 (449·4–782·3)	122·6% (19·3 to 259·5%)	62·3 (43·7–76·4)	13·9% (-34·7 to 64·8%)	0·68
	World Bank High Income	1920·9 (1379·9–2092·2)	29·5% (-18·1 to 41·4%)	123·2 (91·5–133·4)	-10·4% (-42·9 to -2·6%)	0·67
	World Bank Upper Middle Income	3722·2 (2788·2–4206)	30·9% (-19·1 to 57·1%)	126·9 (94·8–142·9)	-12% (-45·8 to 5·4%)	0·76
	World Bank Lower Middle Income	2560·3 (1954·2–2955·2)	59·8% (-6 to 114·3%)	85·1 (64·7–98·2)	0·7% (-36·7 to 27·2%)	0·71
	World Bank Low Income	452·1 (330·3–578·7)	93·9% (7·7 to 217·5%)	70 (50·4–87·8)	3·6% (-38·4 to 51·6%)	0·70
Stroke	Global	143232·2 (133095·8–153241·8)	32·4% (22 to 42·2%)	1768·1 (1640·6–1889·4)	-35·2% (-40·5 to -30·5%)	0·76
	Southeast Asia, East Asia, and Oceania	66050 (59613·2–72976·5)	48·3% (29 to 69·3%)	2563·5 (2312·5–2827·2)	-35·8% (-44·5 to -27%)	0·66
	Central Europe, Eastern Europe, and Central Asia	14351·9 (13236·4–15424·1)	-8·9% (-14·7 to -3%)	2327·5 (2144–2500·4)	-32·4% (-36·6 to -28%)	0·70
	High-income	13703·4 (12355·7–14781·7)	-16·4% (-20·3 to -12·9%)	653·2 (596·1–704·9)	-52·9% (-54·8 to -50·9%)	0·84
	Latin America and Caribbean	6196·6 (5710·4–6688·8)	22·2% (13·5 to 31·4%)	1060 (976·7–1144·7)	-50·3% (-53·8 to -46·7%)	0·83
	North Africa and Middle East	7946 (7060·2–8870·8)	43·3% (27·2 to 61·4%)	1826·2 (1635·3–2026·2)	-32% (-39·1 to -23·3%)	1·04
	South Asia	24119·3 (21416·4–26859·9)	70·4% (47·3 to 95·6%)	1702·6 (1510·9–1891)	-30·6% (-40·6 to -19·8%)	0·87
	Sub-Saharan Africa	10865 (9479·7–12395·1)	61·2% (40·8 to 84·4%)	2216·8 (1955·9–2495·9)	-22·8% (-31·8 to -12·2%)	0·90
	World Bank High Income	15753·7 (14247·6–17024)	-16% (-19·9 to -12·5%)	705·1 (643·6–762·9)	-52·4% (-54·5 to -50·4%)	0·83
	World Bank Upper Middle Income	67195·7 (60574·6–73873·7)	27·6% (12·4 to 44·2%)	2067·2 (1863·6–2269·6)	-42% (-48·7 to -34·9%)	0·68
	World Bank Lower Middle Income	51615 (47355·1–55856·7)	64·6% (48·8 to 79·5%)	2177·1 (1999·5–2348·3)	-25·6% (-33·1 to -17·8%)	0·85
	World Bank Low Income	8585·9 (7346·5–9955·9)	59·2% (39·1 to 81·7%)	2591·6 (2230·8–2981·7)	-21·8% (-30·5 to -12%)	0·90
Ischaemic stroke	Global	63478·3 (57827·4–68986·5)	56·7% (43·3 to 67·9%)	798·8 (727·5–866·9)	-28·5% (-34·7 to -23·2%)	0·83
	Southeast Asia, East Asia, and Oceania	28355·4 (25100·2–31773·5)	138·8% (95·3 to 172·6%)	1144 (1016·4–1276·1)	-3·2% (-21·4 to 10%)	0·73
	Central Europe, Eastern Europe, and Central Asia	9122·6 (8355–9868·2)	-9·3% (-16·1 to -2·8%)	1444·2 (1321·2–1560·7)	-35·2% (-39·8 to -30·7%)	0·78
	High-income	7774·2 (6824·5–8572·7)	-18·2% (-22·8 to -14·2%)	334·7 (295–371·2)	-56·9% (-59·4 to -54·6%)	0·88
	Latin America and Caribbean	2582·8 (2358·5–2802·4)	39% (27·9 to 51·2%)	458 (417·2–496·6)	-49·4% (-53·4 to -45%)	0·80
	North Africa and Middle East	4751·4 (4227·7–5271·9)	120% (89 to 148·1%)	1183·6 (1060·8–1307)	-8·8% (-19·6 to 2·2%)	1·09
	South Asia	7745·9 (6612–9077·2)	122·3% (83·9 to 159·8%)	605·3 (521·1–706·7)	-19·4% (-34·1 to -4·1%)	0·84
	Sub-Saharan Africa	3145·9 (2777·4–3620·1)	102·3% (77·8 to 126%)	766·5 (680·9–868·6)	-3·8% (-16·1 to 7·4%)	1·08
	World Bank High Income	9137·4 (8085·3–10026)	-17·2% (-21·7 to -13·1%)	374·6 (332–414)	-55·8% (-58·2 to -53·5%)	0·88

Table S10 Global and regional burden, mortality, incidence, and prevalence of neurological conditions in different regions of the world

		All Ages		Age-standardised		
		Number (thousand)	Percent change	Rate per 100 000	Percent change	Sex ratio
**Measure**		2019	From 1990 to 2019	2019	From 1990 to 2019	2019
Cause	Region	Mean (95% UI)	Mean (95% UI)	Mean (95% UI)	Mean (95% UI)	F:M
	World Bank Upper Middle Income	33246 (29880·2–36557·8)	75·2% (53·8 to 94·2%)	1044·2 (934·2–1144·9)	-26% (-35·1 to -18·2%)	0·77
	World Bank Lower Middle Income	18608·3 (16613–20667·5)	100·4% (76·5 to 120·9%)	876 (784·4–965·2)	-16·9% (-26·7 to -6·5%)	0·90
	World Bank Low Income	2455·7 (2117·3–2893·7)	106·7% (83·9 to 130·6%)	863·8 (740–1008·7)	-2·3% (-12·7 to 8·5%)	1·01
Intracerebral haemorrhage	Global	68572·5 (63272·3–73682)	25·3% (12·2 to 36·3%)	832·8 (769·2–894·7)	-36·7% (-43·3 to -31%)	0·68
	Southeast Asia, East Asia, and Oceania	34034·3 (30443·4–37734·8)	30·7% (10·8 to 50·5%)	1280 (1147·9–1417)	-43·2% (-52·3 to -34·5%)	0·59
	Central Europe, Eastern Europe, and Central Asia	4223 (3867·2–4548·8)	-11·4% (-19·2 to -3·7%)	705·2 (645·6–760·3)	-30·2% (-36·4 to -24·2%)	0·57
	High-income	4061 (3756·2–4310·9)	-20·3% (-24·6 to -16·2%)	208·3 (196·2–220·4)	-53·1% (-55·1 to -50·9%)	0·65
	Latin America and Caribbean	2611·3 (2385·7–2852)	4·4% (-5 to 14·8%)	437 (399·1–477)	-55·9% (-59·8 to -51·5%)	0·73
	North Africa and Middle East	2702·2 (2343·7–3120·3)	3·4% (-12·3 to 21·4%)	548·4 (479·5–623·1)	-51·7% (-58·2 to -43·8%)	0·93
	South Asia	13740·6 (11774·7–15890·6)	55·7% (31·8 to 79·7%)	931·4 (798·1–1075·7)	-35·4% (-45·5 to -25·1%)	0·88
	Sub-Saharan Africa	7200·1 (6126·6–8347)	47·7% (27 to 71·9%)	1372·6 (1177·7–1584·4)	-30·4% (-39·5 to -20·2%)	0·82
	World Bank High Income	4627 (4293·3–4918·2)	-20·6% (-24·9 to -16·8%)	223·3 (209·7–237·3)	-52·6% (-54·9 to -50·3%)	0·64
	World Bank Upper Middle Income	29543·7 (26176–33096·9)	12·3% (-5·2 to 30·2%)	888·3 (787·4–993·2)	-47·7% (-55·7 to -39·7%)	0·56
	World Bank Lower Middle Income	28752·2 (25833·6–31579·3)	53·7% (36·5 to 69·9%)	1144·8 (1029–1257·6)	-29·5% (-37·8 to -21·5%)	0·81
	World Bank Low Income	5606·1 (4742·9–6615·2)	45·4% (25·3 to 69·2%)	1601·9 (1366–1878·7)	-28·8% (-37·6 to -18·5%)	0·85
Subarachnoid haemorrhage	Global	11181·4 (9893·4–12668·9)	-13·8% (-25·7 to 17%)	136·5 (120·8–154·7)	-54·2% (-60·6 to -37·5%)	0·89
	Southeast Asia, East Asia, and Oceania	3660·2 (3083·4–4334·7)	-44·8% (-58·7 to -6·2%)	139·5 (117·9–164·7)	-74·8% (-81·2 to -56·6%)	0·71
	Central Europe, Eastern Europe, and Central Asia	1006·3 (909·5–1111·8)	9·1% (-3·4 to 23·6%)	178·1 (160·9–198·3)	-11·2% (-21·5 to 1%)	0·73
	High-income	1868·1 (1710·2–2025·1)	4·5% (-0·9 to 12%)	110·2 (101·7–118·9)	-33·6% (-37 to -28·4%)	1·16
	Latin America and Caribbean	1002·6 (911–1107·4)	40·3% (25 to 57·8%)	165·1 (149·8–182·5)	-30·6% (-38·3 to -21·8%)	1·29
	North Africa and Middle East	492·4 (408·3–617·2)	-36% (-50·6 to 6·8%)	94·2 (79–116·8)	-62·8% (-71·6 to -41%)	1·08
	South Asia	2632·9 (1703–3567·1)	42·6% (11·4 to 136·3%)	165·9 (106·3–224·5)	-36·6% (-51·2 to 5·5%)	0·94
	Sub-Saharan Africa	519 (315·8–1049)	67·6% (27·6 to 133·8%)	77·7 (45·3–155·7)	-24·5% (-37·9 to 1·6%)	0·62
	World Bank High Income	1989·3 (1819·3–2157·5)	4·6% (-0·7 to 12%)	107·2 (98·8–115·6)	-34·2% (-37·4 to -29%)	1·16
	World Bank Upper Middle Income	4406 (3829·6–5013·8)	-40·2% (-52·7 to -5·7%)	134·7 (117·2–152·9)	-70·4% (-76·6 to -52·7%)	0·81
	World Bank Lower Middle Income	4254·5 (3188·4–5488·9)	26·8% (3 to 88·7%)	156·3 (117–200·7)	-36·9% (-49·9 to -6%)	0·89
	World Bank Low Income	524·1 (302·8–1007·1)	50·3% (18·1 to 106·3%)	125·9 (71·8–238·4)	-30% (-42·5 to -7·8%)	0·79
Neurological disorders*	Global	97724·4 (55942·8–159416·8)	69·9% (58·7 to 90%)	1253·6 (719·7–2039·8)	-0·8% (-5·1 to 3·1%)	1·22
	Southeast Asia, East Asia, and Oceania	25859·8 (13866·4–43640·3)	70·2% (53·2 to 100·8%)	1134 (609·5–1887·4)	0·4% (-7·4 to 6·9%)	1·24
	Central Europe, Eastern Europe, and Central Asia	6580·3 (4025–10385·4)	21·4% (11·9 to 35·5%)	1301·9 (775·1–2061·4)	0·7% (-2·8 to 4·4%)	1·24
	High-income	21294·8 (12967·1–33532)	58·2% (40·1 to 84·6%)	1315·6 (731·2–2146·3)	1·1% (-1 to 3·8%)	1·37
	Latin America and Caribbean	7896·1 (4420·2–12972)	78·9% (62 to 107·7%)	1352·4 (767·1–2203·8)	-2·3% (-8·6 to 2·8%)	1·24
	North Africa and Middle East	7156 (3769·7–12184·4)	91·1% (72 to 109·7%)	1382·1 (776·6–2273·8)	-3·9% (-10·3 to 3·5%)	1·25

Table S10 Global and regional burden, mortality, incidence, and prevalence of neurological conditions in different regions of the world

		**All Ages**		**Age-standardised**		
		Number (thousand)	Percent change	Rate per 100 000	Percent change	Sex ratio
**Measure**		2019	From 1990 to 2019	2019	From 1990 to 2019	2019
Cause	Region	Mean (95% UI)	Mean (95% UI)	Mean (95% UI)	Mean (95% UI)	F:M
	South Asia	19068·9 (9725·7–32172·8)	77% (52·9 to 102·6%)	1189·8 (648·5–1975·5)	-4·3% (-13·7 to 5%)	1·20
	Sub-Saharan Africa	9868·5 (5609·8–16119·2)	119·2% (98·1 to 140%)	1283·3 (772·9–2013·8)	0·7% (-5·8 to 8·1%)	1·09
	World Bank High Income	23193·6 (14090–36738·4)	58·2% (41 to 83·7%)	1319·7 (738·4–2145·1)	0·9% (-1·4 to 3·6%)	1·35
	World Bank Upper Middle Income	34291·3 (19343·5–55999·5)	63·3% (47·8 to 90·1%)	1199·5 (670·6–1953·1)	-0·8% (-7·9 to 4·8%)	1·23
	World Bank Lower Middle Income	34025·6 (17416·8–57071·1)	80·5% (64·3 to 99·3%)	1240·6 (681–2047·5)	-2·1% (-8·4 to 5%)	1·20
	World Bank Low Income	6158·7 (3509·2–10018·6)	107·8% (81·8 to 131·2%)	1226·8 (724·6–1939·7)	-1·9% (-9·4 to 4·9%)	1·12
Alzheimer's disease and other dementias	Global	25277 (11204·5–54558·2)	161·6% (149·7 to 175·7%)	338·6 (151–731·3)	3·7% (-0·3 to 8·1%)	1·19
	Southeast Asia, East Asia, and Oceania	7765·2 (3410·6–17185·4)	211·5% (178 to 254·4%)	361·2 (159·6–784·8)	5·3% (-5·7 to 18·7%)	1·26
	Central Europe, Eastern Europe, and Central Asia	2092·9 (961·1–4507)	71·9% (63·5 to 81·4%)	335 (154·1–720·4)	4·3% (0 to 8·7%)	1·11
	High-income	8678·2 (4036·8–17955·9)	127·3% (117·1 to 137·5%)	329·5 (153·9–685·4)	2·4% (-1·4 to 5·6%)	1·20
	Latin America and Caribbean	1922 (821·6–4193·6)	238·7% (219·9 to 260·6%)	351·8 (150·7–765·5)	2·7% (-2·4 to 8·7%)	1·05
	North Africa and Middle East	1208·1 (532–2672·8)	177·3% (161·1 to 215·8%)	387 (172–848·5)	-1·1% (-6·6 to 12·1%)	1·12
	South Asia	2617·6 (1061–6225·2)	260·7% (225 to 300·6%)	262·1 (105·6–617·4)	10% (0·1 to 21·5%)	1·06
	Sub-Saharan Africa	993·1 (406·6–2267·4)	144·1% (128·4 to 161%)	344·5 (140·3–797·7)	8·5% (2·4 to 15·5%)	1·21
	World Bank High Income	9244·2 (4299·4–19139·6)	126·6% (116·4 to 136·8%)	331·6 (154·5–692·5)	2·2% (-1·4 to 5·4%)	1·20
	World Bank Upper Middle Income	10365·3 (4584·1–22624)	194·9% (170 to 224·2%)	359·6 (160–781·7)	4·7% (-2·9 to 13·7%)	1·17
	World Bank Lower Middle Income	4955·3 (2039·9–11483·4)	178·1% (161 to 202·7%)	298·1 (122–689·3)	3·2% (-2·9 to 12·9%)	1·13
	World Bank Low Income	699·5 (291–1608·7)	148·7% (132·3 to 167·6%)	348·6 (144·7–801·4)	5·6% (-0·8 to 13%)	1·21
Parkinson's disease	Global	6292·6 (5769·2–6827·2)	128·9% (113·3 to 142·5%)	80 (73·3–86·6)	2·4% (-4·4 to 8·2%)	0·56
	Southeast Asia, East Asia, and Oceania	2044·9 (1794–2305·7)	141·2% (111·1 to 172·6%)	83·8 (73·7–94·1)	-8·6% (-19·7 to 2·6%)	0·56
	Central Europe, Eastern Europe, and Central Asia	467·2 (426·9–508·2)	50·2% (41·5 to 60·3%)	72·7 (66·4–79·1)	4·9% (-0·9 to 12·1%)	0·57
	High-income	1822·1 (1648·7–1954·3)	107·3% (97·9 to 113·9%)	76·5 (69·5–82·3)	9·8% (5·9 to 12·7%)	0·49
	Latin America and Caribbean	412·5 (372·9–452·1)	208·4% (188·6 to 230·4%)	74·3 (67·2–81·4)	5·3% (-1·4 to 12·7%)	0·59
	North Africa and Middle East	300·7 (266·3–365·4)	163·8% (134·8 to 202·1%)	84·4 (74·7–103·2)	0·9% (-10·2 to 15·7%)	0·68
	South Asia	984·6 (854·6–1124·9)	182·8% (135·4 to 231·5%)	82·1 (71–94)	-2·9% (-18·5 to 14·2%)	0·67
	Sub-Saharan Africa	260·7 (231·4–300·6)	125·1% (95·6 to 155·2%)	75·6 (67–88·2)	7·2% (-6·3 to 20·9%)	0·66
	World Bank High Income	1942·4 (1761·3–2087·9)	106% (96·9 to 112·3%)	76·6 (69·7–82·5)	9·2% (5·5 to 12·2%)	0·49
	World Bank Upper Middle Income	2509·8 (2240·6–2799·6)	132·6% (108·6 to 155·9%)	79·5 (70·8–88·5)	-5·9% (-15·3 to 3·1%)	0·56
	World Bank Lower Middle Income	1646·2 (1483–1820·6)	159·4% (125·5 to 189·4%)	83·4 (75·1–92)	5·2% (-8·5 to 17%)	0·66
	World Bank Low Income	190·8 (165·6–225·5)	108·8% (83·6 to 134·4%)	76·9 (67–91·6)	-2·4% (-13·5 to 8·9%)	0·64
Idiopathic epilepsy	Global	13077·6 (9986·7–16734·1)	15·9% (0 to 42·1%)	170·6 (130·4–218·3)	-16·5% (-27·4 to 1·7%)	0·82
	Southeast Asia, East Asia, and Oceania	2259·6 (1627·7–3002·3)	-10·6% (-28·5 to 14·2%)	108 (77·8–144·4)	-25·1% (-40·5 to -4·7%)	0·79

Table S10 Global and regional burden, mortality, incidence, and prevalence of neurological conditions in different regions of the world

**Measure**		**All Ages**		**Age-standardised**		
		Number (thousand)	Percent change	Rate per 100 000	Percent change	Sex ratio
		2019	From 1990 to 2019	2019	From 1990 to 2019	2019
Cause	Region	Mean (95% UI)	Mean (95% UI)	Mean (95% UI)	Mean (95% UI)	F:M
	Central Europe, Eastern Europe, and Central Asia	704·5 (523·2–930·3)	-5·8% (-19·5 to 9·3%)	170·4 (127·1–227)	-5·2% (-19 to 9·8%)	0·73
	High-income	1295·1 (894·3–1900·4)	15% (-3·8 to 35·3%)	112·5 (76·5–165·9)	-8·4% (-23·4 to 7·7%)	0·86
	Latin America and Caribbean	1287 (944·9–1721·7)	13·1% (-6·2 to 38·4%)	219·9 (161·2–295·2)	-23·9% (-36·9 to -7·6%)	0·84
	North Africa and Middle East	955·3 (682·8–1293·2)	13·8% (-13·8 to 56%)	158·3 (112·6–213·2)	-26·2% (-43·6 to -1·1%)	0·87
	South Asia	3697·9 (2884·5–4712·2)	9·7% (-13·9 to 54·7%)	207·4 (162·1–263·8)	-29·3% (-43·7 to -3·6%)	0·98
	Sub-Saharan Africa	2878·2 (2129·4–3822·1)	87·2% (54·3 to 144·2%)	270·1 (203·1–355·3)	-11·8% (-26·2 to 10·6%)	0·64
	World Bank High Income	1538·6 (1059·2–2235·1)	17·5% (0·1 to 36·8%)	121·2 (82·2–176·2)	-6·9% (-20·8 to 8·6%)	0·83
	World Bank Upper Middle Income	3549 (2603·4–4672·1)	-10·8% (-26·2 to 9%)	137·7 (100·7–182·1)	-25·6% (-38·2 to -9·6%)	0·81
	World Bank Lower Middle Income	6201·9 (4795·7–7893·3)	25·5% (3·2 to 68·4%)	196·3 (151·4–250·3)	-20% (-33·3 to 4·5%)	0·86
	World Bank Low Income	1779·9 (1288·5–2380·3)	69·1% (34·9 to 130·5%)	249·5 (183·5–329·2)	-16·6% (-32·1 to 8·6%)	0·70
Multiple sclerosis	Global	1159·8 (1001·2–1381·9)	59·7% (46·6 to 72·7%)	14 (12–16·6)	-13·2% (-20·9 to -6·3%)	1·57
	Southeast Asia, East Asia, and Oceania	108 (91·1–139·8)	46·5% (14·5 to 110·9%)	4 (3·3–5·1)	-21% (-37·7 to 12·5%)	0·98
	Central Europe, Eastern Europe, and Central Asia	132·8 (99·3–214·1)	-7% (-23 to 29·4%)	25·4 (18·9–41·2)	-19·9% (-34 to 12·6%)	1·31
	High-income	550 (451·1–647·2)	52·9% (24·1 to 63·2%)	36·5 (30–43·3)	7·8% (-10·4 to 14·1%)	1·85
	Latin America and Caribbean	59·3 (50–72)	158·7% (108·2 to 190%)	9·6 (8·1–11·7)	19·4% (-4·7 to 33·9%)	1·44
	North Africa and Middle East	115·9 (93·1–144·8)	146% (98·9 to 205·5%)	19·9 (16·1–24·7)	6·2% (-11·6 to 30·5%)	1·58
	South Asia	144·1 (119·7–177·5)	134·3% (86·2 to 210·3%)	8·6 (7·1–10·5)	10·3% (-11·7 to 47·5%)	1·42
	Sub-Saharan Africa	49·8 (40·8–62·4)	172·1% (107·2 to 239·4%)	7·2 (6–9·1)	14·9% (-10·8 to 44·2%)	1·69
	World Bank High Income	591·2 (490·1–695·4)	48·2% (21·8 to 58%)	35·5 (29·4–42·3)	2·5% (-13·8 to 9·7%)	1·84
	World Bank Upper Middle Income	271·4 (226·1–346·5)	46·4% (27·6 to 75·8%)	8·3 (6·9–10·5)	-17·5% (-28·1 to -2%)	1·30
	World Bank Lower Middle Income	262·3 (223·2–319·6)	106% (74 to 152·7%)	9 (7·7–11)	-0·2% (-15 to 21·7%)	1·38
	World Bank Low Income	34·2 (26·2–44·7)	145·8% (90·1 to 196·7%)	7·4 (5·6–9·6)	7·5% (-15 to 29·6%)	1·58
Migraine	Global	42077·7 (6418·4–95645·2)	56·6% (52·6 to 62·1%)	525·5 (78·8–1194)	1·5% (-4·4 to 3·3%)	1·70
	Southeast Asia, East Asia, and Oceania	11650·2 (1650·6–27010·1)	49·5% (41·1 to 59·8%)	488·1 (64·8–1135)	6·7% (-3 to 10·5%)	1·65
	Central Europe, Eastern Europe, and Central Asia	2468·6 (656·6–5214·9)	4·1% (1·7 to 9·5%)	541·2 (131·6–1161·6)	-0·5% (-2·7 to 0·3%)	1·86
	High-income	6617·2 (1167·8–14754·8)	17·2% (13·8 to 28%)	587·6 (89·5–1321·7)	0·6% (-1 to 2·2%)	2·06
	Latin America and Caribbean	3388·3 (455·7–7790·2)	63·7% (56·7 to 91·3%)	557·2 (74·1–1281·5)	2·2% (-0·1 to 4·2%)	1·94
	North Africa and Middle East	3793·2 (645·3–8665·8)	102·1% (93·2 to 125·1%)	601·4 (107–1371·8)	0% (-1·6 to 1·6%)	1·70
	South Asia	9779·9 (1150·3–22319·5)	86·3% (79·3 to 99·4%)	526·3 (66·1–1196·4)	0·6% (-2·2 to 3·6%)	1·54
	Sub-Saharan Africa	4380·3 (668·4–9895·8)	135·7% (132·2 to 138·9%)	461 (81·5–1033·8)	1·2% (-0·2 to 2·3%)	1·54
	World Bank High Income	7368·2 (1321·7–16430·4)	19·6% (16 to 30·6%)	584·6 (90·8–1312·1)	0·5% (-1·4 to 2·2%)	2·02
	World Bank Upper Middle Income	14488 (2455·8–32795·2)	44·8% (37·8 to 53%)	503·1 (78·6–1148·7)	5·7% (-5·5 to 9·7%)	1·75
	World Bank Lower Middle Income	17514·6 (2229·5–40394·2)	84·1% (78·8 to 93·5%)	544 (74·1–1244·9)	0·1% (-2·3 to 1·8%)	1·57
	World Bank Low Income	2682·5 (416·6–6084·3)	130·4% (126·5 to 133·4%)	432·4 (77·5–968·1)	1·4% (0 to 2·4%)	1·58

Table S10 Global and regional burden, mortality, incidence, and prevalence of neurological conditions in different regions of the world

		All Ages		Age-standardised		
		Number (thousand)	Percent change	Rate per 100 000	Percent change	Sex ratio
**Measure**		2019	From 1990 to 2019	2019	From 1990 to 2019	2019
Cause	Region	Mean (95% UI)	Mean (95% UI)	Mean (95% UI)	Mean (95% UI)	F:M
Tension-type headache	Global	4541·7 (1395·5–14981·3)	57·8% (45·1 to 65·9%)	56·2 (17–188·5)	-2·5% (-5·4 to 1·1%)	1·25
	Southeast Asia, East Asia, and Oceania	1126·6 (354·3–3731·9)	52·5% (26·4 to 74·1%)	46 (13·8–161·6)	1·7% (-5·8 to 14·3%)	1·10
	Central Europe, Eastern Europe, and Central Asia	411·7 (145·4–1182·2)	6·7% (-0·7 to 12·1%)	85·9 (28·8–257·4)	-1·4% (-4 to 1·5%)	1·43
	High-income	846·4 (251·2–2751)	23·9% (12·5 to 31·8%)	68·7 (19·2–230·1)	0% (-4·6 to 2·6%)	1·36
	Latin America and Caribbean	331·7 (95·8–1195·2)	76·7% (50·4 to 99·5%)	54·3 (15·6–196·1)	0·3% (-4·4 to 4·2%)	1·33
	North Africa and Middle East	416·6 (138·3–1196·8)	115·5% (84·9 to 132·3%)	68·1 (22·8–195·5)	1% (-9·5 to 8·7%)	1·28
	South Asia	930·2 (251·8–3573·8)	92·1% (70·8 to 107·2%)	51·7 (14·3–193·5)	0·5% (-6·1 to 7·5%)	1·21
	Sub-Saharan Africa	478·5 (142·5–1776·1)	133·2% (122·4 to 143·2%)	55 (17·7–184·3)	0·2% (-4·5 to 4·7%)	1·27
	World Bank High Income	942·1 (286·1–3013·9)	25·8% (13·1 to 34·1%)	68·5 (19·6–226·6)	-0·5% (-5·8 to 2%)	1·36
	World Bank Upper Middle Income	1594·4 (533·9–4790·4)	46·5% (25·3 to 59·9%)	53·3 (16·9–170)	-1·4% (-7·6 to 7·4%)	1·21
	World Bank Lower Middle Income	1706·7 (486·1–6242·3)	87·7% (70·5 to 98·5%)	54·9 (15·9–191·2)	-1·1% (-5·7 to 4·1%)	1·23
	World Bank Low Income	296·1 (87·9–1098·6)	127·2% (116·2 to 137·7%)	51·6 (16·5–173·5)	0·4% (-4·5 to 5%)	1·27
Motor neuron disease	Global	1034·6 (979·9–1085·4)	65·7% (55·5 to 77·7%)	12·7 (12–13·3)	-4·5% (-10·1 to 1·9%)	0·71
	Southeast Asia, East Asia, and Oceania	134 (117·9–150·6)	-18·2% (-31·5 to -3·9%)	5·5 (4·9–6·1)	-45% (-53·5 to -35·5%)	0·61
	Central Europe, Eastern Europe, and Central Asia	54·9 (50·1–59·8)	83·9% (58·5 to 108%)	10·1 (9·3–11)	45·2% (26·4 to 63·3%)	0·69
	High-income	629·5 (594–658·1)	87·8% (79·1 to 95·6%)	36·8 (35–38·5)	12·9% (8 to 17·7%)	0·70
	Latin America and Caribbean	89·3 (79·3–98·7)	222·3% (180·5 to 262·9%)	14·9 (13·2–16·5)	69·4% (47·3 to 90%)	0·69
	North Africa and Middle East	41·6 (33·8–50·6)	22·5% (-28·9 to 88·7%)	7·8 (6·4–9·5)	-16·6% (-44·8 to 17·9%)	0·73
	South Asia	72·6 (58–88·7)	168·8% (121 to 219·5%)	4·5 (3·6–5·5)	45·8% (18·1 to 78·8%)	0·81
	Sub-Saharan Africa	12·6 (10·5–15)	89·4% (52·8 to 127·7%)	1·6 (1·3–1·9)	-16·3% (-34·8 to 5·1%)	1·08
	World Bank High Income	646·1 (609·2–676·3)	86·7% (77·8 to 94·6%)	34·8 (33–36·5)	11·9% (7 to 16·9%)	0·71
	World Bank Upper Middle Income	261 (238·7–284·1)	17·4% (1 to 36·1%)	8·6 (7·8–9·3)	-22·3% (-32·7 to -10·3%)	0·66
	World Bank Lower Middle Income	116·1 (101·3–133·1)	138·5% (104·4 to 173·1%)	4·1 (3·6–4·7)	30·3% (9·4 to 52·3%)	0·78
	World Bank Low Income	10·8 (9–13·1)	56% (18·1 to 92·3%)	2 (1·7–2·5)	-14·4% (-32·1 to 3·5%)	1·02
Other neurological disorders	Global	4263·4 (3458·9–5174·1)	56·5% (41·8 to 74·3%)	55·9 (45·2–68·3)	10·8% (0·4 to 22·9%)	0·83
	Southeast Asia, East Asia, and Oceania	771·2 (613·5–948·2)	37·9% (17·7 to 61·6%)	37·5 (29–47·1)	13% (-3·8 to 33·7%)	0·77
	Central Europe, Eastern Europe, and Central Asia	247·8 (206–299·3)	14·5% (3·3 to 26·7%)	61·1 (49·6–75·2)	14·2% (1·4 to 28·1%)	0·74
	High-income	856·3 (762·7–976)	38·3% (32 to 45·5%)	67·6 (58·1–80·6)	0·3% (-6·3 to 9%)	0·78
	Latin America and Caribbean	406·1 (325–507·4)	52·8% (34·1 to 76·6%)	70·4 (55·9–88·4)	5·9% (-6·8 to 21·2%)	0·81
	North Africa and Middle East	324·7 (250·4–417·3)	59·4% (26·5 to 98·9%)	55·2 (43–70·4)	6·3% (-13·5 to 30·6%)	0·76
	South Asia	842·1 (623·2–1115·9)	66·4% (40 to 120·6%)	47·1 (35–62)	13·2% (-4·9 to 44·2%)	0·96
	Sub-Saharan Africa	815·2 (589·6–1087·3)	130% (85·4 to 183·9%)	68·2 (51·6–88·8)	12·1% (-7·3 to 35·1%)	0·88
	World Bank High Income	921 (812·7–1057·4)	36·6% (30 to 43·9%)	66·9 (56·8–80)	-0·5% (-7·7 to 8·8%)	0·78
	World Bank Upper Middle Income	1252·4 (1012·2–1518)	34·8% (19·5 to 52·8%)	49·5 (39·2–61·1)	11·8% (-1·8 to 27·9%)	0·78
	World Bank Lower Middle Income	1622·4 (1227·5–2090·3)	80·9% (55·6 to 120·6%)	50·8 (38·9–64·4)	19·6% (2·7 to 43·6%)	0·90

Table S10 Global and regional burden, mortality, incidence, and prevalence of neurological conditions in different regions of the world

		All Ages		Age-standardised		
		Number (thousand)	Percent change	Rate per 100 000	Percent change	Sex ratio
**Measure**		2019	From 1990 to 2019	2019	From 1990 to 2019	2019
Cause	Region	Mean (95% UI)	Mean (95% UI)	Mean (95% UI)	Mean (95% UI)	F:M
	World Bank Low Income	464·8 (332·6–633·8)	108·1% (65·6 to 166·2%)	58·3 (43–78·1)	6·7% (-12·6 to 33·3%)	0·86
Headache disorders	Global	46619·4 (9772·9–100161·7)	56·7% (52·4 to 62·1%)	581·8 (119·6–1255·6)	1·1% (-4·2 to 2·9%)	1·65
	Southeast Asia, East Asia, and Oceania	12776·9 (2520·4–28023·2)	49·8% (40·9 to 60%)	534·1 (98·6–1189·2)	6·3% (-2·7 to 10·1%)	1·59
	Central Europe, Eastern Europe, and Central Asia	2880·3 (987·5–5766·8)	4·5% (2 to 9·3%)	627·1 (196–1287·8)	-0·6% (-2·6 to 0·3%)	1·79
	High-income	7463·5 (1712·2–15883)	18% (14·5 to 27·9%)	656·3 (135·9–1424·6)	0·5% (-1·3 to 2%)	1·97
	Latin America and Caribbean	3720 (683–8205·9)	64·8% (57·2 to 90·5%)	611·5 (111·4–1348·8)	2·1% (-0·4 to 4·1%)	1·88
	North Africa and Middle East	4209·8 (990·1–9068·3)	103·3% (94·5 to 123·7%)	669·6 (159·1–1431·3)	0·1% (-2·4 to 2·4%)	1·65
	South Asia	10710 (1765·8–23536·8)	86·8% (79·5 to 99·8%)	578 (100·5–1258·7)	0·6% (-2·4 to 3·7%)	1·51
	Sub-Saharan Africa	4858·8 (1002·7–10481·4)	135·4% (130·5 to 138·7%)	516·1 (123·2–1089·1)	1·1% (-0·8 to 2·4%)	1·51
	World Bank High Income	8310·2 (1933–17641·9)	20·2% (16·7 to 30·3%)	653·1 (138·2–1410·8)	0·4% (-1·6 to 2%)	1·94
	World Bank Upper Middle Income	16082·4 (3701·8–34346)	44·9% (37·6 to 52·8%)	556·4 (118·8–1199·2)	4·9% (-5·3 to 9·1%)	1·69
	World Bank Lower Middle Income	19221·3 (3425–41915·3)	84·4% (78·7 to 93·6%)	598·8 (112·2–1302·8)	0% (-2·5 to 1·7%)	1·54
	World Bank Low Income	2978·5 (625·3–6424)	130·1% (125 to 133·4%)	484·1 (115·9–1022·8)	1·3% (-0·8 to 2·6%)	1·54
**Deaths**						
Meningitis	Global	236·2 (204·4–277·4)	-45·4% (-53·5 to -35·8%)	3·3 (2·8–3·9)	-56% (-62·5 to -48·3%)	0·84
	Southeast Asia, East Asia, and Oceania	18·2 (16·3–20·5)	-76·3% (-80·3 to -71·7%)	1 (0·9–1·2)	-77·4% (-81·2 to -73·2%)	0·68
	Central Europe, Eastern Europe, and Central Asia	2·7 (2·4–3)	-67% (-70·3 to -63·3%)	0·6 (0·6–0·7)	-71·4% (-74·5 to -67·9%)	0·59
	High-income	3·8 (3·6–4)	-50·3% (-52·8 to -48·2%)	0·3 (0·3–0·3)	-68·9% (-70·7 to -67·3%)	0·75
	Latin America and Caribbean	5·6 (4·7–6·5)	-69·2% (-74·8 to -63%)	1 (0·9–1·2)	-75·2% (-79·7 to -70·3%)	0·69
	North Africa and Middle East	6·3 (5·3–7·4)	-64·1% (-72·2 to -54·5%)	1·2 (1–1·4)	-71·6% (-77·1 to -64·9%)	0·88
	South Asia	55·5 (48·4–64·4)	-57·5% (-65 to -49%)	3·5 (3–4)	-69% (-74 to -63·3%)	1·02
	Sub-Saharan Africa	144·1 (117·3–175·5)	-17% (-32·2 to 3·7%)	14·3 (12·2–16·7)	-51·3% (-58·6 to -42%)	0·81
	World Bank High Income	4·1 (3·8–4·3)	-52·7% (-55 to -50·5%)	0·3 (0·2–0·3)	-70·5% (-72·3 to -68·9%)	0·73
	World Bank Upper Middle Income	18·1 (16·5–19·8)	-73·3% (-77 to -69·3%)	0·7 (0·6–0·8)	-78·3% (-81·4 to -75%)	0·69
	World Bank Lower Middle Income	132·5 (114–155·8)	-45·1% (-53·8 to -34·3%)	4·5 (3·9–5·3)	-59·2% (-64·8 to -52·3%)	0·90
	World Bank Low Income	81·4 (66·9–99·1)	-29% (-42·3 to -12·6%)	12·3 (10·5–14·5)	-55·8% (-62·5 to -47·5%)	0·78
Encephalitis	Global	89·9 (76·5–122·9)	-23·7% (-38·9 to 12·9%)	1·2 (1–1·6)	-45·7% (-56·1 to -21·1%)	0·84
	Southeast Asia, East Asia, and Oceania	11·1 (9·3–12·8)	-40·1% (-53·8 to -5·5%)	0·6 (0·5–0·7)	-47·3% (-58·9 to -25·8%)	0·73
	Central Europe, Eastern Europe, and Central Asia	3·7 (3·2–4·3)	-16·1% (-25·2 to 1·2%)	0·9 (0·8–1)	-19% (-28·1 to -0·7%)	0·72
	High-income	3·3 (2·6–3·5)	79·8% (35·9 to 98·6%)	0·2 (0·2–0·2)	11·7% (-10·9 to 22·4%)	0·77
	Latin America and Caribbean	2·9 (2·4–3·5)	3·9% (-13·6 to 25·4%)	0·5 (0·4–0·6)	-21·4% (-34·6 to -5·8%)	0·76
	North Africa and Middle East	3·9 (3·1–5·1)	26·2% (-9·4 to 71·2%)	0·7 (0·6–0·9)	-24·9% (-42·7 to -1·9%)	1·03
	South Asia	59·9 (48·6–90·5)	-29% (-46·2 to 12·5%)	4·2 (3·4–6·2)	-52·9% (-63·7 to -27%)	0·97
	Sub-Saharan Africa	5 (4–6·7)	85·9% (40·5 to 132%)	0·6 (0·5–0·8)	-2·2% (-20 to 16·2%)	0·70
	World Bank High Income	3·7 (3–3·9)	54·1% (29·7 to 68·6%)	0·2 (0·2–0·2)	-4·9% (-15·7 to 3%)	0·77
	World Bank Upper Middle Income	10·5 (9·2–12·3)	-37·5% (-48·4 to -11·3%)	0·4 (0·4–0·5)	-47·8% (-57·2 to -26·2%)	0·78

Table S10 Global and regional burden, mortality, incidence, and prevalence of neurological conditions in different regions of the world

		All Ages		Age-standardised		
		Number (thousand)	Percent change	Rate per 100 000	Percent change	Sex ratio
**Measure**		2019	From 1990 to 2019	2019	From 1990 to 2019	2019
Cause	Region	Mean (95% UI)	Mean (95% UI)	Mean (95% UI)	Mean (95% UI)	F:M
	World Bank Lower Middle Income	70·6 (58·3–102·6)	-25·5% (-41·9 to 14·9%)	2·8 (2·3–4)	-47·2% (-58·4 to -21·7%)	0·90
	World Bank Low Income	5·1 (4·2–6·1)	32·2% (-5·3 to 110·7%)	1 (0·8–1·2)	-25% (-41·2 to 2·6%)	0·80
Tetanus	Global	34·7 (25·9–48·5)	-87·4% (-90·5 to -81·8%)	0·5 (0·4–0·7)	-89·5% (-91·9 to -84·9%)	0·81
	Southeast Asia, East Asia, and Oceania	5·1 (2·7–6·4)	-90% (-94·5 to -85·5%)	0·2 (0·1–0·3)	-92·9% (-95·5 to -89·9%)	0·53
	Central Europe, Eastern Europe, and Central Asia	<0·1 (<0·1–0·1)	-85·8% (-89·4 to -69·8%)	<0·1 (<0·1–<0·1)	-88·2% (-91·1 to -75·5%)	0·64
	High-income	0·1 (0–0·1)	-79·9% (-84·1 to -59·1%)	<0·1 (<0·1–<0·1)	-88·6% (-90·9 to -76·8%)	0·69
	Latin America and Caribbean	0·4 (0·3–0·8)	-87·3% (-91·6 to -76·2%)	0·1 (0·1–0·1)	-91·8% (-94·5 to -85·2%)	0·68
	North Africa and Middle East	1·3 (0·8–2)	-84·1% (-91·3 to -69%)	0·2 (0·2–0·4)	-88·9% (-93·3 to -80·6%)	0·84
	South Asia	11·7 (8·3–16·1)	-93·2% (-95·1 to -90·3%)	0·7 (0·5–1)	-93·8% (-95·5 to -91·4%)	1·07
	Sub-Saharan Africa	16 (11–25·2)	-60·6% (-74·5 to -36·1%)	1·5 (0·9–2·4)	-78·1% (-84·4 to -69·2%)	0·70
	World Bank High Income	0·1 (0·1–0·2)	-79·4% (-90·5 to -65%)	<0·1 (<0·1–<0·1)	-88·4% (-95·4 to -78·7%)	0·61
	World Bank Upper Middle Income	1 (0·7–1·6)	-95·7% (-96·9 to -92·5%)	<0·1 (<0·1–0·1)	-97% (-97·8 to -94·8%)	0·43
	World Bank Lower Middle Income	23·1 (16·4–30·7)	-89·2% (-92·2 to -85·1%)	0·8 (0·6–1·1)	-91·1% (-93·2 to -88%)	0·83
	World Bank Low Income	10·4 (6·8–17·4)	-72·5% (-82 to -48·1%)	1·5 (0·9–2·3)	-83·1% (-88·1 to -74·3%)	0·71
Brain and central nervous system cancer	Global	246·3 (185·6–270·9)	76·4% (11 to 104·9%)	3 (2·3–3·4)	-1·2% (-36·8 to 13·9%)	0·71
	Southeast Asia, East Asia, and Oceania	79·6 (58·7–93·5)	72·8% (15·7 to 116·3%)	3·1 (2·3–3·7)	-6·2% (-37·2 to 15·4%)	0·75
	Central Europe, Eastern Europe, and Central Asia	24·5 (17·6–28·2)	42·7% (-9·4 to 68%)	4·4 (3·2–5·1)	17·4% (-24·9 to 37·6%)	0·64
	High-income	62·8 (42·5–69·2)	56·4% (-4·4 to 72·8%)	3·6 (2·5–3·9)	-2·5% (-39 to 6·6%)	0·65
	Latin America and Caribbean	19·4 (12·6–22·7)	183% (37 to 269·4%)	3·3 (2·1–3·9)	41·9% (-31 to 84·8%)	0·76
	North Africa and Middle East	17·8 (12·1–20·9)	111·5% (30·3 to 173·4%)	3·7 (2·5–4·3)	3% (-33·4 to 30·2%)	0·87
	South Asia	30·7 (23·9–36·8)	89·7% (17·9 to 154·8%)	1·9 (1·5–2·3)	7·3% (-29·6 to 33·9%)	0·70
	Sub-Saharan Africa	11·4 (8–14)	141·1% (35·3 to 256·3%)	1·6 (1·1–1·9)	20·4% (-26·5 to 60·5%)	0·71
	World Bank High Income	68·7 (46·7–75·6)	55·6% (-6·2 to 72·9%)	3·6 (2·5–3·9)	-3·7% (-40·5 to 6%)	0·65
	World Bank Upper Middle Income	111·1 (81·1–126·4)	79·7% (14·3 to 113·4%)	3·5 (2·5–4)	0·6% (-36·4 to 19·5%)	0·75
	World Bank Lower Middle Income	57·9 (44·3–66·1)	95·4% (23·6 to 143·8%)	2·1 (1·6–2·4)	8·4% (-26·6 to 29·9%)	0·74
	World Bank Low Income	8·5 (6·1–10·6)	112·4% (24·3 to 218·2%)	1·8 (1·3–2·2)	11·4% (-30·4 to 51·9%)	0·72
Stroke	Global	6552·7 (5995·2–7015·1)	43·3% (31 to 55·4%)	84·2 (76·8–90·2)	-36·4% (-41·6 to -31·2%)	0·76
	Southeast Asia, East Asia, and Oceania	2999·6 (2667·7–3332·5)	68·8% (42·6 to 94·2%)	129·4 (114·8–143·6)	-34·5% (-45 to -24·7%)	0·64
	Central Europe, Eastern Europe, and Central Asia	761·7 (691·2–817·9)	-1·7% (-8·5 to 4·9%)	121·6 (110·2–130·6)	-33·5% (-37·9 to -29·2%)	0·78
	High-income	816·8 (697·4–887·7)	-8% (-15·7 to -2·9%)	32·1 (28·1–34·6)	-56·6% (-59·2 to -54·5%)	0·82
	Latin America and Caribbean	281·7 (252·9–304·8)	44·3% (32·7 to 55·9%)	50·1 (44·8–54·2)	-49·3% (-53·1 to -45·4%)	0·82
	North Africa and Middle East	312·2 (278·4–349·7)	75·5% (56·2 to 98·8%)	87·7 (78·2–97·6)	-27·8% (-35·4 to -16%)	1·07
	South Asia	978·9 (864·8–1095·4)	86·9% (57·8 to 116·7%)	80·2 (70·9–89·7)	-32·6% (-43·7 to -20·5%)	0·87
	Sub-Saharan Africa	401·8 (353·1–455·4)	69·3% (48·3 to 93·6%)	106·7 (94·2–120)	-18·3% (-28 to -8%)	0·94
	World Bank High Income	915·3 (786·2–989·5)	-8·7% (-15·8 to -3·7%)	34·2 (29·8–36·8)	-56·5% (-58·9 to -54·4%)	0·82
	World Bank Upper Middle Income	3235·7 (2883·7–3569·7)	45·8% (26·8 to 64·6%)	106·8 (94·9–117·8)	-40·6% (-47·9 to -33·2%)	0·68

Table S10 Global and regional burden, mortality, incidence, and prevalence of neurological conditions in different regions of the world

**Measure**		**All Ages** Number (thousand) 2019	Percent change From 1990 to 2019	**Age-standardised** Rate per 100 000 2019	Percent change From 1990 to 2019	Sex ratio 2019
Cause	Region	Mean (95% UI)	Mean (95% UI)	Mean (95% UI)	Mean (95% UI)	F:M
	World Bank Lower Middle Income	2076·8 (1908·6–2248·7)	78·5% (58·8 to 99·7%)	103·6 (94·9–111·9)	-26·4% (-34·6 to -17%)	0·89
	World Bank Low Income	321·5 (273·1–372·2)	71·6% (51·9 to 94%)	122·8 (104·7–141·2)	-17·6% (-26·3 to -7·8%)	0·92
Ischaemic stroke	Global	3293·4 (2973·5–3536·1)	60·7% (45·8 to 74·7%)	43·5 (39·1–46·8)	-33·6% (-39·2 to -28·2%)	0·81
	Southeast Asia, East Asia, and Oceania	1357·9 (1194·3–1519)	168·6% (110·6 to 216·8%)	61·8 (54·2–69)	-3·1% (-24·2 to 14·4%)	0·65
	Central Europe, Eastern Europe, and Central Asia	546·1 (490·7–589·3)	-0·8% (-8·9 to 6·7%)	86·7 (77·8–93·6)	-35% (-39·9 to -30·2%)	0·85
	High-income	517·3 (433·6–567·5)	-13·2% (-21·1 to -7·7%)	18·9 (16–20·6)	-61·9% (-64·6 to -59·8%)	0·87
	Latin America and Caribbean	149 (129·8–162)	60·3% (44·3 to 76·2%)	27·3 (23·7–29·7)	-48·9% (-53·5 to -44%)	0·82
	North Africa and Middle East	210·1 (187·1–234)	141·8% (110·1 to 175%)	62·9 (56·3–69·9)	-9·1% (-20·7 to 4%)	1·10
	South Asia	377·8 (321·5–443·3)	144·2% (95·6 to 196·6%)	35·2 (30–40·9)	-22·8% (-37·9 to -4·8%)	0·84
	Sub-Saharan Africa	135·1 (118·3–155·8)	113·6% (82·2 to 143·5%)	43·5 (38·3–49·3)	-0·5% (-14·8 to 13·3%)	1·07
	World Bank High Income	589·9 (496·3–644·6)	-12·7% (-20·3 to -7·6%)	20·6 (17·5–22·4)	-61% (-63·6 to -59%)	0·88
	World Bank Upper Middle Income	1708·2 (1522·4–1878·2)	89·5% (64·2 to 111·8%)	58·3 (51·8–64)	-28·4% (-37·8 to -20·3%)	0·74
	World Bank Lower Middle Income	888·5 (792·1–984)	109·3% (80·8 to 139·2%)	50·3 (44·9–55·3)	-20·4% (-30·4 to -8·5%)	0·93
	World Bank Low Income	105·2 (88·4–125·1)	122·9% (95·1 to 152·2%)	47·4 (39·5–56·1)	0% (-11·9 to 12·7%)	0·97
Intracerebral haemorrhage	Global	2886·2 (2644·5–3099·4)	37·5% (21·7 to 50·9%)	36 (33–38·7)	-35·6% (-42·8 to -29·2%)	0·70
	Southeast Asia, East Asia, and Oceania	1511·6 (1346·2–1680·8)	47·3% (23·3 to 70·6%)	62·3 (55·5–69·3)	-42·7% (-52·4 to -34·2%)	0·63
	Central Europe, Eastern Europe, and Central Asia	180 (163·5–194·2)	-8·4% (-17·1 to 0·5%)	29 (26·3–31·3)	-33·2% (-39·4 to -26·6%)	0·62
	High-income	228·9 (202·4–246·9)	-2·9% (-10·6 to 3·2%)	9·9 (8·9–10·6)	-49·9% (-53·1 to -47·1%)	0·68
	Latin America and Caribbean	102·3 (93·1–112·1)	20·5% (9·4 to 33·1%)	17·7 (16–19·3)	-54·4% (-58·5 to -49·6%)	0·73
	North Africa and Middle East	88·5 (77·9–101·1)	18% (1·5 to 37·8%)	21·6 (19–24·3)	-51·4% (-58·1 to -41·3%)	0·99
	South Asia	521·3 (442·8–608·6)	65·4% (38·3 to 92·8%)	39·3 (33·2–45·8)	-38·2% (-48·2 to -27·7%)	0·90
	Sub-Saharan Africa	253·6 (217·3–292·8)	52·6% (32·8 to 75·3%)	60·6 (52·2–69·8)	-27·4% (-36·4 to -16·8%)	0·87
	World Bank High Income	251 (222–271)	-5·9% (-13·1 to 0·3%)	10·2 (9·2–11)	-50·8% (-53·9 to -48·2%)	0·67
	World Bank Upper Middle Income	1368·2 (1210·1–1529·7)	30% (9·4 to 49·6%)	43·5 (38·4–48·5)	-45·1% (-54·1 to -37%)	0·60
	World Bank Lower Middle Income	1063·8 (949–1176·1)	63·9% (43·3 to 83·4%)	48·1 (42·9–53·2)	-30·4% (-39·5 to -21·5%)	0·86
	World Bank Low Income	201·6 (171·5–236·4)	54·7% (35·6 to 77·6%)	70·9 (59·8–82·7)	-25·5% (-33·8 to -15·5%)	0·90
Subarachnoid haemorrhage	Global	373·1 (330–415·9)	-12·1% (-25·3 to 26·3%)	4·7 (4·1–5·2)	-57·4% (-63·8 to -38·8%)	0·86
	Southeast Asia, East Asia, and Oceania	130·1 (106·1–156·5)	-46·9% (-61·4 to -1·5%)	5·3 (4·3–6·3)	-79% (-84·7 to -61·9%)	0·67
	Central Europe, Eastern Europe, and Central Asia	35·6 (31·7–38·9)	27·7% (10·3 to 49·8%)	5·9 (5·3–6·5)	-4·3% (-17·5 to 12·3%)	0·74
	High-income	70·6 (62·9–75·2)	24·8% (14·7 to 32·9%)	3·4 (3·1–3·6)	-31·3% (-35·8 to -27·2%)	1·04
	Latin America and Caribbean	30·5 (27·2–33·7)	75·7% (53·1 to 99·5%)	5·1 (4·6–5·7)	-23·2% (-34·3 to -12·2%)	1·29
	North Africa and Middle East	13·6 (11·2–16·8)	-15% (-35·7 to 38·8%)	3·2 (2·6–3·9)	-59% (-70·7 to -31·6%)	1·05
	South Asia	79·7 (48·6–110·2)	47·8% (13·8 to 152·9%)	5·6 (3·4–7·8)	-42% (-56 to -1·4%)	0·96
	Sub-Saharan Africa	13·1 (7·2–27·5)	64·6% (31·7 to 128·1%)	2·6 (1·3–5·4)	-24·7% (-38·1 to -1·5%)	0·63
	World Bank High Income	74·4 (66·3–79)	24·6% (14·8 to 32·7%)	3·3 (3–3·5)	-31·6% (-36 to -27·5%)	1·03
	World Bank Upper Middle Income	159·3 (134·3–184·6)	-39·8% (-53 to 1·8%)	5 (4·2–5·8)	-73·8% (-79·6 to -55·7%)	0·77

Table S10 Global and regional burden, mortality, incidence, and prevalence of neurological conditions in different regions of the world

		All Ages		Age-standardised		
		Number (thousand)	Percent change	Rate per 100 000	Percent change	Sex ratio
**Measure**		2019	From 1990 to 2019	2019	From 1990 to 2019	2019
Cause	Region	Mean (95% UI)	Mean (95% UI)	Mean (95% UI)	Mean (95% UI)	F:M
	World Bank Lower Middle Income	124·5 (89·7–161·8)	37·9% (8·4 to 111·7%)	5·2 (3·8–6·8)	-38·6% (-52·3 to -6·4%)	0·92
	World Bank Low Income	14·7 (7·9–28·5)	49·3% (19·6 to 99·1%)	4·5 (2·4–8·5)	-31·4% (-44·3 to -9·7%)	0·79
Neurological disorders*	Global	2221·3 (1027·9–4759·8)	154·3% (120·5 to 174·1%)	30·7 (13·8–66·3)	1·2% (-2·8 to 7·7%)	0·98
	Southeast Asia, East Asia, and Oceania	564·2 (238·2–1246·4)	176·5% (117 to 224·4%)	29·3 (11·8–66·4)	-4·1% (-16·3 to 10%)	1·04
	Central Europe, Eastern Europe, and Central Asia	175·9 (76·9–395)	77·2% (61·4 to 88·9%)	29·6 (13–65·3)	4·4% (-1·1 to 11·2%)	0·90
	High-income	839·7 (375·2–1778·7)	149·4% (137·2 to 159·6%)	31·4 (15–64·8)	5·5% (0 to 12%)	0·96
	Latin America and Caribbean	172·3 (76·9–368)	229·3% (181·8 to 258%)	31·7 (14–67·9)	0·3% (-6 to 8·6%)	0·91
	North Africa and Middle East	99·6 (47·3–213·2)	153·3% (111 to 207·8%)	33·2 (14·3–73·9)	-2·8% (-9·6 to 13·9%)	1·01
	South Asia	265·3 (143–544·5)	174% (99·4 to 254%)	27·7 (13·1–60·5)	3·3% (-11·8 to 19·5%)	0·98
	Sub-Saharan Africa	104·2 (57·1–209·1)	132·5% (98·1 to 164·1%)	34 (14·7–77)	9·1% (-0·1 to 20·1%)	0·98
	World Bank High Income	887·8 (396·8–1884·5)	147·5% (134·8 to 157·8%)	31·4 (14·9–65)	4·8% (-0·5 to 11%)	0·97
	World Bank Upper Middle Income	795·5 (342·3–1750·7)	168·8% (118·6 to 204·3%)	29·9 (12·4–66·5)	-3% (-11·6 to 6·7%)	0·97
	World Bank Lower Middle Income	466·9 (241·4–974)	149·4% (102·9 to 193·4%)	29·4 (13·3–64·1)	3·3% (-5·1 to 17·9%)	1·00
	World Bank Low Income	69·9 (37·4–141·1)	126·3% (84·5 to 160·6%)	33·2 (14·3–75·2)	4·3% (-5·4 to 14·2%)	0·99
Alzheimer's disease and other dementias	Global	1623·3 (407·5–4205·7)	189·4% (173·5 to 214·5%)	22·9 (5·8–59·2)	3% (-1·2 to 9·8%)	1·17
	Southeast Asia, East Asia, and Oceania	428·7 (102·7–1121)	231·2% (191·3 to 292%)	23·3 (5·6–60·8)	0·5% (-10·8 to 19%)	1·25
	Central Europe, Eastern Europe, and Central Asia	133·1 (32·4–356·7)	90·8% (78·4 to 106·9%)	22·2 (5·5–58·7)	3·9% (-1·3 to 10·6%)	1·05
	High-income	642·2 (167·9–1599·7)	163·2% (145·2 to 187·1%)	22·5 (5·8–56·7)	3·2% (-1·4 to 9·9%)	1·19
	Latin America and Caribbean	129·4 (32·7–331·5)	278·9% (251·7 to 319·2%)	24 (6·1–61·6)	0·5% (-5·3 to 9·2%)	1·03
	North Africa and Middle East	70·5 (17·2–185·8)	191·6% (169·8 to 253·2%)	25·5 (6·3–67·1)	-2·3% (-9 to 17·3%)	1·12
	South Asia	158·1 (37·7–437·1)	335·4% (282·5 to 409·4%)	19·2 (4·6–52·1)	14·1% (1 to 33%)	1·07
	Sub-Saharan Africa	61·2 (15·2–166·3)	165% (144·8 to 189·5%)	25·5 (6·3–69·1)	11·9% (4·2 to 22·9%)	1·16
	World Bank High Income	679 (177·2–1694·8)	162·1% (144·5 to 185·4%)	22·6 (5·8–56·9)	2·9% (-1·8 to 9·2%)	1·19
	World Bank Upper Middle Income	604·4 (149·1–1576·6)	217·3% (189·2 to 259·8%)	23·3 (5·7–60·3)	0·6% (-7·3 to 12·1%)	1·14
	World Bank Lower Middle Income	297·3 (72·4–811·7)	209·8% (186·2 to 253·8%)	21·4 (5·1–56·2)	6·3% (-1·7 to 22%)	1·13
	World Bank Low Income	41·8 (10·4–113·7)	177% (154·5 to 205·2%)	25·2 (6·3–67·8)	8·6% (-0·1 to 19·9%)	1·17
Parkinson's disease	Global	362·9 (326·9–388·2)	146·5% (128·1 to 162·8%)	4·8 (4·3–5·1)	3·8% (-3·3 to 10·1%)	0·53
	Southeast Asia, East Asia, and Oceania	103·4 (90·2–114·9)	152·4% (116·1 to 190·8%)	4·7 (4·1–5·3)	-10·9% (-23·1 to 2·1%)	0·51
	Central Europe, Eastern Europe, and Central Asia	28·1 (25·3–30·3)	63% (51·9 to 75·8%)	4·4 (4–4·7)	7% (0·1 to 15·3%)	0·55
	High-income	120·1 (105·1–128·2)	133·2% (119·5 to 141·6%)	4·7 (4·1–5)	13·4% (8·6 to 16·9%)	0·45
	Latin America and Caribbean	24·8 (21·9–27·2)	228·8% (204·2 to 256·4%)	4·6 (4–5)	3·6% (-3·8 to 12%)	0·61

Table S10 Global and regional burden, mortality, incidence, and prevalence of neurological conditions in different regions of the world

		All Ages		Age-standardised		
		Number (thousand)	Percent change	Rate per 100 000	Percent change	Sex ratio
**Measure**		2019	From 1990 to 2019	2019	From 1990 to 2019	2019
Cause	Region	Mean (95% UI)	Mean (95% UI)	Mean (95% UI)	Mean (95% UI)	F:M
	North Africa and Middle East	16·8 (14·6–21·6)	179·4% (145·9 to 225·5%)	5·3 (4·6–6·9)	2·3% (-10 to 19%)	0·66
	South Asia	55 (47·4–63·6)	208·7% (153·2 to 271%)	5·2 (4·4–6)	-2·9% (-19·8 to 16·2%)	0·65
	Sub-Saharan Africa	14·7 (13–17·5)	139% (105·8 to 174·5%)	4·9 (4·3–5·9)	11·3% (-3·5 to 27·3%)	0·67
	World Bank High Income	126·9 (111·6–135·5)	130·7% (116·6 to 139·2%)	4·7 (4·1–4·9)	12·4% (7·4 to 16·1%)	0·45
	World Bank Upper Middle Income	134·4 (119·8–147·2)	145·2% (117·3 to 173%)	4·6 (4·1–5)	-7·7% (-17·5 to 1·8%)	0·53
	World Bank Lower Middle Income	91·1 (81·9–100·7)	178% (137·6 to 213·6%)	5·2 (4·7–5·8)	7·5% (-7·5 to 20·8%)	0·65
	World Bank Low Income	10·2 (8·8–12·4)	125·4% (96·9 to 154·6%)	4·7 (4·1–5·9)	0·8% (-10·9 to 13·2%)	0·62
Idiopathic epilepsy	Global	114 (100·2–129·9)	13·9% (-1·4 to 51·6%)	1·5 (1·3–1·7)	-24·8% (-34·2 to -1·6%)	0·71
	Southeast Asia, East Asia, and Oceania	16·7 (14·5–19·5)	-27·7% (-40·5 to -6·4%)	0·7 (0·6–0·8)	-48·1% (-57·1 to -32·9%)	0·56
	Central Europe, Eastern Europe, and Central Asia	6·3 (5·7–6·9)	5·8% (-3·2 to 15·2%)	1·4 (1·2–1·5)	-0·8% (-9·5 to 8·6%)	0·53
	High-income	14·3 (11·8–15·3)	61·2% (26·5 to 71·8%)	0·9 (0·8–0·9)	-0·3% (-17·2 to 4·7%)	0·69
	Latin America and Caribbean	9·1 (8·2–10·3)	28·4% (13 to 49·4%)	1·5 (1·4–1·7)	-23·8% (-32·6 to -12·1%)	0·60
	North Africa and Middle East	6·6 (5·4–7·6)	5·3% (-16·7 to 70·4%)	1·2 (1–1·4)	-33·4% (-46·2 to 2·1%)	0·88
	South Asia	39·5 (31·8–47·5)	7·9% (-14·4 to 67·1%)	2·5 (2–3)	-36·8% (-48·4 to -9·3%)	1·11
	Sub-Saharan Africa	21·5 (17·7–27·5)	76·4% (38·1 to 160%)	2·7 (2·2–3·4)	-13·5% (-28·6 to 16·2%)	0·39
	World Bank High Income	16·6 (13·8–17·7)	59·2% (27·6 to 70·3%)	0·9 (0·8–1)	-0·2% (-15·5 to 6·3%)	0·66
	World Bank Upper Middle Income	27 (24·4–30·1)	-18·3% (-29·2 to 0·1%)	1 (0·9–1·1)	-40·8% (-48·4 to -27·5%)	0·59
	World Bank Lower Middle Income	56·2 (46·1–68·9)	18·5% (-3·9 to 79·6%)	2 (1·6–2·5)	-28·2% (-40·3 to 2·3%)	0·87
	World Bank Low Income	14·2 (12–17·1)	55·6% (22·8 to 138%)	2·6 (2·2–3·1)	-19·1% (-33 to 8·9%)	0·47
Multiple sclerosis	Global	22·4 (20·2–27·8)	68% (40·1 to 84%)	0·3 (0·2–0·3)	-14% (-29·1 to -5·9%)	1·35
	Southeast Asia, East Asia, and Oceania	2·7 (2·2–3·6)	59·9% (20·4 to 138·3%)	0·1 (0·1–0·1)	-22·5% (-40·9 to 13·7%)	0·84
	Central Europe, Eastern Europe, and Central Asia	2·7 (2–4·9)	-5·5% (-25·7 to 42%)	0·5 (0·4–0·9)	-23% (-40·1 to 18%)	1·26
	High-income	10·6 (7·6–12·5)	68·5% (7·7 to 92·8%)	0·6 (0·4–0·7)	5·1% (-29·9 to 18·3%)	1·58
	Latin America and Caribbean	1·2 (1–1·5)	187·4% (110·3 to 238%)	0·2 (0·2–0·2)	20·2% (-12·4 to 41·3%)	1·19
	North Africa and Middle East	1·4 (1·2–1·8)	147·3% (83·1 to 248%)	0·3 (0·2–0·3)	5·1% (-25 to 47·6%)	1·21
	South Asia	2·9 (2·4–3·7)	145% (84 to 267·6%)	0·2 (0·2–0·2)	8·2% (-18·3 to 64·4%)	1·26
	Sub-Saharan Africa	1 (0·8–1·3)	167·9% (95·7 to 264%)	0·2 (0·1–0·2)	15·7% (-12·7 to 58·1%)	1·49
	World Bank High Income	11·4 (8·5–13·8)	60·1% (6·1 to 80·7%)	0·6 (0·4–0·7)	-1% (-31·8 to 10·2%)	1·58
	World Bank Upper Middle Income	5·3 (4·5–7·3)	48·5% (24·5 to 87·4%)	0·2 (0·1–0·2)	-24·6% (-36·7 to -5·1%)	1·08
	World Bank Lower Middle Income	5·1 (4·3–6·6)	113·7% (73·4 to 180·8%)	0·2 (0·2–0·2)	-1·1% (-18·8 to 30·6%)	1·20
	World Bank Low Income	0·6 (0·4–0·9)	132·6% (76·7 to 204·5%)	0·2 (0·1–0·2)	4·3% (-18·4 to 37%)	1·36
Motor neuron disease	Global	39·1 (36·6–41·1)	121·4% (108 to 135·4%)	0·5 (0·4–0·5)	12·4% (5·7 to 19·3%)	0·70
	Southeast Asia, East Asia, and Oceania	3·6 (3·1–4·1)	24·2% (3·6 to 47·5%)	0·1 (0·1–0·2)	-31·9% (-43 to -19·9%)	0·59
	Central Europe, Eastern Europe, and Central Asia	1·8 (1·6–2)	149·4% (106·9 to 187·1%)	0·3 (0·3–0·3)	90·2% (59·5 to 118%)	0·67
	High-income	27·5 (25·5–29)	127·3% (114·8 to 138·2%)	1·4 (1·3–1·4)	30·6% (24·1 to 36·4%)	0·70
	Latin America and Caribbean	2·9 (2·6–3·2)	354·4% (296·6 to 404%)	0·5 (0·4–0·5)	99·8% (74·8 to 120·8%)	0·72

Table S10 Global and regional burden, mortality, incidence, and prevalence of neurological conditions in different regions of the world

		All Ages		Age-standardised		
		Number (thousand)	Percent change	Rate per 100 000	Percent change	Sex ratio
**Measure**		2019	From 1990 to 2019	2019	From 1990 to 2019	2019
Cause	Region	Mean (95% UI)	Mean (95% UI)	Mean (95% UI)	Mean (95% UI)	F:M
	North Africa and Middle East	1·1 (0·9–1·3)	90·4% (23·9 to 177·6%)	0·2 (0·2–0·3)	9·3% (-26·7 to 51·1%)	0·73
	South Asia	1·9 (1·5–2·4)	234·9% (165·3 to 323·9%)	0·1 (0·1–0·2)	54·3% (19 to 102%)	0·76
	Sub-Saharan Africa	0·2 (0·2–0·3)	73·2% (28·3 to 126·4%)	<0·1 (<0·1–<0·1)	-23·5% (-44·5 to 3·7%)	0·87
	World Bank High Income	28·1 (26–29·7)	127% (114·4 to 138·3%)	1·3 (1·2–1·4)	29·9% (23·3 to 36%)	0·71
	World Bank Upper Middle Income	7·7 (7–8·3)	88·5% (65·6 to 114·8%)	0·2 (0·2–0·3)	4·5% (-7·9 to 18·4%)	0·66
	World Bank Lower Middle Income	3·1 (2·6–3·5)	189·1% (137·2 to 247·4%)	0·1 (0·1–0·1)	40·8% (12·7 to 73%)	0·72
	World Bank Low Income	0·2 (0·2–0·3)	67·4% (27·5 to 109·9%)	0·1 (0–0·1)	-11% (-32·2 to 12·2%)	0·86
Other neurological disorders	Global	59·6 (55·1–64·6)	74·8% (59·5 to 91·6%)	0·8 (0·7–0·8)	-0·2% (-8·2 to 9·2%)	0·74
	Southeast Asia, East Asia, and Oceania	9·1 (7·9–10·4)	53·1% (27·7 to 84·3%)	0·4 (0·3–0·4)	-4·5% (-19·9 to 14·7%)	0·62
	Central Europe, Eastern Europe, and Central Asia	3·9 (3·6–4·2)	42·9% (30·9 to 54·5%)	0·8 (0·7–0·8)	19·7% (9·7 to 29·6%)	0·62
	High-income	24·9 (23–26)	78·6% (70·9 to 85%)	1·3 (1·3–1·4)	3·2% (0·3 to 6·4%)	0·68
	Latin America and Caribbean	4·9 (4·5–5·4)	99·5% (82 to 119·2%)	0·8 (0·8–0·9)	3·1% (-5·7 to 12·9%)	0·69
	North Africa and Middle East	3·3 (2·9–3·8)	85·9% (42·8 to 136·2%)	0·7 (0·6–0·8)	17·4% (-7·9 to 49·9%)	0·67
	South Asia	7·9 (5·9–10·6)	80·9% (51 to 141·8%)	0·5 (0·4–0·7)	11·2% (-11·6 to 46·3%)	1·15
	Sub-Saharan Africa	5·6 (4·6–6·9)	94% (42·6 to 152·9%)	0·7 (0·6–0·8)	4·2% (-18·3 to 31·3%)	0·86
	World Bank High Income	25·8 (23·9–26·9)	75·2% (67·8 to 81·6%)	1·3 (1·2–1·3)	1% (-2 to 4·3%)	0·68
	World Bank Upper Middle Income	16·6 (15·3–18)	68·1% (50·2 to 89·1%)	0·6 (0·5–0·6)	5·6% (-5·5 to 18·4%)	0·66
	World Bank Lower Middle Income	14·2 (11·4–17·8)	85·2% (51·2 to 129·4%)	0·5 (0·4–0·6)	14·1% (-7·3 to 41·9%)	0·95
	World Bank Low Income	3 (2·4–3·6)	64% (26·7 to 115·9%)	0·5 (0·4–0·6)	-5·1% (-23·5 to 19·8%)	0·85
**Incidence**						
Meningitis	Global	2507·2 (2113·1–2988·7)	-23·8% (-26·5 to -20·4%)	35·4 (29·6–42·5)	-35·9% (-37 to -34·6%)	0·92
	Southeast Asia, East Asia, and Oceania	177·1 (146·7–211·8)	-60·9% (-63·5 to -57·6%)	10·9 (8·8–13·3)	-57·6% (-59·3 to -55·3%)	0·75
	Central Europe, Eastern Europe, and Central Asia	70·8 (57–86)	-45·7% (-48·6 to -42·5%)	20·9 (16·6–25·6)	-39·2% (-43·1 to -35·5%)	0·79
	High-income	96 (78·2–113·9)	-41·5% (-45·2 to -37%)	10·8 (8·7–13·1)	-49·1% (-51·2 to -46·5%)	0·85
	Latin America and Caribbean	93 (76–110·9)	-33·5% (-39·8 to -25·8%)	16·8 (13·7–20)	-46·3% (-49·9 to -42·3%)	0·86
	North Africa and Middle East	128·5 (106·7–152·7)	-9·9% (-16·3 to -1·9%)	22·5 (18·9–26·4)	-37% (-39·9 to -33·9%)	1·12
	South Asia	741·6 (610·3–883·3)	-35·3% (-38·7 to -31·2%)	43·6 (36·2–52·3)	-49·2% (-50·7 to -47·5%)	1·04
	Sub-Saharan Africa	1200·1 (997·8–1452)	7·8% (4·8 to 11·2%)	96·3 (82·6–111·7)	-42·4% (-43·4 to -41·5%)	0·87
	World Bank High Income	108·3 (87·9–128·8)	-39·9% (-43·6 to -35·3%)	11·1 (8·9–13·6)	-47·1% (-48·9 to -44·6%)	0·85
	World Bank Upper Middle Income	262·9 (216·8–311·1)	-51% (-55·1 to -45·9%)	12 (9·8–14·4)	-52% (-54·7 to -48·6%)	0·89
	World Bank Lower Middle Income	1400·3 (1163·6–1685·8)	-24·6% (-27·3 to -21·1%)	44·8 (37·4–53·8)	-42·6% (-43·8 to -41·4%)	0·96
	World Bank Low Income	734·4 (615·3–878·6)	2·9% (-0·9 to 6·8%)	90·2 (77·6–103·6)	-43·5% (-44·8 to -42·3%)	0·86
Encephalitis	Global	1444·7 (1280·1–1614·9)	12·5% (8·1 to 17·1%)	19·3 (17·1–21·7)	-16·5% (-17·8 to -15·2%)	0·92
	Southeast Asia, East Asia, and Oceania	297·2 (257–339·9)	-9% (-14 to -3·8%)	16·8 (14·3–19·6)	-10·8% (-13·6 to -7·7%)	0·79
	Central Europe, Eastern Europe, and Central Asia	39·4 (34·8–43·8)	-9% (-11·6 to -6·2%)	10 (8·8–11·4)	-5·9% (-7·6 to -4·3%)	0·70
	High-income	66·4 (59·4–73·6)	18·3% (12·9 to 24·4%)	5·7 (5–6·5)	-10·3% (-12·5 to -8%)	1·04

Table S10 Global and regional burden, mortality, incidence, and prevalence of neurological conditions in different regions of the world

**Measure**		**All Ages**		**Age-standardised**		
		Number (thousand)	Percent change	Rate per 100 000	Percent change	Sex ratio
		2019	From 1990 to 2019	2019	From 1990 to 2019	2019
Cause	Region	Mean (95% UI)	Mean (95% UI)	Mean (95% UI)	Mean (95% UI)	F:M
	Latin America and Caribbean	59·4 (51·2–68·3)	-4·1% (-9·4 to 0·9%)	11 (9·4–12·7)	-25·3% (-28 to -22·2%)	0·96
	North Africa and Middle East	58·1 (49·8–67)	46·2% (40·2 to 53·3%)	9·9 (8·5–11·3)	-4·4% (-5·8 to -3%)	1·21
	South Asia	743·1 (669·1–816·1)	11% (5·5 to 16·5%)	46·7 (42·3–51·2)	-26·7% (-27·9 to -25·5%)	0·99
	Sub-Saharan Africa	181·1 (153–212·8)	108·3% (104·7 to 112%)	16·5 (14·4–18·7)	-1·2% (-1·9 to -0·4%)	0·92
	World Bank High Income	71·7 (64·1–79·5)	16·3% (10·6 to 22·2%)	5·7 (5–6·5)	-11·2% (-13·4 to -9%)	1·04
	World Bank Upper Middle Income	339·4 (295·6–386·7)	-8·1% (-13 to -2·9%)	15·4 (13–17·9)	-11·2% (-14 to -8·1%)	0·85
	World Bank Lower Middle Income	913·2 (814·7–1015)	15·7% (10·7 to 21%)	31·9 (28·8–35·2)	-22·3% (-23·7 to -20·9%)	0·93
	World Bank Low Income	119·8 (102·1–140·2)	88·6% (84·2 to 93·2%)	16·9 (14·9–19)	-6·8% (-8 to -5·6%)	0·91
Tetanus	Global	73·7 (53·3–101·1)	-88% (-91·1 to -83·2%)	1 (0·7–1·4)	-90% (-92·4 to -86·1%)	0·78
	Southeast Asia, East Asia, and Oceania	14·9 (8–19·5)	-90·4% (-94·6 to -85·9%)	0·7 (0·4–0·9)	-92·8% (-95·4 to -89·8%)	0·55
	Central Europe, Eastern Europe, and Central Asia	0·1 (0–0·1)	-85% (-88·8 to -66·9%)	<0·1 (<0·1–<0·1)	-87·3% (-90·4 to -73·8%)	0·61
	High-income	0·3 (0·3–0·6)	-80·3% (-85 to -59·4%)	<0·1 (<0·1–<0·1)	-88·9% (-91·5 to -76·4%)	0·71
	Latin America and Caribbean	0·7 (0·5–1·4)	-87·2% (-91·4 to -75·8%)	0·1 (0·1–0·3)	-91·6% (-94·3 to -84·2%)	0·64
	North Africa and Middle East	1·9 (1·2–2·9)	-83% (-90·6 to -68%)	0·3 (0·2–0·5)	-88·4% (-92·9 to -79%)	0·82
	South Asia	28·5 (19·5–41·3)	-92·4% (-94·6 to -89·1%)	1·8 (1·2–2·6)	-93% (-94·9 to -90·2%)	1·07
	Sub-Saharan Africa	27·2 (17·5–42·4)	-60% (-74·7 to -37·2%)	2·5 (1·5–4)	-77·9% (-84·4 to -69%)	0·66
	World Bank High Income	0·4 (0·3–0·7)	-79·9% (-86·6 to -69·4%)	<0·1 (<0·1–<0·1)	-88·7% (-93·3 to -82·6%)	0·68
	World Bank Upper Middle Income	2·6 (1·8–4·2)	-96·4% (-97·6 to -93·8%)	0·1 (0·1–0·1)	-97·3% (-98·1 to -95·5%)	0·44
	World Bank Lower Middle Income	53·2 (37·1–71·3)	-88·8% (-92 to -84·5%)	1·8 (1·3–2·4)	-90·8% (-93·1 to -87·5%)	0·81
	World Bank Low Income	17·4 (10·8–28·4)	-73·2% (-82·3 to -51·2%)	2·4 (1·5–3·7)	-83·2% (-88·4 to -74·7%)	0·66
Brain and central nervous system cancer	Global	348 (262·1–388·9)	94·4% (22 to 128·3%)	4·3 (3·3–4·9)	13·8% (-27·3 to 32·8%)	0·80
	Southeast Asia, East Asia, and Oceania	113·6 (87–134·3)	103·8% (34·7 to 159·5%)	4·7 (3·6–5·5)	22·2% (-19·7 to 52·5%)	0·98
	Central Europe, Eastern Europe, and Central Asia	28 (20·5–31·8)	42·9% (-7·3 to 66·2%)	5·3 (3·9–6)	21·5% (-21·1 to 41·1%)	0·68
	High-income	104 (71·4–121·9)	78% (8·6 to 110·7%)	6·7 (4·7–7·8)	18·9% (-25·9 to 40·9%)	0·74
	Latin America and Caribbean	23·7 (15·7–27·9)	177·2% (34·7 to 263·4%)	4 (2·7–4·8)	50·5% (-26·4 to 96·5%)	0·80
	North Africa and Middle East	27·5 (18·6–32·6)	152·5% (49·6 to 233·1%)	5·2 (3·5–6·1)	28% (-19·6 to 63·3%)	0·84
	South Asia	37·2 (28·4–44·8)	86·6% (12·2 to 156·2%)	2·3 (1·7–2·7)	11·7% (-28·4 to 44·4%)	0·70
	Sub-Saharan Africa	13·9 (9·7–17·1)	136·9% (31 to 259%)	1·7 (1·2–2)	19·4% (-27·8 to 64·5%)	0·70
	World Bank High Income	111·5 (76·6–130·2)	77% (7·6 to 108·5%)	6·6 (4·6–7·6)	17·6% (-27 to 38·4%)	0·75
	World Bank Upper Middle Income	155 (113·6–177·1)	107·6% (30·9 to 147·9%)	5·2 (3·8–5·9)	27·9% (-19·5 to 52·6%)	0·92
	World Bank Lower Middle Income	70·9 (54·3–81·9)	95·5% (20·3 to 150·2%)	2·5 (1·9–2·9)	12·8% (-25·4 to 37·7%)	0·75
	World Bank Low Income	10·4 (7·5–13)	106·3% (19·3 to 217·5%)	1·9 (1·4–2·4)	9·3% (-32·5 to 52·4%)	0·72
Stroke	Global	12224·6 (11041·8–13589·3)	70·1% (66·6 to 73·4%)	150·8 (136·5–167·5)	-16·9% (-18·4 to -15·3%)	0·99
	Southeast Asia, East Asia, and Oceania	5415·4 (4789·4–6219·4)	118·7% (107·1 to 130·4%)	206·2 (183·9–234·4)	-8% (-12·8 to -3·2%)	0·92
	Central Europe, Eastern Europe, and Central Asia	1080·9 (973·6–1205·9)	-4·4% (-6·5 to -2·2%)	179·6 (163·2–199)	-27·9% (-28·9 to -26·8%)	0·91
	High-income	1710·5 (1545·2–1899·3)	10·4% (7 to 14·1%)	87·6 (79·6–96·4)	-34% (-36 to -31·9%)	1·10

Table S10 Global and regional burden, mortality, incidence, and prevalence of neurological conditions in different regions of the world

		All Ages		Age-standardised		
		Number (thousand)	Percent change	Rate per 100 000	Percent change	Sex ratio
**Measure**		2019	From 1990 to 2019	2019	From 1990 to 2019	2019
Cause	Region	Mean (95% UI)	Mean (95% UI)	Mean (95% UI)	Mean (95% UI)	F:M
	Latin America and Caribbean	638·1 (581·3–703·2)	53·9% (50 to 57·8%)	109·2 (99·5–120·3)	-35·5% (-36·6 to -34·3%)	0·96
	North Africa and Middle East	829·8 (758·4–912·8)	130·7% (124·4 to 137·7%)	183 (166·7–201·7)	-5·4% (-7·4 to -3·3%)	1·13
	South Asia	1698·6 (1524·2–1894·1)	110·1% (104·8 to 115%)	117·3 (105·9–130·4)	-10·2% (-11·4 to -9·1%)	1·04
	Sub-Saharan Africa	851·3 (777·1–933·6)	89·8% (86·9 to 92·5%)	160 (147·1–175·3)	-13·9% (-15·2 to -12·7%)	1·07
	World Bank High Income	1939·7 (1756·8–2152·2)	11·5% (8·2 to 15%)	92·6 (84·2–102)	-33·4% (-35·4 to -31·4%)	1·08
	World Bank Upper Middle Income	5884·1 (5210–6712·2)	83·4% (75·4 to 91·1%)	179·7 (160·4–203·2)	-17·4% (-20·6 to -14·1%)	0·94
	World Bank Lower Middle Income	3782·2 (3447·6–4190·7)	98·1% (95·2 to 101·1%)	154·8 (141·3–171)	-11·6% (-12·6 to -10·8%)	1·01
	World Bank Low Income	612 (564·9–666·6)	88·2% (85·3 to 91·1%)	171·2 (159–185·8)	-12·5% (-13·8 to -11·1%)	1·04
Ischaemic stroke	Global	7630·8 (6569·2–8960·4)	87·6% (83·5 to 91·7%)	94·5 (81·9–110·8)	-10% (-12 to -8%)	1·07
	Southeast Asia, East Asia, and Oceania	3643·3 (3052–4352)	201% (188 to 214·4%)	138 (116·7–164·8)	26·8% (23 to 30·9%)	1·04
	Central Europe, Eastern Europe, and Central Asia	755·3 (650·3–876·1)	-3·3% (-6·1 to -0·5%)	123·5 (107·1–142·7)	-28·5% (-29·8 to -27·1%)	1·00
	High-income	1075·6 (922·8–1252·3)	0·6% (-3·2 to 4·8%)	53·1 (45·6–61·4)	-41·1% (-43·2 to -38·7%)	1·09
	Latin America and Caribbean	382·6 (331·3–448·2)	59·3% (54 to 64·5%)	66·4 (57·4–77·6)	-35% (-36·6 to -33·4%)	0·89
	North Africa and Middle East	602·5 (531·3–682·5)	166·9% (157·2 to 177·2%)	135·5 (119·7–153·6)	8·8% (6·3 to 11·4%)	1·16
	South Asia	708·1 (601·3–831·2)	119·6% (108·5 to 129·9%)	50·5 (43–58·9)	-4·5% (-6 to -3%)	1·01
	Sub-Saharan Africa	463·4 (399·7–536·6)	111·2% (107·9 to 114·3%)	86·1 (75–98·9)	-2·1% (-3·1 to -1%)	1·20
	World Bank High Income	1250·2 (1079·5–1449·8)	3·2% (-0·4 to 7·4%)	57·9 (50–66·8)	-39·3% (-41·3 to -37%)	1·08
	World Bank Upper Middle Income	4161·5 (3508·9–4931·2)	130·2% (122·5 to 139%)	126·6 (108·3–149·8)	2·1% (-0·9 to 5·1%)	1·04
	World Bank Lower Middle Income	1890·7 (1634·2–2191·2)	111·3% (105·4 to 116·6%)	79·6 (69·3–92·3)	-6·6% (-8·2 to -5%)	1·07
	World Bank Low Income	324·8 (282·9–372·6)	111·7% (107·6 to 116·2%)	89·5 (78·7–102·5)	-0·3% (-2·1 to 1·7%)	1·16
Intracerebral haemorrhage	Global	3409·1 (2970·5–3909·2)	43·2% (40·7 to 45·5%)	41·8 (36·5–47·9)	-29·2% (-30·5 to -27·8%)	0·78
	Southeast Asia, East Asia, and Oceania	1451·1 (1252·3–1686·5)	38·4% (33·8 to 43·6%)	55·9 (48·4–64·3)	-42·4% (-45·1 to -39·6%)	0·64
	Central Europe, Eastern Europe, and Central Asia	209·1 (188–234·3)	-14·3% (-16·2 to -12%)	35·8 (32·2–40)	-33·1% (-34·4 to -31·9%)	0·66
	High-income	271·1 (237·2–307·8)	-0·1% (-3·1 to 3·4%)	14 (12·3–15·8)	-41·2% (-42·7 to -39·7%)	0·76
	Latin America and Caribbean	160·5 (140·7–183)	29·3% (25·3 to 33·4%)	27·1 (23·8–31)	-44·4% (-45·7 to -43%)	0·90
	North Africa and Middle East	163·2 (149·2–179·8)	65·1% (60·7 to 69·7%)	35 (31·8–38·6)	-32·6% (-34·5 to -30·6%)	1·00
	South Asia	815·7 (692·8–959·2)	109·3% (104·4 to 114·2%)	55·8 (47·5–65·6)	-13·1% (-14·9 to -11·3%)	1·06
	Sub-Saharan Africa	338·4 (303·9–377·2)	65·3% (62·3 to 68·1%)	65·5 (58·9–73)	-26% (-27 to -24·9%)	0·94
	World Bank High Income	308·4 (271·9–348·7)	0·1% (-2·7 to 3·3%)	14·9 (13·3–16·8)	-40·5% (-42 to -39·1%)	0·74
	World Bank Upper Middle Income	1279·2 (1097–1488·3)	17·1% (13·2 to 21·3%)	39·6 (34·1–45·9)	-46·7% (-48·8 to -44·5%)	0·64
	World Bank Lower Middle Income	1572·1 (1362·8–1817·7)	89·8% (86·7 to 92·6%)	63·2 (54·9–72·7)	-15·5% (-16·8 to -14·4%)	0·93
	World Bank Low Income	247·2 (226·1–269·8)	64·1% (61·3 to 67·1%)	71·2 (65·3–77·9)	-24% (-25·2 to -22·8%)	0·92
Subarachnoid haemorrhage	Global	1184·6 (1005·9–1390·4)	60·7% (56·5 to 64·7%)	14·5 (12·3–16·9)	-16·9% (-18·8 to -15·3%)	1·21
	Southeast Asia, East Asia, and Oceania	321 (271–378·1)	47·8% (39·4 to 55·7%)	12·3 (10·6–14·4)	-32·5% (-36·8 to -28·9%)	1·05
	Central Europe, Eastern Europe, and Central Asia	116·5 (99·2–137·4)	10·8% (8 to 13·8%)	20·3 (17·4–23·8)	-10·9% (-12·4 to -9·5%)	0·90
	High-income	363·8 (308·5–430·3)	74·5% (67·3 to 81·2%)	20·5 (17·4–23·9)	8·7% (5·9 to 11·7%)	1·44

Table S10 Global and regional burden, mortality, incidence, and prevalence of neurological conditions in different regions of the world

		All Ages		Age-standardised		
		Number (thousand)	Percent change	Rate per 100 000	Percent change	Sex ratio
**Measure**		2019	From 1990 to 2019	2019	From 1990 to 2019	2019
Cause	Region	Mean (95% UI)	Mean (95% UI)	Mean (95% UI)	Mean (95% UI)	F:M
	Latin America and Caribbean	95 (81·5–111·6)	88·4% (79·5 to 96·8%)	15·7 (13·5–18·4)	-14·1% (-16·2 to -11·9%)	1·46
	North Africa and Middle East	64·1 (54·6–75·6)	82·9% (75·3 to 92·5%)	12·5 (10·7–14·7)	-26·4% (-29·2 to -23·3%)	1·24
	South Asia	174·7 (148–208·1)	81·6% (77·7 to 85·6%)	11·1 (9·4–13·2)	-18·7% (-20·3 to -17%)	1·09
	Sub-Saharan Africa	49·5 (41–59·6)	101·9% (97·2 to 107%)	8·5 (6·9–10·2)	-11·4% (-12·7 to -10%)	0·90
	World Bank High Income	381·2 (322·9–451)	72·5% (65·5 to 78·9%)	19·8 (16·7–23·1)	6·6% (3·9 to 9·4%)	1·43
	World Bank Upper Middle Income	443·3 (377–522·7)	43·6% (37·6 to 49%)	13·5 (11·7–15·9)	-29% (-32·4 to -26·5%)	1·15
	World Bank Lower Middle Income	319·4 (270·7–378·3)	71·9% (69 to 75·2%)	12 (10·2–14·2)	-20·9% (-22·4 to -19·5%)	1·07
	World Bank Low Income	40·1 (33·8–47·6)	90·3% (85·4 to 95·3%)	10·5 (8·7–12·5)	-13·3% (-15·1 to -11·4%)	0·97
Neurological disorders*	Global	805178·5 (725838·1–888847·4)	49·1% (46·1 to 52·4%)	10259·5 (9223·2–11324·2)	-0·1% (-0·6 to 0·5%)	1·11
	Southeast Asia, East Asia, and Oceania	194539·5 (174829·5–214928·3)	36·6% (30·3 to 42·9%)	8814·5 (7936–9750·4)	4·9% (3·2 to 6·7%)	1·19
	Central Europe, Eastern Europe, and Central Asia	52731·7 (47396–58219·9)	0·5% (-1·6 to 2·9%)	12359·3 (11058·2–13681·2)	0% (-1 to 0·9%)	1·09
	High-income	137733·4 (124215·6–151650)	18·4% (15·5 to 21·5%)	12412 (11123·1–13713·2)	0·2% (-0·9 to 1·2%)	1·15
	Latin America and Caribbean	62722·6 (56051·7–69366·1)	54·5% (48·8 to 59·8%)	10557·5 (9444·7–11680·6)	0·5% (-0·4 to 1·4%)	1·11
	North Africa and Middle East	61542·7 (54639·6–68418·4)	89·1% (82·4 to 95·8%)	10090·8 (9024·1–11159·3)	0·8% (0 to 1·6%)	1·07
	South Asia	200573·3 (179176·1–222566·4)	76·4% (72·7 to 80·3%)	10892·1 (9821–12030·4)	0% (-0·4 to 0·4%)	1·03
	Sub-Saharan Africa	95335·2 (83533·8–106945·6)	128·2% (126·7 to 129·6%)	9445·5 (8443–10478)	-0·4% (-0·7 to -0·2%)	1·05
	World Bank High Income	150554 (135429·6–165808·3)	19% (16·1 to 22%)	12200 (10936·3–13478·3)	-0·6% (-1·6 to 0·4%)	1·15
	World Bank Upper Middle Income	252144·5 (226768·2–278851·9)	33% (27·7 to 38·6%)	9327·5 (8375·4–10322·8)	3·1% (1·9 to 4·4%)	1·17
	World Bank Lower Middle Income	342907 (306288·3–380531·5)	74·2% (70·7 to 77·6%)	10707 (9632·9–11814·4)	-0·7% (-1 to -0·5%)	1·06
	World Bank Low Income	59122·8 (51834·6–66755·7)	122·4% (120·7 to 124·2%)	8942·8 (7955·3–9950)	-0·4% (-0·8 to 0%)	1·05
Alzheimer's disease and other dementias	Global	7236·4 (6217·2–8232·7)	147·7% (142 to 153·6%)	95 (81·6–107·9)	1·5% (0·2 to 2·8%)	1·24
	Southeast Asia, East Asia, and Oceania	2267·3 (1915·4–2613·3)	231% (222·2 to 240·6%)	99·6 (84·6–114)	11·7% (9·2 to 14·1%)	1·28
	Central Europe, Eastern Europe, and Central Asia	653·9 (547·5–754·3)	62% (56·3 to 68%)	103·6 (87·4–118·8)	3·2% (1·6 to 4·9%)	1·13
	High-income	2513·7 (2178·2–2835)	107·2% (99·6 to 115%)	99·8 (86·5–112·6)	0·2% (-1·4 to 1·9%)	1·29
	Latin America and Caribbean	504·7 (436·1–573·9)	225% (216·2 to 234·8%)	91·3 (78·8–104·3)	1·5% (-0·1 to 3·1%)	1·10
	North Africa and Middle East	361·2 (309·7–413·1)	177·5% (171·2 to 184·3%)	110·2 (93·9–125·6)	0·8% (-0·7 to 2·6%)	1·09
	South Asia	678·7 (581·6–781·9)	211·4% (202 to 221·5%)	63·6 (54·2–73)	-0·8% (-2·4 to 0·8%)	1·06
	Sub-Saharan Africa	256·9 (222·9–292·7)	122·1% (117·1 to 127·1%)	82·4 (71–94·2)	-0·7% (-2·2 to 0·6%)	1·28
	World Bank High Income	2679·2 (2317·6–3021·1)	107·4% (99·9 to 115·3%)	100·2 (86·7–113·1)	0·3% (-1·3 to 1·9%)	1·28
	World Bank Upper Middle Income	3051·3 (2583·7–3516·6)	199% (191·4 to 206·7%)	102 (86·7–116·7)	8·6% (6·8 to 10·4%)	1·21
	World Bank Lower Middle Income	1318·2 (1130·5–1512·6)	150·2% (145·7 to 155%)	74·6 (63·6–85·4)	-5·8% (-7·1 to -4·5%)	1·14
	World Bank Low Income	184·3 (159·5–210·9)	128·8% (122·7 to 135·3%)	85·3 (73·3–97·3)	-1·1% (-2·6 to 0·3%)	1·26

Table S10 Global and regional burden, mortality, incidence, and prevalence of neurological conditions in different regions of the world

**Measure**		**All Ages** Number (thousand) 2019 Mean (95% UI)	Percent change From 1990 to 2019 Mean (95% UI)	**Age-standardised** Rate per 100 000 2019 Mean (95% UI)	Percent change From 1990 to 2019 Mean (95% UI)	Sex ratio 2019 F:M
Cause	Region					
Parkinson's disease	Global	1081·7 (953·3–1211·2)	159·7% (153 to 167%)	13·4 (11·8–15)	19·6% (17 to 22·3%)	0·56
	Southeast Asia, East Asia, and Oceania	377·8 (320·5–434·2)	194·7% (187·7 to 202·2%)	14·5 (12·3–16·6)	13·7% (11 to 16·6%)	0·63
	Central Europe, Eastern Europe, and Central Asia	69·6 (61·8–77·7)	41·4% (37·2 to 46%)	10·9 (9·7–12·1)	2·6% (0 to 5·1%)	0·60
	High-income	364·5 (329·4–399·2)	147·6% (130·4 to 164·6%)	16·4 (14·8–17·9)	38% (29 to 46·7%)	0·42
	Latin America and Caribbean	63·6 (56·7–70·6)	222·1% (214·5 to 230·9%)	11·2 (10–12·4)	14% (11·9 to 16%)	0·63
	North Africa and Middle East	42·8 (38·3–47·3)	192·5% (184·3 to 201·9%)	11·4 (10·3–12·5)	12·7% (9·9 to 15·7%)	0·68
	South Asia	128·5 (108·4–149·5)	204·1% (194·6 to 213·2%)	10·1 (8·5–11·6)	8·7% (7·2 to 10·6%)	0·70
	Sub-Saharan Africa	34·9 (31·1–38·9)	130·1% (125·8 to 134·7%)	9·2 (8·2–10·2)	6·1% (4·8 to 7·5%)	0·72
	World Bank High Income	385·3 (348·8–421·1)	144·9% (128·2 to 161·3%)	16·2 (14·7–17·7)	36·3% (27·7 to 44·7%)	0·43
	World Bank Upper Middle Income	449·2 (383·4–515·3)	175·5% (169·3 to 182·3%)	13·6 (11·7–15·6)	14·7% (12·2 to 17·3%)	0·62
	World Bank Lower Middle Income	222·7 (193·1–253·7)	162·2% (157·3 to 167·2%)	10·5 (9·1–11·9)	8·3% (7·2 to 9·6%)	0·70
	World Bank Low Income	24 (21·5–26·6)	119·3% (112·6 to 127%)	9 (8·1–9·8)	1·1% (-1 to 3·6%)	0·70
Idiopathic epilepsy	Global	2898·2 (2098·7–3823·4)	55·9% (32·4 to 83·7%)	38·8 (28–51·3)	16·9% (0·1 to 36·5%)	0·86
	Southeast Asia, East Asia, and Oceania	551 (390·4–731·6)	53·1% (19·7 to 105%)	28·8 (19·8–38·2)	39·2% (10·1 to 83·5%)	0·90
	Central Europe, Eastern Europe, and Central Asia	150·9 (104·6–203·9)	2·8% (-15·3 to 23·3%)	39·3 (27·2–53·4)	8% (-11·5 to 29·3%)	0·76
	High-income	422·2 (287·9–563·2)	24·5% (3·6 to 45·2%)	42·9 (28·5–59·1)	6·2% (-10·8 to 23·4%)	0·86
	Latin America and Caribbean	289·7 (203·4–398·6)	33·2% (6·5 to 70·4%)	51·2 (35·5–69·6)	-0·9% (-20·2 to 25·4%)	0·90
	North Africa and Middle East	295·5 (195–407·7)	67·4% (20·3 to 135·5%)	48·2 (32·2–66·4)	8·3% (-21·5 to 51·4%)	0·85
	South Asia	629 (423·2–854·1)	69·6% (23·9 to 146·5%)	35·1 (23·9–47·3)	15·8% (-15·1 to 67·5%)	0·85
	Sub-Saharan Africa	559·9 (362·6–790·4)	125% (79·2 to 192·5%)	46·7 (31·6–63·6)	6·7% (-14·9 to 38·7%)	0·80
	World Bank High Income	479·6 (323·4–643·8)	28·2% (7·2 to 48·5%)	44·1 (28·7–60·3)	9·5% (-8·2 to 25·8%)	0·85
	World Bank Upper Middle Income	865 (608·9–1152·7)	37% (12·5 to 68·7%)	36·5 (25·2–48·7)	23·9% (1·4 to 51·2%)	0·87
	World Bank Lower Middle Income	1228·7 (849·1–1654)	73·8% (38·2 to 128·1%)	38·3 (26·7–51·2)	16·7% (-6·5 to 51·9%)	0·86
	World Bank Low Income	323·1 (196·4–470·4)	121·2% (63·6 to 222·8%)	40·3 (25·3–57·2)	7·2% (-21·1 to 55·1%)	0·80
Multiple sclerosis	Global	59·3 (51·8–66·9)	41·8% (37·7 to 45·7%)	0·7 (0·6–0·8)	-7·3% (-8·7 to -5·8%)	1·69
	Southeast Asia, East Asia, and Oceania	4·4 (3·6–5·2)	54·9% (41·8 to 66·6%)	0·2 (0·1–0·2)	-3·3% (-5·1 to -1·5%)	1·18
	Central Europe, Eastern Europe, and Central Asia	5·3 (4·7–6)	-11·7% (-14·5 to -9·3%)	1·3 (1·1–1·4)	-11·3% (-12·4 to -10·3%)	1·21
	High-income	26·5 (23·9–29)	19% (13·6 to 23·9%)	2·7 (2·4–2·9)	15% (10·5 to 19·1%)	2·04
	Latin America and Caribbean	3·4 (2·8–4·1)	99·5% (90·3 to 108·5%)	0·6 (0·5–0·7)	10·5% (8·6 to 12·6%)	1·72
	North Africa and Middle East	9·2 (7·9–10·5)	120% (110 to 129·4%)	1·4 (1·2–1·6)	5·5% (4 to 6·8%)	1·75
	South Asia	7·4 (6–9)	109·2% (103·6 to 114·7%)	0·4 (0·3–0·5)	9·1% (7·7 to 10·5%)	1·52
	Sub-Saharan Africa	3 (2·4–3·6)	149·2% (145·7 to 153%)	0·3 (0·3–0·4)	5·9% (4·8 to 7·2%)	1·91
	World Bank High Income	28·3 (25·6–31·1)	19·1% (14 to 23·6%)	2·5 (2·3–2·8)	11·8% (7·9 to 15·6%)	2·05

Table S10 Global and regional burden, mortality, incidence, and prevalence of neurological conditions in different regions of the world

		All Ages		Age-standardised		
		Number (thousand)	Percent change	Rate per 100 000	Percent change	Sex ratio
**Measure**		2019	From 1990 to 2019	2019	From 1990 to 2019	2019
Cause	Region	Mean (95% UI)	Mean (95% UI)	Mean (95% UI)	Mean (95% UI)	F:M
	World Bank Upper Middle Income	14·9 (12·8–16·9)	48·4% (42 to 54·5%)	0·5 (0·4–0·6)	6·6% (4·5 to 9·4%)	1·48
	World Bank Lower Middle Income	13·8 (11·3–16·4)	93·5% (89·1 to 98·2%)	0·4 (0·4–0·5)	2·4% (1·4 to 3·7%)	1·53
	World Bank Low Income	2·3 (1·8–2·7)	161·2% (156·4 to 167·4%)	0·4 (0·3–0·4)	11·9% (10·1 to 14·1%)	1·73
Migraine	Global	87649 (76635·7–98654·6)	40% (36·8 to 43·5%)	1142·5 (995·9–1289·4)	2·1% (1·1 to 2·8%)	1·69
	Southeast Asia, East Asia, and Oceania	22465·6 (19797·2–25160·1)	22·2% (16·5 to 28·1%)	1085·2 (951·1–1220·9)	6·8% (5·1 to 8·6%)	1·70
	Central Europe, Eastern Europe, and Central Asia	4108·8 (3608–4635·2)	-7·1% (-9·3 to -4·8%)	1071 (934·3–1216·4)	-0·1% (-0·4 to 0·3%)	1·86
	High-income	11229·2 (9877·3–12582·3)	5·4% (3·6 to 7·7%)	1241·4 (1079–1400·1)	1·2% (0 to 2·2%)	2·01
	Latin America and Caribbean	6990·8 (6079·9–7888·4)	35·3% (30·5 to 40·6%)	1208·5 (1049·3–1374·9)	1·6% (-0·1 to 3·1%)	1·98
	North Africa and Middle East	7950·9 (6837·6–9083·6)	70·6% (62·7 to 79·1%)	1238·9 (1063·5–1415·6)	0·4% (-0·7 to 1·6%)	1·69
	South Asia	23283·1 (20365·1–26136·7)	63·6% (57·2 to 69·6%)	1213·2 (1063·8–1358·6)	-0·2% (-2·4 to 1·8%)	1·53
	Sub-Saharan Africa	11620·6 (9811·2–13428·9)	129·3% (126·3 to 132·3%)	992·7 (859·9–1132·8)	0·9% (0·1 to 1·6%)	1·54
	World Bank High Income	12488·2 (10994·7–14040)	6·5% (4·4 to 9%)	1236·8 (1074·5–1397·1)	1·3% (0·3 to 2·2%)	1·99
	World Bank Upper Middle Income	27121·9 (23923·5–30452·8)	19% (14·2 to 24·4%)	1089·4 (953·9–1227·1)	5·8% (4·2 to 7·6%)	1·82
	World Bank Lower Middle Income	40861·2 (35590·1–46159·9)	64·4% (59·2 to 69·2%)	1211·8 (1057·7–1361·7)	-0·3% (-1·6 to 0·8%)	1·56
	World Bank Low Income	7126·1 (5930–8313·2)	123·8% (121·2 to 126·2%)	937·4 (800·9–1076·2)	0·7% (0·2 to 1·1%)	1·57
Tension-type headache	Global	706190·1 (626723·6–788575·3)	49·6% (46·2 to 53·3%)	8968·2 (7931·9–9990·5)	-0·4% (-1 to 0·2%)	1·05
	Southeast Asia, East Asia, and Oceania	168861·3 (149198·7–189297·5)	37·4% (30·5 to 44·7%)	7585·7 (6721·2–8479·1)	4·4% (2·6 to 6·5%)	1·14
	Central Europe, Eastern Europe, and Central Asia	47740 (42530–53277·5)	0·6% (-1·6 to 3·4%)	11132·6 (9878·8–12448)	-0·1% (-1·2 to 0·9%)	1·04
	High-income	123147·2 (110015·6–136995·2)	18·4% (15·4 to 22%)	11007 (9758·4–12289·1)	0% (-1·2 to 1·2%)	1·09
	Latin America and Caribbean	54866·4 (48544·6–61500·3)	56·6% (50·4 to 63%)	9194·1 (8124·3–10313·7)	0·3% (-0·6 to 1·4%)	1·03
	North Africa and Middle East	52879·8 (46137–59485·2)	91·9% (84 to 99·8%)	8680·1 (7631·6–9732·5)	0·9% (-0·1 to 1·7%)	1·01
	South Asia	175839·8 (155563·4–197126·6)	77·9% (73·6 to 82·3%)	9569·3 (8497·7–10681·1)	0% (-0·4 to 0·3%)	0·98
	Sub-Saharan Africa	82855·6 (71291·4–94414·4)	128% (126·5 to 129·6%)	8313·5 (7335·4–9340·4)	-0·6% (-0·9 to -0·4%)	1·01
	World Bank High Income	134462·4 (119979·6–149502·6)	19% (16 to 22·6%)	10798·5 (9576·8–12055·5)	-0·8% (-2 to 0·2%)	1·09
	World Bank Upper Middle Income	220625·1 (196068·9–246983·6)	33·7% (27·9 to 40·2%)	8084·9 (7158·9–9035)	2·6% (1·1 to 4%)	1·10
	World Bank Lower Middle Income	299249·9 (263442·6–334738·4)	75·3% (71·4 to 79·2%)	9371 (8317·3–10454·1)	-0·8% (-1·1 to -0·6%)	1·01
	World Bank Low Income	51459·9 (44233·5–58852·5)	122·2% (120·3 to 124·2%)	7869·8 (6908·2–8882·1)	-0·6% (-0·9 to -0·2%)	1·01
Motor neuron disease	Global	63·7 (57·3–71·3)	79% (74·1 to 84%)	0·8 (0·7–0·9)	0·3% (-0·4 to 0·9%)	0·77
	Southeast Asia, East Asia, and Oceania	12·1 (10–15)	55·5% (45 to 65·6%)	0·5 (0·4–0·6)	-8·9% (-10·4 to -7·3%)	0·83

Table S10 Global and regional burden, mortality, incidence, and prevalence of neurological conditions in different regions of the world

		All Ages		Age-standardised		
		Number (thousand)	Percent change	Rate per 100 000	Percent change	Sex ratio
**Measure**		2019	From 1990 to 2019	2019	From 1990 to 2019	2019
Cause	Region	Mean (95% UI)	Mean (95% UI)	Mean (95% UI)	Mean (95% UI)	F:M
	Central Europe, Eastern Europe, and Central Asia	3 (2·6–3·5)	27·8% (23·6 to 32·7%)	0·6 (0·5–0·7)	9·8% (7·5 to 12·3%)	0·78
	High-income	30·1 (28·5–31·6)	89·2% (85·3 to 93·4%)	1·7 (1·6–1·8)	14·6% (13 to 16·3%)	0·70
	Latin America and Caribbean	4 (3·5–4·5)	118·2% (103·3 to 133·9%)	0·7 (0·6–0·8)	21·4% (17·4 to 25·4%)	0·81
	North Africa and Middle East	3·4 (2·9–4·1)	83·1% (70·8 to 94·9%)	0·6 (0·5–0·7)	1·9% (0·3 to 3·6%)	0·82
	South Asia	6·9 (5·6–8·4)	75·6% (66 to 84·5%)	0·4 (0·3–0·5)	-1·9% (-3·4 to -0·3%)	0·91
	Sub-Saharan Africa	4·3 (3·5–5·2)	117·3% (112·1 to 122·1%)	0·6 (0·5–0·7)	0·4% (-0·2 to 1·1%)	0·93
	World Bank High Income	30·9 (29·3–32·6)	88·6% (84·5 to 92·6%)	1·6 (1·5–1·7)	14·1% (12·6 to 15·7%)	0·70
	World Bank Upper Middle Income	17·2 (14·6–20·5)	61·7% (52·7 to 70·7%)	0·6 (0·5–0·7)	-0·1% (-2·1 to 1·8%)	0·82
	World Bank Lower Middle Income	12·4 (10·2–15·1)	75·9% (66·9 to 84·3%)	0·4 (0·4–0·5)	-2·1% (-3 to -1%)	0·89
	World Bank Low Income	3·1 (2·6–3·8)	111·6% (106·5 to 116·4%)	0·6 (0·5–0·8)	1·8% (1 to 2·7%)	0·93
Headache disorders	Global	793839·1 (714299·8–877018·8)	48·5% (45·4 to 51·7%)	10110·7 (9070·7–11167·1)	-0·2% (-0·7 to 0·4%)	1·11
	Southeast Asia, East Asia, and Oceania	191326·9 (171561·9–211839·5)	35·5% (29·3 to 42%)	8670·9 (7789·1–9596·3)	4·7% (3·1 to 6·5%)	1·20
	Central Europe, Eastern Europe, and Central Asia	51848·9 (46467·7–57362)	0% (-2·1 to 2·4%)	12203·6 (10908·5–13536·1)	-0·1% (-1·1 to 0·8%)	1·09
	High-income	134376·4 (120812–148269·9)	17·2% (14·4 to 20·4%)	12248·5 (10944·5–13542·9)	0·1% (-1 to 1·2%)	1·15
	Latin America and Caribbean	61857·2 (55187·7–68524·1)	53·9% (48·2 to 59·3%)	10402·6 (9289·8–11520·2)	0·5% (-0·4 to 1·4%)	1·11
	North Africa and Middle East	60830·7 (53891·3–67676·1)	88·8% (82 to 95·6%)	9919 (8853·1–10989·5)	0·8% (-0·1 to 1·5%)	1·07
	South Asia	199122·9 (177628–221237·2)	76·1% (72·3 to 80%)	10782·5 (9709–11924·4)	0% (-0·4 to 0·4%)	1·03
	Sub-Saharan Africa	94476·2 (82667–106164·1)	128·2% (126·7 to 129·6%)	9306·2 (8303·4–10341·2)	-0·5% (-0·7 to -0·2%)	1·05
	World Bank High Income	146950·6 (131956·5–162241·3)	17·9% (15·1 to 20·9%)	12035·3 (10759·2–13320·2)	-0·6% (-1·6 to 0·3%)	1·16
	World Bank Upper Middle Income	247747 (222409·9–274188·2)	31·9% (26·5 to 37·5%)	9174·3 (8230·4–10161·3)	2·9% (1·7 to 4·3%)	1·17
	World Bank Lower Middle Income	340111·1 (303054·8–377611·4)	73·9% (70·4 to 77·3%)	10582·8 (9504·8–11684·9)	-0·8% (-1·1 to -0·5%)	1·06
	World Bank Low Income	58586 (51180·8–66226·4)	122·4% (120·7 to 124·2%)	8807·2 (7816·7–9825)	-0·4% (-0·8 to -0·1%)	1·05
**Prevalence**						
Meningitis	Global	7683·5 (6590·3–9132·2)	-23·7% (-26 to -21·1%)	99·9 (85·5–118·8)	-47·2% (-48·7 to -45·5%)	0·98
	Southeast Asia, East Asia, and Oceania	690·2 (590·4–814·5)	-63·4% (-65·3 to -61%)	32·5 (27·7–38·4)	-71% (-72·4 to -69·3%)	0·82
	Central Europe, Eastern Europe, and Central Asia	253·8 (215·2–300·1)	-42·7% (-45·7 to -39·6%)	59·7 (50·6–70·7)	-43% (-46 to -39·9%)	0·90
	High-income	313 (270·6–360·6)	-45·1% (-47·6 to -42·4%)	25·9 (22·3–30·1)	-57·4% (-59·1 to -55·6%)	0·86
	Latin America and Caribbean	270 (234·3–310·4)	-27·1% (-31·6 to -21·9%)	45·2 (39·3–52·1)	-54·8% (-57·3 to -52%)	0·93
	North Africa and Middle East	356·2 (306·7–419·9)	5·7% (-1·6 to 14%)	60·5 (52·2–71·1)	-44·7% (-48·4 to -40·7%)	1·12
	South Asia	2675·6 (2262·5–3190·2)	-29% (-32·7 to -25·1%)	149·6 (126·9–178)	-59·8% (-61·9 to -57·5%)	1·07
	Sub-Saharan Africa	3124·7 (2646·9–3729·8)	15·8% (10·6 to 21·6%)	318·8 (273·6–375·8)	-48·5% (-50·7 to -46%)	0·91
	World Bank High Income	351·4 (303·8–406)	-43·4% (-45·7 to -40·9%)	26·4 (22·7–30·7)	-56·1% (-57·7 to -54·5%)	0·86
	World Bank Upper Middle Income	849·2 (732·7–984·4)	-57·7% (-60 to -54·8%)	31·3 (27–36·4)	-67·6% (-69·2 to -65·7%)	0·97

Table S10 Global and regional burden, mortality, incidence, and prevalence of neurological conditions in different regions of the world

		All Ages		Age-standardised		
		Number (thousand)	Percent change	Rate per 100 000	Percent change	Sex ratio
**Measure**		2019	From 1990 to 2019	2019	From 1990 to 2019	2019
Cause	Region	Mean (95% UI)	Mean (95% UI)	Mean (95% UI)	Mean (95% UI)	F:M
	World Bank Lower Middle Income	4402·9 (3752·3–5266·2)	-21·7% (-24·7 to -18·5%)	139·9 (119·7–166·8)	-55·1% (-56·8 to -53·2%)	1·00
	World Bank Low Income	2075·9 (1772–2460·4)	14·3% (7·4 to 22·3%)	324·6 (279·5–380·9)	-47·6% (-50·6 to -44%)	0·90
Encephalitis	Global	4499·4 (3372·1–5573·2)	4·7% (1·6 to 8·9%)	56·8 (42·6–70·3)	-31·9% (-33·9 to -28·5%)	0·99
	Southeast Asia, East Asia, and Oceania	1318·3 (1002–1645·4)	-16·2% (-19·2 to -12%)	57·1 (43·8–70·9)	-39·3% (-41·9 to -35·6%)	0·86
	Central Europe, Eastern Europe, and Central Asia	108·2 (80·6–134·3)	-5·3% (-7 to -3·7%)	23·8 (18–29·3)	-10·1% (-11·7 to -8·1%)	0·82
	High-income	157·4 (120·5–193·8)	4·7% (2·1 to 7·2%)	12 (9·4–14·6)	-22% (-23·6 to -20·3%)	1·10
	Latin America and Caribbean	192·8 (146·3–238·9)	16·3% (13·1 to 19·7%)	32·3 (24·5–40·1)	-29·3% (-31·8 to -26·5%)	1·16
	North Africa and Middle East	167·5 (127·8–205·9)	79·3% (73·4 to 86·9%)	28 (21·2–34·6)	-10·4% (-13·5 to -5·9%)	1·30
	South Asia	2039·9 (1521·1–2522·9)	3·8% (-0·4 to 9·6%)	119·2 (88·3–147·8)	-44·3% (-46·3 to -40·9%)	1·05
	Sub-Saharan Africa	515·4 (382·8–646)	119·3% (113·3 to 129·5%)	58·1 (42–73·7)	-4·7% (-7·2 to -0·5%)	0·98
	World Bank High Income	171·1 (131·2–210·8)	4·7% (2·1 to 7·3%)	11·9 (9·3–14·5)	-22·4% (-24 to -20·7%)	1·10
	World Bank Upper Middle Income	1412·3 (1073·9–1759)	-13·6% (-16·6 to -9·6%)	49·8 (38·2–61·7)	-36·7% (-39·1 to -33·1%)	0·92
	World Bank Lower Middle Income	2528·8 (1886·9–3124·5)	9·3% (5·3 to 15%)	84·6 (62·7–104·9)	-39·7% (-41·8 to -36·4%)	1·00
	World Bank Low Income	385·7 (286·4–482·2)	110·5% (102·8 to 122·3%)	66·8 (48·3–84·2)	-5·2% (-8·5 to -0·1%)	0·99
Tetanus	Global	61·7 (44·6–81·2)	-64·4% (-68·1 to -61·2%)	0·8 (0·6–1·1)	-73·7% (-76·2 to -71·5%)	1·01
	Southeast Asia, East Asia, and Oceania	12·9 (9–17·4)	-77·5% (-80·4 to -74·7%)	0·6 (0·4–0·8)	-81·6% (-83·9 to -79·4%)	1·39
	Central Europe, Eastern Europe, and Central Asia	<0·1 (<0·1–<0·1)	-78·4% (-83·9 to -62·5%)	<0·1 (<0·1–<0·1)	-80·1% (-85·3 to -66·9%)	0·80
	High-income	<0·1 (<0·1–0·1)	-67% (-75·2 to -51·8%)	<0·1 (<0·1–<0·1)	-74·5% (-81·8 to -64·7%)	0·37
	Latin America and Caribbean	0·4 (0·2–0·6)	-61·2% (-70 to -53·2%)	0·1 (0–0·1)	-74·4% (-80·2 to -69·1%)	0·79
	North Africa and Middle East	0·7 (0·5–1·1)	-35·7% (-53·8 to -16·2%)	0·1 (0·1–0·2)	-61·7% (-70·5 to -51·9%)	0·63
	South Asia	32·6 (23·7–42·7)	-66·6% (-70·3 to -63·5%)	1·8 (1·3–2·4)	-78·5% (-80·6 to -76·9%)	1·00
	Sub-Saharan Africa	15 (10·9–19·6)	-5·8% (-19·3 to 4·3%)	1·4 (1–1·8)	-56·1% (-61·1 to -52·1%)	0·82
	World Bank High Income	0·1 (0·1–0·1)	-52·8% (-66·7 to -38·1%)	<0·1 (<0·1–<0·1)	-61·3% (-74·8 to -48·6%)	0·57
	World Bank Upper Middle Income	4·7 (3–6·8)	-86·1% (-89·1 to -82·9%)	0·2 (0·1–0·3)	-89·2% (-91·5 to -86·7%)	0·80
	World Bank Lower Middle Income	48·5 (35·3–63·4)	-62·8% (-66·8 to -59·5%)	1·5 (1·1–2)	-76·5% (-78·6 to -74·7%)	1·05
	World Bank Low Income	8·4 (6·1–10·9)	-1·7% (-20·6 to 16·6%)	1·2 (0·9–1·6)	-47·7% (-55·4 to -41·6%)	0·85
Brain and central nervous system cancer	Global	1065·3 (800·4–1199·9)	151·5% (50·8 to 195·7%)	13·5 (10·1–15·2)	59·7% (-2·4 to 86·2%)	1·01
	Southeast Asia, East Asia, and Oceania	364·6 (283·6–436·3)	225·7% (102·4 to 346·8%)	16·3 (12·5–19·2)	137·2% (45·3 to 222%)	1·48
	Central Europe, Eastern Europe, and Central Asia	49·4 (37·1–55·8)	53·7% (2·1 to 77·5%)	10·6 (8–12)	42·5% (-5·9 to 64·7%)	0·79
	High-income	411·2 (277·3–488)	118·9% (31·8 to 163·4%)	30·6 (21·4–36·3)	56·3% (-3·4 to 87·4%)	0·86
	Latin America and Caribbean	49·5 (33·2–58·9)	190·3% (41 to 286%)	8·5 (5·6–10·1)	85·2% (-9·9 to 146·2%)	0·91
	North Africa and Middle East	97·2 (64·2–115·6)	280·5% (113·9 to 424·7%)	16·5 (10·8–19·5)	119·1% (30·2 to 189·1%)	0·81
	South Asia	67·3 (51·8–81)	77·5% (1 to 162·8%)	3·9 (3–4·7)	19·9% (-27·7 to 64·7%)	0·71
	Sub-Saharan Africa	26·2 (18·8–33·1)	134·3% (24·6 to 275·2%)	2·6 (1·8–3·2)	20·2% (-31·1 to 73·9%)	0·70
	World Bank High Income	432 (294·6–511·1)	119·9% (32·8 to 163·4%)	29·4 (20·6–34·7)	57% (-2·5 to 86·8%)	0·87
	World Bank Upper Middle Income	474·2 (357·7–548)	220·3% (94·5 to 293·2%)	17·3 (12·9–19·9)	137·9% (43·1 to 190·5%)	1·29

Table S10 Global and regional burden, mortality, incidence, and prevalence of neurological conditions in different regions of the world

**Measure**		**All Ages**		**Age-standardised**		
		Number (thousand)	Percent change	Rate per 100 000	Percent change	Sex ratio
		2019	From 1990 to 2019	2019	From 1990 to 2019	2019
Cause	Region	Mean (95% UI)	Mean (95% UI)	Mean (95% UI)	Mean (95% UI)	F:M
	World Bank Lower Middle Income	139·1 (107·2–161·6)	101·5% (16·3 to 171·6%)	4·6 (3·5–5·3)	26·9% (-21 to 61·6%)	0·79
	World Bank Low Income	19·6 (14·3–25·1)	97·8% (10·7 to 218%)	2·9 (2·1–3·7)	5·9% (-36·8 to 54·7%)	0·72
Stroke	Global	101474·6 (93211·9–110526·3)	85·3% (82·6 to 88·2%)	1240·3 (1139·7–1353)	-6·1% (-7·2 to -5%)	1·14
	Southeast Asia, East Asia, and Oceania	40296·6 (36493·8–44443·6)	136·9% (126·1 to 148·1%)	1517·9 (1377·7–1679·5)	9·3% (5·2 to 13·7%)	1·14
	Central Europe, Eastern Europe, and Central Asia	7627·4 (6942·9–8379·5)	1·7% (0 to 3·3%)	1283·4 (1173·2–1400·8)	-19·2% (-20·5 to -17·8%)	1·17
	High-income	19336·4 (17636·2–21205·1)	38·9% (35·5 to 42·2%)	1031·4 (948·8–1123·2)	-13·9% (-16·1 to -11·9%)	1·14
	Latin America and Caribbean	6641·7 (6100·9–7213)	72·5% (69·4 to 76%)	1114·2 (1023·6–1210·9)	-26·5% (-27·9 to -25·2%)	1·16
	North Africa and Middle East	7323·4 (6794·7–7863·1)	142·1% (137·8 to 146·3%)	1537·5 (1421·9–1659·9)	-0·5% (-2·3 to 1·1%)	1·24
	South Asia	12784·1 (11531–14080·9)	119·8% (115·6 to 124·2%)	810·1 (730·5–892·8)	-0·6% (-2 to 0·8%)	1·09
	Sub-Saharan Africa	7465 (6847·8–8101)	106·2% (103·4 to 108·7%)	1283·5 (1185·4–1385·2)	-8·4% (-9·4 to -7·5%)	1·18
	World Bank High Income	21470 (19663·6–23527·1)	39·4% (36·2 to 42·6%)	1065·4 (982·2–1157·8)	-13·7% (-15·7 to -11·7%)	1·14
	World Bank Upper Middle Income	45603·3 (41291·5–50255·8)	99·7% (92·2 to 107·6%)	1376·4 (1248·1–1516·3)	-2·9% (-5·9 to 0·2%)	1·17
	World Bank Lower Middle Income	29398·6 (26844·8–31983·4)	109·7% (107·4 to 112·2%)	1120·2 (1023·6–1222·1)	-3·7% (-4·7 to -2·8%)	1·11
	World Bank Low Income	4946·4 (4592·8–5310·6)	100·4% (97·2 to 103·6%)	1277·9 (1190·5–1367·9)	-8·2% (-9·5 to -7%)	1·20
Ischaemic stroke	Global	77192·5 (68857·2–86457·6)	95·3% (91·7 to 99·3%)	951 (849·8–1064·1)	-1·9% (-3·4 to -0·4%)	1·19
	Southeast Asia, East Asia, and Oceania	31681·7 (27554·1–36036·4)	176·2% (159·9 to 194·3%)	1220·2 (1067·6–1388·5)	25·9% (19·8 to 32·5%)	1·29
	Central Europe, Eastern Europe, and Central Asia	5913·4 (5217·5–6686·8)	1·2% (-0·8 to 3·1%)	985·7 (873·4–1107·1)	-20·5% (-22·1 to -18·7%)	1·22
	High-income	15094·1 (13423·3–16968·2)	36·5% (32·5 to 40·9%)	786·1 (700·8–878·1)	-16·4% (-19·1 to -13·7%)	1·05
	Latin America and Caribbean	4698 (4169·8–5255·7)	75·4% (71·5 to 80%)	796·8 (707·6–890·4)	-26·2% (-28·1 to -24·3%)	1·04
	North Africa and Middle East	5998·8 (5474–6566·7)	157·6% (152·8 to 162·7%)	1303·6 (1183·2–1435·4)	5·9% (3·6 to 8%)	1·25
	South Asia	8311·8 (7164·9–9433·9)	129·6% (122·7 to 135·8%)	532·4 (461·5–606·5)	4·6% (3 to 6%)	1·05
	Sub-Saharan Africa	5494·7 (4870·4–6108·4)	118·3% (115·8 to 120·6%)	943·4 (847·8–1045·7)	-2·5% (-3·4 to -1·7%)	1·32
	World Bank High Income	16808·3 (14986·1–18843·2)	37·2% (33·2 to 41·2%)	815 (728·9–907·5)	-16·1% (-18·7 to -13·5%)	1·06
	World Bank Upper Middle Income	36852·5 (32321·9–41736·5)	123·1% (112·3 to 134·7%)	1125 (987·3–1271·4)	7·3% (3·1 to 12·1%)	1·25
	World Bank Lower Middle Income	19886·8 (17496–22232·2)	119·8% (116·4 to 122·9%)	775·7 (685–874·5)	0·7% (-0·6 to 2·1%)	1·16
	World Bank Low Income	3605·1 (3238·3–3957·3)	115·3% (112 to 118·3%)	934·8 (848·1–1020·8)	-1·7% (-3·2 to -0·4%)	1·36
Intracerebral haemorrhage	Global	20663·9 (18016·2–23417·9)	57·8% (56·1 to 59·6%)	248·8 (217·1–281·4)	-16·8% (-18·2 to -15·4%)	0·90
	Southeast Asia, East Asia, and Oceania	8073·5 (6957·9–9260·1)	60·6% (57·3 to 64·3%)	289·3 (251·3–330·3)	-23·4% (-25·9 to -20·9%)	0·70
	Central Europe, Eastern Europe, and Central Asia	1221·2 (1069·3–1390)	-3·9% (-6 to -1·9%)	216·8 (190–245·5)	-19·2% (-21·3 to -17·3%)	0·84
	High-income	2305 (2040·6–2592·2)	21·4% (18·4 to 24·4%)	139·1 (122·5–156·6)	-18·9% (-21 to -16·6%)	1·08
	Latin America and Caribbean	1530·3 (1349·4–1720·9)	52·7% (48·3 to 56·8%)	253 (223·4–284·2)	-31·7% (-33·9 to -29·5%)	1·33
	North Africa and Middle East	1301·3 (1169·2–1441·4)	89·8% (86 to 93·8%)	241·6 (217·5–265·7)	-21·1% (-23·1 to -19·2%)	1·07

Table S10 Global and regional burden, mortality, incidence, and prevalence of neurological conditions in different regions of the world

		All Ages		Age-standardised		
		Number (thousand)	Percent change	Rate per 100 000	Percent change	Sex ratio
**Measure**		2019	From 1990 to 2019	2019	From 1990 to 2019	2019
Cause	Region	Mean (95% UI)	Mean (95% UI)	Mean (95% UI)	Mean (95% UI)	F:M
	South Asia	4142·7 (3526·6–4806)	102% (98·5 to 105·8%)	257·7 (219–298·2)	-7·6% (-9·1 to -6%)	1·14
	Sub-Saharan Africa	2090 (1864·8–2330·1)	79·5% (76 to 82·8%)	347·5 (309·6–388·7)	-21·4% (-22·5 to -20·1%)	0·87
	World Bank High Income	2638·6 (2332·6–2962·4)	24·3% (21·4 to 27%)	149 (131·3–168·5)	-16·4% (-18·8 to -13·8%)	1·04
	World Bank Upper Middle Income	7703·5 (6686·3–8783·7)	40·3% (37 to 43·7%)	229·2 (199·9–260·1)	-28·4% (-30·8 to -25·9%)	0·79
	World Bank Lower Middle Income	8916·9 (7678–10185·3)	91·5% (89·1 to 93·7%)	324 (279·4–369·2)	-10·3% (-11·2 to -9·2%)	0·96
	World Bank Low Income	1390·5 (1258–1533·9)	70·1% (66·9 to 73·7%)	342·6 (308–379·9)	-22·2% (-23·5 to -20·9%)	0·86
Subarachnoid haemorrhage	Global	8396·5 (7188·2–9833·3)	64·5% (60·4 to 68%)	101·6 (87·1–118·5)	-12·9% (-15 to -11·5%)	1·44
	Southeast Asia, East Asia, and Oceania	2415·6 (2052–2857·4)	66·2% (59·5 to 71·6%)	89·3 (75·9–105)	-17·6% (-21·1 to -15·2%)	1·13
	Central Europe, Eastern Europe, and Central Asia	784·4 (668·1–930·7)	6·9% (5·1 to 8·9%)	139·9 (119·8–165)	-11·2% (-12·4 to -9·9%)	1·39
	High-income	2729·3 (2324·3–3236·1)	66·3% (59·5 to 73%)	159·8 (136·7–187·5)	6% (2·2 to 8·7%)	1·79
	Latin America and Caribbean	700·4 (599·5–821·3)	87·9% (82·3 to 93·6%)	113·8 (97·8–133)	-14·5% (-16·4 to -12·6%)	1·78
	North Africa and Middle East	376·9 (320·2–444·6)	103·4% (85 to 112·9%)	64 (54·5–75·3)	-18·1% (-26·3 to -14·1%)	1·58
	South Asia	1068·3 (895·6–1292·8)	87·6% (84·8 to 90·4%)	62·4 (52·4–75)	-9·8% (-11·5 to -8·2%)	1·09
	Sub-Saharan Africa	321·7 (272·6–381)	118·3% (114·5 to 122%)	49·4 (41·9–57·9)	-6·3% (-7·5 to -5·2%)	1·10
	World Bank High Income	2887·4 (2461·6–3411·7)	64% (57·4 to 70·4%)	155·1 (132·7–181·8)	3·7% (-0·1 to 6·2%)	1·80
	World Bank Upper Middle Income	3113·5 (2653·5–3684·1)	52·8% (48·1 to 57%)	93·5 (79·6–109·9)	-20·1% (-22·7 to -18%)	1·34
	World Bank Lower Middle Income	2141·6 (1822·6–2537·9)	81·5% (79·1 to 83·8%)	74·7 (63·4–88·2)	-14·5% (-16·1 to -13·2%)	1·17
	World Bank Low Income	249·6 (213·5–291·5)	104·7% (101 to 108·8%)	58·3 (50·1–68·1)	-7·9% (-9·3 to -6·2%)	1·14
Neurological disorders*	Global	2658932·4 (2452847·9–2858528·8)	54·9% (52·4 to 57·4%)	33451·9 (30870·7–36082)	0·4% (-0·1 to 0·9%)	1·19
	Southeast Asia, East Asia, and Oceania	685311·8 (632012·6–737143·4)	46% (40·9 to 51·1%)	29629·5 (27228·8–32045·9)	5·6% (4·2 to 7·4%)	1·26
	Central Europe, Eastern Europe, and Central Asia	170256·3 (157476·9–182658·3)	3·6% (1·9 to 5·3%)	38238·6 (35315·8–41193·8)	0·1% (-0·5 to 0·8%)	1·17
	High-income	461946·8 (429491–493936·9)	21·6% (19·5 to 23·6%)	39417·1 (36416·2–42300·7)	0·5% (-0·2 to 1·2%)	1·23
	Latin America and Caribbean	209057·1 (193343·7–225622·4)	62·6% (58·4 to 66·5%)	34661·9 (32067·7–37417)	0·2% (-0·5 to 1·1%)	1·22
	North Africa and Middle East	208460·5 (190465·7–226945·6)	101·2% (95·9 to 106·6%)	34170·6 (31389·5–37068·5)	1·4% (0·6 to 2·3%)	1·19
	South Asia	632154·3 (581340·6–683250·5)	84% (80·8 to 87·2%)	34526·3 (31816–37231·5)	0% (-0·8 to 0·8%)	1·12
	Sub-Saharan Africa	291745·5 (263012·9–320857·4)	130·8% (129·4 to 132·4%)	30661·7 (28059·7–33299·7)	-0·2% (-0·6 to 0·2%)	1·13
	World Bank High Income	506563·2 (470908·3–541601)	22·7% (20·6 to 24·8%)	38838·4 (35884·3–41709)	-0·2% (-0·8 to 0·5%)	1·24
	World Bank Upper Middle Income	874705·6 (809482·2–941170·3)	41·6% (37·4 to 46·1%)	30981·9 (28555·6–33367·7)	4% (2·7 to 5·3%)	1·25
	World Bank Lower Middle Income	1094803·5 (1004711·5–1184738·7)	81·2% (78·5 to 84%)	34537·4 (31861·5–37262·9)	-0·5% (-1 to 0%)	1·14
	World Bank Low Income	181354·4 (161856·9–201444)	125% (123·6 to 126·6%)	29116·4 (26502–31862·9)	-0·2% (-0·8 to 0·2%)	1·14
Alzheimer's disease and other dementias	Global	51624·2 (44277–59021·5)	160·9% (156 to 166·3%)	682·5 (585·2–782·7)	5·7% (4·3 to 7%)	1·28
	Southeast Asia, East Asia, and Oceania	16388 (13798·5–19015·6)	264·1% (254 to 274·4%)	740·9 (625·7–855·2)	24·4% (21·7 to 27%)	1·33

Table S10 Global and regional burden, mortality, incidence, and prevalence of neurological conditions in different regions of the world

		**All Ages**		**Age-standardised**		
		Number (thousand)	Percent change	Rate per 100 000	Percent change	Sex ratio
**Measure**		2019	From 1990 to 2019	2019	From 1990 to 2019	2019
Cause	Region	Mean (95% UI)	Mean (95% UI)	Mean (95% UI)	Mean (95% UI)	F:M
	Central Europe, Eastern Europe, and Central Asia	4603·3 (3852–5389·2)	66·7% (61·9 to 72%)	733·6 (615·5–854·9)	4·7% (3 to 6·4%)	1·20
	High-income	18291·5 (15842·7–20800·8)	118% (111·3 to 124·9%)	715 (623·2–813·2)	2·8% (1 to 4·9%)	1·29
	Latin America and Caribbean	3516·7 (3040·7–4011·7)	235·8% (227·1 to 244·9%)	637·3 (550·6–727·8)	5·8% (4·2 to 7·3%)	1·13
	North Africa and Middle East	2485·1 (2117·2–2865)	184·5% (178·1 to 190·7%)	777·6 (660·8–896)	3% (1·5 to 4·6%)	1·11
	South Asia	4636·2 (3926·9–5335·6)	219% (210·3 to 229·1%)	428·4 (365–494)	1·6% (0·2 to 3%)	1·08
	Sub-Saharan Africa	1703·4 (1467·1–1954·6)	123·4% (119·3 to 127·7%)	548·3 (470·7–628·6)	0·6% (-0·8 to 1·9%)	1·38
	World Bank High Income	19475·2 (16835·3–22168·6)	118% (111·3 to 124·7%)	717·4 (623·9–816·3)	2·8% (1 to 4·8%)	1·30
	World Bank Upper Middle Income	21910·3 (18485·4–25275·2)	222·6% (215·5 to 230·2%)	743·5 (629–857·1)	16·5% (14·7 to 18·4%)	1·25
	World Bank Lower Middle Income	8981·6 (7631·5–10351·3)	155·1% (151·1 to 159·5%)	508·3 (434·1–585·5)	-4·5% (-5·8 to -3·1%)	1·18
	World Bank Low Income	1233·3 (1056·8–1416·7)	132·3% (127 to 138·2%)	576·6 (494·6–661·1)	0·8% (-0·7 to 2·4%)	1·35
Parkinson's disease	Global	8511 (7288·5–9841·4)	155·5% (150·4 to 161%)	106·3 (91·2–122·2)	15·9% (13·3 to 18·5%)	0·68
	Southeast Asia, East Asia, and Oceania	3480·4 (2898·8–4148·4)	240·3% (231·2 to 250·4%)	135·3 (113·6–159·6)	30·2% (26·6 to 34·4%)	0·68
	Central Europe, Eastern Europe, and Central Asia	562·8 (481·9–648·2)	42·4% (38·2 to 47·8%)	87·8 (75·2–101·1)	1·7% (-1·5 to 5·2%)	0·65
	High-income	2471·1 (2181·3–2765·5)	95·9% (84·3 to 107·6%)	108·7 (95·8–121·8)	6·9% (1·1 to 12·7%)	0·65
	Latin America and Caribbean	512·1 (440·8–590·9)	238·2% (228·6 to 248·5%)	90·2 (77·7–103·8)	19·9% (16·7 to 23%)	0·63
	North Africa and Middle East	309·9 (265–362·8)	199·5% (188·8 to 211·2%)	82·6 (70·2–95·6)	15·4% (11·5 to 20%)	0·74
	South Asia	930·1 (770·2–1111·8)	216·5% (206·8 to 227·3%)	72·7 (60·3–86)	16·6% (14·3 to 18·8%)	0·76
	Sub-Saharan Africa	244·6 (208–288·1)	136·7% (131·8 to 142·3%)	64·1 (54·4–74·3)	9·7% (7·8 to 11·6%)	0·69
	World Bank High Income	2653·5 (2345–2966)	97% (85·1 to 108·9%)	109·2 (96·3–122·4)	7·4% (1·5 to 13·3%)	0·66
	World Bank Upper Middle Income	4016·3 (3358·7–4768)	213·3% (205·3 to 222·1%)	122·4 (102·8–144·2)	29% (25·3 to 32·7%)	0·66
	World Bank Lower Middle Income	1661·1 (1403–1962·3)	167% (162 to 172·6%)	78·8 (66·1–92·5)	10% (8·3 to 11·7%)	0·75
	World Bank Low Income	176 (147·8–206·4)	124·2% (116·2 to 133·1%)	65·9 (55·4–76·3)	3·5% (0 to 7·4%)	0·73
Idiopathic epilepsy	Global	25111·1 (19033·6–31433)	63·9% (39·3 to 93%)	326·3 (247·8–408·3)	13% (-3·4 to 31·8%)	0·92
	Southeast Asia, East Asia, and Oceania	5090·4 (3744·6–6535·6)	67·2% (30·1 to 122·6%)	242·3 (177·5–311·3)	34·4% (5·9 to 77·4%)	0·92
	Central Europe, Eastern Europe, and Central Asia	1548·3 (1097·2–1988·8)	9·5% (-9·7 to 31·7%)	361·6 (254–471·3)	7·2% (-11·5 to 29·2%)	0·87
	High-income	3930·7 (2702·6–5086·3)	33·1% (8·6 to 60%)	336·8 (231·4–442·1)	5·2% (-14 to 25·5%)	0·96
	Latin America and Caribbean	2897·9 (2115·2–3716·1)	44% (14·4 to 83·4%)	496 (361·4–635·8)	-6% (-24·2 to 20·2%)	0·98
	North Africa and Middle East	1990·9 (1360·2–2600)	77·6% (26·2 to 150·6%)	336·9 (231·8–437·4)	8·6% (-23 to 52·4%)	0·88
	South Asia	5312·5 (3773·4–6915·7)	86·7% (37·8 to 170·5%)	300·8 (215·3–386·2)	13·1% (-15·6 to 63·6%)	0·91
	Sub-Saharan Africa	4340·2 (3000·2–5914·1)	124·5% (78·1 to 192·8%)	414·3 (292·3–544·6)	1·9% (-18·9 to 32·4%)	0·87
	World Bank High Income	4547·3 (3181·3–5876·6)	37·1% (13 to 61·4%)	354·6 (244·3–467·2)	8·2% (-10·3 to 26·2%)	0·94
	World Bank Upper Middle Income	8198·2 (6041·5–10387·8)	46·1% (19·9 to 79·1%)	315·7 (234·1–400·3)	17·3% (-3·3 to 42·5%)	0·93
	World Bank Lower Middle Income	9908·9 (7360·7–12777·3)	88·3% (50·6 to 147·4%)	317·3 (236·8–405)	14·7% (-8·3 to 50·5%)	0·91
	World Bank Low Income	2441·4 (1579·1–3433·9)	117·3% (59·9 to 216%)	345·9 (227·7–477·6)	0·6% (-26·5 to 46·2%)	0·89
Multiple	Global	1756·8 (1531·9–1973·6)	71·7% (66·3 to 76·8%)	21·3 (18·5–23·9)	-6·2% (-8·7 to -3·8%)	1·99

Table S10 Global and regional burden, mortality, incidence, and prevalence of neurological conditions in different regions of the world

Measure		All Ages		Age-standardised		
		Number (thousand)	Percent change	Rate per 100 000	Percent change	Sex ratio
		2019	From 1990 to 2019	2019	From 1990 to 2019	2019
Cause	Region	Mean (95% UI)	Mean (95% UI)	Mean (95% UI)	Mean (95% UI)	F:M
sclerosis	Southeast Asia, East Asia, and Oceania	64·6 (50·2–80·5)	100·1% (86·8 to 112·7%)	2·5 (1·9–3·1)	19·9% (18 to 22%)	1·45
	Central Europe, Eastern Europe, and Central Asia	146·3 (126–166·5)	8·3% (6·2 to 10·2%)	28·4 (24·3–32·5)	-5% (-6·5 to -3·4%)	1·32
	High-income	1062·1 (957·9–1169·8)	59·1% (50·7 to 66·6%)	74·5 (66·8–82·5)	17·5% (11·8 to 22·8%)	2·21
	Latin America and Caribbean	78·5 (63–94·1)	160·7% (152 to 168·7%)	12·7 (10·2–15·2)	20·6% (18·5 to 22·8%)	1·97
	North Africa and Middle East	222·7 (190·7–256·8)	171·3% (165·8 to 176·5%)	39 (33·6–44·7)	11·5% (10 to 12·8%)	1·98
	South Asia	136·6 (107·6–167·8)	135·4% (129·8 to 140·7%)	8 (6·3–9·7)	14·3% (13 to 15·8%)	1·76
	Sub-Saharan Africa	45·9 (35·9–56·8)	158·3% (154·6 to 162·4%)	6·2 (4·9–7·5)	11·2% (9·8 to 13%)	2·20
	World Bank High Income	1114·9 (1002·9–1227·9)	59·1% (51 to 66·3%)	70·3 (63–78)	14·9% (9·6 to 19·9%)	2·23
	World Bank Upper Middle Income	339·1 (286·7–392·3)	85·3% (80·4 to 90·1%)	10·4 (8·8–12·1)	5·9% (4·2 to 7·6%)	1·69
	World Bank Lower Middle Income	266·3 (212·1–325·1)	112·4% (108·8 to 116·1%)	9·1 (7·3–11)	2·7% (1·2 to 4·1%)	1·74
	World Bank Low Income	35·5 (28–43·8)	161·6% (156·9 to 168%)	7·2 (5·8–8·7)	14·4% (12·6 to 16·7%)	1·97
Migraine	Global	1128087·3 (979598·8–1298138·1)	56·3% (52·3 to 60·5%)	14107·3 (12270·3–16239)	1·7% (0·7 to 2·8%)	1·73
	Southeast Asia, East Asia, and Oceania	310834·2 (270520·3–360356·1)	49·2% (41·7 to 57%)	13058·2 (11309·8–15039·5)	6·9% (4·5 to 9·6%)	1·70
	Central Europe, Eastern Europe, and Central Asia	61838·4 (54163·3–71248·3)	3·1% (0·8 to 5·3%)	13767 (11945·1–15870·9)	-0·4% (-0·8 to -0·1%)	1·89
	High-income	177198·5 (154611·4–205095·8)	16·7% (13·4 to 20·2%)	15898 (13829·5–18359·3)	0·8% (-0·6 to 2·2%)	2·08
	Latin America and Caribbean	90994 (78883–106306·2)	62·7% (56·4 to 69·6%)	14973·8 (12981–17494·5)	2% (0·1 to 3·9%)	1·99
	North Africa and Middle East	96931·5 (83756·7–112609·4)	100·9% (92·7 to 108·8%)	15355 (13305·5–17806)	-0·1% (-1·5 to 1·4%)	1·76
	South Asia	272364·6 (235734–313550·1)	84·9% (78·2 to 91·4%)	14665·7 (12739–16845·3)	0% (-2·7 to 2·9%)	1·56
	Sub-Saharan Africa	117926·2 (100150·9–138972·4)	134·6% (132 to 137·4%)	12277·2 (10570–14226·1)	0·8% (-0·1 to 1·8%)	1·56
	World Bank High Income	196511·3 (171633·9–227483·1)	18·8% (15·5 to 22·2%)	15744·2 (13686·9–18125·7)	0·5% (-0·8 to 1·8%)	2·05
	World Bank Upper Middle Income	381854·1 (332207·5–442329)	44·5% (38·1 to 51·5%)	13318·2 (11581·9–15401)	6·3% (4·2 to 8·6%)	1·81
	World Bank Lower Middle Income	476963 (412558·5–549991·2)	83·2% (78·1 to 88·3%)	14807·7 (12872–17010·8)	-0·2% (-1·8 to 1·6%)	1·59
	World Bank Low Income	72108·8 (60626–85649·5)	128·9% (127·4 to 130·4%)	11518·6 (9835·6–13421·6)	0·8% (0·3 to 1·4%)	1·60
Tension-type headache	Global	1995172·5 (1751946·8–2242204·9)	52·6% (49·2 to 55·9%)	25113·5 (22020·8–28316·2)	-0·8% (-1·5 to 0%)	1·06
	Southeast Asia, East Asia, and Oceania	474689·4 (417821·5–533103·3)	43·5% (36·1 to 51·1%)	20786·3 (18180·6–23472·7)	5·6% (3·4 to 8·3%)	1·15
	Central Europe, Eastern Europe, and Central Asia	139748·9 (124072·5–155545·9)	2·3% (-0·3 to 5·1%)	31756·6 (28039·7–35733·2)	0% (-1·1 to 1·2%)	1·05
	High-income	374424·5 (333244·3–416231·4)	20% (16·7 to 23·4%)	32263·7 (28503·7–36000)	0% (-1 to 1·3%)	1·09
	Latin America and Caribbean	155507·4 (135667·9–175082·9)	61% (54·6 to 67·3%)	25764·8 (22483·8–29108)	-0·8% (-1·9 to 0·4%)	1·03
	North Africa and Middle East	149061·7 (128455·9–170990·9)	102·2% (93·5 to 111%)	24504·5 (21304·8–27987·5)	2% (0·7 to 3·4%)	1·02

Table S10 Global and regional burden, mortality, incidence, and prevalence of neurological conditions in different regions of the world

		All Ages		Age-standardised		
		Number (thousand)	Percent change	Rate per 100 000	Percent change	Sex ratio
**Measure**		2019	From 1990 to 2019	2019	From 1990 to 2019	2019
Cause	Region	Mean (95% UI)	Mean (95% UI)	Mean (95% UI)	Mean (95% UI)	F:M
	South Asia	481422·4 (421988·6–544314·7)	83·8% (79·8 to 87·8%)	26254·7 (23110·1–29418·7)	-0·1% (-0·5 to 0·3%)	0·99
	Sub-Saharan Africa	220318·3 (187735·8–255957·4)	129·9% (128·4 to 131·4%)	23094·2 (20058–26210·8)	-0·7% (-1·1 to -0·4%)	1·01
	World Bank High Income	407166·5 (362328·9–453198·5)	20·7% (17·3 to 24%)	31474·7 (27779·7–35146·1)	-1·1% (-2·1 to 0%)	1·10
	World Bank Upper Middle Income	624591·3 (550243·3–698925·7)	38·7% (32·5 to 44·9%)	22303·2 (19499·4–25184·3)	3% (1·3 to 5·1%)	1·11
	World Bank Lower Middle Income	826372·9 (721726·7–936807·4)	80·3% (76·6 to 84%)	26042·8 (22872·1–29328·5)	-0·9% (-1·2 to -0·6%)	1·01
	World Bank Low Income	135924·1 (113741·4–159753·3)	123·9% (122·2 to 125·7%)	21687·4 (18624·2–24822·1)	-0·8% (-1·3 to -0·2%)	1·01
Motor neuron disease	Global	268·7 (231·9–310·7)	68·9% (62·5 to 75·7%)	3·4 (2·9–3·9)	1·9% (0·6 to 3·4%)	0·82
	Southeast Asia, East Asia, and Oceania	55·1 (45·1–67·2)	47% (36·3 to 59·8%)	2·4 (2–3)	9·6% (7·8 to 11·7%)	0·93
	Central Europe, Eastern Europe, and Central Asia	14·8 (12·5–17·6)	6·7% (2·5 to 11·4%)	3·4 (2·8–4·1)	4% (2·4 to 5·7%)	0·99
	High-income	121·3 (107·8–135)	77·5% (70·5 to 84·7%)	7·7 (6·9–8·6)	19·4% (15·8 to 24%)	0·76
	Latin America and Caribbean	15·3 (12·8–18·3)	77% (65·6 to 90·1%)	2·6 (2·2–3·1)	11·3% (8·7 to 14·2%)	0·95
	North Africa and Middle East	15·6 (12·7–19)	103·3% (92·1 to 113·8%)	2·6 (2·1–3·1)	3·5% (2 to 5·1%)	0·86
	South Asia	32·4 (25·6–40·7)	92% (86 to 98·6%)	1·8 (1·4–2·2)	10·3% (8·7 to 12·1%)	0·87
	Sub-Saharan Africa	14·2 (11·2–17·9)	129·7% (126·5 to 132·6%)	1·5 (1·2–1·9)	2·5% (1·8 to 3·1%)	0·89
	World Bank High Income	125·4 (111·5–139·9)	75·4% (68·5 to 82·4%)	7·4 (6·6–8·2)	17·7% (14·4 to 21·9%)	0·77
	World Bank Upper Middle Income	77·3 (63·8–93·7)	46·9% (37·1 to 58%)	2·8 (2·3–3·4)	10·1% (8·5 to 11·9%)	0·93
	World Bank Lower Middle Income	56·1 (44·8–70·2)	84·2% (78·2 to 90·5%)	1·8 (1·4–2·2)	5·4% (4·6 to 6·3%)	0·89
	World Bank Low Income	9·6 (7·6–11·9)	120% (115·7 to 124%)	1·6 (1·3–1·9)	1·2% (0·1 to 2·4%)	0·89
Other neurological disorders	Global	56·9 (39·1–77·8)	66·3% (56·8 to 77%)	0·7 (0·5–1)	6·4% (3·6 to 9·5%)	0·82
	Southeast Asia, East Asia, and Oceania	7·2 (4·8–10·2)	46·8% (29 to 66·9%)	0·3 (0·2–0·5)	9·8% (5·7 to 14·4%)	0·90
	Central Europe, Eastern Europe, and Central Asia	3 (2–4·2)	5·6% (0·4 to 11·2%)	0·7 (0·4–0·9)	0·2% (-1 to 1·6%)	0·89
	High-income	17·5 (11·7–23·7)	64·4% (51·3 to 80%)	1·3 (0·9–1·7)	15·9% (9·4 to 23·5%)	0·82
	Latin America and Caribbean	5·7 (3·8–7·8)	51·3% (40 to 64·9%)	1 (0·7–1·3)	-10·7% (-17 to -5%)	0·76
	North Africa and Middle East	3·9 (2·6–5·5)	96·6% (81 to 113·8%)	0·7 (0·5–1)	0·3% (-0·9 to 1·5%)	1·00
	South Asia	11·5 (7·8–15·9)	80·3% (66·6 to 94·8%)	0·7 (0·5–0·9)	1·4% (-2·9 to 5·6%)	0·76
	Sub-Saharan Africa	8·1 (5·4–11·3)	119·3% (114·9 to 123·9%)	0·8 (0·6–1·2)	-1% (-1·4 to -0·5%)	0·84
	World Bank High Income	18 (12·1–24·5)	64·1% (51·1 to 79·7%)	1·2 (0·8–1·6)	15·1% (8·8 to 22·6%)	0·83
	World Bank Upper Middle Income	14·4 (9·7–20)	43·2% (30·5 to 56·8%)	0·5 (0·4–0·7)	4·3% (0·9 to 7·9%)	0·87
	World Bank Lower Middle Income	20 (13·5–27·7)	80·1% (69·7 to 91·3%)	0·7 (0·4–0·9)	3·1% (0·4 to 5·7%)	0·82
	World Bank Low Income	4·3 (2·9–6·1)	118·7% (113·6 to 124·2%)	0·7 (0·5–0·9)	1·6% (0·9 to 2·3%)	0·89
Headache disorders	Global	2602898·1 (2396738·3–2805119·6)	54% (51·5 to 56·5%)	32716·8 (30148·4–35335)	0·2% (-0·3 to 0·8%)	1·20
	Southeast Asia, East Asia, and Oceania	668443·8 (615598·4–720601·6)	44·2% (39·1 to 49·5%)	28868·6 (26461·8–31286·3)	5·2% (3·7 to 7%)	1·27
	Central Europe, Eastern Europe, and Central Asia	166128·1 (153146·5–178791·1)	2·8% (1·2 to 4·6%)	37506 (34525·4–40423·8)	0% (-0·6 to 0·8%)	1·18

Table S10 Global and regional burden, mortality, incidence, and prevalence of neurological conditions in different regions of the world

		**All Ages**		**Age-standardised**		
		Number (thousand)	Percent change	Rate per 100 000	Percent change	Sex ratio
**Measure**		2019	From 1990 to 2019	2019	From 1990 to 2019	2019
Cause	Region	Mean (95% UI)	Mean (95% UI)	Mean (95% UI)	Mean (95% UI)	F:M
	High-income	445981·2 (412399·9–477975)	20% (17·8 to 22%)	38660·3 (35647·5–41594·4)	0·4% (-0·3 to 1·1%)	1·24
	Latin America and Caribbean	204541·3 (188760·4–221579)	61·7% (57·5 to 65·6%)	33858·3 (31282·1–36692·1)	0·2% (-0·6 to 1%)	1·23
	North Africa and Middle East	205280·4 (186994·5–223750·1)	100·9% (95·4 to 106·3%)	33389·7 (30530·9–36328·1)	1·3% (0·5 to 2·2%)	1·19
	South Asia	625189·3 (573755·6–676224·4)	83·5% (80·4 to 86·7%)	34008·4 (31276·1–36720·4)	-0·1% (-0·9 to 0·7%)	1·12
	Sub-Saharan Africa	287333·9 (258492·8–316694·9)	131% (129·7 to 132·2%)	29968·5 (27357·4–32625·5)	-0·3% (-0·6 to 0·1%)	1·13
	World Bank High Income	489368·8 (452237·2–525167·9)	21·1% (19 to 23·2%)	38069·5 (35083·1–40952·7)	-0·3% (-1 to 0·4%)	1·24
	World Bank Upper Middle Income	851904·5 (785822·5–918508·7)	40% (35·8 to 44·6%)	30187·3 (27747·4–32604·4)	3·6% (2·3 to 5·1%)	1·26
	World Bank Lower Middle Income	1081546·1 (991376·7–1172010·4)	80·9% (78·1 to 83·6%)	33954·8 (31261·2–36715·9)	-0·6% (-1·1 to -0·1%)	1·14
	World Bank Low Income	178601·6 (158915·2–198547·4)	125·1% (123·8 to 126·4%)	28435·4 (25758–31187·2)	-0·3% (-0·7 to 0·1%)	1·14
**YLDs (Years Lived with Disability)**						
Meningitis	Global	683·3 (480·9–924·3)	-19·2% (-22·5 to -15·4%)	9 (6·3–12·2)	-41·2% (-43·6 to -38·4%)	1·02
	Southeast Asia, East Asia, and Oceania	62·6 (43·3–85)	-62·9% (-65·2 to -60·2%)	3·1 (2·1–4·2)	-68% (-69·7 to -65·8%)	0·91
	Central Europe, Eastern Europe, and Central Asia	22·3 (15·6–29·9)	-42% (-44·9 to -39%)	5·6 (3·9–7·6)	-39·5% (-42·7 to -36·3%)	0·99
	High-income	28·3 (19·7–37·5)	-46·2% (-48·6 to -43·7%)	2·5 (1·8–3·3)	-56·5% (-58·4 to -54·6%)	0·92
	Latin America and Caribbean	23·4 (16·6–31)	-27·3% (-31·9 to -22·2%)	3·9 (2·8–5·2)	-51·6% (-54·4 to -48·4%)	1·01
	North Africa and Middle East	36·2 (25·1–48·7)	17·1% (9·2 to 26·8%)	6 (4·2–8)	-34·6% (-39 to -29·4%)	1·16
	South Asia	254·4 (178·2–346)	-20·2% (-25·9 to -13·5%)	13·9 (9·7–18·7)	-51·3% (-54·7 to -47·2%)	1·09
	Sub-Saharan Africa	256·1 (179·2–347·5)	25·5% (17·9 to 35·4%)	23·8 (17–31·9)	-42·5% (-45·7 to -38·3%)	0·95
	World Bank High Income	31·7 (22·2–42)	-44·4% (-46·8 to -42·1%)	2·6 (1·8–3·4)	-55·2% (-57·1 to -53·4%)	0·92
	World Bank Upper Middle Income	75·6 (53–100·7)	-58·1% (-60·6 to -55%)	2·9 (2–3·9)	-65·4% (-67·3 to -63%)	1·03
	World Bank Lower Middle Income	400·9 (280·5–544·6)	-14·8% (-19·6 to -9%)	12·4 (8·7–16·7)	-48% (-50·8 to -44·4%)	1·04
	World Bank Low Income	174·7 (123·7–236·7)	27·4% (17·9 to 39·5%)	24·9 (17·8–33·3)	-39·8% (-43·9 to -34·4%)	0·95
Encephalitis	Global	482·4 (343·3–647·3)	5·2% (0·7 to 9·8%)	6·1 (4·4–8·3)	-28·8% (-31·6 to -25·6%)	1·03
	Southeast Asia, East Asia, and Oceania	131·8 (91·3–177·7)	-23·9% (-29·6 to -17·9%)	5·9 (4·1–8)	-40·5% (-44·6 to -36%)	0·92
	Central Europe, Eastern Europe, and Central Asia	10·7 (7·5–14·4)	-9% (-13 to -5·7%)	2·5 (1·7–3·4)	-9·8% (-13·4 to -6·6%)	0·91
	High-income	16·3 (11·4–21·8)	1·3% (-2·1 to 4·5%)	1·3 (0·9–1·8)	-21·8% (-24·2 to -19·6%)	1·18
	Latin America and Caribbean	19·5 (13·5–26·4)	10·4% (4·6 to 16·8%)	3·3 (2·3–4·4)	-29·1% (-33 to -25·1%)	1·23
	North Africa and Middle East	19·8 (13·8–26·8)	84·9% (76·6 to 93·9%)	3·2 (2·2–4·3)	-4·1% (-8·1 to 0·4%)	1·35
	South Asia	228·4 (163·3–307·1)	12% (5·1 to 18·9%)	12·9 (9·2–17·2)	-37·1% (-40·6 to -33·4%)	1·07
	Sub-Saharan Africa	55·8 (39–75·8)	119·5% (109·1 to 130·7%)	5·7 (4–7·7)	-4% (-8·4 to 0·5%)	1·03
	World Bank High Income	17·7 (12·4–23·7)	1·2% (-2·2 to 4·6%)	1·3 (0·9–1·7)	-22·4% (-24·9 to -20·1%)	1·18
	World Bank Upper Middle Income	140·2 (97·2–189·2)	-21·4% (-27·1 to -15·4%)	5·1 (3·6–6·9)	-38·3% (-42·7 to -33·8%)	0·97
	World Bank Lower Middle Income	281 (199·4–376·4)	15·7% (9·3 to 22·1%)	9 (6·4–12·1)	-34% (-37·5 to -30·6%)	1·04

Table S10 Global and regional burden, mortality, incidence, and prevalence of neurological conditions in different regions of the world

		All Ages		Age-standardised		
		Number (thousand)	Percent change	Rate per 100 000	Percent change	Sex ratio
**Measure**		2019	From 1990 to 2019	2019	From 1990 to 2019	2019
Cause	Region	Mean (95% UI)	Mean (95% UI)	Mean (95% UI)	Mean (95% UI)	F:M
	World Bank Low Income	43·3 (30·1–58·9)	118·4% (105·5 to 131·8%)	6·8 (4·8–9·2)	-0·8% (-6·5 to 5·2%)	1·04
Tetanus	Global	1·7 (1–2·7)	-76·5% (-81·8 to -71%)	<0·1 (<0·1–<0·1)	-81·7% (-85·3 to -77·9%)	0·94
	Southeast Asia, East Asia, and Oceania	0·4 (0·2–0·6)	-83·5% (-87·6 to -79·7%)	<0·1 (<0·1–<0·1)	-86·8% (-89·8 to -83·8%)	1·12
	Central Europe, Eastern Europe, and Central Asia	<0·1 (<0·1–<0·1)	-83·9% (-87·8 to -66·5%)	<0·1 (<0·1–<0·1)	-86·1% (-89·4 to -72·7%)	0·65
	High-income	<0·1 (<0·1–<0·1)	-79% (-83·7 to -60·2%)	<0·1 (<0·1–<0·1)	-87·2% (-90·2 to -76%)	0·62
	Latin America and Caribbean	<0·1 (<0·1–<0·1)	-79·2% (-85·7 to -70%)	<0·1 (<0·1–<0·1)	-86·3% (-90·4 to -80·5%)	0·73
	North Africa and Middle East	<0·1 (<0·1–<0·1)	-70·2% (-81·9 to -50·5%)	<0·1 (<0·1–<0·1)	-80·9% (-87·4 to -70·7%)	0·71
	South Asia	0·9 (0·5–1·4)	-80·1% (-85·3 to -74·7%)	<0·1 (<0·1–0·1)	-85·2% (-88·4 to -82·3%)	1·01
	Sub-Saharan Africa	0·5 (0·3–0·7)	-35·7% (-54·2 to -16·8%)	<0·1 (<0·1–0·1)	-67·5% (-75·4 to -60%)	0·76
	World Bank High Income	<0·1 (<0·1–<0·1)	-74·8% (-82·3 to -64·5%)	<0·1 (<0·1–<0·1)	-82·7% (-89·2 to -74·4%)	0·68
	World Bank Upper Middle Income	0·1 (0·1–0·2)	-90·7% (-93·5 to -87·5%)	<0·1 (<0·1–<0·1)	-92·8% (-94·9 to -90·2%)	0·76
	World Bank Lower Middle Income	1·3 (0·8–2·1)	-76·2% (-81·9 to -70·6%)	<0·1 (<0·1–0·1)	-83·2% (-86·5 to -80·2%)	0·98
	World Bank Low Income	0·3 (0·2–0·4)	-50·4% (-65·3 to -27·5%)	<0·1 (<0·1–0·1)	-70·7% (-78·4 to -61·8%)	0·78
Brain and central nervous system cancer	Global	129·4 (83·9–175)	118·1% (34·2 to 156·9%)	1·6 (1·1–2·2)	29·5% (-18·9 to 52%)	0·86
	Southeast Asia, East Asia, and Oceania	43·1 (28·2–60·1)	150% (62·5 to 225·7%)	1·8 (1·2–2·5)	56·5% (1·1 to 101·5%)	1·14
	Central Europe, Eastern Europe, and Central Asia	8·6 (5·5–11·8)	47·1% (-3·7 to 70·6%)	1·7 (1·1–2·3)	27·7% (-16·2 to 48%)	0·71
	High-income	44 (26·9–60·8)	96·9% (19·3 to 135·8%)	3 (1·8–4·1)	34·9% (-16·1 to 60·9%)	0·78
	Latin America and Caribbean	7·6 (4·7–10·7)	189·3% (40·8 to 280·8%)	1·3 (0·8–1·8)	61·4% (-21·5 to 113%)	0·82
	North Africa and Middle East	10·6 (6·2–15)	203·1% (78·6 to 311·3%)	1·9 (1·1–2·7)	55·6% (-2·6 to 105·3%)	0·78
	South Asia	11·2 (7·4–15·4)	87·5% (12·1 to 159·7%)	0·7 (0·4–0·9)	14·4% (-27·1 to 49·2%)	0·69
	Sub-Saharan Africa	4·2 (2·6–5·9)	137·8% (31 to 263·7%)	0·5 (0·3–0·7)	20% (-28·3 to 66·9%)	0·69
	World Bank High Income	46·7 (28·5–64·4)	96·5% (19·5 to 133·9%)	2·9 (1·8–4)	34·1% (-16·6 to 59%)	0·78
	World Bank Upper Middle Income	57·5 (37·5–78·7)	149·4% (55·4 to 201·3%)	2 (1·3–2·7)	60·6% (-0·4 to 94·4%)	1·03
	World Bank Lower Middle Income	22 (14·5–29·7)	100·8% (22·3 to 158·6%)	0·8 (0·5–1)	17·2% (-23 to 43·7%)	0·75
	World Bank Low Income	3·1 (2–4·4)	105·2% (18·2 to 218·8%)	0·6 (0·4–0·8)	8·9% (-33 to 52·6%)	0·71
Stroke	Global	17741·7 (12759·4–22587·4)	88·9% (85·3 to 92·7%)	218·1 (156·7–277)	-4·7% (-6·1 to -3·3%)	1·30
	Southeast Asia, East Asia, and Oceania	8389·8 (5940·5–10818·6)	140·2% (128·4 to 152·7%)	320·7 (227–412)	11·3% (6·9 to 16%)	1·30
	Central Europe, Eastern Europe, and Central Asia	1495·7 (1077·1–1904·4)	1·7% (0 to 3·4%)	252·2 (181·4–321·6)	-19·3% (-20·9 to -17·9%)	1·33
	High-income	3150·8 (2286–3990·4)	41·2% (37·5 to 45%)	163·6 (118·1–206·4)	-14·4% (-16·7 to -12·3%)	1·26
	Latin America and Caribbean	631·8 (465·1–796·2)	76·3% (72·9 to 80·3%)	107·1 (78·6–134·9)	-25·7% (-27·2 to -24·2%)	1·37
	North Africa and Middle East	1113·7 (812·1–1400·8)	140·2% (135·3 to 145·3%)	239·4 (176·1–301·4)	-0·7% (-2·6 to 1·2%)	1·43
	South Asia	1902·1 (1371·3–2435·5)	120·3% (115·5 to 125·7%)	121·3 (87·8–155·1)	0·2% (-1·4 to 1·8%)	1·25
	Sub-Saharan Africa	1057·8 (768·3–1329·3)	107·2% (104·3 to 110%)	187·1 (137·2–234·9)	-7·6% (-8·6 to -6·5%)	1·37
	World Bank High Income	3545·8 (2568·3–4486·7)	40·6% (37·1 to 44·2%)	171·8 (124·1–216·4)	-14·7% (-16·9 to -12·7%)	1·27
	World Bank Upper Middle Income	8583·8 (6097·7–10998·2)	106·9% (97·7 to 116·6%)	261·1 (185·2–335·2)	0·1% (-3·6 to 4%)	1·32
	World Bank Lower Middle Income	4868·4 (3489–6186·2)	107·1% (104·4 to 109·8%)	188·2 (135·3–238·9)	-4·9% (-6 to -3·8%)	1·27

Table S10 Global and regional burden, mortality, incidence, and prevalence of neurological conditions in different regions of the world

Measure		All Ages		Age-standardised		
		Number (thousand)	Percent change	Rate per 100 000	Percent change	Sex ratio
		2019	From 1990 to 2019	2019	From 1990 to 2019	2019
Cause	Region	Mean (95% UI)	Mean (95% UI)	Mean (95% UI)	Mean (95% UI)	F:M
	World Bank Low Income	735·4 (533·3–923·5)	101·4% (97·7 to 105%)	196·6 (144·5–244·5)	-7·2% (-8·7 to -5·6%)	1·40
Ischaemic stroke	Global	13128·5 (9349·9–16930·4)	102% (97·4 to 106·9%)	162·3 (115·8–209·9)	0·1% (-1·8 to 2%)	1·36
	Southeast Asia, East Asia, and Oceania	6400·7 (4450·5–8359·1)	182·5% (166 to 200·2%)	248·5 (174·6–323·6)	27·3% (21·1 to 34·3%)	1·45
	Central Europe, Eastern Europe, and Central Asia	1128·4 (809·8–1465·6)	2% (-0·3 to 4%)	187·2 (133·9–241·9)	-20·4% (-22·3 to -18·7%)	1·39
	High-income	2376·4 (1723·7–3030·4)	39·2% (34·7 to 43·7%)	119 (85·4–152·7)	-17·3% (-20 to -14·5%)	1·16
	Latin America and Caribbean	433·3 (313·9–550)	80·9% (76·3 to 86·1%)	74·3 (53·9–94·5)	-25·7% (-27·6 to -23·8%)	1·24
	North Africa and Middle East	877·3 (639·8–1114·1)	157·6% (151·5 to 164·4%)	195·6 (144·4–249·1)	5·2% (2·8 to 7·6%)	1·45
	South Asia	1172·1 (824·7–1509·8)	133·9% (126·5 to 141·8%)	76·2 (53·6–98·6)	5·2% (3·2 to 7·1%)	1·21
	Sub-Saharan Africa	740·3 (529·9–939·5)	118·6% (115·6 to 121·9%)	133·4 (95·8–168·6)	-2% (-3·1 to -0·8%)	1·52
	World Bank High Income	2688·1 (1950·5–3434·5)	39% (34·8 to 43·3%)	125·9 (90·2–161·3)	-17·3% (-19·8 to -14·7%)	1·19
	World Bank Upper Middle Income	6780·2 (4777·2–8828)	134·9% (122·7 to 148·3%)	207·4 (145·3–269·1)	11·1% (6·3 to 16·6%)	1·43
	World Bank Lower Middle Income	3143·5 (2221·3–4045·7)	118·4% (114·6 to 122%)	125·4 (89·6–162·6)	-1·3% (-2·8 to 0·3%)	1·34
	World Bank Low Income	511·1 (367·9–645·6)	116% (111·5 to 120·9%)	140·3 (102·7–176·6)	-0·8% (-2·9 to 1·1%)	1·59
Intracerebral haemorrhage	Global	3266·3 (2334–4173·7)	57·2% (54·9 to 59·5%)	39·4 (28·1–50·2)	-17·7% (-19·2 to -16·1%)	0·99
	Southeast Asia, East Asia, and Oceania	1521 (1080·1–1970·3)	60·4% (56·5 to 64·5%)	54·7 (38·8–70·7)	-23·7% (-26·3 to -21·2%)	0·81
	Central Europe, Eastern Europe, and Central Asia	222 (157·2–287·6)	-3·4% (-6·2 to -0·8%)	39·3 (27·6–50·4)	-19·2% (-21·9 to -16·6%)	0·96
	High-income	352·5 (252–452·3)	24·9% (21·1 to 28·8%)	20·6 (14·6–26·2)	-18·5% (-21 to -16·1%)	1·19
	Latin America and Caribbean	135·9 (97·9–173·8)	57·6% (51·9 to 63·1%)	22·6 (16·3–28·9)	-30·3% (-32·7 to -27·8%)	1·62
	North Africa and Middle East	182·2 (129·1–229·6)	88·9% (82·6 to 95·8%)	34·4 (24·4–43·5)	-21·5% (-24·2 to -18·4%)	1·26
	South Asia	578·9 (410·1–750)	104·7% (99·4 to 110·3%)	36·2 (25·7–46·9)	-6·9% (-9·2 to -4·4%)	1·34
	Sub-Saharan Africa	273·8 (197·3–342·1)	80·1% (76 to 84·3%)	46·7 (33·7–59)	-20·7% (-22·2 to -19·1%)	1·02
	World Bank High Income	406·7 (289·7–518·4)	25·8% (22·2 to 29·3%)	22·3 (15·8–28·2)	-17·1% (-19·8 to -14·5%)	1·16
	World Bank Upper Middle Income	1279·9 (910–1646·1)	39·6% (35·7 to 43·9%)	37·9 (26·9–48·7)	-29·8% (-32·4 to -27·1%)	0·85
	World Bank Lower Middle Income	1388·4 (983·4–1785·4)	91·5% (88·3 to 95·1%)	50·9 (36·3–65·7)	-10·5% (-11·9 to -9%)	1·09
	World Bank Low Income	189·2 (135·7–237·8)	70·3% (65·7 to 75·1%)	47·7 (34·4–60·5)	-21·7% (-23·8 to -19·7%)	1·00
Subarachnoid haemorrhage	Global	1346·9 (964·2–1782·2)	65·4% (60·7 to 69·6%)	16·4 (11·7–21·6)	-12·9% (-15·2 to -11·2%)	1·63
	Southeast Asia, East Asia, and Oceania	468·1 (330·9–620·1)	67·5% (60·3 to 74·5%)	17·4 (12·3–23·1)	-17·3% (-21 to -14·3%)	1·31
	Central Europe, Eastern Europe, and Central Asia	145·4 (102·9–191·1)	8·1% (5 to 11·2%)	25·7 (18·3–33·8)	-10·8% (-13 to -8·4%)	1·61
	High-income	422 (302·6–556·4)	74·4% (66·2 to 82·4%)	24 (17·2–31·6)	8·7% (4·3 to 11·9%)	2·05
	Latin America and Caribbean	62·6 (45·1–82·4)	92·3% (85 to 100·2%)	10·2 (7·4–13·4)	-13·2% (-16·1 to -10·4%)	2·07
	North Africa and Middle East	54·2 (37·9–72·3)	103·1% (83·8 to 116·5%)	9·3 (6·6–12·4)	-17·9% (-26·2 to -12·2%)	1·77
	South Asia	151·1 (105·7–203·3)	90·2% (84·1 to 96·6%)	8·9 (6·2–12)	-9% (-12·3 to -5·7%)	1·26
	Sub-Saharan Africa	43·6 (31·2–57·5)	118·9% (112·9 to 125·4%)	6·9 (5–8·9)	-5·6% (-8·1 to -2·9%)	1·28
	World Bank High Income	451 (322·8–592·9)	70·3% (62·6 to 77·9%)	23·6 (16·9–31·1)	5·6% (1·5 to 8·6%)	2·06
	World Bank Upper Middle Income	523·6 (372·1–692·3)	51·5% (45·5 to 56·8%)	15·7 (11·2–20·9)	-21·6% (-24·5 to -19·2%)	1·47

Table S10 Global and regional burden, mortality, incidence, and prevalence of neurological conditions in different regions of the world

		All Ages		Age-standardised		
		Number (thousand)	Percent change	Rate per 100 000	Percent change	Sex ratio
**Measure**		2019	From 1990 to 2019	2019	From 1990 to 2019	2019
Cause	Region	Mean (95% UI)	Mean (95% UI)	Mean (95% UI)	Mean (95% UI)	F:M
	World Bank Lower Middle Income	336·5 (238·6–451·6)	80·8% (76·6 to 85·2%)	11·9 (8·5–15·9)	-15·4% (-17·6 to -13·1%)	1·36
	World Bank Low Income	35·2 (25·2–45·8)	101·8% (93·4 to 110%)	8·5 (6·1–11)	-8·6% (-12·3 to -4·9%)	1·33
Neurological disorders*	Global	65626 (28649·3–122710)	64·9% (58·3 to 81·4%)	831·3 (365·8–1542·4)	2·9% (-0·7 to 6·8%)	1·43
	Southeast Asia, East Asia, and Oceania	17553·3 (7346·6–33447·1)	64·2% (53·2 to 96·7%)	755·8 (321·7–1444·3)	10·4% (5·2 to 17·8%)	1·41
	Central Europe, Eastern Europe, and Central Asia	4202 (2161·2–7257)	11·4% (6·6 to 21·3%)	882·5 (430–1564·3)	0·3% (-3·7 to 4·9%)	1·51
	High-income	11939 (6080·6–21042·8)	35·3% (26·3 to 59·7%)	898·5 (377·4–1685)	1·4% (-1·7 to 5·4%)	1·69
	Latin America and Caribbean	5390·3 (2370·9–10064·2)	63·7% (51·3 to 82·8%)	904·1 (405·6–1677·9)	-1·5% (-9·8 to 5%)	1·52
	North Africa and Middle East	5447·8 (2227·5–10439·2)	99·6% (83·8 to 121·6%)	935 (419·6–1737·7)	0·2% (-6·5 to 8·3%)	1·42
	South Asia	13815·8 (4897·8–26963·1)	87·6% (72·8 to 108·7%)	778·8 (299·7–1483·9)	1·3% (-5·8 to 10·3%)	1·33
	Sub-Saharan Africa	7277·7 (3231·2–13267·4)	131·6% (110·5 to 154·4%)	798·9 (381·9–1413·6)	1% (-5·7 to 9·1%)	1·29
	World Bank High Income	13192·1 (6688·8–23330·1)	36·9% (28 to 60·5%)	901·3 (384·4–1675·8)	1·5% (-1·6 to 5·9%)	1·66
	World Bank Upper Middle Income	22834·6 (10450·8–41922·5)	55·6% (46·3 to 77·1%)	800·9 (363–1486·8)	6·5% (0·5 to 11·7%)	1·45
	World Bank Lower Middle Income	25126 (9332·5–49163·9)	85·8% (75·5 to 101·1%)	821·5 (333·4–1568·3)	0·8% (-3·8 to 6·9%)	1·36
	World Bank Low Income	4435·8 (1953·2–8119·1)	126·5% (99·6 to 155·7%)	747·8 (355·9–1336·9)	0·9% (-7·8 to 10·6%)	1·32
Alzheimer's disease and other dementias	Global	7417·1 (5226·8–9927·7)	164·7% (158·7 to 171%)	98·9 (69·5–132·6)	5·5% (4 to 6·8%)	1·27
	Southeast Asia, East Asia, and Oceania	2316·8 (1631·4–3125)	273% (260·1 to 286·1%)	107 (75·3–144·2)	24·6% (21·7 to 27·4%)	1·31
	Central Europe, Eastern Europe, and Central Asia	679·3 (473·6–924·6)	71% (65·2 to 77·4%)	108·2 (75·6–146·6)	5·2% (3·4 to 6·9%)	1·20
	High-income	2698·2 (1904·5–3596·3)	121·4% (114·1 to 129%)	104 (73·4–138·9)	2·8% (1 to 4·7%)	1·29
	Latin America and Caribbean	504 (356·4–674·7)	244·3% (234·3 to 255·7%)	91·9 (64·9–123·1)	6·5% (4·8 to 8·2%)	1·13
	North Africa and Middle East	352·2 (248·1–474·3)	187·7% (180 to 195·6%)	112·8 (79·1–151·9)	2·8% (0·9 to 4·6%)	1·10
	South Asia	628·3 (444·1–839·5)	228·2% (216·5 to 241·4%)	59·9 (42·4–79·9)	1·6% (-0·1 to 3·3%)	1·04
	Sub-Saharan Africa	238·3 (167·8–321·2)	126·8% (122 to 132%)	79·6 (56·1–106·3)	1·4% (-0·1 to 2·8%)	1·38
	World Bank High Income	2870·9 (2024·6–3822·1)	121·6% (114·2 to 129%)	104·4 (73·7–139·3)	2·8% (1·1 to 4·7%)	1·30
	World Bank Upper Middle Income	3131·1 (2195·7–4219)	229·2% (220·3 to 238·5%)	107·9 (75·7–145·5)	16·3% (14·2 to 18·4%)	1·24
	World Bank Lower Middle Income	1238·5 (877·3–1658·3)	157·4% (152·6 to 162·9%)	72·3 (51–96·6)	-5·1% (-6·6 to -3·6%)	1·16
	World Bank Low Income	173·1 (121·7–234·2)	138·1% (131·1 to 145·6%)	83·9 (59·1–112·4)	1·5% (-0·2 to 3·2%)	1·35
Parkinson's disease	Global	1210·1 (841·2–1640·7)	154·7% (149·4 to 160·9%)	15·1 (10·4–20·3)	16·2% (13·5 to 19%)	0·67
	Southeast Asia, East Asia, and Oceania	502·1 (341–694·5)	238·9% (227·1 to 250·6%)	19·3 (13·3–26·7)	30·8% (26·3 to 35·4%)	0·66
	Central Europe, Eastern Europe, and Central Asia	79·7 (55·1–108)	42·6% (37·5 to 48·7%)	12·5 (8·6–16·9)	2·3% (-1·4 to 6·3%)	0·66
	High-income	344·2 (246·2–459·2)	93·9% (82·4 to 105·6%)	15·3 (10·9–20·4)	6·7% (0·7 to 12·4%)	0·65
	Latin America and Caribbean	73·1 (50·6–99·1)	236% (224 to 248·3%)	12·8 (8·9–17·4)	20·2% (16·4 to 24·2%)	0·63
	North Africa and Middle East	44·5 (30·7–60·5)	197·3% (182·1 to 214·4%)	11·7 (8–15·7)	14·8% (9·2 to 21·2%)	0·73
	South Asia	130·9 (88·8–181·1)	213% (199·7 to 227·7%)	10·1 (6·9–13·8)	16·8% (13·2 to 20·7%)	0·75

Table S10 Global and regional burden, mortality, incidence, and prevalence of neurological conditions in different regions of the world

		All Ages		Age-standardised		
		Number (thousand)	Percent change	Rate per 100 000	Percent change	Sex ratio
**Measure**		2019	From 1990 to 2019	2019	From 1990 to 2019	2019
Cause	Region	Mean (95% UI)	Mean (95% UI)	Mean (95% UI)	Mean (95% UI)	F:M
	Sub-Saharan Africa	35·6 (24·5–48·5)	137·6% (130·9 to 145·1%)	9·1 (6·2–12·2)	10·1% (7·5 to 12·8%)	0·69
	World Bank High Income	370 (264·4–493·4)	95·2% (83·4 to 106·7%)	15·4 (11–20·5)	7·2% (1·1 to 13·2%)	0·66
	World Bank Upper Middle Income	577·9 (392·9–797·8)	212·5% (202·8 to 222·3%)	17·5 (12–24·2)	29·7% (25·7 to 33·8%)	0·65
	World Bank Lower Middle Income	235·9 (160·9–322·2)	165·3% (158·1 to 172·9%)	11 (7·6–14·9)	9·9% (7·4 to 12·5%)	0·74
	World Bank Low Income	25·6 (17·6–35·3)	124·4% (113·6 to 136·3%)	9·4 (6·5–12·7)	3·9% (-0·5 to 9·1%)	0·72
Idiopathic epilepsy	Global	7740·8 (4810·3–11216·7)	43·7% (18·7 to 76·3%)	101·1 (63·1–146·8)	1·4% (-15·4 to 23·2%)	0·91
	Southeast Asia, East Asia, and Oceania	1499 (892·6–2234·9)	32·5% (-3·4 to 85·7%)	72·6 (43·3–106·4)	9·7% (-19·5 to 53%)	0·92
	Central Europe, Eastern Europe, and Central Asia	415 (234·7–633·6)	-4·9% (-26·9 to 20·6%)	99·2 (57–154·3)	-5·2% (-28·4 to 20·9%)	0·87
	High-income	883·3 (484·8–1481·1)	17·8% (-10·1 to 49·4%)	78·1 (41·8–131·1)	-5·1% (-26·5 to 19·4%)	0·95
	Latin America and Caribbean	858 (514·5–1295·1)	19·2% (-9·5 to 60·1%)	147 (88–222·7)	-20·8% (-39·7 to 5·1%)	0·97
	North Africa and Middle East	603·1 (342·3–938·7)	47·5% (-1 to 119%)	101 (57·3–156·5)	-9·3% (-39·8 to 33·8%)	0·87
	South Asia	1827 (1118·8–2751·1)	59·1% (12·2 to 142%)	102·3 (62·6–153·1)	-2·5% (-30·5 to 49·3%)	0·89
	Sub-Saharan Africa	1655·3 (970·1–2541·9)	108·6% (63·5 to 180·3%)	154 (91·2–232)	-5·4% (-25·5 to 26·2%)	0·86
	World Bank High Income	1040·7 (574·3–1729·4)	20·9% (-6·3 to 49·6%)	83·7 (45·4–138·6)	-2·8% (-24·2 to 20·8%)	0·93
	World Bank Upper Middle Income	2311 (1394·8–3430·9)	16·9% (-10·4 to 54%)	90·1 (54–134·7)	-3·9% (-26·6 to 25%)	0·93
	World Bank Lower Middle Income	3419·4 (2089·2–5050·1)	65·3% (27·5 to 124·4%)	108·2 (66·9–159·4)	1·6% (-22·2 to 38·1%)	0·89
	World Bank Low Income	964·8 (524·8–1539·1)	101·9% (47·3 to 202·1%)	133·7 (74–209·7)	-6·6% (-32 to 37·4%)	0·88
Multiple sclerosis	Global	451·2 (320·7–591·5)	71·3% (65·3 to 77·1%)	5·5 (3·9–7·1)	-5·8% (-8·6 to -2·9%)	1·93
	Southeast Asia, East Asia, and Oceania	18·2 (12–25·8)	100·1% (86·8 to 112·7%)	0·7 (0·5–1)	19·9% (18 to 22%)	1·45
	Central Europe, Eastern Europe, and Central Asia	38·6 (27–50·9)	8·5% (4·5 to 12·8%)	7·5 (5·3–9·9)	-4·5% (-7·9 to -1·1%)	1·31
	High-income	264·3 (189·2–338)	57·4% (48·7 to 66·1%)	18·8 (13·5–24·1)	17·3% (11·2 to 23·7%)	2·15
	Latin America and Caribbean	21·2 (14·4–29·1)	158% (146·8 to 170·2%)	3·4 (2·3–4·7)	19·9% (15·6 to 24·4%)	1·91
	North Africa and Middle East	57·7 (40·8–76·8)	169·3% (156·5 to 183·1%)	10 (7·1–13·3)	10·9% (6·1 to 16·3%)	1·88
	South Asia	38·4 (25·5–54)	135·2% (129·9 to 140·7%)	2·2 (1·5–3·1)	14·3% (12·9 to 15·9%)	1·76
	Sub-Saharan Africa	12·8 (8·5–18·3)	157·3% (151·9 to 163·8%)	1·7 (1·2–2·4)	10·7% (8·4 to 13·6%)	2·18
	World Bank High Income	278 (198·7–356·2)	57·6% (49·2 to 65·9%)	17·7 (12·7–22·7)	14·8% (9·1 to 20·7%)	2·16
	World Bank Upper Middle Income	90 (62·6–119·3)	84·4% (77·7 to 91·8%)	2·8 (1·9–3·7)	6·2% (3·1 to 9·5%)	1·64
	World Bank Lower Middle Income	73·2 (49·3–101·7)	112·8% (105·9 to 119·3%)	2·5 (1·7–3·4)	3·2% (0 to 6·1%)	1·71
	World Bank Low Income	9·7 (6·4–13·7)	159·7% (148·4 to 172·6%)	2 (1·3–2·7)	13·6% (8·8 to 18·9%)	1·94
Migraine	Global	42077·7 (6418·4–95645·2)	56·6% (52·6 to 62·1%)	525·5 (78·8–1194)	1·5% (-4·4 to 3·3%)	1·70
	Southeast Asia, East Asia, and Oceania	11650·2 (1650·6–27010·1)	49·5% (41·1 to 59·8%)	488·1 (64·8–1135)	6·7% (-3 to 10·5%)	1·65
	Central Europe, Eastern Europe, and Central Asia	2468·6 (656·6–5214·9)	4·1% (1·7 to 9·5%)	541·2 (131·6–1161·6)	-0·5% (-2·7 to 0·3%)	1·86
	High-income	6617·2 (1167·8–14754·8)	17·2% (13·8 to 28%)	587·6 (89·5–1321·7)	0·6% (-1 to 2·2%)	2·06

Table S10 Global and regional burden, mortality, incidence, and prevalence of neurological conditions in different regions of the world

		**All Ages**		**Age-standardised**		
		Number (thousand)	Percent change	Rate per 100 000	Percent change	Sex ratio
**Measure**		2019	From 1990 to 2019	2019	From 1990 to 2019	2019
Cause	Region	Mean (95% UI)	Mean (95% UI)	Mean (95% UI)	Mean (95% UI)	F:M
	Latin America and Caribbean	3388·3 (455·7–7790·2)	63·7% (56·7 to 91·3%)	557·2 (74·1–1281·5)	2·2% (-0·1 to 4·2%)	1·94
	North Africa and Middle East	3793·2 (645·3–8665·8)	102·1% (93·2 to 125·1%)	601·4 (107–1371·8)	0% (-1·6 to 1·6%)	1·70
	South Asia	9779·9 (1150·3–22319·5)	86·3% (79·3 to 99·4%)	526·3 (66·1–1196·4)	0·6% (-2·2 to 3·6%)	1·54
	Sub-Saharan Africa	4380·3 (668·4–9895·8)	135·7% (132·2 to 138·9%)	461 (81·5–1033·8)	1·2% (-0·2 to 2·3%)	1·54
	World Bank High Income	7368·2 (1321·7–16430·4)	19·6% (16 to 30·6%)	584·6 (90·8–1312·1)	0·5% (-1·4 to 2·2%)	2·02
	World Bank Upper Middle Income	14488 (2455·8–32795·2)	44·8% (37·8 to 53%)	503·1 (78·6–1148·7)	5·7% (-5·5 to 9·7%)	1·75
	World Bank Lower Middle Income	17514·6 (2229·5–40394·2)	84·1% (78·8 to 93·5%)	544 (74·1–1244·9)	0·1% (-2·3 to 1·8%)	1·57
	World Bank Low Income	2682·5 (416·6–6084·3)	130·4% (126·5 to 133·4%)	432·4 (77·5–968·1)	1·4% (0 to 2·4%)	1·58
Tension-type headache	Global	4541·7 (1395·5–14981·3)	57·8% (45·1 to 65·9%)	56·2 (17–188·5)	-2·5% (-5·4 to 1·1%)	1·25
	Southeast Asia, East Asia, and Oceania	1126·6 (354·3–3731·9)	52·5% (26·4 to 74·1%)	46 (13·8–161·6)	1·7% (-5·8 to 14·3%)	1·10
	Central Europe, Eastern Europe, and Central Asia	411·7 (145·4–1182·2)	6·7% (-0·7 to 12·1%)	85·9 (28·8–257·4)	-1·4% (-4 to 1·5%)	1·43
	High-income	846·4 (251·2–2751)	23·9% (12·5 to 31·8%)	68·7 (19·2–230·1)	0% (-4·6 to 2·6%)	1·36
	Latin America and Caribbean	331·7 (95·8–1195·2)	76·7% (50·4 to 99·5%)	54·3 (15·6–196·1)	0·3% (-4·4 to 4·2%)	1·33
	North Africa and Middle East	416·6 (138·3–1196·8)	115·5% (84·9 to 132·3%)	68·1 (22·8–195·5)	1% (-9·5 to 8·7%)	1·28
	South Asia	930·2 (251·8–3573·8)	92·1% (70·8 to 107·2%)	51·7 (14·3–193·5)	0·5% (-6·1 to 7·5%)	1·21
	Sub-Saharan Africa	478·5 (142·5–1776·1)	133·2% (122·4 to 143·2%)	55 (17·7–184·3)	0·2% (-4·5 to 4·7%)	1·27
	World Bank High Income	942·1 (286·1–3013·9)	25·8% (13·1 to 34·1%)	68·5 (19·6–226·6)	-0·5% (-5·8 to 2%)	1·36
	World Bank Upper Middle Income	1594·4 (533·9–4790·4)	46·5% (25·3 to 59·9%)	53·3 (16·9–170)	-1·4% (-7·6 to 7·4%)	1·21
	World Bank Lower Middle Income	1706·7 (486·1–6242·3)	87·7% (70·5 to 98·5%)	54·9 (15·9–191·2)	-1·1% (-5·7 to 4·1%)	1·23
	World Bank Low Income	296·1 (87·9–1098·6)	127·2% (116·2 to 137·7%)	51·6 (16·5–173·5)	0·4% (-4·5 to 5%)	1·27
Motor neuron disease	Global	57·1 (40–76·3)	68·8% (62·4 to 75·6%)	0·7 (0·5–1)	1·9% (0·6 to 3·3%)	0·82
	Southeast Asia, East Asia, and Oceania	11·7 (8–16·4)	47% (36·3 to 59·8%)	0·5 (0·4–0·7)	9·6% (7·8 to 11·7%)	0·93
	Central Europe, Eastern Europe, and Central Asia	3·1 (2·2–4·3)	6·7% (2·5 to 11·4%)	0·7 (0·5–1)	4% (2·4 to 5·7%)	0·99
	High-income	25·7 (17·9–33·9)	77·3% (70·4 to 84·5%)	1·6 (1·1–2·1)	19·3% (15·7 to 23·9%)	0·76
	Latin America and Caribbean	3·2 (2·3–4·4)	77% (65·6 to 90·1%)	0·5 (0·4–0·7)	11·3% (8·7 to 14·2%)	0·95
	North Africa and Middle East	3·3 (2·3–4·6)	103·3% (92·1 to 113·8%)	0·5 (0·4–0·8)	3·6% (2 to 5·1%)	0·86
	South Asia	6·9 (4·7–9·7)	92% (86 to 98·6%)	0·4 (0·3–0·5)	10·3% (8·7 to 12·1%)	0·87
	Sub-Saharan Africa	3 (2–4·2)	129·7% (126·5 to 132·6%)	0·3 (0·2–0·4)	2·5% (1·8 to 3·1%)	0·89
	World Bank High Income	26·6 (18·6–35)	75·3% (68·4 to 82·3%)	1·6 (1·1–2)	17·6% (14·3 to 21·9%)	0·77
	World Bank Upper Middle Income	16·4 (11·3–22·9)	46·9% (37·1 to 58%)	0·6 (0·4–0·8)	10·1% (8·5 to 11·9%)	0·93
	World Bank Lower Middle Income	11·9 (8·1–16·7)	84·2% (78·2 to 90·5%)	0·4 (0·3–0·5)	5·4% (4·6 to 6·3%)	0·89
	World Bank Low Income	2 (1·4–2·8)	119·9% (115·7 to 124%)	0·3 (0·2–0·5)	1·2% (0·1 to 2·4%)	0·89
Other [illegible]	Global	2130·4 (1392·1–3016·4)	93·3% (65·4 to 128·5%)	28·3 (18·4–40·4)	40·6% (20·9 to 64·7%)	0·94

Table S10 Global and regional burden, mortality, incidence, and prevalence of neurological conditions in different regions of the world

		**All Ages**		**Age-standardised**		
		Number (thousand)	Percent change	Rate per 100 000	Percent change	Sex ratio
**Measure**		2019	From 1990 to 2019	2019	From 1990 to 2019	2019
Cause	Region	Mean (95% UI)	Mean (95% UI)	Mean (95% UI)	Mean (95% UI)	F:M
neurological disorders	Southeast Asia, East Asia, and Oceania	428·6 (278·7–604·8)	80% (40·7 to 135·4%)	21·5 (13·5–31·2)	51·9% (19·4 to 96·5%)	0·96
	Central Europe, Eastern Europe, and Central Asia	106 (67·5–154·4)	24% (2·1 to 51·4%)	27·2 (16·6–41·1)	31% (3·6 to 63·2%)	0·94
	High-income	259·8 (170·4–379·6)	56·9% (35 to 81·2%)	24·4 (15·1–37·3)	32·6% (10·7 to 57·2%)	1·04
	Latin America and Caribbean	210·7 (131·4–311)	54·8% (21 to 100·9%)	36·8 (23–54·7)	12·4% (-12·2 to 44·6%)	0·97
	North Africa and Middle East	177·2 (106·1–265·1)	98·6% (41·9 to 177·9%)	29·4 (18–43·9)	25·5% (-8·9 to 71%)	0·92
	South Asia	474·4 (288·6–707)	106·9% (51·2 to 203·4%)	25·8 (15·9–38·1)	33·4% (-1·7 to 88·8%)	0·90
	Sub-Saharan Africa	473·7 (281·7–730·9)	198·7% (135·8 to 295·5%)	38·2 (23·3–57·9)	32·9% (6·3 to 72·7%)	0·90
	World Bank High Income	295·6 (192·6–429·3)	58·5% (36·9 to 81·9%)	25·4 (15·6–38·9)	34·3% (12·6 to 58·7%)	1·02
	World Bank Upper Middle Income	625·8 (401·8–893·3)	56·5% (26·9 to 94·4%)	25·7 (16–37·5)	35·6% (8·3 to 67·3%)	0·96
	World Bank Lower Middle Income	925·8 (574·2–1361·7)	121·5% (74·1 to 192·6%)	28·3 (17·8–41·2)	39·9% (10·7 to 78·9%)	0·91
	World Bank Low Income	281·9 (156·1–448·1)	189·7% (113·1 to 324·2%)	34·5 (19·8–53·6)	32·2% (-0·9 to 88·2%)	0·92
Headache disorders	Global	46619·4 (9772·9–100161·7)	56·7% (52·4 to 62·1%)	581·8 (119·6–1255·6)	1·1% (-4·2 to 2·9%)	1·65
	Southeast Asia, East Asia, and Oceania	12776·9 (2520·4–28023·2)	49·8% (40·9 to 60%)	534·1 (98·6–1189·2)	6·3% (-2·7 to 10·1%)	1·59
	Central Europe, Eastern Europe, and Central Asia	2880·3 (987·5–5766·8)	4·5% (2 to 9·3%)	627·1 (196–1287·8)	-0·6% (-2·6 to 0·3%)	1·79
	High-income	7463·5 (1712·2–15883)	18% (14·5 to 27·9%)	656·3 (135·9–1424·6)	0·5% (-1·3 to 2%)	1·97
	Latin America and Caribbean	3720 (683–8205·9)	64·8% (57·2 to 90·5%)	611·5 (111·4–1348·8)	2·1% (-0·4 to 4·1%)	1·88
	North Africa and Middle East	4209·8 (990·1–9068·3)	103·3% (94·5 to 123·7%)	669·6 (159·1–1431·3)	0·1% (-2·4 to 2·4%)	1·65
	South Asia	10710 (1765·8–23536·8)	86·8% (79·5 to 99·8%)	578 (100·5–1258·7)	0·6% (-2·4 to 3·7%)	1·51
	Sub-Saharan Africa	4858·8 (1002·7–10481·4)	135·4% (130·5 to 138·7%)	516·1 (123·2–1089·1)	1·1% (-0·8 to 2·4%)	1·51
	World Bank High Income	8310·2 (1933–17641·9)	20·2% (16·7 to 30·3%)	653·1 (138·2–1410·8)	0·4% (-1·6 to 2%)	1·94
	World Bank Upper Middle Income	16082·4 (3701·8–34346)	44·9% (37·6 to 52·8%)	556·4 (118·8–1199·2)	4·9% (-5·3 to 9·1%)	1·69
	World Bank Lower Middle Income	19221·3 (3425–41915·3)	84·4% (78·7 to 93·6%)	598·8 (112·2–1302·8)	0% (-2·5 to 1·7%)	1·54
	World Bank Low Income	2978·5 (625·3–6424)	130·1% (125 to 133·4%)	484·1 (115·9–1022·8)	1·3% (-0·8 to 2·6%)	1·54
**YLLs (Years of Life Lost)**						
Meningitis	Global	15649·9 (13103–18930·7)	-52·1% (-60·4 to -42·7%)	225 (187·3–273·1)	-57·7% (-65 to -48·8%)	0·85
	Southeast Asia, East Asia, and Oceania	1055 (918·3–1208·2)	-82·5% (-85·7 to -78·7%)	66·5 (57·3–76·8)	-80·2% (-84 to -75·9%)	0·70
	Central Europe, Eastern Europe, and Central Asia	126·8 (113·6–141·3)	-77·1% (-79·6 to -74%)	34·5 (30·5–39·1)	-77·4% (-80·2 to -74·1%)	0·64
	High-income	129·4 (122·1–136)	-66·8% (-68·7 to -64·9%)	13·7 (12·7–14·7)	-74% (-76 to -72%)	0·78
	Latin America and Caribbean	332·2 (271·7–397·2)	-76·3% (-81·3 to -70·8%)	63·5 (51·6–76·7)	-78·4% (-83 to -73·2%)	0·71
	North Africa and Middle East	376·5 (310·2–457·5)	-72·5% (-79·3 to -64·6%)	64·1 (53–77·6)	-77·2% (-82·4 to -70·9%)	0·89
	South Asia	3457·6 (2935·3–4130)	-64·2% (-71·6 to -55·7%)	204 (172·5–244·6)	-69·9% (-76 to -63·2%)	1·00
	Sub-Saharan Africa	10172·3 (8053·5–12712·2)	-23·6% (-38·7 to -2·8%)	757·7 (617·6–922·1)	-56·9% (-64·6 to -46·4%)	0·80
	World Bank High Income	135·8 (127·8–143·1)	-68·4% (-70·4 to -66·6%)	12·6 (11·7–13·6)	-75·6% (-77·6 to -73·7%)	0·77
	World Bank Upper Middle Income	866·2 (777·8–962·7)	-83% (-85·7 to -80%)	39·4 (35·1–44·3)	-83·2% (-86 to -80·1%)	0·72

Table S10 Global and regional burden, mortality, incidence, and prevalence of neurological conditions in different regions of the world

Measure		All Ages		Age-standardised		
		Number (thousand)	Percent change	Rate per 100 000	Percent change	Sex ratio
		2019	From 1990 to 2019	2019	From 1990 to 2019	2019
Cause	Region	Mean (95% UI)	Mean (95% UI)	Mean (95% UI)	Mean (95% UI)	F:M
	World Bank Lower Middle Income	8968·6 (7480·9–10810·6)	-51% (-59·6 to -39·8%)	284·5 (237·8–343)	-60·4% (-67·2 to -51·9%)	0·89
	World Bank Low Income	5669·5 (4508·8–7103·1)	-35·8% (-48·8 to -19·1%)	645·2 (530·5–782·8)	-61·9% (-68·8 to -53·2%)	0·78
Encephalitis	Global	4315 (3623·2–5928·3)	-46·2% (-58·5 to -17·4%)	59·2 (49·5–80·9)	-55·9% (-65·7 to -33·2%)	0·86
	Southeast Asia, East Asia, and Oceania	624·9 (488·6–726·6)	-54·8% (-66·1 to -27·9%)	38 (28·2–45)	-51·2% (-63·1 to -27·8%)	0·74
	Central Europe, Eastern Europe, and Central Asia	188·1 (161·2–221·7)	-28·5% (-37·5 to -8·1%)	52·9 (44·7–62·9)	-23·3% (-34·3 to 0·2%)	0·78
	High-income	95·2 (79·7–102)	26·7% (5·8 to 38·1%)	8·8 (7·4–9·6)	-0·6% (-15·1 to 9·4%)	0·83
	Latin America and Caribbean	168·3 (136·9–206·6)	-19·5% (-35·5 to 3·2%)	31·5 (25·5–38·8)	-29·7% (-43·9 to -10·8%)	0·80
	North Africa and Middle East	231·3 (179·8–314·1)	9·8% (-25·4 to 58·2%)	38·8 (30·3–52·5)	-21·8% (-45·9 to 9·5%)	1·07
	South Asia	2695·6 (2135·3–4145·7)	-52·7% (-66 to -22·5%)	161·4 (128·7–246·4)	-62·4% (-71·9 to -39·3%)	0·95
	Sub-Saharan Africa	311·6 (239–429·6)	71·8% (23·1 to 124·4%)	26·6 (21·2–35·3)	-7·8% (-30·1 to 14·1%)	0·68
	World Bank High Income	112 (98·1–122)	4·2% (-6·7 to 13%)	9·6 (8·5–10·8)	-20% (-27·8 to -12·7%)	0·83
	World Bank Upper Middle Income	548 (473·7–664·8)	-54·9% (-64·1 to -29·2%)	25·6 (21·9–31·6)	-54·3% (-63·9 to -28·2%)	0·81
	World Bank Lower Middle Income	3360 (2747·2–4819·3)	-47·8% (-61 to -17·6%)	111·5 (91·5–159·6)	-59·2% (-68·9 to -38·4%)	0·88
	World Bank Low Income	293·2 (237·6–373·7)	15·5% (-21·6 to 103·2%)	40 (32·8–48·5)	-32·8% (-51·4 to 7·1%)	0·84
Tetanus	Global	2314·6 (1768·8–3277)	-89·4% (-92·1 to -83·8%)	33·7 (25·6–47·8)	-90·3% (-92·7 to -85·1%)	0·86
	Southeast Asia, East Asia, and Oceania	222·1 (134·6–275·3)	-93·9% (-96·6 to -91·2%)	11·6 (7·6–14·4)	-94·5% (-96·5 to -92·1%)	0·64
	Central Europe, Eastern Europe, and Central Asia	0·9 (0·6–1·9)	-84·3% (-88 to -68·6%)	0·2 (0·1–0·4)	-84·8% (-88·2 to -70·2%)	0·63
	High-income	1·4 (1·1–3)	-83·8% (-87·1 to -66·5%)	0·1 (0·1–0·2)	-88·9% (-91·2 to -77·3%)	0·67
	Latin America and Caribbean	23·4 (14·7–44·1)	-89·7% (-93·9 to -80·4%)	4·5 (2·7–8·7)	-91·5% (-94·9 to -83·5%)	1·07
	North Africa and Middle East	87·4 (53·6–139·5)	-85·8% (-92·9 to -68·7%)	15 (9·2–24·1)	-88·2% (-93·9 to -76·1%)	0·85
	South Asia	827·8 (566–1156·9)	-94·2% (-96 to -91·2%)	50·6 (34·6–71)	-94·3% (-96·2 to -91·5%)	1·00
	Sub-Saharan Africa	1151·6 (809·3–1864·4)	-62·8% (-76·5 to -33·7%)	84·4 (57·6–133·2)	-78·9% (-85·9 to -67·7%)	0·75
	World Bank High Income	3·2 (2–5·2)	-85·9% (-95·3 to -69·1%)	0·2 (0·2–0·4)	-91·3% (-97·3 to -79·1%)	0·60
	World Bank Upper Middle Income	39·9 (29·9–62·4)	-97·7% (-98·3 to -96·1%)	1·6 (1·3–2·4)	-98% (-98·5 to -96·7%)	0·50
	World Bank Lower Middle Income	1512·8 (1109·6–2026·8)	-91·1% (-93·8 to -87·3%)	49 (35·9–65·4)	-92·2% (-94·5 to -89·2%)	0·85
	World Bank Low Income	757·4 (508·5–1317·6)	-74·5% (-83·9 to -46·8%)	82·3 (53·6–138·9)	-84·4% (-89·6 to -71·7%)	0·77
Brain and central nervous system cancer	Global	8530·5 (6625·1–9447·6)	39·7% (-13·7 to 66%)	107·4 (83·1–119)	-10·8% (-43·7 to 4·8%)	0·71
	Southeast Asia, East Asia, and Oceania	2629·6 (1975·7–3089·8)	21·9% (-21·3 to 64·2%)	110·5 (83·3–129·4)	-19·1% (-47·6 to 7·4%)	0·76
	Central Europe, Eastern Europe, and Central Asia	815 (604·1–926·1)	18·4% (-21·2 to 36·2%)	166·9 (125·5–190·2)	5·7% (-29·9 to 21·5%)	0·65
	High-income	1694·8 (1218–1829·5)	29·8% (-17 to 40·7%)	119·6 (88·4–128·4)	-9·2% (-41·3 to -2·1%)	0·66
	Latin America and Caribbean	704·1 (476·3–823·2)	110% (3 to 174·9%)	120 (80·9–140·8)	28·4% (-36·9 to 67·7%)	0·76
	North Africa and Middle East	705·6 (486–836·9)	69·9% (0·4 to 130·4%)	126·4 (86·6–149·3)	-5·6% (-40·2 to 22·6%)	0·86
	South Asia	1371·9 (1074·7–1650·1)	47·5% (-13·9 to 119·2%)	80·4 (63–97)	-1·3% (-38·3 to 34·8%)	0·67
	Sub-Saharan Africa	609·5 (446·6–776·8)	122·6% (19·2 to 259·5%)	61·8 (43·3–75·8)	13·9% (-34·7 to 64·8%)	0·68
	World Bank High Income	1874·2 (1354·3–2033·1)	28·4% (-18·7 to 40%)	120·3 (89·3–129·9)	-11·1% (-43·3 to -3·4%)	0·66
	World Bank Upper Middle Income	3664·6 (2736·9–4153·2)	29·9% (-19·7 to 56·2%)	125 (93–140·9)	-12·6% (-46·1 to 4·7%)	0·76

Table S10 Global and regional burden, mortality, incidence, and prevalence of neurological conditions in different regions of the world

		**All Ages**		**Age-standardised**		
		Number (thousand)	Percent change	Rate per 100 000	Percent change	Sex ratio
**Measure**		2019	From 1990 to 2019	2019	From 1990 to 2019	2019
Cause	Region	Mean (95% UI)	Mean (95% UI)	Mean (95% UI)	Mean (95% UI)	F:M
	World Bank Lower Middle Income	2538·3 (1939·6–2932·5)	59·5% (-6·2 to 114%)	84·4 (64·2–97·3)	0·6% (-36·8 to 27·1%)	0·71
	World Bank Low Income	449 (327·7–574·2)	93·8% (7·6 to 217·6%)	69·4 (50–87)	3·6% (-38·4 to 51·6%)	0·70
Stroke	Global	125490·4 (116154·7–134427·9)	27% (15·3 to 37·8%)	1550 (1434·5–1660·7)	-38% (-43·7 to -32·9%)	0·70
	Southeast Asia, East Asia, and Oceania	57660·2 (51365·6–64077·9)	40·4% (20·1 to 62·6%)	2242·9 (1996·9–2488·9)	-39·4% (-48·9 to -30%)	0·59
	Central Europe, Eastern Europe, and Central Asia	12856·1 (11824·3–13810·1)	-10% (-16·4 to -3·4%)	2075·3 (1910·3–2228·8)	-33·7% (-38·4 to -28·8%)	0·65
	High-income	10552·5 (9480·3–11225·3)	-25·5% (-30·1 to -21·7%)	489·7 (450·5–517·9)	-59·1% (-60·6 to -57·2%)	0·73
	Latin America and Caribbean	5564·9 (5108·9–6048·1)	18% (8·6 to 27·8%)	952·9 (874·5–1035·8)	-52·1% (-55·8 to -48·2%)	0·79
	North Africa and Middle East	6832·3 (6014·8–7787·8)	34·5% (18 to 53·6%)	1586·8 (1407–1782·8)	-35·1% (-42·5 to -25·9%)	0·99
	South Asia	22217·3 (19621·1–24900·1)	67·1% (42·6 to 93·9%)	1581·2 (1396·9–1772·1)	-32·2% (-42·6 to -21·1%)	0·85
	Sub-Saharan Africa	9807·2 (8403·9–11288)	57·4% (35·8 to 82·4%)	2029·7 (1779–2312·4)	-24% (-33·5 to -12·6%)	0·86
	World Bank High Income	12207·9 (11059·2–12975·5)	-24·8% (-29·1 to -21·1%)	533·3 (492·6–563·6)	-58·3% (-60 to -56·1%)	0·72
	World Bank Upper Middle Income	58612 (52137·9–65038·2)	20·8% (4·4 to 38·4%)	1806 (1608·7–2001·1)	-45·4% (-52·5 to -37·8%)	0·61
	World Bank Lower Middle Income	46746·6 (42624·9–50644·2)	61·2% (44·4 to 77·3%)	1988·9 (1817·9–2152·7)	-27·1% (-35·1 to -18·7%)	0·82
	World Bank Low Income	7850·4 (6614·1–9236·6)	56·2% (34·8 to 80·2%)	2395 (2026·2–2794·7)	-22·8% (-32·1 to -12·3%)	0·86
Ischaemic stroke	Global	50349·7 (46232·4–54066·7)	48·1% (32·3 to 61·3%)	636·5 (582–683·1)	-33·4% (-40 to -27·6%)	0·73
	Southeast Asia, East Asia, and Oceania	21954·8 (19316–24774·8)	128·4% (78·2 to 169%)	895·5 (788·4–1006·2)	-9·3% (-29·1 to 7·3%)	0·60
	Central Europe, Eastern Europe, and Central Asia	7994·2 (7300·2–8627·8)	-10·7% (-18·2 to -3·3%)	1257 (1147·5–1356·3)	-37% (-42·2 to -31·9%)	0·71
	High-income	5397·8 (4700·8–5817·2)	-30·8% (-36 to -26·9%)	215·7 (190·5–231·3)	-65·9% (-68 to -64·2%)	0·76
	Latin America and Caribbean	2149·5 (1934·9–2331·4)	32·8% (20·2 to 46·9%)	383·7 (345–416·4)	-52·4% (-56·7 to -47·6%)	0·73
	North Africa and Middle East	3874·1 (3427·8–4371·1)	112·9% (77·1 to 146·1%)	987·9 (880·9–1105·9)	-11·1% (-23·3 to 1·3%)	1·03
	South Asia	6573·8 (5508·5–7883·9)	120·4% (77 to 165·3%)	529·1 (448·4–627·8)	-22% (-37·4 to -5·4%)	0·80
	Sub-Saharan Africa	2405·6 (2097·3–2871·8)	97·8% (68 to 128·2%)	633 (552·8–734·5)	-4·2% (-18·3 to 9·4%)	1·01
	World Bank High Income	6449·2 (5649·4–6947·7)	-29·1% (-34·3 to -25·3%)	248·7 (222·1–266·3)	-64·2% (-66·3 to -62·4%)	0·76
	World Bank Upper Middle Income	26465·8 (23484·9–29270)	64·4% (41 to 85·3%)	836·8 (744·2–923·6)	-31·7% (-41·3 to -23·3%)	0·66
	World Bank Lower Middle Income	15464·8 (13644·3–17321·1)	97·1% (69·4 to 121·8%)	750·6 (667·4–832·4)	-19·1% (-29·9 to -7·4%)	0·84
	World Bank Low Income	1944·6 (1625·6–2349·2)	104·4% (76·9 to 134·4%)	723·5 (608·7–867·2)	-2·6% (-14·5 to 10·4%)	0·93
Intracerebral haemorrhage	Global	65306·2 (60073·8–70392·3)	24% (10·4 to 35·4%)	793·4 (731·2–854·6)	-37·4% (-44·2 to -31·5%)	0·66
	Southeast Asia, East Asia, and Oceania	32513·3 (28980·3–36184·3)	29·5% (9 to 50·2%)	1225·2 (1091·4–1363·2)	-43·9% (-53·1 to -34·9%)	0·58
	Central Europe, Eastern Europe, and Central Asia	4001 (3657·5–4317·3)	-11·8% (-19·8 to -3·7%)	665·9 (608·3–719·7)	-30·8% (-37·1 to -24·4%)	0·55
	High-income	3708·6 (3426·1–3934·9)	-22·9% (-27·5 to -18·9%)	187·7 (176·1–198·5)	-55·1% (-57·3 to -52·9%)	0·61
	Latin America and Caribbean	2475·4 (2258·9–2720)	2·5% (-7·2 to 13·4%)	414·3 (378·4–455·3)	-56·8% (-60·9 to -52·3%)	0·70
	North Africa and Middle East	2520 (2171·8–2943·1)	0·1% (-15·7 to 18·7%)	514 (447·9–593)	-52·9% (-59·7 to -44·9%)	0·91
	South Asia	13161·7 (11226·1–15312·2)	54% (29·8 to 78·9%)	895·1 (764–1041·2)	-36·2% (-46·4 to -25·6%)	0·87
	Sub-Saharan Africa	6926·3 (5854·9–8047·9)	46·6% (25·5 to 71·7%)	1325·9 (1130·9–1532·5)	-30·7% (-40·1 to -20·2%)	0·81

Table S10 Global and regional burden, mortality, incidence, and prevalence of neurological conditions in different regions of the world

		**All Ages**		**Age-standardised**		
		Number (thousand)	Percent change	Rate per 100 000	Percent change	Sex ratio
**Measure**		2019	From 1990 to 2019	2019	From 1990 to 2019	2019
Cause	Region	Mean (95% UI)	Mean (95% UI)	Mean (95% UI)	Mean (95% UI)	F:M
	World Bank High Income	4220·3 (3910·7–4484·5)	-23·3% (-27·9 to -19·5%)	201 (188·4–213·5)	-54·7% (-57 to -52·5%)	0·59
	World Bank Upper Middle Income	28263·8 (24959·5–31809·1)	11·3% (-6·5 to 29·8%)	850·3 (750·8–954·5)	-48·3% (-56·5 to -40·1%)	0·55
	World Bank Lower Middle Income	27363·8 (24525·2–30212·7)	52·1% (34 to 69%)	1094 (980·4–1207·1)	-30·2% (-38·7 to -22%)	0·80
	World Bank Low Income	5416·9 (4555·7–6430)	44·7% (23·9 to 69·2%)	1554·1 (1317·5–1829·1)	-29% (-38 to -18·4%)	0·84
Subarachnoid haemorrhage	Global	9834·5 (8614·6–11416·4)	-19·1% (-30·7 to 12·6%)	120·1 (105·5–139·2)	-57% (-63·3 to -39·8%)	0·82
	Southeast Asia, East Asia, and Oceania	3192·1 (2629·6–3858·1)	-49·7% (-63·2 to -10·6%)	122·1 (100·9–146·7)	-77·1% (-83·2 to -59%)	0·65
	Central Europe, Eastern Europe, and Central Asia	860·9 (773·6–958·1)	9·3% (-5·5 to 26·2%)	152·4 (136·5–172·9)	-11·3% (-23·3 to 3·4%)	0·64
	High-income	1446·1 (1353·8–1514·9)	-6·4% (-11·7 to 1%)	86·2 (81·8–90·9)	-40·1% (-43·1 to -34·3%)	1·00
	Latin America and Caribbean	940 (846·7–1042·8)	37·9% (22·1 to 56·3%)	154·8 (139·4–172)	-31·6% (-39·6 to -22·3%)	1·26
	North Africa and Middle East	438·2 (355·9–559·6)	-41% (-55·3 to 0·5%)	84·8 (69·4–107·1)	-64·9% (-73·3 to -43·4%)	1·03
	South Asia	2481·8 (1550·3–3416·1)	40·4% (9·1 to 141·7%)	157 (96·9–216·2)	-37·6% (-52·2 to 7·1%)	0·92
	Sub-Saharan Africa	475·4 (270·4–1004·3)	64% (20·1 to 134·9%)	70·8 (38·7–149)	-26% (-40·5 to 2·5%)	0·58
	World Bank High Income	1538·3 (1435·5–1616·4)	-6% (-11·1 to 2%)	83·6 (79·1–88·5)	-40·6% (-43·5 to -34·5%)	1·00
	World Bank Upper Middle Income	3882·3 (3309·8–4473·3)	-44·7% (-56·5 to -10·2%)	118·9 (101·9–136·5)	-72·7% (-78·5 to -55·1%)	0·75
	World Bank Lower Middle Income	3918 (2859·2–5185)	23·6% (-1 to 89·9%)	144·4 (105–189·5)	-38·2% (-51·5 to -4·7%)	0·86
	World Bank Low Income	488·9 (266·8–970·4)	47·6% (13·1 to 106·8%)	117·4 (62·8–230·1)	-31·2% (-44·7 to -7·7%)	0·76
Neurological disorders*	Global	32098·4 (18750·1–60829·3)	81·3% (45 to 114·6%)	422·3 (243·2–806·2)	-7·4% (-16·5 to 2%)	0·91
	Southeast Asia, East Asia, and Oceania	8306·5 (4104·8–17907·5)	84·2% (29 to 135·9%)	378·2 (182·9–797·3)	-15% (-30·3 to -1·6%)	0·95
	Central Europe, Eastern Europe, and Central Asia	2378·3 (1313·1–4674·3)	44% (26·9 to 57·8%)	419·5 (248·4–793·8)	1·5% (-5·4 to 8·2%)	0·83
	High-income	9355·8 (4981·4–18342·2)	101·5% (83·9 to 110·9%)	417·1 (251·8–754)	0·6% (-2·7 to 3%)	0·88
	Latin America and Caribbean	2505·7 (1436·3–4794·8)	123·6% (76·1 to 166%)	448·2 (252·5–865·1)	-4% (-12·8 to 4·6%)	0·84
	North Africa and Middle East	1708·2 (1049·4–3130·8)	68·3% (24·8 to 124·2%)	447·2 (241·7–897·3)	-11·5% (-23·1 to 7·1%)	0·97
	South Asia	5253·1 (3618·2–8795·2)	54·2% (9·9 to 127·7%)	411 (249·2–764)	-13·4% (-30·8 to 8·3%)	0·98
	Sub-Saharan Africa	2590·8 (1866·2–3969)	90·4% (53·1 to 143·3%)	484·4 (279·7–929·3)	0·3% (-13·1 to 14·4%)	0·83
	World Bank High Income	10001·5 (5358·4–19600·6)	98·9% (81·1 to 108·8%)	418·4 (253·1–759·7)	-0·4% (-3·5 to 2·2%)	0·88
	World Bank Upper Middle Income	11456·7 (5971·3–23998·3)	80·9% (31·4 to 124·7%)	398·6 (206·9–816·7)	-12·7% (-24·7 to -2·5%)	0·90
	World Bank Lower Middle Income	8899·6 (5902·4–15498·6)	66·9% (28 to 121·2%)	419 (246·6–809·3)	-7·4% (-20·7 to 9·8%)	0·95
	World Bank Low Income	1722·8 (1238–2734·1)	71·3% (35·7 to 125·6%)	479 (269·9–934·7)	-5·9% (-19·8 to 7·1%)	0·87
Alzheimer's disease and other dementias	Global	17859·9 (4374·6–47064·8)	160·3% (145·7 to 181·7%)	239·8 (58·5–627·2)	2·9% (-1·5 to 9·8%)	1·15
	Southeast Asia, East Asia, and Oceania	5448·3 (1303·4–15160·3)	191·2% (155·8 to 241·7%)	254·2 (60·6–679·9)	-1·2% (-12·4 to 14·7%)	1·24
	Central Europe, Eastern Europe, and Central Asia	1413·6 (331·5–3778·6)	72·4% (60·8 to 87·3%)	226·8 (52·9–601·1)	3·9% (-1·7 to 10·9%)	1·06
	High-income	5980·1 (1529·1–15164·3)	130·1% (116·2 to 151·2%)	225·5 (57·2–572·7)	2·3% (-2·1 to 8·3%)	1·15
	Latin America and Caribbean	1417·9 (345·7–3739·7)	236·8% (213·6 to 269·3%)	259·9 (63·7–682·7)	1·4% (-4·8 to 10·1%)	1·02
	North Africa and Middle East	855·9 (207·4–2304·9)	173·2% (151·8 to 232·1%)	274·2 (67·3–727·7)	-2·6% (-9·7 to 17·2%)	1·13

Table S10 Global and regional burden, mortality, incidence, and prevalence of neurological conditions in different regions of the world

		**All Ages**		**Age-standardised**		
		Number (thousand)	Percent change	Rate per 100 000	Percent change	Sex ratio
**Measure**		2019	From 1990 to 2019	2019	From 1990 to 2019	2019
Cause	Region	Mean (95% UI)	Mean (95% UI)	Mean (95% UI)	Mean (95% UI)	F:M
	South Asia	1989·3 (475·2–5545)	272·3% (224·7 to 332·4%)	202·2 (48·4–553·4)	12·8% (-0·4 to 30·6%)	1·07
	Sub-Saharan Africa	754·8 (179·2–2009)	150·1% (129·5 to 173·7%)	265 (65·5–718·3)	10·8% (3 to 21·2%)	1·16
	World Bank High Income	6373·3 (1628·7–16173·3)	128·9% (115·3 to 149·9%)	227·2 (57·7–578·6)	2% (-2·3 to 7·7%)	1·15
	World Bank Upper Middle Income	7234·2 (1726·7–19986·6)	182·2% (154·8 to 221·1%)	251·7 (61·2–676·3)	0·4% (-8·3 to 11·8%)	1·15
	World Bank Lower Middle Income	3716·8 (849·7–10334)	185·8% (162·5 to 221·9%)	225·8 (54·2–616·6)	6·1% (-2·1 to 20·2%)	1·12
	World Bank Low Income	526·4 (126·6–1428·7)	152·4% (130·6 to 178·4%)	264·8 (65–719·1)	6·9% (-1·2 to 17·6%)	1·17
Parkinson's disease	Global	5082·5 (4673·4–5409·9)	123·5% (105·6 to 139·9%)	64·9 (59·6–69·2)	-0·3% (-7·9 to 6·5%)	0·54
	Southeast Asia, East Asia, and Oceania	1542·8 (1344·6–1723)	120·5% (87·5 to 156·6%)	64·4 (56·2–71·7)	-16·2% (-28·2 to -3·1%)	0·53
	Central Europe, Eastern Europe, and Central Asia	387·5 (352·4–416·7)	51·9% (41·5 to 64·3%)	60·3 (54·8–64·9)	5·5% (-1·4 to 14%)	0·55
	High-income	1477·8 (1329·1–1560·9)	110·7% (100·4 to 117·8%)	61·2 (55·6–64·3)	10·6% (6·4 to 13·8%)	0·45
	Latin America and Caribbean	339·3 (304·1–371·3)	203·1% (180·1 to 229·6%)	61·4 (55–67·2)	2·6% (-4·9 to 11·3%)	0·59
	North Africa and Middle East	256·2 (225·1–319·5)	158·7% (125·3 to 202·8%)	72·7 (63·9–91·5)	-1% (-13·1 to 15·8%)	0·67
	South Asia	853·8 (737·9–984·5)	178·7% (127·6 to 233·8%)	72 (62·1–83)	-5·2% (-22 to 13·8%)	0·66
	Sub-Saharan Africa	225·1 (198·3–264·3)	123·2% (90·2 to 157·9%)	66·5 (58·5–78·7)	6·8% (-8 to 22·5%)	0·66
	World Bank High Income	1572·3 (1416·3–1660)	108·7% (98·3 to 115·9%)	61·2 (55·6–64·4)	9·8% (5·5 to 13·2%)	0·46
	World Bank Upper Middle Income	1931·9 (1721·3–2117·2)	116% (90·1 to 143·6%)	62 (55·4–67·9)	-12·7% (-22·8 to -2·4%)	0·54
	World Bank Lower Middle Income	1410·3 (1268·9–1552·1)	158·5% (119·6 to 192·9%)	72·4 (65·4–79·8)	4·5% (-10·6 to 17·8%)	0·65
	World Bank Low Income	165·2 (142–196·2)	106·6% (78·8 to 135·8%)	67·5 (57·8–81·3)	-3·2% (-15·3 to 9·3%)	0·63
Idiopathic epilepsy	Global	5336·8 (4722·7–6170)	-9·5% (-24·1 to 27·5%)	69·5 (61·7–80·6)	-33·6% (-44 to -7·6%)	0·71
	Southeast Asia, East Asia, and Oceania	760·6 (662·3–891·8)	-45·6% (-55·9 to -27·5%)	35·4 (31–41·6)	-54·6% (-63 to -39·5%)	0·57
	Central Europe, Eastern Europe, and Central Asia	289·5 (262–319·4)	-6·9% (-15·3 to 2·2%)	71·2 (64·2–79·4)	-5·3% (-14·2 to 4·9%)	0·57
	High-income	411·9 (369–428·9)	9·5% (-5·5 to 14·5%)	34·4 (31·4–35·7)	-15·2% (-25·7 to -11·3%)	0·67
	Latin America and Caribbean	429 (378–491·3)	2·8% (-11·3 to 22·9%)	72·8 (64·1–83·7)	-29·5% (-38·9 to -16·5%)	0·63
	North Africa and Middle East	352·2 (279·1–412·5)	-18·1% (-37·2 to 39%)	57·3 (45·6–67·2)	-44·4% (-55·6 to -11·7%)	0·89
	South Asia	1870·9 (1541·6–2310·1)	-15·8% (-36·2 to 41·5%)	105·1 (86·1–129·8)	-44·3% (-56·5 to -11%)	1·06
	Sub-Saharan Africa	1222·9 (989·8–1571·6)	64·5% (25·6 to 161·4%)	116·2 (95·9–149·2)	-19% (-36·2 to 16·4%)	0·42
	World Bank High Income	497·9 (450·8–524·5)	11% (-2·3 to 18·3%)	37·5 (35–39·7)	-14·9% (-24·1 to -9·2%)	0·66
	World Bank Upper Middle Income	1238 (1109·5–1388·7)	-38·1% (-46·8 to -21·7%)	47·5 (42·6–53·7)	-48% (-55·2 to -33·7%)	0·62
	World Bank Lower Middle Income	2782·5 (2339·8–3471)	-3·1% (-24·4 to 58·3%)	88·1 (73·6–109·4)	-36·6% (-49·3 to -2·5%)	0·83
	World Bank Low Income	815·1 (680·3–999·6)	41·8% (7·7 to 133·6%)	115·9 (98–140·1)	-25·8% (-40·9 to 10·6%)	0·53
Multiple sclerosis	Global	708·6 (645·1–897·8)	53·2% (35·6 to 73·7%)	8·5 (7·7–10·7)	-17·4% (-27·9 to -7·6%)	1·38
	Southeast Asia, East Asia, and Oceania	89·9 (74–120·5)	38·9% (3·5 to 112·3%)	3·3 (2·7–4·3)	-26·3% (-44·6 to 11·3%)	0·91
	Central Europe, Eastern Europe, and Central Asia	94·2 (67·2–173·7)	-12·2% (-32·5 to 35·5%)	17·9 (12·7–33·2)	-25% (-42·8 to 17·7%)	1·32

Table S10 Global and regional burden, mortality, incidence, and prevalence of neurological conditions in different regions of the world

		All Ages		Age-standardised		
		Number (thousand)	Percent change	Rate per 100 000	Percent change	Sex ratio
**Measure**		2019	From 1990 to 2019	2019	From 1990 to 2019	2019
Cause	Region	Mean (95% UI)	Mean (95% UI)	Mean (95% UI)	Mean (95% UI)	F:M
	High-income	285·7 (218·6–353·6)	49% (3·4 to 64·9%)	17·7 (13·8–22·2)	-0·8% (-28 to 8·6%)	1·58
	Latin America and Caribbean	38·1 (31·3–48·4)	159·1% (87·9 to 205·8%)	6·2 (5·1–7·8)	19·1% (-14·2 to 40·9%)	1·24
	North Africa and Middle East	58·2 (45·2–80·4)	126·5% (55·9 to 238%)	9·9 (7·8–13·6)	1·8% (-27·9 to 52·7%)	1·34
	South Asia	105·7 (86·3–134·8)	133·9% (73·8 to 248·1%)	6·3 (5·2–8)	8·9% (-17·9 to 63·7%)	1·32
	Sub-Saharan Africa	36·9 (29·3–49·7)	177·6% (95·4 to 276·3%)	5·5 (4·4–7·4)	16·2% (-15·9 to 57·7%)	1·57
	World Bank High Income	313·2 (244·2–390·5)	40·7% (1·4 to 56·4%)	17·7 (14·1–22·7)	-7·4% (-30·9 to 6·1%)	1·58
	World Bank Upper Middle Income	181·4 (152·5–250·9)	32·8% (9·4 to 72·7%)	5·5 (4·6–7·5)	-25·9% (-38·7 to -5·1%)	1·17
	World Bank Lower Middle Income	189·2 (160·3–245·8)	103·4% (62·1 to 169·6%)	6·5 (5·5–8·4)	-1·4% (-20·3 to 29·8%)	1·28
	World Bank Low Income	24·5 (17·4–34·1)	140·7% (74·1 to 215·9%)	5·4 (3·8–7·4)	5·4% (-20·8 to 37·8%)	1·47
Motor neuron disease	Global	977·5 (926·3–1025·4)	65·5% (54·5 to 78·2%)	11·9 (11·3–12·5)	-4·9% (-10·7 to 1·9%)	0·71
	Southeast Asia, East Asia, and Oceania	122·3 (106·7–138)	-21·6% (-34·6 to -6·5%)	5 (4·4–5·6)	-47·7% (-56·3 to -37·9%)	0·59
	Central Europe, Eastern Europe, and Central Asia	51·8 (46·9–56·6)	92·3% (63·1 to 118·2%)	9·4 (8·5–10·3)	49·8% (28·4 to 69·8%)	0·67
	High-income	603·8 (569·7–631·1)	88·3% (79·2 to 96·4%)	35·2 (33·4–36·7)	12·6% (7·6 to 17·6%)	0·70
	Latin America and Caribbean	86 (76–95·3)	232·6% (186·7 to 276·3%)	14·4 (12·7–16)	72·9% (49·2 to 94·7%)	0·68
	North Africa and Middle East	38·3 (30·6–47·1)	18·4% (-32·8 to 87·7%)	7·3 (5·8–9)	-17·8% (-47·1 to 19·1%)	0·72
	South Asia	65·7 (51·4–81·3)	180·6% (124·6 to 242·8%)	4·1 (3·2–5·1)	50·2% (18·9 to 89·1%)	0·80
	Sub-Saharan Africa	9·6 (8–11·8)	79·6% (36·9 to 127%)	1·2 (1–1·5)	-20·1% (-41 to 5·6%)	1·14
	World Bank High Income	619·4 (584–648·6)	87·2% (77·9 to 95·6%)	33·3 (31·5–34·8)	11·7% (6·6 to 16·7%)	0·71
	World Bank Upper Middle Income	244·6 (223·5–266·1)	15·8% (-1·1 to 35·2%)	8 (7·3–8·7)	-24% (-34·9 to -11·7%)	0·64
	World Bank Lower Middle Income	104·2 (90·2–120·2)	146·8% (106·9 to 189%)	3·8 (3·2–4·3)	33·4% (9·9 to 59·4%)	0·77
	World Bank Low Income	8·8 (7–10·8)	46·1% (4·5 to 88·5%)	1·7 (1·3–2·1)	-16·9% (-36·8 to 3·8%)	1·05
Other neurological disorders	Global	2133 (1915·2–2381·1)	31·4% (16·2 to 49·6%)	27·7 (24·7–31)	-8·9% (-19·1 to 3·5%)	0·72
	Southeast Asia, East Asia, and Oceania	342·6 (298·8–398·1)	6·8% (-12·2 to 28·6%)	16 (14–18·5)	-16% (-30·8 to 1·1%)	0·56
	Central Europe, Eastern Europe, and Central Asia	141·8 (129·6–154·2)	8·2% (-1·8 to 18·3%)	33·9 (30·8–36·9)	3·5% (-6·6 to 13·6%)	0·61
	High-income	596·5 (572·9–615·1)	31·5% (27·2 to 35·9%)	43·2 (41·7–44·7)	-11·8% (-15·6 to -8·3%)	0·66
	Latin America and Caribbean	195·4 (176·4–216·1)	50·7% (35·2 to 67·7%)	33·5 (30·1–37·2)	-0·4% (-10·8 to 10·7%)	0·66
	North Africa and Middle East	147·4 (127·9–171·8)	28·8% (-3·1 to 67·3%)	25·7 (22·4–29·9)	-9·5% (-30 to 15·6%)	0·61
	South Asia	367·8 (266·2–505·5)	32·8% (12·5 to 85·8%)	21·3 (15·5–29·2)	-4·3% (-21·6 to 28%)	1·04
	Sub-Saharan Africa	341·4 (265·4–436·3)	74·4% (23·8 to 135·5%)	30 (24·4–36·7)	-6·5% (-30·4 to 20·9%)	0·86
	World Bank High Income	625·4 (599·7–645·9)	28·2% (23·9 to 32·5%)	41·5 (39·8–43)	-14·2% (-18·1 to -10·8%)	0·66
	World Bank Upper Middle Income	626·6 (576–676·8)	18·5% (4·4 to 34·7%)	23·9 (21·9–25·9)	-6% (-17·4 to 6·9%)	0·62
	World Bank Lower Middle Income	696·6 (538·8–895·5)	45·5% (17·2 to 86·2%)	22·5 (17·5–28·7)	1·1% (-18·5 to 26·2%)	0·89

Table S10 Global and regional burden, mortality, incidence, and prevalence of neurological conditions in different regions of the world

		All Ages		Age-standardised		
		Number (thousand)	Percent change	Rate per 100 000	Percent change	Sex ratio
**Measure**		2019	From 1990 to 2019	2019	From 1990 to 2019	2019
Cause	Region	Mean (95% UI)	Mean (95% UI)	Mean (95% UI)	Mean (95% UI)	F:M
	World Bank Low Income	182·9 (146·3–227·5)	45·1% (9·8 to 99·1%)	23·7 (19·2–29·4)	-16·7% (-35·4 to 7·9%)	0·78

Data in parentheses are 95% uncertainty intervals. Count data in thousands and percentage data are rounded to one decimal place, and sex ratio is rounded to two decimal place·. Percentages and number of DALYs, YLDs, YLLs, deaths, incident cases, and prevalent cases are not mutually exclusive: the sum of percentages and number of DALYs, YLDs, YLLs, deaths, incident cases, and prevalent cases in the columns exceeds the totals for all causes combined because of overlap between various causes. Measures with insufficient data are not reported here. DALYs Disability-Adjusted Life Years; F:M, Female to male ratio; NA, no data available; UI uncertainty interval; YLDs Years Lived with Disability; YLLs Years of Life Lost; *Stroke include ischaemic stroke, intracerebral haemorrhage, and subarachnoid haemorrhage; †Neurological disorders include Alzheimer's disease and other dementias, Parkinson's disease, idiopathic epilepsy, multiple sclerosis, headache disorders (including migraine and tension-type headache), motor neuron disease, and other neurological disorders.

Table S11 Cause- and sex-specific burden, mortality, incidence, and prevalence of neurological conditions in North Africa and Middle East countries
(NEXT PAGE)

Disability-Adjusted Life Years 144
Deaths 152
Incidence 159
Prevalence 168
Years Lived with Disability 178
Years of Life Lost 187

Table S11 Cause- and sex-specific burden, mortality, incidence, and prevalence of neurological conditions in North Africa and Middle East countries

		All Ages		Age-standardised		
		Number (thousand)	Percent change	Rate per 100,000	Percent change	Sex ratio
**Measure**		2019	From 1990 to 2019	2019	From 1990 to 2019	2019
Cause	Location	Mean (95% UI)	Mean (95% UI)	Mean (95% UI)	Mean (95% UI)	F:M
**DALYs (Disability-Adjusted Life Years)**						
Meningitis	Global	16333·2 (13775·1–19609·8)	-51·3% (-59·4 to -42%)	234 (195·8–282·5)	-57·2% (-64·4 to -48·6%)	0·86
	North Africa and Middle East	412·8 (345·2–495)	-70·5% (-77·5 to -62·3%)	70 (58·7–83·7)	-75·8% (-81·1 to -69·5%)	0·91
	Algeria	16·2 (13·3–20·1)	-74·7% (-84·2 to -61·1%)	40 (33·1–49·1)	-80·2% (-87 to -70·7%)	1·01
	Bahrain	0·2 (0·2–0·2)	-42·7% (-55·7 to -26·8%)	18·6 (15·8–21·8)	-74·5% (-79·6 to -67·8%)	0·77
	Egypt	54·6 (37·5–74·3)	-68·1% (-78·3 to -54·2%)	54 (37·4–73·2)	-75·7% (-83·3 to -65·6%)	0·7
	Iran	30·1 (26·2–34·4)	-79% (-84·1 to -71·6%)	38·6 (33·5–44·3)	-79·2% (-84·1 to -72·5%)	0·94
	Iraq	28·4 (22·2–36·3)	-69·6% (-79 to -56·4%)	67·4 (53·4–84·6)	-80% (-85·7 to -72·4%)	0·91
	Jordan	5·9 (4·7–7·5)	5% (-25·9 to 50·7%)	52·6 (42·4–66·4)	-56·5% (-68 to -39·5%)	0·98
	Kuwait	0·7 (0·6–0·9)	-22% (-40·2 to -0·4%)	20·1 (16·2–24·7)	-63·1% (-71·8 to -53·2%)	0·67
	Lebanon	1·5 (1·2–2)	-58·8% (-72·1 to -40·5%)	29·5 (22·2–38·5)	-68·6% (-78 to -56·1%)	0·8
	Libya	2·2 (1·7–2·8)	-68% (-78·4 to -53·9%)	36·2 (28·4–45·8)	-69·9% (-78·5 to -58·4%)	0·94
	Morocco	22·1 (15·5–30)	-82·9% (-89·4 to -74·2%)	68·4 (47·7–94·1)	-82% (-88·5 to -73·2%)	0·74
	Palestine	2 (1·6–2·4)	-75% (-83·4 to -62%)	40·5 (34·1–48·3)	-82·6% (-87·6 to -75·4%)	0·79
	Oman	0·7 (0·6–0·9)	-63·1% (-74·1 to -46·1%)	24·5 (20·8–29·5)	-73·9% (-80·2 to -63·3%)	1·35
	Qatar	0·5 (0·4–0·6)	34·9% (-9·8 to 93·7%)	25·4 (20·7–31·2)	-73·1% (-81·8 to -62·7%)	2·08
	Saudi Arabia	8·5 (6·6–11·1)	-41·8% (-56·5 to -19·7%)	26·7 (21·3–33·5)	-73·3% (-79·4 to -63·2%)	1·22
	Syrian Arab Republic	13·5 (10·4–17·2)	-80·1% (-86·4 to -71·2%)	100 (76·6–129·5)	-70·7% (-79·4 to -58·5%)	0·93
	Tunisia	4·1 (3–5·2)	-76·3% (-84·4 to -64·1%)	37·9 (28·2–48·2)	-77·1% (-84·8 to -66·2%)	0·72
	Türkiye	17·2 (14·4–20·6)	-90·8% (-94·3 to -85·7%)	25·9 (21·4–31·9)	-89·9% (-93·7 to -84·5%)	0·77
	United Arab Emirates	2·8 (1·8–4·1)	26·4% (-21·2 to 80·3%)	32·1 (22·2–43·8)	-73·9% (-81·3 to -65·7%)	0·74
	Yemen	33·1 (22·3–48·2)	-55·4% (-71·8 to -29·7%)	97·4 (68·3–136·9)	-68·5% (-78·6 to -54·1%)	1·04
	Afghanistan	121·3 (88·6–171·7)	-30·5% (-55 to 6·9%)	244·7 (184·6–325·7)	-73·6% (-82·1 to -62·4%)	1·12
	Sudan	46·7 (30·9–71·1)	-79·9% (-87·4 to -69%)	100·3 (68·9–144·1)	-85·1% (-90·2 to -77·6%)	0·75
Encephalitis	Global	4797·4 (4059·5–6418·1)	-43·4% (-55·8 to -15·8%)	65·3 (55·1–87·3)	-54·3% (-63·9 to -32·9%)	0·87
	North Africa and Middle East	251 (200·2–332·9)	13·5% (-21 to 59·6%)	42 (33·6–55·6)	-20·7% (-43·3 to 8·7%)	1·09
	Algeria	8·3 (5·9–14·7)	-2·5% (-38·8 to 49·8%)	20·1 (14·4–35·4)	-28·7% (-52·4 to 3·6%)	0·98
	Bahrain	0·1 (0·1–0·2)	69·5% (28·5 to 110·5%)	11·4 (9·2–13·8)	-25·8% (-43·7 to -8·4%)	0·98
	Egypt	65 (33·5–102·5)	-4·1% (-43·1 to 56%)	62·8 (33·5–98·7)	-31·9% (-58·4 to 4·5%)	0·87
	Iran	12·7 (10·2–14·6)	-13·4% (-37·5 to 14·3%)	16·3 (12·8–19)	-22·1% (-41·5 to -0·8%)	0·93
	Iraq	30·4 (21·1–40·4)	17·8% (-27·9 to 88·4%)	70·5 (50·3–92·5)	-33·8% (-56·9 to -2%)	1·11
	Jordan	1·3 (1–1·7)	2·4% (-35·5 to 154·6%)	10·9 (8·6–14·6)	-60·2% (-74·1 to -9·7%)	1·24
	Kuwait	0·4 (0·3–0·5)	54·1% (26·4 to 92%)	10·5 (8·6–13·3)	-26·5% (-40·1 to -8·6%)	1·27
	Lebanon	0·9 (0·6–1·6)	14·4% (-22 to 59·7%)	17·5 (11·8–30·6)	-19·2% (-42·9 to 10·2%)	1·03
	Libya	1 (0·7–1·9)	5·4% (-30·5 to 52·9%)	17·4 (11·9–31·6)	-11·6% (-37·5 to 25·4%)	0·99
	Morocco	8·6 (5·3–16·3)	-6·2% (-42·2 to 41·4%)	26 (15·6–48·9)	-16·9% (-45·8 to 23·8%)	1·42
	Palestine	0·8 (0·6–1·1)	51·1% (-4·7 to 118·9%)	16·3 (13·1–22·1)	-21·8% (-43·6 to 3·1%)	1·19
	Oman	4·5 (3·3–7·8)	7·8% (-31·4 to 67·1%)	122·1 (94–180·3)	-44·1% (-62·4 to -14·1%)	0·67
	Qatar	0·3 (0·2–0·4)	264·1% (136·5 to 425·7%)	13·3 (9·9–17·6)	-29·3% (-52·7 to 0·9%)	1·34
	Saudi Arabia	9·5 (7·1–12·8)	2·3% (-28·7 to 51·7%)	28·2 (21·6–37·8)	-53% (-68·2 to -29·9%)	1·27
	Syrian Arab Republic	3·7 (2·9–4·9)	-52·8% (-70·3 to -17·7%)	27·4 (21·1–36·6)	-41% (-61·4 to -1·9%)	0·96
	Tunisia	2 (1·3–3·7)	-18·8% (-49·5 to 18·9%)	18·4 (12·1–34·4)	-28·7% (-54·5 to 2·7%)	0·93
	Türkiye	13·2 (10·8–16)	-42·9% (-64·2 to -15%)	18·6 (14·9–22·9)	-47·4% (-66·5 to -22·2%)	0·97
	United Arab Emirates	1·5 (0·9–3)	225·1% (113·7 to 388%)	18·4 (11·9–34·1)	-19·5% (-44·4 to 18%)	0·76
	Yemen	11·4 (6·7–21·4)	52·7% (-12·4 to 168·5%)	31·3 (19·4–57·9)	-7·1% (-41·7 to 47·1%)	1·19
	Afghanistan	60·2 (39·1–116·1)	156·9% (74 to 263·1%)	175·8 (131·7–248·4)	-14·2% (-38·4 to 17·5%)	1·44
	Sudan	14·9 (8·7–27·4)	15·7% (-37·4 to 120·1%)	32·1 (19·6–58·4)	-22·1% (-53·8 to 30·3%)	1·07
Tetanus	Global	2316·4 (1770–3279·4)	-89·4% (-92·1 to -83·8%)	33·7 (25·6–47·9)	-90·2% (-92·7 to -85·1%)	0·86

Table S11 Cause- and sex-specific burden, mortality, incidence, and prevalence of neurological conditions in North Africa and Middle East countries

**Measure**		**All Ages**		**Age-standardised**		
		Number (thousand)	Percent change	Rate per 100,000	Percent change	Sex ratio
		2019	From 1990 to 2019	2019	From 1990 to 2019	2019
Cause	Location	Mean (95% UI)	Mean (95% UI)	Mean (95% UI)	Mean (95% UI)	F:M
	North Africa and Middle East	87·4 (53·6–139·5)	-85·8% (-92·9 to -68·7%)	15 (9·2–24·1)	-88·2% (-93·9 to -76·1%)	0·85
	Algeria	0·5 (0·4–0·9)	-83% (-93·5 to -41·9%)	1·3 (0·9–2·3)	-86·7% (-94·8 to -60·7%)	0·79
	Bahrain	<0·1 (<0·1–<0·1)	37·3% (-40·9 to 115%)	0·7 (0·6–1·2)	-39·3% (-75 to -6·6%)	1·00
	Egypt	7·4 (2·2–22)	-87·3% (-96·4 to -60·1%)	7·7 (2·5–23·2)	-90·8% (-97 to -71·7%)	0·69
	Iran	0·9 (0·7–1·3)	-94·4% (-97·6 to -82·1%)	1·2 (0·9–1·7)	-94·1% (-97·4 to -82·3%)	0·74
	Iraq	1·8 (1–3)	-91·2% (-97·3 to -53·5%)	4 (2·3–6·7)	-93·8% (-98 to -72·8%)	0·56
	Jordan	0·1 (0·1–0·2)	-71·7% (-86·8 to -12·3%)	1 (0·7–1·5)	-85·7% (-93·3 to -59%)	0·72
	Kuwait	<0·1 (<0·1–<0·1)	86·7% (-69·6 to 628·3%)	<0·1 (<0·1–0·1)	-46·1% (-88·3 to 68%)	0·1
	Lebanon	0·8 (0·2–2·7)	-57·9% (-93 to 27·8%)	14·9 (3·2–50·9)	-75·8% (-95·7 to -29·1%)	1·11
	Libya	0·1 (0–0·1)	-69·7% (-88·1 to -8·1%)	1·2 (0·7–2)	-70·5% (-89·8 to -19%)	0·72
	Morocco	7·7 (1·9–28·9)	-95% (-98·8 to -80·8%)	25·7 (6·3–98·1)	-93·9% (-98·5 to -76·2%)	0·84
	Palestine	0·1 (0·1–0·1)	-90·5% (-97·4 to -52·5%)	1·3 (0·9–1·9)	-92·7% (-98 to -67·8%)	0·71
	Oman	<0·1 (<0·1–0·1)	-79·7% (-93·8 to -19·4%)	1 (0·7–1·3)	-90·3% (-96·7 to -62·2%)	0·77
	Qatar	<0·1 (<0·1–<0·1)	195·2% (8·4 to 463·3%)	0·7 (0·5–1)	-50·2% (-83·3 to 5%)	0·91
	Saudi Arabia	0·7 (0·2–1·3)	-94·2% (-99·2 to -68·2%)	2·1 (0·8–3·5)	-96·5% (-99·4 to -81·2%)	0·58
	Syrian Arab Republic	0·3 (0·2–0·6)	-97·6% (-99·2 to -89·5%)	2·4 (1·3–5)	-95·6% (-98·6 to -81·4%)	0·52
	Tunisia	0·1 (0·1–0·2)	-83·7% (-94 to -56·6%)	1 (0·7–1·8)	-83·8% (-94 to -57·1%)	0·8
	Türkiye	0·7 (0·5–1)	-98% (-98·9 to -94·1%)	1 (0·8–1·4)	-98·3% (-99 to -95·5%)	0·83
	United Arab Emirates	1·1 (0·5–1·7)	-8% (-83·2 to 209·2%)	33·2 (8·4–54·9)	-86% (-96·4 to -46·5%)	1·79
	Yemen	4·2 (1·7–8·8)	-85·7% (-95·7 to -34·5%)	11·2 (5–23·3)	-89·9% (-96·5 to -61·6%)	0·51
	Afghanistan	58·1 (32·9–101·7)	-74·6% (-88·5 to -32·2%)	113·9 (70·5–179·3)	-90·2% (-94·9 to -79·6%)	1·03
	Sudan	2·7 (1·1–5·6)	-92·9% (-97·4 to -77·6%)	5·6 (2·6–12·1)	-94·3% (-97·8 to -83·9%)	0·48
Brain and central nervous system cancer	Global	8659·9 (6718–9574·5)	40·5% (-13·2 to 66·9%)	109 (84·6–120·9)	-10·4% (-43·5 to 5·3%)	0·71
	North Africa and Middle East	716·3 (493·9–848·2)	71% (0·9 to 131·7%)	128·3 (87·8–151·3)	-5% (-40 to 23·4%)	0·86
	Algeria	23·7 (14·3–30·2)	56·3% (-16·5 to 120·5%)	58·3 (35·3–73·9)	-6% (-49·9 to 28·7%)	0·98
	Bahrain	1 (0·6–1·4)	186·4% (67·5 to 302·3%)	73 (46·7–93·3)	-25% (-55·8 to 6·5%)	0·83
	Egypt	89·1 (61·4–125·5)	73·5% (-5·9 to 153·7%)	98·8 (68·1–141·1)	-1·8% (-40·8 to 39·9%)	0·83
	Iran	128·5 (67·7–153·6)	40·5% (-27·9 to 81·4%)	156·4 (82–187)	-10·7% (-50·1 to 8·5%)	0·89
	Iraq	79·9 (57·7–103·2)	164·2% (32 to 325·9%)	225·6 (162·9–289)	15·2% (-39·2 to 79·6%)	1·02
	Jordan	9·7 (7–12)	202·2% (91·8 to 309·7%)	97·8 (69·5–120·6)	-10·2% (-43·7 to 22·2%)	0·91
	Kuwait	2·5 (1·7–3·1)	137·7% (45·9 to 204·1%)	66·2 (47–82·8)	-8·6% (-44·1 to 16·6%)	0·65
	Lebanon	6·1 (4·3–7·9)	59·8% (7·6 to 118·8%)	117·3 (82·6–151·5)	-9% (-38·6 to 23·8%)	0·79
	Libya	8·6 (6·3–11·3)	71·6% (16·1 to 161·2%)	134·4 (99·2–175·6)	-4·6% (-32·6 to 37·1%)	0·63
	Morocco	19·6 (13·1–27·1)	56·9% (-9·5 to 134·5%)	55·2 (37·4–74·2)	2·6% (-37·2 to 47·8%)	1·38
	Palestine	8·9 (6·9–11)	114·7% (31·2 to 200·5%)	232 (175·6–279·5)	-9·1% (-39·8 to 24·2%)	0·82
	Oman	2·5 (1·5–3·3)	151·6% (26·6 to 282·6%)	72·6 (43·8–91·3)	11% (-44·4 to 65·5%)	0·97
	Qatar	1·7 (1·1–2·8)	433·1% (209 to 716·6%)	85·5 (61·1–128·4)	-20·3% (-53·1 to 20·6%)	1·24
	Saudi Arabia	26·5 (19·2–39·8)	250·7% (91·7 to 558·9%)	79·4 (59·2–120·9)	32·3% (-29·6 to 148·9%)	0·92
	Syrian Arab Republic	20·4 (14·7–27·5)	34·4% (-23 to 107·6%)	144·8 (104·7–194·1)	-6·8% (-38·4 to 37·2%)	0·83
	Tunisia	5·1 (3·4–7)	44·3% (-15·1 to 113·1%)	41·8 (27·6–57·1)	-5·4% (-42·2 to 37·8%)	1·19
	Türkiye	132·9 (63·5–183·7)	24·5% (-34·5 to 93·9%)	158·3 (76·7–215·5)	-20·4% (-55·4 to 19·1%)	0·77
	United Arab Emirates	14·3 (8·2–21·1)	462% (270·9 to 714·7%)	151·5 (91·5–206·7)	-10·7% (-45·3 to 30·6%)	0·8
	Yemen	31·6 (18·7–46·4)	137% (23·7 to 365·4%)	123·3 (74·9–181·5)	15·2% (-35·9 to 99·7%)	0·76
	Afghanistan	54·5 (29·1–96)	152·7% (57 to 371·2%)	176 (99·6–323·9)	-5·8% (-38·6 to 56%)	0·81
	Sudan	48·4 (29·1–71·3)	70·3% (-20 to 292·9%)	135·8 (84·2–196·4)	-1·5% (-48·8 to 87·4%)	0·69
Stroke*	Global	143232·2 (133095·8–153241·8)	32·4% (22 to 42·2%)	1768·1 (1640·6–1889·4)	-35·2% (-40·5 to -30·5%)	0·76
	North Africa and Middle East	7946 (7060·2–8870·8)	43·3% (27·2 to 61·4%)	1826·2 (1635·3–2026·2)	-32% (-39·1 to -23·3%)	1·04

Table S11 Cause- and sex-specific burden, mortality, incidence, and prevalence of neurological conditions in North Africa and Middle East countries

		All Ages		Age-standardised		
		Number (thousand)	Percent change	Rate per 100,000	Percent change	Sex ratio
**Measure**		2019	From 1990 to 2019	2019	From 1990 to 2019	2019
Cause	Location	Mean (95% UI)	Mean (95% UI)	Mean (95% UI)	Mean (95% UI)	F:M
	Algeria	543·9 (451–649·2)	47% (18·7 to 82·1%)	1755·4 (1459·6–2081·9)	-44·6% (-55 to -31·9%)	1·25
	Bahrain	8 (6·7–9·7)	111% (71·2 to 164·3%)	940·9 (796·8–1146·2)	-52·5% (-61·2 to -42·5%)	1·27
	Egypt	1494 (1146·4–1946·5)	-7·4% (-31·5 to 33·5%)	2138 (1636·8–2809·8)	-35·5% (-50 to -17%)	1·14
	Iran	884·8 (812·2–943·7)	48·2% (33·4 to 63·4%)	1262·2 (1153·5–1346·3)	-45·7% (-51 to -38·3%)	0·98
	Iraq	682·9 (548–826·3)	117·8% (72 to 174·5%)	2922·9 (2399–3459·1)	-19·4% (-35·5 to -0·8%)	0·82
	Jordan	86·1 (72·9–100·2)	133·8% (94 to 178·9%)	1448 (1220·4–1679·5)	-50·2% (-58·7 to -41·1%)	1·17
	Kuwait	25·5 (21·8–29·8)	232·8% (186·6 to 287·4%)	938·4 (795–1093·3)	-11·7% (-24 to 3·3%)	0·68
	Lebanon	39·2 (30·9–48·7)	63% (27·7 to 105·4%)	752·9 (593·3–935·9)	-33·7% (-47·6 to -16%)	1·08
	Libya	82·3 (64·2–104·7)	52·5% (15·8 to 109·4%)	1570·5 (1237·3–2001·2)	-21·3% (-37·5 to 1·4%)	1·22
	Morocco	666·3 (534·6–808·8)	72·1% (36·4 to 111·6%)	2257·8 (1838·8–2702·5)	-17·2% (-33·9 to 0·9%)	1·06
	Palestine	44·9 (39·5–50·8)	63·5% (29·3 to 104·3%)	2128·3 (1878·2–2399·7)	-32·5% (-46·4 to -15%)	0·99
	Oman	30·9 (27–36·7)	26·6% (-0·4 to 64·9%)	1884·3 (1667·3–2127·1)	-38·7% (-51·9 to -20·7%)	0·95
	Qatar	9 (7·3–11·1)	256·6% (174·3 to 355·9%)	904·5 (747·6–1130·3)	-44·7% (-57·5 to -29·5%)	1·72
	Saudi Arabia	417·6 (326·2–509·9)	129% (67·6 to 211·5%)	2114·9 (1685·1–2485·5)	-29·1% (-46·4 to -5·6%)	1·07
	Syrian Arab Republic	238·3 (184·9–304·4)	-5·7% (-29·3 to 27·5%)	2018·1 (1589·6–2547·1)	-40·2% (-55·3 to -19·9%)	0·99
	Tunisia	176·9 (135·4–225·5)	83·2% (38·9 to 138·2%)	1477·5 (1138·5–1875)	-24·7% (-42·8 to -2%)	0·92
	Türkiye	993·1 (820·9–1177·5)	75·3% (32·7 to 117%)	1162·6 (965–1380·4)	-23·5% (-42·5 to -5·2%)	0·95
	United Arab Emirates	95·5 (70·9–128·9)	416·5% (265·8 to 618·4%)	1925·6 (1517–2457·9)	-46% (-58·5 to -30·5%)	1·02
	Yemen	388·5 (302·6–500·1)	88·5% (44 to 152·4%)	2765·5 (2161·6–3520·5)	-24·5% (-41 to -1·9%)	1·07
	Afghanistan	507·8 (375·9–659·8)	56·3% (20·5 to 98·4%)	3498·2 (2508·8–4500·4)	-21·8% (-39·4 to -3·6%)	1·42
	Sudan	522·4 (385·9–715·6)	21·5% (-7·2 to 57·4%)	2585·5 (1970·5–3552·9)	-33% (-45·3 to -16·9%)	1·15
Ischaemic stroke	Global	63478·3 (57827·4–68986·5)	56·7% (43·3 to 67·9%)	798·8 (727·5–866·9)	-28·5% (-34·7 to -23·2%)	0·83
	North Africa and Middle East	4751·4 (4227·7–5271·9)	120% (89 to 148·1%)	1183·6 (1060·8–1307)	-8·8% (-19·6 to 2·2%)	1·09
	Algeria	372·7 (302·7–448·8)	126·7% (81·1 to 182·3%)	1275·1 (1052·8–1530·2)	-24·7% (-38·7 to -8·3%)	1·27
	Bahrain	4·2 (3·5–5)	165·2% (120·6 to 220%)	613·6 (521·7–746·7)	-43·9% (-53·5 to -33%)	1·29
	Egypt	863·1 (669–1115·5)	99% (43·1 to 170·4%)	1381·5 (1070·2–1804·1)	13·7% (-15·5 to 43·8%)	1·3
	Iran	668·9 (608–717·6)	68·5% (51·1 to 87·3%)	984·4 (890–1054·7)	-42·8% (-48·2 to -34·9%)	1·03
	Iraq	373·2 (305·8–438·9)	153·9% (101·6 to 214·7%)	1828·9 (1525·3–2123·6)	-7% (-25·1 to 13·7%)	0·89
	Jordan	59·9 (50·6–69·6)	161·4% (114·5 to 207·5%)	1083·5 (911·7–1252·3)	-46·3% (-56·1 to -36·4%)	1·21
	Kuwait	15·2 (13–18)	252·5% (206·4 to 307%)	645·4 (546·5–754·4)	-12% (-24 to 2·4%)	0·74
	Lebanon	31·1 (24·6–38·2)	125·1% (71·9 to 176·4%)	597·8 (473·3–734·3)	-15·1% (-34·7 to 4·6%)	1·08
	Libya	55·4 (43·1–71)	164% (105·4 to 245·1%)	1122·6 (875·9–1435·2)	11·2% (-12 to 42·4%)	1·24
	Morocco	451·7 (364·1–544·5)	167·3% (107·4 to 225·5%)	1603·6 (1305–1921·7)	18·5% (-6·7 to 42·2%)	1·09
	Palestine	31·2 (27·2–35·4)	101·3% (59·7 to 150·7%)	1590·9 (1386·2–1793·7)	-17·8% (-34·6 to 3%)	1·03
	Oman	17·3 (14·8–19·9)	79·5% (38·7 to 135·1%)	1291·3 (1116·3–1468·2)	-18·3% (-35·2 to 6·7%)	0·7
	Qatar	3·8 (3–4·6)	333·4% (238·6 to 427·2%)	577·3 (476·3–725)	-35·5% (-49·9 to -19·3%)	1·79
	Saudi Arabia	207·9 (160·1–249·4)	153·3% (90·1 to 234·4%)	1300·8 (1026·9–1516·1)	-17·4% (-36·3 to 5·8%)	1·09
	Syrian Arab Republic	116·5 (92·6–145·6)	43·2% (7·6 to 90·1%)	1069·9 (858·8–1325·1)	-25·1% (-44·7 to -2·2%)	1·16
	Tunisia	128·1 (98·9–163·2)	178·2% (108·2 to 261·1%)	1090·4 (848·4–1382·7)	4·1% (-22 to 34·2%)	0·94
	Türkiye	551·1 (460–649·2)	133·2% (71·4 to 186·1%)	662·7 (552·9–779·5)	-8% (-32·7 to 14·3%)	1·05
	United Arab Emirates	54·4 (41·4–69·8)	620·9% (416·4 to 889·1%)	1415·2 (1128·2–1749·3)	-33·9% (-48·7 to -15·7%)	1·05
	Yemen	212·2 (168·3–276·2)	232·1% (159·4 to 334·1%)	1716·7 (1370·2–2197·1)	19·8% (-4·2 to 51·1%)	1·1
	Afghanistan	224·6 (170·4–298·1)	135·5% (80·7 to 197·7%)	1865·5 (1381·8–2444·2)	27·3% (-0·6 to 58·5%)	1·37
	Sudan	304·2 (232·8–427)	108·8% (61·1 to 165·4%)	1692·7 (1303·5–2360·6)	8·1% (-12·2 to 33·5%)	1·18
Intracerebral haemorrhage	Global	68572·5 (63272·3–73682)	25·3% (12·2 to 36·3%)	832·8 (769·2–894·7)	-36·7% (-43·3 to -31%)	0·68
	North Africa and Middle East	2702·2 (2343·7–3120·3)	3·4% (-12·3 to 21·4%)	548·4 (479·5–623·1)	-51·7% (-58·2 to -43·8%)	0·93
	Algeria	142·3 (112·7–177·6)	-16·1% (-35·6 to 10·1%)	403·4 (322·4–501·3)	-67·9% (-75·6 to -58·4%)	1·17

Table S11 Cause- and sex-specific burden, mortality, incidence, and prevalence of neurological conditions in North Africa and Middle East countries

		All Ages		Age-standardised		
		Number (thousand)	Percent change	Rate per 100,000	Percent change	Sex ratio
**Measure**		2019	From 1990 to 2019	2019	From 1990 to 2019	2019
Cause	Location	Mean (95% UI)	Mean (95% UI)	Mean (95% UI)	Mean (95% UI)	F:M
	Bahrain	3 (2·4–3·7)	78·6% (36·6 to 133·3%)	264·6 (217·2–326·5)	-64·2% (-72 to -54·2%)	1·28
	Egypt	525·9 (380·6–714·4)	-36·5% (-56·7 to -7·1%)	641·8 (460·5–884·3)	-59·8% (-70·7 to -45·5%)	0·88
	Iran	172·6 (162·4–183·2)	6·8% (-6·5 to 20·4%)	223·3 (209·6–236·3)	-53·6% (-59·9 to -47·4%)	0·79
	Iraq	287·2 (222–364·5)	99·7% (51·7 to 165·7%)	1022 (808·4–1271·1)	-30% (-47 to -9·1%)	0·7
	Jordan	22·9 (19·3–27·1)	83·5% (47·7 to 127·3%)	325·6 (273·5–385·1)	-60% (-67·8 to -50·4%)	1·04
	Kuwait	8·4 (6·9–10)	252·7% (182·6 to 330·1%)	246 (201·3–294)	-4·2% (-23·3 to 18%)	0·49
	Lebanon	6·7 (5–9)	-19% (-40·5 to 9·9%)	128·4 (95·9–170·4)	-64% (-73·7 to -51%)	1·02
	Libya	22·6 (16·9–30·6)	-2·1% (-28·9 to 43%)	376·2 (284·9–504·4)	-51·2% (-63·2 to -34·3%)	1·16
	Morocco	181·5 (138·9–232·6)	3·6% (-24·8 to 39·1%)	554 (425·3–704·4)	-51% (-64·1 to -34·3%)	0·97
	Palestine	12·2 (10·4–14·2)	11·2% (-14·1 to 46·7%)	487·3 (416·5–568·3)	-56·7% (-66·5 to -43%)	0·86
	Oman	10·8 (9·3–13·4)	-1·9% (-25·1 to 32·7%)	504·1 (437·1–621·6)	-59·8% (-69·5 to -46·5%)	1·9
	Qatar	3·5 (2·7–4·6)	205·4% (117·4 to 322·7%)	243·6 (195·1–315·2)	-56·3% (-67·8 to -41·8%)	1·57
	Saudi Arabia	192·7 (149·1–243·1)	113·8% (51 to 215·6%)	764·7 (597–929·3)	-41·3% (-57·6 to -15·5%)	1·00
	Syrian Arab Republic	106·5 (80·7–141·6)	-11·4% (-36·4 to 24·2%)	839 (646·4–1101·7)	-48·5% (-62·7 to -28·8%)	0·83
	Tunisia	40·6 (29·9–53·5)	2·1% (-27·6 to 42·4%)	322·5 (238·9–422·5)	-56·5% (-69·3 to -38·5%)	0·84
	Türkiye	350·8 (284·1–427·5)	37% (0·3 to 74·7%)	399 (323·7–485·6)	-36·9% (-54·1 to -20%)	0·84
	United Arab Emirates	35 (23·8–53·1)	294·6% (149·3 to 494·6%)	449·8 (317·9–664·8)	-64·1% (-75·4 to -49·2%)	0·88
	Yemen	146·7 (108·2–195·4)	25% (-9·2 to 75·9%)	884·3 (663·2–1178·9)	-53·7% (-65·8 to -36·8%)	1·00
	Afghanistan	241·9 (176·7–325)	22·9% (-7·3 to 64·9%)	1397·7 (991–1864·5)	-46% (-59·2 to -29·6%)	1·46
	Sudan	185·7 (126·4–260·2)	-20·6% (-43·3 to 7·9%)	769·9 (528·1–1070·3)	-60·6% (-70 to -48%)	1·1
Subarachnoid haemorrhage	Global	11181·4 (9893·4–12668·9)	-13·8% (-25·7 to 17%)	136·5 (120·8–154·7)	-54·2% (-60·6 to -37·5%)	0·89
	North Africa and Middle East	492·4 (408·3–617·2)	-36% (-50·6 to 6·8%)	94·2 (79–116·8)	-62·8% (-71·6 to -41%)	1·08
	Algeria	28·9 (22·1–38·1)	-19·8% (-42·2 to 28%)	76·8 (58·2–100·2)	-64·6% (-74·6 to -44·4%)	1·21
	Bahrain	0·9 (0·7–1·2)	55·4% (12·2 to 135·2%)	62·6 (48·6–85·6)	-58·1% (-70·1 to -41·3%)	1·09
	Egypt	104·9 (74·6–145)	-70·2% (-82·6 to -30·8%)	114·7 (82·7–158)	-77·4% (-85·6 to -52·9%)	0·87
	Iran	43·2 (38·4–50·6)	12·4% (-15·5 to 70·6%)	54·6 (48·5–62·6)	-55·7% (-68·1 to -31·7%)	1·02
	Iraq	22·6 (16·9–30·6)	-1·1% (-34·8 to 60·2%)	72·1 (54·4–97·2)	-64·1% (-76·6 to -41·4%)	1·09
	Jordan	3·3 (2·7–4·1)	129·6% (69·5 to 228·8%)	38·9 (30·6–47·5)	-49·8% (-63·8 to -27·7%)	1·17
	Kuwait	1·8 (1·5–2·2)	92·1% (52·5 to 179·1%)	47 (38·9–56·6)	-34·9% (-48·1 to -11·3%)	1·06
	Lebanon	1·4 (1–1·9)	-27·4% (-55·8 to 16·4%)	26·7 (18·6–35·8)	-64·3% (-78·7 to -41·8%)	1·39
	Libya	4·4 (3·2–6·3)	-56% (-73 to -4·1%)	71·7 (51·6–101·4)	-66·7% (-77·7 to -42·9%)	1·23
	Morocco	33·2 (23–48·6)	-23% (-47·9 to 27·2%)	100·3 (70·3–143·9)	-58·6% (-72·4 to -30·7%)	1·22
	Palestine	1·5 (1·3–1·8)	49·3% (10·4 to 117·4%)	50·1 (42·3–59)	-46% (-61·6 to -22·2%)	1·32
	Oman	2·7 (2–4·3)	-25·7% (-54·9 to 55·5%)	88·9 (59·7–133·6)	-62·4% (-76·8 to -29·6%)	1·32
	Qatar	1·7 (1·2–2·2)	240% (109·5 to 413·8%)	83·6 (62·6–109·6)	-54·2% (-72·3 to -26·8%)	1·7
	Saudi Arabia	17 (12·3–22·5)	66·7% (-0·4 to 170·7%)	49·5 (36·1–64·1)	-52·5% (-71·9 to -22·6%)	1·57
	Syrian Arab Republic	15·3 (11·7–19·1)	-70·1% (-80 to -49·5%)	109·2 (84·6–135·2)	-65·7% (-76 to -47·7%)	0·88
	Tunisia	8·2 (5·8–11·2)	-23·9% (-51·2 to 27·2%)	64·5 (45·6–88·2)	-62·6% (-76·7 to -38·2%)	1·09
	Türkiye	91·2 (72–114·2)	22·8% (-22 to 111·1%)	100·9 (80–125·8)	-39·9% (-61·8 to 5·4%)	0·87
	United Arab Emirates	6·1 (3·4–9·8)	193·2% (77·1 to 409·8%)	60·6 (33·3–95·3)	-64·4% (-76·8 to -43·5%)	1·42
	Yemen	29·6 (15·7–48·4)	18·9% (-19·5 to 104·4%)	164·5 (87·2–275·6)	-48·9% (-63·9 to -20%)	1·25
	Afghanistan	41·4 (13·9–70·9)	26% (-12·4 to 98·7%)	235 (76·3–400·7)	-43·8% (-59·4 to -14·3%)	1·61
	Sudan	32·6 (18·5–55·8)	-35·4% (-56·2 to 35·5%)	122·9 (71·2–208·7)	-63·7% (-75 to -32·7%)	1·16
Neurological disorders†	Global	97724·4 (55942·8–159416·8)	69·9% (58·7 to 90%)	1253·6 (719·7–2039·8)	-0·8% (-5·1 to 3·1%)	1·22
	North Africa and Middle East	7156 (3769·7–12184·4)	91·1% (72 to 109·7%)	1382·1 (776·6–2273·8)	-3·9% (-10·3 to 3·5%)	1·25
	Algeria	494·8 (254·6–842·9)	82·8% (47·3 to 123·8%)	1365·1 (738·5–2292)	-8·4% (-21·4 to 5%)	1·29
	Bahrain	16·1 (7·7–28·6)	199·3% (134·7 to 269·8%)	1387·6 (768·6–2266·4)	-8% (-23·9 to 8·1%)	1·26

Table S11 Cause- and sex-specific burden, mortality, incidence, and prevalence of neurological conditions in North Africa and Middle East countries

		All Ages		Age-standardised		
		Number (thousand)	Percent change	Rate per 100,000	Percent change	Sex ratio
**Measure**		2019	From 1990 to 2019	2019	From 1990 to 2019	2019
Cause	Location	Mean (95% UI)	Mean (95% UI)	Mean (95% UI)	Mean (95% UI)	F:M
	Egypt	1050·5 (470·2–1877·2)	92% (55·8 to 126·9%)	1336·2 (709·8–2284·8)	0·9% (-13·5 to 15·6%)	1·3
	Iran	1103·5 (584·2–1853)	80·4% (60·7 to 111·9%)	1394·2 (785·8–2300·9)	-4·1% (-10·3 to 4·7%)	1·25
	Iraq	437·8 (204·7–786·4)	153·1% (104·7 to 201·9%)	1331·8 (715·6–2250·9)	-1·7% (-14 to 12·3%)	1·23
	Jordan	118·1 (54·4–213·6)	243·5% (184·7 to 317·9%)	1291·8 (687·7–2194·4)	-4·7% (-17·1 to 8·6%)	1·28
	Kuwait	49·3 (22·5–88·6)	181·9% (131·6 to 238·7%)	1294·9 (693·8–2173·2)	-4·3% (-17·6 to 9·3%)	1·26
	Lebanon	69·8 (37–117·5)	93·9% (63 to 142·7%)	1349·3 (721·7–2279·8)	-3·4% (-16·8 to 12·3%)	1·26
	Libya	84·6 (43·8–145·1)	95% (63·4 to 133·8%)	1378·2 (759·2–2303·8)	-3% (-15·6 to 11·8%)	1·24
	Morocco	435·8 (225·1–747·8)	71·5% (46 to 110·2%)	1355·8 (744·6–2284·9)	1·9% (-12·1 to 17%)	1·24
	Palestine	50·3 (24·7–87·5)	142·1% (92·8 to 184·1%)	1376 (773·8–2255·8)	-5·1% (-17·7 to 8·1%)	1·2
	Oman	43·2 (18·5–79·8)	167·7% (114·1 to 223·1%)	1340·6 (758·2–2231·2)	1% (-12·7 to 19·5%)	1·22
	Qatar	26·8 (11·4–50·2)	546·8% (399·9 to 684·3%)	1344·2 (754·1–2196)	-2·2% (-17·2 to 16·1%)	1·34
	Saudi Arabia	410·5 (195·2–722·1)	160·3% (106·3 to 223·5%)	1428·4 (794·9–2355·6)	0·1% (-16·7 to 20·3%)	1·18
	Syrian Arab Republic	165·3 (80·7–293·6)	43·5% (20·1 to 79·2%)	1327·1 (692·4–2305·8)	1·4% (-11·9 to 16·8%)	1·29
	Tunisia	154·4 (80·2–263·2)	74·3% (47·6 to 117·8%)	1332·6 (699·8–2234·9)	-2·1% (-15·5 to 11·4%)	1·26
	Türkiye	1232 (709·1–2000·5)	49·8% (23 to 84·9%)	1477·4 (862·5–2368·4)	-8·7% (-22·9 to 6·2%)	1·25
	United Arab Emirates	104·9 (51·5–182·4)	439·5% (320·2 to 578·7%)	1432 (843·2–2254·4)	-6·4% (-24·3 to 12%)	1·14
	Yemen	298·4 (137·8–541·5)	137·1% (81·9 to 190·8%)	1324·5 (723·4–2244·2)	-2·7% (-15·4 to 12·6%)	1·24
	Afghanistan	382·7 (190·2–668·3)	154·8% (86·6 to 226·8%)	1503 (853·8–2492·1)	-10·1% (-23·8 to 4·4%)	1·25
	Sudan	420·3 (204·7–745·9)	84·3% (37·5 to 130·9%)	1353·8 (761·5–2268·2)	-7·1% (-20·6 to 6·3%)	1·2
Alzheimer's disease and other dementias	Global	25277 (11204·5–54558·2)	161·6% (149·7 to 175·7%)	338·6 (151–731·3)	3·7% (-0·3 to 8·1%)	1·19
	North Africa and Middle East	1208·1 (532–2672·8)	177·3% (161·1 to 215·8%)	387 (172–848·5)	-1·1% (-6·6 to 12·1%)	1·12
	Algeria	95·6 (41·6–217·3)	228·9% (176·4 to 291·6%)	401·3 (176·1–912·8)	-5% (-17·6 to 8·7%)	1·12
	Bahrain	1·7 (0·7–3·9)	398·1% (321·9 to 486·1%)	403·1 (176·2–888·6)	-3·8% (-15 to 9·1%)	1·13
	Egypt	135·6 (59·5–305·5)	112·3% (83 to 155·6%)	362·4 (160·1–793·2)	0·2% (-12·4 to 18·8%)	1·14
	Iran	228·1 (100·8–497·8)	314·3% (277 to 401·2%)	386 (170·3–844)	0% (-5·4 to 21·3%)	1·11
	Iraq	57·5 (25·2–128·2)	157·1% (125 to 195·1%)	382 (168·2–826·8)	1·9% (-9·7 to 16·3%)	1·08
	Jordan	14·8 (6·6–32·9)	385·3% (325·6 to 465·5%)	369·7 (165·2–822·8)	-4·5% (-15·9 to 11·3%)	1·12
	Kuwait	6·9 (3·1–14·7)	387·2% (341·5 to 440%)	387·3 (174–837·8)	-2·6% (-11·2 to 6·9%)	1·1
	Lebanon	19·7 (8·6–43·4)	208·8% (166·4 to 308·3%)	395·6 (172·4–866·4)	-2·3% (-15 to 29·1%)	1·16
	Libya	16·6 (7·1–37·2)	161·3% (122·6 to 212·3%)	414·3 (176·1–925·9)	-4·2% (-18·1 to 14·3%)	1·11
	Morocco	86·2 (37·1–195·5)	147% (117·2 to 177·1%)	385·8 (166–876·4)	0·3% (-10·6 to 11%)	1·04
	Palestine	5·8 (2·5–13·5)	117·2% (87 to 155·6%)	388 (170·4–895·9)	-4·4% (-17 to 12%)	1·05
	Oman	3·3 (1·4–7·8)	126·2% (93·7 to 183·8%)	422 (179·8–967·9)	-3·3% (-16·4 to 26·2%)	1·13
	Qatar	1·2 (0·5–2·9)	600% (460·9 to 778·2%)	407·2 (171·7–948·7)	3·4% (-11·6 to 19·9%)	1·13
	Saudi Arabia	32·5 (13·9–73)	122·7% (86·3 to 186·1%)	389·7 (168·5–863·1)	-2% (-15·8 to 26·4%)	1·1
	Syrian Arab Republic	30·7 (13·2–70·3)	112% (74·2 to 170·1%)	391·1 (167–907·7)	2·9% (-14 to 29·8%)	1·14
	Tunisia	42 (18·3–95)	212·4% (160 to 293·7%)	400·1 (174·9–889·4)	-2% (-17 to 21·4%)	1·1
	Türkiye	313·7 (142·3–713·2)	182·1% (142·9 to 226·4%)	395·4 (179·6–892·7)	-2% (-15·1 to 12·9%)	1·17
	United Arab Emirates	4·1 (1·6–10)	607·7% (462·1 to 810·2%)	366·1 (157·8–831)	-5·4% (-17·1 to 14·2%)	1·08
	Yemen	31·8 (13·7–72)	202% (163·7 to 252·4%)	383·7 (166–893·1)	-0·4% (-11·5 to 14·1%)	1·07
	Afghanistan	30·9 (12·7–73·8)	52·6% (31·4 to 71·9%)	432·7 (179–1002)	-3·7% (-15·3 to 6·8%)	1·05
	Sudan	48·1 (21·1–107·8)	107·9% (88·1 to 135·5%)	365·5 (160·3–840·7)	-3·9% (-12·2 to 7%)	1·08
Parkinson's disease	Global	6292·6 (5769·2–6827·2)	128·9% (113·3 to 142·5%)	80 (73·3–86·6)	2·4% (-4·4 to 8·2%)	0·56
	North Africa and Middle East	300·7 (266·3–365·4)	163·8% (134·8 to 202·1%)	84·4 (74·7–103·2)	0·9% (-10·2 to 15·7%)	0·68
	Algeria	21·7 (17·6–26·3)	161·4% (106 to 229·3%)	78·9 (64·1–95·1)	-18·1% (-35·7 to 3·6%)	0·83
	Bahrain	0·5 (0·3–0·6)	299·2% (213·3 to 401·9%)	88·5 (64·1–106·6)	-13·5% (-32·1 to 7·5%)	0·69
	Egypt	48·3 (37·8–67·9)	135·2% (88·1 to 190·9%)	98·6 (77·1–142·4)	11·7% (-9·4 to 36·5%)	1·05

Table S11 Cause- and sex-specific burden, mortality, incidence, and prevalence of neurological conditions in North Africa and Middle East countries

		**All Ages**		**Age-standardised**		
		Number (thousand)	Percent change	Rate per 100,000	Percent change	Sex ratio
**Measure**		2019	From 1990 to 2019	2019	From 1990 to 2019	2019
Cause	Location	Mean (95% UI)	Mean (95% UI)	Mean (95% UI)	Mean (95% UI)	F:M
	Iran	48·8 (42·4–53·9)	253·3% (207·9 to 313·5%)	76·8 (66·9–84·9)	3·1% (-12·9 to 20·5%)	0·68
	Iraq	15 (12·2–20·3)	209·1% (147·3 to 290·7%)	85·6 (71–115·2)	17·5% (-5·7 to 47·1%)	0·43
	Jordan	3·5 (3–4·1)	335·8% (254·4 to 432·2%)	74·2 (63–86·7)	-14% (-29·8 to 5%)	0·69
	Kuwait	1·1 (0·9–1·3)	221% (180·8 to 271·8%)	56·1 (47·2–66)	-26·8% (-36·5 to -14·7%)	0·48
	Lebanon	3·7 (3–4·8)	142·1% (82·7 to 213·3%)	69·5 (57·3–91·6)	-14·2% (-35·2 to 11·2%)	0·58
	Libya	3·7 (2·8–4·7)	206·8% (134·1 to 306·4%)	84·8 (65·3–108·4)	10·9% (-15·2 to 45·8%)	0·69
	Morocco	25·3 (20·6–29·6)	221·2% (160·6 to 300·2%)	96·6 (79·3–111·9)	39·6% (14·4 to 73·3%)	0·59
	Palestine	1·6 (1·1–1·9)	127·5% (75·6 to 210·6%)	93·1 (64·9–106·8)	-4·9% (-26·4 to 29·1%)	0·56
	Oman	1·1 (0·8–1·3)	168·6% (99·7 to 253·4%)	125·3 (79–144·5)	30·3% (-2·2 to 70·4%)	0·52
	Qatar	0·5 (0·4–0·6)	517% (361·6 to 711·4%)	148·6 (96–194·6)	14·8% (-11·6 to 49·8%)	1·03
	Saudi Arabia	11·6 (9·4–13·5)	145·8% (88·1 to 247·9%)	111 (83·8–129·8)	1·6% (-19·9 to 43·5%)	0·4
	Syrian Arab Republic	7·8 (5·5–9·8)	157·5% (96·9 to 243·4%)	83·9 (58·4–104·9)	13·7% (-12·2 to 49·7%)	0·67
	Tunisia	8·3 (6·4–10·7)	205·8% (138·1 to 290·2%)	72·7 (56·2–93·7)	11·1% (-13·8 to 41%)	0·58
	Türkiye	62·2 (48·7–105·2)	147·6% (97·6 to 201·9%)	76·1 (59·4–129·7)	-8·2% (-25·9 to 11·9%)	0·67
	United Arab Emirates	2·9 (2·1–3·9)	716·5% (504·6 to 1058·9%)	130·5 (87–167·2)	-10·6% (-29·6 to 21·4%)	0·61
	Yemen	8·2 (6·4–10·7)	216·7% (147·7 to 319·7%)	76·8 (60·8–98·7)	15·5% (-7·8 to 49·7%)	0·66
	Afghanistan	11·6 (8·8–14·9)	52·5% (18·5 to 92·8%)	113·8 (88·7–143·3)	-6% (-25·7 to 16·3%)	0·74
	Sudan	13·2 (10·4–16·8)	81·9% (38·4 to 143·1%)	85·5 (67·8–107·5)	-4·3% (-26·5 to 26·1%)	0·58
Idiopathic epilepsy	Global	13077·6 (9986·7–16734·1)	15·9% (0 to 42·1%)	170·6 (130·4–218·3)	-16·5% (-27·4 to 1·7%)	0·82
	North Africa and Middle East	955·3 (682·8–1293·2)	13·8% (-13·8 to 56%)	158·3 (112·6–213·2)	-26·2% (-43·6 to -1·1%)	0·87
	Algeria	60·5 (29·8–101·8)	-8·6% (-58·6 to 82·6%)	146·2 (70·8–246·9)	-38% (-72·5 to 25·8%)	0·96
	Bahrain	2·3 (1·1–4·1)	83·1% (-17·9 to 303·1%)	186·8 (86·8–326·3)	-31·7% (-70 to 51·3%)	0·94
	Egypt	111·7 (44·2–202·5)	34·2% (-51·1 to 227·5%)	110·6 (43·3–200)	-19·5% (-70·4 to 99·5%)	0·74
	Iran	113·3 (78·4–155·6)	-22·8% (-43·4 to 6·4%)	140·2 (96·1–192·5)	-34·8% (-51·4 to -13%)	0·84
	Iraq	56·3 (24·8–98·7)	59·3% (-31·4 to 252·4%)	131 (57·8–229·5)	-29·4% (-69·3 to 55·2%)	0·93
	Jordan	14·1 (6·2–26·7)	131·8% (-9·3 to 452%)	119·4 (51·7–224·9)	-22·1% (-69·1 to 83·1%)	0·89
	Kuwait	5·5 (2·4–10·5)	74·2% (-22·8 to 296·6%)	138·9 (58·5–268·6)	-22·2% (-66·5 to 81·8%)	0·9
	Lebanon	6·8 (3·2–12·3)	11·9% (-52 to 153·2%)	132·9 (62·6–243·4)	-25·6% (-67·3 to 68·6%)	0·84
	Libya	9·3 (5·2–14·9)	1·9% (-46·8 to 108·6%)	141 (77·2–226·1)	-29% (-63·5 to 47·3%)	0·86
	Morocco	47·9 (17·3–89·9)	17·6% (-58·2 to 176·2%)	135·2 (48·7–253·5)	-11·7% (-68·2 to 108·6%)	1·07
	Palestine	8·2 (4·5–13·3)	69·8% (-11·5 to 223·1%)	163·6 (91·1–262·6)	-24·2% (-60·6 to 43·1%)	0·85
	Oman	4·5 (1·6–9)	91·1% (-38·5 to 468·1%)	107·8 (37·2–214·7)	-9·8% (-70·4 to 160·3%)	0·87
	Qatar	3·1 (1·2–6·1)	326·2% (61·4 to 1037%)	126·5 (47·7–250·5)	-28·2% (-73·2 to 94·6%)	1·1
	Saudi Arabia	81·4 (40·2–138)	128·5% (4·1 to 416·5%)	226·7 (107·8–394·2)	-0·3% (-56·7 to 123·2%)	1·06
	Syrian Arab Republic	15·9 (6·8–28·7)	-10·5% (-65·7 to 124·2%)	109·2 (47–198·2)	-17·2% (-67·5 to 102·6%)	0·89
	Tunisia	13·3 (6·6–23·5)	-11·9% (-58·9 to 78·9%)	119·5 (58·8–210·4)	-26·5% (-66·1 to 50·4%)	0·89
	Türkiye	163 (83·7–270·9)	-19·8% (-60·9 to 58·6%)	213·7 (113·7–354·5)	-30·4% (-65·1 to 38·4%)	0·78
	United Arab Emirates	18·9 (9–30·7)	253·5% (60·3 to 637%)	216·3 (98·8–364·2)	-28·2% (-69·6 to 62·2%)	0·79
	Yemen	49·5 (26·5–81·7)	41·5% (-27·2 to 217·2%)	147·6 (78·5–243·3)	-27·4% (-62·7 to 53%)	0·89
	Afghanistan	94 (50·2–142·6)	88% (10·5 to 308·6%)	224·7 (120·4–348·1)	-38·8% (-64·2 to 28·3%)	1·13
	Sudan	74·7 (43·3–119·5)	5·7% (-46·7 to 158·2%)	168·7 (97·5–271)	-37·1% (-67·9 to 39·6%)	0·82
Multiple sclerosis	Global	1159·8 (1001·2–1381·9)	59·7% (46·6 to 72·7%)	14 (12–16·6)	-13·2% (-20·9 to -6·3%)	1·57
	North Africa and Middle East	115·9 (93·1–144·8)	146% (98·9 to 205·5%)	19·9 (16·1–24·7)	6·2% (-11·6 to 30·5%)	1·58
	Algeria	8·5 (5·7–11·2)	178·8% (106·5 to 257·5%)	20·1 (13·6–26·4)	12·2% (-14·2 to 41·1%)	1·73
	Bahrain	0·2 (0·2–0·3)	446% (337·3 to 558%)	12·5 (9·1–16·2)	12·3% (-7·6 to 32·6%)	1·8
	Egypt	14·3 (8·6–35·9)	95·5% (-16·2 to 400·6%)	15·3 (9·6–32·4)	6·4% (-44·2 to 137·3%)	1·55
	Iran	26·9 (21·8–34·9)	143·4% (103·1 to 191·7%)	29·2 (23·7–37·4)	0·5% (-18·6 to 20·3%)	1·44

Table S11 Cause- and sex-specific burden, mortality, incidence, and prevalence of neurological conditions in North Africa and Middle East countries

		All Ages		Age-standardised		
		Number (thousand)	Percent change	Rate per 100,000	Percent change	Sex ratio
**Measure**		2019	From 1990 to 2019	2019	From 1990 to 2019	2019
Cause	Location	Mean (95% UI)	Mean (95% UI)	Mean (95% UI)	Mean (95% UI)	F:M
	Iraq	4·9 (3·6–6·5)	254% (186·9 to 340·3%)	14·3 (10·6–18·6)	12·4% (-8·4 to 39·2%)	1·57
	Jordan	1·9 (1·4–2·4)	325% (236·9 to 413%)	19 (13·6–23·9)	-6·9% (-24·9 to 11·7%)	1·86
	Kuwait	0·9 (0·6–1·2)	414·3% (336·1 to 505·8%)	17·3 (12·8–22·6)	38·2% (21·1 to 58·7%)	1·5
	Lebanon	1·3 (0·9–1·6)	146·5% (97·3 to 209·4%)	23·5 (16·4–30·3)	20·9% (-2·5 to 51·3%)	1·58
	Libya	1·6 (1·1–2·2)	265% (176·8 to 418·4%)	21·8 (14·9–30·2)	34·2% (2·3 to 87·9%)	1·5
	Morocco	7·4 (5·2–10)	148·6% (94·9 to 227·4%)	20·2 (14·1–27)	25·1% (-1·6 to 63·3%)	1·69
	Palestine	0·7 (0·6–0·9)	239·9% (151·8 to 357·1%)	19·5 (15·3–24·3)	8·5% (-18·1 to 44·4%)	1·32
	Oman	0·7 (0·5–1)	301·1% (176·6 to 453·3%)	17·1 (11·6–23·2)	20·3% (-15·8 to 68·5%)	1·47
	Qatar	0·5 (0·4–0·7)	1040·4% (822·7 to 1331·2%)	16·8 (12·3–21·8)	33·7% (10·4 to 61·8%)	2·31
	Saudi Arabia	4·6 (3·3–6)	357·9% (248·8 to 567·7%)	11·7 (8·7–15)	23% (-5 to 77%)	1·63
	Syrian Arab Republic	2·2 (1·6–2·8)	118·3% (77·3 to 168·1%)	15·2 (11·3–19·6)	15·4% (-5·9 to 40·2%)	1·75
	Tunisia	2·9 (2·1–3·9)	158·7% (103·5 to 228·3%)	22·2 (15·5–29·2)	25·6% (1 to 58·9%)	1·65
	Türkiye	19·4 (15·3–24·6)	68·9% (7 to 127·5%)	21 (16·3–27·6)	-11·8% (-37·6 to 20%)	1·66
	United Arab Emirates	2·8 (1·7–4·4)	935·7% (601·3 to 1345·8%)	20·6 (12·3–32·5)	19% (-16·5 to 59·7%)	1·22
	Yemen	3·1 (2–4·5)	266·8% (156·3 to 435·7%)	14·1 (9·2–19·6)	26·4% (-9·6 to 72·1%)	1·35
	Afghanistan	6·6 (3·9–12·4)	237·7% (138 to 395·7%)	27·1 (16·8–48·7)	10·9% (-21·5 to 51·8%)	1·86
	Sudan	4·2 (2·6–6)	175·8% (93·2 to 310·7%)	14 (8·9–19·2)	20·2% (-13·9 to 70·1%)	1·35
Migraine	Global	42077·7 (6418·4–95645·2)	56·6% (52·6 to 62·1%)	525·5 (78·8–1194)	1·5% (-4·4 to 3·3%)	1·70
	North Africa and Middle East	3793·2 (645·3–8665·8)	102·1% (93·2 to 125·1%)	601·4 (107–1371·8)	0% (-1·6 to 1·6%)	1·70
	Algeria	260 (44·3–590)	89% (76·7 to 120·2%)	602·5 (103·9–1372·1)	-0·2% (-3·2 to 3·1%)	1·67
	Bahrain	9·5 (1·8–21·2)	219·3% (197 to 279·3%)	564·5 (100·5–1278·1)	-0·7% (-3·9 to 2·8%)	1·66
	Egypt	619·6 (96·7–1426·4)	98·2% (84·3 to 113·8%)	621·1 (104–1420·2)	1·9% (-5·1 to 9·1%)	1·74
	Iran	571·7 (107–1282·2)	77·6% (63·7 to 117·4%)	628·3 (115·2–1411·2)	-0·3% (-3·6 to 4·1%)	1·66
	Iraq	253·2 (40·1–580·5)	181·6% (170·8 to 206·2%)	595·4 (102·8–1355·6)	0·3% (-2·8 to 3·2%)	1·67
	Jordan	70·1 (11·9–160·3)	250·2% (230·2 to 298·6%)	590·3 (104–1339·1)	-0·8% (-4 to 2·5%)	1·66
	Kuwait	29·8 (5·2–67·4)	190·2% (171·1 to 225·4%)	578·3 (99·7–1315·1)	3·2% (-2·3 to 8·6%)	1·68
	Lebanon	32 (5·6–73·1)	76·4% (68·2 to 92·7%)	605·8 (104–1385·5)	0·5% (-2·7 to 3·5%)	1·67
	Libya	44·9 (7·7–101·7)	103·6% (88·6 to 141·3%)	594·7 (104–1347)	1·3% (-1·9 to 4·4%)	1·67
	Morocco	226·8 (38·7–515·5)	58·5% (50·7 to 80·5%)	601·7 (104·2–1370·5)	-0·3% (-3·3 to 3%)	1·66
	Palestine	28·5 (4·4–66·3)	177·7% (166·5 to 199·2%)	596·1 (102·5–1357·3)	-1·1% (-4·1 to 1·9%)	1·68
	Oman	28·4 (4·9–64·4)	185% (166·5 to 226·5%)	554·2 (98·7–1256·8)	-1·4% (-4·5 to 1·9%)	1·67
	Qatar	18·1 (3·2–40·5)	601·2% (567·3 to 669·2%)	523 (94·3–1174·9)	-2·7% (-6·2 to 0·8%)	1·66
	Saudi Arabia	234·2 (41·4–528·2)	176·4% (152·8 to 229·1%)	562 (99·4–1276·9)	-1·2% (-5·9 to 3·8%)	1·55
	Syrian Arab Republic	91·6 (15·5–212·2)	39·1% (31·2 to 62·3%)	609·9 (105·1–1394·5)	1·7% (-1·9 to 5·2%)	1·68
	Tunisia	74·3 (13·2–168·6)	54·9% (45·1 to 82·1%)	606·3 (104·4–1382·7)	0·3% (-2·6 to 3·4%)	1·67
	Türkiye	528·1 (101·8–1197·4)	56·6% (45·4 to 81·9%)	589·6 (110·2–1340·7)	0·2% (-4·5 to 5·2%)	1·88
	United Arab Emirates	60·2 (12–134)	473% (424 to 588·1%)	535·6 (97·8–1203·7)	-0·7% (-4·3 to 3·1%)	1·66
	Yemen	175·4 (26·6–404·4)	169% (158·9 to 186·6%)	596·7 (104·2–1356·8)	0·1% (-2·9 to 3·2%)	1·65
	Afghanistan	198·8 (30·2–469·5)	244·1% (220·3 to 261·8%)	587·4 (103–1342·7)	-1·3% (-4·9 to 3·2%)	1·67
	Sudan	234·3 (36·7–542)	122·2% (114·9 to 132·9%)	601·9 (104·6–1369·5)	-0·1% (-3·2 to 3·2%)	1·67
Tension-type headache	Global	4541·7 (1395·5–14981·3)	57·8% (45·1 to 65·9%)	56·2 (17–188·5)	-2·5% (-5·4 to 1·1%)	1·25
	North Africa and Middle East	416·6 (138·3–1196·8)	115·5% (84·9 to 132·3%)	68·1 (22·8–195·5)	1% (-9·5 to 8·7%)	1·28
	Algeria	28·5 (9·6–79·5)	106·7% (73·1 to 140·3%)	66·7 (22·6–189)	0·3% (-10·2 to 7·9%)	1·29
	Bahrain	1·1 (0·4–3·1)	254·2% (197·7 to 330·7%)	64·6 (21·3–189·3)	-0·3% (-10·7 to 7·7%)	1·29
	Egypt	65·2 (21–196·7)	100·8% (75 to 120·6%)	69·1 (22·6–207·8)	1·4% (-10·1 to 10·8%)	1·29
	Iran	70·2 (23–207·1)	103·8% (70·9 to 131·5%)	77·6 (24·9–236·4)	5·2% (-5·7 to 15·9%)	1·14
	Iraq	26·3 (8·7–77)	193·4% (151·1 to 230·5%)	66·2 (22·1–190·3)	0·7% (-11·1 to 9·1%)	1·29

Table S11 Cause- and sex-specific burden, mortality, incidence, and prevalence of neurological conditions in North Africa and Middle East countries

		All Ages		Age-standardised		
		Number (thousand)	Percent change	Rate per 100,000	Percent change	Sex ratio
**Measure**		2019	From 1990 to 2019	2019	From 1990 to 2019	2019
Cause	Location	Mean (95% UI)	Mean (95% UI)	Mean (95% UI)	Mean (95% UI)	F:M
	Jordan	7·4 (2·5–21·8)	276·6% (221·8 to 332·8%)	66·2 (22·7–191·4)	0·1% (-10·9 to 8·3%)	1·29
	Kuwait	3·2 (1·1–9)	204·5% (159·1 to 260·4%)	62·8 (20·8–186·4)	1·5% (-10·4 to 11·9%)	1·4
	Lebanon	3·6 (1·2–10)	86·4% (59 to 108·4%)	67 (22·7–191·7)	0·8% (-10·8 to 9·4%)	1·29
	Libya	4·9 (1·7–13·9)	122·3% (84·9 to 164·2%)	66·4 (22·8–191)	1·1% (-11·2 to 9·5%)	1·29
	Morocco	24·8 (8·5–69·4)	70·5% (47·5 to 93·4%)	66·5 (22·7–187·3)	0·2% (-9·8 to 8·1%)	1·29
	Palestine	2·9 (1–8·6)	187·4% (144·1 to 219·3%)	66·2 (22·1–187·5)	-0·3% (-13 to 9·3%)	1·3
	Oman	3·2 (1–9)	208·1% (154·1 to 256·4%)	64·3 (21·6–190·1)	0% (-15·4 to 10·8%)	1·29
	Qatar	2·1 (0·7–6)	643·8% (552 to 739·5%)	62 (20·7–184·7)	-1·4% (-11·6 to 7·2%)	1·29
	Saudi Arabia	25·6 (9·1–70·1)	202% (136·9 to 255%)	63 (22–180·3)	-0·3% (-18·6 to 10·8%)	1·3
	Syrian Arab Republic	9·8 (3·4–28·4)	51·2% (24·9 to 78·8%)	67 (22·9–189·7)	1·1% (-10·5 to 9·5%)	1·29
	Tunisia	8·4 (2·9–23·5)	70·6% (42·7 to 97·2%)	67 (22·6–192·5)	0·7% (-10·7 to 8·5%)	1·3
	Türkiye	60·7 (21·7–160·1)	71·5% (44·9 to 93·8%)	66·6 (23·6–180)	0·8% (-10 to 10·1%)	1·36
	United Arab Emirates	7·4 (2·4–20·5)	542·2% (427·1 to 676·1%)	63·2 (20·7–187·8)	0% (-12·6 to 8·9%)	1·29
	Yemen	17·6 (5·8–51·7)	176·7% (140 to 209·4%)	66·1 (22·8–185·6)	0·6% (-11·1 to 9·8%)	1·28
	Afghanistan	19·6 (6·3–57·7)	231·4% (191·2 to 263·2%)	65·2 (22·1–183·3)	-0·1% (-9·6 to 7·9%)	1·29
	Sudan	23·6 (7·8–69·1)	125% (95·3 to 148·4%)	66·4 (23·2–189)	0·5% (-11·8 to 9·8%)	1·29
Motor neuron disease	Global	1034·6 (979·9–1085·4)	65·7% (55·5 to 77·7%)	12·7 (12–13·3)	-4·5% (-10·1 to 1·9%)	0·71
	North Africa and Middle East	41·6 (33·8–50·6)	22·5% (-28·9 to 88·7%)	7·8 (6·4–9·5)	-16·6% (-44·8 to 17·9%)	0·73
	Algeria	1 (0·8–1·3)	115·9% (60·6 to 191·1%)	2·5 (2–3·2)	-2% (-28·3 to 36·3%)	1·15
	Bahrain	<0·1 (<0·1–<0·1)	114·5% (70·4 to 169·7%)	2·1 (1·7–2·5)	-54·1% (-64·1 to -42·6%)	1·12
	Egypt	6·5 (3·8–10·1)	221·4% (80·1 to 420·3%)	7·9 (4·6–12·4)	69·4% (-4·4 to 176·8%)	0·68
	Iran	3·8 (3·4–4·4)	170·1% (88·7 to 257·6%)	4·7 (4·1–5·4)	43·7% (1 to 90·3%)	0·9
	Iraq	0·6 (0·5–0·8)	188% (107·4 to 289·5%)	1·8 (1·4–2·3)	10·6% (-27 to 58·4%)	1·1
	Jordan	0·3 (0·3–0·4)	424·1% (275·5 to 637·8%)	3·2 (2·5–4·2)	35·8% (-4·5 to 97%)	0·53
	Kuwait	0·2 (0·2–0·3)	-14% (-39·2 to 17·5%)	5·9 (4·3–7·5)	-61·7% (-72·5 to -48·2%)	0·37
	Lebanon	0·2 (0·1–0·3)	92·6% (25·8 to 185·4%)	3·3 (2·2–5·1)	4·5% (-32·1 to 55·6%)	0·86
	Libya	0·2 (0·1–0·4)	215·6% (71·3 to 427·3%)	3·7 (2·3–5·4)	39·5% (-25·6 to 142%)	1·02
	Morocco	0·9 (0·7–1·2)	130·5% (67·1 to 211·2%)	2·5 (1·9–3·2)	23·8% (-11·8 to 68%)	1·07
	Palestine	0·1 (0·1–0·1)	216·8% (132·7 to 328·8%)	2·1 (1·7–2·5)	31·7% (-8·3 to 85·9%)	1·44
	Oman	0·1 (0·1–0·1)	208·9% (89·5 to 344·2%)	3·2 (1·9–4·4)	17·5% (-32·8 to 84·6%)	1·3
	Qatar	<0·1 (<0·1–0·1)	619·8% (433 to 857·2%)	2·1 (1·6–2·7)	-4·8% (-34·3 to 34·6%)	1·26
	Saudi Arabia	0·9 (0·7–1·3)	226·6% (85 to 448%)	3·1 (2·2–4·2)	18·7% (-35 to 116%)	0·88
	Syrian Arab Republic	0·4 (0·3–0·7)	106·2% (31 to 220·1%)	2·9 (1·9–4·4)	23·9% (-21·2 to 93·2%)	0·87
	Tunisia	0·3 (0·2–0·5)	115·1% (41·1 to 224·9%)	2·7 (2–3·6)	15·9% (-24 to 76·4%)	0·88
	Türkiye	23·6 (18·6–29·4)	-14·4% (-54·1 to 51·4%)	31·7 (24·6–40·4)	-25% (-58 to 20·9%)	0·63
	United Arab Emirates	0·6 (0·3–1·1)	961·7% (388 to 1953·3%)	5·9 (2·6–11·2)	21·9% (-44·4 to 124·6%)	0·98
	Yemen	0·4 (0·3–0·5)	196·5% (107·9 to 322·1%)	1·8 (1·3–2·5)	19·9% (-23·6 to 83·1%)	1·11
	Afghanistan	0·6 (0·5–0·8)	144·4% (73·9 to 237·9%)	2·6 (1·8–3·6)	-6·9% (-34·5 to 34·1%)	1·24
	Sudan	0·6 (0·4–0·8)	138·9% (63 to 241·8%)	2·1 (1·5–2·8)	17·3% (-23·4 to 74·9%)	1·08
Other neurological disorders	Global	4263·4 (3458·9–5174·1)	56·5% (41·8 to 74·3%)	55·9 (45·2–68·3)	10·8% (0·4 to 22·9%)	0·83
	North Africa and Middle East	324·7 (250·4–417·3)	59·4% (26·5 to 98·9%)	55·2 (43–70·4)	6·3% (-13·5 to 30·6%)	0·76
	Algeria	19 (11·3–30·2)	53·7% (-21·7 to 206·7%)	46·8 (28·1–73·9)	4·8% (-45·2 to 93·8%)	0·86
	Bahrain	0·8 (0·5–1·2)	145% (44 to 321·6%)	65·6 (41·9–99·2)	0·3% (-39·8 to 65·6%)	0·91
	Egypt	49·3 (28·7–76·3)	96·3% (6·1 to 252·8%)	51·2 (31·7–76)	25·2% (-28·1 to 108·4%)	0·58
	Iran	40·7 (31·2–51·9)	48·5% (19·8 to 84·1%)	51·5 (39·2–66·3)	20·2% (-2·5 to 47·4%)	0·82
	Iraq	24 (15–36·5)	140·8% (37·7 to 315·4%)	55·4 (36·1–82·1)	14·4% (-32·3 to 91·1%)	0·99
	Jordan	5·8 (3·5–9·4)	204·1% (65·9 to 443·5%)	49·9 (31·3–77·7)	10·7% (-35·4 to 83·8%)	0·85
	Kuwait	1·8 (1–3)	108·4% (16·3 to 277·9%)	48·3 (28·3–81·4)	-3·2% (-44·8 to 69·7%)	0·84

Table S11 Cause- and sex-specific burden, mortality, incidence, and prevalence of neurological conditions in North Africa and Middle East countries

		All Ages		Age-standardised		
		Number (thousand)	Percent change	Rate per 100,000	Percent change	Sex ratio
**Measure**		2019	From 1990 to 2019	2019	From 1990 to 2019	2019
Cause	Location	Mean (95% UI)	Mean (95% UI)	Mean (95% UI)	Mean (95% UI)	F:M
	Lebanon	2·6 (1·6–4·1)	90·1% (11·5 to 257·2%)	51·7 (32·2–80·9)	28·4% (-24·8 to 133·1%)	0·65
	Libya	3·3 (2·2–4·7)	73·8% (2·4 to 219·8%)	51·5 (34·7–73·7)	23·2% (-25 to 115·9%)	0·71
	Morocco	16·5 (9·2–26·3)	71·6% (-14·5 to 239·1%)	47·3 (26·7–75·3)	32·3% (-31·9 to 148·1%)	0·8
	Palestine	2·4 (1·4–3·9)	134·8% (25·7 to 342·6%)	47·5 (29·7–71·6)	13·9% (-34 to 103·6%)	0·78
	Oman	1·8 (1–2·9)	171·8% (39·3 to 463·2%)	46·8 (27·9–75·1)	43·2% (-21·9 to 164·3%)	0·95
	Qatar	1·2 (0·7–1·9)	393·3% (181·8 to 777·7%)	57·8 (36·9–87)	-3·3% (-42·3 to 67·3%)	1·27
	Saudi Arabia	19·7 (10·6–32·9)	138·3% (11·4 to 384%)	61·3 (34·4–101·1)	27·1% (-37·2 to 145·4%)	1·02
	Syrian Arab Republic	6·9 (4·3–10·4)	8% (-40·5 to 96·8%)	48 (30·5–71·5)	14·1% (-34·4 to 96·1%)	0·93
	Tunisia	4·7 (3–7·1)	56·2% (-11·3 to 182·6%)	42 (25·4–65·3)	24·4% (-29·8 to 119·4%)	0·8
	Türkiye	61·4 (42·7–85)	-13·6% (-43·8 to 34·7%)	83·4 (57·4–117·1)	-20·8% (-48·4 to 20·7%)	0·72
	United Arab Emirates	8·1 (5·2–11·7)	577·3% (281·2 to 1086·5%)	93·8 (59·5–135·8)	31% (-23·2 to 121·3%)	0·53
	Yemen	12·4 (6·2–21·5)	140·2% (8·3 to 487·5%)	37·9 (21·8–61·2)	20·1% (-39·2 to 161·1%)	0·84
	Afghanistan	20·5 (10·5–34·7)	221·5% (56·3 to 543·9%)	49·6 (28·8–79·6)	5·6% (-41·9 to 94·8%)	0·87
	Sudan	21·4 (12·2–34·8)	132·1% (17·3 to 374·3%)	49·7 (30·3–76·2)	33% (-28·5 to 152·1%)	0·63
Headache disorders	Global	46619·4 (9772·9–100161·7)	56·7% (52·4 to 62·1%)	581·8 (119·6–1255·6)	1·1% (-4·2 to 2·9%)	1·65
	North Africa and Middle East	4209·8 (990·1–9068·3)	103·3% (94·5 to 123·7%)	669·6 (159·1–1431·3)	0·1% (-2·4 to 2·4%)	1·65
	Algeria	288·4 (67·5–615·9)	90·7% (78·3 to 119·7%)	669·2 (155·7–1442·3)	-0·1% (-3·5 to 3·1%)	1·62
	Bahrain	10·6 (2·6–22·3)	222·7% (200·3 to 275·9%)	629 (149·4–1360·3)	-0·6% (-3·9 to 2·8%)	1·62
	Egypt	684·8 (147·1–1492)	98·4% (84·9 to 112·8%)	690·3 (156·4–1488·7)	1·9% (-4·8 to 8·6%)	1·69
	Iran	641·8 (160·7–1355·8)	80·2% (66·5 to 117·2%)	705·8 (173·2–1503·1)	0·3% (-3·3 to 5·8%)	1·59
	Iraq	279·5 (60·1–621·2)	182·7% (170·9 to 204·1%)	661·6 (153–1427·4)	0·3% (-3·3 to 3·5%)	1·63
	Jordan	77·5 (17·4–171·6)	252·5% (233·2 to 298·1%)	656·4 (153·8–1428·7)	-0·7% (-3·9 to 2·5%)	1·62
	Kuwait	33 (7·6–71·3)	191·5% (173·1 to 225·4%)	641·1 (146·8–1408)	3·1% (-2·3 to 7·9%)	1·65
	Lebanon	35·5 (8·4–77·1)	77·3% (68·9 to 92·6%)	672·7 (153·9–1457·6)	0·5% (-3·1 to 3·7%)	1·63
	Libya	49·8 (11·8–107·6)	105·3% (90·3 to 140·3%)	661·1 (155·4–1425·1)	1·3% (-2·2 to 4·6%)	1·63
	Morocco	251·6 (58·5–545)	59·6% (51·6 to 78·5%)	668·2 (156·2–1445·1)	-0·2% (-3·6 to 3·1%)	1·62
	Palestine	31·4 (6·8–70·6)	178·6% (166·2 to 198·3%)	662·3 (157·1–1439·2)	-1% (-4·4 to 2·1%)	1·63
	Oman	31·6 (7·5–67·8)	187·2% (168·1 to 223·2%)	618·5 (146·4–1332·9)	-1·2% (-4·9 to 2·6%)	1·63
	Qatar	20·2 (5–42·9)	605·4% (571·7 to 667·4%)	585 (140·4–1252)	-2·6% (-5·8 to 0·9%)	1·62
	Saudi Arabia	259·8 (62·7–548·8)	178·7% (155·8 to 227·9%)	625 (149·7–1343·4)	-1·1% (-6·2 to 3·8%)	1·52
	Syrian Arab Republic	101·4 (22·9–223·8)	40·2% (32·3 to 61·8%)	676·9 (157·1–1474·2)	1·7% (-2·1 to 5·1%)	1·63
	Tunisia	82·7 (20·2–176·6)	56·3% (46·6 to 81·3%)	673·3 (158·7–1448·7)	0·3% (-3·1 to 3·5%)	1·63
	Türkiye	588·7 (155·4–1275·3)	58% (47·2 to 81%)	656·1 (167·7–1433)	0·3% (-4·6 to 5%)	1·82
	United Arab Emirates	67·6 (17·6–141·9)	479·9% (432·5 to 583%)	598·8 (142·4–1275·1)	-0·7% (-5 to 3%)	1·62
	Yemen	193 (40·6–431·5)	169·7% (159·4 to 187·2%)	662·8 (157·9–1435·6)	0·1% (-3·4 to 3·6%)	1·61
	Afghanistan	218·4 (44·8–493·2)	242·9% (219·8 to 259·7%)	652·5 (156·7–1422·4)	-1·2% (-4·5 to 2·9%)	1·62
	Sudan	258 (54–579)	122·5% (114·3 to 132·3%)	668·3 (155·5–1438·9)	-0·1% (-3·7 to 3·7%)	1·62
**Deaths**						
Meningitis	Global	236·2 (204·4–277·4)	-45·4% (-53·5 to -35·8%)	3·3 (2·8–3·9)	-56% (-62·5 to -48·3%)	0·84
	North Africa and Middle East	6·3 (5·3–7·4)	-64·1% (-72·2 to -54·5%)	1·2 (1–1·4)	-71·6% (-77·1 to -64·9%)	0·88
	Algeria	0·3 (0·2–0·4)	-65·2% (-78·1 to -47%)	0·9 (0·7–1·1)	-74·7% (-82·4 to -64%)	1·06
	Bahrain	<0·1 (<0·1–<0·1)	-33·2% (-50·1 to -13%)	0·4 (0·3–0·5)	-73·1% (-78·8 to -66·1%)	0·85
	Egypt	0·8 (0·5–1·1)	-63·9% (-76·2 to -46·5%)	0·8 (0·6–1·2)	-72·2% (-81·4 to -58·8%)	0·65
	Iran	0·6 (0·5–0·6)	-67·7% (-75 to -57·1%)	0·8 (0·7–0·9)	-72·3% (-78 to -64·3%)	0·81
	Iraq	0·4 (0·3–0·5)	-63·2% (-73·7 to -48·1%)	1·3 (1–1·5)	-74·8% (-81·1 to -66·3%)	0·78
	Jordan	0·1 (0·1–0·1)	11·3% (-20·7 to 59·6%)	0·9 (0·8–1·2)	-58·7% (-68·7 to -43·7%)	1·03
	Kuwait	<0·1 (<0·1–<0·1)	-5·6% (-28·6 to 20·3%)	0·4 (0·3–0·5)	-59·4% (-68·3 to -49·6%)	0·59
	Lebanon	<0·1 (<0·1–<0·1)	-40% (-59·3 to -15·2%)	0·7 (0·5–0·9)	-64% (-75·3 to -50·8%)	0·69

Table S11 Cause- and sex-specific burden, mortality, incidence, and prevalence of neurological conditions in North Africa and Middle East countries

		All Ages		Age-standardised		
		Number (thousand)	Percent change	Rate per 100,000	Percent change	Sex ratio
**Measure**		2019	From 1990 to 2019	2019	From 1990 to 2019	2019
Cause	Location	Mean (95% UI)	Mean (95% UI)	Mean (95% UI)	Mean (95% UI)	F:M
	Libya	<0·1 (<0·1–0·1)	-52·1% (-68·5 to -30·9%)	0·8 (0·6–1)	-61·9% (-73·8 to -47·3%)	0·91
	Morocco	0·4 (0·3–0·5)	-74·8% (-83·3 to -63·5%)	1·3 (1–1·7)	-74·7% (-82·4 to -65·3%)	0·79
	Palestine	<0·1 (<0·1–<0·1)	-72·2% (-81·1 to -58·8%)	0·9 (0·8–1·1)	-77·8% (-83·4 to -70·1%)	0·62
	Oman	<0·1 (<0·1–<0·1)	-52·8% (-65·4 to -32·6%)	0·9 (0·8–1·1)	-64·2% (-73·3 to -49·6%)	0·83
	Qatar	<0·1 (<0·1–<0·1)	8·8% (-31·3 to 63%)	0·6 (0·4–0·8)	-73·4% (-84·2 to -60·4%)	5·72
	Saudi Arabia	0·2 (0·1–0·2)	-30·7% (-49·2 to -1·3%)	0·9 (0·7–1·1)	-69·7% (-77·9 to -55·6%)	1·35
	Syrian Arab Republic	0·2 (0·2–0·3)	-74·4% (-82·4 to -63·1%)	1·7 (1·4–2·2)	-64·9% (-74·9 to -51·9%)	0·94
	Tunisia	0·1 (0·1–0·1)	-61·5% (-75·4 to -40·8%)	0·8 (0·6–1)	-69·3% (-79·3 to -55·4%)	0·68
	Türkiye	0·4 (0·3–0·4)	-85·3% (-90·5 to -77·2%)	0·5 (0·4–0·6)	-86·7% (-91·2 to -80·4%)	0·78
	United Arab Emirates	0·1 (0–0·1)	70·1% (4·9 to 145·4%)	0·8 (0·5–1·2)	-67·5% (-76·9 to -58·1%)	0·73
	Yemen	0·5 (0·3–0·7)	-47·4% (-66·3 to -19·7%)	2 (1·4–2·7)	-60·2% (-72 to -44·3%)	0·96
	Afghanistan	1·6 (1·1–2·2)	-27·6% (-52 to 6·8%)	4·3 (3·1–5·7)	-67·3% (-77·2 to -55·4%)	1·16
	Sudan	0·7 (0·5–1)	-76·6% (-84·7 to -64·3%)	1·9 (1·3–2·5)	-80·2% (-86·3 to -71·3%)	0·76
Encephalitis	Global	89·9 (76·5–122·9)	-23·7% (-38·9 to 12·9%)	1·2 (1–1·6)	-45·7% (-56·1 to -21·1%)	0·84
	North Africa and Middle East	3·9 (3·1–5·1)	26·2% (-9·4 to 71·2%)	0·7 (0·6–0·9)	-24·9% (-42·7 to -1·9%)	1·03
	Algeria	0·1 (0·1–0·3)	18·7% (-23·4 to 79·4%)	0·4 (0·3–0·7)	-23·2% (-44·7 to 7·1%)	0·92
	Bahrain	<0·1 (<0·1–<0·1)	95% (41·5 to 152·2%)	0·2 (0·2–0·3)	-24·2% (-42·9 to -3·5%)	0·74
	Egypt	0·9 (0·5–1·4)	3·6% (-36·7 to 62·8%)	1 (0·6–1·4)	-30% (-54·8 to 2·7%)	0·94
	Iran	0·2 (0·2–0·2)	18% (-13·1 to 52·2%)	0·3 (0·2–0·3)	-15·6% (-37·9 to 4·8%)	0·84
	Iraq	0·5 (0·3–0·6)	31·1% (-15·5 to 97·9%)	1·3 (1–1·6)	-30·8% (-51·1 to -2·8%)	1·05
	Jordan	<0·1 (<0·1–<0·1)	-4·5% (-43·3 to 173·6%)	0·2 (0·1–0·2)	-65% (-78·2 to -13·6%)	1·09
	Kuwait	<0·1 (<0·1–<0·1)	73% (40 to 119·9%)	0·2 (0·1–0·2)	-22·3% (-36·3 to -4·5%)	1·2
	Lebanon	<0·1 (<0·1–<0·1)	38·4% (-6 to 106·6%)	0·3 (0·2–0·5)	-19% (-42·9 to 19·7%)	0·87
	Libya	<0·1 (<0·1–<0·1)	34·3% (-8·3 to 94·4%)	0·3 (0·2–0·7)	-9·6% (-35 to 33·9%)	0·89
	Morocco	0·1 (0·1–0·3)	18·5% (-23·8 to 72·5%)	0·5 (0·3–1)	-10·9% (-36·4 to 24·4%)	1·2
	Palestine	<0·1 (<0·1–<0·1)	50·3% (-3 to 118·4%)	0·3 (0·2–0·4)	-22% (-43 to 6·7%)	0·97
	Oman	0·1 (0·1–0·1)	21·6% (-19·6 to 83·9%)	3·2 (2·5–3·9)	-43·4% (-62·2 to -9·9%)	0·61
	Qatar	<0·1 (<0·1–<0·1)	299·1% (154·3 to 482·7%)	0·3 (0·2–0·4)	-24·6% (-49 to 11·7%)	1·33
	Saudi Arabia	0·2 (0·1–0·3)	19% (-19 to 87·5%)	0·7 (0·6–0·9)	-51·8% (-67·4 to -22·7%)	1·24
	Syrian Arab Republic	0·1 (0–0·1)	-42·5% (-64·6 to 1·8%)	0·5 (0·4–0·7)	-40·9% (-61·5 to -1·3%)	0·96
	Tunisia	<0·1 (<0·1–0·1)	8·4% (-32·7 to 62·4%)	0·3 (0·2–0·6)	-23·8% (-48·9 to 11·9%)	0·84
	Türkiye	0·2 (0·2–0·3)	-31% (-56·3 to 4·8%)	0·3 (0·2–0·4)	-50·3% (-66·7 to -25·5%)	0·88
	United Arab Emirates	<0·1 (<0·1–0·1)	294% (144·7 to 494·9%)	0·3 (0·2–0·6)	-18·7% (-41·6 to 15·4%)	0·69
	Yemen	0·1 (0·1–0·3)	62·9% (-5·7 to 178·4%)	0·5 (0·3–1)	-2% (-33·4 to 47·6%)	0·98
	Afghanistan	1 (0·7–1·6)	124·7% (57·2 to 209%)	4·8 (3·6–6·2)	-11·1% (-36·6 to 19·6%)	1·15
	Sudan	0·2 (0·1–0·4)	23·2% (-32·4 to 123·2%)	0·5 (0·3–1)	-15·8% (-47·2 to 30·9%)	0·92
Tetanus	Global	34·7 (25·9–48·5)	-87·4% (-90·5 to -81·8%)	0·5 (0·4–0·7)	-89·5% (-91·9 to -84·9%)	0·81
	North Africa and Middle East	1·3 (0·8–2)	-84·1% (-91·3 to -69%)	0·2 (0·2–0·4)	-88·9% (-93·3 to -80·6%)	0·84
	Algeria	<0·1 (<0·1–<0·1)	-77·1% (-90·6 to -38·5%)	<0·1 (<0·1–0·1)	-84·3% (-93·4 to -61·1%)	0·75
	Bahrain	<0·1 (<0·1–<0·1)	62·3% (-29·1 to 156·3%)	<0·1 (<0·1–<0·1)	-44·1% (-76·2 to -11·3%)	0·98
	Egypt	0·1 (0–0·4)	-83·9% (-94·4 to -50·8%)	0·2 (0·1–0·5)	-89·2% (-95·3 to -68·8%)	0·78
	Iran	<0·1 (<0·1–<0·1)	-91·5% (-96·4 to -75·4%)	<0·1 (<0·1–<0·1)	-92·3% (-96·6 to -81·3%)	0·7
	Iraq	<0·1 (<0·1–<0·1)	-90% (-96·7 to -53·6%)	0·1 (0–0·1)	-92·7% (-97·3 to -72·4%)	0·45
	Jordan	<0·1 (<0·1–<0·1)	-64·2% (-82·6 to 3·4%)	<0·1 (<0·1–<0·1)	-82·4% (-91·2 to -58·2%)	0·8
	Kuwait	<0·1 (<0·1–<0·1)	89·1% (-77·3 to 574%)	<0·1 (<0·1–<0·1)	-62·6% (-93·9 to -13·5%)	0·04
	Lebanon	<0·1 (<0·1–0·1)	-52·6% (-90·5 to 57·5%)	0·5 (0·1–1·9)	-79·9% (-95·6 to -34·5%)	0·75
	Libya	<0·1 (<0·1–<0·1)	-57% (-83·6 to 8·3%)	<0·1 (<0·1–0·1)	-68·6% (-89·3 to -26·5%)	0·69
	Morocco	0·1 (0–0·4)	-93·7% (-98·5 to -77%)	0·4 (0·1–1·3)	-92·6% (-98·1 to -76·1%)	0·72
	Palestine	<0·1 (<0·1–<0·1)	-88·4% (-96·7 to -47·6%)	<0·1 (<0·1–<0·1)	-90·6% (-97·2 to -66·4%)	0·7

Table S11 Cause- and sex-specific burden, mortality, incidence, and prevalence of neurological conditions in North Africa and Middle East countries

		All Ages		Age-standardised		
		Number (thousand)	Percent change	Rate per 100,000	Percent change	Sex ratio
**Measure**		2019	From 1990 to 2019	2019	From 1990 to 2019	2019
Cause	Location	Mean (95% UI)	Mean (95% UI)	Mean (95% UI)	Mean (95% UI)	F:M
	Oman	<0·1 (<0·1–<0·1)	-78·3% (-92·1 to -17·8%)	<0·1 (<0·1–<0·1)	-92·9% (-98·7 to -57·8%)	0·46
	Qatar	<0·1 (<0·1–<0·1)	225·3% (13·4 to 520·2%)	<0·1 (<0·1–<0·1)	-50·7% (-85·6 to 38·3%)	0·72
	Saudi Arabia	<0·1 (<0·1–<0·1)	-91·4% (-98·9 to -52·9%)	<0·1 (<0·1–0·1)	-95·5% (-99·1 to -73·3%)	0·62
	Syrian Arab Republic	<0·1 (<0·1–<0·1)	-96·4% (-98·9 to -86·6%)	<0·1 (<0·1–0·1)	-93·7% (-98 to -78·8%)	0·53
	Tunisia	<0·1 (<0·1–<0·1)	-75·5% (-90·8 to -38·4%)	<0·1 (<0·1–<0·1)	-80·6% (-92·5 to -51·2%)	0·75
	Türkiye	<0·1 (<0·1–<0·1)	-97·7% (-98·6 to -94·1%)	<0·1 (<0·1–<0·1)	-98·7% (-99·3 to -96%)	0·82
	United Arab Emirates	<0·1 (<0·1–0·1)	2·1% (-78·1 to 288·8%)	2 (0·3–3·5)	-85·5% (-95·9 to -33·6%)	1·78
	Yemen	0·1 (0–0·1)	-83·6% (-94·6 to -35·4%)	0·2 (0·1–0·5)	-87·7% (-95·1 to -59·4%)	0·49
	Afghanistan	0·8 (0·5–1·3)	-73·8% (-86·9 to -43·4%)	2·5 (1·4–3·8)	-87·7% (-92·8 to -77·3%)	1·01
	Sudan	<0·1 (<0·1–0·1)	-91·8% (-96·7 to -76·9%)	0·1 (0·1–0·2)	-93·1% (-97 to -85·4%)	0·48
Brain and central nervous system cancer	Global	246·3 (185·6–270·9)	76·4% (11 to 104·9%)	3 (2·3–3·4)	-1·2% (-36·8 to 13·9%)	0·71
	North Africa and Middle East	17·8 (12·1–20·9)	111·5% (30·3 to 173·4%)	3·7 (2·5–4·3)	3% (-33·4 to 30·2%)	0·87
	Algeria	0·6 (0·4–0·7)	102% (12·2 to 175·5%)	1·5 (1–1·9)	1·2% (-43·3 to 38·9%)	0·96
	Bahrain	<0·1 (<0·1–<0·1)	232·8% (94·8 to 386%)	2·1 (1·4–2·7)	-25·6% (-54·8 to 8·1%)	0·84
	Egypt	2·1 (1·4–3)	101·3% (25·7 to 185%)	2·7 (1·8–4·1)	5·4% (-28·7 to 47·6%)	0·84
	Iran	3·5 (1·8–4·2)	97·5% (5 to 138·5%)	4·6 (2·3–5·5)	-1·1% (-43·8 to 22·9%)	0·89
	Iraq	1·8 (1·3–2·4)	215·1% (65·6 to 389·7%)	6·4 (4·7–8·2)	26% (-31·9 to 93·6%)	0·94
	Jordan	0·2 (0·2–0·3)	261·5% (125·1 to 391·7%)	2·8 (2–3·5)	-5·6% (-42·1 to 30·6%)	0·9
	Kuwait	0·1 (0–0·1)	205% (87·4 to 288·9%)	1·9 (1·3–2·4)	1·6% (-38·5 to 31·3%)	0·67
	Lebanon	0·2 (0·1–0·2)	82·5% (24·5 to 153·2%)	3·2 (2·3–4·2)	-9% (-37·6 to 25·4%)	0·79
	Libya	0·2 (0·2–0·3)	114·8% (50·6 to 210·9%)	3·7 (2·8–4·9)	-0·9% (-28·4 to 40·2%)	0·63
	Morocco	0·5 (0·3–0·6)	91·3% (24·4 to 168·9%)	1·4 (1–1·9)	5·9% (-28·1 to 45·2%)	1·24
	Palestine	0·2 (0·2–0·3)	137·9% (52·4 to 224·3%)	7·2 (5·2–8·7)	-3·7% (-34·8 to 31·5%)	0·79
	Oman	0·1 (0–0·1)	176·7% (39·6 to 318·9%)	2·1 (1·4–2·6)	16·6% (-39 to 73·2%)	0·93
	Qatar	<0·1 (<0·1–0·1)	485·3% (234·8 to 814·8%)	2·8 (2·1–4)	-16·8% (-46·7 to 21·7%)	1·41
	Saudi Arabia	0·6 (0·4–0·9)	291·2% (111·7 to 651%)	2·3 (1·7–3·5)	32·8% (-27·6 to 148·5%)	0·93
	Syrian Arab Republic	0·6 (0·4–0·8)	73·8% (6·8 to 160·3%)	4·5 (3–6)	-2·6% (-34 to 43·1%)	0·85
	Tunisia	0·1 (0·1–0·2)	91·6% (22·4 to 175·2%)	1·1 (0·8–1·5)	0·5% (-34·6 to 44·2%)	1·18
	Türkiye	4·1 (1·8–5·7)	74·1% (-4·1 to 156·7%)	4·7 (2·1–6·6)	-10·8% (-48·4 to 27·2%)	0·79
	United Arab Emirates	0·3 (0·2–0·5)	572·5% (332·1 to 902·9%)	4·2 (2·6–5·7)	-6·6% (-43·9 to 46·8%)	0·79
	Yemen	0·7 (0·4–0·9)	174·2% (48·6 to 386·6%)	3·4 (2·2–5·1)	21·7% (-29·2 to 93%)	0·74
	Afghanistan	1 (0·6–1·8)	140·1% (58 to 293·1%)	4·7 (2·8–8·5)	3·2% (-32·7 to 59·4%)	0·77
	Sudan	1 (0·6–1·4)	93·2% (-1·7 to 278·8%)	3·6 (2·3–5·1)	8·2% (-38·1 to 84%)	0·7
Stroke*	Global	6552·7 (5995·2–7015·1)	43·3% (31 to 55·4%)	84·2 (76·8–90·2)	-36·4% (-41·6 to -31·2%)	0·76
	North Africa and Middle East	312·2 (278·4–349·7)	75·5% (56·2 to 98·8%)	87·7 (78·2–97·6)	-27·8% (-35·4 to -16%)	1·07
	Algeria	24·8 (20–30·2)	77·1% (38·3 to 122·6%)	101·5 (82·6–121·6)	-43·4% (-54·9 to -30·2%)	1·35
	Bahrain	0·2 (0·2–0·3)	109·6% (64·1 to 168·7%)	52·8 (43·5–66·9)	-48·5% (-58·4 to -36·5%)	1·22
	Egypt	45·8 (33·6–63·2)	32·2% (-2·7 to 75·5%)	85·7 (63·1–118·9)	-22·6% (-42·6 to 1·6%)	1·4
	Iran	40·9 (36·7–43·8)	88·6% (67·7 to 118·5%)	66·2 (58·7–71·3)	-45·1% (-50·6 to -35·4%)	1·05
	Iraq	26·3 (21·4–31·1)	123·8% (77·4 to 177·3%)	143·3 (119·2–166·1)	-13·7% (-30·2 to 4·5%)	0·83
	Jordan	3·4 (2·8–4)	136·2% (89·6 to 188·2%)	75·7 (61·3–89)	-49·8% (-59·4 to -39·2%)	1·2
	Kuwait	0·9 (0·8–1·1)	301·8% (235·8 to 381·5%)	46·5 (38–55·2)	-9·6% (-24·2 to 7·7%)	0·65
	Lebanon	1·8 (1·2–2·3)	81% (30·1 to 143·2%)	35·2 (24·5–45·6)	-39·5% (-55·5 to -19·2%)	0·97
	Libya	3·1 (2·4–4)	97·1% (51·4 to 161·9%)	69·4 (53–90·1)	-17·9% (-36·4 to 7·9%)	1·12
	Morocco	29 (23·3–35·1)	96·6% (55·4 to 142·5%)	116·4 (94·2–139·3)	-11·9% (-29·1 to 8%)	0·99
	Palestine	2 (1·7–2·3)	59·8% (25·5 to 102·3%)	122·4 (105·6–138·2)	-29·1% (-43·5 to -10·1%)	0·94
	Oman	1 (0·9–1·2)	36·7% (4·9 to 81·9%)	103·7 (90·7–118·7)	-28·8% (-45 to -3·7%)	0·85
	Qatar	0·2 (0·1–0·2)	197·2% (112·5 to 306·7%)	52·9 (42·5–69·2)	-36·7% (-54 to -15·7%)	1·91

Table S11 Cause- and sex-specific burden, mortality, incidence, and prevalence of neurological conditions in North Africa and Middle East countries

		**All Ages**		**Age-standardised**		
		Number (thousand)	Percent change	Rate per 100,000	Percent change	Sex ratio
**Measure**		2019	From 1990 to 2019	2019	From 1990 to 2019	2019
Cause	Location	Mean (95% UI)	Mean (95% UI)	Mean (95% UI)	Mean (95% UI)	F:M
	Saudi Arabia	12·7 (9·8–15·4)	83·2% (32·6 to 152·7%)	102·7 (80·4–120·5)	-32·5% (-49·1 to -10%)	1·03
	Syrian Arab Republic	9·2 (7·1–11·8)	24·4% (-8·8 to 68·6%)	99 (78·3–124·9)	-32·2% (-50·2 to -8·6%)	1·19
	Tunisia	8·7 (6·6–11·1)	114·5% (59 to 186%)	80 (60·6–101·4)	-25% (-43·8 to -0·6%)	0·88
	Türkiye	48·9 (39·2–59·5)	121% (59 to 179·6%)	60·6 (48·7–73·6)	-16·5% (-39·5 to 6·2%)	0·99
	United Arab Emirates	2·2 (1·5–3)	348·7% (202 to 555%)	91·3 (70·2–118·7)	-50% (-62 to -34·9%)	0·96
	Yemen	14·4 (10·9–18·9)	108·6% (63·7 to 174·6%)	135·7 (102·4–176·6)	-22·1% (-38·2 to 0·7%)	1·04
	Afghanistan	16·8 (11·7–21·9)	39·3% (7·3 to 74·3%)	161·5 (110·3–208·5)	-18·7% (-36·1 to -1·6%)	1·35
	Sudan	19·6 (14·4–27·8)	34·7% (8·4 to 70%)	125·2 (92·6–174·9)	-29·4% (-42·2 to -12%)	1·13
Ischaemic stroke	Global	3293·4 (2973·5–3536·1)	60·7% (45·8 to 74·7%)	43·5 (39·1–46·8)	-33·6% (-39·2 to -28·2%)	0·81
	North Africa and Middle East	210·1 (187·1–234)	141·8% (110·1 to 175%)	62·9 (56·3–69·9)	-9·1% (-20·7 to 4%)	1·1
	Algeria	18·7 (14·8–22·9)	154·5% (98·1 to 225·9%)	80·1 (64·4–96·8)	-27·9% (-42·2 to -10·5%)	1·38
	Bahrain	0·1 (0·1–0·2)	144·8% (93·3 to 209·7%)	37·5 (30·5–47·6)	-41·4% (-53·2 to -27·1%)	1·23
	Egypt	30·1 (21·9–41·5)	137% (65·8 to 212·8%)	62·3 (46–86·1)	15·3% (-17·3 to 51·5%)	1·51
	Iran	33·2 (29·5–35·7)	109·7% (83·3 to 144·4%)	54·8 (48·3–59·2)	-42·7% (-49·7 to -32·3%)	1·09
	Iraq	16·7 (13·7–19·4)	150·2% (98·3 to 210·1%)	101 (83·9–116)	-1·9% (-21·6 to 19%)	0·86
	Jordan	2·5 (2–2·9)	155·9% (98·6 to 214·9%)	59·2 (47·4–69·3)	-46·9% (-57·8 to -35·2%)	1·25
	Kuwait	0·6 (0·5–0·7)	317·1% (246·1 to 401·3%)	34·2 (27·7–41)	-10·5% (-25·6 to 6·9%)	0·7
	Lebanon	1·5 (1–1·9)	140·4% (63·2 to 215·4%)	29·4 (19·6–38·2)	-25% (-48 to -1·1%)	0·96
	Libya	2·3 (1·7–3·1)	181·8% (117·4 to 274·3%)	54·1 (40·9–72·5)	5·7% (-18·7 to 38·2%)	1·11
	Morocco	21·2 (17–25·8)	184% (118·6 to 248·7%)	88·9 (71·8–108·4)	19% (-6·7 to 43·8%)	1·01
	Palestine	1·5 (1·3–1·7)	89·4% (48·2 to 142·7%)	96·5 (82–109·6)	-15·9% (-33 to 6·2%)	0·96
	Oman	0·6 (0·6–0·8)	82·1% (38·5 to 148·3%)	76·6 (63·8–89·9)	-7·7% (-30·7 to 24·4%)	0·64
	Qatar	0·1 (0·1–0·1)	195·5% (108·4 to 300·9%)	36·9 (29·4–48·4)	-28·6% (-47·9 to -4·3%)	1·93
	Saudi Arabia	7 (5·3–8·4)	93·4% (39·7 to 159·4%)	70·1 (53·5–82·2)	-22·8% (-41·9 to 2·1%)	1·05
	Syrian Arab Republic	5 (3·8–6·3)	58·3% (10·8 to 111·7%)	58·3 (46·2–73·1)	-20·6% (-42·8 to 6·2%)	1·35
	Tunisia	6·7 (5–8·7)	204·9% (114·3 to 304·8%)	63·2 (47·3–80·4)	-2·2% (-29·8 to 27·8%)	0·9
	Türkiye	30·2 (24·1–36·7)	173·4% (88·9 to 259·4%)	38·3 (30·5–46·5)	-3·7% (-32·5 to 26·5%)	1·05
	United Arab Emirates	1·2 (0·9–1·7)	458·2% (277·6 to 709·3%)	71·4 (55·2–91·1)	-41·1% (-55·1 to -23·6%)	0·99
	Yemen	8·9 (6·9–11·7)	247·2% (169·8 to 350·3%)	93·3 (71·2–121·2)	16·4% (-7·7 to 47·9%)	1·07
	Afghanistan	8·7 (6·2–11·6)	107·2% (59·7 to 164·3%)	98 (69·2–129·4)	23·6% (-3·3 to 55·2%)	1·32
	Sudan	13·1 (9·8–18·9)	115·4% (70·7 to 171·4%)	90·3 (66·7–129)	4·9% (-14·6 to 29·6%)	1·14
Intracerebral haemorrhage	Global	2886·2 (2644·5–3099·4)	37·5% (21·7 to 50·9%)	36 (33–38·7)	-35·6% (-42·8 to -29·2%)	0·7
	North Africa and Middle East	88·5 (77·9–101·1)	18% (1·5 to 37·8%)	21·6 (19–24·3)	-51·4% (-58·1 to -41·3%)	0·99
	Algeria	5·3 (4·1–6·7)	-7·7% (-30·8 to 21·9%)	18·5 (14·5–23·6)	-69·3% (-76·8 to -60·1%)	1·24
	Bahrain	0·1 (0·1–0·1)	76·1% (33·5 to 133·1%)	13·1 (10·7–16·9)	-61·2% (-70 to -50·1%)	1·24
	Egypt	13·7 (9·5–19·5)	-19% (-42·5 to 13·9%)	20·7 (14·5–30·1)	-56·1% (-69·1 to -38·9%)	1·13
	Iran	6·3 (5·8–6·7)	34% (14·5 to 54·5%)	9·3 (8·4–9·9)	-52·5% (-59·6 to -43·2%)	0·86
	Iraq	9 (7·1–11·2)	102·5% (52·5 to 163·8%)	40 (32·2–48)	-28·3% (-45·3 to -7·8%)	0·75
	Jordan	0·8 (0·7–1)	93·7% (53·3 to 142·9%)	15·2 (12·6–18·1)	-58·3% (-66·7 to -47·5%)	1·08
	Kuwait	0·3 (0·2–0·3)	313·5% (222·5 to 417·9%)	10·8 (8·5–13·1)	-3·7% (-26·2 to 21·5%)	0·48
	Lebanon	0·3 (0·2–0·4)	-16·7% (-40·7 to 19·7%)	5 (3·7–6·9)	-69·1% (-78·1 to -55·1%)	0·98
	Libya	0·7 (0·5–0·9)	15·4% (-15·4 to 60%)	13·3 (9·7–18·1)	-52·9% (-65·2 to -35·6%)	1·16
	Morocco	6·7 (5·1–8·4)	10·4% (-19·5 to 48%)	23·6 (18·4–29·1)	-51·2% (-63·6 to -34·6%)	0·9
	Palestine	0·5 (0·4–0·5)	7·6% (-17 to 41·9%)	24·1 (20·3–28)	-55·6% (-65·6 to -42·4%)	0·83
	Oman	0·3 (0·3–0·4)	-1·8% (-25·4 to 31·6%)	24 (20·4–30·5)	-56·6% (-67·7 to -40%)	1·89
	Qatar	0·1 (0·1–0·1)	187·7% (95·8 to 302·8%)	12·6 (9·8–16·3)	-49·3% (-64 to -28·1%)	1·83
	Saudi Arabia	5·3 (4–6·6)	74·4% (23·4 to 158·7%)	31·1 (24–37·4)	-46·3% (-60·6 to -22·4%)	0·96

Table S11 Cause- and sex-specific burden, mortality, incidence, and prevalence of neurological conditions in North Africa and Middle East countries

		All Ages		Age-standardised		
		Number (thousand)	Percent change	Rate per 100,000	Percent change	Sex ratio
**Measure**		2019	From 1990 to 2019	2019	From 1990 to 2019	2019
Cause	Location	Mean (95% UI)	Mean (95% UI)	Mean (95% UI)	Mean (95% UI)	F:M
	Syrian Arab Republic	3·9 (3–5·2)	11·3% (-20·6 to 55·8%)	37·9 (29·1–49·4)	-42·3% (-58·3 to -20·1%)	1·00
	Tunisia	1·7 (1·3–2·2)	10·3% (-23·7 to 59·9%)	14·5 (10·8–19·1)	-59·4% (-71·5 to -41·3%)	0·82
	Türkiye	15·6 (12·4–19·2)	74·2% (26 to 123·4%)	18·7 (15–22·9)	-30·5% (-49 to -9·9%)	0·91
	United Arab Emirates	0·8 (0·5–1·3)	264·2% (123 to 462·5%)	17·9 (12·5–27·2)	-67·8% (-77·7 to -54·3%)	0·83
	Yemen	4·6 (3·4–6·2)	25·2% (-7·6 to 69·9%)	36 (26·8–46·8)	-55·8% (-66·7 to -40·7%)	0·97
	Afghanistan	7 (4·9–9·3)	2·4% (-23·5 to 35·6%)	54·1 (36·6–72·4)	-47·4% (-60 to -33·1%)	1·38
	Sudan	5·7 (3·8–8)	-21·7% (-40·6 to 3·9%)	30·4 (20·9–42·2)	-61·5% (-70·1 to -49%)	1·11
Subarachnoid haemorrhage	Global	373·1 (330–415·9)	-12·1% (-25·3 to 26·3%)	4·7 (4·1–5·2)	-57·4% (-63·8 to -38·8%)	0·86
	North Africa and Middle East	13·6 (11·2–16·8)	-15% (-35·7 to 38·8%)	3·2 (2·6–3·9)	-59% (-70·7 to -31·6%)	1·05
	Algeria	0·9 (0·7–1·2)	-8·1% (-36·8 to 47·8%)	2·9 (2·1–3·7)	-64·6% (-75·9 to -42·9%)	1·14
	Bahrain	<0·1 (<0·1–<0·1)	84·3% (23·7 to 177%)	2·2 (1·7–3·3)	-52·9% (-69·4 to -32·1%)	0·97
	Egypt	2 (1·4–2·9)	-60% (-74·5 to -18·4%)	2·8 (1·9–3·9)	-71·4% (-81·7 to -44·7%)	1·06
	Iran	1·5 (1·3–1·7)	23·2% (-13·9 to 95·6%)	2·1 (1·8–2·4)	-60·6% (-73·8 to -34·9%)	0·98
	Iraq	0·6 (0·4–0·8)	-8·1% (-42·6 to 65%)	2·3 (1·7–3·3)	-68·4% (-80·4 to -40·9%)	0·91
	Jordan	0·1 (0·1–0·1)	107·4% (41·9 to 224·3%)	1·2 (0·9–1·6)	-54·9% (-69·5 to -30·4%)	0·96
	Kuwait	<0·1 (<0·1–0·1)	130·4% (74·7 to 243·4%)	1·6 (1·3–2)	-24·8% (-43 to 3·4%)	0·95
	Lebanon	<0·1 (<0·1–0·1)	-32·3% (-66·5 to 27·1%)	0·7 (0·4–1)	-72·7% (-86·7 to -47·5%)	1·18
	Libya	0·1 (0·1–0·2)	-33·9% (-57·7 to 27·1%)	2 (1·4–2·9)	-60·9% (-73·2 to -36·3%)	1·17
	Morocco	1·1 (0·8–1·6)	-9·3% (-39·6 to 56·5%)	3·9 (2·8–5·6)	-56·8% (-71·3 to -21·3%)	1·08
	Palestine	<0·1 (<0·1–<0·1)	28·5% (-13·1 to 95·7%)	1·8 (1·5–2·2)	-49·4% (-66·2 to -22·8%)	1·29
	Oman	0·1 (0–0·1)	-15·5% (-50·8 to 71·4%)	3·1 (1·8–4·7)	-58·1% (-77·1 to -20·2%)	1·47
	Qatar	<0·1 (<0·1–<0·1)	225·6% (85·7 to 425·6%)	3·5 (2·6–4·8)	-51·2% (-72·8 to -12·8%)	2·11
	Saudi Arabia	0·3 (0·2–0·5)	41·4% (-22·5 to 148·8%)	1·5 (1·1–2)	-57·5% (-76·8 to -25·9%)	1·27
	Syrian Arab Republic	0·3 (0·3–0·4)	-55·5% (-70·1 to -25·7%)	2·8 (2·1–3·7)	-59·5% (-73·3 to -35·3%)	0·87
	Tunisia	0·3 (0·2–0·4)	-13·3% (-48·6 to 52·4%)	2·3 (1·6–3·2)	-64·2% (-79·3 to -35·6%)	0·97
	Türkiye	3·1 (2·4–3·9)	46·3% (-11·3 to 180·6%)	3·6 (2·8–4·5)	-39·1% (-63·8 to 20·4%)	0·84
	United Arab Emirates	0·1 (0·1–0·2)	205·7% (71·8 to 453·8%)	2 (0·9–3·2)	-65·2% (-78·1 to -42·5%)	1·24
	Yemen	0·8 (0·4–1·4)	31·6% (-6·7 to 111·7%)	6·4 (3·2–11·1)	-49·2% (-63·4 to -17·8%)	1·11
	Afghanistan	1·2 (0·4–2·1)	8·3% (-24·9 to 76·4%)	9·4 (2·8–17)	-42·7% (-58·3 to -11·8%)	1·48
	Sudan	0·9 (0·5–1·5)	-31·1% (-52·4 to 35·5%)	4·5 (2·4–7·6)	-63·4% (-75·2 to -30·9%)	1·04
Neurological disorders†*	Global	2221·3 (1027·9–4759·8)	154·3% (120·5 to 174·1%)	30·7 (13·8–66·3)	1·2% (-2·8 to 7·7%)	0·98
	North Africa and Middle East	99·6 (47·3–213·2)	153·3% (111 to 207·8%)	33·2 (14·3–73·9)	-2·8% (-9·6 to 13·9%)	1·01
	Algeria	7·2 (3·2–16·7)	200% (114 to 293·4%)	34·2 (13·6–81·7)	-10·5% (-26·8 to 6·2%)	1·07
	Bahrain	0·1 (0·1–0·3)	273·8% (183 to 359·2%)	36 (15·2–80·7)	-7·1% (-21·6 to 10·1%)	1·02
	Egypt	10·7 (5·1–22·2)	109·8% (70 to 162·7%)	31·2 (12·8–70·1)	1·1% (-15·1 to 25·8%)	1·11
	Iran	18·3 (8–41·4)	276·2% (178·8 to 397·5%)	32·1 (13·2–73·9)	-1·4% (-8·6 to 27·8%)	1·01
	Iraq	4·7 (2·1–9·9)	134% (95·7 to 183·2%)	32·4 (13·3–72)	1·2% (-13·6 to 22·6%)	0·88
	Jordan	1·1 (0·5–2·4)	313% (232·7 to 404·2%)	30·3 (12·4–67·6)	-9·4% (-23·2 to 11·5%)	1·02
	Kuwait	0·5 (0·2–1·2)	331% (223·4 to 405%)	29·9 (11·4–69)	-7·2% (-20 to 4·2%)	0·97
	Lebanon	1·6 (0·6–3·8)	210·9% (145·6 to 344·6%)	33 (12·6–78·5)	-4·9% (-21·7 to 37·3%)	1·03
	Libya	1·4 (0·6–3·2)	154·3% (102·3 to 218·1%)	35·8 (14·3–80·8)	-1·9% (-19·7 to 23·8%)	1·00
	Morocco	6·9 (3–15·7)	160·3% (118·3 to 199·1%)	33·1 (13·6–78)	6·7% (-8·8 to 26·7%)	0·9
	Palestine	0·5 (0·3–1·1)	96·8% (60·8 to 145·7%)	34·5 (14·9–80·3)	-6·6% (-22·6 to 16·4%)	0·89
	Oman	0·3 (0·1–0·5)	131·4% (91·2 to 213·3%)	39·1 (17·1–90·1)	4·7% (-12·2 to 49·9%)	0·9
	Qatar	0·1 (0–0·2)	390·2% (267·2 to 553·5%)	40·3 (18·1–90·3)	10·9% (-10·4 to 40·8%)	1·12
	Saudi Arabia	3·1 (1·7–5·9)	116·5% (71·7 to 197·8%)	36·3 (16·7–78·1)	-1·9% (-19·5 to 35·2%)	0·89
	Syrian Arab Republic	2·2 (1–5·1)	77·4% (35·5 to 138·7%)	33 (13·1–78·3)	4·9% (-16 to 40%)	1·04
	Tunisia	3·2 (1·3–7·4)	225·2% (139 to 332·1%)	32·7 (12·4–75·9)	-2·1% (-22·3 to 32·4%)	0·95

Table S11 Cause- and sex-specific burden, mortality, incidence, and prevalence of neurological conditions in North Africa and Middle East countries

		**All Ages**		**Age-standardised**		
		Number (thousand)	Percent change	Rate per 100,000	Percent change	Sex ratio
**Measure**		2019	From 1990 to 2019	2019	From 1990 to 2019	2019
Cause	Location	Mean (95% UI)	Mean (95% UI)	Mean (95% UI)	Mean (95% UI)	F:M
	Türkiye	26·6 (12·1–60·8)	137·3% (80 to 193·6%)	34·5 (15·5–78·3)	-6·9% (-22·9 to 11·4%)	1·04
	United Arab Emirates	0·5 (0·4–0·8)	544% (363·6 to 791·4%)	35·6 (16·8–76·6)	-7% (-22·2 to 23·5%)	0·89
	Yemen	2·7 (1·3–5·8)	170% (94·7 to 258·2%)	32·1 (12·5–77·4)	1·8% (-13·6 to 25·9%)	0·95
	Afghanistan	3·4 (2–6·5)	62·7% (37·1 to 99·1%)	41·1 (17·7–92·4)	-6·7% (-22·5 to 7·6%)	0·98
	Sudan	4·4 (2·1–9·4)	85% (31·1 to 138·4%)	31·4 (13–72·5)	-6·9% (-21·2 to 10·2%)	0·92
Alzheimer's disease and other dementias	Global	1623·3 (407·5–4205·7)	189·4% (173·5 to 214·5%)	22·9 (5·8–59·2)	3% (-1·2 to 9·8%)	1·17
	North Africa and Middle East	70·5 (17·2–185·8)	191·6% (169·8 to 253·2%)	25·5 (6·3–67·1)	-2·3% (-9 to 17·3%)	1·12
	Algeria	5·2 (1·3–15)	289·7% (212·2 to 394·7%)	27 (6·6–74·5)	-6·3% (-20·6 to 11%)	1·12
	Bahrain	0·1 (0–0·2)	374·9% (290·8 to 482·7%)	27·3 (6·6–72)	-4·4% (-18·2 to 13·2%)	1·14
	Egypt	6·9 (1·6–18·5)	110·8% (72 to 172·7%)	23·6 (5·4–63·1)	-1·7% (-17·6 to 23·2%)	1·14
	Iran	13·9 (3·4–37·2)	382% (336·4 to 552·3%)	25·3 (6·4–67·6)	-0·6% (-7·1 to 34·5%)	1·1
	Iraq	3·3 (0·8–8·5)	135·7% (99·4 to 182·6%)	25·3 (6·1–65·7)	-0·5% (-14·6 to 18·8%)	1·05
	Jordan	0·8 (0·2–2·2)	356·6% (286·2 to 471·1%)	24 (6–62)	-7·6% (-21·4 to 15·9%)	1·12
	Kuwait	0·4 (0·1–1·1)	439·9% (380·6 to 519·8%)	25·1 (6·5–64)	-2·1% (-12·8 to 10·4%)	1·08
	Lebanon	1·3 (0·3–3·4)	246% (186·4 to 425·1%)	26·8 (6·5–70·8)	-2·6% (-19·7 to 48%)	1·19
	Libya	1·1 (0·3–2·8)	162·3% (114 to 232·1%)	28·6 (7·2–72·8)	-3·8% (-21·4 to 22%)	1·11
	Morocco	4·9 (1·1–13·8)	161·9% (120·2 to 204·4%)	25·4 (5·9–70·2)	0·7% (-14·2 to 16·1%)	1·01
	Palestine	0·3 (0·1–0·9)	98% (65·5 to 145%)	25·9 (6·1–72·1)	-6·1% (-21 to 15·6%)	1·02
	Oman	0·2 (0–0·4)	117·5% (79·4 to 214·4%)	28·8 (6·7–81)	-3·5% (-20·3 to 46·7%)	1·11
	Qatar	<0·1 (<0·1–0·1)	409·8% (276·9 to 594·1%)	27·9 (6·5–76)	5·8% (-14·1 to 28·2%)	1·12
	Saudi Arabia	1·7 (0·4–4·5)	106·2% (67·4 to 196·6%)	26·3 (6·3–68·4)	-2·1% (-19·6 to 41%)	1·09
	Syrian Arab Republic	1·6 (0·4–4·6)	75% (34·9 to 142·1%)	26 (6·3–70·3)	3·1% (-18·3 to 44·3%)	1·14
	Tunisia	2·5 (0·6–6·8)	270·1% (189 to 428%)	26·5 (6·3–70·3)	-2·8% (-22·7 to 37%)	1·07
	Türkiye	19·7 (5·1–53·6)	190·5% (141·7 to 254%)	25·8 (6·6–70)	-3·8% (-20·1 to 16·3%)	1·19
	United Arab Emirates	0·1 (0–0·4)	496·6% (359·4 to 708·4%)	24·2 (5·9–64·2)	-6·1% (-19·9 to 26·8%)	1·07
	Yemen	1·7 (0·4–4·7)	227·9% (174·9 to 303·6%)	25·5 (6·2–71·1)	0·7% (-14 to 21·5%)	1·05
	Afghanistan	1·8 (0·4–4·8)	59·1% (32 to 85·4%)	30·8 (7·5–82·3)	-3·3% (-18·5 to 10%)	1·05
	Sudan	2·8 (0·7–8)	132·5% (103·4 to 176·2%)	23·9 (5·6–66)	-5·1% (-16·2 to 11·2%)	1·06
Parkinson's disease	Global	362·9 (326·9–388·2)	146·5% (128·1 to 162·8%)	4·8 (4·3–5·1)	3·8% (-3·3 to 10·1%)	0·53
	North Africa and Middle East	16·8 (14·6–21·6)	179·4% (145·9 to 225·5%)	5·3 (4·6–6·9)	2·3% (-10 to 19%)	0·66
	Algeria	1·3 (1–1·6)	192·2% (125·1 to 277·6%)	5·4 (4·4–6·7)	-18·7% (-36·3 to 4·6%)	0·84
	Bahrain	<0·1 (<0·1–<0·1)	286·6% (187·6 to 403·7%)	6 (4·2–7·4)	-7·7% (-29·9 to 17·9%)	0·7
	Egypt	2·4 (1·8–3·7)	133·4% (84·4 to 193·2%)	6 (4·6–9·3)	12·2% (-10·6 to 38·2%)	1·18
	Iran	2·8 (2·4–3·2)	343·1% (276·8 to 429·9%)	4·8 (4·1–5·3)	6·7% (-12·6 to 28·4%)	0·67
	Iraq	0·8 (0·7–1·2)	202% (137 to 289·8%)	5·7 (4·6–7·4)	22·8% (-3·6 to 58%)	0·39
	Jordan	0·2 (0·2–0·2)	332·8% (245·4 to 442·6%)	4·9 (4·1–5·8)	-13·2% (-30 to 8·6%)	0·67
	Kuwait	0·1 (0–0·1)	253·2% (199·8 to 317·8%)	3·5 (2·8–4·2)	-25·1% (-36·3 to -11·7%)	0·46
	Lebanon	0·2 (0·2–0·3)	171·3% (98·1 to 266·4%)	4·4 (3·5–6)	-14·1% (-37·2 to 15·7%)	0·55
	Libya	0·2 (0·2–0·3)	202·9% (125 to 314·9%)	5 (3·7–6·5)	9·9% (-18·1 to 50·5%)	0·62
	Morocco	1·4 (1·1–1·6)	244·3% (174·9 to 340·4%)	6 (4·9–7)	46·6% (16·7 to 85·9%)	0·54
	Palestine	0·1 (0·1–0·1)	118·5% (64·7 to 206·9%)	6·2 (4–7·2)	-2·5% (-26·2 to 36·2%)	0·55
	Oman	0·1 (0–0·1)	185·9% (105·3 to 287·6%)	9·1 (4·9–10·7)	44·7% (6·3 to 96%)	0·45
	Qatar	<0·1 (<0·1–<0·1)	450·7% (294·9 to 662·2%)	11·1 (6·5–14·7)	35·3% (1·2 to 78·3%)	1·00
	Saudi Arabia	0·6 (0·4–0·7)	108% (58·2 to 207·5%)	7·3 (5–8·7)	0·2% (-22·2 to 47·7%)	0·36
	Syrian Arab Republic	0·4 (0·3–0·6)	151% (86·5 to 240·1%)	5·7 (3·7–7·2)	21·1% (-8·4 to 61·8%)	0·69
	Tunisia	0·5 (0·4–0·6)	237·3% (150·4 to 341·6%)	4·6 (3·4–6)	12% (-16·4 to 45·8%)	0·52
	Türkiye	3·8 (2·9–7·1)	164·1% (106 to 229·6%)	4·9 (3·7–9·1)	-6·8% (-27 to 16·1%)	0·67
	United Arab Emirates	0·1 (0·1–0·1)	527·4% (356 to 824·1%)	8 (4·8–10·4)	-12·8% (-32·3 to 23·5%)	0·58
	Yemen	0·4 (0·3–0·5)	246% (169·5 to 358·9%)	4·6 (3·6–6)	21·9% (-3·2 to 58·7%)	0·59

Table S11 Cause- and sex-specific burden, mortality, incidence, and prevalence of neurological conditions in North Africa and Middle East countries

		All Ages		Age-standardised		
		Number (thousand)	Percent change	Rate per 100,000	Percent change	Sex ratio
**Measure**		2019	From 1990 to 2019	2019	From 1990 to 2019	2019
Cause	Location	Mean (95% UI)	Mean (95% UI)	Mean (95% UI)	Mean (95% UI)	F:M
	Afghanistan	0·6 (0·4–0·7)	50·8% (17·4 to 90·2%)	6·8 (5·3–8·5)	-2·3% (-22·7 to 20·3%)	0·68
	Sudan	0·7 (0·6–0·9)	94·9% (46·1 to 165·3%)	5·2 (4·1–6·5)	0·1% (-24·7 to 36·8%)	0·53
Idiopathic epilepsy	Global	114 (100·2–129·9)	13·9% (-1·4 to 51·6%)	1·5 (1·3–1·7)	-24·8% (-34·2 to -1·6%)	0·71
	North Africa and Middle East	6·6 (5·4–7·6)	5·3% (-16·7 to 70·4%)	1·2 (1–1·4)	-33·4% (-46·2 to 2·1%)	0·88
	Algeria	0·4 (0·3–0·5)	-16·3% (-39·6 to 30·7%)	1·1 (0·8–1·3)	-49·1% (-62·4 to -24·2%)	1·02
	Bahrain	<0·1 (<0·1–<0·1)	102·2% (56·6 to 171·7%)	1·7 (1·3–2·1)	-34·3% (-49·1 to -13·8%)	0·82
	Egypt	0·4 (0·3–0·6)	8·4% (-21·3 to 66·4%)	0·5 (0·3–0·7)	-34·5% (-52 to -4·4%)	0·68
	Iran	0·7 (0·6–0·8)	-33·7% (-49·3 to 7·6%)	0·8 (0·7–1)	-51·1% (-60·7 to -26·1%)	0·74
	Iraq	0·3 (0·2–0·4)	31·1% (-6·5 to 101%)	0·8 (0·6–1)	-42·7% (-58 to -15·7%)	0·91
	Jordan	0·1 (0·1–0·1)	107% (54·6 to 194·2%)	0·6 (0·5–0·8)	-38·7% (-52·8 to -12·8%)	0·82
	Kuwait	<0·1 (<0·1–<0·1)	65·6% (38 to 112·8%)	0·7 (0·6–0·9)	-30·6% (-42·2 to -13%)	0·8
	Lebanon	<0·1 (<0·1–0·1)	11·6% (-19·2 to 62·4%)	0·9 (0·6–1·2)	-36·7% (-54·2 to -10·2%)	0·7
	Libya	0·1 (0·1–0·1)	23·2% (-11·1 to 80·8%)	1·1 (0·8–1·5)	-25·7% (-44·4 to 3·3%)	0·82
	Morocco	0·3 (0·1–0·8)	19·9% (-21·2 to 82·6%)	0·9 (0·3–2·5)	-21·8% (-45·3 to 13·6%)	1·32
	Palestine	0·1 (0·1–0·1)	56·4% (16·7 to 142·5%)	1·6 (1·4–2)	-29·9% (-47·5 to 3%)	0·74
	Oman	<0·1 (<0·1–<0·1)	42·5% (-3·6 to 152·9%)	0·4 (0·3–0·5)	-37·8% (-56·6 to -1·6%)	0·75
	Qatar	<0·1 (<0·1–<0·1)	269·9% (170·8 to 453·3%)	0·5 (0·4–0·7)	-40·4% (-55·9 to -15·8%)	1·8
	Saudi Arabia	0·6 (0·4–0·8)	141·4% (41·8 to 298·1%)	1·8 (1·4–2·4)	-14·4% (-49·4 to 37·2%)	1·1
	Syrian Arab Republic	0·1 (0·1–0·1)	-8·3% (-37·9 to 63·4%)	0·6 (0·5–0·8)	-33·3% (-53·5 to 12·7%)	0·82
	Tunisia	0·1 (0·1–0·1)	-17% (-43·8 to 26%)	0·9 (0·6–1·2)	-40·6% (-58·7 to -11·3%)	0·76
	Türkiye	1·4 (1·1–1·7)	-17% (-41·6 to 61·2%)	1·7 (1·4–2·1)	-34·1% (-56·8 to 25·7%)	0·75
	United Arab Emirates	0·1 (0·1–0·2)	340·1% (173·1 to 610·8%)	1·4 (0·9–2·1)	-31·9% (-52·9 to 1·1%)	0·68
	Yemen	0·4 (0·3–0·5)	31·4% (-15 to 180·6%)	1·4 (0·9–1·9)	-29·8% (-52·2 to 19·6%)	0·85
	Afghanistan	0·9 (0·4–1·2)	62% (19·4 to 184·5%)	2·5 (1·3–3·5)	-41·3% (-55 to -8·7%)	1·13
	Sudan	0·6 (0·4–0·8)	-13·4% (-49·1 to 109·2%)	1·6 (1–2·1)	-45·5% (-65·4 to 4·4%)	0·76
Multiple sclerosis	Global	22·4 (20·2–27·8)	68% (40·1 to 84%)	0·3 (0·2–0·3)	-14% (-29·1 to -5·9%)	1·35
	North Africa and Middle East	1·4 (1·2–1·8)	147·3% (83·1 to 248%)	0·3 (0·2–0·3)	5·1% (-25 to 47·6%)	1·21
	Algeria	0·1 (0·1–0·1)	166·5% (59·6 to 303%)	0·3 (0·1–0·4)	1·6% (-38·3 to 50·7%)	1·43
	Bahrain	<0·1 (<0·1–<0·1)	422·7% (202·3 to 628·6%)	0·1 (0·1–0·2)	-6·6% (-44·1 to 28·6%)	1·49
	Egypt	0·2 (0·1–0·4)	96·5% (-24·9 to 377·8%)	0·2 (0·1–0·4)	5·9% (-48 to 122·5%)	1·18
	Iran	0·4 (0·3–0·6)	175·2% (96·6 to 288·4%)	0·5 (0·4–0·7)	4·8% (-28·2 to 55·7%)	1·06
	Iraq	<0·1 (<0·1–0·1)	225·3% (107·9 to 436·1%)	0·1 (0·1–0·2)	4·8% (-31·5 to 69·7%)	1·05
	Jordan	<0·1 (<0·1–<0·1)	308·5% (164·5 to 467·6%)	0·2 (0·1–0·2)	-14·5% (-43·1 to 18%)	1·5
	Kuwait	<0·1 (<0·1–<0·1)	300·3% (202·6 to 437·9%)	0·1 (0·1–0·2)	0·8% (-23 to 34·7%)	1·09
	Lebanon	<0·1 (<0·1–<0·1)	128·8% (49·9 to 257·5%)	0·3 (0·1–0·4)	7·5% (-29·5 to 66·7%)	1·15
	Libya	<0·1 (<0·1–<0·1)	288·9% (132·2 to 591·5%)	0·3 (0·2–0·5)	38·4% (-17·3 to 139·5%)	1·21
	Morocco	0·1 (0·1–0·1)	177·1% (74·4 to 334·2%)	0·3 (0·1–0·4)	31·5% (-16·5 to 103·4%)	1·34
	Palestine	<0·1 (<0·1–<0·1)	214·7% (89·1 to 448·7%)	0·2 (0·2–0·4)	1·6% (-37·7 to 77·2%)	0·94
	Oman	<0·1 (<0·1–<0·1)	248·6% (73·1 to 534·5%)	0·3 (0·1–0·4)	16·2% (-42·4 to 115·1%)	1·14
	Qatar	<0·1 (<0·1–<0·1)	594·2% (327·3 to 934·6%)	0·1 (0·1–0·1)	-14·3% (-44·8 to 29·6%)	1·75
	Saudi Arabia	<0·1 (<0·1–0·1)	348·6% (142·5 to 1082·3%)	0·1 (0·1–0·2)	17·7% (-33·3 to 198·9%)	1·1
	Syrian Arab Republic	<0·1 (<0·1–<0·1)	123·7% (39·3 to 273·7%)	0·1 (0·1–0·2)	4·4% (-34·3 to 71·8%)	1·31
	Tunisia	<0·1 (<0·1–0·1)	178·5% (75·4 to 355·8%)	0·3 (0·1–0·4)	24·3% (-22 to 102·2%)	1·24
	Türkiye	0·2 (0·2–0·3)	53·9% (-13·4 to 151·5%)	0·2 (0·2–0·3)	-23·2% (-53·2 to 26%)	1·37
	United Arab Emirates	<0·1 (<0·1–0·1)	1132·7% (586·4 to 1830·3%)	0·4 (0·2–0·7)	24·9% (-27·7 to 81·3%)	0·95
	Yemen	<0·1 (<0·1–0·1)	270·7% (103·4 to 567·6%)	0·2 (0·1–0·3)	28·9% (-26·4 to 121·5%)	1·04
	Afghanistan	0·1 (0–0·2)	186·7% (81 to 382·9%)	0·4 (0·2–0·9)	8·8% (-28·9 to 66·6%)	1·63
	Sudan	0·1 (0–0·1)	166·2% (46·7 to 377·5%)	0·2 (0·1–0·3)	19·7% (-31·4 to 105·2%)	1·02
Motor neuron	Global	39·1 (36·6–41·1)	121·4% (108 to 135·4%)	0·5 (0·4–0·5)	12·4% (5·7 to 19·3%)	0·7

Table S11 Cause- and sex-specific burden, mortality, incidence, and prevalence of neurological conditions in North Africa and Middle East countries

		**All Ages**		**Age-standardised**		
		Number (thousand)	Percent change	Rate per 100,000	Percent change	Sex ratio
**Measure**		2019	From 1990 to 2019	2019	From 1990 to 2019	2019
Cause	Location	Mean (95% UI)	Mean (95% UI)	Mean (95% UI)	Mean (95% UI)	F:M
disease	North Africa and Middle East	1·1 (0·9–1·3)	90·4% (23·9 to 177·6%)	0·2 (0·2–0·3)	9·3% (-26·7 to 51·1%)	0·73
	Algeria	<0·1 (<0·1–<0·1)	149·1% (67·4 to 278·1%)	0·1 (0–0·1)	-2·1% (-34·8 to 49·6%)	1·03
	Bahrain	<0·1 (<0·1–<0·1)	108·8% (52·1 to 183·7%)	<0·1 (<0·1–0·1)	-63·6% (-73·4 to -51·1%)	0·96
	Egypt	0·2 (0·1–0·3)	287·6% (104·4 to 557·7%)	0·2 (0·1–0·4)	85% (-4·9 to 213·5%)	0·77
	Iran	0·1 (0·1–0·1)	268·2% (140 to 424·8%)	0·1 (0·1–0·2)	61·9% (4 to 134%)	0·9
	Iraq	<0·1 (<0·1–<0·1)	240·1% (91·6 to 463·7%)	<0·1 (<0·1–0·1)	19·7% (-35·8 to 111·1%)	0·94
	Jordan	<0·1 (<0·1–<0·1)	455·5% (263·9 to 761%)	0·1 (0–0·1)	21·1% (-22·7 to 91·8%)	0·55
	Kuwait	<0·1 (<0·1–<0·1)	12·5% (-20·2 to 52·2%)	0·1 (0·1–0·2)	-54·4% (-66·8 to -38·7%)	0·32
	Lebanon	<0·1 (<0·1–<0·1)	111·7% (19·5 to 245·5%)	0·1 (0–0·1)	7·3% (-39·3 to 75·9%)	0·73
	Libya	<0·1 (<0·1–<0·1)	297·9% (82·7 to 653·6%)	0·1 (0·1–0·1)	51·4% (-30·9 to 187%)	0·94
	Morocco	<0·1 (<0·1–<0·1)	193·5% (84 to 332·1%)	0·1 (0–0·1)	35% (-15·5 to 101·1%)	0·94
	Palestine	<0·1 (<0·1–<0·1)	285% (131·8 to 525·1%)	<0·1 (<0·1–0·1)	50·6% (-15·5 to 165·2%)	1·27
	Oman	<0·1 (<0·1–<0·1)	235·5% (71·8 to 471·5%)	0·1 (0–0·1)	25·2% (-37·3 to 123·4%)	1·24
	Qatar	<0·1 (<0·1–<0·1)	697·7% (382·4 to 1191·9%)	<0·1 (<0·1–0·1)	-4·2% (-46 to 63·4%)	1·27
	Saudi Arabia	<0·1 (<0·1–<0·1)	287·5% (87 to 737·1%)	0·1 (0·1–0·1)	24% (-40·7 to 186·8%)	0·74
	Syrian Arab Republic	<0·1 (<0·1–<0·1)	187·2% (55·7 to 392%)	0·1 (0–0·1)	34·7% (-25·8 to 136·8%)	0·73
	Tunisia	<0·1 (<0·1–<0·1)	178·8% (57·8 to 386·7%)	0·1 (0–0·1)	25·3% (-30 to 119·5%)	0·74
	Türkiye	0·6 (0·5–0·8)	44·6% (-12·4 to 119·7%)	0·8 (0·6–0·9)	-4·7% (-38·7 to 39·2%)	0·61
	United Arab Emirates	<0·1 (<0·1–<0·1)	1180·6% (407·6 to 2470·6%)	0·2 (0·1–0·3)	24·9% (-49·3 to 143·6%)	0·93
	Yemen	<0·1 (<0·1–<0·1)	223% (90·7 to 460·4%)	<0·1 (<0·1–0·1)	24% (-31·2 to 125·4%)	0·96
	Afghanistan	<0·1 (<0·1–<0·1)	92·7% (24·9 to 201·1%)	0·1 (0–0·1)	-8·7% (-41·4 to 43·1%)	1·12
	Sudan	<0·1 (<0·1–<0·1)	147·7% (49·3 to 304·6%)	0·1 (0–0·1)	21·6% (-27·9 to 102·9%)	0·93
Other neurological disorders	Global	59·6 (55·1–64·6)	74·8% (59·5 to 91·6%)	0·8 (0·7–0·8)	-0·2% (-8·2 to 9·2%)	0·74
	North Africa and Middle East	3·3 (2·9–3·8)	85·9% (42·8 to 136·2%)	0·7 (0·6–0·8)	17·4% (-7·9 to 49·9%)	0·67
	Algeria	0·2 (0·1–0·2)	95·3% (32·9 to 164·6%)	0·5 (0·4–0·5)	10·4% (-20·6 to 45·5%)	0·75
	Bahrain	<0·1 (<0·1–<0·1)	199·2% (131·5 to 284·7%)	0·8 (0·7–1)	-0·8% (-21·5 to 24·7%)	0·7
	Egypt	0·5 (0·3–0·7)	141·1% (70·4 to 228·5%)	0·7 (0·5–1)	54·1% (9 to 104·8%)	0·42
	Iran	0·4 (0·4–0·4)	138·6% (82·4 to 224·6%)	0·6 (0·5–0·6)	39·2% (8·1 to 95·9%)	0·68
	Iraq	0·2 (0·2–0·3)	144% (72·2 to 285·9%)	0·6 (0·4–0·7)	12·1% (-19·5 to 74·4%)	0·95
	Jordan	<0·1 (<0·1–0·1)	201% (120·6 to 307·9%)	0·5 (0·4–0·6)	-0·3% (-23·7 to 30·5%)	0·62
	Kuwait	<0·1 (<0·1–<0·1)	81·1% (39·7 to 130·5%)	0·4 (0·3–0·4)	-24·6% (-40·4 to -5·3%)	0·67
	Lebanon	<0·1 (<0·1–<0·1)	171·8% (93·4 to 278·8%)	0·6 (0·5–0·8)	46% (3·4 to 100·9%)	0·39
	Libya	<0·1 (<0·1–0·1)	190·1% (89·2 to 360%)	0·7 (0·5–0·8)	58·5% (6·7 to 145·6%)	0·56
	Morocco	0·2 (0·1–0·2)	129·5% (71·1 to 211·1%)	0·5 (0·3–0·6)	48·6% (12 to 96·5%)	0·56
	Palestine	<0·1 (<0·1–<0·1)	116·4% (43·9 to 267·2%)	0·5 (0·4–0·6)	15·9% (-19 to 95·8%)	0·66
	Oman	<0·1 (<0·1–<0·1)	215·4% (88·9 to 389%)	0·5 (0·4–0·6)	62·6% (1·1 to 157·2%)	1·12
	Qatar	<0·1 (<0·1–<0·1)	348·7% (207 to 595·6%)	0·7 (0·5–0·9)	-10·8% (-38 to 41·8%)	3·08
	Saudi Arabia	0·2 (0·1–0·2)	143·7% (49·6 to 327·5%)	0·7 (0·5–0·8)	19·2% (-23·7 to 114·1%)	1·09
	Syrian Arab Republic	0·1 (0·1–0·1)	21·2% (-17·9 to 89·9%)	0·6 (0·4–0·7)	12·7% (-21·6 to 76·8%)	1·04
	Tunisia	0·1 (0–0·1)	121·4% (46 to 215%)	0·4 (0·3–0·6)	35·9% (-9 to 91·1%)	0·55
	Türkiye	0·9 (0·7–1·1)	14·3% (-17·1 to 60·8%)	1·1 (0·9–1·4)	-14·7% (-36·2 to 19%)	0·72
	United Arab Emirates	0·1 (0·1–0·2)	1061·4% (587·3 to 1672·4%)	1·5 (0·9–2·1)	67·4% (6·1 to 138·3%)	0·33
	Yemen	0·1 (0·1–0·1)	151·4% (47·2 to 333·8%)	0·3 (0·3–0·5)	34·3% (-13·1 to 129%)	0·74
	Afghanistan	0·1 (0·1–0·2)	151·2% (77·1 to 268·8%)	0·5 (0·4–0·7)	5·5% (-23·1 to 49·9%)	0·69
	Sudan	0·2 (0·1–0·2)	145·6% (34·7 to 301·2%)	0·5 (0·4–0·8)	57·6% (-3·2 to 131·8%)	0·46
**Incidence**						
Meningitis	Global	2507·2 (2113·1–2988·7)	-23·8% (-26·5 to -20·4%)	35·4 (29·6–42·5)	-35·9% (-37 to -34·6%)	0·92

Table S11 Cause- and sex-specific burden, mortality, incidence, and prevalence of neurological conditions in North Africa and Middle East countries

		**All Ages**		**Age-standardised**		
		Number (thousand)	Percent change	Rate per 100,000	Percent change	Sex ratio
**Measure**		2019	From 1990 to 2019	2019	From 1990 to 2019	2019
Cause	Location	Mean (95% UI)	Mean (95% UI)	Mean (95% UI)	Mean (95% UI)	F:M
	North Africa and Middle East	128·5 (106·7–152·7)	-9·9% (-16·3 to -1·9%)	22·5 (18·9–26·4)	-37% (-39·9 to -33·9%)	1·12
	Algeria	7·9 (6·4–9·5)	-5·1% (-13·2 to 4·1%)	19·8 (16·4–23·5)	-33·8% (-38·6 to -28·8%)	1·03
	Bahrain	0·2 (0·2–0·3)	27·6% (9·2 to 50·4%)	19·5 (15·7–23·5)	-46% (-51·2 to -40·6%)	0·87
	Egypt	21·6 (17·6–26·8)	-6% (-16·6 to 5·7%)	21·9 (18·1–26·7)	-33·2% (-38·9 to -26·9%)	0·99
	Iran	19·8 (16·1–24·1)	-16% (-25·5 to -2·6%)	25·8 (21·1–31)	-23·5% (-29·3 to -17·2%)	2·00
	Iraq	9·4 (7·8–11·1)	5·8% (-5·5 to 17·5%)	22·9 (19·3–26·8)	-40·2% (-44·8 to -36·1%)	0·99
	Jordan	3 (2·5–3·7)	128·8% (107·8 to 154·7%)	27·4 (22·5–33·1)	-10·8% (-16·6 to -3·7%)	1·04
	Kuwait	0·7 (0·5–0·8)	64·1% (48·1 to 82·4%)	19·2 (15·6–23·2)	-20·5% (-24·8 to -15·4%)	1·09
	Lebanon	1 (0·8–1·2)	-2% (-12·5 to 9·9%)	20 (16·5–23·7)	-27·8% (-33 to -22·6%)	1·04
	Libya	1 (0·8–1·2)	-27·8% (-37·6 to -15·7%)	17·7 (14·6–21·4)	-34·7% (-40·1 to -28·9%)	1·06
	Morocco	7 (5·8–8·3)	-41·1% (-46·9 to -35·1%)	22·1 (18·5–26·2)	-45·9% (-49·8 to -41·6%)	0·93
	Palestine	0·9 (0·7–1·1)	-1% (-12·7 to 12·1%)	18·6 (15·5–22·2)	-44·1% (-48·5 to -39%)	1·03
	Oman	0·3 (0·2–0·3)	-3·7% (-13·9 to 9·4%)	8·6 (7·1–10·4)	-40·6% (-45·2 to -36·4%)	0·77
	Qatar	1·3 (1–1·6)	365·1% (306·3 to 439·9%)	56·3 (46·3–68·2)	-17·5% (-22·9 to -12·5%)	1·05
	Saudi Arabia	4·4 (3·4–5·4)	12·6% (-0·7 to 29·9%)	17 (13·6–20·6)	-33·5% (-39·9 to -26·1%)	1·12
	Syrian Arab Republic	3 (2·5–3·6)	-52·1% (-57·6 to -45·4%)	23·7 (19·9–27·8)	-37·1% (-41·5 to -32·2%)	1·01
	Tunisia	2·3 (1·9–2·7)	-10·2% (-19·6 to 0·7%)	21·8 (17·9–25·8)	-21·8% (-27·9 to -16·3%)	1·12
	Türkiye	4·6 (3·8–5·4)	-60·2% (-65·5 to -53·4%)	6·7 (5·5–8)	-63·2% (-66·9 to -59·1%)	0·77
	United Arab Emirates	1·2 (1–1·5)	126% (94·5 to 165·4%)	28·2 (24·1–33)	-36% (-40·1 to -31·9%)	1·13
	Yemen	7·7 (6·3–9·4)	15·2% (3·4 to 29·5%)	23·5 (20–27·6)	-34·6% (-39·3 to -29·3%)	0·96
	Afghanistan	25·2 (21·8–28·9)	75·6% (64·8 to 88·3%)	86·2 (76·3–96·4)	-35·7% (-38·3 to -32·9%)	1·07
	Sudan	6 (4·9–7·2)	-60·6% (-63·9 to -57·1%)	14 (11·9–16·4)	-74·4% (-75·9 to -72·6%)	0·92
Encephalitis	Global	1444·7 (1280·1–1614·9)	12·5% (8·1 to 17·1%)	19·3 (17·1–21·7)	-16·5% (-17·8 to -15·2%)	0·92
	North Africa and Middle East	58·1 (49·8–67)	46·2% (40·2 to 53·3%)	9·9 (8·5–11·3)	-4·4% (-5·8 to -3%)	1·21
	Algeria	3·4 (2·9–4)	39·9% (30·1 to 49·8%)	8·4 (7·2–9·7)	-2·4% (-6·5 to 1·9%)	1·15
	Bahrain	0·1 (0·1–0·1)	123·8% (102·1 to 145·6%)	8·2 (7–9·6)	0·5% (-3·8 to 4·8%)	1·19
	Egypt	10 (8·5–11·8)	39·1% (31·4 to 47·1%)	10 (8·6–11·6)	-10·6% (-15·2 to -5·9%)	1·12
	Iran	6·8 (5·8–7·8)	13·4% (5·7 to 22·6%)	8·7 (7·4–10·2)	1·1% (0 to 2·3%)	1·16
	Iraq	4·6 (3·9–5·3)	76·6% (66·4 to 88·5%)	11·1 (9·6–12·7)	-12·1% (-15·9 to -8%)	1·23
	Jordan	0·9 (0·8–1·1)	145·3% (130·3 to 162·4%)	8·2 (7–9·6)	-5·4% (-9·3 to -0·7%)	1·17
	Kuwait	0·3 (0·3–0·4)	114·9% (98·8 to 132·8%)	8·3 (7·1–9·8)	0·6% (-4·3 to 5%)	1·18
	Lebanon	0·6 (0·6–0·7)	31·4% (24·7 to 39·7%)	12·4 (10·9–14·1)	-12·5% (-15·7 to -8·5%)	1·38
	Libya	0·4 (0·3–0·4)	13·8% (3·2 to 27%)	6·3 (5·4–7·4)	-1·2% (-5·3 to 2·9%)	1·12
	Morocco	2·9 (2·5–3·4)	18·3% (11·8 to 26%)	8·6 (7·3–10)	-2·8% (-6·8 to 1·4%)	1·23
	Palestine	0·4 (0·4–0·5)	100·1% (87·3 to 113·4%)	8·4 (7·1–9·8)	-0·8% (-4·8 to 3·9%)	1·14
	Oman	0·6 (0·5–0·6)	50% (39·8 to 62%)	15·8 (14·1–17·6)	-21·3% (-24·5 to -17·7%)	0·88
	Qatar	0·2 (0·1–0·2)	394·9% (348·9 to 438·7%)	8·3 (7–9·6)	-1·8% (-5·9 to 2·7%)	1·18
	Saudi Arabia	2·7 (2·3–3·1)	57% (40·6 to 73·5%)	9·2 (7·9–10·7)	-11% (-15·2 to -6·3%)	1·24
	Syrian Arab Republic	1·2 (1–1·4)	-17·5% (-24·6 to -8·8%)	8·6 (7·3–10·1)	-6·9% (-11·2 to -2·6%)	1·16
	Tunisia	0·9 (0·8–1)	15·7% (6·9 to 26%)	8·4 (7·1–9·8)	-1·2% (-5·7 to 3·7%)	1·19
	Türkiye	8·1 (7–9·2)	11·8% (3·3 to 21·4%)	11·2 (9·6–13)	-2·8% (-7·1 to 2%)	1·23
	United Arab Emirates	0·5 (0·4–0·6)	241·7% (200·6 to 288·3%)	8·4 (7·1–9·8)	-0·6% (-5·8 to 4·5%)	1·15
	Yemen	2·9 (2·4–3·4)	89·1% (75·4 to 102·9%)	8·4 (7·2–9·8)	-2% (-6·8 to 2·6%)	1·15
	Afghanistan	7 (6·2–8)	225·2% (209·5 to 242·2%)	19·4 (17·5–21·5)	0·1% (-3·8 to 4·2%)	1·46
	Sudan	3·6 (3–4·3)	71·3% (59·9 to 82·7%)	8·3 (7–9·6)	-3·4% (-7·6 to 0·9%)	1·17
Tetanus	Global	73·7 (53·3–101·1)	-88% (-91·1 to -83·2%)	1 (0·7–1·4)	-90% (-92·4 to -86·1%)	0·78
	North Africa and Middle East	1·9 (1·2–2·9)	-83% (-90·6 to -68%)	0·3 (0·2–0·5)	-88·4% (-92·9 to -79%)	0·82

Table S11 Cause- and sex-specific burden, mortality, incidence, and prevalence of neurological conditions in North Africa and Middle East countries

		All Ages		Age-standardised		
		Number (thousand)	Percent change	Rate per 100,000	Percent change	Sex ratio
**Measure**		2019	From 1990 to 2019	2019	From 1990 to 2019	2019
Cause	Location	Mean (95% UI)	Mean (95% UI)	Mean (95% UI)	Mean (95% UI)	F:M
	Algeria	<0·1 (<0·1–<0·1)	-75·3% (-90·4 to -33·4%)	<0·1 (<0·1–0·1)	-83·1% (-93 to -57·7%)	0·74
	Bahrain	<0·1 (<0·1–<0·1)	71% (-23·8 to 170%)	<0·1 (<0·1–<0·1)	-39·3% (-73·2 to -5·8%)	0·93
	Egypt	0·2 (0·1–0·6)	-82·7% (-94·1 to -48·7%)	0·3 (0·1–0·7)	-88·5% (-95·1 to -67·3%)	0·76
	Iran	<0·1 (<0·1–<0·1)	-90·7% (-96·2 to -72·8%)	<0·1 (<0·1–0·1)	-91·7% (-96·3 to -79·4%)	0·69
	Iraq	<0·1 (<0·1–0·1)	-89·1% (-96·5 to -53·5%)	0·1 (0·1–0·2)	-92·2% (-97·2 to -72·2%)	0·43
	Jordan	<0·1 (<0·1–<0·1)	-58·9% (-81·7 to 18·5%)	<0·1 (<0·1–<0·1)	-80·1% (-90·8 to -53·3%)	0·79
	Kuwait	<0·1 (<0·1–<0·1)	92% (-77·1 to 609·3%)	<0·1 (<0·1–<0·1)	-60·9% (-93·8 to -5·7%)	0·04
	Lebanon	<0·1 (<0·1–0·1)	-51% (-90·3 to 59·8%)	0·7 (0·2–2·9)	-78·6% (-95·3 to -30·4%)	0·77
	Libya	<0·1 (<0·1–<0·1)	-54·9% (-82·9 to 11·7%)	<0·1 (<0·1–0·1)	-67·1% (-88·8 to -23·8%)	0·69
	Morocco	0·2 (0–0·6)	-93·2% (-98·3 to -75·6%)	0·6 (0·1–1·9)	-92·1% (-97·9 to -74%)	0·7
	Palestine	<0·1 (<0·1–<0·1)	-87% (-96·4 to -40%)	<0·1 (<0·1–0·1)	-89·7% (-96·9 to -64·2%)	0·69
	Oman	<0·1 (<0·1–<0·1)	-75·2% (-91·2 to -7·3%)	<0·1 (<0·1–0·1)	-92·1% (-98·5 to -53·2%)	0·47
	Qatar	<0·1 (<0·1–<0·1)	246·7% (18·5 to 553·5%)	<0·1 (<0·1–<0·1)	-47·2% (-84·6 to 40·9%)	0·73
	Saudi Arabia	<0·1 (<0·1–<0·1)	-90·3% (-98·7 to -46·9%)	0·1 (0–0·1)	-95% (-99·1 to -69·9%)	0·59
	Syrian Arab Republic	<0·1 (<0·1–<0·1)	-96% (-98·8 to -84·9%)	0·1 (0–0·1)	-93% (-97·7 to -76·5%)	0·52
	Tunisia	<0·1 (<0·1–<0·1)	-73·4% (-89·8 to -33·3%)	<0·1 (<0·1–0·1)	-78·8% (-92·1 to -47%)	0·74
	Türkiye	<0·1 (<0·1–<0·1)	-97·5% (-98·5 to -93·3%)	<0·1 (<0·1–<0·1)	-98·5% (-99·2 to -95·5%)	0·8
	United Arab Emirates	<0·1 (<0·1–0·1)	3·7% (-77·7 to 292%)	2·9 (0·4–5·3)	-85·1% (-95·9 to -34·4%)	1·75
	Yemen	0·1 (0–0·2)	-82·3% (-94·2 to -29·8%)	0·3 (0·2–0·7)	-87% (-94·8 to -58·9%)	0·47
	Afghanistan	1·1 (0·6–1·9)	-71·2% (-85·3 to -40%)	3·4 (2–5·2)	-86·8% (-92·3 to -75·7%)	1·00
	Sudan	0·1 (0–0·1)	-91% (-96·5 to -75·3%)	0·2 (0·1–0·4)	-92·6% (-96·8 to -84·1%)	0·47
Brain and central nervous system cancer	Global	348 (262·1–388·9)	94·4% (22 to 128·3%)	4·3 (3·3–4·9)	13·8% (-27·3 to 32·8%)	0·8
	North Africa and Middle East	27·5 (18·6–32·6)	152·5% (49·6 to 233·1%)	5·2 (3·5–6·1)	28% (-19·6 to 63·3%)	0·84
	Algeria	0·9 (0·6–1·2)	136·7% (28·9 to 225·3%)	2·3 (1·5–2·9)	28% (-30·1 to 75·1%)	0·94
	Bahrain	<0·1 (<0·1–0·1)	358% (177·2 to 552·5%)	3·6 (2·4–4·6)	9·6% (-34·1 to 55·8%)	0·79
	Egypt	2·9 (2–4·1)	128·8% (34·3 to 228%)	3·5 (2·4–5·1)	20·9% (-22·4 to 69·8%)	0·82
	Iran	5·8 (2·9–7)	130·3% (23·4 to 189·2%)	7·3 (3·7–8·8)	29·6% (-25·3 to 57·5%)	0·86
	Iraq	2·8 (2–3·6)	264% (85·4 to 481·7%)	8·5 (6·3–10·9)	45·7% (-22 to 125%)	0·96
	Jordan	0·4 (0·3–0·5)	377·4% (199·9 to 546·7%)	4·4 (3·1–5·4)	26% (-21·9 to 71·5%)	0·88
	Kuwait	0·1 (0·1–0·2)	300·1% (144·2 to 423·9%)	4 (2·8–5)	44·3% (-12·5 to 86·6%)	0·61
	Lebanon	0·4 (0·3–0·4)	195% (101·3 to 302·7%)	6·7 (4·8–8·6)	56·4% (6·3 to 111·2%)	0·75
	Libya	0·3 (0·2–0·4)	130·5% (60·2 to 239·2%)	5·2 (3·9–6·8)	15·6% (-17·6 to 64·1%)	0·61
	Morocco	0·6 (0·4–0·9)	101·7% (22·8 to 189·8%)	1·8 (1·3–2·4)	19·5% (-22·3 to 67·3%)	1·28
	Palestine	0·3 (0·2–0·4)	175·7% (74 to 272·6%)	9·4 (7·1–11·2)	9·9% (-27·2 to 49%)	0·78
	Oman	0·1 (0·1–0·2)	343·4% (121 to 582·6%)	3·7 (2·3–4·7)	69·9% (-13·9 to 153·1%)	0·9
	Qatar	0·1 (0·1–0·2)	982·3% (534·6 to 1563%)	5·3 (3·8–7·6)	36·2% (-15·7 to 100%)	1·17
	Saudi Arabia	1·3 (0·9–2)	625·3% (283·3 to 1290·8%)	4·1 (3·1–6·2)	136% (25·5 to 341·7%)	0·99
	Syrian Arab Republic	0·8 (0·6–1·1)	94·4% (18·5 to 193·9%)	6·1 (4·3–8·1)	19·5% (-19·3 to 76·6%)	0·82
	Tunisia	0·2 (0·2–0·3)	132·6% (41·8 to 241·1%)	1·9 (1·3–2·6)	39·4% (-12·3 to 103·2%)	1·14
	Türkiye	6·4 (3–8·8)	120·2% (21·9 to 234·3%)	7·6 (3·7–10·3)	25·9% (-26·3 to 82·2%)	0·75
	United Arab Emirates	0·5 (0·3–0·7)	641·7% (380·4 to 976·5%)	5·7 (3·5–7·8)	8·7% (-35·1 to 65·6%)	0·78
	Yemen	0·8 (0·5–1·2)	177·9% (48·6 to 409·1%)	3·9 (2·4–5·7)	26·2% (-26·8 to 104·5%)	0·73
	Afghanistan	1·3 (0·7–2·3)	152% (64 to 330·3%)	5·1 (3–9·3)	2·3% (-33·1 to 60·9%)	0·78
	Sudan	1·4 (0·8–2)	111% (2·4 to 346·1%)	4·4 (2·6–6·2)	16·6% (-35·3 to 106·5%)	0·69
Stroke*	Global	12224·6 (11041·8–13589·3)	70·1% (66·6 to 73·4%)	150·8 (136·5–167·5)	-16·9% (-18·4 to -15·3%)	0·99
	North Africa and Middle East	829·8 (758·4–912·8)	130·7% (124·4 to 137·7%)	183 (166·7–201·7)	-5·4% (-7·4 to -3·3%)	1·13
	Algeria	61·1 (55·1–67·9)	118·1% (106·4 to 132%)	181·7 (164·3–202·2)	-16·9% (-21·1 to -12·3%)	1·15
	Bahrain	1·2 (1·1–1·4)	240·4% (215·8 to 266·4%)	113·4 (102·3–126·6)	-32·8% (-36·8 to -28·7%)	1·26

Table S11 Cause- and sex-specific burden, mortality, incidence, and prevalence of neurological conditions in North Africa and Middle East countries

		**All Ages**		**Age-standardised**		
		Number (thousand)	Percent change	Rate per 100,000	Percent change	Sex ratio
**Measure**		2019	From 1990 to 2019	2019	From 1990 to 2019	2019
Cause	Location	Mean (95% UI)	Mean (95% UI)	Mean (95% UI)	Mean (95% UI)	F:M
	Egypt	152·7 (136·4–170·2)	138·9% (124·4 to 155·3%)	228·4 (205–255·3)	9·6% (3·5 to 16·5%)	1·3
	Iran	102·8 (90·1–117·8)	112·9% (103·9 to 123·9%)	138·8 (121·8–159·6)	-16·7% (-18·1 to -15·1%)	1·07
	Iraq	60·4 (54·9–66·1)	153·6% (141·4 to 166·3%)	241·6 (216·9–267)	-11·7% (-16·4 to -6·7%)	1·1
	Jordan	15·5 (13·8–17·3)	238% (216·8 to 261·6%)	225·9 (200·5–253·4)	-26·2% (-30·9 to -20·8%)	1·11
	Kuwait	4 (3·6–4·5)	270·9% (249·2 to 294·7%)	125·8 (113·7–140·4)	-2·7% (-8·2 to 3·3%)	0·86
	Lebanon	7·8 (7–8·7)	112% (99·5 to 126·2%)	150·5 (135·7–167·8)	-5·3% (-9·9 to 0·3%)	1·36
	Libya	9·4 (8·5–10·4)	180·8% (162·4 to 200·3%)	170·9 (153·6–190·3)	7·9% (2·1 to 14·6%)	1·5
	Morocco	63·7 (57·1–70·8)	106·6% (93·9 to 120·9%)	199·8 (179·8–222)	-4·4% (-9·8 to 1·5%)	1·2
	Palestine	4·7 (4·3–5·3)	143·1% (127·3 to 157·8%)	189·7 (170·1–212·2)	-7·6% (-13·7 to -1·7%)	1·18
	Oman	3·7 (3·3–4·2)	125·4% (112·1 to 138·6%)	196·2 (174·8–222·1)	-10·3% (-15·9 to -4·6%)	1·00
	Qatar	1·9 (1·7–2·2)	487·3% (447·3 to 527·8%)	127·1 (114·7–141·6)	-26·1% (-30·3 to -21·7%)	1·3
	Saudi Arabia	41·1 (37·4–45·2)	254·9% (234 to 278·8%)	193·9 (176·5–213·7)	11·9% (6·7 to 18%)	1·14
	Syrian Arab Republic	22·2 (20·2–24·4)	61·4% (53·1 to 70·1%)	179·3 (164–196·4)	-18·6% (-22·5 to -14·7%)	1·08
	Tunisia	19·7 (17·6–22·1)	136·2% (122·1 to 151·7%)	159·6 (144–178·9)	-1·2% (-6·6 to 4·3%)	1·13
	Türkiye	125·3 (114·5–138·1)	117·4% (106·2 to 130·1%)	145·6 (133·4–160·7)	-2·8% (-7·6 to 1·9%)	1·06
	United Arab Emirates	14·5 (12·8–16·5)	710·5% (643·9 to 778·5%)	262·2 (233·8–294·3)	-14·8% (-19·2 to -9·9%)	1·15
	Yemen	32·1 (29·3–35)	145·9% (134·6 to 157·8%)	207·9 (190·1–228·3)	-9·5% (-14 to -4·7%)	1·12
	Afghanistan	36·8 (33·6–40·3)	111·8% (99·3 to 123·9%)	217·7 (200·8–238·5)	-7·7% (-11·8 to -3·2%)	1·11
	Sudan	48·6 (44·3–53·8)	101% (91 to 112·3%)	227·1 (205–253·2)	-4·8% (-9·7 to 0·9%)	1·22
Ischaemic stroke	Global	7630·8 (6569·2–8960·4)	87·6% (83·5 to 91·7%)	94·5 (81·9–110·8)	-10% (-12 to -8%)	1·07
	North Africa and Middle East	602·5 (531·3–682·5)	166·9% (157·2 to 177·2%)	135·5 (119·7–153·6)	8·8% (6·3 to 11·4%)	1·16
	Algeria	46·6 (40·7–53·3)	161·6% (142·6 to 182·8%)	140·1 (122·9–160·1)	-1·4% (-7·3 to 5·6%)	1·17
	Bahrain	0·9 (0·7–1)	261·5% (226·2 to 300·9%)	82 (71·4–94·5)	-30·1% (-35·1 to -24·2%)	1·26
	Egypt	116·4 (100·5–133·8)	200·2% (176·1 to 227·7%)	176·6 (153·4–202·3)	35·8% (25·7 to 46%)	1·32
	Iran	82·5 (69·4–97·6)	122·7% (111·2 to 136·5%)	112·3 (94·7–133·4)	-14·2% (-16 to -12·5%)	1·1
	Iraq	42·4 (36·9–48)	168·6% (150·2 to 187·7%)	178·1 (154–203·1)	-5% (-11·7 to 2·3%)	1·19
	Jordan	12 (10·4–13·8)	245·7% (218·2 to 275·8%)	181·4 (156·6–208·7)	-24·9% (-30·9 to -18·1%)	1·12
	Kuwait	2·7 (2·4–3·2)	271·6% (243 to 304·6%)	91·5 (79·6–105·3)	-4% (-10·7 to 4·2%)	0·86
	Lebanon	6·2 (5·4–7)	143·8% (126·1 to 164·3%)	119 (104·1–135·6)	7·9% (1·1 to 15·5%)	1·39
	Libya	7·2 (6·3–8·2)	230·5% (201·8 to 261·7%)	133·9 (117–152·9)	25·3% (16·7 to 34·8%)	1·55
	Morocco	48·4 (42–55·5)	152·2% (130·5 to 175·1%)	152·9 (132·9–174·8)	14·9% (5·8 to 23·9%)	1·24
	Palestine	3·6 (3·2–4·2)	164% (142·7 to 185·3%)	149·6 (130–172·4)	2·5% (-5·9 to 11·2%)	1·21
	Oman	2·7 (2·3–3·1)	156·8% (136·8 to 175·9%)	148·9 (128·2–173·3)	3·3% (-4·6 to 11·8%)	0·87
	Qatar	1·2 (0·9–1·4)	515·8% (455·6 to 575·7%)	87·5 (75·7–101·9)	-24·8% (-30·6 to -18·4%)	1·33
	Saudi Arabia	28·1 (24·7–32)	280·6% (248·3 to 315·6%)	140·4 (123·8–159·3)	20·7% (12·7 to 29·8%)	1·18
	Syrian Arab Republic	14·8 (12·9–17)	85·1% (70·9 to 99·2%)	120·3 (105·4–136·8)	-9·9% (-16·4 to -3·4%)	1·15
	Tunisia	15·2 (13·2–17·7)	180·6% (159·2 to 204%)	123·4 (108·1–142·9)	15·9% (7·6 to 23·8%)	1·15
	Türkiye	81·6 (71·5–93·6)	138·2% (120 to 158·2%)	95·4 (83·8–109·5)	4·7% (-3 to 12·8%)	1·1
	United Arab Emirates	10·9 (9·3–12·8)	847·6% (743·8 to 952·1%)	208·2 (180·3–240·9)	-5% (-11·4 to 2·3%)	1·18
	Yemen	21·9 (19·2–24·8)	216·3% (194·7 to 239·4%)	147·6 (129·8–168·3)	17·1% (9·1 to 25·7%)	1·15
	Afghanistan	22·2 (19·4–25·6)	154·7% (130·9 to 180·7%)	140·3 (123·4–159·4)	18·9% (10·7 to 27·7%)	1·09
	Sudan	34·5 (30·2–39·6)	157·6% (139·6 to 178·8%)	166·7 (145–190·9)	22·7% (14·3 to 32·1%)	1·25
Intracerebral haemorrhage	Global	3409·1 (2970·5–3909·2)	43·2% (40·7 to 45·5%)	41·8 (36·5–47·9)	-29·2% (-30·5 to -27·8%)	0·78
	North Africa and Middle East	163·2 (149·2–179·8)	65·1% (60·7 to 69·7%)	35 (31·8–38·6)	-32·6% (-34·5 to -30·6%)	1·00
	Algeria	9·8 (8·7–10·9)	30·1% (22 to 38%)	29·1 (25·8–32·5)	-49·8% (-53·1 to -46·8%)	1·06
	Bahrain	0·2 (0·2–0·3)	145·9% (125·7 to 167·4%)	21·8 (19·3–24·6)	-46·4% (-50·1 to -43%)	1·24
	Egypt	27·1 (24·4–30·2)	43·3% (34·7 to 52·5%)	39·6 (35·9–44·1)	-34·6% (-38·2 to -30·4%)	1·17

Table S11 Cause- and sex-specific burden, mortality, incidence, and prevalence of neurological conditions in North Africa and Middle East countries

		All Ages		Age-standardised		
		Number (thousand)	Percent change	Rate per 100,000	Percent change	Sex ratio
**Measure**		2019	From 1990 to 2019	2019	From 1990 to 2019	2019
Cause	Location	Mean (95% UI)	Mean (95% UI)	Mean (95% UI)	Mean (95% UI)	F:M
	Iran	12·8 (10·8–15·1)	68·6% (58·3 to 79·7%)	17·3 (14·6–20·6)	-28% (-30·2 to -25·5%)	0·86
	Iraq	14·7 (13·5–16)	132·8% (122·2 to 146·8%)	52·7 (48·2–57·9)	-22·7% (-26·6 to -18·3%)	0·83
	Jordan	2·2 (1·9–2·5)	166·1% (147·1 to 186%)	30·2 (26·9–34)	-39·6% (-43·5 to -35%)	0·94
	Kuwait	0·7 (0·6–0·8)	256·5% (228·2 to 284·7%)	21·4 (19·2–24·2)	-0·5% (-7·4 to 7%)	0·64
	Lebanon	1 (0·9–1·1)	24·9% (16·6 to 33·8%)	19 (16·9–21·4)	-43·7% (-47·2 to -40·1%)	1·28
	Libya	1·4 (1·3–1·6)	74·6% (64·1 to 86%)	25·3 (22·6–28·3)	-31·6% (-35·3 to -27·9%)	1·33
	Morocco	10·8 (9·7–12·1)	27·4% (20·2 to 35·1%)	33·7 (30·3–37·7)	-40% (-43·3 to -36·3%)	1·02
	Palestine	0·7 (0·7–0·8)	66·5% (55·8 to 78·5%)	28·3 (25·8–31·3)	-38·8% (-42·9 to -34·8%)	0·95
	Oman	0·7 (0·6–0·8)	50·9% (40·6 to 63·9%)	35·3 (31·4–39·6)	-39·3% (-43·1 to -35·4%)	1·6
	Qatar	0·4 (0·3–0·5)	380·1% (337·6 to 438·3%)	24·5 (21·9–27·6)	-34% (-38·3 to -29·3%)	1·16
	Saudi Arabia	9·7 (8·9–10·6)	204% (184·5 to 224·3%)	42·8 (38·9–47·5)	-6% (-11·1 to -0·8%)	0·99
	Syrian Arab Republic	5·7 (5·2–6·3)	22·3% (15·6 to 30%)	47·4 (43·3–52)	-34% (-37·8 to -30·4%)	0·89
	Tunisia	2·9 (2·5–3·3)	47·1% (37·3 to 56·5%)	23·9 (21·1–27·3)	-37·5% (-41·4 to -33·6%)	0·98
	Türkiye	30 (26·9–33·5)	93·1% (79·1 to 107·3%)	35·1 (31·4–39·1)	-12·4% (-18·3 to -6·4%)	0·92
	United Arab Emirates	2·6 (2·3–2·9)	432·8% (394·6 to 479·2%)	42·3 (38·1–47)	-41·9% (-45·3 to -38·6%)	0·94
	Yemen	7·4 (6·8–8·2)	55·2% (47·8 to 62·4%)	44·7 (40·7–48·8)	-45·8% (-48·6 to -42·9%)	0·96
	Afghanistan	11·7 (10·7–12·8)	63·2% (53·8 to 73·7%)	61 (56–66·3)	-37·6% (-40·2 to -34·7%)	1·11
	Sudan	10·6 (9·6–11·6)	24·2% (17·5 to 30·6%)	45·8 (41·6–50·2)	-44·3% (-47·4 to -41·5%)	1·12
Subarachnoid haemorrhage	Global	1184·6 (1005·9–1390·4)	60·7% (56·5 to 64·7%)	14·5 (12·3–16·9)	-16·9% (-18·8 to -15·3%)	1·21
	North Africa and Middle East	64·1 (54·6–75·6)	82·9% (75·3 to 92·5%)	12·5 (10·7–14·7)	-26·4% (-29·2 to -23·3%)	1·24
	Algeria	4·7 (4–5·6)	76·1% (61 to 91·9%)	12·5 (10·6–14·6)	-33% (-38·1 to -27·6%)	1·2
	Bahrain	0·2 (0·1–0·2)	354·7% (294·1 to 430·2%)	9·6 (7·9–11·9)	-11·1% (-19·6 to 0·8%)	1·32
	Egypt	9·2 (7·9–10·9)	47·8% (37·6 to 59·3%)	12·2 (10·5–14·3)	-31·6% (-36·3 to -26·4%)	1·35
	Iran	7·5 (6·2–9·2)	106% (93·3 to 120%)	9·2 (7·6–11·1)	-20·9% (-23·4 to -17·9%)	1·21
	Iraq	3·2 (2·7–3·8)	90·7% (72·6 to 109·4%)	10·8 (9·1–12·8)	-40·3% (-45·7 to -34·7%)	1·26
	Jordan	1·3 (1–1·6)	350·1% (300·9 to 401·4%)	14·3 (11·7–17·6)	-2·3% (-12·5 to 7·5%)	1·3
	Kuwait	0·6 (0·4–0·7)	287·1% (244·6 to 338·5%)	12·9 (10·5–16·2)	3·1% (-6·4 to 15·2%)	1·35
	Lebanon	0·7 (0·5–0·8)	79·6% (58·5 to 103%)	12·5 (10·2–15·7)	-15·3% (-24·8 to -4·5%)	1·25
	Libya	0·7 (0·6–0·9)	119·8% (99·5 to 144·3%)	11·7 (9·8–14)	-19·8% (-26·1 to -12·7%)	1·32
	Morocco	4·5 (3·8–5·3)	42·4% (32·8 to 53·1%)	13·3 (11·3–15·4)	-32·8% (-37·3 to -28·1%)	1·28
	Palestine	0·4 (0·3–0·5)	176·1% (152·5 to 205·6%)	11·8 (9·8–14·6)	-9·4% (-16·3 to -0·8%)	1·33
	Oman	0·4 (0·3–0·5)	127·6% (104 to 153·5%)	12 (10·1–14·2)	-26·9% (-32·7 to -21·1%)	1·35
	Qatar	0·3 (0·3–0·4)	548·7% (485 to 610·3%)	15·1 (12·9–17·5)	-18·7% (-25·3 to -13%)	1·33
	Saudi Arabia	3·3 (2·7–4·1)	227·3% (196·2 to 266·1%)	10·7 (8·8–13·3)	-5·9% (-13·1 to 4·2%)	1·27
	Syrian Arab Republic	1·6 (1·3–1·9)	55·3% (40·2 to 72·7%)	11·6 (9·8–14)	-22·3% (-28·4 to -15·2%)	1·18
	Tunisia	1·6 (1·3–1·9)	67% (54·7 to 81·4%)	12·3 (10·3–14·7)	-26·8% (-31·7 to -20·9%)	1·29
	Türkiye	13·7 (11·9–15·9)	74·8% (62·2 to 88·6%)	15·1 (13·1–17·4)	-18·8% (-24·8 to -12·2%)	1·14
	United Arab Emirates	1·1 (0·8–1·3)	562·9% (494·7 to 631·6%)	11·7 (9·9–13·7)	-25·2% (-29·9 to -20·7%)	1·35
	Yemen	2·7 (2·3–3·1)	104·9% (90·7 to 122·2%)	15·5 (13·2–18·2)	-26·3% (-31·3 to -20·2%)	1·27
	Afghanistan	2·9 (2·5–3·4)	93·4% (75·2 to 115·5%)	16·3 (13·8–19·3)	-19·2% (-24 to -12·1%)	1·3
	Sudan	3·5 (3–4·1)	54·4% (43·3 to 65·9%)	14·6 (12·5–17)	-28·8% (-33·5 to -23·9%)	1·26
Neurological disorders†*	Global	805178·5 (725838·1–888847·4)	49·1% (46·1 to 52·4%)	10259·5 (9223·2–11324·2)	-0·1% (-0·6 to 0·5%)	1·11
	North Africa and Middle East	61542·7 (54639·6–68418·4)	89·1% (82·4 to 95·8%)	10090·8 (9024·1–11159·3)	0·8% (0 to 1·6%)	1·07
	Algeria	4123·9 (3650·4–4589·5)	72·9% (64·3 to 81·9%)	9906·8 (8817·7–10981·7)	-0·1% (-0·6 to 0·4%)	1·07
	Bahrain	149 (131·2–167·7)	201·1% (179·3 to 223·1%)	9902·6 (8826·1–10988·3)	-0·1% (-0·7 to 0·5%)	1·07

Table S11 Cause- and sex-specific burden, mortality, incidence, and prevalence of neurological conditions in North Africa and Middle East countries

		All Ages		Age-standardised		
		Number (thousand)	Percent change	Rate per 100,000	Percent change	Sex ratio
**Measure**		2019	From 1990 to 2019	2019	From 1990 to 2019	2019
Cause	Location	Mean (95% UI)	Mean (95% UI)	Mean (95% UI)	Mean (95% UI)	F:M
	Egypt	10069·8 (8899·2–11220·2)	88·4% (81·1 to 96·2%)	10257·3 (9189·4–11341·2)	1·1% (-2·1 to 4·8%)	1·08
	Iran	9645·9 (8632·3–10716·6)	62·8% (52·2 to 72·8%)	11293·3 (10132·6–12499·6)	4·5% (1·6 to 6·9%)	1·06
	Iraq	4137·8 (3642·8–4637)	159·3% (151·1 to 167·8%)	9902·6 (8818·8–10985·9)	0% (-0·5 to 0·5%)	1·07
	Jordan	1148·7 (1012·6–1286·1)	230% (218·2 to 242·7%)	9888·5 (8805·2–10979·7)	0% (-0·4 to 0·5%)	1·07
	Kuwait	451·9 (399·8–508·4)	162·4% (148·6 to 176·8%)	9888·6 (8803·3–10919·9)	-0·1% (-2·6 to 2·5%)	1·08
	Lebanon	514·3 (459·3–572·6)	68·3% (61·8 to 74·7%)	9912·9 (8824·5–10995·6)	0·2% (-0·4 to 0·7%)	1·07
	Libya	694·2 (614·9–775)	77·3% (66·9 to 88·3%)	9902·4 (8808·5–10993·8)	0% (-0·4 to 0·5%)	1·07
	Morocco	3608·8 (3200·6–4010·2)	50% (44·6 to 55·5%)	9904·2 (8826·3–10974·4)	0% (-0·5 to 0·5%)	1·07
	Palestine	478·4 (419·8–539·4)	161·1% (155·1 to 167·9%)	9904·4 (8818·6–11008·6)	-0·1% (-0·6 to 0·4%)	1·07
	Oman	462·9 (402·1–528·3)	157·7% (141 to 173·5%)	9864·6 (8795·8–10966·6)	0% (-0·5 to 0·5%)	1·07
	Qatar	297·6 (254·9–343·2)	581·2% (557·6 to 604·4%)	9777·4 (8710·9–10885·3)	-0·5% (-1·1 to 0%)	1·07
	Saudi Arabia	3568 (3139·3–4012·1)	144·8% (128·1 to 162·2%)	9582·1 (8516·5–10684·8)	-0·8% (-3·4 to 2%)	1·06
	Syrian Arab Republic	1467·2 (1302·7–1640·9)	25·4% (18·6 to 32·1%)	9915 (8823·2–10985·6)	0·2% (-0·2 to 0·7%)	1·07
	Tunisia	1171·3 (1049·6–1300)	45·3% (38·4 to 52·8%)	9908·4 (8818·4–10995·9)	0·1% (-0·3 to 0·6%)	1·07
	Türkiye	8130·9 (7269–9079·5)	47·2% (39·6 to 54·4%)	9588·8 (8564·7–10698·3)	0·9% (-1·9 to 3·6%)	1·08
	United Arab Emirates	973·7 (829·2–1124·9)	435·9% (391·1 to 480·3%)	9865·6 (8796·4–10962·3)	0% (-0·7 to 0·6%)	1·07
	Yemen	2994·1 (2628·3–3374·9)	150% (143·6 to 156·7%)	9895·4 (8807·4–10988·4)	0% (-0·4 to 0·4%)	1·07
	Afghanistan	3489·7 (3052·2–3961·4)	238·5% (228·4 to 247·1%)	9901·9 (8816·5–10966·1)	0% (-0·5 to 0·6%)	1·07
	Sudan	3902·3 (3426·3–4410·5)	114% (111·1 to 116·9%)	9879·3 (8797·9–10946·5)	-0·1% (-0·6 to 0·3%)	1·07
Alzheimer's disease and other dementias	Global	7236·4 (6217·2–8232·7)	147·7% (142 to 153·6%)	95 (81·6–107·9)	1·5% (0·2 to 2·8%)	1·24
	North Africa and Middle East	361·2 (309·7–413·1)	177·5% (171·2 to 184·3%)	110·2 (93·9–125·6)	0·8% (-0·7 to 2·6%)	1·09
	Algeria	28·2 (23·7–32·8)	222·4% (198·4 to 248%)	110 (93·8–126)	-0·1% (-3·1 to 3·1%)	1·08
	Bahrain	0·6 (0·5–0·7)	450·2% (419·6 to 484%)	111·9 (96·1–128·1)	1·4% (-1·4 to 4·7%)	1·08
	Egypt	44 (37·5–50·8)	117·7% (109·8 to 124·7%)	107·3 (91·6–122·8)	2·6% (-1·1 to 5·7%)	1·09
	Iran	67·9 (57·9–77·4)	291·5% (266·1 to 320·5%)	111·7 (95–127·3)	0·4% (-1·3 to 1·9%)	1·08
	Iraq	17·5 (14·9–20)	165·3% (156·4 to 175·8%)	108·8 (93·1–124·3)	2·2% (-0·6 to 5·3%)	1·09
	Jordan	4·7 (4–5·5)	425·7% (403·9 to 446·7%)	107·9 (91·9–123·3)	1·8% (-1·3 to 4·8%)	1·08
	Kuwait	2·1 (1·8–2·3)	391·7% (373·8 to 411·3%)	111·2 (95·4–127·1)	0·7% (-2·3 to 4%)	1·1
	Lebanon	5·6 (4·8–6·5)	207·7% (191·4 to 222·2%)	110·6 (95·2–126·5)	1·6% (-1·8 to 4·7%)	1·09
	Libya	4·4 (3·8–5)	168% (159·4 to 176·1%)	107·4 (90·9–122·9)	-1·9% (-5 to 0·9%)	1·08
	Morocco	25·6 (21·8–29·5)	140·2% (131 to 149·4%)	109·1 (92·7–124·8)	-1% (-4·1 to 2·3%)	1·08
	Palestine	1·8 (1·5–2)	136·8% (128·6 to 144·9%)	109·3 (93·5–124·8)	0·6% (-2·4 to 3·8%)	1·07
	Oman	1 (0·9–1·2)	144·6% (135·1 to 154%)	110·1 (93·9–125·2)	-0·6% (-3·5 to 2·4%)	1·1
	Qatar	0·4 (0·4–0·5)	701·7% (645·5 to 764%)	109 (92·9–124·7)	2·5% (-1 to 6·3%)	1·08
	Saudi Arabia	10·2 (8·9–11·7)	136·6% (124·3 to 151%)	108·1 (92–123·6)	-1·3% (-4·3 to 1·9%)	1·1

Table S11 Cause- and sex-specific burden, mortality, incidence, and prevalence of neurological conditions in North Africa and Middle East countries

		**All Ages**		**Age-standardised**		
		Number (thousand)	Percent change	Rate per 100,000	Percent change	Sex ratio
**Measure**		2019	From 1990 to 2019	2019	From 1990 to 2019	2019
Cause	Location	Mean (95% UI)	Mean (95% UI)	Mean (95% UI)	Mean (95% UI)	F:M
	Syrian Arab Republic	9·4 (8–10·9)	119·3% (108·2 to 130·6%)	109·7 (93·7–125·5)	0·8% (-2·6 to 4·4%)	1·1
	Tunisia	12 (10·3–13·8)	203·8% (188·7 to 220·8%)	110·7 (94·6–126·2)	0·2% (-2·7 to 3·1%)	1·09
	Türkiye	91 (78·3–103·6)	187·5% (177·6 to 195·8%)	112·9 (96·7–128·9)	1·5% (-1·8 to 4·5%)	1·1
	United Arab Emirates	1·4 (1·1–1·6)	669·5% (600·8 to 739·7%)	103·1 (86·7–119·4)	-0·9% (-3·8 to 2·1%)	1·09
	Yemen	9·7 (8·3–11·1)	187·9% (177 to 199·2%)	109·3 (93·5–124·4)	-2·7% (-5·7 to 0·3%)	1·07
	Afghanistan	8·5 (7·1–9·8)	55·2% (48·4 to 62·1%)	109·4 (92·9–125·6)	-1·9% (-5·1 to 1·6%)	1·07
	Sudan	14·9 (12·7–17·1)	107·3% (97·2 to 117·9%)	108·2 (92·1–123·5)	-1·3% (-4·4 to 1·8%)	1·08
Parkinson's disease	Global	1081·7 (953·3–1211·2)	159·7% (153 to 167%)	13·4 (11·8–15)	19·6% (17 to 22·3%)	0·56
	North Africa and Middle East	42·8 (38·3–47·3)	192·5% (184·3 to 201·9%)	11·4 (10·3–12·5)	12·7% (9·9 to 15·7%)	0·68
	Algeria	3·2 (2·8–3·7)	200·8% (176·4 to 228·3%)	11 (9·8–12·3)	6·6% (-0·9 to 15·4%)	0·75
	Bahrain	0·1 (0·1–0·1)	466·8% (391·3 to 546%)	13·7 (12–15·5)	12·8% (1·4 to 26%)	0·69
	Egypt	6·3 (5·5–7·2)	142·4% (121·3 to 162·3%)	12 (10·5–13·6)	12·1% (3·7 to 20%)	0·91
	Iran	7·4 (6·3–8·7)	256·7% (226·9 to 285·1%)	11·2 (9·4–13·2)	13·7% (11·1 to 16·4%)	0·68
	Iraq	2 (1·7–2·2)	183·6% (163·9 to 208·4%)	10 (8·8–11·3)	1·6% (-5·3 to 9·9%)	0·62
	Jordan	0·6 (0·5–0·6)	408% (353·5 to 457·6%)	10·3 (9·1–11·6)	-3% (-12·7 to 6%)	0·75
	Kuwait	0·2 (0·2–0·2)	288·1% (244·9 to 338·3%)	8·4 (7·4–9·6)	-11·5% (-23·7 to 2·8%)	0·65
	Lebanon	0·5 (0·5–0·6)	189·9% (158·6 to 222·5%)	10·2 (9·2–11·4)	8·8% (-1 to 19·2%)	0·68
	Libya	0·5 (0·5–0·6)	208·6% (188·1 to 233·7%)	11·3 (9·9–12·5)	11·7% (3·9 to 20·6%)	0·66
	Morocco	2·7 (2·4–3)	176·5% (152·1 to 204·6%)	9·8 (8·8–10·9)	21% (10·5 to 32·2%)	0·59
	Palestine	0·2 (0·2–0·2)	160·2% (139·7 to 183·5%)	11·3 (10·1–12·7)	3·8% (-3·7 to 12·4%)	0·62
	Oman	0·2 (0·2–0·2)	222·3% (196·6 to 253·5%)	17·6 (15·6–20·1)	33·3% (23·2 to 45·2%)	0·54
	Qatar	0·1 (0·1–0·1)	796·5% (707·7 to 879·6%)	18·4 (16·3–20·7)	26·5% (16·2 to 38·3%)	0·87
	Saudi Arabia	2 (1·7–2·2)	224·7% (195·2 to 256·1%)	16 (14·4–18·2)	26·7% (16·2 to 38·2%)	0·51
	Syrian Arab Republic	1·2 (1–1·3)	153·7% (130·8 to 177·6%)	11·6 (10·3–12·9)	9·1% (0·1 to 19·3%)	0·71
	Tunisia	1·2 (1·1–1·4)	195·9% (169·2 to 227·2%)	10·5 (9·4–11·8)	12·5% (3·4 to 24·2%)	0·64
	Türkiye	10·2 (9–11·5)	220·8% (198·1 to 247·2%)	12·3 (10·8–13·8)	19·9% (11·6 to 29·7%)	0·65
	United Arab Emirates	0·5 (0·4–0·6)	854·7% (769·4 to 943·5%)	19 (17·1–21·2)	14·3% (7·1 to 21·3%)	0·75
	Yemen	1 (0·9–1·1)	207·8% (183·7 to 237·2%)	8·8 (7·8–9·8)	11·5% (3·4 to 21·9%)	0·68
	Afghanistan	1·1 (1–1·2)	63·2% (48·2 to 78·2%)	10·5 (9·5–11·6)	-2·8% (-8·8 to 4%)	0·62
	Sudan	1·6 (1·4–1·7)	94·7% (77·4 to 114·4%)	9·4 (8·4–10·6)	-1·2% (-8·9 to 7·9%)	0·62
Idiopathic epilepsy	Global	2898·2 (2098·7–3823·4)	55·9% (32·4 to 83·7%)	38·8 (28–51·3)	16·9% (0·1 to 36·5%)	0·86
	North Africa and Middle East	295·5 (195–407·7)	67·4% (20·3 to 135·5%)	48·2 (32·2–66·4)	8·3% (-21·5 to 51·4%)	0·85
	Algeria	20·2 (5·5–34·2)	44·1% (-64·1 to 502·9%)	48·2 (13·1–81·3)	-0·3% (-74·5 to 315·8%)	0·88
	Bahrain	0·8 (0·2–1·3)	134% (-28 to 809·6%)	61·2 (18·2–101·5)	-5·2% (-70·4 to 270·5%)	0·87
	Egypt	40·8 (10·7–70·8)	59·9% (-54·8 to 647·6%)	39·3 (10·5–68·1)	-0·4% (-72 to 360·2%)	0·79
	Iran	44·7 (30·1–62·1)	26·5% (-6·9 to 71·9%)	55·6 (37·2–77·3)	8·3% (-18·6 to 44%)	0·87
	Iraq	20·2 (4·9–36·1)	116·9% (-40·6 to 800·7%)	45·3 (11–79·1)	1·3% (-72·3 to 319·5%)	0·88
	Jordan	5·7 (1·5–9·7)	183·9% (-25·7 to 975·2%)	46·5 (12·5–79)	4·7% (-72·3 to 291·9%)	0·86
	Kuwait	2·2 (0·6–3·7)	115·4% (-38·4 to 703·8%)	56 (15·2–90·4)	-0·9% (-72 to 282·6%)	0·87
	Lebanon	2·6 (0·7–4·4)	50% (-61·3 to 581·9%)	50·9 (14·3–85·5)	7·2% (-72·2 to 387·8%)	0·85
	Libya	2·9 (0·9–4·8)	19·4% (-63·1 to 350·8%)	45·6 (14·8–75)	-8·1% (-69·9 to 242·5%)	0·84
	Morocco	15·5 (4·1–27·5)	39·5% (-66·3 to 514·5%)	44·2 (11·7–78·4)	13·7% (-72·8 to 390·2%)	0·92
	Palestine	2·5 (0·6–4·4)	126·4% (-42·8 to 926·7%)	45·6 (12–79·7)	5·7% (-72·6 to 387·6%)	0·87
	Oman	2·2 (0·7–3·8)	134·3% (-32·3 to 837·3%)	50·3 (15·1–85·3)	19·4% (-65·9 to 372·9%)	0·87
	Qatar	1·4 (0·4–2·4)	465·9% (62·6 to 2033·6%)	56·4 (16·9–93·7)	2% (-69·9 to 273·5%)	0·95
	Saudi Arabia	23·9 (6·5–39·4)	153·9% (-31·8 to 928·7%)	71·1 (20·2–117·3)	33% (-62·7 to 418·5%)	0·92
	Syrian Arab Republic	5·9 (1·7–10·2)	1·6% (-73·8 to 372·8%)	41·7 (12·2–71·8)	12·6% (-70·5 to 417·6%)	0·88
	Tunisia	4·7 (1·4–7·8)	35·2% (-66·3 to 480·4%)	43·8 (12·5–72·7)	17·2% (-71·6 to 406·5%)	0·91

Table S11 Cause- and sex-specific burden, mortality, incidence, and prevalence of neurological conditions in North Africa and Middle East countries

		All Ages		Age-standardised		
		Number (thousand)	Percent change	Rate per 100,000	Percent change	Sex ratio
**Measure**		2019	From 1990 to 2019	2019	From 1990 to 2019	2019
Cause	Location	Mean (95% UI)	Mean (95% UI)	Mean (95% UI)	Mean (95% UI)	F:M
	Türkiye	47·3 (14–76)	47·5% (-54·5 to 511%)	63·5 (18·8–103·2)	28·1% (-60·8 to 432·1%)	0·81
	United Arab Emirates	5·3 (1·5–9)	307·6% (17·5 to 1375·9%)	65·1 (18·4–106·5)	-6% (-73·4 to 237·2%)	0·82
	Yemen	12·7 (3·1–24)	118·1% (-53·8 to 1156·3%)	35·3 (8·5–66)	4·1% (-78 to 476%)	0·85
	Afghanistan	16·1 (2·9–31·8)	234·2% (-28·5 to 2078·6%)	35·5 (6·5–69·7)	-2·6% (-79·7 to 535%)	0·98
	Sudan	17·4 (4·2–32·4)	104·9% (-50·1 to 982·3%)	38 (9·2–69·8)	10·1% (-72·8 to 488·9%)	0·85
Multiple sclerosis	Global	59·3 (51·8–66·9)	41·8% (37·7 to 45·7%)	0·7 (0·6–0·8)	-7·3% (-8·7 to -5·8%)	1·69
	North Africa and Middle East	9·2 (7·9–10·5)	120% (110 to 129·4%)	1·4 (1·2–1·6)	5·5% (4 to 6·8%)	1·75
	Algeria	0·7 (0·6–0·8)	138·2% (115·5 to 160%)	1·5 (1·3–1·8)	21·3% (15·6 to 28%)	1·82
	Bahrain	<0·1 (<0·1–<0·1)	271% (222·2 to 329%)	1·2 (1–1·5)	27·9% (21 to 34·9%)	1·91
	Egypt	0·8 (0·7–1)	132·1% (123·1 to 142·2%)	0·8 (0·7–1)	17·6% (13·8 to 21·8%)	1·73
	Iran	1·9 (1·6–2·2)	78·9% (60·5 to 97·2%)	2 (1·7–2·2)	-5·2% (-8·4 to -2%)	1·77
	Iraq	0·6 (0·5–0·7)	238·3% (218·6 to 256·6%)	1·3 (1·1–1·6)	13·8% (9·2 to 18·5%)	1·78
	Jordan	0·2 (0·2–0·2)	245·5% (204·9 to 283·8%)	1·6 (1·3–1·9)	-2·4% (-12·7 to 7·8%)	1·94
	Kuwait	0·1 (0·1–0·1)	302·1% (270·4 to 337·5%)	1·8 (1·5–2·1)	49·9% (41·5 to 58·8%)	1·6
	Lebanon	0·1 (0·1–0·1)	135·2% (114·3 to 158%)	1·8 (1·5–2·2)	26·5% (20·2 to 34·6%)	1·8
	Libya	0·1 (0·1–0·1)	169·2% (143·6 to 194·3%)	1·5 (1·3–1·7)	25·9% (19·4 to 30·9%)	1·67
	Morocco	0·6 (0·5–0·7)	87·7% (75·1 to 101·5%)	1·5 (1·2–1·8)	20·3% (14 to 26·9%)	1·8
	Palestine	0·1 (0·1–0·1)	235·8% (219·6 to 254·9%)	1·5 (1·2–1·7)	16·3% (12·3 to 21·4%)	1·63
	Oman	0·1 (0·1–0·1)	340·4% (304·2 to 374·4%)	1·2 (1–1·4)	28% (21·8 to 34·8%)	1·53
	Qatar	0·1 (0·1–0·1)	1046·5% (915·9 to 1227·2%)	1·8 (1·6–2)	44·3% (27·3 to 63·6%)	2·42
	Saudi Arabia	0·5 (0·4–0·6)	273·2% (242·2 to 306%)	1 (0·8–1·2)	22·9% (17·5 to 28·3%)	1·98
	Syrian Arab Republic	0·2 (0·2–0·3)	50·7% (38·9 to 64·2%)	1·5 (1·2–1·8)	20·4% (15·3 to 25·1%)	1·94
	Tunisia	0·2 (0·2–0·3)	85·5% (69·4 to 102·4%)	1·7 (1·4–2·1)	23·2% (17·7 to 28·2%)	1·8
	Türkiye	1·6 (1·5–1·6)	53·6% (48·8 to 58·7%)	1·7 (1·6–1·8)	-2% (-4·3 to 0·4%)	1·67
	United Arab Emirates	0·2 (0·1–0·2)	498·6% (387·4 to 612·9%)	1·1 (0·9–1·2)	5% (-1·1 to 12·2%)	1·48
	Yemen	0·3 (0·2–0·3)	233·9% (217·3 to 251·1%)	0·9 (0·7–1)	17·1% (11·5 to 22·6%)	1·56
	Afghanistan	0·6 (0·5–0·7)	342·8% (318 to 368·5%)	1·6 (1·4–1·9)	12·3% (7·4 to 17·5%)	1·87
	Sudan	0·4 (0·3–0·4)	169·4% (156·9 to 182·3%)	0·9 (0·7–1)	16·9% (12·1 to 22%)	1·58
Migraine	Global	87649 (76635·7–98654·6)	40% (36·8 to 43·5%)	1142·5 (995·9–1289·4)	2·1% (1·1 to 2·8%)	1·69
	North Africa and Middle East	7950·9 (6837·6–9083·6)	70·6% (62·7 to 79·1%)	1238·9 (1063·5–1415·6)	0·4% (-0·7 to 1·6%)	1·69
	Algeria	527 (448–606·8)	50·7% (41·4 to 60·7%)	1234·6 (1047·1–1422·7)	0·1% (0 to 0·1%)	1·66
	Bahrain	16·7 (14·3–19·3)	151·6% (132·1 to 176·2%)	1189·7 (1004·9–1375·8)	0·4% (0 to 0·8%)	1·66
	Egypt	1383·8 (1182·6–1579·4)	85·2% (73·6 to 98·1%)	1285·1 (1107·4–1462·9)	3·3% (-2·1 to 9·3%)	1·72
	Iran	1093·3 (967·5–1223·9)	31·4% (21·9 to 41·7%)	1274·7 (1119·7–1427)	-0·7% (-3·4 to 2·3%)	1·75
	Iraq	569·3 (479·2–662·2)	140% (127·9 to 151·9%)	1229·4 (1042·5–1418·4)	-0·1% (-0·2 to -0·1%)	1·66
	Jordan	155 (130·1–180·1)	199·1% (186·6 to 212·3%)	1213·3 (1027·6–1402·1)	-0·6% (-0·8 to -0·5%)	1·66
	Kuwait	56 (47·9–65·3)	140·8% (123·9 to 159·7%)	1223·6 (1039·8–1415·4)	2·8% (-0·5 to 6·6%)	1·64
	Lebanon	63·4 (54–72·9)	51·9% (44·8 to 59·7%)	1235·1 (1046·9–1423·5)	-0·4% (-0·5 to -0·2%)	1·66
	Libya	87 (74·6–100)	51·3% (37·8 to 65·4%)	1227·8 (1041·4–1416·6)	1·1% (0·8 to 1·4%)	1·66
	Morocco	456·4 (389·5–524·5)	32·8% (26·8 to 39%)	1236·4 (1048·9–1424·6)	-0·4% (-0·4 to -0·3%)	1·66
	Palestine	68·4 (56·9–80·3)	148·6% (140·3 to 156·8%)	1232·3 (1045–1421)	-0·3% (-0·5 to -0·2%)	1·66
	Oman	55·9 (47·2–65·1)	119·2% (97·7 to 141·3%)	1153·5 (973·2–1340·3)	-1% (-1·4 to -0·7%)	1·66
	Qatar	32·3 (27·2–37·9)	494·1% (456 to 527·1%)	1106·2 (930·6–1292·4)	-2·3% (-3 to -1·6%)	1·66
	Saudi Arabia	449·3 (384–519·7)	110·6% (89·9 to 133·2%)	1179·2 (1004·5–1362·2)	-0·6% (-3·9 to 2·4%)	1·56
	Syrian Arab Republic	191·1 (160·8–221·4)	8% (0·1 to 15·9%)	1261·9 (1074·4–1450·5)	2·4% (1·9 to 2·9%)	1·66
	Tunisia	139·8 (120·1–160)	23·2% (16 to 31%)	1239·1 (1051·9–1427·3)	0·2% (0·1 to 0·3%)	1·66
	Türkiye	961·5 (826·6–1099·3)	23·7% (15·5 to 32·7%)	1200·2 (1023·9–1384·3)	0·3% (-3 to 4·4%)	1·8

Table S11 Cause- and sex-specific burden, mortality, incidence, and prevalence of neurological conditions in North Africa and Middle East countries

		All Ages		Age-standardised		
		Number (thousand)	Percent change	Rate per 100,000	Percent change	Sex ratio
**Measure**		2019	From 1990 to 2019	2019	From 1990 to 2019	2019
Cause	Location	Mean (95% UI)	Mean (95% UI)	Mean (95% UI)	Mean (95% UI)	F:M
	United Arab Emirates	103·2 (86–123·5)	338·5% (291·1 to 393·5%)	1148·7 (968·7–1333·5)	0·3% (-0·2 to 1%)	1·66
	Yemen	442 (365·1–521·9)	136·6% (126·1 to 146·9%)	1236·1 (1049·1–1424·3)	-0·1% (-0·3 to 0%)	1·66
	Afghanistan	524·9 (430·1–623·8)	255% (247·6 to 264·2%)	1229 (1042·8–1418)	-2·2% (-2·7 to -1·8%)	1·66
	Sudan	566·6 (470·3–665)	110% (105·7 to 113·9%)	1240 (1052·1–1428·4)	-0·3% (-0·4 to -0·1%)	1·66
Tension-type headache	Global	706190·1 (626723·6–788575·3)	49·6% (46·2 to 53·3%)	8968·2 (7931·9–9990·5)	-0·4% (-1 to 0·2%)	1·05
	North Africa and Middle East	52879·8 (46137–59485·2)	91·9% (84 to 99·8%)	8680·1 (7631·6–9732·5)	0·9% (-0·1 to 1·7%)	1·01
	Algeria	3544·5 (3089·9–4021·8)	76·2% (66·4 to 87%)	8501 (7440·7–9578·6)	-0·1% (-0·1 to -0·1%)	1·01
	Bahrain	130·8 (112·6–149·9)	208·6% (184·1 to 235·6%)	8524·4 (7476–9592·8)	-0·2% (-0·3 to 0%)	1·01
	Egypt	8593·6 (7460·4–9681·3)	88·9% (80·8 to 98%)	8812·2 (7762·7–9893·9)	0·7% (-2·7 to 5%)	1·01
	Iran	8430·1 (7411·2–9501·1)	67·4% (55·2 to 78·9%)	9837·5 (8687·5–11030·7)	5·3% (1·9 to 7·9%)	1·00
	Iraq	3528 (3048–4011·2)	162·9% (153·4 to 172·6%)	8507·1 (7448·5–9584·3)	0% (0 to 0%)	1·01
	Jordan	982·5 (849·6–1113·7)	235·1% (221 to 250·2%)	8508·4 (7455–9588·3)	0·1% (0 to 0·2%)	1·01
	Kuwait	391·3 (340·7–446·6)	165·4% (150 to 182·2%)	8487·2 (7489·2–9540·2)	-0·6% (-3·3 to 2·4%)	1·02
	Lebanon	442 (387·1–499·4)	69·9% (62·6 to 77%)	8503·8 (7444·7–9581·2)	0·2% (0·1 to 0·3%)	1·01
	Libya	599·2 (520·2–678·7)	81·7% (69·1 to 94·3%)	8508·4 (7450·6–9585·8)	-0·1% (-0·2 to 0·1%)	1·01
	Morocco	3107·8 (2711·4–3503)	52·4% (46·3 to 59%)	8502·5 (7441·7–9579·2)	0% (0 to 0%)	1·01
	Palestine	405·5 (349·9–462·3)	163·7% (156·7 to 171·4%)	8503·8 (7444·3–9580·2)	-0·1% (-0·2 to 0%)	1·01
	Oman	403·5 (342·7–467·6)	164·3% (145·6 to 183·1%)	8531·4 (7483·3–9610·9)	0% (-0·2 to 0·1%)	1·01
	Qatar	263·2 (222·4–306·8)	594·1% (567·9 to 620·2%)	8485·2 (7444·1–9569·2)	-0·4% (-0·7 to -0·1%)	1·01
	Saudi Arabia	3081·9 (2653·4–3512·2)	150·6% (131·2 to 170·4%)	8206·1 (7168·6–9215·6)	-1% (-4 to 2%)	1·01
	Syrian Arab Republic	1259·3 (1101·1–1426·1)	28·2% (20·3 to 35·8%)	8488 (7422·9–9561·3)	-0·1% (-0·3 to 0·1%)	1·01
	Tunisia	1013·2 (890·1–1140·5)	48% (40 to 56·9%)	8502 (7441·4–9578·4)	0% (0 to 0·1%)	1·01
	Türkiye	7018·7 (6183·8–7932·9)	50% (41·3 to 59%)	8197·3 (7199·6–9252·4)	0·7% (-2·5 to 3·6%)	1·01
	United Arab Emirates	863·1 (722·1–1015·4)	451·2% (400·5 to 502·6%)	8528·1 (7490·2–9612·7)	-0·1% (-0·3 to 0·1%)	1·01
	Yemen	2528·3 (2163·2–2899)	152·5% (145·1 to 159·9%)	8504·5 (7443·4–9580·9)	0% (0 to 0·1%)	1·01
	Afghanistan	2938·3 (2512·7–3388·7)	237% (225·6 to 247%)	8515·2 (7457·9–9593·9)	0·4% (0·2 to 0·6%)	1·01
	Sudan	3301·3 (2844–3772·5)	114·8% (111·7 to 117·8%)	8482·4 (7418·8–9558·5)	-0·1% (-0·2 to -0·1%)	1·01
Motor neuron disease	Global	63·7 (57·3–71·3)	79% (74·1 to 84%)	0·8 (0·7–0·9)	0·3% (-0·4 to 0·9%)	0·77
	North Africa and Middle East	3·4 (2·9–4·1)	83·1% (70·8 to 94·9%)	0·6 (0·5–0·7)	1·9% (0·3 to 3·6%)	0·82
	Algeria	0·2 (0·2–0·3)	85·6% (68·4 to 104·6%)	0·5 (0·4–0·6)	0·8% (-2·8 to 4·5%)	0·89
	Bahrain	<0·1 (<0·1–<0·1)	254·2% (199·1 to 312·3%)	0·5 (0·4–0·6)	4·4% (0·6 to 8·3%)	0·89
	Egypt	0·5 (0·4–0·6)	72·4% (60·2 to 83·7%)	0·5 (0·5–0·7)	-1·8% (-5·2 to 2%)	0·88
	Iran	0·5 (0·4–0·6)	69·8% (50·1 to 87·8%)	0·6 (0·5–0·7)	4·1% (2·2 to 6·2%)	0·86
	Iraq	0·2 (0·2–0·3)	132·4% (110·9 to 154%)	0·6 (0·5–0·7)	4·1% (0·7 to 7·7%)	0·87
	Jordan	0·1 (0–0·1)	221% (185 to 257·9%)	0·5 (0·5–0·6)	2·4% (-1·9 to 6·6%)	0·89
	Kuwait	<0·1 (<0·1–<0·1)	142·6% (113·4 to 171·4%)	0·5 (0·4–0·6)	-12% (-16·5 to -7·4%)	0·83
	Lebanon	<0·1 (<0·1–<0·1)	63·7% (51·1 to 75·5%)	0·5 (0·4–0·6)	-0·6% (-3·8 to 2·8%)	0·87
	Libya	<0·1 (<0·1–<0·1)	65·1% (41·3 to 88%)	0·5 (0·4–0·6)	1·4% (-2·7 to 5·5%)	0·93
	Morocco	0·2 (0·2–0·2)	49·4% (34·4 to 64·1%)	0·6 (0·5–0·7)	-2·1% (-5·8 to 1·6%)	0·88
	Palestine	<0·1 (<0·1–<0·1)	119·4% (98·4 to 141·2%)	0·6 (0·5–0·7)	0·5% (-2·8 to 4·1%)	0·89
	Oman	<0·1 (<0·1–<0·1)	128% (101·1 to 156·7%)	0·5 (0·4–0·6)	0·6% (-3 to 4·1%)	0·86
	Qatar	<0·1 (<0·1–<0·1)	541% (467·6 to 611·6%)	0·5 (0·4–0·6)	1·3% (-2·6 to 5·5%)	0·89
	Saudi Arabia	0·1 (0·1–0·2)	127·1% (94·5 to 161·5%)	0·5 (0·4–0·6)	3·1% (-1·3 to 7·2%)	0·82

Table S11 Cause- and sex-specific burden, mortality, incidence, and prevalence of neurological conditions in North Africa and Middle East countries

		All Ages		Age-standardised		
		Number (thousand)	Percent change	Rate per 100,000	Percent change	Sex ratio
**Measure**		2019	From 1990 to 2019	2019	From 1990 to 2019	2019
Cause	Location	Mean (95% UI)	Mean (95% UI)	Mean (95% UI)	Mean (95% UI)	F:M
	Syrian Arab Republic	0·1 (0·1–0·1)	18·3% (1 to 35·5%)	0·6 (0·5–0·7)	0% (-3·6 to 4·2%)	0·9
	Tunisia	0·1 (0·1–0·1)	53·6% (37·7 to 69%)	0·5 (0·5–0·7)	-1·8% (-5·2 to 1·9%)	0·87
	Türkiye	0·7 (0·6–0·8)	63·7% (53·4 to 74·5%)	0·9 (0·8–1)	7·9% (3·8 to 12%)	0·68
	United Arab Emirates	<0·1 (<0·1–0·1)	516·2% (409·8 to 613·1%)	0·5 (0·4–0·6)	8·5% (4·1 to 12·8%)	0·86
	Yemen	0·1 (0·1–0·2)	111·3% (93 to 130·7%)	0·5 (0·4–0·7)	1% (-2·7 to 5·3%)	0·88
	Afghanistan	0·2 (0·2–0·3)	203·3% (184·4 to 223·2%)	0·7 (0·6–0·8)	2·1% (-1 to 5·8%)	0·9
	Sudan	0·2 (0·1–0·2)	80·9% (68·1 to 93·4%)	0·5 (0·4–0·6)	-1·7% (-5·2 to 2·6%)	0·88
Headache disorders	Global	793839·1 (714299·8–877018·8)	48·5% (45·4 to 51·7%)	10110·7 (9070·7–11167·1)	-0·2% (-0·7 to 0·4%)	1·11
	North Africa and Middle East	60830·7 (53891·3–67676·1)	88·8% (82 to 95·6%)	9919 (8853·1–10989·5)	0·8% (-0·1 to 1·5%)	1·07
	Algeria	4071·5 (3596·7–4536)	72·4% (63·8 to 81·7%)	9735·6 (8650–10821·3)	-0·1% (-0·1 to 0%)	1·07
	Bahrain	147·5 (129·6–166·4)	200·9% (179·2 to 223·7%)	9714·1 (8647·6–10789·3)	-0·1% (-0·2 to 0%)	1·07
	Egypt	9977·4 (8813·7–11125·9)	88·4% (81·1 to 96·3%)	10097·3 (9024·8–11179·1)	1% (-2·1 to 4·7%)	1·08
	Iran	9523·5 (8508–10591·8)	62·3% (51·6 to 72·1%)	11112·1 (9952·4–12306·5)	4·6% (1·6 to 6·9%)	1·06
	Iraq	4097·3 (3604·7–4592·2)	159·5% (151·2 to 168·2%)	9736·6 (8654·2–10821·6)	0% (0 to 0%)	1·07
	Jordan	1137·5 (1001·8–1274)	229·7% (217·7 to 242·6%)	9721·7 (8637·2–10804·7)	0% (-0·1 to 0·1%)	1·07
	Kuwait	447·3 (394–503·2)	162% (148·3 to 176%)	9710·8 (8639·4–10763·2)	-0·1% (-2·6 to 2·5%)	1·08
	Lebanon	505·4 (450·6–563·9)	67·4% (61·1 to 73·5%)	9738·8 (8650·3–10829·1)	0·1% (0 to 0·2%)	1·07
	Libya	686·2 (606·5–767·2)	77·2% (66·6 to 88·4%)	9736·2 (8655·1–10820·8)	0·1% (-0·1 to 0·2%)	1·07
	Morocco	3564·2 (3157·9–3966·9)	49·6% (44·3 to 55·2%)	9739 (8652·6–10825·3)	-0·1% (-0·1 to 0%)	1·07
	Palestine	473·9 (414·8–534·7)	161·4% (155·4 to 168%)	9736·1 (8651·9–10822)	-0·1% (-0·2 to 0%)	1·07
	Oman	459·4 (397·9–524·3)	157·8% (141·4 to 173·4%)	9684·9 (8625·4–10764)	-0·2% (-0·3 to 0%)	1·07
	Qatar	295·5 (252·8–340·8)	581·5% (557·2 to 604·5%)	9591·5 (8532·6–10666·3)	-0·6% (-0·9 to -0·3%)	1·07
	Saudi Arabia	3531·2 (3088·6–3972·7)	144·7% (127·8 to 162·2%)	9385·4 (8318–10477·2)	-1% (-3·5 to 1·8%)	1·06
	Syrian Arab Republic	1450·4 (1285·1–1623·1)	25·1% (18·2 to 31·8%)	9749·8 (8662–10834·3)	0·2% (0 to 0·4%)	1·07
	Tunisia	1153 (1029·2–1281·5)	44·5% (37·3 to 51·9%)	9741 (8652·7–10828·7)	0·1% (0 to 0·1%)	1·07
	Türkiye	7980·1 (7127–8917·3)	46·3% (39 to 53·5%)	9397·5 (8376·7–10486·8)	0·7% (-2·1 to 3·2%)	1·09
	United Arab Emirates	966·3 (822·4–1117·1)	436·5% (392·1 to 481·4%)	9676·8 (8629·4–10745·8)	0% (-0·2 to 0·2%)	1·07
	Yemen	2970·2 (2605–3349·3)	150% (143·5 to 156·6%)	9740·6 (8655·2–10825·6)	0% (-0·1 to 0·1%)	1·07
	Afghanistan	3463·2 (3022·7–3937·2)	239·6% (230 to 248·4%)	9744·2 (8662·8–10828·1)	0·1% (-0·1 to 0·3%)	1·07
	Sudan	3867·9 (3387·7–4370·9)	114·1% (111·4 to 116·6%)	9722·3 (8634·6–10808)	-0·2% (-0·2 to -0·1%)	1·07
**Prevalence**						
Meningitis	Global	7683·5 (6590·3–9132·2)	-23·7% (-26 to -21·1%)	99·9 (85·5–118·8)	-47·2% (-48·7 to -45·5%)	0·98
	North Africa and Middle East	356·2 (306·7–419·9)	5·7% (-1·6 to 14%)	60·5 (52·2–71·1)	-44·7% (-48·4 to -40·7%)	1·12
	Algeria	21·6 (17·7–26·8)	21·7% (-1·1 to 48·1%)	53·2 (43·5–65·7)	-32·3% (-45·1 to -17·5%)	1·00
	Bahrain	0·6 (0·5–0·7)	76·6% (45 to 110·6%)	40·1 (32·7–48·6)	-46·2% (-56·2 to -35·5%)	0·83

Table S11 Cause- and sex-specific burden, mortality, incidence, and prevalence of neurological conditions in North Africa and Middle East countries

		All Ages		Age-standardised		
		Number (thousand)	Percent change	Rate per 100,000	Percent change	Sex ratio
**Measure**		2019	From 1990 to 2019	2019	From 1990 to 2019	2019
Cause	Location	Mean (95% UI)	Mean (95% UI)	Mean (95% UI)	Mean (95% UI)	F:M
	Egypt	60·6 (49·3–75·6)	5·3% (-15·3 to 29·8%)	63·8 (51·9–79·7)	-42·7% (-54·3 to -29·3%)	0·98
	Iran	58·7 (50·1–69·3)	3% (-5·1 to 12·2%)	69·4 (59·2–82)	-35·7% (-40·3 to -30·8%)	2·02
	Iraq	23·9 (19·7–29·1)	46·7% (18·5 to 80·2%)	59·8 (49·5–72·5)	-42% (-53·5 to -28·6%)	0·98
	Jordan	9·5 (7·8–11·6)	174·9% (126·3 to 229·7%)	86·2 (71·1–105·2)	-17·7% (-32·3 to -1·2%)	1·01
	Kuwait	1·7 (1·4–2)	102·1% (65 to 145·4%)	37·6 (31–46·1)	-25·8% (-39·2 to -9·4%)	1·08
	Lebanon	2·9 (2·4–3·6)	2·5% (-15·3 to 24·6%)	55·7 (45·8–68·5)	-39·8% (-50·5 to -26·9%)	1·02
	Libya	3·4 (2·8–4·3)	52·6% (23·9 to 86·7%)	50·3 (41·7–63·7)	-14·6% (-31 to 3·9%)	1·05
	Morocco	24·5 (19·8–30·6)	-27·1% (-41·4 to -10·6%)	69·2 (55·9–86·4)	-51·8% (-61·5 to -40·5%)	0·9
	Palestine	3·2 (2·7–4)	32·6% (8·9 to 60·5%)	71·5 (58·7–88·5)	-47·2% (-56·9 to -35·8%)	1·05
	Oman	0·7 (0·6–0·9)	55·8% (26·2 to 90·2%)	17·1 (13·6–21·7)	-38·1% (-50·1 to -23·9%)	0·78
	Qatar	2·6 (2·1–3·1)	421·2% (327·5 to 527·1%)	89 (73·1–108·5)	-26·2% (-39·7 to -10·5%)	0·96
	Saudi Arabia	10·8 (8·6–13·3)	81·2% (46·1 to 117·4%)	31·2 (25–38·3)	-29·1% (-43·3 to -14·4%)	1·03
	Syrian Arab Republic	11·7 (9·7–14·4)	-31·5% (-44·6 to -16·5%)	82·1 (68·7–101)	-45% (-55·3 to -32·4%)	1·00
	Tunisia	7·5 (6·2–9·1)	5·1% (-14·9 to 28·5%)	63·4 (52–76·9)	-31·3% (-44·6 to -16·1%)	1·14
	Türkiye	12·6 (10·4–15·5)	-53·5% (-63 to -42·9%)	14·9 (12·3–18·4)	-69% (-75·3 to -62%)	0·76
	United Arab Emirates	3·2 (2·6–4)	333·9% (251·6 to 421·7%)	38·3 (31·2–47·4)	-21% (-36·2 to -5%)	1·08
	Yemen	23·5 (18·7–29·6)	71·4% (33·2 to 115·3%)	84·5 (67·6–106·3)	-29·7% (-45·4 to -11·7%)	0·94
	Afghanistan	56·5 (46·4–68·9)	71·4% (34·9 to 114·7%)	217·1 (178·4–261·1)	-41% (-53·6 to -26·1%)	1·11
	Sudan	16·3 (13·1–20·4)	-55·8% (-64·7 to -43·9%)	44·1 (35·4–54·9)	-79% (-83·3 to -73·3%)	0·92
Encephalitis	Global	4499·4 (3372·1–5573·2)	4·7% (1·6 to 8·9%)	56·8 (42·6–70·3)	-31·9% (-33·9 to -28·5%)	0·99
	North Africa and Middle East	167·5 (127·8–205·9)	79·3% (73·4 to 86·9%)	28 (21·2–34·6)	-10·4% (-13·5 to -5·9%)	1·3
	Algeria	9·6 (7·4–11·9)	87·5% (76·2 to 99·4%)	23·2 (17·7–28·8)	-0·2% (-6·2 to 6·6%)	1·2
	Bahrain	0·3 (0·2–0·3)	212·3% (193·4 to 232·4%)	17·1 (13·3–21·4)	-4·4% (-10·7 to 2%)	1·22
	Egypt	27·2 (20·8–33·7)	52·4% (42·3 to 64·1%)	28·5 (21·5–35·6)	-20·2% (-25·6 to -14%)	1·19
	Iran	20·9 (16·2–25·6)	52·4% (47·1 to 59·2%)	24·2 (18·8–29·7)	-10·8% (-13·8 to -6·1%)	1·24
	Iraq	11·2 (8·6–13·8)	131·8% (119·3 to 146·5%)	28·4 (21·2–35·2)	-13·3% (-18·3 to -7·4%)	1·29
	Jordan	2·9 (2·2–3·5)	194·6% (179 to 209·4%)	25·8 (19·8–32·2)	-14·3% (-19·2 to -9·7%)	1·23
	Kuwait	0·7 (0·6–0·9)	173·5% (154 to 193·4%)	16·1 (12·5–20)	-3·3% (-10·5 to 4·2%)	1·26
	Lebanon	1·7 (1·3–2·1)	36·5% (29·2 to 44%)	32·1 (24·7–39·8)	-23·2% (-27·5 to -18·4%)	1·5
	Libya	1·3 (1–1·6)	151·6% (137·9 to 165·6%)	18·8 (14·2–23·5)	33·2% (26·1 to 40·4%)	1·2
	Morocco	9·8 (7·4–12·3)	43·3% (35·1 to 52·7%)	27 (20·3–33·7)	-10·3% (-15·8 to -4%)	1·29
	Palestine	1·5 (1·2–1·9)	153·8% (139·4 to 169%)	33·9 (25·9–42·1)	-4·8% (-10·5 to 1%)	1·2
	Oman	1·2 (1–1·5)	115·7% (99·9 to 131·1%)	28·9 (22·3–35·6)	-18·2% (-24·5 to -11·5%)	1·00
	Qatar	0·4 (0·3–0·5)	521·9% (468·5 to 576%)	13 (10–16·2)	-10·8% (-18·7 to -2·6%)	1·24
	Saudi Arabia	6·2 (4·8–7·8)	146·9% (128·5 to 165%)	17·2 (13·2–21·7)	-7·3% (-14·1 to -0·2%)	1·26
	Syrian Arab Republic	4·6 (3·5–5·7)	17·4% (9·8 to 25·1%)	31·5 (23·9–39·6)	-12·7% (-18·7 to -6·8%)	1·23
	Tunisia	3·1 (2·4–3·8)	41·2% (33·7 to 49·1%)	25·6 (19·5–31·7)	-11·4% (-16·1 to -6·4%)	1·27
	Türkiye	23·7 (18·2–29·3)	39·7% (31·6 to 48%)	27·2 (21–33·5)	-12·3% (-17·9 to -6·4%)	1·31
	United Arab Emirates	1·5 (1·2–1·9)	596·8% (542·3 to 656·7%)	15·3 (11·6–19·2)	23·9% (13·7 to 35·1%)	1·22
	Yemen	9 (6·7–11·5)	175·4% (154·6 to 195·5%)	32·5 (23·6–41·7)	9·1% (0·5 to 17·5%)	1·23
	Afghanistan	19·8 (14·6–25·3)	232·3% (204·9 to 263·8%)	69·4 (48·6–89·3)	4·4% (-4·1 to 13·2%)	1·58
	Sudan	10·5 (8–13·3)	88·7% (75 to 106%)	28·9 (21·5–36·5)	-12·4% (-18·8 to -4·3%)	1·25
Tetanus	Global	61·7 (44·6–81·2)	-64·4% (-68·1 to -61·2%)	0·8 (0·6–1·1)	-73·7% (-76·2 to -71·5%)	1·01
	North Africa and Middle East	0·7 (0·5–1·1)	-35·7% (-53·8 to -16·2%)	0·1 (0·1–0·2)	-61·7% (-70·5 to -51·9%)	0·63
	Algeria	<0·1 (<0·1–<0·1)	-10·2% (-45·5 to 17·6%)	<0·1 (<0·1–<0·1)	-45·1% (-65·9 to -28·6%)	0·68
	Bahrain	<0·1 (<0·1–<0·1)	168·5% (143·6 to 196·5%)	0·1 (0–0·1)	-5·9% (-15·2 to 3·8%)	0·76
	Egypt	0·1 (0·1–0·2)	-36·6% (-54·2 to -18%)	0·1 (0·1–0·2)	-63·5% (-73·5 to -53·2%)	0·34
	Iran	<0·1 (<0·1–<0·1)	-58·9% (-72·9 to -44·9%)	<0·1 (<0·1–<0·1)	-69·4% (-78·2 to -61·1%)	0·59

Table S11 Cause- and sex-specific burden, mortality, incidence, and prevalence of neurological conditions in North Africa and Middle East countries

		All Ages		Age-standardised		
		Number (thousand)	Percent change	Rate per 100,000	Percent change	Sex ratio
**Measure**		2019	From 1990 to 2019	2019	From 1990 to 2019	2019
Cause	Location	Mean (95% UI)	Mean (95% UI)	Mean (95% UI)	Mean (95% UI)	F:M
	Iraq	0·1 (<0·1–0·1)	-23·6% (-51·5 to 6·3%)	0·1 (0·1–0·2)	-63·5% (-73·9 to -53·4%)	0·45
	Jordan	<0·1 (<0·1–<0·1)	71·8% (23·6 to 119%)	<0·1 (<0·1–0·1)	-40·8% (-55·8 to -26·1%)	0·58
	Kuwait	<0·1 (<0·1–<0·1)	136% (117 to 157·4%)	0·1 (0–0·1)	-6·2% (-13·7 to 2·4%)	0·92
	Lebanon	<0·1 (<0·1–<0·1)	-39·7% (-80·8 to 55%)	0·1 (0–0·2)	-72·6% (-90·5 to -27·6%)	0·76
	Libya	<0·1 (<0·1–<0·1)	15·8% (-16·4 to 39·3%)	<0·1 (<0·1–<0·1)	-26·3% (-48·5 to -11·7%)	0·78
	Morocco	0·1 (0·1–0·2)	-48·9% (-67·7 to -28·2%)	0·4 (0·2–0·5)	-57·8% (-71·1 to -43·8%)	0·76
	Palestine	<0·1 (<0·1–<0·1)	-7% (-58·9 to 44·8%)	<0·1 (<0·1–0·1)	-52·6% (-74·1 to -36·1%)	0·61
	Oman	<0·1 (<0·1–<0·1)	44·3% (-13·4 to 94·5%)	<0·1 (<0·1–<0·1)	-50·9% (-79·9 to -20·2%)	0·8
	Qatar	<0·1 (<0·1–<0·1)	454·9% (372·5 to 504·4%)	<0·1 (<0·1–0·1)	-11·4% (-30·2 to -2·7%)	2·29
	Saudi Arabia	<0·1 (<0·1–<0·1)	-39·1% (-76·9 to 7%)	<0·1 (<0·1–0·1)	-71·4% (-87·9 to -51%)	0·84
	Syrian Arab Republic	<0·1 (<0·1–<0·1)	-73·6% (-84·1 to -61·8%)	0·1 (0–0·1)	-72·3% (-81·1 to -63·1%)	0·77
	Tunisia	<0·1 (<0·1–<0·1)	-13·1% (-43·5 to 11·7%)	<0·1 (<0·1–<0·1)	-35·6% (-57·7 to -18·4%)	0·75
	Türkiye	<0·1 (<0·1–0·1)	-58·7% (-76·1 to -34·5%)	0·1 (0–0·1)	-74·2% (-86·2 to -54·7%)	0·83
	United Arab Emirates	<0·1 (<0·1–<0·1)	59% (-27 to 218·5%)	0·2 (0·1–0·3)	-82·6% (-92·8 to -36·4%)	1·64
	Yemen	<0·1 (<0·1–0·1)	-21·7% (-65·9 to 29·9%)	0·1 (0·1–0·2)	-58·7% (-79·3 to -40·8%)	0·34
	Afghanistan	0·2 (0·2–0·3)	-14% (-47·3 to 48·1%)	0·6 (0·4–0·8)	-67·3% (-77·7 to -51·9%)	0·86
	Sudan	<0·1 (<0·1–0·1)	-41·6% (-69·4 to -8·5%)	0·1 (0·1–0·2)	-65·6% (-79·8 to -52·8%)	0·37
Brain and central nervous system cancer	Global	1065·3 (800·4–1199·9)	151·5% (50·8 to 195·7%)	13·5 (10·1–15·2)	59·7% (-2·4 to 86·2%)	1·01
	North Africa and Middle East	97·2 (64·2–115·6)	280·5% (113·9 to 424·7%)	16·5 (10·8–19·5)	119·1% (30·2 to 189·1%)	0·81
	Algeria	3·5 (2·1–4·6)	240·6% (80·2 to 401·8%)	8·3 (4·9–11·1)	118·4% (15·6 to 213·7%)	0·93
	Bahrain	0·2 (0·1–0·3)	723·9% (389·9 to 1098·2%)	14·8 (9·4–19·3)	147·5% (49·6 to 250·5%)	0·73
	Egypt	8·4 (5·6–11·7)	226·4% (63·4 to 401·9%)	8·7 (5·9–12)	81·5% (1·2 to 172%)	0·79
	Iran	23·2 (12·1–29·5)	221·6% (78·6 to 352·2%)	27·7 (14·4–35·1)	129·4% (36·1 to 203·2%)	0·85
	Iraq	9·5 (6·8–12·6)	422·3% (154·6 to 768·9%)	23·5 (17–31·2)	123·7% (14·7 to 254·7%)	1·00
	Jordan	1·8 (1·3–2·3)	709·6% (408·1 to 1031·3%)	15·8 (11·4–20)	146·1% (57·1 to 240·4%)	0·84
	Kuwait	0·8 (0·6–1·1)	417% (212·8 to 610·5%)	20·8 (14·7–26·2)	120·7% (35·2 to 197·4%)	0·56
	Lebanon	1·8 (1·2–2·3)	535·7% (309·1 to 812·8%)	34 (23·6–44·8)	284·7% (150·6 to 439·7%)	0·7
	Libya	1 (0·7–1·4)	173·7% (77·5 to 332·5%)	15·3 (11–20·2)	69·6% (17·6 to 153·3%)	0·58
	Morocco	1·6 (1·1–2·3)	158·2% (39 to 300·6%)	4·6 (3–6·5)	79·1% (0·8 to 165·4%)	1·37
	Palestine	1·1 (0·9–1·5)	280·7% (122 to 456·2%)	25 (18·9–31·3)	60·2% (2·7 to 120·3%)	0·76
	Oman	0·7 (0·4–0·9)	754·3% (329·7 to 1289·4%)	15·7 (9·1–20·4)	255·2% (76·4 to 453·4%)	0·85
	Qatar	0·6 (0·4–1)	2220% (1205·8 to 3531·3%)	24·3 (17–36·4)	230·5% (93·2 to 395%)	0·94
	Saudi Arabia	6·8 (4·8–10·2)	1355·6% (660·2 to 2697·9%)	17·9 (13·1–26·5)	444·1% (181·8 to 910·9%)	1·06
	Syrian Arab Republic	2·5 (1·8–3·3)	166·4% (57·3 to 321·2%)	17·5 (12·8–23·6)	109·7% (38·8 to 216·7%)	0·78
	Tunisia	0·9 (0·6–1·3)	239·2% (90·3 to 430·9%)	8·1 (5·2–11·4)	157·8% (49 to 292·8%)	1·1
	Türkiye	23·1 (11·3–32·3)	279·2% (97·5 to 516%)	29 (14·5–40)	173·1% (53 to 322·7%)	0·72
	United Arab Emirates	1·5 (0·9–2·3)	820·1% (481·5 to 1255·4%)	16·2 (9·5–22·6)	60·9% (-6·8 to 134·6%)	0·78
	Yemen	1·9 (1·1–2·8)	210% (60 to 521·8%)	6·8 (4·1–9·9)	47·8% (-18·6 to 161·2%)	0·73
	Afghanistan	2·5 (1·3–4·4)	183% (72 to 445·1%)	7·6 (4·3–13·6)	2·9% (-33 to 74·3%)	0·79
	Sudan	3·7 (2·1–5·3)	166·2% (16·8 to 550·4%)	9·1 (5·4–13·2)	47% (-25·8 to 197·7%)	0·67
Stroke*	Global	101474·6 (93211·9–110526·3)	85·3% (82·6 to 88·2%)	1240·3 (1139·7–1353)	-6·1% (-7·2 to -5%)	1·14
	North Africa and Middle East	7323·4 (6794·7–7863·1)	142·1% (137·8 to 146·3%)	1537·5 (1421·9–1659·9)	-0·5% (-2·3 to 1·1%)	1·24
	Algeria	549·7 (506·6–593)	129% (118·8 to 139·1%)	1540·3 (1417·2–1668)	-10·7% (-14·5 to -7%)	1·26
	Bahrain	14·5 (13·4–15·8)	292·5% (272·2 to 313·4%)	1136·4 (1049–1234·8)	-24·2% (-27·5 to -20·8%)	1·35
	Egypt	1269·8 (1170·8–1374·6)	151·8% (140·2 to 163·9%)	1806·1 (1658·1–1974·5)	18·5% (12·9 to 23·9%)	1·35
	Iran	963·5 (859·2–1079·7)	123·4% (115·9 to 131·2%)	1253·8 (1113·5–1418·4)	-13·3% (-15·9 to -10·7%)	1·16
	Iraq	520 (483·1–557·3)	169·6% (159 to 180·7%)	1968·8 (1823·4–2122·1)	-9·6% (-13·3 to -5·4%)	1·18

Table S11 Cause- and sex-specific burden, mortality, incidence, and prevalence of neurological conditions in North Africa and Middle East countries

Measure		All Ages		Age-standardised		
		Number (thousand)	Percent change	Rate per 100,000	Percent change	Sex ratio
		2019	From 1990 to 2019	2019	From 1990 to 2019	2019
Cause	Location	Mean (95% UI)	Mean (95% UI)	Mean (95% UI)	Mean (95% UI)	F:M
	Jordan	134·6 (123·3–145·5)	258·4% (239·7 to 275%)	1793·9 (1621·2–1952·9)	-23·4% (-27·7 to -19·5%)	1·25
	Kuwait	42·7 (39·3–46·4)	267·6% (248·5 to 287·1%)	1230·6 (1134·6–1332·5)	-5·9% (-10·4 to -1·4%)	1·05
	Lebanon	74·8 (69·4–80·8)	124·2% (114·6 to 134·6%)	1425·1 (1320·3–1538·1)	2·2% (-1·8 to 6·6%)	1·39
	Libya	90·4 (83·4–96·9)	201·2% (186·1 to 214·9%)	1588·4 (1461·7–1712·9)	14% (8·9 to 18·7%)	1·59
	Morocco	557·1 (515·5–599·7)	120·3% (110·8 to 131%)	1695·1 (1563·4–1833·2)	3·4% (-0·7 to 8·2%)	1·33
	Palestine	41·3 (37·8–45·1)	167·1% (153·9 to 180·9%)	1510·8 (1381·5–1666·5)	-3·7% (-8·5 to 1·4%)	1·33
	Oman	37·7 (34·6–41·2)	167·7% (154·3 to 182%)	1525·6 (1399·9–1686·6)	-8·5% (-12·4 to -4·5%)	1·1
	Qatar	22·3 (20·3–24·5)	568·9% (532·5 to 607·4%)	1226·3 (1127·4–1327·2)	-22·2% (-25·6 to -18·9%)	1·31
	Saudi Arabia	480·5 (442·2–523·2)	238·8% (214·5 to 262·7%)	1967·7 (1818·1–2143·5)	7·9% (-0·1 to 15·2%)	1·33
	Syrian Arab Republic	199·6 (184·4–215·1)	78·9% (71·6 to 86·2%)	1518·7 (1403·8–1637·5)	-13·9% (-17·3 to -10·5%)	1·14
	Tunisia	159·9 (147·4–173·4)	184·9% (171 to 200·3%)	1260·8 (1162·6–1366)	22·1% (16·3 to 28·2%)	1·19
	Türkiye	1080·4 (1002·6–1164·9)	103·3% (94·4 to 112·7%)	1213·6 (1124·7–1309·4)	-8·7% (-12·4 to -4·6%)	1·21
	United Arab Emirates	136·8 (125·5–148·3)	721·6% (675·3 to 767·1%)	2225·7 (2037·3–2436·3)	-7·5% (-11·6 to -3·6%)	1·22
	Yemen	260·9 (242–280·8)	174·8% (161·2 to 188·1%)	1609·4 (1480·2–1745·5)	0·8% (-4·2 to 5·7%)	1·25
	Afghanistan	281·2 (260·9–302·7)	130·1% (117·5 to 141·2%)	1657·6 (1533·4–1779·5)	3·8% (-1·2 to 8%)	1·31
	Sudan	398 (367·1–427·9)	124·6% (114·5 to 134·3%)	1785·8 (1645·2–1936·9)	8·2% (3·7 to 12·8%)	1·33
Ischaemic stroke	Global	77192·5 (68857·2–86457·6)	95·3% (91·7 to 99·3%)	951 (849·8–1064·1)	-1·9% (-3·4 to -0·4%)	1·19
	North Africa and Middle East	5998·8 (5474–6566·7)	157·6% (152·8 to 162·7%)	1303·6 (1183·2–1435·4)	5·9% (3·6 to 8%)	1·25
	Algeria	470 (427·5–513·8)	150·1% (138·7 to 161·4%)	1347·1 (1227–1477·6)	-2·6% (-6·7 to 1·7%)	1·26
	Bahrain	11·2 (10·1–12·4)	297·3% (276·9 to 317·4%)	949·2 (859·7–1048·7)	-23·8% (-27·5 to -20·2%)	1·34
	Egypt	1089·8 (991–1195)	181·7% (168·2 to 194·4%)	1597 (1436·4–1765·4)	31·7% (24·9 to 38·1%)	1·37
	Iran	849 (736·7–971·9)	123·9% (115·1 to 132·9%)	1127·4 (977–1300·9)	-13·1% (-16·1 to -10·1%)	1·16
	Iraq	414·4 (378·2–449·6)	169·7% (158·8 to 182·2%)	1656·7 (1500·2–1820)	-7·6% (-11·8 to -2·6%)	1·27
	Jordan	113·8 (102·4–124·4)	256·8% (238·1 to 273·9%)	1591·7 (1411·6–1753·9)	-23·9% (-28·6 to -19·4%)	1·27
	Kuwait	33 (29·8–36·3)	264·5% (248·5 to 281%)	1032·1 (934·4–1132·1)	-6·5% (-11 to -2·1%)	1·02
	Lebanon	65·7 (59·9–71·6)	141·5% (131·1 to 153%)	1254 (1145–1367·4)	8·4% (4 to 13·1%)	1·38
	Libya	76·4 (69·4–83)	223·3% (207·5 to 239%)	1395·3 (1262·3–1526·8)	23·5% (17·7 to 29·4%)	1·63
	Morocco	476·8 (435·2–521·4)	144% (133 to 156·2%)	1480·4 (1346·4–1625·7)	13·5% (8·4 to 19·4%)	1·35
	Palestine	35·2 (31·8–39)	168·2% (155·5 to 180·7%)	1341·8 (1205·3–1496·6)	-0·6% (-5·9 to 4·6%)	1·33
	Oman	29·8 (27–33)	176·1% (164 to 190%)	1327·2 (1192·6–1493·3)	-2·9% (-7·6 to 1·9%)	1·01
	Qatar	15·9 (14·1–17·9)	593·8% (566·3 to 621·8%)	1000·1 (908·5–1098·6)	-20·9% (-24·8 to -17·5%)	1·31
	Saudi Arabia	321·1 (290·9–356·3)	249·5% (214·9 to 281·5%)	1504·9 (1360·8–1680·6)	13·5% (1·9 to 23·8%)	1·37
	Syrian Arab Republic	156·7 (142·9–171·2)	94% (86·3 to 102%)	1224·1 (1107·6–1338·3)	-8·4% (-12·3 to -4·5%)	1·19
	Tunisia	141·9 (129–155·7)	191·6% (178·5 to 206·8%)	1130·1 (1028·1–1240·4)	25·6% (20·1 to 31·5%)	1·17
	Türkiye	838·4 (764·1–920·8)	114·7% (104·8 to 124·9%)	956·3 (872·9–1049·7)	-5·2% (-9·5 to -0·7%)	1·21
	United Arab Emirates	108·9 (98·6–119·9)	779·7% (733·4 to 827·4%)	2005·1 (1808·4–2238·2)	-3·3% (-7·9 to 1·1%)	1·23
	Yemen	210·9 (192·4–230·5)	215·7% (201·3 to 229·9%)	1355·2 (1220·3–1492·9)	15·9% (9·8 to 21·5%)	1·27
	Afghanistan	208 (188·8–227)	148·9% (135 to 162·7%)	1309·1 (1187·6–1435·3)	18·2% (12·4 to 23·2%)	1·28
	Sudan	325·7 (297·5–355·5)	153·9% (143·4 to 165%)	1520·7 (1375·6–1681·8)	23·8% (18·5 to 29·5%)	1·35
Intracerebral haemorrhage	Global	20663·9 (18016·2–23417·9)	57·8% (56·1 to 59·6%)	248·8 (217·1–281·4)	-16·8% (-18·2 to -15·4%)	0·9
	North Africa and Middle East	1301·3 (1169·2–1441·4)	89·8% (86 to 93·8%)	241·6 (217·5–265·7)	-21·1% (-23·1 to -19·2%)	1·07
	Algeria	78·7 (70·9–86·7)	50% (43·8 to 56·9%)	201·9 (182·3–221·3)	-39·9% (-42·7 to -37·1%)	1·14
	Bahrain	2·9 (2·5–3·2)	223·4% (207·8 to 239·9%)	186 (166·5–205·7)	-31·2% (-34·4 to -28·1%)	1·3
	Egypt	191·8 (173·5–212·1)	69% (62·2 to 75·9%)	236·1 (213·7–260·5)	-21·7% (-25·2 to -18·5%)	1·19
	Iran	115·8 (100–132·7)	89·9% (83·3 to 96·5%)	138·3 (120·1–157·2)	-16·3% (-19·5 to -13·1%)	0·99
	Iraq	110·4 (101·1–120)	164·6% (154·8 to 175·5%)	346·5 (316·6–378·4)	-16% (-19·1 to -12·4%)	0·81
	Jordan	19·7 (17·3–22·3)	218·2% (201·4 to 234·9%)	213·9 (189·8–238·7)	-29·2% (-33·2 to -25%)	1·07

Table S11 Cause- and sex-specific burden, mortality, incidence, and prevalence of neurological conditions in North Africa and Middle East countries

		**All Ages**		**Age-standardised**		
		Number (thousand)	Percent change	Rate per 100,000	Percent change	Sex ratio
**Measure**		2019	From 1990 to 2019	2019	From 1990 to 2019	2019
Cause	Location	Mean (95% UI)	Mean (95% UI)	Mean (95% UI)	Mean (95% UI)	F:M
	Kuwait	7·7 (6·7–8·7)	239·2% (220·8 to 258%)	181·9 (160·3–202·9)	-5·9% (-11 to -1·2%)	0·93
	Lebanon	9 (8–10)	51·2% (44·4 to 58%)	170·3 (151·9–188·9)	-25·4% (-29 to -21·9%)	1·31
	Libya	13·2 (11·9–14·5)	104·8% (97 to 113·1%)	202·1 (182·3–222·3)	-21·3% (-24·3 to -18%)	1·35
	Morocco	81·1 (73·4–90)	46·4% (40·9 to 51·9%)	227·7 (205·7–251·9)	-29% (-31·8 to -26%)	1·14
	Palestine	6·1 (5·3–7·1)	137·1% (123·4 to 153%)	176·6 (155·3–203·6)	-21·6% (-26·1 to -16·1%)	1·13
	Oman	7·3 (6·3–8·3)	123·3% (111·7 to 136·7%)	215·3 (191·2–240·9)	-30·2% (-33·6 to -26·2%)	1·57
	Qatar	5·1 (4·4–5·8)	492·5% (460·9 to 527·5%)	209·1 (184·9–232·2)	-28·6% (-32·2 to -24·8%)	1·19
	Saudi Arabia	153·8 (128·3–183·3)	202·7% (182·9 to 223%)	493·7 (421·2–576·3)	-5·3% (-10·8 to 0%)	1·18
	Syrian Arab Republic	42·2 (38·2–46·3)	34·9% (29·2 to 42·7%)	298·5 (270·2–327·4)	-30·1% (-33·2 to -26·4%)	0·89
	Tunisia	16·6 (14·3–18·8)	118·3% (97·6 to 140%)	128·2 (110–144·9)	2% (-6·9 to 12·1%)	1·12
	Türkiye	212·8 (190·9–233·9)	73·1% (65·5 to 81·5%)	234·6 (210·6–257·5)	-15·9% (-19·7 to -11·7%)	1·08
	United Arab Emirates	25 (22·3–28·1)	509·4% (482·8 to 536·8%)	282·6 (253·7–312·3)	-30·3% (-33·4 to -27·3%)	1·08
	Yemen	51·2 (46·6–56·5)	82·4% (74·1 to 92·2%)	260 (236·1–286)	-37% (-39·8 to -33·6%)	1·04
	Afghanistan	74·9 (67·2–82·1)	90·7% (79·7 to 103%)	355·1 (316·2–392)	-27% (-30·2 to -23·5%)	1·33
	Sudan	74·7 (67·9–82·2)	54·2% (47 to 62·2%)	279·6 (253·9–307·1)	-31·9% (-34·9 to -28·8%)	1·14
Subarachnoid haemorrhage	Global	8396·5 (7188·2–9833·3)	64·5% (60·4 to 68%)	101·6 (87·1–118·5)	-12·9% (-15 to -11·5%)	1·44
	North Africa and Middle East	376·9 (320·2–444·6)	103·4% (85 to 112·9%)	64 (54·5–75·3)	-18·1% (-26·3 to -14·1%)	1·58
	Algeria	27·3 (21·8–32·4)	97·8% (51·7 to 114·1%)	64·7 (51·8–76·8)	-24·2% (-43 to -17·1%)	1·5
	Bahrain	1·1 (0·9–1·3)	361·9% (326·7 to 396·5%)	53·2 (44·2–63·1)	-6·4% (-11·9 to -1·2%)	1·52
	Egypt	54·4 (45·4–64·2)	68·6% (46·1 to 79·3%)	61·4 (51·6–72·5)	-21·5% (-33 to -16·4%)	1·64
	Iran	45·4 (37·3–54·9)	127·3% (117·8 to 136·1%)	49·4 (40·8–59·3)	-7·1% (-8·9 to -5·2%)	1·56
	Iraq	21 (16·9–24·9)	141·6% (88·5 to 162%)	58·9 (46–70)	-26·1% (-45·7 to -19·5%)	1·61
	Jordan	7·8 (6·6–9·3)	347·5% (322·3 to 392·6%)	74·9 (62·3–88·5)	-1·1% (-5·6 to 8·6%)	1·51
	Kuwait	3·9 (3·2–4·6)	281·6% (257·9 to 310·1%)	71·5 (59–84·8)	2·1% (-3 to 10%)	1·64
	Lebanon	3·6 (3–4·3)	81% (63·8 to 91·7%)	67·8 (56·1–80·9)	-9·6% (-18·9 to -4·4%)	1·62
	Libya	4·8 (3·9–5·7)	146·3% (106·5 to 162·2%)	63·7 (51·5–75·1)	-11·4% (-27·1 to -6·2%)	1·6
	Morocco	24·6 (20·5–29·4)	58·4% (35·6 to 68·5%)	66·1 (55·6–78·6)	-22·9% (-34·4 to -17·4%)	1·55
	Palestine	2·5 (2·1–3)	207·6% (193·3 to 224·2%)	65·6 (54·2–77·7)	-4·3% (-9 to 0·6%)	1·64
	Oman	2·6 (2·2–3·1)	179·6% (153·1 to 197·5%)	58 (48·9–68·6)	-18·9% (-29·7 to -13·9%)	1·67
	Qatar	2·4 (2–2·9)	577% (448·3 to 625·5%)	73·6 (57·6–87·5)	-17·4% (-38·8 to -11·6%)	1·54
	Saudi Arabia	23·5 (19·5–27·7)	252·7% (233·5 to 271·3%)	58·5 (48·5–69·5)	-3·1% (-9·2 to 1%)	1·64
	Syrian Arab Republic	9·4 (7·4–11·3)	61·6% (28·4 to 73·6%)	63·1 (50·8–74·9)	-15·5% (-32·3 to -9·8%)	1·44
	Tunisia	8·7 (7–10·3)	119% (70·2 to 145·7%)	65·2 (53·3–76·9)	3·7% (-19 to 15·8%)	1·63
	Türkiye	74 (62·7–86·5)	63·7% (55·6 to 73·9%)	78·4 (66·2–91·3)	-19·2% (-23·3 to -14·1%)	1·52
	United Arab Emirates	8 (6·7–9·5)	644·4% (588·5 to 698·8%)	58 (49–68·4)	-15·4% (-23·2 to -10·8%)	1·69
	Yemen	15 (12·7–17·6)	138·3% (125·8 to 151·7%)	68·9 (58·1–81·4)	-17·6% (-21·9 to -12·6%)	1·61
	Afghanistan	16·4 (13·7–19·5)	170·2% (152·7 to 189·5%)	66·9 (56·4–79·3)	-10·6% (-14·9 to -6·4%)	1·58
	Sudan	20·1 (17–23·6)	87·1% (77·4 to 98·7%)	67·6 (57·5–79·7)	-19·5% (-23·9 to -14·5%)	1·57
Neurological disorders†*	Global	2658932·4 (2452847·9–2858528·8)	54·9% (52·4 to 57·4%)	33451·9 (30870·7–36082)	0·4% (-0·1 to 0·9%)	1·19
	North Africa and Middle East	208460·5 (190465·7–226945·6)	101·2% (95·9 to 106·6%)	34170·6 (31389·5–37068·5)	1·4% (0·6 to 2·3%)	1·19
	Algeria	14066·4 (12788·1–15403)	86·2% (78·9 to 93·9%)	33570·1 (30564·3–36622·8)	0·1% (-0·7 to 1%)	1·19
	Bahrain	524·4 (473·9–576·7)	221·5% (205·6 to 238·1%)	33112·7 (30197·4–36147)	-0·1% (-1·2 to 1%)	1·18
	Egypt	34352·8 (31415·8–37326·1)	96·6% (89 to 104·2%)	35726·9 (32855·2–38512·6)	2·8% (-0·8 to 6·7%)	1·2
	Iran	33461·6 (30981·9–35941·1)	81·4% (73·2 to 90%)	38297·2 (35449·7–41091·4)	5·3% (3·4 to 7·3%)	1·15

Table S11 Cause- and sex-specific burden, mortality, incidence, and prevalence of neurological conditions in North Africa and Middle East countries

		All Ages		Age-standardised		
		Number (thousand)	Percent change	Rate per 100,000	Percent change	Sex ratio
**Measure**		2019	From 1990 to 2019	2019	From 1990 to 2019	2019
Cause	Location	Mean (95% UI)	Mean (95% UI)	Mean (95% UI)	Mean (95% UI)	F:M
	Iraq	13749·7 (12424·5–15155·1)	174·7% (168·3 to 181·5%)	33513·6 (30526·4–36599·7)	0·2% (-0·6 to 1·1%)	1·18
	Jordan	3820·3 (3444·4–4203·3)	248·2% (237·8 to 259·4%)	33366·2 (30403·8–36446·1)	0·1% (-0·7 to 0·9%)	1·19
	Kuwait	1592·6 (1444·6–1756·4)	180·3% (168·3 to 192·5%)	33272 (30301·2–36326·1)	0·8% (-1·7 to 3·5%)	1·2
	Lebanon	1765·5 (1607·1–1930·3)	76·4% (71·6 to 81·4%)	33723·8 (30708·5–36796·6)	0·5% (-0·4 to 1·4%)	1·18
	Libya	2417·2 (2195–2643·4)	96·6% (87·7 to 105·5%)	33496·5 (30469–36534·7)	0·7% (-0·1 to 1·4%)	1·18
	Morocco	12337 (11206·9–13482·8)	58·5% (53·8 to 63%)	33537 (30530·4–36612·6)	0·2% (-0·6 to 1%)	1·19
	Palestine	1555·7 (1395·3–1715·1)	172·9% (167·6 to 178·5%)	33527·9 (30565·1–36597·7)	-0·1% (-0·9 to 0·8%)	1·18
	Oman	1575·2 (1415–1754·3)	180·2% (168·3 to 193%)	32834·2 (29837·3–35871·3)	0·1% (-0·9 to 1%)	1·18
	Qatar	1029·9 (924·9–1150·2)	608·8% (592 to 626%)	32245·1 (29313·4–35220·7)	-0·7% (-1·7 to 0·3%)	1·19
	Saudi Arabia	12255·8 (11107·4–13550·3)	166·8% (153·5 to 180·3%)	31719·5 (28912–34577·5)	-1% (-3·6 to 1·9%)	1·16
	Syrian Arab Republic	4994 (4532·8–5479·4)	37·4% (32·5 to 42·4%)	33730·3 (30733·5–36791·7)	1·1% (0·2 to 1·9%)	1·19
	Tunisia	4068·6 (3719·3–4444·1)	55·6% (49·5 to 61·5%)	33620·3 (30668–36658·7)	0·5% (-0·2 to 1·3%)	1·19
	Türkiye	28054 (25592·4–30582·4)	57·3% (50·3 to 63·9%)	31966·2 (29201·8–34866·9)	1% (-1·6 to 3·6%)	1·24
	United Arab Emirates	3418·9 (3055·9–3814·3)	475·3% (441·9 to 508·3%)	32631·3 (29615·7–35673·5)	-0·1% (-1·3 to 1%)	1·18
	Yemen	9573·6 (8555–10612·4)	163·6% (158·4 to 168·8%)	33439·2 (30457·6–36527·8)	0·1% (-0·6 to 0·9%)	1·19
	Afghanistan	10980·2 (9799·2–12197·3)	234·7% (227·3 to 241·4%)	33401·5 (30465·4–36481)	-0·4% (-1·3 to 0·4%)	1·19
	Sudan	12655·4 (11340·5–13986·7)	119·4% (116·7 to 122·3%)	33434·1 (30397·1–36506·1)	0·1% (-0·7 to 0·9%)	1·19
Alzheimer's disease and other dementias	Global	51624·2 (44277–59021·5)	160·9% (156 to 166·3%)	682·5 (585·2–782·7)	5·7% (4·3 to 7%)	1·28
	North Africa and Middle East	2485·1 (2117·2–2865)	184·5% (178·1 to 190·7%)	777·6 (660·8–896)	3% (1·5 to 4·6%)	1·11
	Algeria	193·1 (162·8–224)	231·7% (211·8 to 253·3%)	777·5 (659·7–898·6)	1·5% (-1·9 to 4·8%)	1·1
	Bahrain	3·8 (3·2–4·3)	450·3% (424·1 to 479·2%)	801·1 (684·2–925·8)	3·5% (0·2 to 7·1%)	1·1
	Egypt	295·8 (250·9–342·6)	121·4% (113·8 to 129·1%)	750·4 (635·6–866·1)	5·2% (1·4 to 8·7%)	1·12
	Iran	470·9 (397·9–542·9)	303·8% (282·7 to 328·6%)	789 (667·8–910·4)	1·6% (-0·3 to 3·2%)	1·1
	Iraq	118·9 (101·5–137·3)	165·6% (156·2 to 176·3%)	764·1 (647·4–882)	3·8% (0·7 to 7·5%)	1·11
	Jordan	32 (27–37·4)	432·9% (411·5 to 453·7%)	763·4 (645·9–890)	3·8% (-0·1 to 7·8%)	1·1
	Kuwait	14·3 (12·3–16·4)	401·3% (382·3 to 422%)	798·8 (682·4–922)	1·3% (-2·1 to 4·8%)	1·12
	Lebanon	39·9 (34·1–46·4)	221% (204·8 to 235·4%)	792·4 (677·6–916·5)	4·2% (0·5 to 8·4%)	1·11
	Libya	30·5 (25·8–35·2)	169·1% (159·2 to 178·8%)	755·9 (635·1–874·7)	-1·1% (-4·6 to 2·6%)	1·1
	Morocco	174·6 (147·7–202·3)	145% (135·7 to 155%)	761·7 (643·9–884·8)	0·5% (-2·9 to 4·3%)	1·1
	Palestine	12 (10·3–14)	135·9% (126·9 to 144·7%)	771·8 (652·9–891)	2·1% (-1·8 to 5·8%)	1·09
	Oman	6·7 (5·7–7·8)	143·4% (135·2 to 153·2%)	789·5 (668·6–913·6)	1·1% (-2·4 to 4·6%)	1·11
	Qatar	2·7 (2·3–3·2)	685·6% (629·8 to 752·1%)	779·2 (654·8–905·3)	3·9% (0·1 to 8·2%)	1·1
	Saudi Arabia	67·8 (58–77·8)	133·2% (122·1 to 147%)	770·3 (648·2–891·9)	0·8% (-2·6 to 5·3%)	1·12
	Syrian Arab Republic	63·7 (53·7–74)	117·3% (106·6 to 128·4%)	770·3 (653·1–892·3)	2·3% (-1·7 to 6·3%)	1·12
	Tunisia	84·6 (72–98)	215·4% (200·5 to 231·5%)	791·8 (670·4–916)	2·1% (-1·3 to 5·5%)	1·12
	Türkiye	643·1 (550·3–741·8)	198·7% (188·4 to 208·2%)	805·1 (686·6–926·2)	4·7% (1 to 8·1%)	1·13
	United Arab Emirates	8·1 (6·6–9·6)	636·5% (579·7 to 696·4%)	721·3 (602·2–842·2)	-0·1% (-3·5 to 3·2%)	1·11

Table S11 Cause- and sex-specific burden, mortality, incidence, and prevalence of neurological conditions in North Africa and Middle East countries

		All Ages		Age-standardised		
		Number (thousand)	Percent change	Rate per 100,000	Percent change	Sex ratio
**Measure**		2019	From 1990 to 2019	2019	From 1990 to 2019	2019
Cause	Location	Mean (95% UI)	Mean (95% UI)	Mean (95% UI)	Mean (95% UI)	F:M
	Yemen	64·8 (54·8–74·7)	190·8% (179·6 to 203%)	752·3 (639·3–869·6)	-2·7% (-6 to 1·1%)	1·09
	Afghanistan	54·8 (45·9–63·6)	54·1% (47·3 to 60·4%)	735·4 (616·4–852·4)	-1·6% (-5·3 to 2·4%)	1·09
	Sudan	100·5 (85·7–116)	111·9% (101·4 to 122%)	746·2 (630·2–861·7)	0·2% (-3·3 to 3·8%)	1·1
Parkinson's disease	Global	8511 (7288·5–9841·4)	155·5% (150·4 to 161%)	106·3 (91·2–122·2)	15·9% (13·3 to 18·5%)	0·68
	North Africa and Middle East	309·9 (265–362·8)	199·5% (188·8 to 211·2%)	82·6 (70·2–95·6)	15·4% (11·5 to 20%)	0·74
	Algeria	24 (19·9–28·8)	217·4% (181 to 255·3%)	80·4 (66·5–96·8)	10·2% (-1·1 to 22%)	0·78
	Bahrain	0·7 (0·6–0·8)	493·1% (408·4 to 582·9%)	94 (75·9–113·4)	12·5% (-5·2 to 33·3%)	0·73
	Egypt	43·8 (36·1–52·6)	148·8% (120 to 177·7%)	84·4 (69·1–101)	17·3% (3·6 to 30·7%)	0·85
	Iran	56·5 (47·3–66·9)	260·9% (235 to 285·6%)	85·1 (70·2–101·3)	13·1% (10·4 to 15·8%)	0·75
	Iraq	14·4 (11·8–17·1)	193·4% (168·2 to 226·9%)	74·2 (61·1–87·9)	5·1% (-4·3 to 17·1%)	0·67
	Jordan	4·1 (3·4–4·9)	425·7% (364·4 to 488·4%)	77 (63·4–91·5)	3% (-11·5 to 16·6%)	0·79
	Kuwait	1·6 (1·3–1·9)	315·1% (261·1 to 379·4%)	71·4 (59·1–86·1)	-8·5% (-23·9 to 9·9%)	0·72
	Lebanon	4·1 (3·5–4·8)	192·1% (157·6 to 228·7%)	77·6 (66·5–90·9)	9·3% (-3·1 to 23·6%)	0·75
	Libya	3·8 (3·2–4·5)	213·5% (184·3 to 251·5%)	84·1 (69·3–98·3)	13·5% (2·3 to 27·9%)	0·74
	Morocco	20·5 (17·2–23·8)	179·5% (147·3 to 219·8%)	73·7 (61·9–85·8)	20·7% (6·3 to 39%)	0·69
	Palestine	1·5 (1·3–1·8)	158·4% (129·8 to 188·4%)	78·3 (65·2–92·4)	2·1% (-8·8 to 14·4%)	0·69
	Oman	1·4 (1·1–1·7)	213·3% (180·9 to 249%)	112·5 (92·9–135·2)	28% (14 to 44·8%)	0·62
	Qatar	0·8 (0·6–1)	811·6% (709·6 to 914·7%)	119·3 (98·3–142·9)	20·2% (6·9 to 36·3%)	0·89
	Saudi Arabia	13·3 (11–15·9)	233·9% (195·1 to 279·3%)	107·6 (90·4–127·8)	27·7% (13·1 to 45%)	0·6
	Syrian Arab Republic	8·3 (6·9–9·9)	160·5% (132·4 to 191·7%)	81·6 (67·8–97)	11·6% (-1·3 to 26·8%)	0·75
	Tunisia	9·3 (7·8–10·9)	208·5% (170·6 to 249·5%)	79·6 (66·9–93·2)	15·1% (1·8 to 30·4%)	0·73
	Türkiye	72·5 (59·4–86·8)	225% (194·8 to 259·1%)	86·9 (71–104·1)	22·3% (10·9 to 35·5%)	0·71
	United Arab Emirates	3·1 (2·5–3·9)	901% (789·1 to 1022·1%)	116 (97·6–138·8)	11·8% (0·6 to 23·6%)	0·79
	Yemen	7·2 (5·9–8·7)	205·1% (175·4 to 242%)	63·6 (51·9–76·4)	9·7% (-1·8 to 24·7%)	0·76
	Afghanistan	7·4 (6·1–8·8)	61·8% (44·4 to 82·4%)	69·3 (58·2–82·4)	-3·3% (-12·9 to 8%)	0·72
	Sudan	11·4 (9·5–13·7)	102·4% (78·9 to 129·8%)	69·7 (58·3–83·4)	2·1% (-9·9 to 16·1%)	0·7
Idiopathic epilepsy	Global	25111·1 (19033·6–31433)	63·9% (39·3 to 93%)	326·3 (247·8–408·3)	13% (-3·4 to 31·8%)	0·92
	North Africa and Middle East	1990·9 (1360·2–2600)	77·6% (26·2 to 150·6%)	336·9 (231·8–437·4)	8·6% (-23 to 52·4%)	0·88
	Algeria	131·1 (38·6–215)	40·1% (-62·6 to 496·7%)	322·5 (94·5–529·8)	-7·9% (-76·4 to 293%)	0·93
	Bahrain	5·5 (1·7–9·1)	143·6% (-26·4 to 883·4%)	450·6 (140·2–726·5)	-10·6% (-72·9 to 252·3%)	0·94
	Egypt	274·8 (77·9–460·8)	84·3% (-49·4 to 746%)	275·4 (79·2–459·6)	8·2% (-70·5 to 395%)	0·79
	Iran	281·7 (193–373·6)	28·3% (-5·1 to 72·5%)	352·8 (240·3–465·2)	1·5% (-24 to 35·3%)	0·88
	Iraq	126·9 (33·6–217·4)	125·5% (-39·4 to 851·8%)	299·1 (77·8–510·2)	-3·3% (-74·2 to 300·6%)	0·93
	Jordan	35·4 (9·7–59·7)	204·5% (-20·6 to 1036·4%)	302·9 (83·8–498·4)	3·5% (-73·1 to 291·1%)	0·9
	Kuwait	15·2 (4·3–24·1)	119·1% (-37·4 to 718·6%)	388·9 (106·7–618·4)	-4·5% (-73·2 to 267·1%)	0·92
	Lebanon	17·5 (5·1–28·2)	56·4% (-59·4 to 598·3%)	343·8 (99·5–558·2)	3·3% (-72·7 to 371·4%)	0·89
	Libya	18·5 (6·1–29·2)	25·6% (-59·8 to 364%)	287·1 (93·7–450·4)	-15·5% (-72·8 to 217·9%)	0·88
	Morocco	103·3 (24·8–182·3)	55·2% (-62·8 to 593%)	295·3 (70·8–519·2)	16·6% (-72·4 to 416·5%)	0·98
	Palestine	15·2 (4–25·7)	137·7% (-41·1 to 1001·8%)	301·3 (78·4–510·7)	2·2% (-74·3 to 374·9%)	0·93
	Oman	14 (4·3–22·8)	153% (-24·4 to 892·3%)	339·1 (106·3–548·8)	20·3% (-65·1 to 372·1%)	0·91
	Qatar	9·5 (3·1–15·5)	484·5% (71·8 to 2028·4%)	397·3 (130·2–630·9)	-1·7% (-71 to 262·2%)	1·04
	Saudi Arabia	187·6 (56·2–294)	209·3% (-14·6 to 1107·1%)	563·3 (162·9–883·2)	41·8% (-60·1 to 462·6%)	1·02
	Syrian Arab Republic	39·1 (11·4–66·2)	18·9% (-68·8 to 447·1%)	269·8 (79·6–454·1)	13·8% (-70·2 to 417·4%)	0·93
	Tunisia	31·1 (8·9–50·4)	47·6% (-64 to 534·6%)	279·7 (79·6–456·8)	16·3% (-72·2 to 395·8%)	0·96
	Türkiye	373·6 (110·8–580·8)	57·9% (-53·3 to 564·8%)	476 (143·4–741·4)	23·8% (-62·6 to 422·5%)	0·8
	United Arab Emirates	38 (11·1–60·6)	306·1% (16 to 1404·2%)	491·9 (143–776·3)	-13·1% (-74·9 to 216·9%)	0·84
	Yemen	72·9 (18–135·4)	113·8% (-55·2 to 1082%)	220·1 (53·2–403·6)	-3·3% (-79·5 to 439·8%)	0·89
	Afghanistan	93·8 (16·7–183·1)	204·7% (-33·9 to 1912·6%)	234·4 (41·5–453·3)	-9·9% (-80·5 to 489·9%)	1·09

Table S11 Cause- and sex-specific burden, mortality, incidence, and prevalence of neurological conditions in North Africa and Middle East countries

		**All Ages**		**Age-standardised**		
		Number (thousand)	Percent change	Rate per 100,000	Percent change	Sex ratio
**Measure**		2019	From 1990 to 2019	2019	From 1990 to 2019	2019
Cause	Location	Mean (95% UI)	Mean (95% UI)	Mean (95% UI)	Mean (95% UI)	F:M
	Sudan	104·1 (25·3–187·3)	110·9% (-49·8 to 1029·8%)	245·5 (60·3–444·7)	8·5% (-73·9 to 482%)	0·87
Multiple sclerosis	Global	1756·8 (1531·9–1973·6)	71·7% (66·3 to 76·8%)	21·3 (18·5–23·9)	-6·2% (-8·7 to -3·8%)	1·99
	North Africa and Middle East	222·7 (190·7–256·8)	171·3% (165·8 to 176·5%)	39 (33·6–44·7)	11·5% (10 to 12·8%)	1·98
	Algeria	17·6 (14·4–21·3)	222·5% (203·8 to 242%)	41·9 (34·2–50·6)	26·4% (20·4 to 33·6%)	1·98
	Bahrain	0·6 (0·5–0·7)	484·5% (438·6 to 535·1%)	32·7 (26·3–40·3)	29·1% (21·6 to 36·4%)	2·06
	Egypt	17·8 (14–21·6)	160·1% (150 to 171·7%)	20·6 (16·4–25)	22·2% (17·7 to 27·4%)	1·98
	Iran	46·9 (39·9–53·7)	135·2% (124·4 to 145·7%)	51·2 (44·1–58·5)	-4·9% (-8·2 to -1·6%)	2·09
	Iraq	12·8 (10·1–16·1)	274·2% (256·6 to 293·6%)	37·8 (30–46·9)	19·5% (14·2 to 25·2%)	1·93
	Jordan	4·6 (3·7–5·6)	345·7% (289·6 to 401·1%)	47 (37·9–56·8)	-1·2% (-13·3 to 10·6%)	2·07
	Kuwait	2·7 (2·2–3·3)	485·7% (448·8 to 526·8%)	53 (43·7–63·5)	59·7% (50·5 to 69·9%)	1·68
	Lebanon	2·9 (2·4–3·5)	179·2% (162·9 to 199·5%)	53·9 (43·9–64·2)	34·7% (27·2 to 43·9%)	1·95
	Libya	3 (2·5–3·6)	261·2% (240·2 to 282·5%)	41·1 (34·2–48·9)	31·6% (24·9 to 38·4%)	1·82
	Morocco	15 (12·2–18·3)	151·6% (136·6 to 168·2%)	40·9 (33·3–49·7)	26·4% (19·3 to 34·6%)	1·96
	Palestine	1·5 (1·2–1·8)	268% (252·9 to 286·8%)	41·5 (33·6–50·2)	18·5% (13·9 to 24·3%)	1·78
	Oman	1·5 (1·2–1·8)	352·9% (326·2 to 381·2%)	34·3 (28·3–41)	33·8% (27·2 to 41·9%)	1·69
	Qatar	1·8 (1·6–2)	1183·7% (988·2 to 1408·8%)	56·1 (49·2–62·9)	54·1% (34 to 77·5%)	2·58
	Saudi Arabia	10·8 (8·3–13·6)	364·9% (340 to 389·3%)	28·4 (22·4–35·2)	29·7% (23·9 to 35·9%)	2·13
	Syrian Arab Republic	5·9 (4·7–7·2)	130·5% (117·5 to 143·2%)	42·1 (33·4–51·5)	23·8% (18·6 to 28·4%)	2·1
	Tunisia	6·6 (5·4–7·9)	170·3% (156·3 to 186·2%)	49·7 (40·8–59·8)	28·4% (22·4 to 34·2%)	1·95
	Türkiye	45·8 (43·9–47·9)	110·2% (105·2 to 115·9%)	48·7 (46·7–50·9)	2·5% (0·1 to 5%)	1·78
	United Arab Emirates	3·1 (2·6–3·7)	709·6% (610·3 to 804·1%)	23·8 (20·5–27·5)	-0·9% (-9·2 to 7·7%)	1·82
	Yemen	5 (4–6·2)	253·5% (235·8 to 271·5%)	23·3 (18·6–28·4)	22·9% (16·8 to 29·2%)	1·72
	Afghanistan	10 (8·2–12·1)	259·8% (239·6 to 281·6%)	43·1 (36–51·2)	19·9% (13·6 to 25·7%)	2·06
	Sudan	6·7 (5·3–8·3)	180% (166·4 to 195%)	23·1 (18·5–27·9)	23·9% (18·2 to 30·7%)	1·74
Migraine	Global	1128087·3 (979598·8–1298138·1)	56·3% (52·3 to 60·5%)	14107·3 (12270·3–16239)	1·7% (0·7 to 2·8%)	1·73
	North Africa and Middle East	96931·5 (83756·7–112609·4)	100·9% (92·7 to 108·8%)	15355 (13305·5–17806)	-0·1% (-1·5 to 1·4%)	1·76
	Algeria	6629·2 (5653–7727·2)	87·9% (76·8 to 98·6%)	15365·7 (13120·8–17933·6)	-0·1% (-0·2 to 0%)	1·72
	Bahrain	239·7 (205·3–279·9)	215·9% (197·3 to 237·3%)	14419·8 (12318·8–16876·2)	-0·7% (-1 to -0·3%)	1·72
	Egypt	15818·7 (13828·6–18240·8)	97·4% (83·2 to 112·2%)	15809·3 (13960·9–18055·5)	1·8% (-5 to 9%)	1·82
	Iran	14579·4 (12748–16638)	75·8% (63·4 to 88·4%)	16048·8 (14063·2–18371·8)	-0·5% (-4 to 2·8%)	1·76
	Iraq	6522·5 (5506·5–7700·2)	179·5% (170·3 to 187·6%)	15300·4 (13067–17857·9)	-0·1% (-0·1 to -0·1%)	1·72
	Jordan	1789·5 (1517·3–2114·9)	247·9% (233·8 to 262·4%)	15033 (12863·2–17565·4)	-1% (-1·1 to -0·8%)	1·72
	Kuwait	764·4 (652·8–896·7)	189·8% (171·5 to 208·5%)	14884·8 (12735·9–17378·9)	3·7% (-0·5 to 7·9%)	1·69
	Lebanon	819·1 (700·6–950·6)	75·4% (68·5 to 81·6%)	15516·6 (13250·8–18080·2)	0·2% (0 to 0·4%)	1·72
	Libya	1150 (980·2–1340·8)	102·9% (88·9 to 116·4%)	15252·6 (13031·9–17806·7)	1·9% (1·6 to 2·2%)	1·72
	Morocco	5801·2 (4955·3–6762·3)	57·5% (50·8 to 63·8%)	15400·4 (13149·2–17963·3)	-0·3% (-0·4 to -0·3%)	1·72
	Palestine	736·1 (619·2–875·3)	176·4% (169·4 to 183%)	15338·5 (13096·5–17900)	-1·2% (-1·4 to -0·9%)	1·72
	Oman	721·3 (607·2–849·2)	182·6% (166·6 to 197·2%)	14081·2 (12028–16515·9)	-1·5% (-1·8 to -1·2%)	1·72

Table S11 Cause- and sex-specific burden, mortality, incidence, and prevalence of neurological conditions in North Africa and Middle East countries

		All Ages		Age-standardised		
		Number (thousand)	Percent change	Rate per 100,000	Percent change	Sex ratio
**Measure**		2019	From 1990 to 2019	2019	From 1990 to 2019	2019
Cause	Location	Mean (95% UI)	Mean (95% UI)	Mean (95% UI)	Mean (95% UI)	F:M
	Qatar	457·4 (385·3–540·2)	596·8% (576 to 614·3%)	13295·3 (11309·2–15700·9)	-2·7% (-3·3 to -2·2%)	1·72
	Saudi Arabia	5954·6 (5057·3–6988·8)	173·9% (151·3 to 195·1%)	14325·3 (12266·3–16709·6)	-1·3% (-5·2 to 2·8%)	1·57
	Syrian Arab Republic	2354 (2000·1–2775·1)	38·7% (31·9 to 46·1%)	15691 (13390·1–18304·1)	2·5% (2 to 2·9%)	1·72
	Tunisia	1889·8 (1626·6–2191·2)	54% (45·3 to 62·5%)	15455 (13195·2–18014·9)	0·4% (0·4 to 0·5%)	1·72
	Türkiye	13296·1 (11425·7–15392·4)	55·1% (45·5 to 65·2%)	14888·4 (12817·8–17240·8)	0% (-4·1 to 4·2%)	1·93
	United Arab Emirates	1521·8 (1290·3–1798·7)	466·6% (428·2 to 509·6%)	13693·4 (11674·5–16104·4)	-0·7% (-1·1 to -0·3%)	1·72
	Yemen	4554 (3811·6–5444·9)	168·1% (161·7 to 173·9%)	15398·5 (13147·1–17961·2)	0% (-0·1 to 0·1%)	1·72
	Afghanistan	5201·9 (4319·1–6245·2)	240·2% (231·8 to 247·4%)	15315·6 (13076·7–17870·6)	-2·3% (-2·7 to -1·8%)	1·72
	Sudan	6032·2 (5066·7–7200·2)	121·7% (118·9 to 124·1%)	15412 (13164·6–17974·7)	-0·2% (-0·3 to -0·1%)	1·72
Tension-type headache	Global	1995172·5 (1751946·8–2242204·9)	52·6% (49·2 to 55·9%)	25113·5 (22020·8–28316·2)	-0·8% (-1·5 to 0%)	1·06
	North Africa and Middle East	149061·7 (128455·9–170990·9)	102·2% (93·5 to 111%)	24504·5 (21304·8–27987·5)	2% (0·7 to 3·4%)	1·02
	Algeria	9888 (8443·6–11527·6)	85·5% (74·2 to 98·1%)	23647·6 (20341·2–27276·9)	-0·1% (-0·1 to 0%)	1·02
	Bahrain	377·8 (318·5–442·8)	224·3% (196·2 to 253%)	23716 (20422–27274·1)	-0·1% (-0·3 to 0·1%)	1·02
	Egypt	25094·8 (21538·9–28759·9)	97·1% (85·8 to 108·5%)	26290·9 (22878·1–29775·3)	3·6% (-1·6 to 9·5%)	1·04
	Iran	25773 (22795·1–28770)	88·3% (74·4 to 101·8%)	29640·4 (26202·1–32949·4)	9·7% (6·5 to 13·1%)	0·99
	Iraq	9672·8 (8160·2–11338·8)	173·9% (163·8 to 183·3%)	23667·9 (20363·1–27281·6)	0% (0 to 0%)	1·02
	Jordan	2700·1 (2285·9–3158·5)	248·3% (232·6 to 266·5%)	23661·5 (20365·4–27259·2)	0·1% (0 to 0·2%)	1·02
	Kuwait	1117·9 (946·8–1313·4)	175·2% (156·1 to 195·5%)	23533 (20134·8–26965·1)	-0·9% (-4·9 to 3%)	1·05
	Lebanon	1237·3 (1062–1432·4)	75·6% (67·8 to 83·6%)	23655 (20353·1–27281·3)	0·1% (0 to 0·3%)	1·02
	Libya	1698·7 (1444·4–1978·9)	94·9% (81·4 to 109·5%)	23667·9 (20357·9–27281·4)	-0·1% (-0·2 to 0·1%)	1·02
	Morocco	8691·6 (7444·1–10049·1)	58·2% (51·7 to 65·2%)	23654·8 (20352·1–27282·8)	0% (-0·1 to 0%)	1·02
	Palestine	1094·4 (921–1287·9)	172·3% (164·6 to 180·4%)	23661·8 (20360·3–27277·7)	0% (-0·2 to 0·1%)	1·02
	Oman	1138·7 (948·5–1361·3)	181·2% (161·5 to 199·9%)	23692·5 (20423·5–27194·2)	-0·1% (-0·3 to 0%)	1·02
	Qatar	756·4 (622·3–910·8)	614·6% (587·5 to 637·7%)	23538·3 (20220·1–27033·6)	-0·5% (-0·9 to -0·2%)	1·02
	Saudi Arabia	8354·7 (7033·6–9767·9)	162·3% (142·1 to 182·5%)	21757·9 (18684·4–24972·1)	-2·7% (-7 to 2%)	1·01
	Syrian Arab Republic	3507·8 (2996·7–4066·4)	36·6% (28·8 to 44·8%)	23640·6 (20306·7–27263·7)	0% (-0·3 to 0·3%)	1·02
	Tunisia	2863·7 (2464·9–3296·8)	55% (45·8 to 64·3%)	23657·2 (20350·7–27289)	0·1% (0 to 0·1%)	1·02
	Türkiye	19035·8 (16410–21823·2)	57·2% (45·8 to 67·8%)	21726·3 (18745·7–24838·8)	1% (-2·9 to 5·2%)	1·02
	United Arab Emirates	2512·6 (2048·3–3008·5)	483·1% (427 to 537·9%)	23676 (20386·9–27204·5)	-0·2% (-0·5 to 0%)	1·02

Table S11 Cause- and sex-specific burden, mortality, incidence, and prevalence of neurological conditions in North Africa and Middle East countries

		All Ages		Age-standardised		
		Number (thousand)	Percent change	Rate per 100,000	Percent change	Sex ratio
**Measure**		2019	From 1990 to 2019	2019	From 1990 to 2019	2019
Cause	Location	Mean (95% UI)	Mean (95% UI)	Mean (95% UI)	Mean (95% UI)	F:M
	Yemen	6749 (5678·8–8024)	162·7% (155·4 to 169·6%)	23663 (20359·2–27287·1)	0% (-0·1 to 0·1%)	1·02
	Afghanistan	7750·9 (6436·5–9231·4)	234·6% (222·8 to 246·5%)	23693·8 (20386·8–27306·9)	0·4% (0·1 to 0·7%)	1·02
	Sudan	8894 (7481·3–10475·8)	118·3% (115·4 to 121·4%)	23598·8 (20291–27242·1)	-0·1% (-0·2 to -0·1%)	1·02
Motor neuron disease	Global	268·7 (231·9–310·7)	68·9% (62·5 to 75·7%)	3·4 (2·9–3·9)	1·9% (0·6 to 3·4%)	0·82
	North Africa and Middle East	15·6 (12·7–19)	103·3% (92·1 to 113·8%)	2·6 (2·1–3·1)	3·5% (2 to 5·1%)	0·86
	Algeria	1 (0·8–1·2)	89·8% (74·6 to 104·9%)	2·4 (1·9–2·8)	2% (-1·6 to 5·7%)	0·85
	Bahrain	<0·1 (<0·1–0·1)	235·2% (197·8 to 274·8%)	2·8 (2·3–3·4)	3·7% (-0·5 to 7·9%)	0·86
	Egypt	2·1 (1·7–2·6)	96·4% (87·1 to 105·5%)	2·2 (1·8–2·7)	4·2% (0·6 to 8·2%)	0·87
	Iran	2·5 (2–3·2)	86·9% (70·6 to 103·5%)	2·9 (2·3–3·5)	6·6% (4·9 to 8·4%)	0·89
	Iraq	1 (0·8–1·3)	170·7% (154·7 to 186·6%)	2·5 (2·1–3·1)	2·6% (-1·2 to 6·4%)	0·88
	Jordan	0·3 (0·2–0·4)	252% (224·4 to 277·9%)	2·7 (2·2–3·3)	3% (-1·7 to 7·2%)	0·84
	Kuwait	0·2 (0·1–0·2)	166·7% (143·3 to 192·9%)	3·1 (2·6–3·8)	-4·2% (-9·8 to 1·1%)	0·85
	Lebanon	0·1 (0·1–0·2)	77·2% (65·9 to 87·5%)	2·8 (2·3–3·4)	2·8% (-1 to 6·9%)	0·87
	Libya	0·2 (0·1–0·2)	81·7% (64·2 to 98·3%)	2·3 (1·9–2·8)	-4·9% (-8·6 to -0·9%)	0·91
	Morocco	0·9 (0·7–1·1)	62·3% (51·4 to 73·3%)	2·4 (1·9–2·9)	4% (0 to 8%)	0·84
	Palestine	0·1 (0·1–0·1)	175·2% (158·3 to 189·6%)	2·6 (2·1–3·2)	4% (0 to 7·7%)	0·89
	Oman	0·1 (0·1–0·1)	182·6% (159·7 to 207·3%)	2·3 (1·9–2·8)	2·7% (-1·3 to 6·9%)	0·87
	Qatar	0·1 (0·1–0·1)	631·5% (587·1 to 678·9%)	2·8 (2·3–3·5)	4·2% (-0·3 to 8·7%)	0·86
	Saudi Arabia	0·9 (0·7–1·2)	166·9% (141·5 to 192·2%)	2·4 (2–2·9)	2·9% (-0·9 to 6·8%)	0·85
	Syrian Arab Republic	0·4 (0·3–0·5)	28·3% (17·7 to 41·1%)	2·5 (2·1–3·1)	0·4% (-3·4 to 4·6%)	0·87
	Tunisia	0·3 (0·3–0·4)	61·1% (48·2 to 75·5%)	2·7 (2·2–3·3)	4·1% (-0·5 to 8·1%)	0·89
	Türkiye	3·2 (2·6–3·7)	77·2% (64·6 to 89·4%)	3·6 (3–4·2)	10·2% (6·2 to 14·5%)	0·83
	United Arab Emirates	0·3 (0·2–0·4)	472·2% (400 to 543·2%)	2·7 (2·2–3·2)	-0·5% (-4·4 to 4·5%)	0·87
	Yemen	0·5 (0·4–0·6)	153·1% (137·6 to 168·2%)	1·6 (1·3–2)	-0·8% (-4·5 to 3·2%)	0·86
	Afghanistan	0·7 (0·5–0·8)	238·4% (219·8 to 260%)	1·9 (1·6–2·4)	0·2% (-4 to 4·2%)	0·82
	Sudan	0·6 (0·5–0·8)	123·4% (113·4 to 134·2%)	1·7 (1·4–2·1)	4·1% (0·4 to 8·2%)	0·86
Other neurological disorders	Global	56·9 (39·1–77·8)	66·3% (56·8 to 77%)	0·7 (0·5–1)	6·4% (3·6 to 9·5%)	0·82
	North Africa and Middle East	3·9 (2·6–5·5)	96·6% (81 to 113·8%)	0·7 (0·5–1)	0·3% (-0·9 to 1·5%)	1·00
	Algeria	0·3 (0·2–0·4)	91·2% (70·5 to 114·3%)	0·7 (0·5–1)	-0·1% (-0·2 to 0·1%)	1·00
	Bahrain	<0·1 (<0·1–<0·1)	251·2% (197·9 to 315·7%)	0·7 (0·5–0·9)	-0·4% (-0·9 to 0%)	1·00
	Egypt	0·6 (0·4–0·9)	88·8% (80·3 to 97·3%)	0·7 (0·5–1)	0% (-0·2 to 0·3%)	1·00
	Iran	0·5 (0·4–0·8)	69·6% (46·7 to 96·5%)	0·6 (0·4–0·9)	0·2% (-0·1 to 0·4%)	0·97
	Iraq	0·3 (0·2–0·4)	156·2% (137·6 to 177·1%)	0·7 (0·5–1)	0% (0 to 0%)	1·00
	Jordan	0·1 (0·1–0·1)	288·5% (245 to 338·6%)	0·9 (0·6–1·2)	11·4% (3·6 to 20·5%)	1·02
	Kuwait	<0·1 (<0·1–<0·1)	197·2% (159·5 to 237·8%)	0·7 (0·5–1)	2% (-4 to 9·2%)	0·99
	Lebanon	<0·1 (<0·1–<0·1)	74·9% (63·2 to 87·3%)	0·7 (0·5–1)	0·2% (-0·1 to 0·6%)	1·00
	Libya	<0·1 (<0·1–0·1)	126·5% (89·7 to 177·6%)	0·6 (0·4–0·9)	19·1% (6·4 to 36·4%)	0·98
	Morocco	0·2 (0·2–0·3)	63% (48·7 to 78·5%)	0·7 (0·5–1)	0% (-0·1 to 0·1%)	1·00
	Palestine	<0·1 (<0·1–<0·1)	154% (137·8 to 173·2%)	0·7 (0·5–1)	-0·5% (-1 to -0·1%)	1·00
	Oman	<0·1 (<0·1–<0·1)	153·2% (121·5 to 186·7%)	0·7 (0·5–0·9)	-0·2% (-0·5 to 0·1%)	1·00
	Qatar	<0·1 (<0·1–<0·1)	591·6% (536·1 to 640·5%)	0·7 (0·5–0·9)	0·3% (-0·3 to 1%)	1·00
	Saudi Arabia	0·2 (0·1–0·3)	153·9% (116·9 to 194·2%)	0·7 (0·5–1)	0·3% (0·1 to 0·4%)	1·00
	Syrian Arab Republic	0·1 (0·1–0·1)	36·5% (18·7 to 57·6%)	0·7 (0·5–1)	-0·1% (-0·4 to 0·3%)	1·00
	Tunisia	0·1 (0·1–0·1)	64·4% (46·9 to 83%)	0·7 (0·5–1)	0% (-0·2 to 0·1%)	1·00
	Türkiye	0·6 (0·4–0·9)	62·4% (44·5 to 81%)	0·7 (0·5–1)	0·8% (-5 to 7·6%)	1·03
	United Arab Emirates	0·1 (0–0·1)	496·2% (395·1 to 607%)	0·7 (0·5–0·9)	-0·1% (-0·6 to 0·4%)	1·00
	Yemen	0·2 (0·1–0·3)	140·3% (125·8 to 156·2%)	0·7 (0·5–1)	0·3% (0 to 0·5%)	1·00

Table S11 Cause- and sex-specific burden, mortality, incidence, and prevalence of neurological conditions in North Africa and Middle East countries

		All Ages		Age-standardised		
		Number (thousand)	Percent change	Rate per 100,000	Percent change	Sex ratio
**Measure**		2019	From 1990 to 2019	2019	From 1990 to 2019	2019
Cause	Location	Mean (95% UI)	Mean (95% UI)	Mean (95% UI)	Mean (95% UI)	F:M
	Afghanistan	0·2 (0·1–0·3)	207·4% (183·1 to 228·1%)	0·7 (0·5–1)	-0·3% (-0·8 to 0·2%)	1·00
	Sudan	0·2 (0·2–0·3)	106·7% (99·4 to 114·7%)	0·7 (0·5–1)	0% (-0·1 to 0·2%)	1·00
Headache disorders	Global	2602898·1 (2396738·3–2805119·6)	54% (51·5 to 56·5%)	32716·8 (30148·4–35335)	0·2% (-0·3 to 0·8%)	1·2
	North Africa and Middle East	205280·4 (186994·5–223750·1)	100·9% (95·4 to 106·3%)	33389·7 (30530·9–36328·1)	1·3% (0·5 to 2·2%)	1·19
	Algeria	13831·1 (12549·8–15188·5)	85·9% (78·2 to 93·4%)	32788·5 (29733·8–35917·9)	0·1% (-0·4 to 0·7%)	1·19
	Bahrain	517·8 (467–570·9)	221·6% (205 to 238·3%)	32232·2 (29237–35273·3)	-0·1% (-0·6 to 0·5%)	1·19
	Egypt	33951·1 (31033·2–36963)	96·5% (89·3 to 104·5%)	35022·4 (32158–37899·9)	2·7% (-0·8 to 6·7%)	1·21
	Iran	32958·4 (30482·4–35496·7)	81% (72·7 to 89·6%)	37542·1 (34654·1–40354·8)	5·4% (3·5 to 7·5%)	1·15
	Iraq	13572·5 (12216·2–14975·5)	175·3% (169·1 to 181·8%)	32763·7 (29727·1–35826·9)	0·2% (-0·3 to 0·7%)	1·19
	Jordan	3770·8 (3396·2–4150·4)	247·8% (237·5 to 258·9%)	32603·8 (29560·1–35676·2)	0% (-0·6 to 0·5%)	1·19
	Kuwait	1571·2 (1423·1–1732·6)	179·9% (167·6 to 192·2%)	32425·7 (29466·2–35506·5)	0·8% (-1·8 to 3·3%)	1·2
	Lebanon	1724·5 (1566·6–1888·7)	75·3% (70·5 to 80·2%)	32916·6 (29885·3–36014·1)	0·4% (-0·1 to 0·9%)	1·19
	Libya	2382·1 (2156·1–2608·3)	96·7% (87·5 to 105·6%)	32752·8 (29720–35810·4)	0·8% (0·3 to 1·3%)	1·19
	Morocco	12136·8 (10998·9–13271·3)	57·9% (53·2 to 62·5%)	32787·8 (29774–35877·7)	0·1% (-0·4 to 0·6%)	1·19
	Palestine	1536 (1378–1697)	173·5% (168·3 to 178·6%)	32770·6 (29756·4–35868·7)	-0·1% (-0·7 to 0·4%)	1·19
	Oman	1560 (1397·9–1741·2)	180·6% (168·5 to 193·2%)	32016·9 (29024·5–35062·5)	-0·1% (-0·8 to 0·5%)	1·19
	Qatar	1020·5 (913·9–1140·9)	609·4% (592·3 to 625·7%)	31367·7 (28394·9–34336·2)	-0·8% (-1·5 to -0·2%)	1·19
	Saudi Arabia	12076·9 (10926·9–13362)	166·6% (153·2 to 180%)	30759·7 (27871·4–33712·7)	-1·5% (-4 to 1·2%)	1·16
	Syrian Arab Republic	4919 (4454·6–5399·6)	37·1% (32 to 42·2%)	32983·9 (29977·1–36072·6)	1% (0·4 to 1·5%)	1·19
	Tunisia	3985·5 (3630·6–4359·6)	54·5% (48·3 to 60·4%)	32857·4 (29808·3–35970·3)	0·4% (-0·1 to 0·9%)	1·19
	Türkiye	27316·9 (24809–29912·1)	56·1% (49·3 to 62·8%)	31032·8 (28267·3–33987·3)	0·7% (-1·8 to 3·4%)	1·25
	United Arab Emirates	3386·5 (3025·4–3794)	477·2% (443·3 to 509%)	31755·5 (28744·2–34841·8)	0% (-0·6 to 0·6%)	1·19
	Yemen	9474·7 (8476·3–10502·6)	163·9% (159·2 to 168·7%)	32765·4 (29726·4–35887·4)	0·2% (-0·3 to 0·7%)	1·19
	Afghanistan	10870·8 (9667·2–12101·4)	236·4% (229·2 to 243%)	32717·2 (29722·6–35827·7)	-0·4% (-0·9 to 0·2%)	1·19
	Sudan	12508·7 (11209·3–13860·2)	119·5% (117·3 to 122%)	32741·2 (29701·9–35840·2)	0·1% (-0·5 to 0·6%)	1·19
**YLDs (Years Lived with Disability)**						
Meningitis	Global	683·3 (480·9–924·3)	-19·2% (-22·5 to -15·4%)	9 (6·3–12·2)	-41·2% (-43·6 to -38·4%)	1·02
	North Africa and Middle East	36·2 (25·1–48·7)	17·1% (9·2 to 26·8%)	6 (4·2–8)	-34·6% (-39 to -29·4%)	1·16
	Algeria	2·2 (1·5–3)	32·6% (17·8 to 48%)	5·4 (3·7–7·3)	-20·4% (-29·4 to -11·2%)	1·03
	Bahrain	0·1 (0–0·1)	69·2% (45·3 to 101·3%)	3·9 (2·7–5·4)	-44·7% (-51·7 to -35·7%)	0·85
	Egypt	6·3 (4·3–8·6)	24·3% (7·7 to 42·2%)	6·3 (4·3–8·6)	-29·9% (-39 to -20·2%)	1·02
	Iran	5·9 (4·1–7·9)	4·6% (-5·1 to 15·9%)	7 (4·9–9·3)	-28·1% (-34 to -21·5%)	2·07

Table S11 Cause- and sex-specific burden, mortality, incidence, and prevalence of neurological conditions in North Africa and Middle East countries

		All Ages		Age-standardised		
		Number (thousand)	Percent change	Rate per 100,000	Percent change	Sex ratio
**Measure**		2019	From 1990 to 2019	2019	From 1990 to 2019	2019
Cause	Location	Mean (95% UI)	Mean (95% UI)	Mean (95% UI)	Mean (95% UI)	F:M
	Iraq	2·5 (1·7–3·3)	67·1% (49·1 to 87·9%)	5·8 (4–7·8)	-30·1% (-37·7 to -21·7%)	1·04
	Jordan	1 (0·7–1·4)	178·2% (129·5 to 241·1%)	8·6 (5·8–12)	-11·4% (-25·3 to 7·1%)	1·03
	Kuwait	0·2 (0·1–0·2)	109·8% (85·4 to 135·6%)	3·6 (2·5–4·9)	-19·5% (-29 to -9·2%)	1·14
	Lebanon	0·3 (0·2–0·4)	9·3% (-5·5 to 26·6%)	5·7 (3·9–7·8)	-31·5% (-40·5 to -21·3%)	1·07
	Libya	0·3 (0·2–0·5)	57·8% (41 to 77·2%)	5 (3·5–6·8)	-5·1% (-14·7 to 5·9%)	1·09
	Morocco	2·4 (1·7–3·3)	-18·3% (-30·3 to -2·8%)	6·8 (4·7–9·3)	-42·1% (-49·9 to -32%)	0·94
	Palestine	0·4 (0·3–0·5)	39·3% (21·3 to 59·7%)	7·6 (5·2–10·4)	-42% (-49 to -33·9%)	1·04
	Oman	0·1 (0–0·1)	59·2% (39·1 to 81·2%)	1·6 (1·1–2·2)	-32·6% (-40·7 to -23·8%)	0·81
	Qatar	0·2 (0·2–0·3)	433·6% (314·1 to 580·1%)	8 (5·4–10·9)	-22·2% (-36·6 to -5·3%)	0·96
	Saudi Arabia	1 (0·7–1·4)	80·9% (59·7 to 105·7%)	2·9 (2–4)	-25·2% (-34·1 to -15·2%)	1·07
	Syrian Arab Republic	1·2 (0·8–1·6)	-30·8% (-40·3 to -19·4%)	8·2 (5·6–11·2)	-38·4% (-46·4 to -29·7%)	1·00
	Tunisia	0·8 (0·5–1)	6·7% (-6·7 to 24·3%)	6·5 (4·5–9·1)	-25% (-33·6 to -12·7%)	1·18
	Türkiye	1·2 (0·8–1·7)	-49·8% (-56·7 to -42%)	1·5 (1–2·1)	-64·1% (-69 to -58·5%)	0·78
	United Arab Emirates	0·3 (0·2–0·4)	333·9% (292·3 to 376·9%)	3·6 (2·5–4·9)	-16·1% (-24·9 to -6·1%)	1·12
	Yemen	2·3 (1·6–3·2)	86·1% (63·1 to 112·3%)	7·4 (5·2–10·2)	-20% (-29·5 to -9·3%)	1·00
	Afghanistan	5·8 (4·1–7·8)	108·3% (79·6 to 142·8%)	19·1 (13·9–25·3)	-33·2% (-42 to -22·8%)	1·13
	Sudan	1·6 (1·1–2·3)	-47·7% (-55 to -38·7%)	4·1 (2·8–5·5)	-74·4% (-77·6 to -70·4%)	0·96
Encephalitis	Global	482·4 (343·3–647·3)	5·2% (0·7 to 9·8%)	6·1 (4·4–8·3)	-28·8% (-31·6 to -25·6%)	1·03
	North Africa and Middle East	19·8 (13·8–26·8)	84·9% (76·6 to 93·9%)	3·2 (2·2–4·3)	-4·1% (-8·1 to 0·4%)	1·35
	Algeria	1·2 (0·8–1·6)	94·5% (76·8 to 113·5%)	2·7 (1·9–3·7)	8·6% (-1·1 to 18·5%)	1·24
	Bahrain	<0·1 (<0·1–<0·1)	187·6% (156·4 to 223·9%)	1·9 (1·3–2·6)	-7·3% (-17 to 4·6%)	1·24
	Egypt	3·2 (2·2–4·4)	62·8% (49 to 78·3%)	3·2 (2·2–4·4)	-13·2% (-20·2 to -5·3%)	1·24
	Iran	2·4 (1·7–3·3)	47·4% (37·4 to 57·9%)	2·8 (1·9–3·8)	-8·6% (-14·1 to -3·1%)	1·33
	Iraq	1·3 (0·9–1·8)	149·5% (130·6 to 169·4%)	3·2 (2·2–4·3)	-4·5% (-11·4 to 2·7%)	1·37
	Jordan	0·4 (0·2–0·5)	197·7% (169·8 to 217·4%)	3·1 (2·1–4·2)	-9·9% (-17·7 to -4%)	1·26
	Kuwait	0·1 (0·1–0·1)	178·7% (147·6 to 207·1%)	1·7 (1·2–2·4)	1·8% (-9 to 12·2%)	1·33
	Lebanon	0·2 (0·1–0·3)	40·1% (25·7 to 54·9%)	3·7 (2·6–5·1)	-17·9% (-26·4 to -8·5%)	1·58
	Libya	0·2 (0·1–0·2)	153·6% (135·9 to 178·4%)	2·2 (1·5–2·9)	39·6% (30·6 to 51·4%)	1·24
	Morocco	1·2 (0·8–1·6)	47·7% (36·5 to 60·2%)	3·1 (2·2–4·3)	-3·2% (-10·4 to 4·8%)	1·32
	Palestine	0·2 (0·1–0·3)	153·5% (127 to 181·8%)	4·2 (2·9–5·8)	-3·1% (-12·4 to 6·9%)	1·22
	Oman	0·1 (0·1–0·2)	112·6% (90·4 to 136·2%)	3 (2·1–4·1)	-17·2% (-25·6 to -8·2%)	1·05
	Qatar	<0·1 (<0·1–0·1)	540·5% (486·5 to 583·1%)	1·4 (0·9–1·9)	-6% (-13·9 to 1·1%)	1·26
	Saudi Arabia	0·7 (0·5–0·9)	140·7% (117·8 to 165%)	1·8 (1·3–2·5)	-7·6% (-16·3 to 1·9%)	1·32
	Syrian Arab Republic	0·6 (0·4–0·8)	16·4% (8 to 25·4%)	3·7 (2·6–5·1)	-7·5% (-13·3 to -0·9%)	1·26
	Tunisia	0·4 (0·3–0·5)	36·2% (23·4 to 51%)	3·1 (2·1–4·2)	-10·5% (-18·6 to -1·5%)	1·33
	Türkiye	2·7 (1·8–3·7)	35·4% (19 to 50·4%)	3·1 (2·1–4·3)	-10·6% (-21·2 to -0·5%)	1·34
	United Arab Emirates	0·2 (0·1–0·2)	591·3% (545·5 to 639·8%)	1·6 (1·1–2·2)	28·8% (19·3 to 38%)	1·28
	Yemen	1·1 (0·7–1·5)	187·7% (163·7 to 215·1%)	3·5 (2·5–4·8)	16·7% (8·3 to 26·2%)	1·32
	Afghanistan	2·5 (1·7–3·3)	288·8% (253·9 to 328·3%)	7·5 (5·4–10·1)	12·6% (2·4 to 23·8%)	1·58
	Sudan	1·3 (0·9–1·8)	114·1% (95·8 to 132·8%)	3·3 (2·3–4·5)	0·2% (-7·7 to 8·4%)	1·27
Tetanus	Global	1·7 (1–2·7)	-76·5% (-81·8 to -71%)	<0·1 (<0·1–<0·1)	-81·7% (-85·3 to -77·9%)	0·94
	North Africa and Middle East	<0·1 (<0·1–<0·1)	-70·2% (-81·9 to -50·5%)	<0·1 (<0·1–<0·1)	-80·9% (-87·4 to -70·7%)	0·71
	Algeria	<0·1 (<0·1–<0·1)	-46·5% (-76·6 to -2·4%)	<0·1 (<0·1–<0·1)	-65·9% (-84·6 to -41·1%)	0·7
	Bahrain	<0·1 (<0·1–<0·1)	136·8% (87 to 169·8%)	<0·1 (<0·1–<0·1)	-11·7% (-35·5 to 0%)	0·77
	Egypt	<0·1 (<0·1–<0·1)	-65·9% (-81·4 to -38·2%)	<0·1 (<0·1–<0·1)	-79·4% (-88 to -61·9%)	0·47
	Iran	<0·1 (<0·1–<0·1)	-79% (-90 to -59·4%)	<0·1 (<0·1–<0·1)	-82·7% (-90·7 to -70·6%)	0·62
	Iraq	<0·1 (<0·1–<0·1)	-64·2% (-85·1 to -12·2%)	<0·1 (<0·1–<0·1)	-78·7% (-89·9 to -58·3%)	0·45
	Jordan	<0·1 (<0·1–<0·1)	11·7% (-38·3 to 76·3%)	<0·1 (<0·1–<0·1)	-54·7% (-72·7 to -35·8%)	0·63
	Kuwait	<0·1 (<0·1–<0·1)	123% (90·9 to 149·1%)	<0·1 (<0·1–<0·1)	-9·2% (-21·4 to 1·3%)	0·91

Table S11 Cause- and sex-specific burden, mortality, incidence, and prevalence of neurological conditions in North Africa and Middle East countries

		All Ages		Age-standardised		
		Number (thousand)	Percent change	Rate per 100,000	Percent change	Sex ratio
**Measure**		2019	From 1990 to 2019	2019	From 1990 to 2019	2019
Cause	Location	Mean (95% UI)	Mean (95% UI)	Mean (95% UI)	Mean (95% UI)	F:M
	Lebanon	<0·1 (<0·1–<0·1)	-48·7% (-87·9 to 58·3%)	<0·1 (<0·1–<0·1)	-77·4% (-94·2 to -30·2%)	0·76
	Libya	<0·1 (<0·1–<0·1)	-24·6% (-59·7 to 14·3%)	<0·1 (<0·1–<0·1)	-46·2% (-73·2 to -18·6%)	0·74
	Morocco	<0·1 (<0·1–<0·1)	-81·5% (-91·7 to -62·2%)	<0·1 (<0·1–<0·1)	-82% (-91·4 to -65·1%)	0·74
	Palestine	<0·1 (<0·1–<0·1)	-58·1% (-87·2 to 12·5%)	<0·1 (<0·1–<0·1)	-72·5% (-90·1 to -44·6%)	0·64
	Oman	<0·1 (<0·1–<0·1)	-25·8% (-69·3 to 53·4%)	<0·1 (<0·1–<0·1)	-78·1% (-93·8 to -34·9%)	0·69
	Qatar	<0·1 (<0·1–<0·1)	322·8% (141·9 to 430%)	<0·1 (<0·1–<0·1)	-27·4% (-63·8 to -3·7%)	1·62
	Saudi Arabia	<0·1 (<0·1–<0·1)	-76% (-93·8 to -17·7%)	<0·1 (<0·1–<0·1)	-87·9% (-96·6 to -59·1%)	0·76
	Syrian Arab Republic	<0·1 (<0·1–<0·1)	-87·9% (-94·9 to -69·9%)	<0·1 (<0·1–<0·1)	-83·4% (-92·1 to -67·4%)	0·7
	Tunisia	<0·1 (<0·1–<0·1)	-45·6% (-75·1 to -9·6%)	<0·1 (<0·1–<0·1)	-56·9% (-80·3 to -30·5%)	0·75
	Türkiye	<0·1 (<0·1–<0·1)	-87·3% (-94 to -72·3%)	<0·1 (<0·1–<0·1)	-92·6% (-96·6 to -81·5%)	0·82
	United Arab Emirates	<0·1 (<0·1–<0·1)	19·4% (-65·4 to 265·1%)	<0·1 (<0·1–<0·1)	-83% (-95 to -29·7%)	1·71
	Yemen	<0·1 (<0·1–<0·1)	-64·9% (-87·5 to 3·4%)	<0·1 (<0·1–<0·1)	-77·7% (-91·4 to -48·7%)	0·4
	Afghanistan	<0·1 (<0·1–<0·1)	-59·9% (-78·4 to -18·6%)	<0·1 (<0·1–0·1)	-82·7% (-89·1 to -70·5%)	0·94
	Sudan	<0·1 (<0·1–<0·1)	-76·4% (-90·3 to -41·6%)	<0·1 (<0·1–<0·1)	-83·6% (-92·6 to -68·2%)	0·41
Brain and central nervous system cancer	Global	129·4 (83·9–175)	118·1% (34·2 to 156·9%)	1·6 (1·1–2·2)	29·5% (-18·9 to 52%)	0·86
	North Africa and Middle East	10·6 (6·2–15)	203·1% (78·6 to 311·3%)	1·9 (1·1–2·7)	55·6% (-2·6 to 105·3%)	0·78
	Algeria	0·4 (0·2–0·6)	183·7% (52·9 to 298·7%)	0·9 (0·5–1·4)	59% (-14·1 to 120·7%)	0·89
	Bahrain	<0·1 (<0·1–<0·1)	510·4% (260·3 to 799·8%)	1·5 (0·9–2·2)	50·6% (-10·4 to 121·5%)	0·72
	Egypt	1 (0·6–1·6)	164·1% (48 to 286·3%)	1·2 (0·7–1·8)	39·2% (-12·6 to 102%)	0·78
	Iran	2·4 (1·1–3·5)	174% (49·3 to 259·1%)	2·9 (1·3–4·3)	62·6% (-5·7 to 104·8%)	0·81
	Iraq	1 (0·6–1·5)	318·3% (111·4 to 590·4%)	2·9 (1·8–4·2)	64·4% (-13·6 to 160·6%)	0·95
	Jordan	0·2 (0·1–0·3)	509% (285·8 to 731·1%)	1·7 (1·1–2·5)	62·9% (0·1 to 124·2%)	0·81
	Kuwait	0·1 (0–0·1)	380·1% (193·4 to 556·2%)	1·9 (1·2–2·9)	81·7% (11·4 to 148·3%)	0·55
	Lebanon	0·2 (0·1–0·2)	318·6% (176·1 to 489%)	3·1 (1·9–4·6)	128·8% (50·9 to 223·1%)	0·69
	Libya	0·1 (0·1–0·2)	154·9% (69·8 to 287·6%)	1·9 (1·2–2·7)	33·2% (-5·8 to 94·8%)	0·58
	Morocco	0·2 (0·1–0·3)	123·8% (33·3 to 228·5%)	0·6 (0·4–0·9)	36·2% (-14 to 92·3%)	1·26
	Palestine	0·1 (0·1–0·2)	216·7% (92·4 to 357·9%)	3·2 (2–4·3)	25·1% (-19·5 to 76·2%)	0·74
	Oman	0·1 (0–0·1)	514·4% (193·5 to 920·7%)	1·6 (0·9–2·4)	130·8% (13·7 to 260·4%)	0·82
	Qatar	<0·1 (<0·1–0·1)	1494·2% (769·7 to 2457·2%)	2·4 (1·5–3·8)	97·1% (16·7 to 204·4%)	1·00
	Saudi Arabia	0·6 (0·4–1)	932·3% (436 to 1905·1%)	1·8 (1·2–2·9)	237·6% (77·8 to 536·9%)	0·96
	Syrian Arab Republic	0·3 (0·2–0·4)	122·3% (34·4 to 241%)	2·1 (1·4–3·2)	42·3% (-4·4 to 112·2%)	0·76
	Tunisia	0·1 (0·1–0·2)	187·5% (71·7 to 334·5%)	0·8 (0·5–1·3)	83·1% (10·5 to 174·5%)	1·05
	Türkiye	2·5 (1·1–3·9)	178·5% (50·6 to 349·9%)	3 (1·3–4·7)	67·7% (-3·5 to 160·9%)	0·69
	United Arab Emirates	0·2 (0·1–0·3)	736·5% (427·5 to 1139·8%)	2·1 (1·1–3·2)	25·8% (-25·8 to 91·7%)	0·75
	Yemen	0·3 (0·1–0·4)	191·3% (55 to 447·4%)	1·2 (0·7–1·9)	32·2% (-24·3 to 118·2%)	0·71
	Afghanistan	0·4 (0·2–0·7)	158·9% (67·4 to 354·3%)	1·5 (0·8–2·9)	2·9% (-32·8 to 63·2%)	0·77
	Sudan	0·5 (0·2–0·7)	131·1% (10·5 to 404%)	1·4 (0·8–2·1)	25·8% (-31·3 to 129·2%)	0·67
Stroke*	Global	17741·7 (12759·4–22587·4)	88·9% (85·3 to 92·7%)	218·1 (156·7–277)	-4·7% (-6·1 to -3·3%)	1·3
	North Africa and Middle East	1113·7 (812·1–1400·8)	140·2% (135·3 to 145·3%)	239·4 (176·1–301·4)	-0·7% (-2·6 to 1·2%)	1·43
	Algeria	85 (62·2–107·5)	128·1% (116·1 to 140·6%)	242·8 (178·6–307·3)	-10·6% (-14·9 to -6%)	1·44
	Bahrain	2·1 (1·5–2·6)	275·8% (251·4 to 302·4%)	173·8 (126–219·2)	-25·9% (-30 to -21·8%)	1·56
	Egypt	193·9 (141·7–245·2)	149·5% (136·2 to 163%)	282·6 (206·4–356·5)	18% (11·8 to 24·2%)	1·57
	Iran	148 (106·3–188·6)	125·2% (116·8 to 134%)	196·4 (140·3–251·9)	-12·7% (-15·5 to -10%)	1·34
	Iraq	78·4 (56·7–98·6)	167% (154·2 to 181·1%)	306·4 (223·5–385·2)	-8·7% (-13·3 to -3·7%)	1·39
	Jordan	20·5 (14·9–25·8)	256·2% (235·6 to 277·3%)	284·5 (207·6–358·1)	-23·7% (-28·5 to -19·2%)	1·47
	Kuwait	6·3 (4·5–8)	262·6% (237·2 to 288·5%)	189·4 (138·2–238)	-6·8% (-12·1 to -1·3%)	1·2
	Lebanon	11·8 (8·7–14·8)	127·4% (116 to 140·3%)	224·6 (165·6–281·1)	1·8% (-2·8 to 7·2%)	1·62
	Libya	13·7 (10–17·2)	193% (177·2 to 209·7%)	248·1 (179·9–311)	12·8% (6·9 to 18·8%)	1·85

Table S11 Cause- and sex-specific burden, mortality, incidence, and prevalence of neurological conditions in North Africa and Middle East countries

		**All Ages**		**Age-standardised**		
		Number (thousand)	Percent change	Rate per 100,000	Percent change	Sex ratio
**Measure**		2019	From 1990 to 2019	2019	From 1990 to 2019	2019
Cause	Location	Mean (95% UI)	Mean (95% UI)	Mean (95% UI)	Mean (95% UI)	F:M
	Morocco	85·2 (62·1–106·7)	119·2% (108·3 to 131·5%)	265·1 (194·2–333)	3·4% (-1·4 to 9·1%)	1·53
	Palestine	6·3 (4·6–8)	161% (146·3 to 176·7%)	238 (173·6–302·4)	-3·9% (-9·4 to 1·6%)	1·54
	Oman	5·5 (3·9–7·1)	162% (145·6 to 179%)	237·2 (174·1–301·6)	-8·1% (-12·8 to -3%)	1·27
	Qatar	3·1 (2·2–4)	555·1% (505·9 to 612·7%)	182·2 (132·5–228·2)	-24·2% (-28·2 to -19·9%)	1·5
	Saudi Arabia	68·9 (49–87·5)	226·9% (200·7 to 255·2%)	299·4 (218·9–378·4)	7·2% (-1·5 to 15·6%)	1·55
	Syrian Arab Republic	30 (22–38)	77·7% (68·4 to 86·5%)	234·1 (172·3–295·5)	-13·7% (-17·7 to -9·6%)	1·33
	Tunisia	24·9 (18·4–31·3)	180·2% (164·4 to 196·5%)	199·4 (146·5–250·1)	21·2% (14·7 to 27·8%)	1·39
	Türkiye	168 (122·3–211·3)	104·2% (93·2 to 116%)	191·2 (139·3–239·6)	-8·6% (-13·2 to -3·6%)	1·39
	United Arab Emirates	18·8 (13·5–24·2)	686·9% (628·8 to 746·8%)	336·4 (245·1–425·6)	-10·2% (-14·8 to -5·7%)	1·41
	Yemen	39·8 (29–50·1)	175·4% (160·3 to 191·5%)	249·6 (183·1–315·4)	1·2% (-4·2 to 7%)	1·43
	Afghanistan	41·5 (30·3–52)	127·5% (113·7 to 141·6%)	248·5 (183·7–312)	3·4% (-2·1 to 8·1%)	1·5
	Sudan	60·6 (43·9–76·3)	122·8% (111·4 to 134·4%)	276·5 (201·3–351·5)	8·2% (2·6 to 13·3%)	1·54
Ischaemic stroke	Global	13128·5 (9349·9–16930·4)	102% (97·4 to 106·9%)	162·3 (115·8–209·9)	0·1% (-1·8 to 2%)	1·36
	North Africa and Middle East	877·3 (639·8–1114·1)	157·6% (151·5 to 164·4%)	195·6 (144·4–249·1)	5·2% (2·8 to 7·6%)	1·45
	Algeria	69·8 (50·8–89·3)	151·1% (135·5 to 167·4%)	204·2 (149·2–261·1)	-3·1% (-8·5 to 2·6%)	1·45
	Bahrain	1·5 (1·1–2)	287·4% (256·8 to 320·9%)	139·9 (101·5–178·9)	-25·5% (-30·1 to -20·7%)	1·56
	Egypt	159 (115·8–203·1)	179·1% (161·3 to 197·2%)	239·8 (175·1–307·5)	30% (22·2 to 37·9%)	1·58
	Iran	124·9 (88·9–160·3)	130% (120·3 to 141·2%)	169·1 (121·1–219·4)	-12·7% (-15·8 to -9·5%)	1·35
	Iraq	60·3 (43·3–76·4)	169·8% (154·6 to 186·7%)	249·5 (181·9–315·5)	-6·3% (-11·5 to -0·1%)	1·48
	Jordan	16·7 (12–21·1)	258·7% (234·4 to 283·5%)	243 (176·8–308·3)	-23·7% (-29·1 to -18·5%)	1·48
	Kuwait	4·7 (3·4–5·9)	265·9% (237·1 to 297·9%)	153·2 (111·1–193·1)	-7·5% (-13·9 to -1·4%)	1·19
	Lebanon	10 (7·3–12·6)	146·5% (132·8 to 161·4%)	190·1 (140–240·3)	7·8% (1·9 to 14·1%)	1·62
	Libya	11·2 (8·1–14·2)	220% (199·6 to 240·5%)	209·8 (152·5–264·3)	21·6% (14·6 to 28·9%)	1·89
	Morocco	70·2 (50·8–88·4)	143·8% (129·5 to 159·4%)	223 (161·5–281)	12·6% (6·4 to 19·5%)	1·56
	Palestine	5·1 (3·7–6·5)	163·5% (146·1 to 183·8%)	203·2 (147·6–260·1)	-0·9% (-7·4 to 5·9%)	1·56
	Oman	4·2 (3–5·4)	173% (151·6 to 194·5%)	197·7 (145·4–253·1)	-2·6% (-8·7 to 3·8%)	1·18
	Qatar	2·1 (1·5–2·7)	577·8% (513·3 to 654·7%)	143·4 (105–182·3)	-23·4% (-28·4 to -18·4%)	1·51
	Saudi Arabia	44·8 (32–57·5)	240·3% (202·3 to 280·5%)	222 (162·1–281·5)	12·5% (0·7 to 24·5%)	1·59
	Syrian Arab Republic	22·8 (16·7–29)	94·5% (82·4 to 106·8%)	182·8 (133·9–233·7)	-8·8% (-13·6 to -3·7%)	1·39
	Tunisia	21·3 (15·5–26·9)	194·8% (176·7 to 214·1%)	171·2 (125·8–216·1)	25·1% (18 to 33%)	1·37
	Türkiye	126·6 (92·7–160·6)	117·6% (102·4 to 133·2%)	145·8 (106·5–184)	-5·7% (-11·7 to 0·5%)	1·4
	United Arab Emirates	14·4 (10·4–18·7)	754·8% (678·1 to 839·9%)	289·4 (210–368)	-5·9% (-11·6 to -0·7%)	1·42
	Yemen	30·5 (22·1–38·8)	216·5% (196·9 to 238·2%)	202·9 (148·1–258·8)	15·1% (7·9 to 22·6%)	1·47
	Afghanistan	29 (21–37)	142·9% (123·4 to 161·9%)	189·7 (139·8–240·7)	17·3% (9·8 to 23·9%)	1·48
	Sudan	47·3 (33·9–60·2)	150·9% (135·3 to 167·7%)	226·9 (164·1–290·5)	22·6% (15·5 to 30·3%)	1·57
Intracerebral haemorrhage	Global	3266·3 (2334–4173·7)	57·2% (54·9 to 59·5%)	39·4 (28·1–50·2)	-17·7% (-19·2 to -16·1%)	0·99
	North Africa and Middle East	182·2 (129·1–229·6)	88·9% (82·6 to 95·8%)	34·4 (24·4–43·5)	-21·5% (-24·2 to -18·4%)	1·26
	Algeria	11·2 (7·8–14·4)	50·3% (35·3 to 65·8%)	29·2 (20·5–37·5)	-39·8% (-45·5 to -34%)	1·33
	Bahrain	0·4 (0·3–0·5)	217·3% (175 to 264·1%)	26·2 (18·1–34)	-32·1% (-39·1 to -24·6%)	1·51
	Egypt	27 (19·1–34·6)	68·2% (50·9 to 86·9%)	33·8 (24·2–43·2)	-22·2% (-29·7 to -14%)	1·39
	Iran	16·5 (11·5–21·1)	93% (84 to 102·4%)	19·9 (14·1–25·6)	-15·1% (-19·1 to -10·9%)	1·17
	Iraq	15·1 (10·6–19·3)	161·7% (136·8 to 188·3%)	48·4 (34·2–61·7)	-16·2% (-23·4 to -8·3%)	0·97
	Jordan	2·7 (1·9–3·6)	216·8% (181·1 to 254·4%)	30·7 (21·8–39·7)	-29·4% (-36·7 to -21·5%)	1·25
	Kuwait	1·1 (0·7–1·4)	239·5% (187 to 294·7%)	25·9 (18·2–33·8)	-6·3% (-17·3 to 4·8%)	1·09
	Lebanon	1·3 (0·9–1·7)	52·5% (36 to 70·8%)	24·7 (17·4–31·6)	-25·7% (-33·5 to -17%)	1·53
	Libya	1·9 (1·3–2·4)	103·6% (80·7 to 127·9%)	29 (20·5–37·8)	-21·7% (-29·4 to -13·3%)	1·58
	Morocco	11·5 (8·1–14·7)	46·4% (32·2 to 61·3%)	32·6 (23–41·7)	-29·1% (-35·7 to -22·3%)	1·32
	Palestine	0·9 (0·6–1·1)	134% (107·2 to 161·3%)	25·4 (17·9–32·8)	-22% (-30·1 to -12·9%)	1·32

Table S11 Cause- and sex-specific burden, mortality, incidence, and prevalence of neurological conditions in North Africa and Middle East countries

		All Ages		Age-standardised		
		Number (thousand)	Percent change	Rate per 100,000	Percent change	Sex ratio
**Measure**		2019	From 1990 to 2019	2019	From 1990 to 2019	2019
Cause	Location	Mean (95% UI)	Mean (95% UI)	Mean (95% UI)	Mean (95% UI)	F:M
	Oman	1 (0·7–1·3)	120·8% (94·1 to 152·2%)	31·1 (22·2–40)	-30·2% (-36·7 to -22·6%)	1·84
	Qatar	0·7 (0·5–0·9)	486·1% (398·9 to 596·6%)	28·6 (20·2–36·5)	-30% (-37·3 to -22·4%)	1·39
	Saudi Arabia	20·8 (14·3–27·6)	198·4% (164·8 to 235·6%)	68·9 (48·4–89·2)	-5·7% (-13·5 to 3·3%)	1·39
	Syrian Arab Republic	5·9 (4·1–7·5)	35·3% (22·2 to 49·3%)	42·1 (29·6–53·2)	-29·9% (-36·5 to -23·2%)	1·06
	Tunisia	2·4 (1·7–3·2)	117·3% (89·7 to 151%)	18·6 (12·9–24·6)	1% (-11·9 to 16·4%)	1·33
	Türkiye	30·8 (21·6–39·3)	74·8% (56·5 to 93·9%)	34·2 (24·1–43·7)	-15·9% (-24·3 to -6·6%)	1·27
	United Arab Emirates	3·2 (2·3–4·2)	492·2% (407·8 to 599·6%)	38·7 (27·7–49·6)	-32% (-37·9 to -25·2%)	1·26
	Yemen	7·1 (5·1–9·1)	82% (63·7 to 101·4%)	36·7 (26·3–46·8)	-37·2% (-42·9 to -30·4%)	1·21
	Afghanistan	10·2 (7·3–13)	87·6% (68·5 to 109·1%)	49·1 (35·3–62·2)	-27·6% (-33·8 to -21·2%)	1·54
	Sudan	10·5 (7·4–13·5)	53·2% (37·9 to 69%)	39·7 (28·1–50·3)	-32·1% (-38·3 to -25·6%)	1·35
Subarachnoid haemorrhage	Global	1346·9 (964·2–1782·2)	65·4% (60·7 to 69·6%)	16·4 (11·7–21·6)	-12·9% (-15·2 to -11·2%)	1·63
	North Africa and Middle East	54·2 (37·9–72·3)	103·1% (83·8 to 116·5%)	9·3 (6·6–12·4)	-17·9% (-26·2 to -12·2%)	1·77
	Algeria	4 (2·7–5·4)	98·4% (53·6 to 135·2%)	9·5 (6·5–13)	-23·7% (-41·8 to -9·3%)	1·67
	Bahrain	0·1 (0·1–0·2)	353·6% (288·7 to 432·3%)	7·7 (5·2–10·4)	-7·2% (-19·9 to 7·6%)	1·74
	Egypt	7·9 (5·4–10·6)	68·8% (40 to 96·8%)	9 (6·1–12·1)	-21·3% (-34·6 to -8·4%)	1·84
	Iran	6·7 (4·7–9)	129·1% (116·4 to 142·7%)	7·3 (5·2–9·9)	-6·6% (-10·8 to -2·3%)	1·75
	Iraq	3 (2·1–4·2)	141·8% (92·3 to 183·1%)	8·6 (5·9–11·8)	-25·3% (-44·6 to -11·8%)	1·81
	Jordan	1·1 (0·8–1·5)	345% (283·5 to 415·2%)	10·8 (7·5–14·5)	-1·4% (-14·7 to 14·6%)	1·7
	Kuwait	0·5 (0·4–0·7)	283·9% (223·4 to 357·4%)	10·3 (7·2–13·9)	3% (-11·2 to 20·2%)	1·85
	Lebanon	0·5 (0·4–0·7)	81·8% (54·2 to 113·8%)	9·9 (6·8–13·6)	-9·9% (-23·7 to 6·3%)	1·81
	Libya	0·7 (0·5–0·9)	145·8% (102·5 to 188·2%)	9·3 (6·4–12·8)	-11% (-27·8 to 4%)	1·78
	Morocco	3·5 (2·4–4·8)	58·3% (30·8 to 84%)	9·6 (6·6–12·8)	-22·8% (-36·1 to -9·7%)	1·73
	Palestine	0·4 (0·2–0·5)	202·8% (161·5 to 251·9%)	9·5 (6·5–12·9)	-5·3% (-18·9 to 11%)	1·83
	Oman	0·4 (0·3–0·5)	176·3% (133·9 to 220%)	8·3 (5·8–11·3)	-19·2% (-32·4 to -5·8%)	1·87
	Qatar	0·3 (0·2–0·4)	571·8% (450·8 to 693·6%)	10·2 (7–13·8)	-17·9% (-36·9 to -2·8%)	1·75
	Saudi Arabia	3·3 (2·3–4·6)	249·5% (196·9 to 308·4%)	8·5 (5·9–11·6)	-2·9% (-16·7 to 12·6%)	1·85
	Syrian Arab Republic	1·4 (0·9–1·9)	62·7% (29·4 to 92·5%)	9·2 (6·4–12·4)	-14·4% (-30·5 to 0·6%)	1·64
	Tunisia	1·3 (0·9–1·7)	118·1% (71·9 to 163·3%)	9·6 (6·6–13)	2·9% (-19·3 to 24·5%)	1·82
	Türkiye	10·6 (7·3–14·4)	63·5% (38·4 to 92·1%)	11·3 (7·8–15·2)	-19·5% (-31·6 to -4·8%)	1·73
	United Arab Emirates	1·1 (0·8–1·6)	634·3% (522·4 to 767·7%)	8·2 (5·7–11·3)	-15·8% (-27 to -3·1%)	1·9
	Yemen	2·2 (1·5–2·9)	140·2% (106·5 to 178·1%)	10 (7–13·5)	-16·6% (-28·6 to -3·4%)	1·78
	Afghanistan	2·3 (1·6–3·1)	166·4% (129·5 to 213·7%)	9·6 (6·7–12·9)	-10·6% (-23·4 to 4·2%)	1·75
	Sudan	2·9 (2–3·9)	87·6% (60·3 to 116·6%)	9·9 (6·9–13·3)	-18·9% (-31·2 to -5·4%)	1·75
Neurological disorders†*	Global	65626 (28649·3–122710)	64·9% (58·3 to 81·4%)	831·3 (365·8–1542·4)	2·9% (-0·7 to 6·8%)	1·43
	North Africa and Middle East	5447·8 (2227·5–10439·2)	99·6% (83·8 to 121·6%)	935 (419·6–1737·7)	0·2% (-6·5 to 8·3%)	1·42
	Algeria	374·2 (157·4–723·9)	84·9% (44 to 148%)	928·2 (416·8–1744·2)	-2·3% (-20 to 19·5%)	1·42
	Bahrain	13·3 (5·2–25·4)	204·4% (128·4 to 304·6%)	923·6 (424·3–1686·2)	-4·4% (-29 to 24·2%)	1·39
	Egypt	852 (300·4–1665·6)	94·8% (48·2 to 146·5%)	931·2 (371·4–1745·9)	2% (-16·8 to 23·4%)	1·44
	Iran	833·3 (360·8–1572·9)	76·6% (61·6 to 100%)	975·8 (451·6–1800)	-0·6% (-6·7 to 6·2%)	1·39
	Iraq	353·6 (132·5–707)	168·9% (104·9 to 239·6%)	913 (394·4–1730·9)	-0·1% (-18·2 to 20·6%)	1·42
	Jordan	97·6 (36·3–192·4)	245·4% (173·6 to 348·2%)	907·9 (394·5–1704·5)	-0·8% (-17·2 to 18·5%)	1·42
	Kuwait	41·2 (15·6–81)	185·4% (126·4 to 264·3%)	916·1 (399·6–1722·5)	1·2% (-18·1 to 24·3%)	1·42
	Lebanon	48·7 (20·4–92·6)	81·3% (48·1 to 132·4%)	931·4 (391–1772·2)	-0·2% (-17·7 to 20·5%)	1·42
	Libya	62·7 (24·3–122·2)	92·6% (53·5 to 140·6%)	904·5 (393·2–1713·4)	-2% (-18·5 to 16·5%)	1·42
	Morocco	327·2 (124·9–641·4)	62·1% (28·2 to 109·9%)	925·7 (383·4–1774·1)	1·1% (-18·1 to 23·1%)	1·42
	Palestine	39·9 (15–78·1)	166·7% (106 to 236·2%)	915·4 (406·2–1702·6)	-0·9% (-17·1 to 18%)	1·42
	Oman	38·1 (13·6–75·2)	175·9% (112·6 to 250·9%)	877·8 (389·3–1634·8)	0·7% (-17·7 to 24·3%)	1·41
	Qatar	24·4 (9·3–47·7)	572% (407·4 to 748·7%)	861·3 (393·5–1543·5)	-3·5% (-24·6 to 22·3%)	1·44

Table S11 Cause- and sex-specific burden, mortality, incidence, and prevalence of neurological conditions in North Africa and Middle East countries

		**All Ages**		**Age-standardised**		
		Number (thousand)	Percent change	Rate per 100,000	Percent change	Sex ratio
**Measure**		2019	From 1990 to 2019	2019	From 1990 to 2019	2019
Cause	Location	Mean (95% UI)	Mean (95% UI)	Mean (95% UI)	Mean (95% UI)	F:M
	Saudi Arabia	338·3 (128·9–653·2)	172·5% (108·9 to 275·5%)	949·3 (419–1737·1)	3·3% (-18·3 to 35·6%)	1·33
	Syrian Arab Republic	128·5 (49–254)	38·9% (11·1 to 84·7%)	916·3 (384·9–1745·3)	1·8% (-14·4 to 23%)	1·44
	Tunisia	109·3 (46·3–207·6)	64·6% (41·2 to 114·7%)	916·1 (393·8–1740)	1·6% (-12·7 to 20·5%)	1·45
	Türkiye	833·8 (374–1538·2)	61·1% (30·1 to 120·6%)	966·5 (437·5–1760)	1% (-18·7 to 29·4%)	1·46
	United Arab Emirates	84·7 (33·2–163·2)	430·1% (298·9 to 626·6%)	917 (419·7–1693·9)	-4·9% (-28·6 to 25·3%)	1·35
	Yemen	238·4 (80·9–479·5)	158·2% (97·3 to 245%)	887·5 (363·5–1694·8)	-1·1% (-17·1 to 19·2%)	1·42
	Afghanistan	279·5 (101·6–561)	224·6% (127·3 to 339·8%)	896·8 (387·4–1711·2)	-2·4% (-22·3 to 23·3%)	1·45
	Sudan	323·5 (113·5–649·9)	118·3% (70·7 to 191·4%)	902·9 (375·3–1718·2)	0·3% (-16 to 24·4%)	1·42
Alzheimer's disease and other dementias	Global	7417·1 (5226·8–9927·7)	164·7% (158·7 to 171%)	98·9 (69·5–132·6)	5·5% (4 to 6·8%)	1·27
	North Africa and Middle East	352·2 (248·1–474·3)	187·7% (180 to 195·6%)	112·8 (79·1–151·9)	2·8% (0·9 to 4·6%)	1·1
	Algeria	27·4 (19·4–37)	244·1% (216·3 to 275·2%)	113·1 (79·6–152·1)	1% (-3·2 to 5·2%)	1·09
	Bahrain	0·5 (0·3–0·7)	440% (403·7 to 480·9%)	113·7 (80·5–151·6)	2·9% (-1 to 7·3%)	1·09
	Egypt	41·2 (29·3–55·5)	119·1% (108·5 to 130%)	110 (76·8–148·3)	4·8% (0·4 to 9·7%)	1·1
	Iran	67·3 (47·3–90·6)	325·9% (297·8 to 357·1%)	113·9 (80–154)	2·2% (0 to 4%)	1·08
	Iraq	16·6 (11·8–22·4)	167·2% (154·7 to 181·3%)	110·5 (78·3–149·5)	6·3% (2·1 to 10·9%)	1·09
	Jordan	4·5 (3·1–6·1)	431% (401·7 to 459·4%)	111·6 (78·2–151·8)	3·7% (-1 to 8·3%)	1·09
	Kuwait	2 (1·4–2·7)	406·6% (380·4 to 434·2%)	116·3 (81·6–156·8)	0·6% (-3·7 to 5·4%)	1·11
	Lebanon	5·7 (4–7·7)	227·4% (206·9 to 246·6%)	113·8 (79·9–152·6)	3·3% (-1·1 to 8·2%)	1·1
	Libya	4·4 (3·1–5·8)	167·3% (155·4 to 180·6%)	109 (76·7–145·9)	-2·1% (-6·2 to 2·7%)	1·09
	Morocco	24·7 (17·4–33·3)	145·7% (134 to 158·8%)	110·9 (77·8–149·4)	-0·2% (-4·4 to 4·5%)	1·08
	Palestine	1·7 (1·2–2·3)	131·4% (119·3 to 143·7%)	111·6 (78·9–150·6)	1·5% (-3·4 to 6·1%)	1·08
	Oman	0·9 (0·6–1·2)	140·1% (127·7 to 155·2%)	113·4 (79·6–152)	0·8% (-3·4 to 5·3%)	1·1
	Qatar	0·3 (0·2–0·5)	637·3% (552·5 to 735·9%)	110·5 (77·2–147·4)	2·8% (-1·9 to 8%)	1·09
	Saudi Arabia	9·1 (6·4–12·3)	128·3% (112·8 to 144·1%)	109·4 (76·6–146·3)	1·2% (-3·3 to 5·8%)	1·11
	Syrian Arab Republic	8·8 (6·2–11·9)	109·5% (95·8 to 123·9%)	110·8 (77·7–149·2)	0·3% (-4 to 5·3%)	1·1
	Tunisia	12·1 (8·5–16·3)	221·5% (202·1 to 243%)	114·9 (80·6–155·1)	1·3% (-2·8 to 5·4%)	1·12
	Türkiye	92·7 (65·1–124·2)	199·6% (185·3 to 212·4%)	116·9 (82–157·2)	3·9% (-0·7 to 8·2%)	1·11
	United Arab Emirates	1 (0·7–1·5)	610·5% (527·9 to 705·4%)	103·7 (72·7–139·5)	-0·4% (-4·8 to 3·8%)	1·09
	Yemen	9·1 (6·4–12·2)	195·2% (180·2 to 211·8%)	109·2 (76·5–146·7)	-2·6% (-7·1 to 2%)	1·08
	Afghanistan	7·5 (5·3–10·2)	54·5% (46·3 to 63·3%)	103·8 (73·3–140·2)	-2·3% (-6·5 to 2·7%)	1·07
	Sudan	14·3 (10·2–19·2)	115% (101·6 to 129·3%)	108·5 (76·5–145·8)	-0·1% (-4·3 to 4·3%)	1·09
Parkinson's disease	Global	1210·1 (841·2–1640·7)	154·7% (149·4 to 160·9%)	15·1 (10·4–20·3)	16·2% (13·5 to 19%)	0·67
	North Africa and Middle East	44·5 (30·7–60·5)	197·3% (182·1 to 214·4%)	11·7 (8–15·7)	14·8% (9·2 to 21·2%)	0·73
	Algeria	3·4 (2·4–4·7)	213·3% (165·9 to 268·6%)	11·4 (7·9–15·5)	9·2% (-6·4 to 25·7%)	0·77
	Bahrain	0·1 (0·1–0·1)	494·3% (387·6 to 617·3%)	13 (8·8–17·7)	11·2% (-7·8 to 34·3%)	0·73
	Egypt	6·4 (4·3–8·9)	147·6% (106·3 to 196·4%)	12 (8·2–16·5)	16·5% (-1·6 to 37·1%)	0·84
	Iran	8·1 (5·5–11·1)	255·1% (228·5 to 282·5%)	12 (8·1–16·6)	13·2% (8·7 to 17·3%)	0·74
	Iraq	2·1 (1·4–2·8)	200% (156·4 to 252·7%)	10·5 (7·3–14·2)	6·8% (-7·7 to 24·8%)	0·67
	Jordan	0·6 (0·4–0·8)	425% (342·9 to 517%)	10·9 (7·5–15·1)	2·8% (-13·4 to 19·7%)	0·78
	Kuwait	0·2 (0·2–0·3)	311·7% (238·6 to 396·9%)	10·1 (6·8–13·9)	-9·2% (-26·3 to 11·5%)	0·72
	Lebanon	0·6 (0·4–0·8)	184% (140·8 to 236·9%)	10·8 (7·5–14·9)	7·8% (-8·5 to 26·4%)	0·75
	Libya	0·5 (0·4–0·8)	210·8% (170 to 263·7%)	11·8 (8–16·3)	12% (-2·8 to 31%)	0·74
	Morocco	3 (2–4·2)	176·2% (132·8 to 232·9%)	10·5 (7·2–14·6)	19·4% (0·8 to 42·1%)	0·69
	Palestine	0·2 (0·1–0·3)	159·2% (115·7 to 204·1%)	11 (7·6–15·1)	1·2% (-13·4 to 18%)	0·69
	Oman	0·2 (0·1–0·3)	214% (165·6 to 267·9%)	15·6 (10·6–21·4)	26·7% (8·5 to 46·6%)	0·63
	Qatar	0·1 (0·1–0·2)	807·3% (650 to 989·8%)	16·4 (11·3–22·5)	18% (0·7 to 36·9%)	0·88
	Saudi Arabia	1·9 (1·3–2·7)	239·4% (184·2 to 304·5%)	14·8 (10·2–20·4)	27·1% (8·4 to 48·9%)	0·6
	Syrian Arab Republic	1·2 (0·8–1·7)	158·5% (118·1 to 207·3%)	11·5 (8–15·9)	10·1% (-6·3 to 30·5%)	0·74

Table S11 Cause- and sex-specific burden, mortality, incidence, and prevalence of neurological conditions in North Africa and Middle East countries

		All Ages		Age-standardised		
		Number (thousand)	Percent change	Rate per 100,000	Percent change	Sex ratio
**Measure**		2019	From 1990 to 2019	2019	From 1990 to 2019	2019
Cause	Location	Mean (95% UI)	Mean (95% UI)	Mean (95% UI)	Mean (95% UI)	F:M
	Tunisia	1·3 (0·9–1·8)	202·6% (153·7 to 262·6%)	11·2 (7·7–15·2)	13·9% (-3·3 to 34·8%)	0·73
	Türkiye	10·3 (7–14·2)	220·9% (175·2 to 275·1%)	12·3 (8·4–16·8)	21·5% (4·3 to 41·8%)	0·71
	United Arab Emirates	0·5 (0·3–0·7)	907·8% (719·4 to 1151·3%)	16·1 (11·3–22·3)	10·9% (-4·2 to 28·2%)	0·78
	Yemen	1 (0·7–1·5)	204·1% (154 to 263·5%)	9 (6·2–12·4)	9·6% (-7·1 to 29·6%)	0·76
	Afghanistan	1·1 (0·7–1·5)	63·8% (37·9 to 94·7%)	9·6 (6·5–13·3)	-4·1% (-17·2 to 11·4%)	0·72
	Sudan	1·7 (1·1–2·3)	102·3% (69 to 143%)	9·9 (6·8–14)	1·8% (-14·6 to 21·1%)	0·7
Idiopathic epilepsy	Global	7740·8 (4810·3–11216·7)	43·7% (18·7 to 76·3%)	101·1 (63·1–146·8)	1·4% (-15·4 to 23·2%)	0·91
	North Africa and Middle East	603·1 (342·3–938·7)	47·5% (-1 to 119%)	101 (57·3–156·5)	-9·3% (-39·8 to 33·8%)	0·87
	Algeria	38·7 (8·7–80)	15·9% (-75·9 to 408·9%)	94·6 (21·2–196·2)	-22·9% (-83·9 to 237%)	0·91
	Bahrain	1·5 (0·4–3·3)	93·9% (-54·7 to 821·7%)	125·4 (29·5–263·3)	-27·4% (-82·5 to 245·2%)	0·92
	Egypt	87·3 (21–178)	54·2% (-65·2 to 647·7%)	86·5 (20·9–177·8)	-9·2% (-79·5 to 337·3%)	0·77
	Iran	79·9 (45·7–122·2)	5·9% (-27·2 to 51·5%)	100 (57·2–152·2)	-14·4% (-41·4 to 21·7%)	0·87
	Iraq	39·7 (8·4–82·1)	95·1% (-53·5 to 838·5%)	92·3 (20–189·2)	-15·6% (-80·5 to 286·1%)	0·91
	Jordan	10·5 (2·4–23·1)	154·2% (-40·2 to 939·2%)	88·5 (20·3–192·5)	-12·8% (-79·2 to 245·1%)	0·89
	Kuwait	4 (1–9)	92·1% (-51·6 to 741·6%)	103·4 (23·9–231)	-15·2% (-78·7 to 270·3%)	0·91
	Lebanon	4·6 (1·1–10)	24·1% (-71·5 to 456%)	91·5 (21·8–198·7)	-16·9% (-81·2 to 270·9%)	0·88
	Libya	5·5 (1·6–10·9)	7·8% (-69·3 to 350·6%)	85·6 (25–167·6)	-26·2% (-78·6 to 201%)	0·86
	Morocco	33·7 (7·3–72·1)	30·9% (-74·5 to 553·2%)	96 (21–203·5)	-0·5% (-80·9 to 388·9%)	0·96
	Palestine	4·7 (1·1–9·8)	108·4% (-54·9 to 925·8%)	92 (22·5–190·6)	-10·2% (-80·5 to 341·2%)	0·92
	Oman	3·9 (0·9–8·3)	107·9% (-53·3 to 851·7%)	92·8 (22·2–199·6)	-0·4% (-77·1 to 347%)	0·89
	Qatar	2·5 (0·6–5·6)	355·7% (9·8 to 1964·2%)	103·9 (25·2–227·9)	-22·8% (-80·9 to 249·8%)	1·01
	Saudi Arabia	51·6 (12·7–107·9)	142·1% (-39·5 to 872·6%)	152·9 (37·8–320)	12·9% (-72·7 to 356·7%)	1·01
	Syrian Arab Republic	11·6 (2·7–24·3)	-3·4% (-79·6 to 382·7%)	79·9 (18·6–167·4)	-6·2% (-79·8 to 370·4%)	0·92
	Tunisia	8·7 (2–19·2)	23·8% (-69·5 to 529·6%)	78·3 (18·3–168)	-0·5% (-75·3 to 381·1%)	0·95
	Türkiye	102·4 (25–214)	18·7% (-71·1 to 432·2%)	131·3 (32·4–273·9)	-5·2% (-76·5 to 327·2%)	0·79
	United Arab Emirates	11·9 (3·1–23·5)	251·2% (-12·1 to 1308·2%)	152·1 (38·2–303·4)	-23·7% (-81·4 to 211·6%)	0·83
	Yemen	25·7 (5·5–56·3)	85·9% (-62·5 to 936·9%)	76·5 (16·8–162·9)	-15·5% (-83·6 to 372·7%)	0·87
	Afghanistan	37·8 (6·4–83·6)	185·9% (-42 to 2058·3%)	92 (15·6–202·9)	-16·3% (-83·1 to 512·7%)	1·07
	Sudan	36·3 (8–79·4)	84·4% (-56·9 to 958·2%)	84·3 (18·4–183·6)	-5·1% (-77·8 to 449·1%)	0·86
Multiple sclerosis	Global	451·2 (320·7–591·5)	71·3% (65·3 to 77·1%)	5·5 (3·9–7·1)	-5·8% (-8·6 to -2·9%)	1·93
	North Africa and Middle East	57·7 (40·8–76·8)	169·3% (156·5 to 183·1%)	10 (7·1–13·3)	10·9% (6·1 to 16·3%)	1·88
	Algeria	4·5 (3·1–6·3)	218·1% (167·6 to 278%)	10·8 (7·3–14·9)	25·3% (6·4 to 48·3%)	1·86
	Bahrain	0·2 (0·1–0·2)	464·7% (361·2 to 595·2%)	8·4 (5·5–11·6)	25·3% (6·2 to 47·9%)	1·96
	Egypt	4·8 (3·2–6·8)	160·1% (121·2 to 202·7%)	5·5 (3·7–7·8)	22·6% (4·4 to 43·5%)	1·87
	Iran	12 (8·4–15·9)	133·3% (117·5 to 150·8%)	13 (9·1–17·2)	-4·8% (-10·5 to 1·6%)	2·00
	Iraq	3·3 (2·2–4·8)	273% (215 to 339·5%)	9·7 (6·4–13·7)	19·1% (0·9 to 40·6%)	1·83
	Jordan	1·2 (0·8–1·7)	343·4% (251·7 to 443·3%)	12·1 (7·9–16·6)	-1% (-21·1 to 20%)	1·96
	Kuwait	0·7 (0·5–1)	468% (369·2 to 600·9%)	13·5 (9·1–18·5)	56·2% (32·3 to 85·2%)	1·63
	Lebanon	0·7 (0·5–1)	174·4% (129 to 232·9%)	13·6 (9·1–18·6)	33% (11·9 to 61·6%)	1·84
	Libya	0·8 (0·5–1·1)	253·4% (192·9 to 321·9%)	10·5 (7·2–14·6)	29·1% (8·3 to 54·8%)	1·72
	Morocco	3·9 (2·5–5·4)	145·6% (107 to 193·4%)	10·4 (6·9–14·5)	23·9% (4·6 to 46·7%)	1·85
	Palestine	0·4 (0·3–0·5)	263·1% (206 to 331·9%)	10·5 (7–14·5)	16·9% (-0·6 to 39·5%)	1·68
	Oman	0·4 (0·3–0·6)	346·2% (275·8 to 434%)	8·8 (5·9–12·2)	30·8% (10·3 to 55·9%)	1·61
	Qatar	0·5 (0·3–0·6)	1172·4% (915·4 to 1561·2%)	14·1 (9·8–18·9)	52·2% (26 to 86·9%)	2·49
	Saudi Arabia	2·8 (1·8–4·1)	353·6% (279·3 to 435·7%)	7·3 (4·8–10·2)	25·4% (6·8 to 45·1%)	2·03
	Syrian Arab Republic	1·5 (1–2·1)	124·9% (89·3 to 167·5%)	10·8 (7·3–15)	22·4% (3·8 to 43·8%)	1·98
	Tunisia	1·7 (1·1–2·3)	165% (124 to 214·2%)	12·7 (8·7–17·3)	27·1% (6·9 to 50·3%)	1·86
	Türkiye	11·8 (8·3–15·3)	109·3% (78 to 144·8%)	12·5 (8·8–16·3)	2·9% (-11·9 to 20%)	1·69

Table S11 Cause- and sex-specific burden, mortality, incidence, and prevalence of neurological conditions in North Africa and Middle East countries

		All Ages		Age-standardised		
		Number (thousand)	Percent change	Rate per 100,000	Percent change	Sex ratio
**Measure**		2019	From 1990 to 2019	2019	From 1990 to 2019	2019
Cause	Location	Mean (95% UI)	Mean (95% UI)	Mean (95% UI)	Mean (95% UI)	F:M
	United Arab Emirates	0·8 (0·5–1·2)	699% (539·2 to 921·1%)	6·3 (4·3–8·6)	-1·4% (-17·9 to 19·9%)	1·79
	Yemen	1·4 (0·9–1·9)	249·8% (197·8 to 314·9%)	6·2 (4·1–8·6)	21·6% (4·4 to 42·9%)	1·64
	Afghanistan	2·6 (1·7–3·6)	260·4% (199 to 333·6%)	10·8 (7·1–14·8)	17·9% (-1·6 to 41·8%)	1·94
	Sudan	1·8 (1·2–2·5)	176·2% (132·5 to 220·5%)	6·1 (4·1–8·5)	22·1% (2·7 to 41·7%)	1·68
Migraine	Global	42077·7 (6418·4–95645·2)	56·6% (52·6 to 62·1%)	525·5 (78·8–1194)	1·5% (-4·4 to 3·3%)	1·7
	North Africa and Middle East	3793·2 (645·3–8665·8)	102·1% (93·2 to 125·1%)	601·4 (107–1371·8)	0% (-1·6 to 1·6%)	1·7
	Algeria	260 (44·3–590)	89% (76·7 to 120·2%)	602·5 (103·9–1372·1)	-0·2% (-3·2 to 3·1%)	1·67
	Bahrain	9·5 (1·8–21·2)	219·3% (197 to 279·3%)	564·5 (100·5–1278·1)	-0·7% (-3·9 to 2·8%)	1·66
	Egypt	619·6 (96·7–1426·4)	98·2% (84·3 to 113·8%)	621·1 (104–1420·2)	1·9% (-5·1 to 9·1%)	1·74
	Iran	571·7 (107–1282·2)	77·6% (63·7 to 117·4%)	628·3 (115·2–1411·2)	-0·3% (-3·6 to 4·1%)	1·66
	Iraq	253·2 (40·1–580·5)	181·6% (170·8 to 206·2%)	595·4 (102·8–1355·6)	0·3% (-2·8 to 3·2%)	1·67
	Jordan	70·1 (11·9–160·3)	250·2% (230·2 to 298·6%)	590·3 (104–1339·1)	-0·8% (-4 to 2·5%)	1·66
	Kuwait	29·8 (5·2–67·4)	190·2% (171·1 to 225·4%)	578·3 (99·7–1315·1)	3·2% (-2·3 to 8·6%)	1·68
	Lebanon	32 (5·6–73·1)	76·4% (68·2 to 92·7%)	605·8 (104–1385·5)	0·5% (-2·7 to 3·5%)	1·67
	Libya	44·9 (7·7–101·7)	103·6% (88·6 to 141·3%)	594·7 (104–1347)	1·3% (-1·9 to 4·4%)	1·67
	Morocco	226·8 (38·7–515·5)	58·5% (50·7 to 80·5%)	601·7 (104·2–1370·5)	-0·3% (-3·3 to 3%)	1·66
	Palestine	28·5 (4·4–66·3)	177·7% (166·5 to 199·2%)	596·1 (102·5–1357·3)	-1·1% (-4·1 to 1·9%)	1·68
	Oman	28·4 (4·9–64·4)	185% (166·5 to 226·5%)	554·2 (98·7–1256·8)	-1·4% (-4·5 to 1·9%)	1·67
	Qatar	18·1 (3·2–40·5)	601·2% (567·3 to 669·2%)	523 (94·3–1174·9)	-2·7% (-6·2 to 0·8%)	1·66
	Saudi Arabia	234·2 (41·4–528·2)	176·4% (152·8 to 229·1%)	562 (99·4–1276·9)	-1·2% (-5·9 to 3·8%)	1·55
	Syrian Arab Republic	91·6 (15·5–212·2)	39·1% (31·2 to 62·3%)	609·9 (105·1–1394·5)	1·7% (-1·9 to 5·2%)	1·68
	Tunisia	74·3 (13·2–168·6)	54·9% (45·1 to 82·1%)	606·3 (104·4–1382·7)	0·3% (-2·6 to 3·4%)	1·67
	Türkiye	528·1 (101·8–1197·4)	56·6% (45·4 to 81·9%)	589·6 (110·2–1340·7)	0·2% (-4·5 to 5·2%)	1·88
	United Arab Emirates	60·2 (12–134)	473% (424 to 588·1%)	535·6 (97·8–1203·7)	-0·7% (-4·3 to 3·1%)	1·66
	Yemen	175·4 (26·6–404·4)	169% (158·9 to 186·6%)	596·7 (104·2–1356·8)	0·1% (-2·9 to 3·2%)	1·65
	Afghanistan	198·8 (30·2–469·5)	244·1% (220·3 to 261·8%)	587·4 (103–1342·7)	-1·3% (-4·9 to 3·2%)	1·67
	Sudan	234·3 (36·7–542)	122·2% (114·9 to 132·9%)	601·9 (104·6–1369·5)	-0·1% (-3·2 to 3·2%)	1·67
Tension-type headache	Global	4541·7 (1395·5–14981·3)	57·8% (45·1 to 65·9%)	56·2 (17–188·5)	-2·5% (-5·4 to 1·1%)	1·25
	North Africa and Middle East	416·6 (138·3–1196·8)	115·5% (84·9 to 132·3%)	68·1 (22·8–195·5)	1% (-9·5 to 8·7%)	1·28
	Algeria	28·5 (9·6–79·5)	106·7% (73·1 to 140·3%)	66·7 (22·6–189)	0·3% (-10·2 to 7·9%)	1·29
	Bahrain	1·1 (0·4–3·1)	254·2% (197·7 to 330·7%)	64·6 (21·3–189·3)	-0·3% (-10·7 to 7·7%)	1·29
	Egypt	65·2 (21–196·7)	100·8% (75 to 120·6%)	69·1 (22·6–207·8)	1·4% (-10·1 to 10·8%)	1·29
	Iran	70·2 (23–207·1)	103·8% (70·9 to 131·5%)	77·6 (24·9–236·4)	5·2% (-5·7 to 15·9%)	1·14
	Iraq	26·3 (8·7–77)	193·4% (151·1 to 230·5%)	66·2 (22·1–190·3)	0·7% (-11·1 to 9·1%)	1·29
	Jordan	7·4 (2·5–21·8)	276·6% (221·8 to 332·8%)	66·2 (22·7–191·4)	0·1% (-10·9 to 8·3%)	1·29
	Kuwait	3·2 (1·1–9)	204·5% (159·1 to 260·4%)	62·8 (20·8–186·4)	1·5% (-10·4 to 11·9%)	1·4
	Lebanon	3·6 (1·2–10)	86·4% (59 to 108·4%)	67 (22·7–191·7)	0·8% (-10·8 to 9·4%)	1·29
	Libya	4·9 (1·7–13·9)	122·3% (84·9 to 164·2%)	66·4 (22·8–191)	1·1% (-11·2 to 9·5%)	1·29
	Morocco	24·8 (8·5–69·4)	70·5% (47·5 to 93·4%)	66·5 (22·7–187·3)	0·2% (-9·8 to 8·1%)	1·29
	Palestine	2·9 (1–8·6)	187·4% (144·1 to 219·3%)	66·2 (22·1–187·5)	-0·3% (-13 to 9·3%)	1·3
	Oman	3·2 (1–9)	208·1% (154·1 to 256·4%)	64·3 (21·6–190·1)	0% (-15·4 to 10·8%)	1·29
	Qatar	2·1 (0·7–6)	643·8% (552 to 739·5%)	62 (20·7–184·7)	-1·4% (-11·6 to 7·2%)	1·29
	Saudi Arabia	25·6 (9·1–70·1)	202% (136·9 to 255%)	63 (22–180·3)	-0·3% (-18·6 to 10·8%)	1·3
	Syrian Arab Republic	9·8 (3·4–28·4)	51·2% (24·9 to 78·8%)	67 (22·9–189·7)	1·1% (-10·5 to 9·5%)	1·29
	Tunisia	8·4 (2·9–23·5)	70·6% (42·7 to 97·2%)	67 (22·6–192·5)	0·7% (-10·7 to 8·5%)	1·3
	Türkiye	60·7 (21·7–160·1)	71·5% (44·9 to 93·8%)	66·6 (23·6–180)	0·8% (-10 to 10·1%)	1·36
	United Arab Emirates	7·4 (2·4–20·5)	542·2% (427·1 to 676·1%)	63·2 (20·7–187·8)	0% (-12·6 to 8·9%)	1·29

Table S11 Cause- and sex-specific burden, mortality, incidence, and prevalence of neurological conditions in North Africa and Middle East countries

		All Ages		Age-standardised		
		Number (thousand)	Percent change	Rate per 100,000	Percent change	Sex ratio
**Measure**		2019	From 1990 to 2019	2019	From 1990 to 2019	2019
Cause	Location	Mean (95% UI)	Mean (95% UI)	Mean (95% UI)	Mean (95% UI)	F:M
	Yemen	17·6 (5·8–51·7)	176·7% (140 to 209·4%)	66·1 (22·8–185·6)	0·6% (-11·1 to 9·8%)	1·28
	Afghanistan	19·6 (6·3–57·7)	231·4% (191·2 to 263·2%)	65·2 (22·1–183·3)	-0·1% (-9·6 to 7·9%)	1·29
	Sudan	23·6 (7·8–69·1)	125% (95·3 to 148·4%)	66·4 (23·2–189)	0·5% (-11·8 to 9·8%)	1·29
Motor neuron disease	Global	57·1 (40–76·3)	68·8% (62·4 to 75·6%)	0·7 (0·5–1)	1·9% (0·6 to 3·3%)	0·82
	North Africa and Middle East	3·3 (2·3–4·6)	103·3% (92·1 to 113·8%)	0·5 (0·4–0·8)	3·6% (2 to 5·1%)	0·86
	Algeria	0·2 (0·1–0·3)	89·9% (74·6 to 104·9%)	0·5 (0·3–0·7)	2% (-1·6 to 5·7%)	0·85
	Bahrain	<0·1 (<0·1–<0·1)	235·2% (197·8 to 274·8%)	0·6 (0·4–0·8)	3·7% (-0·5 to 7·9%)	0·86
	Egypt	0·5 (0·3–0·6)	96·4% (87·1 to 105·5%)	0·5 (0·3–0·7)	4·2% (0·6 to 8·2%)	0·87
	Iran	0·5 (0·4–0·8)	86·9% (70·6 to 103·5%)	0·6 (0·4–0·9)	6·6% (4·9 to 8·4%)	0·89
	Iraq	0·2 (0·1–0·3)	170·7% (154·7 to 186·6%)	0·5 (0·4–0·7)	2·6% (-1·2 to 6·4%)	0·88
	Jordan	0·1 (0–0·1)	252·1% (224·4 to 277·9%)	0·6 (0·4–0·8)	3% (-1·7 to 7·2%)	0·84
	Kuwait	<0·1 (<0·1–<0·1)	166·7% (143·3 to 192·9%)	0·7 (0·5–0·9)	-4·2% (-9·8 to 1·1%)	0·85
	Lebanon	<0·1 (<0·1–<0·1)	77·2% (65·9 to 87·5%)	0·6 (0·4–0·8)	2·8% (-1 to 6·9%)	0·87
	Libya	<0·1 (<0·1–<0·1)	81·7% (64·2 to 98·3%)	0·5 (0·3–0·7)	-4·9% (-8·6 to -0·9%)	0·91
	Morocco	0·2 (0·1–0·3)	62·3% (51·4 to 73·3%)	0·5 (0·3–0·7)	4% (0 to 8%)	0·84
	Palestine	<0·1 (<0·1–<0·1)	175·2% (158·3 to 189·6%)	0·5 (0·4–0·8)	4% (0 to 7·7%)	0·89
	Oman	<0·1 (<0·1–<0·1)	182·6% (159·7 to 207·3%)	0·5 (0·3–0·7)	2·7% (-1·3 to 6·9%)	0·87
	Qatar	<0·1 (<0·1–<0·1)	631·4% (587·1 to 678·9%)	0·6 (0·4–0·8)	4·2% (-0·3 to 8·7%)	0·86
	Saudi Arabia	0·2 (0·1–0·3)	166·9% (141·5 to 192·2%)	0·5 (0·4–0·7)	2·9% (-0·9 to 6·8%)	0·85
	Syrian Arab Republic	0·1 (0·1–0·1)	28·3% (17·7 to 41·1%)	0·5 (0·4–0·8)	0·5% (-3·4 to 4·6%)	0·87
	Tunisia	0·1 (0–0·1)	61·1% (48·2 to 75·5%)	0·6 (0·4–0·8)	4·1% (-0·5 to 8·1%)	0·89
	Türkiye	0·7 (0·5–0·9)	77·3% (64·6 to 89·4%)	0·8 (0·5–1)	10·2% (6·2 to 14·5%)	0·83
	United Arab Emirates	0·1 (0–0·1)	472·1% (400 to 543·2%)	0·6 (0·4–0·8)	-0·6% (-4·4 to 4·5%)	0·87
	Yemen	0·1 (0·1–0·1)	153% (137·6 to 168·2%)	0·3 (0·2–0·5)	-0·8% (-4·5 to 3·2%)	0·86
	Afghanistan	0·1 (0·1–0·2)	238·3% (219·8 to 260%)	0·4 (0·3–0·6)	0·2% (-4 to 4·2%)	0·82
	Sudan	0·1 (0·1–0·2)	123·4% (113·4 to 134·2%)	0·4 (0·2–0·5)	4·1% (0·4 to 8·2%)	0·86
Other neurological disorders	Global	2130·4 (1392·1–3016·4)	93·3% (65·4 to 128·5%)	28·3 (18·4–40·4)	40·6% (20·9 to 64·7%)	0·94
	North Africa and Middle East	177·2 (106·1–265·1)	98·6% (41·9 to 177·9%)	29·4 (18–43·9)	25·5% (-8·9 to 71%)	0·92
	Algeria	11·6 (4–22·1)	61·2% (-48·7 to 478·3%)	28·6 (10·1–54·1)	12·4% (-63 to 244%)	0·96
	Bahrain	0·4 (0·1–0·8)	168·8% (-11·7 to 924·4%)	33·5 (10·8–66·6)	8·3% (-65·7 to 260·8%)	0·98
	Egypt	27·1 (8·4–51·8)	120·5% (-34·1 to 711%)	26·4 (9·5–48·8)	30·6% (-56·7 to 292·5%)	0·81
	Iran	23·8 (14·3–35·3)	40·7% (3·5 to 90·8%)	30·4 (18·3–45·5)	19·7% (-12·2 to 57·7%)	0·94
	Iraq	12·2 (3·6–24)	175·6% (-13·8 to 915·1%)	27·9 (9·7–53·7)	24·2% (-56·8 to 272·3%)	0·95
	Jordan	3·3 (1·1–6·8)	263·1% (14·7 to 1066·3%)	27·8 (10·1–54·6)	26·7% (-54·8 to 240·1%)	0·95
	Kuwait	1·2 (0·4–2·4)	176·9% (-1 to 807·8%)	31·2 (11·4–65)	25% (-53·9 to 247·1%)	0·96
	Lebanon	1·4 (0·5–2·8)	73% (-37·5 to 470·2%)	28·3 (10·4–55·2)	21·8% (-55 to 271·5%)	0·95
	Libya	1·6 (0·7–3)	47·7% (-42·9 to 404·3%)	25·9 (10·8–47·7)	10·2% (-56·3 to 209·3%)	0·92
	Morocco	10·2 (3·3–20·3)	80·6% (-47·6 to 585·6%)	29·1 (9·6–58)	40·2% (-57·1 to 346·5%)	0·99
	Palestine	1·5 (0·4–2·9)	195·7% (-14·6 to 1003·2%)	27·5 (9·6–51·9)	27·6% (-55·3 to 278·1%)	0·97
	Oman	1·1 (0·4–2·2)	171·8% (-21·8 to 887·6%)	28·1 (10·4–55·7)	41·7% (-53·9 to 325%)	0·94
	Qatar	0·7 (0·3–1·4)	536·4% (124·1 to 2135·2%)	30·8 (11·5–61·1)	16·1% (-56·9 to 248·4%)	1·04
	Saudi Arabia	12·8 (4·1–26·1)	195·5% (-14·4 to 925·6%)	39·3 (13·3–79·7)	54·1% (-52·8 to 379·6%)	0·99
	Syrian Arab Republic	3·9 (1·3–7·3)	42·2% (-55·8 to 438·3%)	25·9 (9·4–49·2)	37·1% (-54·2 to 305·1%)	0·97
	Tunisia	2·7 (1·1–5·1)	70·6% (-35·2 to 451·1%)	25 (9·6–47·4)	38·2% (-48·7 to 285·9%)	1·02
	Türkiye	27·2 (10·1–51·7)	46·3% (-49 to 378·4%)	36·7 (13–71·8)	26·5% (-57·8 to 276·8%)	0·84
	United Arab Emirates	2·8 (0·9–5·4)	347·5% (39 to 1491·1%)	39·3 (12·8–76·3)	9·5% (-65·6 to 251·2%)	0·86
	Yemen	8·2 (2·2–17)	162·8% (-28·4 to 1089%)	23·5 (7·9–45·9)	20·8% (-61·6 to 293·2%)	0·92
	Afghanistan	12 (2·9–25·7)	308·4% (0 to 1736·2%)	27·6 (8·7–55·4)	20·4% (-63·8 to 330·8%)	1·09

Table S11 Cause- and sex-specific burden, mortality, incidence, and prevalence of neurological conditions in North Africa and Middle East countries

		All Ages		Age-standardised		
		Number (thousand)	Percent change	Rate per 100,000	Percent change	Sex ratio
**Measure**		2019	From 1990 to 2019	2019	From 1990 to 2019	2019
Cause	Location	Mean (95% UI)	Mean (95% UI)	Mean (95% UI)	Mean (95% UI)	F:M
	Sudan	11·4 (3·2–23·9)	158·3% (-26·2 to 1024·1%)	25·4 (9–50·9)	31·9% (-56 to 326·6%)	0·9
Headache disorders	Global	46619·4 (9772·9–100161·7)	56·7% (52·4 to 62·1%)	581·8 (119·6–1255·6)	1·1% (-4·2 to 2·9%)	1·65
	North Africa and Middle East	4209·8 (990·1–9068·3)	103·3% (94·5 to 123·7%)	669·6 (159·1–1431·3)	0·1% (-2·4 to 2·4%)	1·65
	Algeria	288·4 (67·5–615·9)	90·7% (78·3 to 119·7%)	669·2 (155·7–1442·3)	-0·1% (-3·5 to 3·1%)	1·62
	Bahrain	10·6 (2·6–22·3)	222·7% (200·3 to 275·9%)	629 (149·4–1360·3)	-0·6% (-3·9 to 2·8%)	1·62
	Egypt	684·8 (147·1–1492)	98·4% (84·9 to 112·8%)	690·3 (156·4–1488·7)	1·9% (-4·8 to 8·6%)	1·69
	Iran	641·8 (160·7–1355·8)	80·2% (66·5 to 117·2%)	705·8 (173·2–1503·1)	0·3% (-3·3 to 5·8%)	1·59
	Iraq	279·5 (60·1–621·2)	182·7% (170·9 to 204·1%)	661·6 (153–1427·4)	0·3% (-3·3 to 3·5%)	1·63
	Jordan	77·5 (17·4–171·6)	252·5% (233·2 to 298·1%)	656·4 (153·8–1428·7)	-0·7% (-3·9 to 2·5%)	1·62
	Kuwait	33 (7·6–71·3)	191·5% (173·1 to 225·4%)	641·1 (146·8–1408)	3·1% (-2·3 to 7·9%)	1·65
	Lebanon	35·5 (8·4–77·1)	77·3% (68·9 to 92·6%)	672·7 (153·9–1457·6)	0·5% (-3·1 to 3·7%)	1·63
	Libya	49·8 (11·8–107·6)	105·3% (90·3 to 140·3%)	661·1 (155·4–1425·1)	1·3% (-2·2 to 4·6%)	1·63
	Morocco	251·6 (58·5–545)	59·6% (51·6 to 78·5%)	668·2 (156·2–1445·1)	-0·2% (-3·6 to 3·1%)	1·62
	Palestine	31·4 (6·8–70·6)	178·6% (166·2 to 198·3%)	662·3 (157·1–1439·2)	-1% (-4·4 to 2·1%)	1·63
	Oman	31·6 (7·5–67·8)	187·2% (168·1 to 223·2%)	618·5 (146·4–1332·9)	-1·2% (-4·9 to 2·6%)	1·63
	Qatar	20·2 (5–42·9)	605·4% (571·7 to 667·4%)	585 (140·4–1252)	-2·6% (-5·8 to 0·9%)	1·62
	Saudi Arabia	259·8 (62·7–548·8)	178·7% (155·8 to 227·9%)	625 (149·7–1343·4)	-1·1% (-6·2 to 3·8%)	1·52
	Syrian Arab Republic	101·4 (22·9–223·8)	40·2% (32·3 to 61·8%)	676·9 (157·1–1474·2)	1·7% (-2·1 to 5·1%)	1·63
	Tunisia	82·7 (20·2–176·6)	56·3% (46·6 to 81·3%)	673·3 (158·7–1448·7)	0·3% (-3·1 to 3·5%)	1·63
	Türkiye	588·7 (155·4–1275·3)	58% (47·2 to 81%)	656·1 (167·7–1433)	0·3% (-4·6 to 5%)	1·82
	United Arab Emirates	67·6 (17·6–141·9)	479·9% (432·5 to 583%)	598·8 (142·4–1275·1)	-0·7% (-5 to 3%)	1·62
	Yemen	193 (40·6–431·5)	169·7% (159·4 to 187·2%)	662·8 (157·9–1435·6)	0·1% (-3·4 to 3·6%)	1·61
	Afghanistan	218·4 (44·8–493·2)	242·9% (219·8 to 259·7%)	652·5 (156·7–1422·4)	-1·2% (-4·5 to 2·9%)	1·62
	Sudan	258 (54–579)	122·5% (114·3 to 132·3%)	668·3 (155·5–1438·9)	-0·1% (-3·7 to 3·7%)	1·62
**YLLs (Years of Life Lost)**						
Meningitis	Global	15649·9 (13103–18930·7)	-52·1% (-60·4 to -42·7%)	225 (187·3–273·1)	-57·7% (-65 to -48·8%)	0·85
	North Africa and Middle East	376·5 (310·2–457·5)	-72·5% (-79·3 to -64·6%)	64·1 (53–77·6)	-77·2% (-82·4 to -70·9%)	0·89
	Algeria	14 (11·2–17·7)	-77·6% (-86·5 to -65%)	34·6 (27·8–43·5)	-82·2% (-88·8 to -73·4%)	1·01
	Bahrain	0·1 (0·1–0·2)	-54·4% (-66·4 to -39·6%)	14·7 (12·2–17·6)	-77·7% (-82·9 to -71·1%)	0·75
	Egypt	48·3 (31·7–67·1)	-70·9% (-81·2 to -56·9%)	47·7 (31·3–66·4)	-77·7% (-85·5 to -67·2%)	0·67
	Iran	24·2 (21–27·7)	-82·4% (-87 to -76·1%)	31·7 (27·2–36·5)	-82·1% (-86·5 to -75·8%)	0·79
	Iraq	25·9 (19·8–33·8)	-71·8% (-80·9 to -58·8%)	61·6 (47·4–78·2)	-81·3% (-86·9 to -73·7%)	0·9
	Jordan	4·9 (3·9–6·5)	-6·7% (-36·5 to 38·4%)	44 (34·6–57·4)	-60·4% (-71·9 to -42·9%)	0·97
	Kuwait	0·6 (0·5–0·7)	-33·4% (-51·4 to -11·7%)	16·5 (12·7–21·1)	-67% (-75·7 to -56·6%)	0·59
	Lebanon	1·2 (0·9–1·7)	-64·2% (-77 to -45·1%)	23·8 (16·8–32·5)	-72·3% (-81·8 to -59·2%)	0·74
	Libya	1·8 (1·4–2·4)	-72·1% (-81·8 to -58·5%)	31·2 (23·6–40·4)	-72·9% (-81·3 to -61·5%)	0·91
	Morocco	19·6 (13·1–27·7)	-84·5% (-90·6 to -76%)	61·6 (40·9–87·5)	-83·3% (-89·8 to -74·7%)	0·72
	Palestine	1·6 (1·3–2·1)	-79% (-86·9 to -66·3%)	32·8 (26·9–40)	-85% (-89·8 to -77·9%)	0·74
	Oman	0·6 (0·5–0·8)	-66% (-76·5 to -49·6%)	22·9 (19·3–27·9)	-74·9% (-81·3 to -64·3%)	1·4
	Qatar	0·3 (0·2–0·4)	-17·1% (-48·5 to 28·8%)	17·5 (13·4–22·3)	-79·3% (-86·8 to -69·8%)	2·9
	Saudi Arabia	7·5 (5·5–10·1)	-46·8% (-61·4 to -25%)	23·7 (18·4–30·4)	-75·2% (-81·4 to -65·4%)	1·23
	Syrian Arab Republic	12·3 (9·3–16)	-81·4% (-87·6 to -72·5%)	91·8 (68·7–121)	-72% (-80·9 to -59·6%)	0·92
	Tunisia	3·3 (2·3–4·4)	-79·9% (-87·6 to -68·2%)	31·3 (21·8–41·7)	-80% (-87·5 to -69·4%)	0·65
	Türkiye	16 (13·2–19·4)	-91·4% (-94·7 to -86·4%)	24·4 (20–30·4)	-90·3% (-94·1 to -85%)	0·77
	United Arab Emirates	2·5 (1·5–3·8)	16·6% (-31·4 to 70·4%)	28·5 (19–40·4)	-75·9% (-83·4 to -67·4%)	0·7
	Yemen	30·8 (20·1–46)	-57·8% (-73·6 to -32·2%)	90 (61·5–129·3)	-70% (-80·1 to -55·4%)	1·04
	Afghanistan	115·5 (82·3–166·4)	-32·8% (-56·4 to 4·7%)	225·5 (164·4–306·1)	-74·9% (-83·5 to -63·5%)	1·12

Table S11 Cause- and sex-specific burden, mortality, incidence, and prevalence of neurological conditions in North Africa and Middle East countries

		**All Ages**		**Age-standardised**		
		Number (thousand)	Percent change	Rate per 100,000	Percent change	Sex ratio
**Measure**		2019	From 1990 to 2019	2019	From 1990 to 2019	2019
Cause	Location	Mean (95% UI)	Mean (95% UI)	Mean (95% UI)	Mean (95% UI)	F:M
	Sudan	45 (29·4–69·6)	-80·4% (-87·9 to -69·3%)	96·3 (64·8–140·3)	-85·3% (-90·4 to -77·7%)	0·74
Encephalitis	Global	4315 (3623·2–5928·3)	-46·2% (-58·5 to -17·4%)	59·2 (49·5–80·9)	-55·9% (-65·7 to -33·2%)	0·86
	North Africa and Middle East	231·3 (179·8–314·1)	9·8% (-25·4 to 58·2%)	38·8 (30·3–52·5)	-21·8% (-45·9 to 9·5%)	1·07
	Algeria	7·1 (4·7–13·7)	-9·7% (-47·2 to 43·8%)	17·3 (11·7–32·9)	-32·4% (-58 to 3%)	0·95
	Bahrain	0·1 (0·1–0·1)	52·7% (9·2 to 97%)	9·5 (7·5–12)	-28·6% (-48·3 to -9·3%)	0·94
	Egypt	61·8 (29·7–99·3)	-6·1% (-45·9 to 55·8%)	59·5 (29·6–95·5)	-32·7% (-60·1 to 5·5%)	0·86
	Iran	10·2 (7·7–12·1)	-21·1% (-45·2 to 8·7%)	13·5 (9·8–16·2)	-24·4% (-45·9 to 0·9%)	0·86
	Iraq	29·1 (19·8–39·1)	15·1% (-30·7 to 86·4%)	67·4 (47–89)	-34·7% (-58·1 to -2·2%)	1·1
	Jordan	0·9 (0·7–1·3)	-18·6% (-52·9 to 144·1%)	7·8 (5·8–11·4)	-67·4% (-80·4 to -9·5%)	1·23
	Kuwait	0·3 (0·3–0·4)	38·3% (9·1 to 81%)	8·8 (7·1–11·4)	-30·4% (-44·7 to -10·3%)	1·26
	Lebanon	0·7 (0·4–1·4)	8·8% (-34·1 to 66·7%)	13·7 (8·2–26·8)	-19·5% (-48·9 to 19·7%)	0·92
	Libya	0·9 (0·6–1·8)	-4·4% (-39·8 to 44·2%)	15·2 (9·9–29·6)	-15·9% (-43 to 23·6%)	0·96
	Morocco	7·4 (4·1–15)	-11·2% (-49·8 to 40·9%)	22·9 (12·6–45·6)	-18·4% (-51·2 to 26·5%)	1·43
	Palestine	0·6 (0·4–0·9)	33·7% (-22 to 111·2%)	12·1 (9·1–17·7)	-26·7% (-52·2 to 5·4%)	1·18
	Oman	4·4 (3·2–7·6)	6·2% (-33 to 66·1%)	119·1 (91·1–177·2)	-44·5% (-63 to -14·1%)	0·66
	Qatar	0·2 (0·2–0·4)	238·1% (108·4 to 412·1%)	11·9 (8·7–16·3)	-31·3% (-56·3 to 1·4%)	1·35
	Saudi Arabia	8·8 (6·5–12)	-2·1% (-33·1 to 48%)	26·4 (19·8–35·6)	-54·5% (-69·8 to -31%)	1·26
	Syrian Arab Republic	3·2 (2·3–4·4)	-57·2% (-74·4 to -20·4%)	23·7 (17·6–33)	-44·1% (-65·5 to -0·8%)	0·92
	Tunisia	1·6 (1–3·3)	-25·7% (-57·6 to 15·8%)	15·4 (9·1–31·5)	-31·5% (-59·2 to 5·4%)	0·87
	Türkiye	10·6 (8·1–13·1)	-50·2% (-70·5 to -21·4%)	15·6 (11·8–19·6)	-51·4% (-70·6 to -23·6%)	0·91
	United Arab Emirates	1·3 (0·7–2·8)	204·8% (88·7 to 370·6%)	16·8 (10·4–32)	-22·3% (-48·1 to 16·4%)	0·72
	Yemen	10·3 (5·7–20·5)	45·6% (-19·9 to 167·3%)	27·8 (16·2–54·6)	-9·4% (-46·2 to 51·2%)	1·18
	Afghanistan	57·7 (36·8–113·5)	153·3% (68·1 to 262·9%)	168·2 (124·6–241·3)	-15·1% (-39·9 to 17·6%)	1·43
	Sudan	13·6 (7·4–25·9)	10·8% (-42·9 to 121·2%)	28·8 (16·4–54·8)	-24% (-57·6 to 34·5%)	1·05
Tetanus	Global	2314·6 (1768·8–3277)	-89·4% (-92·1 to -83·8%)	33·7 (25·6–47·8)	-90·3% (-92·7 to -85·1%)	0·86
	North Africa and Middle East	87·4 (53·6–139·5)	-85·8% (-92·9 to -68·7%)	15 (9·2–24·1)	-88·2% (-93·9 to -76·1%)	0·85
	Algeria	0·5 (0·4–0·9)	-83% (-93·5 to -41·9%)	1·3 (0·9–2·3)	-86·7% (-94·8 to -60·7%)	0·79
	Bahrain	<0·1 (<0·1–<0·1)	37·1% (-41 to 114·9%)	0·7 (0·6–1·2)	-39·4% (-75 to -6·6%)	1·00
	Egypt	7·4 (2·2–22)	-87·3% (-96·4 to -60·1%)	7·7 (2·5–23·2)	-90·8% (-97 to -71·7%)	0·69
	Iran	0·9 (0·7–1·3)	-94·4% (-97·6 to -82·2%)	1·2 (0·9–1·7)	-94·1% (-97·4 to -82·4%)	0·74
	Iraq	1·8 (1–3)	-91·2% (-97·3 to -53·5%)	4 (2·3–6·7)	-93·8% (-98 to -72·8%)	0·56
	Jordan	0·1 (0·1–0·2)	-71·8% (-86·8 to -12·3%)	1 (0·7–1·5)	-85·7% (-93·3 to -59%)	0·72
	Kuwait	<0·1 (<0·1–<0·1)	84·7% (-77·8 to 686·7%)	<0·1 (<0·1–0·1)	-47·7% (-91 to 74·2%)	0·06
	Lebanon	0·8 (0·2–2·7)	-57·9% (-93 to 27·8%)	14·9 (3·2–50·9)	-75·8% (-95·7 to -29·1%)	1·11
	Libya	0·1 (0–0·1)	-69·7% (-88·1 to -8·1%)	1·2 (0·7–2)	-70·5% (-89·8 to -19%)	0·72
	Morocco	7·7 (1·9–28·9)	-95% (-98·8 to -80·8%)	25·7 (6·3–98·1)	-93·9% (-98·5 to -76·2%)	0·84
	Palestine	0·1 (0·1–0·1)	-90·5% (-97·4 to -52·6%)	1·3 (0·9–1·9)	-92·7% (-98 to -67·8%)	0·71
	Oman	<0·1 (<0·1–0·1)	-79·7% (-93·8 to -19·4%)	1 (0·7–1·3)	-90·3% (-96·7 to -62·2%)	0·77
	Qatar	<0·1 (<0·1–<0·1)	195·1% (8·3 to 463·4%)	0·7 (0·5–1)	-50·2% (-83·3 to 5%)	0·91
	Saudi Arabia	0·7 (0·2–1·3)	-94·2% (-99·2 to -68·2%)	2·1 (0·8–3·5)	-96·5% (-99·4 to -81·2%)	0·58
	Syrian Arab Republic	0·3 (0·2–0·6)	-97·6% (-99·2 to -89·5%)	2·4 (1·3–5)	-95·6% (-98·6 to -81·4%)	0·52
	Tunisia	0·1 (0·1–0·2)	-83·7% (-94 to -56·6%)	1 (0·7–1·8)	-83·8% (-94 to -57·1%)	0·8
	Türkiye	0·7 (0·5–1)	-98% (-98·9 to -94·1%)	1 (0·8–1·4)	-98·3% (-99 to -95·6%)	0·83
	United Arab Emirates	1·1 (0·5–1·7)	-8% (-83·2 to 209·2%)	33·2 (8·4–54·9)	-86% (-96·4 to -46·5%)	1·79
	Yemen	4·2 (1·7–8·8)	-85·7% (-95·7 to -34·5%)	11·2 (5–23·3)	-89·9% (-96·5 to -61·6%)	0·51
	Afghanistan	58·1 (32·9–101·6)	-74·6% (-88·5 to -32·2%)	113·9 (70·4–179·3)	-90·2% (-94·9 to -79·6%)	1·03
	Sudan	2·7 (1·1–5·6)	-92·9% (-97·4 to -77·6%)	5·6 (2·6–12·1)	-94·3% (-97·8 to -83·9%)	0·48
Brain and central nervous system	Global	8530·5 (6625·1–9447·6)	39·7% (-13·7 to 66%)	107·4 (83·1–119)	-10·8% (-43·7 to 4·8%)	0·71

Table S11 Cause- and sex-specific burden, mortality, incidence, and prevalence of neurological conditions in North Africa and Middle East countries

**Measure**		**All Ages**		**Age-standardised**		
		Number (thousand)	Percent change	Rate per 100,000	Percent change	Sex ratio
		2019	From 1990 to 2019	2019	From 1990 to 2019	2019
Cause	Location	Mean (95% UI)	Mean (95% UI)	Mean (95% UI)	Mean (95% UI)	F:M
cancer	North Africa and Middle East	705·6 (486–836·9)	69·9% (0·4 to 130·4%)	126·4 (86·6–149·3)	-5·6% (-40·2 to 22·6%)	0·86
	Algeria	23·3 (14·1–29·7)	55·2% (-17·1 to 119%)	57·4 (34·8–72·8)	-6·6% (-50·2 to 27·9%)	0·98
	Bahrain	1 (0·6–1·3)	183·3% (66·3 to 297·5%)	71·5 (45·8–91·6)	-25·8% (-56·1 to 5·3%)	0·83
	Egypt	88 (60·6–123·9)	72·8% (-6·2 to 152·9%)	97·6 (67·4–139·6)	-2·2% (-41 to 39·4%)	0·83
	Iran	126·2 (66·6–150·7)	39·2% (-28·5 to 80%)	153·5 (80·7–183·6)	-11·4% (-50·6 to 7·8%)	0·89
	Iraq	78·8 (57·2–102)	162·9% (31·3 to 323·7%)	222·7 (161·1–285·4)	14·7% (-39·3 to 78·9%)	1·02
	Jordan	9·6 (6·8–11·8)	199·4% (90 to 306%)	96·1 (68·3–118·7)	-10·9% (-44·2 to 21·4%)	0·92
	Kuwait	2·4 (1·7–3)	134·1% (43·7 to 198·9%)	64·3 (45·7–80·2)	-10% (-45·1 to 14·8%)	0·65
	Lebanon	5·9 (4·1–7·6)	57·1% (5·6 to 115·1%)	114·1 (80·2–146·9)	-10·5% (-39·7 to 21·6%)	0·79
	Libya	8·5 (6·2–11·2)	70·9% (15·5 to 160·1%)	132·5 (97·6–173·2)	-5% (-32·8 to 36·5%)	0·63
	Morocco	19·4 (13–26·8)	56·4% (-9·8 to 133·7%)	54·6 (36·9–73·8)	2·3% (-37·4 to 47·3%)	1·39
	Palestine	8·8 (6·8–10·9)	113·7% (30·7 to 199·1%)	228·8 (173·5–275·6)	-9·4% (-40·1 to 23·6%)	0·82
	Oman	2·5 (1·4–3·2)	148% (25·1 to 276·2%)	71 (42·8–89·6)	9·7% (-45·2 to 63·5%)	0·97
	Qatar	1·6 (1·1–2·7)	422·6% (204·5 to 702·6%)	83·1 (59·5–125·1)	-21·6% (-53·8 to 18·8%)	1·25
	Saudi Arabia	25·8 (18·8–38·9)	245·2% (88·5 to 548·5%)	77·6 (57·8–117·9)	30·4% (-30·5 to 145·4%)	0·92
	Syrian Arab Republic	20·1 (14·4–27·2)	33·6% (-23·6 to 106·3%)	142·6 (103–191·1)	-7·3% (-38·8 to 36·5%)	0·83
	Tunisia	5 (3·3–6·8)	42·9% (-16·3 to 111%)	41 (27–56)	-6·3% (-42·7 to 36·2%)	1·2
	Türkiye	130·4 (62·3–180·1)	23·2% (-35·1 to 91·7%)	155·2 (75·2–211·6)	-21·2% (-55·8 to 17·8%)	0·77
	United Arab Emirates	14·1 (8·1–21)	459·7% (269·5 to 712·1%)	149·4 (90·4–203·9)	-11% (-45·5 to 29·8%)	0·8
	Yemen	31·3 (18·6–46)	136·6% (23·5 to 365·2%)	122·1 (74·2–179·5)	15% (-36 to 99·4%)	0·76
	Afghanistan	54·1 (28·9–95·3)	152·6% (56·9 to 371·5%)	174·6 (98·6–321·4)	-5·9% (-38·6 to 55·9%)	0·81
	Sudan	48 (28·8–70·5)	69·9% (-20·1 to 292·2%)	134·4 (83·4–194·7)	-1·7% (-49 to 87·2%)	0·69
Stroke*	Global	125490·4 (116154·7–134427·9)	27% (15·3 to 37·8%)	1550 (1434·5–1660·7)	-38% (-43·7 to -32·9%)	0·7
	North Africa and Middle East	6832·3 (6014·8–7787·8)	34·5% (18 to 53·6%)	1586·8 (1407–1782·8)	-35·1% (-42·5 to -25·9%)	0·99
	Algeria	458·9 (365·7–567·4)	37·9% (8·1 to 76·4%)	1512·6 (1223–1842·1)	-47·8% (-58·8 to -34%)	1·22
	Bahrain	5·9 (4·8–7·5)	82·8% (42·3 to 141·6%)	767 (633·5–977·6)	-56·1% (-65 to -45%)	1·21
	Egypt	1300·1 (960·6–1749·9)	-15·4% (-39·9 to 25·9%)	1855·4 (1367·5–2533·1)	-39·7% (-55·1 to -19·7%)	1·08
	Iran	736·7 (679·9–781·7)	38·6% (23 to 55·4%)	1065·8 (976·7–1134·4)	-49·2% (-54·5 to -41·4%)	0·93
	Iraq	604·5 (471·7–746)	112·7% (62·5 to 175·2%)	2616·5 (2102·7–3132·9)	-20·5% (-38·2 to 0%)	0·77
	Jordan	65·6 (54·3–78·1)	111·1% (67·1 to 162%)	1163·5 (954·9–1374·6)	-54·1% (-63·2 to -43·8%)	1·11
	Kuwait	19·2 (15·8–23·1)	224·1% (165·5 to 293·9%)	749 (616·5–896·1)	-12·8% (-27·8 to 5·7%)	0·58
	Lebanon	27·4 (19·3–35·9)	45·3% (2·7 to 96·7%)	528·3 (372·5–692·1)	-42·3% (-59·3 to -21·3%)	0·91
	Libya	68·6 (51·2–90·6)	39·1% (0·8 to 99·7%)	1322·4 (1000·5–1742·3)	-25·6% (-43·4 to 0·2%)	1·13
	Morocco	581·1 (451·5–721·7)	66·8% (27·8 to 110·2%)	1992·7 (1584·1–2446·6)	-19·4% (-37·5 to 0·5%)	1·01
	Palestine	38·6 (33–43·9)	54% (18·4 to 97·9%)	1890·3 (1632·7–2140·5)	-35% (-49·6 to -16·4%)	0·94
	Oman	25·3 (21·8–30·6)	13·8% (-13·2 to 53·4%)	1647·1 (1439·9–1874·5)	-41·5% (-55 to -21·9%)	0·91
	Qatar	5·9 (4·3–7·9)	187·7% (103·6 to 298·9%)	722·3 (572·2–941)	-48·2% (-62 to -31%)	1·78
	Saudi Arabia	348·7 (259·5–437·9)	116·2% (51·2 to 209·1%)	1815·5 (1408·2–2165·5)	-32·8% (-51 to -7·4%)	1·00
	Syrian Arab Republic	208·3 (157·6–274)	-11·7% (-36 to 23·3%)	1784 (1385·4–2304·3)	-42·5% (-58·2 to -20·6%)	0·96
	Tunisia	152 (112·9–200·8)	73·3% (26·9 to 133·5%)	1278·1 (957·8–1669)	-28·9% (-48 to -4·6%)	0·86
	Türkiye	825·1 (660·4–1008·3)	70·3% (22·4 to 119·6%)	971·3 (778·9–1182·1)	-25·9% (-46·7 to -4·8%)	0·89
	United Arab Emirates	76·7 (53–110·4)	376·4% (212·6 to 600·9%)	1589·3 (1200–2106·9)	-50·2% (-63·3 to -33·3%)	0·94
	Yemen	348·7 (262·5–463·4)	81·9% (35·4 to 149·9%)	2515·8 (1917–3278·3)	-26·4% (-43·7 to -2·2%)	1·04
	Afghanistan	466·3 (332·5–617·4)	52% (14·2 to 96·2%)	3249·7 (2264·5–4235·5)	-23·2% (-41·6 to -4%)	1·42
	Sudan	461·8 (329–661)	14·7% (-15·3 to 52·6%)	2309 (1690·7–3286·2)	-35·9% (-49·3 to -18·7%)	1·11
Ischaemic stroke	Global	50349·7 (46232·4–54066·7)	48·1% (32·3 to 61·3%)	636·5 (582–683·1)	-33·4% (-40 to -27·6%)	0·73
	North Africa and Middle East	3874·1 (3427·8–4371·1)	112·9% (77·1 to 146·1%)	987·9 (880·9–1105·9)	-11·1% (-23·3 to 1·3%)	1·03

Table S11 Cause- and sex-specific burden, mortality, incidence, and prevalence of neurological conditions in North Africa and Middle East countries

		All Ages		Age-standardised		
		Number (thousand)	Percent change	Rate per 100,000	Percent change	Sex ratio
**Measure**		2019	From 1990 to 2019	2019	From 1990 to 2019	2019
Cause	Location	Mean (95% UI)	Mean (95% UI)	Mean (95% UI)	Mean (95% UI)	F:M
	Algeria	302·9 (238–378)	121·8% (69·2 to 188%)	1071 (848·5–1326·6)	-27·8% (-43·4 to -9·4%)	1·24
	Bahrain	2·6 (2·1–3·3)	123·7% (71·8 to 193·8%)	473·6 (390–608·3)	-47·7% (-58·7 to -35%)	1·21
	Egypt	704·1 (521·9–961)	86·9% (27·6 to 165·9%)	1141·7 (834·6–1566·8)	10·8% (-22 to 46%)	1·25
	Iran	544 (496·6–578·7)	58·8% (39·2 to 79·3%)	815·3 (737·4–870·1)	-46·6% (-52·8 to -38%)	0·98
	Iraq	312·9 (249·3–376·9)	151·1% (90·4 to 223·9%)	1579·4 (1289–1862·9)	-7·1% (-28·1 to 17·2%)	0·82
	Jordan	43·3 (35·6–51·1)	136·7% (84·1 to 191·8%)	840·5 (689·2–990·8)	-50·6% (-61·4 to -39·3%)	1·14
	Kuwait	10·6 (8·7–12·9)	246·8% (184·7 to 326·6%)	492·2 (402·7–595·2)	-13·3% (-28·9 to 5·9%)	0·63
	Lebanon	21·1 (14·3–27·4)	116·3% (46·7 to 187%)	407·8 (275·3–528·6)	-22·7% (-46·2 to 3·1%)	0·9
	Libya	44·2 (32·7–59·7)	152·8% (85·7 to 252·6%)	912·8 (682·1–1217·5)	9% (-17·8 to 46·7%)	1·13
	Morocco	381·4 (298–470·1)	172·2% (101·8 to 245·1%)	1380·6 (1094·3–1686·2)	19·5% (-9·3 to 48%)	1·03
	Palestine	26·1 (22·1–29·8)	92·3% (46·2 to 150%)	1387·8 (1182·5–1583·7)	-19·8% (-38 to 3·7%)	0·97
	Oman	13·1 (11–15·5)	61·9% (19·4 to 124·2%)	1093·5 (932·1–1272·3)	-20·6% (-39·3 to 8·4%)	0·64
	Qatar	1·7 (1·3–2·4)	199% (99 to 324·1%)	433·9 (344·6–570·4)	-38·7% (-55·7 to -18%)	1·89
	Saudi Arabia	163·1 (119·6–201·3)	136·7% (62·8 to 229·7%)	1078·7 (809·9–1276·7)	-21·7% (-43 to 4·6%)	1·01
	Syrian Arab Republic	93·7 (70·7–121·9)	34·6% (-5 to 89·5%)	887·1 (687–1136·2)	-27·8% (-49·7 to -0·9%)	1·12
	Tunisia	106·8 (79·9–140·3)	175·1% (94·2 to 273·1%)	919·3 (689·9–1202·8)	0·9% (-28 to 35·7%)	0·87
	Türkiye	424·5 (337·5–520)	138·3% (59·8 to 211·6%)	516·9 (411·8–632·2)	-8·6% (-38·7 to 19·9%)	0·97
	United Arab Emirates	40 (27·9–55·1)	582·3% (344·3 to 936·2%)	1125·8 (857·3–1440)	-38·6% (-54·7 to -17·6%)	0·96
	Yemen	181·7 (139·4–241·2)	234·9% (150·9 to 360%)	1513·8 (1168·7–2002)	20·4% (-6·8 to 57·4%)	1·06
	Afghanistan	195·5 (141·6–268·2)	134·5% (74·8 to 207·3%)	1675·8 (1191·4–2257·4)	28·6% (-2·2 to 64·4%)	1·36
	Sudan	256·9 (187·2–378)	102·5% (50·2 to 167·8%)	1465·8 (1081·7–2132·7)	6·2% (-16·1 to 35·3%)	1·12
Intracerebral haemorrhage	Global	65306·2 (60073·8–70392·3)	24% (10·4 to 35·4%)	793·4 (731·2–854·6)	-37·4% (-44·2 to -31·5%)	0·66
	North Africa and Middle East	2520 (2171·8–2943·1)	0·1% (-15·7 to 18·7%)	514 (447·9–593)	-52·9% (-59·7 to -44·9%)	0·91
	Algeria	131·1 (101·7–165·7)	-19·2% (-39·1 to 7·8%)	374·3 (293–473·1)	-69% (-76·8 to -59·3%)	1·16
	Bahrain	2·6 (2–3·3)	67·6% (24·5 to 126·6%)	238·4 (192·5–299·8)	-65·9% (-74 to -55·4%)	1·26
	Egypt	498·9 (354·5–683·9)	-38·6% (-58·8 to -8·7%)	608 (426·1–849·3)	-60·8% (-72 to -46%)	0·86
	Iran	156·2 (147·5–165·4)	2% (-11 to 15·5%)	203·3 (190·9–215·2)	-55·6% (-61·9 to -49·2%)	0·76
	Iraq	272·1 (207·2–348)	97·1% (48 to 165·7%)	973·6 (760·3–1219·7)	-30·5% (-48 to -8·6%)	0·68
	Jordan	20·1 (16·6–24·1)	73·5% (36·2 to 118·7%)	294·9 (244·2–353)	-61·7% (-69·8 to -52%)	1·02
	Kuwait	7·4 (5·9–9)	254·6% (175·8 to 343·4%)	220·2 (175·7–269·5)	-4% (-25·4 to 20·7%)	0·44
	Lebanon	5·4 (3·8–7·6)	-27·2% (-50·1 to 4·9%)	103·7 (72·5–145·4)	-67·9% (-77·7 to -53·8%)	0·93
	Libya	20·7 (15–28·7)	-6·5% (-34·2 to 39·9%)	347·2 (254–476·4)	-52·7% (-65·3 to -34·7%)	1·13
	Morocco	170 (126·5–220·3)	1·6% (-27·3 to 38·3%)	521·4 (392·9–671)	-52% (-65·3 to -34·6%)	0·95
	Palestine	11·3 (9·5–13·3)	7% (-18·3 to 42·8%)	462 (389·4–544)	-57·7% (-67·7 to -43·6%)	0·84
	Oman	9·8 (8·4–12·4)	-7·1% (-29·6 to 28·1%)	473 (407·1–592·1)	-60·9% (-70·6 to -47·4%)	1·9
	Qatar	2·8 (2·1–3·9)	174·5% (83·4 to 292·8%)	215 (165·9–284·7)	-58·4% (-70·5 to -42·9%)	1·59
	Saudi Arabia	171·9 (126·8–221·2)	106·7% (41 to 213·9%)	695·8 (533·8–855·4)	-43·4% (-60·1 to -16%)	0·97
	Syrian Arab Republic	100·6 (75·7–135·8)	-13·1% (-38·9 to 24·2%)	796·9 (605·4–1057·1)	-49·2% (-63·8 to -28·7%)	0·82
	Tunisia	38·2 (27·6–51)	-1·2% (-31·3 to 40%)	303·9 (221·8–403·7)	-58% (-70·6 to -40%)	0·81
	Türkiye	320·1 (253·6–397·6)	34·2% (-3·3 to 74·6%)	364·9 (289·8–451·6)	-38·3% (-56·1 to -20·1%)	0·8
	United Arab Emirates	31·8 (20·8–50·2)	281·6% (130·5 to 495·2%)	411·1 (282·8–629·3)	-65·7% (-77·3 to -50·2%)	0·85
	Yemen	139·6 (101·6–188·1)	23% (-12·1 to 75·9%)	847·6 (623·1–1140·1)	-54·2% (-66·6 to -36·7%)	0·99
	Afghanistan	231·7 (166·2–313·9)	21·1% (-10 to 64·3%)	1348·6 (941·2–1813·7)	-46·5% (-60 to -29·7%)	1·46
	Sudan	175·2 (115·8–250·4)	-22·8% (-45·8 to 6·5%)	730·2 (488·8–1029)	-61·5% (-71·1 to -48·6%)	1·09
Subarachnoid haemorrhage	Global	9834·5 (8614·6–11416·4)	-19·1% (-30·7 to 12·6%)	120·1 (105·5–139·2)	-57% (-63·3 to -39·8%)	0·82
	North Africa and Middle East	438·2 (355·9–559·6)	-41% (-55·3 to 0·5%)	84·8 (69·4–107·1)	-64·9% (-73·3 to -43·4%)	1·03
	Algeria	24·9 (18·2–34·2)	-26·7% (-49·3 to 20·6%)	67·4 (49·6–90·1)	-67·1% (-77·2 to -46·7%)	1·15

Table S11 Cause- and sex-specific burden, mortality, incidence, and prevalence of neurological conditions in North Africa and Middle East countries

		**All Ages**		**Age-standardised**		
		Number (thousand)	Percent change	Rate per 100,000	Percent change	Sex ratio
**Measure**		2019	From 1990 to 2019	2019	From 1990 to 2019	2019
Cause	Location	Mean (95% UI)	Mean (95% UI)	Mean (95% UI)	Mean (95% UI)	F:M
	Bahrain	0·7 (0·5–1)	37·2% (-6·2 to 119·3%)	55 (41·9–77·4)	-61% (-73 to -43·7%)	1·02
	Egypt	97·1 (67·3–138)	-72% (-84·1 to -33·8%)	105·7 (74·2–149·7)	-78·7% (-86·7 to -53·8%)	0·82
	Iran	36·6 (32·3–43·6)	2·8% (-24·5 to 62·9%)	47·2 (41·9–54·8)	-59% (-71·2 to -34·3%)	0·94
	Iraq	19·6 (13·9–27·5)	-9·4% (-42·4 to 54·8%)	63·4 (45·7–89·1)	-66·5% (-79·1 to -42·8%)	1·02
	Jordan	2·2 (1·7–2·8)	84·9% (27·4 to 189·2%)	28·1 (20·8–34·8)	-57·8% (-71 to -34·1%)	1·01
	Kuwait	1·3 (1–1·6)	58% (17·6 to 159%)	36·6 (29·5–46·1)	-41% (-55·4 to -13·9%)	0·9
	Lebanon	0·9 (0·5–1·3)	-46·5% (-73·5 to -0·1%)	16·8 (9·5–25·3)	-73·7% (-87·1 to -50·4%)	1·2
	Libya	3·7 (2·5–5·5)	-61·8% (-77·8 to -10·3%)	62·4 (42·4–92·6)	-69·6% (-80·3 to -45·5%)	1·16
	Morocco	29·6 (19·4–45·1)	-27·5% (-51·9 to 25·2%)	90·6 (61–134·7)	-60·6% (-74·2 to -30·8%)	1·18
	Palestine	1·2 (0·9–1·4)	29% (-10·1 to 98·3%)	40·6 (33–48·7)	-51% (-66·6 to -25·4%)	1·22
	Oman	2·4 (1·6–4)	-33·3% (-61·6 to 47·5%)	80·6 (51·4–125·1)	-64·4% (-79 to -30·7%)	1·27
	Qatar	1·4 (1–1·8)	203·9% (73·8 to 389·9%)	73·4 (53·3–97·8)	-56·9% (-74·6 to -27·3%)	1·7
	Saudi Arabia	13·7 (9·2–19·2)	48% (-17·9 to 162%)	41 (28·5–55·1)	-57% (-76·5 to -24·9%)	1·52
	Syrian Arab Republic	14 (10·4–17·8)	-72·3% (-82 to -52·7%)	100 (75·4–126)	-67·5% (-77·7 to -49·3%)	0·83
	Tunisia	6·9 (4·6–9·8)	-32% (-58·1 to 19·4%)	54·9 (37–77)	-66·3% (-79·7 to -41·1%)	1·01
	Türkiye	80·5 (61·6–103·1)	18·9% (-27·9 to 116·3%)	89·5 (69·3–113·8)	-41·8% (-64·7 to 8·3%)	0·8
	United Arab Emirates	5 (2·3–8·9)	158·9% (42·8 to 384·3%)	52·3 (24·9–85·9)	-67·3% (-80 to -45·5%)	1·35
	Yemen	27·5 (13·5–46)	14·3% (-25·3 to 99·8%)	154·5 (76·3–264)	-50·2% (-65·3 to -20·4%)	1·23
	Afghanistan	39·1 (11·8–68·6)	22·2% (-15·8 to 93·1%)	225·4 (67·3–391·3)	-44·6% (-61 to -14·5%)	1·6
	Sudan	29·7 (15·6–53)	-39·2% (-59·5 to 28·9%)	113 (60·8–198·4)	-65·4% (-76·4 to -33·3%)	1·12
Neurological disorders†*	Global	32098·4 (18750·1–60829·3)	81·3% (45 to 114·6%)	422·3 (243·2–806·2)	-7·4% (-16·5 to 2%)	0·91
	North Africa and Middle East	1708·2 (1049·4–3130·8)	68·3% (24·8 to 124·2%)	447·2 (241·7–897·3)	-11·5% (-23·1 to 7·1%)	0·97
	Algeria	120·5 (65·6–244·8)	76·5% (15·8 to 149·8%)	436·9 (213·5–944)	-19·1% (-37·2 to -0·3%)	1·06
	Bahrain	2·8 (1·8–5)	177·7% (104·4 to 266·1%)	464·1 (236·7–947·9)	-14·5% (-29·9 to 1·7%)	1·02
	Egypt	198·5 (116–369·3)	80·9% (36·9 to 132·9%)	405·1 (199·8–823·1)	-1·6% (-20·5 to 23·9%)	1·04
	Iran	270·1 (147·8–532·9)	93·5% (24·4 to 184·4%)	418·4 (213·6–866·4)	-11·4% (-24·7 to 14%)	0·97
	Iraq	84·1 (50·5–154·9)	103% (54 to 160·3%)	418·7 (207·7–864·7)	-5·2% (-22·9 to 15·3%)	0·89
	Jordan	20·4 (12·1–39)	234·3% (153 to 323·6%)	384 (185–824·8)	-12·8% (-28·2 to 7·1%)	1·00
	Kuwait	8·1 (4·3–16·1)	165·2% (85·4 to 248·6%)	378·8 (172·1–818·2)	-15·4% (-30·9 to -2·5%)	0·94
	Lebanon	21·1 (10·3–46)	130·7% (67·3 to 224·2%)	417·9 (201·9–909·6)	-9·8% (-26·4 to 24·9%)	0·96
	Libya	21·9 (11·9–42·8)	102% (46·8 to 168·6%)	473·7 (234·6–985·4)	-4·9% (-24·5 to 21%)	0·97
	Morocco	108·6 (55·9–219·9)	107·6% (62·2 to 147%)	430·1 (205·5–908·5)	3·7% (-13·6 to 22%)	0·93
	Palestine	10·4 (7·2–18·4)	78·8% (37·8 to 135·8%)	460·6 (252·7–984·1)	-12·5% (-30·3 to 10·9%)	0·87
	Oman	5 (3·1–9·4)	118·9% (77·3 to 181·2%)	462·8 (225·9–999·7)	1·4% (-15·7 to 39·5%)	0·93
	Qatar	2·4 (1·6–4·1)	370·4% (237·2 to 547·8%)	482·8 (243·5–1022·1)	0·2% (-21·4 to 25·4%)	1·16
	Saudi Arabia	72·2 (48·1–118·9)	115% (55·8 to 200·1%)	479·1 (260·8–954·7)	-5·6% (-25·5 to 29·5%)	0·91
	Syrian Arab Republic	36·7 (18·4–76·7)	62% (10·4 to 128·3%)	410·7 (188·7–907·5)	0·5% (-23·9 to 36·2%)	1·01
	Tunisia	45·1 (21–96·2)	103·5% (31·5 to 186·1%)	416·5 (189·5–892·1)	-9·4% (-31·7 to 20·7%)	0·94
	Türkiye	398·2 (225·2–780·4)	30·7% (-14·4 to 93·1%)	511 (293·3–978·2)	-22·8% (-39·9 to -2%)	0·94
	United Arab Emirates	20·3 (13·6–29·8)	482·3% (297·7 to 728·9%)	515·1 (295·4–990·3)	-9·1% (-27·7 to 15·9%)	0·81
	Yemen	59·9 (37·2–106·7)	79% (15·7 to 196·2%)	437 (215·1–925·6)	-5·7% (-25·8 to 23%)	0·96
	Afghanistan	103·1 (67–153·5)	61% (24·8 to 135·6%)	606·2 (341·8–1162·2)	-19·5% (-37·6 to 2·3%)	1·01
	Sudan	96·8 (60·9–163·2)	21·2% (-24·2 to 109·6%)	451 (240·8–919·7)	-19% (-40·9 to 5·1%)	0·87
Alzheimer's disease and other dementias	Global	17859·9 (4374·6–47064·8)	160·3% (145·7 to 181·7%)	239·8 (58·5–627·2)	2·9% (-1·5 to 9·8%)	1·15
	North Africa and Middle East	855·9 (207·4–2304·9)	173·2% (151·8 to 232·1%)	274·2 (67·3–727·7)	-2·6% (-9·7 to 17·2%)	1·13
	Algeria	68·2 (16·7–189·7)	223·2% (150·9 to 312·3%)	288·2 (70·3–791·7)	-7·2% (-23·2 to 12·4%)	1·14
	Bahrain	1·2 (0·3–3·4)	382·8% (289·1 to 512·1%)	289·4 (70·1–764·3)	-6·2% (-20·9 to 12·1%)	1·15

Table S11 Cause- and sex-specific burden, mortality, incidence, and prevalence of neurological conditions in North Africa and Middle East countries

		All Ages		Age-standardised		
		Number (thousand)	Percent change	Rate per 100,000	Percent change	Sex ratio
**Measure**		2019	From 1990 to 2019	2019	From 1990 to 2019	2019
Cause	Location	Mean (95% UI)	Mean (95% UI)	Mean (95% UI)	Mean (95% UI)	F:M
	Egypt	94·4 (21·9–259·4)	109·5% (67·1 to 173·8%)	252·4 (56·8–672·4)	-1·7% (-19·3 to 25·3%)	1·16
	Iran	160·8 (38·2–426·3)	309·6% (265·3 to 447·6%)	272·1 (66·1–723)	-0·9% (-7·7 to 32%)	1·12
	Iraq	40·9 (9·7–107·6)	153·1% (109·1 to 209·2%)	271·4 (64·8–723·4)	0·3% (-16 to 21·3%)	1·07
	Jordan	10·3 (2·5–28·6)	367·9% (287·4 to 478·3%)	258·1 (63–715·8)	-7·7% (-22·3 to 14·6%)	1·13
	Kuwait	4·8 (1·2–12·6)	379·4% (320·7 to 456·3%)	271 (67·9–731)	-3·9% (-15·3 to 10%)	1·1
	Lebanon	14 (3·3–38·8)	201·8% (146·8 to 347·3%)	281·8 (67·2–770·7)	-4·4% (-20·7 to 40·6%)	1·18
	Libya	12·2 (3–33·4)	159·2% (106·9 to 231·1%)	305·3 (74·8–829·1)	-5% (-23·6 to 21·6%)	1·12
	Morocco	61·5 (13·9–170·8)	147·5% (105·1 to 196·1%)	274·9 (62·5–760·6)	0·5% (-15·5 to 18·8%)	1·03
	Palestine	4·1 (1–11·9)	111·9% (74 to 165·2%)	276·4 (63·8–791·3)	-6·6% (-22·5 to 16·2%)	1·05
	Oman	2·4 (0·6–6·7)	121·4% (82·1 to 204·3%)	308·6 (71–852·2)	-4·7% (-21·2 to 39·9%)	1·14
	Qatar	0·9 (0·2–2·5)	586·4% (396·5 to 851·4%)	296·7 (70·2–820·8)	3·7% (-16·9 to 28·2%)	1·15
	Saudi Arabia	23·4 (5·6–63·7)	120·5% (73·4 to 215·2%)	280·3 (67–747·3)	-3·2% (-21·1 to 39·5%)	1·1
	Syrian Arab Republic	21·9 (4·8–61·9)	113% (60·9 to 198·9%)	280·2 (65·9–791)	4% (-19·1 to 47·9%)	1·16
	Tunisia	29·9 (7·1–79·8)	208·9% (135·9 to 338·8%)	285·2 (67–745·2)	-3·3% (-25 to 34·3%)	1·09
	Türkiye	220·9 (55·7–628·9)	175·4% (126 to 237·4%)	278·5 (70·2–786·3)	-4·3% (-21·4 to 16·6%)	1·19
	United Arab Emirates	3·1 (0·7–8·9)	606·8% (406·4 to 889·8%)	262·4 (62·9–724·5)	-7·2% (-22·3 to 25·2%)	1·08
	Yemen	22·7 (5·2–64·6)	204·8% (151 to 283·8%)	274·5 (63·9–761·5)	0·5% (-15·7 to 23·6%)	1·07
	Afghanistan	23·4 (5·5–64·8)	52% (22·7 to 79·9%)	328·9 (79·5–892·5)	-4·1% (-20·5 to 10·9%)	1·04
	Sudan	33·9 (7·8–93·2)	105·1% (77·7 to 146·5%)	257 (59·6–730·3)	-5·5% (-17·2 to 11·7%)	1·07
Parkinson's disease	Global	5082·5 (4673·4–5409·9)	123·5% (105·6 to 139·9%)	64·9 (59·6–69·2)	-0·3% (-7·9 to 6·5%)	0·54
	North Africa and Middle East	256·2 (225·1–319·5)	158·7% (125·3 to 202·8%)	72·7 (63·9–91·5)	-1% (-13·1 to 15·8%)	0·67
	Algeria	18·3 (14·4–22·7)	153·4% (90·7 to 234·1%)	67·5 (53·3–83·2)	-21·4% (-39·6 to 2·4%)	0·84
	Bahrain	0·4 (0·2–0·5)	266·9% (169·3 to 389·4%)	75·5 (52·4–93·7)	-16·7% (-37·1 to 7·3%)	0·69
	Egypt	42 (31·6–61·9)	133·4% (80·9 to 196%)	86·6 (65·3–130·4)	11·1% (-12·2 to 39·4%)	1·09
	Iran	40·7 (34·6–44·9)	253% (199·1 to 325·2%)	64·8 (55·1–71·4)	1·4% (-17·1 to 21·9%)	0·67
	Iraq	12·9 (10·3–18·5)	210·7% (139·7 to 303·4%)	75·1 (60·6–104·5)	19·2% (-7·3 to 53·8%)	0·4
	Jordan	2·9 (2·5–3·5)	321·2% (232·5 to 428·2%)	63·3 (52·9–75·2)	-16·4% (-33·5 to 4·8%)	0·67
	Kuwait	0·8 (0·7–1)	202·2% (154·4 to 263%)	46 (37·5–55·1)	-29·8% (-40·8 to -16·3%)	0·44
	Lebanon	3·1 (2·5–4·2)	135·7% (69·9 to 219·5%)	58·7 (46·9–79·9)	-17·4% (-40·2 to 11·5%)	0·56
	Libya	3·2 (2·3–4·2)	206·1% (123·4 to 324·3%)	73 (53·5–95·8)	10·7% (-18·9 to 51·8%)	0·68
	Morocco	22·3 (17·7–26·3)	228·2% (160·1 to 323·9%)	86·1 (69–100·3)	42·6% (14 to 83·9%)	0·58
	Palestine	1·4 (0·9–1·6)	123·3% (67·5 to 219·4%)	82·1 (54·5–95·3)	-5·7% (-29·1 to 33·6%)	0·55
	Oman	0·9 (0·6–1)	160·2% (84 to 258·2%)	109·7 (65–128·8)	30·8% (-5·2 to 77%)	0·5
	Qatar	0·4 (0·3–0·5)	463·5% (290·7 to 692·3%)	132·3 (80·5–177·6)	14·4% (-14·8 to 53·3%)	1·05
	Saudi Arabia	9·7 (7·6–11·5)	133% (72·6 to 247·2%)	96·2 (69·4–113·5)	-1·5% (-25 to 46·7%)	0·37
	Syrian Arab Republic	6·6 (4·3–8·6)	157·3% (86·7 to 255·3%)	72·5 (47·6–93·1)	14·2% (-15·7 to 56·3%)	0·66
	Tunisia	6·9 (5·1–9·4)	206·4% (125 to 308·2%)	61·4 (44·9–82·7)	10·6% (-18·1 to 45·8%)	0·55
	Türkiye	51·9 (39·3–96·4)	136·8% (82·2 to 201%)	63·8 (48·4–119·1)	-12·3% (-31·8 to 10·9%)	0·67
	United Arab Emirates	2·4 (1·6–3·4)	687·2% (456·3 to 1074·3%)	114·3 (70·7–151)	-13% (-33·7 to 23·8%)	0·59
	Yemen	7·1 (5·5–9·7)	218·6% (142·5 to 341·6%)	67·7 (52·8–89·4)	16·4% (-9·6 to 55·9%)	0·64
	Afghanistan	10·5 (7·8–13·7)	51·5% (14·8 to 96·2%)	104·2 (79·3–133·3)	-6·2% (-27·4 to 18·8%)	0·74
	Sudan	11·6 (8·8–15·1)	79·3% (32·6 to 148%)	75·6 (58·5–96·3)	-5% (-29·6 to 30·5%)	0·57
Idiopathic epilepsy	Global	5336·8 (4722·7–6170)	-9·5% (-24·1 to 27·5%)	69·5 (61·7–80·6)	-33·6% (-44 to -7·6%)	0·71
	North Africa and Middle East	352·2 (279·1–412·5)	-18·1% (-37·2 to 39%)	57·3 (45·6–67·2)	-44·4% (-55·6 to -11·7%)	0·89
	Algeria	21·8 (16·7–27·2)	-33·4% (-53·1 to 9%)	51·6 (39·6–64·2)	-54·4% (-67·1 to -27·6%)	1·05
	Bahrain	0·8 (0·6–1)	65% (28·5 to 120·6%)	61·4 (49·5–77·6)	-39·1% (-52 to -18·5%)	0·98
	Egypt	24·4 (16–36·7)	-8·4% (-35·4 to 47·7%)	24 (16·1–35·9)	-42·9% (-59 to -10·4%)	0·62
	Iran	33·4 (30·1–39·3)	-53·1% (-65·8 to -19·2%)	40·2 (36–47·4)	-59% (-69·4 to -34·5%)	0·77

Table S11 Cause- and sex-specific burden, mortality, incidence, and prevalence of neurological conditions in North Africa and Middle East countries

		**All Ages**		**Age-standardised**		
		Number (thousand)	Percent change	Rate per 100,000	Percent change	Sex ratio
**Measure**		2019	From 1990 to 2019	2019	From 1990 to 2019	2019
Cause	Location	Mean (95% UI)	Mean (95% UI)	Mean (95% UI)	Mean (95% UI)	F:M
	Iraq	16·6 (12·4–21·7)	10·7% (-22·1 to 78·1%)	38·7 (29·1–50·1)	-49·1% (-63·3 to -22·8%)	0·98
	Jordan	3·7 (2·8–4·5)	85·1% (36·2 to 165·2%)	30·9 (23·6–37·7)	-40·4% (-55·1 to -15%)	0·89
	Kuwait	1·5 (1·2–1·8)	38·3% (14·1 to 78·1%)	35·5 (29·4–43·8)	-37·1% (-47·8 to -20·3%)	0·88
	Lebanon	2·1 (1·5–3)	-7·7% (-34·7 to 38·7%)	41·5 (29·8–57·5)	-39·6% (-56·7 to -9·9%)	0·76
	Libya	3·8 (2·8–5·1)	-5·7% (-33·4 to 41·7%)	55·4 (39·9–75·2)	-32·9% (-50·9 to -3·5%)	0·86
	Morocco	14·2 (5–37·8)	-5·2% (-40 to 50·5%)	39·2 (13·8–104·9)	-30·8% (-54·5 to 4·4%)	1·42
	Palestine	3·5 (3–4·3)	36·1% (-0·9 to 123%)	71·6 (60·9–86·9)	-36·9% (-52·6 to -3·6%)	0·78
	Oman	0·7 (0·5–0·9)	29·1% (-13·6 to 138·5%)	15 (12·4–19·6)	-43·2% (-60·6 to -5·2%)	0·77
	Qatar	0·6 (0·5–0·8)	237% (146·6 to 406·7%)	22·6 (17·5–30)	-45·8% (-59·9 to -19·9%)	1·56
	Saudi Arabia	29·8 (21·7–41·7)	108·2% (24 to 251·9%)	73·8 (55·9–100·8)	-19·6% (-51·6 to 32·3%)	1·18
	Syrian Arab Republic	4·2 (3·2–5·7)	-25·6% (-49·9 to 35·1%)	29·3 (22·2–39)	-37·3% (-56·7 to 10·3%)	0·82
	Tunisia	4·7 (3·3–6·5)	-42·6% (-62·6 to -8·6%)	41·2 (28·8–57·6)	-50·9% (-67·6 to -23·8%)	0·79
	Türkiye	60·6 (50·2–72·8)	-48·2% (-64·1 to 6·5%)	82·5 (67·7–98·8)	-51·1% (-66·2 to -1·7%)	0·75
	United Arab Emirates	7 (4·3–10·9)	257·4% (124·3 to 474·8%)	64·2 (41·6–96·7)	-36·9% (-57·1 to -3·7%)	0·69
	Yemen	23·8 (15·4–33·8)	12·4% (-28·4 to 172·2%)	71·1 (46·5–100·1)	-36·9% (-58·2 to 23·2%)	0·92
	Afghanistan	56·2 (28·5–79·5)	52·8% (5·4 to 208·8%)	132·6 (65·9–185·9)	-48·4% (-61·9 to -8·1%)	1·17
	Sudan	38·4 (24·1–53·5)	-24·7% (-56 to 107·4%)	84·4 (54·1–116·2)	-52·9% (-71·8 to 6%)	0·77
Multiple sclerosis	Global	708·6 (645·1–897·8)	53·2% (35·6 to 73·7%)	8·5 (7·7–10·7)	-17·4% (-27·9 to -7·6%)	1·38
	North Africa and Middle East	58·2 (45·2–80·4)	126·5% (55·9 to 238%)	9·9 (7·8–13·6)	1·8% (-27·9 to 52·7%)	1·34
	Algeria	4 (2–5·8)	144·3% (39·6 to 288%)	9·3 (4·6–13·5)	0·1% (-42·7 to 55·6%)	1·6
	Bahrain	0·1 (0–0·1)	412·5% (190·6 to 618·1%)	4·1 (2·5–5·5)	-7·1% (-44·4 to 28%)	1·51
	Egypt	9·5 (4·4–30)	73·8% (-47·9 to 495·6%)	9·7 (4·7–26·4)	-1·1% (-64·7 to 191·7%)	1·4
	Iran	14·9 (12·3–22)	152·2% (86·7 to 244·2%)	16·2 (13·4–23·5)	5·1% (-26·7 to 48·4%)	1·12
	Iraq	1·6 (1·1–2·3)	219·6% (103·3 to 437·3%)	4·7 (3·2–6·7)	0·8% (-35·9 to 66·6%)	1·17
	Jordan	0·7 (0·4–0·9)	297% (146 to 459%)	6·9 (4·3–8·9)	-15·7% (-45·8 to 17·1%)	1·71
	Kuwait	0·2 (0·1–0·3)	282·6% (188·1 to 408·8%)	3·9 (2·9–5·5)	-1·4% (-26·3 to 31·7%)	1·12
	Lebanon	0·5 (0·3–0·8)	116% (38·7 to 240·2%)	9·9 (5·1–14·5)	7·4% (-32 to 68·3%)	1·3
	Libya	0·8 (0·4–1·4)	276·4% (125·3 to 571%)	11·3 (5·5–19)	39·3% (-20 to 143·8%)	1·33
	Morocco	3·6 (1·8–5·7)	151·9% (53·5 to 315·9%)	9·7 (4·9–15·2)	26·3% (-22 to 105·7%)	1·54
	Palestine	0·3 (0·3–0·5)	216·9% (81·8 to 464·4%)	9 (6·9–12·8)	0% (-39·5 to 73·8%)	1·00
	Oman	0·3 (0·2–0·5)	258·7% (74·9 to 559·7%)	8·2 (3·9–13·5)	10·8% (-46·2 to 103·4%)	1·33
	Qatar	0·1 (0·1–0·1)	594·9% (327·6 to 922·8%)	2·7 (1·9–3·7)	-18·7% (-49·7 to 21·5%)	1·5
	Saudi Arabia	1·7 (1·2–2·6)	365·1% (151·5 to 1122·9%)	4·3 (3–6·2)	19·2% (-34·5 to 205·4%)	1·13
	Syrian Arab Republic	0·6 (0·4–0·9)	104·2% (23 to 241·6%)	4·4 (3–6)	1·1% (-37·4 to 69·5%)	1·31
	Tunisia	1·3 (0·6–1·9)	150·6% (52·4 to 321·6%)	9·5 (4·8–14·4)	23·7% (-25·4 to 102·9%)	1·42
	Türkiye	7·7 (5·5–11·2)	30·3% (-42·8 to 137·8%)	8·5 (5·9–13·9)	-27% (-62·8 to 40·7%)	1·61
	United Arab Emirates	2 (0·9–3·6)	1081·6% (551·8 to 1757·5%)	14·3 (6–26·2)	31% (-26·2 to 94·8%)	1·01
	Yemen	1·7 (0·8–3·1)	281·2% (93·8 to 661·2%)	7·9 (3·8–12·8)	30·5% (-29·5 to 133·4%)	1·16
	Afghanistan	4 (1·4–9·8)	224·7% (96·9 to 486·4%)	16·3 (6·7–36·8)	6·8% (-31·4 to 74·9%)	1·81
	Sudan	2·4 (1–4·1)	175·4% (41·8 to 425·8%)	7·9 (3·5–12·6)	18·8% (-35·9 to 113·6%)	1·15
Motor neuron disease	Global	977·5 (926·3–1025·4)	65·5% (54·5 to 78·2%)	11·9 (11·3–12·5)	-4·9% (-10·7 to 1·9%)	0·71
	North Africa and Middle East	38·3 (30·6–47·1)	18·4% (-32·8 to 87·7%)	7·3 (5·8–9)	-17·8% (-47·1 to 19·1%)	0·72
	Algeria	0·8 (0·6–1·1)	123·9% (53·5 to 228·4%)	2 (1·5–2·7)	-3% (-34·5 to 46·4%)	1·23
	Bahrain	<0·1 (<0·1–<0·1)	86% (35·2 to 150·7%)	1·5 (1·2–1·8)	-62·5% (-71·9 to -50·5%)	1·24
	Egypt	6 (3·3–9·6)	237·5% (78·8 to 465·4%)	7·5 (4·1–11·9)	76·4% (-5·4 to 195·5%)	0·67
	Iran	3·3 (2·9–3·8)	191·4% (89·1 to 307·9%)	4·1 (3·6–4·7)	51·5% (0·1 to 113·1%)	0·9
	Iraq	0·4 (0·3–0·6)	197·8% (78·6 to 360·2%)	1·3 (0·9–1·8)	14·2% (-35·6 to 91%)	1·2
	Jordan	0·3 (0·2–0·4)	493·6% (282·5 to 804·1%)	2·6 (2–3·6)	45·7% (-6·6 to 129·7%)	0·47
	Kuwait	0·2 (0·1–0·2)	-23·4% (-47·6 to 7·8%)	5·3 (3·7–6·8)	-64·4% (-75·2 to -50·4%)	0·33

Table S11 Cause- and sex-specific burden, mortality, incidence, and prevalence of neurological conditions in North Africa and Middle East countries

		All Ages		Age-standardised		
		Number (thousand)	Percent change	Rate per 100,000	Percent change	Sex ratio
**Measure**		2019	From 1990 to 2019	2019	From 1990 to 2019	2019
Cause	Location	Mean (95% UI)	Mean (95% UI)	Mean (95% UI)	Mean (95% UI)	F:M
	Lebanon	0·1 (0·1–0·2)	96·5% (12·6 to 215·5%)	2·7 (1·6–4·4)	4·9% (-40·2 to 68·1%)	0·85
	Libya	0·2 (0·1–0·3)	260% (70·3 to 566·3%)	3·2 (1·8–4·9)	50·1% (-29·3 to 183·3%)	1·03
	Morocco	0·7 (0·5–1)	158·1% (67·8 to 277·2%)	2 (1·4–2·7)	30·1% (-17·5 to 90·3%)	1·13
	Palestine	0·1 (0–0·1)	239% (117·3 to 435·5%)	1·5 (1·2–1·9)	45·5% (-11·5 to 143·9%)	1·72
	Oman	0·1 (0–0·1)	218·5% (67·6 to 419·9%)	2·7 (1·4–3·9)	20·7% (-36·9 to 109·9%)	1·39
	Qatar	<0·1 (<0·1–<0·1)	612·3% (342·6 to 1009·8%)	1·5 (1·1–2·1)	-7·9% (-45·3 to 50·3%)	1·45
	Saudi Arabia	0·7 (0·5–1·1)	247·2% (68·1 to 594·8%)	2·6 (1·8–3·7)	22·5% (-40·3 to 166·4%)	0·89
	Syrian Arab Republic	0·3 (0·2–0·6)	138·8% (32·1 to 304·8%)	2·3 (1·4–3·8)	31·1% (-26·3 to 121·1%)	0·87
	Tunisia	0·3 (0·2–0·4)	135·3% (34·9 to 295·4%)	2·1 (1·4–3)	19·7% (-31·3 to 104%)	0·88
	Türkiye	22·9 (18–28·8)	-15·7% (-55·2 to 50·7%)	31 (23·8–39·6)	-25·6% (-58·5 to 21·2%)	0·62
	United Arab Emirates	0·5 (0·2–1)	1091·1% (375·2 to 2343·4%)	5·3 (2–10·6)	24·9% (-47·6 to 142·8%)	0·99
	Yemen	0·3 (0·2–0·4)	214·8% (96·5 to 413·9%)	1·4 (1–2·1)	26·1% (-27·5 to 120·6%)	1·18
	Afghanistan	0·5 (0·4–0·7)	126·7% (53·4 to 236·3%)	2·2 (1·5–3·2)	-8·2% (-39·2 to 41·1%)	1·34
	Sudan	0·5 (0·3–0·7)	143·8% (50·9 to 286·3%)	1·7 (1·1–2·5)	20·5% (-28 to 97·1%)	1·14
Other neurological disorders	Global	2133 (1915·2–2381·1)	31·4% (16·2 to 49·6%)	27·7 (24·7–31)	-8·9% (-19·1 to 3·5%)	0·72
	North Africa and Middle East	147·4 (127·9–171·8)	28·8% (-3·1 to 67·3%)	25·7 (22·4–29·9)	-9·5% (-30 to 15·6%)	0·61
	Algeria	7·4 (6·2–8·9)	43·2% (-7·6 to 100·8%)	18·3 (15·2–21·7)	-5·3% (-35·7 to 28·1%)	0·72
	Bahrain	0·4 (0·3–0·4)	122·4% (70·4 to 191·1%)	32·2 (26·9–38·3)	-6·8% (-26·3 to 19·5%)	0·84
	Egypt	22·2 (15–31·6)	73·1% (17 to 150·5%)	24·8 (16·8–35·2)	19·9% (-17·1 to 65·9%)	0·39
	Iran	16·9 (15·7–18·2)	61·1% (18·9 to 116·6%)	21·1 (19·4–22·6)	20·8% (-8·5 to 62·8%)	0·66
	Iraq	11·8 (9–14·9)	113·1% (48·2 to 236·7%)	27·5 (21·2–34·5)	6% (-24·3 to 66·1%)	1·02
	Jordan	2·5 (2·1–3·1)	150·6% (78·6 to 250·6%)	22·1 (18–27·1)	-4·5% (-29·8 to 29·7%)	0·74
	Kuwait	0·6 (0·5–0·8)	41·3% (4·8 to 85·4%)	17·1 (13·7–21·3)	-31·5% (-48·1 to -10·8%)	0·66
	Lebanon	1·2 (0·9–1·6)	115·2% (50 to 201·4%)	23·4 (16·9–31·7)	37·4% (-3·3 to 90·7%)	0·4
	Libya	1·7 (1·3–2·2)	109·8% (36 to 234·8%)	25·6 (19·3–33·1)	39·9% (-6·8 to 117·6%)	0·54
	Morocco	6·3 (4·6–8·6)	58·9% (12·2 to 128·7%)	18·2 (13·1–24·5)	21·5% (-12·2 to 69·2%)	0·56
	Palestine	1 (0·8–1·2)	79·5% (13·7 to 211·1%)	20 (16·6–23·7)	-0·7% (-33·2 to 67·7%)	0·57
	Oman	0·7 (0·5–0·8)	171·9% (63·8 to 318·8%)	18·7 (15–22·9)	45·4% (-9·7 to 120·5%)	0·97
	Qatar	0·5 (0·4–0·6)	273·2% (153·8 to 481·7%)	27 (20·7–35·8)	-18·7% (-43·7 to 23·2%)	1·57
	Saudi Arabia	6·8 (5·2–9)	74·6% (5·7 to 198·8%)	22 (17·5–27·6)	-3·2% (-39·1 to 61·5%)	1·07
	Syrian Arab Republic	3 (2·3–4)	-17·4% (-46·6 to 33·4%)	22 (16·8–28·7)	-4·7% (-35 to 50·1%)	0·89
	Tunisia	2 (1·4–2·7)	40% (-9·2 to 106·1%)	17·1 (12·3–23·3)	8·7% (-28·7 to 55·2%)	0·56
	Türkiye	34·2 (27·9–41·9)	-34·8% (-54·1 to -5·4%)	46·7 (38·8–56·7)	-38·7% (-56·1 to -12·2%)	0·64
	United Arab Emirates	5·3 (3·2–8)	836·4% (460 to 1357·3%)	54·5 (33·9–79·3)	52·7% (-2·2 to 120·7%)	0·33
	Yemen	4·2 (2·8–6·1)	106% (15·6 to 271·1%)	14·4 (10·1–20·4)	19% (-25·5 to 104·8%)	0·73
	Afghanistan	8·4 (5·9–11·7)	146·6% (63·7 to 282·3%)	22 (16·4–30·1)	-8·4% (-35·7 to 34%)	0·64
	Sudan	10·1 (6·3–15·1)	108·2% (7·4 to 272·2%)	24·3 (15·7–35·3)	34·3% (-24·9 to 114·5%)	0·42

Data in parentheses are 95% uncertainty intervals. Count data in thousands and percentage data are rounded to one decimal place, and sex ratio is rounded to two decimal place. Percentages and number of DALYs, YLDs, YLLs, deaths, incident cases, and prevalent cases are not mutually exclusive: the sum of percentages and number of DALYs, YLDs, YLLs, deaths, incident cases, and prevalent cases in the columns exceeds the totals for all causes combined because of overlap between various causes. Measures with insufficient data are not reported here. DALYs Disability-Adjusted Life Years; F:M, Female to male ratio; NA, no data available; UI uncertainty interval; YLDs Years Lived with Disability; YLLs Years of Life Lost; *Stroke include ischaemic stroke, intracerebral haemorrhage, and subarachnoid haemorrhage; †Neurological disorders include Alzheimer's disease and other dementias, Parkinson's disease, idiopathic epilepsy, multiple sclerosis, headache disorders (including migraine and tension-type headache), motor neuron disease, and other neurological disorders.

Table S12 Burden, mortality, incidence, and prevalence of neurological conditions in North Africa and Middle East countries
(NEXT PAGE)

Disability-Adjusted Life Years 196
Deaths 205
Incidence 214
Prevalence 224
Years Lived with Disability 235
Years of Life Lost 245

Table S12 Burden, mortality, incidence, and prevalence of neurological conditions in North Africa and Middle East countries

		All Ages		Age-standardised	
		Number	Percent change	Rate per 100,000	Percent change
**Measure**		2019	From 1990 to 2019	2019	From 1990 to 2019
Cause	Location	Mean (95% UI)	Mean (95% UI)	Mean (95% UI)	Mean (95% UI)
**DALYs (Disability-Adjusted Life Years)**					
Global	Meningitis	16333198 (13775122–19609767)	-51·3% (-59·4 to -42%)	234 (195·8–282·5)	-57·2% (-64·4 to -48·6%)
	Encephalitis	4797407 (4059493–6418088)	-43·4% (-55·8 to -15·8%)	65·3 (55·1–87·3)	-54·3% (-63·9 to -32·9%)
	Tetanus	2316381 (1770002–3279408)	-89·4% (-92·1 to -83·8%)	33·7 (25·6–47·9)	-90·2% (-92·7 to -85·1%)
	Brain and central nervous system cancer	8659871 (6718029–9574458)	40·5% (-13·2 to 66·9%)	109 (84·6–120·9)	-10·4% (-43·5 to 5·3%)
	Stroke*	143232184 (133095809–153241824)	32·4% (22 to 42·2%)	1768·1 (1640·6–1889·4)	-35·2% (-40·5 to -30·5%)
	Ischaemic stroke	63478272 (57827413–68986524)	56·7% (43·3 to 67·9%)	798·8 (727·5–866·9)	-28·5% (-34·7 to -23·2%)
	Intracerebral haemorrhage	68572498 (63272310–73681973)	25·3% (12·2 to 36·3%)	832·8 (769·2–894·7)	-36·7% (-43·3 to -31%)
	Subarachnoid haemorrhage	11181414 (9893414–12668877)	-13·8% (-25·7 to 17%)	136·5 (120·8–154·7)	-54·2% (-60·6 to -37·5%)
	Neurological disorders†	97724412 (55942819–159416793)	69·9% (58·7 to 90%)	1253·6 (719·7–2039·8)	-0·8% (-5·1 to 3·1%)
	Alzheimer's disease and other dementias	25276989 (11204523–54558243)	161·6% (149·7 to 175·7%)	338·6 (151–731·3)	3·7% (-0·3 to 8·1%)
	Parkinson's disease	6292616 (5769210–6827207)	128·9% (113·3 to 142·5%)	80 (73·3–86·6)	2·4% (-4·4 to 8·2%)
	Idiopathic epilepsy	13077624 (9986730–16734086)	15·9% (0 to 42·1%)	170·6 (130·4–218·3)	-16·5% (-27·4 to 1·7%)
	Multiple sclerosis	1159832 (1001180–1381870)	59·7% (46·6 to 72·7%)	14 (12–16·6)	-13·2% (-20·9 to -6·3%)
	Migraine	42077666 (6418383–95645211)	56·6% (52·6 to 62·1%)	525·5 (78·8–1194)	1·5% (-4·4 to 3·3%)
	Tension-type headache	4541689 (1395546–14981336)	57·8% (45·1 to 65·9%)	56·2 (17–188·5)	-2·5% (-5·4 to 1·1%)
	Motor neuron disease	1034607 (979911–1085401)	65·7% (55·5 to 77·7%)	12·7 (12–13·3)	-4·5% (-10·1 to 1·9%)
	Other neurological disorders	4263390 (3458864–5174136)	56·5% (41·8 to 74·3%)	55·9 (45·2–68·3)	10·8% (0·4 to 22·9%)
	Headache disorders	46619355 (9772903–100161726)	56·7% (52·4 to 62·1%)	581·8 (119·6–1255·6)	1·1% (-4·2 to 2·9%)
North Africa and Middle East	Meningitis	412772 (345204–495004)	-70·5% (-77·5 to -62·3%)	70 (58·7–83·7)	-75·8% (-81·1 to -69·5%)
	Encephalitis	251049 (200161–332874)	13·5% (-21 to 59·6%)	42 (33·6–55·6)	-20·7% (-43·3 to 8·7%)
	Tetanus	87412 (53608–139520)	-85·8% (-92·9 to -68·7%)	15 (9·2–24·1)	-88·2% (-93·9 to -76·1%)
	Brain and central nervous system cancer	716271 (493932–848226)	71% (0·9 to 131·7%)	128·3 (87·8–151·3)	-5% (-40 to 23·4%)
	Stroke*	7946004 (7060209–8870766)	43·3% (27·2 to 61·4%)	1826·2 (1635·3–2026·2)	-32% (-39·1 to -23·3%)
	Ischaemic stroke	4751376 (4227735–5271899)	120% (89 to 148·1%)	1183·6 (1060·8–1307)	-8·8% (-19·6 to 2·2%)
	Intracerebral haemorrhage	2702244 (2343718–3120307)	3·4% (-12·3 to 21·4%)	548·4 (479·5–623·1)	-51·7% (-58·2 to -43·8%)
	Subarachnoid haemorrhage	492385 (408280–617218)	-36% (-50·6 to 6·8%)	94·2 (79–116·8)	-62·8% (-71·6 to -41%)
	Neurological disorders†	7156041 (3769688–12184398)	91·1% (72 to 109·7%)	1382·1 (776·6–2273·8)	-3·9% (-10·3 to 3·5%)
	Alzheimer's disease and other dementias	1208064 (532026–2672826)	177·3% (161·1 to 215·8%)	387 (172–848·5)	-1·1% (-6·6 to 12·1%)
	Parkinson's disease	300698 (266277–365360)	163·8% (134·8 to 202·1%)	84·4 (74·7–103·2)	0·9% (-10·2 to 15·7%)
	Idiopathic epilepsy	955271 (682842–1293169)	13·8% (-13·8 to 56%)	158·3 (112·6–213·2)	-26·2% (-43·6 to -1·1%)
	Multiple sclerosis	115886 (93053–144758)	146% (98·9 to 205·5%)	19·9 (16·1–24·7)	6·2% (-11·6 to 30·5%)
	Migraine	3793207 (645291–8665809)	102·1% (93·2 to 125·1%)	601·4 (107–1371·8)	0% (-1·6 to 1·6%)
	Tension-type headache	416595 (138258–1196825)	115·5% (84·9 to 132·3%)	68·1 (22·8–195·5)	1% (-9·5 to 8·7%)

Table S12 Burden, mortality, incidence, and prevalence of neurological conditions in North Africa and Middle East countries

		All Ages		Age-standardised	
		Number	Percent change	Rate per 100,000	Percent change
**Measure**		2019	From 1990 to 2019	2019	From 1990 to 2019
Cause	Location	Mean (95% UI)	Mean (95% UI)	Mean (95% UI)	Mean (95% UI)
	Motor neuron disease	41628 (33815–50605)	22·5% (-28·9 to 88·7%)	7·8 (6·4–9·5)	-16·6% (-44·8 to 17·9%)
	Other neurological disorders	324691 (250401–417325)	59·4% (26·5 to 98·9%)	55·2 (43–70·4)	6·3% (-13·5 to 30·6%)
	Headache disorders	4209802 (990143–9068319)	103·3% (94·5 to 123·7%)	669·6 (159·1–1431·3)	0·1% (-2·4 to 2·4%)
Algeria	Meningitis	16224 (13337–20098)	-74·7% (-84·2 to -61·1%)	40 (33·1–49·1)	-80·2% (-87 to -70·7%)
	Encephalitis	8284 (5881–14740)	-2·5% (-38·8 to 49·8%)	20·1 (14·4–35·4)	-28·7% (-52·4 to 3·6%)
	Tetanus	540 (393–943)	-83% (-93·5 to -41·9%)	1·3 (0·9–2·3)	-86·7% (-94·8 to -60·7%)
	Brain and central nervous system cancer	23674 (14317–30164)	56·3% (-16·5 to 120·5%)	58·3 (35·3–73·9)	-6% (-49·9 to 28·7%)
	Stroke*	543945 (450965–649230)	47% (18·7 to 82·1%)	1755·4 (1459·6–2081·9)	-44·6% (-55 to -31·9%)
	Ischaemic stroke	372749 (302683–448803)	126·7% (81·1 to 182·3%)	1275·1 (1052·8–1530·2)	-24·7% (-38·7 to -8·3%)
	Intracerebral haemorrhage	142325 (112671–177630)	-16·1% (-35·6 to 10·1%)	403·4 (322·4–501·3)	-67·9% (-75·6 to -58·4%)
	Subarachnoid haemorrhage	28872 (22118–38126)	-19·8% (-42·2 to 28%)	76·8 (58·2–100·2)	-64·6% (-74·6 to -44·4%)
	Neurological disorders†	494778 (254585–842878)	82·8% (47·3 to 123·8%)	1365·1 (738·5–2292)	-8·4% (-21·4 to 5%)
	Alzheimer's disease and other dementias	95573 (41570–217272)	228·9% (176·4 to 291·6%)	401·3 (176·1–912·8)	-5% (-17·6 to 8·7%)
	Parkinson's disease	21708 (17579–26334)	161·4% (106 to 229·3%)	78·9 (64·1–95·1)	-18·1% (-35·7 to 3·6%)
	Idiopathic epilepsy	60483 (29802–101772)	-8·6% (-58·6 to 82·6%)	146·2 (70·8–246·9)	-38% (-72·5 to 25·8%)
	Multiple sclerosis	8523 (5733–11226)	178·8% (106·5 to 257·5%)	20·1 (13·6–26·4)	12·2% (-14·2 to 41·1%)
	Migraine	259963 (44334–590017)	89% (76·7 to 120·2%)	602·5 (103·9–1372·1)	-0·2% (-3·2 to 3·1%)
	Tension-type headache	28460 (9650–79524)	106·7% (73·1 to 140·3%)	66·7 (22·6–189)	0·3% (-10·2 to 7·9%)
	Motor neuron disease	1023 (806–1285)	115·9% (60·6 to 191·1%)	2·5 (2–3·2)	-2% (-28·3 to 36·3%)
	Other neurological disorders	19045 (11271–30215)	53·7% (-21·7 to 206·7%)	46·8 (28·1–73·9)	4·8% (-45·2 to 93·8%)
	Headache disorders	288424 (67480–615898)	90·7% (78·3 to 119·7%)	669·2 (155·7–1442·3)	-0·1% (-3·5 to 3·1%)
Bahrain	Meningitis	208 (175–248)	-42·7% (-55·7 to -26·8%)	18·6 (15·8–21·8)	-74·5% (-79·6 to -67·8%)
	Encephalitis	141 (113–171)	69·5% (28·5 to 110·5%)	11·4 (9·2–13·8)	-25·8% (-43·7 to -8·4%)
	Tetanus	10 (8–14)	37·3% (-40·9 to 115%)	0·7 (0·6–1·2)	-39·3% (-75 to -6·6%)
	Brain and central nervous system cancer	1022 (618–1352)	186·4% (67·5 to 302·3%)	73 (46·7–93·3)	-25% (-55·8 to 6·5%)
	Stroke*	7987 (6663–9690)	111% (71·2 to 164·3%)	940·9 (796·8–1146·2)	-52·5% (-61·2 to -42·5%)
	Ischaemic stroke	4155 (3482–5019)	165·2% (120·6 to 220%)	613·6 (521·7–746·7)	-43·9% (-53·5 to -33%)
	Intracerebral haemorrhage	2952 (2394–3662)	78·6% (36·6 to 133·3%)	264·6 (217·2–326·5)	-64·2% (-72 to -54·2%)
	Subarachnoid haemorrhage	880 (677–1193)	55·4% (12·2 to 135·2%)	62·6 (48·6–85·6)	-58·1% (-70·1 to -41·3%)
	Neurological disorders†	16094 (7722–28555)	199·3% (134·7 to 269·8%)	1387·6 (768·6–2266·4)	-8% (-23·9 to 8·1%)
	Alzheimer's disease and other dementias	1694 (733–3909)	398·1% (321·9 to 486·1%)	403·1 (176·2–888·6)	-3·8% (-15 to 9·1%)
	Parkinson's disease	467 (340–573)	299·2% (213·3 to 401·9%)	88·5 (64·1–106·6)	-13·5% (-32·1 to 7·5%)
	Idiopathic epilepsy	2334 (1088–4053)	83·1% (-17·9 to 303·1%)	186·8 (86·8–326·3)	-31·7% (-70 to 51·3%)
	Multiple sclerosis	232 (166–309)	446% (337·3 to 558%)	12·5 (9·1–16·2)	12·3% (-7·6 to 32·6%)
	Migraine	9458 (1762–21196)	219·3% (197 to 279·3%)	564·5 (100·5–1278·1)	-0·7% (-3·9 to 2·8%)
	Tension-type headache	1117 (386–3084)	254·2% (197·7 to 330·7%)	64·6 (21·3–189·3)	-0·3% (-10·7 to 7·7%)
	Motor neuron disease	33 (27–40)	114·5% (70·4 to 169·7%)	2·1 (1·7–2·5)	-54·1% (-64·1 to -42·6%)
	Other neurological disorders	759 (484–1160)	145% (44 to 321·6%)	65·6 (41·9–99·2)	0·3% (-39·8 to 65·6%)
	Headache disorders	10575 (2641–22348)	222·7% (200·3 to 275·9%)	629 (149·4–1360·3)	-0·6% (-3·9 to 2·8%)
Egypt	Meningitis	54627 (37527–74295)	-68·1% (-78·3 to -54·2%)	54 (37·4–73·2)	-75·7% (-83·3 to -65·6%)
	Encephalitis	65047 (33529–102544)	-4·1% (-43·1 to 56%)	62·8 (33·5–98·7)	-31·9% (-58·4 to 4·5%)
	Tetanus	7406 (2206–21957)	-87·3% (-96·4 to -60·1%)	7·7 (2·5–23·2)	-90·8% (-97 to -71·7%)
	Brain and central nervous system cancer	89056 (61392–125531)	73·5% (-5·9 to 153·7%)	98·8 (68·1–141·1)	-1·8% (-40·8 to 39·9%)

Table S12 Burden, mortality, incidence, and prevalence of neurological conditions in North Africa and Middle East countries

		All Ages		Age-standardised	
		Number	Percent change	Rate per 100,000	Percent change
**Measure**		2019	From 1990 to 2019	2019	From 1990 to 2019
Cause	Location	Mean (95% UI)	Mean (95% UI)	Mean (95% UI)	Mean (95% UI)
	Stroke*	1493970 (1146402–1946512)	-7·4% (-31·5 to 33·5%)	2138 (1636·8–2809·8)	-35·5% (-50 to -17%)
	Ischaemic stroke	863083 (669024–1115487)	99% (43·1 to 170·4%)	1381·5 (1070·2–1804·1)	13·7% (-15·5 to 43·8%)
	Intracerebral haemorrhage	525945 (380648–714428)	-36·5% (-56·7 to -7·1%)	641·8 (460·5–884·3)	-59·8% (-70·7 to -45·5%)
	Subarachnoid haemorrhage	104943 (74559–145019)	-70·2% (-82·6 to -30·8%)	114·7 (82·7–158)	-77·4% (-85·6 to -52·9%)
	Neurological disorders†	1050487 (470197–1877241)	92% (55·8 to 126·9%)	1336·2 (709·8–2284·8)	0·9% (-13·5 to 15·6%)
	Alzheimer's disease and other dementias	135572 (59535–305476)	112·3% (83 to 155·6%)	362·4 (160·1–793·2)	0·2% (-12·4 to 18·8%)
	Parkinson's disease	48343 (37833–67875)	135·2% (88·1 to 190·9%)	98·6 (77·1–142·4)	11·7% (-9·4 to 36·5%)
	Idiopathic epilepsy	111651 (44159–202534)	34·2% (-51·1 to 227·5%)	110·6 (43·3–200)	-19·5% (-70·4 to 99·5%)
	Multiple sclerosis	14335 (8608–35878)	95·5% (-16·2 to 400·6%)	15·3 (9·6–32·4)	6·4% (-44·2 to 137·3%)
	Migraine	619621 (96677–1426392)	98·2% (84·3 to 113·8%)	621·1 (104–1420·2)	1·9% (-5·1 to 9·1%)
	Tension-type headache	65173 (20989–196744)	100·8% (75 to 120·6%)	69·1 (22·6–207·8)	1·4% (-10·1 to 10·8%)
	Motor neuron disease	6483 (3807–10066)	221·4% (80·1 to 420·3%)	7·9 (4·6–12·4)	69·4% (-4·4 to 176·8%)
	Other neurological disorders	49309 (28736–76336)	96·3% (6·1 to 252·8%)	51·2 (31·7–76)	25·2% (-28·1 to 108·4%)
	Headache disorders	684794 (147091–1491960)	98·4% (84·9 to 112·8%)	690·3 (156·4–1488·7)	1·9% (-4·8 to 8·6%)
Iran (Islamic Republic of)	Meningitis	30092 (26169–34399)	-79% (-84·1 to -71·6%)	38·6 (33·5–44·3)	-79·2% (-84·1 to -72·5%)
	Encephalitis	12690 (10236–14589)	-13·4% (-37·5 to 14·3%)	16·3 (12·8–19)	-22·1% (-41·5 to -0·8%)
	Tetanus	911 (693–1254)	-94·4% (-97·6 to -82·1%)	1·2 (0·9–1·7)	-94·1% (-97·4 to -82·3%)
	Brain and central nervous system cancer	128547 (67682–153606)	40·5% (-27·9 to 81·4%)	156·4 (82–187)	-10·7% (-50·1 to 8·5%)
	Stroke*	884768 (812248–943655)	48·2% (33·4 to 63·4%)	1262·2 (1153·5–1346·3)	-45·7% (-51 to -38·3%)
	Ischaemic stroke	668904 (607976–717571)	68·5% (51·1 to 87·3%)	984·4 (890–1054·7)	-42·8% (-48·2 to -34·9%)
	Intracerebral haemorrhage	172626 (162359–183216)	6·8% (-6·5 to 20·4%)	223·3 (209·6–236·3)	-53·6% (-59·9 to -47·4%)
	Subarachnoid haemorrhage	43238 (38360–50622)	12·4% (-15·5 to 70·6%)	54·6 (48·5–62·6)	-55·7% (-68·1 to -31·7%)
	Neurological disorders†	1103459 (584174–1853014)	80·4% (60·7 to 111·9%)	1394·2 (785·8–2300·9)	-4·1% (-10·3 to 4·7%)
	Alzheimer's disease and other dementias	228128 (100792–497824)	314·3% (277 to 401·2%)	386 (170·3–844)	0% (-5·4 to 21·3%)
	Parkinson's disease	48779 (42378–53932)	253·3% (207·9 to 313·5%)	76·8 (66·9–84·9)	3·1% (-12·9 to 20·5%)
	Idiopathic epilepsy	113267 (78371–155611)	-22·8% (-43·4 to 6·4%)	140·2 (96·1–192·5)	-34·8% (-51·4 to -13%)
	Multiple sclerosis	26915 (21772–34918)	143·4% (103·1 to 191·7%)	29·2 (23·7–37·4)	0·5% (-18·6 to 20·3%)
	Migraine	571655 (107029–1282201)	77·6% (63·7 to 117·4%)	628·3 (115·2–1411·2)	-0·3% (-3·6 to 4·1%)
	Tension-type headache	70182 (23015–207136)	103·8% (70·9 to 131·5%)	77·6 (24·9–236·4)	5·2% (-5·7 to 15·9%)
	Motor neuron disease	3832 (3359–4363)	170·1% (88·7 to 257·6%)	4·7 (4·1–5·4)	43·7% (1 to 90·3%)
	Other neurological disorders	40701 (31189–51857)	48·5% (19·8 to 84·1%)	51·5 (39·2–66·3)	20·2% (-2·5 to 47·4%)
	Headache disorders	641837 (160733–1355804)	80·2% (66·5 to 117·2%)	705·8 (173·2–1503·1)	0·3% (-3·3 to 5·8%)
Iraq	Meningitis	28390 (22192–36269)	-69·6% (-79 to -56·4%)	67·4 (53·4–84·6)	-80% (-85·7 to -72·4%)
	Encephalitis	30409 (21118–40443)	17·8% (-27·9 to 88·4%)	70·5 (50·3–92·5)	-33·8% (-56·9 to -2%)
	Tetanus	1783 (1014–3003)	-91·2% (-97·3 to -53·5%)	4 (2·3–6·7)	-93·8% (-98 to -72·8%)
	Brain and central nervous system cancer	79865 (57728–103168)	164·2% (32 to 325·9%)	225·6 (162·9–289)	15·2% (-39·2 to 79·6%)
	Stroke*	682943 (548026–826273)	117·8% (72 to 174·5%)	2922·9 (2399–3459·1)	-19·4% (-35·5 to -0·8%)
	Ischaemic stroke	373159 (305766–438919)	153·9% (101·6 to 214·7%)	1828·9 (1525·3–2123·6)	-7% (-25·1 to 13·7%)
	Intracerebral haemorrhage	287202 (221961–364476)	99·7% (51·7 to 165·7%)	1022 (808·4–1271·1)	-30% (-47 to -9·1%)
	Subarachnoid haemorrhage	22582 (16870–30599)	-1·1% (-34·8 to 60·2%)	72·1 (54·4–97·2)	-64·1% (-76·6 to -41·4%)
	Neurological disorders†	437763 (204665–786450)	153·1% (104·7 to 201·9%)	1331·8 (715·6–2250·9)	-1·7% (-14 to 12·3%)
	Alzheimer's disease and other dementias	57498 (25205–128176)	157·1% (125 to 195·1%)	382 (168·2–826·8)	1·9% (-9·7 to 16·3%)

Table S12 Burden, mortality, incidence, and prevalence of neurological conditions in North Africa and Middle East countries

		All Ages		Age-standardised	
		Number	Percent change	Rate per 100,000	Percent change
**Measure**		2019	From 1990 to 2019	2019	From 1990 to 2019
Cause	Location	Mean (95% UI)	Mean (95% UI)	Mean (95% UI)	Mean (95% UI)
	Parkinson's disease	14973 (12204–20254)	209·1% (147·3 to 290·7%)	85·6 (71–115·2)	17·5% (-5·7 to 47·1%)
	Idiopathic epilepsy	56263 (24821–98677)	59·3% (-31·4 to 252·4%)	131 (57·8–229·5)	-29·4% (-69·3 to 55·2%)
	Multiple sclerosis	4893 (3596–6482)	254% (186·9 to 340·3%)	14·3 (10·6–18·6)	12·4% (-8·4 to 39·2%)
	Migraine	253176 (40135–580495)	181·6% (170·8 to 206·2%)	595·4 (102·8–1355·6)	0·3% (-2·8 to 3·2%)
	Tension-type headache	26343 (8663–77009)	193·4% (151·1 to 230·5%)	66·2 (22·1–190·3)	0·7% (-11·1 to 9·1%)
	Motor neuron disease	640 (495–796)	188% (107·4 to 289·5%)	1·8 (1·4–2·3)	10·6% (-27 to 58·4%)
	Other neurological disorders	23978 (15008–36485)	140·8% (37·7 to 315·4%)	55·4 (36·1–82·1)	14·4% (-32·3 to 91·1%)
	Headache disorders	279519 (60086–621233)	182·7% (170·9 to 204·1%)	661·6 (153–1427·4)	0·3% (-3·3 to 3·5%)
Jordan	Meningitis	5929 (4734–7496)	5% (-25·9 to 50·7%)	52·6 (42·4–66·4)	-56·5% (-68 to -39·5%)
	Encephalitis	1262 (993–1679)	2·4% (-35·5 to 154·6%)	10·9 (8·6–14·6)	-60·2% (-74·1 to -9·7%)
	Tetanus	118 (87–174)	-71·7% (-86·8 to -12·3%)	1 (0·7–1·5)	-85·7% (-93·3 to -59%)
	Brain and central nervous system cancer	9737 (6956–12042)	202·2% (91·8 to 309·7%)	97·8 (69·5–120·6)	-10·2% (-43·7 to 22·2%)
	Stroke*	86118 (72852–100150)	133·8% (94 to 178·9%)	1448 (1220·4–1679·5)	-50·2% (-58·7 to -41·1%)
	Ischaemic stroke	59926 (50568–69639)	161·4% (114·5 to 207·5%)	1083·5 (911·7–1252·3)	-46·3% (-56·1 to -36·4%)
	Intracerebral haemorrhage	22856 (19316–27054)	83·5% (47·7 to 127·3%)	325·6 (273·5–385·1)	-60% (-67·8 to -50·4%)
	Subarachnoid haemorrhage	3335 (2693–4084)	129·6% (69·5 to 228·8%)	38·9 (30·6–47·5)	-49·8% (-63·8 to -27·7%)
	Neurological disorders†	118079 (54388–213589)	243·5% (184·7 to 317·9%)	1291·8 (687·7–2194·4)	-4·7% (-17·1 to 8·6%)
	Alzheimer's disease and other dementias	14828 (6605–32942)	385·3% (325·6 to 465·5%)	369·7 (165·2–822·8)	-4·5% (-15·9 to 11·3%)
	Parkinson's disease	3534 (3007–4138)	335·8% (254·4 to 432·2%)	74·2 (63–86·7)	-14% (-29·8 to 5%)
	Idiopathic epilepsy	14136 (6162–26671)	131·8% (-9·3 to 452%)	119·4 (51·7–224·9)	-22·1% (-69·1 to 83·1%)
	Multiple sclerosis	1903 (1351–2405)	325% (236·9 to 413%)	19 (13·6–23·9)	-6·9% (-24·9 to 11·7%)
	Migraine	70088 (11911–160288)	250·2% (230·2 to 298·6%)	590·3 (104–1339·1)	-0·8% (-4 to 2·5%)
	Tension-type headache	7406 (2514–21769)	276·6% (221·8 to 332·8%)	66·2 (22·7–191·4)	0·1% (-10·9 to 8·3%)
	Motor neuron disease	338 (265–446)	424·1% (275·5 to 637·8%)	3·2 (2·5–4·2)	35·8% (-4·5 to 97%)
	Other neurological disorders	5844 (3502–9358)	204·1% (65·9 to 443·5%)	49·9 (31·3–77·7)	10·7% (-35·4 to 83·8%)
	Headache disorders	77495 (17367–171586)	252·5% (233·2 to 298·1%)	656·4 (153·8–1428·7)	-0·7% (-3·9 to 2·5%)
Kuwait	Meningitis	749 (606–917)	-22% (-40·2 to -0·4%)	20·1 (16·2–24·7)	-63·1% (-71·8 to -53·2%)
	Encephalitis	403 (333–511)	54·1% (26·4 to 92%)	10·5 (8·6–13·3)	-26·5% (-40·1 to -8·6%)
	Tetanus	1 (0–3)	86·7% (-69·6 to 628·3%)	0 (0–0·1)	-46·1% (-88·3 to 68%)
	Brain and central nervous system cancer	2478 (1736–3106)	137·7% (45·9 to 204·1%)	66·2 (47–82·8)	-8·6% (-44·1 to 16·6%)
	Stroke*	25463 (21787–29824)	232·8% (186·6 to 287·4%)	938·4 (795–1093·3)	-11·7% (-24 to 3·3%)
	Ischaemic stroke	15225 (12984–17974)	252·5% (206·4 to 307%)	645·4 (546·5–754·4)	-12% (-24 to 2·4%)
	Intracerebral haemorrhage	8432 (6893–10043)	252·7% (182·6 to 330·1%)	246 (201·3–294)	-4·2% (-23·3 to 18%)
	Subarachnoid haemorrhage	1807 (1476–2200)	92·1% (52·5 to 179·1%)	47 (38·9–56·6)	-34·9% (-48·1 to -11·3%)
	Neurological disorders†	49272 (22503–88625)	181·9% (131·6 to 238·7%)	1294·9 (693·8–2173·2)	-4·3% (-17·6 to 9·3%)
	Alzheimer's disease and other dementias	6860 (3079–14690)	387·2% (341·5 to 440%)	387·3 (174–837·8)	-2·6% (-11·2 to 6·9%)
	Parkinson's disease	1052 (894–1255)	221% (180·8 to 271·8%)	56·1 (47·2–66)	-26·8% (-36·5 to -14·7%)
	Idiopathic epilepsy	5505 (2389–10479)	74·2% (-22·8 to 296·6%)	138·9 (58·5–268·6)	-22·2% (-66·5 to 81·8%)
	Multiple sclerosis	894 (644–1180)	414·3% (336·1 to 505·8%)	17·3 (12·8–22·6)	38·2% (21·1 to 58·7%)
	Migraine	29758 (5159–67375)	190·2% (171·1 to 225·4%)	578·3 (99·7–1315·1)	3·2% (-2·3 to 8·6%)
	Tension-type headache	3205 (1084–8967)	204·5% (159·1 to 260·4%)	62·8 (20·8–186·4)	1·5% (-10·4 to 11·9%)
	Motor neuron disease	210 (155–262)	-14% (-39·2 to 17·5%)	5·9 (4·3–7·5)	-61·7% (-72·5 to -48·2%)
	Other neurological disorders	1788 (1032–3017)	108·4% (16·3 to 277·9%)	48·3 (28·3–81·4)	-3·2% (-44·8 to 69·7%)
	Headache disorders	32963 (7555–71305)	191·5% (173·1 to 225·4%)	641·1 (146·8–1408)	3·1% (-2·3 to 7·9%)

Table S12 Burden, mortality, incidence, and prevalence of neurological conditions in North Africa and Middle East countries

Measure		All Ages		Age-standardised	
		Number	Percent change	Rate per 100,000	Percent change
		2019	From 1990 to 2019	2019	From 1990 to 2019
Cause	Location	Mean (95% UI)	Mean (95% UI)	Mean (95% UI)	Mean (95% UI)
Lebanon	Meningitis	1528 (1154–1994)	-58·8% (-72·1 to -40·5%)	29·5 (22·2–38·5)	-68·6% (-78 to -56·1%)
	Encephalitis	902 (609–1583)	14·4% (-22 to 59·7%)	17·5 (11·8–30·6)	-19·2% (-42·9 to 10·2%)
	Tetanus	767 (166–2657)	-57·9% (-93 to 27·8%)	14·9 (3·2–50·9)	-75·8% (-95·7 to -29·1%)
	Brain and central nervous system cancer	6094 (4252–7858)	59·8% (7·6 to 118·8%)	117·3 (82·6–151·5)	-9% (-38·6 to 23·8%)
	Stroke*	39216 (30874–48709)	63% (27·7 to 105·4%)	752·9 (593·3–935·9)	-33·7% (-47·6 to -16%)
	Ischaemic stroke	31068 (24587–38151)	125·1% (71·9 to 176·4%)	597·8 (473·3–734·3)	-15·1% (-34·7 to 4·6%)
	Intracerebral haemorrhage	6732 (5020–8959)	-19% (-40·5 to 9·9%)	128·4 (95·9–170·4)	-64% (-73·7 to -51%)
	Subarachnoid haemorrhage	1416 (991–1898)	-27·4% (-55·8 to 16·4%)	26·7 (18·6–35·8)	-64·3% (-78·7 to -41·8%)
	Neurological disorders†	69765 (36994–117533)	93·9% (63 to 142·7%)	1349·3 (721·7–2279·8)	-3·4% (-16·8 to 12·3%)
	Alzheimer's disease and other dementias	19727 (8592–43392)	208·8% (166·4 to 308·3%)	395·6 (172·4–866·4)	-2·3% (-15 to 29·1%)
	Parkinson's disease	3662 (3016–4825)	142·1% (82·7 to 213·3%)	69·5 (57·3–91·6)	-14·2% (-35·2 to 11·2%)
	Idiopathic epilepsy	6786 (3216–12311)	11·9% (-52 to 153·2%)	132·9 (62·6–243·4)	-25·6% (-67·3 to 68·6%)
	Multiple sclerosis	1270 (891–1630)	146·5% (97·3 to 209·4%)	23·5 (16·4–30·3)	20·9% (-2·5 to 51·3%)
	Migraine	31980 (5563–73108)	76·4% (68·2 to 92·7%)	605·8 (104–1385·5)	0·5% (-2·7 to 3·5%)
	Tension-type headache	3565 (1217–10006)	86·4% (59 to 108·4%)	67 (22·7–191·7)	0·8% (-10·8 to 9·4%)
	Motor neuron disease	169 (112–261)	92·6% (25·8 to 185·4%)	3·3 (2·2–5·1)	4·5% (-32·1 to 55·6%)
	Other neurological disorders	2606 (1643–4080)	90·1% (11·5 to 257·2%)	51·7 (32·2–80·9)	28·4% (-24·8 to 133·1%)
	Headache disorders	35546 (8399–77063)	77·3% (68·9 to 92·6%)	672·7 (153·9–1457·6)	0·5% (-3·1 to 3·7%)
Libya	Meningitis	2179 (1718–2754)	-68% (-78·4 to -53·9%)	36·2 (28·4–45·8)	-69·9% (-78·5 to -58·4%)
	Encephalitis	1025 (707–1917)	5·4% (-30·5 to 52·9%)	17·4 (11·9–31·6)	-11·6% (-37·5 to 25·4%)
	Tetanus	67 (44–116)	-69·7% (-88·1 to -8·1%)	1·2 (0·7–2)	-70·5% (-89·8 to -19%)
	Brain and central nervous system cancer	8639 (6346–11310)	71·6% (16·1 to 161·2%)	134·4 (99·2–175·6)	-4·6% (-32·6 to 37·1%)
	Stroke*	82320 (64181–104686)	52·5% (15·8 to 109·4%)	1570·5 (1237·3–2001·2)	-21·3% (-37·5 to 1·4%)
	Ischaemic stroke	55357 (43126–71016)	164% (105·4 to 245·1%)	1122·6 (875·9–1435·2)	11·2% (-12 to 42·4%)
	Intracerebral haemorrhage	22583 (16898–30609)	-2·1% (-28·9 to 43%)	376·2 (284·9–504·4)	-51·2% (-63·2 to -34·3%)
	Subarachnoid haemorrhage	4380 (3189–6255)	-56% (-73 to -4·1%)	71·7 (51·6–101·4)	-66·7% (-77·7 to -42·9%)
	Neurological disorders†	84590 (43792–145104)	95% (63·4 to 133·8%)	1378·2 (759·2–2303·8)	-3% (-15·6 to 11·8%)
	Alzheimer's disease and other dementias	16595 (7063–37243)	161·3% (122·6 to 212·3%)	414·3 (176·1–925·9)	-4·2% (-18·1 to 14·3%)
	Parkinson's disease	3712 (2836–4719)	206·8% (134·1 to 306·4%)	84·8 (65·3–108·4)	10·9% (-15·2 to 45·8%)
	Idiopathic epilepsy	9315 (5179–14869)	1·9% (-46·8 to 108·6%)	141 (77·2–226·1)	-29% (-63·5 to 47·3%)
	Multiple sclerosis	1604 (1091–2241)	265% (176·8 to 418·4%)	21·8 (14·9–30·2)	34·2% (2·3 to 87·9%)
	Migraine	44893 (7730–101676)	103·6% (88·6 to 141·3%)	594·7 (104–1347)	1·3% (-1·9 to 4·4%)
	Tension-type headache	4920 (1694–13899)	122·3% (84·9 to 164·2%)	66·4 (22·8–191)	1·1% (-11·2 to 9·5%)
	Motor neuron disease	243 (149–363)	215·6% (71·3 to 427·3%)	3·7 (2·3–5·4)	39·5% (-25·6 to 142%)
	Other neurological disorders	3308 (2226–4717)	73·8% (2·4 to 219·8%)	51·5 (34·7–73·7)	23·2% (-25 to 115·9%)
	Headache disorders	49812 (11832–107596)	105·3% (90·3 to 140·3%)	661·1 (155·4–1425·1)	1·3% (-2·2 to 4·6%)
Morocco	Meningitis	22079 (15488–30007)	-82·9% (-89·4 to -74·2%)	68·4 (47·7–94·1)	-82% (-88·5 to -73·2%)
	Encephalitis	8593 (5253–16290)	-6·2% (-42·2 to 41·4%)	26 (15·6–48·9)	-16·9% (-45·8 to 23·8%)
	Tetanus	7669 (1916–28940)	-95% (-98·8 to -80·8%)	25·7 (6·3–98·1)	-93·9% (-98·5 to -76·2%)
	Brain and central nervous system cancer	19615 (13105–27097)	56·9% (-9·5 to 134·5%)	55·2 (37·4–74·2)	2·6% (-37·2 to 47·8%)
	Stroke*	666284 (534574–808810)	72·1% (36·4 to 111·6%)	2257·8 (1838·8–2702·5)	-17·2% (-33·9 to 0·9%)
	Ischaemic stroke	451674 (364060–544462)	167·3% (107·4 to 225·5%)	1603·6 (1305–1921·7)	18·5% (-6·7 to 42·2%)
	Intracerebral haemorrhage	181455 (138916–232630)	3·6% (-24·8 to 39·1%)	554 (425·3–704·4)	-51% (-64·1 to -34·3%)

Table S12 Burden, mortality, incidence, and prevalence of neurological conditions in North Africa and Middle East countries

**Measure** Cause	Location	**All Ages** Number 2019 Mean (95% UI)	Percent change From 1990 to 2019 Mean (95% UI)	**Age-standardised** Rate per 100,000 2019 Mean (95% UI)	Percent change From 1990 to 2019 Mean (95% UI)
	Subarachnoid haemorrhage	33155 (23008–48586)	-23% (-47·9 to 27·2%)	100·3 (70·3–143·9)	-58·6% (-72·4 to -30·7%)
	Neurological disorders†	435807 (225061–747805)	71·5% (46 to 110·2%)	1355·8 (744·6–2284·9)	1·9% (-12·1 to 17%)
	Alzheimer's disease and other dementias	86183 (37096–195521)	147% (117·2 to 177·1%)	385·8 (166–876·4)	0·3% (-10·6 to 11%)
	Parkinson's disease	25269 (20568–29607)	221·2% (160·6 to 300·2%)	96·6 (79·3–111·9)	39·6% (14·4 to 73·3%)
	Idiopathic epilepsy	47904 (17349–89938)	17·6% (-58·2 to 176·2%)	135·2 (48·7–253·5)	-11·7% (-68·2 to 108·6%)
	Multiple sclerosis	7437 (5171–10001)	148·6% (94·9 to 227·4%)	20·2 (14·1–27)	25·1% (-1·6 to 63·3%)
	Migraine	226776 (38690–515523)	58·5% (50·7 to 80·5%)	601·7 (104·2–1370·5)	-0·3% (-3·3 to 3%)
	Tension-type headache	24818 (8463–69426)	70·5% (47·5 to 93·4%)	66·5 (22·7–187·3)	0·2% (-9·8 to 8·1%)
	Motor neuron disease	903 (687–1185)	130·5% (67·1 to 211·2%)	2·5 (1·9–3·2)	23·8% (-11·8 to 68%)
	Other neurological disorders	16517 (9202–26306)	71·6% (-14·5 to 239·1%)	47·3 (26·7–75·3)	32·3% (-31·9 to 148·1%)
	Headache disorders	251594 (58542–544979)	59·6% (51·6 to 78·5%)	668·2 (156·2–1445·1)	-0·2% (-3·6 to 3·1%)
Palestine	Meningitis	1996 (1640–2450)	-75% (-83·4 to -62%)	40·5 (34·1–48·3)	-82·6% (-87·6 to -75·4%)
	Encephalitis	824 (642–1137)	51·1% (-4·7 to 118·9%)	16·3 (13·1–22·1)	-21·8% (-43·6 to 3·1%)
	Tetanus	71 (51–106)	-90·5% (-97·4 to -52·5%)	1·3 (0·9–1·9)	-92·7% (-98 to -67·8%)
	Brain and central nervous system cancer	8903 (6939–11031)	114·7% (31·2 to 200·5%)	232 (175·6–279·5)	-9·1% (-39·8 to 24·2%)
	Stroke*	44902 (39457–50763)	63·5% (29·3 to 104·3%)	2128·3 (1878·2–2399·7)	-32·5% (-46·4 to -15%)
	Ischaemic stroke	31202 (27203–35384)	101·3% (59·7 to 150·7%)	1590·9 (1386·2–1793·7)	-17·8% (-34·6 to 3%)
	Intracerebral haemorrhage	12183 (10390–14189)	11·2% (-14·1 to 46·7%)	487·3 (416·5–568·3)	-56·7% (-66·5 to -43%)
	Subarachnoid haemorrhage	1516 (1277–1778)	49·3% (10·4 to 117·4%)	50·1 (42·3–59)	-46% (-61·6 to -22·2%)
	Neurological disorders†	50324 (24673–87487)	142·1% (92·8 to 184·1%)	1376 (773·8–2255·8)	-5·1% (-17·7 to 8·1%)
	Alzheimer's disease and other dementias	5818 (2538–13511)	117·2% (87 to 155·6%)	388 (170·4–895·9)	-4·4% (-17 to 12%)
	Parkinson's disease	1614 (1142–1855)	127·5% (75·6 to 210·6%)	93·1 (64·9–106·8)	-4·9% (-26·4 to 29·1%)
	Idiopathic epilepsy	8232 (4516–13294)	69·8% (-11·5 to 223·1%)	163·6 (91·1–262·6)	-24·2% (-60·6 to 43·1%)
	Multiple sclerosis	726 (572–901)	239·9% (151·8 to 357·1%)	19·5 (15·3–24·3)	8·5% (-18·1 to 44·4%)
	Migraine	28492 (4390–66341)	177·7% (166·5 to 199·2%)	596·1 (102·5–1357·3)	-1·1% (-4·1 to 1·9%)
	Tension-type headache	2914 (952–8613)	187·4% (144·1 to 219·3%)	66·2 (22·1–187·5)	-0·3% (-13 to 9·3%)
	Motor neuron disease	84 (69–102)	216·8% (132·7 to 328·8%)	2·1 (1·7–2·5)	31·7% (-8·3 to 85·9%)
	Other neurological disorders	2443 (1404–3855)	134·8% (25·7 to 342·6%)	47·5 (29·7–71·6)	13·9% (-34 to 103·6%)
	Headache disorders	31406 (6765–70639)	178·6% (166·2 to 198·3%)	662·3 (157·1–1439·2)	-1% (-4·4 to 2·1%)
Oman	Meningitis	708 (600–870)	-63·1% (-74·1 to -46·1%)	24·5 (20·8–29·5)	-73·9% (-80·2 to -63·3%)
	Encephalitis	4505 (3291–7755)	7·8% (-31·4 to 67·1%)	122·1 (94–180·3)	-44·1% (-62·4 to -14·1%)
	Tetanus	40 (32–50)	-79·7% (-93·8 to -19·4%)	1 (0·7–1·3)	-90·3% (-96·7 to -62·2%)
	Brain and central nervous system cancer	2520 (1453–3280)	151·6% (26·6 to 282·6%)	72·6 (43·8–91·3)	11% (-44·4 to 65·5%)
	Stroke*	30859 (27010–36651)	26·6% (-0·4 to 64·9%)	1884·3 (1667·3–2127·1)	-38·7% (-51·9 to -20·7%)
	Ischaemic stroke	17267 (14836–19890)	79·5% (38·7 to 135·1%)	1291·3 (1116·3–1468·2)	-18·3% (-35·2 to 6·7%)
	Intracerebral haemorrhage	10843 (9315–13404)	-1·9% (-25·1 to 32·7%)	504·1 (437·1–621·6)	-59·8% (-69·5 to -46·5%)
	Subarachnoid haemorrhage	2749 (1961–4314)	-25·7% (-54·9 to 55·5%)	88·9 (59·7–133·6)	-62·4% (-76·8 to -29·6%)
	Neurological disorders†	43187 (18527–79809)	167·7% (114·1 to 223·1%)	1340·6 (758·2–2231·2)	1% (-12·7 to 19·5%)
	Alzheimer's disease and other dementias	3313 (1388–7765)	126·2% (93·7 to 183·8%)	422 (179·8–967·9)	-3·3% (-16·4 to 26·2%)
	Parkinson's disease	1097 (785–1265)	168·6% (99·7 to 253·4%)	125·3 (79–144·5)	30·3% (-2·2 to 70·4%)
	Idiopathic epilepsy	4544 (1615–9015)	91·1% (-38·5 to 468·1%)	107·8 (37·2–214·7)	-9·8% (-70·4 to 160·3%)
	Multiple sclerosis	728 (501–973)	301·1% (176·6 to 453·3%)	17·1 (11·6–23·2)	20·3% (-15·8 to 68·5%)
	Migraine	28437 (4879–64400)	185% (166·5 to 226·5%)	554·2 (98·7–1256·8)	-1·4% (-4·5 to 1·9%)

Table S12 Burden, mortality, incidence, and prevalence of neurological conditions in North Africa and Middle East countries

**Measure**		**All Ages**		**Age-standardised**	
		Number	Percent change	Rate per 100,000	Percent change
		2019	From 1990 to 2019	2019	From 1990 to 2019
Cause	Location	Mean (95% UI)	Mean (95% UI)	Mean (95% UI)	Mean (95% UI)
	Tension-type headache	3196 (1042–8985)	208·1% (154·1 to 256·4%)	64·3 (21·6–190·1)	0% (-15·4 to 10·8%)
	Motor neuron disease	100 (68–130)	208·9% (89·5 to 344·2%)	3·2 (1·9–4·4)	17·5% (-32·8 to 84·6%)
	Other neurological disorders	1771 (1005–2906)	171·8% (39·3 to 463·2%)	46·8 (27·9–75·1)	43·2% (-21·9 to 164·3%)
	Headache disorders	31633 (7474–67794)	187·2% (168·1 to 223·2%)	618·5 (146·4–1332·9)	-1·2% (-4·9 to 2·6%)
Qatar	Meningitis	516 (413–641)	34·9% (-9·8 to 93·7%)	25·4 (20·7–31·2)	-73·1% (-81·8 to -62·7%)
	Encephalitis	279 (207–394)	264·1% (136·5 to 425·7%)	13·3 (9·9–17·6)	-29·3% (-52·7 to 0·9%)
	Tetanus	17 (13–23)	195·2% (8·4 to 463·3%)	0·7 (0·5–1)	-50·2% (-83·3 to 5%)
	Brain and central nervous system cancer	1692 (1130–2801)	433·1% (209 to 716·6%)	85·5 (61·1–128·4)	-20·3% (-53·1 to 20·6%)
	Stroke*	8998 (7254–11097)	256·6% (174·3 to 355·9%)	904·5 (747·6–1130·3)	-44·7% (-57·5 to -29·5%)
	Ischaemic stroke	3791 (2990–4599)	333·4% (238·6 to 427·2%)	577·3 (476·3–725)	-35·5% (-49·9 to -19·3%)
	Intracerebral haemorrhage	3518 (2730–4574)	205·4% (117·4 to 322·7%)	243·6 (195·1–315·2)	-56·3% (-67·8 to -41·8%)
	Subarachnoid haemorrhage	1689 (1246–2192)	240% (109·5 to 413·8%)	83·6 (62·6–109·6)	-54·2% (-72·3 to -26·8%)
	Neurological disorders†	26834 (11350–50198)	546·8% (399·9 to 684·3%)	1344·2 (754·1–2196)	-2·2% (-17·2 to 16·1%)
	Alzheimer's disease and other dementias	1203 (489–2863)	600% (460·9 to 778·2%)	407·2 (171·7–948·7)	3·4% (-11·6 to 19·9%)
	Parkinson's disease	488 (360–643)	517% (361·6 to 711·4%)	148·6 (96–194·6)	14·8% (-11·6 to 49·8%)
	Idiopathic epilepsy	3147 (1212–6098)	326·2% (61·4 to 1037%)	126·5 (47·7–250·5)	-28·2% (-73·2 to 94·6%)
	Multiple sclerosis	540 (388–720)	1040·4% (822·7 to 1331·2%)	16·8 (12·3–21·8)	33·7% (10·4 to 61·8%)
	Migraine	18109 (3233–40461)	601·2% (567·3 to 669·2%)	523 (94·3–1174·9)	-2·7% (-6·2 to 0·8%)
	Tension-type headache	2135 (699–6004)	643·8% (552 to 739·5%)	62 (20·7–184·7)	-1·4% (-11·6 to 7·2%)
	Motor neuron disease	50 (39–64)	619·8% (433 to 857·2%)	2·1 (1·6–2·7)	-4·8% (-34·3 to 34·6%)
	Other neurological disorders	1162 (702–1855)	393·3% (181·8 to 777·7%)	57·8 (36·9–87)	-3·3% (-42·3 to 67·3%)
	Headache disorders	20244 (5008–42949)	605·4% (571·7 to 667·4%)	585 (140·4–1252)	-2·6% (-5·8 to 0·9%)
Saudi Arabia	Meningitis	8491 (6565–11135)	-41·8% (-56·5 to -19·7%)	26·7 (21·3–33·5)	-73·3% (-79·4 to -63·2%)
	Encephalitis	9462 (7139–12792)	2·3% (-28·7 to 51·7%)	28·2 (21·6–37·8)	-53% (-68·2 to -29·9%)
	Tetanus	749 (246–1293)	-94·2% (-99·2 to -68·2%)	2·1 (0·8–3·5)	-96·5% (-99·4 to -81·2%)
	Brain and central nervous system cancer	26453 (19219–39839)	250·7% (91·7 to 558·9%)	79·4 (59·2–120·9)	32·3% (-29·6 to 148·9%)
	Stroke*	417599 (326188–509857)	129% (67·6 to 211·5%)	2114·9 (1685·1–2485·5)	-29·1% (-46·4 to -5·6%)
	Ischaemic stroke	207936 (160132–249405)	153·3% (90·1 to 234·4%)	1300·8 (1026·9–1516·1)	-17·4% (-36·3 to 5·8%)
	Intracerebral haemorrhage	192664 (149104–243126)	113·8% (51 to 215·6%)	764·7 (597–929·3)	-41·3% (-57·6 to -15·5%)
	Subarachnoid haemorrhage	16999 (12299–22487)	66·7% (-0·4 to 170·7%)	49·5 (36·1–64·1)	-52·5% (-71·9 to -22·6%)
	Neurological disorders†	410451 (195196–722127)	160·3% (106·3 to 223·5%)	1428·4 (794·9–2355·6)	0·1% (-16·7 to 20·3%)
	Alzheimer's disease and other dementias	32530 (13935–72984)	122·7% (86·3 to 186·1%)	389·7 (168·5–863·1)	-2% (-15·8 to 26·4%)
	Parkinson's disease	11587 (9409–13541)	145·8% (88·1 to 247·9%)	111 (83·8–129·8)	1·6% (-19·9 to 43·5%)
	Idiopathic epilepsy	81357 (40168–137978)	128·5% (4·1 to 416·5%)	226·7 (107·8–394·2)	-0·3% (-56·7 to 123·2%)
	Multiple sclerosis	4558 (3316–5953)	357·9% (248·8 to 567·7%)	11·7 (8·7–15)	23% (-5 to 77%)
	Migraine	234239 (41410–528197)	176·4% (152·8 to 229·1%)	562 (99·4–1276·9)	-1·2% (-5·9 to 3·8%)
	Tension-type headache	25584 (9050–70083)	202% (136·9 to 255%)	63 (22–180·3)	-0·3% (-18·6 to 10·8%)
	Motor neuron disease	941 (676–1306)	226·6% (85 to 448%)	3·1 (2·2–4·2)	18·7% (-35 to 116%)
	Other neurological disorders	19655 (10586–32871)	138·3% (11·4 to 384%)	61·3 (34·4–101·1)	27·1% (-37·2 to 145·4%)
	Headache disorders	259823 (62718–548786)	178·7% (155·8 to 227·9%)	625 (149·7–1343·4)	-1·1% (-6·2 to 3·8%)
Syrian Arab Republic	Meningitis	13469 (10405–17156)	-80·1% (-86·4 to -71·2%)	100 (76·6–129·5)	-70·7% (-79·4 to -58·5%)
	Encephalitis	3715 (2866–4881)	-52·8% (-70·3 to -17·7%)	27·4 (21·1–36·6)	-41% (-61·4 to -1·9%)
	Tetanus	307 (169–626)	-97·6% (-99·2 to -89·5%)	2·4 (1·3–5)	-95·6% (-98·6 to -81·4%)

Table S12 Burden, mortality, incidence, and prevalence of neurological conditions in North Africa and Middle East countries

		All Ages		Age-standardised	
		Number	Percent change	Rate per 100,000	Percent change
**Measure**		2019	From 1990 to 2019	2019	From 1990 to 2019
Cause	Location	Mean (95% UI)	Mean (95% UI)	Mean (95% UI)	Mean (95% UI)
	Brain and central nervous system cancer	20391 (14676–27540)	34·4% (-23 to 107·6%)	144·8 (104·7–194·1)	-6·8% (-38·4 to 37·2%)
	Stroke*	238333 (184920–304410)	-5·7% (-29·3 to 27·5%)	2018·1 (1589·6–2547·1)	-40·2% (-55·3 to -19·9%)
	Ischaemic stroke	116537 (92554–145609)	43·2% (7·6 to 90·1%)	1069·9 (858·8–1325·1)	-25·1% (-44·7 to -2·2%)
	Intracerebral haemorrhage	106469 (80729–141598)	-11·4% (-36·4 to 24·2%)	839 (646·4–1101·7)	-48·5% (-62·7 to -28·8%)
	Subarachnoid haemorrhage	15327 (11694–19102)	-70·1% (-80 to -49·5%)	109·2 (84·6–135·2)	-65·7% (-76 to -47·7%)
	Neurological disorders†	165257 (80674–293562)	43·5% (20·1 to 79·2%)	1327·1 (692·4–2305·8)	1·4% (-11·9 to 16·8%)
	Alzheimer's disease and other dementias	30688 (13248–70349)	112% (74·2 to 170·1%)	391·1 (167–907·7)	2·9% (-14 to 29·8%)
	Parkinson's disease	7787 (5490–9816)	157·5% (96·9 to 243·4%)	83·9 (58·4–104·9)	13·7% (-12·2 to 49·7%)
	Idiopathic epilepsy	15874 (6769–28704)	-10·5% (-65·7 to 124·2%)	109·2 (47–198·2)	-17·2% (-67·5 to 102·6%)
	Multiple sclerosis	2153 (1605–2776)	118·3% (77·3 to 168·1%)	15·2 (11·3–19·6)	15·4% (-5·9 to 40·2%)
	Migraine	91621 (15453–212202)	39·1% (31·2 to 62·3%)	609·9 (105·1–1394·5)	1·7% (-1·9 to 5·2%)
	Tension-type headache	9810 (3361–28381)	51·2% (24·9 to 78·8%)	67 (22·9–189·7)	1·1% (-10·5 to 9·5%)
	Motor neuron disease	424 (278–650)	106·2% (31 to 220·1%)	2·9 (1·9–4·4)	23·9% (-21·2 to 93·2%)
	Other neurological disorders	6900 (4257–10445)	8% (-40·5 to 96·8%)	48 (30·5–71·5)	14·1% (-34·4 to 96·1%)
	Headache disorders	101431 (22875–223796)	40·2% (32·3 to 61·8%)	676·9 (157·1–1474·2)	1·7% (-2·1 to 5·1%)
Tunisia	Meningitis	4056 (3030–5173)	-76·3% (-84·4 to -64·1%)	37·9 (28·2–48·2)	-77·1% (-84·8 to -66·2%)
	Encephalitis	1987 (1319–3724)	-18·8% (-49·5 to 18·9%)	18·4 (12·1–34·4)	-28·7% (-54·5 to 2·7%)
	Tetanus	106 (70–195)	-83·7% (-94 to -56·6%)	1 (0·7–1·8)	-83·8% (-94 to -57·1%)
	Brain and central nervous system cancer	5077 (3366–6958)	44·3% (-15·1 to 113·1%)	41·8 (27·6–57·1)	-5·4% (-42·2 to 37·8%)
	Stroke*	176891 (135395–225499)	83·2% (38·9 to 138·2%)	1477·5 (1138·5–1875)	-24·7% (-42·8 to -2%)
	Ischaemic stroke	128086 (98885–163158)	178·2% (108·2 to 261·1%)	1090·4 (848·4–1382·7)	4·1% (-22 to 34·2%)
	Intracerebral haemorrhage	40607 (29901–53483)	2·1% (-27·6 to 42·4%)	322·5 (238·9–422·5)	-56·5% (-69·3 to -38·5%)
	Subarachnoid haemorrhage	8198 (5771–11166)	-23·9% (-51·2 to 27·2%)	64·5 (45·6–88·2)	-62·6% (-76·7 to -38·2%)
	Neurological disorders†	154375 (80217–263164)	74·3% (47·6 to 117·8%)	1332·6 (699·8–2234·9)	-2·1% (-15·5 to 11·4%)
	Alzheimer's disease and other dementias	42020 (18281–94965)	212·4% (160 to 293·7%)	400·1 (174·9–889·4)	-2% (-17 to 21·4%)
	Parkinson's disease	8271 (6418–10676)	205·8% (138·1 to 290·2%)	72·7 (56·2–93·7)	11·1% (-13·8 to 41%)
	Idiopathic epilepsy	13345 (6574–23467)	-11·9% (-58·9 to 78·9%)	119·5 (58·8–210·4)	-26·5% (-66·1 to 50·4%)
	Multiple sclerosis	2938 (2061–3853)	158·7% (103·5 to 228·3%)	22·2 (15·5–29·2)	25·6% (1 to 58·9%)
	Migraine	74318 (13231–168600)	54·9% (45·1 to 82·1%)	606·3 (104·4–1382·7)	0·3% (-2·6 to 3·4%)
	Tension-type headache	8414 (2880–23528)	70·6% (42·7 to 97·2%)	67 (22·6–192·5)	0·7% (-10·7 to 8·5%)
	Motor neuron disease	346 (249–473)	115·1% (41·1 to 224·9%)	2·7 (2–3·6)	15·9% (-24 to 76·4%)
	Other neurological disorders	4722 (2962–7075)	56·2% (-11·3 to 182·6%)	42 (25·4–65·3)	24·4% (-29·8 to 119·4%)
	Headache disorders	82732 (20234–176614)	56·3% (46·6 to 81·3%)	673·3 (158·7–1448·7)	0·3% (-3·1 to 3·5%)
Türkiye	Meningitis	17218 (14424–20635)	-90·8% (-94·3 to -85·7%)	25·9 (21·4–31·9)	-89·9% (-93·7 to -84·5%)
	Encephalitis	13246 (10795–15985)	-42·9% (-64·2 to -15%)	18·6 (14·9–22·9)	-47·4% (-66·5 to -22·2%)
	Tetanus	711 (543–962)	-98% (-98·9 to -94·1%)	1 (0·8–1·4)	-98·3% (-99 to -95·5%)
	Brain and central nervous system cancer	132889 (63488–183730)	24·5% (-34·5 to 93·9%)	158·3 (76·7–215·5)	-20·4% (-55·4 to 19·1%)
	Stroke*	993082 (820881–1177528)	75·3% (32·7 to 117%)	1162·6 (965–1380·4)	-23·5% (-42·5 to -5·2%)
	Ischaemic stroke	551064 (459967–649248)	133·2% (71·4 to 186·1%)	662·7 (552·9–779·5)	-8% (-32·7 to 14·3%)
	Intracerebral haemorrhage	350826 (284141–427496)	37% (0·3 to 74·7%)	399 (323·7–485·6)	-36·9% (-54·1 to -20%)
	Subarachnoid haemorrhage	91192 (72042–114240)	22·8% (-22 to 111·1%)	100·9 (80–125·8)	-39·9% (-61·8 to 5·4%)
	Neurological disorders†	1231991 (709138–2000451)	49·8% (23 to 84·9%)	1477·4 (862·5–2368·4)	-8·7% (-22·9 to 6·2%)
	Alzheimer's disease and other dementias	313664 (142254–713153)	182·1% (142·9 to 226·4%)	395·4 (179·6–892·7)	-2% (-15·1 to 12·9%)

Table S12 Burden, mortality, incidence, and prevalence of neurological conditions in North Africa and Middle East countries

		All Ages		Age-standardised	
		Number	Percent change	Rate per 100,000	Percent change
**Measure**		2019	From 1990 to 2019	2019	From 1990 to 2019
Cause	Location	Mean (95% UI)	Mean (95% UI)	Mean (95% UI)	Mean (95% UI)
	Parkinson's disease	62178 (48654–105236)	147·6% (97·6 to 201·9%)	76·1 (59·4–129·7)	-8·2% (-25·9 to 11·9%)
	Idiopathic epilepsy	162977 (83748–270865)	-19·8% (-60·9 to 58·6%)	213·7 (113·7–354·5)	-30·4% (-65·1 to 38·4%)
	Multiple sclerosis	19425 (15255–24609)	68·9% (7 to 127·5%)	21 (16·3–27·6)	-11·8% (-37·6 to 20%)
	Migraine	528078 (101819–1197352)	56·6% (45·4 to 81·9%)	589·6 (110·2–1340·7)	0·2% (-4·5 to 5·2%)
	Tension-type headache	60666 (21747–160117)	71·5% (44·9 to 93·8%)	66·6 (23·6–180)	0·8% (-10 to 10·1%)
	Motor neuron disease	23574 (18647–29385)	-14·4% (-54·1 to 51·4%)	31·7 (24·6–40·4)	-25% (-58 to 20·9%)
	Other neurological disorders	61429 (42698–84967)	-13·6% (-43·8 to 34·7%)	83·4 (57·4–117·1)	-20·8% (-48·4 to 20·7%)
	Headache disorders	588744 (155386–1275312)	58% (47·2 to 81%)	656·1 (167·7–1433)	0·3% (-4·6 to 5%)
United Arab Emirates	Meningitis	2834 (1849–4062)	26·4% (-21·2 to 80·3%)	32·1 (22·2–43·8)	-73·9% (-81·3 to -65·7%)
	Encephalitis	1474 (881–2971)	225·1% (113·7 to 388%)	18·4 (11·9–34·1)	-19·5% (-44·4 to 18%)
	Tetanus	1060 (485–1742)	-8% (-83·2 to 209·2%)	33·2 (8·4–54·9)	-86% (-96·4 to -46·5%)
	Brain and central nervous system cancer	14328 (8218–21137)	462% (270·9 to 714·7%)	151·5 (91·5–206·7)	-10·7% (-45·3 to 30·6%)
	Stroke*	95518 (70890–128945)	416·5% (265·8 to 618·4%)	1925·6 (1517–2457·9)	-46% (-58·5 to -30·5%)
	Ischaemic stroke	54428 (41443–69792)	620·9% (416·4 to 889·1%)	1415·2 (1128·2–1749·3)	-33·9% (-48·7 to -15·7%)
	Intracerebral haemorrhage	35021 (23807–53069)	294·6% (149·3 to 494·6%)	449·8 (317·9–664·8)	-64·1% (-75·4 to -49·2%)
	Subarachnoid haemorrhage	6068 (3360–9834)	193·2% (77·1 to 409·8%)	60·6 (33·3–95·3)	-64·4% (-76·8 to -43·5%)
	Neurological disorders†	104945 (51493–182388)	439·5% (320·2 to 578·7%)	1432 (843·2–2254·4)	-6·4% (-24·3 to 12%)
	Alzheimer's disease and other dementias	4085 (1620–9979)	607·7% (462·1 to 810·2%)	366·1 (157·8–831)	-5·4% (-17·1 to 14·2%)
	Parkinson's disease	2896 (2100–3866)	716·5% (504·6 to 1058·9%)	130·5 (87–167·2)	-10·6% (-29·6 to 21·4%)
	Idiopathic epilepsy	18904 (8991–30669)	253·5% (60·3 to 637%)	216·3 (98·8–364·2)	-28·2% (-69·6 to 62·2%)
	Multiple sclerosis	2827 (1713–4429)	935·7% (601·3 to 1345·8%)	20·6 (12·3–32·5)	19% (-16·5 to 59·7%)
	Migraine	60153 (11995–134011)	473% (424 to 588·1%)	535·6 (97·8–1203·7)	-0·7% (-4·3 to 3·1%)
	Tension-type headache	7426 (2396–20517)	542·2% (427·1 to 676·1%)	63·2 (20·7–187·8)	0% (-12·6 to 8·9%)
	Motor neuron disease	561 (258–1065)	961·7% (388 to 1953·3%)	5·9 (2·6–11·2)	21·9% (-44·4 to 124·6%)
	Other neurological disorders	8094 (5181–11697)	577·3% (281·2 to 1086·5%)	93·8 (59·5–135·8)	31% (-23·2 to 121·3%)
	Headache disorders	67579 (17571–141865)	479·9% (432·5 to 583%)	598·8 (142·4–1275·1)	-0·7% (-5 to 3%)
Yemen	Meningitis	33109 (22291–48225)	-55·4% (-71·8 to -29·7%)	97·4 (68·3–136·9)	-68·5% (-78·6 to -54·1%)
	Encephalitis	11411 (6705–21416)	52·7% (-12·4 to 168·5%)	31·3 (19·4–57·9)	-7·1% (-41·7 to 47·1%)
	Tetanus	4196 (1662–8780)	-85·7% (-95·7 to -34·5%)	11·2 (5–23·3)	-89·9% (-96·5 to -61·6%)
	Brain and central nervous system cancer	31600 (18698–46415)	137% (23·7 to 365·4%)	123·3 (74·9–181·5)	15·2% (-35·9 to 99·7%)
	Stroke*	388486 (302567–500074)	88·5% (44 to 152·4%)	2765·5 (2161·6–3520·5)	-24·5% (-41 to -1·9%)
	Ischaemic stroke	212195 (168265–276241)	232·1% (159·4 to 334·1%)	1716·7 (1370·2–2197·1)	19·8% (-4·2 to 51·1%)
	Intracerebral haemorrhage	146684 (108214–195409)	25% (-9·2 to 75·9%)	884·3 (663·2–1178·9)	-53·7% (-65·8 to -36·8%)
	Subarachnoid haemorrhage	29608 (15705–48366)	18·9% (-19·5 to 104·4%)	164·5 (87·2–275·6)	-48·9% (-63·9 to -20%)
	Neurological disorders†	298357 (137796–541512)	137·1% (81·9 to 190·8%)	1324·5 (723·4–2244·2)	-2·7% (-15·4 to 12·6%)
	Alzheimer's disease and other dementias	31778 (13661–72000)	202% (163·7 to 252·4%)	383·7 (166–893·1)	-0·4% (-11·5 to 14·1%)
	Parkinson's disease	8163 (6383–10687)	216·7% (147·7 to 319·7%)	76·8 (60·8–98·7)	15·5% (-7·8 to 49·7%)
	Idiopathic epilepsy	49541 (26539–81681)	41·5% (-27·2 to 217·2%)	147·6 (78·5–243·3)	-27·4% (-62·7 to 53%)
	Multiple sclerosis	3096 (1957–4548)	266·8% (156·3 to 435·7%)	14·1 (9·2–19·6)	26·4% (-9·6 to 72·1%)
	Migraine	175369 (26571–404422)	169% (158·9 to 186·6%)	596·7 (104·2–1356·8)	0·1% (-2·9 to 3·2%)
	Tension-type headache	17617 (5766–51725)	176·7% (140 to 209·4%)	66·1 (22·8–185·6)	0·6% (-11·1 to 9·8%)
	Motor neuron disease	396 (300–514)	196·5% (107·9 to 322·1%)	1·8 (1·3–2·5)	19·9% (-23·6 to 83·1%)
	Other neurological disorders	12397 (6185–21451)	140·2% (8·3 to 487·5%)	37·9 (21·8–61·2)	20·1% (-39·2 to 161·1%)

Table S12 Burden, mortality, incidence, and prevalence of neurological conditions in North Africa and Middle East countries

		All Ages		Age-standardised	
		Number	Percent change	Rate per 100,000	Percent change
**Measure**		2019	From 1990 to 2019	2019	From 1990 to 2019
Cause	Location	Mean (95% UI)	Mean (95% UI)	Mean (95% UI)	Mean (95% UI)
	Headache disorders	192986 (40627–431548)	169·7% (159·4 to 187·2%)	662·8 (157·9–1435·6)	0·1% (-3·4 to 3·6%)
Afghanistan	Meningitis	121279 (88624–171666)	-30·5% (-55 to 6·9%)	244·7 (184·6–325·7)	-73·6% (-82·1 to -62·4%)
	Encephalitis	60188 (39100–116114)	156·9% (74 to 263·1%)	175·8 (131·7–248·4)	-14·2% (-38·4 to 17·5%)
	Tetanus	58111 (32892–101654)	-74·6% (-88·5 to -32·2%)	113·9 (70·5–179·3)	-90·2% (-94·9 to -79·6%)
	Brain and central nervous system cancer	54529 (29084–95999)	152·7% (57 to 371·2%)	176 (99·6–323·9)	-5·8% (-38·6 to 56%)
	Stroke*	507837 (375947–659772)	56·3% (20·5 to 98·4%)	3498·2 (2508·8–4500·4)	-21·8% (-39·4 to -3·6%)
	Ischaemic stroke	224570 (170361–298131)	135·5% (80·7 to 197·7%)	1865·5 (1381·8–2444·2)	27·3% (-0·6 to 58·5%)
	Intracerebral haemorrhage	241903 (176658–324979)	22·9% (-7·3 to 64·9%)	1397·7 (991–1864·5)	-46% (-59·2 to -29·6%)
	Subarachnoid haemorrhage	41364 (13929–70872)	26% (-12·4 to 98·7%)	235 (76·3–400·7)	-43·8% (-59·4 to -14·3%)
	Neurological disorders†	382680 (190210–668251)	154·8% (86·6 to 226·8%)	1503 (853·8–2492·1)	-10·1% (-23·8 to 4·4%)
	Alzheimer's disease and other dementias	30938 (12731–73803)	52·6% (31·4 to 71·9%)	432·7 (179–1002)	-3·7% (-15·3 to 6·8%)
	Parkinson's disease	11574 (8845–14866)	52·5% (18·5 to 92·8%)	113·8 (88·7–143·3)	-6% (-25·7 to 16·3%)
	Idiopathic epilepsy	94040 (50171–142571)	88% (10·5 to 308·6%)	224·7 (120·4–348·1)	-38·8% (-64·2 to 28·3%)
	Multiple sclerosis	6575 (3900–12354)	237·7% (138 to 395·7%)	27·1 (16·8–48·7)	10·9% (-21·5 to 51·8%)
	Migraine	198849 (30218–469528)	244·1% (220·3 to 261·8%)	587·4 (103–1342·7)	-1·3% (-4·9 to 3·2%)
	Tension-type headache	19586 (6272–57731)	231·4% (191·2 to 263·2%)	65·2 (22·1–183·3)	-0·1% (-9·6 to 7·9%)
	Motor neuron disease	628 (484–827)	144·4% (73·9 to 237·9%)	2·6 (1·8–3·6)	-6·9% (-34·5 to 34·1%)
	Other neurological disorders	20488 (10466–34679)	221·5% (56·3 to 543·9%)	49·6 (28·8–79·6)	5·6% (-41·9 to 94·8%)
	Headache disorders	218435 (44781–493226)	242·9% (219·8 to 259·7%)	652·5 (156·7–1422·4)	-1·2% (-4·5 to 2·9%)
Sudan	Meningitis	46672 (30936–71077)	-79·9% (-87·4 to -69%)	100·3 (68·9–144·1)	-85·1% (-90·2 to -77·6%)
	Encephalitis	14946 (8675–27391)	15·7% (-37·4 to 120·1%)	32·1 (19·6–58·4)	-22·1% (-53·8 to 30·3%)
	Tetanus	2686 (1121–5579)	-92·9% (-97·4 to -77·6%)	5·6 (2·6–12·1)	-94·3% (-97·8 to -83·9%)
	Brain and central nervous system cancer	48434 (29080–71332)	70·3% (-20 to 292·9%)	135·8 (84·2–196·4)	-1·5% (-48·8 to 87·4%)
	Stroke*	522412 (385879–715571)	21·5% (-7·2 to 57·4%)	2585·5 (1970·5–3552·9)	-33% (-45·3 to -16·9%)
	Ischaemic stroke	304172 (232832–427020)	108·8% (61·1 to 165·4%)	1692·7 (1303·5–2360·6)	8·1% (-12·2 to 33·5%)
	Intracerebral haemorrhage	185673 (126437–260181)	-20·6% (-43·3 to 7·9%)	769·9 (528·1–1070·3)	-60·6% (-70 to -48%)
	Subarachnoid haemorrhage	32567 (18464–55769)	-35·4% (-56·2 to 35·5%)	122·9 (71·2–208·7)	-63·7% (-75 to -32·7%)
	Neurological disorders†	420277 (204680–745904)	84·3% (37·5 to 130·9%)	1353·8 (761·5–2268·2)	-7·1% (-20·6 to 6·3%)
	Alzheimer's disease and other dementias	48141 (21134–107828)	107·9% (88·1 to 135·5%)	365·5 (160·3–840·7)	-3·9% (-12·2 to 7%)
	Parkinson's disease	13238 (10419–16768)	81·9% (38·4 to 143·1%)	85·5 (67·8–107·5)	-4·3% (-26·5 to 26·1%)
	Idiopathic epilepsy	74696 (43254–119485)	5·7% (-46·7 to 158·2%)	168·7 (97·5–271)	-37·1% (-67·9 to 39·6%)
	Multiple sclerosis	4194 (2566–6043)	175·8% (93·2 to 310·7%)	14 (8·9–19·2)	20·2% (-13·9 to 70·1%)
	Migraine	234320 (36676–542009)	122·2% (114·9 to 132·9%)	601·9 (104·6–1369·5)	-0·1% (-3·2 to 3·2%)
	Tension-type headache	23633 (7813–69143)	125% (95·3 to 148·4%)	66·4 (23·2–189)	0·5% (-11·8 to 9·8%)
	Motor neuron disease	608 (449–804)	138·9% (63 to 241·8%)	2·1 (1·5–2·8)	17·3% (-23·4 to 74·9%)
	Other neurological disorders	21446 (12180–34810)	132·1% (17·3 to 374·3%)	49·7 (30·3–76·2)	33% (-28·5 to 152·1%)
	Headache disorders	257953 (53961–578953)	122·5% (114·3 to 132·3%)	668·3 (155·5–1438·9)	-0·1% (-3·7 to 3·7%)
**Deaths**					
Global	Meningitis	236222 (204381–277426)	-45·4% (-53·5 to -35·8%)	3·3 (2·8–3·9)	-56% (-62·5 to -48·3%)
	Encephalitis	89897 (76532–122874)	-23·7% (-38·9 to 12·9%)	1·2 (1–1·6)	-45·7% (-56·1 to -21·1%)
	Tetanus	34684 (25943–48457)	-87·4% (-90·5 to -81·8%)	0·5 (0·4–0·7)	-89·5% (-91·9 to -84·9%)
	Brain and central nervous system cancer	246253 (185642–270930)	76·4% (11 to 104·9%)	3 (2·3–3·4)	-1·2% (-36·8 to 13·9%)
	Stroke*	6552725 (5995200–7015139)	43·3% (31 to 55·4%)	84·2 (76·8–90·2)	-36·4% (-41·6 to -31·2%)

Table S12 Burden, mortality, incidence, and prevalence of neurological conditions in North Africa and Middle East countries

		**All Ages**		**Age-standardised**	
		Number	Percent change	Rate per 100,000	Percent change
**Measure**		2019	From 1990 to 2019	2019	From 1990 to 2019
Cause	Location	Mean (95% UI)	Mean (95% UI)	Mean (95% UI)	Mean (95% UI)
	Ischaemic stroke	3293397 (2973537–3536076)	60·7% (45·8 to 74·7%)	43·5 (39·1–46·8)	-33·6% (-39·2 to -28·2%)
	Intracerebral haemorrhage	2886196 (2644484–3099351)	37·5% (21·7 to 50·9%)	36 (33–38·7)	-35·6% (-42·8 to -29·2%)
	Subarachnoid haemorrhage	373131 (330033–415937)	-12·1% (-25·3 to 26·3%)	4·7 (4·1–5·2)	-57·4% (-63·8 to -38·8%)
	Neurological disorders†	2221323 (1027903–4759829)	154·3% (120·5 to 174·1%)	30·7 (13·8–66·3)	1·2% (-2·8 to 7·7%)
	Alzheimer's disease and other dementias	1623276 (407465–4205719)	189·4% (173·5 to 214·5%)	22·9 (5·8–59·2)	3% (-1·2 to 9·8%)
	Parkinson's disease	362907 (326855–388200)	146·5% (128·1 to 162·8%)	4·8 (4·3–5·1)	3·8% (-3·3 to 10·1%)
	Idiopathic epilepsy	114011 (100178–129928)	13·9% (-1·4 to 51·6%)	1·5 (1·3–1·7)	-24·8% (-34·2 to -1·6%)
	Multiple sclerosis	22439 (20226–27792)	68% (40·1 to 84%)	0·3 (0·2–0·3)	-14% (-29·1 to -5·9%)
	Motor neuron disease	39081 (36567–41130)	121·4% (108 to 135·4%)	0·5 (0·4–0·5)	12·4% (5·7 to 19·3%)
	Other neurological disorders	59610 (55082–64551)	74·8% (59·5 to 91·6%)	0·8 (0·7–0·8)	-0·2% (-8·2 to 9·2%)
North Africa and Middle East	Meningitis	6275 (5332–7405)	-64·1% (-72·2 to -54·5%)	1·2 (1–1·4)	-71·6% (-77·1 to -64·9%)
	Encephalitis	3926 (3148–5118)	26·2% (-9·4 to 71·2%)	0·7 (0·6–0·9)	-24·9% (-42·7 to -1·9%)
	Tetanus	1289 (823–2015)	-84·1% (-91·3 to -69%)	0·2 (0·2–0·4)	-88·9% (-93·3 to -80·6%)
	Brain and central nervous system cancer	17773 (12096–20936)	111·5% (30·3 to 173·4%)	3·7 (2·5–4·3)	3% (-33·4 to 30·2%)
	Stroke*	312220 (278450–349726)	75·5% (56·2 to 98·8%)	87·7 (78·2–97·6)	-27·8% (-35·4 to -16%)
	Ischaemic stroke	210108 (187141–234048)	141·8% (110·1 to 175%)	62·9 (56·3–69·9)	-9·1% (-20·7 to 4%)
	Intracerebral haemorrhage	88542 (77871–101100)	18% (1·5 to 37·8%)	21·6 (19–24·3)	-51·4% (-58·1 to -41·3%)
	Subarachnoid haemorrhage	13570 (11176–16789)	-15% (-35·7 to 38·8%)	3·2 (2·6–3·9)	-59% (-70·7 to -31·6%)
	Neurological disorders†	99628 (47340–213242)	153·3% (111 to 207·8%)	33·2 (14·3–73·9)	-2·8% (-9·6 to 13·9%)
	Alzheimer's disease and other dementias	70483 (17247–185789)	191·6% (169·8 to 253·2%)	25·5 (6·3–67·1)	-2·3% (-9 to 17·3%)
	Parkinson's disease	16784 (14607–21649)	179·4% (145·9 to 225·5%)	5·3 (4·6–6·9)	2·3% (-10 to 19%)
	Idiopathic epilepsy	6570 (5357–7629)	5·3% (-16·7 to 70·4%)	1·2 (1–1·4)	-33·4% (-46·2 to 2·1%)
	Multiple sclerosis	1436 (1176–1814)	147·3% (83·1 to 248%)	0·3 (0·2–0·3)	5·1% (-25 to 47·6%)
	Motor neuron disease	1068 (855–1318)	90·4% (23·9 to 177·6%)	0·2 (0·2–0·3)	9·3% (-26·7 to 51·1%)
	Other neurological disorders	3287 (2889–3778)	85·9% (42·8 to 136·2%)	0·7 (0·6–0·8)	17·4% (-7·9 to 49·9%)
Algeria	Meningitis	292 (234–363)	-65·2% (-78·1 to -47%)	0·9 (0·7–1·1)	-74·7% (-82·4 to -64%)
	Encephalitis	134 (94–253)	18·7% (-23·4 to 79·4%)	0·4 (0·3–0·7)	-23·2% (-44·7 to 7·1%)
	Tetanus	10 (7–20)	-77·1% (-90·6 to -38·5%)	0 (0–0·1)	-84·3% (-93·4 to -61·1%)
	Brain and central nervous system cancer	557 (355–702)	102% (12·2 to 175·5%)	1·5 (1–1·9)	1·2% (-43·3 to 38·9%)
	Stroke*	24811 (19995–30219)	77·1% (38·3 to 122·6%)	101·5 (82·6–121·6)	-43·4% (-54·9 to -30·2%)
	Ischaemic stroke	18650 (14812–22930)	154·5% (98·1 to 225·9%)	80·1 (64·4–96·8)	-27·9% (-42·2 to -10·5%)
	Intracerebral haemorrhage	5276 (4129–6725)	-7·7% (-30·8 to 21·9%)	18·5 (14·5–23·6)	-69·3% (-76·8 to -60·1%)
	Subarachnoid haemorrhage	885 (658–1175)	-8·1% (-36·8 to 47·8%)	2·9 (2·1–3·7)	-64·6% (-75·9 to -42·9%)
	Neurological disorders†	7191 (3170–16735)	200% (114 to 293·4%)	34·2 (13·6–81·7)	-10·5% (-26·8 to 6·2%)
	Alzheimer's disease and other dementias	5209 (1256–14968)	289·7% (212·2 to 394·7%)	27 (6·6–74·5)	-6·3% (-20·6 to 11%)
	Parkinson's disease	1283 (1019–1583)	192·2% (125·1 to 277·6%)	5·4 (4·4–6·7)	-18·7% (-36·3 to 4·6%)
	Idiopathic epilepsy	410 (313–514)	-16·3% (-39·6 to 30·7%)	1·1 (0·8–1·3)	-49·1% (-62·4 to -24·2%)
	Multiple sclerosis	101 (52–142)	166·5% (59·6 to 303%)	0·3 (0·1–0·4)	1·6% (-38·3 to 50·7%)
	Motor neuron disease	23 (17–31)	149·1% (67·4 to 278·1%)	0·1 (0–0·1)	-2·1% (-34·8 to 49·6%)
	Other neurological disorders	165 (136–198)	95·3% (32·9 to 164·6%)	0·5 (0·4–0·5)	10·4% (-20·6 to 45·5%)

Table S12 Burden, mortality, incidence, and prevalence of neurological conditions in North Africa and Middle East countries

		All Ages		Age-standardised	
		Number	Percent change	Rate per 100,000	Percent change
**Measure**		2019	From 1990 to 2019	2019	From 1990 to 2019
Cause	Location	Mean (95% UI)	Mean (95% UI)	Mean (95% UI)	Mean (95% UI)
Bahrain	Meningitis	3 (3–4)	-33·2% (-50·1 to -13%)	0·4 (0·3–0·5)	-73·1% (-78·8 to -66·1%)
	Encephalitis	2 (2–3)	95% (41·5 to 152·2%)	0·2 (0·2–0·3)	-24·2% (-42·9 to -3·5%)
	Tetanus	0 (0–0)	62·3% (-29·1 to 156·3%)	0 (0–0)	-44·1% (-76·2 to -11·3%)
	Brain and central nervous system cancer	26 (16–34)	232·8% (94·8 to 386%)	2·1 (1·4–2·7)	-25·6% (-54·8 to 8·1%)
	Stroke*	245 (200–310)	109·6% (64·1 to 168·7%)	52·8 (43·5–66·9)	-48·5% (-58·4 to -36·5%)
	Ischaemic stroke	136 (111–177)	144·8% (93·3 to 209·7%)	37·5 (30·5–47·6)	-41·4% (-53·2 to -27·1%)
	Intracerebral haemorrhage	88 (70–113)	76·1% (33·5 to 133·1%)	13·1 (10·7–16·9)	-61·2% (-70 to -50·1%)
	Subarachnoid haemorrhage	20 (15–31)	84·3% (23·7 to 177%)	2·2 (1·7–3·3)	-52·9% (-69·4 to -32·1%)
	Neurological disorders†	123 (64–251)	273·8% (183 to 359·2%)	36 (15·2–80·7)	-7·1% (-21·6 to 10·1%)
	Alzheimer's disease and other dementias	74 (18–202)	374·9% (290·8 to 482·7%)	27·3 (6·6–72)	-4·4% (-18·2 to 13·2%)
	Parkinson's disease	21 (15–27)	286·6% (187·6 to 403·7%)	6 (4·2–7·4)	-7·7% (-29·9 to 17·9%)
	Idiopathic epilepsy	17 (13–22)	102·2% (56·6 to 171·7%)	1·7 (1·3–2·1)	-34·3% (-49·1 to -13·8%)
	Multiple sclerosis	2 (1–3)	422·7% (202·3 to 628·6%)	0·1 (0·1–0·2)	-6·6% (-44·1 to 28·6%)
	Motor neuron disease	1 (0–1)	108·8% (52·1 to 183·7%)	0 (0–0·1)	-63·6% (-73·4 to -51·1%)
	Other neurological disorders	8 (6–10)	199·2% (131·5 to 284·7%)	0·8 (0·7–1)	-0·8% (-21·5 to 24·7%)
Egypt	Meningitis	764 (512–1067)	-63·9% (-76·2 to -46·5%)	0·8 (0·6–1·2)	-72·2% (-81·4 to -58·8%)
	Encephalitis	910 (508–1371)	3·6% (-36·7 to 62·8%)	1 (0·6–1·4)	-30% (-54·8 to 2·7%)
	Tetanus	136 (47–393)	-83·9% (-94·4 to -50·8%)	0·2 (0·1–0·5)	-89·2% (-95·3 to -68·8%)
	Brain and central nervous system cancer	2066 (1425–3046)	101·3% (25·7 to 185%)	2·7 (1·8–4·1)	5·4% (-28·7 to 47·6%)
	Stroke*	45767 (33557–63157)	32·2% (-2·7 to 75·5%)	85·7 (63·1–118·9)	-22·6% (-42·6 to 1·6%)
	Ischaemic stroke	30078 (21909–41497)	137% (65·8 to 212·8%)	62·3 (46–86·1)	15·3% (-17·3 to 51·5%)
	Intracerebral haemorrhage	13654 (9489–19503)	-19% (-42·5 to 13·9%)	20·7 (14·5–30·1)	-56·1% (-69·1 to -38·9%)
	Subarachnoid haemorrhage	2035 (1418–2867)	-60% (-74·5 to -18·4%)	2·8 (1·9–3·9)	-71·4% (-81·7 to -44·7%)
	Neurological disorders†	10675 (5110–22175)	109·8% (70 to 162·7%)	31·2 (12·8–70·1)	1·1% (-15·1 to 25·8%)
	Alzheimer's disease and other dementias	6918 (1554–18509)	110·8% (72 to 172·7%)	23·6 (5·4–63·1)	-1·7% (-17·6 to 23·2%)
	Parkinson's disease	2439 (1842–3675)	133·4% (84·4 to 193·2%)	6 (4·6–9·3)	12·2% (-10·6 to 38·2%)
	Idiopathic epilepsy	441 (302–644)	8·4% (-21·3 to 66·4%)	0·5 (0·3–0·7)	-34·5% (-52 to -4·4%)
	Multiple sclerosis	191 (96–426)	96·5% (-24·9 to 377·8%)	0·2 (0·1–0·4)	5·9% (-48 to 122·5%)
	Motor neuron disease	177 (98–284)	287·6% (104·4 to 557·7%)	0·2 (0·1–0·4)	85% (-4·9 to 213·5%)
	Other neurological disorders	510 (341–722)	141·1% (70·4 to 228·5%)	0·7 (0·5–1)	54·1% (9 to 104·8%)
Iran (Islamic Republic of)	Meningitis	577 (515–647)	-67·7% (-75 to -57·1%)	0·8 (0·7–0·9)	-72·3% (-78 to -64·3%)
	Encephalitis	217 (157–244)	18% (-13·1 to 52·2%)	0·3 (0·2–0·3)	-15·6% (-37·9 to 4·8%)
	Tetanus	17 (12–24)	-91·5% (-96·4 to -75·4%)	0 (0–0)	-92·3% (-96·6 to -81·3%)
	Brain and central nervous system cancer	3494 (1751–4173)	97·5% (5 to 138·5%)	4·6 (2·3–5·5)	-1·1% (-43·8 to 22·9%)
	Stroke*	40912 (36741–43849)	88·6% (67·7 to 118·5%)	66·2 (58·7–71·3)	-45·1% (-50·6 to -35·4%)
	Ischaemic stroke	33151 (29508–35673)	109·7% (83·3 to 144·4%)	54·8 (48·3–59·2)	-42·7% (-49·7 to -32·3%)
	Intracerebral haemorrhage	6308 (5792–6705)	34% (14·5 to 54·5%)	9·3 (8·4–9·9)	-52·5% (-59·6 to -43·2%)
	Subarachnoid haemorrhage	1453 (1269–1653)	23·2% (-13·9 to 95·6%)	2·1 (1·8–2·4)	-60·6% (-73·8 to -34·9%)
	Neurological disorders†	18338 (7975–41415)	276·2% (178·8 to 397·5%)	32·1 (13·2–73·9)	-1·4% (-8·6 to 27·8%)
	Alzheimer's disease and other dementias	13910 (3444–37233)	382% (336·4 to 552·3%)	25·3 (6·4–67·6)	-0·6% (-7·1 to 34·5%)
	Parkinson's disease	2848 (2432–3155)	343·1% (276·8 to 429·9%)	4·8 (4·1–5·3)	6·7% (-12·6 to 28·4%)
	Idiopathic epilepsy	662 (605–776)	-33·7% (-49·3 to 7·6%)	0·8 (0·7–1)	-51·1% (-60·7 to -26·1%)

Table S12 Burden, mortality, incidence, and prevalence of neurological conditions in North Africa and Middle East countries

		All Ages		Age-standardised	
		Number	Percent change	Rate per 100,000	Percent change
**Measure**		2019	From 1990 to 2019	2019	From 1990 to 2019
Cause	Location	Mean (95% UI)	Mean (95% UI)	Mean (95% UI)	Mean (95% UI)
	Multiple sclerosis	396 (328–587)	175·2% (96·6 to 288·4%)	0·5 (0·4–0·7)	4·8% (-28·2 to 55·7%)
	Motor neuron disease	103 (89–119)	268·2% (140 to 424·8%)	0·1 (0·1–0·2)	61·9% (4 to 134%)
	Other neurological disorders	419 (390–449)	138·6% (82·4 to 224·6%)	0·6 (0·5–0·6)	39·2% (8·1 to 95·9%)
Iraq	Meningitis	427 (336–535)	-63·2% (-73·7 to -48·1%)	1·3 (1–1·5)	-74·8% (-81·1 to -66·3%)
	Encephalitis	457 (334–590)	31·1% (-15·5 to 97·9%)	1·3 (1–1·6)	-30·8% (-51·1 to -2·8%)
	Tetanus	24 (15–39)	-90% (-96·7 to -53·6%)	0·1 (0–0·1)	-92·7% (-97·3 to -72·4%)
	Brain and central nervous system cancer	1842 (1330–2362)	215·1% (65·6 to 389·7%)	6·4 (4·7–8·2)	26% (-31·9 to 93·6%)
	Stroke*	26256 (21422–31075)	123·8% (77·4 to 177·3%)	143·3 (119·2–166·1)	-13·7% (-30·2 to 4·5%)
	Ischaemic stroke	16678 (13716–19426)	150·2% (98·3 to 210·1%)	101 (83·9–116)	-1·9% (-21·6 to 19%)
	Intracerebral haemorrhage	9012 (7054–11186)	102·5% (52·5 to 163·8%)	40 (32·2–48)	-28·3% (-45·3 to -7·8%)
	Subarachnoid haemorrhage	566 (410–796)	-8·1% (-42·6 to 65%)	2·3 (1·7–3·3)	-68·4% (-80·4 to -40·9%)
	Neurological disorders†	4654 (2139–9861)	134% (95·7 to 183·2%)	32·4 (13·3–72)	1·2% (-13·6 to 22·6%)
	Alzheimer's disease and other dementias	3259 (779–8524)	135·7% (99·4 to 182·6%)	25·3 (6·1–65·7)	-0·5% (-14·6 to 18·8%)
	Parkinson's disease	842 (684–1150)	202% (137 to 289·8%)	5·7 (4·6–7·4)	22·8% (-3·6 to 58%)
	Idiopathic epilepsy	301 (225–393)	31·1% (-6·5 to 101%)	0·8 (0·6–1)	-42·7% (-58 to -15·7%)
	Multiple sclerosis	40 (28–57)	225·3% (107·9 to 436·1%)	0·1 (0·1–0·2)	4·8% (-31·5 to 69·7%)
	Motor neuron disease	11 (8–15)	240·1% (91·6 to 463·7%)	0 (0–0·1)	19·7% (-35·8 to 111·1%)
	Other neurological disorders	201 (155–254)	144% (72·2 to 285·9%)	0·6 (0·4–0·7)	12·1% (-19·5 to 74·4%)
Jordan	Meningitis	83 (66–104)	11·3% (-20·7 to 59·6%)	0·9 (0·8–1·2)	-58·7% (-68·7 to -43·7%)
	Encephalitis	15 (11–22)	-4·5% (-43·3 to 173·6%)	0·2 (0·1–0·2)	-65% (-78·2 to -13·6%)
	Tetanus	2 (1–3)	-64·2% (-82·6 to 3·4%)	0 (0–0)	-82·4% (-91·2 to -58·2%)
	Brain and central nervous system cancer	228 (162–282)	261·5% (125·1 to 391·7%)	2·8 (2–3·5)	-5·6% (-42·1 to 30·6%)
	Stroke*	3367 (2758–3983)	136·2% (89·6 to 188·2%)	75·7 (61·3–89)	-49·8% (-59·4 to -39·2%)
	Ischaemic stroke	2472 (2020–2919)	155·9% (98·6 to 214·9%)	59·2 (47·4–69·3)	-46·9% (-57·8 to -35·2%)
	Intracerebral haemorrhage	820 (676–980)	93·7% (53·3 to 142·9%)	15·2 (12·6–18·1)	-58·3% (-66·7 to -47·5%)
	Subarachnoid haemorrhage	76 (55–94)	107·4% (41·9 to 224·3%)	1·2 (0·9–1·6)	-54·9% (-69·5 to -30·4%)
	Neurological disorders†	1094 (503–2425)	313% (232·7 to 404·2%)	30·3 (12·4–67·6)	-9·4% (-23·2 to 11·5%)
	Alzheimer's disease and other dementias	767 (188–2164)	356·6% (286·2 to 471·1%)	24 (6–62)	-7·6% (-21·4 to 15·9%)
	Parkinson's disease	192 (160–228)	332·8% (245·4 to 442·6%)	4·9 (4·1–5·8)	-13·2% (-30 to 8·6%)
	Idiopathic epilepsy	67 (51–81)	107% (54·6 to 194·2%)	0·6 (0·5–0·8)	-38·7% (-52·8 to -12·8%)
	Multiple sclerosis	17 (11–23)	308·5% (164·5 to 467·6%)	0·2 (0·1–0·2)	-14·5% (-43·1 to 18%)
	Motor neuron disease	6 (4–7)	455·5% (263·9 to 761%)	0·1 (0–0·1)	21·1% (-22·7 to 91·8%)
	Other neurological disorders	45 (38–55)	201% (120·6 to 307·9%)	0·5 (0·4–0·6)	-0·3% (-23·7 to 30·5%)
Kuwait	Meningitis	12 (10–15)	-5·6% (-28·6 to 20·3%)	0·4 (0·3–0·5)	-59·4% (-68·3 to -49·6%)
	Encephalitis	6 (5–8)	73% (40 to 119·9%)	0·2 (0·1–0·2)	-22·3% (-36·3 to -4·5%)
	Tetanus	0 (0–0)	89·1% (-77·3 to 574%)	0 (0–0)	-62·6% (-93·9 to -13·5%)
	Brain and central nervous system cancer	59 (42–74)	205% (87·4 to 288·9%)	1·9 (1·3–2·4)	1·6% (-38·5 to 31·3%)
	Stroke*	920 (757–1093)	301·8% (235·8 to 381·5%)	46·5 (38–55·2)	-9·6% (-24·2 to 7·7%)
	Ischaemic stroke	617 (504–742)	317·1% (246·1 to 401·3%)	34·2 (27·7–41)	-10·5% (-25·6 to 6·9%)
	Intracerebral haemorrhage	263 (207–319)	313·5% (222·5 to 417·9%)	10·8 (8·5–13·1)	-3·7% (-26·2 to 21·5%)
	Subarachnoid haemorrhage	40 (33–51)	130·4% (74·7 to 243·4%)	1·6 (1·3–2)	-24·8% (-43 to 3·4%)
	Neurological disorders†	532 (216–1200)	331% (223·4 to 405%)	29·9 (11·4–69)	-7·2% (-20 to 4·2%)

Table S12 Burden, mortality, incidence, and prevalence of neurological conditions in North Africa and Middle East countries

		**All Ages**		**Age-standardised**	
		Number	Percent change	Rate per 100,000	Percent change
**Measure**		2019	From 1990 to 2019	2019	From 1990 to 2019
Cause	Location	Mean (95% UI)	Mean (95% UI)	Mean (95% UI)	Mean (95% UI)
	Alzheimer's disease and other dementias	425 (110–1086)	439·9% (380·6 to 519·8%)	25·1 (6·5–64)	-2·1% (-12·8 to 10·4%)
	Parkinson's disease	58 (47–69)	253·2% (199·8 to 317·8%)	3·5 (2·8–4·2)	-25·1% (-36·3 to -11·7%)
	Idiopathic epilepsy	28 (23–35)	65·6% (38 to 112·8%)	0·7 (0·6–0·9)	-30·6% (-42·2 to -13%)
	Multiple sclerosis	5 (4–7)	300·3% (202·6 to 437·9%)	0·1 (0·1–0·2)	0·8% (-23 to 34·7%)
	Motor neuron disease	4 (3–5)	12·5% (-20·2 to 52·2%)	0·1 (0·1–0·2)	-54·4% (-66·8 to -38·7%)
	Other neurological disorders	12 (10–14)	81·1% (39·7 to 130·5%)	0·4 (0·3–0·4)	-24·6% (-40·4 to -5·3%)
Lebanon	Meningitis	34 (25–45)	-40% (-59·3 to -15·2%)	0·7 (0·5–0·9)	-64% (-75·3 to -50·8%)
	Encephalitis	15 (10–28)	38·4% (-6 to 106·6%)	0·3 (0·2–0·5)	-19% (-42·9 to 19·7%)
	Tetanus	23 (5–99)	-52·6% (-90·5 to 57·5%)	0·5 (0·1–1·9)	-79·9% (-95·6 to -34·5%)
	Brain and central nervous system cancer	168 (122–220)	82·5% (24·5 to 153·2%)	3·2 (2·3–4·2)	-9% (-37·6 to 25·4%)
	Stroke*	1764 (1236–2298)	81% (30·1 to 143·2%)	35·2 (24·5–45·6)	-39·5% (-55·5 to -19·2%)
	Ischaemic stroke	1470 (986–1903)	140·4% (63·2 to 215·4%)	29·4 (19·6–38·2)	-25% (-48 to -1·1%)
	Intracerebral haemorrhage	257 (188–351)	-16·7% (-40·7 to 19·7%)	5 (3·7–6·9)	-69·1% (-78·1 to -55·1%)
	Subarachnoid haemorrhage	37 (21–54)	-32·3% (-66·5 to 27·1%)	0·7 (0·4–1)	-72·7% (-86·7 to -47·5%)
	Neurological disorders†	1578 (622–3760)	210·9% (145·6 to 344·6%)	33 (12·6–78·5)	-4·9% (-21·7 to 37·3%)
	Alzheimer's disease and other dementias	1255 (306–3375)	246% (186·4 to 425·1%)	26·8 (6·5–70·8)	-2·6% (-19·7 to 48%)
	Parkinson's disease	226 (180–312)	171·3% (98·1 to 266·4%)	4·4 (3·5–6)	-14·1% (-37·2 to 15·7%)
	Idiopathic epilepsy	46 (34–63)	11·6% (-19·2 to 62·4%)	0·9 (0·6–1·2)	-36·7% (-54·2 to -10·2%)
	Multiple sclerosis	15 (8–21)	128·8% (49·9 to 257·5%)	0·3 (0·1–0·4)	7·5% (-29·5 to 66·7%)
	Motor neuron disease	4 (2–8)	111·7% (19·5 to 245·5%)	0·1 (0–0·1)	7·3% (-39·3 to 75·9%)
	Other neurological disorders	31 (24–41)	171·8% (93·4 to 278·8%)	0·6 (0·5–0·8)	46% (3·4 to 100·9%)
Libya	Meningitis	43 (32–56)	-52·1% (-68·5 to -30·9%)	0·8 (0·6–1)	-61·9% (-73·8 to -47·3%)
	Encephalitis	19 (12–37)	34·3% (-8·3 to 94·4%)	0·3 (0·2–0·7)	-9·6% (-35 to 33·9%)
	Tetanus	1 (1–3)	-57% (-83·6 to 8·3%)	0 (0–0·1)	-68·6% (-89·3 to -26·5%)
	Brain and central nervous system cancer	217 (161–289)	114·8% (50·6 to 210·9%)	3·7 (2·8–4·9)	-0·9% (-28·4 to 40·2%)
	Stroke*	3086 (2364–4016)	97·1% (51·4 to 161·9%)	69·4 (53–90·1)	-17·9% (-36·4 to 7·9%)
	Ischaemic stroke	2301 (1732–3073)	181·8% (117·4 to 274·3%)	54·1 (40·9–72·5)	5·7% (-18·7 to 38·2%)
	Intracerebral haemorrhage	678 (497–936)	15·4% (-15·4 to 60%)	13·3 (9·7–18·1)	-52·9% (-65·2 to -35·6%)
	Subarachnoid haemorrhage	107 (73–156)	-33·9% (-57·7 to 27·1%)	2 (1·4–2·9)	-60·9% (-73·2 to -36·3%)
	Neurological disorders†	1438 (613–3179)	154·3% (102·3 to 218·1%)	35·8 (14·3–80·8)	-1·9% (-19·7 to 23·8%)
	Alzheimer's disease and other dementias	1094 (276–2789)	162·3% (114 to 232·1%)	28·6 (7·2–72·8)	-3·8% (-21·4 to 22%)
	Parkinson's disease	203 (150–265)	202·9% (125 to 314·9%)	5 (3·7–6·5)	9·9% (-18·1 to 50·5%)
	Idiopathic epilepsy	75 (55–101)	23·2% (-11·1 to 80·8%)	1·1 (0·8–1·5)	-25·7% (-44·4 to 3·3%)
	Multiple sclerosis	21 (11–35)	288·9% (132·2 to 591·5%)	0·3 (0·2–0·5)	38·4% (-17·3 to 139·5%)
	Motor neuron disease	6 (3–9)	297·9% (82·7 to 653·6%)	0·1 (0·1–0·1)	51·4% (-30·9 to 187%)
	Other neurological disorders	39 (29–51)	190·1% (89·2 to 360%)	0·7 (0·5–0·8)	58·5% (6·7 to 145·6%)
Morocco	Meningitis	400 (288–511)	-74·8% (-83·3 to -63·5%)	1·3 (1–1·7)	-74·7% (-82·4 to -65·3%)
	Encephalitis	150 (83–309)	18·5% (-23·8 to 72·5%)	0·5 (0·3–1)	-10·9% (-36·4 to 24·4%)
	Tetanus	113 (30–374)	-93·7% (-98·5 to -77%)	0·4 (0·1–1·3)	-92·6% (-98·1 to -76·1%)
	Brain and central nervous system cancer	479 (344–639)	91·3% (24·4 to 168·9%)	1·4 (1–1·9)	5·9% (-28·1 to 45·2%)
	Stroke*	29033 (23331–35135)	96·6% (55·4 to 142·5%)	116·4 (94·2–139·3)	-11·9% (-29·1 to 8%)
	Ischaemic stroke	21220 (16956–25800)	184% (118·6 to 248·7%)	88·9 (71·8–108·4)	19% (-6·7 to 43·8%)
	Intracerebral haemorrhage	6700 (5090–8430)	10·4% (-19·5 to 48%)	23·6 (18·4–29·1)	-51·2% (-63·6 to -34·6%)

Table S12 Burden, mortality, incidence, and prevalence of neurological conditions in North Africa and Middle East countries

		All Ages		Age-standardised	
		Number	Percent change	Rate per 100,000	Percent change
**Measure**		2019	From 1990 to 2019	2019	From 1990 to 2019
Cause	Location	Mean (95% UI)	Mean (95% UI)	Mean (95% UI)	Mean (95% UI)
	Subarachnoid haemorrhage	1113 (763–1621)	-9·3% (-39·6 to 56·5%)	3·9 (2·8–5·6)	-56·8% (-71·3 to -21·3%)
	Neurological disorders†	6917 (3047–15662)	160·3% (118·3 to 199·1%)	33·1 (13·6–78)	6·7% (-8·8 to 26·7%)
	Alzheimer's disease and other dementias	4945 (1122–13768)	161·9% (120·2 to 204·4%)	25·4 (5·9–70·2)	0·7% (-14·2 to 16·1%)
	Parkinson's disease	1390 (1122–1614)	244·3% (174·9 to 340·4%)	6 (4·9–7)	46·6% (16·7 to 85·9%)
	Idiopathic epilepsy	304 (101–846)	19·9% (-21·2 to 82·6%)	0·9 (0·3–2·5)	-21·8% (-45·3 to 13·6%)
	Multiple sclerosis	99 (51–148)	177·1% (74·4 to 334·2%)	0·3 (0·1–0·4)	31·5% (-16·5 to 103·4%)
	Motor neuron disease	22 (15–30)	193·5% (84 to 332·1%)	0·1 (0–0·1)	35% (-15·5 to 101·1%)
	Other neurological disorders	157 (111–204)	129·5% (71·1 to 211·1%)	0·5 (0·3–0·6)	48·6% (12 to 96·5%)
Palestine	Meningitis	28 (23–33)	-72·2% (-81·1 to -58·8%)	0·9 (0·8–1·1)	-77·8% (-83·4 to -70·1%)
	Encephalitis	10 (8–15)	50·3% (-3 to 118·4%)	0·3 (0·2–0·4)	-22% (-43 to 6·7%)
	Tetanus	1 (1–1)	-88·4% (-96·7 to -47·6%)	0 (0–0)	-90·6% (-97·2 to -66·4%)
	Brain and central nervous system cancer	210 (159–251)	137·9% (52·4 to 224·3%)	7·2 (5·2–8·7)	-3·7% (-34·8 to 31·5%)
	Stroke*	2019 (1740–2286)	59·8% (25·5 to 102·3%)	122·4 (105·6–138·2)	-29·1% (-43·5 to -10·1%)
	Ischaemic stroke	1511 (1288–1720)	89·4% (48·2 to 142·7%)	96·5 (82–109·6)	-15·9% (-33 to 6·2%)
	Intracerebral haemorrhage	468 (395–549)	7·6% (-17 to 41·9%)	24·1 (20·3–28)	-55·6% (-65·6 to -42·4%)
	Subarachnoid haemorrhage	40 (32–48)	28·5% (-13·1 to 95·7%)	1·8 (1·5–2·2)	-49·4% (-66·2 to -22·8%)
	Neurological disorders†	509 (261–1116)	96·8% (60·8 to 145·7%)	34·5 (14·9–80·3)	-6·6% (-22·6 to 16·4%)
	Alzheimer's disease and other dementias	326 (76–926)	98% (65·5 to 145%)	25·9 (6·1–72·1)	-6·1% (-21 to 15·6%)
	Parkinson's disease	92 (61–107)	118·5% (64·7 to 206·9%)	6·2 (4–7·2)	-2·5% (-26·2 to 36·2%)
	Idiopathic epilepsy	63 (54–77)	56·4% (16·7 to 142·5%)	1·6 (1·4–2)	-29·9% (-47·5 to 3%)
	Multiple sclerosis	8 (6–12)	214·7% (89·1 to 448·7%)	0·2 (0·2–0·4)	1·6% (-37·7 to 77·2%)
	Motor neuron disease	1 (1–2)	285% (131·8 to 525·1%)	0 (0–0·1)	50·6% (-15·5 to 165·2%)
	Other neurological disorders	17 (15–20)	116·4% (43·9 to 267·2%)	0·5 (0·4–0·6)	15·9% (-19 to 95·8%)
Oman	Meningitis	14 (12–16)	-52·8% (-65·4 to -32·6%)	0·9 (0·8–1·1)	-64·2% (-73·3 to -49·6%)
	Encephalitis	83 (63–126)	21·6% (-19·6 to 83·9%)	3·2 (2·5–3·9)	-43·4% (-62·2 to -9·9%)
	Tetanus	1 (1–1)	-78·3% (-92·1 to -17·8%)	0 (0–0)	-92·9% (-98·7 to -57·8%)
	Brain and central nervous system cancer	54 (32–70)	176·7% (39·6 to 318·9%)	2·1 (1·4–2·6)	16·6% (-39 to 73·2%)
	Stroke*	1030 (895–1196)	36·7% (4·9 to 81·9%)	103·7 (90·7–118·7)	-28·8% (-45 to -3·7%)
	Ischaemic stroke	650 (557–758)	82·1% (38·5 to 148·3%)	76·6 (63·8–89·9)	-7·7% (-30·7 to 24·4%)
	Intracerebral haemorrhage	323 (276–408)	-1·8% (-25·4 to 31·6%)	24 (20·4–30·5)	-56·6% (-67·7 to -40%)
	Subarachnoid haemorrhage	58 (37–98)	-15·5% (-50·8 to 71·4%)	3·1 (1·8–4·7)	-58·1% (-77·1 to -20·2%)
	Neurological disorders†	257 (130–543)	131·4% (91·2 to 213·3%)	39·1 (17·1–90·1)	4·7% (-12·2 to 49·9%)
	Alzheimer's disease and other dementias	163 (37–446)	117·5% (79·4 to 214·4%)	28·8 (6·7–81)	-3·5% (-20·3 to 46·7%)
	Parkinson's disease	60 (37–70)	185·9% (105·3 to 287·6%)	9·1 (4·9–10·7)	44·7% (6·3 to 96%)
	Idiopathic epilepsy	12 (10–17)	42·5% (-3·6 to 152·9%)	0·4 (0·3–0·5)	-37·8% (-56·6 to -1·6%)
	Multiple sclerosis	8 (4–12)	248·6% (73·1 to 534·5%)	0·3 (0·1–0·4)	16·2% (-42·4 to 115·1%)
	Motor neuron disease	2 (1–3)	235·5% (71·8 to 471·5%)	0·1 (0–0·1)	25·2% (-37·3 to 123·4%)
	Other neurological disorders	13 (10–16)	215·4% (88·9 to 389%)	0·5 (0·4–0·6)	62·6% (1·1 to 157·2%)
Qatar	Meningitis	5 (4–7)	8·8% (-31·3 to 63%)	0·6 (0·4–0·8)	-73·4% (-84·2 to -60·4%)
	Encephalitis	4 (3–6)	299·1% (154·3 to 482·7%)	0·3 (0·2–0·4)	-24·6% (-49 to 11·7%)
	Tetanus	0 (0–0)	225·3% (13·4 to 520·2%)	0 (0–0)	-50·7% (-85·6 to 38·3%)
	Brain and central nervous system cancer	37 (24–62)	485·3% (234·8 to 814·8%)	2·8 (2·1–4)	-16·8% (-46·7 to 21·7%)

Table S12 Burden, mortality, incidence, and prevalence of neurological conditions in North Africa and Middle East countries

		All Ages		Age-standardised	
		Number	Percent change	Rate per 100,000	Percent change
**Measure**		2019	From 1990 to 2019	2019	From 1990 to 2019
Cause	Location	Mean (95% UI)	Mean (95% UI)	Mean (95% UI)	Mean (95% UI)
	Stroke*	181 (136–239)	197·2% (112·5 to 306·7%)	52·9 (42·5–69·2)	-36·7% (-54 to -15·7%)
	Ischaemic stroke	70 (53–97)	195·5% (108·4 to 300·9%)	36·9 (29·4–48·4)	-28·6% (-47·9 to -4·3%)
	Intracerebral haemorrhage	77 (57–106)	187·7% (95·8 to 302·8%)	12·6 (9·8–16·3)	-49·3% (-64 to -28·1%)
	Subarachnoid haemorrhage	34 (24–46)	225·6% (85·7 to 425·6%)	3·5 (2·6–4·8)	-51·2% (-72·8 to -12·8%)
	Neurological disorders†	83 (46–161)	390·2% (267·2 to 553·5%)	40·3 (18·1–90·3)	10·9% (-10·4 to 40·8%)
	Alzheimer's disease and other dementias	41 (10–120)	409·8% (276·9 to 594·1%)	27·9 (6·5–76)	5·8% (-14·1 to 28·2%)
	Parkinson's disease	19 (13–26)	450·7% (294·9 to 662·2%)	11·1 (6·5–14·7)	35·3% (1·2 to 78·3%)
	Idiopathic epilepsy	11 (8–15)	269·9% (170·8 to 453·3%)	0·5 (0·4–0·7)	-40·4% (-55·9 to -15·8%)
	Multiple sclerosis	2 (1–3)	594·2% (327·3 to 934·6%)	0·1 (0·1–0·1)	-14·3% (-44·8 to 29·6%)
	Motor neuron disease	1 (1–1)	697·7% (382·4 to 1191·9%)	0 (0–0·1)	-4·2% (-46 to 63·4%)
	Other neurological disorders	9 (6–12)	348·7% (207 to 595·6%)	0·7 (0·5–0·9)	-10·8% (-38 to 41·8%)
Saudi Arabia	Meningitis	180 (134–240)	-30·7% (-49·2 to -1·3%)	0·9 (0·7–1·1)	-69·7% (-77·9 to -55·6%)
	Encephalitis	186 (137–254)	19% (-19 to 87·5%)	0·7 (0·6–0·9)	-51·8% (-67·4 to -22·7%)
	Tetanus	14 (5–25)	-91·4% (-98·9 to -52·9%)	0 (0–0·1)	-95·5% (-99·1 to -73·3%)
	Brain and central nervous system cancer	597 (437–915)	291·2% (111·7 to 651%)	2·3 (1·7–3·5)	32·8% (-27·6 to 148·5%)
	Stroke*	12669 (9750–15359)	83·2% (32·6 to 152·7%)	102·7 (80·4–120·5)	-32·5% (-49·1 to -10%)
	Ischaemic stroke	7034 (5262–8441)	93·4% (39·7 to 159·4%)	70·1 (53·5–82·2)	-22·8% (-41·9 to 2·1%)
	Intracerebral haemorrhage	5292 (3981–6647)	74·4% (23·4 to 158·7%)	31·1 (24–37·4)	-46·3% (-60·6 to -22·4%)
	Subarachnoid haemorrhage	344 (235–473)	41·4% (-22·5 to 148·8%)	1·5 (1·1–2)	-57·5% (-76·8 to -25·9%)
	Neurological disorders†	3083 (1725–5891)	116·5% (71·7 to 197·8%)	36·3 (16·7–78·1)	-1·9% (-19·5 to 35·2%)
	Alzheimer's disease and other dementias	1707 (411–4518)	106·2% (67·4 to 196·6%)	26·3 (6·3–68·4)	-2·1% (-19·6 to 41%)
	Parkinson's disease	562 (414–665)	108% (58·2 to 207·5%)	7·3 (5–8·7)	0·2% (-22·2 to 47·7%)
	Idiopathic epilepsy	602 (439–838)	141·4% (41·8 to 298·1%)	1·8 (1·4–2·4)	-14·4% (-49·4 to 37·2%)
	Multiple sclerosis	40 (27–60)	348·6% (142·5 to 1082·3%)	0·1 (0·1–0·2)	17·7% (-33·3 to 198·9%)
	Motor neuron disease	20 (13–29)	287·5% (87 to 737·1%)	0·1 (0·1–0·1)	24% (-40·7 to 186·8%)
	Other neurological disorders	151 (115–201)	143·7% (49·6 to 327·5%)	0·7 (0·5–0·8)	19·2% (-23·7 to 114·1%)
Syrian Arab Republic	Meningitis	214 (165–276)	-74·4% (-82·4 to -63·1%)	1·7 (1·4–2·2)	-64·9% (-74·9 to -51·9%)
	Encephalitis	59 (44–82)	-42·5% (-64·6 to 1·8%)	0·5 (0·4–0·7)	-40·9% (-61·5 to -1·3%)
	Tetanus	5 (3–11)	-96·4% (-98·9 to -86·6%)	0 (0–0·1)	-93·7% (-98 to -78·8%)
	Brain and central nervous system cancer	566 (388–768)	73·8% (6·8 to 160·3%)	4·5 (3–6)	-2·6% (-34 to 43·1%)
	Stroke*	9186 (7117–11838)	24·4% (-8·8 to 68·6%)	99 (78·3–124·9)	-32·2% (-50·2 to -8·6%)
	Ischaemic stroke	4954 (3849–6348)	58·3% (10·8 to 111·7%)	58·3 (46·2–73·1)	-20·6% (-42·8 to 6·2%)
	Intracerebral haemorrhage	3895 (2952–5174)	11·3% (-20·6 to 55·8%)	37·9 (29·1–49·4)	-42·3% (-58·3 to -20·1%)
	Subarachnoid haemorrhage	336 (250–438)	-55·5% (-70·1 to -25·7%)	2·8 (2·1–3·7)	-59·5% (-73·3 to -35·3%)
	Neurological disorders†	2208 (956–5130)	77·4% (35·5 to 138·7%)	33 (13·1–78·3)	4·9% (-16 to 40%)
	Alzheimer's disease and other dementias	1592 (368–4596)	75% (34·9 to 142·1%)	26 (6·3–70·3)	3·1% (-18·3 to 44·3%)
	Parkinson's disease	434 (286–555)	151% (86·5 to 240·1%)	5·7 (3·7–7·2)	21·1% (-8·4 to 61·8%)
	Idiopathic epilepsy	87 (63–117)	-8·3% (-37·9 to 63·4%)	0·6 (0·5–0·8)	-33·3% (-53·5 to 12·7%)
	Multiple sclerosis	18 (12–25)	123·7% (39·3 to 273·7%)	0·1 (0·1–0·2)	4·4% (-34·3 to 71·8%)
	Motor neuron disease	10 (6–17)	187·2% (55·7 to 392%)	0·1 (0–0·1)	34·7% (-25·8 to 136·8%)
	Other neurological disorders	67 (52–86)	21·2% (-17·9 to 89·9%)	0·6 (0·4–0·7)	12·7% (-21·6 to 76·8%)
Tunisia	Meningitis	85 (61–115)	-61·5% (-75·4 to -40·8%)	0·8 (0·6–1)	-69·3% (-79·3 to -55·4%)

Table S12 Burden, mortality, incidence, and prevalence of neurological conditions in North Africa and Middle East countries

**Measure**		**All Ages**		**Age-standardised**	
		Number	Percent change	Rate per 100,000	Percent change
		2019	From 1990 to 2019	2019	From 1990 to 2019
Cause	Location	Mean (95% UI)	Mean (95% UI)	Mean (95% UI)	Mean (95% UI)
	Encephalitis	36 (23–72)	8·4% (-32·7 to 62·4%)	0·3 (0·2–0·6)	-23·8% (-48·9 to 11·9%)
	Tetanus	2 (1–5)	-75·5% (-90·8 to -38·4%)	0 (0–0)	-80·6% (-92·5 to -51·2%)
	Brain and central nervous system cancer	138 (94–186)	91·6% (22·4 to 175·2%)	1·1 (0·8–1·5)	0·5% (-34·6 to 44·2%)
	Stroke*	8713 (6627–11147)	114·5% (59 to 186%)	80 (60·6–101·4)	-25% (-43·8 to -0·6%)
	Ischaemic stroke	6748 (5045–8677)	204·9% (114·3 to 304·8%)	63·2 (47·3–80·4)	-2·2% (-29·8 to 27·8%)
	Intracerebral haemorrhage	1692 (1254–2247)	10·3% (-23·7 to 59·9%)	14·5 (10·8–19·1)	-59·4% (-71·5 to -41·3%)
	Subarachnoid haemorrhage	273 (186–385)	-13·3% (-48·6 to 52·4%)	2·3 (1·6–3·2)	-64·2% (-79·3 to -35·6%)
	Neurological disorders†	3229 (1267–7379)	225·2% (139 to 332·1%)	32·7 (12·4–75·9)	-2·1% (-22·3 to 32·4%)
	Alzheimer's disease and other dementias	2547 (597–6753)	270·1% (189 to 428%)	26·5 (6·3–70·3)	-2·8% (-22·7 to 37%)
	Parkinson's disease	485 (356–646)	237·3% (150·4 to 341·6%)	4·6 (3·4–6)	12% (-16·4 to 45·8%)
	Idiopathic epilepsy	101 (71–139)	-17% (-43·8 to 26%)	0·9 (0·6–1·2)	-40·6% (-58·7 to -11·3%)
	Multiple sclerosis	35 (18–52)	178·5% (75·4 to 355·8%)	0·3 (0·1–0·4)	24·3% (-22 to 102·2%)
	Motor neuron disease	9 (6–13)	178·8% (57·8 to 386·7%)	0·1 (0–0·1)	25·3% (-30 to 119·5%)
	Other neurological disorders	53 (38–72)	121·4% (46 to 215%)	0·4 (0·3–0·6)	35·9% (-9 to 91·1%)
Türkiye	Meningitis	351 (288–422)	-85·3% (-90·5 to -77·2%)	0·5 (0·4–0·6)	-86·7% (-91·2 to -80·4%)
	Encephalitis	231 (179–287)	-31% (-56·3 to 4·8%)	0·3 (0·2–0·4)	-50·3% (-66·7 to -25·5%)
	Tetanus	16 (11–23)	-97·7% (-98·6 to -94·1%)	0 (0–0)	-98·7% (-99·3 to -96%)
	Brain and central nervous system cancer	4074 (1833–5724)	74·1% (-4·1 to 156·7%)	4·7 (2·1–6·6)	-10·8% (-48·4 to 27·2%)
	Stroke*	48947 (39204–59511)	121% (59 to 179·6%)	60·6 (48·7–73·6)	-16·5% (-39·5 to 6·2%)
	Ischaemic stroke	30216 (24111–36742)	173·4% (88·9 to 259·4%)	38·3 (30·5–46·5)	-3·7% (-32·5 to 26·5%)
	Intracerebral haemorrhage	15611 (12430–19184)	74·2% (26 to 123·4%)	18·7 (15–22·9)	-30·5% (-49 to -9·9%)
	Subarachnoid haemorrhage	3120 (2421–3942)	46·3% (-11·3 to 180·6%)	3·6 (2·8–4·5)	-39·1% (-63·8 to 20·4%)
	Neurological disorders†	26636 (12094–60752)	137·3% (80 to 193·6%)	34·5 (15·5–78·3)	-6·9% (-22·9 to 11·4%)
	Alzheimer's disease and other dementias	19721 (5070–53643)	190·5% (141·7 to 254%)	25·8 (6·6–70)	-3·8% (-20·1 to 16·3%)
	Parkinson's disease	3838 (2916–7139)	164·1% (106 to 229·6%)	4·9 (3·7–9·1)	-6·8% (-27 to 16·1%)
	Idiopathic epilepsy	1361 (1110–1654)	-17% (-41·6 to 61·2%)	1·7 (1·4–2·1)	-34·1% (-56·8 to 25·7%)
	Multiple sclerosis	206 (151–292)	53·9% (-13·4 to 151·5%)	0·2 (0·2–0·3)	-23·2% (-53·2 to 26%)
	Motor neuron disease	624 (481–789)	44·6% (-12·4 to 119·7%)	0·8 (0·6–0·9)	-4·7% (-38·7 to 39·2%)
	Other neurological disorders	886 (723–1085)	14·3% (-17·1 to 60·8%)	1·1 (0·9–1·4)	-14·7% (-36·2 to 19%)
United Arab Emirates	Meningitis	54 (32–80)	70·1% (4·9 to 145·4%)	0·8 (0·5–1·2)	-67·5% (-76·9 to -58·1%)
	Encephalitis	25 (13–53)	294% (144·7 to 494·9%)	0·3 (0·2–0·6)	-18·7% (-41·6 to 15·4%)
	Tetanus	33 (12–55)	2·1% (-78·1 to 288·8%)	2 (0·3–3·5)	-85·5% (-95·9 to -33·6%)
	Brain and central nervous system cancer	314 (180–465)	572·5% (332·1 to 902·9%)	4·2 (2·6–5·7)	-6·6% (-43·9 to 46·8%)
	Stroke*	2168 (1544–3026)	348·7% (202 to 555%)	91·3 (70·2–118·7)	-50% (-62 to -34·9%)
	Ischaemic stroke	1240 (891–1675)	458·2% (277·6 to 709·3%)	71·4 (55·2–91·1)	-41·1% (-55·1 to -23·6%)
	Intracerebral haemorrhage	812 (534–1257)	264·2% (123 to 462·5%)	17·9 (12·5–27·2)	-67·8% (-77·7 to -54·3%)
	Subarachnoid haemorrhage	117 (53–208)	205·7% (71·8 to 453·8%)	2 (0·9–3·2)	-65·2% (-78·1 to -42·5%)
	Neurological disorders†	541 (352–839)	544% (363·6 to 791·4%)	35·6 (16·8–76·6)	-7% (-22·2 to 23·5%)
	Alzheimer's disease and other dementias	138 (31–390)	496·6% (359·4 to 708·4%)	24·2 (5·9–64·2)	-6·1% (-19·9 to 26·8%)
	Parkinson's disease	90 (60–123)	527·4% (356 to 824·1%)	8 (4·8–10·4)	-12·8% (-32·3 to 23·5%)
	Idiopathic epilepsy	141 (86–220)	340·1% (173·1 to 610·8%)	1·4 (0·9–2·1)	-31·9% (-52·9 to 1·1%)
	Multiple sclerosis	44 (20–80)	1132·7% (586·4 to 1830·3%)	0·4 (0·2–0·7)	24·9% (-27·7 to 81·3%)

Table S12 Burden, mortality, incidence, and prevalence of neurological conditions in North Africa and Middle East countries

		All Ages		Age-standardised	
		Number	Percent change	Rate per 100,000	Percent change
**Measure**		2019	From 1990 to 2019	2019	From 1990 to 2019
Cause	Location	Mean (95% UI)	Mean (95% UI)	Mean (95% UI)	Mean (95% UI)
	Motor neuron disease	13 (5–26)	1180·6% (407·6 to 2470·6%)	0·2 (0·1–0·3)	24·9% (-49·3 to 143·6%)
	Other neurological disorders	115 (69–173)	1061·4% (587·3 to 1672·4%)	1·5 (0·9–2·1)	67·4% (6·1 to 138·3%)
Yemen	Meningitis	479 (330–684)	-47·4% (-66·3 to -19·7%)	2 (1·4–2·7)	-60·2% (-72 to -44·3%)
	Encephalitis	148 (86–291)	62·9% (-5·7 to 178·4%)	0·5 (0·3–1)	-2% (-33·4 to 47·6%)
	Tetanus	58 (27–119)	-83·6% (-94·6 to -35·4%)	0·2 (0·1–0·5)	-87·7% (-95·1 to -59·4%)
	Brain and central nervous system cancer	653 (398–950)	174·2% (48·6 to 386·6%)	3·4 (2·2–5·1)	21·7% (-29·2 to 93%)
	Stroke*	14375 (10909–18865)	108·6% (63·7 to 174·6%)	135·7 (102·4–176·6)	-22·1% (-38·2 to 0·7%)
	Ischaemic stroke	8921 (6871–11744)	247·2% (169·8 to 350·3%)	93·3 (71·2–121·2)	16·4% (-7·7 to 47·9%)
	Intracerebral haemorrhage	4607 (3395–6206)	25·2% (-7·6 to 69·9%)	36 (26·8–46·8)	-55·8% (-66·7 to -40·7%)
	Subarachnoid haemorrhage	846 (418–1437)	31·6% (-6·7 to 111·7%)	6·4 (3·2–11·1)	-49·2% (-63·4 to -17·8%)
	Neurological disorders†	2651 (1275–5794)	170% (94·7 to 258·2%)	32·1 (12·5–77·4)	1·8% (-13·6 to 25·9%)
	Alzheimer's disease and other dementias	1731 (403–4750)	227·9% (174·9 to 303·6%)	25·5 (6·2–71·1)	0·7% (-14 to 21·5%)
	Parkinson's disease	413 (322–540)	246% (169·5 to 358·9%)	4·6 (3·6–6)	21·9% (-3·2 to 58·7%)
	Idiopathic epilepsy	382 (251–536)	31·4% (-15 to 180·6%)	1·4 (0·9–1·9)	-29·8% (-52·2 to 19·6%)
	Multiple sclerosis	41 (20–67)	270·7% (103·4 to 567·6%)	0·2 (0·1–0·3)	28·9% (-26·4 to 121·5%)
	Motor neuron disease	7 (5–11)	223% (90·7 to 460·4%)	0 (0–0·1)	24% (-31·2 to 125·4%)
	Other neurological disorders	76 (54–108)	151·4% (47·2 to 333·8%)	0·3 (0·3–0·5)	34·3% (-13·1 to 129%)
Afghanistan	Meningitis	1563 (1134–2159)	-27·6% (-52 to 6·8%)	4·3 (3·1–5·7)	-67·3% (-77·2 to -55·4%)
	Encephalitis	1016 (727–1612)	124·7% (57·2 to 209%)	4·8 (3·6–6·2)	-11·1% (-36·6 to 19·6%)
	Tetanus	792 (476–1309)	-73·8% (-86·9 to -43·4%)	2·5 (1·4–3·8)	-87·7% (-92·8 to -77·3%)
	Brain and central nervous system cancer	1014 (569–1848)	140·1% (58 to 293·1%)	4·7 (2·8–8·5)	3·2% (-32·7 to 59·4%)
	Stroke*	16816 (11747–21934)	39·3% (7·3 to 74·3%)	161·5 (110·3–208·5)	-18·7% (-36·1 to -1·6%)
	Ischaemic stroke	8673 (6211–11616)	107·2% (59·7 to 164·3%)	98 (69·2–129·4)	23·6% (-3·3 to 55·2%)
	Intracerebral haemorrhage	6961 (4904–9333)	2·4% (-23·5 to 35·6%)	54·1 (36·6–72·4)	-47·4% (-60 to -33·1%)
	Subarachnoid haemorrhage	1183 (363–2053)	8·3% (-24·9 to 76·4%)	9·4 (2·8–17)	-42·7% (-58·3 to -11·8%)
	Neurological disorders†	3434 (1983–6537)	62·7% (37·1 to 99·1%)	41·1 (17·7–92·4)	-6·7% (-22·5 to 7·6%)
	Alzheimer's disease and other dementias	1775 (422–4801)	59·1% (32 to 85·4%)	30·8 (7·5–82·3)	-3·3% (-18·5 to 10%)
	Parkinson's disease	560 (431–715)	50·8% (17·4 to 90·2%)	6·8 (5·3–8·5)	-2·3% (-22·7 to 20·3%)
	Idiopathic epilepsy	860 (434–1211)	62% (19·4 to 184·5%)	2·5 (1·3–3·5)	-41·3% (-55 to -8·7%)
	Multiple sclerosis	90 (36–205)	186·7% (81 to 382·9%)	0·4 (0·2–0·9)	8·8% (-28·9 to 66·6%)
	Motor neuron disease	11 (8–17)	92·7% (24·9 to 201·1%)	0·1 (0–0·1)	-8·7% (-41·4 to 43·1%)
	Other neurological disorders	138 (101–185)	151·2% (77·1 to 268·8%)	0·5 (0·4–0·7)	5·5% (-23·1 to 49·9%)
Sudan	Meningitis	662 (452–951)	-76·6% (-84·7 to -64·3%)	1·9 (1·3–2·5)	-80·2% (-86·3 to -71·3%)
	Encephalitis	198 (115–375)	23·2% (-32·4 to 123·2%)	0·5 (0·3–1)	-15·8% (-47·2 to 30·9%)
	Tetanus	38 (18–82)	-91·8% (-96·7 to -76·9%)	0·1 (0·1–0·2)	-93·1% (-97 to -85·4%)
	Brain and central nervous system cancer	961 (600–1387)	93·2% (-1·7 to 278·8%)	3·6 (2·3–5·1)	8·2% (-38·1 to 84%)
	Stroke*	19638 (14425–27765)	34·7% (8·4 to 70%)	125·2 (92·6–174·9)	-29·4% (-42·2 to -12%)
	Ischaemic stroke	13107 (9770–18878)	115·4% (70·7 to 171·4%)	90·3 (66·7–129)	4·9% (-14·6 to 29·6%)
	Intracerebral haemorrhage	5660 (3818–7963)	-21·7% (-40·6 to 3·9%)	30·4 (20·9–42·2)	-61·5% (-70·1 to -49%)
	Subarachnoid haemorrhage	872 (472–1495)	-31·1% (-52·4 to 35·5%)	4·5 (2·4–7·6)	-63·4% (-75·2 to -30·9%)
	Neurological disorders†	4356 (2098–9385)	85% (31·1 to 138·4%)	31·4 (13–72·5)	-6·9% (-21·2 to 10·2%)
	Alzheimer's disease and other dementias	2815 (663–8000)	132·5% (103·4 to 176·2%)	23·9 (5·6–66)	-5·1% (-16·2 to 11·2%)

Table S12 Burden, mortality, incidence, and prevalence of neurological conditions in North Africa and Middle East countries

		All Ages		Age-standardised	
		Number	Percent change	Rate per 100,000	Percent change
**Measure**		2019	From 1990 to 2019	2019	From 1990 to 2019
Cause	Location	Mean (95% UI)	Mean (95% UI)	Mean (95% UI)	Mean (95% UI)
	Parkinson's disease	710 (557–898)	94·9% (46·1 to 165·3%)	5·2 (4·1–6·5)	0·1% (-24·7 to 36·8%)
	Idiopathic epilepsy	592 (383–816)	-13·4% (-49·1 to 109·2%)	1·6 (1–2·1)	-45·5% (-65·4 to 4·4%)
	Multiple sclerosis	56 (25–89)	166·2% (46·7 to 377·5%)	0·2 (0·1–0·3)	19·7% (-31·4 to 105·2%)
	Motor neuron disease	12 (8–17)	147·7% (49·3 to 304·6%)	0·1 (0–0·1)	21·6% (-27·9 to 102·9%)
	Other neurological disorders	171 (111–248)	145·6% (34·7 to 301·2%)	0·5 (0·4–0·8)	57·6% (-3·2 to 131·8%)
**Incidence**					
Global	Meningitis	2507224 (2113069–2988698)	-23·8% (-26·5 to -20·4%)	35·4 (29·6–42·5)	-35·9% (-37 to -34·6%)
	Encephalitis	1444722 (1280147–1614941)	12·5% (8·1 to 17·1%)	19·3 (17·1–21·7)	-16·5% (-17·8 to -15·2%)
	Tetanus	73662 (53347–101149)	-88% (-91·1 to -83·2%)	1 (0·7–1·4)	-90% (-92·4 to -86·1%)
	Brain and central nervous system cancer	347992 (262084–388896)	94·4% (22 to 128·3%)	4·3 (3·3–4·9)	13·8% (-27·3 to 32·8%)
	Stroke*	12224551 (11041816–13589312)	70·1% (66·6 to 73·4%)	150·8 (136·5–167·5)	-16·9% (-18·4 to -15·3%)
	Ischaemic stroke	7630803 (6569220–8960358)	87·6% (83·5 to 91·7%)	94·5 (81·9–110·8)	-10% (-12 to -8%)
	Intracerebral haemorrhage	3409122 (2970474–3909194)	43·2% (40·7 to 45·5%)	41·8 (36·5–47·9)	-29·2% (-30·5 to -27·8%)
	Subarachnoid haemorrhage	1184625 (1005873–1390420)	60·7% (56·5 to 64·7%)	14·5 (12·3–16·9)	-16·9% (-18·8 to -15·3%)
	Neurological disorders†	805178458 (725838131–888847392)	49·1% (46·1 to 52·4%)	10259·5 (9223·2–11324·2)	-0·1% (-0·6 to 0·5%)
	Alzheimer's disease and other dementias	7236385 (6217239–8232672)	147·7% (142 to 153·6%)	95 (81·6–107·9)	1·5% (0·2 to 2·8%)
	Parkinson's disease	1081723 (953265–1211202)	159·7% (153 to 167%)	13·4 (11·8–15)	19·6% (17 to 22·3%)
	Idiopathic epilepsy	2898222 (2098718–3823376)	55·9% (32·4 to 83·7%)	38·8 (28–51·3)	16·9% (0·1 to 36·5%)
	Multiple sclerosis	59345 (51818–66943)	41·8% (37·7 to 45·7%)	0·7 (0·6–0·8)	-7·3% (-8·7 to -5·8%)
	Migraine	87648969 (76635688–98654602)	40% (36·8 to 43·5%)	1142·5 (995·9–1289·4)	2·1% (1·1 to 2·8%)
	Tension-type headache	706190114 (626723555–788575302)	49·6% (46·2 to 53·3%)	8968·2 (7931·9–9990·5)	-0·4% (-1 to 0·2%)
	Motor neuron disease	63700 (57296–71343)	79% (74·1 to 84%)	0·8 (0·7–0·9)	0·3% (-0·4 to 0·9%)
	Other neurological disorders	0 (0–0)	0% (0 to 0%)	0 (0–0)	0% (0 to 0%)
	Headache disorders	793839083 (714299832–877018808)	48·5% (45·4 to 51·7%)	10110·7 (9070·7–11167·1)	-0·2% (-0·7 to 0·4%)
North Africa and Middle East	Meningitis	128547 (106665–152723)	-9·9% (-16·3 to -1·9%)	22·5 (18·9–26·4)	-37% (-39·9 to -33·9%)
	Encephalitis	58113 (49849–67005)	46·2% (40·2 to 53·3%)	9·9 (8·5–11·3)	-4·4% (-5·8 to -3%)
	Tetanus	1860 (1177–2923)	-83% (-90·6 to -68%)	0·3 (0·2–0·5)	-88·4% (-92·9 to -79%)
	Brain and central nervous system cancer	27529 (18554–32579)	152·5% (49·6 to 233·1%)	5·2 (3·5–6·1)	28% (-19·6 to 63·3%)
	Stroke*	829803 (758352–912812)	130·7% (124·4 to 137·7%)	183 (166·7–201·7)	-5·4% (-7·4 to -3·3%)
	Ischaemic stroke	602510 (531317–682539)	166·9% (157·2 to 177·2%)	135·5 (119·7–153·6)	8·8% (6·3 to 11·4%)
	Intracerebral haemorrhage	163240 (149166–179814)	65·1% (60·7 to 69·7%)	35 (31·8–38·6)	-32·6% (-34·5 to -30·6%)
	Subarachnoid haemorrhage	64053 (54559–75578)	82·9% (75·3 to 92·5%)	12·5 (10·7–14·7)	-26·4% (-29·2 to -23·3%)
	Neurological disorders†	61542741 (54639556–68418425)	89·1% (82·4 to 95·8%)	10090·8 (9024·1–11159·3)	0·8% (0 to 1·6%)
	Alzheimer's disease and other dementias	361192 (309692–413138)	177·5% (171·2 to 184·3%)	110·2 (93·9–125·6)	0·8% (-0·7 to 2·6%)
	Parkinson's disease	42804 (38332–47338)	192·5% (184·3 to 201·9%)	11·4 (10·3–12·5)	12·7% (9·9 to 15·7%)
	Idiopathic epilepsy	295453 (195044–407682)	67·4% (20·3 to 135·5%)	48·2 (32·2–66·4)	8·3% (-21·5 to 51·4%)

Table S12 Burden, mortality, incidence, and prevalence of neurological conditions in North Africa and Middle East countries

		All Ages		Age-standardised	
		Number	Percent change	Rate per 100,000	Percent change
**Measure**		2019	From 1990 to 2019	2019	From 1990 to 2019
Cause	Location	Mean (95% UI)	Mean (95% UI)	Mean (95% UI)	Mean (95% UI)
	Multiple sclerosis	9218 (7879–10525)	120% (110 to 129·4%)	1·4 (1·2–1·6)	5·5% (4 to 6·8%)
	Migraine	7950885 (6837588–9083619)	70·6% (62·7 to 79·1%)	1238·9 (1063·5–1415·6)	0·4% (-0·7 to 1·6%)
	Tension-type headache	52879780 (46137009–59485212)	91·9% (84 to 99·8%)	8680·1 (7631·6–9732·5)	0·9% (-0·1 to 1·7%)
	Motor neuron disease	3409 (2881–4056)	83·1% (70·8 to 94·9%)	0·6 (0·5–0·7)	1·9% (0·3 to 3·6%)
	Other neurological disorders	0 (0–0)	0% (0 to 0%)	0 (0–0)	0% (0 to 0%)
	Headache disorders	60830665 (53891298–67676102)	88·8% (82 to 95·6%)	9919 (8853·1–10989·5)	0·8% (-0·1 to 1·5%)
Algeria	Meningitis	7858 (6442–9451)	-5·1% (-13·2 to 4·1%)	19·8 (16·4–23·5)	-33·8% (-38·6 to -28·8%)
	Encephalitis	3407 (2900–3956)	39·9% (30·1 to 49·8%)	8·4 (7·2–9·7)	-2·4% (-6·5 to 1·9%)
	Tetanus	16 (11–32)	-75·3% (-90·4 to -33·4%)	0 (0–0·1)	-83·1% (-93 to -57·7%)
	Brain and central nervous system cancer	906 (567–1162)	136·7% (28·9 to 225·3%)	2·3 (1·5–2·9)	28% (-30·1 to 75·1%)
	Stroke*	61052 (55145–67937)	118·1% (106·4 to 132%)	181·7 (164·3–202·2)	-16·9% (-21·1 to -12·3%)
	Ischaemic stroke	46561 (40719–53253)	161·6% (142·6 to 182·8%)	140·1 (122·9–160·1)	-1·4% (-7·3 to 5·6%)
	Intracerebral haemorrhage	9769 (8701–10901)	30·1% (22 to 38%)	29·1 (25·8–32·5)	-49·8% (-53·1 to -46·8%)
	Subarachnoid haemorrhage	4722 (3970–5563)	76·1% (61 to 91·9%)	12·5 (10·6–14·6)	-33% (-38·1 to -27·6%)
	Neurological disorders†	4123947 (3650397–4589463)	72·9% (64·3 to 81·9%)	9906·8 (8817·7–10981·7)	-0·1% (-0·6 to 0·4%)
	Alzheimer's disease and other dementias	28168 (23651–32803)	222·4% (198·4 to 248%)	110 (93·8–126)	-0·1% (-3·1 to 3·1%)
	Parkinson's disease	3215 (2838–3684)	200·8% (176·4 to 228·3%)	11 (9·8–12·3)	6·6% (-0·9 to 15·4%)
	Idiopathic epilepsy	20216 (5528–34174)	44·1% (-64·1 to 502·9%)	48·2 (13·1–81·3)	-0·3% (-74·5 to 315·8%)
	Multiple sclerosis	684 (567–810)	138·2% (115·5 to 160%)	1·5 (1·3–1·8)	21·3% (15·6 to 28%)
	Migraine	526992 (447986–606761)	50·7% (41·4 to 60·7%)	1234·6 (1047·1–1422·7)	0·1% (0 to 0·1%)
	Tension-type headache	3544462 (3089897–4021845)	76·2% (66·4 to 87%)	8501 (7440·7–9578·6)	-0·1% (-0·1 to -0·1%)
	Motor neuron disease	211 (174–254)	85·6% (68·4 to 104·6%)	0·5 (0·4–0·6)	0·8% (-2·8 to 4·5%)
	Other neurological disorders	0 (0–0)	0% (0 to 0%)	0 (0–0)	0% (0 to 0%)
	Headache disorders	4071454 (3596687–4535972)	72·4% (63·8 to 81·7%)	9735·6 (8650–10821·3)	-0·1% (-0·1 to 0%)
Bahrain	Meningitis	208 (165–256)	27·6% (9·2 to 50·4%)	19·5 (15·7–23·5)	-46% (-51·2 to -40·6%)
	Encephalitis	93 (79–107)	123·8% (102·1 to 145·6%)	8·2 (7–9·6)	0·5% (-3·8 to 4·8%)
	Tetanus	0 (0–0)	71% (-23·8 to 170%)	0 (0–0)	-39·3% (-73·2 to -5·8%)
	Brain and central nervous system cancer	47 (28–61)	358% (177·2 to 552·5%)	3·6 (2·4–4·6)	9·6% (-34·1 to 55·8%)
	Stroke*	1236 (1090–1394)	240·4% (215·8 to 266·4%)	113·4 (102·3–126·6)	-32·8% (-36·8 to -28·7%)
	Ischaemic stroke	854 (724–1019)	261·5% (226·2 to 300·9%)	82 (71·4–94·5)	-30·1% (-35·1 to -24·2%)
	Intracerebral haemorrhage	230 (202–261)	145·9% (125·7 to 167·4%)	21·8 (19·3–24·6)	-46·4% (-50·1 to -43%)
	Subarachnoid haemorrhage	153 (121–196)	354·7% (294·1 to 430·2%)	9·6 (7·9–11·9)	-11·1% (-19·6 to 0·8%)
	Neurological disorders†	148982 (131155–167732)	201·1% (179·3 to 223·1%)	9902·6 (8826·1–10988·3)	-0·1% (-0·7 to 0·5%)
	Alzheimer's disease and other dementias	573 (492–659)	450·2% (419·6 to 484%)	111·9 (96·1–128·1)	1·4% (-1·4 to 4·7%)
	Parkinson's disease	94 (78–110)	466·8% (391·3 to 546%)	13·7 (12–15·5)	12·8% (1·4 to 26%)
	Idiopathic epilepsy	758 (221–1274)	134% (-28 to 809·6%)	61·2 (18·2–101·5)	-5·2% (-70·4 to 270·5%)
	Multiple sclerosis	21 (17–26)	271% (222·2 to 329%)	1·2 (1–1·5)	27·9% (21 to 34·9%)
	Migraine	16696 (14309–19280)	151·6% (132·1 to 176·2%)	1189·7 (1004·9–1375·8)	0·4% (0 to 0·8%)
	Tension-type headache	130833 (112605–149916)	208·6% (184·1 to 235·6%)	8524·4 (7476–9592·8)	-0·2% (-0·3 to 0%)
	Motor neuron disease	7 (5–9)	254·2% (199·1 to 312·3%)	0·5 (0·4–0·6)	4·4% (0·6 to 8·3%)

Table S12 Burden, mortality, incidence, and prevalence of neurological conditions in North Africa and Middle East countries

Measure		All Ages Number 2019	Percent change From 1990 to 2019	Age-standardised Rate per 100,000 2019	Percent change From 1990 to 2019
Cause	Location	Mean (95% UI)	Mean (95% UI)	Mean (95% UI)	Mean (95% UI)
	Other neurological disorders	0 (0–0)	0% (0 to 0%)	0 (0–0)	0% (0 to 0%)
	Headache disorders	147529 (129607–166427)	200·9% (179·2 to 223·7%)	9714·1 (8647·6–10789·3)	-0·1% (-0·2 to 0%)
Egypt	Meningitis	21599 (17577–26759)	-6% (-16·6 to 5·7%)	21·9 (18·1–26·7)	-33·2% (-38·9 to -26·9%)
	Encephalitis	10024 (8491–11839)	39·1% (31·4 to 47·1%)	10 (8·6–11·6)	-10·6% (-15·2 to -5·9%)
	Tetanus	213 (72–628)	-82·7% (-94·1 to -48·7%)	0·3 (0·1–0·7)	-88·5% (-95·1 to -67·3%)
	Brain and central nervous system cancer	2910 (2013–4118)	128·8% (34·3 to 228%)	3·5 (2·4–5·1)	20·9% (-22·4 to 69·8%)
	Stroke*	152702 (136448–170214)	138·9% (124·4 to 155·3%)	228·4 (205–255·3)	9·6% (3·5 to 16·5%)
	Ischaemic stroke	116369 (100531–133786)	200·2% (176·1 to 227·7%)	176·6 (153·4–202·3)	35·8% (25·7 to 46%)
	Intracerebral haemorrhage	27106 (24428–30214)	43·3% (34·7 to 52·5%)	39·6 (35·9–44·1)	-34·6% (-38·2 to -30·4%)
	Subarachnoid haemorrhage	9227 (7905–10872)	47·8% (37·6 to 59·3%)	12·2 (10·5–14·3)	-31·6% (-36·3 to -26·4%)
	Neurological disorders†	10069794 (8899171–11220191)	88·4% (81·1 to 96·2%)	10257·3 (9189·4–11341·2)	1·1% (-2·1 to 4·8%)
	Alzheimer's disease and other dementias	44042 (37497–50789)	117·7% (109·8 to 124·7%)	107·3 (91·6–122·8)	2·6% (-1·1 to 5·7%)
	Parkinson's disease	6280 (5471–7205)	142·4% (121·3 to 162·3%)	12 (10·5–13·6)	12·1% (3·7 to 20%)
	Idiopathic epilepsy	40757 (10706–70842)	59·9% (-54·8 to 647·6%)	39·3 (10·5–68·1)	-0·4% (-72 to 360·2%)
	Multiple sclerosis	818 (658–986)	132·1% (123·1 to 142·2%)	0·8 (0·7–1)	17·6% (13·8 to 21·8%)
	Migraine	1383829 (1182631–1579436)	85·2% (73·6 to 98·1%)	1285·1 (1107·4–1462·9)	3·3% (-2·1 to 9·3%)
	Tension-type headache	8593585 (7460381–9681319)	88·9% (80·8 to 98%)	8812·2 (7762·7–9893·9)	0·7% (-2·7 to 5%)
	Motor neuron disease	482 (400–580)	72·4% (60·2 to 83·7%)	0·5 (0·5–0·7)	-1·8% (-5·2 to 2%)
	Other neurological disorders	0 (0–0)	0% (0 to 0%)	0 (0–0)	0% (0 to 0%)
	Headache disorders	9977414 (8813692–11125950)	88·4% (81·1 to 96·3%)	10097·3 (9024·8–11179·1)	1% (-2·1 to 4·7%)
Iran (Islamic Republic of)	Meningitis	19839 (16076–24050)	-16% (-25·5 to -2·6%)	25·8 (21·1–31)	-23·5% (-29·3 to -17·2%)
	Encephalitis	6769 (5769–7841)	13·4% (5·7 to 22·6%)	8·7 (7·4–10·2)	1·1% (0 to 2·3%)
	Tetanus	27 (19–41)	-90·7% (-96·2 to -72·8%)	0 (0–0·1)	-91·7% (-96·3 to -79·4%)
	Brain and central nervous system cancer	5811 (2942–7046)	130·3% (23·4 to 189·2%)	7·3 (3·7–8·8)	29·6% (-25·3 to 57·5%)
	Stroke*	102778 (90115–117821)	112·9% (103·9 to 123·9%)	138·8 (121·8–159·6)	-16·7% (-18·1 to -15·1%)
	Ischaemic stroke	82457 (69393–97578)	122·7% (111·2 to 136·5%)	112·3 (94·7–133·4)	-14·2% (-16 to -12·5%)
	Intracerebral haemorrhage	12798 (10809–15134)	68·6% (58·3 to 79·7%)	17·3 (14·6–20·6)	-28% (-30·2 to -25·5%)
	Subarachnoid haemorrhage	7523 (6200–9169)	106% (93·3 to 120%)	9·2 (7·6–11·1)	-20·9% (-23·4 to -17·9%)
	Neurological disorders†	9645919 (8632330–10716566)	62·8% (52·2 to 72·8%)	11293·3 (10132·6–12499·6)	4·5% (1·6 to 6·9%)
	Alzheimer's disease and other dementias	67888 (57885–77446)	291·5% (266·1 to 320·5%)	111·7 (95–127·3)	0·4% (-1·3 to 1·9%)
	Parkinson's disease	7444 (6276–8663)	256·7% (226·9 to 285·1%)	11·2 (9·4–13·2)	13·7% (11·1 to 16·4%)
	Idiopathic epilepsy	44729 (30094–62083)	26·5% (-6·9 to 71·9%)	55·6 (37·2–77·3)	8·3% (-18·6 to 44%)
	Multiple sclerosis	1913 (1643–2191)	78·9% (60·5 to 97·2%)	2 (1·7–2·2)	-5·2% (-8·4 to -2%)
	Migraine	1093330 (967519–1223899)	31·4% (21·9 to 41·7%)	1274·7 (1119·7–1427)	-0·7% (-3·4 to 2·3%)
	Tension-type headache	8430126 (7411247–9501060)	67·4% (55·2 to 78·9%)	9837·5 (8687·5–11030·7)	5·3% (1·9 to 7·9%)
	Motor neuron disease	490 (409–596)	69·8% (50·1 to 87·8%)	0·6 (0·5–0·7)	4·1% (2·2 to 6·2%)
	Other neurological disorders	0 (0–0)	0% (0 to 0%)	0 (0–0)	0% (0 to 0%)
	Headache disorders	9523455 (8507965–10591783)	62·3% (51·6 to 72·1%)	11112·1 (9952·4–12306·5)	4·6% (1·6 to 6·9%)

Table S12 Burden, mortality, incidence, and prevalence of neurological conditions in North Africa and Middle East countries

		All Ages		Age-standardised	
		Number	Percent change	Rate per 100,000	Percent change
**Measure**		2019	From 1990 to 2019	2019	From 1990 to 2019
Cause	Location	Mean (95% UI)	Mean (95% UI)	Mean (95% UI)	Mean (95% UI)
Iraq	Meningitis	9393 (7789–11141)	5·8% (-5·5 to 17·5%)	22·9 (19·3–26·8)	-40·2% (-44·8 to -36·1%)
	Encephalitis	4582 (3921–5321)	76·6% (66·4 to 88·5%)	11·1 (9·6–12·7)	-12·1% (-15·9 to -8%)
	Tetanus	38 (23–65)	-89·1% (-96·5 to -53·5%)	0·1 (0·1–0·2)	-92·2% (-97·2 to -72·2%)
	Brain and central nervous system cancer	2797 (2019–3628)	264% (85·4 to 481·7%)	8·5 (6·3–10·9)	45·7% (-22 to 125%)
	Stroke*	60403 (54854–66101)	153·6% (141·4 to 166·3%)	241·6 (216·9–267)	-11·7% (-16·4 to -6·7%)
	Ischaemic stroke	42447 (36923–48014)	168·6% (150·2 to 187·7%)	178·1 (154–203·1)	-5% (-11·7 to 2·3%)
	Intracerebral haemorrhage	14714 (13481–16029)	132·8% (122·2 to 146·8%)	52·7 (48·2–57·9)	-22·7% (-26·6 to -18·3%)
	Subarachnoid haemorrhage	3242 (2740–3831)	90·7% (72·6 to 109·4%)	10·8 (9·1–12·8)	-40·3% (-45·7 to -34·7%)
	Neurological disorders†	4137753 (3642822–4636981)	159·3% (151·1 to 167·8%)	9902·6 (8818·8–10985·9)	0% (-0·5 to 0·5%)
	Alzheimer's disease and other dementias	17479 (14946–20004)	165·3% (156·4 to 175·8%)	108·8 (93·1–124·3)	2·2% (-0·6 to 5·3%)
	Parkinson's disease	1951 (1712–2207)	183·6% (163·9 to 208·4%)	10 (8·8–11·3)	1·6% (-5·3 to 9·9%)
	Idiopathic epilepsy	20248 (4904–36053)	116·9% (-40·6 to 800·7%)	45·3 (11–79·1)	1·3% (-72·3 to 319·5%)
	Multiple sclerosis	607 (481–749)	238·3% (218·6 to 256·6%)	1·3 (1·1–1·6)	13·8% (9·2 to 18·5%)
	Migraine	569281 (479195–662177)	140% (127·9 to 151·9%)	1229·4 (1042·5–1418·4)	-0·1% (-0·2 to -0·1%)
	Tension-type headache	3527971 (3048007–4011221)	162·9% (153·4 to 172·6%)	8507·1 (7448·5–9584·3)	0% (0 to 0%)
	Motor neuron disease	215 (178–260)	132·4% (110·9 to 154%)	0·6 (0·5–0·7)	4·1% (0·7 to 7·7%)
	Other neurological disorders	0 (0–0)	0% (0 to 0%)	0 (0–0)	0% (0 to 0%)
	Headache disorders	4097252 (3604695–4592180)	159·5% (151·2 to 168·2%)	9736·6 (8654·2–10821·6)	0% (0 to 0%)
Jordan	Meningitis	3034 (2459–3737)	128·8% (107·8 to 154·7%)	27·4 (22·5–33·1)	-10·8% (-16·6 to -3·7%)
	Encephalitis	939 (785–1120)	145·3% (130·3 to 162·4%)	8·2 (7–9·6)	-5·4% (-9·3 to -0·7%)
	Tetanus	3 (2–4)	-58·9% (-81·7 to 18·5%)	0 (0–0)	-80·1% (-90·8 to -53·3%)
	Brain and central nervous system cancer	409 (295–508)	377·4% (199·9 to 546·7%)	4·4 (3·1–5·4)	26% (-21·9 to 71·5%)
	Stroke*	15457 (13809–17262)	238% (216·8 to 261·6%)	225·9 (200·5–253·4)	-26·2% (-30·9 to -20·8%)
	Ischaemic stroke	12026 (10423–13812)	245·7% (218·2 to 275·8%)	181·4 (156·6–208·7)	-24·9% (-30·9 to -18·1%)
	Intracerebral haemorrhage	2162 (1916–2472)	166·1% (147·1 to 186%)	30·2 (26·9–34)	-39·6% (-43·5 to -35%)
	Subarachnoid haemorrhage	1270 (1034–1570)	350·1% (300·9 to 401·4%)	14·3 (11·7–17·6)	-2·3% (-12·5 to 7·5%)
	Neurological disorders†	1148696 (1012559–1286134)	230% (218·2 to 242·7%)	9888·5 (8805·2–10979·7)	0% (-0·4 to 0·5%)
	Alzheimer's disease and other dementias	4712 (4020–5451)	425·7% (403·9 to 446·7%)	107·9 (91·9–123·3)	1·8% (-1·3 to 4·8%)
	Parkinson's disease	563 (491–642)	408% (353·5 to 457·6%)	10·3 (9·1–11·6)	-3% (-12·7 to 6%)
	Idiopathic epilepsy	5674 (1526–9673)	183·9% (-25·7 to 975·2%)	46·5 (12·5–79)	4·7% (-72·3 to 291·9%)
	Multiple sclerosis	202 (163–241)	245·5% (204·9 to 283·8%)	1·6 (1·3–1·9)	-2·4% (-12·7 to 7·8%)
	Migraine	155009 (130146–180094)	199·1% (186·6 to 212·3%)	1213·3 (1027·6–1402·1)	-0·6% (-0·8 to -0·5%)
	Tension-type headache	982482 (849563–1113702)	235·1% (221 to 250·2%)	8508·4 (7455–9588·3)	0·1% (0 to 0·2%)
	Motor neuron disease	55 (46–66)	221% (185 to 257·9%)	0·5 (0·5–0·6)	2·4% (-1·9 to 6·6%)
	Other neurological disorders	0 (0–0)	0% (0 to 0%)	0 (0–0)	0% (0 to 0%)
	Headache disorders	1137490 (1001771–1274002)	229·7% (217·7 to 242·6%)	9721·7 (8637·2–10804·7)	0% (-0·1 to 0·1%)
Kuwait	Meningitis	669 (534–826)	64·1% (48·1 to 82·4%)	19·2 (15·6–23·2)	-20·5% (-24·8 to -15·4%)
	Encephalitis	307 (259–359)	114·9% (98·8 to 132·8%)	8·3 (7·1–9·8)	0·6% (-4·3 to 5%)
	Tetanus	0 (0–0)	92% (-77·1 to 609·3%)	0 (0–0)	-60·9% (-93·8 to -5·7%)
	Brain and central nervous system cancer	144 (101–181)	300·1% (144·2 to 423·9%)	4 (2·8–5)	44·3% (-12·5 to 86·6%)

Table S12 Burden, mortality, incidence, and prevalence of neurological conditions in North Africa and Middle East countries

		All Ages		Age-standardised	
		Number	Percent change	Rate per 100,000	Percent change
**Measure**		2019	From 1990 to 2019	2019	From 1990 to 2019
Cause	Location	Mean (95% UI)	Mean (95% UI)	Mean (95% UI)	Mean (95% UI)
	Stroke*	4005 (3598–4495)	270·9% (249·2 to 294·7%)	125·8 (113·7–140·4)	-2·7% (-8·2 to 3·3%)
	Ischaemic stroke	2745 (2374–3199)	271·6% (243 to 304·6%)	91·5 (79·6–105·3)	-4% (-10·7 to 4·2%)
	Intracerebral haemorrhage	704 (626–803)	256·5% (228·2 to 284·7%)	21·4 (19·2–24·2)	-0·5% (-7·4 to 7%)
	Subarachnoid haemorrhage	556 (438–711)	287·1% (244·6 to 338·5%)	12·9 (10·5–16·2)	3·1% (-6·4 to 15·2%)
	Neurological disorders†	451938 (399757–508423)	162·4% (148·6 to 176·8%)	9888·6 (8803·3–10919·9)	-0·1% (-2·6 to 2·5%)
	Alzheimer's disease and other dementias	2054 (1797–2316)	391·7% (373·8 to 411·3%)	111·2 (95·4–127·1)	0·7% (-2·3 to 4%)
	Parkinson's disease	190 (165–222)	288·1% (244·9 to 338·3%)	8·4 (7·4–9·6)	-11·5% (-23·7 to 2·8%)
	Idiopathic epilepsy	2250 (614–3660)	115·4% (-38·4 to 703·8%)	56 (15·2–90·4)	-0·9% (-72 to 282·6%)
	Multiple sclerosis	101 (82–120)	302·1% (270·4 to 337·5%)	1·8 (1·5–2·1)	49·9% (41·5 to 58·8%)
	Migraine	56016 (47909–65273)	140·8% (123·9 to 159·7%)	1223·6 (1039·8–1415·4)	2·8% (-0·5 to 6·6%)
	Tension-type headache	391309 (340667–446614)	165·4% (150 to 182·2%)	8487·2 (7489·2–9540·2)	-0·6% (-3·3 to 2·4%)
	Motor neuron disease	19 (15–24)	142·6% (113·4 to 171·4%)	0·5 (0·4–0·6)	-12% (-16·5 to -7·4%)
	Other neurological disorders	0 (0–0)	0% (0 to 0%)	0 (0–0)	0% (0 to 0%)
	Headache disorders	447324 (393997–503177)	162% (148·3 to 176%)	9710·8 (8639·4–10763·2)	-0·1% (-2·6 to 2·5%)
Lebanon	Meningitis	1024 (846–1215)	-2% (-12·5 to 9·9%)	20 (16·5–23·7)	-27·8% (-33 to -22·6%)
	Encephalitis	635 (556–721)	31·4% (24·7 to 39·7%)	12·4 (10·9–14·1)	-12·5% (-15·7 to -8·5%)
	Tetanus	37 (8–145)	-51% (-90·3 to 59·8%)	0·7 (0·2–2·9)	-78·6% (-95·3 to -30·4%)
	Brain and central nervous system cancer	350 (252–447)	195% (101·3 to 302·7%)	6·7 (4·8–8·6)	56·4% (6·3 to 111·2%)
	Stroke*	7800 (7030–8706)	112% (99·5 to 126·2%)	150·5 (135·7–167·8)	-5·3% (-9·9 to 0·3%)
	Ischaemic stroke	6161 (5384–7048)	143·8% (126·1 to 164·3%)	119 (104·1–135·6)	7·9% (1·1 to 15·5%)
	Intracerebral haemorrhage	979 (866–1103)	24·9% (16·6 to 33·8%)	19 (16·9–21·4)	-43·7% (-47·2 to -40·1%)
	Subarachnoid haemorrhage	660 (539–834)	79·6% (58·5 to 103%)	12·5 (10·2–15·7)	-15·3% (-24·8 to -4·5%)
	Neurological disorders†	514250 (459288–572600)	68·3% (61·8 to 74·7%)	9912·9 (8824·5–10995·6)	0·2% (-0·4 to 0·7%)
	Alzheimer's disease and other dementias	5623 (4787–6485)	207·7% (191·4 to 222·2%)	110·6 (95·2–126·5)	1·6% (-1·8 to 4·7%)
	Parkinson's disease	536 (481–603)	189·9% (158·6 to 222·5%)	10·2 (9·2–11·4)	8·8% (-1 to 19·2%)
	Idiopathic epilepsy	2594 (728–4401)	50% (-61·3 to 581·9%)	50·9 (14·3–85·5)	7·2% (-72·2 to 387·8%)
	Multiple sclerosis	102 (84–121)	135·2% (114·3 to 158%)	1·8 (1·5–2·2)	26·5% (20·2 to 34·6%)
	Migraine	63410 (53956–72932)	51·9% (44·8 to 59·7%)	1235·1 (1046·9–1423·5)	-0·4% (-0·5 to -0·2%)
	Tension-type headache	441957 (387091–499441)	69·9% (62·6 to 77%)	8503·8 (7444·7–9581·2)	0·2% (0·1 to 0·3%)
	Motor neuron disease	28 (23–33)	63·7% (51·1 to 75·5%)	0·5 (0·4–0·6)	-0·6% (-3·8 to 2·8%)
	Other neurological disorders	0 (0–0)	0% (0 to 0%)	0 (0–0)	0% (0 to 0%)
	Headache disorders	505367 (450608–563886)	67·4% (61·1 to 73·5%)	9738·8 (8650·3–10829·1)	0·1% (0 to 0·2%)
Libya	Meningitis	965 (783–1160)	-27·8% (-37·6 to -15·7%)	17·7 (14·6–21·4)	-34·7% (-40·1 to -28·9%)
	Encephalitis	360 (306–417)	13·8% (3·2 to 27%)	6·3 (5·4–7·4)	-1·2% (-5·3 to 2·9%)
	Tetanus	2 (1–5)	-54·9% (-82·9 to 11·7%)	0 (0–0·1)	-67·1% (-88·8 to -23·8%)
	Brain and central nervous system cancer	319 (237–423)	130·5% (60·2 to 239·2%)	5·2 (3·9–6·8)	15·6% (-17·6 to 64·1%)
	Stroke*	9358 (8475–10432)	180·8% (162·4 to 200·3%)	170·9 (153·6–190·3)	7·9% (2·1 to 14·6%)
	Ischaemic stroke	7177 (6299–8209)	230·5% (201·8 to 261·7%)	133·9 (117–152·9)	25·3% (16·7 to 34·8%)
	Intracerebral haemorrhage	1434 (1285–1592)	74·6% (64·1 to 86%)	25·3 (22·6–28·3)	-31·6% (-35·3 to -27·9%)
	Subarachnoid haemorrhage	747 (617–917)	119·8% (99·5 to 144·3%)	11·7 (9·8–14)	-19·8% (-26·1 to -12·7%)
	Neurological disorders†	694187 (614912–774991)	77·3% (66·9 to 88·3%)	9902·4 (8808·5–10993·8)	0% (-0·4 to 0·5%)
	Alzheimer's disease and other dementias	4408 (3768–5005)	168% (159·4 to 176·1%)	107·4 (90·9–122·9)	-1·9% (-5 to 0·9%)
	Parkinson's disease	515 (457–572)	208·6% (188·1 to 233·7%)	11·3 (9·9–12·5)	11·7% (3·9 to 20·6%)

Table S12 Burden, mortality, incidence, and prevalence of neurological conditions in North Africa and Middle East countries

Measure		All Ages		Age-standardised	
		Number	Percent change	Rate per 100,000	Percent change
		2019	From 1990 to 2019	2019	From 1990 to 2019
Cause	Location	Mean (95% UI)	Mean (95% UI)	Mean (95% UI)	Mean (95% UI)
	Idiopathic epilepsy	2904 (941–4821)	19·4% (-63·1 to 350·8%)	45·6 (14·8–75)	-8·1% (-69·9 to 242·5%)
	Multiple sclerosis	119 (101–140)	169·2% (143·6 to 194·3%)	1·5 (1·3–1·7)	25·9% (19·4 to 30·9%)
	Migraine	87010 (74572–100028)	51·3% (37·8 to 65·4%)	1227·8 (1041·4–1416·6)	1·1% (0·8 to 1·4%)
	Tension-type headache	599202 (520188–678679)	81·7% (69·1 to 94·3%)	8508·4 (7450·6–9585·8)	-0·1% (-0·2 to 0·1%)
	Motor neuron disease	29 (24–35)	65·1% (41·3 to 88%)	0·5 (0·4–0·6)	1·4% (-2·7 to 5·5%)
	Other neurological disorders	0 (0–0)	0% (0 to 0%)	0 (0–0)	0% (0 to 0%)
	Headache disorders	686212 (606467–767209)	77·2% (66·6 to 88·4%)	9736·2 (8655·1–10820·8)	0·1% (-0·1 to 0·2%)
Morocco	Meningitis	7009 (5794–8281)	-41·1% (-46·9 to -35·1%)	22·1 (18·5–26·2)	-45·9% (-49·8 to -41·6%)
	Encephalitis	2914 (2471–3374)	18·3% (11·8 to 26%)	8·6 (7·3–10)	-2·8% (-6·8 to 1·4%)
	Tetanus	169 (44–567)	-93·2% (-98·3 to -75·6%)	0·6 (0·1–1·9)	-92·1% (-97·9 to -74%)
	Brain and central nervous system cancer	639 (446–862)	101·7% (22·8 to 189·8%)	1·8 (1·3–2·4)	19·5% (-22·3 to 67·3%)
	Stroke*	63692 (57107–70794)	106·6% (93·9 to 120·9%)	199·8 (179·8–222)	-4·4% (-9·8 to 1·5%)
	Ischaemic stroke	48382 (42016–55500)	152·2% (130·5 to 175·1%)	152·9 (132·9–174·8)	14·9% (5·8 to 23·9%)
	Intracerebral haemorrhage	10826 (9693–12112)	27·4% (20·2 to 35·1%)	33·7 (30·3–37·7)	-40% (-43·3 to -36·3%)
	Subarachnoid haemorrhage	4485 (3794–5253)	42·4% (32·8 to 53·1%)	13·3 (11·3–15·4)	-32·8% (-37·3 to -28·1%)
	Neurological disorders†	3608793 (3200563–4010183)	50% (44·6 to 55·5%)	9904·2 (8826·3–10974·4)	0% (-0·5 to 0·5%)
	Alzheimer's disease and other dementias	25591 (21785–29492)	140·2% (131 to 149·4%)	109·1 (92·7–124·8)	-1% (-4·1 to 2·3%)
	Parkinson's disease	2733 (2425–3050)	176·5% (152·1 to 204·6%)	9·8 (8·8–10·9)	21% (10·5 to 32·2%)
	Idiopathic epilepsy	15481 (4080–27531)	39·5% (-66·3 to 514·5%)	44·2 (11·7–78·4)	13·7% (-72·8 to 390·2%)
	Multiple sclerosis	565 (471–673)	87·7% (75·1 to 101·5%)	1·5 (1·2–1·8)	20·3% (14 to 26·9%)
	Migraine	456426 (389533–524531)	32·8% (26·8 to 39%)	1236·4 (1048·9–1424·6)	-0·4% (-0·4 to -0·3%)
	Tension-type headache	3107802 (2711391–3502989)	52·4% (46·3 to 59%)	8502·5 (7441·7–9579·2)	0% (0 to 0%)
	Motor neuron disease	195 (161–239)	49·4% (34·4 to 64·1%)	0·6 (0·5–0·7)	-2·1% (-5·8 to 1·6%)
	Other neurological disorders	0 (0–0)	0% (0 to 0%)	0 (0–0)	0% (0 to 0%)
	Headache disorders	3564228 (3157937–3966864)	49·6% (44·3 to 55·2%)	9739 (8652·6–10825·3)	-0·1% (-0·1 to 0%)
Palestine	Meningitis	912 (725–1116)	-1% (-12·7 to 12·1%)	18·6 (15·5–22·2)	-44·1% (-48·5 to -39%)
	Encephalitis	435 (357–523)	100·1% (87·3 to 113·4%)	8·4 (7·1–9·8)	-0·8% (-4·8 to 3·9%)
	Tetanus	2 (1–2)	-87% (-96·4 to -40%)	0 (0–0·1)	-89·7% (-96·9 to -64·2%)
	Brain and central nervous system cancer	324 (249–395)	175·7% (74 to 272·6%)	9·4 (7·1–11·2)	9·9% (-27·2 to 49%)
	Stroke*	4745 (4261–5288)	143·1% (127·3 to 157·8%)	189·7 (170·1–212·2)	-7·6% (-13·7 to -1·7%)
	Ischaemic stroke	3632 (3175–4171)	164% (142·7 to 185·3%)	149·6 (130–172·4)	2·5% (-5·9 to 11·2%)
	Intracerebral haemorrhage	727 (650–832)	66·5% (55·8 to 78·5%)	28·3 (25·8–31·3)	-38·8% (-42·9 to -34·8%)
	Subarachnoid haemorrhage	386 (316–475)	176·1% (152·5 to 205·6%)	11·8 (9·8–14·6)	-9·4% (-16·3 to -0·8%)
	Neurological disorders†	478406 (419814–539351)	161·1% (155·1 to 167·9%)	9904·4 (8818·6–11008·6)	-0·1% (-0·6 to 0·4%)
	Alzheimer's disease and other dementias	1766 (1512–2023)	136·8% (128·6 to 144·9%)	109·3 (93·5–124·8)	0·6% (-2·4 to 3·8%)
	Parkinson's disease	217 (193–244)	160·2% (139·7 to 183·5%)	11·3 (10·1–12·7)	3·8% (-3·7 to 12·4%)
	Idiopathic epilepsy	2473 (625–4398)	126·4% (-42·8 to 926·7%)	45·6 (12–79·7)	5·7% (-72·6 to 387·6%)
	Multiple sclerosis	74 (60–90)	235·8% (219·6 to 254·9%)	1·5 (1·2–1·7)	16·3% (12·3 to 21·4%)
	Migraine	68357 (56868–80265)	148·6% (140·3 to 156·8%)	1232·3 (1045–1421)	-0·3% (-0·5 to -0·2%)
	Tension-type headache	405495 (349922–462326)	163·7% (156·7 to 171·4%)	8503·8 (7444·3–9580·2)	-0·1% (-0·2 to 0%)
	Motor neuron disease	24 (20–29)	119·4% (98·4 to 141·2%)	0·6 (0·5–0·7)	0·5% (-2·8 to 4·1%)
	Other neurological disorders	0 (0–0)	0% (0 to 0%)	0 (0–0)	0% (0 to 0%)

Table S12 Burden, mortality, incidence, and prevalence of neurological conditions in North Africa and Middle East countries

		**All Ages**		**Age-standardised**	
		Number	Percent change	Rate per 100,000	Percent change
**Measure**		2019	From 1990 to 2019	2019	From 1990 to 2019
Cause	Location	Mean (95% UI)	Mean (95% UI)	Mean (95% UI)	Mean (95% UI)
	Headache disorders	473852 (414848–534653)	161·4% (155·4 to 168%)	9736·1 (8651·9–10822)	-0·1% (-0·2 to 0%)
Oman	Meningitis	274 (216–341)	-3·7% (-13·9 to 9·4%)	8·6 (7·1–10·4)	-40·6% (-45·2 to -36·4%)
	Encephalitis	568 (495–645)	50% (39·8 to 62%)	15·8 (14·1–17·6)	-21·3% (-24·5 to -17·7%)
	Tetanus	1 (1–2)	-75·2% (-91·2 to -7·3%)	0 (0–0·1)	-92·1% (-98·5 to -53·2%)
	Brain and central nervous system cancer	123 (72–161)	343·4% (121 to 582·6%)	3·7 (2·3–4·7)	69·9% (-13·9 to 153·1%)
	Stroke*	3701 (3308–4161)	125·4% (112·1 to 138·6%)	196·2 (174·8–222·1)	-10·3% (-15·9 to -4·6%)
	Ischaemic stroke	2666 (2292–3116)	156·8% (136·8 to 175·9%)	148·9 (128·2–173·3)	3·3% (-4·6 to 11·8%)
	Intracerebral haemorrhage	669 (590–756)	50·9% (40·6 to 63·9%)	35·3 (31·4–39·6)	-39·3% (-43·1 to -35·4%)
	Subarachnoid haemorrhage	366 (301–451)	127·6% (104 to 153·5%)	12 (10·1–14·2)	-26·9% (-32·7 to -21·1%)
	Neurological disorders†	462926 (402131–528279)	157·7% (141 to 173·5%)	9864·6 (8795·8–10966·6)	0% (-0·5 to 0·5%)
	Alzheimer's disease and other dementias	1020 (873–1171)	144·6% (135·1 to 154%)	110·1 (93·9–125·2)	-0·6% (-3·5 to 2·4%)
	Parkinson's disease	213 (185–245)	222·3% (196·6 to 253·5%)	17·6 (15·6–20·1)	33·3% (23·2 to 45·2%)
	Idiopathic epilepsy	2223 (686–3757)	134·3% (-32·3 to 837·3%)	50·3 (15·1–85·3)	19·4% (-65·9 to 372·9%)
	Multiple sclerosis	82 (67–100)	340·4% (304·2 to 374·4%)	1·2 (1–1·4)	28% (21·8 to 34·8%)
	Migraine	55853 (47175–65125)	119·2% (97·7 to 141·3%)	1153·5 (973·2–1340·3)	-1% (-1·4 to -0·7%)
	Tension-type headache	403518 (342668–467602)	164·3% (145·6 to 183·1%)	8531·4 (7483·3–9610·9)	0% (-0·2 to 0·1%)
	Motor neuron disease	18 (14–22)	128% (101·1 to 156·7%)	0·5 (0·4–0·6)	0·6% (-3 to 4·1%)
	Other neurological disorders	0 (0–0)	0% (0 to 0%)	0 (0–0)	0% (0 to 0%)
	Headache disorders	459371 (397916–524267)	157·8% (141·4 to 173·4%)	9684·9 (8625·4–10764)	-0·2% (-0·3 to 0%)
Qatar	Meningitis	1264 (987–1594)	365·1% (306·3 to 439·9%)	56·3 (46·3–68·2)	-17·5% (-22·9 to -12·5%)
	Encephalitis	170 (141–199)	394·9% (348·9 to 438·7%)	8·3 (7–9·6)	-1·8% (-5·9 to 2·7%)
	Tetanus	1 (0–1)	246·7% (18·5 to 553·5%)	0 (0–0)	-47·2% (-84·6 to 40·9%)
	Brain and central nervous system cancer	97 (63–161)	982·3% (534·6 to 1563%)	5·3 (3·8–7·6)	36·2% (-15·7 to 100%)
	Stroke*	1889 (1659–2171)	487·3% (447·3 to 527·8%)	127·1 (114·7–141·6)	-26·1% (-30·3 to -21·7%)
	Ischaemic stroke	1165 (949–1435)	515·8% (455·6 to 575·7%)	87·5 (75·7–101·9)	-24·8% (-30·6 to -18·4%)
	Intracerebral haemorrhage	385 (328–454)	380·1% (337·6 to 438·3%)	24·5 (21·9–27·6)	-34% (-38·3 to -29·3%)
	Subarachnoid haemorrhage	339 (274–412)	548·7% (485 to 610·3%)	15·1 (12·9–17·5)	-18·7% (-25·3 to -13%)
	Neurological disorders†	297563 (254878–343236)	581·2% (557·6 to 604·4%)	9777·4 (8710·9–10885·3)	-0·5% (-1·1 to 0%)
	Alzheimer's disease and other dementias	434 (362–505)	701·7% (645·5 to 764%)	109 (92·9–124·7)	2·5% (-1 to 6·3%)
	Parkinson's disease	110 (92–132)	796·5% (707·7 to 879·6%)	18·4 (16·3–20·7)	26·5% (16·2 to 38·3%)
	Idiopathic epilepsy	1413 (442–2362)	465·9% (62·6 to 2033·6%)	56·4 (16·9–93·7)	2% (-69·9 to 273·5%)
	Multiple sclerosis	85 (73–99)	1046·5% (915·9 to 1227·2%)	1·8 (1·6–2)	44·3% (27·3 to 63·6%)
	Migraine	32281 (27198–37927)	494·1% (456 to 527·1%)	1106·2 (930·6–1292·4)	-2·3% (-3 to -1·6%)
	Tension-type headache	263229 (222394–306753)	594·1% (567·9 to 620·2%)	8485·2 (7444·1–9569·2)	-0·4% (-0·7 to -0·1%)
	Motor neuron disease	11 (8–14)	541% (467·6 to 611·6%)	0·5 (0·4–0·6)	1·3% (-2·6 to 5·5%)
	Other neurological disorders	0 (0–0)	0% (0 to 0%)	0 (0–0)	0% (0 to 0%)
	Headache disorders	295510 (252820–340824)	581·5% (557·2 to 604·5%)	9591·5 (8532·6–10666·3)	-0·6% (-0·9 to -0·3%)
Saudi Arabia	Meningitis	4378 (3416–5388)	12·6% (-0·7 to 29·9%)	17 (13·6–20·6)	-33·5% (-39·9 to -26·1%)
	Encephalitis	2662 (2268–3079)	57% (40·6 to 73·5%)	9·2 (7·9–10·7)	-11% (-15·2 to -6·3%)
	Tetanus	24 (8–42)	-90·3% (-98·7 to -46·9%)	0·1 (0–0·1)	-95% (-99·1 to -69·9%)
	Brain and central nervous system cancer	1296 (944–1962)	625·3% (283·3 to 1290·8%)	4·1 (3·1–6·2)	136% (25·5 to 341·7%)
	Stroke*	41061 (37416–45161)	254·9% (234 to 278·8%)	193·9 (176·5–213·7)	11·9% (6·7 to 18%)
	Ischaemic stroke	28087 (24748–32005)	280·6% (248·3 to 315·6%)	140·4 (123·8–159·3)	20·7% (12·7 to 29·8%)
	Intracerebral haemorrhage	9675 (8851–10619)	204% (184·5 to 224·3%)	42·8 (38·9–47·5)	-6% (-11·1 to -0·8%)

Table S12 Burden, mortality, incidence, and prevalence of neurological conditions in North Africa and Middle East countries

		All Ages		Age-standardised	
		Number	Percent change	Rate per 100,000	Percent change
**Measure**		2019	From 1990 to 2019	2019	From 1990 to 2019
Cause	Location	Mean (95% UI)	Mean (95% UI)	Mean (95% UI)	Mean (95% UI)
	Subarachnoid haemorrhage	3299 (2654–4136)	227·3% (196·2 to 266·1%)	10·7 (8·8–13·3)	-5·9% (-13·1 to 4·2%)
	Neurological disorders†	3567976 (3139311–4012111)	144·8% (128·1 to 162·2%)	9582·1 (8516·5–10684·8)	-0·8% (-3·4 to 2%)
	Alzheimer's disease and other dementias	10230 (8861–11651)	136·6% (124·3 to 151%)	108·1 (92–123·6)	-1·3% (-4·3 to 1·9%)
	Parkinson's disease	1952 (1713–2220)	224·7% (195·2 to 256·1%)	16 (14·4–18·2)	26·7% (16·2 to 38·2%)
	Idiopathic epilepsy	23922 (6464–39404)	153·9% (-31·8 to 928·7%)	71·1 (20·2–117·3)	33% (-62·7 to 418·5%)
	Multiple sclerosis	487 (387–593)	273·2% (242·2 to 306%)	1 (0·8–1·2)	22·9% (17·5 to 28·3%)
	Migraine	449325 (383954–519673)	110·6% (89·9 to 133·2%)	1179·2 (1004·5–1362·2)	-0·6% (-3·9 to 2·4%)
	Tension-type headache	3081911 (2653414–3512212)	150·6% (131·2 to 170·4%)	8206·1 (7168·6–9215·6)	-1% (-4 to 2%)
	Motor neuron disease	149 (119–186)	127·1% (94·5 to 161·5%)	0·5 (0·4–0·6)	3·1% (-1·3 to 7·2%)
	Other neurological disorders	0 (0–0)	0% (0 to 0%)	0 (0–0)	0% (0 to 0%)
	Headache disorders	3531235 (3088625–3972683)	144·7% (127·8 to 162·2%)	9385·4 (8318–10477·2)	-1% (-3·5 to 1·8%)
Syrian Arab Republic	Meningitis	3021 (2512–3583)	-52·1% (-57·6 to -45·4%)	23·7 (19·9–27·8)	-37·1% (-41·5 to -32·2%)
	Encephalitis	1171 (993–1369)	-17·5% (-24·6 to -8·8%)	8·6 (7·3–10·1)	-6·9% (-11·2 to -2·6%)
	Tetanus	8 (5–18)	-96% (-98·8 to -84·9%)	0·1 (0–0·1)	-93% (-97·7 to -76·5%)
	Brain and central nervous system cancer	804 (568–1083)	94·4% (18·5 to 193·9%)	6·1 (4·3–8·1)	19·5% (-19·3 to 76·6%)
	Stroke*	22164 (20152–24408)	61·4% (53·1 to 70·1%)	179·3 (164–196·4)	-18·6% (-22·5 to -14·7%)
	Ischaemic stroke	14837 (12917–16977)	85·1% (70·9 to 99·2%)	120·3 (105·4–136·8)	-9·9% (-16·4 to -3·4%)
	Intracerebral haemorrhage	5745 (5231–6338)	22·3% (15·6 to 30%)	47·4 (43·3–52)	-34% (-37·8 to -30·4%)
	Subarachnoid haemorrhage	1583 (1311–1935)	55·3% (40·2 to 72·7%)	11·6 (9·8–14)	-22·3% (-28·4 to -15·2%)
	Neurological disorders†	1467183 (1302744–1640873)	25·4% (18·6 to 32·1%)	9915 (8823·2–10985·6)	0·2% (-0·2 to 0·7%)
	Alzheimer's disease and other dementias	9408 (8003–10910)	119·3% (108·2 to 130·6%)	109·7 (93·7–125·5)	0·8% (-2·6 to 4·4%)
	Parkinson's disease	1177 (1034–1333)	153·7% (130·8 to 177·6%)	11·6 (10·3–12·9)	9·1% (0·1 to 19·3%)
	Idiopathic epilepsy	5930 (1736–10195)	1·6% (-73·8 to 372·8%)	41·7 (12·2–71·8)	12·6% (-70·5 to 417·6%)
	Multiple sclerosis	210 (172–253)	50·7% (38·9 to 64·2%)	1·5 (1·2–1·8)	20·4% (15·3 to 25·1%)
	Migraine	191070 (160765–221406)	8% (0·1 to 15·9%)	1261·9 (1074·4–1450·5)	2·4% (1·9 to 2·9%)
	Tension-type headache	1259310 (1101071–1426066)	28·2% (20·3 to 35·8%)	8488 (7422·9–9561·3)	-0·1% (-0·3 to 0·1%)
	Motor neuron disease	79 (64–96)	18·3% (1 to 35·5%)	0·6 (0·5–0·7)	0% (-3·6 to 4·2%)
	Other neurological disorders	0 (0–0)	0% (0 to 0%)	0 (0–0)	0% (0 to 0%)
	Headache disorders	1450380 (1285098–1623077)	25·1% (18·2 to 31·8%)	9749·8 (8662–10834·3)	0·2% (0 to 0·4%)
Tunisia	Meningitis	2268 (1852–2674)	-10·2% (-19·6 to 0·7%)	21·8 (17·9–25·8)	-21·8% (-27·9 to -16·3%)
	Encephalitis	902 (772–1037)	15·7% (6·9 to 26%)	8·4 (7·1–9·8)	-1·2% (-5·7 to 3·7%)
	Tetanus	4 (2–7)	-73·4% (-89·8 to -33·3%)	0 (0–0·1)	-78·8% (-92·1 to -47%)
	Brain and central nervous system cancer	231 (156–316)	132·6% (41·8 to 241·1%)	1·9 (1·3–2·6)	39·4% (-12·3 to 103·2%)
	Stroke*	19659 (17619–22111)	136·2% (122·1 to 151·7%)	159·6 (144–178·9)	-1·2% (-6·6 to 4·3%)
	Ischaemic stroke	15194 (13233–17660)	180·6% (159·2 to 204%)	123·4 (108·1–142·9)	15·9% (7·6 to 23·8%)
	Intracerebral haemorrhage	2884 (2533–3290)	47·1% (37·3 to 56·5%)	23·9 (21·1–27·3)	-37·5% (-41·4 to -33·6%)
	Subarachnoid haemorrhage	1581 (1313–1909)	67% (54·7 to 81·4%)	12·3 (10·3–14·7)	-26·8% (-31·7 to -20·9%)
	Neurological disorders†	1171253 (1049587–1299980)	45·3% (38·4 to 52·8%)	9908·4 (8818·4–10995·9)	0·1% (-0·3 to 0·6%)

Table S12 Burden, mortality, incidence, and prevalence of neurological conditions in North Africa and Middle East countries

Measure		All Ages		Age-standardised	
		Number	Percent change	Rate per 100,000	Percent change
		2019	From 1990 to 2019	2019	From 1990 to 2019
Cause	Location	Mean (95% UI)	Mean (95% UI)	Mean (95% UI)	Mean (95% UI)
	Alzheimer's disease and other dementias	12027 (10263–13810)	203·8% (188·7 to 220·8%)	110·7 (94·6–126·2)	0·2% (-2·7 to 3·1%)
	Parkinson's disease	1234 (1096–1388)	195·9% (169·2 to 227·2%)	10·5 (9·4–11·8)	12·5% (3·4 to 24·2%)
	Idiopathic epilepsy	4724 (1367–7778)	35·2% (-66·3 to 480·4%)	43·8 (12·5–72·7)	17·2% (-71·6 to 406·5%)
	Multiple sclerosis	214 (177–252)	85·5% (69·4 to 102·4%)	1·7 (1·4–2·1)	23·2% (17·7 to 28·2%)
	Migraine	139776 (120065–160031)	23·2% (16 to 31%)	1239·1 (1051·9–1427·3)	0·2% (0·1 to 0·3%)
	Tension-type headache	1013213 (890081–1140492)	48% (40 to 56·9%)	8502 (7441·4–9578·4)	0% (0 to 0·1%)
	Motor neuron disease	64 (53–78)	53·6% (37·7 to 69%)	0·5 (0·5–0·7)	-1·8% (-5·2 to 1·9%)
	Other neurological disorders	0 (0–0)	0% (0 to 0%)	0 (0–0)	0% (0 to 0%)
	Headache disorders	1152989 (1029232–1281523)	44·5% (37·3 to 51·9%)	9741 (8652·7–10828·7)	0·1% (0 to 0·1%)
Türkiye	Meningitis	4631 (3846–5447)	-60·2% (-65·5 to -53·4%)	6·7 (5·5–8)	-63·2% (-66·9 to -59·1%)
	Encephalitis	8083 (6976–9240)	11·8% (3·3 to 21·4%)	11·2 (9·6–13)	-2·8% (-7·1 to 2%)
	Tetanus	26 (18–38)	-97·5% (-98·5 to -93·3%)	0 (0–0)	-98·5% (-99·2 to -95·5%)
	Brain and central nervous system cancer	6355 (2999–8787)	120·2% (21·9 to 234·3%)	7·6 (3·7–10·3)	25·9% (-26·3 to 82·2%)
	Stroke*	125345 (114547–138123)	117·4% (106·2 to 130·1%)	145·6 (133·4–160·7)	-2·8% (-7·6 to 1·9%)
	Ischaemic stroke	81599 (71499–93648)	138·2% (120 to 158·2%)	95·4 (83·8–109·5)	4·7% (-3 to 12·8%)
	Intracerebral haemorrhage	30027 (26923–33453)	93·1% (79·1 to 107·3%)	35·1 (31·4–39·1)	-12·4% (-18·3 to -6·4%)
	Subarachnoid haemorrhage	13719 (11850–15875)	74·8% (62·2 to 88·6%)	15·1 (13·1–17·4)	-18·8% (-24·8 to -12·2%)
	Neurological disorders†	8130897 (7269011–9079477)	47·2% (39·6 to 54·4%)	9588·8 (8564·7–10698·3)	0·9% (-1·9 to 3·6%)
	Alzheimer's disease and other dementias	90963 (78271–103566)	187·5% (177·6 to 195·8%)	112·9 (96·7–128·9)	1·5% (-1·8 to 4·5%)
	Parkinson's disease	10209 (8951–11496)	220·8% (198·1 to 247·2%)	12·3 (10·8–13·8)	19·9% (11·6 to 29·7%)
	Idiopathic epilepsy	47294 (13980–76026)	47·5% (-54·5 to 511%)	63·5 (18·8–103·2)	28·1% (-60·8 to 432·1%)
	Multiple sclerosis	1562 (1484–1647)	53·6% (48·8 to 58·7%)	1·7 (1·6–1·8)	-2% (-4·3 to 0·4%)
	Migraine	961458 (826618–1099316)	23·7% (15·5 to 32·7%)	1200·2 (1023·9–1384·3)	0·3% (-3 to 4·4%)
	Tension-type headache	7018674 (6183837–7932907)	50% (41·3 to 59%)	8197·3 (7199·6–9252·4)	0·7% (-2·5 to 3·6%)
	Motor neuron disease	737 (640–840)	63·7% (53·4 to 74·5%)	0·9 (0·8–1)	7·9% (3·8 to 12%)
	Other neurological disorders	0 (0–0)	0% (0 to 0%)	0 (0–0)	0% (0 to 0%)
	Headache disorders	7980132 (7127013–8917328)	46·3% (39 to 53·5%)	9397·5 (8376·7–10486·8)	0·7% (-2·1 to 3·2%)
United Arab Emirates	Meningitis	1235 (997–1517)	126% (94·5 to 165·4%)	28·2 (24·1–33)	-36% (-40·1 to -31·9%)
	Encephalitis	534 (444–621)	241·7% (200·6 to 288·3%)	8·4 (7·1–9·8)	-0·6% (-5·8 to 4·5%)
	Tetanus	49 (19–82)	3·7% (-77·7 to 292%)	2·9 (0·4–5·3)	-85·1% (-95·9 to -34·4%)
	Brain and central nervous system cancer	479 (275–704)	641·7% (380·4 to 976·5%)	5·7 (3·5–7·8)	8·7% (-35·1 to 65·6%)
	Stroke*	14482 (12788–16501)	710·5% (643·9 to 778·5%)	262·2 (233·8–294·3)	-14·8% (-19·2 to -9·9%)
	Ischaemic stroke	10857 (9258–12752)	847·6% (743·8 to 952·1%)	208·2 (180·3–240·9)	-5% (-11·4 to 2·3%)
	Intracerebral haemorrhage	2561 (2257–2883)	432·8% (394·6 to 479·2%)	42·3 (38·1–47)	-41·9% (-45·3 to -38·6%)
	Subarachnoid haemorrhage	1064 (849–1323)	562·9% (494·7 to 631·6%)	11·7 (9·9–13·7)	-25·2% (-29·9 to -20·7%)
	Neurological disorders†	973713 (829158–1124932)	435·9% (391·1 to 480·3%)	9865·6 (8796·4–10962·3)	0% (-0·7 to 0·6%)
	Alzheimer's disease and other dementias	1369 (1110–1622)	669·5% (600·8 to 739·7%)	103·1 (86·7–119·4)	-0·9% (-3·8 to 2·1%)
	Parkinson's disease	471 (402–563)	854·7% (769·4 to 943·5%)	19 (17·1–21·2)	14·3% (7·1 to 21·3%)
	Idiopathic epilepsy	5334 (1502–8982)	307·6% (17·5 to 1375·9%)	65·1 (18·4–106·5)	-6% (-73·4 to 237·2%)
	Multiple sclerosis	152 (120–190)	498·6% (387·4 to 612·9%)	1·1 (0·9–1·2)	5% (-1·1 to 12·2%)

Table S12 Burden, mortality, incidence, and prevalence of neurological conditions in North Africa and Middle East countries

		**All Ages**		**Age-standardised**	
		Number	Percent change	Rate per 100,000	Percent change
**Measure**		2019	From 1990 to 2019	2019	From 1990 to 2019
Cause	Location	Mean (95% UI)	Mean (95% UI)	Mean (95% UI)	Mean (95% UI)
	Migraine	103202 (85999–123478)	338·5% (291·1 to 393·5%)	1148·7 (968·7–1333·5)	0·3% (-0·2 to 1%)
	Tension-type headache	863143 (722148–1015406)	451·2% (400·5 to 502·6%)	8528·1 (7490·2–9612·7)	-0·1% (-0·3 to 0·1%)
	Motor neuron disease	42 (31–56)	516·2% (409·8 to 613·1%)	0·5 (0·4–0·6)	8·5% (4·1 to 12·8%)
	Other neurological disorders	0 (0–0)	0% (0 to 0%)	0 (0–0)	0% (0 to 0%)
	Headache disorders	966345 (822373–1117064)	436·5% (392·1 to 481·4%)	9676·8 (8629·4–10745·8)	0% (-0·2 to 0·2%)
Yemen	Meningitis	7684 (6256–9394)	15·2% (3·4 to 29·5%)	23·5 (20–27·6)	-34·6% (-39·3 to -29·3%)
	Encephalitis	2888 (2393–3447)	89·1% (75·4 to 102·9%)	8·4 (7·2–9·8)	-2% (-6·8 to 2·6%)
	Tetanus	86 (39–184)	-82·3% (-94·2 to -29·8%)	0·3 (0·2–0·7)	-87% (-94·8 to -58·9%)
	Brain and central nervous system cancer	842 (503–1211)	177·9% (48·6 to 409·1%)	3·9 (2·4–5·7)	26·2% (-26·8 to 104·5%)
	Stroke*	32051 (29342–34986)	145·9% (134·6 to 157·8%)	207·9 (190·1–228·3)	-9·5% (-14 to -4·7%)
	Ischaemic stroke	21937 (19200–24782)	216·3% (194·7 to 239·4%)	147·6 (129·8–168·3)	17·1% (9·1 to 25·7%)
	Intracerebral haemorrhage	7447 (6770–8165)	55·2% (47·8 to 62·4%)	44·7 (40·7–48·8)	-45·8% (-48·6 to -42·9%)
	Subarachnoid haemorrhage	2667 (2281–3109)	104·9% (90·7 to 122·2%)	15·5 (13·2–18·2)	-26·3% (-31·3 to -20·2%)
	Neurological disorders†	2994086 (2628346–3374853)	150% (143·6 to 156·7%)	9895·4 (8807·4–10988·4)	0% (-0·4 to 0·4%)
	Alzheimer's disease and other dementias	9717 (8284–11147)	187·9% (177 to 199·2%)	109·3 (93·5–124·4)	-2·7% (-5·7 to 0·3%)
	Parkinson's disease	1005 (878–1130)	207·8% (183·7 to 237·2%)	8·8 (7·8–9·8)	11·5% (3·4 to 21·9%)
	Idiopathic epilepsy	12744 (3088–23978)	118·1% (-53·8 to 1156·3%)	35·3 (8·5–66)	4·1% (-78 to 476%)
	Multiple sclerosis	268 (216–324)	233·9% (217·3 to 251·1%)	0·9 (0·7–1)	17·1% (11·5 to 22·6%)
	Migraine	441953 (365086–521903)	136·6% (126·1 to 146·9%)	1236·1 (1049·1–1424·3)	-0·1% (-0·3 to 0%)
	Tension-type headache	2528256 (2163210–2899036)	152·5% (145·1 to 159·9%)	8504·5 (7443·4–9580·9)	0% (0 to 0·1%)
	Motor neuron disease	144 (116–173)	111·3% (93 to 130·7%)	0·5 (0·4–0·7)	1% (-2·7 to 5·3%)
	Other neurological disorders	0 (0–0)	0% (0 to 0%)	0 (0–0)	0% (0 to 0%)
	Headache disorders	2970209 (2605021–3349282)	150% (143·5 to 156·6%)	9740·6 (8655·2–10825·6)	0% (-0·1 to 0·1%)
Afghanistan	Meningitis	25187 (21806–28902)	75·6% (64·8 to 88·3%)	86·2 (76·3–96·4)	-35·7% (-38·3 to -32·9%)
	Encephalitis	7014 (6176–8040)	225·2% (209·5 to 242·2%)	19·4 (17·5–21·5)	0·1% (-3·8 to 4·2%)
	Tetanus	1096 (645–1872)	-71·2% (-85·3 to -40%)	3·4 (2–5·2)	-86·8% (-92·3 to -75·7%)
	Brain and central nervous system cancer	1264 (694–2276)	152% (64 to 330·3%)	5·1 (3–9·3)	2·3% (-33·1 to 60·9%)
	Stroke*	36785 (33645–40296)	111·8% (99·3 to 123·9%)	217·7 (200·8–238·5)	-7·7% (-11·8 to -3·2%)
	Ischaemic stroke	22218 (19362–25555)	154·7% (130·9 to 180·7%)	140·3 (123·4–159·4)	18·9% (10·7 to 27·7%)
	Intracerebral haemorrhage	11653 (10651–12823)	63·2% (53·8 to 73·7%)	61 (56–66·3)	-37·6% (-40·2 to -34·7%)
	Subarachnoid haemorrhage	2914 (2471–3422)	93·4% (75·2 to 115·5%)	16·3 (13·8–19·3)	-19·2% (-24 to -12·1%)
	Neurological disorders†	3489660 (3052224–3961436)	238·5% (228·4 to 247·1%)	9901·9 (8816·5–10966·1)	0% (-0·5 to 0·6%)
	Alzheimer's disease and other dementias	8459 (7105–9788)	55·2% (48·4 to 62·1%)	109·4 (92·9–125·6)	-1·9% (-5·1 to 1·6%)
	Parkinson's disease	1102 (984–1236)	63·2% (48·2 to 78·2%)	10·5 (9·5–11·6)	-2·8% (-8·8 to 4%)
	Idiopathic epilepsy	16073 (2945–31796)	234·2% (-28·5 to 2078·6%)	35·5 (6·5–69·7)	-2·6% (-79·7 to 535%)
	Multiple sclerosis	588 (490–704)	342·8% (318 to 368·5%)	1·6 (1·4–1·9)	12·3% (7·4 to 17·5%)
	Migraine	524948 (430066–623815)	255% (247·6 to 264·2%)	1229 (1042·8–1418)	-2·2% (-2·7 to -1·8%)
	Tension-type headache	2938263 (2512679–3388697)	237% (225·6 to 247%)	8515·2 (7457·9–9593·9)	0·4% (0·2 to 0·6%)
	Motor neuron disease	226 (182–278)	203·3% (184·4 to 223·2%)	0·7 (0·6–0·8)	2·1% (-1 to 5·8%)
	Other neurological disorders	0 (0–0)	0% (0 to 0%)	0 (0–0)	0% (0 to 0%)

Table S12 Burden, mortality, incidence, and prevalence of neurological conditions in North Africa and Middle East countries

		All Ages		Age-standardised	
		Number	Percent change	Rate per 100,000	Percent change
**Measure**		2019	From 1990 to 2019	2019	From 1990 to 2019
Cause	Location	Mean (95% UI)	Mean (95% UI)	Mean (95% UI)	Mean (95% UI)
	Headache disorders	3463211 (3022728–3937188)	239·6% (230 to 248·4%)	9744·2 (8662·8–10828·1)	0·1% (-0·1 to 0·3%)
Sudan	Meningitis	5966 (4872–7163)	-60·6% (-63·9 to -57·1%)	14 (11·9–16·4)	-74·4% (-75·9 to -72·6%)
	Encephalitis	3596 (2974–4272)	71·3% (59·9 to 82·7%)	8·3 (7–9·6)	-3·4% (-7·6 to 0·9%)
	Tetanus	58 (27–122)	-91% (-96·5 to -75·3%)	0·2 (0·1–0·4)	-92·6% (-96·8 to -84·1%)
	Brain and central nervous system cancer	1354 (800–1961)	111% (2·4 to 346·1%)	4·4 (2·6–6·2)	16·6% (-35·3 to 106·5%)
	Stroke*	48595 (44282–53816)	101% (91 to 112·3%)	227·1 (205–253·2)	-4·8% (-9·7 to 0·9%)
	Ischaemic stroke	34529 (30159–39646)	157·6% (139·6 to 178·8%)	166·7 (145–190·9)	22·7% (14·3 to 32·1%)
	Intracerebral haemorrhage	10580 (9645–11599)	24·2% (17·5 to 30·6%)	45·8 (41·6–50·2)	-44·3% (-47·4 to -41·5%)
	Subarachnoid haemorrhage	3486 (3000–4062)	54·4% (43·3 to 65·9%)	14·6 (12·5–17)	-28·8% (-33·5 to -23·9%)
	Neurological disorders†	3902295 (3426301–4410513)	114% (111·1 to 116·9%)	9879·3 (8797·9–10946·5)	-0·1% (-0·6 to 0·3%)
	Alzheimer's disease and other dementias	14895 (12715–17101)	107·3% (97·2 to 117·9%)	108·2 (92·1–123·5)	-1·3% (-4·4 to 1·8%)
	Parkinson's disease	1552 (1379–1745)	94·7% (77·4 to 114·4%)	9·4 (8·4–10·6)	-1·2% (-8·9 to 7·9%)
	Idiopathic epilepsy	17413 (4224–32374)	104·9% (-50·1 to 982·3%)	38 (9·2–69·8)	10·1% (-72·8 to 488·9%)
	Multiple sclerosis	352 (282–426)	169·4% (156·9 to 182·3%)	0·9 (0·7–1)	16·9% (12·1 to 22%)
	Migraine	566587 (470251–665008)	110% (105·7 to 113·9%)	1240 (1052·1–1428·4)	-0·3% (-0·4 to -0·1%)
	Tension-type headache	3301315 (2843973–3772470)	114·8% (111·7 to 117·8%)	8482·4 (7418·8–9558·5)	-0·1% (-0·2 to -0·1%)
	Motor neuron disease	182 (147–220)	80·9% (68·1 to 93·4%)	0·5 (0·4–0·6)	-1·7% (-5·2 to 2·6%)
	Other neurological disorders	0 (0–0)	0% (0 to 0%)	0 (0–0)	0% (0 to 0%)
	Headache disorders	3867901 (3387749–4370946)	114·1% (111·4 to 116·6%)	9722·3 (8634·6–10808)	-0·2% (-0·2 to -0·1%)
**Prevalence**					
Global	Meningitis	7683540 (6590288–9132199)	-23·7% (-26 to -21·1%)	99·9 (85·5–118·8)	-47·2% (-48·7 to -45·5%)
	Encephalitis	4499425 (3372091–5573174)	4·7% (1·6 to 8·9%)	56·8 (42·6–70·3)	-31·9% (-33·9 to -28·5%)
	Tetanus	61660 (44603–81241)	-64·4% (-68·1 to -61·2%)	0·8 (0·6–1·1)	-73·7% (-76·2 to -71·5%)
	Brain and central nervous system cancer	1065294 (800441–1199906)	151·5% (50·8 to 195·7%)	13·5 (10·1–15·2)	59·7% (-2·4 to 86·2%)
	Stroke*	101474558 (93211910–110526302)	85·3% (82·6 to 88·2%)	1240·3 (1139·7–1353)	-6·1% (-7·2 to -5%)
	Ischaemic stroke	77192499 (68857178–86457604)	95·3% (91·7 to 99·3%)	951 (849·8–1064·1)	-1·9% (-3·4 to -0·4%)
	Intracerebral haemorrhage	20663889 (18016197–23417927)	57·8% (56·1 to 59·6%)	248·8 (217·1–281·4)	-16·8% (-18·2 to -15·4%)
	Subarachnoid haemorrhage	8396541 (7188193–9833329)	64·5% (60·4 to 68%)	101·6 (87·1–118·5)	-12·9% (-15 to -11·5%)
	Neurological disorders†	2658932358 (2452847930–2858528818)	54·9% (52·4 to 57·4%)	33451·9 (30870·7–36082)	0·4% (-0·1 to 0·9%)
	Alzheimer's disease and other dementias	51624193 (44276969–59021502)	160·9% (156 to 166·3%)	682·5 (585·2–782·7)	5·7% (4·3 to 7%)
	Parkinson's disease	8511022 (7288530–9841378)	155·5% (150·4 to 161%)	106·3 (91·2–122·2)	15·9% (13·3 to 18·5%)
	Idiopathic epilepsy	25111110 (19033571–31433013)	63·9% (39·3 to 93%)	326·3 (247·8–408·3)	13% (-3·4 to 31·8%)
	Multiple sclerosis	1756792 (1531919–1973623)	71·7% (66·3 to 76·8%)	21·3 (18·5–23·9)	-6·2% (-8·7 to -3·8%)
	Migraine	1128087261 (979598830–1298138078)	56·3% (52·3 to 60·5%)	14107·3 (12270·3–16239)	1·7% (0·7 to 2·8%)
	Tension-type headache	1995172549 (1751946846–2242204885)	52·6% (49·2 to 55·9%)	25113·5 (22020·8–28316·2)	-0·8% (-1·5 to 0%)

Table S12 Burden, mortality, incidence, and prevalence of neurological conditions in North Africa and Middle East countries

		All Ages		Age-standardised	
		Number	Percent change	Rate per 100,000	Percent change
**Measure**		2019	From 1990 to 2019	2019	From 1990 to 2019
Cause	Location	Mean (95% UI)	Mean (95% UI)	Mean (95% UI)	Mean (95% UI)
	Motor neuron disease	268674 (231894–310664)	68·9% (62·5 to 75·7%)	3·4 (2·9–3·9)	1·9% (0·6 to 3·4%)
	Other neurological disorders	56882 (39066–77832)	66·3% (56·8 to 77%)	0·7 (0·5–1)	6·4% (3·6 to 9·5%)
	Headache disorders	2602898103 (2396738294–2805119610)	54% (51·5 to 56·5%)	32716·8 (30148·4–35335)	0·2% (-0·3 to 0·8%)
North Africa and Middle East	Meningitis	356233 (306659–419924)	5·7% (-1·6 to 14%)	60·5 (52·2–71·1)	-44·7% (-48·4 to -40·7%)
	Encephalitis	167496 (127752–205936)	79·3% (73·4 to 86·9%)	28 (21·2–34·6)	-10·4% (-13·5 to -5·9%)
	Tetanus	748 (500–1074)	-35·7% (-53·8 to -16·2%)	0·1 (0·1–0·2)	-61·7% (-70·5 to -51·9%)
	Brain and central nervous system cancer	97195 (64216–115621)	280·5% (113·9 to 424·7%)	16·5 (10·8–19·5)	119·1% (30·2 to 189·1%)
	Stroke*	7323421 (6794727–7863138)	142·1% (137·8 to 146·3%)	1537·5 (1421·9–1659·9)	-0·5% (-2·3 to 1·1%)
	Ischaemic stroke	5998774 (5473979–6566718)	157·6% (152·8 to 162·7%)	1303·6 (1183·2–1435·4)	5·9% (3·6 to 8%)
	Intracerebral haemorrhage	1301257 (1169230–1441443)	89·8% (86 to 93·8%)	241·6 (217·5–265·7)	-21·1% (-23·1 to -19·2%)
	Subarachnoid haemorrhage	376883 (320184–444637)	103·4% (85 to 112·9%)	64 (54·5–75·3)	-18·1% (-26·3 to -14·1%)
	Neurological disorders†	208460497 (190465729–226945563)	101·2% (95·9 to 106·6%)	34170·6 (31389·5–37068·5)	1·4% (0·6 to 2·3%)
	Alzheimer's disease and other dementias	2485062 (2117243–2864990)	184·5% (178·1 to 190·7%)	777·6 (660·8–896)	3% (1·5 to 4·6%)
	Parkinson's disease	309887 (264964–362774)	199·5% (188·8 to 211·2%)	82·6 (70·2–95·6)	15·4% (11·5 to 20%)
	Idiopathic epilepsy	1990924 (1360240–2600038)	77·6% (26·2 to 150·6%)	336·9 (231·8–437·4)	8·6% (-23 to 52·4%)
	Multiple sclerosis	222696 (190733–256781)	171·3% (165·8 to 176·5%)	39 (33·6–44·7)	11·5% (10 to 12·8%)
	Migraine	96931476 (83756714–112609351)	100·9% (92·7 to 108·8%)	15355 (13305·5–17806)	-0·1% (-1·5 to 1·4%)
	Tension-type headache	149061721 (128455947–170990926)	102·2% (93·5 to 111%)	24504·5 (21304·8–27987·5)	2% (0·7 to 3·4%)
	Motor neuron disease	15574 (12657–19025)	103·3% (92·1 to 113·8%)	2·6 (2·1–3·1)	3·5% (2 to 5·1%)
	Other neurological disorders	3937 (2627–5514)	96·6% (81 to 113·8%)	0·7 (0·5–1)	0·3% (-0·9 to 1·5%)
	Headache disorders	205280354 (186994538–223750113)	100·9% (95·4 to 106·3%)	33389·7 (30530·9–36328·1)	1·3% (0·5 to 2·2%)
Algeria	Meningitis	21625 (17717–26763)	21·7% (-1·1 to 48·1%)	53·2 (43·5–65·7)	-32·3% (-45·1 to -17·5%)
	Encephalitis	9650 (7397–11935)	87·5% (76·2 to 99·4%)	23·2 (17·7–28·8)	-0·2% (-6·2 to 6·6%)
	Tetanus	12 (7–20)	-10·2% (-45·5 to 17·6%)	0 (0–0)	-45·1% (-65·9 to -28·6%)
	Brain and central nervous system cancer	3451 (2055–4639)	240·6% (80·2 to 401·8%)	8·3 (4·9–11·1)	118·4% (15·6 to 213·7%)
	Stroke*	549736 (506627–592960)	129% (118·8 to 139·1%)	1540·3 (1417·2–1668)	-10·7% (-14·5 to -7%)
	Ischaemic stroke	469983 (427467–513763)	150·1% (138·7 to 161·4%)	1347·1 (1227–1477·6)	-2·6% (-6·7 to 1·7%)
	Intracerebral haemorrhage	78698 (70922–86722)	50% (43·8 to 56·9%)	201·9 (182·3–221·3)	-39·9% (-42·7 to -37·1%)
	Subarachnoid haemorrhage	27321 (21829–32379)	97·8% (51·7 to 114·1%)	64·7 (51·8–76·8)	-24·2% (-43 to -17·1%)
	Neurological disorders†	14066371 (12788073–15402985)	86·2% (78·9 to 93·9%)	33570·1 (30564·3–36622·8)	0·1% (-0·7 to 1%)
	Alzheimer's disease and other dementias	193058 (162815–224021)	231·7% (211·8 to 253·3%)	777·5 (659·7–898·6)	1·5% (-1·9 to 4·8%)
	Parkinson's disease	23956 (19889–28839)	217·4% (181 to 255·3%)	80·4 (66·5–96·8)	10·2% (-1·1 to 22%)
	Idiopathic epilepsy	131094 (38570–214998)	40·1% (-62·6 to 496·7%)	322·5 (94·5–529·8)	-7·9% (-76·4 to 293%)
	Multiple sclerosis	17581 (14373–21340)	222·5% (203·8 to 242%)	41·9 (34·2–50·6)	26·4% (20·4 to 33·6%)
	Migraine	6629210 (5653007–7727155)	87·9% (76·8 to 98·6%)	15365·7 (13120·8–17933·6)	-0·1% (-0·2 to 0%)
	Tension-type headache	9888031 (8443602–11527577)	85·5% (74·2 to 98·1%)	23647·6 (20341·2–27276·9)	-0·1% (-0·1 to 0%)

Table S12 Burden, mortality, incidence, and prevalence of neurological conditions in North Africa and Middle East countries

		**All Ages**		**Age-standardised**	
		Number	Percent change	Rate per 100,000	Percent change
**Measure**		2019	From 1990 to 2019	2019	From 1990 to 2019
Cause	Location	Mean (95% UI)	Mean (95% UI)	Mean (95% UI)	Mean (95% UI)
	Motor neuron disease	997 (799–1217)	89·8% (74·6 to 104·9%)	2·4 (1·9–2·8)	2% (-1·6 to 5·7%)
	Other neurological disorders	277 (185–389)	91·2% (70·5 to 114·3%)	0·7 (0·5–1)	-0·1% (-0·2 to 0·1%)
	Headache disorders	13831109 (12549817–15188452)	85·9% (78·2 to 93·4%)	32788·5 (29733·8–35917·9)	0·1% (-0·4 to 0·7%)
Bahrain	Meningitis	595 (485–726)	76·6% (45 to 110·6%)	40·1 (32·7–48·6)	-46·2% (-56·2 to -35·5%)
	Encephalitis	266 (208–332)	212·3% (193·4 to 232·4%)	17·1 (13·3–21·4)	-4·4% (-10·7 to 2%)
	Tetanus	1 (0–2)	168·5% (143·6 to 196·5%)	0·1 (0–0·1)	-5·9% (-15·2 to 3·8%)
	Brain and central nervous system cancer	206 (123–279)	723·9% (389·9 to 1098·2%)	14·8 (9·4–19·3)	147·5% (49·6 to 250·5%)
	Stroke*	14539 (13369–15844)	292·5% (272·2 to 313·4%)	1136·4 (1049–1234·8)	-24·2% (-27·5 to -20·8%)
	Ischaemic stroke	11186 (10069–12387)	297·3% (276·9 to 317·4%)	949·2 (859·7–1048·7)	-23·8% (-27·5 to -20·2%)
	Intracerebral haemorrhage	2862 (2545–3203)	223·4% (207·8 to 239·9%)	186 (166·5–205·7)	-31·2% (-34·4 to -28·1%)
	Subarachnoid haemorrhage	1059 (873–1262)	361·9% (326·7 to 396·5%)	53·2 (44·2–63·1)	-6·4% (-11·9 to -1·2%)
	Neurological disorders†	524416 (473877–576693)	221·5% (205·6 to 238·1%)	33112·7 (30197·4–36147)	-0·1% (-1·2 to 1%)
	Alzheimer's disease and other dementias	3757 (3198–4348)	450·3% (424·1 to 479·2%)	801·1 (684·2–925·8)	3·5% (0·2 to 7·1%)
	Parkinson's disease	674 (552–825)	493·1% (408·4 to 582·9%)	94 (75·9–113·4)	12·5% (-5·2 to 33·3%)
	Idiopathic epilepsy	5530 (1707–9062)	143·6% (-26·4 to 883·4%)	450·6 (140·2–726·5)	-10·6% (-72·9 to 252·3%)
	Multiple sclerosis	592 (471–734)	484·5% (438·6 to 535·1%)	32·7 (26·3–40·3)	29·1% (21·6 to 36·4%)
	Migraine	239676 (205264–279890)	215·9% (197·3 to 237·3%)	14419·8 (12318·8–16876·2)	-0·7% (-1 to -0·3%)
	Tension-type headache	377845 (318458–442847)	224·3% (196·2 to 253%)	23716 (20422–27274·1)	-0·1% (-0·3 to 0·1%)
	Motor neuron disease	46 (36–58)	235·2% (197·8 to 274·8%)	2·8 (2·3–3·4)	3·7% (-0·5 to 7·9%)
	Other neurological disorders	10 (7–15)	251·2% (197·9 to 315·7%)	0·7 (0·5–0·9)	-0·4% (-0·9 to 0%)
	Headache disorders	517784 (467000–570886)	221·6% (205 to 238·3%)	32232·2 (29237–35273·3)	-0·1% (-0·6 to 0·5%)
Egypt	Meningitis	60577 (49274–75589)	5·3% (-15·3 to 29·8%)	63·8 (51·9–79·7)	-42·7% (-54·3 to -29·3%)
	Encephalitis	27214 (20780–33672)	52·4% (42·3 to 64·1%)	28·5 (21·5–35·6)	-20·2% (-25·6 to -14%)
	Tetanus	128 (76–195)	-36·6% (-54·2 to -18%)	0·1 (0·1–0·2)	-63·5% (-73·5 to -53·2%)
	Brain and central nervous system cancer	8411 (5551–11708)	226·4% (63·4 to 401·9%)	8·7 (5·9–12)	81·5% (1·2 to 172%)
	Stroke*	1269834 (1170811–1374639)	151·8% (140·2 to 163·9%)	1806·1 (1658·1–1974·5)	18·5% (12·9 to 23·9%)
	Ischaemic stroke	1089819 (991038–1194962)	181·7% (168·2 to 194·4%)	1597 (1436·4–1765·4)	31·7% (24·9 to 38·1%)
	Intracerebral haemorrhage	191761 (173529–212078)	69% (62·2 to 75·9%)	236·1 (213·7–260·5)	-21·7% (-25·2 to -18·5%)
	Subarachnoid haemorrhage	54368 (45386–64247)	68·6% (46·1 to 79·3%)	61·4 (51·6–72·5)	-21·5% (-33 to -16·4%)
	Neurological disorders†	34352847 (31415782–37326127)	96·6% (89 to 104·2%)	35726·9 (32855·2–38512·6)	2·8% (-0·8 to 6·7%)
	Alzheimer's disease and other dementias	295756 (250876–342572)	121·4% (113·8 to 129·1%)	750·4 (635·6–866·1)	5·2% (1·4 to 8·7%)
	Parkinson's disease	43837 (36133–52603)	148·8% (120 to 177·7%)	84·4 (69·1–101)	17·3% (3·6 to 30·7%)
	Idiopathic epilepsy	274828 (77862–460774)	84·3% (-49·4 to 746%)	275·4 (79·2–459·6)	8·2% (-70·5 to 395%)
	Multiple sclerosis	17764 (14045–21567)	160·1% (150 to 171·7%)	20·6 (16·4–25)	22·2% (17·7 to 27·4%)
	Migraine	15818651 (13828617–18240820)	97·4% (83·2 to 112·2%)	15809·3 (13960·9–18055·5)	1·8% (-5 to 9%)
	Tension-type headache	25094802 (21538924–28759918)	97·1% (85·8 to 108·5%)	26290·9 (22878·1–29775·3)	3·6% (-1·6 to 9·5%)
	Motor neuron disease	2135 (1705–2641)	96·4% (87·1 to 105·5%)	2·2 (1·8–2·7)	4·2% (0·6 to 8·2%)
	Other neurological disorders	619 (413–862)	88·8% (80·3 to 97·3%)	0·7 (0·5–1)	0% (-0·2 to 0·3%)

Table S12 Burden, mortality, incidence, and prevalence of neurological conditions in North Africa and Middle East countries

		**All Ages**		**Age-standardised**	
		Number	Percent change	Rate per 100,000	Percent change
**Measure**		2019	From 1990 to 2019	2019	From 1990 to 2019
Cause	Location	Mean (95% UI)	Mean (95% UI)	Mean (95% UI)	Mean (95% UI)
	Headache disorders	33951125 (31033203–36963035)	96·5% (89·3 to 104·5%)	35022·4 (32158–37899·9)	2·7% (-0·8 to 6·7%)
Iran (Islamic Republic of)	Meningitis	58747 (50149–69255)	3% (-5·1 to 12·2%)	69·4 (59·2–82)	-35·7% (-40·3 to -30·8%)
	Encephalitis	20904 (16204–25628)	52·4% (47·1 to 59·2%)	24·2 (18·8–29·7)	-10·8% (-13·8 to -6·1%)
	Tetanus	20 (11–32)	-58·9% (-72·9 to -44·9%)	0 (0–0)	-69·4% (-78·2 to -61·1%)
	Brain and central nervous system cancer	23225 (12066–29504)	221·6% (78·6 to 352·2%)	27·7 (14·4–35·1)	129·4% (36·1 to 203·2%)
	Stroke*	963512 (859232–1079662)	123·4% (115·9 to 131·2%)	1253·8 (1113·5–1418·4)	-13·3% (-15·9 to -10·7%)
	Ischaemic stroke	849042 (736693–971863)	123·9% (115·1 to 132·9%)	1127·4 (977–1300·9)	-13·1% (-16·1 to -10·1%)
	Intracerebral haemorrhage	115797 (99985–132722)	89·9% (83·3 to 96·5%)	138·3 (120·1–157·2)	-16·3% (-19·5 to -13·1%)
	Subarachnoid haemorrhage	45366 (37280–54943)	127·3% (117·8 to 136·1%)	49·4 (40·8–59·3)	-7·1% (-8·9 to -5·2%)
	Neurological disorders†	33461615 (30981852–35941056)	81·4% (73·2 to 90%)	38297·2 (35449·7–41091·4)	5·3% (3·4 to 7·3%)
	Alzheimer's disease and other dementias	470948 (397868–542859)	303·8% (282·7 to 328·6%)	789 (667·8–910·4)	1·6% (-0·3 to 3·2%)
	Parkinson's disease	56514 (47335–66920)	260·9% (235 to 285·6%)	85·1 (70·2–101·3)	13·1% (10·4 to 15·8%)
	Idiopathic epilepsy	281691 (192954–373635)	28·3% (-5·1 to 72·5%)	352·8 (240·3–465·2)	1·5% (-24 to 35·3%)
	Multiple sclerosis	46854 (39948–53742)	135·2% (124·4 to 145·7%)	51·2 (44·1–58·5)	-4·9% (-8·2 to -1·6%)
	Migraine	14579445 (12748031–16637964)	75·8% (63·4 to 88·4%)	16048·8 (14063·2–18371·8)	-0·5% (-4 to 2·8%)
	Tension-type headache	25772952 (22795090–28770049)	88·3% (74·4 to 101·8%)	29640·4 (26202·1–32949·4)	9·7% (6·5 to 13·1%)
	Motor neuron disease	2546 (2012–3187)	86·9% (70·6 to 103·5%)	2·9 (2·3–3·5)	6·6% (4·9 to 8·4%)
	Other neurological disorders	540 (356–773)	69·6% (46·7 to 96·5%)	0·6 (0·4–0·9)	0·2% (-0·1 to 0·4%)
	Headache disorders	32958409 (30482353–35496741)	81% (72·7 to 89·6%)	37542·1 (34654·1–40354·8)	5·4% (3·5 to 7·5%)
Iraq	Meningitis	23872 (19687–29101)	46·7% (18·5 to 80·2%)	59·8 (49·5–72·5)	-42% (-53·5 to -28·6%)
	Encephalitis	11216 (8551–13841)	131·8% (119·3 to 146·5%)	28·4 (21·2–35·2)	-13·3% (-18·3 to -7·4%)
	Tetanus	46 (29–67)	-23·6% (-51·5 to 6·3%)	0·1 (0·1–0·2)	-63·5% (-73·9 to -53·4%)
	Brain and central nervous system cancer	9453 (6780–12580)	422·3% (154·6 to 768·9%)	23·5 (17–31·2)	123·7% (14·7 to 254·7%)
	Stroke*	520023 (483072–557268)	169·6% (159 to 180·7%)	1968·8 (1823·4–2122·1)	-9·6% (-13·3 to -5·4%)
	Ischaemic stroke	414374 (378236–449645)	169·7% (158·8 to 182·2%)	1656·7 (1500·2–1820)	-7·6% (-11·8 to -2·6%)
	Intracerebral haemorrhage	110425 (101055–120024)	164·6% (154·8 to 175·5%)	346·5 (316·6–378·4)	-16% (-19·1 to -12·4%)
	Subarachnoid haemorrhage	21047 (16904–24891)	141·6% (88·5 to 162%)	58·9 (46–70)	-26·1% (-45·7 to -19·5%)
	Neurological disorders†	13749679 (12424473–15155117)	174·7% (168·3 to 181·5%)	33513·6 (30526·4–36599·7)	0·2% (-0·6 to 1·1%)
	Alzheimer's disease and other dementias	118870 (101493–137301)	165·6% (156·2 to 176·3%)	764·1 (647·4–882)	3·8% (0·7 to 7·5%)
	Parkinson's disease	14382 (11802–17136)	193·4% (168·2 to 226·9%)	74·2 (61·1–87·9)	5·1% (-4·3 to 17·1%)
	Idiopathic epilepsy	126929 (33569–217387)	125·5% (-39·4 to 851·8%)	299·1 (77·8–510·2)	-3·3% (-74·2 to 300·6%)
	Multiple sclerosis	12797 (10065–16073)	274·2% (256·6 to 293·6%)	37·8 (30–46·9)	19·5% (14·2 to 25·2%)
	Migraine	6522476 (5506459–7700191)	179·5% (170·3 to 187·6%)	15300·4 (13067–17857·9)	-0·1% (-0·1 to -0·1%)
	Tension-type headache	9672804 (8160231–11338809)	173·9% (163·8 to 183·3%)	23667·9 (20363·1–27281·6)	0% (0 to 0%)
	Motor neuron disease	1025 (822–1264)	170·7% (154·7 to 186·6%)	2·5 (2·1–3·1)	2·6% (-1·2 to 6·4%)
	Other neurological disorders	253 (169–354)	156·2% (137·6 to 177·1%)	0·7 (0·5–1)	0% (0 to 0%)
	Headache disorders	13572476 (12216221–14975491)	175·3% (169·1 to 181·8%)	32763·7 (29727·1–35826·9)	0·2% (-0·3 to 0·7%)
Jordan	Meningitis	9454 (7810–11615)	174·9% (126·3 to 229·7%)	86·2 (71·1–105·2)	-17·7% (-32·3 to -1·2%)

Table S12 Burden, mortality, incidence, and prevalence of neurological conditions in North Africa and Middle East countries

		All Ages		Age-standardised	
		Number	Percent change	Rate per 100,000	Percent change
**Measure**		2019	From 1990 to 2019	2019	From 1990 to 2019
Cause	Location	Mean (95% UI)	Mean (95% UI)	Mean (95% UI)	Mean (95% UI)
	Encephalitis	2852 (2206–3537)	194·6% (179 to 209·4%)	25·8 (19·8–32·2)	-14·3% (-19·2 to -9·7%)
	Tetanus	4 (2–7)	71·8% (23·6 to 119%)	0 (0–0·1)	-40·8% (-55·8 to -26·1%)
	Brain and central nervous system cancer	1782 (1268–2270)	709·6% (408·1 to 1031·3%)	15·8 (11·4–20)	146·1% (57·1 to 240·4%)
	Stroke*	134580 (123260–145467)	258·4% (239·7 to 275%)	1793·9 (1621·2–1952·9)	-23·4% (-27·7 to -19·5%)
	Ischaemic stroke	113823 (102391–124390)	256·8% (238·1 to 273·9%)	1591·7 (1411·6–1753·9)	-23·9% (-28·6 to -19·4%)
	Intracerebral haemorrhage	19651 (17293–22289)	218·2% (201·4 to 234·9%)	213·9 (189·8–238·7)	-29·2% (-33·2 to -25%)
	Subarachnoid haemorrhage	7841 (6569–9273)	347·5% (322·3 to 392·6%)	74·9 (62·3–88·5)	-1·1% (-5·6 to 8·6%)
	Neurological disorders†	3820339 (3444388–4203289)	248·2% (237·8 to 259·4%)	33366·2 (30403·8–36446·1)	0·1% (-0·7 to 0·9%)
	Alzheimer's disease and other dementias	31996 (26991–37367)	432·9% (411·5 to 453·7%)	763·4 (645·9–890)	3·8% (-0·1 to 7·8%)
	Parkinson's disease	4127 (3383–4884)	425·7% (364·4 to 488·4%)	77 (63·4–91·5)	3% (-11·5 to 16·6%)
	Idiopathic epilepsy	35446 (9652–59654)	204·5% (-20·6 to 1036·4%)	302·9 (83·8–498·4)	3·5% (-73·1 to 291·1%)
	Multiple sclerosis	4613 (3653–5615)	345·7% (289·6 to 401·1%)	47 (37·9–56·8)	-1·2% (-13·3 to 10·6%)
	Migraine	1789505 (1517280–2114905)	247·9% (233·8 to 262·4%)	15033 (12863·2–17565·4)	-1% (-1·1 to -0·8%)
	Tension-type headache	2700147 (2285860–3158502)	248·3% (232·6 to 266·5%)	23661·5 (20365·4–27259·2)	0·1% (0 to 0·2%)
	Motor neuron disease	308 (248–380)	252% (224·4 to 277·9%)	2·7 (2·2–3·3)	3% (-1·7 to 7·2%)
	Other neurological disorders	92 (62–128)	288·5% (245 to 338·6%)	0·9 (0·6–1·2)	11·4% (3·6 to 20·5%)
	Headache disorders	3770800 (3396204–4150448)	247·8% (237·5 to 258·9%)	32603·8 (29560·1–35676·2)	0% (-0·6 to 0·5%)
Kuwait	Meningitis	1650 (1360–2032)	102·1% (65 to 145·4%)	37·6 (31–46·1)	-25·8% (-39·2 to -9·4%)
	Encephalitis	742 (579–916)	173·5% (154 to 193·4%)	16·1 (12·5–20)	-3·3% (-10·5 to 4·2%)
	Tetanus	4 (2–6)	136% (117 to 157·4%)	0·1 (0–0·1)	-6·2% (-13·7 to 2·4%)
	Brain and central nervous system cancer	833 (578–1059)	417% (212·8 to 610·5%)	20·8 (14·7–26·2)	120·7% (35·2 to 197·4%)
	Stroke*	42739 (39334–46356)	267·6% (248·5 to 287·1%)	1230·6 (1134·6–1332·5)	-5·9% (-10·4 to -1·4%)
	Ischaemic stroke	32970 (29762–36320)	264·5% (248·5 to 281%)	1032·1 (934·4–1132·1)	-6·5% (-11 to -2·1%)
	Intracerebral haemorrhage	7685 (6691–8702)	239·2% (220·8 to 258%)	181·9 (160·3–202·9)	-5·9% (-11 to -1·2%)
	Subarachnoid haemorrhage	3852 (3238–4565)	281·6% (257·9 to 310·1%)	71·5 (59–84·8)	2·1% (-3 to 10%)
	Neurological disorders†	1592630 (1444629–1756386)	180·3% (168·3 to 192·5%)	33272 (30301·2–36326·1)	0·8% (-1·7 to 3·5%)
	Alzheimer's disease and other dementias	14284 (12285–16371)	401·3% (382·3 to 422%)	798·8 (682·4–922)	1·3% (-2·1 to 4·8%)
	Parkinson's disease	1581 (1292–1923)	315·1% (261·1 to 379·4%)	71·4 (59·1–86·1)	-8·5% (-23·9 to 9·9%)
	Idiopathic epilepsy	15171 (4283–24128)	119·1% (-37·4 to 718·6%)	388·9 (106·7–618·4)	-4·5% (-73·2 to 267·1%)
	Multiple sclerosis	2717 (2228–3296)	485·7% (448·8 to 526·8%)	53 (43·7–63·5)	59·7% (50·5 to 69·9%)
	Migraine	764424 (652804–896685)	189·8% (171·5 to 208·5%)	14884·8 (12735·9–17378·9)	3·7% (-0·5 to 7·9%)
	Tension-type headache	1117900 (946810–1313413)	175·2% (156·1 to 195·5%)	23533 (20134·8–26965·1)	-0·9% (-4·9 to 3%)
	Motor neuron disease	152 (121–190)	166·7% (143·3 to 192·9%)	3·1 (2·6–3·8)	-4·2% (-9·8 to 1·1%)
	Other neurological disorders	30 (20–45)	197·2% (159·5 to 237·8%)	0·7 (0·5–1)	2% (-4 to 9·2%)
	Headache disorders	1571200 (1423071–1732552)	179·9% (167·6 to 192·2%)	32425·7 (29466·2–35506·5)	0·8% (-1·8 to 3·3%)
Lebanon	Meningitis	2893 (2379–3561)	2·5% (-15·3 to 24·6%)	55·7 (45·8–68·5)	-39·8% (-50·5 to -26·9%)
	Encephalitis	1684 (1292–2085)	36·5% (29·2 to 44%)	32·1 (24·7–39·8)	-23·2% (-27·5 to -18·4%)
	Tetanus	3 (1–9)	-39·7% (-80·8 to 55%)	0·1 (0–0·2)	-72·6% (-90·5 to -27·6%)
	Brain and central nervous system cancer	1781 (1230–2343)	535·7% (309·1 to 812·8%)	34 (23·6–44·8)	284·7% (150·6 to 439·7%)

Table S12 Burden, mortality, incidence, and prevalence of neurological conditions in North Africa and Middle East countries

		**All Ages**		**Age-standardised**	
		Number	Percent change	Rate per 100,000	Percent change
**Measure**		2019	From 1990 to 2019	2019	From 1990 to 2019
Cause	Location	Mean (95% UI)	Mean (95% UI)	Mean (95% UI)	Mean (95% UI)
	Stroke*	74841 (69385–80771)	124·2% (114·6 to 134·6%)	1425·1 (1320·3–1538·1)	2·2% (-1·8 to 6·6%)
	Ischaemic stroke	65698 (59943–71554)	141·5% (131·1 to 153%)	1254 (1145–1367·4)	8·4% (4 to 13·1%)
	Intracerebral haemorrhage	8994 (8003–9978)	51·2% (44·4 to 58%)	170·3 (151·9–188·9)	-25·4% (-29 to -21·9%)
	Subarachnoid haemorrhage	3628 (3015–4328)	81% (63·8 to 91·7%)	67·8 (56·1–80·9)	-9·6% (-18·9 to -4·4%)
	Neurological disorders†	1765467 (1607119–1930344)	76·4% (71·6 to 81·4%)	33723·8 (30708·5–36796·6)	0·5% (-0·4 to 1·4%)
	Alzheimer's disease and other dementias	39913 (34066–46450)	221% (204·8 to 235·4%)	792·4 (677·6–916·5)	4·2% (0·5 to 8·4%)
	Parkinson's disease	4065 (3476–4757)	192·1% (157·6 to 228·7%)	77·6 (66·5–90·9)	9·3% (-3·1 to 23·6%)
	Idiopathic epilepsy	17459 (5094–28152)	56·4% (-59·4 to 598·3%)	343·8 (99·5–558·2)	3·3% (-72·7 to 371·4%)
	Multiple sclerosis	2918 (2372–3504)	179·2% (162·9 to 199·5%)	53·9 (43·9–64·2)	34·7% (27·2 to 43·9%)
	Migraine	819115 (700606–950606)	75·4% (68·5 to 81·6%)	15516·6 (13250·8–18080·2)	0·2% (0 to 0·4%)
	Tension-type headache	1237281 (1062030–1432419)	75·6% (67·8 to 83·6%)	23655 (20353·1–27281·3)	0·1% (0 to 0·3%)
	Motor neuron disease	148 (121–181)	77·2% (65·9 to 87·5%)	2·8 (2·3–3·4)	2·8% (-1 to 6·9%)
	Other neurological disorders	36 (24–50)	74·9% (63·2 to 87·3%)	0·7 (0·5–1)	0·2% (-0·1 to 0·6%)
	Headache disorders	1724497 (1566585–1888655)	75·3% (70·5 to 80·2%)	32916·6 (29885·3–36014·1)	0·4% (-0·1 to 0·9%)
Libya	Meningitis	3369 (2778–4257)	52·6% (23·9 to 86·7%)	50·3 (41·7–63·7)	-14·6% (-31 to 3·9%)
	Encephalitis	1302 (988–1628)	151·6% (137·9 to 165·6%)	18·8 (14·2–23·5)	33·2% (26·1 to 40·4%)
	Tetanus	2 (1–3)	15·8% (-16·4 to 39·3%)	0 (0–0)	-26·3% (-48·5 to -11·7%)
	Brain and central nervous system cancer	1010 (727–1362)	173·7% (77·5 to 332·5%)	15·3 (11–20·2)	69·6% (17·6 to 153·3%)
	Stroke*	90351 (83440–96922)	201·2% (186·1 to 214·9%)	1588·4 (1461·7–1712·9)	14% (8·9 to 18·7%)
	Ischaemic stroke	76359 (69400–82982)	223·3% (207·5 to 239%)	1395·3 (1262·3–1526·8)	23·5% (17·7 to 29·4%)
	Intracerebral haemorrhage	13165 (11865–14473)	104·8% (97 to 113·1%)	202·1 (182·3–222·3)	-21·3% (-24·3 to -18%)
	Subarachnoid haemorrhage	4772 (3877–5658)	146·3% (106·5 to 162·2%)	63·7 (51·5–75·1)	-11·4% (-27·1 to -6·2%)
	Neurological disorders†	2417198 (2194984–2643442)	96·6% (87·7 to 105·5%)	33496·5 (30469–36534·7)	0·7% (-0·1 to 1·4%)
	Alzheimer's disease and other dementias	30490 (25757–35237)	169·1% (159·2 to 178·8%)	755·9 (635·1–874·7)	-1·1% (-4·6 to 2·6%)
	Parkinson's disease	3830 (3183–4453)	213·5% (184·3 to 251·5%)	84·1 (69·3–98·3)	13·5% (2·3 to 27·9%)
	Idiopathic epilepsy	18517 (6097–29168)	25·6% (-59·8 to 364%)	287·1 (93·7–450·4)	-15·5% (-72·8 to 217·9%)
	Multiple sclerosis	2974 (2455–3556)	261·2% (240·2 to 282·5%)	41·1 (34·2–48·9)	31·6% (24·9 to 38·4%)
	Migraine	1150029 (980241–1340837)	102·9% (88·9 to 116·4%)	15252·6 (13031·9–17806·7)	1·9% (1·6 to 2·2%)
	Tension-type headache	1698733 (1444446–1978942)	94·9% (81·4 to 109·5%)	23667·9 (20357·9–27281·4)	-0·1% (-0·2 to 0·1%)
	Motor neuron disease	164 (132–204)	81·7% (64·2 to 98·3%)	2·3 (1·9–2·8)	-4·9% (-8·6 to -0·9%)
	Other neurological disorders	40 (26–57)	126·5% (89·7 to 177·6%)	0·6 (0·4–0·9)	19·1% (6·4 to 36·4%)
	Headache disorders	2382119 (2156062–2608297)	96·7% (87·5 to 105·6%)	32752·8 (29720–35810·4)	0·8% (0·3 to 1·3%)
Morocco	Meningitis	24546 (19831–30631)	-27·1% (-41·4 to -10·6%)	69·2 (55·9–86·4)	-51·8% (-61·5 to -40·5%)
	Encephalitis	9842 (7414–12268)	43·3% (35·1 to 52·7%)	27 (20·3–33·7)	-10·3% (-15·8 to -4%)
	Tetanus	132 (85–187)	-48·9% (-67·7 to -28·2%)	0·4 (0·2–0·5)	-57·8% (-71·1 to -43·8%)
	Brain and central nervous system cancer	1647 (1052–2316)	158·2% (39 to 300·6%)	4·6 (3–6·5)	79·1% (0·8 to 165·4%)
	Stroke*	557119 (515485–599727)	120·3% (110·8 to 131%)	1695·1 (1563·4–1833·2)	3·4% (-0·7 to 8·2%)
	Ischaemic stroke	476785 (435246–521362)	144% (133 to 156·2%)	1480·4 (1346·4–1625·7)	13·5% (8·4 to 19·4%)
	Intracerebral haemorrhage	81150 (73393–90013)	46·4% (40·9 to 51·9%)	227·7 (205·7–251·9)	-29% (-31·8 to -26%)

Table S12 Burden, mortality, incidence, and prevalence of neurological conditions in North Africa and Middle East countries

		All Ages		Age-standardised	
		Number	Percent change	Rate per 100,000	Percent change
**Measure**		2019	From 1990 to 2019	2019	From 1990 to 2019
Cause	Location	Mean (95% UI)	Mean (95% UI)	Mean (95% UI)	Mean (95% UI)
	Subarachnoid haemorrhage	24641 (20517–29395)	58·4% (35·6 to 68·5%)	66·1 (55·6–78·6)	-22·9% (-34·4 to -17·4%)
	Neurological disorders†	12336972 (11206895–13482839)	58·5% (53·8 to 63%)	33537 (30530·4–36612·6)	0·2% (-0·6 to 1%)
	Alzheimer's disease and other dementias	174576 (147734–202323)	145% (135·7 to 155%)	761·7 (643·9–884·8)	0·5% (-2·9 to 4·3%)
	Parkinson's disease	20470 (17155–23848)	179·5% (147·3 to 219·8%)	73·7 (61·9–85·8)	20·7% (6·3 to 39%)
	Idiopathic epilepsy	103282 (24771–182256)	55·2% (-62·8 to 593%)	295·3 (70·8–519·2)	16·6% (-72·4 to 416·5%)
	Multiple sclerosis	15001 (12205–18295)	151·6% (136·6 to 168·2%)	40·9 (33·3–49·7)	26·4% (19·3 to 34·6%)
	Migraine	5801216 (4955330–6762318)	57·5% (50·8 to 63·8%)	15400·4 (13149·2–17963·3)	-0·3% (-0·4 to -0·3%)
	Tension-type headache	8691626 (7444134–10049060)	58·2% (51·7 to 65·2%)	23654·8 (20352·1–27282·8)	0% (-0·1 to 0%)
	Motor neuron disease	863 (701–1067)	62·3% (51·4 to 73·3%)	2·4 (1·9–2·9)	4% (0 to 8%)
	Other neurological disorders	242 (161–343)	63% (48·7 to 78·5%)	0·7 (0·5–1)	0% (-0·1 to 0·1%)
	Headache disorders	12136822 (10998942–13271258)	57·9% (53·2 to 62·5%)	32787·8 (29774–35877·7)	0·1% (-0·4 to 0·6%)
Palestine	Meningitis	3238 (2656–4044)	32·6% (8·9 to 60·5%)	71·5 (58·7–88·5)	-47·2% (-56·9 to -35·8%)
	Encephalitis	1535 (1189–1893)	153·8% (139·4 to 169%)	33·9 (25·9–42·1)	-4·8% (-10·5 to 1%)
	Tetanus	2 (1–3)	-7% (-58·9 to 44·8%)	0 (0–0·1)	-52·6% (-74·1 to -36·1%)
	Brain and central nervous system cancer	1143 (885–1498)	280·7% (122 to 456·2%)	25 (18·9–31·3)	60·2% (2·7 to 120·3%)
	Stroke*	41305 (37846–45067)	167·1% (153·9 to 180·9%)	1510·8 (1381·5–1666·5)	-3·7% (-8·5 to 1·4%)
	Ischaemic stroke	35217 (31813–38979)	168·2% (155·5 to 180·7%)	1341·8 (1205·3–1496·6)	-0·6% (-5·9 to 4·6%)
	Intracerebral haemorrhage	6105 (5301–7123)	137·1% (123·4 to 153%)	176·6 (155·3–203·6)	-21·6% (-26·1 to -16·1%)
	Subarachnoid haemorrhage	2509 (2061–2972)	207·6% (193·3 to 224·2%)	65·6 (54·2–77·7)	-4·3% (-9 to 0·6%)
	Neurological disorders†	1555737 (1395251–1715093)	172·9% (167·6 to 178·5%)	33527·9 (30565·1–36597·7)	-0·1% (-0·9 to 0·8%)
	Alzheimer's disease and other dementias	12043 (10286–13959)	135·9% (126·9 to 144·7%)	771·8 (652·9–891)	2·1% (-1·8 to 5·8%)
	Parkinson's disease	1517 (1259–1792)	158·4% (129·8 to 188·4%)	78·3 (65·2–92·4)	2·1% (-8·8 to 14·4%)
	Idiopathic epilepsy	15155 (4009–25681)	137·7% (-41·1 to 1001·8%)	301·3 (78·4–510·7)	2·2% (-74·3 to 374·9%)
	Multiple sclerosis	1498 (1205–1827)	268% (252·9 to 286·8%)	41·5 (33·6–50·2)	18·5% (13·9 to 24·3%)
	Migraine	736087 (619212–875301)	176·4% (169·4 to 183%)	15338·5 (13096·5–17900)	-1·2% (-1·4 to -0·9%)
	Tension-type headache	1094411 (920963–1287920)	172·3% (164·6 to 180·4%)	23661·8 (20360·3–27277·7)	0% (-0·2 to 0·1%)
	Motor neuron disease	120 (95–150)	175·2% (158·3 to 189·6%)	2·6 (2·1–3·2)	4% (0 to 7·7%)
	Other neurological disorders	29 (19–40)	154% (137·8 to 173·2%)	0·7 (0·5–1)	-0·5% (-1 to -0·1%)
	Headache disorders	1535980 (1377994–1696993)	173·5% (168·3 to 178·6%)	32770·6 (29756·4–35868·7)	-0·1% (-0·7 to 0·4%)
Oman	Meningitis	735 (580–945)	55·8% (26·2 to 90·2%)	17·1 (13·6–21·7)	-38·1% (-50·1 to -23·9%)
	Encephalitis	1233 (960–1509)	115·7% (99·9 to 131·1%)	28·9 (22·3–35·6)	-18·2% (-24·5 to -11·5%)
	Tetanus	1 (1–2)	44·3% (-13·4 to 94·5%)	0 (0–0)	-50·9% (-79·9 to -20·2%)
	Brain and central nervous system cancer	673 (380–900)	754·3% (329·7 to 1289·4%)	15·7 (9·1–20·4)	255·2% (76·4 to 453·4%)
	Stroke*	37722 (34606–41220)	167·7% (154·3 to 182%)	1525·6 (1399·9–1686·6)	-8·5% (-12·4 to -4·5%)
	Ischaemic stroke	29829 (26974–32984)	176·1% (164 to 190%)	1327·2 (1192·6–1493·3)	-2·9% (-7·6 to 1·9%)
	Intracerebral haemorrhage	7278 (6350–8298)	123·3% (111·7 to 136·7%)	215·3 (191·2–240·9)	-30·2% (-33·6 to -26·2%)
	Subarachnoid haemorrhage	2619 (2167–3072)	179·6% (153·1 to 197·5%)	58 (48·9–68·6)	-18·9% (-29·7 to -13·9%)
	Neurological disorders†	1575176 (1414954–1754275)	180·2% (168·3 to 193%)	32834·2 (29837·3–35871·3)	0·1% (-0·9 to 1%)

Table S12 Burden, mortality, incidence, and prevalence of neurological conditions in North Africa and Middle East countries

		All Ages		Age-standardised	
		Number	Percent change	Rate per 100,000	Percent change
**Measure**		2019	From 1990 to 2019	2019	From 1990 to 2019
Cause	Location	Mean (95% UI)	Mean (95% UI)	Mean (95% UI)	Mean (95% UI)
	Alzheimer's disease and other dementias	6746 (5733–7781)	143·4% (135·2 to 153·2%)	789·5 (668·6–913·6)	1·1% (-2·4 to 4·6%)
	Parkinson's disease	1362 (1125–1661)	213·3% (180·9 to 249%)	112·5 (92·9–135·2)	28% (14 to 44·8%)
	Idiopathic epilepsy	14018 (4253–22808)	153% (-24·4 to 892·3%)	339·1 (106·3–548·8)	20·3% (-65·1 to 372·1%)
	Multiple sclerosis	1483 (1206–1835)	352·9% (326·2 to 381·2%)	34·3 (28·3–41)	33·8% (27·2 to 41·9%)
	Migraine	721298 (607176–849217)	182·6% (166·6 to 197·2%)	14081·2 (12028–16515·9)	-1·5% (-1·8 to -1·2%)
	Tension-type headache	1138730 (948472–1361335)	181·2% (161·5 to 199·9%)	23692·5 (20423·5–27194·2)	-0·1% (-0·3 to 0%)
	Motor neuron disease	116 (91–145)	182·6% (159·7 to 207·3%)	2·3 (1·9–2·8)	2·7% (-1·3 to 6·9%)
	Other neurological disorders	27 (18–39)	153·2% (121·5 to 186·7%)	0·7 (0·5–0·9)	-0·2% (-0·5 to 0·1%)
	Headache disorders	1559952 (1397888–1741248)	180·6% (168·5 to 193·2%)	32016·9 (29024·5–35062·5)	-0·1% (-0·8 to 0·5%)
Qatar	Meningitis	2554 (2108–3120)	421·2% (327·5 to 527·1%)	89 (73·1–108·5)	-26·2% (-39·7 to -10·5%)
	Encephalitis	382 (297–476)	521·9% (468·5 to 576%)	13 (10–16·2)	-10·8% (-18·7 to -2·6%)
	Tetanus	1 (0–1)	454·9% (372·5 to 504·4%)	0 (0–0·1)	-11·4% (-30·2 to -2·7%)
	Brain and central nervous system cancer	586 (374–975)	2220% (1205·8 to 3531·3%)	24·3 (17–36·4)	230·5% (93·2 to 395%)
	Stroke*	22335 (20296–24525)	568·9% (532·5 to 607·4%)	1226·3 (1127·4–1327·2)	-22·2% (-25·6 to -18·9%)
	Ischaemic stroke	15886 (14141–17914)	593·8% (566·3 to 621·8%)	1000·1 (908·5–1098·6)	-20·9% (-24·8 to -17·5%)
	Intracerebral haemorrhage	5075 (4419–5756)	492·5% (460·9 to 527·5%)	209·1 (184·9–232·2)	-28·6% (-32·2 to -24·8%)
	Subarachnoid haemorrhage	2422 (1952–2857)	577% (448·3 to 625·5%)	73·6 (57·6–87·5)	-17·4% (-38·8 to -11·6%)
	Neurological disorders†	1029865 (924870–1150192)	608·8% (592 to 626%)	32245·1 (29313·4–35220·7)	-0·7% (-1·7 to 0·3%)
	Alzheimer's disease and other dementias	2696 (2251–3172)	685·6% (629·8 to 752·1%)	779·2 (654·8–905·3)	3·9% (0·1 to 8·2%)
	Parkinson's disease	750 (594–951)	811·6% (709·6 to 914·7%)	119·3 (98·3–142·9)	20·2% (6·9 to 36·3%)
	Idiopathic epilepsy	9532 (3056–15453)	484·5% (71·8 to 2028·4%)	397·3 (130·2–630·9)	-1·7% (-71 to 262·2%)
	Multiple sclerosis	1784 (1551–2001)	1183·7% (988·2 to 1408·8%)	56·1 (49·2–62·9)	54·1% (34 to 77·5%)
	Migraine	457439 (385311–540225)	596·8% (576 to 614·3%)	13295·3 (11309·2–15700·9)	-2·7% (-3·3 to -2·2%)
	Tension-type headache	756418 (622260–910774)	614·6% (587·5 to 637·7%)	23538·3 (20220·1–27033·6)	-0·5% (-0·9 to -0·2%)
	Motor neuron disease	93 (73–119)	631·5% (587·1 to 678·9%)	2·8 (2·3–3·5)	4·2% (-0·3 to 8·7%)
	Other neurological disorders	18 (12–26)	591·6% (536·1 to 640·5%)	0·7 (0·5–0·9)	0·3% (-0·3 to 1%)
	Headache disorders	1020526 (913853–1140900)	609·4% (592·3 to 625·7%)	31367·7 (28394·9–34336·2)	-0·8% (-1·5 to -0·2%)
Saudi Arabia	Meningitis	10756 (8555–13346)	81·2% (46·1 to 117·4%)	31·2 (25–38·3)	-29·1% (-43·3 to -14·4%)
	Encephalitis	6242 (4833–7806)	146·9% (128·5 to 165%)	17·2 (13·2–21·7)	-7·3% (-14·1 to -0·2%)
	Tetanus	17 (9–28)	-39·1% (-76·9 to 7%)	0 (0–0·1)	-71·4% (-87·9 to -51%)
	Brain and central nervous system cancer	6790 (4813–10153)	1355·6% (660·2 to 2697·9%)	17·9 (13·1–26·5)	444·1% (181·8 to 910·9%)
	Stroke*	480500 (442193–523176)	238·8% (214·5 to 262·7%)	1967·7 (1818·1–2143·5)	7·9% (-0·1 to 15·2%)
	Ischaemic stroke	321122 (290893–356283)	249·5% (214·9 to 281·5%)	1504·9 (1360·8–1680·6)	13·5% (1·9 to 23·8%)
	Intracerebral haemorrhage	153819 (128262–183295)	202·7% (182·9 to 223%)	493·7 (421·2–576·3)	-5·3% (-10·8 to 0%)
	Subarachnoid haemorrhage	23492 (19521–27716)	252·7% (233·5 to 271·3%)	58·5 (48·5–69·5)	-3·1% (-9·2 to 1%)
	Neurological disorders†	12255833 (11107362–13550299)	166·8% (153·5 to 180·3%)	31719·5 (28912–34577·5)	-1% (-3·6 to 1·9%)
	Alzheimer's disease and other dementias	67805 (58042–77811)	133·2% (122·1 to 147%)	770·3 (648·2–891·9)	0·8% (-2·6 to 5·3%)
	Parkinson's disease	13291 (11019–15910)	233·9% (195·1 to 279·3%)	107·6 (90·4–127·8)	27·7% (13·1 to 45%)
	Idiopathic epilepsy	187641 (56191–294049)	209·3% (-14·6 to 1107·1%)	563·3 (162·9–883·2)	41·8% (-60·1 to 462·6%)

Table S12 Burden, mortality, incidence, and prevalence of neurological conditions in North Africa and Middle East countries

		All Ages		Age-standardised	
		Number	Percent change	Rate per 100,000	Percent change
**Measure**		2019	From 1990 to 2019	2019	From 1990 to 2019
Cause	Location	Mean (95% UI)	Mean (95% UI)	Mean (95% UI)	Mean (95% UI)
	Multiple sclerosis	10757 (8342–13614)	364·9% (340 to 389·3%)	28·4 (22·4–35·2)	29·7% (23·9 to 35·9%)
	Migraine	5954561 (5057344–6988764)	173·9% (151·3 to 195·1%)	14325·3 (12266·3–16709·6)	-1·3% (-5·2 to 2·8%)
	Tension-type headache	8354742 (7033634–9767868)	162·3% (142·1 to 182·5%)	21757·9 (18684·4–24972·1)	-2·7% (-7 to 2%)
	Motor neuron disease	927 (738–1158)	166·9% (141·5 to 192·2%)	2·4 (2–2·9)	2·9% (-0·9 to 6·8%)
	Other neurological disorders	227 (150–330)	153·9% (116·9 to 194·2%)	0·7 (0·5–1)	0·3% (0·1 to 0·4%)
	Headache disorders	12076851 (10926929–13362048)	166·6% (153·2 to 180%)	30759·7 (27871·4–33712·7)	-1·5% (-4 to 1·2%)
Syrian Arab Republic	Meningitis	11671 (9722–14373)	-31·5% (-44·6 to -16·5%)	82·1 (68·7–101)	-45% (-55·3 to -32·4%)
	Encephalitis	4573 (3488–5719)	17·4% (9·8 to 25·1%)	31·5 (23·9–39·6)	-12·7% (-18·7 to -6·8%)
	Tetanus	9 (5–13)	-73·6% (-84·1 to -61·8%)	0·1 (0–0·1)	-72·3% (-81·1 to -63·1%)
	Brain and central nervous system cancer	2461 (1818–3349)	166·4% (57·3 to 321·2%)	17·5 (12·8–23·6)	109·7% (38·8 to 216·7%)
	Stroke*	199599 (184381–215112)	78·9% (71·6 to 86·2%)	1518·7 (1403·8–1637·5)	-13·9% (-17·3 to -10·5%)
	Ischaemic stroke	156735 (142891–171185)	94% (86·3 to 102%)	1224·1 (1107·6–1338·3)	-8·4% (-12·3 to -4·5%)
	Intracerebral haemorrhage	42182 (38241–46305)	34·9% (29·2 to 42·7%)	298·5 (270·2–327·4)	-30·1% (-33·2 to -26·4%)
	Subarachnoid haemorrhage	9383 (7415–11295)	61·6% (28·4 to 73·6%)	63·1 (50·8–74·9)	-15·5% (-32·3 to -9·8%)
	Neurological disorders†	4994027 (4532771–5479405)	37·4% (32·5 to 42·4%)	33730·3 (30733·5–36791·7)	1·1% (0·2 to 1·9%)
	Alzheimer's disease and other dementias	63678 (53661–73950)	117·3% (106·6 to 128·4%)	770·3 (653·1–892·3)	2·3% (-1·7 to 6·3%)
	Parkinson's disease	8349 (6932–9929)	160·5% (132·4 to 191·7%)	81·6 (67·8–97)	11·6% (-1·3 to 26·8%)
	Idiopathic epilepsy	39099 (11405–66196)	18·9% (-68·8 to 447·1%)	269·8 (79·6–454·1)	13·8% (-70·2 to 417·4%)
	Multiple sclerosis	5899 (4683–7200)	130·5% (117·5 to 143·2%)	42·1 (33·4–51·5)	23·8% (18·6 to 28·4%)
	Migraine	2353979 (2000112–2775087)	38·7% (31·9 to 46·1%)	15691 (13390·1–18304·1)	2·5% (2 to 2·9%)
	Tension-type headache	3507816 (2996722–4066438)	36·6% (28·8 to 44·8%)	23640·6 (20306·7–27263·7)	0% (-0·3 to 0·3%)
	Motor neuron disease	367 (297–452)	28·3% (17·7 to 41·1%)	2·5 (2·1–3·1)	0·4% (-3·4 to 4·6%)
	Other neurological disorders	98 (65–136)	36·5% (18·7 to 57·6%)	0·7 (0·5–1)	-0·1% (-0·4 to 0·3%)
	Headache disorders	4919044 (4454649–5399577)	37·1% (32 to 42·2%)	32983·9 (29977·1–36072·6)	1% (0·4 to 1·5%)
Tunisia	Meningitis	7524 (6184–9135)	5·1% (-14·9 to 28·5%)	63·4 (52–76·9)	-31·3% (-44·6 to -16·1%)
	Encephalitis	3109 (2368–3846)	41·2% (33·7 to 49·1%)	25·6 (19·5–31·7)	-11·4% (-16·1 to -6·4%)
	Tetanus	3 (2–5)	-13·1% (-43·5 to 11·7%)	0 (0–0)	-35·6% (-57·7 to -18·4%)
	Brain and central nervous system cancer	932 (602–1310)	239·2% (90·3 to 430·9%)	8·1 (5·2–11·4)	157·8% (49 to 292·8%)
	Stroke*	159928 (147442–173396)	184·9% (171 to 200·3%)	1260·8 (1162·6–1366)	22·1% (16·3 to 28·2%)
	Ischaemic stroke	141938 (129035–155655)	191·6% (178·5 to 206·8%)	1130·1 (1028·1–1240·4)	25·6% (20·1 to 31·5%)
	Intracerebral haemorrhage	16645 (14281–18819)	118·3% (97·6 to 140%)	128·2 (110–144·9)	2% (-6·9 to 12·1%)
	Subarachnoid haemorrhage	8671 (7021–10281)	119% (70·2 to 145·7%)	65·2 (53·3–76·9)	3·7% (-19 to 15·8%)
	Neurological disorders†	4068566 (3719308–4444144)	55·6% (49·5 to 61·5%)	33620·3 (30668–36658·7)	0·5% (-0·2 to 1·3%)
	Alzheimer's disease and other dementias	84642 (72004–97973)	215·4% (200·5 to 231·5%)	791·8 (670·4–916)	2·1% (-1·3 to 5·5%)
	Parkinson's disease	9312 (7834–10877)	208·5% (170·6 to 249·5%)	79·6 (66·9–93·2)	15·1% (1·8 to 30·4%)
	Idiopathic epilepsy	31108 (8948–50416)	47·6% (-64 to 534·6%)	279·7 (79·6–456·8)	16·3% (-72·2 to 395·8%)
	Multiple sclerosis	6567 (5389–7931)	170·3% (156·3 to 186·2%)	49·7 (40·8–59·8)	28·4% (22·4 to 34·2%)
	Migraine	1889805 (1626638–2191245)	54% (45·3 to 62·5%)	15455 (13195·2–18014·9)	0·4% (0·4 to 0·5%)

Table S12 Burden, mortality, incidence, and prevalence of neurological conditions in North Africa and Middle East countries

		All Ages		Age-standardised	
		Number	Percent change	Rate per 100,000	Percent change
**Measure**		2019	From 1990 to 2019	2019	From 1990 to 2019
Cause	Location	Mean (95% UI)	Mean (95% UI)	Mean (95% UI)	Mean (95% UI)
	Tension-type headache	2863725 (2464872–3296804)	55% (45·8 to 64·3%)	23657·2 (20350·7–27289)	0·1% (0 to 0·1%)
	Motor neuron disease	332 (269–408)	61·1% (48·2 to 75·5%)	2·7 (2·2–3·3)	4·1% (-0·5 to 8·1%)
	Other neurological disorders	83 (55–116)	64·4% (46·9 to 83%)	0·7 (0·5–1)	0% (-0·2 to 0·1%)
	Headache disorders	3985492 (3630553–4359580)	54·5% (48·3 to 60·4%)	32857·4 (29808·3–35970·3)	0·4% (-0·1 to 0·9%)
Türkiye	Meningitis	12559 (10364–15525)	-53·5% (-63 to -42·9%)	14·9 (12·3–18·4)	-69% (-75·3 to -62%)
	Encephalitis	23653 (18209–29257)	39·7% (31·6 to 48%)	27·2 (21–33·5)	-12·3% (-17·9 to -6·4%)
	Tetanus	43 (23–75)	-58·7% (-76·1 to -34·5%)	0·1 (0–0·1)	-74·2% (-86·2 to -54·7%)
	Brain and central nervous system cancer	23091 (11335–32329)	279·2% (97·5 to 516%)	29 (14·5–40)	173·1% (53 to 322·7%)
	Stroke*	1080379 (1002616–1164932)	103·3% (94·4 to 112·7%)	1213·6 (1124·7–1309·4)	-8·7% (-12·4 to -4·6%)
	Ischaemic stroke	838412 (764091–920762)	114·7% (104·8 to 124·9%)	956·3 (872·9–1049·7)	-5·2% (-9·5 to -0·7%)
	Intracerebral haemorrhage	212849 (190914–233873)	73·1% (65·5 to 81·5%)	234·6 (210·6–257·5)	-15·9% (-19·7 to -11·7%)
	Subarachnoid haemorrhage	74005 (62671–86497)	63·7% (55·6 to 73·9%)	78·4 (66·2–91·3)	-19·2% (-23·3 to -14·1%)
	Neurological disorders†	28053956 (25592370–30582393)	57·3% (50·3 to 63·9%)	31966·2 (29201·8–34866·9)	1% (-1·6 to 3·6%)
	Alzheimer's disease and other dementias	643123 (550258–741760)	198·7% (188·4 to 208·2%)	805·1 (686·6–926·2)	4·7% (1 to 8·1%)
	Parkinson's disease	72458 (59364–86774)	225% (194·8 to 259·1%)	86·9 (71–104·1)	22·3% (10·9 to 35·5%)
	Idiopathic epilepsy	373568 (110799–580823)	57·9% (-53·3 to 564·8%)	476 (143·4–741·4)	23·8% (-62·6 to 422·5%)
	Multiple sclerosis	45814 (43903–47892)	110·2% (105·2 to 115·9%)	48·7 (46·7–50·9)	2·5% (0·1 to 5%)
	Migraine	13296101 (11425706–15392415)	55·1% (45·5 to 65·2%)	14888·4 (12817·8–17240·8)	0% (-4·1 to 4·2%)
	Tension-type headache	19035787 (16409964–21823161)	57·2% (45·8 to 67·8%)	21726·3 (18745·7–24838·8)	1% (-2·9 to 5·2%)
	Motor neuron disease	3163 (2637–3746)	77·2% (64·6 to 89·4%)	3·6 (3–4·2)	10·2% (6·2 to 14·5%)
	Other neurological disorders	623 (416–872)	62·4% (44·5 to 81%)	0·7 (0·5–1)	0·8% (-5 to 7·6%)
	Headache disorders	27316884 (24809006–29912076)	56·1% (49·3 to 62·8%)	31032·8 (28267·3–33987·3)	0·7% (-1·8 to 3·4%)
United Arab Emirates	Meningitis	3196 (2562–3961)	333·9% (251·6 to 421·7%)	38·3 (31·2–47·4)	-21% (-36·2 to -5%)
	Encephalitis	1528 (1169–1905)	596·8% (542·3 to 656·7%)	15·3 (11·6–19·2)	23·9% (13·7 to 35·1%)
	Tetanus	6 (4–9)	59% (-27 to 218·5%)	0·2 (0·1–0·3)	-82·6% (-92·8 to -36·4%)
	Brain and central nervous system cancer	1511 (860–2264)	820·1% (481·5 to 1255·4%)	16·2 (9·5–22·6)	60·9% (-6·8 to 134·6%)
	Stroke*	136808 (125534–148340)	721·6% (675·3 to 767·1%)	2225·7 (2037·3–2436·3)	-7·5% (-11·6 to -3·6%)
	Ischaemic stroke	108862 (98572–119869)	779·7% (733·4 to 827·4%)	2005·1 (1808·4–2238·2)	-3·3% (-7·9 to 1·1%)
	Intracerebral haemorrhage	24981 (22321–28054)	509·4% (482·8 to 536·8%)	282·6 (253·7–312·3)	-30·3% (-33·4 to -27·3%)
	Subarachnoid haemorrhage	8045 (6737–9531)	644·4% (588·5 to 698·8%)	58 (49–68·4)	-15·4% (-23·2 to -10·8%)
	Neurological disorders†	3418888 (3055926–3814303)	475·3% (441·9 to 508·3%)	32631·3 (29615·7–35673·5)	-0·1% (-1·3 to 1%)
	Alzheimer's disease and other dementias	8095 (6634–9647)	636·5% (579·7 to 696·4%)	721·3 (602·2–842·2)	-0·1% (-3·5 to 3·2%)
	Parkinson's disease	3131 (2506–3880)	901% (789·1 to 1022·1%)	116 (97·6–138·8)	11·8% (0·6 to 23·6%)
	Idiopathic epilepsy	38016 (11150–60612)	306·1% (16 to 1404·2%)	491·9 (143–776·3)	-13·1% (-74·9 to 216·9%)
	Multiple sclerosis	3103 (2586–3694)	709·6% (610·3 to 804·1%)	23·8 (20·5–27·5)	-0·9% (-9·2 to 7·7%)
	Migraine	1521822 (1290266–1798697)	466·6% (428·2 to 509·6%)	13693·4 (11674·5–16104·4)	-0·7% (-1·1 to -0·3%)
	Tension-type headache	2512643 (2048289–3008501)	483·1% (427 to 537·9%)	23676 (20386·9–27204·5)	-0·2% (-0·5 to 0%)
	Motor neuron disease	297 (229–384)	472·2% (400 to 543·2%)	2·7 (2·2–3·2)	-0·5% (-4·4 to 4·5%)

Table S12 Burden, mortality, incidence, and prevalence of neurological conditions in North Africa and Middle East countries

		**All Ages**		**Age-standardised**	
		Number	Percent change	Rate per 100,000	Percent change
**Measure**		2019	From 1990 to 2019	2019	From 1990 to 2019
Cause	Location	Mean (95% UI)	Mean (95% UI)	Mean (95% UI)	Mean (95% UI)
	Other neurological disorders	63 (39–97)	496·2% (395·1 to 607%)	0·7 (0·5–0·9)	-0·1% (-0·6 to 0·4%)
	Headache disorders	3386525 (3025434–3793999)	477·2% (443·3 to 509%)	31755·5 (28744·2–34841·8)	0% (-0·6 to 0·6%)
Yemen	Meningitis	23455 (18740–29582)	71·4% (33·2 to 115·3%)	84·5 (67·6–106·3)	-29·7% (-45·4 to -11·7%)
	Encephalitis	9044 (6746–11507)	175·4% (154·6 to 195·5%)	32·5 (23·6–41·7)	9·1% (0·5 to 17·5%)
	Tetanus	43 (27–64)	-21·7% (-65·9 to 29·9%)	0·1 (0·1–0·2)	-58·7% (-79·3 to -40·8%)
	Brain and central nervous system cancer	1936 (1105–2806)	210% (60 to 521·8%)	6·8 (4·1–9·9)	47·8% (-18·6 to 161·2%)
	Stroke*	260915 (242029–280821)	174·8% (161·2 to 188·1%)	1609·4 (1480·2–1745·5)	0·8% (-4·2 to 5·7%)
	Ischaemic stroke	210911 (192387–230501)	215·7% (201·3 to 229·9%)	1355·2 (1220·3–1492·9)	15·9% (9·8 to 21·5%)
	Intracerebral haemorrhage	51230 (46578–56482)	82·4% (74·1 to 92·2%)	260 (236·1–286)	-37% (-39·8 to -33·6%)
	Subarachnoid haemorrhage	15013 (12661–17634)	138·3% (125·8 to 151·7%)	68·9 (58·1–81·4)	-17·6% (-21·9 to -12·6%)
	Neurological disorders†	9573564 (8555001–10612384)	163·6% (158·4 to 168·8%)	33439·2 (30457·6–36527·8)	0·1% (-0·6 to 0·9%)
	Alzheimer's disease and other dementias	64757 (54820–74749)	190·8% (179·6 to 203%)	752·3 (639·3–869·6)	-2·7% (-6 to 1·1%)
	Parkinson's disease	7214 (5874–8683)	205·1% (175·4 to 242%)	63·6 (51·9–76·4)	9·7% (-1·8 to 24·7%)
	Idiopathic epilepsy	72874 (17982–135412)	113·8% (-55·2 to 1082%)	220·1 (53·2–403·6)	-3·3% (-79·5 to 439·8%)
	Multiple sclerosis	5042 (4003–6214)	253·5% (235·8 to 271·5%)	23·3 (18·6–28·4)	22·9% (16·8 to 29·2%)
	Migraine	4554030 (3811556–5444867)	168·1% (161·7 to 173·9%)	15398·5 (13147·1–17961·2)	0% (-0·1 to 0·1%)
	Tension-type headache	6749015 (5678757–8023953)	162·7% (155·4 to 169·6%)	23663 (20359·2–27287·1)	0% (-0·1 to 0·1%)
	Motor neuron disease	470 (372–588)	153·1% (137·6 to 168·2%)	1·6 (1·3–2)	-0·8% (-4·5 to 3·2%)
	Other neurological disorders	180 (120–252)	140·3% (125·8 to 156·2%)	0·7 (0·5–1)	0·3% (0 to 0·5%)
	Headache disorders	9474680 (8476336–10502588)	163·9% (159·2 to 168·7%)	32765·4 (29726·4–35887·4)	0·2% (-0·3 to 0·7%)
Afghanistan	Meningitis	56549 (46438–68857)	71·4% (34·9 to 114·7%)	217·1 (178·4–261·1)	-41% (-53·6 to -26·1%)
	Encephalitis	19845 (14553–25342)	232·3% (204·9 to 263·8%)	69·4 (48·6–89·3)	4·4% (-4·1 to 13·2%)
	Tetanus	229 (161–311)	-14% (-47·3 to 48·1%)	0·6 (0·4–0·8)	-67·3% (-77·7 to -51·9%)
	Brain and central nervous system cancer	2524 (1341–4399)	183% (72 to 445·1%)	7·6 (4·3–13·6)	2·9% (-33 to 74·3%)
	Stroke*	281177 (260881–302740)	130·1% (117·5 to 141·2%)	1657·6 (1533·4–1779·5)	3·8% (-1·2 to 8%)
	Ischaemic stroke	208005 (188770–227010)	148·9% (135 to 162·7%)	1309·1 (1187·6–1435·3)	18·2% (12·4 to 23·2%)
	Intracerebral haemorrhage	74850 (67151–82123)	90·7% (79·7 to 103%)	355·1 (316·2–392)	-27% (-30·2 to -23·5%)
	Subarachnoid haemorrhage	16373 (13652–19482)	170·2% (152·7 to 189·5%)	66·9 (56·4–79·3)	-10·6% (-14·9 to -6·4%)
	Neurological disorders†	10980202 (9799203–12197339)	234·7% (227·3 to 241·4%)	33401·5 (30465·4–36481)	-0·4% (-1·3 to 0·4%)
	Alzheimer's disease and other dementias	54772 (45887–63556)	54·1% (47·3 to 60·4%)	735·4 (616·4–852·4)	-1·6% (-5·3 to 2·4%)
	Parkinson's disease	7352 (6145–8807)	61·8% (44·4 to 82·4%)	69·3 (58·2–82·4)	-3·3% (-12·9 to 8%)
	Idiopathic epilepsy	93796 (16679–183102)	204·7% (-33·9 to 1912·6%)	234·4 (41·5–453·3)	-9·9% (-80·5 to 489·9%)
	Multiple sclerosis	9996 (8248–12114)	259·8% (239·6 to 281·6%)	43·1 (36–51·2)	19·9% (13·6 to 25·7%)
	Migraine	5201896 (4319114–6245247)	240·2% (231·8 to 247·4%)	15315·6 (13076·7–17870·6)	-2·3% (-2·7 to -1·8%)
	Tension-type headache	7750880 (6436495–9231384)	234·6% (222·8 to 246·5%)	23693·8 (20386·8–27306·9)	0·4% (0·1 to 0·7%)
	Motor neuron disease	652 (521–815)	238·4% (219·8 to 260%)	1·9 (1·6–2·4)	0·2% (-4 to 4·2%)
	Other neurological disorders	210 (139–295)	207·4% (183·1 to 228·1%)	0·7 (0·5–1)	-0·3% (-0·8 to 0·2%)
	Headache disorders	10870791 (9667151–12101396)	236·4% (229·2 to 243%)	32717·2 (29722·6–35827·7)	-0·4% (-0·9 to 0·2%)

Table S12 Burden, mortality, incidence, and prevalence of neurological conditions in North Africa and Middle East countries

**Measure**		**All Ages**		**Age-standardised**	
		Number	Percent change	Rate per 100,000	Percent change
		2019	From 1990 to 2019	2019	From 1990 to 2019
Cause	Location	Mean (95% UI)	Mean (95% UI)	Mean (95% UI)	Mean (95% UI)
Sudan	Meningitis	16303 (13100–20405)	-55·8% (-64·7 to -43·9%)	44·1 (35·4–54·9)	-79% (-83·3 to -73·3%)
	Encephalitis	10509 (7968–13256)	88·7% (75 to 106%)	28·9 (21·5–36·5)	-12·4% (-18·8 to -4·3%)
	Tetanus	43 (27–65)	-41·6% (-69·4 to -8·5%)	0·1 (0·1–0·2)	-65·6% (-79·8 to -52·8%)
	Brain and central nervous system cancer	3651 (2077–5305)	166·2% (16·8 to 550·4%)	9·1 (5·4–13·2)	47% (-25·8 to 197·7%)
	Stroke*	398038 (367084–427930)	124·6% (114·5 to 134·3%)	1785·8 (1645·2–1936·9)	8·2% (3·7 to 12·8%)
	Ischaemic stroke	325721 (297455–355521)	153·9% (143·4 to 165%)	1520·7 (1375·6–1681·8)	23·8% (18·5 to 29·5%)
	Intracerebral haemorrhage	74732 (67854–82225)	54·2% (47 to 62·2%)	279·6 (253·9–307·1)	-31·9% (-34·9 to -28·8%)
	Subarachnoid haemorrhage	20074 (16980–23622)	87·1% (77·4 to 98·7%)	67·6 (57·5–79·7)	-19·5% (-23·9 to -14·5%)
	Neurological disorders†	12655355 (11340532–13986720)	119·4% (116·7 to 122·3%)	33434·1 (30397·1–36506·1)	0·1% (-0·7 to 0·9%)
	Alzheimer's disease and other dementias	100531 (85731–115957)	111·9% (101·4 to 122%)	746·2 (630·2–861·7)	0·2% (-3·3 to 3·8%)
	Parkinson's disease	11401 (9527–13651)	102·4% (78·9 to 129·8%)	69·7 (58·3–83·4)	2·1% (-9·9 to 16·1%)
	Idiopathic epilepsy	104147 (25345–187318)	110·9% (-49·8 to 1029·8%)	245·5 (60·3–444·7)	8·5% (-73·9 to 482%)
	Multiple sclerosis	6715 (5309–8260)	180% (166·4 to 195%)	23·1 (18·5–27·9)	23·9% (18·2 to 30·7%)
	Migraine	6032231 (5066737–7200217)	121·7% (118·9 to 124·1%)	15412 (13164·6–17974·7)	-0·2% (-0·3 to -0·1%)
	Tension-type headache	8893989 (7481341–10475764)	118·3% (115·4 to 121·4%)	23598·8 (20291–27242·1)	-0·1% (-0·2 to -0·1%)
	Motor neuron disease	637 (502–799)	123·4% (113·4 to 134·2%)	1·7 (1·4–2·1)	4·1% (0·4 to 8·2%)
	Other neurological disorders	236 (158–328)	106·7% (99·4 to 114·7%)	0·7 (0·5–1)	0% (-0·1 to 0·2%)
	Headache disorders	12508726 (11209326–13860164)	119·5% (117·3 to 122%)	32741·2 (29701·9–35840·2)	0·1% (-0·5 to 0·6%)
**YLDs (Years Lived with Disability)**					
Global	Meningitis	683333 (480874–924320)	-19·2% (-22·5 to -15·4%)	9 (6·3–12·2)	-41·2% (-43·6 to -38·4%)
	Encephalitis	482436 (343282–647343)	5·2% (0·7 to 9·8%)	6·1 (4·4–8·3)	-28·8% (-31·6 to -25·6%)
	Tetanus	1745 (1022–2663)	-76·5% (-81·8 to -71%)	0 (0–0)	-81·7% (-85·3 to -77·9%)
	Brain and central nervous system cancer	129402 (83870–174962)	118·1% (34·2 to 156·9%)	1·6 (1·1–2·2)	29·5% (-18·9 to 52%)
	Stroke*	17741742 (12759419–22587415)	88·9% (85·3 to 92·7%)	218·1 (156·7–277)	-4·7% (-6·1 to -3·3%)
	Ischaemic stroke	13128534 (9349917–16930382)	102% (97·4 to 106·9%)	162·3 (115·8–209·9)	0·1% (-1·8 to 2%)
	Intracerebral haemorrhage	3266283 (2333985–4173735)	57·2% (54·9 to 59·5%)	39·4 (28·1–50·2)	-17·7% (-19·2 to -16·1%)
	Subarachnoid haemorrhage	1346925 (964157–1782242)	65·4% (60·7 to 69·6%)	16·4 (11·7–21·6)	-12·9% (-15·2 to -11·2%)
	Neurological disorders†	65625969 (28649298–122710043)	64·9% (58·3 to 81·4%)	831·3 (365·8–1542·4)	2·9% (-0·7 to 6·8%)
	Alzheimer's disease and other dementias	7417069 (5226780–9927706)	164·7% (158·7 to 171%)	98·9 (69·5–132·6)	5·5% (4 to 6·8%)
	Parkinson's disease	1210093 (841166–1640683)	154·7% (149·4 to 160·9%)	15·1 (10·4–20·3)	16·2% (13·5 to 19%)
	Idiopathic epilepsy	7740804 (4810323–11216664)	43·7% (18·7 to 76·3%)	101·1 (63·1–146·8)	1·4% (-15·4 to 23·2%)
	Multiple sclerosis	451204 (320712–591541)	71·3% (65·3 to 77·1%)	5·5 (3·9–7·1)	-5·8% (-8·6 to -2·9%)
	Migraine	42077666 (6418383–95645211)	56·6% (52·6 to 62·1%)	525·5 (78·8–1194)	1·5% (-4·4 to 3·3%)
	Tension-type headache	4541689 (1395546–14981336)	57·8% (45·1 to 65·9%)	56·2 (17–188·5)	-2·5% (-5·4 to 1·1%)
	Motor neuron disease	57068 (39982–76338)	68·8% (62·4 to 75·6%)	0·7 (0·5–1)	1·9% (0·6 to 3·3%)
	Other neurological disorders	2130376 (1392089–3016404)	93·3% (65·4 to 128·5%)	28·3 (18·4–40·4)	40·6% (20·9 to 64·7%)

Table S12 Burden, mortality, incidence, and prevalence of neurological conditions in North Africa and Middle East countries

		**All Ages**		**Age-standardised**	
		Number	Percent change	Rate per 100,000	Percent change
**Measure**		2019	From 1990 to 2019	2019	From 1990 to 2019
Cause	Location	Mean (95% UI)	Mean (95% UI)	Mean (95% UI)	Mean (95% UI)
	Headache disorders	46619355 (9772903–100161726)	56·7% (52·4 to 62·1%)	581·8 (119·6–1255·6)	1·1% (-4·2 to 2·9%)
North Africa and Middle East	Meningitis	36232 (25118–48690)	17·1% (9·2 to 26·8%)	6 (4·2–8)	-34·6% (-39 to -29·4%)
	Encephalitis	19799 (13819–26804)	84·9% (76·6 to 93·9%)	3·2 (2·2–4·3)	-4·1% (-8·1 to 0·4%)
	Tetanus	27 (15–42)	-70·2% (-81·9 to -50·5%)	0 (0–0)	-80·9% (-87·4 to -70·7%)
	Brain and central nervous system cancer	10647 (6244–14986)	203·1% (78·6 to 311·3%)	1·9 (1·1–2·7)	55·6% (-2·6 to 105·3%)
	Stroke*	1113741 (812092–1400797)	140·2% (135·3 to 145·3%)	239·4 (176·1–301·4)	-0·7% (-2·6 to 1·2%)
	Ischaemic stroke	877321 (639827–1114121)	157·6% (151·5 to 164·4%)	195·6 (144·4–249·1)	5·2% (2·8 to 7·6%)
	Intracerebral haemorrhage	182217 (129142–229566)	88·9% (82·6 to 95·8%)	34·4 (24·4–43·5)	-21·5% (-24·2 to -18·4%)
	Subarachnoid haemorrhage	54203 (37875–72271)	103·1% (83·8 to 116·5%)	9·3 (6·6–12·4)	-17·9% (-26·2 to -12·2%)
	Neurological disorders†	5447847 (2227535–10439230)	99·6% (83·8 to 121·6%)	935 (419·6–1737·7)	0·2% (-6·5 to 8·3%)
	Alzheimer's disease and other dementias	352185 (248064–474309)	187·7% (180 to 195·6%)	112·8 (79·1–151·9)	2·8% (0·9 to 4·6%)
	Parkinson's disease	44505 (30653–60492)	197·3% (182·1 to 214·4%)	11·7 (8–15·7)	14·8% (9·2 to 21·2%)
	Idiopathic epilepsy	603114 (342286–938664)	47·5% (-1 to 119%)	101 (57·3–156·5)	-9·3% (-39·8 to 33·8%)
	Multiple sclerosis	57687 (40798–76752)	169·3% (156·5 to 183·1%)	10 (7·1–13·3)	10·9% (6·1 to 16·3%)
	Migraine	3793207 (645291–8665809)	102·1% (93·2 to 125·1%)	601·4 (107–1371·8)	0% (-1·6 to 1·6%)
	Tension-type headache	416595 (138258–1196825)	115·5% (84·9 to 132·3%)	68·1 (22·8–195·5)	1% (-9·5 to 8·7%)
	Motor neuron disease	3308 (2269–4604)	103·3% (92·1 to 113·8%)	0·5 (0·4–0·8)	3·6% (2 to 5·1%)
	Other neurological disorders	177246 (106129–265092)	98·6% (41·9 to 177·9%)	29·4 (18–43·9)	25·5% (-8·9 to 71%)
	Headache disorders	4209802 (990143–9068319)	103·3% (94·5 to 123·7%)	669·6 (159·1–1431·3)	0·1% (-2·4 to 2·4%)
Algeria	Meningitis	2230 (1544–3012)	32·6% (17·8 to 48%)	5·4 (3·7–7·3)	-20·4% (-29·4 to -11·2%)
	Encephalitis	1151 (800–1568)	94·5% (76·8 to 113·5%)	2·7 (1·9–3·7)	8·6% (-1·1 to 18·5%)
	Tetanus	0 (0–1)	-46·5% (-76·6 to -2·4%)	0 (0–0)	-65·9% (-84·6 to -41·1%)
	Brain and central nervous system cancer	363 (193–552)	183·7% (52·9 to 298·7%)	0·9 (0·5–1·4)	59% (-14·1 to 120·7%)
	Stroke*	85024 (62206–107505)	128·1% (116·1 to 140·6%)	242·8 (178·6–307·3)	-10·6% (-14·9 to -6%)
	Ischaemic stroke	69844 (50800–89336)	151·1% (135·5 to 167·4%)	204·2 (149·2–261·1)	-3·1% (-8·5 to 2·6%)
	Intracerebral haemorrhage	11224 (7842–14388)	50·3% (35·3 to 65·8%)	29·2 (20·5–37·5)	-39·8% (-45·5 to -34%)
	Subarachnoid haemorrhage	3955 (2721–5400)	98·4% (53·6 to 135·2%)	9·5 (6·5–13)	-23·7% (-41·8 to -9·3%)
	Neurological disorders†	374247 (157441–723907)	84·9% (44 to 148%)	928·2 (416·8–1744·2)	-2·3% (-20 to 19·5%)
	Alzheimer's disease and other dementias	27351 (19435–36980)	244·1% (216·3 to 275·2%)	113·1 (79·6–152·1)	1% (-3·2 to 5·2%)
	Parkinson's disease	3441 (2387–4723)	213·3% (165·9 to 268·6%)	11·4 (7·9–15·5)	9·2% (-6·4 to 25·7%)
	Idiopathic epilepsy	38664 (8737–80014)	15·9% (-75·9 to 408·9%)	94·6 (21·2–196·2)	-22·9% (-83·9 to 237%)
	Multiple sclerosis	4546 (3054–6303)	218·1% (167·6 to 278%)	10·8 (7·3–14·9)	25·3% (6·4 to 48·3%)
	Migraine	259963 (44334–590017)	89% (76·7 to 120·2%)	602·5 (103·9–1372·1)	-0·2% (-3·2 to 3·1%)
	Tension-type headache	28460 (9650–79524)	106·7% (73·1 to 140·3%)	66·7 (22·6–189)	0·3% (-10·2 to 7·9%)
	Motor neuron disease	212 (145–296)	89·9% (74·6 to 104·9%)	0·5 (0·3–0·7)	2% (-1·6 to 5·7%)
	Other neurological disorders	11609 (3995–22111)	61·2% (-48·7 to 478·3%)	28·6 (10·1–54·1)	12·4% (-63 to 244%)
	Headache disorders	288424 (67480–615898)	90·7% (78·3 to 119·7%)	669·2 (155·7–1442·3)	-0·1% (-3·5 to 3·1%)
Bahrain	Meningitis	58 (40–80)	69·2% (45·3 to 101·3%)	3·9 (2·7–5·4)	-44·7% (-51·7 to -35·7%)
	Encephalitis	30 (21–42)	187·6% (156·4 to 223·9%)	1·9 (1·3–2·6)	-7·3% (-17 to 4·6%)
	Tetanus	0 (0–0)	136·8% (87 to 169·8%)	0 (0–0)	-11·7% (-35·5 to 0%)
	Brain and central nervous system cancer	21 (11–31)	510·4% (260·3 to 799·8%)	1·5 (0·9–2·2)	50·6% (-10·4 to 121·5%)
	Stroke*	2076 (1491–2641)	275·8% (251·4 to 302·4%)	173·8 (126–219·2)	-25·9% (-30 to -21·8%)

Table S12 Burden, mortality, incidence, and prevalence of neurological conditions in North Africa and Middle East countries					
		**All Ages**		**Age-standardised**	
		Number	Percent change	Rate per 100,000	Percent change
**Measure**		2019	From 1990 to 2019	2019	From 1990 to 2019
Cause	Location	Mean (95% UI)	Mean (95% UI)	Mean (95% UI)	Mean (95% UI)
	Ischaemic stroke	1541 (1097–1972)	287·4% (256·8 to 320·9%)	139·9 (101·5–178·9)	-25·5% (-30·1 to -20·7%)
	Intracerebral haemorrhage	387 (268–510)	217·3% (175 to 264·1%)	26·2 (18·1–34)	-32·1% (-39·1 to -24·6%)
	Subarachnoid haemorrhage	148 (100–202)	353·6% (288·7 to 432·3%)	7·7 (5·2–10·4)	-7·2% (-19·9 to 7·6%)
	Neurological disorders†	13285 (5189–25427)	204·4% (128·4 to 304·6%)	923·6 (424·3–1686·2)	-4·4% (-29 to 24·2%)
	Alzheimer's disease and other dementias	492 (346–665)	440% (403·7 to 480·9%)	113·7 (80·5–151·6)	2·9% (-1 to 7·3%)
	Parkinson's disease	99 (64–142)	494·3% (387·6 to 617·3%)	13 (8·8–17·7)	11·2% (-7·8 to 34·3%)
	Idiopathic epilepsy	1549 (373–3291)	93·9% (-54·7 to 821·7%)	125·4 (29·5–263·3)	-27·4% (-82·5 to 245·2%)
	Multiple sclerosis	154 (101–218)	464·7% (361·2 to 595·2%)	8·4 (5·5–11·6)	25·3% (6·2 to 47·9%)
	Migraine	9458 (1762–21196)	219·3% (197 to 279·3%)	564·5 (100·5–1278·1)	-0·7% (-3·9 to 2·8%)
	Tension-type headache	1117 (386–3084)	254·2% (197·7 to 330·7%)	64·6 (21·3–189·3)	-0·3% (-10·7 to 7·7%)
	Motor neuron disease	10 (7–14)	235·2% (197·8 to 274·8%)	0·6 (0·4–0·8)	3·7% (-0·5 to 7·9%)
	Other neurological disorders	405 (139–807)	168·8% (-11·7 to 924·4%)	33·5 (10·8–66·6)	8·3% (-65·7 to 260·8%)
	Headache disorders	10575 (2641–22348)	222·7% (200·3 to 275·9%)	629 (149·4–1360·3)	-0·6% (-3·9 to 2·8%)
Egypt	Meningitis	6334 (4295–8627)	24·3% (7·7 to 42·2%)	6·3 (4·3–8·6)	-29·9% (-39 to -20·2%)
	Encephalitis	3213 (2215–4397)	62·8% (49 to 78·3%)	3·2 (2·2–4·4)	-13·2% (-20·2 to -5·3%)
	Tetanus	4 (2–8)	-65·9% (-81·4 to -38·2%)	0 (0–0)	-79·4% (-88 to -61·9%)
	Brain and central nervous system cancer	1037 (634–1565)	164·1% (48 to 286·3%)	1·2 (0·7–1·8)	39·2% (-12·6 to 102%)
	Stroke*	193859 (141703–245188)	149·5% (136·2 to 163%)	282·6 (206·4–356·5)	18% (11·8 to 24·2%)
	Ischaemic stroke	158984 (115787–203081)	179·1% (161·3 to 197·2%)	239·8 (175·1–307·5)	30% (22·2 to 37·9%)
	Intracerebral haemorrhage	27020 (19059–34612)	68·2% (50·9 to 86·9%)	33·8 (24·2–43·2)	-22·2% (-29·7 to -14%)
	Subarachnoid haemorrhage	7856 (5370–10558)	68·8% (40 to 96·8%)	9 (6·1–12·1)	-21·3% (-34·6 to -8·4%)
	Neurological disorders†	851984 (300357–1665555)	94·8% (48·2 to 146·5%)	931·2 (371·4–1745·9)	2% (-16·8 to 23·4%)
	Alzheimer's disease and other dementias	41201 (29329–55508)	119·1% (108·5 to 130%)	110 (76·8–148·3)	4·8% (0·4 to 9·7%)
	Parkinson's disease	6366 (4279–8932)	147·6% (106·3 to 196·4%)	12 (8·2–16·5)	16·5% (-1·6 to 37·1%)
	Idiopathic epilepsy	87277 (21019–178024)	54·2% (-65·2 to 647·7%)	86·5 (20·9–177·8)	-9·2% (-79·5 to 337·3%)
	Multiple sclerosis	4792 (3178–6761)	160·1% (121·2 to 202·7%)	5·5 (3·7–7·8)	22·6% (4·4 to 43·5%)
	Migraine	619621 (96677–1426392)	98·2% (84·3 to 113·8%)	621·1 (104–1420·2)	1·9% (-5·1 to 9·1%)
	Tension-type headache	65173 (20989–196744)	100·8% (75 to 120·6%)	69·1 (22·6–207·8)	1·4% (-10·1 to 10·8%)
	Motor neuron disease	454 (308–632)	96·4% (87·1 to 105·5%)	0·5 (0·3–0·7)	4·2% (0·6 to 8·2%)
	Other neurological disorders	27101 (8400–51840)	120·5% (-34·1 to 711%)	26·4 (9·5–48·8)	30·6% (-56·7 to 292·5%)
	Headache disorders	684794 (147091–1491960)	98·4% (84·9 to 112·8%)	690·3 (156·4–1488·7)	1·9% (-4·8 to 8·6%)
Iran (Islamic Republic of)	Meningitis	5917 (4098–7913)	4·6% (-5·1 to 15·9%)	7 (4·9–9·3)	-28·1% (-34 to -21·5%)
	Encephalitis	2444 (1678–3343)	47·4% (37·4 to 57·9%)	2·8 (1·9–3·8)	-8·6% (-14·1 to -3·1%)
	Tetanus	1 (0–1)	-79% (-90 to -59·4%)	0 (0–0)	-82·7% (-90·7 to -70·6%)
	Brain and central nervous system cancer	2379 (1087–3523)	174% (49·3 to 259·1%)	2·9 (1·3–4·3)	62·6% (-5·7 to 104·8%)
	Stroke*	148037 (106315–188599)	125·2% (116·8 to 134%)	196·4 (140·3–251·9)	-12·7% (-15·5 to -10%)
	Ischaemic stroke	124907 (88889–160270)	130% (120·3 to 141·2%)	169·1 (121·1–219·4)	-12·7% (-15·8 to -9·5%)
	Intracerebral haemorrhage	16457 (11518–21066)	93% (84 to 102·4%)	19·9 (14·1–25·6)	-15·1% (-19·1 to -10·9%)
	Subarachnoid haemorrhage	6673 (4684–9048)	129·1% (116·4 to 142·7%)	7·3 (5·2–9·9)	-6·6% (-10·8 to -2·3%)
	Neurological disorders†	833316 (360833–1572932)	76·6% (61·6 to 100%)	975·8 (451·6–1800)	-0·6% (-6·7 to 6·2%)
	Alzheimer's disease and other dementias	67291 (47273–90622)	325·9% (297·8 to 357·1%)	113·9 (80–154)	2·2% (0 to 4%)
	Parkinson's disease	8067 (5467–11099)	255·1% (228·5 to 282·5%)	12 (8·1–16·6)	13·2% (8·7 to 17·3%)
	Idiopathic epilepsy	79851 (45748–122172)	5·9% (-27·2 to 51·5%)	100 (57·2–152·2)	-14·4% (-41·4 to 21·7%)

Table S12 Burden, mortality, incidence, and prevalence of neurological conditions in North Africa and Middle East countries

Measure		All Ages		Age-standardised	
		Number	Percent change	Rate per 100,000	Percent change
		2019	From 1990 to 2019	2019	From 1990 to 2019
Cause	Location	Mean (95% UI)	Mean (95% UI)	Mean (95% UI)	Mean (95% UI)
	Multiple sclerosis	11971 (8380–15879)	133·3% (117·5 to 150·8%)	13 (9·1–17·2)	-4·8% (-10·5 to 1·6%)
	Migraine	571655 (107029–1282201)	77·6% (63·7 to 117·4%)	628·3 (115·2–1411·2)	-0·3% (-3·6 to 4·1%)
	Tension-type headache	70182 (23015–207136)	103·8% (70·9 to 131·5%)	77·6 (24·9–236·4)	5·2% (-5·7 to 15·9%)
	Motor neuron disease	541 (370–758)	86·9% (70·6 to 103·5%)	0·6 (0·4–0·9)	6·6% (4·9 to 8·4%)
	Other neurological disorders	23759 (14285–35287)	40·7% (3·5 to 90·8%)	30·4 (18·3–45·5)	19·7% (-12·2 to 57·7%)
	Headache disorders	641837 (160733–1355804)	80·2% (66·5 to 117·2%)	705·8 (173·2–1503·1)	0·3% (-3·3 to 5·8%)
Iraq	Meningitis	2459 (1672–3320)	67·1% (49·1 to 87·9%)	5·8 (4–7·8)	-30·1% (-37·7 to -21·7%)
	Encephalitis	1335 (933–1813)	149·5% (130·6 to 169·4%)	3·2 (2·2–4·3)	-4·5% (-11·4 to 2·7%)
	Tetanus	1 (1–2)	-64·2% (-85·1 to -12·2%)	0 (0–0)	-78·7% (-89·9 to -58·3%)
	Brain and central nervous system cancer	1033 (636–1504)	318·3% (111·4 to 590·4%)	2·9 (1·8–4·2)	64·4% (-13·6 to 160·6%)
	Stroke*	78443 (56662–98553)	167% (154·2 to 181·1%)	306·4 (223·5–385·2)	-8·7% (-13·3 to -3·7%)
	Ischaemic stroke	60290 (43323–76438)	169·8% (154·6 to 186·7%)	249·5 (181·9–315·5)	-6·3% (-11·5 to -0·1%)
	Intracerebral haemorrhage	15125 (10610–19282)	161·7% (136·8 to 188·3%)	48·4 (34·2–61·7)	-16·2% (-23·4 to -8·3%)
	Subarachnoid haemorrhage	3028 (2077–4156)	141·8% (92·3 to 183·1%)	8·6 (5·9–11·8)	-25·3% (-44·6 to -11·8%)
	Neurological disorders†	353613 (132455–707045)	168·9% (104·9 to 239·6%)	913 (394·4–1730·9)	-0·1% (-18·2 to 20·6%)
	Alzheimer's disease and other dementias	16645 (11832–22420)	167·2% (154·7 to 181·3%)	110·5 (78·3–149·5)	6·3% (2·1 to 10·9%)
	Parkinson's disease	2072 (1426–2825)	200% (156·4 to 252·7%)	10·5 (7·3–14·2)	6·8% (-7·7 to 24·8%)
	Idiopathic epilepsy	39664 (8430–82092)	95·1% (-53·5 to 838·5%)	92·3 (20–189·2)	-15·6% (-80·5 to 286·1%)
	Multiple sclerosis	3320 (2197–4752)	273% (215 to 339·5%)	9·7 (6·4–13·7)	19·1% (0·9 to 40·6%)
	Migraine	253176 (40135–580495)	181·6% (170·8 to 206·2%)	595·4 (102·8–1355·6)	0·3% (-2·8 to 3·2%)
	Tension-type headache	26343 (8663–77009)	193·4% (151·1 to 230·5%)	66·2 (22·1–190·3)	0·7% (-11·1 to 9·1%)
	Motor neuron disease	218 (148–303)	170·7% (154·7 to 186·6%)	0·5 (0·4–0·7)	2·6% (-1·2 to 6·4%)
	Other neurological disorders	12176 (3620–23961)	175·6% (-13·8 to 915·1%)	27·9 (9·7–53·7)	24·2% (-56·8 to 272·3%)
	Headache disorders	279519 (60086–621233)	182·7% (170·9 to 204·1%)	661·6 (153–1427·4)	0·3% (-3·3 to 3·5%)
Jordan	Meningitis	997 (660–1408)	178·2% (129·5 to 241·1%)	8·6 (5·8–12)	-11·4% (-25·3 to 7·1%)
	Encephalitis	356 (246–484)	197·7% (169·8 to 217·4%)	3·1 (2·1–4·2)	-9·9% (-17·7 to -4%)
	Tetanus	0 (0–0)	11·7% (-38·3 to 76·3%)	0 (0–0)	-54·7% (-72·7 to -35·8%)
	Brain and central nervous system cancer	174 (104–252)	509% (285·8 to 731·1%)	1·7 (1·1–2·5)	62·9% (0·1 to 124·2%)
	Stroke*	20515 (14870–25763)	256·2% (235·6 to 277·3%)	284·5 (207·6–358·1)	-23·7% (-28·5 to -19·2%)
	Ischaemic stroke	16657 (12027–21062)	258·7% (234·4 to 283·5%)	243 (176·8–308·3)	-23·7% (-29·1 to -18·5%)
	Intracerebral haemorrhage	2747 (1923–3585)	216·8% (181·1 to 254·4%)	30·7 (21·8–39·7)	-29·4% (-36·7 to -21·5%)
	Subarachnoid haemorrhage	1111 (765–1522)	345% (283·5 to 415·2%)	10·8 (7·5–14·5)	-1·4% (-14·7 to 14·6%)
	Neurological disorders†	97636 (36331–192445)	245·4% (173·6 to 348·2%)	907·9 (394·5–1704·5)	-0·8% (-17·2 to 18·5%)
	Alzheimer's disease and other dementias	4479 (3147–6083)	431% (401·7 to 459·4%)	111·6 (78·2–151·8)	3·7% (-1 to 8·3%)
	Parkinson's disease	600 (414–838)	425% (342·9 to 517%)	10·9 (7·5–15·1)	2·8% (-13·4 to 19·7%)
	Idiopathic epilepsy	10477 (2356–23076)	154·2% (-40·2 to 939·2%)	88·5 (20·3–192·5)	-12·8% (-79·2 to 245·1%)
	Multiple sclerosis	1198 (774–1659)	343·4% (251·7 to 443·3%)	12·1 (7·9–16·6)	-1% (-21·1 to 20%)
	Migraine	70088 (11911–160288)	250·2% (230·2 to 298·6%)	590·3 (104–1339·1)	-0·8% (-4 to 2·5%)
	Tension-type headache	7406 (2514–21769)	276·6% (221·8 to 332·8%)	66·2 (22·7–191·4)	0·1% (-10·9 to 8·3%)
	Motor neuron disease	65 (45–91)	252·1% (224·4 to 277·9%)	0·6 (0·4–0·8)	3% (-1·7 to 7·2%)
	Other neurological disorders	3322 (1084–6819)	263·1% (14·7 to 1066·3%)	27·8 (10·1–54·6)	26·7% (-54·8 to 240·1%)
	Headache disorders	77495 (17367–171586)	252·5% (233·2 to 298·1%)	656·4 (153·8–1428·7)	-0·7% (-3·9 to 2·5%)
Kuwait	Meningitis	160 (111–217)	109·8% (85·4 to 135·6%)	3·6 (2·5–4·9)	-19·5% (-29 to -9·2%)
	Encephalitis	82 (56–113)	178·7% (147·6 to 207·1%)	1·7 (1·2–2·4)	1·8% (-9 to 12·2%)

Table S12 Burden, mortality, incidence, and prevalence of neurological conditions in North Africa and Middle East countries

		All Ages		Age-standardised	
		Number	Percent change	Rate per 100,000	Percent change
**Measure**		2019	From 1990 to 2019	2019	From 1990 to 2019
Cause	Location	Mean (95% UI)	Mean (95% UI)	Mean (95% UI)	Mean (95% UI)
	Tetanus	0 (0–0)	123% (90·9 to 149·1%)	0 (0–0)	-9·2% (-21·4 to 1·3%)
	Brain and central nervous system cancer	73 (44–111)	380·1% (193·4 to 556·2%)	1·9 (1·2–2·9)	81·7% (11·4 to 148·3%)
	Stroke*	6281 (4549–7999)	262·6% (237·2 to 288·5%)	189·4 (138·2–238)	-6·8% (-12·1 to -1·3%)
	Ischaemic stroke	4675 (3370–5942)	265·9% (237·1 to 297·9%)	153·2 (111·1–193·1)	-7·5% (-13·9 to -1·4%)
	Intracerebral haemorrhage	1061 (734–1403)	239·5% (187 to 294·7%)	25·9 (18·2–33·8)	-6·3% (-17·3 to 4·8%)
	Subarachnoid haemorrhage	545 (377–739)	283·9% (223·4 to 357·4%)	10·3 (7·2–13·9)	3% (-11·2 to 20·2%)
	Neurological disorders†	41195 (15568–81016)	185·4% (126·4 to 264·3%)	916·1 (399·6–1722·5)	1·2% (-18·1 to 24·3%)
	Alzheimer's disease and other dementias	2044 (1442–2740)	406·6% (380·4 to 434·2%)	116·3 (81·6–156·8)	0·6% (-3·7 to 5·4%)
	Parkinson's disease	232 (156–329)	311·7% (238·6 to 396·9%)	10·1 (6·8–13·9)	-9·2% (-26·3 to 11·5%)
	Idiopathic epilepsy	4048 (964–9024)	92·1% (-51·6 to 741·6%)	103·4 (23·9–231)	-15·2% (-78·7 to 270·3%)
	Multiple sclerosis	701 (466–983)	468% (369·2 to 600·9%)	13·5 (9·1–18·5)	56·2% (32·3 to 85·2%)
	Migraine	29758 (5159–67375)	190·2% (171·1 to 225·4%)	578·3 (99·7–1315·1)	3·2% (-2·3 to 8·6%)
	Tension-type headache	3205 (1084–8967)	204·5% (159·1 to 260·4%)	62·8 (20·8–186·4)	1·5% (-10·4 to 11·9%)
	Motor neuron disease	32 (22–45)	166·7% (143·3 to 192·9%)	0·7 (0·5–0·9)	-4·2% (-9·8 to 1·1%)
	Other neurological disorders	1175 (429–2398)	176·9% (-1 to 807·8%)	31·2 (11·4–65)	25% (-53·9 to 247·1%)
	Headache disorders	32963 (7555–71305)	191·5% (173·1 to 225·4%)	641·1 (146·8–1408)	3·1% (-2·3 to 7·9%)
Lebanon	Meningitis	297 (204–404)	9·3% (-5·5 to 26·6%)	5·7 (3·9–7·8)	-31·5% (-40·5 to -21·3%)
	Encephalitis	196 (136–267)	40·1% (25·7 to 54·9%)	3·7 (2·6–5·1)	-17·9% (-26·4 to -8·5%)
	Tetanus	0 (0–1)	-48·7% (-87·9 to 58·3%)	0 (0–0)	-77·4% (-94·2 to -30·2%)
	Brain and central nervous system cancer	164 (100–244)	318·6% (176·1 to 489%)	3·1 (1·9–4·6)	128·8% (50·9 to 223·1%)
	Stroke*	11799 (8708–14761)	127·4% (116 to 140·3%)	224·6 (165·6–281·1)	1·8% (-2·8 to 7·2%)
	Ischaemic stroke	9967 (7343–12584)	146·5% (132·8 to 161·4%)	190·1 (140–240·3)	7·8% (1·9 to 14·1%)
	Intracerebral haemorrhage	1302 (922–1675)	52·5% (36 to 70·8%)	24·7 (17·4–31·6)	-25·7% (-33·5 to -17%)
	Subarachnoid haemorrhage	530 (363–729)	81·8% (54·2 to 113·8%)	9·9 (6·8–13·6)	-9·9% (-23·7 to 6·3%)
	Neurological disorders†	48653 (20353–92622)	81·3% (48·1 to 132·4%)	931·4 (391–1772·2)	-0·2% (-17·7 to 20·5%)
	Alzheimer's disease and other dementias	5722 (4024–7672)	227·4% (206·9 to 246·6%)	113·8 (79·9–152·6)	3·3% (-1·1 to 8·2%)
	Parkinson's disease	569 (396–783)	184% (140·8 to 236·9%)	10·8 (7·5–14·9)	7·8% (-8·5 to 26·4%)
	Idiopathic epilepsy	4637 (1101–9995)	24·1% (-71·5 to 456%)	91·5 (21·8–198·7)	-16·9% (-81·2 to 270·9%)
	Multiple sclerosis	739 (493–1016)	174·4% (129 to 232·9%)	13·6 (9·1–18·6)	33% (11·9 to 61·6%)
	Migraine	31980 (5563–73108)	76·4% (68·2 to 92·7%)	605·8 (104–1385·5)	0·5% (-2·7 to 3·5%)
	Tension-type headache	3565 (1217–10006)	86·4% (59 to 108·4%)	67 (22·7–191·7)	0·8% (-10·8 to 9·4%)
	Motor neuron disease	31 (22–44)	77·2% (65·9 to 87·5%)	0·6 (0·4–0·8)	2·8% (-1 to 6·9%)
	Other neurological disorders	1409 (532–2801)	73% (-37·5 to 470·2%)	28·3 (10·4–55·2)	21·8% (-55 to 271·5%)
	Headache disorders	35546 (8399–77063)	77·3% (68·9 to 92·6%)	672·7 (153·9–1457·6)	0·5% (-3·1 to 3·7%)
Libya	Meningitis	342 (237–460)	57·8% (41 to 77·2%)	5 (3·5–6·8)	-5·1% (-14·7 to 5·9%)
	Encephalitis	153 (105–209)	153·6% (135·9 to 178·4%)	2·2 (1·5–2·9)	39·6% (30·6 to 51·4%)
	Tetanus	0 (0–0)	-24·6% (-59·7 to 14·3%)	0 (0–0)	-46·2% (-73·2 to -18·6%)
	Brain and central nervous system cancer	119 (76–173)	154·9% (69·8 to 287·6%)	1·9 (1·2–2·7)	33·2% (-5·8 to 94·8%)
	Stroke*	13742 (9969–17230)	193% (177·2 to 209·7%)	248·1 (179·9–311)	12·8% (6·9 to 18·8%)
	Ischaemic stroke	11200 (8057–14164)	220% (199·6 to 240·5%)	209·8 (152·5–264·3)	21·6% (14·6 to 28·9%)
	Intracerebral haemorrhage	1857 (1298–2418)	103·6% (80·7 to 127·9%)	29 (20·5–37·8)	-21·7% (-29·4 to -13·3%)
	Subarachnoid haemorrhage	685 (470–950)	145·8% (102·5 to 188·2%)	9·3 (6·4–12·8)	-11% (-27·8 to 4%)
	Neurological disorders†	62689 (24320–122168)	92·6% (53·5 to 140·6%)	904·5 (393·2–1713·4)	-2% (-18·5 to 16·5%)

Table S12 Burden, mortality, incidence, and prevalence of neurological conditions in North Africa and Middle East countries

		All Ages		Age-standardised	
		Number	Percent change	Rate per 100,000	Percent change
**Measure**		2019	From 1990 to 2019	2019	From 1990 to 2019
Cause	Location	Mean (95% UI)	Mean (95% UI)	Mean (95% UI)	Mean (95% UI)
	Alzheimer's disease and other dementias	4356 (3058–5850)	167·3% (155·4 to 180·6%)	109 (76·7–145·9)	-2·1% (-6·2 to 2·7%)
	Parkinson's disease	549 (370–760)	210·8% (170 to 263·7%)	11·8 (8–16·3)	12% (-2·8 to 31%)
	Idiopathic epilepsy	5538 (1612–10918)	7·8% (-69·3 to 350·6%)	85·6 (25–167·6)	-26·2% (-78·6 to 201%)
	Multiple sclerosis	768 (520–1067)	253·4% (192·9 to 321·9%)	10·5 (7·2–14·6)	29·1% (8·3 to 54·8%)
	Migraine	44893 (7730–101676)	103·6% (88·6 to 141·3%)	594·7 (104–1347)	1·3% (-1·9 to 4·4%)
	Tension-type headache	4920 (1694–13899)	122·3% (84·9 to 164·2%)	66·4 (22·8–191)	1·1% (-11·2 to 9·5%)
	Motor neuron disease	35 (24–49)	81·7% (64·2 to 98·3%)	0·5 (0·3–0·7)	-4·9% (-8·6 to -0·9%)
	Other neurological disorders	1630 (664–3036)	47·7% (-42·9 to 404·3%)	25·9 (10·8–47·7)	10·2% (-56·3 to 209·3%)
	Headache disorders	49812 (11832–107596)	105·3% (90·3 to 140·3%)	661·1 (155·4–1425·1)	1·3% (-2·2 to 4·6%)
Morocco	Meningitis	2442 (1667–3340)	-18·3% (-30·3 to -2·8%)	6·8 (4·7–9·3)	-42·1% (-49·9 to -32%)
	Encephalitis	1159 (806–1589)	47·7% (36·5 to 60·2%)	3·1 (2·2–4·3)	-3·2% (-10·4 to 4·8%)
	Tetanus	4 (2–7)	-81·5% (-91·7 to -62·2%)	0 (0–0)	-82% (-91·4 to -65·1%)
	Brain and central nervous system cancer	218 (131–332)	123·8% (33·3 to 228·5%)	0·6 (0·4–0·9)	36·2% (-14 to 92·3%)
	Stroke*	85229 (62093–106733)	119·2% (108·3 to 131·5%)	265·1 (194·2–333)	3·4% (-1·4 to 9·1%)
	Ischaemic stroke	70229 (50759–88434)	143·8% (129·5 to 159·4%)	223 (161·5–281)	12·6% (6·4 to 19·5%)
	Intracerebral haemorrhage	11457 (8143–14677)	46·4% (32·2 to 61·3%)	32·6 (23–41·7)	-29·1% (-35·7 to -22·3%)
	Subarachnoid haemorrhage	3543 (2444–4750)	58·3% (30·8 to 84%)	9·6 (6·6–12·8)	-22·8% (-36·1 to -9·7%)
	Neurological disorders†	327176 (124928–641409)	62·1% (28·2 to 109·9%)	925·7 (383·4–1774·1)	1·1% (-18·1 to 23·1%)
	Alzheimer's disease and other dementias	24732 (17391–33330)	145·7% (134 to 158·8%)	110·9 (77·8–149·4)	-0·2% (-4·4 to 4·5%)
	Parkinson's disease	2959 (2025–4161)	176·2% (132·8 to 232·9%)	10·5 (7·2–14·6)	19·4% (0·8 to 42·1%)
	Idiopathic epilepsy	33678 (7332–72053)	30·9% (-74·5 to 553·2%)	96 (21–203·5)	-0·5% (-80·9 to 388·9%)
	Multiple sclerosis	3853 (2549–5380)	145·6% (107 to 193·4%)	10·4 (6·9–14·5)	23·9% (4·6 to 46·7%)
	Migraine	226776 (38690–515523)	58·5% (50·7 to 80·5%)	601·7 (104·2–1370·5)	-0·3% (-3·3 to 3%)
	Tension-type headache	24818 (8463–69426)	70·5% (47·5 to 93·4%)	66·5 (22·7–187·3)	0·2% (-9·8 to 8·1%)
	Motor neuron disease	183 (125–256)	62·3% (51·4 to 73·3%)	0·5 (0·3–0·7)	4% (0 to 8%)
	Other neurological disorders	10177 (3267–20278)	80·6% (-47·6 to 585·6%)	29·1 (9·6–58)	40·2% (-57·1 to 346·5%)
	Headache disorders	251594 (58542–544979)	59·6% (51·6 to 78·5%)	668·2 (156·2–1445·1)	-0·2% (-3·6 to 3·1%)
Palestine	Meningitis	372 (255–515)	39·3% (21·3 to 59·7%)	7·6 (5·2–10·4)	-42% (-49 to -33·9%)
	Encephalitis	201 (139–280)	153·5% (127 to 181·8%)	4·2 (2·9–5·8)	-3·1% (-12·4 to 6·9%)
	Tetanus	0 (0–0)	-58·1% (-87·2 to 12·5%)	0 (0–0)	-72·5% (-90·1 to -44·6%)
	Brain and central nervous system cancer	121 (79–167)	216·7% (92·4 to 357·9%)	3·2 (2–4·3)	25·1% (-19·5 to 76·2%)
	Stroke*	6341 (4621–7995)	161% (146·3 to 176·7%)	238 (173·6–302·4)	-3·9% (-9·4 to 1·6%)
	Ischaemic stroke	5124 (3703–6496)	163·5% (146·1 to 183·8%)	203·2 (147·6–260·1)	-0·9% (-7·4 to 5·9%)
	Intracerebral haemorrhage	858 (601–1132)	134% (107·2 to 161·3%)	25·4 (17·9–32·8)	-22% (-30·1 to -12·9%)
	Subarachnoid haemorrhage	359 (242–486)	202·8% (161·5 to 251·9%)	9·5 (6·5–12·9)	-5·3% (-18·9 to 11%)
	Neurological disorders†	39889 (14998–78109)	166·7% (106 to 236·2%)	915·4 (406·2–1702·6)	-0·9% (-17·1 to 18%)
	Alzheimer's disease and other dementias	1679 (1195–2282)	131·4% (119·3 to 143·7%)	111·6 (78·9–150·6)	1·5% (-3·4 to 6·1%)
	Parkinson's disease	218 (149–307)	159·2% (115·7 to 204·1%)	11 (7·6–15·1)	1·2% (-13·4 to 18%)
	Idiopathic epilepsy	4710 (1129–9817)	108·4% (-54·9 to 925·8%)	92 (22·5–190·6)	-10·2% (-80·5 to 341·2%)
	Multiple sclerosis	386 (259–538)	263·1% (206 to 331·9%)	10·5 (7–14·5)	16·9% (-0·6 to 39·5%)
	Migraine	28492 (4390–66341)	177·7% (166·5 to 199·2%)	596·1 (102·5–1357·3)	-1·1% (-4·1 to 1·9%)
	Tension-type headache	2914 (952–8613)	187·4% (144·1 to 219·3%)	66·2 (22·1–187·5)	-0·3% (-13 to 9·3%)
	Motor neuron disease	25 (17–35)	175·2% (158·3 to 189·6%)	0·5 (0·4–0·8)	4% (0 to 7·7%)

Table S12 Burden, mortality, incidence, and prevalence of neurological conditions in North Africa and Middle East countries

		**All Ages**		**Age-standardised**	
		Number	Percent change	Rate per 100,000	Percent change
**Measure**		2019	From 1990 to 2019	2019	From 1990 to 2019
Cause	Location	Mean (95% UI)	Mean (95% UI)	Mean (95% UI)	Mean (95% UI)
	Other neurological disorders	1464 (445–2897)	195·7% (-14·6 to 1003·2%)	27·5 (9·6–51·9)	27·6% (-55·3 to 278·1%)
	Headache disorders	31406 (6765–70639)	178·6% (166·2 to 198·3%)	662·3 (157·1–1439·2)	-1% (-4·4 to 2·1%)
Oman	Meningitis	71 (48–100)	59·2% (39·1 to 81·2%)	1·6 (1·1–2·2)	-32·6% (-40·7 to -23·8%)
	Encephalitis	137 (95–190)	112·6% (90·4 to 136·2%)	3 (2·1–4·1)	-17·2% (-25·6 to -8·2%)
	Tetanus	0 (0–0)	-25·8% (-69·3 to 53·4%)	0 (0–0)	-78·1% (-93·8 to -34·9%)
	Brain and central nervous system cancer	59 (30–92)	514·4% (193·5 to 920·7%)	1·6 (0·9–2·4)	130·8% (13·7 to 260·4%)
	Stroke*	5523 (3941–7070)	162% (145·6 to 179%)	237·2 (174·1–301·6)	-8·1% (-12·8 to -3%)
	Ischaemic stroke	4152 (2977–5371)	173% (151·6 to 194·5%)	197·7 (145·4–253·1)	-2·6% (-8·7 to 3·8%)
	Intracerebral haemorrhage	1005 (708–1327)	120·8% (94·1 to 152·2%)	31·1 (22·2–40)	-30·2% (-36·7 to -22·6%)
	Subarachnoid haemorrhage	366 (253–495)	176·3% (133·9 to 220%)	8·3 (5·8–11·3)	-19·2% (-32·4 to -5·8%)
	Neurological disorders†	38140 (13649–75171)	175·9% (112·6 to 250·9%)	877·8 (389·3–1634·8)	0·7% (-17·7 to 24·3%)
	Alzheimer's disease and other dementias	907 (642–1232)	140·1% (127·7 to 155·2%)	113·4 (79·6–152)	0·8% (-3·4 to 5·3%)
	Parkinson's disease	199 (133–284)	214% (165·6 to 267·9%)	15·6 (10·6–21·4)	26·7% (8·5 to 46·6%)
	Idiopathic epilepsy	3890 (946–8304)	107·9% (-53·3 to 851·7%)	92·8 (22·2–199·6)	-0·4% (-77·1 to 347%)
	Multiple sclerosis	393 (262–556)	346·2% (275·8 to 434%)	8·8 (5·9–12·2)	30·8% (10·3 to 55·9%)
	Migraine	28437 (4879–64400)	185% (166·5 to 226·5%)	554·2 (98·7–1256·8)	-1·4% (-4·5 to 1·9%)
	Tension-type headache	3196 (1042–8985)	208·1% (154·1 to 256·4%)	64·3 (21·6–190·1)	0% (-15·4 to 10·8%)
	Motor neuron disease	25 (17–34)	182·6% (159·7 to 207·3%)	0·5 (0·3–0·7)	2·7% (-1·3 to 6·9%)
	Other neurological disorders	1095 (363–2211)	171·8% (-21·8 to 887·6%)	28·1 (10·4–55·7)	41·7% (-53·9 to 325%)
	Headache disorders	31633 (7474–67794)	187·2% (168·1 to 223·2%)	618·5 (146·4–1332·9)	-1·2% (-4·9 to 2·6%)
Qatar	Meningitis	235 (156–332)	433·6% (314·1 to 580·1%)	8 (5·4–10·9)	-22·2% (-36·6 to -5·3%)
	Encephalitis	42 (29–57)	540·5% (486·5 to 583·1%)	1·4 (0·9–1·9)	-6% (-13·9 to 1·1%)
	Tetanus	0 (0–0)	322·8% (141·9 to 430%)	0 (0–0)	-27·4% (-63·8 to -3·7%)
	Brain and central nervous system cancer	49 (27–89)	1494·2% (769·7 to 2457·2%)	2·4 (1·5–3·8)	97·1% (16·7 to 204·4%)
	Stroke*	3099 (2242–3978)	555·1% (505·9 to 612·7%)	182·2 (132·5–228·2)	-24·2% (-28·2 to -19·9%)
	Ischaemic stroke	2104 (1497–2721)	577·8% (513·3 to 654·7%)	143·4 (105–182·3)	-23·4% (-28·4 to -18·4%)
	Intracerebral haemorrhage	669 (464–884)	486·1% (398·9 to 596·6%)	28·6 (20·2–36·5)	-30% (-37·3 to -22·4%)
	Subarachnoid haemorrhage	327 (223–443)	571·8% (450·8 to 693·6%)	10·2 (7–13·8)	-17·9% (-36·9 to -2·8%)
	Neurological disorders†	24393 (9303–47736)	572% (407·4 to 748·7%)	861·3 (393·5–1543·5)	-3·5% (-24·6 to 22·3%)
	Alzheimer's disease and other dementias	338 (231–473)	637·3% (552·5 to 735·9%)	110·5 (77·2–147·4)	2·8% (-1·9 to 8%)
	Parkinson's disease	112 (72–165)	807·3% (650 to 989·8%)	16·4 (11·3–22·5)	18% (0·7 to 36·9%)
	Idiopathic epilepsy	2530 (608–5562)	355·7% (9·8 to 1964·2%)	103·9 (25·2–227·9)	-22·8% (-80·9 to 249·8%)
	Multiple sclerosis	465 (313–641)	1172·4% (915·4 to 1561·2%)	14·1 (9·8–18·9)	52·2% (26 to 86·9%)
	Migraine	18109 (3233–40461)	601·2% (567·3 to 669·2%)	523 (94·3–1174·9)	-2·7% (-6·2 to 0·8%)
	Tension-type headache	2135 (699–6004)	643·8% (552 to 739·5%)	62 (20·7–184·7)	-1·4% (-11·6 to 7·2%)
	Motor neuron disease	20 (13–28)	631·4% (587·1 to 678·9%)	0·6 (0·4–0·8)	4·2% (-0·3 to 8·7%)
	Other neurological disorders	684 (252–1366)	536·4% (124·1 to 2135·2%)	30·8 (11·5–61·1)	16·1% (-56·9 to 248·4%)
	Headache disorders	20244 (5008–42949)	605·4% (571·7 to 667·4%)	585 (140·4–1252)	-2·6% (-5·8 to 0·9%)
Saudi Arabia	Meningitis	1038 (713–1412)	80·9% (59·7 to 105·7%)	2·9 (2–4)	-25·2% (-34·1 to -15·2%)
	Encephalitis	680 (473–947)	140·7% (117·8 to 165%)	1·8 (1·3–2·5)	-7·6% (-16·3 to 1·9%)
	Tetanus	1 (0–1)	-76% (-93·8 to -17·7%)	0 (0–0)	-87·9% (-96·6 to -59·1%)
	Brain and central nervous system cancer	619 (372–994)	932·3% (436 to 1905·1%)	1·8 (1·2–2·9)	237·6% (77·8 to 536·9%)
	Stroke*	68914 (48974–87456)	226·9% (200·7 to 255·2%)	299·4 (218·9–378·4)	7·2% (-1·5 to 15·6%)
	Ischaemic stroke	44814 (32037–57463)	240·3% (202·3 to 280·5%)	222 (162·1–281·5)	12·5% (0·7 to 24·5%)

Table S12 Burden, mortality, incidence, and prevalence of neurological conditions in North Africa and Middle East countries

Measure		All Ages		Age-standardised	
		Number	Percent change	Rate per 100,000	Percent change
		2019	From 1990 to 2019	2019	From 1990 to 2019
Cause	Location	Mean (95% UI)	Mean (95% UI)	Mean (95% UI)	Mean (95% UI)
	Intracerebral haemorrhage	20788 (14337–27557)	198·4% (164·8 to 235·6%)	68·9 (48·4–89·2)	-5·7% (-13·5 to 3·3%)
	Subarachnoid haemorrhage	3312 (2288–4602)	249·5% (196·9 to 308·4%)	8·5 (5·9–11·6)	-2·9% (-16·7 to 12·6%)
	Neurological disorders†	338288 (128872–653216)	172·5% (108·9 to 275·5%)	949·3 (419–1737·1)	3·3% (-18·3 to 35·6%)
	Alzheimer's disease and other dementias	9112 (6427–12276)	128·3% (112·8 to 144·1%)	109·4 (76·6–146·3)	1·2% (-3·3 to 5·8%)
	Parkinson's disease	1928 (1291–2728)	239·4% (184·2 to 304·5%)	14·8 (10·2–20·4)	27·1% (8·4 to 48·9%)
	Idiopathic epilepsy	51551 (12678–107945)	142·1% (-39·5 to 872·6%)	152·9 (37·8–320)	12·9% (-72·7 to 356·7%)
	Multiple sclerosis	2841 (1830–4067)	353·6% (279·3 to 435·7%)	7·3 (4·8–10·2)	25·4% (6·8 to 45·1%)
	Migraine	234239 (41410–528197)	176·4% (152·8 to 229·1%)	562 (99·4–1276·9)	-1·2% (-5·9 to 3·8%)
	Tension-type headache	25584 (9050–70083)	202% (136·9 to 255%)	63 (22–180·3)	-0·3% (-18·6 to 10·8%)
	Motor neuron disease	197 (135–277)	166·9% (141·5 to 192·2%)	0·5 (0·4–0·7)	2·9% (-0·9 to 6·8%)
	Other neurological disorders	12836 (4055–26144)	195·5% (-14·4 to 925·6%)	39·3 (13·3–79·7)	54·1% (-52·8 to 379·6%)
	Headache disorders	259823 (62718–548786)	178·7% (155·8 to 227·9%)	625 (149·7–1343·4)	-1·1% (-6·2 to 3·8%)
Syrian Arab Republic	Meningitis	1196 (807–1614)	-30·8% (-40·3 to -19·4%)	8·2 (5·6–11·2)	-38·4% (-46·4 to -29·7%)
	Encephalitis	551 (387–752)	16·4% (8 to 25·4%)	3·7 (2·6–5·1)	-7·5% (-13·3 to -0·9%)
	Tetanus	0 (0–0)	-87·9% (-94·9 to -69·9%)	0 (0–0)	-83·4% (-92·1 to -67·4%)
	Brain and central nervous system cancer	293 (185–435)	122·3% (34·4 to 241%)	2·1 (1·4–3·2)	42·3% (-4·4 to 112·2%)
	Stroke*	30049 (22030–37974)	77·7% (68·4 to 86·5%)	234·1 (172·3–295·5)	-13·7% (-17·7 to -9·6%)
	Ischaemic stroke	22824 (16673–29014)	94·5% (82·4 to 106·8%)	182·8 (133·9–233·7)	-8·8% (-13·6 to -3·7%)
	Intracerebral haemorrhage	5870 (4115–7482)	35·3% (22·2 to 49·3%)	42·1 (29·6–53·2)	-29·9% (-36·5 to -23·2%)
	Subarachnoid haemorrhage	1355 (917–1857)	62·7% (29·4 to 92·5%)	9·2 (6·4–12·4)	-14·4% (-30·5 to 0·6%)
	Neurological disorders†	128515 (48961–253992)	38·9% (11·1 to 84·7%)	916·3 (384·9–1745·3)	1·8% (-14·4 to 23%)
	Alzheimer's disease and other dementias	8807 (6210–11896)	109·5% (95·8 to 123·9%)	110·8 (77·7–149·2)	0·3% (-4 to 5·3%)
	Parkinson's disease	1198 (821–1670)	158·5% (118·1 to 207·3%)	11·5 (8–15·9)	10·1% (-6·3 to 30·5%)
	Idiopathic epilepsy	11625 (2685–24285)	-3·4% (-79·6 to 382·7%)	79·9 (18·6–167·4)	-6·2% (-79·8 to 370·4%)
	Multiple sclerosis	1511 (1018–2113)	124·9% (89·3 to 167·5%)	10·8 (7·3–15)	22·4% (3·8 to 43·8%)
	Migraine	91621 (15453–212202)	39·1% (31·2 to 62·3%)	609·9 (105·1–1394·5)	1·7% (-1·9 to 5·2%)
	Tension-type headache	9810 (3361–28381)	51·2% (24·9 to 78·8%)	67 (22·9–189·7)	1·1% (-10·5 to 9·5%)
	Motor neuron disease	78 (53–110)	28·3% (17·7 to 41·1%)	0·5 (0·4–0·8)	0·5% (-3·4 to 4·6%)
	Other neurological disorders	3865 (1320–7291)	42·2% (-55·8 to 438·3%)	25·9 (9·4–49·2)	37·1% (-54·2 to 305·1%)
	Headache disorders	101431 (22875–223796)	40·2% (32·3 to 61·8%)	676·9 (157·1–1474·2)	1·7% (-2·1 to 5·1%)
Tunisia	Meningitis	765 (524–1050)	6·7% (-6·7 to 24·3%)	6·5 (4·5–9·1)	-25% (-33·6 to -12·7%)
	Encephalitis	372 (257–506)	36·2% (23·4 to 51%)	3·1 (2·1–4·2)	-10·5% (-18·6 to -1·5%)
	Tetanus	0 (0–0)	-45·6% (-75·1 to -9·6%)	0 (0–0)	-56·9% (-80·3 to -30·5%)
	Brain and central nervous system cancer	98 (59–151)	187·5% (71·7 to 334·5%)	0·8 (0·5–1·3)	83·1% (10·5 to 174·5%)
	Stroke*	24933 (18357–31268)	180·2% (164·4 to 196·5%)	199·4 (146·5–250·1)	21·2% (14·7 to 27·8%)
	Ischaemic stroke	21265 (15507–26856)	194·8% (176·7 to 214·1%)	171·2 (125·8–216·1)	25·1% (18 to 33%)
	Intracerebral haemorrhage	2401 (1672–3170)	117·3% (89·7 to 151%)	18·6 (12·9–24·6)	1% (-11·9 to 16·4%)
	Subarachnoid haemorrhage	1266 (873–1737)	118·1% (71·9 to 163·3%)	9·6 (6·6–13)	2·9% (-19·3 to 24·5%)
	Neurological disorders†	109299 (46257–207558)	64·6% (41·2 to 114·7%)	916·1 (393·8–1740)	1·6% (-12·7 to 20·5%)
	Alzheimer's disease and other dementias	12094 (8498–16332)	221·5% (202·1 to 243%)	114·9 (80·6–155·1)	1·3% (-2·8 to 5·4%)
	Parkinson's disease	1324 (903–1807)	202·6% (153·7 to 262·6%)	11·2 (7·7–15·2)	13·9% (-3·3 to 34·8%)
	Idiopathic epilepsy	8669 (2011–19193)	23·8% (-69·5 to 529·6%)	78·3 (18·3–168)	-0·5% (-75·3 to 381·1%)
	Multiple sclerosis	1683 (1146–2277)	165% (124 to 214·2%)	12·7 (8·7–17·3)	27·1% (6·9 to 50·3%)

Table S12 Burden, mortality, incidence, and prevalence of neurological conditions in North Africa and Middle East countries					
		**All Ages**		**Age-standardised**	
		Number	Percent change	Rate per 100,000	Percent change
**Measure**		2019	From 1990 to 2019	2019	From 1990 to 2019
Cause	Location	Mean (95% UI)	Mean (95% UI)	Mean (95% UI)	Mean (95% UI)
	Migraine	74318 (13231–168600)	54·9% (45·1 to 82·1%)	606·3 (104·4–1382·7)	0·3% (-2·6 to 3·4%)
	Tension-type headache	8414 (2880–23528)	70·6% (42·7 to 97·2%)	67 (22·6–192·5)	0·7% (-10·7 to 8·5%)
	Motor neuron disease	71 (49–99)	61·1% (48·2 to 75·5%)	0·6 (0·4–0·8)	4·1% (-0·5 to 8·1%)
	Other neurological disorders	2727 (1117–5118)	70·6% (-35·2 to 451·1%)	25 (9·6–47·4)	38·2% (-48·7 to 285·9%)
	Headache disorders	82732 (20234–176614)	56·3% (46·6 to 81·3%)	673·3 (158·7–1448·7)	0·3% (-3·1 to 3·5%)
Türkiye	Meningitis	1235 (835–1710)	-49·8% (-56·7 to -42%)	1·5 (1–2·1)	-64·1% (-69 to -58·5%)
	Encephalitis	2677 (1834–3686)	35·4% (19 to 50·4%)	3·1 (2·1–4·3)	-10·6% (-21·2 to -0·5%)
	Tetanus	1 (1–2)	-87·3% (-94 to -72·3%)	0 (0–0)	-92·6% (-96·6 to -81·5%)
	Brain and central nervous system cancer	2518 (1094–3941)	178·5% (50·6 to 349·9%)	3 (1·3–4·7)	67·7% (-3·5 to 160·9%)
	Stroke*	168016 (122306–211256)	104·2% (93·2 to 116%)	191·2 (139·3–239·6)	-8·6% (-13·2 to -3·6%)
	Ischaemic stroke	126600 (92718–160564)	117·6% (102·4 to 133·2%)	145·8 (106·5–184)	-5·7% (-11·7 to 0·5%)
	Intracerebral haemorrhage	30767 (21581–39288)	74·8% (56·5 to 93·9%)	34·2 (24·1–43·7)	-15·9% (-24·3 to -6·6%)
	Subarachnoid haemorrhage	10649 (7316–14357)	63·5% (38·4 to 92·1%)	11·3 (7·8–15·2)	-19·5% (-31·6 to -4·8%)
	Neurological disorders†	833810 (374048–1538176)	61·1% (30·1 to 120·6%)	966·5 (437·5–1760)	1% (-18·7 to 29·4%)
	Alzheimer's disease and other dementias	92726 (65127–124219)	199·6% (185·3 to 212·4%)	116·9 (82–157·2)	3·9% (-0·7 to 8·2%)
	Parkinson's disease	10292 (7018–14162)	220·9% (175·2 to 275·1%)	12·3 (8·4–16·8)	21·5% (4·3 to 41·8%)
	Idiopathic epilepsy	102409 (24971–214021)	18·7% (-71·1 to 432·2%)	131·3 (32·4–273·9)	-5·2% (-76·5 to 327·2%)
	Multiple sclerosis	11771 (8260–15345)	109·3% (78 to 144·8%)	12·5 (8·8–16·3)	2·9% (-11·9 to 20%)
	Migraine	528078 (101819–1197352)	56·6% (45·4 to 81·9%)	589·6 (110·2–1340·7)	0·2% (-4·5 to 5·2%)
	Tension-type headache	60666 (21747–160117)	71·5% (44·9 to 93·8%)	66·6 (23·6–180)	0·8% (-10 to 10·1%)
	Motor neuron disease	672 (464–916)	77·3% (64·6 to 89·4%)	0·8 (0·5–1)	10·2% (6·2 to 14·5%)
	Other neurological disorders	27197 (10055–51718)	46·3% (-49 to 378·4%)	36·7 (13–71·8)	26·5% (-57·8 to 276·8%)
	Headache disorders	588744 (155386–1275312)	58% (47·2 to 81%)	656·1 (167·7–1433)	0·3% (-4·6 to 5%)
United Arab Emirates	Meningitis	302 (208–415)	333·9% (292·3 to 376·9%)	3·6 (2·5–4·9)	-16·1% (-24·9 to -6·1%)
	Encephalitis	165 (113–229)	591·3% (545·5 to 639·8%)	1·6 (1·1–2·2)	28·8% (19·3 to 38%)
	Tetanus	0 (0–1)	19·4% (-65·4 to 265·1%)	0 (0–0)	-83% (-95 to -29·7%)
	Brain and central nervous system cancer	180 (89–302)	736·5% (427·5 to 1139·8%)	2·1 (1·1–3·2)	25·8% (-25·8 to 91·7%)
	Stroke*	18775 (13548–24191)	686·9% (628·8 to 746·8%)	336·4 (245·1–425·6)	-10·2% (-14·8 to -5·7%)
	Ischaemic stroke	14430 (10385–18659)	754·8% (678·1 to 839·9%)	289·4 (210–368)	-5·9% (-11·6 to -0·7%)
	Intracerebral haemorrhage	3249 (2285–4213)	492·2% (407·8 to 599·6%)	38·7 (27·7–49·6)	-32% (-37·9 to -25·2%)
	Subarachnoid haemorrhage	1097 (754–1555)	634·3% (522·4 to 767·7%)	8·2 (5·7–11·3)	-15·8% (-27 to -3·1%)
	Neurological disorders†	84688 (33224–163183)	430·1% (298·9 to 626·6%)	917 (419·7–1693·9)	-4·9% (-28·6 to 25·3%)
	Alzheimer's disease and other dementias	1032 (696–1462)	610·5% (527·9 to 705·4%)	103·7 (72·7–139·5)	-0·4% (-4·8 to 3·8%)
	Parkinson's disease	474 (311–710)	907·8% (719·4 to 1151·3%)	16·1 (11·3–22·3)	10·9% (-4·2 to 28·2%)
	Idiopathic epilepsy	11873 (3086–23522)	251·2% (-12·1 to 1308·2%)	152·1 (38·2–303·4)	-23·7% (-81·4 to 211·6%)
	Multiple sclerosis	832 (544–1183)	699% (539·2 to 921·1%)	6·3 (4·3–8·6)	-1·4% (-17·9 to 19·9%)
	Migraine	60153 (11995–134011)	473% (424 to 588·1%)	535·6 (97·8–1203·7)	-0·7% (-4·3 to 3·1%)
	Tension-type headache	7426 (2396–20517)	542·2% (427·1 to 676·1%)	63·2 (20·7–187·8)	0% (-12·6 to 8·9%)
	Motor neuron disease	63 (43–91)	472·1% (400 to 543·2%)	0·6 (0·4–0·8)	-0·6% (-4·4 to 4·5%)
	Other neurological disorders	2834 (932–5379)	347·5% (39 to 1491·1%)	39·3 (12·8–76·3)	9·5% (-65·6 to 251·2%)
	Headache disorders	67579 (17571–141865)	479·9% (432·5 to 583%)	598·8 (142·4–1275·1)	-0·7% (-5 to 3%)
Yemen	Meningitis	2295 (1582–3166)	86·1% (63·1 to 112·3%)	7·4 (5·2–10·2)	-20% (-29·5 to -9·3%)
	Encephalitis	1071 (745–1479)	187·7% (163·7 to 215·1%)	3·5 (2·5–4·8)	16·7% (8·3 to 26·2%)

Table S12 Burden, mortality, incidence, and prevalence of neurological conditions in North Africa and Middle East countries

		**All Ages**		**Age-standardised**	
		Number	Percent change	Rate per 100,000	Percent change
**Measure**		2019	From 1990 to 2019	2019	From 1990 to 2019
Cause	Location	Mean (95% UI)	Mean (95% UI)	Mean (95% UI)	Mean (95% UI)
	Tetanus	1 (1–3)	-64·9% (-87·5 to 3·4%)	0 (0–0)	-77·7% (-91·4 to -48·7%)
	Brain and central nervous system cancer	272 (145–430)	191·3% (55 to 447·4%)	1·2 (0·7–1·9)	32·2% (-24·3 to 118·2%)
	Stroke*	39779 (29049–50057)	175·4% (160·3 to 191·5%)	249·6 (183·1–315·4)	1·2% (-4·2 to 7%)
	Ischaemic stroke	30505 (22098–38798)	216·5% (196·9 to 238·2%)	202·9 (148·1–258·8)	15·1% (7·9 to 22·6%)
	Intracerebral haemorrhage	7123 (5082–9074)	82% (63·7 to 101·4%)	36·7 (26·3–46·8)	-37·2% (-42·9 to -30·4%)
	Subarachnoid haemorrhage	2151 (1490–2876)	140·2% (106·5 to 178·1%)	10 (7–13·5)	-16·6% (-28·6 to -3·4%)
	Neurological disorders†	238440 (80945–479466)	158·2% (97·3 to 245%)	887·5 (363·5–1694·8)	-1·1% (-17·1 to 19·2%)
	Alzheimer's disease and other dementias	9054 (6367–12226)	195·2% (180·2 to 211·8%)	109·2 (76·5–146·7)	-2·6% (-7·1 to 2%)
	Parkinson's disease	1048 (702–1482)	204·1% (154 to 263·5%)	9 (6·2–12·4)	9·6% (-7·1 to 29·6%)
	Idiopathic epilepsy	25729 (5544–56291)	85·9% (-62·5 to 936·9%)	76·5 (16·8–162·9)	-15·5% (-83·6 to 372·7%)
	Multiple sclerosis	1351 (896–1903)	249·8% (197·8 to 314·9%)	6·2 (4·1–8·6)	21·6% (4·4 to 42·9%)
	Migraine	175369 (26571–404422)	169% (158·9 to 186·6%)	596·7 (104·2–1356·8)	0·1% (-2·9 to 3·2%)
	Tension-type headache	17617 (5766–51725)	176·7% (140 to 209·4%)	66·1 (22·8–185·6)	0·6% (-11·1 to 9·8%)
	Motor neuron disease	100 (68–140)	153% (137·6 to 168·2%)	0·3 (0·2–0·5)	-0·8% (-4·5 to 3·2%)
	Other neurological disorders	8171 (2243–16997)	162·8% (-28·4 to 1089%)	23·5 (7·9–45·9)	20·8% (-61·6 to 293·2%)
	Headache disorders	192986 (40627–431548)	169·7% (159·4 to 187·2%)	662·8 (157·9–1435·6)	0·1% (-3·4 to 3·6%)
Afghanistan	Meningitis	5811 (4106–7806)	108·3% (79·6 to 142·8%)	19·1 (13·9–25·3)	-33·2% (-42 to -22·8%)
	Encephalitis	2459 (1749–3338)	288·8% (253·9 to 328·3%)	7·5 (5·4–10·1)	12·6% (2·4 to 23·8%)
	Tetanus	12 (6–19)	-59·9% (-78·4 to -18·6%)	0 (0–0·1)	-82·7% (-89·1 to -70·5%)
	Brain and central nervous system cancer	386 (195–728)	158·9% (67·4 to 354·3%)	1·5 (0·8–2·9)	2·9% (-32·8 to 63·2%)
	Stroke*	41547 (30305–52032)	127·5% (113·7 to 141·6%)	248·5 (183·7–312)	3·4% (-2·1 to 8·1%)
	Ischaemic stroke	29039 (21015–36955)	142·9% (123·4 to 161·9%)	189·7 (139·8–240·7)	17·3% (9·8 to 23·9%)
	Intracerebral haemorrhage	10209 (7293–13030)	87·6% (68·5 to 109·1%)	49·1 (35·3–62·2)	-27·6% (-33·8 to -21·2%)
	Subarachnoid haemorrhage	2299 (1599–3105)	166·4% (129·5 to 213·7%)	9·6 (6·7–12·9)	-10·6% (-23·4 to 4·2%)
	Neurological disorders†	279547 (101550–560990)	224·6% (127·3 to 339·8%)	896·8 (387·4–1711·2)	-2·4% (-22·3 to 23·3%)
	Alzheimer's disease and other dementias	7490 (5271–10167)	54·5% (46·3 to 63·3%)	103·8 (73·3–140·2)	-2·3% (-6·5 to 2·7%)
	Parkinson's disease	1059 (715–1488)	63·8% (37·9 to 94·7%)	9·6 (6·5–13·3)	-4·1% (-17·2 to 11·4%)
	Idiopathic epilepsy	37823 (6439–83555)	185·9% (-42 to 2058·3%)	92 (15·6–202·9)	-16·3% (-83·1 to 512·7%)
	Multiple sclerosis	2557 (1653–3578)	260·4% (199 to 333·6%)	10·8 (7·1–14·8)	17·9% (-1·6 to 41·8%)
	Migraine	198849 (30218–469528)	244·1% (220·3 to 261·8%)	587·4 (103–1342·7)	-1·3% (-4·9 to 3·2%)
	Tension-type headache	19586 (6272–57731)	231·4% (191·2 to 263·2%)	65·2 (22·1–183·3)	-0·1% (-9·6 to 7·9%)
	Motor neuron disease	138 (94–195)	238·3% (219·8 to 260%)	0·4 (0·3–0·6)	0·2% (-4 to 4·2%)
	Other neurological disorders	12045 (2865–25726)	308·4% (0 to 1736·2%)	27·6 (8·7–55·4)	20·4% (-63·8 to 330·8%)
	Headache disorders	218435 (44781–493226)	242·9% (219·8 to 259·7%)	652·5 (156·7–1422·4)	-1·2% (-4·5 to 2·9%)
Sudan	Meningitis	1639 (1137–2271)	-47·7% (-55 to -38·7%)	4·1 (2·8–5·5)	-74·4% (-77·6 to -70·4%)
	Encephalitis	1303 (908–1779)	114·1% (95·8 to 132·8%)	3·3 (2·3–4·5)	0·2% (-7·7 to 8·4%)
	Tetanus	1 (1–2)	-76·4% (-90·3 to -41·6%)	0 (0–0)	-83·6% (-92·6 to -68·2%)
	Brain and central nervous system cancer	458 (246–701)	131·1% (10·5 to 404%)	1·4 (0·8–2·1)	25·8% (-31·3 to 129·2%)
	Stroke*	60626 (43937–76264)	122·8% (111·4 to 134·4%)	276·5 (201·3–351·5)	8·2% (2·6 to 13·3%)
	Ischaemic stroke	47279 (33937–60223)	150·9% (135·3 to 167·7%)	226·9 (164·1–290·5)	22·6% (15·5 to 30·3%)
	Intracerebral haemorrhage	10455 (7363–13471)	53·2% (37·9 to 69%)	39·7 (28·1–50·3)	-32·1% (-38·3 to -25·6%)
	Subarachnoid haemorrhage	2893 (2009–3932)	87·6% (60·3 to 116·6%)	9·9 (6·9–13·3)	-18·9% (-31·2 to -5·4%)
	Neurological disorders†	323508 (113529–649897)	118·3% (70·7 to 191·4%)	902·9 (375·3–1718·2)	0·3% (-16 to 24·4%)

Table S12 Burden, mortality, incidence, and prevalence of neurological conditions in North Africa and Middle East countries

		All Ages		Age-standardised	
		Number	Percent change	Rate per 100,000	Percent change
**Measure**		2019	From 1990 to 2019	2019	From 1990 to 2019
Cause	Location	Mean (95% UI)	Mean (95% UI)	Mean (95% UI)	Mean (95% UI)
	Alzheimer's disease and other dementias	14276 (10170–19240)	115% (101·6 to 129·3%)	108·5 (76·5–145·8)	-0·1% (-4·3 to 4·3%)
	Parkinson's disease	1655 (1112–2320)	102·3% (69 to 143%)	9·9 (6·8–14)	1·8% (-14·6 to 21·1%)
	Idiopathic epilepsy	36309 (8020–79414)	84·4% (-56·9 to 958·2%)	84·3 (18·4–183·6)	-5·1% (-77·8 to 449·1%)
	Multiple sclerosis	1798 (1181–2524)	176·2% (132·5 to 220·5%)	6·1 (4·1–8·5)	22·1% (2·7 to 41·7%)
	Migraine	234320 (36676–542009)	122·2% (114·9 to 132·9%)	601·9 (104·6–1369·5)	-0·1% (-3·2 to 3·2%)
	Tension-type headache	23633 (7813–69143)	125% (95·3 to 148·4%)	66·4 (23·2–189)	0·5% (-11·8 to 9·8%)
	Motor neuron disease	135 (91–188)	123·4% (113·4 to 134·2%)	0·4 (0·2–0·5)	4·1% (0·4 to 8·2%)
	Other neurological disorders	11382 (3219–23893)	158·3% (-26·2 to 1024·1%)	25·4 (9–50·9)	31·9% (-56 to 326·6%)
	Headache disorders	257953 (53961–578953)	122·5% (114·3 to 132·3%)	668·3 (155·5–1438·9)	-0·1% (-3·7 to 3·7%)
**YLLs (Years of Life Lost)**					
Global	Meningitis	15649865 (13102961–18930650)	-52·1% (-60·4 to -42·7%)	225 (187·3–273·1)	-57·7% (-65 to -48·8%)
	Encephalitis	4314970 (3623222–5928341)	-46·2% (-58·5 to -17·4%)	59·2 (49·5–80·9)	-55·9% (-65·7 to -33·2%)
	Tetanus	2314636 (1768800–3276980)	-89·4% (-92·1 to -83·8%)	33·7 (25·6–47·8)	-90·3% (-92·7 to -85·1%)
	Brain and central nervous system cancer	8530468 (6625077–9447558)	39·7% (-13·7 to 66%)	107·4 (83·1–119)	-10·8% (-43·7 to 4·8%)
	Stroke*	125490442 (116154749–134427938)	27% (15·3 to 37·8%)	1550 (1434·5–1660·7)	-38% (-43·7 to -32·9%)
	Ischaemic stroke	50349738 (46232449–54066668)	48·1% (32·3 to 61·3%)	636·5 (582–683·1)	-33·4% (-40 to -27·6%)
	Intracerebral haemorrhage	65306215 (60073845–70392269)	24% (10·4 to 35·4%)	793·4 (731·2–854·6)	-37·4% (-44·2 to -31·5%)
	Subarachnoid haemorrhage	9834489 (8614600–11416373)	-19·1% (-30·7 to 12·6%)	120·1 (105·5–139·2)	-57% (-63·3 to -39·8%)
	Neurological disorders†	32098442 (18750071–60829291)	81·3% (45 to 114·6%)	422·3 (243·2–806·2)	-7·4% (-16·5 to 2%)
	Alzheimer's disease and other dementias	17859920 (4374613–47064787)	160·3% (145·7 to 181·7%)	239·8 (58·5–627·2)	2·9% (-1·5 to 9·8%)
	Parkinson's disease	5082522 (4673385–5409853)	123·5% (105·6 to 139·9%)	64·9 (59·6–69·2)	-0·3% (-7·9 to 6·5%)
	Idiopathic epilepsy	5336821 (4722718–6170046)	-9·5% (-24·1 to 27·5%)	69·5 (61·7–80·6)	-33·6% (-44 to -7·6%)
	Multiple sclerosis	708628 (645137–897753)	53·2% (35·6 to 73·7%)	8·5 (7·7–10·7)	-17·4% (-27·9 to -7·6%)
	Motor neuron disease	977539 (926348–1025430)	65·5% (54·5 to 78·2%)	11·9 (11·3–12·5)	-4·9% (-10·7 to 1·9%)
	Other neurological disorders	2133013 (1915236–2381088)	31·4% (16·2 to 49·6%)	27·7 (24·7–31)	-8·9% (-19·1 to 3·5%)
North Africa and Middle East	Meningitis	376540 (310241–457477)	-72·5% (-79·3 to -64·6%)	64·1 (53–77·6)	-77·2% (-82·4 to -70·9%)
	Encephalitis	231250 (179810–314123)	9·8% (-25·4 to 58·2%)	38·8 (30·3–52·5)	-21·8% (-45·9 to 9·5%)
	Tetanus	87385 (53584–139487)	-85·8% (-92·9 to -68·7%)	15 (9·2–24·1)	-88·2% (-93·9 to -76·1%)
	Brain and central nervous system cancer	705624 (485965–836908)	69·9% (0·4 to 130·4%)	126·4 (86·6–149·3)	-5·6% (-40·2 to 22·6%)
	Stroke*	6832264 (6014765–7787849)	34·5% (18 to 53·6%)	1586·8 (1407–1782·8)	-35·1% (-42·5 to -25·9%)
	Ischaemic stroke	3874055 (3427786–4371090)	112·9% (77·1 to 146·1%)	987·9 (880·9–1105·9)	-11·1% (-23·3 to 1·3%)
	Intracerebral haemorrhage	2520027 (2171781–2943061)	0·1% (-15·7 to 18·7%)	514 (447·9–593)	-52·9% (-59·7 to -44·9%)
	Subarachnoid haemorrhage	438182 (355907–559639)	-41% (-55·3 to 0·5%)	84·8 (69·4–107·1)	-64·9% (-73·3 to -43·4%)
	Neurological disorders†	1708193 (1049368–3130756)	68·3% (24·8 to 124·2%)	447·2 (241·7–897·3)	-11·5% (-23·1 to 7·1%)
	Alzheimer's disease and other dementias	855879 (207406–2304917)	173·2% (151·8 to 232·1%)	274·2 (67·3–727·7)	-2·6% (-9·7 to 17·2%)

Table S12 Burden, mortality, incidence, and prevalence of neurological conditions in North Africa and Middle East countries

		All Ages		Age-standardised	
		Number	Percent change	Rate per 100,000	Percent change
**Measure**		2019	From 1990 to 2019	2019	From 1990 to 2019
Cause	Location	Mean (95% UI)	Mean (95% UI)	Mean (95% UI)	Mean (95% UI)
	Parkinson's disease	256193 (225088–319531)	158·7% (125·3 to 202·8%)	72·7 (63·9–91·5)	-1% (-13·1 to 15·8%)
	Idiopathic epilepsy	352158 (279073–412488)	-18·1% (-37·2 to 39%)	57·3 (45·6–67·2)	-44·4% (-55·6 to -11·7%)
	Multiple sclerosis	58198 (45154–80375)	126·5% (55·9 to 238%)	9·9 (7·8–13·6)	1·8% (-27·9 to 52·7%)
	Motor neuron disease	38320 (30630–47074)	18·4% (-32·8 to 87·7%)	7·3 (5·8–9)	-17·8% (-47·1 to 19·1%)
	Other neurological disorders	147446 (127889–171848)	28·8% (-3·1 to 67·3%)	25·7 (22·4–29·9)	-9·5% (-30 to 15·6%)
Algeria	Meningitis	13994 (11227–17746)	-77·6% (-86·5 to -65%)	34·6 (27·8–43·5)	-82·2% (-88·8 to -73·4%)
	Encephalitis	7133 (4745–13687)	-9·7% (-47·2 to 43·8%)	17·3 (11·7–32·9)	-32·4% (-58 to 3%)
	Tetanus	539 (393–942)	-83% (-93·5 to -41·9%)	1·3 (0·9–2·3)	-86·7% (-94·8 to -60·7%)
	Brain and central nervous system cancer	23311 (14132–29666)	55·2% (-17·1 to 119%)	57·4 (34·8–72·8)	-6·6% (-50·2 to 27·9%)
	Stroke*	458921 (365717–567391)	37·9% (8·1 to 76·4%)	1512·6 (1223–1842·1)	-47·8% (-58·8 to -34%)
	Ischaemic stroke	302904 (238050–377992)	121·8% (69·2 to 188%)	1071 (848·5–1326·6)	-27·8% (-43·4 to -9·4%)
	Intracerebral haemorrhage	131101 (101693–165721)	-19·2% (-39·1 to 7·8%)	374·3 (293–473·1)	-69% (-76·8 to -59·3%)
	Subarachnoid haemorrhage	24917 (18170–34201)	-26·7% (-49·3 to 20·6%)	67·4 (49·6–90·1)	-67·1% (-77·2 to -46·7%)
	Neurological disorders†	120531 (65613–244802)	76·5% (15·8 to 149·8%)	436·9 (213·5–944)	-19·1% (-37·2 to -0·3%)
	Alzheimer's disease and other dementias	68222 (16660–189706)	223·2% (150·9 to 312·3%)	288·2 (70·3–791·7)	-7·2% (-23·2 to 12·4%)
	Parkinson's disease	18267 (14367–22680)	153·4% (90·7 to 234·1%)	67·5 (53·3–83·2)	-21·4% (-39·6 to 2·4%)
	Idiopathic epilepsy	21819 (16684–27174)	-33·4% (-53·1 to 9%)	51·6 (39·6–64·2)	-54·4% (-67·1 to -27·6%)
	Multiple sclerosis	3976 (1993–5812)	144·3% (39·6 to 288%)	9·3 (4·6–13·5)	0·1% (-42·7 to 55·6%)
	Motor neuron disease	811 (613–1058)	123·9% (53·5 to 228·4%)	2 (1·5–2·7)	-3% (-34·5 to 46·4%)
	Other neurological disorders	7435 (6159–8871)	43·2% (-7·6 to 100·8%)	18·3 (15·2–21·7)	-5·3% (-35·7 to 28·1%)
Bahrain	Meningitis	150 (124–180)	-54·4% (-66·4 to -39·6%)	14·7 (12·2–17·6)	-77·7% (-82·9 to -71·1%)
	Encephalitis	111 (87–140)	52·7% (9·2 to 97%)	9·5 (7·5–12)	-28·6% (-48·3 to -9·3%)
	Tetanus	10 (8–14)	37·1% (-41 to 114·9%)	0·7 (0·6–1·2)	-39·4% (-75 to -6·6%)
	Brain and central nervous system cancer	1001 (606–1327)	183·3% (66·3 to 297·5%)	71·5 (45·8–91·6)	-25·8% (-56·1 to 5·3%)
	Stroke*	5911 (4755–7465)	82·8% (42·3 to 141·6%)	767 (633·5–977·6)	-56·1% (-65 to -45%)
	Ischaemic stroke	2614 (2086–3342)	123·7% (71·8 to 193·8%)	473·6 (390–608·3)	-47·7% (-58·7 to -35%)
	Intracerebral haemorrhage	2564 (2018–3270)	67·6% (24·5 to 126·6%)	238·4 (192·5–299·8)	-65·9% (-74 to -55·4%)
	Subarachnoid haemorrhage	732 (537–1043)	37·2% (-6·2 to 119·3%)	55 (41·9–77·4)	-61% (-73 to -43·7%)
	Neurological disorders†	2810 (1778–5047)	177·7% (104·4 to 266·1%)	464·1 (236·7–947·9)	-14·5% (-29·9 to 1·7%)
	Alzheimer's disease and other dementias	1202 (287–3440)	382·8% (289·1 to 512·1%)	289·4 (70·1–764·3)	-6·2% (-20·9 to 12·1%)
	Parkinson's disease	368 (248–469)	266·9% (169·3 to 389·4%)	75·5 (52·4–93·7)	-16·7% (-37·1 to 7·3%)
	Idiopathic epilepsy	785 (618–1037)	65% (28·5 to 120·6%)	61·4 (49·5–77·6)	-39·1% (-52 to -18·5%)
	Multiple sclerosis	78 (45–106)	412·5% (190·6 to 618·1%)	4·1 (2·5–5·5)	-7·1% (-44·4 to 28%)
	Motor neuron disease	23 (18–29)	86% (35·2 to 150·7%)	1·5 (1·2–1·8)	-62·5% (-71·9 to -50·5%)
	Other neurological disorders	354 (295–429)	122·4% (70·4 to 191·1%)	32·2 (26·9–38·3)	-6·8% (-26·3 to 19·5%)
Egypt	Meningitis	48294 (31689–67133)	-70·9% (-81·2 to -56·9%)	47·7 (31·3–66·4)	-77·7% (-85·5 to -67·2%)
	Encephalitis	61833 (29716–99309)	-6·1% (-45·9 to 55·8%)	59·5 (29·6–95·5)	-32·7% (-60·1 to 5·5%)
	Tetanus	7402 (2203–21951)	-87·3% (-96·4 to -60·1%)	7·7 (2·5–23·2)	-90·8% (-97 to -71·7%)
	Brain and central nervous system cancer	88019 (60623–123901)	72·8% (-6·2 to 152·9%)	97·6 (67·4–139·6)	-2·2% (-41 to 39·4%)
	Stroke*	1300110 (960642–1749931)	-15·4% (-39·9 to 25·9%)	1855·4 (1367·5–2533·1)	-39·7% (-55·1 to -19·7%)
	Ischaemic stroke	704099 (521871–961041)	86·9% (27·6 to 165·9%)	1141·7 (834·6–1566·8)	10·8% (-22 to 46%)
	Intracerebral haemorrhage	498924 (354451–683948)	-38·6% (-58·8 to -8·7%)	608 (426·1–849·3)	-60·8% (-72 to -46%)
	Subarachnoid haemorrhage	97087 (67306–137958)	-72% (-84·1 to -33·8%)	105·7 (74·2–149·7)	-78·7% (-86·7 to -53·8%)

Table S12 Burden, mortality, incidence, and prevalence of neurological conditions in North Africa and Middle East countries

		All Ages		Age-standardised	
		Number	Percent change	Rate per 100,000	Percent change
**Measure**		2019	From 1990 to 2019	2019	From 1990 to 2019
Cause	Location	Mean (95% UI)	Mean (95% UI)	Mean (95% UI)	Mean (95% UI)
	Neurological disorders†	198502 (115959–369299)	80·9% (36·9 to 132·9%)	405·1 (199·8–823·1)	-1·6% (-20·5 to 23·9%)
	Alzheimer's disease and other dementias	94371 (21935–259421)	109·5% (67·1 to 173·8%)	252·4 (56·8–672·4)	-1·7% (-19·3 to 25·3%)
	Parkinson's disease	41977 (31577–61942)	133·4% (80·9 to 196%)	86·6 (65·3–130·4)	11·1% (-12·2 to 39·4%)
	Idiopathic epilepsy	24374 (16027–36689)	-8·4% (-35·4 to 47·7%)	24 (16·1–35·9)	-42·9% (-59 to -10·4%)
	Multiple sclerosis	9543 (4393–30024)	73·8% (-47·9 to 495·6%)	9·7 (4·7–26·4)	-1·1% (-64·7 to 191·7%)
	Motor neuron disease	6029 (3334–9618)	237·5% (78·8 to 465·4%)	7·5 (4·1–11·9)	76·4% (-5·4 to 195·5%)
	Other neurological disorders	22208 (14954–31604)	73·1% (17 to 150·5%)	24·8 (16·8–35·2)	19·9% (-17·1 to 65·9%)
Iran (Islamic Republic of)	Meningitis	24175 (21013–27651)	-82·4% (-87 to -76·1%)	31·7 (27·2–36·5)	-82·1% (-86·5 to -75·8%)
	Encephalitis	10246 (7698–12077)	-21·1% (-45·2 to 8·7%)	13·5 (9·8–16·2)	-24·4% (-45·9 to 0·9%)
	Tetanus	910 (692–1253)	-94·4% (-97·6 to -82·2%)	1·2 (0·9–1·7)	-94·1% (-97·4 to -82·4%)
	Brain and central nervous system cancer	126167 (66631–150728)	39·2% (-28·5 to 80%)	153·5 (80·7–183·6)	-11·4% (-50·6 to 7·8%)
	Stroke*	736731 (679915–781711)	38·6% (23 to 55·4%)	1065·8 (976·7–1134·4)	-49·2% (-54·5 to -41·4%)
	Ischaemic stroke	543997 (496639–578722)	58·8% (39·2 to 79·3%)	815·3 (737·4–870·1)	-46·6% (-52·8 to -38%)
	Intracerebral haemorrhage	156169 (147538–165405)	2% (-11 to 15·5%)	203·3 (190·9–215·2)	-55·6% (-61·9 to -49·2%)
	Subarachnoid haemorrhage	36565 (32315–43627)	2·8% (-24·5 to 62·9%)	47·2 (41·9–54·8)	-59% (-71·2 to -34·3%)
	Neurological disorders†	270143 (147800–532947)	93·5% (24·4 to 184·4%)	418·4 (213·6–866·4)	-11·4% (-24·7 to 14%)
	Alzheimer's disease and other dementias	160837 (38235–426338)	309·6% (265·3 to 447·6%)	272·1 (66·1–723)	-0·9% (-7·7 to 32%)
	Parkinson's disease	40712 (34551–44885)	253% (199·1 to 325·2%)	64·8 (55·1–71·4)	1·4% (-17·1 to 21·9%)
	Idiopathic epilepsy	33416 (30081–39269)	-53·1% (-65·8 to -19·2%)	40·2 (36–47·4)	-59% (-69·4 to -34·5%)
	Multiple sclerosis	14945 (12308–21975)	152·2% (86·7 to 244·2%)	16·2 (13·4–23·5)	5·1% (-26·7 to 48·4%)
	Motor neuron disease	3291 (2875–3758)	191·4% (89·1 to 307·9%)	4·1 (3·6–4·7)	51·5% (0·1 to 113·1%)
	Other neurological disorders	16942 (15688–18168)	61·1% (18·9 to 116·6%)	21·1 (19·4–22·6)	20·8% (-8·5 to 62·8%)
Iraq	Meningitis	25931 (19826–33764)	-71·8% (-80·9 to -58·8%)	61·6 (47·4–78·2)	-81·3% (-86·9 to -73·7%)
	Encephalitis	29074 (19822–39114)	15·1% (-30·7 to 86·4%)	67·4 (47–89)	-34·7% (-58·1 to -2·2%)
	Tetanus	1781 (1012–3002)	-91·2% (-97·3 to -53·5%)	4 (2·3–6·7)	-93·8% (-98 to -72·8%)
	Brain and central nervous system cancer	78832 (57161–101955)	162·9% (31·3 to 323·7%)	222·7 (161·1–285·4)	14·7% (-39·3 to 78·9%)
	Stroke*	604499 (471681–745962)	112·7% (62·5 to 175·2%)	2616·5 (2102·7–3132·9)	-20·5% (-38·2 to 0%)
	Ischaemic stroke	312868 (249314–376904)	151·1% (90·4 to 223·9%)	1579·4 (1289–1862·9)	-7·1% (-28·1 to 17·2%)
	Intracerebral haemorrhage	272077 (207250–348016)	97·1% (48 to 165·7%)	973·6 (760·3–1219·7)	-30·5% (-48 to -8·6%)
	Subarachnoid haemorrhage	19554 (13921–27517)	-9·4% (-42·4 to 54·8%)	63·4 (45·7–89·1)	-66·5% (-79·1 to -42·8%)
	Neurological disorders†	84150 (50537–154877)	103% (54 to 160·3%)	418·7 (207·7–864·7)	-5·2% (-22·9 to 15·3%)
	Alzheimer's disease and other dementias	40853 (9715–107617)	153·1% (109·1 to 209·2%)	271·4 (64·8–723·4)	0·3% (-16 to 21·3%)
	Parkinson's disease	12901 (10288–18484)	210·7% (139·7 to 303·4%)	75·1 (60·6–104·5)	19·2% (-7·3 to 53·8%)
	Idiopathic epilepsy	16599 (12416–21675)	10·7% (-22·1 to 78·1%)	38·7 (29·1–50·1)	-49·1% (-63·3 to -22·8%)
	Multiple sclerosis	1573 (1093–2283)	219·6% (103·3 to 437·3%)	4·7 (3·2–6·7)	0·8% (-35·9 to 66·6%)
	Motor neuron disease	422 (306–573)	197·8% (78·6 to 360·2%)	1·3 (0·9–1·8)	14·2% (-35·6 to 91%)
	Other neurological disorders	11802 (8983–14891)	113·1% (48·2 to 236·7%)	27·5 (21·2–34·5)	6% (-24·3 to 66·1%)
Jordan	Meningitis	4932 (3860–6486)	-6·7% (-36·5 to 38·4%)	44 (34·6–57·4)	-60·4% (-71·9 to -42·9%)
	Encephalitis	906 (661–1303)	-18·6% (-52·9 to 144·1%)	7·8 (5·8–11·4)	-67·4% (-80·4 to -9·5%)
	Tetanus	118 (87–174)	-71·8% (-86·8 to -12·3%)	1 (0·7–1·5)	-85·7% (-93·3 to -59%)
	Brain and central nervous system cancer	9563 (6825–11843)	199·4% (90 to 306%)	96·1 (68·3–118·7)	-10·9% (-44·2 to 21·4%)
	Stroke*	65603 (54338–78113)	111·1% (67·1 to 162%)	1163·5 (954·9–1374·6)	-54·1% (-63·2 to -43·8%)

Table S12 Burden, mortality, incidence, and prevalence of neurological conditions in North Africa and Middle East countries

		All Ages		Age-standardised	
		Number	Percent change	Rate per 100,000	Percent change
**Measure**		2019	From 1990 to 2019	2019	From 1990 to 2019
Cause	Location	Mean (95% UI)	Mean (95% UI)	Mean (95% UI)	Mean (95% UI)
	Ischaemic stroke	43269 (35599–51125)	136·7% (84·1 to 191·8%)	840·5 (689·2–990·8)	-50·6% (-61·4 to -39·3%)
	Intracerebral haemorrhage	20109 (16625–24114)	73·5% (36·2 to 118·7%)	294·9 (244·2–353)	-61·7% (-69·8 to -52%)
	Subarachnoid haemorrhage	2224 (1725–2759)	84·9% (27·4 to 189·2%)	28·1 (20·8–34·8)	-57·8% (-71 to -34·1%)
	Neurological disorders†	20443 (12115–38987)	234·3% (153 to 323·6%)	384 (185–824·8)	-12·8% (-28·2 to 7·1%)
	Alzheimer's disease and other dementias	10349 (2456–28594)	367·9% (287·4 to 478·3%)	258·1 (63–715·8)	-7·7% (-22·3 to 14·6%)
	Parkinson's disease	2934 (2457–3511)	321·2% (232·5 to 428·2%)	63·3 (52·9–75·2)	-16·4% (-33·5 to 4·8%)
	Idiopathic epilepsy	3660 (2795–4476)	85·1% (36·2 to 165·2%)	30·9 (23·6–37·7)	-40·4% (-55·1 to -15%)
	Multiple sclerosis	706 (422–922)	297% (146 to 459%)	6·9 (4·3–8·9)	-15·7% (-45·8 to 17·1%)
	Motor neuron disease	273 (202–379)	493·6% (282·5 to 804·1%)	2·6 (2–3·6)	45·7% (-6·6 to 129·7%)
	Other neurological disorders	2522 (2050–3103)	150·6% (78·6 to 250·6%)	22·1 (18–27·1)	-4·5% (-29·8 to 29·7%)
Kuwait	Meningitis	589 (460–747)	-33·4% (-51·4 to -11·7%)	16·5 (12·7–21·1)	-67% (-75·7 to -56·6%)
	Encephalitis	321 (260–422)	38·3% (9·1 to 81%)	8·8 (7·1–11·4)	-30·4% (-44·7 to -10·3%)
	Tetanus	1 (0–3)	84·7% (-77·8 to 686·7%)	0 (0–0·1)	-47·7% (-91 to 74·2%)
	Brain and central nervous system cancer	2405 (1687–3006)	134·1% (43·7 to 198·9%)	64·3 (45·7–80·2)	-10% (-45·1 to 14·8%)
	Stroke*	19183 (15796–23106)	224·1% (165·5 to 293·9%)	749 (616·5–896·1)	-12·8% (-27·8 to 5·7%)
	Ischaemic stroke	10550 (8664–12861)	246·8% (184·7 to 326·6%)	492·2 (402·7–595·2)	-13·3% (-28·9 to 5·9%)
	Intracerebral haemorrhage	7371 (5899–8995)	254·6% (175·8 to 343·4%)	220·2 (175·7–269·5)	-4% (-25·4 to 20·7%)
	Subarachnoid haemorrhage	1262 (1010–1605)	58% (17·6 to 159%)	36·6 (29·5–46·1)	-41% (-55·4 to -13·9%)
	Neurological disorders†	8076 (4291–16107)	165·2% (85·4 to 248·6%)	378·8 (172·1–818·2)	-15·4% (-30·9 to -2·5%)
	Alzheimer's disease and other dementias	4815 (1207–12607)	379·4% (320·7 to 456·3%)	271 (67·9–731)	-3·9% (-15·3 to 10%)
	Parkinson's disease	820 (675–986)	202·2% (154·4 to 263%)	46 (37·5–55·1)	-29·8% (-40·8 to -16·3%)
	Idiopathic epilepsy	1457 (1202–1833)	38·3% (14·1 to 78·1%)	35·5 (29·4–43·8)	-37·1% (-47·8 to -20·3%)
	Multiple sclerosis	193 (145–275)	282·6% (188·1 to 408·8%)	3·9 (2·9–5·5)	-1·4% (-26·3 to 31·7%)
	Motor neuron disease	178 (124–227)	-23·4% (-47·6 to 7·8%)	5·3 (3·7–6·8)	-64·4% (-75·2 to -50·4%)
	Other neurological disorders	613 (499–758)	41·3% (4·8 to 85·4%)	17·1 (13·7–21·3)	-31·5% (-48·1 to -10·8%)
Lebanon	Meningitis	1231 (870–1677)	-64·2% (-77 to -45·1%)	23·8 (16·8–32·5)	-72·3% (-81·8 to -59·2%)
	Encephalitis	706 (422–1379)	8·8% (-34·1 to 66·7%)	13·7 (8·2–26·8)	-19·5% (-48·9 to 19·7%)
	Tetanus	766 (166–2656)	-57·9% (-93 to 27·8%)	14·9 (3·2–50·9)	-75·8% (-95·7 to -29·1%)
	Brain and central nervous system cancer	5929 (4138–7638)	57·1% (5·6 to 115·1%)	114·1 (80·2–146·9)	-10·5% (-39·7 to 21·6%)
	Stroke*	27417 (19332–35906)	45·3% (2·7 to 96·7%)	528·3 (372·5–692·1)	-42·3% (-59·3 to -21·3%)
	Ischaemic stroke	21101 (14261–27359)	116·3% (46·7 to 187%)	407·8 (275·3–528·6)	-22·7% (-46·2 to 3·1%)
	Intracerebral haemorrhage	5430 (3796–7649)	-27·2% (-50·1 to 4·9%)	103·7 (72·5–145·4)	-67·9% (-77·7 to -53·8%)
	Subarachnoid haemorrhage	887 (503–1337)	-46·5% (-73·5 to -0·1%)	16·8 (9·5–25·3)	-73·7% (-87·1 to -50·4%)
	Neurological disorders†	21112 (10304–45979)	130·7% (67·3 to 224·2%)	417·9 (201·9–909·6)	-9·8% (-26·4 to 24·9%)
	Alzheimer's disease and other dementias	14005 (3289–38760)	201·8% (146·8 to 347·3%)	281·8 (67·2–770·7)	-4·4% (-20·7 to 40·6%)
	Parkinson's disease	3093 (2474–4206)	135·7% (69·9 to 219·5%)	58·7 (46·9–79·9)	-17·4% (-40·2 to 11·5%)
	Idiopathic epilepsy	2148 (1537–2988)	-7·7% (-34·7 to 38·7%)	41·5 (29·8–57·5)	-39·6% (-56·7 to -9·9%)
	Multiple sclerosis	531 (274–778)	116% (38·7 to 240·2%)	9·9 (5·1–14·5)	7·4% (-32 to 68·3%)
	Motor neuron disease	138 (81–227)	96·5% (12·6 to 215·5%)	2·7 (1·6–4·4)	4·9% (-40·2 to 68·1%)
	Other neurological disorders	1197 (865–1618)	115·2% (50 to 201·4%)	23·4 (16·9–31·7)	37·4% (-3·3 to 90·7%)
Libya	Meningitis	1837 (1380–2396)	-72·1% (-81·8 to -58·5%)	31·2 (23·6–40·4)	-72·9% (-81·3 to -61·5%)
	Encephalitis	872 (571–1766)	-4·4% (-39·8 to 44·2%)	15·2 (9·9–29·6)	-15·9% (-43 to 23·6%)
	Tetanus	66 (44–116)	-69·7% (-88·1 to -8·1%)	1·2 (0·7–2)	-70·5% (-89·8 to -19%)

Table S12 Burden, mortality, incidence, and prevalence of neurological conditions in North Africa and Middle East countries

		**All Ages**		**Age-standardised**	
		Number	Percent change	Rate per 100,000	Percent change
**Measure**		2019	From 1990 to 2019	2019	From 1990 to 2019
Cause	Location	Mean (95% UI)	Mean (95% UI)	Mean (95% UI)	Mean (95% UI)
	Brain and central nervous system cancer	8520 (6244–11164)	70·9% (15·5 to 160·1%)	132·5 (97·6–173·2)	-5% (-32·8 to 36·5%)
	Stroke*	68578 (51189–90565)	39·1% (0·8 to 99·7%)	1322·4 (1000·5–1742·3)	-25·6% (-43·4 to 0·2%)
	Ischaemic stroke	44157 (32728–59668)	152·8% (85·7 to 252·6%)	912·8 (682·1–1217·5)	9% (-17·8 to 46·7%)
	Intracerebral haemorrhage	20726 (15025–28745)	-6·5% (-34·2 to 39·9%)	347·2 (254–476·4)	-52·7% (-65·3 to -34·7%)
	Subarachnoid haemorrhage	3696 (2486–5548)	-61·8% (-77·8 to -10·3%)	62·4 (42·4–92·6)	-69·6% (-80·3 to -45·5%)
	Neurological disorders†	21901 (11896–42823)	102% (46·8 to 168·6%)	473·7 (234·6–985·4)	-4·9% (-24·5 to 21%)
	Alzheimer's disease and other dementias	12239 (2972–33364)	159·2% (106·9 to 231·1%)	305·3 (74·8–829·1)	-5% (-23·6 to 21·6%)
	Parkinson's disease	3163 (2321–4165)	206·1% (123·4 to 324·3%)	73 (53·5–95·8)	10·7% (-18·9 to 51·8%)
	Idiopathic epilepsy	3777 (2759–5119)	-5·7% (-33·4 to 41·7%)	55·4 (39·9–75·2)	-32·9% (-50·9 to -3·5%)
	Multiple sclerosis	836 (411–1423)	276·4% (125·3 to 571%)	11·3 (5·5–19)	39·3% (-20 to 143·8%)
	Motor neuron disease	209 (115–327)	260% (70·3 to 566·3%)	3·2 (1·8–4·9)	50·1% (-29·3 to 183·3%)
	Other neurological disorders	1677 (1252–2176)	109·8% (36 to 234·8%)	25·6 (19·3–33·1)	39·9% (-6·8 to 117·6%)
Morocco	Meningitis	19636 (13062–27667)	-84·5% (-90·6 to -76%)	61·6 (40·9–87·5)	-83·3% (-89·8 to -74·7%)
	Encephalitis	7434 (4105–15000)	-11·2% (-49·8 to 40·9%)	22·9 (12·6–45·6)	-18·4% (-51·2 to 26·5%)
	Tetanus	7666 (1913–28936)	-95% (-98·8 to -80·8%)	25·7 (6·3–98·1)	-93·9% (-98·5 to -76·2%)
	Brain and central nervous system cancer	19397 (12986–26757)	56·4% (-9·8 to 133·7%)	54·6 (36·9–73·8)	2·3% (-37·4 to 47·3%)
	Stroke*	581054 (451454–721688)	66·8% (27·8 to 110·2%)	1992·7 (1584·1–2446·6)	-19·4% (-37·5 to 0·5%)
	Ischaemic stroke	381444 (298001–470143)	172·2% (101·8 to 245·1%)	1380·6 (1094·3–1686·2)	19·5% (-9·3 to 48%)
	Intracerebral haemorrhage	169997 (126492–220310)	1·6% (-27·3 to 38·3%)	521·4 (392·9–671)	-52% (-65·3 to -34·6%)
	Subarachnoid haemorrhage	29612 (19446–45136)	-27·5% (-51·9 to 25·2%)	90·6 (61–134·7)	-60·6% (-74·2 to -30·8%)
	Neurological disorders†	108631 (55917–219853)	107·6% (62·2 to 147%)	430·1 (205·5–908·5)	3·7% (-13·6 to 22%)
	Alzheimer's disease and other dementias	61451 (13881–170845)	147·5% (105·1 to 196·1%)	274·9 (62·5–760·6)	0·5% (-15·5 to 18·8%)
	Parkinson's disease	22310 (17671–26335)	228·2% (160·1 to 323·9%)	86·1 (69–100·3)	42·6% (14 to 83·9%)
	Idiopathic epilepsy	14226 (5014–37834)	-5·2% (-40 to 50·5%)	39·2 (13·8–104·9)	-30·8% (-54·5 to 4·4%)
	Multiple sclerosis	3584 (1808–5653)	151·9% (53·5 to 315·9%)	9·7 (4·9–15·2)	26·3% (-22 to 105·7%)
	Motor neuron disease	720 (505–991)	158·1% (67·8 to 277·2%)	2 (1·4–2·7)	30·1% (-17·5 to 90·3%)
	Other neurological disorders	6339 (4554–8577)	58·9% (12·2 to 128·7%)	18·2 (13·1–24·5)	21·5% (-12·2 to 69·2%)
Palestine	Meningitis	1624 (1290–2076)	-79% (-86·9 to -66·3%)	32·8 (26·9–40)	-85% (-89·8 to -77·9%)
	Encephalitis	622 (448–937)	33·7% (-22 to 111·2%)	12·1 (9·1–17·7)	-26·7% (-52·2 to 5·4%)
	Tetanus	71 (51–106)	-90·5% (-97·4 to -52·6%)	1·3 (0·9–1·9)	-92·7% (-98 to -67·8%)
	Brain and central nervous system cancer	8782 (6846–10894)	113·7% (30·7 to 199·1%)	228·8 (173·5–275·6)	-9·4% (-40·1 to 23·6%)
	Stroke*	38561 (33005–43892)	54% (18·4 to 97·9%)	1890·3 (1632·7–2140·5)	-35% (-49·6 to -16·4%)
	Ischaemic stroke	26078 (22065–29807)	92·3% (46·2 to 150%)	1387·8 (1182·5–1583·7)	-19·8% (-38 to 3·7%)
	Intracerebral haemorrhage	11325 (9548–13292)	7% (-18·3 to 42·8%)	462 (389·4–544)	-57·7% (-67·7 to -43·6%)
	Subarachnoid haemorrhage	1157 (941–1393)	29% (-10·1 to 98·3%)	40·6 (33–48·7)	-51% (-66·6 to -25·4%)
	Neurological disorders†	10435 (7161–18412)	78·8% (37·8 to 135·8%)	460·6 (252·7–984·1)	-12·5% (-30·3 to 10·9%)
	Alzheimer's disease and other dementias	4139 (955–11921)	111·9% (74 to 165·2%)	276·4 (63·8–791·3)	-6·6% (-22·5 to 16·2%)
	Parkinson's disease	1396 (932–1626)	123·3% (67·5 to 219·4%)	82·1 (54·5–95·3)	-5·7% (-29·1 to 33·6%)
	Idiopathic epilepsy	3522 (2976–4335)	36·1% (-0·9 to 123%)	71·6 (60·9–86·9)	-36·9% (-52·6 to -3·6%)
	Multiple sclerosis	340 (259–458)	216·9% (81·8 to 464·4%)	9 (6·9–12·8)	0% (-39·5 to 73·8%)
	Motor neuron disease	59 (47–74)	239% (117·3 to 435·5%)	1·5 (1·2–1·9)	45·5% (-11·5 to 143·9%)
	Other neurological disorders	979 (804–1191)	79·5% (13·7 to 211·1%)	20 (16·6–23·7)	-0·7% (-33·2 to 67·7%)

Table S12 Burden, mortality, incidence, and prevalence of neurological conditions in North Africa and Middle East countries

		All Ages		Age-standardised	
		Number	Percent change	Rate per 100,000	Percent change
**Measure**		2019	From 1990 to 2019	2019	From 1990 to 2019
Cause	Location	Mean (95% UI)	Mean (95% UI)	Mean (95% UI)	Mean (95% UI)
Oman	Meningitis	637 (532–797)	-66% (-76·5 to -49·6%)	22·9 (19·3–27·9)	-74·9% (-81·3 to -64·3%)
	Encephalitis	4369 (3162–7628)	6·2% (-33 to 66·1%)	119·1 (91·1–177·2)	-44·5% (-63 to -14·1%)
	Tetanus	40 (32–50)	-79·7% (-93·8 to -19·4%)	1 (0·7–1·3)	-90·3% (-96·7 to -62·2%)
	Brain and central nervous system cancer	2460 (1421–3200)	148% (25·1 to 276·2%)	71 (42·8–89·6)	9·7% (-45·2 to 63·5%)
	Stroke*	25335 (21843–30592)	13·8% (-13·2 to 53·4%)	1647·1 (1439·9–1874·5)	-41·5% (-55 to -21·9%)
	Ischaemic stroke	13115 (11032–15498)	61·9% (19·4 to 124·2%)	1093·5 (932·1–1272·3)	-20·6% (-39·3 to 8·4%)
	Intracerebral haemorrhage	9838 (8377–12433)	-7·1% (-29·6 to 28·1%)	473 (407·1–592·1)	-60·9% (-70·6 to -47·4%)
	Subarachnoid haemorrhage	2383 (1623–3970)	-33·3% (-61·6 to 47·5%)	80·6 (51·4–125·1)	-64·4% (-79 to -30·7%)
	Neurological disorders†	5047 (3089–9368)	118·9% (77·3 to 181·2%)	462·8 (225·9–999·7)	1·4% (-15·7 to 39·5%)
	Alzheimer's disease and other dementias	2406 (559–6695)	121·4% (82·1 to 204·3%)	308·6 (71–852·2)	-4·7% (-21·2 to 39·9%)
	Parkinson's disease	899 (595–1047)	160·2% (84 to 258·2%)	109·7 (65–128·8)	30·8% (-5·2 to 77%)
	Idiopathic epilepsy	655 (527–911)	29·1% (-13·6 to 138·5%)	15 (12·4–19·6)	-43·2% (-60·6 to -5·2%)
	Multiple sclerosis	336 (159–518)	258·7% (74·9 to 559·7%)	8·2 (3·9–13·5)	10·8% (-46·2 to 103·4%)
	Motor neuron disease	76 (46–106)	218·5% (67·6 to 419·9%)	2·7 (1·4–3·9)	20·7% (-36·9 to 109·9%)
	Other neurological disorders	676 (545–813)	171·9% (63·8 to 318·8%)	18·7 (15–22·9)	45·4% (-9·7 to 120·5%)
Qatar	Meningitis	280 (212–367)	-17·1% (-48·5 to 28·8%)	17·5 (13·4–22·3)	-79·3% (-86·8 to -69·8%)
	Encephalitis	237 (169–351)	238·1% (108·4 to 412·1%)	11·9 (8·7–16·3)	-31·3% (-56·3 to 1·4%)
	Tetanus	17 (13–23)	195·1% (8·3 to 463·4%)	0·7 (0·5–1)	-50·2% (-83·3 to 5%)
	Brain and central nervous system cancer	1643 (1097–2724)	422·6% (204·5 to 702·6%)	83·1 (59·5–125·1)	-21·6% (-53·8 to 18·8%)
	Stroke*	5899 (4347–7879)	187·7% (103·6 to 298·9%)	722·3 (572·2–941)	-48·2% (-62 to -31%)
	Ischaemic stroke	1687 (1251–2371)	199% (99 to 324·1%)	433·9 (344·6–570·4)	-38·7% (-55·7 to -18%)
	Intracerebral haemorrhage	2850 (2061–3897)	174·5% (83·4 to 292·8%)	215 (165·9–284·7)	-58·4% (-70·5 to -42·9%)
	Subarachnoid haemorrhage	1362 (958–1847)	203·9% (73·8 to 389·9%)	73·4 (53·3–97·8)	-56·9% (-74·6 to -27·3%)
	Neurological disorders†	2441 (1551–4070)	370·4% (237·2 to 547·8%)	482·8 (243·5–1022·1)	0·2% (-21·4 to 25·4%)
	Alzheimer's disease and other dementias	865 (194–2513)	586·4% (396·5 to 851·4%)	296·7 (70·2–820·8)	3·7% (-16·9 to 28·2%)
	Parkinson's disease	377 (251–523)	463·5% (290·7 to 692·3%)	132·3 (80·5–177·6)	14·4% (-14·8 to 53·3%)
	Idiopathic epilepsy	617 (465–845)	237% (146·6 to 406·7%)	22·6 (17·5–30)	-45·8% (-59·9 to -19·9%)
	Multiple sclerosis	75 (51–112)	594·9% (327·6 to 922·8%)	2·7 (1·9–3·7)	-18·7% (-49·7 to 21·5%)
	Motor neuron disease	30 (21–41)	612·3% (342·6 to 1009·8%)	1·5 (1·1–2·1)	-7·9% (-45·3 to 50·3%)
	Other neurological disorders	478 (362–650)	273·2% (153·8 to 481·7%)	27 (20·7–35·8)	-18·7% (-43·7 to 23·2%)
Saudi Arabia	Meningitis	7453 (5506–10113)	-46·8% (-61·4 to -25%)	23·7 (18·4–30·4)	-75·2% (-81·4 to -65·4%)
	Encephalitis	8782 (6507–11985)	-2·1% (-33·1 to 48%)	26·4 (19·8–35·6)	-54·5% (-69·8 to -31%)
	Tetanus	749 (245–1293)	-94·2% (-99·2 to -68·2%)	2·1 (0·8–3·5)	-96·5% (-99·4 to -81·2%)
	Brain and central nervous system cancer	25834 (18794–38905)	245·2% (88·5 to 548·5%)	77·6 (57·8–117·9)	30·4% (-30·5 to 145·4%)
	Stroke*	348685 (259472–437865)	116·2% (51·2 to 209·1%)	1815·5 (1408·2–2165·5)	-32·8% (-51 to -7·4%)
	Ischaemic stroke	163122 (119550–201296)	136·7% (62·8 to 229·7%)	1078·7 (809·9–1276·7)	-21·7% (-43 to 4·6%)
	Intracerebral haemorrhage	171876 (126809–221167)	106·7% (41 to 213·9%)	695·8 (533·8–855·4)	-43·4% (-60·1 to -16%)
	Subarachnoid haemorrhage	13687 (9191–19190)	48% (-17·9 to 162%)	41 (28·5–55·1)	-57% (-76·5 to -24·9%)
	Neurological disorders†	72163 (48100–118902)	115% (55·8 to 200·1%)	479·1 (260·8–954·7)	-5·6% (-25·5 to 29·5%)
	Alzheimer's disease and other dementias	23419 (5586–63721)	120·5% (73·4 to 215·2%)	280·3 (67–747·3)	-3·2% (-21·1 to 39·5%)
	Parkinson's disease	9658 (7640–11493)	133% (72·6 to 247·2%)	96·2 (69·4–113·5)	-1·5% (-25 to 46·7%)
	Idiopathic epilepsy	29806 (21684–41678)	108·2% (24 to 251·9%)	73·8 (55·9–100·8)	-19·6% (-51·6 to 32·3%)
	Multiple sclerosis	1717 (1153–2595)	365·1% (151·5 to 1122·9%)	4·3 (3–6·2)	19·2% (-34·5 to 205·4%)

Table S12 Burden, mortality, incidence, and prevalence of neurological conditions in North Africa and Middle East countries

		**All Ages**		**Age-standardised**	
		Number	Percent change	Rate per 100,000	Percent change
**Measure**		2019	From 1990 to 2019	2019	From 1990 to 2019
Cause	Location	Mean (95% UI)	Mean (95% UI)	Mean (95% UI)	Mean (95% UI)
	Motor neuron disease	744 (495–1102)	247·2% (68·1 to 594·8%)	2·6 (1·8–3·7)	22·5% (-40·3 to 166·4%)
	Other neurological disorders	6819 (5187–9015)	74·6% (5·7 to 198·8%)	22 (17·5–27·6)	-3·2% (-39·1 to 61·5%)
Syrian Arab Republic	Meningitis	12273 (9255–15997)	-81·4% (-87·6 to -72·5%)	91·8 (68·7–121)	-72% (-80·9 to -59·6%)
	Encephalitis	3164 (2336–4375)	-57·2% (-74·4 to -20·4%)	23·7 (17·6–33)	-44·1% (-65·5 to -0·8%)
	Tetanus	307 (169–626)	-97·6% (-99·2 to -89·5%)	2·4 (1·3–5)	-95·6% (-98·6 to -81·4%)
	Brain and central nervous system cancer	20098 (14445–27220)	33·6% (-23·6 to 106·3%)	142·6 (103–191·1)	-7·3% (-38·8 to 36·5%)
	Stroke*	208285 (157650–274015)	-11·7% (-36 to 23·3%)	1784 (1385·4–2304·3)	-42·5% (-58·2 to -20·6%)
	Ischaemic stroke	93713 (70730–121893)	34·6% (-5 to 89·5%)	887·1 (687–1136·2)	-27·8% (-49·7 to -0·9%)
	Intracerebral haemorrhage	100600 (75708–135767)	-13·1% (-38·9 to 24·2%)	796·9 (605·4–1057·1)	-49·2% (-63·8 to -28·7%)
	Subarachnoid haemorrhage	13972 (10358–17842)	-72·3% (-82 to -52·7%)	100 (75·4–126)	-67·5% (-77·7 to -49·3%)
	Neurological disorders†	36742 (18448–76713)	62% (10·4 to 128·3%)	410·7 (188·7–907·5)	0·5% (-23·9 to 36·2%)
	Alzheimer's disease and other dementias	21880 (4846–61900)	113% (60·9 to 198·9%)	280·2 (65·9–791)	4% (-19·1 to 47·9%)
	Parkinson's disease	6589 (4310–8576)	157·3% (86·7 to 255·3%)	72·5 (47·6–93·1)	14·2% (-15·7 to 56·3%)
	Idiopathic epilepsy	4249 (3218–5691)	-25·6% (-49·9 to 35·1%)	29·3 (22·2–39)	-37·3% (-56·7 to 10·3%)
	Multiple sclerosis	642 (441–882)	104·2% (23 to 241·6%)	4·4 (3–6)	1·1% (-37·4 to 69·5%)
	Motor neuron disease	346 (204–566)	138·8% (32·1 to 304·8%)	2·3 (1·4–3·8)	31·1% (-26·3 to 121·1%)
	Other neurological disorders	3035 (2315–3951)	-17·4% (-46·6 to 33·4%)	22 (16·8–28·7)	-4·7% (-35 to 50·1%)
Tunisia	Meningitis	3291 (2322–4396)	-79·9% (-87·6 to -68·2%)	31·3 (21·8–41·7)	-80% (-87·5 to -69·4%)
	Encephalitis	1616 (974–3338)	-25·7% (-57·6 to 15·8%)	15·4 (9·1–31·5)	-31·5% (-59·2 to 5·4%)
	Tetanus	106 (70–195)	-83·7% (-94 to -56·6%)	1 (0·7–1·8)	-83·8% (-94 to -57·1%)
	Brain and central nervous system cancer	4979 (3286–6824)	42·9% (-16·3 to 111%)	41 (27–56)	-6·3% (-42·7 to 36·2%)
	Stroke*	151958 (112873–200799)	73·3% (26·9 to 133·5%)	1278·1 (957·8–1669)	-28·9% (-48 to -4·6%)
	Ischaemic stroke	106821 (79854–140307)	175·1% (94·2 to 273·1%)	919·3 (689·9–1202·8)	0·9% (-28 to 35·7%)
	Intracerebral haemorrhage	38206 (27649–51027)	-1·2% (-31·3 to 40%)	303·9 (221·8–403·7)	-58% (-70·6 to -40%)
	Subarachnoid haemorrhage	6932 (4647–9826)	-32% (-58·1 to 19·4%)	54·9 (37–77)	-66·3% (-79·7 to -41·1%)
	Neurological disorders†	45075 (20976–96180)	103·5% (31·5 to 186·1%)	416·5 (189·5–892·1)	-9·4% (-31·7 to 20·7%)
	Alzheimer's disease and other dementias	29927 (7064–79849)	208·9% (135·9 to 338·8%)	285·2 (67–745·2)	-3·3% (-25 to 34·3%)
	Parkinson's disease	6947 (5059–9387)	206·4% (125 to 308·2%)	61·4 (44·9–82·7)	10·6% (-18·1 to 45·8%)
	Idiopathic epilepsy	4677 (3260–6549)	-42·6% (-62·6 to -8·6%)	41·2 (28·8–57·6)	-50·9% (-67·6 to -23·8%)
	Multiple sclerosis	1255 (641–1900)	150·6% (52·4 to 321·6%)	9·5 (4·8–14·4)	23·7% (-25·4 to 102·9%)
	Motor neuron disease	275 (181–402)	135·3% (34·9 to 295·4%)	2·1 (1·4–3)	19·7% (-31·3 to 104%)
	Other neurological disorders	1994 (1435–2726)	40% (-9·2 to 106·1%)	17·1 (12·3–23·3)	8·7% (-28·7 to 55·2%)
Türkiye	Meningitis	15983 (13179–19410)	-91·4% (-94·7 to -86·4%)	24·4 (20–30·4)	-90·3% (-94·1 to -85%)
	Encephalitis	10569 (8148–13138)	-50·2% (-70·5 to -21·4%)	15·6 (11·8–19·6)	-51·4% (-70·6 to -23·6%)
	Tetanus	710 (542–960)	-98% (-98·9 to -94·1%)	1 (0·8–1·4)	-98·3% (-99 to -95·6%)
	Brain and central nervous system cancer	130370 (62327–180115)	23·2% (-35·1 to 91·7%)	155·2 (75·2–211·6)	-21·2% (-55·8 to 17·8%)
	Stroke*	825066 (660411–1008282)	70·3% (22·4 to 119·6%)	971·3 (778·9–1182·1)	-25·9% (-46·7 to -4·8%)
	Ischaemic stroke	424464 (337531–519987)	138·3% (59·8 to 211·6%)	516·9 (411·8–632·2)	-8·6% (-38·7 to 19·9%)
	Intracerebral haemorrhage	320059 (253584–397583)	34·2% (-3·3 to 74·6%)	364·9 (289·8–451·6)	-38·3% (-56·1 to -20·1%)
	Subarachnoid haemorrhage	80543 (61565–103081)	18·9% (-27·9 to 116·3%)	89·5 (69·3–113·8)	-41·8% (-64·7 to 8·3%)
	Neurological disorders†	398180 (225184–780441)	30·7% (-14·4 to 93·1%)	511 (293·3–978·2)	-22·8% (-39·9 to -2%)
	Alzheimer's disease and other dementias	220938 (55718–628880)	175·4% (126 to 237·4%)	278·5 (70·2–786·3)	-4·3% (-21·4 to 16·6%)

Table S12 Burden, mortality, incidence, and prevalence of neurological conditions in North Africa and Middle East countries

		All Ages		Age-standardised	
		Number	Percent change	Rate per 100,000	Percent change
**Measure**		2019	From 1990 to 2019	2019	From 1990 to 2019
Cause	Location	Mean (95% UI)	Mean (95% UI)	Mean (95% UI)	Mean (95% UI)
	Parkinson's disease	51886 (39341–96417)	136·8% (82·2 to 201%)	63·8 (48·4–119·1)	-12·3% (-31·8 to 10·9%)
	Idiopathic epilepsy	60568 (50170–72791)	-48·2% (-64·1 to 6·5%)	82·5 (67·7–98·8)	-51·1% (-66·2 to -1·7%)
	Multiple sclerosis	7654 (5547–11248)	30·3% (-42·8 to 137·8%)	8·5 (5·9–13·9)	-27% (-62·8 to 40·7%)
	Motor neuron disease	22902 (17977–28750)	-15·7% (-55·2 to 50·7%)	31 (23·8–39·6)	-25·6% (-58·5 to 21·2%)
	Other neurological disorders	34231 (27915–41914)	-34·8% (-54·1 to -5·4%)	46·7 (38·8–56·7)	-38·7% (-56·1 to -12·2%)
United Arab Emirates	Meningitis	2532 (1538–3785)	16·6% (-31·4 to 70·4%)	28·5 (19–40·4)	-75·9% (-83·4 to -67·4%)
	Encephalitis	1309 (721–2801)	204·8% (88·7 to 370·6%)	16·8 (10·4–32)	-22·3% (-48·1 to 16·4%)
	Tetanus	1059 (485–1741)	-8% (-83·2 to 209·2%)	33·2 (8·4–54·9)	-86% (-96·4 to -46·5%)
	Brain and central nervous system cancer	14149 (8124–20956)	459·7% (269·5 to 712·1%)	149·4 (90·4–203·9)	-11% (-45·5 to 29·8%)
	Stroke*	76743 (52969–110388)	376·4% (212·6 to 600·9%)	1589·3 (1200–2106·9)	-50·2% (-63·3 to -33·3%)
	Ischaemic stroke	39999 (27917–55080)	582·3% (344·3 to 936·2%)	1125·8 (857·3–1440)	-38·6% (-54·7 to -17·6%)
	Intracerebral haemorrhage	31773 (20820–50238)	281·6% (130·5 to 495·2%)	411·1 (282·8–629·3)	-65·7% (-77·3 to -50·2%)
	Subarachnoid haemorrhage	4972 (2324–8880)	158·9% (42·8 to 384·3%)	52·3 (24·9–85·9)	-67·3% (-80 to -45·5%)
	Neurological disorders†	20257 (13568–29820)	482·3% (297·7 to 728·9%)	515·1 (295·4–990·3)	-9·1% (-27·7 to 15·9%)
	Alzheimer's disease and other dementias	3053 (668–8893)	606·8% (406·4 to 889·8%)	262·4 (62·9–724·5)	-7·2% (-22·3 to 25·2%)
	Parkinson's disease	2422 (1594–3367)	687·2% (456·3 to 1074·3%)	114·3 (70·7–151)	-13% (-33·7 to 23·8%)
	Idiopathic epilepsy	7030 (4322–10921)	257·4% (124·3 to 474·8%)	64·2 (41·6–96·7)	-36·9% (-57·1 to -3·7%)
	Multiple sclerosis	1995 (912–3569)	1081·6% (551·8 to 1757·5%)	14·3 (6–26·2)	31% (-26·2 to 94·8%)
	Motor neuron disease	498 (196–1005)	1091·1% (375·2 to 2343·4%)	5·3 (2–10·6)	24·9% (-47·6 to 142·8%)
	Other neurological disorders	5260 (3193–7965)	836·4% (460 to 1357·3%)	54·5 (33·9–79·3)	52·7% (-2·2 to 120·7%)
Yemen	Meningitis	30814 (20053–46016)	-57·8% (-73·6 to -32·2%)	90 (61·5–129·3)	-70% (-80·1 to -55·4%)
	Encephalitis	10340 (5678–20479)	45·6% (-19·9 to 167·3%)	27·8 (16·2–54·6)	-9·4% (-46·2 to 51·2%)
	Tetanus	4194 (1661–8779)	-85·7% (-95·7 to -34·5%)	11·2 (5–23·3)	-89·9% (-96·5 to -61·6%)
	Brain and central nervous system cancer	31328 (18560–46009)	136·6% (23·5 to 365·2%)	122·1 (74·2–179·5)	15% (-36 to 99·4%)
	Stroke*	348708 (262489–463408)	81·9% (35·4 to 149·9%)	2515·8 (1917–3278·3)	-26·4% (-43·7 to -2·2%)
	Ischaemic stroke	181690 (139380–241207)	234·9% (150·9 to 360%)	1513·8 (1168·7–2002)	20·4% (-6·8 to 57·4%)
	Intracerebral haemorrhage	139561 (101599–188052)	23% (-12·1 to 75·9%)	847·6 (623·1–1140·1)	-54·2% (-66·6 to -36·7%)
	Subarachnoid haemorrhage	27457 (13469–46026)	14·3% (-25·3 to 99·8%)	154·5 (76·3–264)	-50·2% (-65·3 to -20·4%)
	Neurological disorders†	59917 (37226–106735)	79% (15·7 to 196·2%)	437 (215·1–925·6)	-5·7% (-25·8 to 23%)
	Alzheimer's disease and other dementias	22724 (5231–64624)	204·8% (151 to 283·8%)	274·5 (63·9–761·5)	0·5% (-15·7 to 23·6%)
	Parkinson's disease	7114 (5460–9661)	218·6% (142·5 to 341·6%)	67·7 (52·8–89·4)	16·4% (-9·6 to 55·9%)
	Idiopathic epilepsy	23811 (15415–33812)	12·4% (-28·4 to 172·2%)	71·1 (46·5–100·1)	-36·9% (-58·2 to 23·2%)
	Multiple sclerosis	1745 (778–3095)	281·2% (93·8 to 661·2%)	7·9 (3·8–12·8)	30·5% (-29·5 to 133·4%)
	Motor neuron disease	296 (206–417)	214·8% (96·5 to 413·9%)	1·4 (1–2·1)	26·1% (-27·5 to 120·6%)
	Other neurological disorders	4227 (2849–6124)	106% (15·6 to 271·1%)	14·4 (10·1–20·4)	19% (-25·5 to 104·8%)
Afghanistan	Meningitis	115469 (82285–166403)	-32·8% (-56·4 to 4·7%)	225·5 (164·4–306·1)	-74·9% (-83·5 to -63·5%)
	Encephalitis	57729 (36789–113537)	153·3% (68·1 to 262·9%)	168·2 (124·6–241·3)	-15·1% (-39·9 to 17·6%)
	Tetanus	58099 (32883–101635)	-74·6% (-88·5 to -32·2%)	113·9 (70·4–179·3)	-90·2% (-94·9 to -79·6%)
	Brain and central nervous system cancer	54143 (28875–95299)	152·6% (56·9 to 371·5%)	174·6 (98·6–321·4)	-5·9% (-38·6 to 55·9%)
	Stroke*	466290 (332486–617437)	52% (14·2 to 96·2%)	3249·7 (2264·5–4235·5)	-23·2% (-41·6 to -4%)
	Ischaemic stroke	195531 (141603–268203)	134·5% (74·8 to 207·3%)	1675·8 (1191·4–2257·4)	28·6% (-2·2 to 64·4%)
	Intracerebral haemorrhage	231694 (166235–313924)	21·1% (-10 to 64·3%)	1348·6 (941·2–1813·7)	-46·5% (-60 to -29·7%)

Table S12 Burden, mortality, incidence, and prevalence of neurological conditions in North Africa and Middle East countries

		**All Ages**		**Age-standardised**	
		Number	Percent change	Rate per 100,000	Percent change
**Measure**		2019	From 1990 to 2019	2019	From 1990 to 2019
Cause	Location	Mean (95% UI)	Mean (95% UI)	Mean (95% UI)	Mean (95% UI)
	Subarachnoid haemorrhage	39065 (11765–68601)	22·2% (-15·8 to 93·1%)	225·4 (67·3–391·3)	-44·6% (-61 to -14·5%)
	Neurological disorders†	103133 (67039–153499)	61% (24·8 to 135·6%)	606·2 (341·8–1162·2)	-19·5% (-37·6 to 2·3%)
	Alzheimer's disease and other dementias	23448 (5538–64822)	52% (22·7 to 79·9%)	328·9 (79·5–892·5)	-4·1% (-20·5 to 10·9%)
	Parkinson's disease	10515 (7840–13740)	51·5% (14·8 to 96·2%)	104·2 (79·3–133·3)	-6·2% (-27·4 to 18·8%)
	Idiopathic epilepsy	56218 (28474–79484)	52·8% (5·4 to 208·8%)	132·6 (65·9–185·9)	-48·4% (-61·9 to -8·1%)
	Multiple sclerosis	4019 (1431–9775)	224·7% (96·9 to 486·4%)	16·3 (6·7–36·8)	6·8% (-31·4 to 74·9%)
	Motor neuron disease	490 (353–677)	126·7% (53·4 to 236·3%)	2·2 (1·5–3·2)	-8·2% (-39·2 to 41·1%)
	Other neurological disorders	8443 (5904–11671)	146·6% (63·7 to 282·3%)	22 (16·4–30·1)	-8·4% (-35·7 to 34%)
Sudan	Meningitis	45033 (29393–69598)	-80·4% (-87·9 to -69·3%)	96·3 (64·8–140·3)	-85·3% (-90·4 to -77·7%)
	Encephalitis	13643 (7364–25877)	10·8% (-42·9 to 121·2%)	28·8 (16·4–54·8)	-24% (-57·6 to 34·5%)
	Tetanus	2684 (1120–5577)	-92·9% (-97·4 to -77·6%)	5·6 (2·6–12·1)	-94·3% (-97·8 to -83·9%)
	Brain and central nervous system cancer	47975 (28840–70549)	69·9% (-20·1 to 292·2%)	134·4 (83·4–194·7)	-1·7% (-49 to 87·2%)
	Stroke*	461786 (328962–661040)	14·7% (-15·3 to 52·6%)	2309 (1690·7–3286·2)	-35·9% (-49·3 to -18·7%)
	Ischaemic stroke	256894 (187194–378036)	102·5% (50·2 to 167·8%)	1465·8 (1081·7–2132·7)	6·2% (-16·1 to 35·3%)
	Intracerebral haemorrhage	175218 (115804–250413)	-22·8% (-45·8 to 6·5%)	730·2 (488·8–1029)	-61·5% (-71·1 to -48·6%)
	Subarachnoid haemorrhage	29674 (15608–52961)	-39·2% (-59·5 to 28·9%)	113 (60·8–198·4)	-65·4% (-76·4 to -33·3%)
	Neurological disorders††	96769 (60909–163156)	21·2% (-24·2 to 109·6%)	451 (240·8–919·7)	-19% (-40·9 to 5·1%)
	Alzheimer's disease and other dementias	33865 (7813–93233)	105·1% (77·7 to 146·5%)	257 (59·6–730·3)	-5·5% (-17·2 to 11·7%)
	Parkinson's disease	11583 (8809–15062)	79·3% (32·6 to 148%)	75·6 (58·5–96·3)	-5% (-29·6 to 30·5%)
	Idiopathic epilepsy	38387 (24060–53514)	-24·7% (-56 to 107·4%)	84·4 (54·1–116·2)	-52·9% (-71·8 to 6%)
	Multiple sclerosis	2397 (981–4083)	175·4% (41·8 to 425·8%)	7·9 (3·5–12·6)	18·8% (-35·9 to 113·6%)
	Motor neuron disease	472 (328–674)	143·8% (50·9 to 286·3%)	1·7 (1·1–2·5)	20·5% (-28 to 97·1%)
	Other neurological disorders	10089 (6343–15109)	108·2% (7·4 to 272·2%)	24·3 (15·7–35·3)	34·3% (-24·9 to 114·5%)

Data in parentheses are 95% uncertainty intervals. Percentage data are rounded to one decimal place. Percentages and number of DALYs, YLDs, YLLs, deaths, incident cases, and prevalent cases are not mutually exclusive: the sum of percentages and number of DALYs, YLDs, YLLs, deaths, incident cases, and prevalent cases in the columns exceeds the totals for all causes combined because of overlap between various causes. Measures with insufficient data are not reported here. DALYs, Disability-Adjusted Life Years; UI, uncertainty interval; YLDs, Years Lived with Disability; YLLs, Years of Life Lost; * Stroke include ischaemic stroke, intracerebral haemorrhage, and subarachnoid haemorrhage; † Neurological disorders include Alzheimer's disease and other dementias, Parkinson's disease, idiopathic epilepsy, multiple sclerosis, headache disorders (including migraine and tension-type headache), motor neuron disease, and other neurological disorders.

Table S13 Disability, prevalence, and incidence of head and spinal injuries in North Africa and Middle East countries

(NEXT PAGE)

Table S13 Disability, prevalence, and incidence of head and spinal injuries in North Africa and Middle East countries

Location		All Ages			Age-standardised		
		Number	Percent change		Rate per 100,000	Percent change	
		2019	From 1990 to 2019		2019	From 1990 to 2019	
Cause	Measure	Mean (95% UI)	Mean (95% UI)	Rank	Mean (95% UI)	Mean (95% UI)	Rank
**YLDs (Years Lived with Disability)**							
Minor TBI	Global	1366897 (963637–1833553)	70·5% (66·9 to 74%)		16·9 (11·9–22·7)	-4·4% (-5·7 to -3%)	
	North Africa and Middle East	86593 (60649–118700)	120·2% (98·7 to 134·7%)		15·4 (10·9–21)	0·7% (-9·4 to 7·1%)	
	Algeria	4727 (3343–6407)	109·1% (84·5 to 139·4%)	5	11·8 (8·3–15·9)	-6·5% (-16·2 to 5·3%)	8
	Bahrain	182 (123–251)	316·5% (261·9 to 381%)	18	11·1 (7·6–15·1)	3·4% (-7·8 to 14·9%)	13
	Egypt	7891 (5463–10815)	112·3% (86·8 to 142%)	7	9·4 (6·5–12·8)	7·1% (-4·7 to 20·2%)	15
	Iran (Islamic Republic of)	11129 (8111–14902)	33·6% (11·9 to 54·5%)	2	12·9 (9·5–17·3)	-37·8% (-52·2 to -27%)	2
	Iraq	10092 (6104–16454)	115·5% (87·6 to 148·7%)	10	28·7 (17·4–47·3)	-26·7% (-39·5 to -12·7%)	3
	Jordan	947 (643–1309)	282·4% (236·3 to 331·8%)	15	9·5 (6·5–13)	-2·8% (-12·4 to 7·4%)	10
	Kuwait	581 (408–794)	135% (70·4 to 199·6%)	11	12·8 (9–17·3)	-20·7% (-37·8 to -5·4%)	4
	Lebanon	866 (558–1434)	10·6% (-10·9 to 43·9%)	1	16·4 (10·5–27·1)	-40·7% (-52·2 to -24·1%)	1
	Libya	1159 (809–1627)	201% (154·1 to 259·1%)	13	16·9 (11·8–23·5)	29·7% (10 to 56·5%)	19
	Morocco	4180 (2899–5741)	79·8% (60·4 to 99·3%)	3	11·9 (8·2–16·2)	0% (-9·2 to 9·5%)	11
	Palestine	997 (579–1687)	203·2% (163·2 to 253·3%)	14	25·4 (15·1–42·3)	20·4% (4·8 to 39·1%)	17
	Oman	594 (401–826)	187·2% (151·7 to 228·9%)	12	14·7 (10·1–20·3)	-7·1% (-16·1 to 3·1%)	7
	Qatar	429 (293–598)	570·2% (479·3 to 670·4%)	20	15·1 (10·4–20·8)	-10% (-19 to 0%)	6
	Saudi Arabia	8773 (6055–12173)	290·8% (251·8 to 331·7%)	16	25·6 (17·7–35·4)	24·5% (14·3 to 35·2%)	18
	Syrian Arab Republic	4487 (2355–8386)	381·2% (188 to 723%)	19	33·9 (17·7–64)	227·3% (88·4 to 477·2%)	21
	Tunisia	1421 (984–1965)	93% (72·6 to 115·7%)	4	11·3 (7·8–15·5)	2·3% (-7·9 to 13·4%)	12
	Türkiye	10034 (7184–13736)	113·9% (89·8 to 141·7%)	8	11·2 (8–15·3)	16·5% (5 to 30·4%)	16
	United Arab Emirates	1402 (943–1933)	573·3% (479·2 to 684·7%)	21	13·5 (9·2–18·4)	-5·9% (-14·6 to 4·2%)	9
	Yemen	4101 (2470–7070)	301·3% (202·1 to 461·8%)	17	17 (10·6–27·5)	45·5% (10·5 to 98·8%)	20
	Afghanistan	8250 (4003–16254)	115·1% (33·3 to 288·1%)	9	34·8 (16·1–71·7)	-14·9% (-41 to 39·6%)	5
	Sudan	4261 (2975–5853)	111·3% (81·6 to 143·4%)	6	14·5 (10·2–19·7)	4·1% (-6·5 to 16·5%)	14
Moderate/Severe TBI	Global	5710006 (4035548–7732980)	81·1% (78·5 to 84%)		69·7 (49·3–94·3)	1·3% (0 to 2·7%)	
	North Africa and Middle East	366440 (262928–489575)	126% (113·5 to 134·9%)		64·7 (46·6–86·2)	2·1% (-2·6 to 5·7%)	
	Algeria	25209 (17920–33941)	100·8% (89·2 to 114%)	4	62 (44·3–83·5)	-11·3% (-15·9 to -6·3%)	5
	Bahrain	1057 (739–1448)	323·1% (290·3 to 359·8%)	19	60·4 (42·7–82·4)	3·6% (-2·7 to 10·6%)	13
	Egypt	42450 (29730–57678)	107·1% (92·8 to 124%)	6	49·2 (34·5–66·6)	3·4% (-3·2 to 10·6%)	12
	Iran (Islamic Republic of)	59063 (43787–78802)	56·3% (42·3 to 68·7%)	2	67·2 (49·8–89·3)	-25·9% (-33·4 to -20·8%)	2
	Iraq	27218 (18768–38467)	145·2% (113·8 to 174·8%)	10	77·1 (53·4–107·8)	-17·1% (-29 to -6·5%)	4
	Jordan	4937 (3462–6753)	285·5% (258·6 to 315·4%)	17	49·9 (34·8–67·9)	-4·2% (-9·4 to 1·7%)	10
	Kuwait	3130 (2213–4237)	192·9% (172·4 to 214·4%)	14	67·8 (48·4–91·6)	-10·2% (-15·3 to -5%)	7
	Lebanon	3159 (2248–4421)	40·3% (8·9 to 67%)	1	59·3 (42·2–82·9)	-26·9% (-41·4 to -14·3%)	1
	Libya	5161 (3684–6943)	162·4% (143·6 to 185·4%)	11	74·2 (52·8–99·5)	6·3% (-0·6 to 15·8%)	16
	Morocco	22334 (15760–30222)	90·8% (79·1 to 102·5%)	3	62·1 (43·8–84·1)	3% (-2·8 to 8·8%)	11
	Palestine	2731 (1843–4038)	183·8% (156·8 to 209·1%)	12	71·5 (48·5–103·9)	6·9% (-1·5 to 13·9%)	17
	Oman	3652 (2537–5017)	198·1% (178·1 to 217·8%)	16	85·5 (60·2–116·3)	-5·1% (-9·6 to -0·1%)	8
	Qatar	2427 (1693–3334)	555·4% (509·1 to 605·4%)	20	82·1 (58·3–112·1)	-10·6% (-15·3 to -5·6%)	6
	Saudi Arabia	46302 (32141–64030)	315% (290·4 to 340·2%)	18	130·5 (91·7–179·2)	25% (19·6 to 31·1%)	20
	Syrian Arab Republic	11277 (7082–18353)	190·3% (112·9 to 363%)	13	82·5 (51·2–136·2)	80·6% (30·3 to 196·7%)	21
	Tunisia	7959 (5606–10868)	102·6% (90·6 to 115·6%)	5	61·7 (43·4–84·2)	3·8% (-1·7 to 9·7%)	14
	Türkiye	52808 (37969–71236)	124·6% (110·2 to 140·4%)	8	57·9 (41·6–78)	19·4% (12·4 to 27·3%)	19
	United Arab Emirates	8574 (5987–11786)	609% (552·6 to 668·7%)	21	73·8 (51·8–100·8)	-4·7% (-9·8 to 0·7%)	9
	Yemen	12987 (9126–17590)	195·8% (166·6 to 240·6%)	15	58·2 (41·4–78·6)	10·3% (-0·1 to 29·2%)	18
	Afghanistan	17161 (10831–28999)	114% (34 to 237·5%)	7	70·8 (43·7–124·1)	-19·7% (-44·9 to 17·1%)	3
	Sudan	6473 (4602–8820)	125·3% (103·1 to 148·6%)	9	22·5 (16·1–30·6)	5·5% (-3·4 to 15%)	15

Table S13 Disability, prevalence, and incidence of head and spinal injuries in North Africa and Middle East countries

Location		All Ages	Percent change		Age-standardised	Percent change	
		Number	Percent change		Rate per 100,000	Percent change	
**Location**		2019	From 1990 to 2019		2019	From 1990 to 2019	
Cause	Measure	Mean (95% UI)	Mean (95% UI)	Rank	Mean (95% UI)	Mean (95% UI)	Rank
Spinal cord lesion at neck level	Global	4253092 (3040199–5853314)	72·9% (59·4 to 81·2%)		52·3 (37·3–72·1)	4·1% (-2 to 10·1%)	
	North Africa and Middle East	478758 (220027–1142123)	85% (33·5 to 134·8%)		77·7 (36·2–184)	-3·6% (-28·8 to 18·6%)	
	Algeria	16112 (10072–27420)	198·8% (111·7 to 382%)	11	37·4 (23·7–62·4)	36·3% (-1·6 to 118·3%)	16
	Bahrain	507 (353–667)	297·9% (232·5 to 381·3%)	16	27·6 (19·3–35·9)	4·7% (-11 to 24·4%)	10
	Egypt	19407 (13662–25647)	121·4% (81·7 to 175·7%)	7	20·7 (14·7–27·2)	11·4% (-7·7 to 35·6%)	12
	Iran (Islamic Republic of)	45403 (26088–81294)	-8·2% (-31·9 to 36·5%)	2	48·3 (28·1–84·5)	-52·4% (-65·6 to -29·9%)	2
	Iraq	101161 (30457–290074)	76·1% (63·5 to 99·5%)	5	250·3 (76·5–713·1)	-29% (-37·7 to -18%)	4
	Jordan	2590 (1769–3456)	323·5% (249·5 to 427·2%)	17	24 (16·7–31·6)	8·9% (-8 to 31·2%)	11
	Kuwait	2085 (1288–3558)	248·5% (188·7 to 319·3%)	13	40·3 (25·6–66·9)	12·3% (-5·5 to 32·7%)	14
	Lebanon	6683 (1907–20012)	-30·7% (-39·1 to 0·8%)	1	124·9 (35·6–375·8)	-59·3% (-64·8 to -42·7%)	1
	Libya	6296 (2895–14626)	291·7% (199·9 to 372%)	15	85·2 (39·5–199·7)	92·9% (34·9 to 150·8%)	19
	Morocco	10298 (7376–13405)	55·9% (19·7 to 92·1%)	4	27·7 (19·8–35·9)	-7·4% (-25·3 to 10·4%)	6
	Palestine	12738 (3692–36518)	152·4% (126·9 to 173·8%)	8	294·1 (86·7–825·7)	12% (-1·2 to 20·9%)	13
	Oman	1503 (1036–1983)	215·3% (160·8 to 278·1%)	12	31·1 (21·6–40·7)	2% (-12·4 to 18·4%)	8
	Qatar	1086 (750–1434)	551·9% (436·1 to 676·7%)	20	32·9 (23·2–42·7)	-9·4% (-21·7 to 3·9%)	5
	Saudi Arabia	19302 (13513–25372)	288·1% (238·2 to 346·9%)	14	47·8 (33·5–61·8)	17·9% (3·9 to 33·1%)	15
	Syrian Arab Republic	54992 (14594–158293)	957·7% (407·5 to 2102·3%)	21	391·5 (102·9–1140·2)	734·9% (290·4 to 1616·7%)	21
	Tunisia	3261 (2300–4228)	86·9% (57·7 to 116·2%)	6	25·4 (18·1–32·9)	2·8% (-11·4 to 17·7%)	9
	Türkiye	29313 (19188–43188)	158·9% (104·8 to 244·2%)	9	31·9 (20·9–47·3)	47·9% (17·7 to 99·9%)	18
	United Arab Emirates	3664 (2562–4774)	541·4% (419·2 to 694%)	19	28·2 (20–36·2)	-7·3% (-20·9 to 8·8%)	7
	Yemen	24187 (9216–62658)	379·2% (246·7 to 490·5%)	18	87·2 (33·8–224·9)	101·8% (38·9 to 162·4%)	20
	Afghanistan	94455 (22346–283345)	25·1% (-25·6 to 315·1%)	3	325·6 (73·8–997·6)	-52·1% (-68·1 to 36·6%)	3
	Sudan	23229 (13788–46606)	180·4% (136·6 to 222·8%)	10	68·8 (41·8–132·9)	36·7% (12·7 to 61·4%)	17
Spinal cord lesion below neck level	Global	1947717 (1370638–2571462)	51·1% (44·7 to 57·6%)		23·9 (16·8–31·5)	-12% (-15·4 to -8·5%)	
	North Africa and Middle East	85431 (59750–113824)	88·1% (74·3 to 101·8%)		14·5 (10·2–19·3)	-14·1% (-20·4 to -7·9%)	
	Algeria	5431 (3738–7277)	62·3% (38·7 to 89·2%)	7	13 (9–17·5)	-25·5% (-35·4 to -14%)	5
	Bahrain	209 (145–285)	228% (170·4 to 302·9%)	18	11·7 (8·1–15·9)	-15·1% (-28·3 to 0·3%)	13
	Egypt	9565 (6545–12709)	68·2% (39·8 to 99·9%)	8	10·5 (7·2–13·9)	-15·4% (-29·6 to -0·5%)	12
	Iran (Islamic Republic of)	13521 (8937–19264)	48·5% (35·7 to 60·3%)	4	15 (10–21·4)	-28% (-34·3 to -22%)	3
	Iraq	5258 (3619–7084)	132·5% (98·8 to 172·3%)	13	14·3 (9·9–19·3)	-20·7% (-31·5 to -8·4%)	8
	Jordan	1088 (730–1489)	200·7% (150·9 to 256·9%)	17	10·4 (7·1–14·2)	-22·8% (-33·6 to -10·5%)	7
	Kuwait	570 (390–789)	168·6% (124·8 to 218·6%)	15	11·7 (8·1–16·2)	-15·8% (-27·5 to -2·6%)	11
	Lebanon	544 (372–742)	24·1% (4 to 45·4%)	1	10·2 (6·9–13·8)	-35·4% (-45·4 to -24·5%)	1
	Libya	1070 (744–1435)	130·5% (94·9 to 169·6%)	12	14·9 (10·5–19·7)	-3·6% (-16·7 to 11·2%)	19
	Morocco	5522 (3863–7341)	57·3% (33·8 to 81·8%)	6	15 (10·6–19·9)	-12·1% (-24·2 to 0·7%)	17
	Palestine	584 (389–850)	90·4% (28·8 to 168·5%)	9	14·9 (9·9–22·4)	-25·6% (-44·5 to -2·5%)	4
	Oman	613 (408–839)	134·1% (91·5 to 183·7%)	14	13·3 (9·1–17·9)	-24·6% (-36 to -11·4%)	6
	Qatar	439 (287–617)	396·5% (302·9 to 501·6%)	20	13·8 (9·3–19·1)	-32·1% (-42·1 to -21·3%)	2
	Saudi Arabia	8725 (5893–11997)	187·4% (146·5 to 235·9%)	16	22·8 (15·4–30·7)	-12·2% (-24 to 0·8%)	16
	Syrian Arab Republic	1673 (1173–2294)	47·4% (23·8 to 75·6%)	2	11·7 (8·2–16)	-6·4% (-19·9 to 9·5%)	18
	Tunisia	1456 (999–1987)	47·8% (25·3 to 72·8%)	3	11·3 (7·8–15·5)	-20·3% (-31·9 to -7·5%)	9
	Türkiye	10849 (7328–15191)	56·6% (28·6 to 89·6%)	5	11·8 (8–16·5)	-13·3% (-28·3 to 5%)	15
	United Arab Emirates	1800 (1237–2461)	482·4% (373·3 to 611%)	21	14·7 (10·3–19·6)	-17·5% (-29·3 to -5·1%)	10
	Yemen	3284 (2275–4342)	128·1% (92·4 to 171·5%)	11	13·7 (9·5–17·9)	-13·8% (-25·9 to 0%)	14
	Afghanistan	4434 (3065–6190)	251·3% (173·4 to 354·2%)	19	16·4 (11·4–23·2)	14·3% (-8·5 to 49·6%)	21
	Sudan	8709 (6089–11611)	108·5% (81·2 to 140·4%)	10	27·7 (19·5–36·5)	-3·4% (-14·8 to 9%)	20

Table S13 Disability, prevalence, and incidence of head and spinal injuries in North Africa and Middle East countries

		All Ages			Age-standardised		
		Number	Percent change		Rate per 100,000	Percent change	
**Location**		2019	From 1990 to 2019		2019	From 1990 to 2019	
Cause	Measure	Mean (95% UI)	Mean (95% UI)	Rank	Mean (95% UI)	Mean (95% UI)	Rank
Head Injuries	Global	7076902 (4997948–9588098)	79% (76·6 to 81·5%)		86·5 (61·1–117·2)	0·1% (-1 to 1·3%)	
	North Africa and Middle East	453033 (331629–604021)	124·9% (111·3 to 133·7%)		80·1 (58·6–106·9)	1·9% (-3·5 to 5·4%)	
	Algeria	29937 (21475–40072)	102·1% (90·9 to 114·9%)	5	73·8 (52·9–99)	-10·6% (-14·9 to -6%)	6
	Bahrain	1239 (871–1704)	322·1% (292 to 355·1%)	19	71·4 (50·3–97)	3·6% (-2·3 to 9·8%)	13
	Egypt	50341 (35383–68471)	107·9% (95 to 122·9%)	6	58·6 (41·4–79·3)	4% (-2·1 to 10·7%)	14
	Iran (Islamic Republic of)	70192 (52341–93184)	52·2% (39·8 to 63·9%)	2	80·1 (59·6–106·4)	-28·1% (-34·8 to -23·2%)	2
	Iraq	37310 (26823–50871)	136·4% (111·1 to 161·5%)	10	105·8 (76·4–143·3)	-19·9% (-30·1 to -10·6%)	3
	Jordan	5884 (4095–7991)	285% (261·2 to 310·9%)	17	59·4 (41·4–80·3)	-3·9% (-8·6 to 1%)	10
	Kuwait	3711 (2638–5012)	182% (157·4 to 204·3%)	12	80·6 (57·8–108·6)	-12% (-18 to -6·7%)	5
	Lebanon	4026 (2849–5569)	32·7% (7 to 58·8%)	1	75·7 (53·6–105·1)	-30·4% (-42·8 to -18%)	1
	Libya	6320 (4603–8529)	168·7% (151·7 to 189·7%)	11	91·1 (66·4–122·5)	9·9% (3·2 to 18·6%)	16
	Morocco	26514 (18727–35851)	89% (78·7 to 99·1%)	3	73·9 (52–100·1)	2·5% (-2·9 to 7·6%)	11
	Palestine	3728 (2642–5148)	188·7% (163·9 to 212·4%)	13	97 (69·2–133·3)	10·2% (2·1 to 17·8%)	17
	Oman	4246 (2938–5805)	196·5% (179·6 to 214·8%)	14	100·2 (70·5–136·3)	-5·4% (-9·6 to -0·8%)	8
	Qatar	2857 (1994–3911)	557·5% (514·3 to 600·8%)	20	97·2 (68·5–132·2)	-10·5% (-14·8 to -6·2%)	7
	Saudi Arabia	55075 (38616–75312)	310·9% (289·1 to 332·1%)	18	156·1 (109·5–212·2)	25% (20·1 to 29·7%)	20
	Syrian Arab Republic	15764 (10567–23012)	227·2% (146 to 379·1%)	16	116·4 (75·7–170)	107·7% (51·8 to 211·9%)	21
	Tunisia	9380 (6559–12772)	101·1% (90·9 to 112·3%)	4	73 (51·3–99·5)	3·5% (-1·5 to 8·8%)	12
	Türkiye	62842 (45196–84968)	122·8% (109·8 to 137·1%)	9	69·1 (49·6–93·2)	18·9% (12·6 to 26·1%)	19
	United Arab Emirates	9976 (6962–13671)	603·8% (555·7 to 656·9%)	21	87·3 (60·9–119·3)	-4·9% (-9·5 to 0%)	9
	Yemen	17087 (12086–23384)	215·7% (182·1 to 263·2%)	15	75·2 (54·3–101·2)	16·7% (4·8 to 33·9%)	18
	Afghanistan	25411 (16490–40529)	114·3% (34·3 to 250·9%)	7	105·6 (66·4–176·9)	-18·2% (-43·5 to 22·8%)	4
	Sudan	10734 (7768–14444)	119·5% (99·9 to 139·2%)	8	37 (27–49·6)	4·9% (-1·9 to 12·7%)	15
Spinal Injuries	Global	6200809 (4465317–8156193)	65·4% (56·3 to 72%)		76·2 (54·8–100·4)	-1·5% (-5·6 to 3·3%)	
	North Africa and Middle East	564189 (289631–1229092)	85·5% (36·6 to 124·6%)		92·2 (48–198·8)	-5·4% (-27·6 to 12·3%)	
	Algeria	21542 (14186–32871)	146·5% (91·6 to 263·6%)	9	50·5 (33·5–75·6)	12·2% (-11·6 to 62·6%)	16
	Bahrain	716 (501–939)	274·7% (224·5 to 332·1%)	16	39·3 (27·9–51·1)	-2·1% (-13·7 to 11·7%)	11
	Egypt	28971 (20531–37521)	100·5% (75·2 to 133·9%)	7	31·2 (22·2–40·3)	0·7% (-11·7 to 16·9%)	12
	Iran (Islamic Republic of)	58925 (36923–97182)	0·7% (-26·5 to 40%)	2	63·3 (39·9–102)	-48·3% (-62·5 to -29·6%)	3
	Iraq	106419 (35020–295453)	78·2% (65·8 to 104·2%)	6	264·6 (89·6–729·2)	-28·6% (-37·1 to -18·4%)	4
	Jordan	3679 (2567–4814)	277·9% (228 to 346·7%)	17	34·4 (24·2–44·8)	-3·2% (-14·6 to 11·5%)	10
	Kuwait	2655 (1727–4220)	227·6% (179·1 to 283·8%)	13	52 (34·5–79·6)	4·4% (-10·2 to 21%)	13
	Lebanon	7227 (2416–20570)	-28·3% (-37·9 to 4·3%)	1	135 (45·1–386·4)	-58·1% (-63·8 to -41·5%)	1
	Libya	7366 (3820–15730)	255·6% (178·8 to 331·9%)	15	100·1 (52–214·7)	67·9% (21·6 to 123·9%)	19
	Morocco	15820 (11427–20411)	56·4% (29·9 to 80·1%)	4	42·7 (30·9–55)	-9·1% (-21·4 to 2·7%)	7
	Palestine	13321 (4255–37063)	148·8% (118·7 to 168·3%)	10	309 (100·2–843·4)	9·3% (-5·8 to 17·9%)	15
	Oman	2116 (1481–2770)	186·5% (148·6 to 232·1%)	12	44·4 (31·2–57·6)	-7·8% (-17·9 to 4·1%)	8
	Qatar	1525 (1072–2024)	498% (415·6 to 588·7%)	19	46·7 (32·9–61·2)	-17·5% (-26·8 to -8·3%)	5
	Saudi Arabia	28027 (19946–36763)	249·9% (213·8 to 290·2%)	14	70·6 (50·1–92·2)	6·2% (-3·8 to 16·6%)	14
	Syrian Arab Republic	56665 (16102–159728)	794·6% (325·3 to 1703·5%)	21	403·2 (112·6–1150·3)	579·2% (218·2 to 1263·6%)	21
	Tunisia	4717 (3406–6139)	72·8% (51·4 to 94·8%)	5	36·7 (26·5–47·6)	-5·7% (-16 to 5·6%)	9
	Türkiye	40162 (27330–56767)	120·1% (83·7 to 175·5%)	8	43·7 (29·7–61·9)	24·2% (3·7 to 55·5%)	18
	United Arab Emirates	5464 (3826–7080)	520·7% (428·5 to 622·5%)	20	42·9 (30·9–54·8)	-11·1% (-21·3 to -1·1%)	6
	Yemen	27471 (12186–66369)	323·5% (208·3 to 439·2%)	18	100·9 (45·9–237·9)	70·7% (18·8 to 131·1%)	20
	Afghanistan	98889 (26400–287537)	28·8% (-24·1 to 309·3%)	3	342 (87·3–1016·4)	-50·7% (-67·5 to 33%)	2
	Sudan	31938 (20436–55444)	156·3% (122·8 to 192·8%)	11	96·5 (63·1–164·2)	22·2% (5·5 to 42·6%)	17

Table S13 Disability, prevalence, and incidence of head and spinal injuries in North Africa and Middle East countries

Location		All Ages			Age-standardised		
		Number	Percent change		Rate per 100,000	Percent change	
		2019	From 1990 to 2019		2019	From 1990 to 2019	
Cause	Measure	Mean (95% UI)	Mean (95% UI)	Rank	Mean (95% UI)	Mean (95% UI)	Rank
**Prevalence**							
Minor TBI	Global	11482589 (10758798–12351815)	71·3% (67·8 to 74·5%)		141·9 (133–152·7)	-4·4% (-5·7 to -3·2%)	
	North Africa and Middle East	720413 (591293–955610)	119·7% (98·8 to 132·8%)		129 (106·2–170·2)	0·2% (-9·6 to 6·3%)	
	Algeria	39090 (35470–43719)	109% (95·9 to 129·5%)	6	98·1 (89·4–108·9)	-6·4% (-11·4 to 1·7%)	8
	Bahrain	1489 (1369–1612)	314·7% (294·7 to 336·8%)	18	92·6 (85·6–100)	3·2% (-1·1 to 7·8%)	13
	Egypt	65076 (60170–70707)	111·8% (102·8 to 121·1%)	7	78·1 (72·6–84)	7% (3·1 to 11%)	15
	Iran (Islamic Republic of)	92711 (81930–110077)	32·7% (11·3 to 53·3%)	2	108·5 (96·2–128·2)	-38·8% (-52·4 to -27·4%)	2
	Iraq	83833 (53435–142108)	112·7% (87·2 to 139·6%)	8	241·5 (154·3–413·9)	-28·4% (-41·1 to -15·2%)	3
	Jordan	7774 (7129–8520)	281·6% (265·9 to 299·7%)	15	78·7 (72·7–85·4)	-2·9% (-6·2 to 1%)	10
	Kuwait	4771 (4305–5380)	129·6% (66·3 to 182·4%)	11	106·5 (96·7–119·3)	-21·6% (-38·9 to -9·6%)	4
	Lebanon	7301 (5178–12217)	11·6% (-8·8 to 42·1%)	1	138·3 (97·8–231·3)	-40·5% (-51·5 to -25%)	1
	Libya	9647 (7867–12989)	202·8% (167 to 257%)	14	141·4 (116·4–187·5)	30·8% (14·8 to 56·5%)	19
	Morocco	34642 (32135–37604)	80·3% (73·5 to 87·9%)	3	98·8 (91·7–107·1)	0·3% (-2·9 to 3·8%)	11
	Palestine	8308 (5057–14690)	201·7% (174·9 to 237·3%)	13	214·1 (131·3–372·1)	20·5% (9 to 34·4%)	17
	Oman	4866 (4483–5315)	186·1% (174·5 to 198·9%)	12	123·1 (114·3–132·7)	-6·7% (-9·6 to -3·3%)	7
	Qatar	3511 (3190–3893)	570·1% (540·5 to 601·1%)	20	126·8 (117·1–137·9)	-9·4% (-12·8 to -5·8%)	6
	Saudi Arabia	72399 (64683–81721)	289·9% (266·5 to 315·1%)	16	215·3 (195·4–239·6)	25% (18·7 to 31·4%)	18
	Syrian Arab Republic	37575 (19729–71859)	389% (193 to 722·1%)	19	286·9 (147·5–571·1)	234·4% (91·4 to 478·2%)	21
	Tunisia	11811 (10995–12674)	94·2% (86·2 to 102·1%)	4	94·1 (87·5–101·3)	2·7% (-0·5 to 5·7%)	12
	Türkiye	83419 (76697–91188)	115% (103·9 to 127·6%)	9	93·3 (85·8–102)	16·9% (11·6 to 23·5%)	16
	United Arab Emirates	11458 (10596–12412)	573·8% (546·6 to 605·7%)	21	113·4 (105·8–122·1)	-5·4% (-8·2 to -2·5%)	9
	Yemen	34648 (23883–56263)	309% (215·5 to 466%)	17	144·6 (103·2–223·6)	48·1% (14·5 to 102·6%)	20
	Afghanistan	70165 (34848–142450)	116·1% (35·2 to 291·7%)	10	301 (139·5–639·5)	-13·7% (-39·2 to 43·5%)	5
	Sudan	35188 (30134–43909)	108·8% (83·3 to 128%)	5	121 (105·1–149·1)	3·5% (-4 to 11·2%)	14
Moderate/Severe TBI	Global	37505339 (35614732–39599637)	82·8% (80 to 85·7%)		457·4 (434·3–482·6)	1·4% (0·1 to 2·8%)	
	North Africa and Middle East	2376468 (2188401–2700644)	130% (116·8 to 138·2%)		423·2 (390·4–479·2)	3·1% (-2 to 6·1%)	
	Algeria	163516 (154536–173917)	104·2% (97·1 to 112%)	4	404·8 (383·8–429·5)	-10·5% (-13·1 to -7·5%)	5
	Bahrain	6842 (6435–7323)	331% (314·2 to 348%)	19	394·3 (372·7–419·7)	4·5% (1·3 to 7·9%)	14
	Egypt	272495 (258365–287373)	108·1% (101·6 to 115·2%)	6	318·5 (302·7–334·8)	3·6% (0·5 to 6·8%)	12
	Iran (Islamic Republic of)	386217 (357662–420709)	64·8% (50·3 to 75·3%)	2	441·4 (410·4–478·8)	-23·9% (-31·9 to -18·9%)	2
	Iraq	177373 (144014–255710)	144·3% (114 to 171·3%)	10	508·7 (417·6–728·7)	-18·1% (-30 to -8·2%)	4
	Jordan	31647 (29851–33490)	290·5% (279 to 302·4%)	17	324·1 (306·9–341·8)	-3·8% (-6·2 to -1·3%)	10
	Kuwait	20178 (18941–21626)	199·7% (189·1 to 210·7%)	15	443·3 (419·6–473·3)	-8·8% (-11·4 to -6·3%)	7
	Lebanon	20744 (18109–27107)	41·4% (10·6 to 67·1%)	1	389·8 (339·9–510·3)	-26·8% (-41·5 to -14·8%)	1
	Libya	33484 (30875–37616)	166·2% (154·2 to 184·5%)	11	485·9 (450·3–543)	7% (2·4 to 15·1%)	16

Table S13 Disability, prevalence, and incidence of head and spinal injuries in North Africa and Middle East countries

		All Ages			Age-standardised		
		Number	Percent change		Rate per 100,000	Percent change	
**Location**		2019	From 1990 to 2019		2019	From 1990 to 2019	
Cause	Measure	Mean (95% UI)	Mean (95% UI)	Rank	Mean (95% UI)	Mean (95% UI)	Rank
	Morocco	144833 (136919–153465)	93·4% (86·3 to 99·3%)	3	404·5 (383·4–427·9)	3·5% (0·3 to 6·2%)	11
	Palestine	17858 (13854–27341)	187·1% (161·2 to 203·5%)	12	474·3 (371·7–721·2)	8·1% (0 to 12·7%)	17
	Oman	23269 (21831–24840)	199·2% (190·9 to 207·4%)	14	555·9 (526·6–589)	-4·6% (-6·9 to -2·4%)	8
	Qatar	15463 (14427–16703)	558·5% (539·7 to 582%)	20	537·2 (509·1–572·8)	-9·8% (-12·2 to -7%)	6
	Saudi Arabia	297376 (274874–323853)	318% (301·9 to 333·9%)	18	855·4 (798–919·3)	25·6% (22·1 to 29%)	20
	Syrian Arab Republic	74284 (52381–129427)	200·3% (121·1 to 369·7%)	16	546·8 (376·2–974·9)	84·6% (31·4 to 194·7%)	21
	Tunisia	51831 (49256–54732)	106% (100·6 to 111·8%)	5	401·9 (382·3–424·2)	4·3% (2 to 7·1%)	13
	Türkiye	344715 (325611–366874)	128·6% (120·2 to 138·6%)	9	377·8 (357·6–401·5)	20·3% (16 to 25·1%)	19
	United Arab Emirates	54835 (51305–58856)	616·8% (587·1 to 640·8%)	21	480·1 (455·6–509·7)	-4·1% (-6·8 to -2%)	9
	Yemen	83073 (73742–102117)	195·9% (175·2 to 236·2%)	13	379·9 (337·2–466·3)	10·6% (2·2 to 29·3%)	18
	Afghanistan	112371 (77419–202832)	109·3% (33·3 to 229%)	7	473·6 (316·7–887·3)	-20·4% (-45·2 to 15·6%)	3
	Sudan	41654 (38114–47604)	127·1% (119·2 to 136·4%)	8	147·5 (135·4–166·9)	6·5% (2·7 to 11·7%)	15
Spinal cord lesion at neck level	Global	10857565 (9490785–13911786)	82·9% (69·7 to 91·1%)		133·3 (116·3–171·1)	8·7% (2·8 to 14·7%)	
	North Africa and Middle East	1179736 (578725–2753325)	98·8% (42·8 to 150·3%)		192·6 (95·9–445·7)	2·3% (-24·7 to 24·9%)	
	Algeria	40733 (29768–67436)	221·5% (139·2 to 415·9%)		95 (70·4–154·2)	45·6% (10·5 to 129·3%)	17
	Bahrain	1314 (1196–1491)	337·8% (300·2 to 368·3%)	11	72·1 (66–81·2)	14·4% (3·6 to 22·4%)	10
	Egypt	47851 (43430–55034)	138% (119·3 to 172·1%)	16	51·4 (47–58·3)	19·3% (10·8 to 34·9%)	12
	Iran (Islamic Republic of)	118774 (77897–213075)	1·3% (-24·5 to 52·5%)	7	127 (85·4–222·2)	-48·8% (-63·2 to -23·7%)	3
	Iraq	250995 (76003–710887)	88·9% (77·5 to 110·4%)	2	626·6 (193·4–1771·8)	-25·1% (-34·9 to -14%)	4
	Jordan	6598 (5933–7818)	359·7% (320·8 to 442·6%)	5	61·8 (56·2–71·9)	17·5% (8·7 to 35·2%)	11
	Kuwait	5517 (3998–9287)	280·2% (233·8 to 345·5%)	17	107·9 (80·3–176·4)	21·5% (6·5 to 45·7%)	14
	Lebanon	18126 (5491–58095)	-21·4% (-30·5 to 12·7%)	13	338·9 (102·6–1087)	-54·1% (-60 to -36·4%)	1
	Libya	15744 (7745–36439)	317·9% (229·1 to 390·4%)	1	213·7 (105·7–498·9)	103·4% (44·1 to 161·2%)	19
	Morocco	25296 (23066–28856)	69% (34·9 to 92·6%)	14	68·3 (62·4–77·6)	-0·4% (-16·5 to 10·4%)	6
	Palestine	31857 (9434–90472)	169·9% (145·5 to 183·3%)	4	742·4 (223·5–2100·8)	19·5% (6·1 to 26·6%)	13
	Oman	3892 (3564–4215)	244·2% (226·7 to 260·7%)	8	82 (76·4–88·2)	11·7% (7 to 16·9%)	9
	Qatar	2843 (2610–3081)	617·8% (580·9 to 658·7%)	12	87·9 (81·6–95)	0·4% (-4·3 to 5·2%)	7
	Saudi Arabia	50055 (45545–55087)	326·2% (303·3 to 352·5%)	20	126·1 (115·6–137·9)	29·1% (23·1 to 35·6%)	15
	Syrian Arab Republic	138355 (38037–399489)	1049·3% (458·8 to 2231·5%)	15	989·8 (275·3–2883·5)	801·4% (319·5 to 1724%)	21
	Tunisia	8545 (7943–9249)	103·7% (92·5 to 115·1%)	21	66·5 (61·8–72)	10·7% (4·8 to 17·2%)	8
	Türkiye	77214 (61893–109056)	191·5% (147·8 to 272·8%)	6	83·9 (67·3–118·5)	64·9% (38·9 to 114·4%)	18
	United Arab Emirates	9063 (8389–9800)	582·7% (532 to 620·7%)	9	70·6 (66–75·8)	-1·9% (-7·3 to 2·6%)	5
	Yemen	56156 (22113–148124)	404·1% (276·1 to 506·9%)	19	207·3 (84·5–545·3)	114% (45·9 to 178·7%)	20
	Afghanistan	214777 (51501–641727)	28·9% (-23·3 to 330·8%)	18	755·2 (173–2296·9)	-50·1% (-66·8 to 44·9%)	2
	Sudan	54832 (36638–101867)	198·7% (166·9 to 234·5%)	3	164·5 (112·8–296·7)	45·4% (26·3 to 70·1%)	16
Spinal cord lesion below neck level	Global	9777472 (9208477–10448724)	80·1% (76·1 to 84·5%)	10	119·7 (112·8–128)	2·8% (0·9 to 5·1%)	

Table S13 Disability, prevalence, and incidence of head and spinal injuries in North Africa and Middle East countries

Location		All Ages			Age-standardised		
		Number	Percent change		Rate per 100,000	Percent change	
		2019	From 1990 to 2019		2019	From 1990 to 2019	
Cause	Measure	Mean (95% UI)	Mean (95% UI)	Rank	Mean (95% UI)	Mean (95% UI)	Rank
	North Africa and Middle East	418004 (387020–462511)	148·2% (141 to 157·3%)		71·7 (66·4–79·4)	13% (9·8 to 17·6%)	
	Algeria	27040 (24961–29482)	110·4% (99·9 to 120·6%)	6	65·3 (60·4–71·1)	-4·3% (-8·6 to 0%)	3
	Bahrain	1149 (1070–1230)	361·3% (336·4 to 387·6%)	19	65·2 (61–69·6)	18·3% (12·3 to 24·5%)	17
	Egypt	43873 (40983–46736)	116·5% (106·6 to 127·8%)	7	48·7 (45·6–51·7)	8·5% (3·9 to 13·8%)	13
	Iran (Islamic Republic of)	73619 (61638–95401)	103·6% (93 to 116·7%)	5	82·1 (69·1–105·7)	-2·8% (-8·2 to 4·5%)	4
	Iraq	24654 (23214–26281)	192·8% (182·8 to 204·7%)	13	67·9 (64·2–72·2)	-0·6% (-3·8 to 3·1%)	7
	Jordan	5759 (5393–6150)	310% (294 to 330·6%)	17	55·8 (52·5–59·4)	4·4% (0·6 to 9%)	10
	Kuwait	3408 (3177–3653)	225·2% (212·3 to 239·7%)	14	70·8 (66·4–75·4)	1·3% (-2·5 to 5·3%)	9
	Lebanon	3281 (3081–3507)	79·9% (69·7 to 89·7%)	1	61·4 (57·6–65·5)	-6·9% (-12·3 to -1·8%)	2
	Libya	5232 (4863–5774)	181·7% (164·7 to 207·5%)	12	73·3 (68·3–80·3)	16·8% (10·2 to 26·3%)	16
	Morocco	23840 (22369–25585)	96·9% (88·7 to 107%)	2	65·1 (61–69·8)	9·2% (5·1 to 14·4%)	14
	Palestine	2760 (2385–3550)	128·4% (60·1 to 198·9%)	8	71·4 (60·7–96·1)	-10·4% (-30·8 to 8%)	1
	Oman	3539 (3267–3840)	232% (215·9 to 249·7%)	15	78·2 (72·9–83·7)	6·9% (2·3 to 12·1%)	12
	Qatar	2595 (2389–2806)	618·3% (581 to 653·8%)	21	83·6 (77·5–89·7)	-1·5% (-6·2 to 3%)	5
	Saudi Arabia	49177 (45198–53903)	312·1% (288·7 to 337%)	18	130·3 (121–141·7)	25·5% (19·3 to 31·9%)	19
	Syrian Arab Republic	8426 (7804–9100)	98·9% (88·9 to 110·6%)	3	59·1 (54·6–63·9)	25% (18·5 to 33·2%)	18
	Tunisia	8153 (7647–8827)	99·6% (88·9 to 109·6%)	4	63·4 (59·5–68·5)	6·5% (1·3 to 11·7%)	11
	Türkiye	61977 (56066–72328)	139% (120·7 to 170·1%)	9	67·5 (61·1–78·6)	31·1% (21·3 to 46·9%)	21
	United Arab Emirates	8213 (7624–8844)	606·3% (573·7 to 636·9%)	20	68·1 (63·8–72·6)	-0·6% (-4·2 to 3·1%)	6
	Yemen	12455 (11570–13316)	166% (145·8 to 180·6%)	11	52·7 (49·1–56·5)	0·4% (-6·1 to 5%)	8
	Afghanistan	14498 (12307–18547)	284·9% (239 to 370·4%)	16	54·7 (46·1–71·2)	26·8% (10·7 to 57·7%)	20
	Sudan	33931 (31280–36875)	147·5% (134 to 163·4%)	10	109·4 (101·4–118·1)	14·9% (9·2 to 21·4%)	15
Head Injuries	Global	48987927 (46840150–51316804)	79·9% (77·6 to 82·4%)		599·3 (573–627·3)	0% (-1·1 to 1·2%)	
	North Africa and Middle East	3096882 (2843657–3481267)	127·5% (113·2 to 135·7%)		552·2 (507·4–619·2)	2·4% (-3·3 to 5·7%)	
	Algeria	202606 (192787–214115)	105·1% (98·8 to 112·3%)	5	502·8 (479·6–530·1)	-9·8% (-12 to -7%)	6
	Bahrain	8331 (7910–8808)	328% (314·1 to 342·3%)	19	486·9 (464·6–512·4)	4·2% (1·5 to 7·2%)	13
	Egypt	337571 (322203–354072)	108·8% (103·3 to 115·1%)	6	396·6 (378·8–415·3)	4·3% (1·7 to 7%)	14
	Iran (Islamic Republic of)	478928 (445665–520699)	57·4% (45·6 to 67·5%)	2	549·8 (513·4–594·8)	-27·4% (-34·4 to -22·2%)	2
	Iraq	261206 (207437–347346)	133·2% (110 to 156·2%)	10	750·1 (597·1–996·4)	-21·7% (-31·2 to -12·7%)	3
	Jordan	39421 (37539–41373)	288·7% (279·4 to 299·1%)	17	402·8 (385·5–421·6)	-3·6% (-5·7 to -1·6%)	10
	Kuwait	24948 (23651–26471)	183·2% (157·4 to 200%)	12	549·8 (523·6–578·6)	-11·6% (-17·4 to -7·7%)	5
	Lebanon	28045 (23771–37081)	32·2% (7·4 to 57·3%)	1	528·1 (446·9–699·7)	-30·9% (-42·9 to -19·1%)	1
	Libya	43130 (39583–47930)	173·6% (159·7 to 191·3%)	11	627·3 (578·7–693·6)	11·6% (5·9 to 19·3%)	16
	Morocco	179475 (171427–188308)	90·7% (85 to 95·5%)	3	503·3 (481·4–527·5)	2·9% (0·2 to 5%)	11
	Palestine	26166 (19893–36241)	191·6% (168·6 to 209·5%)	13	688·4 (527·1–948·2)	11·7% (4·9 to 17·8%)	17
	Oman	28135 (26674–29779)	196·9% (189 to 204·2%)	14	679 (649·3–713·3)	-5% (-7·1 to -3·1%)	8
	Qatar	18974 (17875–20194)	560·7% (543·2 to 581%)	20	663·9 (631·7–701·2)	-9·7% (-11·8 to -7·3%)	7
	Saudi Arabia	369775 (344765–397295)	312·2% (298·2 to 325·3%)	18	1070·7 (1008·3–1140)	25·5% (22·3 to 28·3%)	20

Table S13 Disability, prevalence, and incidence of head and spinal injuries in North Africa and Middle East countries

Location		All Ages			Age-standardised		
		Number	Percent change		Rate per 100,000	Percent change	
		2019	From 1990 to 2019		2019	From 1990 to 2019	
Cause	Measure	Mean (95% UI)	Mean (95% UI)	Rank	Mean (95% UI)	Mean (95% UI)	Rank
	Syrian Arab Republic	111858 (77475–171069)	245·1% (153·3 to 392·3%)	16	833·7 (566·3–1291·1)	118·2% (55·8 to 220·8%)	21
	Tunisia	63641 (60813–66654)	103·7% (98·7 to 108·9%)	4	496 (474·4–519)	4% (1·9 to 6·4%)	12
	Türkiye	428134 (407264–451661)	125·8% (118·6 to 134·6%)	9	471·1 (448·7–496·4)	19·6% (16·1 to 24·1%)	19
	United Arab Emirates	66292 (62592–70302)	609% (583·8 to 628·9%)	21	593·5 (566·6–623·9)	-4·4% (-6·7 to -2·6%)	9
	Yemen	117721 (101299–141736)	222·1% (190·6 to 269·5%)	15	524·6 (456·2–626·3)	18·9% (7·1 to 35·6%)	18
	Afghanistan	182536 (119628–305576)	111·9% (34·5 to 251%)	7	774·5 (486·3–1367)	-17·9% (-42·8 to 24·8%)	4
	Sudan	76842 (69587–87305)	118·3% (102·2 to 130·2%)	8	268·5 (244·4–303·9)	5·2% (0·6 to 10·1%)	15
Spinal Injuries	Global	20635037 (18925876–23611174)	81·5% (74·2 to 87·1%)		253·1 (231·5–290·4)	5·8% (2·7 to 9·6%)	
	North Africa and Middle East	1597740 (989333–3176105)	109·7% (55·4 to 149·4%)		264·3 (165·5–520·5)	5% (-19·4 to 21·4%)	
	Algeria	67773 (56104–95193)	165·6% (124·4 to 263·4%)	9	160·3 (134–220·9)	20·1% (2·5 to 61·7%)	15
	Bahrain	2464 (2288–2677)	348·4% (320·5 to 373·3%)	18	137·3 (127·8–148·6)	16·2% (8·9 to 22·2%)	14
	Egypt	91724 (85282–100309)	127·2% (115·4 to 145%)	7	100·1 (93·4–108·4)	13·8% (8·4 to 21·8%)	12
	Iran (Islamic Republic of)	192392 (147592–289632)	25·4% (-10·6 to 72·8%)	2	209·1 (162·9–308·2)	-37·1% (-55·4 to -15%)	3
	Iraq	275649 (100535–735044)	95·1% (81·3 to 125·1%)	5	694·5 (261·2–1838·6)	-23·3% (-32·7 to -11·6%)	4
	Jordan	12356 (11428–13714)	335·1% (312·7 to 373·3%)	17	117·6 (109·5–128·8)	10·9% (5·8 to 19·5%)	10
	Kuwait	8925 (7309–12662)	257·1% (228·7 to 305%)	13	178·7 (149·6–246·3)	12·6% (3·7 to 28·4%)	11
	Lebanon	21407 (8783–61368)	-14% (-27 to 31·1%)	1	400·3 (164·1–1148·2)	-50·2% (-57·6 to -27·9%)	1
	Libya	20977 (12852–41589)	272·9% (206·2 to 346·6%)	14	287 (176·8–572·8)	71% (31·2 to 126·2%)	19
	Morocco	49136 (45658–53596)	81·5% (56·8 to 96·1%)	4	133·4 (124·2–145·2)	4·1% (-6·6 to 10·5%)	7
	Palestine	34617 (12023–92970)	166% (136·8 to 180·4%)	10	813·9 (289·2–2164·2)	16·1% (1·3 to 24%)	13
	Oman	7431 (6863–8020)	238·3% (223·6 to 252·6%)	12	160·2 (150·2–171·2)	9·3% (5·5 to 13·7%)	9
	Qatar	5438 (5015–5874)	618% (588·7 to 648%)	20	171·5 (159·9–184·2)	-0·5% (-4·4 to 3·4%)	6
	Saudi Arabia	99232 (90735–108803)	319·1% (298·7 to 340·4%)	15	256·4 (236·7–279·5)	27·2% (21·8 to 32·6%)	16
	Syrian Arab Republic	146780 (46392–407902)	801·9% (347 to 1631·3%)	21	1048·9 (332·6–2942)	567·8% (215·4 to 1184·7%)	21
	Tunisia	16698 (15691–17935)	101·7% (91·9 to 110·6%)	6	129·9 (121·8–139·6)	8·6% (3·7 to 13·4%)	8
	Türkiye	139191 (121472–172113)	165·6% (139·5 to 213·1%)	8	151·4 (132·4–187·8)	47·9% (32·9 to 75·7%)	18
	United Arab Emirates	17277 (16071–18581)	593·7% (558·7 to 620·8%)	19	138·6 (130·6–148·2)	-1·2% (-5·1 to 1·8%)	5
	Yemen	68611 (34516–160572)	333·7% (228·3 to 445·6%)	16	260 (136·8–594·8)	74·1% (25 to 134·1%)	20
	Afghanistan	229275 (64978–655574)	34·5% (-20·8 to 328·2%)	3	810 (224·5–2351)	-48% (-65·9 to 41·6%)	2
	Sudan	88763 (69261–135844)	176·8% (154·2 to 208·3%)	11	274 (218·1–405·4)	31·5% (19·6 to 50·5%)	17
**Incidence**							
Minor TBI	Global	12668525 (9926564–16020674)	29·1% (19·7 to 36·6%)		163·1 (127·9–206·5)	-9·7% (-15·7 to -5·6%)	
	North Africa and Middle East	1008833 (766229–1364308)	67·1% (46 to 87·7%)		161·5 (122·4–220·8)	-1·1% (-14·1 to 13·2%)	
	Algeria	51280 (40057–64923)	37·6% (28·2 to 47·2%)	9	120·8 (94·6–153·1)	-11·4% (-16·8 to -5·4%)	7

Table S13 Disability, prevalence, and incidence of head and spinal injuries in North Africa and Middle East countries

		All Ages			Age-standardised		
		Number	Percent change		Rate per 100,000	Percent change	
**Location**		2019	From 1990 to 2019		2019	From 1990 to 2019	
Cause	Measure	Mean (95% UI)	Mean (95% UI)	Rank	Mean (95% UI)	Mean (95% UI)	Rank
	Bahrain	1695 (1320–2134)	157·9% (138·2 to 177·1%)	15	123 (96·6–156·2)	5·2% (-1·9 to 13%)	15
	Egypt	106781 (83639–134451)	84·5% (71·7 to 99·2%)	10	103 (80·9–129·6)	7·3% (0·5 to 15·6%)	16
	Iran (Islamic Republic of)	102334 (79869–131127)	-36·8% (-57·3 to -16·9%)	1	120·7 (94·2–154·7)	-54·9% (-70 to -40·3%)	2
	Iraq	79695 (61603–103004)	85·4% (54·6 to 111·5%)	11	172·9 (133·1–222·5)	-24·1% (-36·2 to -14·2%)	6
	Jordan	13358 (10382–17077)	184·5% (166·8 to 203·9%)	16	107 (83·2–136·3)	-3·2% (-8·6 to 2·9%)	11
	Kuwait	6024 (4607–7739)	-31% (-67·8 to 33·6%)	2	132·2 (102·6–168·1)	-69·6% (-85·6 to -42·1%)	1
	Lebanon	6246 (4861–8007)	-28·5% (-59·5 to 8·4%)	3	121·8 (94·8–155·1)	-51·8% (-72·3 to -28·3%)	3
	Libya	14238 (10426–21063)	143·2% (88·7 to 262·8%)	14	203·8 (147·6–306·1)	55·9% (19·3 to 138·8%)	18
	Morocco	47920 (37277–61368)	33·2% (24·6 to 42·7%)	8	131·1 (102–167·6)	0·2% (-5·8 to 6·7%)	12
	Palestine	6952 (5430–8746)	26·3% (-27·5 to 91·7%)	5	127·9 (100·2–160·9)	-44·7% (-67·6 to -18·1%)	5
	Oman	7893 (6112–10038)	130·7% (110·9 to 151·6%)	13	160·4 (125·9–202·6)	-6·3% (-12·8 to 0·2%)	10
	Qatar	5894 (4431–7793)	507·3% (454·1 to 560%)	20	172·2 (131·9–224·1)	-8·8% (-15·3 to -2%)	8
	Saudi Arabia	117133 (86100–157862)	197·8% (166·1 to 230·5%)	17	284·5 (210·9–377·8)	22·1% (11·4 to 32%)	17
	Syrian Arab Republic	30687 (19644–53828)	106·8% (42 to 263%)	12	235 (142·5–434·7)	128·9% (47·1 to 316·5%)	20
	Tunisia	14210 (11061–18067)	26·7% (17·9 to 35·4%)	6	125·3 (97·1–160·4)	1% (-4·9 to 7·5%)	13
	Türkiye	97591 (76100–124436)	31·9% (17·9 to 47·6%)	7	120·6 (94·2–153·3)	4·8% (-5·2 to 16·7%)	14
	United Arab Emirates	14587 (11242–18651)	361·1% (328·6 to 396·4%)	18	145·1 (114·4–184)	-6·6% (-10·9 to -1·9%)	9
	Yemen	114021 (60442–238096)	588·9% (261·5 to 1352%)	21	320·2 (173·7–647·9)	178·2% (50·4 to 483·7%)	21
	Afghanistan	117717 (64739–236555)	447·1% (322 to 601·1%)	19	361·4 (178·7–782·8)	107·2% (40·1 to 196·8%)	19
	Sudan	51552 (39111–67761)	6·8% (-44·6 to 69·3%)	4	119·6 (91·7–155·6)	-46·5% (-71·3 to -16·5%)	4
Moderate/Severe TBI	Global	14492183 (12603272–16571211)	60·4% (54·5 to 65·9%)		182·7 (158·9–209·8)	-1·4% (-4 to 0·7%)	
	North Africa and Middle East	902661 (790629–1024620)	33% (10·3 to 57%)		157·1 (137·9–179·1)	-25% (-37·3 to -12·1%)	
	Algeria	58539 (51622–65734)	51·7% (41·7 to 61·1%)	3	145 (128·3–162·5)	-14·5% (-18·5 to -10·3%)	5
	Bahrain	1911 (1683–2162)	177·1% (156·5 to 197·5%)	15	136 (120–153·1)	-5·5% (-10·2 to -0·5%)	12
	Egypt	115686 (101649–130554)	86·7% (75·9 to 97·2%)	10	126·7 (111·2–143·2)	3·7% (-1·1 to 8·8%)	14
	Iran (Islamic Republic of)	122435 (107746–139494)	-61·6% (-73·6 to -43·4%)	1	146·8 (129·6–166·5)	-74% (-81·9 to -62·1%)	1
	Iraq	60597 (53849–68171)	128·5% (110·5 to 141·9%)	13	148 (131·9–165·3)	-11·7% (-17·4 to -7·3%)	6
	Jordan	12681 (11225–14283)	197·7% (181·1 to 214·4%)	16	115·9 (102·9–129·2)	-8·3% (-12·5 to -3·8%)	10
	Kuwait	6513 (5745–7316)	59% (7·2 to 106·8%)	5	147·9 (130·9–164·5)	-36·9% (-56·1 to -20·4%)	2
	Lebanon	6537 (5806–7346)	28% (2 to 48·4%)	2	126·1 (111·8–141·2)	-24% (-38·7 to -12·5%)	3
	Libya	12538 (10974–14700)	108·8% (90·7 to 134·3%)	11	180·8 (159–210·7)	10·2% (1·7 to 23·9%)	16
	Morocco	54268 (47976–60843)	55% (47 to 63·1%)	4	154·8 (136·6–173·5)	3% (-1·3 to 7·1%)	13
	Palestine	5681 (5040–6372)	113·9% (63·7 to 151·3%)	12	126·6 (112·6–141·1)	-10·4% (-29 to 3·1%)	8
	Oman	9225 (7977–10563)	136·4% (118·7 to 154%)	14	225·6 (197·4–254·5)	-7·8% (-13·2 to -2·1%)	11
	Qatar	5995 (5235–6777)	494·4% (457·7 to 532·6%)	21	201·8 (178·6–226·2)	-10·8% (-15·1 to -5·7%)	7
	Saudi Arabia	125089 (108626–142125)	251% (230·8 to 271·9%)	18	343·4 (299·5–385·7)	28·7% (23·3 to 33·6%)	18

Table S13 Disability, prevalence, and incidence of head and spinal injuries in North Africa and Middle East countries

Location		All Ages	Percent change		Age-standardised	Percent change	
		Number			Rate per 100,000		
		2019	From 1990 to 2019		2019	From 1990 to 2019	
Cause	Measure	Mean (95% UI)	Mean (95% UI)	Rank	Mean (95% UI)	Mean (95% UI)	Rank
	Syrian Arab Republic	22143 (17365–31567)	77·7% (44·4 to 150·7%)	8	178·5 (129·9–286·4)	63·9% (21·8 to 156·4%)	20
	Tunisia	17549 (15521–19755)	59·1% (49·6 to 69·2%)	6	148·4 (130·9–166·6)	5·4% (0·9 to 9·9%)	15
	Türkiye	121665 (106815–137945)	80·3% (66·7 to 93·9%)	9	144·5 (127–163·6)	19·9% (12·1 to 27·7%)	17
	United Arab Emirates	18596 (16004–21565)	450·9% (410·4 to 495·7%)	20	184·6 (162·9–208·5)	-8·4% (-12·1 to -4·7%)	9
	Yemen	50877 (39076–74712)	238·1% (167·8 to 390·1%)	17	175·5 (138·7–249·2)	29·8% (5·1 to 82·7%)	19
	Afghanistan	55027 (40875–86053)	317·1% (246·2 to 444·4%)	19	210·5 (135·1–378·6)	64·6% (16·1 to 154·3%)	21
	Sudan	18192 (15010–21945)	65·7% (20·5 to 95·9%)	7	52·4 (43·3–62·4)	-19·7% (-40 to -6·5%)	4
Spinal cord lesion at neck level	Global	492278 (354297–675136)	43·3% (9·8 to 67·6%)		6·2 (4·5–8·6)	-10·6% (-27·7 to 1%)	
	North Africa and Middle East	37699 (20760–81071)	55·3% (-12·6 to 145·9%)		6·6 (3·6–14·3)	-13·3% (-54 to 45·7%)	
	Algeria	1032 (776–1353)	62·5% (49·5 to 77·2%)	10	2·5 (1·9–3·3)	-7·4% (-15·1 to 0·6%)	7
	Bahrain	34 (26–45)	201·5% (172·1 to 230·5%)	13	2·5 (1·9–3·2)	4·2% (-6·1 to 14%)	13
	Egypt	2335 (1712–3223)	126·4% (96·4 to 189·9%)	11	2·5 (1·8–3·4)	23·4% (8·1 to 56·2%)	16
	Iran (Islamic Republic of)	2229 (1676–2908)	-82% (-91·4 to -59·8%)	2	2·7 (2–3·5)	-89·4% (-95·1 to -75·6%)	2
	Iraq	2059 (1241–3940)	41·9% (13·7 to 91·4%)	6	4·8 (2·9–8·9)	-41·2% (-52·2 to -22·7%)	6
	Jordan	241 (186–307)	220·9% (198·2 to 245·4%)	14	2·2 (1·7–2·8)	-1·1% (-8 to 6·1%)	11
	Kuwait	118 (89–153)	-82·1% (-93·6 to -37·9%)	1	2·7 (2–3·4)	-92·1% (-97·3 to -73·4%)	1
	Lebanon	130 (100–168)	-72·6% (-89·2 to -22·9%)	3	2·5 (1·9–3·2)	-82·3% (-93 to -51·3%)	3
	Libya	637 (306–1483)	542·7% (203·5 to 1469·4%)	17	9·4 (4·5–22)	251·6% (64·1 to 771·8%)	18
	Morocco	976 (728–1258)	61·2% (51 to 72·8%)	9	2·8 (2·1–3·6)	8% (1·4 to 14·9%)	15
	Palestine	148 (108–218)	-53·6% (-75·6 to 17·5%)	4	3·1 (2·3–4·5)	-78·2% (-88·7 to -46·3%)	4
	Oman	151 (111–204)	151·3% (130·4 to 178·4%)	12	3·7 (2·7–5)	-1·7% (-10·1 to 8%)	10
	Qatar	109 (83–141)	543·4% (481·6 to 605·7%)	18	3·6 (2·7–4·7)	-3·9% (-12·8 to 4·7%)	8
	Saudi Arabia	2285 (1700–3042)	260·3% (233·4 to 287·8%)	15	6·2 (4·5–8·3)	34·4% (25·8 to 43·2%)	17
	Syrian Arab Republic	2444 (766–6813)	829·2% (215·8 to 2114·1%)	19	23·3 (6·6–66·7)	960·5% (221·7 to 2587·9%)	21
	Tunisia	310 (231–413)	60·3% (45·7 to 75·1%)	8	2·6 (2–3·5)	7·6% (-1·7 to 16·9%)	14
	Türkiye	2391 (1791–3134)	45·4% (3·5 to 87·6%)	7	2·9 (2·1–3·8)	1·3% (-25 to 26·1%)	12
	United Arab Emirates	306 (225–415)	456·4% (419·8 to 497%)	16	3·1 (2·3–4·1)	-3·7% (-9·6 to 2·1%)	9
	Yemen	8190 (2414–23485)	3053·8% (811·8 to 8943%)	21	24 (7·4–68·4)	959·8% (223·3 to 3055·5%)	20
	Afghanistan	9804 (2660–28962)	831·4% (559·5 to 1047·8%)	20	41·7 (9·8–129·8)	369% (166 to 559·6%)	19
	Sudan	1728 (1252–2364)	-26·1% (-69·7 to 60·7%)	5	4·8 (3·4–6·6)	-60·7% (-83·9 to -18·7%)	5
Spinal cord lesion below neck level	Global	416722 (289713–584687)	65·5% (48·9 to 78·9%)		5·3 (3·7–7·4)	-0·1% (-8·4 to 5·5%)	
	North Africa and Middle East	15077 (11480–19576)	-18·2% (-57·7 to 42·1%)		2·6 (2–3·4)	-57·2% (-78·4 to -24·2%)	
	Algeria	903 (671–1192)	62·4% (48 to 77·6%)	6	2·2 (1·7–2·9)	-5·9% (-14 to 1·6%)	2
	Bahrain	30 (23–39)	205·3% (175·9 to 235%)	16	2·2 (1·6–2·8)	7·3% (-2·8 to 16·1%)	12
	Egypt	1725 (1285–2343)	93·4% (80·5 to 107·4%)	8	1·8 (1·4–2·5)	7·8% (1·2 to 15·1%)	13
	Iran (Islamic Republic of)	1935 (1454–2512)	-84·4% (-93·2 to -59·6%)	1	2·3 (1·7–3)	-91% (-96·2 to -76·1%)	1
	Iraq	1016 (782–1311)	147·2% (134·3 to 161·8%)	12	2·4 (1·9–3·1)	-4·5% (-9·2 to 0·5%)	4
	Jordan	214 (164–273)	216·7% (193·8 to 239·8%)	17	1·9 (1·5–2·4)	-1·1% (-8·1 to 5·3%)	8
	Kuwait	104 (79–134)	151·7% (132 to 171·8%)	13	2·3 (1·8–3)	-2·7% (-9·8 to 3·7%)	6
	Lebanon	113 (88–144)	59·3% (34·4 to 87·4%)	5	2·2 (1·7–2·8)	-4·9% (-20·5 to 11·3%)	3
	Libya	178 (134–233)	105·8% (85·6 to 136·4%)	9	2·6 (1·9–3·4)	13% (2·7 to 28·1%)	17
	Morocco	860 (652–1120)	59·1% (48·6 to 71·5%)	4	2·4 (1·8–3·2)	8% (1·6 to 14·8%)	14

Table S13 Disability, prevalence, and incidence of head and spinal injuries in North Africa and Middle East countries

		All Ages			Age-standardised		
		Number	Percent change		Rate per 100,000	Percent change	
**Location**		2019	From 1990 to 2019		2019	From 1990 to 2019	
Cause	Measure	Mean (95% UI)	Mean (95% UI)	Rank	Mean (95% UI)	Mean (95% UI)	Rank
	Palestine	105 (83–133)	182·1% (162·2 to 208·6%)	15	2·3 (1·8–2·9)	15·4% (8 to 25·7%)	18
	Oman	128 (94–172)	154·3% (130·3 to 180·2%)	14	3·1 (2·3–4·3)	0·9% (-8·1 to 10·2%)	10
	Qatar	95 (72–123)	552·3% (487·8 to 614·1%)	21	3·1 (2·3–4·1)	-2·3% (-11·2 to 6·5%)	7
	Saudi Arabia	2019 (1500–2668)	248·3% (220·3 to 275·4%)	19	5·5 (4–7·3)	32% (23·1 to 40·7%)	20
	Syrian Arab Republic	317 (243–414)	50·5% (35·9 to 73·6%)	2	2·4 (1·8–3·1)	34% (17·3 to 67·3%)	21
	Tunisia	263 (195–350)	53·3% (38·3 to 70%)	3	2·2 (1·7–3)	4·4% (-4·9 to 13·8%)	11
	Türkiye	2001 (1516–2619)	85% (61·4 to 115·1%)	7	2·4 (1·8–3·1)	25·7% (10·9 to 43·3%)	19
	United Arab Emirates	256 (189–339)	443·4% (407·2 to 485·6%)	20	2·6 (1·9–3·5)	-3·2% (-9·1 to 2·6%)	5
	Yemen	573 (436–752)	143·8% (131·1 to 156·5%)	11	2 (1·5–2·6)	0·1% (-4·8 to 5%)	9
	Afghanistan	648 (506–815)	245·1% (222 to 274·5%)	18	2 (1·6–2·5)	10·1% (1·5 to 24·5%)	16
	Sudan	1577 (1140–2213)	127·2% (112·6 to 144·4%)	10	4·3 (3·1–6·1)	9·1% (2·2 to 16·9%)	15
Head Injuries	Global	27160708 (23357251–31415052)	44·1% (36·9 to 49·7%)		345·9 (297·8–401)	-5·5% (-8·9 to -3%)	
	North Africa and Middle East	1911495 (1624871–2305162)	49% (31·3 to 67·4%)		318·6 (271·9–382·4)	-14·5% (-24·8 to -4%)	
	Algeria	109818 (95425–126366)	44·8% (36·1 to 53·2%)	7	265·8 (231·2–305·6)	-13·1% (-17·2 to -8·7%)	7
	Bahrain	3605 (3131–4160)	167·7% (150·1 to 185%)	15	259 (224·4–299·4)	-0·7% (-5·5 to 4·4%)	12
	Egypt	222467 (191763–257387)	85·6% (74·8 to 96·2%)	10	229·8 (199·1–263·8)	5·3% (0·5 to 10·6%)	15
	Iran (Islamic Republic of)	224769 (194553–259552)	-53·3% (-64·7 to -38·8%)	1	267·5 (232·3–308·7)	-67·8% (-75·7 to -58%)	1
	Iraq	140292 (119415–166064)	101·8% (77·4 to 121·4%)	12	320·9 (275·6–375·8)	-18·8% (-27·5 to -12·2%)	6
	Jordan	26039 (22338–30461)	190·8% (175·3 to 206·3%)	16	223 (193·7–258·6)	-5·9% (-10 to -1·2%)	11
	Kuwait	12537 (10826–14617)	-2·2% (-45·2 to 51·3%)	3	280·1 (242·7–323·8)	-58·2% (-76 to -37·2%)	2
	Lebanon	12783 (11084–14829)	-7·7% (-38·2 to 21·4%)	2	247·9 (215·1–288·1)	-40·8% (-59·4 to -23·5%)	3
	Libya	26776 (22306–34151)	125·8% (95·7 to 182%)	13	384·6 (320·5–493·9)	30·4% (12·6 to 66%)	18
	Morocco	102188 (88863–118831)	44% (37 to 51·2%)	6	285·9 (249·6–331·2)	1·7% (-2·5 to 5·9%)	13
	Palestine	12633 (10861–14734)	54·8% (1·8 to 106·4%)	8	254·5 (221–295·7)	-31·7% (-53·3 to -12·3%)	5
	Oman	17119 (14700–19857)	133·7% (117·4 to 152·2%)	14	386 (336·3–438·3)	-7·2% (-11·9 to -2%)	10
	Qatar	11890 (10081–14150)	500·7% (462·3 to 540·7%)	21	374 (324·5–433·9)	-9·9% (-14·5 to -5·1%)	8
	Saudi Arabia	242222 (207011–286739)	223·1% (200·7 to 246·1%)	17	627·9 (541·6–735)	25·6% (19·3 to 32·2%)	17
	Syrian Arab Republic	52830 (39544–76766)	93·5% (49·1 to 182·8%)	11	413·5 (297·4–625·7)	95·4% (43·6 to 192·7%)	20
	Tunisia	31759 (27629–36591)	42·7% (34·4 to 51·4%)	5	273·7 (237·6–316·6)	3·3% (-1 to 8·1%)	14
	Türkiye	219256 (191556–254991)	55% (43·4 to 66·5%)	9	265·1 (231·6–308)	12·5% (5·2 to 20%)	16
	United Arab Emirates	33183 (28455–38552)	407·5% (374·1 to 445·5%)	19	329·7 (286·2–378·1)	-7·6% (-11·2 to -3·8%)	9
	Yemen	164898 (108336–287669)	421·8% (240·8 to 822·5%)	20	495·8 (338·5–840·9)	98·1% (35·1 to 236·3%)	21
	Afghanistan	172744 (114294–298507)	397·7% (305·3 to 512·9%)	18	571·9 (346·5–1015·1)	89·2% (38·3 to 157·5%)	19
	Sudan	69744 (56092–87454)	17·7% (-31·8 to 69·8%)	4	171·9 (140·9–212·8)	-40·5% (-64·4 to -16·4%)	4
Spinal Injuries	Global	909001 (706953–1156410)	52·7% (30·4 to 69·9%)		11·5 (9–14·7)	-6·1% (-17·3 to 1·5%)	
	North Africa and Middle East	52776 (34780–97680)	23·6% (-24·7 to 91·9%)		9·2 (6–17)	-32·7% (-60·7 to 8·9%)	
	Algeria	1935 (1588–2368)	62·5% (50·5 to 74·2%)	9	4·8 (3·9–5·8)	-6·7% (-13·1 to -0·4%)	7
	Bahrain	65 (53–78)	203·2% (178·6 to 227·1%)	13	4·6 (3·8–5·6)	5·6% (-2·2 to 13·4%)	12

Table S13 Disability, prevalence, and incidence of head and spinal injuries in North Africa and Middle East countries

		All Ages			Age-standardised		
		Number	Percent change		Rate per 100,000	Percent change	
**Location**		2019	From 1990 to 2019		2019	From 1990 to 2019	
Cause	Measure	Mean (95% UI)	Mean (95% UI)	Rank	Mean (95% UI)	Mean (95% UI)	Rank
	Egypt	4060 (3281–5084)	111·1% (92 to 145·7%)	11	4·3 (3·5–5·4)	16·3% (6·6 to 33·8%)	16
	Iran (Islamic Republic of)	4164 (3373–5116)	-83·2% (-90·8 to -68·2%)	1	5 (4·1–6·1)	-90·2% (-94·8 to -81·2%)	1
	Iraq	3076 (2202–4940)	65·2% (31·9 to 112·4%)	10	7·2 (5·2–11·3)	-32·4% (-44·7 to -15·8%)	6
	Jordan	456 (379–543)	218·9% (200·2 to 239·5%)	14	4·1 (3·4–4·9)	-1·1% (-6·7 to 4·5%)	10
	Kuwait	222 (183–267)	-68·3% (-87·9 to -5·2%)	2	5 (4·1–6)	-86·3% (-94·9 to -60·5%)	2
	Lebanon	244 (201–294)	-55·5% (-81·3 to 1·8%)	3	4·7 (3·9–5·7)	-71·5% (-87·7 to -37·5%)	3
	Libya	815 (471–1647)	338·9% (157·3 to 790%)	16	11·9 (6·8–24·4)	141·6% (40·5 to 405·8%)	18
	Morocco	1836 (1507–2226)	60·2% (51·6 to 69·8%)	7	5·2 (4·3–6·3)	8% (2·9 to 13·7%)	14
	Palestine	254 (207–324)	-29% (-65·1 to 58·3%)	4	5·4 (4·4–6·8)	-66·9% (-83·5 to -29·6%)	4
	Oman	279 (226–355)	152·6% (134·3 to 174·8%)	12	6·8 (5·4–8·5)	-0·5% (-7·3 to 6·9%)	11
	Qatar	204 (168–248)	547·5% (494·8 to 597%)	19	6·7 (5·5–8·1)	-3·2% (-10·3 to 3·5%)	9
	Saudi Arabia	4304 (3458–5275)	254·6% (231·5 to 279·4%)	15	11·7 (9·3–14·4)	33·3% (26·1 to 40·4%)	17
	Syrian Arab Republic	2762 (1061–7102)	482·8% (137·9 to 1262·7%)	18	25·7 (8·8–69·2)	548·3% (131·1 to 1510·3%)	21
	Tunisia	574 (467–697)	57% (45·4 to 69·3%)	6	4·9 (4–6)	6·1% (-0·9 to 13%)	13
	Türkiye	4391 (3619–5398)	61·1% (27·9 to 89·6%)	8	5·3 (4·3–6·5)	11·1% (-9 to 28·1%)	15
	United Arab Emirates	562 (454–695)	450·4% (416·7 to 487·7%)	17	5·6 (4·6–7)	-3·5% (-8·2 to 1·3%)	8
	Yemen	8763 (2941–24072)	1671·3% (510 to 4727·5%)	21	26 (9·3–70·3)	510·5% (122·1 to 1565%)	20
	Afghanistan	10452 (3261–29635)	742·6% (464 to 974%)	20	43·7 (11·8–131·7)	308·3% (115·4 to 502·2%)	19
	Sudan	3305 (2618–4167)	8·9% (-48·2 to 87·2%)	5	9·1 (7·2–11·5)	-43·6% (-72·1 to -7·9%)	5

TBI, traumatic brain injury; UI uncertainty interval; YLDs, Years Lived with Disability. Data in parentheses are 95% uncertainty intervals. Count data are absolute numbers and percentage data are rounded to one decimal place, and sex ratio is rounded to two decimal place. Percentages and number of YLDs, prevalent cases, and incident cases are not mutually exclusive: the sum of percentages and number of YLDs in the columns exceeds the totals for all injuries combined because of overlap between various causes. Rank numbers show the ranks of countries with the highest decline or lowest rise (rank 1) to the highest rise (rank 21) in the number or rate of the specific injury during 1990–2019.

Table S14 Regional incidence, prevalence, and disability of head and spinal injuries in the world and in North Africa and Middle East countries
(NEXT PAGE)

Table S14 Incidence, prevalence, and disability of head and spinal injuries in the world and in North Africa and Middle East countries

		All Ages		Age-standardised		
		Number (thousand)	Percent change	Rate per 100,000	Percent change	Sex ratio
**Location**		2019	From 1990 to 2019	2019	From 1990 to 2019	2019
Cause	Measure	Mean (95% UI)	Mean (95% UI)	Mean (95% UI)	Mean (95% UI)	F:M
**Global**						
Minor TBI	Incidence	12668·5 (9926·6–16020·7)	29·1% (19·7 to 36·6%)	163·1 (127·9–206·5)	-9·7% (-15·7 to -5·6%)	0·59
	Prevalence	11482·6 (10758·8–12351·8)	71·3% (67·8 to 74·5%)	141·9 (133–152·7)	-4·4% (-5·7 to -3·2%)	0·62
	YLDs	1366·9 (963·6–1833·6)	70·5% (66·9 to 74%)	16·9 (11·9–22·7)	-4·4% (-5·7 to -3%)	0·61
Moderate/Severe TBI	Incidence	14492·2 (12603·3–16571·2)	60·4% (54·5 to 65·9%)	182·7 (158·9–209·8)	-1·4% (-4 to 0·7%)	0·61
	Prevalence	37505·3 (35614·7–39599·6)	82·8% (80 to 85·7%)	457·4 (434·3–482·6)	1·4% (0·1 to 2·8%)	0·59
	YLDs	5710 (4035·5–7733)	81·1% (78·5 to 84%)	69·7 (49·3–94·3)	1·3% (0 to 2·7%)	0·58
Spinal cord lesion at neck level	Incidence	492·3 (354·3–675·1)	43·3% (9·8 to 67·6%)	6·2 (4·5–8·6)	-10·6% (-27·7 to 1%)	0·77
	Prevalence	10857·6 (9490·8–13911·8)	82·9% (69·7 to 91·1%)	133·3 (116·3–171·1)	8·7% (2·8 to 14·7%)	0·74
	YLDs	4253·1 (3040·2–5853·3)	72·9% (59·4 to 81·2%)	52·3 (37·3–72·1)	4·1% (-2 to 10·1%)	0·72
Spinal cord lesion below neck level	Incidence	416·7 (289·7–584·7)	65·5% (48·9 to 78·9%)	5·3 (3·7–7·4)	-0·1% (-8·4 to 5·5%)	0·87
	Prevalence	9777·5 (9208·5–10448·7)	80·1% (76·1 to 84·5%)	119·7 (112·8–128)	2·8% (0·9 to 5·1%)	0·81
	YLDs	1947·7 (1370·6–2571·5)	51·1% (44·7 to 57·6%)	23·9 (16·8–31·5)	-12% (-15·4 to -8·5%)	0·78
Head Injuries	Incidence	27160·7 (23357·3–31415·1)	44·1% (36·9 to 49·7%)	345·9 (297·8–401)	-5·5% (-8·9 to -3%)	0·60
	Prevalence	48987·9 (46840·1–51316·8)	79·9% (77·6 to 82·4%)	599·3 (573–627·3)	0% (-1·1 to 1·2%)	0·60
	YLDs	7076·9 (4997·9–9588·1)	79% (76·6 to 81·5%)	86·5 (61·1–117·2)	0·1% (-1 to 1·3%)	0·59
Spinal Injuries	Incidence	909 (707–1156·4)	52·7% (30·4 to 69·9%)	11·5 (9–14·7)	-6·1% (-17·3 to 1·5%)	0·82
	Prevalence	20635 (18925·9–23611·2)	81·5% (74·2 to 87·1%)	253·1 (231·5–290·4)	5·8% (2·7 to 9·6%)	0·77
	YLDs	6200·8 (4465·3–8156·2)	65·4% (56·3 to 72%)	76·2 (54·8–100·4)	-1·5% (-5·6 to 3·3%)	0·74
**North Africa and Middle East**						
Minor TBI	Incidence	1008·8 (766·2–1364·3)	67·1% (46 to 87·7%)	161·5 (122·4–220·8)	-1·1% (-14·1 to 13·2%)	0·58
	Prevalence	720·4 (591·3–955·6)	119·7% (98·8 to 132·8%)	129 (106·2–170·2)	0·2% (-9·6 to 6·3%)	0·58
	YLDs	86·6 (60·6–118·7)	120·2% (98·7 to 134·7%)	15·4 (10·9–21)	0·7% (-9·4 to 7·1%)	0·57
Moderate/Severe TBI	Incidence	902·7 (790·6–1024·6)	33% (10·3 to 57%)	157·1 (137·9–179·1)	-25% (-37·3 to -12·1%)	0·58
	Prevalence	2376·5 (2188·4–2700·6)	130% (116·8 to 138·2%)	423·2 (390·4–479·2)	3·1% (-2 to 6·1%)	0·53
	YLDs	366·4 (262·9–489·6)	126% (113·5 to 134·9%)	64·7 (46·6–86·2)	2·1% (-2·6 to 5·7%)	0·52
Spinal cord lesion at neck level	Incidence	37·7 (20·8–81·1)	55·3% (-12·6 to 145·9%)	6·6 (3·6–14·3)	-13·3% (-54 to 45·7%)	0·77
	Prevalence	1179·7 (578·7–2753·3)	98·8% (42·8 to 150·3%)	192·6 (95·9–445·7)	2·3% (-24·7 to 24·9%)	0·70
	YLDs	478·8 (220–1142·1)	85% (33·5 to 134·8%)	77·7 (36·2–184)	-3·6% (-28·8 to 18·6%)	0·69
Spinal cord lesion below neck level	Incidence	15·1 (11·5–19·6)	-18·2% (-57·7 to 42·1%)	2·6 (2–3·4)	-57·2% (-78·4 to -24·2%)	0·76
	Prevalence	418 (387–462·5)	148·2% (141 to 157·3%)	71·7 (66·4–79·4)	13% (9·8 to 17·6%)	0·72
	YLDs	85·4 (59·7–113·8)	88·1% (74·3 to 101·8%)	14·5 (10·2–19·3)	-14·1% (-20·4 to -7·9%)	0·71
Head Injuries	Incidence	1911·5 (1624·9–2305·2)	49% (31·3 to 67·4%)	318·6 (271·9–382·4)	-14·5% (-24·8 to -4%)	0·58
	Prevalence	3096·9 (2843·7–3481·3)	127·5% (113·2 to 135·7%)	552·2 (507·4–619·2)	2·4% (-3·3 to 5·7%)	0·54
	YLDs	453 (331·6–604)	124·9% (111·3 to 133·7%)	80·1 (58·6–106·9)	1·9% (-3·5 to 5·4%)	0·53
Spinal Injuries	Incidence	52·8 (34·8–97·7)	23·6% (-24·7 to 91·9%)	9·2 (6–17)	-32·7% (-60·7 to 8·9%)	0·76
	Prevalence	1597·7 (989·3–3176·1)	109·7% (55·4 to 149·4%)	264·3 (165·5–520·5)	5% (-19·4 to 21·4%)	0·71
	YLDs	564·2 (289·6–1229·1)	85·5% (36·6 to 124·6%)	92·2 (48–198·8)	-5·4% (-27·6 to 12·3%)	0·70
**Algeria**						
Minor TBI	Incidence	51·3 (40·1–64·9)	37·6% (28·2 to 47·2%)	120·8 (94·6–153·1)	-11·4% (-16·8 to -5·4%)	0·59
	Prevalence	39·1 (35·5–43·7)	109% (95·9 to 129·5%)	98·1 (89·4–108·9)	-6·4% (-11·4 to 1·7%)	0·61
	YLDs	4·7 (3·3–6·4)	109·1% (84·5 to 139·4%)	11·8 (8·3–15·9)	-6·5% (-16·2 to 5·3%)	0·60
Moderate/Severe TBI	Incidence	58·5 (51·6–65·7)	51·7% (41·7 to 61·1%)	145 (128·3–162·5)	-14·5% (-18·5 to -10·3%)	0·61
	Prevalence	163·5 (154·5–173·9)	104·2% (97·1 to 112%)	404·8 (383·8–429·5)	-10·5% (-13·1 to -7·5%)	0·58
	YLDs	25·2 (17·9–33·9)	100·8% (89·2 to 114%)	62 (44·3–83·5)	-11·3% (-15·9 to -6·3%)	0·57
Spinal cord lesion at neck level	Incidence	1 (0·8–1·4)	62·5% (49·5 to 77·2%)	2·5 (1·9–3·3)	-7·4% (-15·1 to 0·6%)	0·69
	Prevalence	40·7 (29·8–67·4)	221·5% (139·2 to 415·9%)	95 (70·4–154·2)	45·6% (10·5 to 129·3%)	0·65
	YLDs	16·1 (10·1–27·4)	198·8% (111·7 to 382%)	37·4 (23·7–62·4)	36·3% (-1·6 to 118·3%)	0·64
Spinal cord lesion below neck level	Incidence	0·9 (0·7–1·2)	62·4% (48 to 77·6%)	2·2 (1·7–2·9)	-5·9% (-14 to 1·6%)	0·79
	Prevalence	27 (25–29·5)	110·4% (99·9 to 120·6%)	65·3 (60·4–71·1)	-4·3% (-8·6 to 0%)	0·76

Table S14 Incidence, prevalence, and disability of head and spinal injuries in the world and in North Africa and Middle East countries

		All Ages		Age-standardised		
		Number (thousand)	Percent change	Rate per 100,000	Percent change	Sex ratio
**Location**		2019	From 1990 to 2019	2019	From 1990 to 2019	2019
Cause	Measure	Mean (95% UI)	Mean (95% UI)	Mean (95% UI)	Mean (95% UI)	F:M
	YLDs	5·4 (3·7–7·3)	62·3% (38·7 to 89·2%)	13 (9–17·5)	-25·5% (-35·4 to -14%)	0·75
Head Injuries	Incidence	109·8 (95·4–126·4)	44·8% (36·1 to 53·2%)	265·8 (231·2–305·6)	-13·1% (-17·2 to -8·7%)	0·60
	Prevalence	202·6 (192·8–214·1)	105·1% (98·8 to 112·3%)	502·8 (479·6–530·1)	-9·8% (-12 to -7%)	0·58
	YLDs	29·9 (21·5–40·1)	102·1% (90·9 to 114·9%)	73·8 (52·9–99)	-10·6% (-14·9 to -6%)	0·57
Spinal Injuries	Incidence	1·9 (1·6–2·4)	62·5% (50·5 to 74·2%)	4·8 (3·9–5·8)	-6·7% (-13·1 to -0·4%)	0·74
	Prevalence	67·8 (56·1–95·2)	165·6% (124·4 to 263·4%)	160·3 (134–220·9)	20·1% (2·5 to 61·7%)	0·70
	YLDs	21·5 (14·2–32·9)	146·5% (91·6 to 263·6%)	50·5 (33·5–75·6)	12·2% (-11·6 to 62·6%)	0·66
**Bahrain**						
Minor TBI	Incidence	1·7 (1·3–2·1)	157·9% (138·2 to 177·1%)	123 (96·6–156·2)	5·2% (-1·9 to 13%)	0·62
	Prevalence	1·5 (1·4–1·6)	314·7% (294·7 to 336·8%)	92·6 (85·6–100)	3·2% (-1·1 to 7·8%)	0·64
	YLDs	0·2 (0·1–0·3)	316·5% (261·9 to 381%)	11·1 (7·6–15·1)	3·4% (-7·8 to 14·9%)	0·63
Moderate/Severe TBI	Incidence	1·9 (1·7–2·2)	177·1% (156·5 to 197·5%)	136 (120–153·1)	-5·5% (-10·2 to -0·5%)	0·58
	Prevalence	6·8 (6·4–7·3)	331% (314·2 to 348%)	394·3 (372·7–419·7)	4·5% (1·3 to 7·9%)	0·57
	YLDs	1·1 (0·7–1·4)	323·1% (290·3 to 359·8%)	60·4 (42·7–82·4)	3·6% (-2·7 to 10·6%)	0·56
Spinal cord lesion at neck level	Incidence	<0·1 (<0·1–<0·1)	201·5% (172·1 to 230·5%)	2·5 (1·9–3·2)	4·2% (-6·1 to 14%)	0·70
	Prevalence	1·3 (1·2–1·5)	337·8% (300·2 to 368·3%)	72·1 (66–81·2)	14·4% (3·6 to 22·4%)	0·69
	YLDs	0·5 (0·4–0·7)	297·9% (232·5 to 381·3%)	27·6 (19·3–35·9)	4·7% (-11 to 24·4%)	0·67
Spinal cord lesion below neck level	Incidence	<0·1 (<0·1–<0·1)	205·3% (175·9 to 235%)	2·2 (1·6–2·8)	7·3% (-2·8 to 16·1%)	0·80
	Prevalence	1·1 (1·1–1·2)	361·3% (336·4 to 387·6%)	65·2 (61–69·6)	18·3% (12·3 to 24·5%)	0·79
	YLDs	0·2 (0·1–0·3)	228% (170·4 to 302·9%)	11·7 (8·1–15·9)	-15·1% (-28·3 to 0·3%)	0·77
Head Injuries	Incidence	3·6 (3·1–4·2)	167·7% (150·1 to 185%)	259 (224·4–299·4)	-0·7% (-5·5 to 4·4%)	0·60
	Prevalence	8·3 (7·9–8·8)	328% (314·1 to 342·3%)	486·9 (464·6–512·4)	4·2% (1·5 to 7·2%)	0·58
	YLDs	1·2 (0·9–1·7)	322·1% (292 to 355·1%)	71·4 (50·3–97)	3·6% (-2·3 to 9·8%)	0·57
Spinal Injuries	Incidence	0·1 (0·1–0·1)	203·2% (178·6 to 227·1%)	4·6 (3·8–5·6)	5·6% (-2·2 to 13·4%)	0·74
	Prevalence	2·5 (2·3–2·7)	348·4% (320·5 to 373·3%)	137·3 (127·8–148·6)	16·2% (8·9 to 22·2%)	0·73
	YLDs	0·7 (0·5–0·9)	274·7% (224·5 to 332·1%)	39·3 (27·9–51·1)	-2·1% (-13·7 to 11·7%)	0·70
**Egypt**						
Minor TBI	Incidence	106·8 (83·6–134·5)	84·5% (71·7 to 99·2%)	103 (80·9–129·6)	7·3% (0·5 to 15·6%)	0·68
	Prevalence	65·1 (60·2–70·7)	111·8% (102·8 to 121·1%)	78·1 (72·6–84)	7% (3·1 to 11%)	0·71
	YLDs	7·9 (5·5–10·8)	112·3% (86·8 to 142%)	9·4 (6·5–12·8)	7·1% (-4·7 to 20·2%)	0·69
Moderate/Severe TBI	Incidence	115·7 (101·6–130·6)	86·7% (75·9 to 97·2%)	126·7 (111·2–143·2)	3·7% (-1·1 to 8·8%)	0·67
	Prevalence	272·5 (258·4–287·4)	108·1% (101·6 to 115·2%)	318·5 (302·7–334·8)	3·6% (0·5 to 6·8%)	0·63
	YLDs	42·5 (29·7–57·7)	107·1% (92·8 to 124%)	49·2 (34·5–66·6)	3·4% (-3·2 to 10·6%)	0·62
Spinal cord lesion at neck level	Incidence	2·3 (1·7–3·2)	126·4% (96·4 to 189·9%)	2·5 (1·8–3·4)	23·4% (8·1 to 56·2%)	0·75
	Prevalence	47·9 (43·4–55)	138% (119·3 to 172·1%)	51·4 (47–58·3)	19·3% (10·8 to 34·9%)	0·75
	YLDs	19·4 (13·7–25·6)	121·4% (81·7 to 175·7%)	20·7 (14·7–27·2)	11·4% (-7·7 to 35·6%)	0·73
Spinal cord lesion below neck level	Incidence	1·7 (1·3–2·3)	93·4% (80·5 to 107·4%)	1·8 (1·4–2·5)	7·8% (1·2 to 15·1%)	0·92
	Prevalence	43·9 (41–46·7)	116·5% (106·6 to 127·8%)	48·7 (45·6–51·7)	8·5% (3·9 to 13·8%)	0·87
	YLDs	9·6 (6·5–12·7)	68·2% (39·8 to 99·9%)	10·5 (7·2–13·9)	-15·4% (-29·6 to -0·5%)	0·85
Head Injuries	Incidence	222·5 (191·8–257·4)	85·6% (74·8 to 96·2%)	229·8 (199·1–263·8)	5·3% (0·5 to 10·6%)	0·68
	Prevalence	337·6 (322·2–354·1)	108·8% (103·3 to 115·1%)	396·6 (378·8–415·3)	4·3% (1·7 to 7%)	0·64
	YLDs	50·3 (35·4–68·5)	107·9% (95 to 122·9%)	58·6 (41·4–79·3)	4% (-2·1 to 10·7%)	0·63
Spinal Injuries	Incidence	4·1 (3·3–5·1)	111·1% (92 to 145·7%)	4·3 (3·5–5·4)	16·3% (6·6 to 33·8%)	0·82
	Prevalence	91·7 (85·3–100·3)	127·2% (115·4 to 145%)	100·1 (93·4–108·4)	13·8% (8·4 to 21·8%)	0·81
	YLDs	29 (20·5–37·5)	100·5% (75·2 to 133·9%)	31·2 (22·2–40·3)	0·7% (-11·7 to 16·9%)	0·77
**Iran**						
Minor TBI	Incidence	102·3 (79·9–131·1)	-36·8% (-57·3 to -16·9%)	120·7 (94·2–154·7)	-54·9% (-70 to -40·3%)	0·53
	Prevalence	92·7 (81·9–110·1)	32·7% (11·3 to 53·3%)	108·5 (96·2–128·2)	-38·8% (-52·4 to -27·4%)	0·53
	YLDs	11·1 (8·1–14·9)	33·6% (11·9 to 54·5%)	12·9 (9·5–17·3)	-37·8% (-52·2 to -27%)	0·52
Moderate/Severe TBI	Incidence	122·4 (107·7–139·5)	-61·6% (-73·6 to -43·4%)	146·8 (129·6–166·5)	-74% (-81·9 to -62·1%)	0·54

Table S14 Incidence, prevalence, and disability of head and spinal injuries in the world and in North Africa and Middle East countries

		**All Ages**		**Age-standardised**		
		Number (thousand)	Percent change	Rate per 100,000	Percent change	Sex ratio
**Location**		2019	From 1990 to 2019	2019	From 1990 to 2019	2019
Cause	Measure	Mean (95% UI)	Mean (95% UI)	Mean (95% UI)	Mean (95% UI)	F:M
	Prevalence	386·2 (357·7–420·7)	64·8% (50·3 to 75·3%)	441·4 (410·4–478·8)	-23·9% (-31·9 to -18·9%)	0·52
	YLDs	59·1 (43·8–78·8)	56·3% (42·3 to 68·7%)	67·2 (49·8–89·3)	-25·9% (-33·4 to -20·8%)	0·51
Spinal cord lesion at neck level	Incidence	2·2 (1·7–2·9)	-82% (-91·4 to -59·8%)	2·7 (2–3·5)	-89·4% (-95·1 to -75·6%)	0·61
	Prevalence	118·8 (77·9–213·1)	1·3% (-24·5 to 52·5%)	127 (85·4–222·2)	-48·8% (-63·2 to -23·7%)	0·62
	YLDs	45·4 (26·1–81·3)	-8·2% (-31·9 to 36·5%)	48·3 (28·1–84·5)	-52·4% (-65·6 to -29·9%)	0·61
Spinal cord lesion below neck level	Incidence	1·9 (1·5–2·5)	-84·4% (-93·2 to -59·6%)	2·3 (1·7–3)	-91% (-96·2 to -76·1%)	0·70
	Prevalence	73·6 (61·6–95·4)	103·6% (93 to 116·7%)	82·1 (69·1–105·7)	-2·8% (-8·2 to 4·5%)	0·70
	YLDs	13·5 (8·9–19·3)	48·5% (35·7 to 60·3%)	15 (10–21·4)	-28% (-34·3 to -22%)	0·69
Head Injuries	Incidence	224·8 (194·6–259·6)	-53·3% (-64·7 to -38·8%)	267·5 (232·3–308·7)	-67·8% (-75·7 to -58%)	0·54
	Prevalence	478·9 (445·7–520·7)	57·4% (45·6 to 67·5%)	549·8 (513·4–594·8)	-27·4% (-34·4 to -22·2%)	0·52
	YLDs	70·2 (52·3–93·2)	52·2% (39·8 to 63·9%)	80·1 (59·6–106·4)	-28·1% (-34·8 to -23·2%)	0·51
Spinal Injuries	Incidence	4·2 (3·4–5·1)	-83·2% (-90·8 to -68·2%)	5 (4·1–6·1)	-90·2% (-94·8 to -81·2%)	0·65
	Prevalence	192·4 (147·6–289·6)	25·4% (-10·6 to 72·8%)	209·1 (162·9–308·2)	-37·1% (-55·4 to -15%)	0·65
	YLDs	58·9 (36·9–97·2)	0·7% (-26·5 to 40%)	63·3 (39·9–102)	-48·3% (-62·5 to -29·6%)	0·63
**Iraq**						
Minor TBI	Incidence	79·7 (61·6–103)	85·4% (54·6 to 111·5%)	172·9 (133·1–222·5)	-24·1% (-36·2 to -14·2%)	0·51
	Prevalence	83·8 (53·4–142·1)	112·7% (87·2 to 139·6%)	241·5 (154·3–413·9)	-28·4% (-41·1 to -15·2%)	0·52
	YLDs	10·1 (6·1–16·5)	115·5% (87·6 to 148·7%)	28·7 (17·4–47·3)	-26·7% (-39·5 to -12·7%)	0·52
Moderate/Severe TBI	Incidence	60·6 (53·8–68·2)	128·5% (110·5 to 141·9%)	148 (131·9–165·3)	-11·7% (-17·4 to -7·3%)	0·51
	Prevalence	177·4 (144–255·7)	144·3% (114 to 171·3%)	508·7 (417·6–728·7)	-18·1% (-30 to -8·2%)	0·47
	YLDs	27·2 (18·8–38·5)	145·2% (113·8 to 174·8%)	77·1 (53·4–107·8)	-17·1% (-29 to -6·5%)	0·46
Spinal cord lesion at neck level	Incidence	2·1 (1·2–3·9)	41·9% (13·7 to 91·4%)	4·8 (2·9–8·9)	-41·2% (-52·2 to -22·7%)	0·58
	Prevalence	251 (76–710·9)	88·9% (77·5 to 110·4%)	626·6 (193·4–1771·8)	-25·1% (-34·9 to -14%)	0·67
	YLDs	101·2 (30·5–290·1)	76·1% (63·5 to 99·5%)	250·3 (76·5–713·1)	-29% (-37·7 to -18%)	0·66
Spinal cord lesion below neck level	Incidence	1 (0·8–1·3)	147·2% (134·3 to 161·8%)	2·4 (1·9–3·1)	-4·5% (-9·2 to 0·5%)	0·66
	Prevalence	24·7 (23·2–26·3)	192·8% (182·8 to 204·7%)	67·9 (64·2–72·2)	-0·6% (-3·8 to 3·1%)	0·68
	YLDs	5·3 (3·6–7·1)	132·5% (98·8 to 172·3%)	14·3 (9·9–19·3)	-20·7% (-31·5 to -8·4%)	0·67
Head Injuries	Incidence	140·3 (119·4–166·1)	101·8% (77·4 to 121·4%)	320·9 (275·6–375·8)	-18·8% (-27·5 to -12·2%)	0·51
	Prevalence	261·2 (207·4–347·3)	133·2% (110 to 156·2%)	750·1 (597·1–996·4)	-21·7% (-31·2 to -12·7%)	0·49
	YLDs	37·3 (26·8–50·9)	136·4% (111·1 to 161·5%)	105·8 (76·4–143·3)	-19·9% (-30·1 to -10·6%)	0·48
Spinal Injuries	Incidence	3·1 (2·2–4·9)	65·2% (31·9 to 112·4%)	7·2 (5·2–11·3)	-32·4% (-44·7 to -15·8%)	0·61
	Prevalence	275·6 (100·5–735)	95·1% (81·3 to 125·1%)	694·5 (261·2–1838·6)	-23·3% (-32·7 to -11·6%)	0·68
	YLDs	106·4 (35–295·5)	78·2% (65·8 to 104·2%)	264·6 (89·6–729·2)	-28·6% (-37·1 to -18·4%)	0·66
**Jordan**						
Minor TBI	Incidence	13·4 (10·4–17·1)	184·5% (166·8 to 203·9%)	107 (83·2–136·3)	-3·2% (-8·6 to 2·9%)	0·62
	Prevalence	7·8 (7·1–8·5)	281·6% (265·9 to 299·7%)	78·7 (72·7–85·4)	-2·9% (-6·2 to 1%)	0·64
	YLDs	0·9 (0·6–1·3)	282·4% (236·3 to 331·8%)	9·5 (6·5–13)	-2·8% (-12·4 to 7·4%)	0·63
Moderate/Severe TBI	Incidence	12·7 (11·2–14·3)	197·7% (181·1 to 214·4%)	115·9 (102·9–129·2)	-8·3% (-12·5 to -3·8%)	0·59
	Prevalence	31·6 (29·9–33·5)	290·5% (279 to 302·4%)	324·1 (306·9–341·8)	-3·8% (-6·2 to -1·3%)	0·58
	YLDs	4·9 (3·5–6·8)	285·5% (258·6 to 315·4%)	49·9 (34·8–67·9)	-4·2% (-9·4 to 1·7%)	0·57
Spinal cord lesion at neck level	Incidence	0·2 (0·2–0·3)	220·9% (198·2 to 245·4%)	2·2 (1·7–2·8)	-1·1% (-8 to 6·1%)	0·70
	Prevalence	6·6 (5·9–7·8)	359·7% (320·8 to 442·6%)	61·8 (56·2–71·9)	17·5% (8·7 to 35·2%)	0·70
	YLDs	2·6 (1·8–3·5)	323·5% (249·5 to 427·2%)	24 (16·7–31·6)	8·9% (-8 to 31·2%)	0·68
Spinal cord lesion below neck level	Incidence	0·2 (0·2–0·3)	216·7% (193·8 to 239·8%)	1·9 (1·5–2·4)	-1·1% (-8·1 to 5·3%)	0·79
	Prevalence	5·8 (5·4–6·2)	310% (294 to 330·6%)	55·8 (52·5–59·4)	4·4% (0·6 to 9%)	0·79
	YLDs	1·1 (0·7–1·5)	200·7% (150·9 to 256·9%)	10·4 (7·1–14·2)	-22·8% (-33·6 to -10·5%)	0·77
Head Injuries	Incidence	26 (22·3–30·5)	190·8% (175·3 to 206·3%)	223 (193·7–258·6)	-5·9% (-10 to -1·2%)	0·60
	Prevalence	39·4 (37·5–41·4)	288·7% (279·4 to 299·1%)	402·8 (385·5–421·6)	-3·6% (-5·7 to -1·6%)	0·59
	YLDs	5·9 (4·1–8)	285% (261·2 to 310·9%)	59·4 (41·4–80·3)	-3·9% (-8·6 to 1%)	0·58
Spinal Injuries	Incidence	0·5 (0·4–0·5)	218·9% (200·2 to 239·5%)	4·1 (3·4–4·9)	-1·1% (-6·7 to 4·5%)	0·74

Table S14 Incidence, prevalence, and disability of head and spinal injuries in the world and in North Africa and Middle East countries

		All Ages		Age-standardised		
		Number (thousand)	Percent change	Rate per 100,000	Percent change	Sex ratio
**Location**		2019	From 1990 to 2019	2019	From 1990 to 2019	2019
Cause	Measure	Mean (95% UI)	Mean (95% UI)	Mean (95% UI)	Mean (95% UI)	F:M
	Prevalence	12·4 (11·4–13·7)	335·1% (312·7 to 373·3%)	117·6 (109·5–128·8)	10·9% (5·8 to 19·5%)	0·74
	YLDs	3·7 (2·6–4·8)	277·9% (228 to 346·7%)	34·4 (24·2–44·8)	-3·2% (-14·6 to 11·5%)	0·71
**Kuwait**						
Minor TBI	Incidence	6 (4·6–7·7)	-31% (-67·8 to 33·6%)	132·2 (102·6–168·1)	-69·6% (-85·6 to -42·1%)	0·54
	Prevalence	4·8 (4·3–5·4)	129·6% (66·3 to 182·4%)	106·5 (96·7–119·3)	-21·6% (-38·9 to -9·6%)	0·55
	YLDs	0·6 (0·4–0·8)	135% (70·4 to 199·6%)	12·8 (9–17·3)	-20·7% (-37·8 to -5·4%)	0·55
Moderate/Severe TBI	Incidence	6·5 (5·7–7·3)	59% (7·2 to 106·8%)	147·9 (130·9–164·5)	-36·9% (-56·1 to -20·4%)	0·48
	Prevalence	20·2 (18·9–21·6)	199·7% (189·1 to 210·7%)	443·3 (419·6–473·3)	-8·8% (-11·4 to -6·3%)	0·49
	YLDs	3·1 (2·2–4·2)	192·9% (172·4 to 214·4%)	67·8 (48·4–91·6)	-10·2% (-15·3 to -5%)	0·49
Spinal cord lesion at neck level	Incidence	0·1 (0·1–0·2)	-82·1% (-93·6 to -37·9%)	2·7 (2–3·4)	-92·1% (-97·3 to -73·4%)	0·59
	Prevalence	5·5 (4–9·3)	280·2% (233·8 to 345·5%)	107·9 (80·3–176·4)	21·5% (6·5 to 45·7%)	0·67
	YLDs	2·1 (1·3–3·6)	248·5% (188·7 to 319·3%)	40·3 (25·6–66·9)	12·3% (-5·5 to 32·7%)	0·66
Spinal cord lesion below neck level	Incidence	0·1 (0·1–0·1)	151·7% (132 to 171·8%)	2·3 (1·8–3)	-2·7% (-9·8 to 3·7%)	0·68
	Prevalence	3·4 (3·2–3·7)	225·2% (212·3 to 239·7%)	70·8 (66·4–75·4)	1·3% (-2·5 to 5·3%)	0·72
	YLDs	0·6 (0·4–0·8)	168·6% (124·8 to 218·6%)	11·7 (8·1–16·2)	-15·8% (-27·5 to -2·6%)	0·71
Head Injuries	Incidence	12·5 (10·8–14·6)	-2·2% (-45·2 to 51·3%)	280·1 (242·7–323·8)	-58·2% (-76 to -37·2%)	0·51
	Prevalence	24·9 (23·7–26·5)	183·2% (157·4 to 200%)	549·8 (523·6–578·6)	-11·6% (-17·4 to -7·7%)	0·50
	YLDs	3·7 (2·6–5)	182% (157·4 to 204·3%)	80·6 (57·8–108·6)	-12% (-18 to -6·7%)	0·50
Spinal Injuries	Incidence	0·2 (0·2–0·3)	-68·3% (-87·9 to -5·2%)	5 (4·1–6)	-86·3% (-94·9 to -60·5%)	0·63
	Prevalence	8·9 (7·3–12·7)	257·1% (228·7 to 305%)	178·7 (149·6–246·3)	12·6% (3·7 to 28·4%)	0·69
	YLDs	2·7 (1·7–4·2)	227·6% (179·1 to 283·8%)	52 (34·5–79·6)	4·4% (-10·2 to 21%)	0·67
**Lebanon**						
Minor TBI	Incidence	6·2 (4·9–8)	-28·5% (-59·5 to 8·4%)	121·8 (94·8–155·1)	-51·8% (-72·3 to -28·3%)	0·58
	Prevalence	7·3 (5·2–12·2)	11·6% (-8·8 to 42·1%)	138·3 (97·8–231·3)	-40·5% (-51·5 to -25%)	0·58
	YLDs	0·9 (0·6–1·4)	10·6% (-10·9 to 43·9%)	16·4 (10·5–27·1)	-40·7% (-52·2 to -24·1%)	0·58
Moderate/Severe TBI	Incidence	6·5 (5·8–7·3)	28% (2 to 48·4%)	126·1 (111·8–141·2)	-24% (-38·7 to -12·5%)	0·59
	Prevalence	20·7 (18·1–27·1)	41·4% (10·6 to 67·1%)	389·8 (339·9–510·3)	-26·8% (-41·5 to -14·8%)	0·53
	YLDs	3·2 (2·2–4·4)	40·3% (8·9 to 67%)	59·3 (42·2–82·9)	-26·9% (-41·4 to -14·3%)	0·52
Spinal cord lesion at neck level	Incidence	0·1 (0·1–0·2)	-72·6% (-89·2 to -22·9%)	2·5 (1·9–3·2)	-82·3% (-93 to -51·3%)	0·71
	Prevalence	18·1 (5·5–58·1)	-21·4% (-30·5 to 12·7%)	338·9 (102·6–1087)	-54·1% (-60 to -36·4%)	0·77
	YLDs	6·7 (1·9–20)	-30·7% (-39·1 to 0·8%)	124·9 (35·6–375·8)	-59·3% (-64·8 to -42·7%)	0·76
Spinal cord lesion below neck level	Incidence	0·1 (0·1–0·1)	59·3% (34·4 to 87·4%)	2·2 (1·7–2·8)	-4·9% (-20·5 to 11·3%)	0·79
	Prevalence	3·3 (3·1–3·5)	79·9% (69·7 to 89·7%)	61·4 (57·6–65·5)	-6·9% (-12·3 to -1·8%)	0·78
	YLDs	0·5 (0·4–0·7)	24·1% (4 to 45·4%)	10·2 (6·9–13·8)	-35·4% (-45·4 to -24·5%)	0·76
Head Injuries	Incidence	12·8 (11·1–14·8)	-7·7% (-38·2 to 21·4%)	247·9 (215·1–288·1)	-40·8% (-59·4 to -23·5%)	0·59
	Prevalence	28 (23·8–37·1)	32·2% (7·4 to 57·3%)	528·1 (446·9–699·7)	-30·9% (-42·9 to -19·1%)	0·54
	YLDs	4 (2·8–5·6)	32·7% (7 to 58·8%)	75·7 (53·6–105·1)	-30·4% (-42·8 to -18%)	0·54
Spinal Injuries	Incidence	0·2 (0·2–0·3)	-55·5% (-81·3 to 1·8%)	4·7 (3·9–5·7)	-71·5% (-87·7 to -37·5%)	0·75
	Prevalence	21·4 (8·8–61·4)	-14% (-27 to 31·1%)	400·3 (164·1–1148·2)	-50·2% (-57·6 to -27·9%)	0·77
	YLDs	7·2 (2·4–20·6)	-28·3% (-37·9 to 4·3%)	135 (45·1–386·4)	-58·1% (-63·8 to -41·5%)	0·76
**Libya**						
Minor TBI	Incidence	14·2 (10·4–21·1)	143·2% (88·7 to 262·8%)	203·8 (147·6–306·1)	55·9% (19·3 to 138·8%)	0·52
	Prevalence	9·6 (7·9–13)	202·8% (167 to 257%)	141·4 (116·4–187·5)	30·8% (14·8 to 56·5%)	0·53
	YLDs	1·2 (0·8–1·6)	201% (154·1 to 259·1%)	16·9 (11·8–23·5)	29·7% (10 to 56·5%)	0·52
Moderate/Severe TBI	Incidence	12·5 (11–14·7)	108·8% (90·7 to 134·3%)	180·8 (159–210·7)	10·2% (1·7 to 23·9%)	0·52
	Prevalence	33·5 (30·9–37·6)	166·2% (154·2 to 184·5%)	485·9 (450·3–543)	7% (2·4 to 15·1%)	0·50
	YLDs	5·2 (3·7–6·9)	162·4% (143·6 to 185·4%)	74·2 (52·8–99·5)	6·3% (-0·6 to 15·8%)	0·49
Spinal cord lesion at neck level	Incidence	0·6 (0·3–1·5)	542·7% (203·5 to 1469·4%)	9·4 (4·5–22)	251·6% (64·1 to 771·8%)	0·57
	Prevalence	15·7 (7·7–36·4)	317·9% (229·1 to 390·4%)	213·7 (105·7–498·9)	103·4% (44·1 to 161·2%)	0·58
	YLDs	6·3 (2·9–14·6)	291·7% (199·9 to 372%)	85·2 (39·5–199·7)	92·9% (34·9 to 150·8%)	0·57

Table S14 Incidence, prevalence, and disability of head and spinal injuries in the world and in North Africa and Middle East countries

		**All Ages**		**Age-standardised**		
		Number (thousand)	Percent change	Rate per 100,000	Percent change	Sex ratio
**Location**		2019	From 1990 to 2019	2019	From 1990 to 2019	2019
Cause	Measure	Mean (95% UI)	Mean (95% UI)	Mean (95% UI)	Mean (95% UI)	F:M
Spinal cord lesion below neck level	Incidence	0·2 (0·1–0·2)	105·8% (85·6 to 136·4%)	2·6 (1·9–3·4)	13% (2·7 to 28·1%)	0·67
	Prevalence	5·2 (4·9–5·8)	181·7% (164·7 to 207·5%)	73·3 (68·3–80·3)	16·8% (10·2 to 26·3%)	0·65
	YLDs	1·1 (0·7–1·4)	130·5% (94·9 to 169·6%)	14·9 (10·5–19·7)	-3·6% (-16·7 to 11·2%)	0·64
Head Injuries	Incidence	26·8 (22·3–34·2)	125·8% (95·7 to 182%)	384·6 (320·5–493·9)	30·4% (12·6 to 66%)	0·52
	Prevalence	43·1 (39·6–47·9)	173·6% (159·7 to 191·3%)	627·3 (578·7–693·6)	11·6% (5·9 to 19·3%)	0·51
	YLDs	6·3 (4·6–8·5)	168·7% (151·7 to 189·7%)	91·1 (66·4–122·5)	9·9% (3·2 to 18·6%)	0·50
Spinal Injuries	Incidence	0·8 (0·5–1·6)	338·9% (157·3 to 790%)	11·9 (6·8–24·4)	141·6% (40·5 to 405·8%)	0·59
	Prevalence	21 (12·9–41·6)	272·9% (206·2 to 346·6%)	287 (176·8–572·8)	71% (31·2 to 126·2%)	0·60
	YLDs	7·4 (3·8–15·7)	255·6% (178·8 to 331·9%)	100·1 (52–214·7)	67·9% (21·6 to 123·9%)	0·58
**Morocco**						
Minor TBI	Incidence	47·9 (37·3–61·4)	33·2% (24·6 to 42·7%)	131·1 (102–167·6)	0·2% (-5·8 to 6·7%)	0·60
	Prevalence	34·6 (32·1–37·6)	80·3% (73·5 to 87·9%)	98·8 (91·7–107·1)	0·3% (-2·9 to 3·8%)	0·61
	YLDs	4·2 (2·9–5·7)	79·8% (60·4 to 99·3%)	11·9 (8·2–16·2)	0% (-9·2 to 9·5%)	0·60
Moderate/Severe TBI	Incidence	54·3 (48–60·8)	55% (47 to 63·1%)	154·8 (136·6–173·5)	3% (-1·3 to 7·1%)	0·59
	Prevalence	144·8 (136·9–153·5)	93·4% (86·3 to 99·3%)	404·5 (383·4–427·9)	3·5% (0·3 to 6·2%)	0·55
	YLDs	22·3 (15·8–30·2)	90·8% (79·1 to 102·5%)	62·1 (43·8–84·1)	3% (-2·8 to 8·8%)	0·54
Spinal cord lesion at neck level	Incidence	1 (0·7–1·3)	61·2% (51 to 72·8%)	2·8 (2·1–3·6)	8% (1·4 to 14·9%)	0·77
	Prevalence	25·3 (23·1–28·9)	69% (34·9 to 92·6%)	68·3 (62·4–77·6)	-0·4% (-16·5 to 10·4%)	0·73
	YLDs	10·3 (7·4–13·4)	55·9% (19·7 to 92·1%)	27·7 (19·8–35·9)	-7·4% (-25·3 to 10·4%)	0·71
Spinal cord lesion below neck level	Incidence	0·9 (0·7–1·1)	59·1% (48·6 to 71·5%)	2·4 (1·8–3·2)	8% (1·6 to 14·8%)	0·88
	Prevalence	23·8 (22·4–25·6)	96·9% (88·7 to 107%)	65·1 (61–69·8)	9·2% (5·1 to 14·4%)	0·82
	YLDs	5·5 (3·9–7·3)	57·3% (33·8 to 81·8%)	15 (10·6–19·9)	-12·1% (-24·2 to 0·7%)	0·80
Head Injuries	Incidence	102·2 (88·9–118·8)	44% (37 to 51·2%)	285·9 (249·6–331·2)	1·7% (-2·5 to 5·9%)	0·60
	Prevalence	179·5 (171·4–188·3)	90·7% (85 to 95·5%)	503·3 (481·4–527·5)	2·9% (0·2 to 5%)	0·56
	YLDs	26·5 (18·7–35·9)	89% (78·7 to 99·1%)	73·9 (52–100·1)	2·5% (-2·9 to 7·6%)	0·55
Spinal Injuries	Incidence	1·8 (1·5–2·2)	60·2% (51·6 to 69·8%)	5·2 (4·3–6·3)	8% (2·9 to 13·7%)	0·82
	Prevalence	49·1 (45·7–53·6)	81·5% (56·8 to 96·1%)	133·4 (124·2–145·2)	4·1% (-6·6 to 10·5%)	0·77
	YLDs	15·8 (11·4–20·4)	56·4% (29·9 to 80·1%)	42·7 (30·9–55)	-9·1% (-21·4 to 2·7%)	0·74
**Palestine**						
Minor TBI	Incidence	7 (5·4–8·7)	26·3% (-27·5 to 91·7%)	127·9 (100·2–160·9)	-44·7% (-67·6 to -18·1%)	0·58
	Prevalence	8·3 (5·1–14·7)	201·7% (174·9 to 237·3%)	214·1 (131·3–372·1)	20·5% (9 to 34·4%)	0·53
	YLDs	1 (0·6–1·7)	203·2% (163·2 to 253·3%)	25·4 (15·1–42·3)	20·4% (4·8 to 39·1%)	0·53
Moderate/Severe TBI	Incidence	5·7 (5–6·4)	113·9% (63·7 to 151·3%)	126·6 (112·6–141·1)	-10·4% (-29 to 3·1%)	0·58
	Prevalence	17·9 (13·9–27·3)	187·1% (161·2 to 203·5%)	474·3 (371·7–721·2)	8·1% (0 to 12·7%)	0·47
	YLDs	2·7 (1·8–4)	183·8% (156·8 to 209·1%)	71·5 (48·5–103·9)	6·9% (-1·5 to 13·9%)	0·47
Spinal cord lesion at neck level	Incidence	0·1 (0·1–0·2)	-53·6% (-75·6 to 17·5%)	3·1 (2·3–4·5)	-78·2% (-88·7 to -46·3%)	0·65
	Prevalence	31·9 (9·4–90·5)	169·9% (145·5 to 183·3%)	742·4 (223·5–2100·8)	19·5% (6·1 to 26·6%)	0·64
	YLDs	12·7 (3·7–36·5)	152·4% (126·9 to 173·8%)	294·1 (86·7–825·7)	12% (-1·2 to 20·9%)	0·64
Spinal cord lesion below neck level	Incidence	0·1 (0·1–0·1)	182·1% (162·2 to 208·6%)	2·3 (1·8–2·9)	15·4% (8 to 25·7%)	0·75
	Prevalence	2·8 (2·4–3·5)	128·4% (60·1 to 198·9%)	71·4 (60·7–96·1)	-10·4% (-30·8 to 8%)	0·68
	YLDs	0·6 (0·4–0·9)	90·4% (28·8 to 168·5%)	14·9 (9·9–22·4)	-25·6% (-44·5 to -2·5%)	0·67
Head Injuries	Incidence	12·6 (10·9–14·7)	54·8% (1·8 to 106·4%)	254·5 (221–295·7)	-31·7% (-53·3 to -12·3%)	0·58
	Prevalence	26·2 (19·9–36·2)	191·6% (168·6 to 209·5%)	688·4 (527·1–948·2)	11·7% (4·9 to 17·8%)	0·49
	YLDs	3·7 (2·6–5·1)	188·7% (163·9 to 212·4%)	97 (69·2–133·3)	10·2% (2·1 to 17·8%)	0·49
Spinal Injuries	Incidence	0·3 (0·2–0·3)	-29% (-65·1 to 58·3%)	5·4 (4·4–6·8)	-66·9% (-83·5 to -29·6%)	0·69
	Prevalence	34·6 (12–93)	166% (136·8 to 180·4%)	813·9 (289·2–2164·2)	16·1% (1·3 to 24%)	0·65
	YLDs	13·3 (4·3–37·1)	148·8% (118·7 to 168·3%)	309 (100·2–843·4)	9·3% (-5·8 to 17·9%)	0·64
**Oman**						
Minor TBI	Incidence	7·9 (6·1–10)	130·7% (110·9 to 151·6%)	160·4 (125·9–202·6)	-6·3% (-12·8 to 0·2%)	0·57
	Prevalence	4·9 (4·5–5·3)	186·1% (174·5 to 198·9%)	123·1 (114·3–132·7)	-6·7% (-9·6 to -3·3%)	0·60

Table S14 Incidence, prevalence, and disability of head and spinal injuries in the world and in North Africa and Middle East countries

		All Ages		Age-standardised		
		Number (thousand)	Percent change	Rate per 100,000	Percent change	Sex ratio
**Location**		2019	From 1990 to 2019	2019	From 1990 to 2019	2019
Cause	Measure	Mean (95% UI)	Mean (95% UI)	Mean (95% UI)	Mean (95% UI)	F:M
	YLDs	0·6 (0·4–0·8)	187·2% (151·7 to 228·9%)	14·7 (10·1–20·3)	-7·1% (-16·1 to 3·1%)	0·59
Moderate/Severe TBI	Incidence	9·2 (8–10·6)	136·4% (118·7 to 154%)	225·6 (197·4–254·5)	-7·8% (-13·2 to -2·1%)	0·60
	Prevalence	23·3 (21·8–24·8)	199·2% (190·9 to 207·4%)	555·9 (526·6–589)	-4·6% (-6·9 to -2·4%)	0·57
	YLDs	3·7 (2·5–5)	198·1% (178·1 to 217·8%)	85·5 (60·2–116·3)	-5·1% (-9·6 to -0·1%)	0·56
Spinal cord lesion at neck level	Incidence	0·2 (0·1–0·2)	151·3% (130·4 to 178·4%)	3·7 (2·7–5)	-1·7% (-10·1 to 8%)	0·66
	Prevalence	3·9 (3·6–4·2)	244·2% (226·7 to 260·7%)	82 (76·4–88·2)	11·7% (7 to 16·9%)	0·63
	YLDs	1·5 (1–2)	215·3% (160·8 to 278·1%)	31·1 (21·6–40·7)	2% (-12·4 to 18·4%)	0·62
Spinal cord lesion below neck level	Incidence	0·1 (0·1–0·2)	154·3% (130·3 to 180·2%)	3·1 (2·3–4·3)	0·9% (-8·1 to 10·2%)	0·78
	Prevalence	3·5 (3·3–3·8)	232% (215·9 to 249·7%)	78·2 (72·9–83·7)	6·9% (2·3 to 12·1%)	0·74
	YLDs	0·6 (0·4–0·8)	134·1% (91·5 to 183·7%)	13·3 (9·1–17·9)	-24·6% (-36 to -11·4%)	0·72
Head Injuries	Incidence	17·1 (14·7–19·9)	133·7% (117·4 to 152·2%)	386 (336·3–438·3)	-7·2% (-11·9 to -2%)	0·59
	Prevalence	28·1 (26·7–29·8)	196·9% (189 to 204·2%)	679 (649·3–713·3)	-5% (-7·1 to -3·1%)	0·58
	YLDs	4·2 (2·9–5·8)	196·5% (179·6 to 214·8%)	100·2 (70·5–136·3)	-5·4% (-9·6 to -0·8%)	0·57
Spinal Injuries	Incidence	0·3 (0·2–0·4)	152·6% (134·3 to 174·8%)	6·8 (5·4–8·5)	-0·5% (-7·3 to 6·9%)	0·71
	Prevalence	7·4 (6·9–8)	238·3% (223·6 to 252·6%)	160·2 (150·2–171·2)	9·3% (5·5 to 13·7%)	0·68
	YLDs	2·1 (1·5–2·8)	186·5% (148·6 to 232·1%)	44·4 (31·2–57·6)	-7·8% (-17·9 to 4·1%)	0·65
**Qatar**						
Minor TBI	Incidence	5·9 (4·4–7·8)	507·3% (454·1 to 560%)	172·2 (131·9–224·1)	-8·8% (-15·3 to -2%)	0·56
	Prevalence	3·5 (3·2–3·9)	570·1% (540·5 to 601·1%)	126·8 (117·1–137·9)	-9·4% (-12·8 to -5·8%)	0·59
	YLDs	0·4 (0·3–0·6)	570·2% (479·3 to 670·4%)	15·1 (10·4–20·8)	-10% (-19 to 0%)	0·58
Moderate/Severe TBI	Incidence	6 (5·2–6·8)	494·4% (457·7 to 532·6%)	201·8 (178·6–226·2)	-10·8% (-15·1 to -5·7%)	0·56
	Prevalence	15·5 (14·4–16·7)	558·5% (539·7 to 582%)	537·2 (509·1–572·8)	-9·8% (-12·2 to -7%)	0·54
	YLDs	2·4 (1·7–3·3)	555·4% (509·1 to 605·4%)	82·1 (58·3–112·1)	-10·6% (-15·3 to -5·6%)	0·53
Spinal cord lesion at neck level	Incidence	0·1 (0·1–0·1)	543·4% (481·6 to 605·7%)	3·6 (2·7–4·7)	-3·9% (-12·8 to 4·7%)	0·62
	Prevalence	2·8 (2·6–3·1)	617·8% (580·9 to 658·7%)	87·9 (81·6–95)	0·4% (-4·3 to 5·2%)	0·64
	YLDs	1·1 (0·7–1·4)	551·9% (436·1 to 676·7%)	32·9 (23·2–42·7)	-9·4% (-21·7 to 3·9%)	0·63
Spinal cord lesion below neck level	Incidence	0·1 (0·1–0·1)	552·3% (487·8 to 614·1%)	3·1 (2·3–4·1)	-2·3% (-11·2 to 6·5%)	0·70
	Prevalence	2·6 (2·4–2·8)	618·3% (581 to 653·8%)	83·6 (77·5–89·7)	-1·5% (-6·2 to 3%)	0·72
	YLDs	0·4 (0·3–0·6)	396·5% (302·9 to 501·6%)	13·8 (9·3–19·1)	-32·1% (-42·1 to -21·3%)	0·71
Head Injuries	Incidence	11·9 (10·1–14·1)	500·7% (462·3 to 540·7%)	374 (324·5–433·9)	-9·9% (-14·5 to -5·1%)	0·56
	Prevalence	19 (17·9–20·2)	560·7% (543·2 to 581%)	663·9 (631·7–701·2)	-9·7% (-11·8 to -7·3%)	0·55
	YLDs	2·9 (2–3·9)	557·5% (514·3 to 600·8%)	97·2 (68·5–132·2)	-10·5% (-14·8 to -6·2%)	0·54
Spinal Injuries	Incidence	0·2 (0·2–0·2)	547·5% (494·8 to 597%)	6·7 (5·5–8·1)	-3·2% (-10·3 to 3·5%)	0·66
	Prevalence	5·4 (5–5·9)	618% (588·7 to 648%)	171·5 (159·9–184·2)	-0·5% (-4·4 to 3·4%)	0·68
	YLDs	1·5 (1·1–2)	498% (415·6 to 588·7%)	46·7 (32·9–61·2)	-17·5% (-26·8 to -8·3%)	0·65
**Saudi Arabia**						
Minor TBI	Incidence	117·1 (86·1–157·9)	197·8% (166·1 to 230·5%)	284·5 (210·9–377·8)	22·1% (11·4 to 32%)	0·40
	Prevalence	72·4 (64·7–81·7)	289·9% (266·5 to 315·1%)	215·3 (195·4–239·6)	25% (18·7 to 31·4%)	0·44
	YLDs	8·8 (6·1–12·2)	290·8% (251·8 to 331·7%)	25·6 (17·7–35·4)	24·5% (14·3 to 35·2%)	0·43
Moderate/Severe TBI	Incidence	125·1 (108·6–142·1)	251% (230·8 to 271·9%)	343·4 (299·5–385·7)	28·7% (23·3 to 33·6%)	0·49
	Prevalence	297·4 (274·9–323·9)	318% (301·9 to 333·9%)	855·4 (798–919·3)	25·6% (22·1 to 29%)	0·47
	YLDs	46·3 (32·1–64)	315% (290·4 to 340·2%)	130·5 (91·7–179·2)	25% (19·6 to 31·1%)	0·46
Spinal cord lesion at neck level	Incidence	2·3 (1·7–3)	260·3% (233·4 to 287·8%)	6·2 (4·5–8·3)	34·4% (25·8 to 43·2%)	0·54
	Prevalence	50·1 (45·5–55·1)	326·2% (303·3 to 352·5%)	126·1 (115·6–137·9)	29·1% (23·1 to 35·6%)	0·51
	YLDs	19·3 (13·5–25·4)	288·1% (238·2 to 346·9%)	47·8 (33·5–61·8)	17·9% (3·9 to 33·1%)	0·50
Spinal cord lesion below neck level	Incidence	2 (1·5–2·7)	248·3% (220·3 to 275·4%)	5·5 (4–7·3)	32% (23·1 to 40·7%)	0·59
	Prevalence	49·2 (45·2–53·9)	312·1% (288·7 to 337%)	130·3 (121–141·7)	25·5% (19·3 to 31·9%)	0·54
	YLDs	8·7 (5·9–12)	187·4% (146·5 to 235·9%)	22·8 (15·4–30·7)	-12·2% (-24 to 0·8%)	0·54
Head Injuries	Incidence	242·2 (207–286·7)	223·1% (200·7 to 246·1%)	627·9 (541·6–735)	25·6% (19·3 to 32·2%)	0·45
	Prevalence	369·8 (344·8–397·3)	312·2% (298·2 to 325·3%)	1070·7 (1008·3–1140)	25·5% (22·3 to 28·3%)	0·46

Table S14 Incidence, prevalence, and disability of head and spinal injuries in the world and in North Africa and Middle East countries

		All Ages		Age-standardised		
		Number (thousand)	Percent change	Rate per 100,000	Percent change	Sex ratio
**Location**		2019	From 1990 to 2019	2019	From 1990 to 2019	2019
Cause	Measure	Mean (95% UI)	Mean (95% UI)	Mean (95% UI)	Mean (95% UI)	F:M
	YLDs	55·1 (38·6–75·3)	310·9% (289·1 to 332·1%)	156·1 (109·5–212·2)	25% (20·1 to 29·7%)	0·45
Spinal Injuries	Incidence	4·3 (3·5–5·3)	254·6% (231·5 to 279·4%)	11·7 (9·3–14·4)	33·3% (26·1 to 40·4%)	0·56
	Prevalence	99·2 (90·7–108·8)	319·1% (298·7 to 340·4%)	256·4 (236·7–279·5)	27·2% (21·8 to 32·6%)	0·53
	YLDs	28 (19·9–36·8)	249·9% (213·8 to 290·2%)	70·6 (50·1–92·2)	6·2% (-3·8 to 16·6%)	0·51
**Syrian Arab Republic**						
Minor TBI	Incidence	30·7 (19·6–53·8)	106·8% (42 to 263%)	235 (142·5–434·7)	128·9% (47·1 to 316·5%)	0·75
	Prevalence	37·6 (19·7–71·9)	389% (193 to 722·1%)	286·9 (147·5–571·1)	234·4% (91·4 to 478·2%)	0·63
	YLDs	4·5 (2·4–8·4)	381·2% (188 to 723%)	33·9 (17·7–64)	227·3% (88·4 to 477·2%)	0·62
Moderate/Severe TBI	Incidence	22·1 (17·4–31·6)	77·7% (44·4 to 150·7%)	178·5 (129·9–286·4)	63·9% (21·8 to 156·4%)	0·69
	Prevalence	74·3 (52·4–129·4)	200·3% (121·1 to 369·7%)	546·8 (376·2–974·9)	84·6% (31·4 to 194·7%)	0·51
	YLDs	11·3 (7·1–18·4)	190·3% (112·9 to 363%)	82·5 (51·2–136·2)	80·6% (30·3 to 196·7%)	0·51
Spinal cord lesion at neck level	Incidence	2·4 (0·8–6·8)	829·2% (215·8 to 2114·1%)	23·3 (6·6–66·7)	960·5% (221·7 to 2587·9%)	0·98
	Prevalence	138·4 (38–399·5)	1049·3% (458·8 to 2231·5%)	989·8 (275·3–2883·5)	801·4% (319·5 to 1724%)	0·55
	YLDs	55 (14·6–158·3)	957·7% (407·5 to 2102·3%)	391·5 (102·9–1140·2)	734·9% (290·4 to 1616·7%)	0·54
Spinal cord lesion below neck level	Incidence	0·3 (0·2–0·4)	50·5% (35·9 to 73·6%)	2·4 (1·8–3·1)	34% (17·3 to 67·3%)	1·06
	Prevalence	8·4 (7·8–9·1)	98·9% (88·9 to 110·6%)	59·1 (54·6–63·9)	25% (18·5 to 33·2%)	0·98
	YLDs	1·7 (1·2–2·3)	47·4% (23·8 to 75·6%)	11·7 (8·2–16)	-6·4% (-19·9 to 9·5%)	0·96
Head Injuries	Incidence	52·8 (39·5–76·8)	93·5% (49·1 to 182·8%)	413·5 (297·4–625·7)	95·4% (43·6 to 192·7%)	0·73
	Prevalence	111·9 (77·5–171·1)	245·1% (153·3 to 392·3%)	833·7 (566·3–1291·1)	118·2% (55·8 to 220·8%)	0·55
	YLDs	15·8 (10·6–23)	227·2% (146 to 379·1%)	116·4 (75·7–170)	107·7% (51·8 to 211·9%)	0·54
Spinal Injuries	Incidence	2·8 (1·1–7·1)	482·8% (137·9 to 1262·7%)	25·7 (8·8–69·2)	548·3% (131·1 to 1510·3%)	0·99
	Prevalence	146·8 (46·4–407·9)	801·9% (347 to 1631·3%)	1048·9 (332·6–2942)	567·8% (215·4 to 1184·7%)	0·57
	YLDs	56·7 (16·1–159·7)	794·6% (325·3 to 1703·5%)	403·2 (112·6–1150·3)	579·2% (218·2 to 1263·6%)	0·55
**Tunisia**						
Minor TBI	Incidence	14·2 (11·1–18·1)	26·7% (17·9 to 35·4%)	125·3 (97·1–160·4)	1% (-4·9 to 7·5%)	0·54
	Prevalence	11·8 (11–12·7)	94·2% (86·2 to 102·1%)	94·1 (87·5–101·3)	2·7% (-0·5 to 5·7%)	0·57
	YLDs	1·4 (1–2)	93% (72·6 to 115·7%)	11·3 (7·8–15·5)	2·3% (-7·9 to 13·4%)	0·56
Moderate/Severe TBI	Incidence	17·5 (15·5–19·8)	59·1% (49·6 to 69·2%)	148·4 (130·9–166·6)	5·4% (0·9 to 9·9%)	0·56
	Prevalence	51·8 (49·3–54·7)	106% (100·6 to 111·8%)	401·9 (382·3–424·2)	4·3% (2 to 7·1%)	0·56
	YLDs	8 (5·6–10·9)	102·6% (90·6 to 115·6%)	61·7 (43·4–84·2)	3·8% (-1·7 to 9·7%)	0·55
Spinal cord lesion at neck level	Incidence	0·3 (0·2–0·4)	60·3% (45·7 to 75·1%)	2·6 (2–3·5)	7·6% (-1·7 to 16·9%)	0·64
	Prevalence	8·5 (7·9–9·2)	103·7% (92·5 to 115·1%)	66·5 (61·8–72)	10·7% (4·8 to 17·2%)	0·67
	YLDs	3·3 (2·3–4·2)	86·9% (57·7 to 116·2%)	25·4 (18·1–32·9)	2·8% (-11·4 to 17·7%)	0·65
Spinal cord lesion below neck level	Incidence	0·3 (0·2–0·4)	53·3% (38·3 to 70%)	2·2 (1·7–3)	4·4% (-4·9 to 13·8%)	0·75
	Prevalence	8·2 (7·6–8·8)	99·6% (88·9 to 109·6%)	63·4 (59·5–68·5)	6·5% (1·3 to 11·7%)	0·75
	YLDs	1·5 (1–2)	47·8% (25·3 to 72·8%)	11·3 (7·8–15·5)	-20·3% (-31·9 to -7·5%)	0·74
Head Injuries	Incidence	31·8 (27·6–36·6)	42·7% (34·4 to 51·4%)	273·7 (237·6–316·6)	3·3% (-1 to 8·1%)	0·55
	Prevalence	63·6 (60·8–66·7)	103·7% (98·7 to 108·9%)	496 (474·4–519)	4% (1·9 to 6·4%)	0·56
	YLDs	9·4 (6·6–12·8)	101·1% (90·9 to 112·3%)	73 (51·3–99·5)	3·5% (-1·5 to 8·8%)	0·55
Spinal Injuries	Incidence	0·6 (0·5–0·7)	57% (45·4 to 69·3%)	4·9 (4–6)	6·1% (-0·9 to 13%)	0·69
	Prevalence	16·7 (15·7–17·9)	101·7% (91·9 to 110·6%)	129·9 (121·8–139·6)	8·6% (3·7 to 13·4%)	0·71
	YLDs	4·7 (3·4–6·1)	72·8% (51·4 to 94·8%)	36·7 (26·5–47·6)	-5·7% (-16 to 5·6%)	0·68
**Türkiye**						
Minor TBI	Incidence	97·6 (76·1–124·4)	31·9% (17·9 to 47·6%)	120·6 (94·2–153·3)	4·8% (-5·2 to 16·7%)	0·62
	Prevalence	83·4 (76·7–91·2)	115% (103·9 to 127·6%)	93·3 (85·8–102)	16·9% (11·6 to 23·5%)	0·64

Table S14 Incidence, prevalence, and disability of head and spinal injuries in the world and in North Africa and Middle East countries

		**All Ages**		**Age-standardised**		
		Number (thousand)	Percent change	Rate per 100,000	Percent change	Sex ratio
**Location**		2019	From 1990 to 2019	2019	From 1990 to 2019	2019
Cause	Measure	Mean (95% UI)	Mean (95% UI)	Mean (95% UI)	Mean (95% UI)	F:M
	YLDs	10 (7·2–13·7)	113·9% (89·8 to 141·7%)	11·2 (8–15·3)	16·5% (5 to 30·4%)	0·63
Moderate/Severe TBI	Incidence	121·7 (106·8–137·9)	80·3% (66·7 to 93·9%)	144·5 (127–163·6)	19·9% (12·1 to 27·7%)	0·70
	Prevalence	344·7 (325·6–366·9)	128·6% (120·2 to 138·6%)	377·8 (357·6–401·5)	20·3% (16 to 25·1%)	0·63
	YLDs	52·8 (38–71·2)	124·6% (110·2 to 140·4%)	57·9 (41·6–78)	19·4% (12·4 to 27·3%)	0·62
Spinal cord lesion at neck level	Incidence	2·4 (1·8–3·1)	45·4% (3·5 to 87·6%)	2·9 (2·1–3·8)	1·3% (-25 to 26·1%)	0·79
	Prevalence	77·2 (61·9–109·1)	191·5% (147·8 to 272·8%)	83·9 (67·3–118·5)	64·9% (38·9 to 114·4%)	0·72
	YLDs	29·3 (19·2–43·2)	158·9% (104·8 to 244·2%)	31·9 (20·9–47·3)	47·9% (17·7 to 99·9%)	0·70
Spinal cord lesion below neck level	Incidence	2 (1·5–2·6)	85% (61·4 to 115·1%)	2·4 (1·8–3·1)	25·7% (10·9 to 43·3%)	0·89
	Prevalence	62 (56·1–72·3)	139% (120·7 to 170·1%)	67·5 (61·1–78·6)	31·1% (21·3 to 46·9%)	0·81
	YLDs	10·8 (7·3–15·2)	56·6% (28·6 to 89·6%)	11·8 (8–16·5)	-13·3% (-28·3 to 5%)	0·79
Head Injuries	Incidence	219·3 (191·6–255)	55% (43·4 to 66·5%)	265·1 (231·6–308)	12·5% (5·2 to 20%)	0·66
	Prevalence	428·1 (407·3–451·7)	125·8% (118·6 to 134·6%)	471·1 (448·7–496·4)	19·6% (16·1 to 24·1%)	0·63
	YLDs	62·8 (45·2–85)	122·8% (109·8 to 137·1%)	69·1 (49·6–93·2)	18·9% (12·6 to 26·1%)	0·62
Spinal Injuries	Incidence	4·4 (3·6–5·4)	61·1% (27·9 to 89·6%)	5·3 (4·3–6·5)	11·1% (-9 to 28·1%)	0·84
	Prevalence	139·2 (121·5–172·1)	165·6% (139·5 to 213·1%)	151·4 (132·4–187·8)	47·9% (32·9 to 75·7%)	0·76
	YLDs	40·2 (27·3–56·8)	120·1% (83·7 to 175·5%)	43·7 (29·7–61·9)	24·2% (3·7 to 55·5%)	0·73
**United Arab Emirates**						
Minor TBI	Incidence	14·6 (11·2–18·7)	361·1% (328·6 to 396·4%)	145·1 (114·4–184)	-6·6% (-10·9 to -1·9%)	0·60
	Prevalence	11·5 (10·6–12·4)	573·8% (546·6 to 605·7%)	113·4 (105·8–122·1)	-5·4% (-8·2 to -2·5%)	0·64
	YLDs	1·4 (0·9–1·9)	573·3% (479·2 to 684·7%)	13·5 (9·2–18·4)	-5·9% (-14·6 to 4·2%)	0·63
Moderate/Severe TBI	Incidence	18·6 (16–21·6)	450·9% (410·4 to 495·7%)	184·6 (162·9–208·5)	-8·4% (-12·1 to -4·7%)	0·62
	Prevalence	54·8 (51·3–58·9)	616·8% (587·1 to 640·8%)	480·1 (455·6–509·7)	-4·1% (-6·8 to -2%)	0·61
	YLDs	8·6 (6–11·8)	609% (552·6 to 668·7%)	73·8 (51·8–100·8)	-4·7% (-9·8 to 0·7%)	0·59
Spinal cord lesion at neck level	Incidence	0·3 (0·2–0·4)	456·4% (419·8 to 497%)	3·1 (2·3–4·1)	-3·7% (-9·6 to 2·1%)	0·67
	Prevalence	9·1 (8·4–9·8)	582·7% (532 to 620·7%)	70·6 (66–75·8)	-1·9% (-7·3 to 2·6%)	0·71
	YLDs	3·7 (2·6–4·8)	541·4% (419·2 to 694%)	28·2 (20–36·2)	-7·3% (-20·9 to 8·8%)	0·70
Spinal cord lesion below neck level	Incidence	0·3 (0·2–0·3)	443·4% (407·2 to 485·6%)	2·6 (1·9–3·5)	-3·2% (-9·1 to 2·6%)	0·78
	Prevalence	8·2 (7·6–8·8)	606·3% (573·7 to 636·9%)	68·1 (63·8–72·6)	-0·6% (-4·2 to 3·1%)	0·80
	YLDs	1·8 (1·2–2·5)	482·4% (373·3 to 611%)	14·7 (10·3–19·6)	-17·5% (-29·3 to -5·1%)	0·78
Head Injuries	Incidence	33·2 (28·5–38·6)	407·5% (374·1 to 445·5%)	329·7 (286·2–378·1)	-7·6% (-11·2 to -3·8%)	0·61
	Prevalence	66·3 (62·6–70·3)	609% (583·8 to 628·9%)	593·5 (566·6–623·9)	-4·4% (-6·7 to -2·6%)	0·61
	YLDs	10 (7–13·7)	603·8% (555·7 to 656·9%)	87·3 (60·9–119·3)	-4·9% (-9·5 to 0%)	0·60
Spinal Injuries	Incidence	0·6 (0·5–0·7)	450·4% (416·7 to 487·7%)	5·6 (4·6–7)	-3·5% (-8·2 to 1·3%)	0·72
	Prevalence	17·3 (16·1–18·6)	593·7% (558·7 to 620·8%)	138·6 (130·6–148·2)	-1·2% (-5·1 to 1·8%)	0·75
	YLDs	5·5 (3·8–7·1)	520·7% (428·5 to 622·5%)	42·9 (30·9–54·8)	-11·1% (-21·3 to -1·1%)	0·73
**Yemen**						
Minor TBI	Incidence	114 (60·4–238·1)	588·9% (261·5 to 1352%)	320·2 (173·7–647·9)	178·2% (50·4 to 483·7%)	0·51
	Prevalence	34·6 (23·9–56·3)	309% (215·5 to 466%)	144·6 (103·2–223·6)	48·1% (14·5 to 102·6%)	0·57
	YLDs	4·1 (2·5–7·1)	301·3% (202·1 to 461·8%)	17 (10·6–27·5)	45·5% (10·5 to 98·8%)	0·56
Moderate/Severe TBI	Incidence	50·9 (39·1–74·7)	238·1% (167·8 to 390·1%)	175·5 (138·7–249·2)	29·8% (5·1 to 82·7%)	0·48
	Prevalence	83·1 (73·7–102·1)	195·9% (175·2 to 236·2%)	379·9 (337·2–466·3)	10·6% (2·2 to 29·3%)	0·52
	YLDs	13 (9·1–17·6)	195·8% (166·6 to 240·6%)	58·2 (41·4–78·6)	10·3% (-0·1 to 29·2%)	0·51
Spinal cord lesion at neck level	Incidence	8·2 (2·4–23·5)	3053·8% (811·8 to 8943%)	24 (7·4–68·4)	959·8% (223·3 to 3055·5%)	0·55
	Prevalence	56·2 (22·1–148·1)	404·1% (276·1 to 506·9%)	207·3 (84·5–545·3)	114% (45·9 to 178·7%)	0·66
	YLDs	24·2 (9·2–62·7)	379·2% (246·7 to 490·5%)	87·2 (33·8–224·9)	101·8% (38·9 to 162·4%)	0·64
Spinal cord lesion below neck level	Incidence	0·6 (0·4–0·8)	143·8% (131·1 to 156·5%)	2 (1·5–2·6)	0·1% (-4·8 to 5%)	0·71
	Prevalence	12·5 (11·6–13·3)	166% (145·8 to 180·6%)	52·7 (49·1–56·5)	0·4% (-6·1 to 5%)	0·72
	YLDs	3·3 (2·3–4·3)	128·1% (92·4 to 171·5%)	13·7 (9·5–17·9)	-13·8% (-25·9 to 0%)	0·70
Head Injuries	Incidence	164·9 (108·3–287·7)	421·8% (240·8 to 822·5%)	495·8 (338·5–840·9)	98·1% (35·1 to 236·3%)	0·50

Table S14 Incidence, prevalence, and disability of head and spinal injuries in the world and in North Africa and Middle East countries

		All Ages		Age-standardised		
		Number (thousand)	Percent change	Rate per 100,000	Percent change	Sex ratio
**Location**		2019	From 1990 to 2019	2019	From 1990 to 2019	2019
Cause	Measure	Mean (95% UI)	Mean (95% UI)	Mean (95% UI)	Mean (95% UI)	F:M
	Prevalence	117·7 (101·3–141·7)	222·1% (190·6 to 269·5%)	524·6 (456·2–626·3)	18·9% (7·1 to 35·6%)	0·53
	YLDs	17·1 (12·1–23·4)	215·7% (182·1 to 263·2%)	75·2 (54·3–101·2)	16·7% (4·8 to 33·9%)	0·52
Spinal Injuries	Incidence	8·8 (2·9–24·1)	1671·3% (510 to 4727·5%)	26 (9·3–70·3)	510·5% (122·1 to 1565%)	0·56
	Prevalence	68·6 (34·5–160·6)	333·7% (228·3 to 445·6%)	260 (136·8–594·8)	74·1% (25 to 134·1%)	0·67
	YLDs	27·5 (12·2–66·4)	323·5% (208·3 to 439·2%)	100·9 (45·9–237·9)	70·7% (18·8 to 131·1%)	0·65
**Afghanistan**						
Minor TBI	Incidence	117·7 (64·7–236·6)	447·1% (322 to 601·1%)	361·4 (178·7–782·8)	107·2% (40·1 to 196·8%)	0·82
	Prevalence	70·2 (34·8–142·5)	116·1% (35·2 to 291·7%)	301 (139·5–639·5)	-13·7% (-39·2 to 43·5%)	0·56
	YLDs	8·3 (4–16·3)	115·1% (33·3 to 288·1%)	34·8 (16·1–71·7)	-14·9% (-41 to 39·6%)	0·56
Moderate/Severe TBI	Incidence	55 (40·9–86·1)	317·1% (246·2 to 444·4%)	210·5 (135·1–378·6)	64·6% (16·1 to 154·3%)	0·53
	Prevalence	112·4 (77·4–202·8)	109·3% (33·3 to 229%)	473·6 (316·7–887·3)	-20·4% (-45·2 to 15·6%)	0·40
	YLDs	17·2 (10·8–29)	114% (34 to 237·5%)	70·8 (43·7–124·1)	-19·7% (-44·9 to 17·1%)	0·39
Spinal cord lesion at neck level	Incidence	9·8 (2·7–29)	831·4% (559·5 to 1047·8%)	41·7 (9·8–129·8)	369% (166 to 559·6%)	1·02
	Prevalence	214·8 (51·5–641·7)	28·9% (-23·3 to 330·8%)	755·2 (173–2296·9)	-50·1% (-66·8 to 44·9%)	0·65
	YLDs	94·5 (22·3–283·3)	25·1% (-25·6 to 315·1%)	325·6 (73·8–997·6)	-52·1% (-68·1 to 36·6%)	0·64
Spinal cord lesion below neck level	Incidence	0·6 (0·5–0·8)	245·1% (222 to 274·5%)	2 (1·6–2·5)	10·1% (1·5 to 24·5%)	0·72
	Prevalence	14·5 (12·3–18·5)	284·9% (239 to 370·4%)	54·7 (46·1–71·2)	26·8% (10·7 to 57·7%)	0·62
	YLDs	4·4 (3·1–6·2)	251·3% (173·4 to 354·2%)	16·4 (11·4–23·2)	14·3% (-8·5 to 49·6%)	0·61
Head Injuries	Incidence	172·7 (114·3–298·5)	397·7% (305·3 to 512·9%)	571·9 (346·5–1015·1)	89·2% (38·3 to 157·5%)	0·70
	Prevalence	182·5 (119·6–305·6)	111·9% (34·5 to 251%)	774·5 (486·3–1367)	-17·9% (-42·8 to 24·8%)	0·46
	YLDs	25·4 (16·5–40·5)	114·3% (34·3 to 250·9%)	105·6 (66·4–176·9)	-18·2% (-43·5 to 22·8%)	0·44
Spinal Injuries	Incidence	10·5 (3·3–29·6)	742·6% (464 to 974%)	43·7 (11·8–131·7)	308·3% (115·4 to 502·2%)	1·00
	Prevalence	229·3 (65–655·6)	34·5% (-20·8 to 328·2%)	810 (224·5–2351)	-48% (-65·9 to 41·6%)	0·65
	YLDs	98·9 (26·4–287·5)	28·8% (-24·1 to 309·3%)	342 (87·3–1016·4)	-50·7% (-67·5 to 33%)	0·64
**Sudan**						
Minor TBI	Incidence	51·6 (39·1–67·8)	6·8% (-44·6 to 69·3%)	119·6 (91·7–155·6)	-46·5% (-71·3 to -16·5%)	0·52
	Prevalence	35·2 (30·1–43·9)	108·8% (83·3 to 128%)	121 (105·1–149·1)	3·5% (-4 to 11·2%)	0·53
	YLDs	4·3 (3–5·9)	111·3% (81·6 to 143·4%)	14·5 (10·2–19·7)	4·1% (-6·5 to 16·5%)	0·52
Moderate/Severe TBI	Incidence	18·2 (15–21·9)	65·7% (20·5 to 95·9%)	52·4 (43·3–62·4)	-19·7% (-40 to -6·5%)	0·53
	Prevalence	41·7 (38·1–47·6)	127·1% (119·2 to 136·4%)	147·5 (135·4–166·9)	6·5% (2·7 to 11·7%)	0·49
	YLDs	6·5 (4·6–8·8)	125·3% (103·1 to 148·6%)	22·5 (16·1–30·6)	5·5% (-3·4 to 15%)	0·48
Spinal cord lesion at neck level	Incidence	1·7 (1·3–2·4)	-26·1% (-69·7 to 60·7%)	4·8 (3·4–6·6)	-60·7% (-83·9 to -18·7%)	0·66
	Prevalence	54·8 (36·6–101·9)	198·7% (166·9 to 234·5%)	164·5 (112·8–296·7)	45·4% (26·3 to 70·1%)	0·70
	YLDs	23·2 (13·8–46·6)	180·4% (136·6 to 222·8%)	68·8 (41·8–132·9)	36·7% (12·7 to 61·4%)	0·69
Spinal cord lesion below neck level	Incidence	1·6 (1·1–2·2)	127·2% (112·6 to 144·4%)	4·3 (3·1–6·1)	9·1% (2·2 to 16·9%)	0·73
	Prevalence	33·9 (31·3–36·9)	147·5% (134 to 163·4%)	109·4 (101·4–118·1)	14·9% (9·2 to 21·4%)	0·70
	YLDs	8·7 (6·1–11·6)	108·5% (81·2 to 140·4%)	27·7 (19·5–36·5)	-3·4% (-14·8 to 9%)	0·69
Head Injuries	Incidence	69·7 (56·1–87·5)	17·7% (-31·8 to 69·8%)	171·9 (140·9–212·8)	-40·5% (-64·4 to -16·4%)	0·52
	Prevalence	76·8 (69·6–87·3)	118·3% (102·2 to 130·2%)	268·5 (244·4–303·9)	5·2% (0·6 to 10·1%)	0·51
	YLDs	10·7 (7·8–14·4)	119·5% (99·9 to 139·2%)	37 (27–49·6)	4·9% (-1·9 to 12·7%)	0·49
Spinal Injuries	Incidence	3·3 (2·6–4·2)	8·9% (-48·2 to 87·2%)	9·1 (7·2–11·5)	-43·6% (-72·1 to -7·9%)	0·69
	Prevalence	88·8 (69·3–135·8)	176·8% (154·2 to 208·3%)	274 (218·1–405·4)	31·5% (19·6 to 50·5%)	0·70
	YLDs	31·9 (20·4–55·4)	156·3% (122·8 to 192·8%)	96·5 (63·1–164·2)	22·2% (5·5 to 42·6%)	0·69

F:M, Female to male ratio; TBI, traumatic brain injury; UI uncertainty interval; YLDs, Years Lived with Disability. Data in parentheses are 95% uncertainty intervals. Count data in thousands and percentage data are rounded to one decimal place, and sex ratio is rounded to two decimal place. Percentages and number of YLDs, incident cases, and prevalent cases are not mutually exclusive: the sum of percentages and number of YLDs in the columns exceeds the totals for all injuries combined because of overlap between various causes. Measures with insufficient data are not reported here.

Table S15 Burden of neurological conditions attributable to risk factors in North Africa and Middle East countries
(NEXT PAGE)

All risk factors (p 277), Behavioural risk factors (p 282), Environmental/occupational risk factors (p 287), Metabolic risk factors (p 290), Air pollution (p 293), Alcohol use (p 296), Child and maternal malnutrition (p 299), Dietary risks (p 300), High body-mass index (p 302), High fasting plasma glucose (p 304), High LDL cholesterol (p 307), High systolic blood pressure (p 308), Kidney dysfunction (p 310), Low physical activity (p 312), Non-optimal temperature (p 312), Other environmental risks (p 314), Tobacco (p 316)

Table S15 Burden of neurological conditions attributable to risk factors in North Africa and Middle East countries

			DALYs (95% UI)			
			PAF (%)	PAF change (%)	Rate per 100,000	Rate change (%)
Risk factors	Causes	Locations	2019	From 1990 to 2019	2019	From 1990 to 2019
**Combined risk factors**						
All risk factors	Meningitis	Global	6·8% (6·1–7·7%)	12% (-2·8 to 28·5%)	15·92 (12·67–20·6)	-52% (-61·5 to -39·2%)
		North Africa and Middle East	7·1% (5·4–9·4%)	-11·4% (-36·7 to 21·3%)	5 (3·51–6·99)	-78·5% (-85·6 to -67·8%)
		Algeria	4·3% (2·6–6·6%)	-9·5% (-53·1 to 79%)	1·72 (0·96–2·86)	-81·8% (-91 to -62·4%)
		Bahrain	1·8% (1·2–2·5%)	-54·2% (-73·9 to -21·6%)	0·33 (0·22–0·48)	-88·3% (-93·6 to -79·2%)
		Egypt	9·5% (4·7–16·6%)	-34·4% (-69·8 to 28·7%)	5·18 (2·15–9·91)	-83·9% (-93·8 to -65·2%)
		Iran	4·4% (3·3–5·5%)	-34·4% (-52·4 to -12·2%)	1·72 (1·22–2·28)	-86·4% (-91·5 to -79·4%)
		Iraq	8·6% (5·5–12·4%)	-17·6% (-56·2 to 48%)	5·8 (3·47–9·21)	-83·5% (-91·6 to -67·4%)
		Jordan	5·9% (3·5–9·7%)	-12·1% (-55·5 to 86·6%)	3·11 (1·73–5·62)	-61·7% (-83 to -11·6%)
		Kuwait	6·2% (3·9–8·6%)	2% (-33·2 to 49·4%)	1·25 (0·68–1·87)	-62·2% (-78·1 to -40·6%)
		Lebanon	3·3% (1·7–5·5%)	-54·1% (-79·4 to -5·6%)	0·96 (0·45–1·72)	-85·5% (-93·9 to -69%)
		Libya	2·4% (1·5–4·1%)	-66·7% (-82·9 to -35·1%)	0·88 (0·5–1·53)	-89·9% (-95 to -80·2%)
		Morocco	10·2% (5·4–17·2%)	-27·3% (-66·4 to 54%)	7 (3·26–13·01)	-86·8% (-94·7 to -68%)
		Palestine	4% (2·6–5·9%)	-29·8% (-62·8 to 30·5%)	1·64 (1–2·5)	-87·7% (-93·8 to -75·9%)
		Oman	4·2% (2·4–7·4%)	-36·2% (-66·6 to 24·9%)	1·04 (0·54–1·94)	-83·3% (-91·7 to -64·3%)
		Qatar	3·4% (2·2–5·2%)	-44% (-69·7 to 0·6%)	0·86 (0·55–1·33)	-84·9% (-92·6 to -71·1%)
		Saudi Arabia	0·5% (0·3–0·8%)	-64·4% (-85 to -25·8%)	0·12 (0·07–0·2)	-90·5% (-95·6 to -80·6%)
		Syrian Arab Republic	2·7% (1·5–4·4%)	-53·2% (-78·4 to 1·8%)	2·64 (1·4–4·55)	-86·3% (-93·7 to -69·1%)
		Tunisia	6·2% (3·7–9·8%)	-7·1% (-52·2 to 86·5%)	2·34 (1·29–3·88)	-78·7% (-89·9 to -55·9%)
		Türkiye	2% (1·3–3·2%)	-37·1% (-66·4 to 19·4%)	0·52 (0·32–0·87)	-93·6% (-96·9 to -86·5%)
		United Arab Emirates	1·4% (0·7–3·5%)	-72·4% (-87 to -45·4%)	0·45 (0·22–1·12)	-92·9% (-96·5 to -85·7%)
		Yemen	5·3% (3–8·5%)	-12·1% (-56·5 to 72·7%)	5·1 (2·78–8·83)	-72·4% (-86·7 to -44·7%)
		Afghanistan	6·2% (3·1–10·9%)	-8·8% (-57·3 to 105·5%)	14·96 (7·35–26·95)	-75·9% (-89·3 to -45%)
		Sudan	5·3% (3–8·9%)	-12·3% (-52·5 to 60·8%)	5·32 (2·78–9·71)	-86·8% (-93·3 to -76·2%)
	Encephalitis	Global	2·5% (1·9–2·9%)	42·9% (2·2 to 91·2%)	1·61 (1·24–2·06)	-34·6% (-56 to 1·4%)
		North Africa and Middle East	3·8% (2·2–6·1%)	7·7% (-32·2 to 72·6%)	1·59 (0·94–2·57)	-15·4% (-52·1 to 49·5%)
		Algeria	2·5% (1·2–4·5%)	22·7% (-45·9 to 179·7%)	0·47 (0·24–0·81)	-12·7% (-64·2 to 112·4%)
		Bahrain	1% (0·6–1·6%)	-33·6% (-66·9 to 30·6%)	0·12 (0·07–0·2)	-50·5% (-77·4 to 0·3%)
		Egypt	6·8% (2·2–13·8%)	3·6% (-55·5 to 123·6%)	4·44 (0·8–10·08)	-29·6% (-73 to 62·2%)
		Iran	3·7% (1·5–5%)	6·9% (-25·9 to 42·1%)	0·61 (0·21–0·87)	-15·8% (-53·7 to 28·2%)
		Iraq	3·4% (1·6–5·9%)	9·1% (-48·9 to 137·9%)	2·43 (0·93–4·56)	-27·5% (-69 to 73·9%)
		Jordan	1·5% (0·8–2·6%)	-32·7% (-72·8 to 35·8%)	0·17 (0·08–0·29)	-73·7% (-90·9 to -5·6%)
		Kuwait	2·3% (1·6–3·1%)	19·4% (-20·8 to 90·1%)	0·24 (0·15–0·34)	-12·2% (-45·8 to 46·1%)
		Lebanon	2·6% (1·3–5%)	-4·8% (-61·1 to 140·3%)	0·45 (0·19–0·87)	-22·1% (-70·8 to 97·2%)
		Libya	2·5% (1·2–4·7%)	-21% (-68·8 to 87%)	0·42 (0·22–0·74)	-28·7% (-74·1 to 73%)
		Morocco	5·7% (2–10·9%)	-2·7% (-52·9 to 106·4%)	1·35 (0·64–2·54)	-19·3% (-65·8 to 82·2%)
		Palestine	2% (1·1–3·3%)	-10·6% (-59 to 100·5%)	0·32 (0·18–0·54)	-29·7% (-70·3 to 56·6%)
		Oman	2% (1–3·7%)	0·5% (-56·9 to 150·7%)	2·45 (1·16–4·45)	-43·8% (-76·9 to 36·5%)
		Qatar	1·6% (0·8–2·5%)	-17·5% (-62·8 to 86·6%)	0·21 (0·11–0·38)	-41·6% (-76·8 to 31·2%)
		Saudi Arabia	0·5% (0·1–1·8%)	-1·3% (-72·5 to 123·4%)	0·15 (0·04–0·6)	-49·8% (-87·4 to 7%)
		Syrian Arab Republic	1·5% (0·9–2·2%)	-24·5% (-61·1 to 37·5%)	0·41 (0·24–0·7)	-55·2% (-79·4 to -8·7%)
		Tunisia	2·8% (1·3–5·3%)	3·3% (-55·6 to 156·6%)	0·48 (0·25–0·86)	-27·1% (-73·1 to 80·6%)
		Türkiye	0·9% (0·4–2%)	-20·5% (-66·5 to 65·3%)	0·17 (0·08–0·36)	-58·2% (-84·1 to -4·5%)
		United Arab Emirates	2·2% (1–4·1%)	-18·5% (-67·4 to 98·4%)	0·38 (0·17–0·71)	-35·1% (-75·7 to 68·2%)
		Yemen	2·3% (1·1–4·7%)	3·4% (-56·1 to 163·3%)	0·69 (0·35–1·29)	-3·7% (-58·3 to 134·1%)
		Afghanistan	1·2% (0·5–2·4%)	5·3% (-57·1 to 151·9%)	2·17 (0·88–5·44)	-10·7% (-63·1 to 115·8%)
		Sudan	2·2% (1·1–4·1%)	0·8% (-58·1 to 164·8%)	0·66 (0·35–1·12)	-22·8% (-67·6 to 98%)
	Stroke	Global	86·4% (83·4–89·2%)	1·2% (-0·01 to 2·5%)	1526·72 (1408·37–1642·57)	-34·5% (-40·2 to -29·2%)
		North Africa and Middle East	85·8% (82·6–89·1%)	9·1% (4·8 to 14·4%)	1566·95 (1393·24–1757·07)	-25·8% (-34·1 to -14%)

Table S15 Burden of neurological conditions attributable to risk factors in North Africa and Middle East countries

Risk factors	Causes	Locations	DALYs (95% UI) PAF (%) 2019	PAF change (%) From 1990 to 2019	Rate per 100,000 2019	Rate change (%) From 1990 to 2019
		Algeria	85·7% (81·2–90·5%)	3·4% (0 to 7·3%)	1503·92 (1242·73–1802·34)	-42·7% (-54·3 to -29·1%)
		Bahrain	86·9% (82·9–90·8%)	0·6% (-1·9 to 3·1%)	818·08 (689·8–989·92)	-52·2% (-61 to -41·9%)
		Egypt	82% (76·6–87%)	40·3% (18·1 to 58%)	1755 (1315·04–2369·7)	-9·2% (-30·9 to 17·3%)
		Iran	84·9% (80·8–89·7%)	3·3% (1·5 to 5·9%)	1072·11 (972·56–1165·18)	-43·9% (-49·4 to -35·4%)
		Iraq	90·3% (87·5–93%)	3·3% (1·1 to 5·5%)	2640·29 (2134·66–3137·47)	-16·7% (-33·9 to 3·1%)
		Jordan	87·8% (83·9–92%)	1·1% (-1 to 3·6%)	1271·73 (1062·95–1477·14)	-49·7% (-58·3 to -40·2%)
		Kuwait	88·6% (85·4–91·7%)	1·3% (-0·4 to 3·1%)	831·04 (699·98–978·99)	-10·5% (-23·2 to 4·5%)
		Lebanon	87·8% (83·7–92·1%)	5·8% (2·9 to 9·1%)	660·81 (511·05–818·29)	-29·8% (-45·1 to -11·7%)
		Libya	86·3% (82·7–89·9%)	13·8% (5·2 to 24%)	1355·5 (1061·21–1716·92)	-10·5% (-29·5 to 16·2%)
		Morocco	88·4% (84·6–92·2%)	3·8% (0·8 to 7·2%)	1995·78 (1606·38–2406·61)	-14·1% (-32 to 6·4%)
		Palestine	85·8% (81·3–91%)	1·7% (-1·1 to 5%)	1825·78 (1582·81–2103·26)	-31·4% (-45·6 to -13·5%)
		Oman	85·4% (81·1–90·1%)	9·1% (4·5 to 14·7%)	1610·32 (1402·57–1845·1)	-33·1% (-48 to -12·1%)
		Qatar	86·5% (82·3–90·5%)	0·7% (-1·9 to 3·6%)	782·5 (640·08–988·22)	-44·3% (-57·4 to -28·6%)
		Saudi Arabia	89·6% (86·4–92·5%)	4·9% (2·6 to 7·5%)	1894·49 (1500·86–2229·97)	-25·6% (-44 to -0·7%)
		Syrian Arab Republic	84·6% (80·9–88·2%)	9·6% (4·1 to 15·5%)	1708·46 (1346·03–2186·72)	-34·5% (-51·5 to -11%)
		Tunisia	85·5% (80·9–90·5%)	4·9% (1·5 to 8·5%)	1263·03 (963·03–1624·7)	-21% (-41·6 to 5·3%)
		Türkiye	85·2% (81·4–89·1%)	2·6% (-0·2 to 6%)	990·97 (816·26–1187·07)	-21·6% (-42·2 to -2·1%)
		United Arab Emirates	89·3% (85·6–92·7%)	0·9% (-1·1 to 3%)	1719·55 (1350·25–2192·67)	-45·5% (-58·5 to -29·9%)
		Yemen	86·4% (83·1–89·9%)	2·0% (-2·0 to 6·9%)	2391·66 (1853·83–3075·25)	-23·1% (-40·5 to 1·9%)
		Afghanistan	89·6% (86·8–92·3%)	2·3% (-0·4 to 5·1%)	3136·6 (2253·15–4043·73)	-19·9% (-38·2 to -0·9%)
		Sudan	89·3% (86·2–92·2%)	5·7% (1·4 to 13·8%)	2309·29 (1736·58–3195·82)	-29·3% (-43·2 to -11·8%)
	Ischaemic stroke	Global	85% (80·2–89·9%)	0·0% (-1·2 to 1·4%)	679·04 (611·57–747·17)	-28·5% (-34·9 to -22·9%)
		North Africa and Middle East	87·1% (82·8–92%)	5% (2·8 to 8·2%)	1030·95 (912·49–1161·56)	-4·2% (-16·2 to 9%)
		Algeria	86% (80·3–92·4%)	3% (-0·1 to 6·8%)	1096·29 (899·86–1331·46)	-22·4% (-37·7 to -4·6%)
		Bahrain	87% (81·6–92·8%)	-0·6% (-3·1 to 1·9%)	533·72 (449·21–646·33)	-44·2% (-53·9 to -33·1%)
		Egypt	86·4% (81·7–90·8%)	20·6% (10·7 to 33·3%)	1194·45 (912·56–1589·29)	37·5% (1·1 to 75·1%)
		Iran	85·6% (80·7–91·4%)	2·5% (0·8 to 5·3%)	842·34 (753·98–927·77)	-41·4% (-46·9 to -32·8%)
		Iraq	90·5% (86·7–94·5%)	2·1% (0·2 to 4·2%)	1656·27 (1358·41–1936·06)	-5% (-24·2 to 16·5%)
		Jordan	87·7% (82·9–93%)	1% (-1·1 to 3·6%)	950·57 (792·13–1107·09)	-45·8% (-55·8 to -35·6%)
		Kuwait	88·9% (84·7–93·3%)	0·2% (-1·5 to 1·9%)	574·01 (476·11–674·09)	-11·8% (-24·1 to 2·7%)
		Lebanon	87·9% (83·2–93·6%)	5·3% (2·3 to 8·8%)	525·76 (403·23–644·29)	-10·6% (-31·2 to 10·5%)
		Libya	88·1% (83·9–92·8%)	6·7% (2·5 to 12·3%)	989·04 (770·36–1273·62)	18·6% (-7·2 to 51·6%)
		Morocco	88·8% (84–93·8%)	3·2% (0·6 to 6·5%)	1424·11 (1149·72–1720·84)	22·2% (-4·4 to 48·1%)
		Palestine	85·9% (80·3–92·7%)	1·2% (-1·5 to 4·4%)	1366·28 (1171·78–1577·86)	-16·8% (-33·6 to 4·7%)

Table S15 Burden of neurological conditions attributable to risk factors in North Africa and Middle East countries

			DALYs (95% UI)			
			PAF (%)	PAF change (%)	Rate per 100,000	Rate change (%)
Risk factors	Causes	Locations	2019	From 1990 to 2019	2019	From 1990 to 2019
		Oman	86·8% (81·3–92·9%)	6·6% (2·7 to 11·1%)	1120·35 (948·02–1304·68)	-13% (-31·3 to 15·5%)
		Qatar	86·4% (80·5–92·4%)	-0·7% (-3·3 to 1·9%)	498·82 (405·5–624·4)	-35·9% (-50·1 to -20%)
		Saudi Arabia	89% (84·9–93·3%)	5% (2·2 to 8%)	1158·37 (900·61–1354·19)	-13·3% (-33·9 to 12·6%)
		Syrian Arab Republic	86·9% (81·6–92·9%)	5% (1·3 to 10·1%)	929·55 (736·23–1168·72)	-21·4% (-42·4 to 4%)
		Tunisia	85·3% (79·6–92·1%)	3·9% (0·9 to 7·3%)	931 (709·53–1185·55)	8·1% (-19·9 to 40·9%)
		Türkiye	84·7% (79·3–90·7%)	0·6% (-2 to 3·7%)	561·71 (464·88–674·12)	-7·5% (-33·4 to 15·5%)
		United Arab Emirates	89·3% (84·8–93·6%)	0·3% (-1·6 to 2·2%)	1263·3 (996·8–1560·33)	-33·7% (-48·9 to -15·3%)
		Yemen	87% (82·8–91·6%)	1·9% (-1·8 to 5·9%)	1493·65 (1176·34–1906·38)	22% (-3·7 to 56%)
		Afghanistan	89·7% (85·8–94%)	2·2% (-0·7 to 5·2%)	1673·01 (1224·87–2198·78)	30·2% (1 to 63·5%)
		Sudan	90·4% (86·6–94·3%)	4·7% (1·1 to 10·3%)	1530·35 (1174·12–2132·97)	13·1% (-9 to 40·6%)
	Intracerebral haemorrhage	Global	88·1% (85·1–90·5%)	2% (0·6 to 3·7%)	733·56 (675·79–792)	-35·4% (-42·5 to -29·1%)
		North Africa and Middle East	84·5% (81·1–87·7%)	8·9% (3·6 to 18·4%)	463·33 (401·03–535·18)	-47·4% (-55·4 to -35·9%)
		Algeria	85·3% (81·1–89%)	2·7% (-0·8 to 7·4%)	344·43 (270·1–433·66)	-67·1% (-75·1 to -56·3%)
		Bahrain	88·3% (84·1–91·7%)	-0·1% (-2·6 to 2·3%)	233·76 (190·52–290·21)	-64·2% (-72·1 to -54·1%)
		Egypt	76·3% (68·7–83·9%)	34·3% (13·8 to 63·6%)	491·4 (342·95–709·93)	-45·5% (-61·8 to -23·7%)
		Iran	83·1% (79·4–86·6%)	5·5% (2·5 to 10·7%)	185·43 (172·49–198·35)	-51·1% (-58·3 to -42·4%)
		Iraq	90·1% (87·1–92·6%)	4% (1·1 to 7·7%)	921·56 (722·77–1159·29)	-27·2% (-46 to -4·1%)
		Jordan	88·2% (84·5–91·5%)	1·3% (-1 to 4%)	287·4 (236·39–341·93)	-59·5% (-67·5 to -49·8%)
		Kuwait	88·3% (84·9–91%)	2·1% (0·1 to 4·3%)	217·18 (176·93–260·49)	-2·2% (-22·3 to 21·2%)
		Lebanon	87·2% (83·3–90·7%)	5·1% (1·8 to 8·8%)	112·05 (83·72–151·37)	-62·2% (-72·6 to -48·1%)
		Libya	83·5% (79·1–88%)	11·8% (2·4 to 27·1%)	314·5 (234·5–432·18)	-45·5% (-60 to -24·8%)
		Morocco	87·9% (83·9–91·4%)	2·8% (-0·9 to 7·5%)	487·1 (373·93–621·39)	-49·7% (-63·3 to -30·8%)
		Palestine	85·8% (81·4–89·8%)	2·5% (-0·8 to 6·2%)	418·18 (354·12–493·39)	-55·6% (-66·1 to -41·5%)
		Oman	84·3% (80–88·8%)	8·1% (3·2 to 14·7%)	425·07 (364–534·71)	-56·6% (-67·6 to -40·3%)
		Qatar	86·8% (82·5–90·6%)	1·7% (-1·3 to 5%)	211·59 (166·85–276·7)	-55·6% (-67·6 to -40·5%)
		Saudi Arabia	90·5% (87·4–93·2%)	4·8% (2·6 to 7·6%)	692·58 (537·92–846·19)	-38·5% (-55·9 to -9·9%)
		Syrian Arab Republic	85·8% (81·8–89·4%)	6·9% (2·1 to 13·1%)	719·83 (547·65–944·57)	-44·9% (-60·4 to -21·9%)
		Tunisia	86·3% (82–90·1%)	5·3% (1·5 to 10·7%)	278·37 (204·86–370·4)	-54·2% (-68·3 to -33·4%)
		Türkiye	86·1% (82–89·9%)	4·6% (0·6 to 10·2%)	343·83 (276·19–421·9)	-34·1% (-53·1 to -14·9%)
		United Arab Emirates	89·8% (86·5–92·8%)	0·5% (-1·7 to 3·2%)	404·56 (285·22–600·65)	-63·9% (-75·5 to -48·2%)
		Yemen	86·1% (82·2–89·3%)	1·1% (-2·8 to 7·3%)	762·31 (558·65–1017·91)	-53·2% (-65·8 to -35·6%)
		Afghanistan	90% (86·9–92·6%)	2·3% (-0·2 to 5%)	1257·48 (885·08–1689·78)	-44·8% (-58·8 to -27·1%)
		Sudan	87·8% (83·9–91%)	4% (-1 to 15·3%)	676·53 (461·03–950·98)	-59·1% (-69·5 to -44·9%)
	Subarachnoid haemorrhage	Global	83·6% (80·3–86·7%)	1·9% (-0·1 to 4·3%)	114·12 (99·76–130·1)	-53·3% (-60 to -36·1%)
		North Africa and Middle East	77·2% (72·5–81·8%)	26·4% (10·7 to 56·8%)	72·67 (60·45–89·48)	-53·1% (-66·3 to -27·2%)
		Algeria	82·2% (77·6–86·3%)	6·9% (0·5 to 16·5%)	63·2 (46·87–83·9)	-62·2% (-73·8 to -41%)
		Bahrain	80·6% (76–85·1%)	17·5% (6·1 to 31·9%)	50·6 (38·49–71·74)	-50·7% (-66·3 to -29·3%)
		Egypt	60·4% (48·4–74·1%)	84·3% (30·8 to 209·8%)	69·16 (46·94–98·93)	-57·6% (-74·8 to -23·6%)
		Iran	81·2% (77·2–85·1%)	4·4% (0·9 to 8·6%)	44·34 (38·81–51·33)	-53·8% (-67·2 to -29·1%)
		Iraq	86·6% (82·7–89·9%)	7·4% (1·6 to 16%)	62·46 (46·4–85·76)	-61·4% (-74·9 to -37·3%)
		Jordan	86·8% (82·5–90·1%)	2% (-0·6 to 5·1%)	33·76 (26·16–41·24)	-48·8% (-63·1 to -26%)
		Kuwait	84·8% (81·4–87·8%)	9·7% (4·8 to 16%)	39·85 (32·59–48·11)	-28·6% (-43·1 to -4%)
		Lebanon	86·1% (82–89·8%)	11·2% (6 to 18%)	23 (16·1–31·94)	-60·3% (-76·6 to -35·5%)
		Libya	72·5% (63·5–81·3%)	48·5% (12·4 to 104·4%)	51·96 (36·84–75·47)	-50·1% (-65·4 to -22·6%)

Table S15 Burden of neurological conditions attributable to risk factors in North Africa and Middle East countries

Risk factors	Causes	Locations	DALYs (95% UI) PAF (%) 2019	PAF change (%) From 1990 to 2019	Rate per 100,000 2019	Rate change (%) From 1990 to 2019
		Morocco	84·3% (79·3–88·3%)	8·3% (0·2 to 19·4%)	84·57 (59·57–123·32)	-55·4% (-71·1 to -23·1%)
		Palestine	82·5% (77·4–86·5%)	1·9% (-2·3 to 7·2%)	41·31 (34·29–49·38)	-45% (-61·1 to -20%)
		Oman	72·4% (64·4–80·9%)	21·7% (5·6 to 46·8%)	64·9 (41·4–103·21)	-54·1% (-72·2 to -13·7%)
		Qatar	86·2% (81·8–89·9%)	4·5% (-0·5 to 10·5%)	72·09 (53·58–94·56)	-52·2% (-71·5 to -22·9%)
		Saudi Arabia	88% (84·4–91%)	7·3% (2·6 to 12·9%)	43·55 (31·54–57·06)	-49% (-70·8 to -15·5%)
		Syrian Arab Republic	54·1% (47–61·6%)	44% (13·4 to 88%)	59·09 (44·57–77·53)	-50·4% (-67·7 to -20·4%)
		Tunisia	83·1% (78·3–87·1%)	12% (3·8 to 24·3%)	53·66 (37·52–73·09)	-58·3% (-74·7 to -29·1%)
		Türkiye	84·6% (80·8–88·2%)	5·1% (1 to 10·6%)	85·42 (66·21–105·65)	-36·8% (-60·3 to 10·3%)
		United Arab Emirates	84·9% (79·3–89·7%)	12·2% (3 to 23·9%)	51·69 (27·03–84·26)	-60% (-75·2 to -34·6%)
		Yemen	82·4% (78–86·2%)	4·5% (-2·9 to 14·7%)	135·7 (70·19–225·17)	-46·8% (-63·3 to -15%)
		Afghanistan	87·8% (83·8–91%)	3·4% (-1 to 9·2%)	206·12 (67·38–351·92)	-41·9% (-59 to -10·3%)
		Sudan	83·3% (76·9–87·8%)	10·2% (-2 to 31·7%)	102·41 (58·58–172·78)	-60·4% (-74·3 to -25·9%)
	Neurological disorders*	Global	9·9% (4·6–18·6%)	7·4% (-1·2 to 14·6%)	122·4 (53·39–286·69)	6% (-2·4 to 13·6%)
		North Africa and Middle East	11·2% (4·5–22·3%)	25·9% (13·4 to 41·8%)	152·62 (56·47–371·89)	20·2% (7·5 to 39·9%)
		Algeria	12·8% (5·1–25·4%)	28·5% (7·5 to 55·3%)	172·61 (63·35–427·52)	16·1% (-2·1 to 40·5%)
		Bahrain	14·8% (6·1–29·7%)	16·7% (-0·9 to 34·6%)	203·7 (76·08–504·82)	6·5% (-11·7 to 23·9%)
		Egypt	11·5% (4·4–23·7%)	38% (15·3 to 68·5%)	150·75 (55·29–362·46)	38·7% (15·7 to 70·7%)
		Iran	10·1% (4·1–20%)	38·7% (20·5 to 61·9%)	138·81 (51·81–339·57)	32% (14·6 to 63·3%)
		Iraq	12·4% (4·7–25·1%)	9·6% (-6·7 to 28·5%)	163·14 (58·46–403·56)	7·4% (-8·2 to 26·4%)
		Jordan	14% (5·8–27·8%)	13·4% (-4·5 to 36%)	176·79 (68·74–424·32)	7% (-9·1 to 30·7%)
		Kuwait	14·9% (6·1–29·3%)	20·4% (1·5 to 44·4%)	189·09 (72·1–447·66)	14% (0·5 to 33·2%)
		Lebanon	15% (6·1–29·4%)	26·2% (8·6 to 51%)	199·52 (79·55–466·17)	21·3% (3·8 to 59·8%)
		Libya	12·7% (4·9–25·1%)	18·3% (-1·4 to 42%)	173·85 (59·87–433·96)	14·4% (-5·7 to 42·5%)
		Morocco	9·8% (3·7–20·3%)	23·2% (0·9 to 46·4%)	131·42 (44·78–341·52)	25·7% (2·6 to 49·4%)
		Palestine	10·8% (4·1–21·9%)	23·4% (2 to 48·4%)	146·9 (53·91–374·07)	16·2% (-6·5 to 45·9%)
		Oman	11·6% (4·5–23·9%)	52·2% (22·9 to 102·9%)	155·21 (52·46–394·55)	53·4% (23 to 113·9%)
		Qatar	15·8% (6·2–31·4%)	38·9% (12·1 to 81·2%)	213·66 (75·8–551·92)	36·4% (7·6 to 77·8%)
		Saudi Arabia	11·8% (4·7–24%)	38·4% (14·1 to 77·5%)	167·38 (58·6–412·97)	38·4% (14·4 to 83·8%)
		Syrian Arab Republic	12·2% (4·5–24·2%)	10·8% (-7·5 to 34·9%)	159·06 (58·66–401·98)	12·5% (-10 to 45·8%)
		Tunisia	13·1% (5·4–25·1%)	19·7% (2 to 43·6%)	171·43 (64·49–400·5)	16·4% (-6·5 to 49·7%)
		Türkiye	11·5% (4·9–21·8%)	19·9% (1·8 to 44·3%)	168·09 (67·41–395·1)	8·2% (-9·4 to 31·7%)
		United Arab Emirates	12·8% (5·2–25·7%)	19·9% (-2·2 to 44·1%)	183·12 (70·89–453·03)	11·9% (-10 to 39·7%)
		Yemen	8·9% (3·5–18·2%)	25·1% (5·3 to 45·1%)	117·04 (42·38–300·14)	22% (-0·4 to 47·3%)
		Afghanistan	8·8% (3·2–19·1%)	64% (37·7 to 108·2%)	133·17 (42·1–359·27)	46·6% (23·8 to 77·7%)
		Sudan	9·8% (3·7–20·4%)	44·9% (19·3 to 77·1%)	130·99 (45·23–336·84)	33·8% (11·8 to 63·7%)
	Alzheimer's disease and other dementias	Global	32·8% (21·8–45·8%)	3% (-5·5 to 9·6%)	111·34 (43·2–273·86)	6·9% (-2·2 to 15·2%)
		North Africa and Middle East	39·9% (26·4–54·7%)	20·3% (9·2 to 30·5%)	154·71 (58·76–375·07)	19% (6·7 to 37·7%)
		Algeria	43·4% (29·2–58·9%)	18% (3·9 to 31·5%)	174·4 (65·84–428·68)	12·1% (-6 to 34·5%)
		Bahrain	50·2% (33–68%)	15·8% (3·3 to 26·2%)	202·75 (75·42–507·7)	11·4% (-5·5 to 29·4%)
		Egypt	43·8% (30·4–57·3%)	38·8% (24 to 57·4%)	158·75 (63·21–371·78)	39% (15·6 to 70·9%)
		Iran	35·8% (22·9–50·6%)	28·9% (12·4 to 43·7%)	138·31 (51·75–336·34)	28·7% (11·3 to 59·1%)
		Iraq	44·5% (30–60·4%)	7·3% (-3·4 to 18·2%)	170·21 (64·71–412·34)	9·4% (-7 to 27·8%)
		Jordan	48·9% (34·6–63·6%)	8·3% (-2·2 to 18·3%)	181·02 (73·93–425·61)	3·5% (-11·6 to 22·9%)
		Kuwait	49·7% (33·6–65·4%)	14·3% (3·7 to 25·5%)	192·73 (76·14–449·12)	11·4% (-1·6 to 27·8%)
		Lebanon	51·1% (36·7–64·9%)	26·9% (15·7 to 40·4%)	202·05 (81·67–468·22)	23·8% (6·5 to 66·9%)
		Libya	42·6% (27·4–58·9%)	16·8% (3·3 to 28·1%)	176·96 (63·31–436·27)	11·9% (-7·7 to 38·5%)
		Morocco	34·4% (20·9–49·7%)	26·2% (7·9 to 44·9%)	133·04 (45·82–340·7)	26·7% (3·7 to 50·4%)
		Palestine	38% (23·8–53·4%)	17·9% (1·7 to 34·2%)	147·89 (54·98–374·49)	12·7% (-9·4 to 42%)
		Oman	37·2% (22·8–53·8%)	56·2% (31·2 to 93·9%)	157·32 (55·53–397·26)	51·2% (21·2 to 108·3%)
		Qatar	53·5% (34·4–71·6%)	32·6% (11·1 to 62·6%)	218·04 (82·09–556·08)	37% (7·5 to 77·7%)

Table S15 Burden of neurological conditions attributable to risk factors in North Africa and Middle East countries

			DALYs (95% UI)			
			PAF (%)	PAF change (%)	Rate per 100,000	Rate change (%)
Risk factors	Causes	Locations	2019	From 1990 to 2019	2019	From 1990 to 2019
		Saudi Arabia	43·8% (27·4–61·3%)	42·2% (28·3 to 66·7%)	170·84 (62·31–417·87)	39·2% (14·9 to 86%)
		Syrian Arab Republic	42% (27·9–57%)	8·4% (-4·2 to 20%)	164·29 (63·23–409·64)	11·6% (-10·6 to 43·4%)
		Tunisia	42·5% (28·3–57·8%)	16·3% (1·1 to 28·3%)	170·12 (63·54–397·23)	13·8% (-8·3 to 44·9%)
		Türkiye	41·2% (26·8–56·1%)	7·1% (-5·6 to 17·6%)	163·27 (63·04–389·57)	4·9% (-12 to 25%)
		United Arab Emirates	49·4% (32·3–66·4%)	29·4% (16·5 to 49·1%)	181·29 (68·34–451·55)	22·5% (2·5 to 52·5%)
		Yemen	31·5% (21·1–43%)	26·1% (10·2 to 41·8%)	121·09 (45·36–304·77)	25·6% (4·2 to 50·6%)
		Afghanistan	31·3% (18·4–46·8%)	49·7% (34 to 73·8%)	135·85 (44·79–362·37)	44% (21·6 to 72·4%)
		Sudan	37·4% (24·3–52·1%)	40·7% (20·2 to 64·1%)	137·19 (51·62–344·06)	35·1% (13·8 to 62%)
	Parkinson's disease	Global	-10% (-15·1–-5·2%)	-22·2% (-27·5 to -17·1%)	-8·02 (-12·33–-4·11)	-20·4% (-27·9 to -12·9%)
		North Africa and Middle East	-9·1% (-13·4–-4·8%)	-19·7% (-30 to -9·3%)	-7·65 (-11·54–-4·01)	-19% (-31·8 to -1·7%)
		Algeria	-9·1% (-13·6–-4·9%)	-24·4% (-39·5 to -8·4%)	-7·22 (-11·49–-3·66)	-38% (-55·3 to -14·6%)
		Bahrain	-8·2% (-12·5–-4·4%)	-28·7% (-46·2 to -5·5%)	-7·31 (-11·81–-3·64)	-38·3% (-57·6 to -11·6%)
		Egypt	-10·7% (-16·1–-5·5%)	21·8% (-1·9 to 50·1%)	-10·55 (-17·15–-5·12)	36·2% (0·6 to 80·7%)
		Iran	-6·4% (-9·8–-3·2%)	-23·3% (-37·6 to -7·8%)	-4·95 (-7·59–-2·39)	-20·8% (-40·1 to 4·9%)
		Iraq	-13% (-19·8–-7%)	-22% (-38·2 to -5%)	-11·14 (-17·84–-5·86)	-8·6% (-34·3 to 25·6%)
		Jordan	-13% (-19·7–-6·5%)	-27·2% (-42·1 to -7·9%)	-9·66 (-15·35–-4·7)	-37·1% (-54·1 to -17·7%)
		Kuwait	-10·5% (-15·4–-5·8%)	-10·8% (-30·1 to 12·8%)	-5·92 (-9·05–-3·05)	-34·7% (-51·7 to -13·6%)
		Lebanon	-17% (-25·5–-9%)	26·7% (0·5 to 57·3%)	-11·82 (-19·08–-6·02)	8·8% (-22·7 to 55·2%)
		Libya	-8·3% (-12·4–-4·4%)	-25·8% (-40·3 to -8·2%)	-7·03 (-11·29–-3·48)	-17·9% (-41·6 to 19·3%)
		Morocco	-5·2% (-8–-2·6%)	-34·1% (-46·6 to -17·7%)	-5 (-7·8–-2·41)	-7·9% (-33·3 to 24·8%)
		Palestine	-10·9% (-16·1–-5·7%)	-17·4% (-37·6 to 6·4%)	-10·11 (-15·79–-4·83)	-21·6% (-45·4 to 21·8%)
		Oman	-3·9% (-5·9–-2·1%)	-30·7% (-48·5 to -6·5%)	-4·9 (-7·63–-2·26)	-9·3% (-41·2 to 40%)
		Qatar	-6·2% (-9·4–-3·2%)	-11·5% (-34·2 to 15·1%)	-9·14 (-15·26–-4·55)	1·3% (-32·7 to 50·8%)
		Saudi Arabia	-5·9% (-8·8–-3·2%)	6·3% (-23·4 to 41·6%)	-6·55 (-10·08–-3·22)	7·7% (-29·9 to 74·6%)
		Syrian Arab Republic	-10·1% (-15·7–-5·2%)	-32% (-46·8 to -16%)	-8·51 (-13·98–-3·93)	-22·7% (-45·7 to 10%)
		Tunisia	-12·1% (-18·6–-6·1%)	-21·5% (-37·9 to -2·7%)	-8·79 (-14·38–-4·17)	-12·8% (-38·8 to 22·4%)
		Türkiye	-10·9% (-16·6–-5·3%)	-36·7% (-49·7 to -22·7%)	-8·21 (-13·68–-4·03)	-41·9% (-57·4 to -21·5%)
		United Arab Emirates	-7·3% (-11·3–-3·7%)	13·5% (-13·9 to 47·3%)	-9·45 (-15·95–-4·54)	1·8% (-31·8 to 50·1%)
		Yemen	-10·8% (-15·6–-5·9%)	-7·1% (-25·1 to 18·1%)	-8·27 (-12·97–-4·37)	7·2% (-23·8 to 55·5%)
		Afghanistan	-4·4% (-6·9–-2·3%)	21·8% (-7·4 to 53·8%)	-5·04 (-8·04–-2·55)	14·4% (-18·8 to 57·9%)
		Sudan	-8·8% (-13·2–-4·8%)	-11% (-33·9 to 16·9%)	-7·55 (-12·17–-3·96)	-15% (-41·1 to 31·7%)
	Idiopathic epilepsy	Global	10·1% (7·3–13%)	11·2% (-2·6 to 23·8%)	17·16 (11·54–23·99)	-7% (-16·7 to 3·9%)
		North Africa and Middle East	2·1% (1·4–3·1%)	11·8% (-16 to 45·8%)	3·4 (1·88–5·53)	-17% (-44·5 to 21·7%)
		Algeria	2·5% (1·4–3·6%)	103·9% (47·9 to 201·7%)	3·57 (1·45–6·69)	25·7% (-45 to 174·2%)
		Bahrain	3·7% (2·4–5·6%)	-48·2% (-62·9 to -34%)	6·94 (2·89–12·81)	-64·8% (-85·2 to -20%)
		Egypt	1% (0·4–1·7%)	21·1% (-31·8 to 123·7%)	1·05 (0·32–2·4)	-3·5% (-66·4 to 165%)
		Iran	1·8% (1·2–2·6%)	367·8% (206·3 to 790·7%)	2·54 (1·48–4·02)	204·6% (90·2 to 478·2%)
		Iraq	1·8% (0·9–2·9%)	-48·6% (-71·1 to -21·2%)	2·34 (0·8–4·95)	-64·2% (-86·1 to -18·8%)
		Jordan	2% (1–3·3%)	36·3% (-17·3 to 139·4%)	2·43 (0·81–5·22)	6·3% (-60·5 to 176·6%)
		Kuwait	0·3% (0–0·6%)		0·35 (0·04–1·02)	
		Lebanon	3·3% (2–4·8%)	-34·3% (-54·8 to -12·5%)	4·27 (1·85–8·28)	-51·4% (-78·9 to 2·9%)
		Libya	1·1% (0·3–1·7%)	353·6% (27·6 to 721·5%)	1·58 (0·46–2·86)	218% (-16·4 to 732·1%)
		Morocco	1·5% (0·8–2·4%)	-33·3% (-55·5 to -8·5%)	2·02 (0·62–4·57)	-41·1% (-80·8 to 45·7%)
		Palestine	4·1% (2·6–6%)	55·8% (16·1 to 143·6%)	6·58 (3·52–11·23)	17·3% (-39·2 to 139·2%)
		Oman	1·4% (0·6–2·3%)	40·5% (-3·6 to 121·4%)	1·45 (0·39–3·28)	26·3% (-57·8 to 272·4%)
		Qatar	2·5% (1·4–3·8%)	-16·9% (-38·9 to 9·4%)	3·12 (0·98–6·66)	-40·8% (-79·2 to 69·9%)
		Saudi Arabia	0·9% (0·2–1·9%)	-42·2% (-67 to -18·2%)	2·04 (0·31–4·94)	-43·1% (-78·4 to 26·9%)
		Syrian Arab Republic	1·5% (0·8–2·3%)	-42% (-60·2 to -22·7%)	1·57 (0·65–2·92)	-52·2% (-79·7 to 11%)
		Tunisia	6·5% (4·3–9%)	89·8% (45·9 to 153·5%)	7·65 (3·66–14·02)	37·5% (-33·7 to 202·8%)

Table S15 Burden of neurological conditions attributable to risk factors in North Africa and Middle East countries

			DALYs (95% UI)			
			PAF (%)	PAF change (%)	Rate per 100,000	Rate change (%)
Risk factors	Causes	Locations	2019	From 1990 to 2019	2019	From 1990 to 2019
		Türkiye	4·6% (2·9–6·4%)	45% (5·9 to 110·1%)	9·8 (4·23–18·05)	1·5% (-52·2 to 125·2%)
		United Arab Emirates	4·2% (2·3–6·5%)	-44·3% (-66·7 to -22·4%)	8·98 (3·52–17·33)	-60·4% (-84 to -12·8%)
		Yemen	1·5% (0·9–2·3%)	-45·2% (-65·5 to -21·1%)	2·23 (0·97–4·09)	-60·1% (-81·7 to -15·8%)
		Afghanistan	0·3% (0·1–0·6%)		0·7 (0·21–1·49)	
		Sudan	0·1% (0–0·2%)	-94·4% (-98·2 to -88·1%)	0·15 (0·04–0·39)	-96·3% (-99 to -91·1%)
	Multiple sclerosis	Global	13·7% (10·4–17·2%)	-25·6% (-30·1 to -19%)	1·92 (1·41–2·54)	-35·4% (-41·4 to -28·2%)
		North Africa and Middle East	10·8% (7·8–14·5%)	-12% (-28·2 to 5%)	2·15 (1·51–2·96)	-6·7% (-28·5 to 8·8%)
		Algeria	9·3% (6·6–12·1%)	-13% (-31·7 to 11·3%)	1·86 (1·19–2·65)	-1·8% (-30·6 to 27·5%)
		Bahrain	10·7% (7·7–13·9%)	-15·3% (-28 to -1·2%)	1·33 (0·85–1·95)	-4·7% (-30 to 22·4%)
		Egypt	10·3% (3·6–17·8%)	24·9% (-45·6 to 145·4%)	1·51 (0·82–3·25)	30·4% (-7 to 67·5%)
		Iran	9·8% (7·2–13·5%)	-6·6% (-22·4 to 14·2%)	2·89 (1·89–4·69)	-6·3% (-34·1 to 15%)
		Iraq	12·1% (8·9–16·1%)	-18·9% (-32·4 to -2·3%)	1·74 (1·13–2·56)	-8·9% (-32·6 to 16·9%)
		Jordan	15·8% (12·1–19·5%)	-3·2% (-18·5 to 16·1%)	3 (2·02–4·11)	-9·9% (-29·5 to 16·2%)
		Kuwait	11·2% (7·8–14·7%)	-25·4% (-36·7 to -11·7%)	1·94 (1·24–2·79)	3% (-15·4 to 26%)
		Lebanon	21·4% (16·2–26·9%)	21·7% (4·7 to 41·8%)	5·02 (3·35–7)	47% (13·9 to 92·5%)
		Libya	10·8% (7·3–14·3%)	-9·5% (-31·6 to 13·1%)	2·34 (1·48–3·23)	20·7% (-13·8 to 67%)
		Morocco	6·8% (4·9–8·8%)	-27·9% (-40·8 to -9·7%)	1·36 (0·87–1·89)	-9·9% (-36·8 to 23·1%)
		Palestine	13% (9·6–17·7%)	-5·3% (-24·9 to 23·5%)	2·54 (1·64–3·81)	2·9% (-34·2 to 43%)
		Oman	7·9% (5·6–10·6%)	-16·9% (-35·2 to 7·2%)	1·34 (0·82–1·93)	-0·3% (-40 to 37·9%)
		Qatar	9·8% (7–12·7%)	0·7% (-16·8 to 21·9%)	1·64 (1·03–2·38)	34·6% (-2·4 to 80·1%)
		Saudi Arabia	8·9% (6·4–11·9%)	27·3% (1·9 to 61·3%)	1·04 (0·66–1·52)	56·6% (9·9 to 129·6%)
		Syrian Arab Republic	11·4% (8·4–14·4%)	-19·7% (-34·7 to -3·7%)	1·72 (1·15–2·47)	-7·3% (-31·4 to 18·8%)
		Tunisia	11·1% (8·1–14·1%)	-11·3% (-26·6 to 11·1%)	2·46 (1·6–3·44)	11·5% (-15·6 to 43·6%)
		Türkiye	15·5% (10·5–19·8%)	-14·4% (-35·8 to 19·7%)	3·24 (2·26–4·44)	-24·4% (-44·7 to -3·9%)
		United Arab Emirates	11·2% (7·7–15·2%)	-9·7% (-27·1 to 9·9%)	2·31 (1·23–3·95)	7·7% (-31·6 to 58·1%)
		Yemen	14·3% (10·2–18·6%)	-7·4% (-23·3 to 13·2%)	1·99 (1·25–2·86)	16·6% (-22·8 to 68%)
		Afghanistan	6·2% (3·9–9·2%)	62·7% (27·1 to 111·4%)	1·66 (0·95–2·79)	81·4% (26·4 to 153·6%)
		Sudan	8·6% (5·6–12%)	-12·1% (-31·4 to 18%)	1·2 (0·68–1·86)	5·8% (-31·7 to 50·8%)
**Cluster of risk factors**						
Behavioural risk factors‡	Meningitis	Global	6·4% (5·7–7·3%)	13·4% (-1·6 to 30·6%)	14·9 (11·92–19·23)	-51·4% (-61 to -38·4%)
		North Africa and Middle East	6·8% (5·2–9%)	-10·3% (-35·5 to 22·9%)	4·79 (3·37–6·71)	-78·3% (-85·5 to -67·4%)
		Algeria	4% (2·4–6·3%)	-8·9% (-52·2 to 78·3%)	1·62 (0·91–2·7)	-81·7% (-91·1 to -62·8%)
		Bahrain	1·7% (1·2–2·4%)	-53·4% (-73·4 to -20%)	0·32 (0·21–0·46)	-88·1% (-93·5 to -78·8%)
		Egypt	9·2% (4·5–16%)	-33·7% (-69·4 to 32·3%)	5 (2·09–9·57)	-83·7% (-93·7 to -65·1%)
		Iran	4·3% (3·2–5·3%)	-34% (-51·9 to -11·9%)	1·64 (1·17–2·18)	-86·3% (-91·5 to -79·3%)
		Iraq	8·3% (5·3–12%)	-16·7% (-55·7 to 49·2%)	5·59 (3·35–8·89)	-83·3% (-91·6 to -67%)
		Jordan	5·7% (3·4–9·4%)	-11·6% (-54·7 to 86·4%)	3·03 (1·68–5·48)	-61·5% (-83·1 to -10·9%)
		Kuwait	5·9% (3·8–8·3%)	3·1% (-32·5 to 51·3%)	1·19 (0·65–1·78)	-61·8% (-77·6 to -40·2%)
		Lebanon	3·2% (1·6–5·4%)	-53·6% (-79·5 to -3·9%)	0·93 (0·43–1·64)	-85·4% (-93·8 to -68·6%)
		Libya	2·4% (1·4–4%)	-66·2% (-82·6 to -33·8%)	0·85 (0·48–1·48)	-89·8% (-95 to -80·2%)
		Morocco	9·8% (5·3–16·6%)	-27·1% (-66·3 to 54%)	6·76 (3·12–12·68)	-86·8% (-94·8 to -67·4%)
		Palestine	3·9% (2·5–5·7%)	-29·4% (-62·3 to 31·1%)	1·58 (0·97–2·42)	-87·6% (-93·8 to -75·6%)
		Oman	4·1% (2·2–7%)	-35% (-65·8 to 26·9%)	1 (0·53–1·87)	-83% (-91·4 to -64%)
		Qatar	3·3% (2·1–5%)	-43·6% (-69·4 to 1·8%)	0·83 (0·53–1·28)	-84·8% (-92·5 to -70·8%)
		Saudi Arabia	0·4% (0·2–0·7%)	-63·5% (-84·3 to -24·5%)	0·12 (0·06–0·19)	-90·2% (-95·5 to -80·2%)
		Syrian Arab Republic	2·6% (1·4–4·3%)	-53·1% (-78·4 to 2·5%)	2·56 (1·37–4·42)	-86·2% (-93·6 to -68·9%)
		Tunisia	5·9% (3·5–9·3%)	-6·6% (-51·7 to 84%)	2·24 (1·22–3·71)	-78·6% (-90 to -55·1%)
		Türkiye	2% (1·3–3·2%)	-36·6% (-66·6 to 20·3%)	0·51 (0·31–0·85)	-93·5% (-96·9 to -86·5%)
		United Arab Emirates	1·4% (0·7–3·4%)	-72·2% (-86·8 to -45·4%)	0·43 (0·21–1·08)	-92·9% (-96·5 to -85·7%)
		Yemen	5·1% (2·9–8·3%)	-10·6% (-55·6 to 76·3%)	4·96 (2·71–8·63)	-71·9% (-86·4 to -43·6%)

Table S15 Burden of neurological conditions attributable to risk factors in North Africa and Middle East countries

			**DALYs (95% UI)**			
			PAF (%)	PAF change (%)	Rate per 100,000	Rate change (%)
Risk factors	Causes	Locations	2019	From 1990 to 2019	2019	From 1990 to 2019
		Afghanistan	5·9% (3–10·4%)	-7·1% (-56·6 to 110·5%)	14·31 (7·06–25·73)	-75·4% (-89 to -43·5%)
		Sudan	5% (2·8–8·3%)	-9·1% (-50·3 to 65·6%)	4·99 (2·59–9·08)	-86·4% (-93 to -75%)
	Encephalitis	Global	2·3% (1·8–2·8%)	43·9% (3 to 92·7%)	1·53 (1·18–1·96)	-34·1% (-55·6 to 2%)
		North Africa and Middle East	3·7% (2·1–5·9%)	8·8% (-31·6 to 74·9%)	1·52 (0·9–2·5)	-14·5% (-51·5 to 51·4%)
		Algeria	2·3% (1·1–4·3%)	23·3% (-45·4 to 180·2%)	0·44 (0·23–0·78)	-12·2% (-64·5 to 112·1%)
		Bahrain	1% (0·6–1·6%)	-32·3% (-66·3 to 33·2%)	0·11 (0·06–0·2)	-49·5% (-77·1 to 2%)
		Egypt	6·6% (2·2–13·4%)	4·9% (-55 to 124·7%)	4·29 (0·77–9·74)	-28·8% (-72·2 to 63·4%)
		Iran	3·5% (1·4–4·8%)	7·6% (-25·3 to 43·9%)	0·58 (0·2–0·84)	-15·2% (-53·5 to 28·8%)
		Iraq	3·3% (1·6–5·7%)	10·3% (-48 to 140·9%)	2·33 (0·89–4·39)	-26·6% (-68·7 to 74·7%)
		Jordan	1·5% (0·8–2·5%)	-32·2% (-72·1 to 35·6%)	0·16 (0·08–0·28)	-73·5% (-90·7 to -6·1%)
		Kuwait	2·2% (1·5–3%)	20·6% (-20·3 to 92·8%)	0·23 (0·15–0·33)	-11·2% (-44·5 to 47·3%)
		Lebanon	2·6% (1·3–4·8%)	-3·6% (-60·4 to 139·5%)	0·43 (0·19–0·85)	-21·1% (-70·3 to 98·9%)
		Libya	2·5% (1·1–4·6%)	-19·8% (-68·5 to 87·3%)	0·4 (0·21–0·72)	-27·6% (-73·3 to 77·2%)
		Morocco	5·4% (2–10·4%)	-2·7% (-52·5 to 103·2%)	1·3 (0·62–2·44)	-19·3% (-65·2 to 84·1%)
		Palestine	1·9% (1·1–3·2%)	-10·2% (-58·5 to 101·8%)	0·31 (0·17–0·52)	-29·3% (-69·2 to 56·7%)
		Oman	1·9% (1–3·6%)	2·7% (-55·6 to 153·6%)	2·36 (1·12–4·28)	-42·6% (-76·3 to 39·3%)
		Qatar	1·5% (0·8–2·4%)	-16·9% (-62·2 to 86·7%)	0·2 (0·1–0·36)	-41·1% (-76·5 to 32·8%)
		Saudi Arabia	0·5% (0·1–1·7%)	1·5% (-71·8 to 128·7%)	0·14 (0·04–0·57)	-48·4% (-87·3 to 10·2%)
		Syrian Arab Republic	1·5% (0·9–2·2%)	-24·1% (-61·5 to 37·8%)	0·4 (0·23–0·69)	-55% (-79·3 to -7·4%)
		Tunisia	2·7% (1·3–5%)	4·1% (-55·7 to 158·3%)	0·46 (0·24–0·84)	-26·6% (-72·3 to 80·1%)
		Türkiye	0·9% (0·4–2%)	-19·9% (-66·5 to 67·6%)	0·17 (0·08–0·35)	-57·9% (-83·7 to -4·3%)
		United Arab Emirates	2·1% (1–3·9%)	-17·9% (-67·6 to 98·6%)	0·37 (0·16–0·69)	-34·6% (-75·4 to 68·7%)
		Yemen	2·3% (1·1–4·5%)	5% (-55·4 to 167·7%)	0·67 (0·34–1·25)	-2·1% (-57·5 to 138·7%)
		Afghanistan	1·1% (0·5–2·3%)	6·7% (-56·6 to 155·4%)	2·07 (0·84–5·18)	-9·4% (-62·7 to 119·3%)
		Sudan	2% (1–3·9%)	3·8% (-56·7 to 169·7%)	0·61 (0·33–1·04)	-20·4% (-66·6 to 104·2%)
	Stroke	Global	46·6% (40·5–53·5%)	-5·4% (-8·4 to -2·6%)	823·57 (707·33–963·08)	-38·7% (-44·6 to -33·2%)
		North Africa and Middle East	34% (29·6–38·9%)	1·3% (-4·8 to 7·5%)	621·84 (517·96–746·83)	-31·1% (-39·8 to -19·7%)
		Algeria	34·1% (28·8–39·9%)	-8·7% (-16·6 to -0·7%)	598·22 (464·26–757·41)	-49·4% (-60 to -35·9%)
		Bahrain	29·8% (24·9–35·6%)	-13·7% (-22 to -6·1%)	280·11 (215·71–361·94)	-59% (-67·5 to -49·6%)
		Egypt	29·2% (24·5–35%)	37·5% (13·1 to 58·5%)	626·69 (445·48–906·17)	-10·9% (-32·9 to 16·4%)
		Iran	31·2% (26·4–36·7%)	-10·8% (-16 to -5·7%)	394·44 (327·43–474·93)	-51·6% (-57·7 to -44·3%)
		Iraq	38·4% (33·2–43·8%)	-2·7% (-9·4 to 4·3%)	1123·49 (857·06–1396·03)	-21·5% (-39·3 to -0·3%)
		Jordan	39·7% (34·6–45·1%)	-7·4% (-12·5 to -2·5%)	575·7 (457·49–701·54)	-53·9% (-62·9 to -44·7%)
		Kuwait	39·3% (33·3–45·8%)	-3·7% (-8·6 to 1·3%)	368·81 (288·68–456)	-14·9% (-27·8 to 0·6%)
		Lebanon	44·5% (39·7–49·8%)	17·8% (9·5 to 26·1%)	335·1 (254·22–430·95)	-21·9% (-39 to -1·9%)
		Libya	35·1% (29·9–41%)	14·7% (2·7 to 26%)	552·04 (406·87–743·3)	-9·8% (-30·4 to 18·5%)
		Morocco	29·4% (24·1–35·1%)	-12·8% (-22·4 to -2·8%)	662·81 (496·68–874·36)	-27·8% (-44·9 to -8%)
		Palestine	38·1% (32·8–43·7%)	-7·3% (-13·2 to -1·2%)	810·72 (655·39–989·01)	-37·4% (-51·3 to -20%)
		Oman	26·9% (21·6–32·7%)	-12·1% (-21·9 to -3·2%)	507·34 (394·09–645·57)	-46·1% (-59 to -27·9%)
		Qatar	27·4% (22·2–33·4%)	-5·9% (-14·9 to 2·2%)	247·75 (179·45–327·6)	-47·9% (-60·7 to -33·5%)
		Saudi Arabia	34·6% (28·7–40·8%)	12% (3·9 to 20·6%)	733·38 (537·4–930·26)	-20·6% (-41·8 to 9%)
		Syrian Arab Republic	37·1% (32·6–42%)	1·5% (-5·9 to 9%)	749·66 (564·29–986·67)	-39·3% (-55·7 to -16%)
		Tunisia	32·7% (28·7–37·3%)	-6·3% (-13·2 to 1·3%)	483·49 (362·33–650·51)	-29·4% (-48 to -4·1%)
		Türkiye	33·6% (29·7–38%)	-12·1% (-19 to -6%)	390·61 (306·85–486·9)	-32·8% (-52·9 to -14·4%)
		United Arab Emirates	37·1% (31·1–43·4%)	5·1% (-3·9 to 13·7%)	714·2 (518·65–943·48)	-43·2% (-58·1 to -25·4%)
		Yemen	44% (38·6–49·2%)	1·1% (-6·3 to 8·9%)	1218·44 (918·14–1614·27)	-23·7% (-42·4 to 2·3%)
		Afghanistan	40% (34–45·9%)	7·1% (0·1 to 16·1%)	1397·87 (987·49–1914·43)	-16·1% (-36·2 to 6%)
		Sudan	38·2% (32·3–44·3%)	0·7% (-9·1 to 11·1%)	989·85 (705·85–1424·5)	-32·6% (-47·6 to -13·6%)
	Ischaemic	Global	42% (34·9–49·1%)	-3·7% (-6·8 to -1%)	335·39 (275·68–404·02)	-31·1% (-38·1 to -25·2%)

Table S15 Burden of neurological conditions attributable to risk factors in North Africa and Middle East countries

			DALYs (95% UI)			
			PAF (%)	PAF change (%)	Rate per 100,000	Rate change (%)
Risk factors	Causes	Locations	2019	From 1990 to 2019	2019	From 1990 to 2019
	stroke	North Africa and Middle East	35·3% (29·2–42%)	-2·5% (-6·4 to 0·7%)	418·1 (334·97–521·45)	-11% (-23·6 to 1·1%)
		Algeria	35·2% (28·6–42·6%)	-7·5% (-13·7 to -1·8%)	449·47 (335·57–583·57)	-30·4% (-44·7 to -13·4%)
		Bahrain	31·2% (24·3–39%)	-11·6% (-19·5 to -5·2%)	191·36 (141–254·69)	-50·4% (-59·8 to -40·8%)
		Egypt	32·1% (25·8–39%)	16·9% (3·9 to 31·1%)	443·61 (309·56–630·09)	33·2% (-4 to 72·5%)
		Iran	32·6% (26·7–39·3%)	-10·4% (-15·4 to -5·7%)	320·47 (259·7–395·55)	-48·7% (-55·3 to -41·5%)
		Iraq	39% (32·5–46·6%)	-4·5% (-10·9 to 1·8%)	714·15 (534·94–901·38)	-11·2% (-30·5 to 10%)
		Jordan	40% (33·8–46·7%)	-6·8% (-12 to -2·1%)	433·53 (337·92–544·63)	-50% (-59·8 to -40·4%)
		Kuwait	39·7% (32·2–47·8%)	-4·5% (-9·6 to 0·3%)	256·02 (194·36–321·04)	-16% (-28·1 to -0·9%)
		Lebanon	45·2% (39·5–51·4%)	16% (9 to 25·6%)	269·91 (200·85–343·6)	-1·5% (-24·2 to 21·4%)
		Libya	36·9% (30·2–43·9%)	6% (-0·9 to 13·2%)	414·97 (296·17–568·92)	17·9% (-7·8 to 52%)
		Morocco	31·3% (24·5–38·7%)	-10·8% (-19 to -3·7%)	502·57 (369·2–674·2)	5·7% (-21·3 to 32·2%)
		Palestine	38·5% (32·2–45·5%)	-6·6% (-12·2 to -1·3%)	612·25 (483·43–763·19)	-23·3% (-39·7 to -2·5%)
		Oman	29·2% (21·9–36·9%)	-14% (-23·2 to -6%)	377·06 (274·5–493·2)	-29·8% (-46·2 to -6·6%)
		Qatar	28·9% (21·4–37·3%)	-6·4% (-14·8 to 0·6%)	167·08 (115·51–234·2)	-39·6% (-53·6 to -24·5%)
		Saudi Arabia	36% (27·6–44·2%)	11·6% (3·8 to 20·2%)	468·52 (331·82–611·03)	-7·8% (-30·3 to 23·7%)
		Syrian Arab Republic	39·2% (32·9–46%)	-2·3% (-8·5 to 3·8%)	420·08 (313·06–543·38)	-26·8% (-46·6 to -2·1%)
		Tunisia	32·5% (27·4–38%)	-6·1% (-12·6 to 0·2%)	355·02 (260·46–480·28)	-2·2% (-29 to 29·7%)
		Türkiye	33·8% (28·1–40·5%)	-11·9% (-19 to -5·8%)	223·76 (170·63–288·85)	-18·9% (-43·4 to 1·2%)
		United Arab Emirates	37·4% (29·6–45·1%)	5% (-3 to 14·3%)	529·53 (384·68–704·14)	-30·6% (-47·9 to -8·3%)
		Yemen	43·7% (37·2–50·6%)	3·4% (-2·1 to 9·3%)	751·05 (569·53–1000·73)	23·9% (-3·3 to 60·5%)
		Afghanistan	39·8% (32·4–47·7%)	7·5% (3·1 to 13%)	742·21 (519·99–1028)	37% (4·9 to 73%)
		Sudan	40·7% (32·8–48·3%)	0·6% (-5·7 to 7·2%)	688·69 (477·74–1021)	8·8% (-13·2 to 38·2%)
	Intracerebral haemorrhage	Global	51·1% (44–58·9%)	-4·9% (-8·2 to -1·8%)	425·25 (358·86–499·8)	-39·7% (-47 to -33·2%)
		North Africa and Middle East	32·1% (27·9–37·1%)	-1·4% (-7·4 to 6·5%)	176·31 (143·96–214·44)	-52·4% (-59·6 to -42·2%)
		Algeria	31% (25·8–36·9%)	-15·5% (-24·6 to -6·2%)	125·32 (91·19–166·21)	-72·9% (-80·3 to -63·4%)
		Bahrain	27·3% (22·4–32·7%)	-22·3% (-30·6 to -13·9%)	72·26 (53·73–96·55)	-72·2% (-79 to -63·1%)
		Egypt	25% (20–30·7%)	24·3% (1 to 55·8%)	161·4 (106·01–249·67)	-49·4% (-65·4 to -26·8%)
		Iran	26·9% (22·8–31·8%)	-15·3% (-22·6 to -7·5%)	60·02 (50·45–71·64)	-60·8% (-67·2 to -53·2%)
		Iraq	37·5% (32·3–43·7%)	-2·8% (-11·6 to 6·7%)	384·11 (285·66–511·82)	-31·9% (-51·5 to -8%)
		Jordan	38·7% (33·4–44·7%)	-9·3% (-16 to -2·7%)	126·23 (97·6–157·03)	-63·7% (-71·5 to -54·4%)
		Kuwait	38·8% (32·4–45·9%)	-2·7% (-9·1 to 4·4%)	95·58 (72·35–121·81)	-6·8% (-26·5 to 17·3%)
		Lebanon	41·8% (37·1–46·9%)	14·6% (5 to 25·7%)	53·81 (39·09–75·58)	-58·7% (-70·8 to -42%)
		Libya	31·2% (25·5–38%)	9% (-4·9 to 26·7%)	117·73 (80·19–173·29)	-46·8% (-62·3 to -23·8%)
		Morocco	24·7% (19·7–30·6%)	-25% (-36·4 to -13·3%)	137·02 (96·8–189·04)	-63·2% (-74·6 to -47·2%)
		Palestine	37% (31·3–43%)	-9·5% (-17·9 to -0·1%)	180·69 (140·58–228·69)	-60·8% (-70·8 to -46·7%)
		Oman	22·1% (16·6–27·5%)	-21·2% (-30·8 to -12·4%)	111·31 (80·25–154)	-68·4% (-76·7 to -55·8%)
		Qatar	24·2% (19·3–29·9%)	-10·8% (-21·6 to 0·5%)	58·89 (41·67–81·26)	-61% (-72·8 to -46·6%)
		Saudi Arabia	32·5% (26·6–39·2%)	10% (-0·1 to 22·7%)	248·66 (175·88–329·91)	-35·4% (-55·3 to -3·9%)
		Syrian Arab Republic	36·4% (30·8–42·2%)	-3·4% (-12 to 4·9%)	305·58 (217·72–415·7)	-50·2% (-64·7 to -27·8%)
		Tunisia	33·6% (28·9–38·9%)	-6·3% (-14·9 to 2·9%)	108·51 (76·44–149)	-59·3% (-72·4 to -39·3%)
		Türkiye	32·9% (29·1–37·3%)	-13·7% (-22·2 to -5·7%)	131·34 (102·41–165·87)	-45·6% (-63·9 to -27·7%)
		United Arab Emirates	36·4% (30·3–42·7%)	2% (-10·3 to 16·7%)	163·97 (108·55–250·12)	-63·4% (-76·6 to -44·9%)
		Yemen	44·9% (38·7–51·3%)	-0·2% (-6·9 to 7%)	397·13 (278·81–548·6)	-53·8% (-66·7 to -35·1%)
		Afghanistan	40·4% (32·4–48%)	7·3% (1·2 to 14·5%)	563·89 (382·96–790·53)	-42% (-57·2 to -22·8%)
		Sudan	33·8% (27·2–40·6%)	-8·3% (-17·2 to 2·8%)	261·22 (169·21–388·16)	-63·9% (-74 to -49·8%)
	Subarachnoid haemorrhage	Global	46·1% (39·2–53·6%)	-9·5% (-13·7 to -4·2%)	62·93 (51·47–77·11)	-58·6% (-65·2 to -41·2%)
		North Africa and Middle East	29·1% (24·7–33·9%)	17·3% (1·5 to 44%)	27·42 (21·48–36·32)	-56·5% (-68·5 to -33·2%)
		Algeria	30·4% (24·8–36·5%)	-11·4% (-23·7 to 2·8%)	23·42 (16·01–33·27)	-68·6% (-79·1 to -50·1%)
		Bahrain	26·2% (20·8–31·8%)	3·5% (-12 to 24%)	16·49 (11·43–24·53)	-56·6% (-71·5 to -36·6%)

Table S15 Burden of neurological conditions attributable to risk factors in North Africa and Middle East countries

Risk factors	Causes	Locations	DALYs (95% UI) PAF (%) 2019	PAF change (%) From 1990 to 2019	Rate per 100,000 2019	Rate change (%) From 1990 to 2019
		Egypt	18·9% (13·9–26%)	81·6% (23·3 to 233·3%)	21·68 (13·63–33·96)	-58·1% (-75·1 to -21·7%)
		Iran	25·6% (21·3–30·9%)	-14·4% (-25 to -0·5%)	13·96 (11·16–17·74)	-62·1% (-73·9 to -40·9%)
		Iraq	34·9% (29·5–41·7%)	10·3% (-4·6 to 26·4%)	25·24 (17·61–36·15)	-60·3% (-75·7 to -35·2%)
		Jordan	41% (35·4–46·9%)	-7·2% (-14·5 to 1%)	15·94 (11·8–20·24)	-53·4% (-67·5 to -32·3%)
		Kuwait	36·7% (30·2–43·5%)	0·3% (-8·6 to 9·9%)	17·22 (13·12–22·08)	-34·7% (-48·6 to -11·7%)
		Lebanon	42·6% (37·5–47·7%)	28·9% (16·3 to 44·8%)	11·39 (7·84–16·16)	-53·9% (-73 to -24·5%)
		Libya	26·9% (21–34·1%)	47·7% (10·2 to 108·9%)	19·34 (12·77–30·58)	-50·2% (-66·8 to -20·1%)
		Morocco	23·1% (17·9–28·7%)	-19·2% (-33 to -0·1%)	23·22 (14·34–37·09)	-66·6% (-79·4 to -40·7%)
		Palestine	35·5% (29·6–41·9%)	-10·1% (-19·9 to 1%)	17·77 (13·57–22·27)	-51·5% (-66·5 to -27·7%)
		Oman	21·2% (16·3–26·9%)	-4·4% (-22·1 to 25·1%)	18·97 (11·24–32·63)	-64·2% (-78·2 to -28·9%)
		Qatar	26% (20·7–32·4%)	-1·3% (-18·4 to 19·8%)	21·79 (14·59–29·86)	-54·9% (-74·6 to -26·5%)
		Saudi Arabia	32·7% (26·6–39·8%)	12·7% (-1·6 to 29·6%)	16·2 (10·78–22·21)	-46·5% (-70·2 to -8·6%)
		Syrian Arab Republic	22% (17·3–27·3%)	45·1% (6·1 to 100·7%)	24 (17·26–33·63)	-50% (-69·2 to -16·2%)
		Tunisia	30·9% (25·1–36·8%)	-4·1% (-17·4 to 13·4%)	19·97 (13·07–28·79)	-64·2% (-78·9 to -37·9%)
		Türkiye	35·2% (31–39·5%)	-7·7% (-17·4 to 4%)	35·51 (26·69–45·73)	-44·5% (-65·8 to -3·8%)
		United Arab Emirates	33·9% (27·5–40·5%)	21·3% (0·9 to 47·7%)	20·7 (10·23–34·7)	-57% (-75·3 to -29·3%)
		Yemen	42·6% (35·5–49·6%)	4·8% (-5·5 to 17·8%)	70·26 (36·18–120·95)	-46·6% (-63·2 to -12·6%)
		Afghanistan	39·1% (30·7–46·9%)	6·7% (-2·4 to 16%)	91·77 (31·12–166·6)	-39·8% (-59·3 to -5·1%)
		Sudan	32·3% (25·2–39·6%)	0·1% (-14·3 to 23·3%)	39·94 (21·06–75·15)	-64·1% (-77·1 to -31·6%)
	Neurological disorders*	Global	5·1% (2·4–9·3%)	-11·1% (-16·8 to -5·3%)	62·36 (29·43–129·99)	-12·4% (-17·9 to -6·1%)
		North Africa and Middle East	4·2% (1·6–8·7%)	-5·5% (-15 to 5%)	57·43 (21·05–137·7)	-9·8% (-18·9 to 4%)
		Algeria	5·3% (2–10·8%)	-6·1% (-22·3 to 16·1%)	71·69 (25·43–174·23)	-15·2% (-30·6 to 5·6%)
		Bahrain	4·8% (1·9–9·8%)	-17·1% (-34·8 to 1%)	66 (24·95–153·87)	-24·2% (-43·2 to -3·8%)
		Egypt	5·2% (1·7–11·4%)	34·5% (11·4 to 60·7%)	68·64 (22·32–171·11)	35·1% (10·9 to 63·3%)
		Iran	3·4% (1·3–6·8%)	-1·2% (-15·8 to 18·4%)	46·44 (17·78–106·51)	-6·2% (-19·8 to 16%)
		Iraq	4·9% (1·7–10·5%)	-15% (-30·6 to -0·2%)	64·74 (20·78–160·07)	-16·5% (-32·9 to -0·3%)
		Jordan	6·1% (2·3–12·8%)	-10·9% (-27·1 to 10·8%)	77·68 (28·33–183·92)	-15·9% (-32·4 to 6·4%)
		Kuwait	5·3% (2–11%)	1% (-18·5 to 29·3%)	67·91 (24·43–158·09)	-4·4% (-20·9 to 17·5%)
		Lebanon	8·3% (3·3–16·9%)	19·6% (-1·1 to 45·4%)	110·61 (41·44–257·97)	15% (-7·2 to 51·9%)
		Libya	3·7% (1·4–7·6%)	-19·2% (-34·4 to 0·3%)	50·91 (17·35–123·46)	-21·9% (-38·1 to 1·3%)
		Morocco	2·6% (0·9–5·5%)	-29·3% (-44·3 to -15·8%)	34·57 (11·69–87·38)	-27·7% (-44·6 to -12·9%)
		Palestine	4·4% (1·7–8·9%)	-6·6% (-22·5 to 16%)	59·57 (21·84–146·77)	-12·1% (-29·7 to 14·2%)
		Oman	2·4% (0·8–5%)	-21·7% (-38 to 0·3%)	32·35 (10·02–78·68)	-21·1% (-38·7 to 4·5%)
		Qatar	4·2% (1·5–8·8%)	43·3% (11·8 to 78·6%)	56·5 (17·03–141·38)	40·3% (3·3 to 85·9%)
		Saudi Arabia	2·7% (0·9–5·7%)	-1·5% (-30·7 to 34·4%)	37·94 (11·38–95·31)	-1·5% (-31·3 to 38·8%)
		Syrian Arab Republic	5·1% (1·8–10·7%)	-17·1% (-33·5 to 1·4%)	66·86 (22·26–162·13)	-15·9% (-35·2 to 10·5%)
		Tunisia	5·3% (2·2–10·3%)	-11·1% (-25·3 to 6·2%)	70·09 (26·33–160·22)	-13·5% (-30·9 to 14·6%)
		Türkiye	4·7% (2·1–8·7%)	-9·8% (-25 to 12%)	68·96 (28·6–152·4)	-18·7% (-34·4 to 7·3%)
		United Arab Emirates	4·1% (1·6–8·6%)	-6·5% (-38·4 to 18·5%)	58·72 (20·72–141·84)	-12·3% (-46·3 to 20·2%)
		Yemen	5% (1·8–10·4%)	7·3% (-14·2 to 28·8%)	65·41 (22·08–162·98)	4·8% (-18·6 to 31·3%)
		Afghanistan	2·5% (0·8–5·3%)	39·8% (7·4 to 89·5%)	37·25 (10·85–97·42)	24·9% (-4·9 to 62·3%)
		Sudan	3·6% (1·2–8%)	-1·2% (-24·7 to 25·5%)	48·17 (14·41–122·46)	-8·6% (-30·7 to 13·5%)
	Alzheimer's disease and other dementias	Global	15·1% (9·7–20·3%)	-17·3% (-22·8 to -13%)	51·3 (20·5–116·23)	-14·2% (-20·2 to -8·4%)
		North Africa and Middle East	15·4% (9·9–20·7%)	-9·8% (-16·6 to -3·1%)	59·52 (23·58–138·55)	-10·8% (-19·1 to 0·9%)
		Algeria	18·3% (12–24·6%)	-15·3% (-26·2 to -3·8%)	73·48 (28·36–173·87)	-19·7% (-34·5 to -3·1%)
		Bahrain	16·2% (9·7–22·5%)	-13·2% (-29·5 to 6·2%)	65·04 (24·5–154·34)	-16·5% (-33·8 to 6·7%)
		Egypt	21·2% (13·7–27·8%)	35·9% (18·8 to 56%)	76·64 (30·52–179·39)	36·1% (12 to 65·5%)
		Iran	11·9% (7·4–16·7%)	-11·1% (-24·2 to 2%)	45·95 (17·5–104·67)	-11·3% (-24·8 to 7·2%)
		Iraq	18·7% (12·3–25%)	-13·4% (-26·3 to -1·8%)	71·81 (28·39–166·42)	-11·6% (-27·2 to 4%)
		Jordan	22·2% (14·7–29·6%)	-16·1% (-28·4 to -3·5%)	81·91 (33·06–185·32)	-19·7% (-35·1 to -3·1%)

Table S15 Burden of neurological conditions attributable to risk factors in North Africa and Middle East countries

Risk factors	Causes	Locations	DALYs (95% UI)<br>PAF (%)<br>2019	PAF change (%)<br>From 1990 to 2019	Rate per 100,000<br>2019	Rate change (%)<br>From 1990 to 2019
		Kuwait	18·4% (11·8–25·6%)	-6·4% (-21 to 10·4%)	71·55 (28·66–161·31)	-8·6% (-24·7 to 10·3%)
		Lebanon	28·6% (18·5–37·6%)	22·3% (3 to 42·1%)	113·14 (44·96–260·28)	19·3% (-3·1 to 61·1%)
		Libya	13% (8·3–17·5%)	-20·8% (-31·4 to -9·4%)	54·03 (20–125·95)	-24·3% (-40 to -4%)
		Morocco	9·4% (5·6–13·4%)	-25·4% (-38·9 to -13·3%)	36·19 (13·29–88·23)	-25·1% (-40·2 to -9·7%)
		Palestine	15·6% (10·1–20·9%)	-12·8% (-27·7 to 2·2%)	60·57 (23·4–143·42)	-16·6% (-32·9 to 7%)
		Oman	8·2% (4·9–11·8%)	-18·9% (-34·6 to 1·2%)	34·46 (12·68–81·66)	-21·6% (-37·8 to 3·2%)
		Qatar	15% (9–21%)	37·9% (10·1 to 68%)	60·88 (22·14–145·63)	42·2% (4·6 to 85·2%)
		Saudi Arabia	10·6% (6·3–15·5%)	5% (-20·1 to 34·6%)	41·4 (15·38–99)	2·7% (-25·9 to 43·7%)
		Syrian Arab Republic	18·5% (11·7–25·1%)	-17·8% (-30·7 to -5·4%)	72·09 (27·43–168·15)	-15·6% (-34·6 to 10%)
		Tunisia	17·1% (11·6–22·7%)	-15·8% (-26·7 to -4·5%)	68·77 (27·05–161·72)	-17·5% (-34·3 to 6·7%)
		Türkiye	16·2% (10·5–21·7%)	-23·1% (-34·6 to -13·1%)	64·14 (24·87–147·81)	-24·6% (-37·9 to -8·4%)
		United Arab Emirates	15·5% (9·4–22·5%)	16·7% (-6·1 to 46·1%)	56·89 (20·99–135·49)	10·7% (-15·4 to 49%)
		Yemen	18·1% (11·5–24·6%)	10·9% (-7·5 to 31%)	69·46 (26·81–167·49)	10·5% (-12·5 to 38·1%)
		Afghanistan	9·2% (5·6–13·3%)	24·6% (-1·8 to 51·6%)	39·94 (13·66–101·34)	19·9% (-8·2 to 51·3%)
		Sudan	14·8% (8·9–21·3%)	0·5% (-18·3 to 22·7%)	54·37 (20·43–131·07)	-3·5% (-23·1 to 20·4%)
	Parkinson's disease	Global	-10% (-15·1–-5·2%)	-22·2% (-27·5 to -17·1%)	-8·02 (-12·33–-4·11)	-20·4% (-27·9 to -12·9%)
		North Africa and Middle East	-9·1% (-13·4–-4·8%)	-19·7% (-30 to -9·3%)	-7·65 (-11·54–-4·01)	-19% (-31·8 to -1·7%)
		Algeria	-9·1% (-13·6–-4·9%)	-24·4% (-39·5 to -8·4%)	-7·22 (-11·49–-3·66)	-38% (-55·3 to -14·6%)
		Bahrain	-8·2% (-12·5–-4·4%)	-28·7% (-46·2 to -5·5%)	-7·31 (-11·81–-3·64)	-38·3% (-57·6 to -11·6%)
		Egypt	-10·7% (-16·1–-5·5%)	21·8% (-1·9 to 50·1%)	-10·55 (-17·15–-5·12)	36·2% (0·6 to 80·7%)
		Iran	-6·4% (-9·8–-3·2%)	-23·3% (-37·6 to -7·8%)	-4·95 (-7·59–-2·39)	-20·8% (-40·1 to 4·9%)
		Iraq	-13% (-19·8–-7%)	-22% (-38·2 to -5%)	-11·14 (-17·84–-5·86)	-8·6% (-34·3 to 25·6%)
		Jordan	-13% (-19·7–-6·5%)	-27·2% (-42·1 to -7·9%)	-9·66 (-15·35–-4·7)	-37·1% (-54·1 to -17·7%)
		Kuwait	-10·5% (-15·4–-5·8%)	-10·8% (-30·1 to 12·8%)	-5·92 (-9·05–-3·05)	-34·7% (-51·7 to -13·6%)
		Lebanon	-17% (-25·5–-9%)	26·7% (0·5 to 57·3%)	-11·82 (-19·08–-6·02)	8·8% (-22·7 to 55·2%)
		Libya	-8·3% (-12·4–-4·4%)	-25·8% (-40·3 to -8·2%)	-7·03 (-11·29–-3·48)	-17·9% (-41·6 to 19·3%)
		Morocco	-5·2% (-8–-2·6%)	-34·1% (-46·6 to -17·7%)	-5 (-7·8–-2·41)	-7·9% (-33·3 to 24·8%)
		Palestine	-10·9% (-16·1–-5·7%)	-17·4% (-37·6 to 6·4%)	-10·11 (-15·79–-4·83)	-21·6% (-45·4 to 21·8%)
		Oman	-3·9% (-5·9–-2·1%)	-30·7% (-48·5 to -6·5%)	-4·9 (-7·63–-2·26)	-9·3% (-41·2 to 40%)
		Qatar	-6·2% (-9·4–-3·2%)	-11·5% (-34·2 to 15·1%)	-9·14 (-15·26–-4·55)	1·3% (-32·7 to 50·8%)
		Saudi Arabia	-5·9% (-8·8–-3·2%)	6·3% (-23·4 to 41·6%)	-6·55 (-10·08–-3·22)	7·7% (-29·9 to 74·6%)
		Syrian Arab Republic	-10·1% (-15·7–-5·2%)	-32% (-46·8 to -16%)	-8·51 (-13·98–-3·93)	-22·7% (-45·7 to 10%)
		Tunisia	-12·1% (-18·6–-6·1%)	-21·5% (-37·9 to -2·7%)	-8·79 (-14·38–-4·17)	-12·8% (-38·8 to 22·4%)
		Türkiye	-10·9% (-16·6–-5·3%)	-36·7% (-49·7 to -22·7%)	-8·21 (-13·68–-4·03)	-41·9% (-57·4 to -21·5%)
		United Arab Emirates	-7·3% (-11·3–-3·7%)	13·5% (-13·9 to 47·3%)	-9·45 (-15·95–-4·54)	1·8% (-31·8 to 50·1%)
		Yemen	-10·8% (-15·6–-5·9%)	-7·1% (-25·1 to 18·1%)	-8·27 (-12·97–-4·37)	7·2% (-23·8 to 55·5%)
		Afghanistan	-4·4% (-6·9–-2·3%)	21·8% (-7·4 to 53·8%)	-5·04 (-8·04–-2·55)	14·4% (-18·8 to 57·9%)
		Sudan	-8·8% (-13·2–-4·8%)	-11% (-33·9 to 16·9%)	-7·55 (-12·17–-3·96)	-15% (-41·1 to 31·7%)
	Idiopathic epilepsy	Global	10·1% (7·3–13%)	11·2% (-2·6 to 23·8%)	17·16 (11·54–23·99)	-7% (-16·7 to 3·9%)
		North Africa and Middle East	2·1% (1·4–3·1%)	11·8% (-16 to 45·8%)	3·4 (1·88–5·53)	-17% (-44·5 to 21·7%)
		Algeria	2·5% (1·4–3·6%)	103·9% (47·9 to 201·7%)	3·57 (1·45–6·69)	25·7% (-45 to 174·2%)
		Bahrain	3·7% (2·4–5·6%)	-48·2% (-62·9 to -34%)	6·94 (2·89–12·81)	-64·8% (-85·2 to -20%)
		Egypt	1% (0·4–1·7%)	21·1% (-31·8 to 123·7%)	1·05 (0·32–2·4)	-3·5% (-66·4 to 165%)
		Iran	1·8% (1·2–2·6%)	367·8% (206·3 to 790·7%)	2·54 (1·48–4·02)	204·6% (90·2 to 478·2%)
		Iraq	1·8% (0·9–2·9%)	-48·6% (-71·1 to -21·2%)	2·34 (0·8–4·95)	-64·2% (-86·1 to -18·8%)
		Jordan	2% (1–3·3%)	36·3% (-17·3 to 139·4%)	2·43 (0·81–5·22)	6·3% (-60·5 to 176·6%)
		Kuwait	0·3% (0–0·6%)		0·35 (0·04–1·02)	
		Lebanon	3·3% (2–4·8%)	-34·3% (-54·8 to -12·5%)	4·27 (1·85–8·28)	-51·4% (-78·9 to 2·9%)
		Libya	1·1% (0·3–1·7%)	353·6% (27·6 to 721·5%)	1·58 (0·46–2·86)	218% (-16·4 to 732·1%)

Table S15 Burden of neurological conditions attributable to risk factors in North Africa and Middle East countries

Risk factors	Causes	Locations	DALYs (95% UI) PAF (%) 2019	PAF change (%) From 1990 to 2019	Rate per 100,000 2019	Rate change (%) From 1990 to 2019
		Morocco	1·5% (0·8–2·4%)	-33·3% (-55·5 to -8·5%)	2·02 (0·62–4·57)	-41·1% (-80·8 to 45·7%)
		Palestine	4·1% (2·6–6%)	55·8% (16·1 to 143·6%)	6·58 (3·52–11·23)	17·3% (-39·2 to 139·2%)
		Oman	1·4% (0·6–2·3%)	40·5% (-3·6 to 121·4%)	1·45 (0·39–3·28)	26·3% (-57·8 to 272·4%)
		Qatar	2·5% (1·4–3·8%)	-16·9% (-38·9 to 9·4%)	3·12 (0·98–6·66)	-40·8% (-79·2 to 69·9%)
		Saudi Arabia	0·9% (0·2–1·9%)	-42·2% (-67 to -18·2%)	2·04 (0·31–4·94)	-43·1% (-78·4 to 26·9%)
		Syrian Arab Republic	1·5% (0·8–2·3%)	-42% (-60·2 to -22·7%)	1·57 (0·65–2·92)	-52·2% (-79·7 to 11%)
		Tunisia	6·5% (4·3–9%)	89·8% (45·9 to 153·5%)	7·65 (3·66–14·02)	37·5% (-33·7 to 202·8%)
		Türkiye	4·6% (2·9–6·4%)	45% (5·9 to 110·1%)	9·8 (4·23–18·05)	1·5% (-52·2 to 125·2%)
		United Arab Emirates	4·2% (2·3–6·5%)	-44·3% (-66·7 to -22·4%)	8·98 (3·52–17·33)	-60·4% (-84 to -12·8%)
		Yemen	1·5% (0·9–2·3%)	-45·2% (-65·5 to -21·1%)	2·23 (0·97–4·09)	-60·1% (-81·7 to -15·8%)
		Afghanistan	0·3% (0·1–0·6%)		0·7 (0·21–1·49)	
		Sudan	0·1% (0–0·2%)	-94·4% (-98·2 to -88·1%)	0·15 (0·04–0·39)	-96·3% (-99 to -91·1%)
	Multiple sclerosis	Global	13·7% (10·4–17·2%)	-25·6% (-30·1 to -19%)	1·92 (1·41–2·54)	-35·4% (-41·4 to -28·2%)
		North Africa and Middle East	10·8% (7·8–14·5%)	-12% (-28·2 to 5%)	2·15 (1·51–2·96)	-6·7% (-28·5 to 8·8%)
		Algeria	9·3% (6·6–12·1%)	-13% (-31·7 to 11·3%)	1·86 (1·19–2·65)	-1·8% (-30·6 to 27·5%)
		Bahrain	10·7% (7·7–13·9%)	-15·3% (-28 to -1·2%)	1·33 (0·85–1·95)	-4·7% (-30 to 22·4%)
		Egypt	10·3% (3·6–17·8%)	24·9% (-45·6 to 145·4%)	1·51 (0·82–3·25)	30·4% (-7 to 67·5%)
		Iran	9·8% (7·2–13·5%)	-6·6% (-22·4 to 14·2%)	2·89 (1·89–4·69)	-6·3% (-34·1 to 15%)
		Iraq	12·1% (8·9–16·1%)	-18·9% (-32·4 to -2·3%)	1·74 (1·13–2·56)	-8·9% (-32·6 to 16·9%)
		Jordan	15·8% (12·1–19·5%)	-3·2% (-18·5 to 16·1%)	3 (2·02–4·11)	-9·9% (-29·5 to 16·2%)
		Kuwait	11·2% (7·8–14·7%)	-25·4% (-36·7 to -11·7%)	1·94 (1·24–2·79)	3% (-15·4 to 26%)
		Lebanon	21·4% (16·2–26·9%)	21·7% (4·7 to 41·8%)	5·02 (3·35–7)	47% (13·9 to 92·5%)
		Libya	10·8% (7·3–14·3%)	-9·5% (-31·6 to 13·1%)	2·34 (1·48–3·23)	20·7% (-13·8 to 67%)
		Morocco	6·8% (4·9–8·8%)	-27·9% (-40·8 to -9·7%)	1·36 (0·87–1·89)	-9·9% (-36·8 to 23·1%)
		Palestine	13% (9·6–17·7%)	-5·3% (-24·9 to 23·5%)	2·54 (1·64–3·81)	2·9% (-34·2 to 43%)
		Oman	7·9% (5·6–10·6%)	-16·9% (-35·2 to 7·2%)	1·34 (0·82–1·93)	-0·3% (-40 to 37·9%)
		Qatar	9·8% (7–12·7%)	0·7% (-16·8 to 21·9%)	1·64 (1·03–2·38)	34·6% (-2·4 to 80·1%)
		Saudi Arabia	8·9% (6·4–11·9%)	27·3% (1·9 to 61·3%)	1·04 (0·66–1·52)	56·6% (9·9 to 129·6%)
		Syrian Arab Republic	11·4% (8·4–14·4%)	-19·7% (-34·7 to -3·7%)	1·72 (1·15–2·47)	-7·3% (-31·4 to 18·8%)
		Tunisia	11·1% (8·1–14·1%)	-11·3% (-26·6 to 11·1%)	2·46 (1·6–3·44)	11·5% (-15·6 to 43·6%)
		Türkiye	15·5% (10·5–19·8%)	-14·4% (-35·8 to 19·7%)	3·24 (2·26–4·44)	-24·4% (-44·7 to -3·9%)
		United Arab Emirates	11·2% (7·7–15·2%)	-9·7% (-27·1 to 9·9%)	2·31 (1·23–3·95)	7·7% (-31·6 to 58·1%)
		Yemen	14·3% (10·2–18·6%)	-7·4% (-23·3 to 13·2%)	1·99 (1·25–2·86)	16·6% (-22·8 to 68%)
		Afghanistan	6·2% (3·9–9·2%)	62·7% (27·1 to 111·4%)	1·66 (0·95–2·79)	81·4% (26·4 to 153·6%)
		Sudan	8·6% (5·6–12%)	-12·1% (-31·4 to 18%)	1·2 (0·68–1·86)	5·8% (-31·7 to 50·8%)
Environmental /occupational risk factors§	Meningitis	Global	2% (1·7–2·2%)	7·5% (-7·8 to 24·1%)	4·62 (3·65–5·97)	-53·9% (-63·3 to -41·2%)
		North Africa and Middle East	1·6% (1·2–2·2%)	-10·2% (-39·3 to 27·6%)	1·11 (0·76–1·62)	-78·3% (-86·3 to -65·8%)
		Algeria	0·8% (0·3–1·5%)	10·5% (-67·9 to 390·3%)	0·3 (0·1–0·61)	-78% (-94 to -3·9%)
		Bahrain	0·4% (0·2–0·7%)	-56·1% (-82 to 7·8%)	0·07 (0·04–0·12)	-88·8% (-95·5 to -71·5%)
		Egypt	2·2% (1–4·1%)	-33·8% (-73 to 42%)	1·21 (0·49–2·46)	-83·7% (-94·2 to -63·3%)
		Iran	0·8% (0·6–1%)	-29·5% (-51·9 to -2·2%)	0·3 (0·2–0·41)	-85·4% (-91·3 to -76·8%)
		Iraq	1·6% (0·9–2·7%)	-26·9% (-65·7 to 58·5%)	1·09 (0·56–1·86)	-85·4% (-94 to -65·4%)
		Jordan	0·8% (0·3–1·5%)	-7·9% (-67·5 to 198·9%)	0·42 (0·17–0·85)	-59·9% (-87·5 to 36·2%)
		Kuwait	1·4% (0·8–2·3%)	2·1% (-49·9 to 94·2%)	0·29 (0·14–0·48)	-62·2% (-81·8 to -26·4%)
		Lebanon	0·4% (0·1–1%)	-53·1% (-89·1 to 97·6%)	0·13 (0·04–0·31)	-85·2% (-96·5 to -37·5%)
		Libya	0·4% (0·2–0·7%)	-70·3% (-89 to -20·9%)	0·14 (0·06–0·28)	-91% (-96·9 to -75·8%)
		Morocco	1·6% (0·7–3·1%)	-20·2% (-73·3 to 140·4%)	1·13 (0·44–2·29)	-85·6% (-95·6 to -54·2%)
		Palestine	0·6% (0·2–1·1%)	-33·9% (-77·3 to 102%)	0·24 (0·08–0·46)	-88·4% (-96 to -66%)
		Oman	0·8% (0·3–1·6%)	-40% (-77·3 to 36·6%)	0·2 (0·07–0·41)	-84·3% (-94·5 to -60·3%)
		Qatar	0·8% (0·5–1·4%)	-44·1% (-73·9 to 13·7%)	0·21 (0·12–0·35)	-85% (-93·1 to -67·7%)

Table S15 Burden of neurological conditions attributable to risk factors in North Africa and Middle East countries

Risk factors	Causes	Locations	DALYs (95% UI) PAF (%) 2019	PAF change (%) From 1990 to 2019	Rate per 100,000 2019	Rate change (%) From 1990 to 2019
		Saudi Arabia	0·1% (0–0·2%)	-62·6% (-87·4 to -0·4%)	0·03 (0·01–0·06)	-90% (-96·4 to -74·6%)
		Syrian Arab Republic	0·4% (0·1–0·7%)	-55·2% (-87·3 to 39·7%)	0·37 (0·12–0·74)	-86·9% (-96·5 to -57·2%)
		Tunisia	0·9% (0·3–1·9%)	-1·9% (-72·9 to 223·1%)	0·36 (0·1–0·75)	-77·4% (-94·5 to -17·8%)
		Türkiye	0·2% (0·1–0·5%)	-39% (-80·5 to 95·9%)	0·06 (0·02–0·13)	-93·8% (-98·2 to -78·9%)
		United Arab Emirates	0·3% (0·1–0·7%)	-71·4% (-90·8 to -25·8%)	0·08 (0·03–0·22)	-92·7% (-97·6 to -80·6%)
		Yemen	1·1% (0·6–1·8%)	-33·8% (-69 to 36·5%)	1·07 (0·56–1·82)	-79·2% (-90·3 to -57·6%)
		Afghanistan	1·6% (0·8–2·8%)	-25·6% (-65·9 to 81·2%)	3·88 (1·8–6·96)	-80·3% (-91·4 to -52·2%)
		Sudan	1·2% (0·6–2·2%)	-34·3% (-70·4 to 43·6%)	1·19 (0·51–2·23)	-90·2% (-95·6 to -79·7%)
	Encephalitis	Global	0·6% (0·5–0·7%)	31·5% (-7·9 to 79·7%)	0·39 (0·3–0·51)	-39·8% (-60 to -6·3%)
		North Africa and Middle East	0·8% (0·5–1·4%)	8·3% (-36·9 to 85·9%)	0·35 (0·21–0·59)	-14·9% (-54·3 to 56·2%)
		Algeria	0·4% (0·1–1%)	50·1% (-63·6 to 590·4%)	0·08 (0·02–0·18)	6% (-74·1 to 359·5%)
		Bahrain	0·2% (0·1–0·4%)	-36·6% (-76·2 to 71·1%)	0·03 (0·01–0·05)	-52·7% (-83·4 to 28·6%)
		Egypt	1·6% (0·5–3·3%)	3·3% (-60·5 to 151%)	1·03 (0·18–2·39)	-29·8% (-76·3 to 74%)
		Iran	0·6% (0·2–0·9%)	14·7% (-26·7 to 63%)	0·1 (0·03–0·15)	-9·6% (-51·9 to 45%)
		Iraq	0·6% (0·3–1·2%)	-2·6% (-61·1 to 146·3%)	0·46 (0·16–0·91)	-35·3% (-76·1 to 67·1%)
		Jordan	0·2% (0·1–0·4%)	-31·2% (-80·4 to 164·7%)	0·02 (0·01–0·04)	-73% (-93·5 to 37·6%)
		Kuwait	0·5% (0·3–0·8%)	20·4% (-47·9 to 150%)	0·06 (0·03–0·09)	-11·4% (-61·6 to 92%)
		Lebanon	0·4% (0·1–0·9%)	-6·4% (-80·4 to 380·2%)	0·06 (0·01–0·15)	-23·6% (-84·9 to 286·8%)
		Libya	0·4% (0·1–0·9%)	-30·9% (-79·1 to 109·7%)	0·07 (0·02–0·14)	-37·5% (-83·3 to 82·4%)
		Morocco	0·9% (0·3–2·1%)	12·5% (-63·5 to 260·1%)	0·22 (0·08–0·47)	-7·1% (-70·9 to 190%)
		Palestine	0·3% (0·1–0·6%)	-15·7% (-77·4 to 179·1%)	0·05 (0·01–0·1)	-34·1% (-83·5 to 126·1%)
		Oman	0·4% (0·2–0·8%)	-8·9% (-70·4 to 177·8%)	0·46 (0·17–0·96)	-49·1% (-85·2 to 57%)
		Qatar	0·4% (0·2–0·7%)	-17·4% (-64·3 to 100·6%)	0·05 (0·02–0·1)	-41·4% (-77 to 37%)
		Saudi Arabia	0·1% (0–0·5%)	-0·6% (-77·4 to 172·4%)	0·04 (0·01–0·15)	-49·5% (-89·5 to 45·4%)
		Syrian Arab Republic	0·2% (0·1–0·4%)	-28·9% (-77·2 to 113·1%)	0·06 (0·02–0·12)	-57·7% (-88·5 to 38·5%)
		Tunisia	0·4% (0·1–1%)	7·3% (-71·6 to 289·8%)	0·07 (0·02–0·17)	-24·2% (-81·6 to 188·9%)
		Türkiye	0·1% (0–0·3%)	-21·5% (-81·4 to 177·3%)	0·02 (0·01–0·05)	-58·4% (-91·2 to 62·5%)
		United Arab Emirates	0·4% (0·1–0·9%)	-15·1% (-75·2 to 156·9%)	0·07 (0·02–0·15)	-32·4% (-81·4 to 130·9%)
		Yemen	0·5% (0·2–1%)	-20·2% (-70·1 to 116·5%)	0·15 (0·07–0·29)	-25·9% (-71 to 86·7%)
		Afghanistan	0·3% (0·1–0·7%)	-12·3% (-63·7 to 116·7%)	0·56 (0·21–1·51)	-25·9% (-70·4 to 85·9%)
		Sudan	0·5% (0·2–1·1%)	-21% (-71·9 to 121·6%)	0·15 (0·07–0·29)	-39·4% (-77 to 76·3%)
	Stroke	Global	37·3% (34·4–40·3%)	-8·2% (-12·3 to -4·2%)	658·89 (586·74–729·11)	-40·6% (-46·5 to -34·5%)
		North Africa and Middle East	37·5% (34·5–40·7%)	-2·2% (-6·9 to 3·9%)	685·01 (590·56–792·82)	-33·5% (-41·7 to -22·2%)
		Algeria	30·8% (24·7–36·6%)	-1·6% (-7·5 to 3·4%)	540·94 (400·88–699·41)	-45·5% (-56·7 to -31·7%)
		Bahrain	37·8% (33–42·2%)	-4·5% (-11·1 to 2·4%)	355·66 (283·74–453·8)	-54·6% (-63·3 to -43·1%)
		Egypt	41% (35·7–45·5%)	26·7% (8 to 42·9%)	878·28 (641·37–1202·41)	-18% (-37·5 to 6·1%)
		Iran	36·6% (33·5–40%)	-4·8% (-7·4 to -2·4%)	461·69 (408·72–516·02)	-48·3% (-53·5 to -40·7%)
		Iraq	38·7% (33·3–43·8%)	-6·9% (-11·5 to -3%)	1132·07 (874·59–1418·34)	-24·9% (-41·3 to -5·6%)
		Jordan	29% (24·8–33·3%)	-1·3% (-5·3 to 3·5%)	419·83 (330·26–512·86)	-50·9% (-59·7 to -40·9%)
		Kuwait	40·7% (35·9–45·4%)	0·6% (-2·4 to 3·9%)	382·13 (311·41–464·04)	-11·2% (-23·9 to 5·1%)
		Lebanon	29·2% (24·5–33·6%)	-5·2% (-10·9 to 0·3%)	220·17 (158·48–284·52)	-37·1% (-50·9 to -19·8%)
		Libya	32·3% (25·3–38·7%)	1·4% (-10·1 to 12·3%)	507·76 (354·43–695·05)	-20·2% (-38·7 to 5·5%)
		Morocco	34% (29·5–38·5%)	-2·3% (-9·6 to 4·6%)	767·32 (587·72–965·54)	-19·1% (-37·3 to 1·3%)
		Palestine	29·7% (23·7–35·4%)	-10·4% (-17·9 to -4·9%)	631·94 (486·13–784·98)	-39·5% (-53·3 to -22·3%)
		Oman	35·2% (27·5–42·2%)	-11·8% (-21·4 to -1·8%)	662·78 (509·66–843·85)	-46% (-59·3 to -25·6%)
		Qatar	40·7% (34·8–45·5%)	-4·4% (-9·2 to 3·9%)	368·3 (286·92–481·18)	-47·1% (-59·7 to -31·1%)
		Saudi Arabia	42·3% (37·6–46·5%)	-0·2% (-6·2 to 6·5%)	895·33 (692·05–1088·55)	-29·2% (-47·8 to -3·3%)
		Syrian Arab Republic	32·1% (27·4–36·6%)	-2·4% (-8·7 to 4·4%)	648·78 (480·88–855·08)	-41·6% (-57·1 to -19·5%)
		Tunisia	30·5% (24·6–36·3%)	-4·7% (-11 to 0·2%)	450·15 (323·15–608·26)	-28·3% (-46·1 to -4·5%)

Table S15 Burden of neurological conditions attributable to risk factors in North Africa and Middle East countries

Risk factors	Causes	Locations	DALYs (95% UI) PAF (%) 2019	PAF change (%) From 1990 to 2019	Rate per 100,000 2019	Rate change (%) From 1990 to 2019
		Türkiye	29·2% (25·5–33%)	-10·4% (-15·5 to -5·4%)	339·71 (267·68–424·6)	-31·5% (-50·3 to -12·7%)
		United Arab Emirates	37·5% (29–45·1%)	0·1% (-6·8 to 14·9%)	723·7 (511·43–1004·27)	-45·9% (-60·1 to -27·4%)
		Yemen	44% (37·8–49·8%)	-15·1% (-26·9 to -5·1%)	1217·98 (915·89–1584·47)	-36% (-52·6 to -13·2%)
		Afghanistan	53·8% (50·1–57·6%)	-8% (-15·2 to -2·2%)	1883·75 (1350·17–2440·09)	-28·1% (-45·5 to -8·6%)
		Sudan	45·4% (39·9–49·7%)	-11·6% (-21·8 to -1·3%)	1174·37 (841·06–1661·13)	-40·8% (-54 to -23·4%)
	Ischaemic stroke	Global	33·1% (30·3–36·2%)	-2·4% (-8·3 to 3·4%)	264·37 (234·11–297·28)	-30·2% (-38·4 to -22·5%)
		North Africa and Middle East	36·4% (33·4–39·7%)	-2·3% (-6·4 to 1·9%)	430·66 (370·68–494·55)	-10·9% (-24 to 2·5%)
		Algeria	29·7% (23·8–35·5%)	1·7% (-3·9 to 7·3%)	378·62 (280·54–494·32)	-23·4% (-39·1 to -4·1%)
		Bahrain	36·1% (31·3–40·4%)	-4·3% (-10·8 to 3%)	221·66 (178·08–283·43)	-46·3% (-56·3 to -33·8%)
		Egypt	42·1% (36·7–46·6%)	14·7% (4·1 to 25·7%)	581·57 (429·89–792·27)	30·6% (-3·9 to 65·8%)
		Iran	35·9% (32·7–39·4%)	-4·7% (-7·2 to -2·5%)	353·43 (309·23–397·35)	-45·4% (-51·1 to -37·7%)
		Iraq	37·1% (31·9–42·1%)	-7·2% (-11·5 to -3·5%)	678·24 (531·62–835·76)	-13·7% (-31·8 to 7%)
		Jordan	28% (23·9–32·2%)	-0·4% (-4·4 to 4·6%)	303·86 (239·73–371·15)	-46·5% (-56·3 to -36%)
		Kuwait	39·4% (34·7–44%)	-0·4% (-3·5 to 3%)	254·53 (206·75–311·34)	-12·3% (-24·8 to 4·1%)
		Lebanon	28·5% (23·9–32·7%)	-2% (-8·1 to 3·5%)	170·38 (123·32–218·36)	-16·7% (-36 to 4%)
		Libya	31·9% (24·9–38·4%)	-1·2% (-10·9 to 6·5%)	358·62 (248·43–496·95)	9·9% (-14·6 to 43%)
		Morocco	32·9% (28·4–37·5%)	0·5% (-7·1 to 7·5%)	528·55 (410·17–669·84)	19% (-9·6 to 47·8%)
		Palestine	28·9% (23–34·5%)	-9·7% (-17·3 to -4·2%)	459·4 (352·19–572·97)	-25·8% (-42·2 to -5·6%)
		Oman	34·6% (27–41·8%)	-11·9% (-21 to -1·7%)	447 (336·09–565·09)	-28·1% (-45·6 to -1·1%)
		Qatar	38·7% (33·3–43·6%)	-4·6% (-9·3 to 3·4%)	223·47 (175·4–292·25)	-38·4% (-52·5 to -21·2%)
		Saudi Arabia	40·3% (35·9–44·5%)	1% (-5·4 to 8·1%)	524·19 (403·52–632·73)	-16·6% (-37·7 to 9·1%)
		Syrian Arab Republic	31·1% (26·4–35·6%)	-4·1% (-9·7 to 2·2%)	333·04 (251·19–436·24)	-28·1% (-47·4 to -2·4%)
		Tunisia	29·5% (23·8–35·2%)	-2·1% (-7·9 to 2·3%)	321·4 (228·49–434·06)	1·8% (-25·2 to 34·1%)
		Türkiye	27·3% (23·8–31%)	-10·3% (-15·5 to -5·4%)	181·28 (143·75–226·3)	-17·5% (-41·4 to 5·3%)
		United Arab Emirates	36·3% (28–43·6%)	1·3% (-6·1 to 17·1%)	514·43 (364·95–692·57)	-33% (-50 to -10·3%)
		Yemen	42·4% (36·6–48·1%)	-14% (-26·9 to -2·9%)	728·59 (552·57–980·97)	2·9% (-23·8 to 37·6%)
		Afghanistan	51·5% (47·4–55·6%)	-6·7% (-15·9 to 0·5%)	961·15 (701·67–1279·22)	18·7% (-9·6 to 51·7%)
		Sudan	44·3% (38·8–48·6%)	-10·5% (-22·7 to 0%)	750·75 (551·67–1085·17)	-3·4% (-25·9 to 26·4%)
	Intracerebral haemorrhage	Global	41·3% (38·3–44·5%)	-9·1% (-13·2 to -5·4%)	344·03 (304·68–381·87)	-42·5% (-49·7 to -35·9%)
		North Africa and Middle East	40% (36·9–43·3%)	-2% (-8·2 to 7·1%)	219·39 (184·56–260·22)	-52·7% (-60·4 to -41·9%)
		Algeria	33·7% (27·3–40·1%)	-0·3% (-6·3 to 5·3%)	136·23 (98·53–180·02)	-68% (-76 to -57·3%)
		Bahrain	41·1% (36·1–45·9%)	-4·5% (-10·8 to 2·7%)	108·74 (84·65–139·91)	-65·8% (-73·8 to -54·6%)
		Egypt	40·2% (34·3–45·9%)	21·8% (5·1 to 47·6%)	258·96 (180·64–384·31)	-50·6% (-65 to -30·8%)
		Iran	38·9% (35·5–42·4%)	-4·5% (-7·6 to -0·5%)	86·91 (77·86–96·47)	-55·7% (-62·1 to -48·3%)
		Iraq	41·4% (35·7–46·9%)	-5·2% (-9·9 to -0·5%)	424·02 (314·38–553·55)	-33·6% (-51·1 to -11·5%)
		Jordan	31·7% (27·1–36·1%)	-1·1% (-4·7 to 3·8%)	103·26 (80·28–128·46)	-60·4% (-68·3 to -50·3%)
		Kuwait	43·6% (38·4–48·6%)	1·4% (-2 to 5·3%)	107·36 (83·54–134·6)	-2·9% (-22·9 to 21·2%)
		Lebanon	32% (26·8–36·7%)	-5·2% (-11·3 to 0·7%)	41·16 (28·88–58·74)	-65·8% (-75·6 to -52·4%)
		Libya	33·8% (26·3–40·3%)	0·5% (-11 to 14·6%)	127·31 (84·43–180·08)	-50·9% (-65 to -30·8%)
		Morocco	36·5% (31·8–41·3%)	-1·4% (-8·9 to 6%)	202·69 (148·84–264·84)	-51·7% (-65·1 to -33·1%)
		Palestine	32·1% (25·7–38·1%)	-8·2% (-15·8 to -2·8%)	156·28 (118·55–196·98)	-60·3% (-70 to -46·9%)
		Oman	36·8% (28·7–44·4%)	-11·7% (-21·1 to -0·6%)	185·54 (138·91–244·68)	-64·6% (-73·6 to -49·8%)
		Qatar	43·6% (37·3–48·6%)	-2·9% (-8·1 to 5·4%)	106·19 (80·18–142·64)	-57·6% (-69·5 to -41·4%)
		Saudi Arabia	45·5% (40·6–49·9%)	0·7% (-5·5 to 6·9%)	348·16 (267·27–438·13)	-40·9% (-58·1 to -12·8%)
		Syrian Arab Republic	34·5% (29·5–39·3%)	-4·1% (-9·9 to 2·5%)	289·52 (209·67–388·95)	-50·6% (-64·3 to -29·6%)
		Tunisia	33·3% (26·9–39·5%)	-3·2% (-9·6 to 2·6%)	107·27 (73·67–149·52)	-57·9% (-70·6 to -38·2%)
		Türkiye	31·4% (27·4–35·3%)	-8·6% (-14 to -2·9%)	125·36 (96·27–159)	-42·3% (-59·5 to -24·6%)

Table S15 Burden of neurological conditions attributable to risk factors in North Africa and Middle East countries

			DALYs (95% UI)			
			PAF (%)	PAF change (%)	Rate per 100,000	Rate change (%)
Risk factors	Causes	Locations	2019	From 1990 to 2019	2019	From 1990 to 2019
		United Arab Emirates	41% (31·8–49%)	1·5% (-5·7 to 16·2%)	184·69 (120·42–289·87)	-63·5% (-75·5 to -46%)
		Yemen	46·8% (40·4–52·7%)	-13·1% (-24·5 to -3·3%)	414·43 (298·52–567·27)	-59·8% (-71·3 to -42·6%)
		Afghanistan	56·7% (52·9–60·6%)	-6·1% (-12·8 to -0·8%)	792·08 (552·74–1058·88)	-49·3% (-62·3 to -32·2%)
		Sudan	47·6% (42·1–52·4%)	-10·5% (-20·7 to 1·4%)	367·12 (242·27–520·5)	-64·8% (-74·1 to -51·6%)
	Subarachnoid haemorrhage	Global	37% (33·4–40·5%)	-16·2% (-20·8 to -11·4%)	50·49 (41·79–60·03)	-61·7% (-67·8 to -45·5%)
		North Africa and Middle East	37·1% (33·5–40·8%)	12·8% (0·2 to 36·4%)	34·96 (27·76–44·83)	-58·2% (-69·6 to -33·8%)
		Algeria	33·9% (27·5–40·6%)	2·8% (-4·6 to 11·9%)	26·1 (17·87–35·85)	-63·7% (-74·9 to -41·9%)
		Bahrain	40·2% (35·4–45·1%)	11·9% (0·3 to 28·3%)	25·26 (18·55–36·27)	-53·1% (-68 to -31·7%)
		Egypt	33% (25·4–41·7%)	60·6% (16·7 to 158·7%)	37·75 (25·22–54·94)	-63·1% (-77·4 to -33·2%)
		Iran	39·1% (35·6–42·6%)	-2·2% (-6·4 to 1·9%)	21·35 (18·3–25·29)	-56·7% (-68·9 to -33·8%)
		Iraq	41·3% (35·7–46·8%)	-0·7% (-7·8 to 7·8%)	29·81 (21·17–41·96)	-64·3% (-77·2 to -41·6%)
		Jordan	32·7% (28·2–37·1%)	-0·6% (-5·2 to 5·1%)	12·71 (9·52–16·13)	-50·1% (-64·2 to -27·2%)
		Kuwait	43·1% (38·4–47·7%)	6·1% (1 to 13·7%)	20·24 (16·19–25·05)	-30·9% (-44·6 to -5·3%)
		Lebanon	32·3% (26·9–37·4%)	-2·7% (-10·1 to 4·9%)	8·63 (5·77–12·27)	-65·3% (-79·7 to -42·3%)
		Libya	30·4% (23·5–37·3%)	28·2% (-1·2 to 74%)	21·83 (14·11–33·17)	-56·9% (-70·3 to -31·8%)
		Morocco	35·9% (31·2–40·9%)	2·7% (-7·1 to 14·1%)	36·08 (24·21–54·22)	-57·5% (-72·8 to -26·6%)
		Palestine	32·5% (26·1–38·1%)	-8·1% (-15·7 to -2%)	16·26 (12·24–21·14)	-50·4% (-65·5 to -27·1%)
		Oman	33·8% (26·2–42·1%)	-0·6% (-15·9 to 22·8%)	30·24 (18·1–50·11)	-62·6% (-77·8 to -27·6%)
		Qatar	46·2% (40·1–51·7%)	1·4% (-5·3 to 10·6%)	38·64 (27·78–51·75)	-53·6% (-72·6 to -24·5%)
		Saudi Arabia	46·4% (41·5–51·1%)	2·3% (-4·8 to 10·5%)	22·98 (16·32–30·7)	-51·4% (-72·2 to -18·4%)
		Syrian Arab Republic	24% (19·6–28·8%)	23·2% (1·2 to 52·8%)	26·21 (18·62–35·78)	-57·6% (-71·8 to -33·6%)
		Tunisia	33·3% (27–39·6%)	1% (-7·9 to 11·9%)	21·48 (14·3–30·37)	-62·4% (-77·2 to -35·4%)
		Türkiye	32·8% (28·6–36·9%)	-6·7% (-12·7 to -1%)	33·07 (24·74–42·34)	-43·9% (-64·9 to -3·1%)
		United Arab Emirates	40·4% (31·7–48·3%)	10·2% (-1·3 to 37·2%)	24·58 (12·51–40·77)	-60·7% (-76·1 to -32·7%)
		Yemen	45·5% (38·9–51·9%)	-10·7% (-23·1 to 1·2%)	74·96 (38·58–124·57)	-54·5% (-69·2 to -26·9%)
		Afghanistan	55·6% (51·4–60·1%)	-5·5% (-14·1 to 1·7%)	130·52 (41·71–225·63)	-46·9% (-63·1 to -17%)
		Sudan	45·9% (40·3–51%)	-5·5% (-19·1 to 13·7%)	56·49 (31·52–97·62)	-66% (-78·2 to -36·2%)
Metabolic risk factors†	Stroke	Global	70·3% (63·8–76·5%)	8·4% (5·4 to 12%)	1242·98 (1093·17–1376·96)	-29·8% (-35·7 to -23·9%)
		North Africa and Middle East	73·9% (67·6–80·1%)	18·3% (12·1 to 26%)	1349·66 (1156·04–1552·32)	-19·6% (-29·1 to -6·3%)
		Algeria	73·3% (65·4–82·2%)	8·7% (2·1 to 17·5%)	1287·26 (1028·15–1590)	-39·7% (-52·4 to -24·6%)
		Bahrain	75·3% (67·7–83·1%)	2·6% (-2·6 to 8·1%)	709·2 (584–883·92)	-51·3% (-60·6 to -40·6%)
		Egypt	70·8% (63·6–77·6%)	62·6% (36·3 to 89·9%)	1516·31 (1124·22–2070·77)	5·2% (-22 to 37·8%)
		Iran	72·3% (64·4–80·5%)	11% (7·1 to 16·7%)	912·16 (796·49–1035·61)	-39·7% (-46·2 to -30·1%)
		Iraq	80·4% (74·4–85·4%)	8·8% (4 to 14·8%)	2349·44 (1872·37–2819·46)	-12·3% (-31 to 9·5%)
		Jordan	76·3% (69–84·1%)	5·2% (0·1 to 11·7%)	1104·15 (914·04–1305·62)	-47·6% (-56·9 to -37·3%)
		Kuwait	75·8% (68·9–82·5%)	3·1% (-0·9 to 7·5%)	711·1 (587·48–850·4)	-8·9% (-22·2 to 7%)
		Lebanon	75·9% (68·2–84·5%)	12·7% (5·7 to 21·1%)	571·61 (422·99–723·84)	-25·3% (-41·5 to -6%)
		Libya	77·2% (70·3–83·6%)	19·6% (9·2 to 32·5%)	1211·99 (935·48–1569·05)	-5·9% (-25·8 to 22·1%)
		Morocco	79·2% (72·4–85·7%)	8·8% (2·8 to 15·6%)	1788·38 (1418·9–2171·68)	-9·9% (-28·4 to 11·7%)
		Palestine	72·4% (64·4–82%)	7·4% (1 to 14·7%)	1541·39 (1292·41–1842·47)	-27·5% (-43·2 to -8·1%)
		Oman	74·9% (67·3–82·9%)	28·7% (17·4 to 41·7%)	1410·95 (1186·57–1652·53)	-21·1% (-38·9 to 6·1%)
		Qatar	74·2% (66·2–81·9%)	2% (-3·3 to 7·4%)	671·12 (536·45–855·68)	-43·6% (-56·7 to -27·6%)
		Saudi Arabia	77·8% (71·5–83·8%)	11·9% (6·2 to 19·3%)	1645·92 (1293·63–1954·58)	-20·7% (-40·7 to 6·7%)

Table S15 Burden of neurological conditions attributable to risk factors in North Africa and Middle East countries

			DALYs (95% UI)			
			PAF (%)	PAF change (%)	Rate per 100,000	Rate change (%)
Risk factors	Causes	Locations	2019	From 1990 to 2019	2019	From 1990 to 2019
		Syrian Arab Republic	72·5% (66–78·9%)	17·2% (8·7 to 27·7%)	1464·84 (1126·86–1894·86)	-29·9% (-47·9 to -2·6%)
		Tunisia	73·8% (65·7–82·3%)	12·4% (5·5 to 20·8%)	1091·13 (811·65–1428·02)	-15·4% (-38·1 to 11·9%)
		Türkiye	72·8% (65·6–79·5%)	7·6% (2·3 to 14·1%)	846·72 (678·85–1036·91)	-17·8% (-39·2 to 2·7%)
		United Arab Emirates	78% (71·2–84·5%)	0·2% (-3·4 to 4·1%)	1503·11 (1159·12–1956·04)	-45·9% (-59 to -30·1%)
		Yemen	68·2% (60·9–75·9%)	15·3% (4·9 to 27·4%)	1888·2 (1417·64–2465·59)	-13·1% (-33·4 to 16·9%)
		Afghanistan	72·9% (65·5–79·7%)	12·2% (4 to 23·3%)	2551·6 (1806·91–3399·42)	-12·2% (-32·7 to 12·5%)
		Sudan	78·3% (71·8–84·3%)	17·6% (9·5 to 29·2%)	2024·05 (1510·29–2822·94)	-21·3% (-37·1 to -0·5%)
	Ischaemic stroke	Global	70·4% (61·2–79·5%)	2·9% (0·2 to 6·1%)	561·99 (478·27–646·59)	-26·4% (-32·6 to -20·2%)
		North Africa and Middle East	75·1% (66·7–84·3%)	12·7% (8 to 18·8%)	888·84 (753·9–1040·54)	2·8% (-11·2 to 18%)
		Algeria	73·4% (63·5–85·2%)	8·7% (1·5 to 18·1%)	936·37 (732·58–1185·69)	-18·1% (-35 to 2·2%)
		Bahrain	74·9% (64·6–86·1%)	0·7% (-4·9 to 6·4%)	459·76 (367·65–584·07)	-43·5% (-54 to -32%)
		Egypt	74·6% (66·7–83·1%)	36·2% (21·7 to 56·1%)	1031·9 (775·36–1392·69)	55·3% (14·6 to 100·9%)
		Iran	72·9% (63·7–83·4%)	9·2% (5·4 to 16%)	717·37 (611·56–838·33)	-37·5% (-44·7 to -27·8%)
		Iraq	80% (72·1–87·7%)	7·6% (2·9 to 13·6%)	1464·3 (1166·94–1744·37)	0·1% (-20·1 to 24%)
		Jordan	76·1% (66·8–86%)	4·8% (-0·3 to 11%)	824·1 (669·81–982·21)	-43·7% (-54·1 to -33·1%)
		Kuwait	76·1% (67·2–85·4%)	1·5% (-2·8 to 5·8%)	491·38 (396·75–587·54)	-10·7% (-23·9 to 4·4%)
		Lebanon	75·8% (66·5–86·6%)	11·6% (4·7 to 21·1%)	453·35 (337·91–572·53)	-5·3% (-27 to 18·9%)
		Libya	78·5% (70·3–87%)	11·6% (5·4 to 20·6%)	881·06 (670·1–1145·34)	24% (-3·1 to 59·9%)
		Morocco	79·3% (70·6–88·3%)	8·2% (2·5 to 15·6%)	1272·24 (1005·64–1555·81)	28·2% (-0·4 to 57·7%)
		Palestine	72·5% (62·7–85·5%)	6·3% (0 to 13·7%)	1153·85 (947·95–1418·5)	-12·6% (-30·3 to 10·4%)
		Oman	75·6% (65·8–87·2%)	23·5% (13 to 36·3%)	976·99 (791·75–1175·46)	0·9% (-21·6 to 35·6%)
		Qatar	73·4% (63–84·8%)	-0·1% (-5·9 to 6·1%)	423·7 (331·27–545·4)	-35·5% (-50·1 to -18·8%)
		Saudi Arabia	77% (68·8–85·5%)	12·3% (5·4 to 20·8%)	1001·63 (772–1200·64)	-7·2% (-30·3 to 21·9%)
		Syrian Arab Republic	74·7% (65·6–85·7%)	11% (4·1 to 20·9%)	799·96 (613·77–1033·8)	-16·9% (-38·9 to 11·9%)
		Tunisia	73·8% (64·1–85·4%)	10·3% (4·2 to 18·7%)	804·76 (592·3–1053·88)	14·9% (-14·8 to 50·3%)
		Türkiye	72·1% (62·5–82·5%)	4·4% (-1 to 11·6%)	478·13 (378·61–595·27)	-3·9% (-30·1 to 20·2%)
		United Arab Emirates	77·9% (69·8–86·2%)	-0·1% (-4 to 3·9%)	1102·48 (852·96–1370·25)	-34% (-49 to -15·8%)
		Yemen	69·6% (60·3–79·6%)	13·4% (4·4 to 25·4%)	1195·08 (902·29–1589·46)	35·8% (5·6 to 77·2%)
		Afghanistan	73·9% (64·9–84·1%)	11% (3·5 to 20·1%)	1378·02 (992·69–1835·48)	41·5% (9·4 to 78·5%)
		Sudan	78·8% (70·6–87·6%)	17·2% (9·4 to 27·8%)	1333·9 (987·59–1858·15)	26·6% (2·1 to 59·6%)
	Intracerebral haemorrhage	Global	70·8% (63–77·4%)	11·6% (7·8 to 16·2%)	589·88 (516·96–664·45)	-29·3% (-37·6 to -22·4%)
		North Africa and Middle East	72·9% (66–78·6%)	19% (11·2 to 31·9%)	399·93 (339·15–470·05)	-42·6% (-51·5 to -29·4%)
		Algeria	73·7% (65·9–80·7%)	8·1% (1·5 to 16·4%)	297·48 (228·62–380·62)	-65·3% (-73·8 to -53·7%)
		Bahrain	77·7% (70·2–84·4%)	3·1% (-2·1 to 9·1%)	205·75 (163·36–260·33)	-63·1% (-71·4 to -52·4%)
		Egypt	66·2% (57·8–74·5%)	57·2% (29·1 to 100·5%)	426·46 (293·04–625·41)	-36·1% (-56·1 to -8·2%)
		Iran	70·7% (63·6–77·2%)	16% (10·3 to 24·4%)	157·87 (140·96–174·54)	-46·3% (-54·6 to -35·3%)
		Iraq	81·1% (74·5–86·1%)	9·8% (4·5 to 17·2%)	829·66 (638·32–1055·76)	-23·1% (-43·1 to 2·2%)
		Jordan	77·1% (69·8–83·5%)	6·4% (0·7 to 13·7%)	251·05 (201·82–302·52)	-57·5% (-66 to -47·1%)

Table S15 Burden of neurological conditions attributable to risk factors in North Africa and Middle East countries

			DALYs (95% UI)			
			PAF (%)	PAF change (%)	Rate per 100,000	Rate change (%)
Risk factors	Causes	Locations	2019	From 1990 to 2019	2019	From 1990 to 2019
		Kuwait	75·6% (68·1–81·7%)	5·1% (0·8 to 10·1%)	186·14 (147·2–226·56)	0·6% (-20·7 to 25·1%)
		Lebanon	76·5% (68·9–82·7%)	13·3% (6·6 to 22·6%)	98·28 (71·52–135·54)	-59·2% (-70·5 to -43%)
		Libya	75·6% (69·3–81·6%)	18·2% (6·3 to 36·6%)	284·87 (210·43–393·2)	-42·3% (-57·6 to -19·4%)
		Morocco	79·5% (72·2–85·3%)	8·2% (1·8 to 15·9%)	440·68 (328·7–565·32)	-47% (-61·8 to -26·8%)
		Palestine	72·5% (64·1–79·8%)	9·1% (2·4 to 16·9%)	353·51 (286·81–428·42)	-52·8% (-64·3 to -37%)
		Oman	74·9% (67·9–81·2%)	30·3% (17·8 to 47·1%)	377·96 (317·53–476·95)	-47·8% (-61·3 to -26·3%)
		Qatar	76% (68·3–82·7%)	4·4% (-1·1 to 10·6%)	185·29 (143·08–244·55)	-54·4% (-67·2 to -38·7%)
		Saudi Arabia	79·4% (72–85·2%)	11·8% (5·9 to 19·8%)	607·28 (466·3–753·34)	-34·4% (-53·2 to -2·7%)
		Syrian Arab Republic	73·6% (65·7–80·3%)	14·7% (6·2 to 25·3%)	617·72 (460·69–819·03)	-40·9% (-58·1 to -16·2%)
		Tunisia	74·5% (66·6–81·4%)	13·5% (5·8 to 23%)	240·53 (172·13–323·69)	-50·7% (-65·9 to -27·6%)
		Türkiye	74·3% (66·3–80·8%)	10·9% (4·1 to 19·7%)	296·38 (233·88–366·17)	-30·1% (-50·8 to -9·8%)
		United Arab Emirates	79% (72·1–84·7%)	-0·5% (-4·7 to 4%)	356·01 (248·01–532·88)	-64·3% (-75·6 to -49%)
		Yemen	66·8% (58·1–74·3%)	13·8% (3·7 to 27·7%)	591·25 (422·61–800·17)	-47·3% (-61·8 to -26·2%)
		Afghanistan	72·4% (64–80%)	11·7% (2·8 to 23·2%)	1012·92 (691·22–1384·65)	-39·7% (-55·2 to -19·4%)
		Sudan	78% (70·6–83·6%)	16% (7·2 to 31·4%)	600·91 (408·26–849·53)	-54·4% (-65·9 to -37·6%)
	Subarachnoid haemorrhage	Global	66·8% (58·6–73·9%)	15·2% (9·9 to 21·8%)	91·11 (76·01–107·33)	-47·2% (-54·9 to -29·6%)
		North Africa and Middle East	64·7% (57·3–71·4%)	39·2% (19·4 to 78%)	60·89 (49·02–76·6)	-48·4% (-63·2 to -19·4%)
		Algeria	69·5% (60·7–77·2%)	12·3% (1·9 to 25·4%)	53·41 (39·29–71·65)	-60·3% (-72·5 to -37·4%)
		Bahrain	69·6% (62·6–76·2%)	22·9% (9·5 to 39·8%)	43·69 (32·78–62·56)	-48·5% (-65·5 to -25·5%)
		Egypt	50·6% (38·1–64·2%)	129·2% (53·5 to 336·1%)	57·96 (37·85–85·36)	-47·1% (-69·4 to -2·6%)
		Iran	67·6% (59·8–74·8%)	14·5% (8·3 to 23·3%)	36·92 (31·19–44·28)	-49·3% (-64·1 to -21·7%)
		Iraq	76·9% (69·2–82·7%)	13·6% (4·9 to 25·4%)	55·47 (40·85–77·53)	-59·2% (-73·6 to -33·4%)
		Jordan	74·5% (66·9–81·3%)	7·7% (1·2 to 16·5%)	29 (22·43–35·79)	-45·9% (-60·9 to -21·4%)
		Kuwait	71·5% (63·8–77·7%)	13·8% (6·4 to 23·2%)	33·57 (26·77–40·92)	-25·9% (-41·4 to 0·3%)
		Lebanon	74·8% (66·6–81·6%)	21·7% (11·5 to 36%)	19·97 (13·71–28·13)	-56·6% (-74·5 to -29·1%)
		Libya	64·3% (53·9–74·4%)	62·1% (18·5 to 135·6%)	46·06 (32·29–67·42)	-45·4% (-63 to -14·9%)
		Morocco	75·3% (66·6–82·2%)	13·7% (2·7 to 28·3%)	75·46 (53·05–109·88)	-53·1% (-69·7 to -18·6%)
		Palestine	67·9% (58·8–75·4%)	8·4% (0·3 to 18·5%)	34·04 (26·94–41·72)	-41·5% (-58·9 to -13·4%)
		Oman	62·4% (53·5–72·2%)	53% (27·1 to 90·8%)	55·99 (34·29–87·8)	-42·2% (-65·6 to 6·2%)
		Qatar	74·3% (66·7–81·1%)	8% (0 to 17·2%)	62·13 (45·08–83·92)	-50·6% (-70·8 to -19·8%)
		Saudi Arabia	74·8% (66·1–81·6%)	14·1% (5·6 to 24·1%)	37·01 (26·46–49·99)	-45·8% (-69·2 to -9·4%)
		Syrian Arab Republic	43·1% (35·5–51%)	64·2% (23·1 to 129·8%)	47·17 (34·12–63·64)	-43·4% (-64·3 to -5·6%)
		Tunisia	71% (62·3–78·4%)	21·9% (8·9 to 40·4%)	45·84 (31·89–63·9)	-54·6% (-72·4 to -21·7%)
		Türkiye	71·5% (62·7–78·6%)	11·1% (3·6 to 20·8%)	72·21 (55·09–90·77)	-33·2% (-57·9 to 17·7%)
		United Arab Emirates	73·2% (65·5–80·4%)	12·1% (1·1 to 25·4%)	44·61 (22·65–72·78)	-60·1% (-75·7 to -34·9%)
		Yemen	62% (52·9–70·6%)	18·5% (3·6 to 37·7%)	101·87 (52·51–171·79)	-39·7% (-59·6 to -2·4%)
		Afghanistan	68·4% (58·7–76·8%)	13·4% (1·2 to 28·7%)	160·66 (52·69–279·88)	-36·2% (-56·2 to -0·4%)
		Sudan	72·6% (63·4–80·1%)	23·5% (6·1 to 53·3%)	89·24 (51·25–150·08)	-55·6% (-71·1 to -17%)
	Neurological disorders*	Global	5·7% (1·8–12·7%)	31·8% (22·2 to 47·4%)	70·7 (20·77–179·6)	30·1% (20·9 to 45·9%)
		North Africa and Middle East	8·2% (2·8–17·4%)	55·3% (38·6 to 85%)	111·71 (34·19–279·74)	48·4% (31·4 to 80·4%)
		Algeria	9% (3–19·6%)	68·8% (40·2 to 128·6%)	121·85 (36·44–312·01)	52·9% (27 to 102·6%)
		Bahrain	11·9% (4·2–24·9%)	40·9% (16·9 to 79·6%)	163·81 (51·36–416·76)	28·5% (9·2 to 56%)
		Egypt	7·8% (2·4–16·9%)	53·3% (19·4 to 108·5%)	101·94 (30·77–248·87)	54·1% (20·1 to 113·2%)
		Iran	7·6% (2·6–16·2%)	71·4% (47·4 to 114·5%)	104·1 (33·44–264·68)	63·4% (40·8 to 114·3%)
		Iraq	9·2% (3–20%)	31% (9·8 to 64·3%)	120·55 (35·98–302·46)	28·3% (6·9 to 60·7%)
		Jordan	10·1% (3·4–21·1%)	38·4% (15 to 75·2%)	127·07 (40·58–314·52)	30·8% (10·5 to 64·5%)
		Kuwait	11·7% (4·1–24·2%)	33·4% (11·4 to 67·9%)	148·04 (49·02–360·41)	26·5% (10·7 to 56·2%)
		Lebanon	9·4% (3·2–20%)	46·1% (22·1 to 86·9%)	124·63 (37·96–311·91)	40·3% (18·9 to 92%)
		Libya	10·3% (3·5–21·9%)	41·6% (20·2 to 76%)	140·67 (41·93–351·92)	37·2% (13·7 to 72·3%)
		Morocco	8% (2·6–17·4%)	61·7% (32·4 to 113·8%)	106·29 (30·86–279·11)	65% (37·8 to 115·9%)

Table S15 Burden of neurological conditions attributable to risk factors in North Africa and Middle East countries

Risk factors	Causes	Locations	DALYs (95% UI) PAF (%) 2019	PAF change (%) From 1990 to 2019	Rate per 100,000 2019	Rate change (%) From 1990 to 2019
		Palestine	7·6% (2·3–17·1%)	54·4% (26·8 to 101·7%)	103·49 (29·37–273·02)	45·8% (19·5 to 93·1%)
		Oman	10% (3·5–21·4%)	98·6% (52·9 to 215·4%)	133·87 (39·27–348·07)	100·6% (55·5 to 222·5%)
		Qatar	13·7% (4·8–28·4%)	44·3% (10·2 to 104·9%)	184·71 (58·75–476·59)	41·8% (7·2 to 96·3%)
		Saudi Arabia	10·2% (3·7–21·5%)	58·2% (27·2 to 118·2%)	144·44 (44·81–358·41)	58·3% (28·7 to 123·6%)
		Syrian Arab Republic	8·6% (2·8–18·3%)	40·8% (16·1 to 80·8%)	111·67 (32·27–291·85)	43·3% (15·2 to 92%)
		Tunisia	9·3% (3·2–20%)	51·6% (27·7 to 93·3%)	121·9 (36·09–303·11)	47·8% (20·1 to 99·6%)
		Türkiye	8% (2·8–17%)	48·8% (24·3 to 89·2%)	117·57 (36·08–290·36)	34·5% (13·2 to 67·1%)
		United Arab Emirates	10·3% (3·5–21·8%)	42·5% (10·8 to 93·2%)	146·98 (48·82–377·67)	32·6% (8·4 to 77·1%)
		Yemen	4·8% (1·3–11·5%)	61·9% (31·3 to 122·2%)	62·61 (16·83–171·33)	57·8% (28·2 to 116·2%)
		Afghanistan	7% (2·1–15·7%)	79·3% (48·3 to 148·3%)	104·97 (28·6–292·15)	60·3% (33·5 to 114·4%)
		Sudan	7·3% (2·3–16·4%)	100·2% (62·1 to 187%)	96·77 (29·85–247·66)	84·9% (50·9 to 163·6%)
	Alzheimer's disease and other dementias	Global	20·8% (9·3–35·5%)	25·3% (16·9 to 38·9%)	70·7 (20·77–179·6)	30·1% (20·9 to 45·9%)
		North Africa and Middle East	28·8% (14·7–45·8%)	50·2% (35·7 to 76·7%)	111·71 (34·19–279·74)	48·4% (31·4 to 80·4%)
		Algeria	30·3% (14·8–48·8%)	60·9% (41·4 to 109·7%)	121·85 (36·44–312·01)	52·9% (27 to 102·6%)
		Bahrain	40·5% (20·9–61·4%)	33·7% (20·8 to 54·9%)	163·81 (51·36–416·76)	28·5% (9·2 to 56%)
		Egypt	28·1% (13·7–44·9%)	53·9% (25·2 to 98·4%)	101·94 (30·77–248·87)	54·1% (20·1 to 113·2%)
		Iran	26·9% (13·2–43·1%)	63·7% (44·4 to 100·8%)	104·1 (33·44–264·68)	63·4% (40·8 to 114·3%)
		Iraq	31·5% (15·5–50·1%)	26·1% (9·1 to 50·2%)	120·55 (35·98–302·46)	28·3% (6·9 to 60·7%)
		Jordan	34·3% (17·8–52·5%)	37·1% (23·1 to 65%)	127·07 (40·58–314·52)	30·8% (10·5 to 64·5%)
		Kuwait	38·2% (19·5–57·6%)	30% (16·7 to 54·2%)	148·04 (49·02–360·41)	26·5% (10·7 to 56·2%)
		Lebanon	31·5% (15·7–50·1%)	43·8% (29·8 to 71·5%)	124·63 (37·96–311·91)	40·3% (18·9 to 92%)
		Libya	33·9% (17·2–52·7%)	43·1% (28·1 to 66·4%)	140·67 (41·93–351·92)	37·2% (13·7 to 72·3%)
		Morocco	27·5% (13·1–44·6%)	64·5% (43·6 to 110·4%)	106·29 (30·86–279·11)	65% (37·8 to 115·9%)
		Palestine	26·6% (11·3–44·9%)	52·6% (33 to 91·1%)	103·49 (29·37–273·02)	45·8% (19·5 to 93·1%)
		Oman	31·6% (16·4–49·7%)	107·4% (67·5 to 219·5%)	133·87 (39·27–348·07)	100·6% (55·5 to 222·5%)
		Qatar	45·3% (22·3–66·2%)	37·1% (7·5 to 84%)	184·71 (58·75–476·59)	41·8% (7·2 to 96·3%)
		Saudi Arabia	37% (19·4–56·2%)	61·7% (41·1 to 108·1%)	144·44 (44·81–358·41)	58·3% (28·7 to 123·6%)
		Syrian Arab Republic	28·5% (13·7–46·2%)	39·1% (22·9 to 67%)	111·67 (32·27–291·85)	43·3% (15·2 to 92%)
		Tunisia	30·4% (14·6–48·7%)	51·2% (35·8 to 82·2%)	121·9 (36·09–303·11)	47·8% (20·1 to 99·6%)
		Türkiye	29·6% (15·2–46·8%)	37·3% (23·5 to 61·8%)	117·57 (36·08–290·36)	34·5% (13·2 to 67·1%)
		United Arab Emirates	40% (20·1–60%)	40·2% (21·3 to 76·5%)	146·98 (48·82–377·67)	32·6% (8·4 to 77·1%)
		Yemen	16·3% (6·2–29·7%)	58·4% (33·8 to 108·8%)	62·61 (16·83–171·33)	57·8% (28·2 to 116·2%)
		Afghanistan	24·2% (10·5–41%)	66·6% (45·5 to 118·1%)	104·97 (28·6–292·15)	60·3% (33·5 to 114·4%)
		Sudan	26·4% (12·3–43·3%)	92·6% (62·8 to 167·5%)	96·77 (29·85–247·66)	84·9% (50·9 to 163·6%)
**Level 2 risk factors**						
Air pollution	Meningitis	Global	2% (1·7–2·2%)	7·5% (-7·8 to 24·1%)	4·62 (3·65–5·97)	-53·9% (-63·3 to -41·2%)
		North Africa and Middle East	1·6% (1·2–2·2%)	-10·2% (-39·3 to 27·6%)	1·11 (0·76–1·62)	-78·3% (-86·3 to -65·8%)
		Algeria	0·8% (0·3–1·5%)	10·5% (-67·9 to 390·3%)	0·3 (0·1–0·61)	-78% (-94 to -3·9%)
		Bahrain	0·4% (0·2–0·7%)	-56·1% (-82 to 7·8%)	0·07 (0·04–0·12)	-88·8% (-95·5 to -71·5%)
		Egypt	2·2% (1–4·1%)	-33·8% (-73 to 42%)	1·21 (0·49–2·46)	-83·7% (-94·2 to -63·3%)
		Iran	0·8% (0·6–1%)	-29·5% (-51·9 to -2·2%)	0·3 (0·2–0·41)	-85·4% (-91·3 to -76·8%)
		Iraq	1·6% (0·9–2·7%)	-26·9% (-65·7 to 58·5%)	1·09 (0·56–1·86)	-85·4% (-94 to -65·4%)
		Jordan	0·8% (0·3–1·5%)	-7·9% (-67·5 to 198·9%)	0·42 (0·17–0·85)	-59·9% (-87·5 to 36·2%)
		Kuwait	1·4% (0·8–2·3%)	2·1% (-49·9 to 94·2%)	0·29 (0·14–0·48)	-62·2% (-81·8 to -26·4%)
		Lebanon	0·4% (0·1–1%)	-53·1% (-89·1 to 97·6%)	0·13 (0·04–0·31)	-85·2% (-96·5 to -37·5%)
		Libya	0·4% (0·2–0·7%)	-70·3% (-89 to -20·9%)	0·14 (0·06–0·28)	-91% (-96·9 to -75·8%)
		Morocco	1·6% (0·7–3·1%)	-20·2% (-73·3 to 140·4%)	1·13 (0·44–2·29)	-85·6% (-95·6 to -54·2%)
		Palestine	0·6% (0·2–1·1%)	-33·9% (-77·3 to 102%)	0·24 (0·08–0·46)	-88·4% (-96 to -66%)
		Oman	0·8% (0·3–1·6%)	-40% (-77·3 to 36·6%)	0·2 (0·07–0·41)	-84·3% (-94·5 to -60·3%)
		Qatar	0·8% (0·5–1·4%)	-44·1% (-73·9 to 13·7%)	0·21 (0·12–0·35)	-85% (-93·1 to -67·7%)

Table S15 Burden of neurological conditions attributable to risk factors in North Africa and Middle East countries

			DALYs (95% UI)			
			PAF (%)	PAF change (%)	Rate per 100,000	Rate change (%)
Risk factors	Causes	Locations	2019	From 1990 to 2019	2019	From 1990 to 2019
		Saudi Arabia	0·1% (0–0·2%)	-62·6% (-87·4 to -0·4%)	0·03 (0·01–0·06)	-90% (-96·4 to -74·6%)
		Syrian Arab Republic	0·4% (0·1–0·7%)	-55·2% (-87·3 to 39·7%)	0·37 (0·12–0·74)	-86·9% (-96·5 to -57·2%)
		Tunisia	0·9% (0·3–1·9%)	-1·9% (-72·9 to 223·1%)	0·36 (0·1–0·75)	-77·4% (-94·5 to -17·8%)
		Türkiye	0·2% (0·1–0·5%)	-39% (-80·5 to 95·9%)	0·06 (0·02–0·13)	-93·8% (-98·2 to -78·9%)
		United Arab Emirates	0·3% (0·1–0·7%)	-71·4% (-90·8 to -25·8%)	0·08 (0·03–0·22)	-92·7% (-97·6 to -80·6%)
		Yemen	1·1% (0·6–1·8%)	-33·8% (-69 to 36·5%)	1·07 (0·56–1·82)	-79·2% (-90·3 to -57·6%)
		Afghanistan	1·6% (0·8–2·8%)	-25·6% (-65·9 to 81·2%)	3·88 (1·8–6·96)	-80·3% (-91·4 to -52·2%)
		Sudan	1·2% (0·6–2·2%)	-34·3% (-70·4 to 43·6%)	1·19 (0·51–2·23)	-90·2% (-95·6 to -79·7%)
	Encephalitis	Global	0·6% (0·5–0·7%)	31·5% (-7·9 to 79·7%)	0·39 (0·3–0·51)	-39·8% (-60 to -6·3%)
		North Africa and Middle East	0·8% (0·5–1·4%)	8·3% (-36·9 to 85·9%)	0·35 (0·21–0·59)	-14·9% (-54·3 to 56·2%)
		Algeria	0·4% (0·1–1%)	50·1% (-63·6 to 590·4%)	0·08 (0·02–0·18)	6% (-74·1 to 359·5%)
		Bahrain	0·2% (0·1–0·4%)	-36·6% (-76·2 to 71·1%)	0·03 (0·01–0·05)	-52·7% (-83·4 to 28·6%)
		Egypt	1·6% (0·5–3·3%)	3·3% (-60·5 to 151%)	1·03 (0·18–2·39)	-29·8% (-76·3 to 74%)
		Iran	0·6% (0·2–0·9%)	14·7% (-26·7 to 63%)	0·1 (0·03–0·15)	-9·6% (-51·9 to 45%)
		Iraq	0·6% (0·3–1·2%)	-2·6% (-61·1 to 146·3%)	0·46 (0·16–0·91)	-35·3% (-76·1 to 67·1%)
		Jordan	0·2% (0·1–0·4%)	-31·2% (-80·4 to 164·7%)	0·02 (0·01–0·04)	-73% (-93·5 to 37·6%)
		Kuwait	0·5% (0·3–0·8%)	20·4% (-47·9 to 150%)	0·06 (0·03–0·09)	-11·4% (-61·6 to 92%)
		Lebanon	0·4% (0·1–0·9%)	-6·4% (-80·4 to 380·2%)	0·06 (0·01–0·15)	-23·6% (-84·9 to 286·8%)
		Libya	0·4% (0·1–0·9%)	-30·9% (-79·1 to 109·7%)	0·07 (0·02–0·14)	-37·5% (-83·3 to 82·4%)
		Morocco	0·9% (0·3–2·1%)	12·5% (-63·5 to 260·1%)	0·22 (0·08–0·47)	-7·1% (-70·9 to 190%)
		Palestine	0·3% (0·1–0·6%)	-15·7% (-77·4 to 179·1%)	0·05 (0·01–0·1)	-34·1% (-83·5 to 126·1%)
		Oman	0·4% (0·2–0·8%)	-8·9% (-70·4 to 177·8%)	0·46 (0·17–0·96)	-49·1% (-85·2 to 57%)
		Qatar	0·4% (0·2–0·7%)	-17·4% (-64·3 to 100·6%)	0·05 (0·02–0·1)	-41·4% (-77 to 37%)
		Saudi Arabia	0·1% (0–0·5%)	-0·6% (-77·4 to 172·4%)	0·04 (0·01–0·15)	-49·5% (-89·5 to 45·4%)
		Syrian Arab Republic	0·2% (0·1–0·4%)	-28·9% (-77·2 to 113·1%)	0·06 (0·02–0·12)	-57·7% (-88·5 to 38·5%)
		Tunisia	0·4% (0·1–1%)	7·3% (-71·6 to 289·8%)	0·07 (0·02–0·17)	-24·2% (-81·6 to 188·9%)
		Türkiye	0·1% (0–0·3%)	-21·5% (-81·4 to 177·3%)	0·02 (0·01–0·05)	-58·4% (-91·2 to 62·5%)
		United Arab Emirates	0·4% (0·1–0·9%)	-15·1% (-75·2 to 156·9%)	0·07 (0·02–0·15)	-32·4% (-81·4 to 130·9%)
		Yemen	0·5% (0·2–1%)	-20·2% (-70·1 to 116·5%)	0·15 (0·07–0·29)	-25·9% (-71 to 86·7%)
		Afghanistan	0·3% (0·1–0·7%)	-12·3% (-63·7 to 116·7%)	0·56 (0·21–1·51)	-25·9% (-70·4 to 85·9%)
		Sudan	0·5% (0·2–1·1%)	-21% (-71·9 to 121·6%)	0·15 (0·07–0·29)	-39·4% (-77 to 76·3%)
	Stroke	Global	29·7% (27·2–32·4%)	-8·6% (-13·9 to -3·1%)	525·57 (463·91–589·93)	-40·8% (-47·4 to -34·3%)
		North Africa and Middle East	29·2% (26·5–32·1%)	-2·5% (-8·3 to 5·1%)	533·24 (450·16–622·16)	-33·7% (-42·6 to -21·4%)
		Algeria	23·4% (17–29·3%)	-0·1% (-7·6 to 6%)	410·3 (278·57–551·21)	-44·6% (-56·3 to -31%)
		Bahrain	32·4% (28–36·5%)	-4·9% (-12·6 to 3·2%)	305·27 (242·73–382·55)	-54·8% (-63·5 to -43·6%)
		Egypt	34·3% (29·1–38·6%)	32·3% (10·2 to 49·4%)	734·11 (528·25–1009·87)	-14·4% (-34·7 to 10%)
		Iran	25·8% (22·8–29%)	-4·8% (-7·8 to -2·1%)	325·8 (281·73–373·28)	-48·3% (-53·6 to -40·5%)
		Iraq	31·4% (26·2–36·4%)	-9·9% (-15·5 to -5·3%)	917·95 (689·76–1178·55)	-27·3% (-43·3 to -7·8%)
		Jordan	22·6% (19·1–26·2%)	1·4% (-3·2 to 7·2%)	327·98 (259·19–404·42)	-49·5% (-58·7 to -39·1%)
		Kuwait	34·3% (30·5–37·8%)	0·6% (-2·3 to 3·9%)	321·62 (267·47–391·12)	-11·1% (-23·6 to 4·3%)
		Lebanon	22·1% (17·6–26·7%)	1·6% (-6·6 to 9·4%)	166·14 (115·93–215·78)	-32·7% (-47·6 to -14·7%)
		Libya	27·1% (20·1–33·4%)	3·9% (-9 to 16·1%)	425·68 (287·87–594·2)	-18·2% (-37·4 to 8·4%)
		Morocco	26·3% (22–30·4%)	-0·7% (-10·9 to 9·4%)	593·27 (444·81–767·08)	-17·8% (-37·4 to 4·6%)
		Palestine	22·8% (17·5–27·8%)	-10·4% (-20·1 to -3·6%)	485·67 (356·72–612·51)	-39·6% (-54·1 to -22·2%)
		Oman	27·5% (21·3–33%)	-14% (-23·7 to -7·1%)	517·75 (390·52–652·4)	-47·3% (-59·8 to -29·8%)
		Qatar	34·4% (30·7–37·7%)	-5·3% (-10·1 to -0·7%)	311·43 (247·97–397·07)	-47·6% (-59·5 to -32·5%)
		Saudi Arabia	35·8% (31·6–39·7%)	1·8% (-6 to 9·1%)	756·82 (586·95–933·06)	-27·8% (-47·1 to -2·5%)
		Syrian Arab Republic	23·3% (19·3–27·5%)	-2·1% (-10·7 to 7·3%)	471·75 (341·09–628·82)	-41·4% (-57·1 to -18·4%)
		Tunisia	22·9% (17·4–28·5%)	-3·9% (-10·8 to 1·6%)	337·89 (232·77–472·49)	-27·7% (-46·3 to -3·9%)

Table S15 Burden of neurological conditions attributable to risk factors in North Africa and Middle East countries

Risk factors	Causes	Locations	DALYs (95% UI) PAF (%) 2019	PAF change (%) From 1990 to 2019	Rate per 100,000 2019	Rate change (%) From 1990 to 2019
		Türkiye	20·4% (17·2–23·8%)	-10·9% (-18·1 to -3·6%)	237·86 (183·09–302·86)	-31·9% (-50·9 to -12·3%)
		United Arab Emirates	30·2% (24·4–35·7%)	3% (-2·3 to 8·1%)	581·53 (421·74–780·36)	-44·3% (-58·2 to -26·9%)
		Yemen	33·7% (27·2–40·1%)	-21·2% (-36·2 to -8%)	934·02 (673·24–1267·34)	-40·6% (-57·8 to -16·7%)
		Afghanistan	42% (38·1–46%)	-13·3% (-22·2 to -5·1%)	1470·15 (1038·17–1910·81)	-32·2% (-49 to -12·5%)
		Sudan	36·3% (31·1–40·6%)	-16·3% (-29·4 to -3·8%)	938·88 (668·03–1337·89)	-44% (-57·4 to -25·9%)
	Ischaemic stroke	Global	25·5% (23–28·1%)	-0·1% (-8·3 to 8·5%)	203·4 (177·76–231·59)	-28·6% (-37·8 to -19·2%)
		North Africa and Middle East	28·1% (25·3–31%)	-2·7% (-8·3 to 2·3%)	332·52 (281·93–387·23)	-11·3% (-24·9 to 2·1%)
		Algeria	22·2% (16–28·2%)	3·5% (-3·9 to 9·9%)	283·77 (194·03–385·09)	-22·1% (-37·7 to -2·7%)
		Bahrain	30·8% (26·7–34·9%)	-5% (-12·4 to 3·2%)	189·18 (151·9–238·37)	-46·7% (-56·8 to -35%)
		Egypt	35·3% (30·1–39·5%)	16·5% (4·5 to 28·3%)	487·64 (355·27–656·08)	32·7% (-2·5 to 67·6%)
		Iran	25·2% (22·2–28·4%)	-5·4% (-8·3 to -2·9%)	248·05 (213·04–286·42)	-45·9% (-52 to -38·1%)
		Iraq	29·7% (24·6–34·6%)	-10·9% (-16·9 to -6·5%)	543·87 (414·45–681·36)	-17·1% (-34·6 to 3%)
		Jordan	21·8% (18·3–25·2%)	2·3% (-2·6 to 8%)	236·08 (185·66–292·64)	-45·1% (-55·3 to -34·3%)
		Kuwait	33·1% (29·3–36·5%)	-0·8% (-3·7 to 2·4%)	213·32 (175·12–262·42)	-12·7% (-24·7 to 1·9%)
		Lebanon	21·3% (16·9–25·9%)	4·9% (-3·5 to 13·5%)	127·57 (89·06–165·75)	-10·9% (-30·8 to 10·6%)
		Libya	26·7% (19·7–33%)	0·2% (-11·3 to 8·9%)	299·73 (201·41–419·31)	11·5% (-14·3 to 44·7%)
		Morocco	25·3% (21·1–29·4%)	1·9% (-9·1 to 11·9%)	405·34 (307·61–519·27)	20·7% (-8·7 to 51·1%)
		Palestine	22% (16·8–26·8%)	-10·1% (-20·1 to -3%)	350·3 (258–442·52)	-26·1% (-43·3 to -6·5%)
		Oman	27% (20·8–32·7%)	-14·6% (-24 to -7·3%)	348·06 (263·87–443·22)	-30·3% (-46·9 to -6·6%)
		Qatar	32·5% (28·7–35·9%)	-5·9% (-10·6 to -1·4%)	187·74 (150·13–237·5)	-39·3% (-52·9 to -23·6%)
		Saudi Arabia	33·7% (29·6–37·6%)	2·6% (-5·6 to 10·1%)	438·62 (340·38–538·64)	-15·3% (-36·7 to 9·4%)
		Syrian Arab Republic	22·5% (18·4–26·6%)	-5·1% (-12·5 to 3·2%)	241·16 (177·7–320·53)	-28·9% (-47·7 to -3·3%)
		Tunisia	21·9% (16·6–27·5%)	-1·7% (-8·4 to 3·3%)	238·79 (164·33–334·46)	2·2% (-24·2 to 34·7%)
		Türkiye	18·9% (15·8–22·1%)	-11·8% (-19·2 to -5·1%)	125·09 (95·45–157·41)	-18·8% (-41·8 to 3·5%)
		United Arab Emirates	29% (23·5–34·4%)	4·2% (-1·3 to 9·2%)	410·8 (301·17–545·79)	-31·2% (-47·6 to -11·2%)
		Yemen	32·3% (25·9–38·6%)	-21·1% (-37·3 to -6·7%)	554·06 (404·3–753·99)	-5·7% (-33·1 to 28·9%)
		Afghanistan	39·8% (35·8–44·1%)	-12·7% (-24·4 to -3·1%)	742·25 (545·08–990·32)	11% (-15·9 to 44·3%)
		Sudan	35·2% (30·2–39·4%)	-16% (-30·2 to -2·4%)	596·27 (430·77–861·56)	-9·3% (-32·4 to 20·7%)
	Intracerebral haemorrhage	Global	33·8% (31–36·6%)	-10·4% (-15·4 to -5·9%)	281·22 (247·98–314·89)	-43·2% (-50·3 to -36·4%)
		North Africa and Middle East	31·6% (28·7–34·6%)	-1·9% (-9·8 to 9·6%)	173·33 (142·8–209·64)	-52·6% (-60·5 to -40·9%)
		Algeria	26·2% (19·3–32·5%)	2·4% (-5·5 to 9%)	105·79 (72·28–150·07)	-67·1% (-75·5 to -56·2%)
		Bahrain	35·5% (30·8–39·8%)	-4·8% (-12·3 to 2·9%)	94·07 (73·72–121·47)	-65·9% (-73·8 to -55·2%)
		Egypt	33·5% (28–38·9%)	27·1% (6·7 to 58·2%)	215·76 (143·82–324·5)	-48·4% (-64·2 to -26·5%)
		Iran	27·8% (24·5–31·1%)	-2·6% (-6·5 to 2·4%)	62·06 (54·15–70·34)	-54·9% (-61·8 to -46·9%)
		Iraq	34·1% (28·7–39·3%)	-7·2% (-13·2 to -1·6%)	349·01 (254·33–465·64)	-35% (-52·4 to -12·5%)
		Jordan	25% (21·2–28·6%)	1·8% (-3·1 to 7·8%)	81·26 (63·87–102·28)	-59·3% (-67·5 to -48·8%)
		Kuwait	36·9% (32·9–40·5%)	2·1% (-1·5 to 5·9%)	90·8 (73·05–113·16)	-2·2% (-21·6 to 21%)
		Lebanon	24·5% (19·7–29·5%)	2·4% (-5·8 to 11%)	31·5 (21·13–45·36)	-63·1% (-73·9 to -48·6%)
		Libya	28·5% (21·5–35·2%)	3·5% (-10·5 to 19·5%)	107·66 (68·74–155·95)	-49·4% (-63·9 to -28·8%)
		Morocco	28·7% (24–33·1%)	1·3% (-9 to 12·1%)	159·27 (114·35–213·71)	-50·4% (-64·6 to -30·2%)
		Palestine	25% (19·4–30·2%)	-7% (-16·8 to 0%)	122 (88·04–155·9)	-59·8% (-70·2 to -45·9%)
		Oman	28·9% (22·8–34·8%)	-13·6% (-22·9 to -6·4%)	145·99 (109·73–192·11)	-65·4% (-74·2 to -52·5%)
		Qatar	37% (32·9–40·3%)	-3·5% (-8·7 to 1·5%)	90·2 (70·3–119·3)	-57·9% (-69·4 to -43%)
		Saudi Arabia	38·9% (34·3–43%)	3·5% (-4·3 to 11·3%)	297·95 (227·24–374·61)	-39·3% (-57·9 to -10·2%)
		Syrian Arab Republic	25·3% (20·9–29·8%)	-4·1% (-12·3 to 5%)	213·01 (149·87–290·31)	-50·6% (-64·6 to -28·5%)
		Tunisia	25·4% (19·4–31·5%)	-0·9% (-8·3 to 6·3%)	82·06 (54·48–115·55)	-56·9% (-70·3 to -36·3%)
		Türkiye	22·1% (18·7–25·7%)	-8·1% (-16·2 to 0·6%)	88·35 (66·81–114·82)	-42% (-59·6 to -23·1%)
		United Arab Emirates	33·4% (27·3–39·7%)	4·9% (-0·9 to 10·5%)	150·42 (99·77–226·05)	-62·4% (-74·7 to -45·6%)
		Yemen	36·3% (29·5–42·8%)	-18·3% (-32·9 to -5·6%)	321·48 (221·59–445·25)	-62·2% (-73·9 to -44·8%)

Table S15 Burden of neurological conditions attributable to risk factors in North Africa and Middle East countries

Risk factors	Causes	Locations	DALYs (95% UI) PAF (%) 2019	PAF change (%) From 1990 to 2019	Rate per 100,000 2019	Rate change (%) From 1990 to 2019
		Afghanistan	44·7% (40·6–48·7%)	-10·7% (-19·3 to -3%)	625·24 (435·78–839·45)	-51·8% (-64·5 to -35·1%)
		Sudan	38·5% (33·2–42·9%)	-14·2% (-26·8 to -0·6%)	296·83 (195·97–425·84)	-66·2% (-75·6 to -52·7%)
	Subarachnoid haemorrhage	Global	30% (26·8–33·1%)	-16·8% (-22·3 to -11·4%)	40·95 (33·38–49·73)	-61·9% (-68·1 to -45·9%)
		North Africa and Middle East	29·1% (25·8–32·3%)	16·4% (-0·1 to 47·7%)	27·38 (21·39–35·06)	-56·9% (-69·5 to -31·9%)
		Algeria	27% (19·9–33·8%)	7·2% (-2·9 to 18·9%)	20·74 (13·39–29·51)	-62·1% (-73·7 to -39·9%)
		Bahrain	35·1% (30·6–39·3%)	15·7% (1·7 to 33·7%)	22·01 (16·31–31·56)	-51·5% (-67·1 to -30·5%)
		Egypt	26·9% (19·9–35%)	84·3% (25·7 to 242·3%)	30·71 (20–44·93)	-57·5% (-74·9 to -23·3%)
		Iran	28·8% (25·4–32·2%)	2·1% (-2·9 to 6·8%)	15·7 (13·31–18·93)	-54·7% (-67·3 to -31·4%)
		Iraq	34·7% (29·3–40·1%)	-0·7% (-9·6 to 10%)	25·07 (17·57–35·86)	-64·2% (-77·1 to -41·8%)
		Jordan	27·3% (23·3–31·3%)	5·2% (-0·4 to 11·5%)	10·63 (7·97–13·5)	-47·2% (-61·5 to -23·8%)
		Kuwait	37·2% (33·5–40·9%)	9·1% (2·8 to 17·1%)	17·49 (14·22–21·6)	-28·9% (-42·9 to -4·1%)
		Lebanon	26·5% (21·3–31·8%)	9·2% (-0·5 to 19·7%)	7·07 (4·62–10·19)	-61% (-76·8 to -36·2%)
		Libya	25·5% (18·6–32·2%)	40·9% (2·2 to 103·3%)	18·29 (11·43–28·02)	-52·5% (-68·1 to -25·2%)
		Morocco	28·5% (23·8–33·2%)	7% (-6·5 to 22·8%)	28·65 (18·92–42·72)	-55·7% (-72·1 to -22·6%)
		Palestine	26·7% (21–32·2%)	-5·5% (-15·2 to 2%)	13·37 (9·81–17·28)	-49% (-64·2 to -25·8%)
		Oman	26·5% (20·4–32·6%)	2% (-14·8 to 25·9%)	23·7 (14·18–39·9)	-61·6% (-77 to -26·4%)
		Qatar	40·1% (35·6–43·7%)	2·4% (-5·5 to 9·5%)	33·49 (24·56–44·23)	-53·2% (-72·4 to -24·7%)
		Saudi Arabia	40·9% (36–45·4%)	6·4% (-2·6 to 15·9%)	20·26 (14·55–27·13)	-49·4% (-70·7 to -14·8%)
		Syrian Arab Republic	16·1% (12·6–20·1%)	42% (6·2 to 95·1%)	17·57 (12·37–24·34)	-51% (-68·6 to -18·4%)
		Tunisia	26·4% (20·5–32·5%)	6·1% (-4·6 to 19·2%)	17·04 (11·09–24·59)	-60·5% (-76·2 to -32·2%)
		Türkiye	24·2% (20·6–27·9%)	-4·7% (-13·4 to 4·4%)	24·42 (17·94–32·47)	-42·6% (-63·8 to -2·7%)
		United Arab Emirates	33·4% (27·3–39·6%)	18·1% (5·6 to 33·1%)	20·31 (10·45–33·68)	-58% (-74·3 to -30·2%)
		Yemen	35·6% (28·8–42·3%)	-15·3% (-31·8 to 0·2%)	58·48 (30·7–98·87)	-56·9% (-71·7 to -28·7%)
		Afghanistan	43·9% (39·4–48·8%)	-10·1% (-21 to -0·2%)	102·66 (33·45–177·87)	-49·5% (-65·4 to -19·4%)
		Sudan	37·3% (32–42·2%)	-8·6% (-25·4 to 16·2%)	45·78 (25·3–77·86)	-67·1% (-79·3 to -37·3%)
Alcohol use	Stroke	Global	5·8% (4·2–7·4%)	16% (2·3 to 32·9%)	102·76 (72·28–133·09)	-24·8% (-35·9 to -12·2%)
		North Africa and Middle East	0·4% (0·2–0·7%)	-30·8% (-55·2 to -8·8%)	7·65 (3·24–12·3)	-52·9% (-69·5 to -37·4%)
		Algeria	0·4% (0–0·7%)	70·1% (-195·8 to 760·5%)	6·72 (0·41–13·23)	-5·7% (-163·2 to 363·6%)
		Bahrain	0·7% (0·2–1·3%)	-72% (-89·3 to -55%)	6·66 (2·24–11·94)	-86·7% (-95 to -77·4%)
		Egypt	0·1% (-0·1–0·4%)	-31·1% (-534·7 to 383·9%)	2·39 (-2·61–8·93)	-55·1% (-397·6 to 223·5%)
		Iran	0·5% (0·3–0·7%)	448·2% (118·3 to 4869·5%)	6·44 (3·88–9·32)	197·8% (20 to 2297·6%)
		Iraq	0·4% (0–0·9%)	-70·1% (-103·5 to -35·8%)	12·02 (-0·73–26·57)	-75·8% (-102·8 to -44·8%)
		Jordan	0·2% (-0·1–0·6%)	57·2% (-1182·4 to 991·6%)	3·48 (-1·62–9·18)	-21·7% (-578·9 to 398·7%)
		Kuwait	0% (-0·1–0·1%)		0·01 (-0·87–1·36)	
		Lebanon	0·6% (0·2–1·1%)	-62·5% (-85·2 to -38·7%)	4·92 (1·47–9·5)	-75% (-91 to -56·5%)
		Libya	0·3% (0–0·5%)	502·8% (-142·2 to 1892·5%)	4·73 (0·15–7·87)	373·1% (-130·7 to 1564·7%)
		Morocco	0·3% (0–0·6%)	-68·1% (-100·1 to -42·5%)	5·99 (-0·01–12·93)	-73·6% (-100·1 to -49·1%)
		Palestine	1·1% (0·7–1·5%)	28·3% (-17·4 to 172·6%)	22·79 (14·3–32·32)	-13·5% (-48·6 to 88·6%)
		Oman	0% (-0·2–0·3%)	-71·7% (-1035·1 to 572·5%)	0·38 (-4·15–5·29)	-82·9% (-661·9 to 391·3%)
		Qatar	0·3% (-0·1–0·7%)	-53·8% (-134·7 to -3·2%)	2·48 (-0·75–6·34)	-74·3% (-116·5 to -43·5%)
		Saudi Arabia	0·2% (-0·1–0·6%)	-67·1% (-214·2 to 136·5%)	4·03 (-2·56–14·19)	-76·6% (-183·4 to 70·1%)
		Syrian Arab Republic	0·7% (0·4–1·1%)	-42% (-64·9 to -22·8%)	14·73 (7·11–24·53)	-65·3% (-81 to -46%)
		Tunisia	1% (0·5–1·6%)	72% (3·5 to 307·8%)	15·04 (6·7–24·81)	29·4% (-29·7 to 211·4%)
		Türkiye	1·2% (0·5–1·8%)	-3·4% (-43·4 to 57·9%)	13·79 (5·97–22·71)	-26·1% (-59 to 29·8%)

Table S15 Burden of neurological conditions attributable to risk factors in North Africa and Middle East countries

Risk factors	Causes	Locations	DALYs (95% UI) PAF (%) 2019	PAF change (%) From 1990 to 2019	Rate per 100,000 2019	Rate change (%) From 1990 to 2019
		United Arab Emirates	0·6% (-0·3–1·6%)	-77·2% (-115·1 to -44·7%)	12·13 (-4·95–31·83)	-87·6% (-108·7 to -68·6%)
		Yemen	0·5% (0·2–0·7%)	-71·7% (-86·7 to -55·4%)	12·57 (4·79–22·38)	-78·6% (-90·1 to -62·2%)
		Afghanistan	0% (-0·1–0·1%)		0·86 (-2·09–4·46)	
		Sudan	0% (-0·1–0%)	-102·3% (-113·8 to -97·4%)	-0·53 (-1·81–0·63)	-101·5% (-109 to -98·2%)
	Ischaemic stroke	Global	2·1% (0·3–3·9%)	26·7% (-125·5 to 275·9%)	16·86 (2·07–31·5)	-9·4% (-117·7 to 163·3%)
		North Africa and Middle East	0% (-0·2–0·3%)	-48·2% (-399·7 to 533·7%)	0·36 (-2·44–3·31)	-52·7% (-370 to 468·5%)
		Algeria	0% (-0·3–0·3%)	-95·3% (-991 to 1125·3%)	-0·06 (-4·05–4·09)	-97·1% (-765·3 to 811·9%)
		Bahrain	0·1% (-0·3–0·5%)	-91·3% (-217·4 to -55·6%)	0·44 (-1·98–3·18)	-95·1% (-168·3 to -73·5%)
		Egypt	-0·1% (-0·4–0·1%)	11·7% (-415·6 to 465·1%)	-1·59 (-5·1–1·99)	27·1% (-456·2 to 547·2%)
		Iran	0·3% (0·1–0·5%)	993·8% (-11892·9 to 7518·4%)	2·84 (0·93–4·99)	526·6% (-6564 to 4368·7%)
		Iraq	-0·1% (-0·4–0·2%)	-136·6% (-750·9 to 590·8%)	-1·93 (-7·62–4·26)	-133·9% (-745·5 to 567·5%)
		Jordan	-0·1% (-0·3–0·3%)	-52·1% (-796·9 to 578·5%)	-0·54 (-3·57–3·03)	-74·5% (-487·4 to 290·1%)
		Kuwait	-0·1% (-0·2–0%)		-0·43 (-1·13–0·32)	
		Lebanon	0·3% (-0·1–0·7%)	-59·5% (-154·5 to -7·9%)	1·66 (-0·83–4·72)	-65·3% (-144·1 to -18·5%)
		Libya	0·2% (0–0·3%)	-4996·8% (-11729·3 to 13571·4%)	1·69 (-0·07–3·28)	-5465·9% (-14472·5 to 14852·3%)
		Morocco	0% (-0·2–0·3%)	-89·8% (-870·6 to 708·5%)	0·31 (-3·77–4·56)	-88% (-1002·1 to 790·8%)
		Palestine	0·5% (0·2–0·9%)	74·3% (-26·1 to 649·8%)	8·65 (3·2–15·05)	42·8% (-40·3 to 532%)
		Oman	-0·2% (-0·4–0·1%)	-3·2% (-175·5 to 179·5%)	-2·16 (-5·86–1·62)	-20·6% (-150·7 to 164%)
		Qatar	-0·1% (-0·5–0·2%)	136·2% (-552·7 to 678·4%)	-0·84 (-3·04–1·41)	50·8% (-379·6 to 392·6%)
		Saudi Arabia	-0·1% (-0·3–0·2%)	-240·4% (-727·8 to 887%)	-0·87 (-3·89–2·68)	-212·9% (-592·5 to 750·3%)
		Syrian Arab Republic	0·3% (0–0·5%)	-45% (-89·1 to -17·1%)	2·77 (0·3–5·87)	-58·7% (-91·3 to -31·3%)
		Tunisia	0·3% (-0·3–0·8%)	-305·2% (-2755·9 to 2828·7%)	2·86 (-2·71–8·76)	-315·6% (-2938·7 to 3213·6%)
		Türkiye	0% (-0·5–0·6%)	-187·1% (-887 to 769·9%)	0·23 (-3·04–3·99)	-186·5% (-843·5 to 727·2%)
		United Arab Emirates	-0·2% (-1·1–0·8%)	-120·8% (-796·3 to 529·3%)	-2·15 (-15·54–11·27)	-113·7% (-565·9 to 320·1%)
		Yemen	0·1% (0–0·3%)	-75·1% (-116·4 to -47%)	2·24 (-0·7–5·96)	-70·1% (-119·3 to -32·2%)
		Afghanistan	-0·1% (-0·2–0%)		-1·36 (-3·05–0·28)	
		Sudan	0% (-0·1–0%)	-125·8% (-487·6 to 156·2%)	-0·85 (-2·12–0·22)	-127·6% (-513·9 to 186·7%)
	Intracerebral haemorrhage	Global	10·3% (7·4–13·4%)	14·8% (3·2 to 27·9%)	85·89 (60·4–111·94)	-27·3% (-37·5 to -15·8%)
		North Africa and Middle East	1·3% (0·8–1·9%)	-2·5% (-21·3 to 16·6%)	7·29 (4·28–10·9)	-52·9% (-63·2 to -41·1%)
		Algeria	1·7% (0·8–2·7%)	125·3% (47 to 335·6%)	6·78 (3·12–11·28)	-27·6% (-55·9 to 46·2%)
		Bahrain	2·4% (1·2–3·7%)	-57·7% (-74·9 to -40·5%)	6·22 (3·15–10·12)	-84·9% (-91·4 to -76·7%)
		Egypt	0·6% (0·1–1·3%)	48·3% (-45·6 to 327·1%)	3·98 (0·61–8·93)	-39·5% (-80 to 68·6%)
		Iran	1·6% (1·1–2·3%)	354·5% (186·9 to 954·1%)	3·6 (2·37–5·16)	110·5% (29·8 to 388·7%)
		Iraq	1·4% (0·5–2·4%)	-54·7% (-81·8 to -20·5%)	13·96 (4·43–27·16)	-68·2% (-87·2 to -40·8%)
		Jordan	1·2% (0·4–2·2%)	53·2% (-26·7 to 240·7%)	4·02 (1·37–7·35)	-38·7% (-71·6 to 45·8%)
		Kuwait	0·2% (0–0·6%)		0·44 (-0·09–1·37)	
		Lebanon	2·5% (1·4–3·9%)	-39·3% (-60·8 to -15·1%)	3·27 (1·66–5·67)	-78·1% (-87·5 to -65·7%)
		Libya	0·8% (0·1–1·2%)	505% (11·7 to 1371·1%)	3·04 (0·47–5·21)	194·8% (-47·9 to 669·4%)
		Morocco	1% (0·3–1·8%)	-42·5% (-73 to -14·8%)	5·69 (1·82–10·88)	-71·7% (-87·3 to -52·6%)

Table S15 Burden of neurological conditions attributable to risk factors in North Africa and Middle East countries

			DALYs (95% UI)			
			PAF (%)	PAF change (%)	Rate per 100,000	Rate change (%)
Risk factors	Causes	Locations	2019	From 1990 to 2019	2019	From 1990 to 2019
		Palestine	2·9% (2–4·1%)	60·8% (14·1 to 183%)	14·13 (9·14–21·28)	-30·4% (-56·4 to 32·7%)
		Oman	0·5% (0·1–1%)	28·4% (-43·7 to 228·3%)	2·54 (0·4–5·34)	-48·5% (-77·8 to 40·5%)
		Qatar	1·4% (0·5–2·4%)	-25·7% (-59 to 14·9%)	3·32 (1·12–6·09)	-67·5% (-83·7 to -46·2%)
		Saudi Arabia	0·6% (0–1·6%)	-49·3% (-87·1 to -18·6%)	4·91 (0·11–12·37)	-70·2% (-93·7 to -44·5%)
		Syrian Arab Republic	1·4% (0·7–2·2%)	-35% (-57·5 to -16·5%)	11·96 (5·96–20·28)	-66·5% (-81·1 to -47·7%)
		Tunisia	3·8% (2·3–5·4%)	116·7% (62·2 to 236·8%)	12·18 (6·89–19·27)	-5·9% (-40·8 to 63·9%)
		Türkiye	3·4% (2–4·9%)	13·6% (-17·8 to 58·8%)	13·56 (7·53–20·58)	-28·3% (-56·7 to 9·3%)
		United Arab Emirates	3·2% (1·1–5·5%)	-51·7% (-77·8 to -21·2%)	14·28 (4·57–27·87)	-82·7% (-93·1 to -66·8%)
		Yemen	1·2% (0·6–1·9%)	-56·6% (-74·6 to -38·1%)	10·33 (4·86–17·74)	-79·9% (-89·5 to -65·8%)
		Afghanistan	0·2% (0–0·4%)		2·23 (-0·06–5·32)	
		Sudan	0% (0–0·1%)	-97·5% (-102 to -92·8%)	0·32 (-0·19–1·06)	-99% (-100·8 to -97%)
	Neurological disorders*	Global	1·5% (0·8–2·5%)	-5·9% (-14·3 to 2·7%)	17·16 (11·54–23·99)	-7% (-16·7 to 3·9%)
		North Africa and Middle East	0·3% (0·1–0·5%)	-13·2% (-39·9 to 23·5%)	3·4 (1·88–5·53)	-17% (-44·5 to 21·7%)
		Algeria	0·3% (0·1–0·6%)	38·4% (-34·9 to 168·7%)	3·57 (1·45–6·69)	25·7% (-45 to 174·2%)
		Bahrain	0·5% (0·2–1%)	-61·4% (-81·4 to -23·3%)	6·94 (2·89–12·81)	-64·8% (-85·2 to -20%)
		Egypt	0·1% (0–0·2%)	-4·1% (-64 to 139·9%)	1·05 (0·32–2·4)	-3·5% (-66·4 to 165%)
		Iran	0·2% (0·1–0·4%)	219·7% (103·6 to 498·4%)	2·54 (1·48–4·02)	204·6% (90·2 to 478·2%)
		Iraq	0·2% (0·1–0·4%)	-63·3% (-84·8 to -23·9%)	2·34 (0·8–4·95)	-64·2% (-86·1 to -18·8%)
		Jordan	0·2% (0·1–0·5%)	12·6% (-54·5 to 162·5%)	2·43 (0·81–5·22)	6·3% (-60·5 to 176·6%)
		Kuwait	0% (0–0·1%)		0·35 (0·04–1·02)	
		Lebanon	0·3% (0·1–0·7%)	-49·3% (-76·6 to -1·7%)	4·27 (1·85–8·28)	-51·4% (-78·9 to 2·9%)
		Libya	0·1% (0–0·2%)	231% (-4·9 to 681·4%)	1·58 (0·46–2·86)	218% (-16·4 to 732·1%)
		Morocco	0·2% (0–0·3%)	-42·8% (-78·1 to 29·1%)	2·02 (0·62–4·57)	-41·1% (-80·8 to 45·7%)
		Palestine	0·5% (0·2–1%)	24·5% (-28·9 to 127%)	6·58 (3·52–11·23)	17·3% (-39·2 to 139·2%)
		Oman	0·1% (0–0·3%)	24·8% (-53·7 to 218·7%)	1·45 (0·39–3·28)	26·3% (-57·8 to 272·4%)
		Qatar	0·2% (0·1–0·5%)	-38·9% (-76·4 to 49·1%)	3·12 (0·98–6·66)	-40·8% (-79·2 to 69·9%)
		Saudi Arabia	0·1% (0–0·4%)	-43·3% (-75·5 to 13·3%)	2·04 (0·31–4·94)	-43·1% (-78·4 to 26·9%)
		Syrian Arab Republic	0·1% (0–0·3%)	-52·9% (-77·7 to -3%)	1·57 (0·65–2·92)	-52·2% (-79·7 to 11%)
		Tunisia	0·6% (0·2–1·3%)	40·7% (-26·1 to 180·2%)	7·65 (3·66–14·02)	37·5% (-33·7 to 202·8%)
		Türkiye	0·7% (0·3–1·4%)	11·9% (-42·3 to 118%)	9·8 (4·23–18·05)	1·5% (-52·2 to 125·2%)
		United Arab Emirates	0·7% (0·3–1·3%)	-57·5% (-80 to -17·8%)	8·98 (3·52–17·33)	-60·4% (-84 to -12·8%)
		Yemen	0·2% (0·1–0·4%)	-58·6% (-79·2 to -20·8%)	2·23 (0·97–4·09)	-60·1% (-81·7 to -15·8%)
		Afghanistan	0% (0–0·1%)		0·7 (0·21–1·49)	
		Sudan	0% (0–0%)	-96% (-98·8 to -91·2%)	0·15 (0·04–0·39)	-96·3% (-99 to -91·1%)
	Idiopathic epilepsy	Global	10·1% (7·3–13%)	11·2% (-2·6 to 23·8%)	17·16 (11·54–23·99)	-7% (-16·7 to 3·9%)
		North Africa and Middle East	2·1% (1·4–3·1%)	11·8% (-16 to 45·8%)	3·4 (1·88–5·53)	-17% (-44·5 to 21·7%)
		Algeria	2·5% (1·4–3·6%)	103·9% (47·9 to 201·7%)	3·57 (1·45–6·69)	25·7% (-45 to 174·2%)
		Bahrain	3·7% (2·4–5·6%)	-48·2% (-62·9 to -34%)	6·94 (2·89–12·81)	-64·8% (-85·2 to -20%)
		Egypt	1% (0·4–1·7%)	21·1% (-31·8 to 123·7%)	1·05 (0·32–2·4)	-3·5% (-66·4 to 165%)
		Iran	1·8% (1·2–2·6%)	367·8% (206·3 to 790·7%)	2·54 (1·48–4·02)	204·6% (90·2 to 478·2%)
		Iraq	1·8% (0·9–2·9%)	-48·6% (-71·1 to -21·2%)	2·34 (0·8–4·95)	-64·2% (-86·1 to -18·8%)
		Jordan	2% (1–3·3%)	36·3% (-17·3 to 139·4%)	2·43 (0·81–5·22)	6·3% (-60·5 to 176·6%)
		Kuwait	0·3% (0–0·6%)		0·35 (0·04–1·02)	
		Lebanon	3·3% (2–4·8%)	-34·3% (-54·8 to -12·5%)	4·27 (1·85–8·28)	-51·4% (-78·9 to 2·9%)
		Libya	1·1% (0·3–1·7%)	353·6% (27·6 to 721·5%)	1·58 (0·46–2·86)	218% (-16·4 to 732·1%)
		Morocco	1·5% (0·8–2·4%)	-33·3% (-55·5 to -8·5%)	2·02 (0·62–4·57)	-41·1% (-80·8 to 45·7%)
		Palestine	4·1% (2·6–6%)	55·8% (16·1 to 143·6%)	6·58 (3·52–11·23)	17·3% (-39·2 to 139·2%)

Table S15 Burden of neurological conditions attributable to risk factors in North Africa and Middle East countries

			DALYs (95% UI)			
			PAF (%)	PAF change (%)	Rate per 100,000	Rate change (%)
Risk factors	Causes	Locations	2019	From 1990 to 2019	2019	From 1990 to 2019
		Oman	1·4% (0·6–2·3%)	40·5% (-3·6 to 121·4%)	1·45 (0·39–3·28)	26·3% (-57·8 to 272·4%)
		Qatar	2·5% (1·4–3·8%)	-16·9% (-38·9 to 9·4%)	3·12 (0·98–6·66)	-40·8% (-79·2 to 69·9%)
		Saudi Arabia	0·9% (0·2–1·9%)	-42·2% (-67 to -18·2%)	2·04 (0·31–4·94)	-43·1% (-78·4 to 26·9%)
		Syrian Arab Republic	1·5% (0·8–2·3%)	-42% (-60·2 to -22·7%)	1·57 (0·65–2·92)	-52·2% (-79·7 to 11%)
		Tunisia	6·5% (4·3–9%)	89·8% (45·9 to 153·5%)	7·65 (3·66–14·02)	37·5% (-33·7 to 202·8%)
		Türkiye	4·6% (2·9–6·4%)	45% (5·9 to 110·1%)	9·8 (4·23–18·05)	1·5% (-52·2 to 125·2%)
		United Arab Emirates	4·2% (2·3–6·5%)	-44·3% (-66·7 to -22·4%)	8·98 (3·52–17·33)	-60·4% (-84 to -12·8%)
		Yemen	1·5% (0·9–2·3%)	-45·2% (-65·5 to -21·1%)	2·23 (0·97–4·09)	-60·1% (-81·7 to -15·8%)
		Afghanistan	0·3% (0·1–0·6%)		0·7 (0·21–1·49)	
		Sudan	0·1% (0–0·2%)	-94·4% (-98·2 to -88·1%)	0·15 (0·04–0·39)	-96·3% (-99 to -91·1%)
Child and maternal malnutrition	Meningitis	Global	6·4% (5·7–7·3%)	13·4% (-1·6 to 30·6%)	14·9 (11·92–19·23)	-51·4% (-61 to -38·4%)
		North Africa and Middle East	6·8% (5·2–9%)	-10·3% (-35·5 to 22·9%)	4·79 (3·37–6·71)	-78·3% (-85·5 to -67·4%)
		Algeria	4% (2·4–6·3%)	-8·9% (-52·2 to 78·3%)	1·62 (0·91–2·7)	-81·7% (-91·1 to -62·8%)
		Bahrain	1·7% (1·2–2·4%)	-53·4% (-73·4 to -20%)	0·32 (0·21–0·46)	-88·1% (-93·5 to -78·8%)
		Egypt	9·2% (4·5–16%)	-33·7% (-69·4 to 32·3%)	5 (2·09–9·57)	-83·7% (-93·7 to -65·1%)
		Iran	4·3% (3·2–5·3%)	-34% (-51·9 to -11·9%)	1·64 (1·17–2·18)	-86·3% (-91·5 to -79·3%)
		Iraq	8·3% (5·3–12%)	-16·7% (-55·7 to 49·2%)	5·59 (3·35–8·89)	-83·3% (-91·6 to -67%)
		Jordan	5·7% (3·4–9·4%)	-11·6% (-54·7 to 86·4%)	3·03 (1·68–5·48)	-61·5% (-83·1 to -10·9%)
		Kuwait	5·9% (3·8–8·3%)	3·1% (-32·5 to 51·3%)	1·19 (0·65–1·78)	-61·8% (-77·6 to -40·2%)
		Lebanon	3·2% (1·6–5·4%)	-53·6% (-79·5 to -3·9%)	0·93 (0·43–1·64)	-85·4% (-93·8 to -68·6%)
		Libya	2·4% (1·4–4%)	-66·2% (-82·6 to -33·8%)	0·85 (0·48–1·48)	-89·8% (-95 to -80·2%)
		Morocco	9·8% (5·3–16·6%)	-27·1% (-66·3 to 54%)	6·76 (3·12–12·68)	-86·8% (-94·8 to -67·4%)
		Palestine	3·9% (2·5–5·7%)	-29·4% (-62·3 to 31·1%)	1·58 (0·97–2·42)	-87·6% (-93·8 to -75·6%)
		Oman	4·1% (2·2–7%)	-35% (-65·8 to 26·9%)	1 (0·53–1·87)	-83% (-91·4 to -64%)
		Qatar	3·3% (2·1–5%)	-43·6% (-69·4 to 1·8%)	0·83 (0·53–1·28)	-84·8% (-92·5 to -70·8%)
		Saudi Arabia	0·4% (0·2–0·7%)	-63·5% (-84·3 to -24·5%)	0·12 (0·06–0·19)	-90·2% (-95·5 to -80·2%)
		Syrian Arab Republic	2·6% (1·4–4·3%)	-53·1% (-78·4 to 2·5%)	2·56 (1·37–4·42)	-86·2% (-93·6 to -68·9%)
		Tunisia	5·9% (3·5–9·3%)	-6·6% (-51·7 to 84%)	2·24 (1·22–3·71)	-78·6% (-90 to -55·1%)
		Türkiye	2% (1·3–3·2%)	-36·6% (-66·6 to 20·3%)	0·51 (0·31–0·85)	-93·5% (-96·9 to -86·5%)
		United Arab Emirates	1·4% (0·7–3·4%)	-72·2% (-86·8 to -45·4%)	0·43 (0·21–1·08)	-92·9% (-96·5 to -85·7%)
		Yemen	5·1% (2·9–8·3%)	-10·6% (-55·6 to 76·3%)	4·96 (2·71–8·63)	-71·9% (-86·4 to -43·6%)
		Afghanistan	5·9% (3–10·4%)	-7·1% (-56·6 to 110·5%)	14·31 (7·06–25·73)	-75·4% (-89 to -43·5%)
		Sudan	5% (2·8–8·3%)	-9·1% (-50·3 to 65·6%)	4·99 (2·59–9·08)	-86·4% (-93 to -75%)
	Encephalitis	Global	2·3% (1·8–2·8%)	43·9% (3 to 92·7%)	1·53 (1·18–1·96)	-34·1% (-55·6 to 2%)
		North Africa and Middle East	3·7% (2·1–5·9%)	8·8% (-31·6 to 74·9%)	1·52 (0·9–2·5)	-14·5% (-51·5 to 51·4%)
		Algeria	2·3% (1·1–4·3%)	23·3% (-45·4 to 180·2%)	0·44 (0·23–0·78)	-12·2% (-64·5 to 112·1%)
		Bahrain	1% (0·6–1·6%)	-32·3% (-66·3 to 33·2%)	0·11 (0·06–0·2)	-49·5% (-77·1 to 2%)
		Egypt	6·6% (2·2–13·4%)	4·9% (-55 to 124·7%)	4·29 (0·77–9·74)	-28·8% (-72·2 to 63·4%)
		Iran	3·5% (1·4–4·8%)	7·6% (-25·3 to 43·9%)	0·58 (0·2–0·84)	-15·2% (-53·5 to 28·8%)
		Iraq	3·3% (1·6–5·7%)	10·3% (-48 to 140·9%)	2·33 (0·89–4·39)	-26·6% (-68·7 to 74·7%)
		Jordan	1·5% (0·8–2·5%)	-32·2% (-72·1 to 35·6%)	0·16 (0·08–0·28)	-73·5% (-90·7 to -6·1%)
		Kuwait	2·2% (1·5–3%)	20·6% (-20·3 to 92·8%)	0·23 (0·15–0·33)	-11·2% (-44·5 to 47·3%)
		Lebanon	2·6% (1·3–4·8%)	-3·6% (-60·4 to 139·5%)	0·43 (0·19–0·85)	-21·1% (-70·3 to 98·9%)
		Libya	2·5% (1·1–4·6%)	-19·8% (-68·5 to 87·3%)	0·4 (0·21–0·72)	-27·6% (-73·3 to 77·2%)
		Morocco	5·4% (2–10·4%)	-2·7% (-52·5 to 103·2%)	1·3 (0·62–2·44)	-19·3% (-65·2 to 84·1%)
		Palestine	1·9% (1·1–3·2%)	-10·2% (-58·5 to 101·8%)	0·31 (0·17–0·52)	-29·3% (-69·2 to 56·7%)
		Oman	1·9% (1–3·6%)	2·7% (-55·6 to 153·6%)	2·36 (1·12–4·28)	-42·6% (-76·3 to 39·3%)
		Qatar	1·5% (0·8–2·4%)	-16·9% (-62·2 to 86·7%)	0·2 (0·1–0·36)	-41·1% (-76·5 to 32·8%)
		Saudi Arabia	0·5% (0·1–1·7%)	1·5% (-71·8 to 128·7%)	0·14 (0·04–0·57)	-48·4% (-87·3 to 10·2%)

Table S15 Burden of neurological conditions attributable to risk factors in North Africa and Middle East countries

Risk factors	Causes	Locations	DALYs (95% UI) PAF (%) 2019	PAF change (%) From 1990 to 2019	Rate per 100,000 2019	Rate change (%) From 1990 to 2019
		Syrian Arab Republic	1·5% (0·9–2·2%)	-24·1% (-61·5 to 37·8%)	0·4 (0·23–0·69)	-55% (-79·3 to -7·4%)
		Tunisia	2·7% (1·3–5%)	4·1% (-55·7 to 158·3%)	0·46 (0·24–0·84)	-26·6% (-72·3 to 80·1%)
		Türkiye	0·9% (0·4–2%)	-19·9% (-66·5 to 67·6%)	0·17 (0·08–0·35)	-57·9% (-83·7 to -4·3%)
		United Arab Emirates	2·1% (1–3·9%)	-17·9% (-67·6 to 98·6%)	0·37 (0·16–0·69)	-34·6% (-75·4 to 68·7%)
		Yemen	2·3% (1·1–4·5%)	5% (-55·4 to 167·7%)	0·67 (0·34–1·25)	-2·1% (-57·5 to 138·7%)
		Afghanistan	1·1% (0·5–2·3%)	6·7% (-56·6 to 155·4%)	2·07 (0·84–5·18)	-9·4% (-62·7 to 119·3%)
		Sudan	2% (1–3·9%)	3·8% (-56·7 to 169·7%)	0·61 (0·33–1·04)	-20·4% (-66·6 to 104·2%)
Dietary risks	Stroke	Global	30% (22·2–39·1%)	-7·9% (-13·3 to -3·5%)	531·25 (389·53–704·59)	-40·4% (-46·8 to -34·1%)
		North Africa and Middle East	16·7% (12·6–21·5%)	-1·7% (-10·2 to 6·8%)	304·56 (223·3–400·76)	-33·2% (-42·5 to -21·5%)
		Algeria	16·9% (12·5–21·7%)	-19·6% (-31·7 to -5·9%)	295·81 (206·66–406·83)	-55·4% (-66·2 to -42%)
		Bahrain	13·1% (9·2–18·2%)	-6·6% (-16·6 to 3·6%)	123·58 (83·57–180·76)	-55·6% (-64·9 to -45%)
		Egypt	11·7% (7·4–17%)	20·4% (-8·9 to 49%)	250·33 (145·11–390·67)	-22·1% (-46·7 to 6·3%)
		Iran	15·3% (11·1–20·2%)	-17·6% (-26·1 to -9·4%)	192·92 (136·37–258·25)	-55·3% (-62·5 to -47·6%)
		Iraq	17% (11·7–23%)	11·1% (-2·2 to 26·6%)	497·79 (325·62–710·38)	-10·4% (-32·7 to 14·8%)
		Jordan	20·3% (15·1–25·7%)	-4·1% (-9·1 to 1·7%)	293·35 (209·99–390·68)	-52·3% (-60·9 to -42·9%)
		Kuwait	20·8% (14·7–28·5%)	-4·6% (-11·9 to 3·1%)	195·01 (133·56–276·94)	-15·8% (-29·1 to -0·1%)
		Lebanon	16·8% (12·2–21·6%)	27·4% (13·1 to 44·6%)	126·67 (82·7–178·68)	-15·5% (-35·7 to 6%)
		Libya	19% (13·9–24·7%)	19·9% (4·3 to 36·6%)	298·38 (201·89–422·4)	-5·7% (-28·6 to 25·7%)
		Morocco	15·4% (11–20·8%)	-16·2% (-31·2 to 1·3%)	347·07 (234·66–492·1)	-30·6% (-49·2 to -7·1%)
		Palestine	20·2% (15–25·8%)	-14·8% (-23·8 to -5·4%)	430·4 (311·83–565·63)	-42·5% (-55·2 to -25·6%)
		Oman	14·6% (10·5–19·4%)	-14% (-25·6 to -1·8%)	275·41 (191·61–378·36)	-47·2% (-60·1 to -29·1%)
		Qatar	11·9% (7·9–17·1%)	-8·1% (-19·1 to 2·5%)	107·2 (65·91–161·05)	-49·2% (-61·9 to -34·5%)
		Saudi Arabia	17% (12·3–23·1%)	11·3% (0·3 to 24·2%)	360·33 (239·33–508·81)	-21% (-42·5 to 7·4%)
		Syrian Arab Republic	19·3% (14·5–24·7%)	17·9% (7·2 to 31·9%)	388·93 (271·68–538·62)	-29·5% (-47·9 to -3·2%)
		Tunisia	14·7% (10·3–19·8%)	-10% (-22·8 to 4·7%)	217·81 (139·4–317·01)	-32·2% (-51·4 to -7%)
		Türkiye	11·5% (8–15·7%)	-5·5% (-13·8 to 3·5%)	134·08 (87·32–191·22)	-27·7% (-46·7 to -9·4%)
		United Arab Emirates	17·8% (12·7–23·5%)	4·4% (-12·1 to 22·1%)	343·26 (223·78–504·76)	-43·6% (-60 to -22·9%)
		Yemen	25·8% (19·9–31·8%)	5% (-5·2 to 15·9%)	714·65 (496·09–1006·84)	-20·7% (-40·7 to 7·3%)
		Afghanistan	29·7% (23·3–36·2%)	-0·7% (-10 to 8·8%)	1038·6 (707·84–1452·73)	-22·3% (-41·5 to -1·1%)
		Sudan	21·9% (16·3–27·8%)	-3·5% (-15·4 to 11·4%)	567·22 (380·9–891·43)	-35·4% (-50·8 to -15·9%)
	Ischaemic stroke	Global	26·7% (18·8–35·6%)	-3·8% (-9·5 to 0·5%)	213·15 (146·68–289·04)	-31·2% (-38·6 to -24·3%)
		North Africa and Middle East	17·4% (12·1–22·4%)	-4·1% (-8·8 to 0%)	206·11 (140·26–270·9)	-12·5% (-23·8 to -0·8%)
		Algeria	17·4% (11·9–22·5%)	-14·8% (-26·2 to -4%)	221·5 (144·16–303·18)	-35·8% (-50·6 to -18·9%)
		Bahrain	13·6% (8·7–18·4%)	-9·1% (-17·8 to -0·8%)	83·74 (52·31–120·85)	-48·9% (-58·8 to -38·2%)
		Egypt	12·7% (7·2–18·1%)	5·6% (-14·1 to 24·6%)	175·65 (93·56–280·9)	20·2% (-18·1 to 60·8%)
		Iran	16·6% (11·8–21·4%)	-15·7% (-24·2 to -8·5%)	163·07 (112·67–213·72)	-51·7% (-59·5 to -43·8%)
		Iraq	17·2% (11·3–23·8%)	3·7% (-8·4 to 18%)	315·16 (193·65–449·31)	-3·5% (-25·3 to 23·3%)
		Jordan	20·8% (14·8–26·5%)	-4·4% (-8·9 to 0·4%)	225·44 (152·86–305·15)	-48·6% (-57·8 to -38·9%)
		Kuwait	20·2% (12·9–28%)	-5·9% (-13·8 to 2·1%)	129·98 (81·36–186·54)	-17·3% (-29·8 to -1%)
		Lebanon	17·7% (12·1–22·4%)	15·3% (7 to 26·2%)	105·53 (65·48–147·89)	-2% (-23·1 to 20·1%)
		Libya	19·9% (13·8–25·7%)	10·5% (0·6 to 22%)	223·69 (143·29–313·93)	22·9% (-5·5 to 58·9%)
		Morocco	16·8% (11·3–22·2%)	-13·4% (-27 to 0·1%)	269·65 (171·39–374·17)	2·6% (-24·8 to 32·6%)
		Palestine	20·6% (14·9–26·4%)	-11·8% (-19·5 to -3·7%)	328·49 (229·76–438·96)	-27·5% (-42·8 to -7·2%)
		Oman	14·9% (9·6–19·8%)	-15·6% (-25·1 to -5·5%)	191·82 (120·45–265·71)	-31% (-47·4 to -8·7%)
		Qatar	12·3% (7·3–17·2%)	-11·1% (-20·8 to -2·3%)	71·05 (40·06–103·25)	-42·6% (-56·2 to -28·1%)
		Saudi Arabia	16·6% (10·2–22·7%)	9·7% (-0·4 to 20·8%)	215·77 (130·31–308·2)	-9·3% (-32·1 to 19·9%)
		Syrian Arab Republic	20·5% (14·7–25·9%)	9·8% (1·7 to 20·4%)	219·59 (146·93–302·96)	-17·7% (-39 to 10·9%)
		Tunisia	15·5% (10–20·4%)	-9·8% (-19·9 to -0·1%)	168·53 (103·9–248·15)	-6·1% (-31·2 to 24·6%)
		Türkiye	14% (9·5–17·9%)	-6·8% (-13·4 to -0·8%)	92·94 (59·16–126·42)	-14·2% (-36·9 to 7·6%)

Table S15 Burden of neurological conditions attributable to risk factors in North Africa and Middle East countries

			DALYs (95% UI)			
			PAF (%)	PAF change (%)	Rate per 100,000	Rate change (%)
Risk factors	Causes	Locations	2019	From 1990 to 2019	2019	From 1990 to 2019
		United Arab Emirates	17·7% (11·2–23·5%)	3·2% (-12·8 to 22·3%)	250·06 (154·81–356·32)	-31·8% (-51·1 to -7·6%)
		Yemen	25·2% (18·6–31·7%)	7·5% (2·9 to 12·4%)	433·05 (299·01–610·32)	28·7% (0·5 to 66%)
		Afghanistan	28·2% (21–35·3%)	4% (-2·6 to 10·6%)	526·05 (347·13–764·9)	32·5% (0·3 to 68%)
		Sudan	22·4% (16–28·8%)	-1·1% (-7·8 to 7·2%)	379·41 (241·08–592·93)	6·9% (-15·3 to 36·4%)
	Intracerebral haemorrhage	Global	32·8% (23·3–43·4%)	-8·5% (-14·3 to -3·6%)	273·5 (188·99–364·6)	-42% (-49·7 to -34·9%)
		North Africa and Middle East	15·5% (10·1–21·3%)	-6·1% (-14·1 to 3·6%)	84·92 (54·4–121·01)	-54·6% (-62·1 to -44·9%)
		Algeria	15·4% (9·3–22%)	-29% (-45·6 to -12·8%)	62·06 (35·47–93·59)	-77·2% (-84·6 to -67·4%)
		Bahrain	12% (6·5–18·3%)	-8·8% (-22·8 to 5·6%)	31·85 (16·53–51·06)	-67·3% (-76·2 to -56·9%)
		Egypt	10·1% (5·1–15·7%)	7·3% (-25·8 to 45·2%)	64·82 (29·78–117·3)	-56·4% (-73·3 to -33·4%)
		Iran	10·7% (5·8–16·7%)	-30·7% (-46·4 to -14·5%)	23·86 (12·91–37·18)	-67·9% (-76·3 to -58·3%)
		Iraq	16·6% (10·3–24·8%)	20·2% (0·8 to 54·4%)	170·12 (98·62–267·17)	-15·8% (-40·5 to 22·9%)
		Jordan	18·4% (11·5–26%)	-6% (-12·6 to 1·8%)	59·85 (37·28–85·7)	-62·4% (-70 to -52·7%)
		Kuwait	22·2% (13·9–31·1%)	-2·8% (-13·1 to 7·2%)	54·56 (32·6–79·41)	-6·9% (-27·5 to 17·7%)
		Lebanon	13·3% (7·2–19·5%)	36·9% (13·8 to 92·7%)	17·13 (8·37–28·92)	-50·5% (-66 to -23·4%)
		Libya	16·9% (10·5–24·3%)	15·1% (-3·7 to 39·4%)	63·66 (36·15–103·02)	-43·8% (-60·3 to -19·3%)
		Morocco	11·8% (6·1–18·2%)	-32·3% (-53·4 to -7·2%)	65·39 (33·06–106·44)	-66·8% (-80·6 to -46·8%)
		Palestine	18·8% (12·2–26·2%)	-22% (-36·8 to -5·3%)	91·77 (56·94–134·37)	-66·1% (-76·5 to -52·1%)
		Oman	14·1% (8·2–20·2%)	-16% (-29·6 to -3·1%)	71·23 (40·46–105·91)	-66·3% (-75·5 to -52·7%)
		Qatar	10·7% (5–17·3%)	-7·9% (-25·9 to 7·4%)	26·02 (11·85–44·37)	-59·7% (-72·4 to -45%)
		Saudi Arabia	17·6% (10·7–25·6%)	14·5% (1·1 to 36·9%)	134·9 (74·85–208·6)	-32·7% (-53·7 to -1%)
		Syrian Arab Republic	18·6% (11·8–25·9%)	15·3% (-0·4 to 37·2%)	156·24 (93·65–241·53)	-40·6% (-58·8 to -13%)
		Tunisia	12·6% (7·2–19%)	-19·1% (-35·6 to -1·9%)	40·62 (21·79–65·72)	-64·8% (-76·8 to -45·6%)
		Türkiye	7·9% (3·6–13·2%)	-15·6% (-34·9 to -0·7%)	31·72 (13·64–56·79)	-46·7% (-66·1 to -28·6%)
		United Arab Emirates	18·1% (10·8–25·7%)	6·2% (-18·6 to 45%)	81·73 (43·95–140·95)	-61·9% (-76·7 to -39·6%)
		Yemen	26·9% (18·6–35·1%)	5·5% (0 to 13·6%)	237·85 (145·2–359·85)	-51·1% (-64·7 to -32·2%)
		Afghanistan	31·5% (22·6–40·2%)	0·3% (-7·1 to 7·4%)	440·02 (275·46–646·6)	-45·8% (-59·9 to -27·4%)
		Sudan	21·1% (13·8–28·6%)	-8·5% (-18·3 to 5%)	162·41 (92·21–262·88)	-64% (-73·8 to -50·1%)
	Subarachnoid haemorrhage	Global	32·7% (23·6–42·3%)	-10·9% (-17 to -5·8%)	44·6 (31·24–59·66)	-59·2% (-65·9 to -43·3%)
		North Africa and Middle East	14·3% (9·2–19·9%)	11·1% (-5·3 to 39·6%)	13·53 (8·28–20·27)	-58·8% (-70·3 to -36·6%)
		Algeria	15·9% (9·7–23%)	-25% (-42·9 to -6·6%)	12·26 (6·89–19·45)	-73·4% (-83·7 to -57·5%)
		Bahrain	12·8% (7·1–19%)	11% (-10·1 to 34·5%)	8 (4·13–12·92)	-53·5% (-69·5 to -33%)
		Egypt	8·6% (4·3–13·8%)	60·5% (-1·1 to 209·4%)	9·86 (4·65–17·08)	-62·9% (-79·3 to -31·7%)
		Iran	11% (6–17%)	-29% (-45·5 to -11·9%)	5·99 (3·25–9·42)	-68·5% (-79·9 to -49·7%)
		Iraq	17·3% (10·9–25·5%)	28·4% (7·3 to 63·9%)	12·52 (6·9–20·51)	-53·7% (-71 to -23·7%)
		Jordan	20·7% (13·2–28·8%)	-2·6% (-9·5 to 6%)	8·05 (4·91–11·67)	-51·1% (-64·7 to -28·2%)
		Kuwait	22·3% (14·2–31·1%)	1·7% (-9·2 to 13·2%)	10·48 (6·46–15·2)	-33·8% (-47·8 to -10·8%)
		Lebanon	15% (8·4–21·3%)	48·5% (23 to 108·6%)	4·02 (2·04–6·37)	-46·8% (-68·9 to 0·2%)
		Libya	15·4% (9·5–22·2%)	55·7% (10·1 to 127·4%)	11·03 (6·09–18·06)	-47·5% (-65·4 to -16·9%)
		Morocco	12% (6·3–18%)	-27·3% (-50·7 to 1·9%)	12·02 (5·76–21·27)	-69·9% (-83·5 to -41·7%)
		Palestine	20·2% (13·2–28%)	-20·8% (-36 to -4·5%)	10·14 (6·28–14·37)	-57·2% (-71·4 to -34·5%)
		Oman	13·8% (8·4–19·9%)	1·5% (-18·5 to 32·1%)	12·36 (6·26–21·69)	-61·9% (-76·9 to -25·2%)
		Qatar	12·1% (5·9–19%)	-1·5% (-21·6 to 17·4%)	10·13 (4·75–16·65)	-54·9% (-74·6 to -26·1%)
		Saudi Arabia	19·5% (12·1–27·5%)	16·4% (2·3 to 36·9%)	9·65 (5·39–14·81)	-44·7% (-69·4 to -3·8%)
		Syrian Arab Republic	12% (7·3–17·4%)	71·8% (24·8 to 143·5%)	13·09 (7·64–20·28)	-40·7% (-62·8 to -0·9%)
		Tunisia	13·4% (7·7–19·6%)	-12·1% (-29·6 to 8·8%)	8·66 (4·48–13·94)	-67·2% (-81·5 to -42·1%)
		Türkiye	9·3% (4·3–14·9%)	-11·6% (-32·2 to 4·7%)	9·42 (4·24–16·12)	-46·6% (-67·6 to -8·5%)
		United Arab Emirates	18·8% (11·2–26%)	17·5% (-10·8 to 60·7%)	11·47 (5·23–20·13)	-58·2% (-76 to -27·6%)
		Yemen	26·6% (18·4–34·8%)	9·1% (-0·3 to 21·8%)	43·74 (20·75–81·04)	-44·3% (-61·9 to -8·8%)
		Afghanistan	31% (21·7–39·7%)	0·4% (-9·3 to 9·7%)	72·52 (24·23–136·85)	-43·3% (-61·6 to -11·5%)
		Sudan	20·6% (13·4–28·2%)	-2·5% (-17·8 to 19·7%)	25·4 (12·59–49·18)	-64·9% (-77·6 to -33·9%)

Table S15 Burden of neurological conditions attributable to risk factors in North Africa and Middle East countries

			DALYs (95% UI)			
			PAF (%)	PAF change (%)	Rate per 100,000	Rate change (%)
Risk factors	Causes	Locations	2019	From 1990 to 2019	2019	From 1990 to 2019
High body-mass index	Stroke	Global	23·6% (15·2–32·2%)	52·7% (32·1 to 88·3%)	416·62 (265·74–581·11)	-1·1% (-16·5 to 24%)
		North Africa and Middle East	33·8% (24·3–42·8%)	37·9% (22·8 to 61·1%)	618 (440·07–822·65)	-6·2% (-21·8 to 16·6%)
		Algeria	30·8% (21·7–39·8%)	29% (12 to 55·4%)	541·64 (362·05–750·9)	-28·6% (-45·9 to -4·3%)
		Bahrain	33·8% (24·3–42·5%)	6% (-2·9 to 16·5%)	317·53 (218·15–428·41)	-49·7% (-59·4 to -36·6%)
		Egypt	38·7% (27·8–47·8%)	65·2% (34·8 to 105·4%)	828·97 (545·9–1208·37)	6·9% (-21·9 to 44·8%)
		Iran	28% (19·9–36·2%)	42% (23·8 to 77·8%)	353·18 (248·49–462·49)	-22·9% (-37·2 to 0·4%)
		Iraq	36·1% (25·4–46·2%)	-0·5% (-9·6 to 11·8%)	1056·12 (692·06–1471·53)	-19·7% (-39·5 to 7·5%)
		Jordan	36% (25·5–45·1%)	17·4% (6·2 to 33·8%)	521·98 (358·2–687·11)	-41·6% (-53·3 to -26%)
		Kuwait	39·3% (28·7–48·3%)	15·6% (7·6 to 26·5%)	368·89 (255·94–482·69)	2·1% (-14·4 to 21·5%)
		Lebanon	32·7% (22·4–42·4%)	23% (8·8 to 44·4%)	246·61 (159·21–364·34)	-18·4% (-38 to 7%)
		Libya	38·5% (27·8–48%)	31·4% (15·6 to 53·9%)	605·34 (392·5–858·32)	3·4% (-20·6 to 38·1%)
		Morocco	30·7% (20·7–40·9%)	26·6% (7·6 to 60·7%)	695·11 (432·3–999·06)	5% (-22·1 to 48·4%)
		Palestine	25·5% (16·9–34·5%)	16·7% (4 to 38·3%)	542·5 (354·31–758·92)	-21·3% (-40·5 to 7%)
		Oman	31·6% (22·1–40·7%)	60·9% (24·4 to 131·7%)	595·49 (401·46–804·2)	-1·4% (-31·5 to 55·7%)
		Qatar	35·9% (26·2–44·8%)	3·8% (-8·9 to 17·6%)	324·23 (225·43–435·18)	-42·6% (-56·6 to -24·5%)
		Saudi Arabia	43·6% (32·3–52·9%)	49·1% (28·1 to 83·2%)	921·86 (636·88–1223·3)	5·8% (-24·5 to 55·6%)
		Syrian Arab Republic	33·7% (23·5–43·4%)	21·7% (8·5 to 42·9%)	680·54 (425·95–986·2)	-27·1% (-46·5 to 5·9%)
		Tunisia	30·4% (20·4–39·8%)	24·1% (9·2 to 46·7%)	449·98 (280·78–668·05)	-6·6% (-32·8 to 31·4%)
		Türkiye	35·5% (24·6–44·9%)	16·4% (4·8 to 34·2%)	413·03 (275·87–564·1)	-11% (-35·2 to 14·4%)
		United Arab Emirates	43% (32·6–51·8%)	31·8% (17 to 53·4%)	828·48 (569·33–1144·45)	-28·8% (-48·8 to -0·7%)
		Yemen	20·4% (12–28·6%)	59·1% (24·6 to 145·3%)	563·96 (299·1–912·08)	20·1% (-19·5 to 108·1%)
		Afghanistan	29·5% (19·9–39·1%)	36·9% (14·7 to 87·8%)	1033·02 (612·12–1572·56)	7·2% (-22·8 to 59·5%)
		Sudan	30·4% (20·5–40·6%)	60·7% (27·8 to 135·9%)	788·05 (470·56–1219·19)	7·6% (-24·6 to 73·4%)
	Ischaemic stroke	Global	16·5% (10·1–23·7%)	33% (22·8 to 52·2%)	132·05 (80·14–195·95)	-4·8% (-14·8 to 10·2%)
		North Africa and Middle East	27·3% (18·8–36%)	50·1% (34 to 77·5%)	323·44 (218·76–443·94)	37·1% (13·1 to 68·5%)
		Algeria	24·8% (16·5–33·7%)	62·2% (39·3 to 104·6%)	316·09 (194·74–460·92)	22% (-7·2 to 66·1%)
		Bahrain	26·2% (17·7–35·1%)	13·2% (2·7 to 26%)	160·36 (103·56–226·87)	-36·4% (-48·4 to -21·2%)
		Egypt	33·9% (23·4–43·5%)	71·2% (45·2 to 110·9%)	468·82 (302·68–687·36)	95·1% (39·5 to 167·3%)
		Iran	23·9% (16·2–32%)	46·4% (26·5 to 82·7%)	235·18 (158·39–319·64)	-16·1% (-31·9 to 8·5%)
		Iraq	27·5% (17·8–37·1%)	1·9% (-8·2 to 15·8%)	503·43 (319·42–714·99)	-5% (-26·3 to 25·4%)
		Jordan	31% (21·3–40%)	24·7% (12·1 to 44·8%)	335·53 (221·94–454·77)	-33% (-46·5 to -15·7%)
		Kuwait	33% (22·8–42·2%)	16·9% (7·4 to 29·1%)	212·66 (143·18–291·13)	2·8% (-12·9 to 23%)
		Lebanon	28·3% (18·9–37·4%)	44·5% (27·5 to 73·4%)	169·14 (107·1–250·31)	22·8% (-6·7 to 58%)
		Libya	33·4% (23·1–43·1%)	44·5% (29·7 to 69%)	375·02 (236·31–545·19)	60·9% (24·2 to 115·4%)
		Morocco	25·3% (16·1–34·9%)	53·6% (29·3 to 99·4%)	405·51 (243·19–598·87)	82·4% (32·1 to 154·1%)
		Palestine	21% (13·3–29·3%)	26·9% (12·9 to 54·3%)	333·62 (207·78–482·39)	4·3% (-20·7 to 42%)
		Oman	26% (17·6–35%)	95·2% (46·6 to 209·5%)	335·2 (211·41–468·71)	59·6% (6·4 to 165·8%)
		Qatar	27·9% (18·8–37·6%)	10·7% (-5·1 to 29·2%)	160·86 (105·22–229·04)	-28·6% (-46·3 to -6·7%)
		Saudi Arabia	34·6% (24·4–43·9%)	70·4% (43·6 to 115·5%)	450·01 (293·46–608·45)	40·8% (-0·3 to 103·7%)
		Syrian Arab Republic	26·1% (17·3–35·6%)	25·2% (11·9 to 47·5%)	279·53 (168·77–414·14)	-6·1% (-30·8 to 31·5%)
		Tunisia	25·1% (16–34·2%)	49·2% (30·1 to 81·2%)	273·44 (161·95–414·9)	55·1% (9·2 to 116·3%)
		Türkiye	26·4% (17·2–35·7%)	22·9% (9·4 to 43·1%)	174·95 (110·78–248·93)	13·2% (-18·4 to 47·4%)
		United Arab Emirates	36·9% (26·8–45·7%)	50·2% (32 to 80·9%)	521·95 (353·67–711·47)	-0·7% (-27·2 to 35·5%)
		Yemen	14·7% (8·3–21·6%)	93·4% (49·7 to 206·9%)	252·92 (132·66–408·98)	131·9% (61·8 to 306·5%)
		Afghanistan	21·3% (13·6–29·2%)	70·8% (39·9 to 140·4%)	396·21 (228·53–604·82)	117·7% (54·2 to 231·9%)
		Sudan	24·1% (15·5–33·6%)	108·1% (65 to 209·3%)	407·82 (234·43–645·81)	124·9% (64·4 to 259·2%)
	Intracerebral [illegible]	Global	28·4% (18–39%)	65·6% (38·8 to 113·3%)	236·21 (149·31–329·45)	4·9% (-14·3 to 36·6%)

Table S15 Burden of neurological conditions attributable to risk factors in North Africa and Middle East countries

			DALYs (95% UI)			
			PAF (%)	PAF change (%)	Rate per 100,000	Rate change (%)
Risk factors	Causes	Locations	2019	From 1990 to 2019	2019	From 1990 to 2019
	haemorrhage	North Africa and Middle East	46% (34·5–56·7%)	47·3% (29·8 to 76·7%)	252·2 (182·48–329·83)	-28·8% (-42·6 to -7·3%)
		Algeria	46·3% (34·3–57·4%)	38·8% (20·8 to 69·6%)	186·94 (127·5–257·28)	-55·5% (-67·8 to -36·9%)
		Bahrain	47·2% (35·2–58·1%)	8·9% (-1 to 21·8%)	124·86 (85·06–168·81)	-61% (-69·8 to -47·9%)
		Egypt	48·6% (36·6–59·5%)	71·5% (35·9 to 128·6%)	312·65 (200·21–475·16)	-30·3% (-52·5 to 5·4%)
		Iran	42·1% (31·2–52·5%)	43·5% (24 to 80·8%)	94·04 (70·09–118·68)	-33·5% (-46·7 to -11·2%)
		Iraq	50·2% (36·4–62·6%)	6% (-3 to 19·6%)	513·96 (342·12–724·8)	-25·7% (-46·1 to 3·9%)
		Jordan	50·2% (37·4–61·3%)	15·3% (5·1 to 31·2%)	163·5 (115·32–214)	-53·9% (-63·6 to -40·3%)
		Kuwait	52·6% (40·3–63·1%)	14% (6·7 to 24·7%)	129·46 (93·41–167·59)	9·2% (-13·4 to 36%)
		Lebanon	48·7% (36–60·1%)	30·2% (14·9 to 55·7%)	62·57 (40·13–93·95)	-53·1% (-67·2 to -31·7%)
		Libya	52·1% (39·7–62·9%)	36·1% (18·3 to 65·3%)	196·26 (130·29–275·76)	-33·6% (-52·2 to -4%)
		Morocco	44% (31·3–56·8%)	37·7% (16·6 to 75·6%)	244·26 (156·38–350·84)	-32·5% (-53·2 to 2·9%)
		Palestine	38·1% (26·5–49·7%)	28% (12·7 to 54·3%)	185·84 (124·49–256·61)	-44·6% (-59·5 to -21·9%)
		Oman	43·9% (32·3–54·6%)	62·7% (27·2 to 124%)	221·17 (154·5–300·22)	-34·7% (-55·8 to 3%)
		Qatar	47·9% (36–59%)	6·3% (-6·8 to 20·8%)	116·72 (79·42–160·29)	-53·6% (-66·4 to -35·8%)
		Saudi Arabia	57·5% (44·3–68·2%)	48·7% (28·6 to 84%)	439·79 (311·35–584·19)	-12·7% (-40 to 38·5%)
		Syrian Arab Republic	43·8% (31·3–55·5%)	22% (9·1 to 44·3%)	367·51 (229·88–532·58)	-37·1% (-55·5 to -5·6%)
		Tunisia	44·8% (32·2–56·8%)	36·4% (19·7 to 61·9%)	144·49 (90·55–213·37)	-40·8% (-60·2 to -11·1%)
		Türkiye	45·9% (32·1–57·8%)	22·4% (8·7 to 42·1%)	183·24 (122·76–258·16)	-22·8% (-46·6 to 1·9%)
		United Arab Emirates	59·7% (47·7–69·8%)	33·1% (19·2 to 55·2%)	268·95 (178·5–413·11)	-52·3% (-68·4 to -27·6%)
		Yemen	29·5% (17·9–40·8%)	83% (41·3 to 181·6%)	261·46 (139·21–418·98)	-15·2% (-45 to 55·5%)
		Afghanistan	39·2% (26·6–51·8%)	50·3% (26·4 to 107%)	547·88 (329·77–836·78)	-18·8% (-43·6 to 26·3%)
		Sudan	42·5% (29·9–54·4%)	76·5% (39·4 to 161·7%)	328·31 (190·23–509·55)	-30·5% (-54·2 to 19·7%)
	Subarachnoid haemorrhage	Global	35·4% (24·4–46·4%)	83·6% (48·4 to 148·8%)	48·36 (32·49–65·61)	-15·4% (-34·3 to 15·6%)
		North Africa and Middle East	45·1% (34–55·2%)	65·6% (37·8 to 115·7%)	42·36 (30·56–56·23)	-38·1% (-55·5 to -5%)
		Algeria	50·2% (38–61·4%)	36·8% (18·1 to 68·5%)	38·61 (25·84–56·11)	-51·5% (-66·9 to -23·2%)
		Bahrain	51·6% (40–61·4%)	31·4% (16·1 to 52·8%)	32·31 (22·45–46·32)	-44·9% (-61·8 to -20·7%)
		Egypt	41·5% (29·4–53·9%)	138·3% (58·6 to 345·7%)	47·5 (29·36–72·42)	-44·9% (-68·4 to 4·8%)
		Iran	43·9% (33–54·5%)	49·3% (28·3 to 88·9%)	23·96 (17·65–31·77)	-33·5% (-52·4 to -0·4%)
		Iraq	53·6% (40·5–65·4%)	15·7% (2·4 to 36·4%)	38·73 (25·07–57·67)	-58·3% (-73 to -33·4%)
		Jordan	59% (46·1–69%)	19·6% (8·3 to 38·2%)	22·96 (16·52–29·43)	-39·9% (-56·4 to -12·7%)
		Kuwait	57% (45–66·3%)	14·1% (5·4 to 24·8%)	26·77 (19·79–34·17)	-25·8% (-41·1 to 4·8%)
		Lebanon	55·8% (42·8–67·1%)	33·9% (16·7 to 61%)	14·9 (9·53–21·93)	-52·1% (-71·3 to -21·3%)
		Libya	47·5% (35·1–59·4%)	77·9% (26·9 to 155·1%)	34·06 (21·93–50·61)	-40% (-59·5 to -5%)
		Morocco	45·1% (31·4–57·8%)	39·7% (14·4 to 76%)	45·34 (27·15–74·09)	-41·6% (-63·9 to 7·2%)
		Palestine	46% (34–57·6%)	26·4% (11·8 to 50·4%)	23·05 (16·15–30·7)	-31·7% (-51·4 to 2·6%)
		Oman	43·8% (32·8–54·5%)	88·6% (43·6 to 171·1%)	39·12 (22·58–65·9)	-29% (-57·8 to 37·3%)
		Qatar	55·8% (43·4–66·4%)	15·1% (2·4 to 32·2%)	46·65 (33·03–64·25)	-47·3% (-68·7 to -17%)
		Saudi Arabia	64·8% (52·2–74·6%)	40·2% (21·1 to 69·2%)	32·06 (22·07–44·16)	-33·3% (-60·9 to 14·9%)
		Syrian Arab Republic	30·6% (21·5–39·4%)	85·8% (36·6 to 153·9%)	33·49 (21·53–47·54)	-35·8% (-57·8 to 6·1%)
		Tunisia	49·7% (36·9–61·4%)	39·8% (20·2 to 68·2%)	32·05 (20·66–45·8)	-47·8% (-69·2 to -10·8%)
		Türkiye	54·3% (40·3–65·6%)	26% (12·1 to 49·6%)	54·84 (37·2–75)	-23·9% (-51·7 to 27·3%)
		United Arab Emirates	61·8% (49·6–71·1%)	42·3% (23·7 to 70%)	37·58 (19·52–61·94)	-49·4% (-69·6 to -15·3%)
		Yemen	30·4% (18·6–42·4%)	85·7% (39·6 to 194·8%)	49·59 (21·88–93·25)	-4·6% (-41·1 to 95%)
		Afghanistan	38·4% (25–52%)	45·5% (17·9 to 113·6%)	88·93 (30·39–166·97)	-16·9% (-45·9 to 39·7%)
		Sudan	42·5% (29·5–55%)	80% (34·4 to 175·3%)	51·92 (27·05–92·14)	-34·1% (-61 to 32·1%)
	Neurological disorders*	Global	3·4% (0·9–8%)	32·1% (19·3 to 58·7%)	42·22 (11·24–107·37)	30·5% (18·3 to 56·4%)
		North Africa and Middle East	5·3% (1·6–11·9%)	55·7% (35·3 to 100%)	72·56 (20·98–181·16)	48·7% (28·5 to 94·4%)
		Algeria	5·5% (1·6–12·6%)	76·2% (37·6 to 162·8%)	74·4 (19·75–183·25)	59·8% (26·6 to 143·2%)
		Bahrain	6·6% (2–14·6%)	33·1% (6·4 to 76%)	90·41 (24·9–226·41)	21·3% (1·5 to 56·7%)
		Egypt	5·5% (1·6–12·4%)	43·9% (10·4 to 114·6%)	71·1 (19·02–173·98)	44·7% (9·7 to 120%)

Table S15 Burden of neurological conditions attributable to risk factors in North Africa and Middle East countries

Risk factors	Causes	Locations	DALYs (95% UI) PAF (%) 2019	PAF change (%) From 1990 to 2019	Rate per 100,000 2019	Rate change (%) From 1990 to 2019
		Iran	4·8% (1·4–10·7%)	75·5% (42·8 to 142·2%)	65·22 (18·7–168·56)	67·3% (36·6 to 135·2%)
		Iraq	5·8% (1·7–13·1%)	20·7% (-0·9 to 64·4%)	75·58 (20·62–188·59)	18·3% (-1·8 to 61·6%)
		Jordan	6·9% (2·1–15·2%)	46·8% (18·4 to 96·1%)	87·46 (24·25–219·23)	38·7% (12·8 to 89·7%)
		Kuwait	7·6% (2·4–16·4%)	36·3% (7·9 to 82·3%)	95·85 (27·3–233·49)	29% (6·1 to 67·6%)
		Lebanon	6% (1·7–13·6%)	45·7% (18·7 to 97·9%)	78·88 (21·75–195·98)	39·9% (14·4 to 96%)
		Libya	6·4% (1·9–14·3%)	34·7% (12·6 to 75·2%)	87·26 (23·81–223·08)	30·5% (6·2 to 71·5%)
		Morocco	4·8% (1·4–10·9%)	63·9% (28·5 to 147%)	64·33 (17·22–166·38)	67·2% (32·4 to 149·3%)
		Palestine	3·8% (1–9·3%)	48·2% (14·9 to 129·2%)	51·91 (12·92–136·79)	39·7% (8·1 to 122·5%)
		Oman	6·6% (2–14·8%)	161·8% (81·5 to 399·3%)	88·35 (24·45–226·53)	164·2% (82 to 416·8%)
		Qatar	8·3% (2·4–18·2%)	44·3% (-14 to 147·8%)	112·23 (29·3–285·75)	41·8% (-13·2 to 138·1%)
		Saudi Arabia	6·7% (2·1–14·9%)	80·2% (36·6 to 176·8%)	95·29 (26·63–234·48)	80·6% (39 to 174·5%)
		Syrian Arab Republic	5·3% (1·5–12%)	38·5% (10·5 to 93·5%)	68·58 (17·58–180·38)	40·9% (9·3 to 102·4%)
		Tunisia	5·5% (1·6–12·5%)	52·8% (23·9 to 110·5%)	72·18 (19·82–179·51)	48·9% (17·9 to 112·6%)
		Türkiye	5·8% (1·8–13%)	51·2% (23·1 to 104·2%)	84·41 (23·5–206·24)	36·6% (11·8 to 80·2%)
		United Arab Emirates	6·6% (1·9–14·6%)	55% (7 to 130·7%)	93·74 (25·63–237·23)	44·3% (5·6 to 112·7%)
		Yemen	2·1% (0·5–5·5%)	68·6% (20·6 to 225%)	27·67 (6·2–78·56)	64·4% (16·9 to 217·1%)
		Afghanistan	3·3% (0·9–7·9%)	87·6% (40·5 to 221·1%)	49·88 (12·56–130·16)	67·8% (26 to 184·2%)
		Sudan	4·1% (1·1–9·4%)	142·5% (73·9 to 326·5%)	54·26 (14·57–141·73)	123·6% (61·7 to 300·4%)
	Alzheimer's disease and other dementias	Global	12·5% (4·9–23·3%)	25·5% (14·1 to 49·8%)	42·22 (11·24–107·37)	30·5% (18·3 to 56·4%)
		North Africa and Middle East	18·7% (8·2–33·1%)	50·5% (31·6 to 90·9%)	72·56 (20·98–181·16)	48·7% (28·5 to 94·4%)
		Algeria	18·5% (8–32·9%)	67·7% (38·5 to 151·7%)	74·4 (19·75–183·25)	59·8% (26·6 to 143·2%)
		Bahrain	22·4% (9·8–38·2%)	26·1% (10·4 to 57·5%)	90·41 (24·9–226·41)	21·3% (1·5 to 56·7%)
		Egypt	19·6% (8·3–34·3%)	44·3% (13·8 to 113·1%)	71·1 (19·02–173·98)	44·7% (9·7 to 120%)
		Iran	16·9% (7·2–29·6%)	67·6% (39·1 to 128·8%)	65·22 (18·7–168·56)	67·3% (36·6 to 135·2%)
		Iraq	19·7% (8·2–34·8%)	16·3% (0·2 to 52·1%)	75·58 (20·62–188·59)	18·3% (-1·8 to 61·6%)
		Jordan	23·6% (10·4–40·2%)	45·6% (22·5 to 90·2%)	87·46 (24·25–219·23)	38·7% (12·8 to 89·7%)
		Kuwait	24·7% (10·9–41·2%)	32·6% (9·6 to 69·7%)	95·85 (27·3–233·49)	29% (6·1 to 67·6%)
		Lebanon	19·9% (8·5–34·9%)	43·4% (24·4 to 84·3%)	78·88 (21·75–195·98)	39·9% (14·4 to 96%)
		Libya	21% (9·4–36·2%)	36·3% (19·4 to 69%)	87·26 (23·81–223·08)	30·5% (6·2 to 71·5%)
		Morocco	16·6% (6·9–29·7%)	66·5% (36·6 to 142%)	64·33 (17·22–166·38)	67·2% (32·4 to 149·3%)
		Palestine	13·4% (4·6–25·9%)	46·6% (20·1 to 123·7%)	51·91 (12·92–136·79)	39·7% (8·1 to 122·5%)
		Oman	20·9% (9·2–35·9%)	173·3% (93·1 to 417·2%)	88·35 (24·45–226·53)	164·2% (82 to 416·8%)
		Qatar	27·5% (11·3–45·9%)	37% (-16·4 to 119·2%)	112·23 (29·3–285·75)	41·8% (-13·2 to 138·1%)
		Saudi Arabia	24·4% (10·8–40·6%)	84·2% (48·9 to 167·3%)	95·29 (26·63–234·48)	80·6% (39 to 174·5%)
		Syrian Arab Republic	17·5% (7·4–31·1%)	36·8% (16·1 to 84·3%)	68·58 (17·58–180·38)	40·9% (9·3 to 102·4%)
		Tunisia	18% (7·5–32·7%)	52·2% (30·2 to 105·3%)	72·18 (19·82–179·51)	48·9% (17·9 to 112·6%)
		Türkiye	21·3% (9–37·4%)	39·6% (20·5 to 75·9%)	84·41 (23·5–206·24)	36·6% (11·8 to 80·2%)
		United Arab Emirates	25·5% (11·1–42·2%)	52·3% (15 to 114%)	93·74 (25·63–237·23)	44·3% (5·6 to 112·7%)
		Yemen	7·2% (2·3–15·2%)	65·3% (19·6 to 213·5%)	27·67 (6·2–78·56)	64·4% (16·9 to 217·1%)
		Afghanistan	11·5% (4·5–21·7%)	74·4% (37 to 194·4%)	49·88 (12·56–130·16)	67·8% (26 to 184·2%)
		Sudan	14·8% (6·1–26·7%)	132·5% (70·9 to 311·6%)	54·26 (14·57–141·73)	123·6% (61·7 to 300·4%)
High fasting plasma glucose	Stroke	Global	20·1% (13·7–29·2%)	39·3% (31·8 to 48·7%)	354·95 (241·94–512·1)	-9·8% (-17·6 to -0·5%)
		North Africa and Middle East	27·6% (17·7–42·8%)	98·2% (68·5 to 132%)	504·87 (318·82–793·93)	34·8% (7·9 to 64·8%)
		Algeria	30·6% (18·6–51·1%)	90·8% (48·2 to 148·3%)	536·74 (315·17–918·49)	5·9% (-25·1 to 46·9%)
		Bahrain	30·7% (19·8–47·9%)	-1·8% (-16·7 to 14·5%)	289·49 (179·88–474·58)	-53·2% (-64·3 to -40·1%)
		Egypt	24·6% (15–39·5%)	258·7% (159·5 to 388·6%)	526·74 (295·21–920·94)	132·5% (50·4 to 233·2%)
		Iran	27·9% (16·5–46·7%)	94·3% (67·6 to 125·1%)	352·2 (207·74–596·84)	5·7% (-13·3 to 29·2%)
		Iraq	30·3% (20·1–44·2%)	55·2% (21·4 to 100·1%)	884·45 (563·87–1343·07)	25% (-12·1 to 74·3%)

Table S15 Burden of neurological conditions attributable to risk factors in North Africa and Middle East countries

			DALYs (95% UI)			
			PAF (%)	PAF change (%)	Rate per 100,000	Rate change (%)
Risk factors	Causes	Locations	2019	From 1990 to 2019	2019	From 1990 to 2019
		Jordan	29·9% (18–47·8%)	19·6% (-5 to 50%)	432·51 (257·92–673·15)	-40·4% (-55·6 to -20·8%)
		Kuwait	30% (19·4–46·4%)	6·3% (-10·5 to 30·3%)	281·08 (178·13–446·73)	-6% (-26·1 to 22·2%)
		Lebanon	31·1% (18·8–53%)	61·9% (26·6 to 115·5%)	234·26 (122·75–400·57)	7·4% (-25·7 to 51·7%)
		Libya	32% (20·1–51·7%)	54·7% (27·9 to 98·5%)	502·49 (284·86–866·15)	21·6% (-9·8 to 69·8%)
		Morocco	32% (19·6–51·7%)	139% (78·9 to 210·3%)	720·21 (429·3–1202·06)	97·7% (40·1 to 173·2%)
		Palestine	34·4% (20·4–57·2%)	42·5% (16·1 to 82·3%)	733·55 (439·8–1237·84)	-3·8% (-30·7 to 35·6%)
		Oman	31·8% (19·5–51·3%)	88·5% (42·9 to 146·5%)	600·57 (370·26–978·15)	15·7% (-20·5 to 73·4%)
		Qatar	29·7% (19·5–45·8%)	-2·1% (-17·6 to 16·9%)	269·33 (169·1–439·41)	-45·8% (-61·2 to -25·7%)
		Saudi Arabia	29·9% (20·7–43·3%)	32·9% (4·9 to 69·2%)	631·39 (410·47–929·63)	-5·6% (-34·8 to 38·4%)
		Syrian Arab Republic	29·2% (19·1–44·6%)	94·8% (55·9 to 148·4%)	589·86 (365·59–968·3)	16·6% (-19·6 to 70·1%)
		Tunisia	31·8% (19·1–52·3%)	55·4% (24·6 to 95·4%)	470·33 (273·47–802·48)	17·2% (-18·8 to 69·6%)
		Türkiye	22·2% (13·7–36%)	64·1% (29·1 to 112%)	258·96 (153·12–425·83)	25·4% (-15·5 to 76·3%)
		United Arab Emirates	30·5% (19·9–45·6%)	1·3% (-16·8 to 23·2%)	588·3 (353·82–922·63)	-45·2% (-60·5 to -26%)
		Yemen	22·7% (13·2–36·6%)	109·5% (53·1 to 175·4%)	626·88 (351·01–1071·41)	58·1% (6·2 to 130·7%)
		Afghanistan	32·8% (22·2–47·9%)	87·2% (47·4 to 142·4%)	1146·74 (695·29–1821·28)	46·5% (1·9 to 107%)
		Sudan	30·6% (19·3–48·6%)	118·6% (64·7 to 189·1%)	789·95 (460·45–1335·49)	46·2% (3·8 to 110·5%)
	Ischaemic stroke	Global	21·8% (11·6–40·4%)	33·5% (25·8 to 42·6%)	173·97 (90·51–316·3)	-4·6% (-13·2 to 5%)
		North Africa and Middle East	29·9% (15·7–53%)	83·6% (61·2 to 111·5%)	353·76 (190·41–627·47)	67·7% (38·9 to 103·2%)
		Algeria	32·1% (17–60·5%)	82·5% (40·7 to 140·2%)	408·83 (207·37–775·79)	37·7% (-0·4 to 92%)
		Bahrain	31·4% (17·4–57·5%)	-6·2% (-21·2 to 14·5%)	193·44 (101·06–355·48)	-47·2% (-60·3 to -32·3%)
		Egypt	28·1% (14·3–50·5%)	179·5% (99 to 278%)	388·95 (186·85–733·84)	219·1% (105·1 to 359·6%)
		Iran	29·1% (15–53·9%)	94·6% (68·1 to 124·4%)	287·01 (145·27–533·19)	11·7% (-9 to 36·4%)
		Iraq	32% (18·2–54·6%)	51·2% (15·9 to 97·3%)	585·61 (327·82–1009·38)	40·7% (-0·5 to 96·4%)
		Jordan	30·6% (15·9–53·8%)	16·8% (-6·8 to 47·3%)	331·46 (170·55–568·24)	-37·3% (-53 to -17·1%)
		Kuwait	31·1% (17·8–54·2%)	2% (-14·3 to 25·3%)	200·83 (110·84–352·13)	-10·2% (-29 to 17·6%)
		Lebanon	32·4% (17·2–59·2%)	54·2% (21·3 to 109·1%)	194 (91·25–359·7)	31·1% (-9·1 to 87%)
		Libya	34·1% (17·9–61·4%)	34·2% (11 to 74·2%)	382·74 (195·84–732·45)	49% (9·4 to 114·4%)
		Morocco	33·5% (16·7–60·8%)	126·3% (75 to 188%)	535·04 (269·99–989·58)	167·7% (89·7 to 264·6%)
		Palestine	35·1% (17·4–66%)	38·3% (14·7 to 77·1%)	559·14 (277·77–1051·09)	13·8% (-15·5 to 59·9%)
		Oman	33·5% (17–62·5%)	83·1% (42·3 to 136·2%)	433·23 (214·32–799·7)	49·8% (4·6 to 120·5%)
		Qatar	30·1% (16·9–55·6%)	-8·6% (-24·7 to 13·9%)	174·18 (95·91–335·88)	-41% (-57·4 to -17·7%)
		Saudi Arabia	30·7% (17·8–51·3%)	26·6% (-5 to 68·5%)	399·14 (223·95–669·16)	4·8% (-30·8 to 55·8%)
		Syrian Arab Republic	32·5% (16·5–58·8%)	79·3% (43·2 to 130·7%)	347·95 (171·61–688·58)	34·5% (-7·6 to 93·9%)
		Tunisia	33·1% (16·9–60·8%)	43% (15·4 to 82·4%)	361·23 (182·79–667·65)	49·2% (4·9 to 113·5%)
		Türkiye	24·1% (12–47·1%)	60·4% (25·4 to 111·2%)	159·78 (77–316·14)	47·5% (-2·2 to 108·7%)
		United Arab Emirates	31·2% (18·2–51·6%)	-2·8% (-21·4 to 22·6%)	442·31 (244·94–758·6)	-35·6% (-53·4 to -9·1%)
		Yemen	24·6% (11·8–47·4%)	103·8% (54·1 to 160·2%)	422·47 (193·65–847·83)	144% (69·9 to 245·1%)
		Afghanistan	34·6% (18·3–61·4%)	71·1% (38·1 to 123·7%)	645·43 (332·72–1187·73)	118·4% (56·6 to 211·1%)
		Sudan	32·8% (16·5–59·5%)	99·3% (49·5 to 167·6%)	554·33 (271·37–1092·51)	115·5% (53·4 to 218·4%)
	Intracerebral haemorrhage	Global	18·9% (12·1–26·9%)	42·2% (33·6 to 53·4%)	157·42 (99·86–227·19)	-9·9% (-20·4 to 0·7%)
		North Africa and Middle East	24·2% (15·6–33·9%)	94·8% (68·2 to 126·8%)	132·49 (81·9–191·04)	-5·9% (-23·4 to 18·3%)
		Algeria	27% (17·5–38·9%)	86·5% (48·6 to 133·3%)	109·19 (64·16–169·56)	-40% (-57·8 to -12·6%)
		Bahrain	30·4% (19·9–43·1%)	0·3% (-15·7 to 19·8%)	80·65 (48·8–123·82)	-64% (-73·3 to -50%)
		Egypt	19·2% (11–29·8%)	233·7% (130·6 to 379%)	124 (62·65–227·74)	35·8% (-15·2 to 110·3%)
		Iran	23·5% (14·7–34·4%)	91% (64·9 to 122·7%)	52·5 (32·92–77·66)	-11·4% (-29·4 to 11·8%)
		Iraq	27·6% (17·8–38·8%)	55·6% (22·6 to 95·5%)	281·93 (168·6–408·57)	8·8% (-26·4 to 58·6%)

Table S15 Burden of neurological conditions attributable to risk factors in North Africa and Middle East countries

Risk factors	Causes	Locations	DALYs (95% UI) PAF (%) 2019	PAF change (%) From 1990 to 2019	Rate per 100,000 2019	Rate change (%) From 1990 to 2019
		Jordan	28·2% (17·8–40·2%)	25·3% (-1·3 to 58·1%)	91·89 (54·66–139·43)	-49·8% (-63·6 to -30·7%)
		Kuwait	27·7% (18·3–39·5%)	14·6% (-6·1 to 41%)	68·31 (42·48–102·39)	9·8% (-19·4 to 49%)
		Lebanon	26·6% (16·8–38·6%)	58·9% (26 to 105·9%)	34·31 (19·07–56·99)	-42·7% (-61·6 to -14·7%)
		Libya	27·6% (17·3–39·9%)	57·3% (23·5 to 114·3%)	104 (57·93–163·49)	-23·1% (-47·2 to 16·2%)
		Morocco	28·8% (17·2–41·5%)	134·3% (85·9 to 195·1%)	159·46 (90·94–244·04)	15% (-21·6 to 69·3%)
		Palestine	32·9% (19·9–47·9%)	46·4% (20 to 87·2%)	160·67 (93·5–243·23)	-36·6% (-55·9 to -6·9%)
		Oman	29·4% (18–42·9%)	81·1% (41·3 to 134·3%)	148·16 (87·55–220·95)	-27·3% (-49·2 to 10%)
		Qatar	29·3% (19·2–42·8%)	3·8% (-14·3 to 28%)	71·34 (44·13–114)	-54·6% (-68·8 to -33·4%)
		Saudi Arabia	28·8% (18·8–40·7%)	38·6% (7·4 to 76·2%)	220·73 (135·31–329·71)	-18·5% (-46·5 to 23·6%)
		Syrian Arab Republic	26·9% (17·1–39·2%)	91·2% (51·6 to 144·3%)	226·81 (130·87–362·17)	-1·1% (-34·3 to 50%)
		Tunisia	28·9% (18·2–42%)	59·2% (25·9 to 97·7%)	93·11 (52·02–146·87)	-30·6% (-53·3 to 7·7%)
		Türkiye	20·6% (12·7–31·1%)	63·8% (31·7 to 112·2%)	82·05 (48·44–126·47)	3·3% (-32·2 to 50·2%)
		United Arab Emirates	29·1% (19·3–40·8%)	3·9% (-17 to 29·4%)	131·05 (73·7–220·16)	-62·7% (-75·3 to -42·9%)
		Yemen	19·7% (11·1–30·4%)	94·3% (48·8 to 154·7%)	174·22 (87·21–282·99)	-10% (-39·8 to 37·8%)
		Afghanistan	30·9% (18·8–44·3%)	89·8% (50·5 to 148·4%)	430·86 (233·25–680·35)	2·4% (-28·5 to 50%)
		Sudan	26·8% (16·2–38·8%)	113·2% (68·1 to 174·3%)	206·16 (111·72–332·58)	-16·1% (-41·7 to 21·2%)
	Subarachnoid haemorrhage	Global	17·3% (11·3–24·3%)	40·5% (30·8 to 52%)	23·57 (14·85–33·87)	-35·7% (-45·2 to -13·8%)
		North Africa and Middle East	19·8% (12·9–28·3%)	121·7% (80·7 to 199·7%)	18·61 (11·36–27·18)	-18·1% (-41·7 to 32·6%)
		Algeria	24·3% (15·8–34·9%)	90·2% (50·5 to 144%)	18·73 (10·74–28·91)	-32·9% (-56·4 to 9·5%)
		Bahrain	24·5% (15·4–35·7%)	29% (1·7 to 65·1%)	15·4 (8·99–25·83)	-46% (-66·6 to -18·1%)
		Egypt	12·1% (6·7–19·2%)	345·8% (161·4 to 889·4%)	13·79 (6·88–23·98)	3% (-46·1 to 116·4%)
		Iran	23·3% (14·8–33·6%)	70·2% (45·7 to 106·1%)	12·69 (7·83–18·27)	-25% (-49·2 to 22·3%)
		Iraq	23·5% (15·1–32·8%)	47·8% (15·1 to 86·2%)	16·91 (9·76–27·04)	-47·1% (-67·3 to -5·6%)
		Jordan	23·5% (15·4–33·3%)	23% (-1·4 to 53%)	9·16 (5·53–13·74)	-38·4% (-59·3 to -8·4%)
		Kuwait	25·4% (16·9–35·4%)	38·8% (14·7 to 68·9%)	11·94 (7·42–17·2)	-9·6% (-32·7 to 25·1%)
		Lebanon	22·3% (13·9–31·9%)	62·2% (24·1 to 111%)	5·95 (3·33–9·75)	-42·3% (-68·3 to 5·7%)
		Libya	22% (13·5–33·4%)	123·2% (49 to 266·7%)	15·76 (8·64–26·31)	-25·2% (-53·5 to 29·7%)
		Morocco	25·6% (15·6–38%)	143·7% (88·6 to 223·2%)	25·7 (13·56–41·84)	-0·2% (-37·9 to 87·8%)
		Palestine	27·4% (16·9–39·5%)	47·3% (17·5 to 92·5%)	13·74 (8·31–20·75)	-20·7% (-48·5 to 30·4%)
		Oman	21·3% (13–30·9%)	93·2% (44·6 to 167·3%)	19·18 (8·99–33·41)	-26·7% (-59·4 to 38·4%)
		Qatar	28·5% (18·2–40·9%)	17·2% (-7·7 to 49·2%)	23·81 (14·08–36·93)	-46·7% (-69·9 to -5·7%)
		Saudi Arabia	23·3% (15·2–33%)	40% (11·4 to 75·5%)	11·52 (6·55–17·79)	-33·7% (-64·4 to 18·3%)
		Syrian Arab Republic	13·8% (8·3–20·6%)	148·2% (72·6 to 285·6%)	15·1 (8·73–24·5)	-14·3% (-48·5 to 55·2%)
		Tunisia	24·8% (15·8–35·8%)	72·8% (34 to 125·4%)	15·99 (9·1–26·23)	-36·1% (-63·1 to 22·2%)
		Türkiye	17% (11–24·6%)	53% (26·8 to 96·1%)	17·13 (10·35–25·76)	-8·4% (-45·5 to 75·1%)
		United Arab Emirates	24·5% (15·8–34·4%)	18% (-6·5 to 49·7%)	14·93 (6·58–26·85)	-57·9% (-74·5 to -28%)
		Yemen	18·2% (10·4–28·6%)	98·6% (49·4 to 164·3%)	30·2 (12·22–61·89)	0·7% (-34·6 to 70·8%)
		Afghanistan	29·8% (17·9–43·7%)	90·9% (45·1 to 155·9%)	70·45 (21·02–143·13)	6·6% (-30·4 to 81·1%)
		Sudan	23·9% (14·3–35·1%)	121·3% (68·1 to 207·8%)	29·47 (13·35–54·84)	-21·4% (-50·9 to 56%)
	Neurological disorders*	Global	2·7% (0·5–7·4%)	35·8% (28·6 to 49·2%)	32·84 (5·53–105·88)	34% (28 to 47·7%)
		North Africa and Middle East	3·5% (0·6–9·7%)	66·6% (54·4 to 89·3%)	48·25 (8·67–151·54)	59·1% (47·1 to 88·7%)
		Algeria	4·3% (0·7–11·9%)	73·1% (50·1 to 114·4%)	58·32 (10·65–189·14)	56·6% (34·7 to 91·6%)
		Bahrain	6·9% (1·4–17·8%)	61·5% (34 to 111·8%)	94·76 (18·78–285·18)	47·2% (25·7 to 81·8%)
		Egypt	3% (0·5–8·4%)	93% (54·9 to 164·8%)	38·5 (6·66–124·65)	93·9% (59·1 to 165·6%)
		Iran	3·4% (0·6–9·4%)	78·9% (64 to 107·2%)	46·91 (8·43–146·09)	70·5% (57·1 to 113·5%)
		Iraq	4·3% (0·8–12·2%)	58% (36·1 to 93·9%)	56·16 (10·27–175·37)	54·7% (36·1 to 87·2%)
		Jordan	4·1% (0·7–11·5%)	34·4% (14·4 to 62·3%)	51·89 (9·32–162·62)	27·1% (9·2 to 57·1%)
		Kuwait	5·5% (1·1–14·5%)	38·9% (18·2 to 75·5%)	69·25 (13·22–210·56)	32% (17 to 58·8%)
		Lebanon	4·3% (0·8–11·8%)	58% (36·7 to 101%)	57·08 (10·16–187·23)	51·7% (32·9 to 109·5%)
		Libya	5% (0·9–13·4%)	65·6% (42·3 to 101·5%)	67·68 (12·26–213·84)	60·6% (35·8 to 100·8%)

Table S15 Burden of neurological conditions attributable to risk factors in North Africa and Middle East countries

			DALYs (95% UI)			
			PAF (%)	PAF change (%)	Rate per 100,000	Rate change (%)
Risk factors	Causes	Locations	2019	From 1990 to 2019	2019	From 1990 to 2019
		Morocco	3·8% (0·7–10·7%)	71·4% (48 to 109·1%)	50·39 (8·76–159·88)	74·8% (54·5 to 105·9%)
		Palestine	4·4% (0·8–12·1%)	69·1% (43·1 to 110·5%)	59·56 (10·71–192·14)	60% (35·4 to 100·6%)
		Oman	4·3% (0·8–11·6%)	57·9% (35 to 102·1%)	57·6 (10·31–187·17)	59·6% (36·1 to 117·3%)
		Qatar	7·4% (1·5–19·2%)	59·5% (23·7 to 133·5%)	100·36 (18·79–302·85)	56·9% (20·5 to 124·5%)
		Saudi Arabia	4·6% (0·9–12·6%)	47% (23·4 to 89·1%)	65·03 (12–204·81)	46·8% (25·6 to 93·8%)
		Syrian Arab Republic	4% (0·7–11·3%)	53·1% (31·6 to 89·8%)	52·33 (9·2–176·09)	55·7% (29·4 to 104·9%)
		Tunisia	4·6% (0·8–12·6%)	61·6% (39 to 98·5%)	60·55 (11·24–188·59)	57·5% (32·1 to 108·1%)
		Türkiye	2·9% (0·5–8·1%)	53·8% (28·7 to 90·4%)	42·17 (7·24–138·49)	39% (19·8 to 68·1%)
		United Arab Emirates	5% (1–13·4%)	39·6% (15·8 to 81·6%)	71·71 (12·97–221·63)	29·8% (9·7 to 67·1%)
		Yemen	2·9% (0·5–8·3%)	61·7% (38·9 to 98·7%)	37·64 (6·28–123·82)	57·5% (36·3 to 92·7%)
		Afghanistan	4·1% (0·7–11·4%)	82·1% (57·3 to 128·5%)	62·39 (10·88–199·36)	62·9% (42·7 to 91·9%)
		Sudan	3·7% (0·6–10·6%)	80·2% (55 to 123·4%)	49·93 (8·96–154·84)	66·7% (50·4 to 97·2%)
	Alzheimer's disease and other dementias	Global	9·7% (2·2–20·7%)	29·3% (24·7 to 39·7%)	32·84 (5·53–105·88)	34% (28 to 47·7%)
		North Africa and Middle East	12·4% (3–25·9%)	61% (52·2 to 77%)	48·25 (8·67–151·54)	59·1% (47·1 to 88·7%)
		Algeria	14·5% (3·6–29·8%)	65·2% (53 to 86·6%)	58·32 (10·65–189·14)	56·6% (34·7 to 91·6%)
		Bahrain	23·4% (6·7–44·2%)	53·3% (40 to 76·8%)	94·76 (18·78–285·18)	47·2% (25·7 to 81·8%)
		Egypt	10·6% (2·5–22·9%)	94·1% (66·6 to 152·9%)	38·5 (6·66–124·65)	93·9% (59·1 to 165·6%)
		Iran	12·1% (2·9–25·4%)	70·8% (60·3 to 88·1%)	46·91 (8·43–146·09)	70·5% (57·1 to 113·5%)
		Iraq	14·7% (3·7–30·1%)	52% (39·4 to 72·8%)	56·16 (10·27–175·37)	54·7% (36·1 to 87·2%)
		Jordan	14% (3·5–28·8%)	33·1% (22·6 to 51·6%)	51·89 (9·32–162·62)	27·1% (9·2 to 57·1%)
		Kuwait	17·8% (4·7–35·7%)	35·4% (24·2 to 58·3%)	69·25 (13·22–210·56)	32% (17 to 58·8%)
		Lebanon	14·4% (3·6–29·6%)	55·4% (45·5 to 72·7%)	57·08 (10·16–187·23)	51·7% (32·9 to 109·5%)
		Libya	16·3% (4·1–33%)	67·2% (54·9 to 88·9%)	67·68 (12·26–213·84)	60·6% (35·8 to 100·8%)
		Morocco	13% (3·1–27%)	74·5% (62 to 95·7%)	50·39 (8·76–159·88)	74·8% (54·5 to 105·9%)
		Palestine	15·3% (3·8–31%)	67% (51·7 to 95%)	59·56 (10·71–192·14)	60% (35·4 to 100·6%)
		Oman	13·6% (3·3–28·2%)	65% (52·6 to 93·6%)	57·6 (10·31–187·17)	59·6% (36·1 to 117·3%)
		Qatar	24·6% (6·6–46·8%)	51·8% (23·9 to 109·7%)	100·36 (18·79–302·85)	56·9% (20·5 to 124·5%)
		Saudi Arabia	16·7% (4·3–33·4%)	50·1% (40·6 to 69·4%)	65·03 (12–204·81)	46·8% (25·6 to 93·8%)
		Syrian Arab Republic	13·3% (3·3–27·9%)	51·1% (40·7 to 72·2%)	52·33 (9·2–176·09)	55·7% (29·4 to 104·9%)
		Tunisia	15·1% (3·8–30·6%)	61·2% (50·8 to 80·4%)	60·55 (11·24–188·59)	57·5% (32·1 to 108·1%)
		Türkiye	10·6% (2·5–22·5%)	41·7% (29·6 to 59·6%)	42·17 (7·24–138·49)	39% (19·8 to 68·1%)
		United Arab Emirates	19·5% (5·2–38·9%)	37·5% (23·3 to 66·1%)	71·71 (12·97–221·63)	29·8% (9·7 to 67·1%)
		Yemen	9·8% (2·2–20·7%)	57·8% (44·2 to 87·5%)	37·64 (6·28–123·82)	57·5% (36·3 to 92·7%)
		Afghanistan	14·4% (3·6–29·7%)	69·3% (57·3 to 89·4%)	62·39 (10·88–199·36)	62·9% (42·7 to 91·9%)
		Sudan	13·6% (3·4–28·3%)	73·7% (61·5 to 95·4%)	49·93 (8·96–154·84)	66·7% (50·4 to 97·2%)
High LDL cholesterol	Stroke	Global	9·6% (5·3–16·6%)	5·5% (0·1 to 13·4%)	170·21 (92·8–294·74)	-31·7% (-37·3 to -23·9%)
		North Africa and Middle East	15% (9·1–24·8%)	45·8% (34·3 to 56·5%)	274·69 (160·6–448·12)	-0·9% (-14 to 13·7%)
		Algeria	15·5% (8·3–26·8%)	49·8% (35 to 70%)	271·99 (137·65–493·84)	-16·8% (-32·6 to 5·3%)
		Bahrain	15·5% (7·5–28·1%)	15·3% (3·7 to 25·7%)	145·63 (68·11–271·24)	-45·2% (-55·6 to -34%)
		Egypt	15·3% (9·7–23·9%)	126·6% (69·6 to 178·3%)	325·37 (192·55–531·13)	46·2% (9·2 to 91%)
		Iran	18·6% (10·8–31%)	2·3% (-3·3 to 6·4%)	234·35 (135·13–399·73)	-44·4% (-50·7 to -37·5%)
		Iraq	14·2% (8·3–24·4%)	18·1% (6·1 to 28·7%)	415·34 (234·43–709·93)	-4·9% (-26·6 to 19·7%)
		Jordan	18·3% (9·8–31·8%)	13·7% (4·5 to 21·3%)	264·81 (137·4–468·41)	-43·4% (-53·7 to -32·7%)
		Kuwait	17·5% (10–29·5%)	0·6% (-8·3 to 8·9%)	164·01 (90·33–282·08)	-11% (-24·9 to 3·1%)
		Lebanon	21·6% (12·4–35·3%)	44·1% (31·7 to 63·9%)	162·52 (86·17–271·76)	-4·6% (-25·1 to 21%)
		Libya	17·6% (11·3–27·8%)	83% (56·5 to 115·5%)	277·11 (162·63–451·78)	43·8% (12·1 to 94·4%)
		Morocco	16·7% (9·8–28%)	53·2% (37·3 to 74·7%)	378·2 (204·88–661·87)	27·1% (-1·6 to 60·2%)
		Palestine	16·4% (8·5–30%)	22·8% (13·1 to 30·8%)	350·07 (174·72–627·82)	-17·1% (-34·8 to 4·3%)
		Oman	16% (7·8–29·3%)	43·4% (14·7 to 67·9%)	302·42 (146·31–570·66)	-12% (-36·5 to 17·2%)
		Qatar	13·2% (6–25·3%)	14·8% (-8·7 to 30·9%)	119·39 (51·45–239·46)	-36·5% (-53·2 to -19·2%)

Table S15 Burden of neurological conditions attributable to risk factors in North Africa and Middle East countries

Risk factors	Causes	Locations	DALYs (95% UI) PAF (%) 2019	PAF change (%) From 1990 to 2019	Rate per 100,000 2019	Rate change (%) From 1990 to 2019
		Saudi Arabia	14·7% (8·7–23·9%)	55·4% (36·9 to 82·2%)	310·63 (174·16–522·36)	10·2% (-18 to 52·2%)
		Syrian Arab Republic	12·9% (7·6–21·3%)	30·2% (16·2 to 42·3%)	259·54 (141·07–442·02)	-22·2% (-43 to 2·5%)
		Tunisia	17·2% (9·5–29·9%)	54·1% (39·1 to 75·8%)	254·39 (128·48–469·75)	16·1% (-12·4 to 56%)
		Türkiye	11·5% (5·9–20·8%)	6·1% (-8·1 to 16·8%)	133·35 (65·42–251·59)	-18·9% (-41·5 to 0·4%)
		United Arab Emirates	21·1% (12·9–34%)	48·9% (31·1 to 75·4%)	403·68 (232·63–670·13)	-19·7% (-39·3 to 9·6%)
		Yemen	14·4% (8·8–23·6%)	78·4% (57·1 to 107·4%)	399·06 (221·29–709·45)	34·7% (4·7 to 80·1%)
		Afghanistan	13·2% (8·3–20·4%)	80·5% (56·9 to 106·7%)	461·25 (265·09–763·51)	41·3% (8·1 to 83·8%)
		Sudan	13·6% (8·2–22·3%)	92·3% (65·4 to 122%)	351·21 (191·37–615·1)	28·8% (1·7 to 68·7%)
	Ischaemic stroke	Global	21·3% (11·7–37%)	-4·4% (-8·2 to 1·9%)	170·21 (92·8–294·74)	-31·7% (-37·3 to -23·9%)
		North Africa and Middle East	23·2% (13·9–38·2%)	8·5% (5 to 14·1%)	274·69 (160·6–448·12)	-0·9% (-14 to 13·7%)
		Algeria	21·3% (11·5–36·7%)	10·3% (2 to 23·8%)	271·99 (137·65–493·84)	-16·8% (-32·6 to 5·3%)
		Bahrain	23·7% (11·6–43%)	-2·5% (-12·1 to 3·9%)	145·63 (68·11–271·24)	-45·2% (-55·6 to -34%)
		Egypt	23·6% (15·1–37%)	28·3% (12·8 to 49·1%)	325·37 (192·55–531·13)	46·2% (9·2 to 91%)
		Iran	23·8% (13·9–39·8%)	-3% (-7·7 to 0·4%)	234·35 (135·13–399·73)	-44·4% (-50·7 to -37·5%)
		Iraq	22·7% (13·2–38·5%)	2·3% (-8·3 to 9·5%)	415·34 (234·43–709·93)	-4·9% (-26·6 to 19·7%)
		Jordan	24·4% (13·2–42·5%)	5·4% (-3·2 to 12·1%)	264·81 (137·4–468·41)	-43·4% (-53·7 to -32·7%)
		Kuwait	25·4% (14·4–42·8%)	1·1% (-6·5 to 6·5%)	164·01 (90·33–282·08)	-11% (-24·9 to 3·1%)
		Lebanon	27·2% (15·4–44·2%)	12·5% (3·4 to 27·5%)	162·52 (86·17–271·76)	-4·6% (-25·1 to 21%)
		Libya	24·7% (15·7–38·8%)	29·4% (16·4 to 50·4%)	277·11 (162·63–451·78)	43·8% (12·1 to 94·4%)
		Morocco	23·5% (13·7–39·3%)	7% (0·1 to 18·1%)	378·2 (204·88–661·87)	27·1% (-1·6 to 60·2%)
		Palestine	22% (11·4–40%)	0·8% (-7·2 to 6·7%)	350·07 (174·72–627·82)	-17·1% (-34·8 to 4·3%)
		Oman	23·4% (11·4–42·7%)	7·8% (-13·3 to 24·7%)	302·42 (146·31–570·66)	-12% (-36·5 to 17·2%)
		Qatar	20·7% (9·5–39·1%)	-1·7% (-19·2 to 9·9%)	119·39 (51·45–239·46)	-36·5% (-53·2 to -19·2%)
		Saudi Arabia	23·9% (14·3–39·3%)	33·4% (21·7 to 54·7%)	310·63 (174·16–522·36)	10·2% (-18 to 52·2%)
		Syrian Arab Republic	24·2% (14·4–39·7%)	3·8% (-4·7 to 11%)	259·54 (141·07–442·02)	-22·2% (-43 to 2·5%)
		Tunisia	23·3% (12·9–39·7%)	11·5% (3·9 to 25·3%)	254·39 (128·48–469·75)	16·1% (-12·4 to 56%)
		Türkiye	20·1% (10·3–36·3%)	-11·8% (-22·3 to -5·1%)	133·35 (65·42–251·59)	-18·9% (-41·5 to 0·4%)
		United Arab Emirates	28·6% (17·4–45·8%)	21·4% (10·5 to 42·2%)	403·68 (232·63–670·13)	-19·7% (-39·3 to 9·6%)
		Yemen	23·2% (14–38·1%)	12·4% (3·9 to 28·8%)	399·06 (221·29–709·45)	34·7% (4·7 to 80·1%)
		Afghanistan	24·8% (15·5–38·5%)	10·7% (2·3 to 24·4%)	461·25 (265·09–763·51)	41·3% (8·1 to 83·8%)
		Sudan	20·7% (12·2–34·5%)	18·9% (9·4 to 36·3%)	351·21 (191·37–615·1)	28·8% (1·7 to 68·7%)
High systolic blood pressure	Stroke	Global	54·8% (47·5–61·5%)	5·5% (1·3 to 10·1%)	969·4 (823·77–1110·2)	-31·7% (-37·9 to -25·5%)
		North Africa and Middle East	52·8% (45·7–59·4%)	10·5% (4·2 to 18·4%)	965·01 (800·6–1143·24)	-24·9% (-34·3 to -12%)
		Algeria	49·7% (40·9–58·5%)	-5·6% (-16·6 to 5·8%)	872·01 (662·49–1114·88)	-47·7% (-59·3 to -34·5%)
		Bahrain	52·1% (43·5–61·1%)	0% (-11·3 to 13·4%)	490·34 (383·49–626·76)	-52·6% (-62·5 to -40·1%)
		Egypt	49·9% (42–56·6%)	59·4% (30 to 92·2%)	1068·48 (757·4–1481·37)	3·2% (-24·4 to 37·1%)
		Iran	48·6% (40·7–56·1%)	5·6% (1·9 to 9·4%)	612·89 (506·32–718·14)	-42·7% (-48·8 to -33·6%)
		Iraq	62·3% (54·5–68·9%)	7·8% (0 to 17·7%)	1822·71 (1421·5–2274·29)	-13·1% (-33·1 to 10%)
		Jordan	52% (42·8–60·6%)	1·8% (-10·3 to 16·1%)	752·3 (585·1–924·87)	-49·4% (-60·1 to -37·2%)
		Kuwait	51% (43·1–59%)	1·5% (-8·1 to 12·5%)	478·76 (375·24–590·88)	-10·3% (-26·2 to 8·7%)
		Lebanon	51·4% (43·1–59·6%)	8·3% (-5·2 to 25·9%)	387·28 (281·36–503·42)	-28·3% (-45·9 to -6·1%)
		Libya	56·9% (48–64·3%)	14·5% (1·1 to 30·1%)	893·19 (668·38–1153·54)	-9·9% (-30·1 to 17·9%)
		Morocco	60·2% (51·3–68·3%)	-2·4% (-11·4 to 6·6%)	1360·9 (1030·45–1725·77)	-19·2% (-36·9 to 1·7%)
		Palestine	46% (36·9–54·7%)	-1·1% (-14 to 12·7%)	979·34 (759·63–1210·42)	-33·3% (-48·8 to -13·4%)
		Oman	52·3% (43·2–60·6%)	33·6% (15 to 58·7%)	986·42 (779·06–1209·09)	-18·2% (-38·9 to 14%)

Table S15 Burden of neurological conditions attributable to risk factors in North Africa and Middle East countries

			DALYs (95% UI)			
			PAF (%)	PAF change (%)	Rate per 100,000	Rate change (%)
Risk factors	Causes	Locations	2019	From 1990 to 2019	2019	From 1990 to 2019
		Qatar	51·4% (42·9–59·9%)	0·3% (-11·1 to 12·7%)	465·01 (351·86–606·99)	-44·5% (-58 to -27·8%)
		Saudi Arabia	53·5% (45·7–61%)	6·5% (-3·8 to 19%)	1131·89 (852·23–1403·64)	-24·5% (-44·5 to 3·1%)
		Syrian Arab Republic	50·6% (42·6–58%)	9·3% (-3·5 to 24·1%)	1021·36 (756·66–1342·5)	-34·7% (-52·5 to -9·3%)
		Tunisia	48·9% (40·2–57·2%)	3·8% (-9·5 to 18·6%)	723·1 (510·79–965·68)	-21·8% (-42·9 to 6·4%)
		Türkiye	52·8% (44·6–60·5%)	-0·4% (-8·9 to 8·4%)	614·52 (476·44–763·74)	-23·9% (-44·4 to -4·9%)
		United Arab Emirates	52·3% (43·6–60·1%)	-13% (-20·4 to -5·9%)	1008·2 (732·4–1350·36)	-53% (-65 to -38·2%)
		Yemen	48·6% (40·3–56·3%)	4·2% (-10·7 to 22%)	1344·79 (963·01–1816·73)	-21·4% (-42 to 7·3%)
		Afghanistan	50·2% (41·7–58·3%)	0·6% (-12·2 to 17·4%)	1759·25 (1176·54–2416·55)	-21·2% (-42·2 to 3·7%)
		Sudan	61·6% (54–68·4%)	10·7% (0·3 to 23·6%)	1592·13 (1151·53–2261·07)	-26% (-41·5 to -4·4%)
	Ischaemic stroke	Global	50·5% (40·3–60%)	0·3% (-3·6 to 4·5%)	403·74 (318·26–487·77)	-28·3% (-34·4 to -22·1%)
		North Africa and Middle East	51·3% (41·6–60·2%)	8·4% (4·2 to 13·7%)	607·76 (477·75–734·53)	-1·1% (-13·7 to 13·3%)
		Algeria	47·9% (37·2–59%)	-3% (-14·3 to 10%)	611·38 (445·22–804·97)	-27% (-42·9 to -6·3%)
		Bahrain	49·4% (38·5–61·2%)	-0·2% (-13·5 to 14·3%)	302·92 (225·87–407·53)	-44% (-55·7 to -29·8%)
		Egypt	51% (40·9–59·5%)	39·4% (18·2 to 68·1%)	704·79 (490·52–973·72)	59% (13·3 to 113·7%)
		Iran	47·4% (37·9–56·5%)	5·4% (2 to 9·5%)	466·36 (369·17–559·51)	-39·7% (-46·5 to -30·5%)
		Iraq	59·7% (49–69%)	7·9% (-0·4 to 19·3%)	1091·5 (829·66–1356·28)	0·4% (-20·5 to 27%)
		Jordan	50% (39·2–60·5%)	2·4% (-10·6 to 17·8%)	541·9 (403·77–685·87)	-45% (-56·8 to -31·7%)
		Kuwait	49·3% (38·6–59·7%)	-0·5% (-10·8 to 11·6%)	318·54 (236·02–406·48)	-12·4% (-28 to 5·6%)
		Lebanon	49·5% (39·6–58·8%)	11·2% (-3·3 to 29·5%)	295·92 (205·28–388·42)	-5·6% (-28·6 to 21·6%)
		Libya	56% (45·4–65%)	8·8% (-2·1 to 22·6%)	628·9 (458·21–827·55)	21% (-6 to 56·9%)
		Morocco	58·3% (47·3–68·3%)	-0·8% (-9·8 to 8·7%)	935·28 (694·8–1203·55)	17·5% (-9·1 to 46·3%)
		Palestine	44·2% (33·7–54·7%)	0·5% (-13·5 to 16·1%)	702·63 (515·7–893·99)	-17·4% (-36·7 to 7·1%)
		Oman	50·6% (39·5–60·8%)	35% (14·4 to 61·8%)	652·76 (492·96–821·83)	10·2% (-19 to 52·7%)
		Qatar	48·6% (37·6–60·2%)	-0·4% (-13·2 to 13·6%)	280·61 (203·62–378·05)	-35·8% (-50·9 to -17·4%)
		Saudi Arabia	50·5% (40·3–60·2%)	9·6% (-2·2 to 24·5%)	657·22 (476·95–832·66)	-9·5% (-32·6 to 18·4%)
		Syrian Arab Republic	48·9% (38·1–58·5%)	5·3% (-7·8 to 20·8%)	523·13 (371·52–699·89)	-21·1% (-44 to 8·3%)
		Tunisia	46·7% (36·3–56·7%)	6·3% (-7·4 to 22%)	509·45 (351·47–692·66)	10·6% (-20·4 to 50·5%)
		Türkiye	49·3% (39–59·2%)	-2% (-11·1 to 7·5%)	326·85 (242·04–422·27)	-9·9% (-35·1 to 13·2%)
		United Arab Emirates	50·2% (40·3–59·7%)	-12·2% (-19·5 to -4·5%)	710·89 (520·32–916·74)	-42% (-56 to -25%)
		Yemen	46% (35·7–56·1%)	7·5% (-8 to 27·1%)	790·54 (544·06–1095·57)	28·8% (-2·6 to 76·1%)
		Afghanistan	46·7% (36·2–56·3%)	4% (-10·2 to 20·6%)	872·47 (576·96–1229·51)	32·6% (-2 to 71·2%)
		Sudan	59·8% (49·2–68·9%)	15·3% (4·2 to 29·2%)	1012·82 (706·92–1466·28)	24·5% (-0·8 to 57·4%)
	Intracerebral haemorrhage	Global	58·8% (49·4–67·1%)	9·5% (5 to 14·7%)	489·23 (406–573·54)	-30·7% (-39·1 to -23·5%)
		North Africa and Middle East	56·4% (47·4–64·3%)	12·1% (5·3 to 23·8%)	309·15 (247·83–378·03)	-45·9% (-54·5 to -33·5%)
		Algeria	54·5% (44·1–64%)	-4·2% (-14·4 to 6·1%)	219·93 (157·97–290·58)	-69·3% (-77·2 to -59·2%)
		Bahrain	58·1% (47·8–68·2%)	0·7% (-9·7 to 13·5%)	153·87 (113·91–198·99)	-63·9% (-72·1 to -52·3%)
		Egypt	49·7% (40·8–58·6%)	54·8% (26·3 to 100%)	320·23 (216·65–473·04)	-37·1% (-57·2 to -8·3%)
		Iran	52·9% (44·3–60·9%)	8·3% (3·7 to 15%)	117·98 (98·25–137·97)	-49·8% (-57·7 to -40·3%)
		Iraq	66·9% (57·5–74·8%)	9·1% (1·4 to 19%)	684·39 (519·1–889·17)	-23·6% (-43·4 to 2·1%)
		Jordan	57·7% (47·6–67·7%)	2·9% (-8·6 to 17%)	188·07 (140·79–233·89)	-58·8% (-68·4 to -47·1%)
		Kuwait	55% (45·6–64·2%)	3·2% (-6·2 to 13·4%)	135·45 (100·62–172·99)	-1·1% (-24 to 24·2%)
		Lebanon	58·6% (49·4–67·4%)	10·7% (-0·6 to 25%)	75·29 (51·2–107·13)	-60·2% (-71·4 to -42·5%)
		Libya	60·2% (50·9–68·5%)	15·7% (1·9 to 36·2%)	226·8 (163·46–313·28)	-43·6% (-59·3 to -19·9%)
		Morocco	65·3% (55–74·5%)	-0·4% (-8·3 to 7·7%)	361·8 (259·52–481·53)	-51·3% (-65 to -32·3%)

Table S15 Burden of neurological conditions attributable to risk factors in North Africa and Middle East countries

Risk factors	Causes	Locations	DALYs (95% UI) PAF (%) 2019	PAF change (%) From 1990 to 2019	Rate per 100,000 2019	Rate change (%) From 1990 to 2019
		Palestine	51·6% (41·3–61·7%)	1·7% (-9·8 to 13·8%)	251·43 (191·58–318·03)	-56% (-67·1 to -41·3%)
		Oman	57·4% (46·9–66·9%)	34·3% (15·4 to 59·5%)	289·41 (225·42–373·93)	-46·2% (-61 to -22·5%)
		Qatar	56·2% (46–66·6%)	3·2% (-7·5 to 15·7%)	137·13 (98·77–182·83)	-54·9% (-68·2 to -38·7%)
		Saudi Arabia	58·6% (48·3–68·2%)	6·2% (-3·7 to 17·6%)	448·16 (329·14–577·75)	-37·8% (-55·9 to -7·2%)
		Syrian Arab Republic	55·1% (45·2–64·7%)	7·7% (-4·2 to 22·3%)	462·41 (335·25–627·51)	-44·5% (-61·2 to -20·7%)
		Tunisia	55·2% (45·7–64·8%)	7·3% (-5 to 22·2%)	178·17 (124·68–248·07)	-53·4% (-68·3 to -30·3%)
		Türkiye	57·6% (47·1–66·9%)	3·3% (-5·5 to 13·6%)	230·01 (169·74–294·58)	-34·9% (-54·7 to -15·5%)
		United Arab Emirates	58·5% (47·8–67·9%)	-11·2% (-19·1 to -4%)	263·65 (174·84–410·7)	-68·1% (-78·7 to -53·9%)
		Yemen	53·1% (42·4–62·5%)	7·1% (-7 to 25·2%)	469·85 (320·69–655·95)	-50·4% (-64·5 to -29·3%)
		Afghanistan	54·5% (43·3–63·8%)	3·5% (-11·2 to 19·6%)	761·9 (490·56–1065·05)	-44·1% (-60·3 to -23·8%)
		Sudan	65·3% (55·8–73·3%)	10·7% (1 to 25·4%)	503·13 (334·51–713·61)	-56·5% (-67·4 to -40·4%)
	Subarachnoid haemorrhage	Global	56% (47·4–64·4%)	10·6% (4·7 to 17·6%)	76·43 (61·04–91·63)	-49·3% (-57·2 to -31·7%)
		North Africa and Middle East	51·1% (42·6–59·1%)	30·7% (11·8 to 67·3%)	48·11 (37·27–61·41)	-51·5% (-65·5 to -23·8%)
		Algeria	52·9% (42·5–62·5%)	0·1% (-12·6 to 15·3%)	40·69 (28·91–55·75)	-64·6% (-75·7 to -44%)
		Bahrain	53·4% (44·5–62·3%)	21·4% (5·1 to 42·7%)	33·55 (23·97–48·78)	-49·1% (-65·7 to -25·9%)
		Egypt	38% (27·4–50·2%)	125·8% (49·8 to 339·5%)	43·46 (28·51–64·68)	-47·8% (-69·6 to -3·3%)
		Iran	52·3% (43·9–60·4%)	6% (-0·5 to 13%)	28·55 (22·95–35·43)	-53% (-66·9 to -26·9%)
		Iraq	64·9% (55·2–73·3%)	14·2% (3·5 to 28·7%)	46·81 (33·36–66·33)	-58·9% (-73·6 to -33·5%)
		Jordan	57·4% (47·2–67%)	4·4% (-8 to 20·1%)	22·33 (16·51–28·51)	-47·6% (-62·8 to -23·7%)
		Kuwait	52·7% (43·7–61·7%)	14·4% (1·1 to 29·4%)	24·76 (18·61–31·41)	-25·5% (-42·3 to 2·4%)
		Lebanon	60·2% (49·7–69·3%)	21·4% (6·6 to 43·1%)	16·08 (10·65–23·56)	-56·7% (-74·9 to -28·3%)
		Libya	52·3% (42·1–63%)	59·3% (13·7 to 135·2%)	37·5 (25·67–56·09)	-46·3% (-63·7 to -16·7%)
		Morocco	63·7% (53·6–73·4%)	5·6% (-6·3 to 20·6%)	63·82 (43·93–94·3)	-56·5% (-72·2 to -24·1%)
		Palestine	50·5% (40·6–59·7%)	1% (-11·6 to 15%)	25·28 (18·92–32·83)	-45·5% (-62·6 to -19·8%)
		Oman	49·2% (40·4–59·9%)	54·9% (26·3 to 101·1%)	44·25 (26·27–71·26)	-41·5% (-65·4 to 12%)
		Qatar	56·5% (47–66·3%)	6·5% (-6·4 to 21·8%)	47·28 (32·85–65·55)	-51·3% (-71·7 to -20·1%)
		Saudi Arabia	53·5% (42·8–64·1%)	4·6% (-10·1 to 20·3%)	26·51 (18·1–36·43)	-50·4% (-72·4 to -16·5%)
		Syrian Arab Republic	32·8% (25·7–40·8%)	53·8% (13·6 to 120·6%)	35·82 (25·22–49·53)	-47% (-67 to -11%)
		Tunisia	55% (45·6–64·6%)	15·5% (-1·3 to 36·3%)	35·48 (24·48–50·9)	-56·9% (-74·3 to -24·9%)
		Türkiye	57·1% (47·3–66·2%)	4·9% (-5 to 15·8%)	57·67 (42·93–74·02)	-37% (-61 to 13%)
		United Arab Emirates	55·1% (45·3–65·3%)	0·3% (-11·6 to 15·4%)	33·67 (16·6–56·74)	-64·2% (-78·3 to -42%)
		Yemen	51·4% (40·5–61·1%)	11% (-6·4 to 33·5%)	84·4 (43·47–143·95)	-43·6% (-62·6 to -8·1%)
		Afghanistan	53·2% (42·2–63·7%)	4·7% (-12·5 to 25·7%)	124·88 (42·15–222·29)	-41·1% (-60·5 to -6·2%)
		Sudan	62·1% (51·3–71%)	16·9% (-0·5 to 45%)	76·18 (42·96–130·2)	-57·9% (-72·9 to -20·8%)
Kidney dysfunction	Stroke	Global	8·2% (6·8–9·6%)	16·6% (12 to 20%)	145·06 (117·59–171·05)	-24·5% (-31·7 to -17·9%)
		North Africa and Middle East	11% (8·7–13·3%)	51·9% (38·8 to 65·2%)	200·82 (155·56–247·94)	3·2% (-12·2 to 21·1%)
		Algeria	10·7% (7·6–13·5%)	43% (24·4 to 57·8%)	188·19 (124·69–256·26)	-20·7% (-38·1 to 1·3%)
		Bahrain	11·9% (8·6–14·7%)	35·5% (21·6 to 46·7%)	111·63 (78·36–152·79)	-35·6% (-49·2 to -20·8%)
		Egypt	11·1% (8·9–13·4%)	108·5% (67·1 to 145·4%)	237·49 (167·07–335·23)	34·9% (0·4 to 75·2%)
		Iran	11·1% (8·3–13·6%)	18·5% (11 to 24·8%)	139·5 (105·24–173·85)	-35·6% (-44·1 to -25%)
		Iraq	12·6% (10–14·9%)	40% (29·9 to 49·5%)	367·22 (272·9–460·37)	12·7% (-11·3 to 40·4%)
		Jordan	12·9% (9·8–15·8%)	33·1% (22·1 to 42·5%)	187·57 (135·73–240·01)	-33·7% (-47·2 to -20·5%)
		Kuwait	11·1% (8·4–13·6%)	24·9% (15·7 to 34·7%)	104·36 (74·35–135·01)	10·3% (-7·1 to 31·3%)
		Lebanon	12% (8·8–14·8%)	48·5% (34·3 to 61·8%)	90·21 (60·13–122·12)	-1·5% (-25 to 25·7%)
		Libya	11·4% (9–13·6%)	58% (41·6 to 76·5%)	179·34 (124·98–238·19)	24·2% (-4·8 to 62·4%)
		Morocco	11·6% (9–14%)	65·8% (47·6 to 82·6%)	260·78 (184·99–334·16)	37·1% (3·5 to 72·3%)
		Palestine	12·7% (9·5–15·5%)	34·9% (22·5 to 44·6%)	269·78 (196–347·03)	-9% (-29·2 to 14·8%)
		Oman	11·9% (8·9–14·4%)	56·5% (37·2 to 74·4%)	223·97 (163·82–287·15)	-4% (-27·2 to 30·1%)
		Qatar	11·1% (7·8–14·1%)	30·9% (14·4 to 43·6%)	100·24 (65·75–137·53)	-27·5% (-46·5 to -4·4%)
		Saudi Arabia	13·5% (10·7–16·1%)	47·8% (37·6 to 58·6%)	285·82 (207·98–363·39)	4·8% (-21·4 to 39·7%)

Table S15 Burden of neurological conditions attributable to risk factors in North Africa and Middle East countries

			DALYs (95% UI)			
			PAF (%)	PAF change (%)	Rate per 100,000	Rate change (%)
Risk factors	Causes	Locations	2019	From 1990 to 2019	2019	From 1990 to 2019
		Syrian Arab Republic	10·5% (8·2–12·5%)	45% (31·4 to 58·4%)	212·3 (151·84–282·21)	-13·3% (-37·4 to 19·3%)
		Tunisia	11·5% (8·6–14·2%)	52% (35·8 to 67·4%)	169·5 (117·61–232·46)	14·4% (-17·3 to 53·7%)
		Türkiye	10·5% (8–12·8%)	55% (37·8 to 71·6%)	121·57 (86·72–160·08)	18·5% (-14·6 to 52%)
		United Arab Emirates	13% (10·2–15·8%)	33·1% (23·2 to 43·4%)	251·38 (174·65–342·61)	-28·1% (-45·5 to -6·6%)
		Yemen	9·4% (7·5–11·4%)	40·8% (26·4 to 57%)	260·75 (184·85–351·87)	6·2% (-18·3 to 40·9%)
		Afghanistan	9·9% (8·1–11·6%)	37·6% (25·2 to 50·7%)	345·8 (241·46–470·41)	7·6% (-17·3 to 35·5%)
		Sudan	10·1% (8–12·2%)	51·9% (34·3 to 71·1%)	260·13 (182·49–369·4)	1·6% (-18·7 to 26·9%)
	Ischaemic stroke	Global	9% (6·7–11·3%)	13·6% (11·5 to 16·8%)	72·17 (52·71–93·11)	-18·8% (-26·1 to -11·9%)
		North Africa and Middle East	12·5% (9·2–15·7%)	38·8% (28·4 to 47·9%)	148·49 (107·88–190)	26·7% (6 to 45·3%)
		Algeria	11·5% (7·4–15·2%)	37·3% (21·8 to 50·1%)	147·26 (89·51–207·24)	3·5% (-19·5 to 30·1%)
		Bahrain	13% (8·5–17%)	30·7% (16·4 to 41·7%)	80·06 (50·76–112·57)	-26·5% (-42·4 to -10·1%)
		Egypt	13·3% (10·2–16·4%)	64·6% (48·6 to 85·3%)	184·51 (127·18–262·21)	87·6% (37·8 to 138·1%)
		Iran	12% (8·6–15·2%)	16·1% (9·2 to 21·5%)	118·34 (84·35–151·74)	-33·5% (-41·9 to -22·5%)
		Iraq	14·1% (10·5–17·6%)	34·5% (24·1 to 43·9%)	258·3 (184·52–337·15)	25·2% (-2·4 to 54·5%)
		Jordan	13·7% (9·7–17·3%)	31·7% (20·1 to 41·4%)	148·9 (99·88–195·47)	-29·2% (-44·3 to -14·9%)
		Kuwait	12·4% (8·7–15·7%)	22·8% (13·3 to 33·1%)	80·01 (54·67–106·58)	8·1% (-9·4 to 29%)
		Lebanon	12·9% (8·9–16·3%)	43·1% (30 to 56·2%)	76·87 (48·13–104·82)	21·5% (-9·3 to 51·3%)
		Libya	12·9% (9·7–16%)	40·9% (30·9 to 52·5%)	144·97 (98·54–195·88)	56·6% (20·5 to 103·6%)
		Morocco	12·7% (9·4–15·8%)	54·8% (39·3 to 69%)	203·89 (138·16–267·25)	83·5% (39·7 to 128·5%)
		Palestine	13·4% (9·2–17%)	31% (20·5 to 40·1%)	213·23 (143·3–282·63)	7·6% (-15·6 to 36·2%)
		Oman	13% (9·1–16·5%)	47·7% (29·8 to 62·6%)	167·69 (113·94–224·14)	20·7% (-9·7 to 61·9%)
		Qatar	12·4% (7·6–16·7%)	22·7% (4 to 34·3%)	71·73 (41·05–102·59)	-20·7% (-41·7 to 3·5%)
		Saudi Arabia	15% (10·8–18·7%)	47·7% (36·4 to 60·3%)	194·76 (132·07–257·63)	22·1% (-8·9 to 58·3%)
		Syrian Arab Republic	12·1% (8·6–15·3%)	34% (20·6 to 45·9%)	129·83 (85·2–179·55)	0·4% (-27·8 to 36·6%)
		Tunisia	12·5% (8·6–15·9%)	43·2% (30·9 to 55·4%)	136·1 (89·41–190·04)	49% (10·5 to 96·5%)
		Türkiye	11·9% (8–15·6%)	46·8% (28·7 to 63·8%)	79·18 (51·63–110·43)	35·2% (-3·4 to 72·9%)
		United Arab Emirates	14·3% (10·6–17·8%)	30·1% (21·5 to 39·5%)	202·2 (136·83–278·44)	-13·9% (-34·1 to 10·8%)
		Yemen	10·7% (8·1–13·4%)	35% (24·1 to 47·7%)	184·54 (124·81–258·6)	61·7% (26·3 to 109·8%)
		Afghanistan	11·6% (8·9–14·3%)	32·8% (23 to 41·8%)	216·38 (143·6–305·49)	69% (30·9 to 113·7%)
		Sudan	11·4% (8·4–14·2%)	42·2% (29·2 to 55·3%)	192·07 (125·5–285·43)	53·5% (22·9 to 91·5%)
	Intracerebral haemorrhage	Global	8·8% (7·3–10·4%)	11·4% (8·6 to 14·7%)	72·89 (59·13–88·21)	-29·4% (-37·3 to -22·6%)
		North Africa and Middle East	9·5% (7·8–11·5%)	40·2% (28·7 to 58·8%)	52·33 (41·03–64·92)	-32·4% (-42·9 to -15·8%)
		Algeria	10·2% (8–12·3%)	34·2% (21·5 to 48·6%)	40·93 (29·18–54·23)	-57% (-67·6 to -42%)
		Bahrain	11·9% (9·3–14·5%)	37·1% (23 to 50·5%)	31·57 (22·6–42·16)	-50·9% (-62·8 to -35·3%)
		Egypt	8·2% (6·4–10·3%)	67·6% (37·3 to 120·1%)	52·98 (33·59–82·97)	-31·9% (-53·4 to -1·3%)
		Iran	9·5% (7·7–11·4%)	17·7% (10·3 to 27·4%)	21·16 (17·07–25·7)	-45·5% (-54 to -34·3%)
		Iraq	10·7% (8·6–12·9%)	30·5% (20·2 to 42·1%)	108·91 (79·5–143·79)	-8·7% (-32·3 to 20·6%)
		Jordan	11·9% (9·4–14·5%)	33% (21·5 to 45·4%)	38·68 (28·5–50·68)	-46·8% (-57·9 to -32·5%)
		Kuwait	9·9% (8–11·9%)	23·8% (14·3 to 34·9%)	24·35 (17·9–32·31)	18·5% (-6·9 to 49·2%)
		Lebanon	10·4% (8·3–12·5%)	30·9% (19·9 to 42·8%)	13·34 (8·6–19·09)	-52·9% (-66 to -33·9%)
		Libya	9·1% (7·4–11%)	36·2% (21 to 58·7%)	34·37 (24·37–48·12)	-33·7% (-51·5 to -8·5%)
		Morocco	10·3% (8·3–12·5%)	47·2% (33·4 to 63%)	56·89 (40·62–76·21)	-28% (-48·1 to -0·2%)
		Palestine	11·6% (9·1–14·1%)	32·8% (22·1 to 43·3%)	56·55 (41·79–73·38)	-42·5% (-56·7 to -23·2%)
		Oman	11·2% (8·7–13·7%)	48·4% (30·9 to 67·6%)	56·28 (40·92–74·23)	-40·5% (-56·4 to -15·6%)
		Qatar	11·7% (8·6–14·7%)	36·6% (15·9 to 56·9%)	28·51 (19·3–40·18)	-40·3% (-57·9 to -16·1%)
		Saudi Arabia	11·9% (9·6–14·5%)	37·2% (27·1 to 48·5%)	91·06 (64·87–118·65)	-19·5% (-42·4 to 17·2%)
		Syrian Arab Republic	9·8% (7·9–12%)	38·5% (25·1 to 54%)	82·47 (55·53–112·21)	-28·7% (-50·5 to 1%)
		Tunisia	10·4% (8·3–12·5%)	35·2% (24·4 to 47·4%)	33·39 (23·19–46·69)	-41·2% (-59·2 to -12·6%)
		Türkiye	10·6% (8·4–12·9%)	52·8% (34·4 to 73·5%)	42·39 (30·72–56·46)	-3·6% (-31·2 to 27·6%)
		United Arab Emirates	10·9% (8·8–13·4%)	19·4% (9·3 to 29·1%)	49·18 (32·14–74·66)	-57·2% (-70·1 to -38·9%)
		Yemen	8·6% (7–10·4%)	25·4% (15·2 to 36·8%)	76·22 (53·3–104·4)	-42% (-57·6 to -19·2%)

Table S15 Burden of neurological conditions attributable to risk factors in North Africa and Middle East countries

			DALYs (95% UI)			
			PAF (%)	PAF change (%)	Rate per 100,000	Rate change (%)
Risk factors	Causes	Locations	2019	From 1990 to 2019	2019	From 1990 to 2019
		Afghanistan	9·3% (7·6–11·2%)	24·1% (16·8 to 31·9%)	129·42 (85·17–182·27)	-33·1% (-50·3 to -12·4%)
		Sudan	8·9% (7·2–10·7%)	32·3% (20·2 to 52·4%)	68·05 (45·31–96·97)	-48·1% (-60·5 to -29·5%)
Low physical activity	Stroke	Global	1·8% (0·3–4·7%)	10·5% (2·8 to 22·1%)	31·16 (5·69–82·02)	-28·4% (-34·3 to -19·7%)
		North Africa and Middle East	5% (1·1–11·2%)	48·9% (35·5 to 63%)	91·71 (18·75–203·41)	1·2% (-11·8 to 16%)
		Algeria	6·3% (1·4–13·5%)	43·9% (29·3 to 68·6%)	110·29 (23·46–231·6)	-20·2% (-35·4 to 0·4%)
		Bahrain	6% (1·3–12·3%)	30·1% (18·9 to 51·3%)	56·44 (12·52–117·47)	-38·3% (-49·3 to -22·7%)
		Egypt	4·7% (1–10·8%)	110·6% (58 to 150·8%)	100·02 (17·45–238·36)	35·8% (-1·8 to 74·6%)
		Iran	5% (1–12·4%)	11·7% (4·9 to 23%)	63·38 (12·02–154·69)	-39·3% (-47·1 to -27·3%)
		Iraq	5·3% (1·2–11·5%)	24·8% (12·4 to 44·4%)	155·48 (34·19–351·11)	0·4% (-19·5 to 25·3%)
		Jordan	5·2% (1·1–11·9%)	26·8% (12·1 to 56·8%)	75·02 (15·32–171·38)	-36·9% (-51·2 to -17·5%)
		Kuwait	6·8% (1·5–13·9%)	3·9% (-6·5 to 17·2%)	64·03 (14·5–129·68)	-8·2% (-22·2 to 9·2%)
		Lebanon	6·6% (1·4–14·4%)	38·3% (22·1 to 59·1%)	49·61 (10·08–109·74)	-8·1% (-32·5 to 15·5%)
		Libya	5·7% (1·2–12·7%)	52·2% (30·8 to 71·8%)	89·11 (17·69–202·67)	19·4% (-7·3 to 53·5%)
		Morocco	5·6% (1·2–12·4%)	56·7% (38·3 to 83·8%)	126·47 (25·16–277·94)	29·5% (2 to 60·6%)
		Palestine	5·8% (1·3–12·7%)	29·8% (17·3 to 52·2%)	122·92 (26·53–276·52)	-12·3% (-30·1 to 12·7%)
		Oman	6% (1·3–12·6%)	71·1% (42·5 to 127·2%)	113·26 (24·78–235·79)	4·9% (-21·8 to 54·9%)
		Qatar	6·4% (1·5–13%)	31·8% (16·6 to 54·9%)	57·81 (13·46–118·27)	-27·1% (-44·5 to -1·1%)
		Saudi Arabia	7·5% (1·9–14·3%)	44·1% (22·8 to 92·4%)	159·92 (40·88–298·44)	2·3% (-24·2 to 50·2%)
		Syrian Arab Republic	4·3% (0·9–9·4%)	39·3% (20·7 to 62·4%)	86·09 (17·45–195·79)	-17·1% (-39 to 11·1%)
		Tunisia	2·8% (0·4–7·6%)	57·7% (36·3 to 94%)	40·64 (6·08–115·44)	19·1% (-13 to 59·9%)
		Türkiye	4% (0·8–9·1%)	38% (18·5 to 70·9%)	46·37 (9·07–110·26)	6·1% (-24 to 42·7%)
		United Arab Emirates	8% (1·9–16%)	29·7% (15·4 to 43·9%)	153·25 (34·19–310·26)	-29·9% (-46·5 to -9·7%)
		Yemen	4·3% (0·9–10%)	61% (32·8 to 87·1%)	117·38 (23·34–274·93)	21·4% (-4·2 to 54·6%)
		Afghanistan	3·7% (0·8–8·9%)	68·2% (42·2 to 99·1%)	129·89 (25·13–306·86)	31·8% (2·6 to 64·1%)
		Sudan	7·9% (2–14·6%)	74% (51·9 to 96·4%)	203·86 (52·24–391·56)	16·4% (-5·1 to 43·8%)
	Ischaemic stroke	Global	3·9% (0·7–10·4%)	0% (-5·9 to 9·8%)	31·16 (5·69–82·02)	-28·4% (-34·3 to -19·7%)
		North Africa and Middle East	7·7% (1·7–17·2%)	10·7% (4·5 to 20·3%)	91·71 (18·75–203·41)	1·2% (-11·8 to 16%)
		Algeria	8·7% (1·9–18·4%)	5·9% (-3·4 to 22·3%)	110·29 (23·46–231·6)	-20·2% (-35·4 to 0·4%)
		Bahrain	9·2% (2·1–18·8%)	10% (2·4 to 25·6%)	56·44 (12·52–117·47)	-38·3% (-49·3 to -22·7%)
		Egypt	7·2% (1·5–16·7%)	18·6% (2·7 to 36·8%)	100·02 (17·45–238·36)	35·8% (-1·8 to 74·6%)
		Iran	6·4% (1·2–15·7%)	5·8% (0·6 to 16·7%)	63·38 (12·02–154·69)	-39·3% (-47·1 to -27·3%)
		Iraq	8·5% (1·9–18·3%)	8% (-0·2 to 23·4%)	155·48 (34·19–351·11)	0·4% (-19·5 to 25·3%)
		Jordan	6·9% (1·5–15·9%)	17·6% (4·4 to 44·6%)	75·02 (15·32–171·38)	-36·9% (-51·2 to -17·5%)
		Kuwait	9·9% (2·2–20·1%)	4·4% (-4·4 to 16·3%)	64·03 (14·5–129·68)	-8·2% (-22·2 to 9·2%)
		Lebanon	8·3% (1·7–18%)	7·8% (-2·9 to 22·6%)	49·61 (10·08–109·74)	-8·1% (-32·5 to 15·5%)
		Libya	8% (1·7–17·6%)	7·5% (-3·2 to 19%)	89·11 (17·69–202·67)	19·4% (-7·3 to 53·5%)
		Morocco	7·9% (1·7–17·4%)	9·2% (-0·1 to 25·9%)	126·47 (25·16–277·94)	29·5% (2 to 60·6%)
		Palestine	7·7% (1·7–16·9%)	6·6% (-2 to 23·6%)	122·92 (26·53–276·52)	-12·3% (-30·1 to 12·7%)
		Oman	8·8% (1·9–18·2%)	28·6% (7·9 to 69·1%)	113·26 (24·78–235·79)	4·9% (-21·8 to 54·9%)
		Qatar	10% (2·4–20·2%)	12·9% (2·3 to 32·3%)	57·81 (13·46–118·27)	-27·1% (-44·5 to -1·1%)
		Saudi Arabia	12·3% (3·1–23%)	23·7% (8·5 to 63·2%)	159·92 (40·88–298·44)	2·3% (-24·2 to 50·2%)
		Syrian Arab Republic	8·1% (1·8–17·7%)	10·9% (1·6 to 27·9%)	86·09 (17·45–195·79)	-17·1% (-39 to 11·1%)
		Tunisia	3·7% (0·6–10·3%)	14·1% (2·6 to 38·7%)	40·64 (6·08–115·44)	19·1% (-13 to 59·9%)
		Türkiye	7% (1·4–16%)	14·8% (1·8 to 38·7%)	46·37 (9·07–110·26)	6·1% (-24 to 42·7%)
		United Arab Emirates	10·8% (2·5–21·5%)	5·8% (-1·9 to 12·9%)	153·25 (34·19–310·26)	-29·9% (-46·5 to -9·7%)
		Yemen	6·9% (1·4–16·2%)	1·3% (-11·5 to 12·7%)	117·38 (23·34–274·93)	21·4% (-4·2 to 54·6%)
		Afghanistan	7% (1·5–16·6%)	2·9% (-7·3 to 15·3%)	129·89 (25·13–306·86)	31·8% (2·6 to 64·1%)
		Sudan	12·1% (3·1–22·5%)	7·6% (1·2 to 18·3%)	203·86 (52·24–391·56)	16·4% (-5·1 to 43·8%)
Non-optimal temperature	Stroke	Global	6·6% (5–8·5%)	-14·7% (-21·7 to -3·7%)	116·95 (89·66–149·32)	-44·7% (-51·4 to -36·3%)

Table S15 Burden of neurological conditions attributable to risk factors in North Africa and Middle East countries

Risk factors	Causes	Locations	DALYs (95% UI) PAF (%) 2019	PAF change (%) From 1990 to 2019	Rate per 100,000 2019	Rate change (%) From 1990 to 2019
		North Africa and Middle East	7·1% (5–9·1%)	0·1% (-12·5 to 18·5%)	129·1 (90–168·54)	-31·9% (-44·4 to -17·3%)
		Algeria	6·7% (3·4–9·8%)	-1·8% (-14·8 to 27·6%)	117·84 (58·87–183·58)	-45·5% (-59·1 to -24·1%)
		Bahrain	5·8% (2–9·3%)	-1% (-35·9 to 34·4%)	54·83 (18·04–95·42)	-52·8% (-69·2 to -33%)
		Egypt	5·3% (2·4–8·9%)	4·9% (-24·8 to 58%)	113·92 (48·24–206·83)	-32% (-56·3 to 7·9%)
		Iran	8·2% (6·4–10%)	-2·9% (-10·3 to 7·5%)	102·97 (79·85–127·24)	-47·3% (-54·7 to -37·3%)
		Iraq	7·1% (4–10%)	10·2% (-7·8 to 30·5%)	206·58 (118·42–311·74)	-11·1% (-34 to 19·2%)
		Jordan	5·5% (2·1–9·3%)	-4·1% (-13·3 to 24·2%)	78·95 (29·87–138·69)	-52·2% (-63·7 to -32·3%)
		Kuwait	7·2% (1·2–12·6%)	7·2% (-30·6 to 31·2%)	67·46 (11·04–121·22)	-5·3% (-40·9 to 21·7%)
		Lebanon	6·5% (4·5–8·7%)	-18·3% (-28·2 to -11%)	49·43 (27·2–72·15)	-45·6% (-61·8 to -26·3%)
		Libya	5% (1·8–8·7%)	3·2% (-30·5 to 24·8%)	78·51 (27·33–143·25)	-18·7% (-49·3 to 19·7%)
		Morocco	6·9% (4·8–9·3%)	-2·8% (-9 to 2·1%)	155·42 (101·26–229·28)	-19·5% (-38·3 to 0·8%)
		Palestine	5·2% (1·1–9·7%)	-6·9% (-33·7 to 38·1%)	109·61 (23·85–208·66)	-37·3% (-56·2 to -4·2%)
		Oman	7·8% (-0·2–14·7%)	-2·6% (-89·9 to 106·8%)	146·2 (-3·56–292·93)	-40·4% (-93·4 to 31·2%)
		Qatar	7·8% (0·5–14·3%)	-3·7% (-99·3 to 77·5%)	70·63 (4·52–138·33)	-46·6% (-99·5 to -0·4%)
		Saudi Arabia	6·5% (2·9–9·9%)	1·9% (-25·4 to 53·5%)	137·79 (57·64–221·22)	-27·6% (-55·9 to 22·4%)
		Syrian Arab Republic	6·7% (3·9–10%)	-1·6% (-9·7 to 12·7%)	136·36 (73·07–214·99)	-41·1% (-58·5 to -16%)
		Tunisia	6% (2·8–9·5%)	0% (-24·6 to 12%)	88·13 (38·96–152·66)	-24·7% (-50·7 to 7·1%)
		Türkiye	9·2% (6·6–11·7%)	-9·4% (-14·6 to -4·1%)	106·88 (73·31–145·65)	-30·7% (-51 to -11%)
		United Arab Emirates	9% (-0·8–17·6%)	-1·5% (-171·2 to 124%)	173 (-14·41–365·4)	-46·5% (-139·8 to 19·3%)
		Yemen	5·4% (2·1–8·4%)	-5·5% (-53·3 to 63·5%)	148·15 (55·48–251·92)	-28·7% (-65·5 to 23·8%)
		Afghanistan	9·4% (6·9–12·6%)	-1·3% (-8·4 to 6·9%)	328·63 (204·92–469·05)	-22·8% (-41·4 to -1·6%)
		Sudan	6·1% (2·1–9·9%)	6% (-48·5 to 72·4%)	157·76 (54·92–295·75)	-28·8% (-66·2 to 9·2%)
	Ischaemic stroke	Global	6·6% (5·1–8·5%)	-20% (-26·3 to -10·7%)	52·73 (40·28–68·57)	-42·8% (-49·6 to -34·4%)
		North Africa and Middle East	6·8% (4·8–8·8%)	-0·4% (-12·5 to 15·6%)	80·34 (56·24–104·65)	-9·2% (-28 to 9·5%)
		Algeria	6·5% (3·3–9·6%)	-0·1% (-13·6 to 30·1%)	83·44 (41·47–130·21)	-24·7% (-44·2 to 4%)
		Bahrain	5·5% (1·9–8·9%)	-0·2% (-34·9 to 35·2%)	33·86 (10·97–59·41)	-43·8% (-63·7 to -21·6%)
		Egypt	5·1% (2·2–8·6%)	9·4% (-20·2 to 64·5%)	70·12 (29·19–125·55)	24·8% (-27·1 to 100%)
		Iran	8% (6·3–9·8%)	-2·8% (-10·5 to 8·5%)	78·79 (60·74–97·69)	-44·4% (-53·3 to -33·5%)
		Iraq	6·8% (3·9–9·7%)	11·7% (-7 to 32·7%)	124·68 (71·93–187·48)	3·8% (-22·8 to 39%)
		Jordan	5·3% (2–9·1%)	-4·1% (-13·2 to 24%)	57·04 (21·45–99·4)	-48·4% (-61·8 to -28%)
		Kuwait	6·9% (1·1–12%)	7% (-31·1 to 31·2%)	44·35 (7·06–80·14)	-5·8% (-42·8 to 20·7%)
		Lebanon	6·3% (4·2–8·5%)	-14·7% (-25·8 to -6·8%)	38·15 (21·45–55·67)	-27·2% (-48·9 to -2·6%)
		Libya	4·8% (1·8–8·4%)	7% (-27·7 to 29·9%)	54·23 (18·73–99·8)	19·2% (-27·3 to 71·5%)
		Morocco	6·7% (4·6–9%)	0·7% (-6·5 to 6·5%)	107·68 (70·15–158·67)	19·3% (-10·7 to 47·5%)
		Palestine	5·1% (1·1–9·5%)	-5·7% (-32·6 to 39·5%)	80·47 (17·32–153·82)	-22·6% (-46·6 to 18·2%)
		Oman	7·5% (-0·2–14·2%)	-0·8% (-89·6 to 111·2%)	97·17 (-2·32–194·21)	-19·2% (-91·4 to 74·2%)
		Qatar	7·3% (0·5–13·6%)	-2·2% (-99·3 to 80%)	42·4 (2·69–83·89)	-36·8% (-99·4 to 15·6%)
		Saudi Arabia	6·3% (2·8–9·6%)	2% (-25·5 to 52·3%)	81·83 (33·77–130·03)	-15·6% (-50·2 to 40·2%)
		Syrian Arab Republic	6·3% (3·7–9·4%)	-1·3% (-10·8 to 13·8%)	67·82 (36·91–106·82)	-26% (-49·6 to 6·2%)
		Tunisia	5·8% (2·7–9·3%)	2·7% (-22·6 to 15·2%)	63·41 (27·79–110·06)	6·9% (-32·8 to 52·8%)
		Türkiye	8·6% (6·2–10·9%)	-7·1% (-15 to 0·2%)	56·87 (39·18–78·07)	-14·6% (-42·9 to 12·4%)
		United Arab Emirates	8·6% (-0·7–17%)	-0·7% (-172·5 to 124%)	122·56 (-10·23–255·24)	-34% (-150·7 to 51·6%)
		Yemen	5·2% (2–8·1%)	-2·5% (-51·5 to 67·8%)	89·09 (33·15–148·36)	16·5% (-38·6 to 96·4%)
		Afghanistan	9·1% (6·7–12·1%)	1·6% (-5·8 to 10·1%)	169·45 (107·06–243·25)	29·3% (-1·8 to 65·8%)
		Sudan	5·9% (2–9·6%)	8·9% (-47·2 to 77·3%)	100·06 (31·58–189·81)	17·9% (-42·6 to 85·7%)
	Intracerebral haemorrhage	Global	6·7% (5–8·5%)	-8·5% (-17·3 to 8·2%)	55·47 (41·16–71·19)	-42·1% (-50·4 to -30·6%)
		North Africa and Middle East	7·6% (5·3–9·8%)	4% (-10·4 to 26·2%)	41·71 (28·68–55·64)	-49·8% (-59 to -36·9%)
		Algeria	7·2% (3·7–10·5%)	0·5% (-12·8 to 30·6%)	29·15 (14·37–44·99)	-67·7% (-77·2 to -54·3%)
		Bahrain	6·4% (2·2–10·3%)	1·8% (-35 to 38·6%)	17·04 (5·63–29·48)	-63·4% (-76·1 to -45·5%)
		Egypt	5·8% (2·7–9·8%)	9·4% (-21·2 to 62·9%)	37·29 (15·74–68·68)	-55·8% (-72·3 to -28·8%)

Table S15 Burden of neurological conditions attributable to risk factors in North Africa and Middle East countries

Risk factors	Causes	Locations	DALYs (95% UI) PAF (%) 2019	PAF change (%) From 1990 to 2019	Rate per 100,000 2019	Rate change (%) From 1990 to 2019
		Iran	8·8% (6·9–10·7%)	-1·4% (-8·4 to 8·2%)	19·63 (15·32–24·06)	-54·3% (-61·7 to -45·8%)
		Iraq	7·5% (4·3–10·6%)	10·8% (-6·7 to 32·2%)	76·89 (43·7–118·32)	-22·4% (-44·1 to 5·8%)
		Jordan	6·1% (2·4–10·6%)	-0·4% (-8·4 to 28·7%)	20 (7·56–35·68)	-60·2% (-69·6 to -43·2%)
		Kuwait	8% (1·4–14%)	9% (-28·2 to 31·8%)	19·81 (3·11–36·07)	4·3% (-37·1 to 37·9%)
		Lebanon	7·5% (5·3–9·9%)	-16·5% (-23·5 to -10·3%)	9·7 (5·71–15·27)	-69·8% (-79 to -56·3%)
		Libya	5·5% (2–9·4%)	5·8% (-28·8 to 26·7%)	20·57 (6·97–38·01)	-48·4% (-68·2 to -21·3%)
		Morocco	7·3% (5·1–9·9%)	-2·1% (-8·1 to 2·2%)	40·67 (25·95–59·37)	-52·1% (-65·6 to -34·4%)
		Palestine	5·5% (1·2–10·5%)	-5·7% (-33·5 to 39·7%)	26·78 (5·76–51·28)	-59·3% (-72·1 to -35·9%)
		Oman	8·3% (-0·2–15·8%)	-0·8% (-89·9 to 111%)	41·94 (-1·02–84·27)	-60·2% (-95·7 to -17·3%)
		Qatar	8·6% (0·6–15·9%)	-2·1% (-99·3 to 81·5%)	21·03 (1·39–41·05)	-57·1% (-99·6 to -17%)
		Saudi Arabia	6·9% (3·1–10·5%)	3·8% (-24·6 to 58·7%)	52·84 (21·72–86·32)	-39% (-62·7 to 7·1%)
		Syrian Arab Republic	7·3% (4·3–10·7%)	1% (-6·8 to 15·6%)	60·9 (31·87–96·51)	-48% (-63·9 to -25·5%)
		Tunisia	6·5% (3–10·4%)	2·4% (-22·5 to 13·1%)	20·94 (9·22–36·61)	-55·6% (-71 to -33%)
		Türkiye	10·1% (7·2–12·8%)	-8·6% (-12·6 to -4·4%)	40·16 (26·97–54·94)	-42·4% (-59·2 to -24·7%)
		United Arab Emirates	9·9% (-0·8–19·1%)	2·1% (-173·2 to 132·8%)	44·78 (-3·59–100·59)	-63·1% (-126·4 to -15·4%)
		Yemen	5·6% (2·2–8·9%)	-4·2% (-52·5 to 67·2%)	49·91 (18·82–87·64)	-55·6% (-79 to -19·9%)
		Afghanistan	9·8% (7·2–13·1%)	-0·4% (-7·5 to 7·7%)	136·39 (83·5–198·16)	-46·2% (-59·8 to -28·2%)
		Sudan	6·5% (2·2–10·5%)	8·4% (-47·5 to 77·6%)	49·93 (16·66–91·2)	-57·1% (-80·3 to -29·9%)
	Subarachnoid haemorrhage	Global	6·4% (4·8–8·2%)	-19·2% (-27·5 to -7·3%)	8·75 (6·57–11·35)	-63% (-69·5 to -47·7%)
		North Africa and Middle East	7·5% (5·4–9·6%)	4·9% (-10·2 to 29·3%)	7·04 (4·86–9·71)	-61% (-71·5 to -33·3%)
		Algeria	6·8% (3·5–9·9%)	-3·1% (-16·4 to 24·8%)	5·25 (2·55–8·36)	-65·7% (-77·2 to -40·7%)
		Bahrain	6·2% (2·1–10·1%)	-0·6% (-35·6 to 34·4%)	3·94 (1·22–7·21)	-58·1% (-75·1 to -33·9%)
		Egypt	5·6% (2·6–9·6%)	6% (-24·4 to 58·5%)	6·5 (2·6–12·07)	-75·9% (-86·4 to -42·5%)
		Iran	8·3% (6·6–10·2%)	-4·5% (-11·6 to 5·2%)	4·55 (3·48–5·89)	-57·7% (-70·9 to -32·4%)
		Iraq	6·9% (3·9–9·8%)	4·3% (-10·8 to 26·2%)	5·01 (2·66–8·3)	-62·4% (-77·3 to -33·3%)
		Jordan	4·9% (1·9–8·4%)	-12·3% (-24·3 to 13·8%)	1·91 (0·72–3·36)	-56% (-71·1 to -25·8%)
		Kuwait	7% (1·1–12·2%)	-1·5% (-32·3 to 19·9%)	3·3 (0·51–5·91)	-35·9% (-58·2 to -1·5%)
		Lebanon	5·8% (3·8–8·1%)	-30·9% (-47·2 to -17%)	1·57 (0·78–2·52)	-75·2% (-88 to -53·4%)
		Libya	5·1% (1·9–9%)	-0·3% (-32·5 to 20·5%)	3·71 (1·28–7·18)	-66·7% (-81·7 to -37%)
		Morocco	7% (4·8–9·5%)	-4·7% (-11·4 to 1·4%)	7·08 (4·12–11·79)	-60·6% (-74·5 to -31·7%)
		Palestine	4·7% (1–8·9%)	-12·1% (-39·4 to 30·1%)	2·36 (0·52–4·64)	-52·5% (-70·7 to -13·8%)
		Oman	8% (-0·2–15·5%)	-3·9% (-89·9 to 104·4%)	7·09 (-0·21–16·61)	-64% (-95·8 to -13·1%)
		Qatar	8·6% (0·6–15·7%)	-2·9% (-99·3 to 79·9%)	7·2 (0·44–14·58)	-55·2% (-99·6 to -8·2%)
		Saudi Arabia	6·3% (2·7–9·6%)	-2·6% (-29·4 to 51·6%)	3·11 (1·19–5·37)	-53·6% (-76·5 to -4·7%)
		Syrian Arab Republic	7% (4·1–10·3%)	-3% (-11 to 11·1%)	7·64 (4·23–12·09)	-66·8% (-77·2 to -47%)
		Tunisia	5·9% (2·7–9·4%)	-4·6% (-28·5 to 7·2%)	3·79 (1·63–6·87)	-64·4% (-80·3 to -35·4%)
		Türkiye	9·8% (7–12·5%)	-9·1% (-14·7 to -2·3%)	9·86 (6·54–13·74)	-45·5% (-66·6 to 0·9%)
		United Arab Emirates	9·3% (-0·8–18·4%)	-2·7% (-170·5 to 113·2%)	5·66 (-0·45–13·58)	-64·9% (-123·4 to -19·3%)
		Yemen	5·5% (2·2–8·6%)	-5·5% (-52·8 to 65·5%)	9·15 (2·48–18·46)	-51·5% (-76·5 to 0·2%)
		Afghanistan	9·6% (7–12·8%)	-1% (-8·4 to 6·7%)	22·79 (6·89–42·36)	-44·3% (-60·1 to -15%)
		Sudan	6·2% (2·2–10·2%)	5·8% (-49·4 to 75·1%)	7·77 (2·14–16·77)	-61·4% (-82·8 to -15·5%)
Other environmental risks	Stroke	Global	4·6% (2·7–6·7%)	-5·1% (-11·8 to 0·8%)	81·97 (47·86–119·11)	-38·5% (-45·6 to -31·2%)
		North Africa and Middle East	5·2% (3·3–7·3%)	-12·9% (-22·2 to -5·2%)	95·53 (57·79–137·74)	-40·8% (-50·5 to -30·4%)
		Algeria	3·2% (1·4–5·1%)	-18·1% (-31 to -8·7%)	55·52 (24·56–93·24)	-54·7% (-65·8 to -42·4%)
		Bahrain	2·2% (0·7–3·9%)	-19·2% (-33 to -9·6%)	20·3 (6·16–38·15)	-61·6% (-70·9 to -52·2%)
		Egypt	5·5% (3·5–7·7%)	14·3% (-5·9 to 33·7%)	118·05 (65·72–186·59)	-25·9% (-45·2 to -2·3%)
		Iran	6·9% (4·7–9·3%)	-13·9% (-21·7 to -7%)	87·46 (57·78–117·89)	-53·2% (-60·6 to -45·7%)
		Iraq	3·9% (2–5·9%)	-11·6% (-22·2 to -3·2%)	113·88 (55·77–181·64)	-28·8% (-45·5 to -9·4%)
		Jordan	2·9% (1·2–4·7%)	-21·2% (-35·2 to -12·4%)	41·44 (16·91–70)	-60·7% (-71 to -51·7%)

Table S15 Burden of neurological conditions attributable to risk factors in North Africa and Middle East countries

Risk factors	Causes	Locations	DALYs (95% UI) PAF (%) 2019	PAF change (%) From 1990 to 2019	Rate per 100,000 2019	Rate change (%) From 1990 to 2019
		Kuwait	2·7% (1·1–4·6%)	-18·1% (-31·9 to -9·8%)	25·79 (10·26–44·16)	-27·7% (-43·4 to -12·5%)
		Lebanon	2·7% (1·2–4·6%)	-30·6% (-45·5 to -20·3%)	20·55 (8·06–37·01)	-53·9% (-68·3 to -40·4%)
		Libya	2·2% (0·8–4%)	-34·4% (-56·3 to -19·8%)	35·29 (11·24–68·24)	-48·3% (-67·9 to -29·9%)
		Morocco	3·8% (1·9–6%)	-18·9% (-30·7 to -9·3%)	85·96 (41·67–138·88)	-32·9% (-49·7 to -14·3%)
		Palestine	3·9% (2·1–5·9%)	-22·7% (-35·7 to -14·6%)	83·3 (43·53–130·17)	-47·8% (-60·9 to -32·8%)
		Oman	3·1% (1·4–4·9%)	-27·2% (-42·2 to -16%)	57·66 (25·62–93·96)	-55·4% (-69·1 to -39·8%)
		Qatar	1·8% (0·4–3·5%)	-5·2% (-17·1 to 64·7%)	16·2 (3·34–33·28)	-47·6% (-61·2 to -3·7%)
		Saudi Arabia	3·9% (1·9–6·1%)	-26·4% (-40·9 to -16·8%)	82·91 (40·04–132·48)	-47·8% (-64·1 to -28·8%)
		Syrian Arab Republic	5·1% (3–7·2%)	-11·7% (-21·5 to -2·4%)	102·23 (57·33–157·04)	-47·2% (-62·5 to -27·4%)
		Tunisia	4·1% (2·2–6·1%)	-21·3% (-34·8 to -11·7%)	60·6 (29·74–97·85)	-40·8% (-59·1 to -20%)
		Türkiye	1·9% (0·5–3·7%)	-27·1% (-48·4 to -17·3%)	22·68 (5·49–45·3)	-44·4% (-64·6 to -27·8%)
		United Arab Emirates	1·7% (0·3–3·4%)	-40·4% (-69·2 to -26·2%)	32·39 (5·88–69·53)	-67·9% (-84·3 to -54·1%)
		Yemen	10·9% (8·2–13·7%)	-3·9% (-13·2 to 6·2%)	301·92 (197·4–425·53)	-27·5% (-46 to -2·3%)
		Afghanistan	12·4% (9·4–15·4%)	6·2% (-3 to 16·3%)	434·86 (284·77–613·55)	-16·9% (-37·4 to 7%)
		Sudan	8·9% (6·4–11·5%)	-8·2% (-18·2 to 3·2%)	230·73 (144·66–348·1)	-38·6% (-52·1 to -21·3%)
	Ischaemic stroke	Global	4% (2·3–6%)	21·2% (12 to 36·6%)	32·21 (18·17–48·71)	-13·4% (-25·7 to 0·9%)
		North Africa and Middle East	5·2% (3·2–7·4%)	-7·1% (-13·5 to -2·2%)	61·25 (36·23–88·95)	-15·2% (-28·5 to -3·3%)
		Algeria	3·2% (1·5–5·1%)	-9·4% (-20·9 to -2·2%)	40·43 (17·59–68·94)	-31·8% (-47·6 to -15·1%)
		Bahrain	2·2% (0·7–3·9%)	-12·1% (-25·2 to -0·9%)	13·21 (4·22–24·75)	-50·6% (-62·2 to -39·5%)
		Egypt	5·8% (3·7–8·2%)	10·2% (-2·6 to 23·8%)	80·62 (45·74–126·67)	25·7% (-9·4 to 61·9%)
		Iran	6·8% (4·5–9·4%)	-9·7% (-17·2 to -1·8%)	67·25 (43·84–92·75)	-48·3% (-57·3 to -39·8%)
		Iraq	3·9% (2–6%)	-5·7% (-15 to 2·9%)	71·04 (35·62–113·25)	-12·3% (-31·8 to 9%)
		Jordan	2·8% (1·2–4·7%)	-17·1% (-31·5 to -8%)	30·72 (12·26–52·13)	-55·4% (-66·6 to -45·7%)
		Kuwait	2·8% (1·1–4·7%)	-13·7% (-27·4 to -4·1%)	17·84 (7·36–30·33)	-24·1% (-39·7 to -8%)
		Lebanon	2·8% (1·2–4·7%)	-25·2% (-40·7 to -15·8%)	16·74 (6·69–29·73)	-36·4% (-55·9 to -17·9%)
		Libya	2·4% (0·8–4·2%)	-31·6% (-54·1 to -19·1%)	26·83 (8·7–50·65)	-23·9% (-52·8 to 3%)
		Morocco	3·8% (1·9–5·9%)	-11·8% (-23·3 to -4·2%)	60·63 (28·96–98·1)	4·6% (-21·6 to 30%)
		Palestine	3·9% (2·1–6%)	-19·2% (-31·6 to -11·7%)	61·91 (32·47–97·06)	-33·5% (-50·8 to -15%)
		Oman	3·1% (1·5–5·2%)	-23·3% (-37·9 to -12%)	40·66 (18·41–67·86)	-37·3% (-55·6 to -14·2%)
		Qatar	1·8% (0·4–3·5%)	0·5% (-13 to 71·6%)	10·2 (2·34–20·74)	-35·3% (-51·9 to 15·6%)
		Saudi Arabia	3·8% (1·9–5·9%)	-18% (-31·2 to -9·1%)	49·96 (24·73–78·44)	-32·3% (-51·5 to -12·4%)
		Syrian Arab Republic	5% (3–7·3%)	-7·7% (-17·3 to 1·3%)	54·04 (30·2–83·26)	-30·8% (-51·6 to -7·3%)
		Tunisia	4·1% (2·2–6·2%)	-13·9% (-25·3 to -6%)	44·58 (21·23–72·76)	-10·3% (-36 to 17·9%)
		Türkiye	2% (0·6–3·7%)	-22·2% (-43·8 to -13%)	12·97 (3·61–25·41)	-28·5% (-54·5 to -7·9%)
		United Arab Emirates	1·7% (0·4–3·5%)	-34·4% (-65·1 to -20·6%)	24·4 (4·85–51·23)	-56·8% (-78·4 to -40·3%)
		Yemen	10·4% (7·5–13·6%)	7·3% (-0·1 to 15·4%)	179·54 (116·54–261·98)	28·5% (-0·3 to 67·1%)
		Afghanistan	11·6% (8·4–14·8%)	16·6% (8·7 to 25·6%)	216·31 (137·82–313·55)	48·3% (14 to 90·9%)
		Sudan	8·8% (6·1–11·7%)	2·3% (-5 to 10·3%)	148·38 (90·57–227·81)	10·5% (-13·1 to 39·3%)
	Intracerebral haemorrhage	Global	5·3% (3–7·8%)	-11·5% (-19·9 to -5·6%)	44·03 (25·12–65·69)	-44% (-52·4 to -36·4%)
		North Africa and Middle East	5·4% (3·2–8%)	-19·1% (-29·6 to -9·5%)	29·89 (17·17–44·76)	-60·9% (-68·6 to -52·2%)
		Algeria	3·2% (1·2–5·7%)	-26·5% (-43·7 to -15·9%)	12·88 (4·76–23·66)	-76·4% (-83·7 to -67·8%)
		Bahrain	2·3% (0·6–4·4%)	-26·8% (-46·4 to -15·3%)	5·97 (1·56–12·63)	-73·7% (-82·1 to -64·7%)
		Egypt	5·2% (3–7·8%)	0·1% (-21·5 to 28·3%)	33·37 (17·23–58·74)	-59·3% (-72·9 to -41·2%)
		Iran	7·4% (4·8–10·4%)	-22·6% (-32·3 to -14%)	16·5 (10·6–23·14)	-64·1% (-70·6 to -56·5%)
		Iraq	4% (1·7–6·6%)	-18·3% (-33·3 to -8·1%)	40·41 (16·84–70·47)	-42·8% (-59·5 to -23·5%)
		Jordan	3% (1–5·4%)	-27·2% (-45·4 to -16·2%)	9·77 (3·34–17·91)	-70·9% (-79·9 to -61·9%)
		Kuwait	2·8% (0·9–5·1%)	-27·1% (-46·5 to -15·8%)	6·88 (2·27–12·72)	-30·2% (-52·3 to -8·2%)
		Lebanon	2·6% (0·9–4·8%)	-41·5% (-60·2 to -29·4%)	3·28 (1·09–6·76)	-78·9% (-87 to -69·8%)
		Libya	2% (0·5–4%)	-46·7% (-70·5 to -29·4%)	7·37 (1·84–15·92)	-74% (-86·4 to -60·8%)
		Morocco	3·9% (1·7–6·4%)	-24·6% (-41 to -14·1%)	21·79 (9·07–37·77)	-63·1% (-74·6 to -48·2%)
		Palestine	4·1% (1·9–6·5%)	-26·5% (-42·6 to -16·8%)	19·78 (9·06–33·03)	-68·1% (-77·9 to -57·4%)

Table S15 Burden of neurological conditions attributable to risk factors in North Africa and Middle East countries

Risk factors	Causes	Locations	DALYs (95% UI) PAF (%) 2019	PAF change (%) From 1990 to 2019	Rate per 100,000 2019	Rate change (%) From 1990 to 2019
		Oman	3% (1·1–5·2%)	-33·7% (-52·8 to -21·1%)	15 (5·51–26·98)	-73·4% (-83·3 to -61·8%)
		Qatar	1·9% (0·3–3·9%)	-9·2% (-25 to 52·7%)	4·59 (0·72–10·19)	-60·3% (-73·2 to -29·2%)
		Saudi Arabia	4·1% (1·8–6·8%)	-32·6% (-51 to -21·2%)	31·41 (13·46–54·04)	-60·5% (-75 to -41·4%)
		Syrian Arab Republic	5·4% (3–8·1%)	-18·2% (-30·3 to -7·9%)	45·05 (23·41–72·58)	-57·9% (-71·1 to -39·8%)
		Tunisia	4·2% (2–6·8%)	-27·7% (-43·3 to -17·2%)	13·67 (5·99–23·64)	-68·6% (-79·9 to -53·5%)
		Türkiye	2% (0·4–4·1%)	-29·7% (-57·1 to -16·4%)	8·05 (1·46–17·22)	-55·8% (-75·2 to -38·3%)
		United Arab Emirates	1·6% (0·2–3·7%)	-49·8% (-81·3 to -33%)	7·21 (0·84–19·06)	-82% (-93·7 to -71%)
		Yemen	11·8% (8·4–15·4%)	-6% (-13·6 to 1·9%)	104·35 (65·96–154·61)	-56·5% (-68·4 to -39%)
		Afghanistan	13·5% (9·7–17·5%)	6·4% (-0·6 to 14·1%)	188·01 (116·69–272·63)	-42·6% (-57·3 to -23·8%)
		Sudan	9·3% (6·1–12·6%)	-12·7% (-23·1 to -1·9%)	71·77 (41·91–112·07)	-65·7% (-75·2 to -53·2%)
	Subarachnoid haemorrhage	Global	4·2% (2·1–6·6%)	-28·2% (-40·6 to -18%)	5·73 (2·8–9·26)	-67·3% (-74·9 to -50·2%)
		North Africa and Middle East	4·6% (2·5–7·1%)	-4·7% (-20·6 to 19·1%)	4·39 (2·14–7·27)	-64·8% (-74·1 to -46·8%)
		Algeria	2·9% (1–5·2%)	-27·2% (-48·5 to -12·5%)	2·2 (0·73–4·21)	-74·2% (-84·5 to -58%)
		Bahrain	1·8% (0·4–3·7%)	-21·7% (-47·7 to -1·8%)	1·12 (0·23–2·53)	-67% (-82 to -50·7%)
		Egypt	3·5% (1·8–5·9%)	31·8% (-17·3 to 155·6%)	4·07 (1·89–7·55)	-69·5% (-82·7 to -41·9%)
		Iran	6·8% (4·2–9·8%)	-23·7% (-37 to -12·3%)	3·71 (2·21–5·45)	-66·2% (-77·3 to -47%)
		Iraq	3·4% (1·2–5·9%)	-17·5% (-38·2 to -3·9%)	2·42 (0·87–4·68)	-70·3% (-82·3 to -49·9%)
		Jordan	2·5% (0·7–4·8%)	-37·9% (-58·5 to -24·6%)	0·96 (0·26–1·94)	-68·8% (-82·6 to -54·5%)
		Kuwait	2·3% (0·7–4·4%)	-28·8% (-51·9 to -12·3%)	1·07 (0·3–2·11)	-53·6% (-70·9 to -36·8%)
		Lebanon	2% (0·6–4·2%)	-45·5% (-67·8 to -29·7%)	0·54 (0·14–1·21)	-80·5% (-91·4 to -66·3%)
		Libya	1·5% (0·4–3·3%)	-31·7% (-62·9 to 5·4%)	1·09 (0·25–2·47)	-76·9% (-88·3 to -62·2%)
		Morocco	3·5% (1·4–6%)	-22·2% (-39·8 to -8·5%)	3·55 (1·29–6·82)	-68% (-79·7 to -46·5%)
		Palestine	3·2% (1·3–5·6%)	-33·1% (-50·4 to -21·8%)	1·61 (0·63–2·93)	-63·8% (-77·7 to -46·2%)
		Oman	2·2% (0·7–4·2%)	-33·6% (-58·1 to -10·2%)	2·01 (0·52–4·68)	-74·7% (-87·9 to -52·8%)
		Qatar	1·7% (0·2–3·8%)	-14·4% (-34·1 to 46·6%)	1·42 (0·17–3·49)	-60·8% (-79·1 to -16·6%)
		Saudi Arabia	3·1% (1·2–5·6%)	-42·7% (-65·2 to -26·3%)	1·55 (0·55–3)	-72·8% (-88·2 to -52·3%)
		Syrian Arab Republic	2·9% (1·4–4·6%)	7·5% (-26·7 to 53·6%)	3·14 (1·46–5·6)	-62·9% (-79·2 to -37·6%)
		Tunisia	3·6% (1·5–6·1%)	-30·2% (-50·7 to -14·5%)	2·35 (0·86–4·43)	-74% (-85·9 to -54·9%)
		Türkiye	1·6% (0·2–3·8%)	-37·6% (-64·8 to -22·6%)	1·66 (0·24–3·94)	-62·6% (-83·9 to -35·4%)
		United Arab Emirates	1·2% (0·1–3·1%)	-50·8% (-80·1 to -29·2%)	0·77 (0·07–2·17)	-82·3% (-94 to -68·4%)
		Yemen	10·9% (7·4–14·5%)	-4·5% (-15 to 7·2%)	18·03 (8·09–33·89)	-51·2% (-66·5 to -22·7%)
		Afghanistan	12·9% (9·1–17·2%)	8·4% (-0·5 to 18·6%)	30·54 (11·05–58·12)	-39·1% (-57·1 to -5·9%)
		Sudan	8·5% (5·4–11·9%)	-8·9% (-24·2 to 9·7%)	10·59 (4·6–19·87)	-67·3% (-78·7 to -39·5%)
Tobacco	Stroke	Global	20% (18·6–21·4%)	-9·6% (-13·8 to -5·3%)	353·87 (316–391·5)	-41·4% (-47·4 to -35·5%)
		North Africa and Middle East	16·9% (15·6–18·1%)	-4·1% (-10·2 to 2%)	307·84 (267·1–351)	-34·8% (-44 to -23·9%)
		Algeria	15·4% (13·9–17·1%)	-11·7% (-19·1 to -3·9%)	270·63 (214·67–337·92)	-51·1% (-61·5 to -37·8%)
		Bahrain	13·7% (12·1–15·6%)	-26·1% (-36·1 to -14·6%)	128·88 (103·8–164)	-64·9% (-72·6 to -55·1%)
		Egypt	16·6% (14·7–19%)	40·6% (17·2 to 63%)	356·02 (258·59–498·99)	-8·8% (-31·7 to 21·6%)
		Iran	14·5% (13·3–15·8%)	-14·6% (-21·9 to -6·5%)	182·93 (162·77–202·43)	-53·6% (-60·5 to -46%)
		Iraq	21·7% (19·5–23·7%)	-13·2% (-21·7 to -3·6%)	633·82 (495·23–781·65)	-30% (-47·4 to -8·6%)
		Jordan	20·7% (18·7–22·6%)	-17·3% (-25·9 to -7·9%)	299·94 (240·03–359·36)	-58·8% (-67·6 to -49%)
		Kuwait	18·5% (16·9–20·2%)	-7·6% (-16·8 to 1·9%)	174·07 (144·23–209·58)	-18·3% (-33·4 to -0·4%)
		Lebanon	28·7% (26·2–31·5%)	20·7% (7·2 to 35·6%)	216·26 (166·97–275·52)	-20% (-39 to 3·5%)
		Libya	15·7% (13·7–18%)	1·3% (-10 to 15%)	246·48 (181·03–335·41)	-20·3% (-39·9 to 8·1%)
		Morocco	11·6% (10·2–13·1%)	-26·2% (-33·9 to -17·6%)	262 (197·05–333·24)	-38·8% (-54·5 to -20·8%)
		Palestine	17·2% (15·5–19·2%)	-10% (-20 to 0·5%)	366·96 (306·71–432·28)	-39·3% (-53·7 to -19·2%)
		Oman	9·2% (8·1–10·5%)	-34·1% (-43·7 to -22·8%)	174·13 (144·52–213·13)	-59·6% (-70·3 to -43·4%)
		Qatar	12·1% (10·7–13·5%)	-16·3% (-28 to -2·6%)	109·11 (85·34–138·58)	-53·7% (-66·3 to -37·6%)
		Saudi Arabia	15·1% (13·2–17%)	9·3% (-6·3 to 27·1%)	318·79 (233·57–399·32)	-22·5% (-45·1 to 10·2%)
		Syrian Arab Republic	18·9% (17·2–20·7%)	-14·9% (-23·8 to -5·3%)	382·18 (284·82–508·54)	-49·1% (-63·6 to -26·9%)
		Tunisia	18·6% (16·8–20·5%)	-11·5% (-19·8 to -2·6%)	275·41 (202·54–364·6)	-33·3% (-52 to -8·7%)

Table S15 Burden of neurological conditions attributable to risk factors in North Africa and Middle East countries

Risk factors	Causes	Locations	DALYs (95% UI) PAF (%) 2019	PAF change (%) From 1990 to 2019	Rate per 100,000 2019	Rate change (%) From 1990 to 2019
		Türkiye	21·3% (19·6–23%)	-22·3% (-29·2 to -14·9%)	247·41 (201·12–297·98)	-40·6% (-58·4 to -23·1%)
		United Arab Emirates	17% (15–19%)	10·1% (-3·4 to 26·4%)	326·93 (240·88–435·43)	-40·6% (-57·6 to -17·6%)
		Yemen	21·7% (19·5–23·9%)	-5·7% (-16·7 to 7·3%)	599·78 (453·71–789·59)	-28·9% (-47·3 to -1%)
		Afghanistan	11·8% (10·3–13·4%)	33·8% (19·7 to 51·4%)	411·89 (293·62–559·68)	4·7% (-22·3 to 39·4%)
		Sudan	14·8% (12·5–17·2%)	-8% (-21·2 to 8·2%)	383·88 (269·68–549·76)	-38·3% (-54 to -18%)
	Ischaemic stroke	Global	17·6% (16·4–18·7%)	-8% (-12·3 to -4%)	140·23 (124·51–155·91)	-34·2% (-41·7 to -28·2%)
		North Africa and Middle East	15·6% (14·4–16·8%)	-7% (-12·4 to -1·8%)	184·39 (160·98–210·02)	-15·1% (-31·8 to -2·3%)
		Algeria	14·5% (12·9–16·1%)	-8·5% (-16·8 to 1·5%)	184·78 (144·73–232·03)	-31·1% (-46·2 to -12%)
		Bahrain	12·4% (10·9–14·3%)	-25·1% (-35·3 to -11·9%)	76·35 (61·83–97·5)	-57·9% (-67·1 to -47%)
		Egypt	16·8% (14·8–19%)	27·3% (8·8 to 46·9%)	232·04 (171·01–319·44)	45·1% (3·3 to 91·2%)
		Iran	13·7% (12·5–15%)	-14·9% (-22·4 to -6·5%)	135·02 (118·97–150·08)	-51·3% (-59 to -43·6%)
		Iraq	19·8% (17·8–21·8%)	-14·6% (-23·5 to -3·8%)	363·31 (288·42–438·92)	-20·5% (-39·1 to 2·3%)
		Jordan	19·2% (17·3–21%)	-17·1% (-25·5 to -7·8%)	208·23 (166·88–248·74)	-55·5% (-65·1 to -45·2%)
		Kuwait	16·9% (15·3–18·6%)	-9·6% (-19·2 to 0·6%)	109·15 (88·86–132·26)	-20·4% (-34·5 to -2·6%)
		Lebanon	27·8% (25·4–30·5%)	27·8% (12·7 to 45·1%)	166·23 (128·64–205·97)	8·5% (-17·5 to 37·7%)
		Libya	15% (13·1–17·1%)	0·1% (-11·6 to 12·5%)	169·09 (122·76–230·36)	11·4% (-17·7 to 49·2%)
		Morocco	10·6% (9·3–12%)	-21·8% (-30·7 to -11·5%)	169·87 (129·2–212·95)	-7·3% (-31·9 to 20·9%)
		Palestine	15·9% (14·2–17·8%)	-8·8% (-19·4 to 2·9%)	253·67 (209·27–300·08)	-25·1% (-43·1 to -1·4%)
		Oman	9·2% (8·1–10·4%)	-36·8% (-46·3 to -25%)	119·18 (97·9–143·3)	-48·4% (-62·3 to -27·8%)
		Qatar	10·3% (9·1–11·7%)	-16·7% (-28·7 to -1·6%)	59·79 (46·94–76·51)	-46·2% (-59·6 to -27·9%)
		Saudi Arabia	13·2% (11·5–15%)	12·9% (-4·1 to 31·7%)	171·58 (126·03–213·53)	-6·8% (-34·6 to 28·8%)
		Syrian Arab Republic	17·3% (15·6–19%)	-18·7% (-27·8 to -8·1%)	185·08 (140·78–241·25)	-39·1% (-57·3 to -14·2%)
		Tunisia	17·5% (15·7–19·4%)	-8·8% (-18·2 to 1·4%)	190·69 (141·54–252·24)	-5% (-32·8 to 28·6%)
		Türkiye	17·6% (16–19·2%)	-24·7% (-32·2 to -15·9%)	116·75 (95·1–141·19)	-30·7% (-52·7 to -10·7%)
		United Arab Emirates	15·7% (13·9–17·6%)	14·6% (0 to 32·7%)	221·74 (167·39–284·63)	-24·3% (-45·4 to 4·5%)
		Yemen	19·9% (17·9–21·9%)	1·7% (-10·8 to 16·2%)	341·06 (265·69–450·12)	21·7% (-9 to 65·4%)
		Afghanistan	10·4% (8·9–11·8%)	38·6% (23·3 to 56·9%)	193·78 (141·61–268·99)	76·2% (31·5 to 133·2%)
		Sudan	13·7% (11·6–16·1%)	-0·8% (-15·8 to 16·1%)	233·26 (163·03–351·84)	7·4% (-19·8 to 44·3%)
	Intracerebral haemorrhage	Global	22·1% (20·4–23·7%)	-8·2% (-13·1 to -3·3%)	183·87 (163·13–204·49)	-41·9% (-49·4 to -34·9%)
		North Africa and Middle East	19·4% (17·9–21%)	1·5% (-6·1 to 9·6%)	106·55 (90·31–125·1)	-51% (-58·9 to -40·7%)
		Algeria	17·9% (15·8–20·2%)	-8·8% (-17·8 to 0·5%)	72·12 (53·89–93·77)	-70·7% (-78·6 to -60·1%)
		Bahrain	16% (14·1–18·3%)	-26·4% (-36·2 to -15·2%)	42·38 (32·66–54·88)	-73·6% (-80·2 to -64·5%)
		Egypt	17% (14·1–20%)	35% (8·2 to 67·9%)	109·94 (71·9–171·21)	-45% (-63·2 to -19·6%)
		Iran	17·4% (15·9–18·8%)	-12·3% (-19·4 to -3·8%)	38·75 (34·91–42·99)	-59·3% (-65·9 to -51·5%)
		Iraq	24·9% (22·5–27·2%)	-10·2% (-19·7 to 0%)	254·64 (191·42–329·95)	-37·1% (-54·7 to -14·2%)
		Jordan	25% (22·6–27·4%)	-14·2% (-23·9 to -4·4%)	81·39 (64·8–99·66)	-65·7% (-73·5 to -56%)
		Kuwait	22·6% (20·6–24·6%)	-4·8% (-14·3 to 5·7%)	55·61 (44·2–68·97)	-8·7% (-29·5 to 17·2%)
		Lebanon	32% (29·2–35·2%)	17·2% (5 to 31%)	41·18 (29·96–57·1)	-57·8% (-70·6 to -40%)
		Libya	17·7% (14·6–21%)	1·3% (-15 to 20·1%)	66·71 (44·97–97·78)	-50·5% (-66 to -27·1%)
		Morocco	14·2% (12·4–16·4%)	-22·4% (-31·7 to -12·6%)	79·05 (56·44–105·4)	-61·9% (-73·8 to -46·7%)
		Palestine	21·2% (18·9–23·8%)	-3·5% (-14·7 to 8·9%)	103·32 (83·46–126·72)	-58·2% (-69·2 to -42·5%)
		Oman	9·2% (8–10·8%)	-33% (-44·7 to -19·5%)	46·65 (37·55–62·91)	-73·1% (-80·9 to -61·3%)
		Qatar	14·6% (12·9–16·4%)	-13·1% (-25·4 to 2·9%)	35·58 (26·44–47·81)	-62% (-73·8 to -47%)
		Saudi Arabia	18·1% (15·9–20·5%)	12% (-4·1 to 31·7%)	138·77 (102–176·23)	-34·4% (-55·3 to 2·7%)
		Syrian Arab Republic	21·8% (19·8–23·8%)	-14·4% (-23 to -5%)	182·87 (133·16–249·79)	-55·8% (-69·1 to -34·5%)
		Tunisia	22·1% (19·8–24·6%)	-6·8% (-17·2 to 3·6%)	71·29 (50·97–97·38)	-59·4% (-72·9 to -39·8%)
		Türkiye	25·4% (23·2–27·5%)	-17·5% (-26·2 to -8·8%)	101·31 (81·14–125·61)	-48·1% (-65·2 to -30·6%)
		United Arab Emirates	20·7% (18·2–23·2%)	12·8% (-2·5 to 30·1%)	93·26 (62·81–138·84)	-59·5% (-74·5 to -38·8%)
		Yemen	24·9% (22·4–27·6%)	-2·6% (-14·3 to 10·6%)	220·58 (158·34–303·79)	-54·9% (-68·3 to -33·9%)
		Afghanistan	13·5% (11·7–15·6%)	41·9% (26·6 to 62·2%)	188·56 (130·84–258·86)	-23·4% (-45·1 to 5·6%)
		Sudan	17% (14–20·2%)	-5·5% (-20·3 to 13%)	131·16 (83·33–194·53)	-62·7% (-74·1 to -45·6%)

Table S15 Burden of neurological conditions attributable to risk factors in North Africa and Middle East countries

			DALYs (95% UI)			
			PAF (%)	PAF change (%)	Rate per 100,000	Rate change (%)
Risk factors	Causes	Locations	2019	From 1990 to 2019	2019	From 1990 to 2019
	Subarachnoid haemorrhage	Global	21·8% (20·1–23·7%)	-13% (-18·8 to -2·3%)	29·77 (25·98–34·23)	-60·2% (-66·8 to -40·1%)
		North Africa and Middle East	18% (16·1–20·1%)	21·3% (0·3 to 50·2%)	16·9 (14·07–21·42)	-55% (-67·9 to -29·2%)
		Algeria	17·8% (15·2–20·7%)	0·4% (-15·5 to 21·9%)	13·73 (9·55–19·72)	-64·4% (-76·6 to -42·7%)
		Bahrain	16·1% (13·7–18·8%)	-3·4% (-22·1 to 20·9%)	10·14 (7·07–15·15)	-59·4% (-74 to -37·6%)
		Egypt	12·2% (9·1–17·3%)	98·4% (31·3 to 254·7%)	14·04 (9·14–22·03)	-54·4% (-73·3 to -14·1%)
		Iran	16·8% (15·1–18·9%)	-4·9% (-20·2 to 17·7%)	9·16 (7·66–11·5)	-57·9% (-71·2 to -34·7%)
		Iraq	21·9% (19·2–25·1%)	1% (-17·4 to 22·8%)	15·87 (11·26–23·42)	-63·6% (-78·5 to -38·5%)
		Jordan	26·5% (23·6–29·3%)	-11·8% (-23·1 to 1·4%)	10·32 (7·54–12·9)	-55·7% (-70·3 to -35%)
		Kuwait	19·8% (17·9–21·9%)	-5·2% (-16·8 to 7·9%)	9·3 (7·38–11·51)	-38·3% (-52·6 to -12·9%)
		Lebanon	33·1% (29·6–37%)	27·3% (11·6 to 45%)	8·85 (6·09–12·7)	-54·5% (-74 to -25·3%)
		Libya	14·8% (12–18·5%)	39·6% (1·3 to 99·7%)	10·69 (7·07–17·27)	-52·9% (-69·9 to -21·6%)
		Morocco	12·9% (10·8–15·3%)	-15·5% (-31·7 to 5·9%)	13·08 (8·3–20·69)	-65·2% (-77·8 to -38%)
		Palestine	19·9% (17·3–22·5%)	0·5% (-14·6 to 19·8%)	9·98 (7·62–12·41)	-45·8% (-62·7 to -16·8%)
		Oman	9·3% (7·7–11·6%)	-15·9% (-37·4 to 21·3%)	8·3 (5·28–15·24)	-68·6% (-81·8 to -34·8%)
		Qatar	16·4% (14·1–18·7%)	-1·9% (-23·5 to 31·2%)	13·75 (9·63–18·78)	-55·2% (-76 to -23·2%)
		Saudi Arabia	17% (14·4–19·8%)	11% (-13·4 to 42·8%)	8·44 (5·71–11·53)	-47·4% (-72·7 to -7·2%)
		Syrian Arab Republic	13% (10·5–16·3%)	27·3% (-10·2 to 79·1%)	14·23 (10·17–19·99)	-56·2% (-74·2 to -23·8%)
		Tunisia	20·7% (16·8–24·4%)	-1·3% (-20·8 to 23·8%)	13·43 (8·64–20·05)	-63% (-78·4 to -34·8%)
		Türkiye	29·1% (26·4–31·6%)	-8·3% (-19·9 to 6·6%)	29·36 (22·47–37·18)	-44·8% (-66 to -2·9%)
		United Arab Emirates	19·5% (16·1–23%)	26·4% (2·8 to 60·4%)	11·92 (5·67–20·05)	-55·3% (-75 to -23·2%)
		Yemen	23·1% (19·7–26·4%)	0·2% (-14·7 to 20·6%)	38·15 (18·99–66·87)	-48·9% (-65·7 to -13·1%)
		Afghanistan	12·5% (9·4–15·1%)	40·2% (19 to 62·5%)	29·55 (10·27–55·61)	-21·1% (-47·2 to 28·7%)
		Sudan	15·6% (11·9–19·3%)	1·7% (-18·3 to 38·3%)	19·47 (9·49–36·91)	-63·8% (-77·5 to -27·7%)
	Neurological disorders*	Global	3·6% (1·2–7·9%)	-13·1% (-19·6 to -6·3%)	45·19 (14·11–110·93)	-14·3% (-20·7 to -7·7%)
		North Africa and Middle East	3·9% (1·4–8·5%)	-4·9% (-15 to 7·7%)	54·03 (17·76–133·86)	-9·3% (-18·6 to 5·1%)
		Algeria	5% (1·8–10·5%)	-7·8% (-25·2 to 17·4%)	68·11 (22·21–168·08)	-16·6% (-31·9 to 2·7%)
		Bahrain	4·3% (1·5–9·3%)	-3·5% (-25·8 to 32·5%)	59·06 (19·52–147·58)	-12·4% (-30·3 to 15·9%)
		Egypt	5·1% (1·7–11·4%)	35·4% (11·7 to 66·1%)	67·6 (21·04–170·41)	36% (12·5 to 64·8%)
		Iran	3·2% (1·2–6·6%)	-5·2% (-19·3 to 11·1%)	43·9 (15·3–103·21)	-9·8% (-22·8 to 9·5%)
		Iraq	4·7% (1·5–10·2%)	-10·3% (-27·2 to 7·9%)	62·4 (18·59–157·58)	-12·1% (-27·6 to 4·4%)
		Jordan	5·9% (2·1–12·6%)	-11·5% (-29·1 to 11·8%)	75·25 (25·73–180·58)	-16·4% (-32·9 to 5%)
		Kuwait	5·3% (1·9–11%)	0·4% (-19·3 to 28·9%)	67·57 (24–157·09)	-4·9% (-21·1 to 17·1%)
		Lebanon	8% (3–16·5%)	26·9% (2·6 to 61·2%)	106·34 (37·65–253·87)	21·7% (-1·5 to 64·9%)
		Libya	3·6% (1·2–7·5%)	-21·2% (-37·2 to -1·5%)	49·33 (15·38–122·14)	-23·8% (-40·5 to -1·8%)
		Morocco	2·4% (0·8–5·3%)	-28·2% (-45·3 to -10·8%)	32·56 (9·63–84·56)	-26·7% (-43·5 to -11·5%)
		Palestine	3·9% (1·2–8·3%)	-9·6% (-29 to 16·9%)	52·99 (15·44–138·04)	-14·8% (-31·8 to 9·4%)
		Oman	2·3% (0·7–5%)	-23·1% (-42·2 to 0·7%)	30·9 (8·53–76·91)	-22·5% (-39·9 to 1·7%)
		Qatar	3·9% (1·2–8·6%)	56·4% (17·4 to 121·7%)	53·38 (13·73–139·17)	52·5% (12·9 to 114·8%)
		Saudi Arabia	2·5% (0·8–5·6%)	3% (-29·9 to 47·1%)	35·89 (9·91–93·98)	2·8% (-27·6 to 47·7%)
		Syrian Arab Republic	5% (1·7–10·6%)	-15·4% (-32·9 to 6·2%)	65·3 (20·64–160·79)	-14·3% (-34·1 to 13·8%)
		Tunisia	4·7% (1·6–9·7%)	-15·2% (-30·7 to 4·2%)	62·44 (20·12–151·74)	-17·3% (-33·8 to 9·5%)
		Türkiye	4% (1·5–8·3%)	-12·8% (-29·5 to 14·7%)	59·17 (20·16–143·58)	-21·3% (-35·1 to -0·3%)
		United Arab Emirates	3·4% (1–8·2%)	20·9% (-11·6 to 68·3%)	49·74 (12·83–129·24)	12·4% (-15·1 to 53·1%)
		Yemen	4·8% (1·7–10·2%)	14·1% (-8·6 to 39·4%)	63·18 (20·28–160·47)	11·1% (-12 to 37·7%)
		Afghanistan	2·4% (0·8–5·3%)	37·1% (3·3 to 82·5%)	36·55 (10·2–96·71)	22·5% (-7·1 to 57·8%)
		Sudan	3·6% (1·2–8%)	7·2% (-16 to 41·1%)	48·02 (14·24–122·21)	-1·2% (-21·5 to 25·8%)
	Alzheimer's disease and other dementias	Global	15·1% (9·7–20·3%)	-17·3% (-22·8 to -13%)	51·3 (20·5–116·23)	-14·2% (-20·2 to -8·4%)
		North Africa and Middle East	15·4% (9·9–20·7%)	-9·8% (-16·6 to -3·1%)	59·52 (23·58–138·55)	-10·8% (-19·1 to 0·9%)
		Algeria	18·3% (12–24·6%)	-15·3% (-26·2 to -3·8%)	73·48 (28·36–173·87)	-19·7% (-34·5 to -3·1%)
		Bahrain	16·2% (9·7–22·5%)	-13·2% (-29·5 to 6·2%)	65·04 (24·5–154·34)	-16·5% (-33·8 to 6·7%)

Table S15 Burden of neurological conditions attributable to risk factors in North Africa and Middle East countries

Risk factors	Causes	Locations	DALYs (95% UI) PAF (%) 2019	PAF change (%) From 1990 to 2019	Rate per 100,000 2019	Rate change (%) From 1990 to 2019
		Egypt	21·2% (13·7–27·8%)	35·9% (18·8 to 56%)	76·64 (30·52–179·39)	36·1% (12 to 65·5%)
		Iran	11·9% (7·4–16·7%)	-11·1% (-24·2 to 2%)	45·95 (17·5–104·67)	-11·3% (-24·8 to 7·2%)
		Iraq	18·7% (12·3–25%)	-13·4% (-26·3 to -1·8%)	71·81 (28·39–166·42)	-11·6% (-27·2 to 4%)
		Jordan	22·2% (14·7–29·6%)	-16·1% (-28·4 to -3·5%)	81·91 (33·06–185·32)	-19·7% (-35·1 to -3·1%)
		Kuwait	18·4% (11·8–25·6%)	-6·4% (-21 to 10·4%)	71·55 (28·66–161·31)	-8·6% (-24·7 to 10·3%)
		Lebanon	28·6% (18·5–37·6%)	22·3% (3 to 42·1%)	113·14 (44·96–260·28)	19·3% (-3·1 to 61·1%)
		Libya	13% (8·3–17·5%)	-20·8% (-31·4 to -9·4%)	54·03 (20–125·95)	-24·3% (-40 to -4%)
		Morocco	9·4% (5·6–13·4%)	-25·4% (-38·9 to -13·3%)	36·19 (13·29–88·23)	-25·1% (-40·2 to -9·7%)
		Palestine	15·6% (10·1–20·9%)	-12·8% (-27·7 to 2·2%)	60·57 (23·4–143·42)	-16·6% (-32·9 to 7%)
		Oman	8·2% (4·9–11·8%)	-18·9% (-34·6 to 1·2%)	34·46 (12·68–81·66)	-21·6% (-37·8 to 3·2%)
		Qatar	15% (9–21%)	37·9% (10·1 to 68%)	60·88 (22·14–145·63)	42·2% (4·6 to 85·2%)
		Saudi Arabia	10·6% (6·3–15·5%)	5% (-20·1 to 34·6%)	41·4 (15·38–99)	2·7% (-25·9 to 43·7%)
		Syrian Arab Republic	18·5% (11·7–25·1%)	-17·8% (-30·7 to -5·4%)	72·09 (27·43–168·15)	-15·6% (-34·6 to 10%)
		Tunisia	17·1% (11·6–22·7%)	-15·8% (-26·7 to -4·5%)	68·77 (27·05–161·72)	-17·5% (-34·3 to 6·7%)
		Türkiye	16·2% (10·5–21·7%)	-23·1% (-34·6 to -13·1%)	64·14 (24·87–147·81)	-24·6% (-37·9 to -8·4%)
		United Arab Emirates	15·5% (9·4–22·5%)	16·7% (-6·1 to 46·1%)	56·89 (20·99–135·49)	10·7% (-15·4 to 49%)
		Yemen	18·1% (11·5–24·6%)	10·9% (-7·5 to 31%)	69·46 (26·81–167·49)	10·5% (-12·5 to 38·1%)
		Afghanistan	9·2% (5·6–13·3%)	24·6% (-1·8 to 51·6%)	39·94 (13·66–101·34)	19·9% (-8·2 to 51·3%)
		Sudan	14·8% (8·9–21·3%)	0·5% (-18·3 to 22·7%)	54·37 (20·43–131·07)	-3·5% (-23·1 to 20·4%)
	Parkinson's disease	Global	-10% (-15·1–-5·2%)	-22·2% (-27·5 to -17·1%)	-8·02 (-12·33–-4·11)	-20·4% (-27·9 to -12·9%)
		North Africa and Middle East	-9·1% (-13·4–-4·8%)	-19·7% (-30 to -9·3%)	-7·65 (-11·54–-4·01)	-19% (-31·8 to -1·7%)
		Algeria	-9·1% (-13·6–-4·9%)	-24·4% (-39·5 to -8·4%)	-7·22 (-11·49–-3·66)	-38% (-55·3 to -14·6%)
		Bahrain	-8·2% (-12·5–-4·4%)	-28·7% (-46·2 to -5·5%)	-7·31 (-11·81–-3·64)	-38·3% (-57·6 to -11·6%)
		Egypt	-10·7% (-16·1–-5·5%)	21·8% (-1·9 to 50·1%)	-10·55 (-17·15–-5·12)	36·2% (0·6 to 80·7%)
		Iran	-6·4% (-9·8–-3·2%)	-23·3% (-37·6 to -7·8%)	-4·95 (-7·59–-2·39)	-20·8% (-40·1 to 4·9%)
		Iraq	-13% (-19·8–-7%)	-22% (-38·2 to -5%)	-11·14 (-17·84–-5·86)	-8·6% (-34·3 to 25·6%)
		Jordan	-13% (-19·7–-6·5%)	-27·2% (-42·1 to -7·9%)	-9·66 (-15·35–-4·7)	-37·1% (-54·1 to -17·7%)
		Kuwait	-10·5% (-15·4–-5·8%)	-10·8% (-30·1 to 12·8%)	-5·92 (-9·05–-3·05)	-34·7% (-51·7 to -13·6%)
		Lebanon	-17% (-25·5–-9%)	26·7% (0·5 to 57·3%)	-11·82 (-19·08–-6·02)	8·8% (-22·7 to 55·2%)
		Libya	-8·3% (-12·4–-4·4%)	-25·8% (-40·3 to -8·2%)	-7·03 (-11·29–-3·48)	-17·9% (-41·6 to 19·3%)
		Morocco	-5·2% (-8–-2·6%)	-34·1% (-46·6 to -17·7%)	-5 (-7·8–-2·41)	-7·9% (-33·3 to 24·8%)
		Palestine	-10·9% (-16·1–-5·7%)	-17·4% (-37·6 to 6·4%)	-10·11 (-15·79–-4·83)	-21·6% (-45·4 to 21·8%)
		Oman	-3·9% (-5·9–-2·1%)	-30·7% (-48·5 to -6·5%)	-4·9 (-7·63–-2·26)	-9·3% (-41·2 to 40%)
		Qatar	-6·2% (-9·4–-3·2%)	-11·5% (-34·2 to 15·1%)	-9·14 (-15·26–-4·55)	1·3% (-32·7 to 50·8%)
		Saudi Arabia	-5·9% (-8·8–-3·2%)	6·3% (-23·4 to 41·6%)	-6·55 (-10·08–-3·22)	7·7% (-29·9 to 74·6%)
		Syrian Arab Republic	-10·1% (-15·7–-5·2%)	-32% (-46·8 to -16%)	-8·51 (-13·98–-3·93)	-22·7% (-45·7 to 10%)
		Tunisia	-12·1% (-18·6–-6·1%)	-21·5% (-37·9 to -2·7%)	-8·79 (-14·38–-4·17)	-12·8% (-38·8 to 22·4%)
		Türkiye	-10·9% (-16·6–-5·3%)	-36·7% (-49·7 to -22·7%)	-8·21 (-13·68–-4·03)	-41·9% (-57·4 to -21·5%)
		United Arab Emirates	-7·3% (-11·3–-3·7%)	13·5% (-13·9 to 47·3%)	-9·45 (-15·95–-4·54)	1·8% (-31·8 to 50·1%)
		Yemen	-10·8% (-15·6–-5·9%)	-7·1% (-25·1 to 18·1%)	-8·27 (-12·97–-4·37)	7·2% (-23·8 to 55·5%)
		Afghanistan	-4·4% (-6·9–-2·3%)	21·8% (-7·4 to 53·8%)	-5·04 (-8·04–-2·55)	14·4% (-18·8 to 57·9%)
		Sudan	-8·8% (-13·2–-4·8%)	-11% (-33·9 to 16·9%)	-7·55 (-12·17–-3·96)	-15% (-41·1 to 31·7%)
	Multiple sclerosis	Global	13·7% (10·4–17·2%)	-25·6% (-30·1 to -19%)	1·92 (1·41–2·54)	-35·4% (-41·4 to -28·2%)
		North Africa and Middle East	10·8% (7·8–14·5%)	-12% (-28·2 to 5%)	2·15 (1·51–2·96)	-6·7% (-28·5 to 8·8%)
		Algeria	9·3% (6·6–12·1%)	-13% (-31·7 to 11·3%)	1·86 (1·19–2·65)	-1·8% (-30·6 to 27·5%)
		Bahrain	10·7% (7·7–13·9%)	-15·3% (-28 to -1·2%)	1·33 (0·85–1·95)	-4·7% (-30 to 22·4%)
		Egypt	10·3% (3·6–17·8%)	24·9% (-45·6 to 145·4%)	1·51 (0·82–3·25)	30·4% (-7 to 67·5%)
		Iran	9·8% (7·2–13·5%)	-6·6% (-22·4 to 14·2%)	2·89 (1·89–4·69)	-6·3% (-34·1 to 15%)
		Iraq	12·1% (8·9–16·1%)	-18·9% (-32·4 to -2·3%)	1·74 (1·13–2·56)	-8·9% (-32·6 to 16·9%)
		Jordan	15·8% (12·1–19·5%)	-3·2% (-18·5 to 16·1%)	3 (2·02–4·11)	-9·9% (-29·5 to 16·2%)
		Kuwait	11·2% (7·8–14·7%)	-25·4% (-36·7 to -11·7%)	1·94 (1·24–2·79)	3% (-15·4 to 26%)

Table S15 Burden of neurological conditions attributable to risk factors in North Africa and Middle East countries

			**DALYs (95% UI)**			
			PAF (%)	PAF change (%)	Rate per 100,000	Rate change (%)
Risk factors	Causes	Locations	2019	From 1990 to 2019	2019	From 1990 to 2019
		Lebanon	21·4% (16·2–26·9%)	21·7% (4·7 to 41·8%)	5·02 (3·35–7)	47% (13·9 to 92·5%)
		Libya	10·8% (7·3–14·3%)	-9·5% (-31·6 to 13·1%)	2·34 (1·48–3·23)	20·7% (-13·8 to 67%)
		Morocco	6·8% (4·9–8·8%)	-27·9% (-40·8 to -9·7%)	1·36 (0·87–1·89)	-9·9% (-36·8 to 23·1%)
		Palestine	13% (9·6–17·7%)	-5·3% (-24·9 to 23·5%)	2·54 (1·64–3·81)	2·9% (-34·2 to 43%)
		Oman	7·9% (5·6–10·6%)	-16·9% (-35·2 to 7·2%)	1·34 (0·82–1·93)	-0·3% (-40 to 37·9%)
		Qatar	9·8% (7–12·7%)	0·7% (-16·8 to 21·9%)	1·64 (1·03–2·38)	34·6% (-2·4 to 80·1%)
		Saudi Arabia	8·9% (6·4–11·9%)	27·3% (1·9 to 61·3%)	1·04 (0·66–1·52)	56·6% (9·9 to 129·6%)
		Syrian Arab Republic	11·4% (8·4–14·4%)	-19·7% (-34·7 to -3·7%)	1·72 (1·15–2·47)	-7·3% (-31·4 to 18·8%)
		Tunisia	11·1% (8·1–14·1%)	-11·3% (-26·6 to 11·1%)	2·46 (1·6–3·44)	11·5% (-15·6 to 43·6%)
		Türkiye	15·5% (10·5–19·8%)	-14·4% (-35·8 to 19·7%)	3·24 (2·26–4·44)	-24·4% (-44·7 to -3·9%)
		United Arab Emirates	11·2% (7·7–15·2%)	-9·7% (-27·1 to 9·9%)	2·31 (1·23–3·95)	7·7% (-31·6 to 58·1%)
		Yemen	14·3% (10·2–18·6%)	-7·4% (-23·3 to 13·2%)	1·99 (1·25–2·86)	16·6% (-22·8 to 68%)
		Afghanistan	6·2% (3·9–9·2%)	62·7% (27·1 to 111·4%)	1·66 (0·95–2·79)	81·4% (26·4 to 153·6%)
		Sudan	8·6% (5·6–12%)	-12·1% (-31·4 to 18%)	1·2 (0·68–1·86)	5·8% (-31·7 to 50·8%)

Data in parentheses are 95% uncertainty intervals. Percentage data is rounded to one decimal place, and rate data per 100,000 is rounded to two decimal place. Percentages of DALYs are not mutually exclusive: the sum of percentages of DALYs in the columns exceeds the totals for all risk factors combined because of overlap between various risk factors. The crude sum of population attributable fraction (PAF) of the risk factors might exceed 100% because the effects of many of these risk factors are mediated partly or fully through another risk factor or risk factors. DALY=disability-adjusted life-year. $PM_{2·5}$=particulate matter with a diameter of <25 µm. *Neurological disorders in the Global Burden of Disease Study 2019 include Alzheimer's disease and other dementias, Parkinson's disease, idiopathic epilepsy, multiple sclerosis, headache disorders (including migraine and tension-type headache), motor neuron disease, and other neurological disorders. Metabolic risk factors† cluster includes high body-mass index, high fasting plasma glucose, high LDL cholesterol, high systolic blood pressure, and kidney dysfunction. ‡Behavioural risk factors cluster includes tobacco (including smoking and second-hand smoking), dietary risks (diet high in sodium, diet low in fruits, diet low in vegetables, diet high in red meat, and diet low in whole grains), low physical activity, alcohol consumption, and child and maternal malnutrition (including low birth weight and short gestation). §Environmental risk factors cluster includes air pollution (including ambient $PM_{2·5}$ pollution and household air pollution from solid fuels), non-optimal temperature (including low ambient temperature and high ambient temperature), and other environmental risks (including lead exposure). Risk factors with insufficient data are not reported here.

Table S16 Regional and national disabilities and deaths related to neurological conditions attributable to risk factors in North Africa and Middle East countries
(NEXT PAGE)

Global (p 322), North Africa and Middle East (p 327), Algeria (p 332), Bahrain (p 337), Egypt (p 342), Iran (p 347), Iraq (p 352), Jordan (p 357), Kuwait (p 362), Lebanon (p 368), Libya (p 373), Morocco (p 378), Palestine (p 383), Oman (p 388), Qatar (p 393), Saudi Arabia (p 398), Syrian Arab Republic (p 403), Tunisia (p 409), Türkiye (p 414), United Arab Emirates (p 419), Yemen (p 424), Afghanistan (p 429), Sudan (p 434)

Table S16 Regional and national disabilities and deaths related to neurological conditions attributable to risk factors in North Africa and Middle East countries

Location<br>Risk factor (alphabetic order)	Cause	DALYs (95% UI)<br>PAF (%)<br>2019	 Rate per 100,000<br>2019	 Rate change (%)<br>From 1990 to 2019	Deaths (95% UI)<br>PAF (%)<br>2019	 Rate per 100,000<br>2019	 Rate change (%)<br>From 1990 to 2019
**Global**							
Air pollution	Meningitis	2% (1·7–2·2%)	4·6 (3·6–6)	-53·9% (-63·3 to -41·2%)	1·6% (1·4–1·8%)	0·1 (0–0·1)	-53·9% (-63·4 to -41·2%)
	Encephalitis	0·6% (0·5–0·7%)	0·4 (0·3–0·5)	-39·8% (-60 to -6·3%)	0·4% (0·3–0·5%)	<0·1 (<0·1–<0·1)	-39·8% (-60 to -6·3%)
	Stroke	29·7% (27·2–32·4%)	525·6 (463·9–589·9)	-40·8% (-47·4 to -34·3%)	25·1% (22·7–27·7%)	21·1 (18·6–23·9)	-42·5% (-49·6 to -35·2%)
	Ischaemic stroke	25·5% (23–28·1%)	203·4 (177·8–231·6)	-28·6% (-37·8 to -19·2%)	20·9% (18·7–23·4%)	9·1 (7·9–10·4)	-33·6% (-43·3 to -23·2%)
	Intracerebral haemorrhage	33·8% (31–36·6%)	281·2 (248–314·9)	-43·2% (-50·3 to -36·4%)	29·9% (27·2–32·8%)	10·8 (9·5–12·1)	-44% (-51·5 to -37%)
	Subarachnoid haemorrhage	30% (26·8–33·1%)	41 (33·4–49·7)	-61·9% (-68·1 to -45·9%)	26·9% (23·7–30%)	1·3 (1–1·5)	-67·1% (-72·9 to -50·5%)
Alcohol use	Stroke	5·8% (4·2–7·4%)	102·8 (72·3–133·1)	-24·8% (-35·9 to -12·2%)	5% (3·5–6·5%)	4·2 (2·9–5·6)	-24·6% (-36·4 to -9·8%)
	Ischaemic stroke	2·1% (0·3–3·9%)	16·9 (2·1–31·5)	-9·4% (-117·7 to 163·3%)	1·7% (0–3·4%)	0·7 (0–1·5)	-13·1% (-204·6 to 200·7%)
	Intracerebral haemorrhage	10·3% (7·4–13·4%)	85·9 (60·4–111·9)	-27·3% (-37·5 to -15·8%)	9·6% (6·8–12·5%)	3·4 (2·4–4·5)	-26·7% (-37·3 to -15·2%)
	Neurological disorders*	1·5% (0·8–2·5%)	17·2 (11·5–24)	-7% (-16·7 to 3·9%)	0·7% (0·3–1·4%)	0·2 (0·1–0·2)	-7·3% (-16·6 to 7·4%)
	Idiopathic epilepsy	10·1% (7·3–13%)	17·2 (11·5–24)	-7% (-16·7 to 3·9%)	12·6% (9·1–16%)	0·2 (0·1–0·2)	-7·3% (-16·6 to 7·4%)
All risk factors	Meningitis	6·8% (6·1–7·7%)	15·9 (12·7–20·6)	-52% (-61·5 to -39·2%)	5·4% (4·8–6·3%)	0·2 (0·1–0·2)	-52% (-61·5 to -39·2%)
	Encephalitis	2·5% (1·9–2·9%)	1·6 (1·2–2·1)	-34·6% (-56 to 1·4%)	1·5% (1·2–1·9%)	<0·1 (<0·1–<0·1)	-34·6% (-56 to 1·4%)
	Stroke	86·4% (83·4–89·2%)	1526·7 (1408·4–1642·6)	-34·5% (-40·2 to -29·2%)	84·3% (80·3–88·5%)	70·9 (64·5–77·1)	-36·5% (-41·8 to -31·1%)
	Ischaemic stroke	85% (80·2–89·9%)	679 (611·6–747·2)	-28·5% (-34·9 to -22·9%)	82·4% (75·5–89·7%)	35·8 (31·7–40·1)	-34·1% (-39·9 to -28·5%)
	Intracerebral haemorrhage	88·1% (85·1–90·5%)	733·6 (675·8–792)	-35·4% (-42·5 to -29·1%)	86·7% (82·5–90·3%)	31·2 (28·5–34)	-35·2% (-42·4 to -28·8%)
	Subarachnoid haemorrhage	83·6% (80·3–86·7%)	114·1 (99·8–130·1)	-53·3% (-60 to -36·1%)	83·5% (78·8–87·6%)	3·9 (3·4–4·4)	-57·4% (-64 to -38·8%)
	Neurological disorders*	9·9% (4·6–18·6%)	122·4 (53·4–286·7)	6% (-2·4 to 13·6%)	20·7% (9·5–33·1%)	6·9 (1·4–20·6)	8·3% (-1 to 21·5%)
	Alzheimer's disease and other dementias	32·8% (21·8–45·8%)	111·3 (43·2–273·9)	6·9% (-2·2 to 15·2%)	30·7% (19·8–43·8%)	7·1 (1·6–20·9)	6·8% (-2·4 to 16·2%)
	Parkinson's disease	-10% (-15·1–-5·2%)	-8 (-12·3–-4·1)	-20·4% (-27·9 to -12·9%)	-8·5% (-13–-4·3%)	-0·4 (-0·6–-0·2)	-21·1% (-29·3 to -12·7%)
	Idiopathic epilepsy	10·1% (7·3–13%)	17·2 (11·5–24)	-7% (-16·7 to 3·9%)	12·6% (9·1–16%)	0·2 (0·1–0·2)	-7·3% (-16·6 to 7·4%)
	Multiple sclerosis	13·7% (10·4–17·2%)	1·9 (1·4–2·5)	-35·4% (-41·4 to -28·2%)	13·4% (10·1–16·9%)	<0·1 (<0·1–0·1)	-35·4% (-45·6 to -27·1%)
Ambient particulate matter pollution	Meningitis	0·5% (0·3–0·7%)	1·2 (0·8–1·8)	-8·4% (-36·9 to 38·4%)	0·4% (0·3–0·6%)	<0·1 (<0·1–<0·1)	-8·4% (-36·9 to 38·3%)
	Encephalitis	0·3% (0·2–0·4%)	0·2 (0·1–0·3)	34·4% (-22·1 to 163·3%)	0·2% (0·1–0·2%)	<0·1 (<0·1–<0·1)	34·4% (-22·1 to 163·3%)
	Stroke	19·7% (16·4–22·5%)	348·1 (283·3–404·4)	9% (-14·3 to 43·3%)	16·9% (14–19·4%)	14·3 (11·8–16·6)	2·2% (-19·5 to 34·8%)
	Ischaemic stroke	18·3% (15·4–20·9%)	146·2 (119·8–171·2)	11·7% (-9·2 to 45·1%)	15·1% (12·7–17·3%)	6·6 (5·5–7·7)	-2% (-20·9 to 27%)
	Intracerebral haemorrhage	21% (17·1–24·3%)	174·8 (140·1–206)	14·8% (-13·4 to 58·8%)	19% (15·6–21·8%)	6·8 (5·5–8·1)	14·1% (-14·2 to 58·8%)
	Subarachnoid haemorrhage	19·8% (16·4–22·9%)	27·1 (21·3–32·7)	-25·4% (-46·6 to 10·9%)	18% (14·9–20·8%)	0·8 (0·7–1)	-32·6% (-53·3 to 6·7%)
Behavioural risk factors‡	Meningitis	6·4% (5·7–7·3%)	14·9 (11·9–19·2)	-51·4% (-61 to -38·4%)	5·1% (4·5–5·9%)	0·2 (0·1–0·2)	-51·4% (-61 to -38·4%)
	Encephalitis	2·3% (1·8–2·8%)	1·5 (1·2–2)	-34·1% (-55·6 to 2%)	1·5% (1·1–1·8%)	<0·1 (<0·1–<0·1)	-34·1% (-55·6 to 2%)

Table S16 Regional and national disabilities and deaths related to neurological conditions attributable to risk factors in North Africa and Middle East countries

Location<br>Risk factor (alphabetic order)	Cause	DALYs (95% UI)<br>PAF (%)<br>2019	Rate per 100,000<br>2019	Rate change (%)<br>From 1990 to 2019	Deaths (95% UI)<br>PAF (%)<br>2019	Rate per 100,000<br>2019	Rate change (%)<br>From 1990 to 2019
	Stroke	46·6% (40·5–53·5%)	823·6 (707·3–963·1)	-38·7% (-44·6 to -33·2%)	41·2% (35·1–48·1%)	34·7 (29–41·5)	-40·3% (-46·1 to -34·6%)
	Ischaemic stroke	42% (34·9–49·1%)	335·4 (275·7–404)	-31·1% (-38·1 to -25·2%)	36·6% (29·5–44·1%)	15·9 (12·6–19·8)	-36·2% (-43·2 to -30·3%)
	Intracerebral haemorrhage	51·1% (44–58·9%)	425·2 (358·9–499·8)	-39·7% (-47 to -33·2%)	46·7% (39·4–54·9%)	16·8 (13·9–20·2)	-39·7% (-47·2 to -33·2%)
	Subarachnoid haemorrhage	46·1% (39·2–53·6%)	62·9 (51·5–77·1)	-58·6% (-65·2 to -41·2%)	42·4% (35·1–50·8%)	2 (1·6–2·5)	-62·6% (-69 to -43·4%)
	Neurological disorders*	5·1% (2·4–9·3%)	62·4 (29·4–130)	-12·4% (-17·9 to -6·1%)	8·1% (3–13·2%)	2·7 (0·4–7·9)	-14·9% (-21·3 to -4·2%)
	Alzheimer's disease and other dementias	15·1% (9·7–20·3%)	51·3 (20·5–116·2)	-14·2% (-20·2 to -8·4%)	12·6% (7·8–17·3%)	2·9 (0·6–8·1)	-15·9% (-22·4 to -8·4%)
	Parkinson's disease	-10% (-15·1–-5·2%)	-8 (-12·3–-4·1)	-20·4% (-27·9 to -12·9%)	-8·5% (-13–-4·3%)	-0·4 (-0·6–-0·2)	-21·1% (-29·3 to -12·7%)
	Idiopathic epilepsy	10·1% (7·3–13%)	17·2 (11·5–24)	-7% (-16·7 to 3·9%)	12·6% (9·1–16%)	0·2 (0·1–0·2)	-7·3% (-16·6 to 7·4%)
	Multiple sclerosis	13·7% (10·4–17·2%)	1·9 (1·4–2·5)	-35·4% (-41·4 to -28·2%)	13·4% (10·1–16·9%)	<0·1 (<0·1–0·1)	-35·4% (-45·6 to -27·1%)
Child and maternal malnutrition	Meningitis	6·4% (5·7–7·3%)	14·9 (11·9–19·2)	-51·4% (-61 to -38·4%)	5·1% (4·5–5·9%)	0·2 (0·1–0·2)	-51·4% (-61 to -38·4%)
	Encephalitis	2·3% (1·8–2·8%)	1·5 (1·2–2)	-34·1% (-55·6 to 2%)	1·5% (1·1–1·8%)	<0·1 (<0·1–<0·1)	-34·1% (-55·6 to 2%)
Diet high in red meat	Stroke	6·9% (4·4–9·1%)	122·6 (77–163·3)	-28·5% (-36 to -17·3%)	5·8% (3·6–7·7%)	4·9 (3–6·5)	-32·1% (-39 to -23·5%)
	Ischaemic stroke	6·3% (3·1–8·3%)	50·1 (25–68·2)	-25·6% (-32·2 to -16·9%)	5% (2·4–6·6%)	2·2 (1–3)	-35% (-41·2 to -27·9%)
	Intracerebral haemorrhage	7·3% (3·7–10·3%)	61·1 (30·7–87·4)	-25·6% (-35·4 to -2·4%)	6·6% (3·1–9·3%)	2·4 (1·1–3·4)	-24·8% (-34·7 to -2·1%)
	Subarachnoid haemorrhage	8·4% (4·3–11·9%)	11·4 (6·1–15·9)	-48·1% (-56·5 to -28·7%)	7·5% (3·8–10·6%)	0·3 (0·2–0·5)	-51·2% (-60·1 to -28·8%)
Diet high in sodium	Stroke	12% (3·9–23·8%)	212·8 (68·4–422·7)	-40·7% (-55·8 to -31·3%)	10·3% (2·9–21·3%)	8·7 (2·5–18·1)	-41·1% (-56·6 to -31·7%)
	Ischaemic stroke	10·4% (3·1–20·5%)	82·9 (24·8–168·4)	-24·7% (-38·3 to -13%)	8·4% (2·2–18·3%)	3·7 (0·9–8)	-29·7% (-43·8 to -18·3%)
	Intracerebral haemorrhage	13·7% (4·6–27·2%)	114·4 (37·5–228·6)	-43·2% (-58·3 to -33·1%)	12·6% (3·8–25·7%)	4·5 (1·3–9·3)	-42·5% (-58 to -32·1%)
	Subarachnoid haemorrhage	11·3% (3·3–23·7%)	15·5 (4·5–32·5)	-67·1% (-81·3 to -48·2%)	10·9% (2·9–23·5%)	0·5 (0·1–1·1)	-69·9% (-83·4 to -49·9%)
Diet low in fibre	Stroke	3·5% (1–6·1%)	62·4 (18–108·3)	-50·4% (-56·2 to -45·2%)	2·9% (0·9–5·1%)	2·5 (0·7–4·3)	-52·4% (-57·5 to -47·7%)
	Ischaemic stroke	2·9% (0·8–5·1%)	23·1 (6·5–40·6)	-44·5% (-50·5 to -39·4%)	2·5% (0·7–4·3%)	1·1 (0·3–1·9)	-48·9% (-54·2 to -44%)
	Intracerebral haemorrhage	4% (1·1–6·8%)	33 (9·5–57·1)	-51·6% (-58·6 to -45·8%)	3·4% (1–5·8%)	1·2 (0·4–2·1)	-52·7% (-59·5 to -46·7%)
	Subarachnoid haemorrhage	4·6% (1·3–7·9%)	6·3 (1·7–11·3)	-60·8% (-67·2 to -47·6%)	4% (1·1–6·8%)	0·2 (0·1–0·3)	-64·7% (-70·6 to -50·7%)
Diet low in fruits	Stroke	7·2% (4·3–10·9%)	127 (75·4–194·2)	-45·1% (-52 to -38·1%)	5·8% (3·4–8·9%)	4·9 (2·8–7·6)	-46·5% (-53·1 to -40·2%)
	Ischaemic stroke	4·4% (1·2–8·1%)	35·5 (10–65·5)	-36·8% (-47·2 to -29·1%)	3·6% (1–6·6%)	1·6 (0·4–2·9)	-41·1% (-49·4 to -34·4%)
	Intracerebral haemorrhage	9·4% (5–15·2%)	77·9 (43·1–127·9)	-45·3% (-52·8 to -37·6%)	8·1% (4·4–13·2%)	2·9 (1·6–4·9)	-45·9% (-53·5 to -37·9%)
	Subarachnoid haemorrhage	9·9% (5·6–16%)	13·6 (7·3–22·3)	-58·6% (-65·2 to -44·6%)	8·7% (4·7–14·1%)	0·4 (0·2–0·7)	-63·2% (-69·6 to -48·1%)
Diet low in vegetables	Stroke	2·8% (1·1–4·6%)	50·2 (18·7–82·6)	-53·3% (-57·5 to -48·5%)	2·3% (0·9–3·7%)	1·9 (0·7–3·1)	-55·7% (-59·7 to -50·7%)
	Ischaemic stroke	1·6% (0·4–2·8%)	12·8 (3–23·1)	-48·5% (-52·7 to -38·6%)	1·4% (0·3–2·5%)	0·6 (0·1–1·1)	-51·4% (-55·5 to -41·4%)
	Intracerebral haemorrhage	3·8% (0·9–7·1%)	31·7 (7·4–58·9)	-53·7% (-59·1 to -47·9%)	3·2% (0·8–6%)	1·2 (0·3–2·2)	-56% (-61·5 to -50·4%)
	Subarachnoid haemorrhage	4·1% (0·9–7·8%)	5·7 (1·1–10·8)	-60·2% (-65·8 to -47%)	3·6% (0·8–6·8%)	0·2 (0–0·3)	-65·3% (-70·3 to -51·9%)
Diet low in whole grains	Stroke	2·3% (0·7–3·3%)	40·3 (12·1–58·8)	-31·7% (-40·9 to -25·9%)	2·2% (0·7–3·1%)	1·8 (0·6–2·7)	-38·8% (-47·7 to -33·4%)

Table S16 Regional and national disabilities and deaths related to neurological conditions attributable to risk factors in North Africa and Middle East countries

**Location**<br>Risk factor (alphabetic order)	Cause	**DALYs (95% UI)**<br>PAF (%)<br>2019	Rate per 100,000<br>2019	Rate change (%)<br>From 1990 to 2019	**Deaths (95% UI)**<br>PAF (%)<br>2019	Rate per 100,000<br>2019	Rate change (%)<br>From 1990 to 2019
	Ischaemic stroke	5% (1·6–7·3%)	40·3 (12·1–58·8)	-31·7% (-40·9 to -25·9%)	4·2% (1·4–6·1%)	1·8 (0·6–2·7)	-38·8% (-47·7 to -33·4%)
Dietary risks	Stroke	30% (22·2–39·1%)	531·3 (389·5–704·6)	-40·4% (-46·8 to -34·1%)	26% (18·8–34·9%)	21·9 (15·5–29·7)	-42·3% (-48·8 to -36·1%)
	Ischaemic stroke	26·7% (18·8–35·6%)	213·2 (146·7–289)	-31·2% (-38·6 to -24·3%)	22·6% (15·4–31·3%)	9·8 (6·6–14)	-37·3% (-44·4 to -30·6%)
	Intracerebral haemorrhage	32·8% (23·3–43·4%)	273·5 (189–364·6)	-42% (-49·7 to -34·9%)	29·7% (20·5–40·7%)	10·7 (7·2–14·8)	-42·2% (-50·4 to -34·7%)
	Subarachnoid haemorrhage	32·7% (23·6–42·3%)	44·6 (31·2–59·7)	-59·2% (-65·9 to -43·3%)	30% (20·7–40·2%)	1·4 (1–1·9)	-63·2% (-69·6 to -45·6%)
Environmental/occupational risk factors§	Meningitis	2% (1·7–2·2%)	4·6 (3·6–6)	-53·9% (-63·3 to -41·2%)	1·6% (1·4–1·8%)	0·1 (0–0·1)	-53·9% (-63·4 to -41·2%)
	Encephalitis	0·6% (0·5–0·7%)	0·4 (0·3–0·5)	-39·8% (-60 to -6·3%)	0·4% (0·3–0·5%)	<0·1 (<0·1–<0·1)	-39·8% (-60 to -6·3%)
	Stroke	37·3% (34·4–40·3%)	658·9 (586·7–729·1)	-40·6% (-46·5 to -34·5%)	34·2% (31·4–37·3%)	28·8 (25·5–32·1)	-41·2% (-47·5 to -35%)
	Ischaemic stroke	33·1% (30·3–36·2%)	264·4 (234·1–297·3)	-30·2% (-38·4 to -22·5%)	30·4% (27·6–33·7%)	13·2 (11·5–14·9)	-34·6% (-42·6 to -26·5%)
	Intracerebral haemorrhage	41·3% (38·3–44·5%)	344 (304·7–381·9)	-42·5% (-49·7 to -35·9%)	38·6% (35·6–41·9%)	13·9 (12·3–15·6)	-42% (-49·5 to -35%)
	Subarachnoid haemorrhage	37% (33·4–40·5%)	50·5 (41·8–60)	-61·7% (-67·8 to -45·5%)	35·7% (32·2–39·1%)	1·7 (1·4–2)	-65·3% (-71·2 to -48·1%)
High body-mass index	Stroke	23·6% (15·2–32·2%)	416·6 (265·7–581·1)	-1·1% (-16·5 to 24%)	15·7% (9·5–22·7%)	13·2 (7·9–19·3)	-8·9% (-22·2 to 12·1%)
	Ischaemic stroke	16·5% (10·1–23·7%)	132·1 (80·1–195·9)	-4·8% (-14·8 to 10·2%)	10·6% (5·8–16·5%)	4·6 (2·5–7·3)	-18·4% (-25·9 to -7·2%)
	Intracerebral haemorrhage	28·4% (18–39%)	236·2 (149·3–329·4)	4·9% (-14·3 to 36·6%)	20·4% (12·4–29·7%)	7·4 (4·4–10·8)	1% (-16·3 to 30·2%)
	Subarachnoid haemorrhage	35·4% (24·4–46·4%)	48·4 (32·5–65·6)	-15·4% (-34·3 to 15·6%)	26·6% (17·6–36·3%)	1·2 (0·8–1·7)	-20·7% (-40 to 12%)
	Neurological disorders*	3·4% (0·9–8%)	42·2 (11·2–107·4)	30·5% (18·3 to 56·4%)	8·5% (2·7–17·1%)	2·8 (0·5–8·4)	26·6% (14·6 to 53%)
	Alzheimer's disease and other dementias	12·5% (4·9–23·3%)	42·2 (11·2–107·4)	30·5% (18·3 to 56·4%)	12·2% (4·5–23%)	2·8 (0·5–8·4)	26·6% (14·6 to 53%)
High fasting plasma glucose	Stroke	20·1% (13·7–29·2%)	355 (241·9–512·1)	-9·8% (-17·6 to -0·5%)	21% (13·3–34·5%)	17·7 (11·3–28·8)	-13·6% (-20·9 to -4·9%)
	Ischaemic stroke	21·8% (11·6–40·4%)	174 (90·5–316·3)	-4·6% (-13·2 to 5%)	22% (10·4–47·4%)	9·6 (4·6–20·8)	-12·1% (-19·5 to -3·5%)
	Intracerebral haemorrhage	18·9% (12·1–26·9%)	157·4 (99·9–227·2)	-9·9% (-20·4 to 0·7%)	20·1% (12·3–30·9%)	7·2 (4·4–11)	-10·6% (-21 to -0·5%)
	Subarachnoid haemorrhage	17·3% (11·3–24·3%)	23·6 (14·8–33·9)	-35·7% (-45·2 to -13·8%)	19·6% (12·7–29·2%)	0·9 (0·6–1·4)	-40% (-49·7 to -16·3%)
	Neurological disorders*	2·7% (0·5–7·4%)	32·8 (5·5–105·9)	34% (28 to 47·7%)	6·8% (1·4–16·1%)	2·2 (0·3–8·2)	32·4% (26 to 47·9%)
	Alzheimer's disease and other dementias	9·7% (2·2–20·7%)	32·8 (5·5–105·9)	34% (28 to 47·7%)	9·7% (2·2–20·7%)	2·2 (0·3–8·2)	32·4% (26 to 47·9%)
High LDL cholesterol	Stroke	9·6% (5·3–16·6%)	170·2 (92·8–294·7)	-31·7% (-37·3 to -23·9%)	9·6% (3·3–20·1%)	8·1 (2·8–17)	-39·2% (-44 to -32·9%)
	Ischaemic stroke	21·3% (11·7–37%)	170·2 (92·8–294·7)	-31·7% (-37·3 to -23·9%)	18·6% (6·4–38·9%)	8·1 (2·8–17)	-39·2% (-44 to -32·9%)
High systolic blood pressure	Stroke	54·8% (47·5–61·5%)	969·4 (823·8–1110·2)	-31·7% (-37·9 to -25·5%)	51·8% (43·6–59·6%)	43·6 (36·2–51·1)	-34·9% (-40·9 to -28·7%)
	Ischaemic stroke	50·5% (40·3–60%)	403·7 (318·3–487·8)	-28·3% (-34·4 to -22·1%)	47·2% (36–59%)	20·5 (15·4–26·1)	-35% (-40·9 to -29%)
	Intracerebral haemorrhage	58·8% (49·4–67·1%)	489·2 (406–573·5)	-30·7% (-39·1 to -23·5%)	56·8% (46·4–66·9%)	20·5 (16·5–24·5)	-31·2% (-39·5 to -23·5%)
	Subarachnoid haemorrhage	56% (47·4–64·4%)	76·4 (61–91·6)	-49·3% (-57·2 to -31·7%)	56% (46·2–65·3%)	2·6 (2–3·1)	-53·5% (-61·2 to -35·3%)
High temperature	Stroke	0·8% (0·1–1·6%)	13·3 (1·4–29)	44·2% (-323·5 to 370·6%)	0·7% (0·1–1·5%)	0·6 (0·1–1·3)	46·5% (-350·9 to 366·2%)
	Ischaemic stroke	0·6% (0·1–1·2%)	4·4 (0·7–9·5)	68·7% (-457·4 to 492%)	0·6% (0·1–1·3%)	0·3 (0–0·6)	68·2% (-514·3 to 465·4%)

Table S16 Regional and national disabilities and deaths related to neurological conditions attributable to risk factors in North Africa and Middle East countries

Location<br>Risk factor (alphabetic order)	Cause	DALYs (95% UI)<br>PAF (%)<br>2019	Rate per 100,000<br>2019	Rate change (%)<br>From 1990 to 2019	Deaths (95% UI)<br>PAF (%)<br>2019	Rate per 100,000<br>2019	Rate change (%)<br>From 1990 to 2019
	Intracerebral haemorrhage	0·9% (0–2%)	7·7 (0·4–17)	37·7% (-365·6 to 312·7%)	0·8% (0·1–1·8%)	0·3 (0–0·6)	36·4% (-339·5 to 270·4%)
	Subarachnoid haemorrhage	0·9% (0·1–1·8%)	1·2 (0·2–2·6)	16·9% (-309·8 to 351·7%)	0·8% (0·1–1·7%)	<0·1 (<0·1–0·1)	13·3% (-442 to 276·6%)
Household air pollution from solid fuels	Meningitis	1·5% (1·2–1·7%)	3·4 (2·6–4·5)	-60·8% (-69·2 to -50·2%)	1·2% (0·9–1·4%)	<0·1 (<0·1–0·1)	-60·8% (-69·2 to -50·2%)
	Encephalitis	0·3% (0·2–0·4%)	0·2 (0·1–0·3)	-60·1% (-74·1 to -39·3%)	0·2% (0·1–0·3%)	<0·1 (<0·1–<0·1)	-60·1% (-74·1 to -39·3%)
	Stroke	10% (6·9–13·7%)	177·5 (122·1–242·8)	-68·8% (-76·2 to -60·7%)	8·2% (5·6–11·3%)	6·9 (4·7–9·5)	-69·8% (-77·2 to -61·5%)
	Ischaemic stroke	7·2% (4·8–10·1%)	57·2 (37·6–80·5)	-62·9% (-72·4 to -52·6%)	5·8% (3·8–8·3%)	2·5 (1·6–3·6)	-63·9% (-73·3 to -53·7%)
	Intracerebral haemorrhage	12·8% (9–17·2%)	106·4 (74·1–143·9)	-69% (-76·2 to -61·3%)	11% (7·6–15%)	3·9 (2·7–5·4)	-70·2% (-77·5 to -62·3%)
	Subarachnoid haemorrhage	10·1% (6·8–14·1%)	13·9 (8·8–20·5)	-80·5% (-85·7 to -71·1%)	8·9% (5·8–12·6%)	0·4 (0·3–0·6)	-83·8% (-88·4 to -75·1%)
Kidney dysfunction	Stroke	8·2% (6·8–9·6%)	145·1 (117·6–171)	-24·5% (-31·7 to -17·9%)	7·5% (5·6–9·2%)	6·3 (4·7–7·8)	-26·7% (-33·8 to -20·1%)
	Ischaemic stroke	9% (6·7–11·3%)	72·2 (52·7–93·1)	-18·8% (-26·1 to -11·9%)	7·6% (4·6–10·4%)	3·3 (2–4·6)	-25·2% (-32·3 to -18%)
	Intracerebral haemorrhage	8·8% (7·3–10·4%)	72·9 (59·1–88·2)	-29·4% (-37·3 to -22·6%)	8·4% (6·7–10·1%)	3 (2·4–3·7)	-28·2% (-36·5 to -20·8%)
Lead exposure	Stroke	4·6% (2·7–6·7%)	82 (47·9–119·1)	-38·5% (-45·6 to -31·2%)	4·6% (2·8–6·6%)	3·8 (2·3–5·5)	-30·5% (-38·7 to -20·9%)
	Ischaemic stroke	4% (2·3–6%)	32·2 (18·2–48·7)	-13·4% (-25·7 to 0·9%)	3·8% (2·2–5·8%)	1·7 (0·9–2·6)	-8·4% (-23 to 12·6%)
	Intracerebral haemorrhage	5·3% (3–7·8%)	44 (25·1–65·7)	-44% (-52·4 to -36·4%)	5·5% (3·2–8·1%)	2 (1·2–2·9)	-36·2% (-45 to -27·6%)
	Subarachnoid haemorrhage	4·2% (2·1–6·6%)	5·7 (2·8–9·3)	-67·3% (-74·9 to -50·2%)	4·6% (2·6–7·1%)	0·2 (0·1–0·3)	-65·9% (-73·1 to -45·1%)
Low birth weight	Meningitis	5·8% (5·2–6·6%)	13·6 (10·9–17·6)	-52·3% (-61·7 to -39·5%)	4·7% (4·1–5·4%)	0·2 (0·1–0·2)	-52·3% (-61·7 to -39·5%)
	Encephalitis	2·2% (1·7–2·6%)	1·4 (1·1–1·9)	-34·8% (-56·2 to 1·5%)	1·4% (1·1–1·7%)	<0·1 (<0·1–<0·1)	-34·8% (-56·2 to 1·5%)
Low birth weight and short gestation	Meningitis	6·4% (5·7–7·3%)	14·9 (11·9–19·2)	-51·4% (-61 to -38·4%)	5·1% (4·5–5·9%)	0·2 (0·1–0·2)	-51·4% (-61 to -38·4%)
	Encephalitis	2·3% (1·8–2·8%)	1·5 (1·2–2)	-34·1% (-55·6 to 2%)	1·5% (1·1–1·8%)	<0·1 (<0·1–<0·1)	-34·1% (-55·6 to 2%)
Low physical activity	Stroke	1·8% (0·3–4·7%)	31·2 (5·7–82)	-28·4% (-34·3 to -19·7%)	2·5% (0·5–6·4%)	2·1 (0·4–5·3)	-32·8% (-38·3 to -23·7%)
	Ischaemic stroke	3·9% (0·7–10·4%)	31·2 (5·7–82)	-28·4% (-34·3 to -19·7%)	4·8% (1–12·3%)	2·1 (0·4–5·3)	-32·8% (-38·3 to -23·7%)
Low temperature	Stroke	5·9% (4·4–7·5%)	103·9 (77–134·5)	-48·7% (-54·6 to -42·7%)	7·3% (5·6–9·3%)	6·2 (4·6–7·9)	-46·6% (-51·6 to -41%)
	Ischaemic stroke	6·1% (4·6–7·9%)	48·4 (36·1–63)	-46% (-52·1 to -39·6%)	8% (6–10·3%)	3·5 (2·6–4·4)	-45·8% (-51·1 to -39·8%)
	Intracerebral haemorrhage	5·8% (4·3–7·4%)	48 (34·6–61·8)	-46·9% (-54·5 to -40%)	6·6% (5·1–8·4%)	2·4 (1·8–3)	-43·7% (-50·8 to -36·9%)
	Subarachnoid haemorrhage	5·6% (4·1–7·3%)	7·6 (5·5–9·8)	-66·6% (-73·1 to -52·2%)	7·1% (5·3–9·2%)	0·3 (0·2–0·4)	-64·8% (-71·1 to -49%)
Metabolic risk factors†	Stroke	70·3% (63·8–76·5%)	1243 (1093·2–1377)	-29·8% (-35·7 to -23·9%)	67·6% (59·3–75·3%)	56·9 (48·9–64·9)	-33% (-38·6 to -27·1%)
	Ischaemic stroke	70·4% (61·2–79·5%)	562 (478·3–646·6)	-26·4% (-32·6 to -20·2%)	66·7% (54·7–79·7%)	29 (23·4–35·2)	-32·8% (-38·6 to -26·7%)
	Intracerebral haemorrhage	70·8% (63–77·4%)	589·9 (517–664·5)	-29·3% (-37·6 to -22·4%)	68·8% (59·9–76·7%)	24·8 (21·2–28)	-29·8% (-38·2 to -22·5%)
	Subarachnoid haemorrhage	66·8% (58·6–73·9%)	91·1 (76–107·3)	-47·2% (-54·9 to -29·6%)	66·2% (57·4–74·1%)	3·1 (2·6–3·6)	-51·9% (-59·5 to -32·5%)
	Neurological disorders*	5·7% (1·8–12·7%)	70·7 (20·8–179·6)	30·1% (20·9 to 45·9%)	14·4% (5·2–26·8%)	4·8 (0·8–14·5)	27·3% (18·2 to 43·6%)
	Alzheimer's disease and other dementias	20·8% (9·3–35·5%)	70·7 (20·8–179·6)	30·1% (20·9 to 45·9%)	20·6% (9·1–35·1%)	4·8 (0·8–14·5)	27·3% (18·2 to 43·6%)
Non-optimal temperature	Stroke	6·6% (5–8·5%)	116·9 (89·7–149·3)	-44·7% (-51·4 to -36·3%)	8% (6·2–10·2%)	6·7 (5·2–8·6)	-43·4% (-49·3 to -35·7%)

Table S16 Regional and national disabilities and deaths related to neurological conditions attributable to risk factors in North Africa and Middle East countries

Location<br>Risk factor (alphabetic order)	Cause	DALYs (95% UI)<br>PAF (%)<br>2019	Rate per 100,000<br>2019	Rate change (%)<br>From 1990 to 2019	Deaths (95% UI)<br>PAF (%)<br>2019	Rate per 100,000<br>2019	Rate change (%)<br>From 1990 to 2019
	Ischaemic stroke	6·6% (5·1–8·5%)	52·7 (40·3–68·6)	-42·8% (-49·6 to -34·4%)	8·5% (6·6–11%)	3·7 (2·9–4·8)	-43·1% (-49 to -35·5%)
	Intracerebral haemorrhage	6·7% (5–8·5%)	55·5 (41·2–71·2)	-42·1% (-50·4 to -30·6%)	7·4% (5·7–9·4%)	2·7 (2–3·4)	-39·8% (-48 to -29·1%)
	Subarachnoid haemorrhage	6·4% (4·8–8·2%)	8·7 (6·6–11·3)	-63% (-69·5 to -47·7%)	7·9% (6–10%)	0·4 (0·3–0·5)	-62·1% (-68·4 to -45·8%)
Other environmental risks	Stroke	4·6% (2·7–6·7%)	82 (47·9–119·1)	-38·5% (-45·6 to -31·2%)	4·6% (2·8–6·6%)	3·8 (2·3–5·5)	-30·5% (-38·7 to -20·9%)
	Ischaemic stroke	4% (2·3–6%)	32·2 (18·2–48·7)	-13·4% (-25·7 to 0·9%)	3·8% (2·2–5·8%)	1·7 (0·9–2·6)	-8·4% (-23 to 12·6%)
	Intracerebral haemorrhage	5·3% (3–7·8%)	44 (25·1–65·7)	-44% (-52·4 to -36·4%)	5·5% (3·2–8·1%)	2 (1·2–2·9)	-36·2% (-45 to -27·6%)
	Subarachnoid haemorrhage	4·2% (2·1–6·6%)	5·7 (2·8–9·3)	-67·3% (-74·9 to -50·2%)	4·6% (2·6–7·1%)	0·2 (0·1–0·3)	-65·9% (-73·1 to -45·1%)
Particulate matter pollution	Meningitis	2% (1·7–2·2%)	4·6 (3·6–6)	-53·9% (-63·3 to -41·2%)	1·6% (1·4–1·8%)	0·1 (0–0·1)	-53·9% (-63·4 to -41·2%)
	Encephalitis	0·6% (0·5–0·7%)	0·4 (0·3–0·5)	-39·8% (-60 to -6·3%)	0·4% (0·3–0·5%)	<0·1 (<0·1–<0·1)	-39·8% (-60 to -6·3%)
	Stroke	29·7% (27·2–32·4%)	525·6 (463·9–589·9)	-40·8% (-47·4 to -34·3%)	25·1% (22·7–27·7%)	21·1 (18·6–23·9)	-42·5% (-49·6 to -35·2%)
	Ischaemic stroke	25·5% (23–28·1%)	203·4 (177·8–231·6)	-28·6% (-37·8 to -19·2%)	20·9% (18·7–23·4%)	9·1 (7·9–10·4)	-33·6% (-43·3 to -23·2%)
	Intracerebral haemorrhage	33·8% (31–36·6%)	281·2 (248–314·9)	-43·2% (-50·3 to -36·4%)	29·9% (27·2–32·8%)	10·8 (9·5–12·1)	-44% (-51·5 to -37%)
	Subarachnoid haemorrhage	30% (26·8–33·1%)	41 (33·4–49·7)	-61·9% (-68·1 to -45·9%)	26·9% (23·7–30%)	1·3 (1–1·5)	-67·1% (-72·9 to -50·5%)
Secondhand smoke	Stroke	3·5% (2·6–4·4%)	61·5 (46–78·8)	-41·8% (-48 to -35·1%)	3% (2·2–3·7%)	2·5 (1·9–3·2)	-41·8% (-48·2 to -34·9%)
	Ischaemic stroke	2·9% (2·2–3·7%)	23·5 (17·3–30)	-30% (-37·7 to -22·1%)	2·5% (1·9–3·2%)	1·1 (0·8–1·4)	-32·6% (-40·5 to -24·2%)
	Intracerebral haemorrhage	3·9% (2·9–4·9%)	32·6 (23·8–42·1)	-43·4% (-50·8 to -36·3%)	3·4% (2·6–4·3%)	1·2 (0·9–1·6)	-43% (-50·4 to -35·2%)
	Subarachnoid haemorrhage	4% (3–5%)	5·4 (3·9–7·1)	-62·7% (-68·6 to -51·8%)	3·5% (2·7–4·4%)	0·2 (0·1–0·2)	-66·7% (-72·4 to -55·1%)
Short gestation	Meningitis	4·4% (3·9–5%)	10·3 (8·2–13·3)	-47·6% (-57·9 to -34·1%)	3·5% (3·1–4·1%)	0·1 (0·1–0·2)	-47·6% (-57·9 to -34·2%)
	Encephalitis	1·5% (1·2–1·8%)	1 (0·8–1·3)	-28·9% (-51·6 to 7·8%)	0·9% (0·7–1·1%)	<0·1 (<0·1–<0·1)	-28·9% (-51·6 to 7·8%)
Smoking	Stroke	17·1% (15·9–18·4%)	302·6 (270·9–337·5)	-41·6% (-47·8 to -35·4%)	13·5% (12·5–14·6%)	11·4 (10·2–12·6)	-42·9% (-49·4 to -36·5%)
	Ischaemic stroke	15·1% (14–16·2%)	120·4 (107–134·7)	-35% (-42·7 to -29·3%)	11·3% (10·3–12·2%)	4·9 (4·3–5·4)	-39·2% (-46·9 to -33%)
	Intracerebral haemorrhage	18·8% (17·4–20·4%)	156·9 (139–175·5)	-41·7% (-49·7 to -34·3%)	15·9% (14·7–17·1%)	5·7 (5·1–6·4)	-41·7% (-49·8 to -33·9%)
	Subarachnoid haemorrhage	18·6% (17–20·3%)	25·3 (22·2–29·4)	-59·9% (-67·1 to -36·8%)	16·2% (14·8–17·9%)	0·8 (0·7–0·9)	-63·2% (-70·4 to -36·8%)
	Neurological disorders*	3·6% (1·2–7·9%)	45·2 (14·1–110·9)	-14·3% (-20·7 to -7·7%)	7·4% (1·7–12·8%)	2·5 (0·3–7·7)	-15·4% (-23·4 to -5·2%)
	Alzheimer's disease and other dementias	15·1% (9·7–20·3%)	51·3 (20·5–116·2)	-14·2% (-20·2 to -8·4%)	12·6% (7·8–17·3%)	2·9 (0·6–8·1)	-15·9% (-22·4 to -8·4%)
	Parkinson's disease	-10% (-15·1–-5·2%)	-8 (-12·3–-4·1)	-20·4% (-27·9 to -12·9%)	-8·5% (-13–-4·3%)	-0·4 (-0·6–-0·2)	-21·1% (-29·3 to -12·7%)
	Multiple sclerosis	13·7% (10·4–17·2%)	1·9 (1·4–2·5)	-35·4% (-41·4 to -28·2%)	13·4% (10·1–16·9%)	<0·1 (<0·1–0·1)	-35·4% (-45·6 to -27·1%)
Tobacco	Stroke	20% (18·6–21·4%)	353·9 (316–391·5)	-41·4% (-47·4 to -35·5%)	16% (14·9–17·3%)	13·5 (12·1–14·9)	-42·6% (-49 to -36·5%)
	Ischaemic stroke	17·6% (16·4–18·7%)	140·2 (124·5–155·9)	-34·2% (-41·7 to -28·2%)	13·5% (12·4–14·6%)	5·9 (5·2–6·5)	-38·1% (-45·8 to -31·9%)
	Intracerebral haemorrhage	22·1% (20·4–23·7%)	183·9 (163·1–204·5)	-41·9% (-49·4 to -34·9%)	18·8% (17·4–20·2%)	6·8 (6–7·5)	-41·8% (-49·6 to -34·7%)
	Subarachnoid haemorrhage	21·8% (20·1–23·7%)	29·8 (26–34·2)	-60·2% (-66·8 to -40·1%)	19·1% (17·5–21%)	0·9 (0·8–1)	-63·7% (-70·3 to -40·9%)
	Neurological disorders*	3·6% (1·2–7·9%)	45·2 (14·1–110·9)	-14·3% (-20·7 to -7·7%)	7·4% (1·7–12·8%)	2·5 (0·3–7·7)	-15·4% (-23·4 to -5·2%)

Table S16 Regional and national disabilities and deaths related to neurological conditions attributable to risk factors in North Africa and Middle East countries

Location<br>Risk factor (alphabetic order)	Cause	DALYs (95% UI)<br>PAF (%)<br>2019	Rate per 100,000<br>2019	Rate change (%)<br>From 1990 to 2019	Deaths (95% UI)<br>PAF (%)<br>2019	Rate per 100,000<br>2019	Rate change (%)<br>From 1990 to 2019
	Alzheimer's disease and other dementias	15·1% (9·7–20·3%)	51·3 (20·5–116·2)	-14·2% (-20·2 to -8·4%)	12·6% (7·8–17·3%)	2·9 (0·6–8·1)	-15·9% (-22·4 to -8·4%)
	Parkinson's disease	-10% (-15·1–-5·2%)	-8 (-12·3–-4·1)	-20·4% (-27·9 to -12·9%)	-8·5% (-13–-4·3%)	-0·4 (-0·6–-0·2)	-21·1% (-29·3 to -12·7%)
	Multiple sclerosis	13·7% (10·4–17·2%)	1·9 (1·4–2·5)	-35·4% (-41·4 to -28·2%)	13·4% (10·1–16·9%)	<0·1 (<0·1–0·1)	-35·4% (-45·6 to -27·1%)
**North Africa and Middle East**							
Air pollution	Meningitis	1·6% (1·2–2·2%)	1·1 (0·8–1·6)	-78·3% (-86·3 to -65·8%)	1% (0·7–1·4%)	<0·1 (<0·1–<0·1)	-78·3% (-86·3 to -65·8%)
	Encephalitis	0·8% (0·5–1·4%)	0·4 (0·2–0·6)	-14·9% (-54·3 to 56·2%)	0·5% (0·3–0·9%)	<0·1 (<0·1–<0·1)	-14·9% (-54·3 to 56·3%)
	Stroke	29·2% (26·5–32·1%)	533·2 (450·2–622·2)	-33·7% (-42·6 to -21·4%)	24·9% (22·4–27·6%)	21·9 (18·5–25·7)	-35·4% (-44·5 to -22·1%)
	Ischaemic stroke	28·1% (25·3–31%)	332·5 (281·9–387·2)	-11·3% (-24·9 to 2·1%)	23·7% (21·2–26·3%)	14·9 (12·5–17·3)	-16·3% (-30·7 to -1·7%)
	Intracerebral haemorrhage	31·6% (28·7–34·6%)	173·3 (142·8–209·6)	-52·6% (-60·5 to -40·9%)	28·2% (25·5–31·2%)	6·1 (5·1–7·3)	-55·8% (-63·3 to -43·8%)
	Subarachnoid haemorrhage	29·1% (25·8–32·3%)	27·4 (21·4–35·1)	-56·9% (-69·5 to -31·9%)	27·1% (24·1–30·1%)	0·9 (0·7–1·1)	-60·7% (-73 to -33·3%)
Alcohol use	Stroke	0·4% (0·2–0·7%)	7·7 (3·2–12·3)	-52·9% (-69·5 to -37·4%)	0·3% (0·1–0·5%)	0·2 (0·1–0·4)	-54·7% (-78·2 to -35·6%)
	Ischaemic stroke	0% (-0·2–0·3%)	0·4 (-2·4–3·3)	-52·7% (-370 to 468·5%)	0% (-0·2–0·2%)	0 (-0·1–0·1)	1603·3% (-412·5 to 507·5%)
	Intracerebral haemorrhage	1·3% (0·8–1·9%)	7·3 (4·3–10·9)	-52·9% (-63·2 to -41·1%)	1·1% (0·6–1·6%)	0·2 (0·1–0·4)	-52·7% (-63·4 to -39·7%)
	Neurological disorders*	0·3% (0·1–0·5%)	3·4 (1·9–5·5)	-17% (-44·5 to 21·7%)	0·1% (0–0·2%)	<0·1 (<0·1–<0·1)	-22·4% (-46·9 to 8·5%)
	Idiopathic epilepsy	2·1% (1·4–3·1%)	3·4 (1·9–5·5)	-17% (-44·5 to 21·7%)	2·4% (1·6–3·4%)	<0·1 (<0·1–<0·1)	-22·4% (-46·9 to 8·5%)
All risk factors	Meningitis	7·1% (5·4–9·4%)	5 (3·5–7)	-78·5% (-85·6 to -67·8%)	4·7% (3·5–6·3%)	0·1 (0–0·1)	-78·5% (-85·6 to -67·8%)
	Encephalitis	3·8% (2·2–6·1%)	1·6 (0·9–2·6)	-15·4% (-52·1 to 49·5%)	2·5% (1·4–3·9%)	<0·1 (<0·1–<0·1)	-15·4% (-52·1 to 49·5%)
	Stroke	85·8% (82·6–89·1%)	1566·9 (1393·2–1757·1)	-25·8% (-34·1 to -14%)	84·7% (79·9–90·3%)	74·3 (65·5–84·4)	-24·9% (-33·5 to -11·6%)
	Ischaemic stroke	87·1% (82·8–92%)	1031 (912·5–1161·6)	-4·2% (-16·2 to 9%)	84·7% (78·3–92·3%)	53·3 (46·8–61)	-6·3% (-18·5 to 7·9%)
	Intracerebral haemorrhage	84·5% (81·1–87·7%)	463·3 (401–535·2)	-47·4% (-55·4 to -35·9%)	85·3% (80·8–89·6%)	18·4 (16–21·1)	-49·2% (-56·8 to -37%)
	Subarachnoid haemorrhage	77·2% (72·5–81·8%)	72·7 (60·5–89·5)	-53·1% (-66·3 to -27·2%)	81% (75·8–85·8%)	2·6 (2·1–3·2)	-55·2% (-68·9 to -24·6%)
	Neurological disorders*	11·2% (4·5–22·3%)	152·6 (56·5–371·9)	20·2% (7·5 to 39·9%)	25·9% (11·4–40·4%)	9·4 (1·8–27·9)	21·6% (6·9 to 55%)
	Alzheimer's disease and other dementias	39·9% (26·4–54·7%)	154·7 (58·8–375·1)	19% (6·7 to 37·7%)	37·9% (24·6–52·9%)	9·7 (2·1–28·4)	19·4% (5·3 to 46·6%)
	Parkinson's disease	-9·1% (-13·4–-4·8%)	-7·6 (-11·5–-4)	-19% (-31·8 to -1·7%)	-8% (-11·8–-4·2%)	-0·4 (-0·6–-0·2)	-18·9% (-33·3 to 1·1%)
	Idiopathic epilepsy	2·1% (1·4–3·1%)	3·4 (1·9–5·5)	-17% (-44·5 to 21·7%)	2·4% (1·6–3·4%)	<0·1 (<0·1–<0·1)	-22·4% (-46·9 to 8·5%)
	Multiple sclerosis	10·8% (7·8–14·5%)	2·2 (1·5–3)	-6·7% (-28·5 to 8·8%)	11·4% (8·2–16·1%)	<0·1 (<0·1–<0·1)	-8·8% (-42 to 18·7%)
Ambient particulate matter pollution	Meningitis	0·9% (0·6–1·3%)	0·6 (0·4–0·9)	-71·5% (-81·8 to -54·2%)	0·6% (0·4–0·9%)	<0·1 (<0·1–<0·1)	-71·5% (-81·8 to -54·2%)
	Encephalitis	0·7% (0·2–1·3%)	0·3 (0·1–0·5)	3·5% (-48·6 to 109·4%)	0·4% (0·2–0·8%)	<0·1 (<0·1–<0·1)	3·5% (-48·6 to 109·5%)
	Stroke	25·5% (22·5–28·5%)	466·4 (391·1–553·9)	2·8% (-14·7 to 26·1%)	22% (19·3–24·6%)	19·3 (16·1–22·8)	2·4% (-15·9 to 26·2%)
	Ischaemic stroke	25% (22·1–27·9%)	296·5 (249·2–349·1)	30·6% (8·7 to 58·7%)	21·2% (18·5–23·8%)	13·3 (11·2–15·8)	26·1% (3·5 to 55·7%)

Table S16 Regional and national disabilities and deaths related to neurological conditions attributable to risk factors in North Africa and Middle East countries

**Location**<br>Risk factor (alphabetic order)	Cause	**DALYs (95% UI)**<br>PAF (%)<br>2019	 Rate per 100,000<br>2019	 Rate change (%)<br>From 1990 to 2019	**Deaths (95% UI)**<br>PAF (%)<br>2019	 Rate per 100,000<br>2019	 Rate change (%)<br>From 1990 to 2019
	Intracerebral haemorrhage	26·8% (23·6–30%)	147 (120·3–178·4)	-23·4% (-38·7 to -3·4%)	24·2% (21·4–27·1%)	5·2 (4·3–6·4)	-26·4% (-41·3 to -6·5%)
	Subarachnoid haemorrhage	24·4% (20·8–27·7%)	23 (18·5–28·8)	-33·7% (-52·9 to 6·2%)	22·7% (19·5–25·8%)	0·7 (0·6–0·9)	-37·4% (-56·9 to 7·8%)
Behavioural risk factors‡	Meningitis	6·8% (5·2–9%)	4·8 (3·4–6·7)	-78·3% (-85·5 to -67·4%)	4·5% (3·3–6%)	0·1 (0–0·1)	-78·3% (-85·5 to -67·4%)
	Encephalitis	3·7% (2·1–5·9%)	1·5 (0·9–2·5)	-14·5% (-51·5 to 51·4%)	2·4% (1·4–3·8%)	<0·1 (<0·1–<0·1)	-14·5% (-51·5 to 51·4%)
	Stroke	34% (29·6–38·9%)	621·8 (518–746·8)	-31·1% (-39·8 to -19·7%)	30·7% (25·7–36·2%)	26·9 (22–33·1)	-30·4% (-39·5 to -17·7%)
	Ischaemic stroke	35·3% (29·2–42%)	418·1 (335–521·4)	-11% (-23·6 to 1·1%)	31·7% (25·3–38·8%)	19·9 (15·5–25·5)	-13·2% (-25·7 to -0·3%)
	Intracerebral haemorrhage	32·1% (27·9–37·1%)	176·3 (144–214·4)	-52·4% (-59·6 to -42·2%)	28·3% (24·4–33·1%)	6·1 (5–7·5)	-54·9% (-61·8 to -44·9%)
	Subarachnoid haemorrhage	29·1% (24·7–33·9%)	27·4 (21·5–36·3)	-56·5% (-68·5 to -33·2%)	26·8% (22·8–31·7%)	0·9 (0·7–1·1)	-59·8% (-71·6 to -34·3%)
	Neurological disorders*	4·2% (1·6–8·7%)	57·4 (21·1–137·7)	-9·8% (-18·9 to 4%)	8·3% (2·6–13·8%)	3·1 (0·4–9·4)	-10·7% (-23·3 to 13·7%)
	Alzheimer's disease and other dementias	15·4% (9·9–20·7%)	59·5 (23·6–138·6)	-10·8% (-19·1 to 0·9%)	13·4% (8·5–18·2%)	3·4 (0·8–9·8)	-11·7% (-21·8 to 5%)
	Parkinson's disease	-9·1% (-13·4–-4·8%)	-7·6 (-11·5–-4)	-19% (-31·8 to -1·7%)	-8% (-11·8–-4·2%)	-0·4 (-0·6–-0·2)	-18·9% (-33·3 to 1·1%)
	Idiopathic epilepsy	2·1% (1·4–3·1%)	3·4 (1·9–5·5)	-17% (-44·5 to 21·7%)	2·4% (1·6–3·4%)	<0·1 (<0·1–<0·1)	-22·4% (-46·9 to 8·5%)
	Multiple sclerosis	10·8% (7·8–14·5%)	2·2 (1·5–3)	-6·7% (-28·5 to 8·8%)	11·4% (8·2–16·1%)	<0·1 (<0·1–<0·1)	-8·8% (-42 to 18·7%)
Child and maternal malnutrition	Meningitis	6·8% (5·2–9%)	4·8 (3·4–6·7)	-78·3% (-85·5 to -67·4%)	4·5% (3·3–6%)	0·1 (0–0·1)	-78·3% (-85·5 to -67·4%)
	Encephalitis	3·7% (2·1–5·9%)	1·5 (0·9–2·5)	-14·5% (-51·5 to 51·4%)	2·4% (1·4–3·8%)	<0·1 (<0·1–<0·1)	-14·5% (-51·5 to 51·4%)
Diet high in red meat	Stroke	3·6% (1·4–5·5%)	66 (25·9–102·1)	-30·9% (-48 to -10%)	2·8% (1·1–4·2%)	2·4 (0·9–3·8)	-31·4% (-49·9 to -11·3%)
	Ischaemic stroke	3·2% (0·6–4·9%)	38·3 (6·5–61·1)	-4·6% (-18·1 to 9·4%)	2·4% (0·4–3·7%)	1·5 (0·3–2·4)	-7·8% (-21·6 to 8·2%)
	Intracerebral haemorrhage	4·3% (0·7–6·8%)	23·5 (3·7–39·4)	-49·6% (-58 to -38·1%)	3·7% (0·6–5·8%)	0·8 (0·1–1·3)	-51·2% (-59·3 to -39·1%)
	Subarachnoid haemorrhage	4·4% (0·7–7%)	4·1 (0·6–6·9)	-52·3% (-65·9 to -26·6%)	3·8% (0·6–6·1%)	0·1 (0–0·2)	-55·5% (-69·2 to -25·2%)
Diet high in sodium	Stroke	1·8% (0·3–7·2%)	33·6 (5·8–133·3)	-28·8% (-40·4 to -8·7%)	1·6% (0·3–6·2%)	1·4 (0·3–5·4)	-29·4% (-42 to -7·5%)
	Ischaemic stroke	1·7% (0·3–6·7%)	20·3 (3·8–80·5)	-5·9% (-25·7 to 18·8%)	1·4% (0·3–5·7%)	0·9 (0·2–3·6)	-8·7% (-29·3 to 16·5%)
	Intracerebral haemorrhage	2·1% (0·3–8·4%)	11·6 (1·7–46·7)	-47·4% (-57·9 to -29·6%)	1·9% (0·3–7·6%)	0·4 (0·1–1·7)	-50·1% (-59·9 to -33·2%)
	Subarachnoid haemorrhage	1·9% (0·3–7·4%)	1·8 (0·3–6·9)	-52% (-66·6 to -18·5%)	1·8% (0·3–7·2%)	0·1 (0–0·2)	-55·5% (-70·1 to -21%)
Diet low in fibre	Stroke	2% (0·5–3·6%)	36·2 (9·2–66·7)	-36·1% (-44 to -23·8%)	1·7% (0·5–3%)	1·5 (0·4–2·7)	-38·8% (-46·4 to -27·2%)
	Ischaemic stroke	1·8% (0·5–3·3%)	21·2 (5·6–39)	-18·2% (-28·1 to -4·8%)	1·5% (0·4–2·7%)	0·9 (0·3–1·7)	-23% (-32·9 to -10·1%)
	Intracerebral haemorrhage	2·4% (0·6–4·3%)	13·1 (3·1–23·9)	-50·5% (-58·7 to -37·9%)	2·1% (0·5–3·7%)	0·4 (0·1–0·8)	-55·2% (-62·1 to -44·4%)
	Subarachnoid haemorrhage	2·1% (0·5–4%)	2 (0·5–4·2)	-54·9% (-65·7 to -32·7%)	2% (0·5–3·7%)	0·1 (0–0·1)	-59·7% (-69·6 to -36%)
Diet low in fruits	Stroke	3·9% (2–5·9%)	70·5 (35·4–112·5)	-47% (-58·1 to -34·3%)	3% (1·5–4·6%)	2·6 (1·3–4·2)	-49·5% (-60·6 to -37·3%)
	Ischaemic stroke	2·9% (0·8–5·3%)	34·1 (9·6–63·6)	-23·1% (-34·1 to -9·1%)	2·2% (0·6–4·1%)	1·4 (0·4–2·6)	-28·1% (-39·5 to -14·1%)
	Intracerebral haemorrhage	5·7% (2·9–9·5%)	31·5 (15·9–54·1)	-58·1% (-64·9 to -48·3%)	4·8% (2·4–8%)	1 (0·5–1·8)	-61·9% (-68·2 to -52·4%)
	Subarachnoid haemorrhage	5·2% (2·8–8·4%)	4·9 (2·4–8·7)	-63·5% (-73·3 to -44·2%)	4·4% (2·3–7·3%)	0·1 (0·1–0·3)	-67·7% (-76·8 to -47·3%)
Diet low in vegetables	Stroke	1·7% (0·7–2·7%)	31·1 (13·4–50·9)	-51·6% (-61·6 to -36·8%)	1·4% (0·6–2·3%)	1·2 (0·5–2)	-53·2% (-63·5 to -38·7%)

Table S16 Regional and national disabilities and deaths related to neurological conditions attributable to risk factors in North Africa and Middle East countries

Location<br>Risk factor (alphabetic order)	Cause	DALYs (95% UI)<br>PAF (%)<br>2019	Rate per 100,000<br>2019	Rate change (%)<br>From 1990 to 2019	Deaths (95% UI)<br>PAF (%)<br>2019	Rate per 100,000<br>2019	Rate change (%)<br>From 1990 to 2019
	Ischaemic stroke	1·3% (0·3–2·3%)	15·4 (4–27·6)	-31·5% (-41·6 to -11·6%)	1·1% (0·3–2%)	0·7 (0·2–1·3)	-33·7% (-44·3 to -15·4%)
	Intracerebral haemorrhage	2·5% (0·7–4·8%)	13·6 (3·5–26·9)	-61·9% (-69·4 to -50·7%)	2·2% (0·6–4·3%)	0·5 (0·1–0·9)	-65·8% (-72·1 to -55·9%)
	Subarachnoid haemorrhage	2·2% (0·6–4·3%)	2·1 (0·5–4·4)	-64·8% (-73·9 to -44·4%)	2·1% (0·6–4·2%)	0·1 (0–0·1)	-68·5% (-76·5 to -48·6%)
Diet low in whole grains	Stroke	5·3% (2·6–7%)	96·7 (46·5–131·4)	-8% (-19·2 to 3·7%)	5·3% (2·9–6·8%)	4·6 (2·5–6·1)	-9·2% (-21·3 to 3·9%)
	Ischaemic stroke	8·2% (4–10·7%)	96·7 (46·5–131·4)	-8% (-19·2 to 3·7%)	7·4% (4–9·5%)	4·6 (2·5–6·1)	-9·2% (-21·3 to 3·9%)
Dietary risks	Stroke	16·7% (12·6–21·5%)	304·6 (223·3–400·8)	-33·2% (-42·5 to -21·5%)	14·6% (11–18·8%)	12·8 (9·4–16·9)	-33·4% (-42·6 to -21·5%)
	Ischaemic stroke	17·4% (12·1–22·4%)	206·1 (140·3–270·9)	-12·5% (-23·8 to -0·8%)	15% (10·7–19·2%)	9·5 (6·5–12·5)	-15·1% (-27·3 to -2·1%)
	Intracerebral haemorrhage	15·5% (10·1–21·3%)	84·9 (54·4–121)	-54·6% (-62·1 to -44·9%)	13·6% (8·8–19%)	2·9 (1·9–4·2)	-57·9% (-65·2 to -48·1%)
	Subarachnoid haemorrhage	14·3% (9·2–19·9%)	13·5 (8·3–20·3)	-58·8% (-70·3 to -36·6%)	13·1% (8·3–18·3%)	0·4 (0·2–0·6)	-62·7% (-73·3 to -40·1%)
Environmental/occupational risk factors§	Meningitis	1·6% (1·2–2·2%)	1·1 (0·8–1·6)	-78·3% (-86·3 to -65·8%)	1% (0·7–1·4%)	<0·1 (<0·1–<0·1)	-78·3% (-86·3 to -65·8%)
	Encephalitis	0·8% (0·5–1·4%)	0·4 (0·2–0·6)	-14·9% (-54·3 to 56·2%)	0·5% (0·3–0·9%)	<0·1 (<0·1–<0·1)	-14·9% (-54·3 to 56·3%)
	Stroke	37·5% (34·5–40·7%)	685 (590·6–792·8)	-33·5% (-41·7 to -22·2%)	34·7% (31·8–37·9%)	30·5 (26·1–35·2)	-32·5% (-40·8 to -19·1%)
	Ischaemic stroke	36·4% (33·4–39·7%)	430·7 (370·7–494·6)	-10·9% (-24 to 2·5%)	33·5% (30·5–36·7%)	21·1 (18·1–24·4)	-13% (-26·6 to 1·8%)
	Intracerebral haemorrhage	40% (36·9–43·3%)	219·4 (184·6–260·2)	-52·7% (-60·4 to -41·9%)	37·9% (34·9–41·3%)	8·2 (6·9–9·7)	-54·4% (-61·8 to -42·9%)
	Subarachnoid haemorrhage	37·1% (33·5–40·8%)	35 (27·8–44·8)	-58·2% (-69·6 to -33·8%)	37% (33·5–40·3%)	1·2 (0·9–1·5)	-59·6% (-71·7 to -31·9%)
High body-mass index	Stroke	33·8% (24·3–42·8%)	618 (440·1–822·7)	-6·2% (-21·8 to 16·6%)	22·8% (15·1–30·7%)	20 (13·3–28·2)	-5·1% (-21·8 to 19·4%)
	Ischaemic stroke	27·3% (18·8–36%)	323·4 (218·8–443·9)	37·1% (13·1 to 68·5%)	17·9% (11·2–25·8%)	11·2 (6·9–16·6)	35·1% (9·5 to 70·8%)
	Intracerebral haemorrhage	46% (34·5–56·7%)	252·2 (182·5–329·8)	-28·8% (-42·6 to -7·3%)	35·2% (25·2–46·3%)	7·6 (5·3–10·5)	-29·8% (-44·3 to -7·1%)
	Subarachnoid haemorrhage	45·1% (34–55·2%)	42·4 (30·6–56·2)	-38·1% (-55·5 to -5%)	35·1% (24·9–46·3%)	1·1 (0·8–1·5)	-40·6% (-59·4 to -2%)
	Neurological disorders*	5·3% (1·6–11·9%)	72·6 (21–181·2)	48·7% (28·5 to 94·4%)	12·9% (4·7–24·2%)	4·6 (0·8–13·6)	47% (23·7 to 100·5%)
	Alzheimer's disease and other dementias	18·7% (8·2–33·1%)	72·6 (21–181·2)	48·7% (28·5 to 94·4%)	18% (7·8–32%)	4·6 (0·8–13·6)	47% (23·7 to 100·5%)
High fasting plasma glucose	Stroke	27·6% (17·7–42·8%)	504·9 (318·8–793·9)	34·8% (7·9 to 64·8%)	29·1% (16·6–52·4%)	25·5 (14·8–45·2)	30·3% (2·9 to 59·5%)
	Ischaemic stroke	29·9% (15·7–53%)	353·8 (190·4–627·5)	67·7% (38·9 to 103·2%)	30% (14·5–62·3%)	18·8 (8·9–38·8)	57·7% (29·6 to 92·4%)
	Intracerebral haemorrhage	24·2% (15·6–33·9%)	132·5 (81·9–191)	-5·9% (-23·4 to 18·3%)	27·2% (16·9–40·9%)	5·9 (3·6–9)	-11·1% (-28·1 to 12·3%)
	Subarachnoid haemorrhage	19·8% (12·9–28·3%)	18·6 (11·4–27·2)	-18·1% (-41·7 to 32·6%)	25% (15·4–38·6%)	0·8 (0·5–1·3)	-22·1% (-46 to 35·4%)
	Neurological disorders*	3·5% (0·6–9·7%)	48·3 (8·7–151·5)	59·1% (47·1 to 88·7%)	8·9% (1·9–19·9%)	3·2 (0·4–11·4)	55·9% (41·5 to 94·6%)
	Alzheimer's disease and other dementias	12·4% (3–25·9%)	48·3 (8·7–151·5)	59·1% (47·1 to 88·7%)	12·3% (3–25·7%)	3·2 (0·4–11·4)	55·9% (41·5 to 94·6%)
High LDL cholesterol	Stroke	15% (9·1–24·8%)	274·7 (160·6–448·1)	-0·9% (-14 to 13·7%)	13·9% (5·5–28·2%)	12·2 (4·7–25·1)	-4·7% (-18·3 to 10·5%)
	Ischaemic stroke	23·2% (13·9–38·2%)	274·7 (160·6–448·1)	-0·9% (-14 to 13·7%)	19·4% (7·6–38·8%)	12·2 (4·7–25·1)	-4·7% (-18·3 to 10·5%)
High systolic blood pressure	Stroke	52·8% (45·7–59·4%)	965 (800·6–1143·2)	-24·9% (-34·3 to -12%)	50·1% (41·6–58·5%)	44 (35·1–53·5)	-25·2% (-35·3 to -10·7%)
	Ischaemic stroke	51·3% (41·6–60·2%)	607·8 (477·7–734·5)	-1·1% (-13·7 to 13·3%)	47·6% (37–58·6%)	30 (22·2–38·6)	-4·5% (-17·5 to 11·3%)

Table S16 Regional and national disabilities and deaths related to neurological conditions attributable to risk factors in North Africa and Middle East countries

Location<br>Risk factor (alphabetic order)	Cause	DALYs (95% UI)<br>PAF (%)<br>2019	Rate per 100,000<br>2019	Rate change (%)<br>From 1990 to 2019	Deaths (95% UI)<br>PAF (%)<br>2019	Rate per 100,000<br>2019	Rate change (%)<br>From 1990 to 2019
	Intracerebral haemorrhage	56·4% (47·4–64·3%)	309·1 (247·8–378)	-45·9% (-54·5 to -33·5%)	56·7% (46·1–66·5%)	12·2 (9·5–15·1)	-48% (-55·8 to -34·9%)
	Subarachnoid haemorrhage	51·1% (42·6–59·1%)	48·1 (37·3–61·4)	-51·5% (-65·5 to -23·8%)	54·2% (43·8–63·4%)	1·7 (1·3–2·3)	-54·4% (-68·7 to -22·2%)
High temperature	Stroke	1·6% (0·5–3·1%)	29·4 (10·2–59·3)	-21·2% (-59·9 to 33·6%)	1·7% (0·6–3·3%)	1·5 (0·5–2·9)	-16·8% (-52·5 to 47·8%)
	Ischaemic stroke	1·5% (0·5–3%)	17·9 (6·1–36·2)	11·1% (-39·8 to 103·6%)	1·7% (0·6–3·2%)	1·1 (0·4–2·1)	8·4% (-39·3 to 97·2%)
	Intracerebral haemorrhage	1·8% (0·6–3·6%)	10 (3·5–20·3)	-43·2% (-71·3 to -8·4%)	1·7% (0·6–3·4%)	0·4 (0·1–0·8)	-45·9% (-71·2 to -6·3%)
	Subarachnoid haemorrhage	1·5% (0·6–3%)	1·5 (0·5–3·1)	-58·5% (-83·3 to -17·7%)	1·5% (0·6–3%)	<0·1 (<0·1–0·1)	-55·6% (-80·6 to -13·8%)
Household air pollution from solid fuels	Meningitis	0·7% (0·4–1·1%)	0·5 (0·3–0·8)	-83·5% (-91·5 to -68·6%)	0·4% (0·2–0·7%)	<0·1 (<0·1–<0·1)	-83·5% (-91·5 to -68·6%)
	Encephalitis	0·2% (0·1–0·3%)	0·1 (0–0·2)	-51·1% (-78 to 26·9%)	0·1% (0–0·2%)	<0·1 (<0·1–<0·1)	-51·1% (-78 to 26·9%)
	Stroke	3·7% (2·5–5%)	66·8 (44·8–93·1)	-80·9% (-84·8 to -76·1%)	3% (2–4·2%)	2·6 (1·7–3·7)	-82·7% (-86 to -78·6%)
	Ischaemic stroke	3% (2·1–4·3%)	36·1 (24·1–52·9)	-75·6% (-80·2 to -69·8%)	2·5% (1·7–3·7%)	1·6 (1–2·4)	-78·2% (-82·3 to -72·7%)
	Intracerebral haemorrhage	4·8% (3·3–6·5%)	26·4 (17·6–37·1)	-84·9% (-88·3 to -80·3%)	4% (2·7–5·6%)	0·9 (0·6–1·2)	-87% (-89·9 to -83·3%)
	Subarachnoid haemorrhage	4·6% (2·5–7·1%)	4·4 (2·1–7·5)	-84·7% (-88·8 to -77·9%)	4·4% (2·2–7·1%)	0·1 (0·1–0·3)	-86·3% (-89·7 to -80·2%)
Kidney dysfunction	Stroke	11% (8·7–13·3%)	200·8 (155·6–247·9)	3·2% (-12·2 to 21·1%)	10·6% (7–13·8%)	9·3 (6·2–12·2)	3% (-15·8 to 24·8%)
	Ischaemic stroke	12·5% (9·2–15·7%)	148·5 (107·9–190)	26·7% (6 to 45·3%)	10·9% (6·2–15·1%)	6·9 (4–9·7)	23·4% (-1·5 to 45·2%)
	Intracerebral haemorrhage	9·5% (7·8–11·5%)	52·3 (41–64·9)	-32·4% (-42·9 to -15·8%)	11·2% (8·6–13·9%)	2·4 (1·8–3·1)	-29·8% (-41·2 to -11·5%)
Lead exposure	Stroke	5·2% (3·3–7·3%)	95·5 (57·8–137·7)	-40·8% (-50·5 to -30·4%)	5·3% (3·4–7·4%)	4·6 (2·9–6·7)	-33% (-44 to -20·6%)
	Ischaemic stroke	5·2% (3·2–7·4%)	61·3 (36·2–89)	-15·2% (-28·5 to -3·3%)	5·1% (3·1–7·4%)	3·2 (1·9–4·7)	-7·7% (-21·8 to 6·6%)
	Intracerebral haemorrhage	5·4% (3·2–8%)	29·9 (17·2–44·8)	-60·9% (-68·6 to -52·2%)	5·8% (3·5–8·5%)	1·3 (0·7–1·8)	-57·9% (-65·3 to -48·3%)
	Subarachnoid haemorrhage	4·6% (2·5–7·1%)	4·4 (2·1–7·3)	-64·8% (-74·1 to -46·8%)	5·6% (3·1–8·3%)	0·2 (0·1–0·3)	-61·3% (-71·9 to -37·9%)
Low birth weight	Meningitis	6·5% (4·9–8·6%)	4·5 (3·2–6·4)	-78·3% (-85·6 to -67·5%)	4·3% (3·1–5·7%)	0·1 (0–0·1)	-78·3% (-85·6 to -67·5%)
	Encephalitis	3·5% (2–5·6%)	1·4 (0·9–2·4)	-15·1% (-52·3 to 50·9%)	2·2% (1·3–3·6%)	<0·1 (<0·1–<0·1)	-15·1% (-52·3 to 50·9%)
Low birth weight and short gestation	Meningitis	6·8% (5·2–9%)	4·8 (3·4–6·7)	-78·3% (-85·5 to -67·4%)	4·5% (3·3–6%)	0·1 (0–0·1)	-78·3% (-85·5 to -67·4%)
	Encephalitis	3·7% (2·1–5·9%)	1·5 (0·9–2·5)	-14·5% (-51·5 to 51·4%)	2·4% (1·4–3·8%)	<0·1 (<0·1–<0·1)	-14·5% (-51·5 to 51·4%)
Low physical activity	Stroke	5% (1·1–11·2%)	91·7 (18·8–203·4)	1·2% (-11·8 to 16%)	6·4% (1·5–13·2%)	5·6 (1·3–11·6)	-3·2% (-15·8 to 12·7%)
	Ischaemic stroke	7·7% (1·7–17·2%)	91·7 (18·8–203·4)	1·2% (-11·8 to 16%)	8·9% (2·1–18·3%)	5·6 (1·3–11·6)	-3·2% (-15·8 to 12·7%)
Low temperature	Stroke	5·5% (3·5–7·7%)	100·9 (62–140·7)	-34·4% (-43·8 to -23·6%)	6·7% (4·5–9·1%)	5·9 (3·8–8)	-26·5% (-36·4 to -12·8%)
	Ischaemic stroke	5·3% (3·4–7·5%)	63·1 (39·4–87·8)	-13·4% (-28·1 to 0·3%)	6·7% (4·5–9·1%)	4·2 (2·7–5·7)	-9·9% (-24 to 5·6%)
	Intracerebral haemorrhage	5·9% (3·6–8·3%)	32·1 (19·1–45·9)	-51·5% (-59·1 to -41·7%)	6·7% (4·4–9%)	1·4 (0·9–2)	-48·8% (-56·7 to -37·3%)
	Subarachnoid haemorrhage	6% (4–8·3%)	5·6 (3·5–8)	-61·6% (-72 to -35%)	7·2% (5·1–9·4%)	0·2 (0·2–0·3)	-55·9% (-70 to -22·2%)
Metabolic risk factors†	Stroke	73·9% (67·6–80·1%)	1349·7 (1156–1552·3)	-19·6% (-29·1 to -6·3%)	71·4% (62·5–81·2%)	62·6 (52·7–74·3)	-19·2% (-29·3 to -4·8%)
	Ischaemic stroke	75·1% (66·7–84·3%)	888·8 (753·9–1040·5)	2·8% (-11·2 to 18%)	70·9% (59·5–84·6%)	44·6 (36·3–54·7)	0·4% (-14 to 17·5%)
	Intracerebral haemorrhage	72·9% (66–78·6%)	399·9 (339·2–470·1)	-42·6% (-51·5 to -29·4%)	73·4% (65·4–80·7%)	15·8 (13·2–18·6)	-44·6% (-53·2 to -30·9%)
	Subarachnoid haemorrhage	64·7% (57·3–71·4%)	60·9 (49–76·6)	-48·4% (-63·2 to -19·4%)	67·6% (58·8–75·7%)	2·1 (1·7–2·7)	-51·4% (-66·6 to -17·8%)

Table S16 Regional and national disabilities and deaths related to neurological conditions attributable to risk factors in North Africa and Middle East countries

Location<br>Risk factor (alphabetic order)	Cause	DALYs (95% UI)<br>PAF (%)<br>2019	Rate per 100,000<br>2019	Rate change (%)<br>From 1990 to 2019	Deaths (95% UI)<br>PAF (%)<br>2019	Rate per 100,000<br>2019	Rate change (%)<br>From 1990 to 2019
	Neurological disorders*	8·2% (2·8–17·4%)	111·7 (34·2–279·7)	48·4% (31·4 to 80·4%)	20·2% (8·1–34·5%)	7·2 (1·3–21·7)	46·4% (27·5 to 85·8%)
	Alzheimer's disease and other dementias	28·8% (14·7–45·8%)	111·7 (34·2–279·7)	48·4% (31·4 to 80·4%)	28·1% (14·2–44·9%)	7·2 (1·3–21·7)	46·4% (27·5 to 85·8%)
Non-optimal temperature	Stroke	7·1% (5–9·1%)	129·1 (90–168·5)	-31·9% (-44·4 to -17·3%)	8·3% (6·1–10·5%)	7·3 (5·2–9·3)	-24·8% (-38·2 to -8%)
	Ischaemic stroke	6·8% (4·8–8·8%)	80·3 (56·2–104·7)	-9·2% (-28 to 9·5%)	8·3% (6·1–10·5%)	5·2 (3·8–6·7)	-6·8% (-24·1 to 12·1%)
	Intracerebral haemorrhage	7·6% (5·3–9·8%)	41·7 (28·7–55·6)	-49·8% (-59 to -36·9%)	8·4% (6–10·5%)	1·8 (1·3–2·3)	-48·2% (-57·5 to -33·6%)
	Subarachnoid haemorrhage	7·5% (5·4–9·6%)	7 (4·9–9·7)	-61% (-71·5 to -33·3%)	8·6% (6·5–10·7%)	0·3 (0·2–0·4)	-55·9% (-69·7 to -21·7%)
Other environmental risks	Stroke	5·2% (3·3–7·3%)	95·5 (57·8–137·7)	-40·8% (-50·5 to -30·4%)	5·3% (3·4–7·4%)	4·6 (2·9–6·7)	-33% (-44 to -20·6%)
	Ischaemic stroke	5·2% (3·2–7·4%)	61·3 (36·2–89)	-15·2% (-28·5 to -3·3%)	5·1% (3·1–7·4%)	3·2 (1·9–4·7)	-7·7% (-21·8 to 6·6%)
	Intracerebral haemorrhage	5·4% (3·2–8%)	29·9 (17·2–44·8)	-60·9% (-68·6 to -52·2%)	5·8% (3·5–8·5%)	1·3 (0·7–1·8)	-57·9% (-65·3 to -48·3%)
	Subarachnoid haemorrhage	4·6% (2·5–7·1%)	4·4 (2·1–7·3)	-64·8% (-74·1 to -46·8%)	5·6% (3·1–8·3%)	0·2 (0·1–0·3)	-61·3% (-71·9 to -37·9%)
Particulate matter pollution	Meningitis	1·6% (1·2–2·2%)	1·1 (0·8–1·6)	-78·3% (-86·3 to -65·8%)	1% (0·7–1·4%)	<0·1 (<0·1–<0·1)	-78·3% (-86·3 to -65·8%)
	Encephalitis	0·8% (0·5–1·4%)	0·4 (0·2–0·6)	-14·9% (-54·3 to 56·2%)	0·5% (0·3–0·9%)	<0·1 (<0·1–<0·1)	-14·9% (-54·3 to 56·3%)
	Stroke	29·2% (26·5–32·1%)	533·2 (450·2–622·2)	-33·7% (-42·6 to -21·4%)	24·9% (22·4–27·6%)	21·9 (18·5–25·7)	-35·4% (-44·5 to -22·1%)
	Ischaemic stroke	28·1% (25·3–31%)	332·5 (281·9–387·2)	-11·3% (-24·9 to 2·1%)	23·7% (21·2–26·3%)	14·9 (12·5–17·3)	-16·3% (-30·7 to -1·7%)
	Intracerebral haemorrhage	31·6% (28·7–34·6%)	173·3 (142·8–209·6)	-52·6% (-60·5 to -40·9%)	28·2% (25·5–31·2%)	6·1 (5·1–7·3)	-55·8% (-63·3 to -43·8%)
	Subarachnoid haemorrhage	29·1% (25·8–32·3%)	27·4 (21·4–35·1)	-56·9% (-69·5 to -31·9%)	27·1% (24·1–30·1%)	0·9 (0·7–1·1)	-60·7% (-73 to -33·3%)
Secondhand smoke	Stroke	3·9% (3–4·9%)	71·9 (53·5–91·9)	-35·7% (-43·7 to -24·8%)	3·4% (2·5–4·2%)	3 (2·2–3·8)	-35·7% (-43·9 to -24·5%)
	Ischaemic stroke	3·6% (2·7–4·5%)	42·9 (31·6–54·7)	-13·2% (-26 to -0·1%)	3·1% (2·3–4%)	2 (1·5–2·5)	-17·3% (-30 to -3·6%)
	Intracerebral haemorrhage	4·5% (3·4–5·7%)	24·9 (18·1–32·3)	-52·6% (-60·2 to -41·9%)	4% (3–5%)	0·9 (0·6–1·1)	-54·5% (-61·4 to -43·7%)
	Subarachnoid haemorrhage	4·4% (3·3–5·6%)	4·1 (2·9–5·6)	-58·5% (-70·5 to -35·3%)	4% (3·1–5%)	0·1 (0·1–0·2)	-60·6% (-72·7 to -35·4%)
Short gestation	Meningitis	4·8% (3·6–6·3%)	3·4 (2·4–4·7)	-75·3% (-83·4 to -62·9%)	3·2% (2·3–4·3%)	<0·1 (<0·1–0·1)	-75·3% (-83·4 to -62·9%)
	Encephalitis	2·7% (1·5–4·4%)	1·1 (0·6–1·8)	-2·9% (-45·9 to 69·7%)	1·7% (1–2·8%)	<0·1 (<0·1–<0·1)	-2·9% (-45·9 to 69·7%)
Smoking	Stroke	13·5% (12·5–14·5%)	246 (214·2–280·1)	-34·9% (-44·3 to -23·8%)	10·3% (9·4–11·2%)	9 (7·8–10·3)	-35·8% (-45·8 to -23·9%)
	Ischaemic stroke	12·4% (11·5–13·4%)	147 (128·6–166·4)	-16% (-32·2 to -2·9%)	9·1% (8·3–10%)	5·7 (5–6·5)	-18·8% (-36·4 to -4·5%)
	Intracerebral haemorrhage	15·6% (14·3–16·9%)	85·5 (72·4–100)	-50·7% (-59·1 to -40·5%)	13·3% (12·1–14·5%)	2·9 (2·4–3·4)	-52·5% (-60·6 to -42%)
	Subarachnoid haemorrhage	14·2% (12·7–16%)	13·4 (11·2–17·2)	-54·1% (-67·6 to -26·6%)	12·8% (11·5–14·6%)	0·4 (0·3–0·5)	-56·8% (-70·4 to -27·7%)
	Neurological disorders*	3·9% (1·4–8·5%)	54 (17·8–133·9)	-9·3% (-18·6 to 5·1%)	8·2% (2·4–13·8%)	3·1 (0·4–9·4)	-10·5% (-23·3 to 13·2%)
	Alzheimer's disease and other dementias	15·4% (9·9–20·7%)	59·5 (23·6–138·6)	-10·8% (-19·1 to 0·9%)	13·4% (8·5–18·2%)	3·4 (0·8–9·8)	-11·7% (-21·8 to 5%)
	Parkinson's disease	-9·1% (-13·4–-4·8%)	-7·6 (-11·5–-4)	-19% (-31·8 to -1·7%)	-8% (-11·8–-4·2%)	-0·4 (-0·6–-0·2)	-18·9% (-33·3 to 1·1%)
	Multiple sclerosis	10·8% (7·8–14·5%)	2·2 (1·5–3)	-6·7% (-28·5 to 8·8%)	11·4% (8·2–16·1%)	<0·1 (<0·1–<0·1)	-8·8% (-42 to 18·7%)
Tobacco	Stroke	16·9% (15·6–18·1%)	307·8 (267·1–351)	-34·8% (-44 to -23·9%)	13·2% (12·1–14·4%)	11·6 (10·1–13·2)	-35·6% (-45·1 to -23·9%)

Table S16 Regional and national disabilities and deaths related to neurological conditions attributable to risk factors in North Africa and Middle East countries

Location<br>Risk factor (alphabetic order)	Cause	DALYs (95% UI)<br>PAF (%)<br>2019	Rate per 100,000<br>2019	Rate change (%)<br>From 1990 to 2019	Deaths (95% UI)<br>PAF (%)<br>2019	Rate per 100,000<br>2019	Rate change (%)<br>From 1990 to 2019
	Ischaemic stroke	15·6% (14·4–16·8%)	184·4 (161–210)	-15·1% (-31·8 to -2·3%)	11·9% (10·8–13·1%)	7·5 (6·4–8·6)	-18·2% (-35·1 to -4·5%)
	Intracerebral haemorrhage	19·4% (17·9–21%)	106·5 (90·3–125·1)	-51% (-58·9 to -40·7%)	16·7% (15·3–18·2%)	3·6 (3·1–4·2)	-52·8% (-60·5 to -42·6%)
	Subarachnoid haemorrhage	18% (16·1–20·1%)	16·9 (14·1–21·4)	-55% (-67·9 to -29·2%)	16·3% (14·7–18·1%)	0·5 (0·4–0·7)	-57·7% (-70·5 to -29·2%)
	Neurological disorders*	3·9% (1·4–8·5%)	54 (17·8–133·9)	-9·3% (-18·6 to 5·1%)	8·2% (2·4–13·8%)	3·1 (0·4–9·4)	-10·5% (-23·3 to 13·2%)
	Alzheimer's disease and other dementias	15·4% (9·9–20·7%)	59·5 (23·6–138·6)	-10·8% (-19·1 to 0·9%)	13·4% (8·5–18·2%)	3·4 (0·8–9·8)	-11·7% (-21·8 to 5%)
	Parkinson's disease	-9·1% (-13·4–-4·8%)	-7·6 (-11·5–-4)	-19% (-31·8 to -1·7%)	-8% (-11·8–-4·2%)	-0·4 (-0·6–-0·2)	-18·9% (-33·3 to 1·1%)
	Multiple sclerosis	10·8% (7·8–14·5%)	2·2 (1·5–3)	-6·7% (-28·5 to 8·8%)	11·4% (8·2–16·1%)	<0·1 (<0·1–<0·1)	-8·8% (-42 to 18·7%)
**Algeria**							
Air pollution	Meningitis	0·8% (0·3–1·5%)	0·3 (0·1–0·6)	-78% (-94 to -3·9%)	0·4% (0·1–0·8%)	<0·1 (<0·1–<0·1)	-78% (-94 to -3·9%)
	Encephalitis	0·4% (0·1–1%)	0·1 (0–0·2)	6% (-74·1 to 359·5%)	0·3% (0·1–0·6%)	<0·1 (<0·1–<0·1)	6% (-74·1 to 359·5%)
	Stroke	23·4% (17–29·3%)	410·3 (278·6–551·2)	-44·6% (-56·3 to -31%)	19·2% (13·7–24·5%)	19·5 (12·9–26·8)	-44·9% (-56·7 to -30·6%)
	Ischaemic stroke	22·2% (16–28·2%)	283·8 (194–385·1)	-22·1% (-37·7 to -2·7%)	18·4% (13–23·5%)	14·8 (9·7–20·2)	-27·5% (-43·2 to -8·3%)
	Intracerebral haemorrhage	26·2% (19·3–32·5%)	105·8 (72·3–150·1)	-67·1% (-75·5 to -56·2%)	22·1% (16–27·9%)	4·1 (2·7–5·8)	-68·9% (-76·7 to -58·7%)
	Subarachnoid haemorrhage	27% (19·9–33·8%)	20·7 (13·4–29·5)	-62·1% (-73·7 to -39·9%)	23·5% (17–29·5%)	0·7 (0·4–1)	-64·4% (-76·1 to -42·1%)
Alcohol use	Stroke	0·4% (0–0·7%)	6·7 (0·4–13·2)	-5·7% (-163·2 to 363·6%)	0·2% (-0·1–0·4%)	0·2 (-0·1–0·4)	-5·8% (-398·5 to 832·9%)
	Ischaemic stroke	0% (-0·3–0·3%)	-0·1 (-4·1–4·1)	-97·1% (-765·3 to 811·9%)	-0·1% (-0·3–0·2%)	-0·1 (-0·3–0·1)	-59·3% (-493·2 to 176·6%)
	Intracerebral haemorrhage	1·7% (0·8–2·7%)	6·8 (3·1–11·3)	-27·6% (-55·9 to 46·2%)	1·2% (0·5–1·9%)	0·2 (0·1–0·4)	-31·7% (-61·4 to 43·5%)
	Neurological disorders*	0·3% (0·1–0·6%)	3·6 (1·5–6·7)	25·7% (-45 to 174·2%)	0·1% (0–0·2%)	<0·1 (<0·1–<0·1)	6·7% (-31·7 to 86·5%)
	Idiopathic epilepsy	2·5% (1·4–3·6%)	3·6 (1·5–6·7)	25·7% (-45 to 174·2%)	2·7% (1·6–4%)	<0·1 (<0·1–<0·1)	6·7% (-31·7 to 86·5%)
All risk factors	Meningitis	4·3% (2·6–6·6%)	1·7 (1–2·9)	-81·8% (-91 to -62·4%)	2·2% (1·3–3·6%)	<0·1 (<0·1–<0·1)	-81·8% (-91 to -62·4%)
	Encephalitis	2·5% (1·2–4·5%)	0·5 (0·2–0·8)	-12·7% (-64·2 to 112·4%)	1·5% (0·7–2·7%)	<0·1 (<0·1–<0·1)	-12·7% (-64·2 to 112·4%)
	Stroke	85·7% (81·2–90·5%)	1503·9 (1242·7–1802·3)	-42·7% (-54·3 to -29·1%)	83·6% (77–91·2%)	84·8 (68·6–104·3)	-42% (-54 to -28%)
	Ischaemic stroke	86% (80·3–92·4%)	1096·3 (899·9–1331·5)	-22·4% (-37·7 to -4·6%)	83·4% (75·3–92·9%)	66·9 (53·2–83·9)	-25·9% (-41·6 to -7·1%)
	Intracerebral haemorrhage	85·3% (81·1–89%)	344·4 (270·1–433·7)	-67·1% (-75·1 to -56·3%)	84·3% (78–89·8%)	15·6 (11·9–20·1)	-68·5% (-76·2 to -58·5%)
	Subarachnoid haemorrhage	82·2% (77·6–86·3%)	63·2 (46·9–83·9)	-62·2% (-73·8 to -41%)	82·5% (76·2–88%)	2·4 (1·7–3·1)	-63·7% (-75·6 to -42·5%)
	Neurological disorders*	12·8% (5·1–25·4%)	172·6 (63·3–427·5)	16·1% (-2·1 to 40·5%)	29·9% (14·2–45·6%)	11·1 (2·2–33·6)	15% (-5·5 to 57·3%)
	Alzheimer's disease and other dementias	43·4% (29·2–58·9%)	174·4 (65·8–428·7)	12·1% (-6 to 34·5%)	42·6% (28·5–57·8%)	11·5 (2·5–34·2)	10·9% (-10·5 to 37·1%)
	Parkinson's disease	-9·1% (-13·6–-4·9%)	-7·2 (-11·5–-3·7)	-38% (-55·3 to -14·6%)	-8·7% (-13–-4·6%)	-0·5 (-0·8–-0·2)	-39·9% (-57·7 to -14·3%)
	Idiopathic epilepsy	2·5% (1·4–3·6%)	3·6 (1·5–6·7)	25·7% (-45 to 174·2%)	2·7% (1·6–4%)	<0·1 (<0·1–<0·1)	6·7% (-31·7 to 86·5%)
	Multiple sclerosis	9·3% (6·6–12·1%)	1·9 (1·2–2·6)	-1·8% (-30·6 to 27·5%)	10·5% (7·3–14·1%)	<0·1 (<0·1–<0·1)	-14·5% (-53·2 to 30·4%)
Ambient particulate matter pollution	Meningitis	0·8% (0·3–1·5%)	0·3 (0·1–0·6)	-70·5% (-92 to 35·1%)	0·4% (0·1–0·8%)	<0·1 (<0·1–<0·1)	-70·5% (-92 to 35·1%)

Table S16 Regional and national disabilities and deaths related to neurological conditions attributable to risk factors in North Africa and Middle East countries

Location<br>Risk factor (alphabetic order)	Cause	DALYs (95% UI)<br>PAF (%)<br>2019	Rate per 100,000<br>2019	Rate change (%)<br>From 1990 to 2019	Deaths (95% UI)<br>PAF (%)<br>2019	Rate per 100,000<br>2019	Rate change (%)<br>From 1990 to 2019
	Encephalitis	0·4% (0·1–1%)	0·1 (0–0·2)	42% (-65·9 to 545·2%)	0·3% (0·1–0·6%)	<0·1 (<0·1–<0·1)	42% (-65·9 to 545·6%)
	Stroke	23·3% (16·9–29·3%)	409 (278–550·1)	-29·3% (-46 to -3·6%)	19·2% (13·6–24·4%)	19·5 (12·8–26·7)	-29·8% (-46·9 to -4·9%)
	Ischaemic stroke	22·2% (16–28·1%)	282·9 (192·9–384·2)	-0·7% (-24·5 to 35%)	18·3% (13–23·5%)	14·7 (9·7–20·2)	-7·8% (-31·1 to 24·2%)
	Intracerebral haemorrhage	26·1% (19·3–32·4%)	105·5 (72–149·5)	-58% (-70 to -40·2%)	22·1% (16–27·8%)	4·1 (2·7–5·8)	-60·3% (-71·5 to -43·4%)
	Subarachnoid haemorrhage	26·9% (19·9–33·7%)	20·7 (13·4–29·5)	-51·3% (-67·3 to -21%)	23·4% (16·9–29·4%)	0·7 (0·4–0·9)	-54·5% (-70·2 to -24·6%)
Behavioural risk factors‡	Meningitis	4% (2·4–6·3%)	1·6 (0·9–2·7)	-81·7% (-91·1 to -62·8%)	2·1% (1·2–3·4%)	<0·1 (<0·1–<0·1)	-81·7% (-91·1 to -62·8%)
	Encephalitis	2·3% (1·1–4·3%)	0·4 (0·2–0·8)	-12·2% (-64·5 to 112·1%)	1·4% (0·7–2·6%)	<0·1 (<0·1–<0·1)	-12·2% (-64·5 to 112·1%)
	Stroke	34·1% (28·8–39·9%)	598·2 (464·3–757·4)	-49·4% (-60 to -35·9%)	31·2% (25·1–37·8%)	31·6 (23·8–41)	-48·2% (-59·3 to -34·9%)
	Ischaemic stroke	35·2% (28·6–42·6%)	449·5 (335·6–583·6)	-30·4% (-44·7 to -13·4%)	32·3% (25·1–40·4%)	25·9 (18·6–34·4)	-33·6% (-48 to -16·6%)
	Intracerebral haemorrhage	31% (25·8–36·9%)	125·3 (91·2–166·2)	-72·9% (-80·3 to -63·4%)	26·8% (22–32·4%)	5 (3·6–6·7)	-74·5% (-81·2 to -65·6%)
	Subarachnoid haemorrhage	30·4% (24·8–36·5%)	23·4 (16–33·3)	-68·6% (-79·1 to -50·1%)	27·4% (22·2–33·3%)	0·8 (0·5–1·1)	-70·4% (-80·6 to -51·4%)
	Neurological disorders*	5·3% (2–10·8%)	71·7 (25·4–174·2)	-15·2% (-30·6 to 5·6%)	11·8% (4·3–18·9%)	4·5 (0·6–13·9)	-18·3% (-36·8 to 18·9%)
	Alzheimer's disease and other dementias	18·3% (12–24·6%)	73·5 (28·4–173·9)	-19·7% (-34·5 to -3·1%)	18·2% (11·7–24·7%)	4·9 (1·1–14·2)	-21·2% (-38·2 to 0·7%)
	Parkinson's disease	-9·1% (-13·6–-4·9%)	-7·2 (-11·5–-3·7)	-38% (-55·3 to -14·6%)	-8·7% (-13–-4·6%)	-0·5 (-0·8–-0·2)	-39·9% (-57·7 to -14·3%)
	Idiopathic epilepsy	2·5% (1·4–3·6%)	3·6 (1·5–6·7)	25·7% (-45 to 174·2%)	2·7% (1·6–4%)	<0·1 (<0·1–<0·1)	6·7% (-31·7 to 86·5%)
	Multiple sclerosis	9·3% (6·6–12·1%)	1·9 (1·2–2·6)	-1·8% (-30·6 to 27·5%)	10·5% (7·3–14·1%)	<0·1 (<0·1–<0·1)	-14·5% (-53·2 to 30·4%)
Child and maternal malnutrition	Meningitis	4% (2·4–6·3%)	1·6 (0·9–2·7)	-81·7% (-91·1 to -62·8%)	2·1% (1·2–3·4%)	<0·1 (<0·1–<0·1)	-81·7% (-91·1 to -62·8%)
	Encephalitis	2·3% (1·1–4·3%)	0·4 (0·2–0·8)	-12·2% (-64·5 to 112·1%)	1·4% (0·7–2·6%)	<0·1 (<0·1–<0·1)	-12·2% (-64·5 to 112·1%)
Diet high in red meat	Stroke	3·4% (1·2–5·2%)	59 (20·7–94·5)	-36·7% (-58·3 to -4·1%)	2·5% (0·8–3·9%)	2·5 (0·8–4·2)	-36·8% (-59·1 to -6·5%)
	Ischaemic stroke	3% (0·4–4·7%)	37·9 (5·6–65·2)	-4·9% (-24·9 to 25·9%)	2·2% (0·4–3·6%)	1·8 (0·3–3·1)	-11·2% (-31 to 21·1%)
	Intracerebral haemorrhage	4·3% (0·6–7%)	17·5 (2·4–31·4)	-61·4% (-71·9 to -44·9%)	3·5% (0·5–5·7%)	0·6 (0·1–1·2)	-63% (-73 to -47·4%)
	Subarachnoid haemorrhage	4·6% (0·6–7·5%)	3·5 (0·4–6·4)	-55·6% (-69·2 to -24·9%)	3·7% (0·5–6·1%)	0·1 (0–0·2)	-58·1% (-71·9 to -29·9%)
Diet high in sodium	Stroke	1·7% (0·3–6·8%)	30·4 (4·5–118·2)	-46·2% (-66·4 to -5·1%)	1·4% (0·3–5·6%)	1·4 (0·3–5·5)	-46·7% (-66·9 to 2·5%)
	Ischaemic stroke	1·6% (0·3–6·3%)	20·4 (3·2–79·2)	-23·2% (-54·1 to 35·9%)	1·3% (0·3–5%)	1 (0·2–4·1)	-28% (-57·7 to 35·9%)
	Intracerebral haemorrhage	2·1% (0·3–8·4%)	8·4 (1–34·7)	-67·4% (-81·2 to -40·2%)	1·8% (0·3–7·1%)	0·3 (0–1·4)	-69·2% (-81·8 to -41·8%)
	Subarachnoid haemorrhage	2·1% (0·2–8·2%)	1·6 (0·2–6·4)	-61·4% (-79·9 to -19·8%)	1·9% (0·3–7·6%)	0·1 (0–0·2)	-63·6% (-80·6 to -20·8%)
Diet low in fibre	Stroke	1·5% (0·4–3·2%)	26 (6–57)	-78·9% (-85·5 to -69·6%)	1·4% (0·3–2·9%)	1·4 (0·4–3)	-76·7% (-84·4 to -65·8%)
	Ischaemic stroke	1·4% (0·3–3%)	18·1 (4·3–38·9)	-69·5% (-79·4 to -56·2%)	1·3% (0·3–2·8%)	1·1 (0·3–2·3)	-68·2% (-79·3 to -52·9%)
	Intracerebral haemorrhage	1·6% (0·4–3·6%)	6·6 (1·4–14·9)	-88% (-92 to -82·3%)	1·5% (0·4–3·3%)	0·3 (0·1–0·6)	-87·7% (-91·9 to -81·8%)
	Subarachnoid haemorrhage	1·7% (0·4–3·8%)	1·3 (0·3–3·1)	-85·9% (-91·6 to -76·5%)	1·6% (0·4–3·3%)	<0·1 (<0·1–0·1)	-86·2% (-91·7 to -76·1%)
Diet low in fruits	Stroke	3·9% (1·7–6·3%)	68·4 (29·3–117·9)	-67·3% (-80·6 to -50·8%)	3·2% (1·3–5·4%)	3·2 (1·3–5·7)	-65·4% (-79·4 to -49·4%)
	Ischaemic stroke	3·1% (0·7–6%)	39·9 (8·1–81·1)	-49·3% (-75·5 to -23·2%)	2·7% (0·6–5·1%)	2·1 (0·5–4·3)	-48·9% (-72·3 to -26%)

Table S16 Regional and national disabilities and deaths related to neurological conditions attributable to risk factors in North Africa and Middle East countries

Location<br>Risk factor (alphabetic order)	Cause	DALYs (95% UI)<br>PAF (%)<br>2019	Rate per 100,000<br>2019	Rate change (%)<br>From 1990 to 2019	Deaths (95% UI)<br>PAF (%)<br>2019	Rate per 100,000<br>2019	Rate change (%)<br>From 1990 to 2019
	Intracerebral haemorrhage	5·9% (2·7–10·4%)	23·7 (10·5–42·6)	-78·7% (-88·2 to -67·8%)	5·1% (2·4–9·3%)	1 (0·4–1·7)	-79% (-88·1 to -68·8%)
	Subarachnoid haemorrhage	6·2% (2·8–11%)	4·8 (2·1–8·8)	-75% (-87·1 to -56·7%)	5·3% (2·5–9·3%)	0·2 (0·1–0·3)	-76·4% (-87·8 to -58·3%)
Diet low in vegetables	Stroke	2·1% (0·6–3·7%)	36·1 (10·5–67·8)	-70·6% (-86·5 to -47·4%)	1·8% (0·6–3·2%)	1·8 (0·6–3·4)	-68·4% (-84·5 to -48%)
	Ischaemic stroke	1·8% (0·2–3·5%)	23·5 (3–46·3)	-48·3% (-75·5 to -18·6%)	1·6% (0·2–3%)	1·3 (0·2–2·6)	-49·1% (-74·1 to -23·2%)
	Intracerebral haemorrhage	2·6% (0·2–6·9%)	10·6 (0·9–28·1)	-84·1% (-94·4 to -68·6%)	2·5% (0·2–6·3%)	0·5 (0–1·2)	-83·7% (-93·3 to -70%)
	Subarachnoid haemorrhage	2·6% (0·2–7%)	2 (0·2–5·5)	-81·7% (-94·4 to -60·5%)	2·5% (0·2–6·4%)	0·1 (0–0·2)	-81·6% (-93·5 to -61·5%)
Diet low in whole grains	Stroke	5·8% (2·8–7·7%)	101·6 (49–145·7)	-24·4% (-40·6 to -6·4%)	5·5% (2·8–7·2%)	5·6 (2·8–7·8)	-29·3% (-45·5 to -11%)
	Ischaemic stroke	8% (3·9–10·5%)	101·6 (49–145·7)	-24·4% (-40·6 to -6·4%)	6·9% (3·6–9·1%)	5·6 (2·8–7·8)	-29·3% (-45·5 to -11%)
Dietary risks	Stroke	16·9% (12·5–21·7%)	295·8 (206·7–406·8)	-55·4% (-66·2 to -42%)	14·8% (11–19%)	15 (10·4–20·5)	-54·3% (-65·6 to -41·5%)
	Ischaemic stroke	17·4% (11·9–22·5%)	221·5 (144·2–303·2)	-35·8% (-50·6 to -18·9%)	15·1% (10·6–19·6%)	12·1 (8–16·6)	-38·8% (-53·7 to -21·2%)
	Intracerebral haemorrhage	15·4% (9·3–22%)	62·1 (35·5–93·6)	-77·2% (-84·6 to -67·4%)	13·7% (8·5–19·8%)	2·5 (1·4–3·8)	-78% (-84·7 to -69·1%)
	Subarachnoid haemorrhage	15·9% (9·7–23%)	12·3 (6·9–19·4)	-73·4% (-83·7 to -57·5%)	14·1% (8·6–20·5%)	0·4 (0·2–0·6)	-74·9% (-84·8 to -58·8%)
Environmental/occupational risk factors§	Meningitis	0·8% (0·3–1·5%)	0·3 (0·1–0·6)	-78% (-94 to -3·9%)	0·4% (0·1–0·8%)	<0·1 (<0·1–<0·1)	-78% (-94 to -3·9%)
	Encephalitis	0·4% (0·1–1%)	0·1 (0–0·2)	6% (-74·1 to 359·5%)	0·3% (0·1–0·6%)	<0·1 (<0·1–<0·1)	6% (-74·1 to 359·5%)
	Stroke	30·8% (24·7–36·6%)	540·9 (400·9–699·4)	-45·5% (-56·7 to -31·7%)	28% (22·5–33·6%)	28·4 (21·1–37)	-44% (-55·9 to -30%)
	Ischaemic stroke	29·7% (23·8–35·5%)	378·6 (280·5–494·3)	-23·4% (-39·1 to -4·1%)	27·2% (21·8–32·7%)	21·8 (16·1–28·5)	-26·8% (-42·7 to -7·3%)
	Intracerebral haemorrhage	33·7% (27·3–40·1%)	136·2 (98·5–180)	-68% (-76 to -57·3%)	30·9% (24·9–36·8%)	5·7 (4·2–7·6)	-69% (-76·8 to -58·8%)
	Subarachnoid haemorrhage	33·9% (27·5–40·6%)	26·1 (17·9–35·8)	-63·7% (-74·9 to -41·9%)	32% (25·9–38·1%)	0·9 (0·6–1·3)	-64·6% (-76·3 to -42·6%)
High body-mass index	Stroke	30·8% (21·7–39·8%)	541·6 (362·1–750·9)	-28·6% (-45·9 to -4·3%)	18·2% (11·4–26·2%)	18·5 (11–28)	-25·7% (-45·1 to 2·9%)
	Ischaemic stroke	24·8% (16·5–33·7%)	316·1 (194·7–460·9)	22% (-7·2 to 66·1%)	14·7% (8·4–22·7%)	11·8 (6·4–19·1)	20·1% (-13 to 72·7%)
	Intracerebral haemorrhage	46·3% (34·3–57·4%)	186·9 (127·5–257·3)	-55·5% (-67·8 to -36·9%)	30·9% (20·6–42·1%)	5·7 (3·6–8·6)	-55·7% (-68·3 to -36·8%)
	Subarachnoid haemorrhage	50·2% (38–61·4%)	38·6 (25·8–56·1)	-51·5% (-66·9 to -23·2%)	35·5% (24·7–47·1%)	1 (0·6–1·5)	-53·6% (-69·5 to -23%)
	Neurological disorders*	5·5% (1·6–12·6%)	74·4 (19·8–183·3)	59·8% (26·6 to 143·2%)	12·9% (4·6–24·3%)	4·7 (0·7–14·2)	64% (24·9 to 169·9%)
	Alzheimer's disease and other dementias	18·5% (8–32·9%)	74·4 (19·8–183·3)	59·8% (26·6 to 143·2%)	17·5% (7·3–31·5%)	4·7 (0·7–14·2)	64% (24·9 to 169·9%)
High fasting plasma glucose	Stroke	30·6% (18·6–51·1%)	536·7 (315·2–918·5)	5·9% (-25·1 to 46·9%)	31% (16·5–61·7%)	31·5 (16·3–62·5)	3·2% (-29·5 to 43·2%)
	Ischaemic stroke	32·1% (17–60·5%)	408·8 (207·4–775·8)	37·7% (-0·4 to 92%)	31·5% (14·3–69·9%)	25·2 (11·1–55·6)	27·7% (-9·9 to 78·8%)
	Intracerebral haemorrhage	27% (17·5–38·9%)	109·2 (64·2–169·6)	-40% (-57·8 to -12·6%)	29·2% (17·9–47·7%)	5·4 (3–9·1)	-43% (-61 to -17·7%)
	Subarachnoid haemorrhage	24·3% (15·8–34·9%)	18·7 (10·7–28·9)	-32·9% (-56·4 to 9·5%)	28·7% (17·9–45·2%)	0·8 (0·5–1·4)	-33·8% (-58·1 to 11·5%)
	Neurological disorders*	4·3% (0·7–11·9%)	58·3 (10·6–189·1)	56·6% (34·7 to 91·6%)	10·7% (2·3–23·8%)	3·9 (0·5–14·7)	53·1% (28·9 to 93·4%)
	Alzheimer's disease and other dementias	14·5% (3·6–29·8%)	58·3 (10·6–189·1)	56·6% (34·7 to 91·6%)	14·6% (3·6–29·7%)	3·9 (0·5–14·7)	53·1% (28·9 to 93·4%)
High LDL cholesterol	Stroke	15·5% (8·3–26·8%)	272 (137·7–493·8)	-16·8% (-32·6 to 5·3%)	14·2% (4·5–31·1%)	14·4 (4·5–33·7)	-22·2% (-37·6 to -0·2%)

Table S16 Regional and national disabilities and deaths related to neurological conditions attributable to risk factors in North Africa and Middle East countries

**Location**<br>Risk factor (alphabetic order)	Cause	**DALYs (95% UI)**<br>PAF (%)<br>2019	 Rate per 100,000<br>2019	 Rate change (%)<br>From 1990 to 2019	**Deaths (95% UI)**<br>PAF (%)<br>2019	 Rate per 100,000<br>2019	 Rate change (%)<br>From 1990 to 2019
	Ischaemic stroke	21·3% (11·5–36·7%)	272 (137·7–493·8)	-16·8% (-32·6 to 5·3%)	18% (5·9–38·9%)	14·4 (4·5–33·7)	-22·2% (-37·6 to -0·2%)
High systolic blood pressure	Stroke	49·7% (40·9–58·5%)	872 (662·5–1114·9)	-47·7% (-59·3 to -34·5%)	47·4% (36·4–58·9%)	48·1 (34·3–65)	-46·5% (-58·9 to -30·8%)
	Ischaemic stroke	47·9% (37·2–59%)	611·4 (445·2–805)	-27% (-42·9 to -6·3%)	45·6% (32·6–59·6%)	36·5 (24·6–51·3)	-29·3% (-46 to -6·6%)
	Intracerebral haemorrhage	54·5% (44·1–64%)	219·9 (158–290·6)	-69·3% (-77·2 to -59·2%)	54·2% (41·1–66·4%)	10 (7–13·5)	-70·3% (-77·6 to -60·4%)
	Subarachnoid haemorrhage	52·9% (42·5–62·5%)	40·7 (28·9–55·7)	-64·6% (-75·7 to -44%)	53·9% (41·8–65·2%)	1·5 (1·1–2·2)	-65·8% (-77·5 to -44·9%)
High temperature	Stroke	2·8% (1–5%)	48·8 (17·6–89)	-45·4% (-62·6 to -13·9%)	3·2% (1·2–5·8%)	3·3 (1·1–5·9)	-40·9% (-57·8 to -7%)
	Ischaemic stroke	2·7% (1–4·9%)	34·5 (12·3–62·3)	-24·5% (-47·8 to 16%)	3·2% (1·2–5·8%)	2·6 (0·9–4·7)	-24·7% (-46·3 to 17·1%)
	Intracerebral haemorrhage	3% (1·1–5·4%)	12·1 (4·4–22·4)	-67·6% (-78·7 to -51·1%)	3·2% (1·2–5·8%)	0·6 (0·2–1·1)	-67·8% (-78·6 to -50·7%)
	Subarachnoid haemorrhage	2·8% (1–5·1%)	2·2 (0·8–4·1)	-65·6% (-79·3 to -36%)	3·2% (1·2–5·8%)	0·1 (0–0·2)	-62·9% (-78·6 to -30·9%)
Household air pollution from solid fuels	Meningitis	0% (0–0%)	<0·1 (<0·1–<0·1)	-99·7% (-99·9 to -98·4%)	0% (0–0%)	<0·1 (<0·1–<0·1)	-99·7% (-99·9 to -98·4%)
	Encephalitis	0% (0–0%)	<0·1 (<0·1–<0·1)	-98·4% (-99·7 to -90·2%)	0% (0–0%)	<0·1 (<0·1–<0·1)	-98·4% (-99·7 to -90·2%)
	Stroke	0·1% (0–0·2%)	1·3 (0·5–2·7)	-99·2% (-99·7 to -98·1%)	0·1% (0–0·1%)	0·1 (0–0·1)	-99·2% (-99·7 to -98·1%)
	Ischaemic stroke	0·1% (0–0·1%)	0·9 (0·3–1·8)	-98·9% (-99·6 to -97·3%)	0·1% (0–0·1%)	<0·1 (<0·1–0·1)	-99% (-99·6 to -97·5%)
	Intracerebral haemorrhage	0·1% (0–0·2%)	0·3 (0·1–0·7)	-99·5% (-99·8 to -98·8%)	0·1% (0–0·1%)	<0·1 (<0·1–<0·1)	-99·6% (-99·8 to -98·9%)
	Subarachnoid haemorrhage	0·1% (0–0·2%)	0·1 (0–0·1)	-99·5% (-99·8 to -98·7%)	0·1% (0–0·2%)	<0·1 (<0·1–<0·1)	-99·5% (-99·8 to -98·7%)
Kidney dysfunction	Stroke	10·7% (7·6–13·5%)	188·2 (124·7–256·3)	-20·7% (-38·1 to 1·3%)	9·4% (4·9–13·6%)	9·5 (4·8–14·4)	-24·6% (-47·3 to -0·8%)
	Ischaemic stroke	11·5% (7·4–15·2%)	147·3 (89·5–207·2)	3·5% (-19·5 to 30·1%)	9·2% (3·6–14·2%)	7·4 (2·9–11·8)	-5·4% (-36·3 to 23%)
	Intracerebral haemorrhage	10·2% (8–12·3%)	40·9 (29·2–54·2)	-57% (-67·6 to -42%)	11·6% (7·9–15%)	2·1 (1·3–3)	-55·6% (-67·3 to -39·7%)
Lead exposure	Stroke	3·2% (1·4–5·1%)	55·5 (24·6–93·2)	-54·7% (-65·8 to -42·4%)	3·3% (1·7–5·3%)	3·4 (1·6–5·6)	-46·9% (-59·6 to -32·6%)
	Ischaemic stroke	3·2% (1·5–5·1%)	40·4 (17·6–68·9)	-31·8% (-47·6 to -15·1%)	3·2% (1·6–5·4%)	2·6 (1·2–4·5)	-26·4% (-43·2 to -6·6%)
	Intracerebral haemorrhage	3·2% (1·2–5·7%)	12·9 (4·8–23·7)	-76·4% (-83·7 to -67·8%)	3·7% (1·6–6·3%)	0·7 (0·3–1·2)	-72·7% (-80·1 to -63·8%)
	Subarachnoid haemorrhage	2·9% (1–5·2%)	2·2 (0·7–4·2)	-74·2% (-84·5 to -58%)	3·6% (1·5–6%)	0·1 (0–0·2)	-69·3% (-80·8 to -50%)
Low birth weight	Meningitis	3·3% (2–5·2%)	1·3 (0·8–2·2)	-83% (-91·7 to -65·3%)	1·7% (1–2·8%)	<0·1 (<0·1–<0·1)	-83% (-91·7 to -65·3%)
	Encephalitis	1·9% (0·9–3·6%)	0·4 (0·2–0·6)	-16·9% (-66·4 to 102·6%)	1·2% (0·6–2·2%)	<0·1 (<0·1–<0·1)	-16·9% (-66·4 to 102·6%)
Low birth weight and short gestation	Meningitis	4% (2·4–6·3%)	1·6 (0·9–2·7)	-81·7% (-91·1 to -62·8%)	2·1% (1·2–3·4%)	<0·1 (<0·1–<0·1)	-81·7% (-91·1 to -62·8%)
	Encephalitis	2·3% (1·1–4·3%)	0·4 (0·2–0·8)	-12·2% (-64·5 to 112·1%)	1·4% (0·7–2·6%)	<0·1 (<0·1–<0·1)	-12·2% (-64·5 to 112·1%)
Low physical activity	Stroke	6·3% (1·4–13·5%)	110·3 (23·5–231·6)	-20·2% (-35·4 to 0·4%)	8·1% (1·9–15·9%)	8·2 (1·9–16·2)	-22·9% (-38·5 to -0·6%)
	Ischaemic stroke	8·7% (1·9–18·4%)	110·3 (23·5–231·6)	-20·2% (-35·4 to 0·4%)	10·3% (2·4–20·2%)	8·2 (1·9–16·2)	-22·9% (-38·5 to -0·6%)
Low temperature	Stroke	4·1% (0·7–7·2%)	71·2 (12·6–130·3)	-45·5% (-60·5 to -28·8%)	4·7% (0·8–8·3%)	4·8 (0·8–8·8)	-41% (-56·9 to -24·4%)
	Ischaemic stroke	4% (0·7–7%)	50·4 (8·5–93·5)	-24·7% (-46·3 to -2%)	4·7% (0·8–8·3%)	3·8 (0·6–7)	-24·7% (-45·9 to -3·5%)
	Intracerebral haemorrhage	4·4% (0·8–7·7%)	17·6 (3·5–31·9)	-67·7% (-77·5 to -56·3%)	4·7% (0·8–8·3%)	0·9 (0·2–1·6)	-68% (-77·5 to -56·5%)
	Subarachnoid haemorrhage	4·1% (0·7–7·3%)	3·2 (0·6–6·1)	-65·7% (-78 to -43·8%)	4·7% (0·8–8·3%)	0·1 (0–0·3)	-63·1% (-76·8 to -39·9%)
Metabolic risk factors†	Stroke	73·3% (65·4–82·2%)	1287·3 (1028·1–1590)	-39·7% (-52·4 to -24·6%)	70% (58·7–83·3%)	71·1 (55·1–91·8)	-39·1% (-53·1 to -23·1%)

Table S16 Regional and national disabilities and deaths related to neurological conditions attributable to risk factors in North Africa and Middle East countries

Location<br>Risk factor (alphabetic order)	Cause	DALYs (95% UI)<br>PAF (%)<br>2019	 Rate per 100,000<br>2019	 Rate change (%)<br>From 1990 to 2019	Deaths (95% UI)<br>PAF (%)<br>2019	 Rate per 100,000<br>2019	 Rate change (%)<br>From 1990 to 2019
	Ischaemic stroke	73·4% (63·5–85·2%)	936·4 (732·6–1185·7)	-18·1% (-35 to 2·2%)	69·5% (55·8–86·2%)	55·7 (41·6–74)	-21·7% (-39·1 to 0·3%)
	Intracerebral haemorrhage	73·7% (65·9–80·7%)	297·5 (228·6–380·6)	-65·3% (-73·8 to -53·7%)	72·5% (62·3–81·6%)	13·4 (9·9–17·5)	-66·7% (-75 to -55·9%)
	Subarachnoid haemorrhage	69·5% (60·7–77·2%)	53·4 (39·3–71·6)	-60·3% (-72·5 to -37·4%)	69·3% (58·5–78·3%)	2 (1·4–2·7)	-61·9% (-74·6 to -39%)
	Neurological disorders*	9% (3–19·6%)	121·9 (36·4–312)	52·9% (27 to 102·6%)	21·7% (8·8–37·5%)	8 (1·4–24·7)	53·4% (24 to 114·7%)
	Alzheimer's disease and other dementias	30·3% (14·8–48·8%)	121·9 (36·4–312)	52·9% (27 to 102·6%)	29·5% (14–47·8%)	8 (1·4–24·7)	53·4% (24 to 114·7%)
Non-optimal temperature	Stroke	6·7% (3·4–9·8%)	117·8 (58·9–183·6)	-45·5% (-59·1 to -24·1%)	7·8% (4–11·3%)	7·9 (4–12·2)	-41% (-55·2 to -19·4%)
	Ischaemic stroke	6·5% (3·3–9·6%)	83·4 (41·5–130·2)	-24·7% (-44·2 to 4%)	7·8% (4–11·3%)	6·2 (3·1–9·6)	-24·8% (-43·9 to 1·5%)
	Intracerebral haemorrhage	7·2% (3·7–10·5%)	29·1 (14·4–45)	-67·7% (-77·2 to -54·3%)	7·8% (4–11·3%)	1·4 (0·7–2·2)	-68% (-77 to -54·4%)
	Subarachnoid haemorrhage	6·8% (3·5–9·9%)	5·3 (2·6–8·4)	-65·7% (-77·2 to -40·7%)	7·8% (4–11·3%)	0·2 (0·1–0·3)	-63·1% (-75·7 to -37·5%)
Other environmental risks	Stroke	3·2% (1·4–5·1%)	55·5 (24·6–93·2)	-54·7% (-65·8 to -42·4%)	3·3% (1·7–5·3%)	3·4 (1·6–5·6)	-46·9% (-59·6 to -32·6%)
	Ischaemic stroke	3·2% (1·5–5·1%)	40·4 (17·6–68·9)	-31·8% (-47·6 to -15·1%)	3·2% (1·6–5·4%)	2·6 (1·2–4·5)	-26·4% (-43·2 to -6·6%)
	Intracerebral haemorrhage	3·2% (1·2–5·7%)	12·9 (4·8–23·7)	-76·4% (-83·7 to -67·8%)	3·7% (1·6–6·3%)	0·7 (0·3–1·2)	-72·7% (-80·1 to -63·8%)
	Subarachnoid haemorrhage	2·9% (1–5·2%)	2·2 (0·7–4·2)	-74·2% (-84·5 to -58%)	3·6% (1·5–6%)	0·1 (0–0·2)	-69·3% (-80·8 to -50%)
Particulate matter pollution	Meningitis	0·8% (0·3–1·5%)	0·3 (0·1–0·6)	-78% (-94 to -3·9%)	0·4% (0·1–0·8%)	<0·1 (<0·1–<0·1)	-78% (-94 to -3·9%)
	Encephalitis	0·4% (0·1–1%)	0·1 (0–0·2)	6% (-74·1 to 359·5%)	0·3% (0·1–0·6%)	<0·1 (<0·1–<0·1)	6% (-74·1 to 359·5%)
	Stroke	23·4% (17–29·3%)	410·3 (278·6–551·2)	-44·6% (-56·3 to -31%)	19·2% (13·7–24·5%)	19·5 (12·9–26·8)	-44·9% (-56·7 to -30·6%)
	Ischaemic stroke	22·2% (16–28·2%)	283·8 (194–385·1)	-22·1% (-37·7 to -2·7%)	18·4% (13–23·5%)	14·8 (9·7–20·2)	-27·5% (-43·2 to -8·3%)
	Intracerebral haemorrhage	26·2% (19·3–32·5%)	105·8 (72·3–150·1)	-67·1% (-75·5 to -56·2%)	22·1% (16–27·9%)	4·1 (2·7–5·8)	-68·9% (-76·7 to -58·7%)
	Subarachnoid haemorrhage	27% (19·9–33·8%)	20·7 (13·4–29·5)	-62·1% (-73·7 to -39·9%)	23·5% (17–29·5%)	0·7 (0·4–1)	-64·4% (-76·1 to -42·1%)
Secondhand smoke	Stroke	4·7% (3·6–5·8%)	82·3 (58·4–109·2)	-46·1% (-58·5 to -31·3%)	3·8% (2·9–4·8%)	3·9 (2·8–5·2)	-44% (-56·7 to -28·8%)
	Ischaemic stroke	4·3% (3·2–5·4%)	54·8 (38·3–73·4)	-20·6% (-38·7 to 0·7%)	3·6% (2·7–4·6%)	2·9 (2·1–3·9)	-24·5% (-41·9 to -4·7%)
	Intracerebral haemorrhage	5·7% (4·3–7·2%)	23 (15·7–31·6)	-67·6% (-76·4 to -55·5%)	4·7% (3·6–5·9%)	0·9 (0·6–1·2)	-68·2% (-76·9 to -56·9%)
	Subarachnoid haemorrhage	5·9% (4·3–7·5%)	4·5 (3–6·5)	-64·6% (-76 to -42·9%)	5·1% (3·8–6·4%)	0·1 (0·1–0·2)	-65·3% (-77·1 to -42·9%)
Short gestation	Meningitis	2·9% (1·7–4·4%)	1·2 (0·6–1·9)	-78·5% (-89·6 to -56·3%)	1·5% (0·8–2·5%)	<0·1 (<0·1–<0·1)	-78·5% (-89·6 to -56·3%)
	Encephalitis	1·6% (0·8–3·1%)	0·3 (0·2–0·6)	2·6% (-59·1 to 150·2%)	1% (0·5–1·8%)	<0·1 (<0·1–<0·1)	2·6% (-59·2 to 150·2%)
Smoking	Stroke	11·2% (10–12·8%)	197·1 (156–245·9)	-53% (-63·5 to -39·8%)	8·5% (7·5–9·9%)	8·7 (6·6–10·9)	-55·2% (-65·8 to -41·3%)
	Ischaemic stroke	10·6% (9·4–12·1%)	135·7 (105·2–172·4)	-34·6% (-49·1 to -14·4%)	8% (6·8–9·3%)	6·4 (4·7–8·2)	-41% (-55·2 to -21·9%)
	Intracerebral haemorrhage	12·8% (10·8–15%)	51·7 (38–67·6)	-72% (-79·8 to -61·5%)	10·5% (8·8–12·4%)	1·9 (1·4–2·6)	-74·2% (-81·6 to -63·8%)
	Subarachnoid haemorrhage	12·6% (9·8–15·5%)	9·7 (6·3–14·5)	-64·5% (-78 to -39·6%)	11·3% (8·5–14·2%)	0·3 (0·2–0·5)	-67·2% (-80·1 to -42·2%)
	Neurological disorders*	5% (1·8–10·5%)	68·1 (22·2–168·1)	-16·6% (-31·9 to 2·7%)	11·7% (4·1–18·9%)	4·4 (0·6–13·9)	-18·4% (-37 to 17·8%)
	Alzheimer's disease and other dementias	18·3% (12–24·6%)	73·5 (28·4–173·9)	-19·7% (-34·5 to -3·1%)	18·2% (11·7–24·7%)	4·9 (1·1–14·2)	-21·2% (-38·2 to 0·7%)

Table S16 Regional and national disabilities and deaths related to neurological conditions attributable to risk factors in North Africa and Middle East countries

**Location**<br>Risk factor (alphabetic order)	Cause	**DALYs (95% UI)**<br>PAF (%)<br>2019	Rate per 100,000<br>2019	Rate change (%)<br>From 1990 to 2019	**Deaths (95% UI)**<br>PAF (%)<br>2019	Rate per 100,000<br>2019	Rate change (%)<br>From 1990 to 2019
	Parkinson's disease	-9·1% (-13·6–-4·9%)	-7·2 (-11·5–-3·7)	-38% (-55·3 to -14·6%)	-8·7% (-13–-4·6%)	-0·5 (-0·8–-0·2)	-39·9% (-57·7 to -14·3%)
	Multiple sclerosis	9·3% (6·6–12·1%)	1·9 (1·2–2·6)	-1·8% (-30·6 to 27·5%)	10·5% (7·3–14·1%)	<0·1 (<0·1–<0·1)	-14·5% (-53·2 to 30·4%)
Tobacco	Stroke	15·4% (13·9–17·1%)	270·6 (214·7–337·9)	-51·1% (-61·5 to -37·8%)	12% (10·6–13·6%)	12·2 (9·5–15·3)	-52·2% (-62·9 to -38·4%)
	Ischaemic stroke	14·5% (12·9–16·1%)	184·8 (144·7–232)	-31·1% (-46·2 to -12%)	11·3% (9·8–12·8%)	9 (6·9–11·4)	-36·7% (-51 to -18·2%)
	Intracerebral haemorrhage	17·9% (15·8–20·2%)	72·1 (53·9–93·8)	-70·7% (-78·6 to -60·1%)	14·7% (12·8–16·7%)	2·7 (2–3·5)	-72·6% (-80·1 to -62·2%)
	Subarachnoid haemorrhage	17·8% (15·2–20·7%)	13·7 (9·5–19·7)	-64·4% (-76·6 to -42·7%)	15·9% (13·2–18·8%)	0·5 (0·3–0·7)	-66·6% (-78·7 to -44·2%)
	Neurological disorders*	5% (1·8–10·5%)	68·1 (22·2–168·1)	-16·6% (-31·9 to 2·7%)	11·7% (4·1–18·9%)	4·4 (0·6–13·9)	-18·4% (-37 to 17·8%)
	Alzheimer's disease and other dementias	18·3% (12–24·6%)	73·5 (28·4–173·9)	-19·7% (-34·5 to -3·1%)	18·2% (11·7–24·7%)	4·9 (1·1–14·2)	-21·2% (-38·2 to 0·7%)
	Parkinson's disease	-9·1% (-13·6–-4·9%)	-7·2 (-11·5–-3·7)	-38% (-55·3 to -14·6%)	-8·7% (-13–-4·6%)	-0·5 (-0·8–-0·2)	-39·9% (-57·7 to -14·3%)
	Multiple sclerosis	9·3% (6·6–12·1%)	1·9 (1·2–2·6)	-1·8% (-30·6 to 27·5%)	10·5% (7·3–14·1%)	<0·1 (<0·1–<0·1)	-14·5% (-53·2 to 30·4%)
**Bahrain**							
Air pollution	Meningitis	0·4% (0·2–0·7%)	0·1 (0–0·1)	-88·8% (-95·5 to -71·5%)	0·2% (0·1–0·3%)	<0·1 (<0·1–<0·1)	-88·8% (-95·5 to -71·5%)
	Encephalitis	0·2% (0·1–0·4%)	<0·1 (<0·1–<0·1)	-52·7% (-83·4 to 28·6%)	0·1% (0·1–0·3%)	<0·1 (<0·1–<0·1)	-52·8% (-83·4 to 28·6%)
	Stroke	32·4% (28–36·5%)	305·3 (242·7–382·6)	-54·8% (-63·5 to -43·6%)	27·7% (23·9–31·5%)	14·6 (11·4–19·4)	-53·1% (-63·3 to -40·9%)
	Ischaemic stroke	30·8% (26·7–34·9%)	189·2 (151·9–238·4)	-46·7% (-56·8 to -35%)	26·3% (22·6–30·1%)	9·9 (7·7–13·2)	-45·9% (-58·5 to -31·7%)
	Intracerebral haemorrhage	35·5% (30·8–39·8%)	94·1 (73·7–121·5)	-65·9% (-73·8 to -55·2%)	30·7% (26·6–34·9%)	4 (3·1–5·3)	-64·7% (-73·1 to -53·1%)
	Subarachnoid haemorrhage	35·1% (30·6–39·3%)	22 (16·3–31·6)	-51·5% (-67·1 to -30·5%)	32·5% (28·2–36·5%)	0·7 (0·5–1·1)	-53·2% (-70 to -29·4%)
Alcohol use	Stroke	0·7% (0·2–1·3%)	6·7 (2·2–11·9)	-86·7% (-95 to -77·4%)	0·3% (0–0·7%)	0·2 (0–0·4)	-90·6% (-100·3 to -80·6%)
	Ischaemic stroke	0·1% (-0·3–0·5%)	0·4 (-2–3·2)	-95·1% (-168·3 to -73·5%)	-0·1% (-0·4–0·2%)	0 (-0·1–0·1)	-108·1% (-307·6 to -76·5%)
	Intracerebral haemorrhage	2·4% (1·2–3·7%)	6·2 (3·1–10·1)	-84·9% (-91·4 to -76·7%)	1·5% (0·7–2·5%)	0·2 (0·1–0·3)	-86·6% (-93 to -78·4%)
	Neurological disorders*	0·5% (0·2–1%)	6·9 (2·9–12·8)	-64·8% (-85·2 to -20%)	0·2% (0·1–0·5%)	0·1 (0–0·1)	-68·1% (-79·3 to -53·2%)
	Idiopathic epilepsy	3·7% (2·4–5·6%)	6·9 (2·9–12·8)	-64·8% (-85·2 to -20%)	3·6% (2·2–5·4%)	0·1 (0–0·1)	-68·1% (-79·3 to -53·2%)
All risk factors	Meningitis	1·8% (1·2–2·5%)	0·3 (0·2–0·5)	-88·3% (-93·6 to -79·2%)	0·9% (0·6–1·3%)	<0·1 (<0·1–<0·1)	-88·3% (-93·6 to -79·2%)
	Encephalitis	1% (0·6–1·6%)	0·1 (0·1–0·2)	-50·5% (-77·4 to 0·3%)	0·7% (0·4–1%)	<0·1 (<0·1–<0·1)	-50·5% (-77·5 to 0·3%)
	Stroke	86·9% (82·9–90·8%)	818·1 (689·8–989·9)	-52·2% (-61 to -41·9%)	84·8% (78·8–91%)	44·8 (36·4–56·8)	-49·1% (-59·1 to -37·3%)
	Ischaemic stroke	87% (81·6–92·8%)	533·7 (449·2–646·3)	-44·2% (-53·9 to -33·1%)	84·6% (76·9–93·1%)	31·8 (25·5–40·5)	-42·1% (-54·2 to -28·9%)
	Intracerebral haemorrhage	88·3% (84·1–91·7%)	233·8 (190·5–290·2)	-64·2% (-72·1 to -54·1%)	85·7% (79·4–91·3%)	11·2 (9–14·5)	-61·8% (-70·6 to -50·6%)
	Subarachnoid haemorrhage	80·6% (76–85·1%)	50·6 (38·5–71·7)	-50·7% (-66·3 to -29·3%)	82·7% (76·5–88·2%)	1·8 (1·3–2·7)	-50·9% (-68·8 to -27·3%)
	Neurological disorders*	14·8% (6·1–29·7%)	203·7 (76·1–504·8)	6·5% (-11·7 to 23·9%)	32·8% (15·1–51·6%)	12·9 (2·5–39·2)	15% (-5·1 to 41·4%)
	Alzheimer's disease and other dementias	50·2% (33–68%)	202·7 (75·4–507·7)	11·4% (-5·5 to 29·4%)	48·3% (30·8–66·1%)	13·2 (2·8–39·5)	13·5% (-6·5 to 35·9%)
	Parkinson's disease	-8·2% (-12·5–-4·4%)	-7·3 (-11·8–-3·6)	-38·3% (-57·6 to -11·6%)	-7·4% (-11·4–-3·8%)	-0·4 (-0·7–-0·2)	-34·2% (-56·4 to -4·9%)

Table S16 Regional and national disabilities and deaths related to neurological conditions attributable to risk factors in North Africa and Middle East countries

**Location**<br>Risk factor (alphabetic order)	Cause	**DALYs (95% UI)**<br>PAF (%)<br>2019	 Rate per 100,000<br>2019	 Rate change (%)<br>From 1990 to 2019	**Deaths (95% UI)**<br>PAF (%)<br>2019	 Rate per 100,000<br>2019	 Rate change (%)<br>From 1990 to 2019
	Idiopathic epilepsy	3·7% (2·4–5·6%)	6·9 (2·9–12·8)	-64·8% (-85·2 to -20%)	3·6% (2·2–5·4%)	0·1 (0–0·1)	-68·1% (-79·3 to -53·2%)
	Multiple sclerosis	10·7% (7·7–13·9%)	1·3 (0·8–2)	-4·7% (-30 to 22·4%)	11·1% (7·6–14·6%)	<0·1 (<0·1–<0·1)	-25·6% (-60·1 to 10%)
Ambient particulate matter pollution	Meningitis	0·4% (0·2–0·6%)	0·1 (0–0·1)	-87·6% (-95 to -68·2%)	0·2% (0·1–0·3%)	<0·1 (<0·1–<0·1)	-87·6% (-95 to -68·2%)
	Encephalitis	0·2% (0·1–0·4%)	<0·1 (<0·1–<0·1)	-47·6% (-81·9 to 42·9%)	0·1% (0·1–0·3%)	<0·1 (<0·1–<0·1)	-47·6% (-81·9 to 42·9%)
	Stroke	32·4% (28–36·4%)	304·4 (241·6–382)	-50·8% (-60·9 to -37·8%)	27·6% (23·8–31·4%)	14·6 (11·4–19·3)	-48·8% (-60·1 to -33·9%)
	Ischaemic stroke	30·7% (26·6–34·8%)	188·6 (151·5–238·1)	-41·8% (-53·9 to -27·7%)	26·2% (22·6–30·1%)	9·9 (7·7–13·2)	-40·9% (-54·4 to -24·4%)
	Intracerebral haemorrhage	35·4% (30·8–39·7%)	93·8 (73·5–121·1)	-62·8% (-71·7 to -51%)	30·6% (26·5–34·6%)	4 (3·1–5·3)	-61·5% (-71·2 to -48·1%)
	Subarachnoid haemorrhage	35% (30·5–39·2%)	22 (16·3–31·5)	-47·2% (-64·1 to -23%)	32·4% (28·1–36·4%)	0·7 (0·5–1·1)	-49% (-67·1 to -23·3%)
Behavioural risk factors‡	Meningitis	1·7% (1·2–2·4%)	0·3 (0·2–0·5)	-88·1% (-93·5 to -78·8%)	0·9% (0·6–1·2%)	<0·1 (<0·1–<0·1)	-88·1% (-93·5 to -78·8%)
	Encephalitis	1% (0·6–1·6%)	0·1 (0·1–0·2)	-49·5% (-77·1 to 2%)	0·6% (0·4–1%)	<0·1 (<0·1–<0·1)	-49·5% (-77·1 to 2%)
	Stroke	29·8% (24·9–35·6%)	280·1 (215·7–361·9)	-59% (-67·5 to -49·6%)	26·6% (20·9–32·8%)	14 (10·1–19·2)	-56·2% (-65·9 to -45%)
	Ischaemic stroke	31·2% (24·3–39%)	191·4 (141–254·7)	-50·4% (-59·8 to -40·8%)	28·2% (20·6–36·6%)	10·6 (7·3–14·9)	-48·2% (-59·9 to -36%)
	Intracerebral haemorrhage	27·3% (22·4–32·7%)	72·3 (53·7–96·5)	-72·2% (-79 to -63·1%)	22·3% (18·1–27·3%)	2·9 (2·2–4)	-71·7% (-78·7 to -62·1%)
	Subarachnoid haemorrhage	26·2% (20·8–31·8%)	16·5 (11·4–24·5)	-56·6% (-71·5 to -36·6%)	23·6% (18·6–29·2%)	0·5 (0·4–0·8)	-58·8% (-74·2 to -37·7%)
	Neurological disorders*	4·8% (1·9–9·8%)	66 (25–153·9)	-24·2% (-43·2 to -3·8%)	8·8% (2·6–14·9%)	3·5 (0·4–10·7)	-12·4% (-33 to 18·9%)
	Alzheimer's disease and other dementias	16·2% (9·7–22·5%)	65 (24·5–154·3)	-16·5% (-33·8 to 6·7%)	14·3% (8·5–20·3%)	3·9 (0·8–11)	-13·3% (-34·4 to 12·4%)
	Parkinson's disease	-8·2% (-12·5–-4·4%)	-7·3 (-11·8–-3·6)	-38·3% (-57·6 to -11·6%)	-7·4% (-11·4–-3·8%)	-0·4 (-0·7–-0·2)	-34·2% (-56·4 to -4·9%)
	Idiopathic epilepsy	3·7% (2·4–5·6%)	6·9 (2·9–12·8)	-64·8% (-85·2 to -20%)	3·6% (2·2–5·4%)	0·1 (0–0·1)	-68·1% (-79·3 to -53·2%)
	Multiple sclerosis	10·7% (7·7–13·9%)	1·3 (0·8–2)	-4·7% (-30 to 22·4%)	11·1% (7·6–14·6%)	<0·1 (<0·1–<0·1)	-25·6% (-60·1 to 10%)
Child and maternal malnutrition	Meningitis	1·7% (1·2–2·4%)	0·3 (0·2–0·5)	-88·1% (-93·5 to -78·8%)	0·9% (0·6–1·2%)	<0·1 (<0·1–<0·1)	-88·1% (-93·5 to -78·8%)
	Encephalitis	1% (0·6–1·6%)	0·1 (0·1–0·2)	-49·5% (-77·1 to 2%)	0·6% (0·4–1%)	<0·1 (<0·1–<0·1)	-49·5% (-77·1 to 2%)
Diet high in red meat	Stroke	4·5% (1·9–6·5%)	42 (17·3–65·3)	-54·1% (-65·9 to -38·7%)	3·3% (1·4–5%)	1·8 (0·7–2·8)	-53·6% (-66·4 to -36·3%)
	Ischaemic stroke	3·8% (0·9–5·9%)	23·4 (5·4–38·1)	-44·3% (-57·6 to -26·4%)	2·8% (0·6–4·5%)	1·1 (0·2–1·8)	-45% (-60·9 to -24·6%)
	Intracerebral haemorrhage	5·6% (1·3–8·8%)	14·8 (3·5–25·1)	-64·7% (-74·7 to -51·3%)	4·5% (0·9–7·1%)	0·6 (0·1–1)	-63·9% (-74·3 to -50·1%)
	Subarachnoid haemorrhage	6% (1·5–9·4%)	3·8 (0·9–6·4)	-49·6% (-67·4 to -24·4%)	5% (1·1–7·9%)	0·1 (0–0·2)	-52·1% (-70·2 to -27·2%)
Diet high in sodium	Stroke	1·8% (0·3–7·2%)	17·1 (2·3–68·4)	-53·3% (-72·3 to -19·7%)	1·4% (0·3–5·8%)	0·8 (0·1–3·1)	-52·6% (-71 to -16%)
	Ischaemic stroke	1·6% (0·3–6·5%)	9·9 (1·5–39·8)	-44·4% (-68·3 to -6%)	1·3% (0·3–5·1%)	0·5 (0·1–1·9)	-44·2% (-67·3 to -2%)
	Intracerebral haemorrhage	2·2% (0·3–8·7%)	5·8 (0·7–24·4)	-64·2% (-79·7 to -33·8%)	1·8% (0·3–7·4%)	0·2 (0–1·1)	-63·8% (-78·7 to -31·6%)
	Subarachnoid haemorrhage	2·2% (0·2–8·5%)	1·4 (0·1–5·5)	-46·9% (-71·9 to 2·1%)	2% (0·3–8%)	<0·1 (<0·1–0·2)	-49·7% (-73 to -3·2%)
Diet low in fibre	Stroke	0·7% (0·3–1·6%)	6·9 (2·3–15·3)	-63·5% (-76·4 to -43·7%)	0·7% (0·2–1·5%)	0·4 (0·1–0·8)	-61·5% (-76·1 to -38·6%)
	Ischaemic stroke	0·7% (0·2–1·5%)	4·3 (1·5–9·6)	-56·7% (-72·1 to -33·4%)	0·6% (0·2–1·4%)	0·2 (0·1–0·5)	-55·7% (-73·8 to -26·7%)
	Intracerebral haemorrhage	0·8% (0·3–1·7%)	2·1 (0·7–4·7)	-72·5% (-83 to -55·4%)	0·7% (0·3–1·6%)	0·1 (0–0·2)	-70·9% (-82 to -54·1%)

Table S16 Regional and national disabilities and deaths related to neurological conditions attributable to risk factors in North Africa and Middle East countries

**Location**<br>Risk factor (alphabetic order)	Cause	**DALYs (95% UI)**<br>PAF (%)<br>2019	 Rate per 100,000<br>2019	 Rate change (%)<br>From 1990 to 2019	**Deaths (95% UI)**<br>PAF (%)<br>2019	 Rate per 100,000<br>2019	 Rate change (%)<br>From 1990 to 2019
	Subarachnoid haemorrhage	0·9% (0·2–1·9%)	0·5 (0·1–1·3)	-62·7% (-77·9 to -34·2%)	0·8% (0·3–1·7%)	<0·1 (<0·1–<0·1)	-63·2% (-78·6 to -34·3%)
Diet low in fruits	Stroke	2·3% (1·1–3·7%)	21·5 (10–36·1)	-60·5% (-73·4 to -42%)	1·7% (0·8–3%)	0·9 (0·4–1·6)	-60·3% (-75·6 to -36·1%)
	Ischaemic stroke	1·7% (0·4–3·3%)	10·3 (2·3–20·7)	-51·2% (-67·6 to -28·2%)	1·3% (0·3–2·7%)	0·5 (0·1–1)	-52% (-71·7 to -22·3%)
	Intracerebral haemorrhage	3·3% (1·5–5·6%)	8·8 (4–15·5)	-68·5% (-79·1 to -52·1%)	2·7% (1·2–4·5%)	0·3 (0·2–0·6)	-68·2% (-80·2 to -50·6%)
	Subarachnoid haemorrhage	3·8% (1·8–6·7%)	2·4 (1·1–4·3)	-55·6% (-72·7 to -32·7%)	3% (1·4–5·2%)	0·1 (0–0·1)	-58·5% (-75·7 to -31·9%)
Diet low in vegetables	Stroke	0·7% (0·2–1·5%)	6·6 (2·1–14·4)	-70·2% (-81·3 to -53·2%)	0·7% (0·2–1·4%)	0·4 (0·1–0·7)	-67·1% (-80·2 to -48·3%)
	Ischaemic stroke	0·7% (0·2–1·4%)	4·2 (1·2–8·8)	-63·2% (-76·7 to -42·4%)	0·7% (0·2–1·4%)	0·2 (0·1–0·5)	-60·6% (-76·1 to -38·5%)
	Intracerebral haemorrhage	0·8% (0·2–2·4%)	2 (0·5–6·6)	-78·6% (-88·2 to -61·2%)	0·8% (0·2–2·4%)	0·1 (0–0·3)	-76·2% (-86·7 to -57·2%)
	Subarachnoid haemorrhage	0·7% (0·2–2·4%)	0·5 (0·1–1·6)	-71% (-84·6 to -44·6%)	0·8% (0·2–2·4%)	<0·1 (<0·1–0·1)	-70% (-84·6 to -41·7%)
Diet low in whole grains	Stroke	4% (1·5–5·6%)	37·7 (14–55·6)	-49·3% (-60·7 to -39%)	3·7% (1·5–5·1%)	2 (0·8–2·9)	-48·6% (-60·4 to -35·7%)
	Ischaemic stroke	6·1% (2·3–8·5%)	37·7 (14–55·6)	-49·3% (-60·7 to -39%)	5·3% (2·1–7·2%)	2 (0·8–2·9)	-48·6% (-60·4 to -35·7%)
Dietary risks	Stroke	13·1% (9·2–18·2%)	123·6 (83·6–180·8)	-55·6% (-64·9 to -45%)	11·1% (7·7–15·4%)	5·8 (3·9–8·7)	-54·5% (-64·7 to -42·9%)
	Ischaemic stroke	13·6% (8·7–18·4%)	83·7 (52·3–120·8)	-48·9% (-58·8 to -38·2%)	11·4% (7·5–15·3%)	4·3 (2·6–6·2)	-48·7% (-60·1 to -35·7%)
	Intracerebral haemorrhage	12% (6·5–18·3%)	31·8 (16·5–51·1)	-67·3% (-76·2 to -56·9%)	10·1% (5·4–15·6%)	1·3 (0·7–2·1)	-66·6% (-75·9 to -55·4%)
	Subarachnoid haemorrhage	12·8% (7·1–19%)	8 (4·1–12·9)	-53·5% (-69·5 to -33%)	11% (6–16·8%)	0·2 (0·1–0·4)	-55·8% (-72·5 to -32·7%)
Environmental/occupational risk factors§	Meningitis	0·4% (0·2–0·7%)	0·1 (0–0·1)	-88·8% (-95·5 to -71·5%)	0·2% (0·1–0·3%)	<0·1 (<0·1–<0·1)	-88·8% (-95·5 to -71·5%)
	Encephalitis	0·2% (0·1–0·4%)	<0·1 (<0·1–<0·1)	-52·7% (-83·4 to 28·6%)	0·1% (0·1–0·3%)	<0·1 (<0·1–<0·1)	-52·8% (-83·4 to 28·6%)
	Stroke	37·8% (33–42·2%)	355·7 (283·7–453·8)	-54·6% (-63·3 to -43·1%)	34·4% (29·5–38·9%)	18·2 (13·9–25)	-51·6% (-61·8 to -37·6%)
	Ischaemic stroke	36·1% (31·3–40·4%)	221·7 (178·1–283·4)	-46·3% (-56·3 to -33·8%)	33·1% (28·1–37·6%)	12·4 (9·5–17·1)	-44·1% (-56·3 to -27·7%)
	Intracerebral haemorrhage	41·1% (36·1–45·9%)	108·7 (84·7–139·9)	-65·8% (-73·8 to -54·6%)	37·3% (32·2–42·1%)	4·9 (3·7–6·5)	-63·7% (-72·3 to -50·8%)
	Subarachnoid haemorrhage	40·2% (35·4–45·1%)	25·3 (18·5–36·3)	-53·1% (-68 to -31·7%)	38·8% (33·6–43·7%)	0·9 (0·6–1·3)	-52·8% (-69·7 to -29·2%)
High body-mass index	Stroke	33·8% (24·3–42·5%)	317·5 (218·2–428·4)	-49·7% (-59·4 to -36·6%)	20·8% (13·2–28·7%)	11 (6·6–16)	-50·2% (-62·1 to -35·4%)
	Ischaemic stroke	26·2% (17·7–35·1%)	160·4 (103·6–226·9)	-36·4% (-48·4 to -21·2%)	15·9% (8·7–24·6%)	6 (3·1–10)	-37·9% (-53·9 to -20%)
	Intracerebral haemorrhage	47·2% (35·2–58·1%)	124·9 (85·1–168·8)	-61% (-69·8 to -47·9%)	31·7% (20·9–43·8%)	4·1 (2·6–6·2)	-61·5% (-71·7 to -47·9%)
	Subarachnoid haemorrhage	51·6% (40–61·4%)	32·3 (22·4–46·3)	-44·9% (-61·8 to -20·7%)	38% (27·1–49·6%)	0·8 (0·5–1·3)	-48·2% (-66·4 to -21·6%)
	Neurological disorders*	6·6% (2–14·6%)	90·4 (24·9–226·4)	21·3% (1·5 to 56·7%)	15·2% (5·2–28·3%)	5·9 (1–17·6)	20·6% (-2·5 to 62·9%)
	Alzheimer's disease and other dementias	22·4% (9·8–38·2%)	90·4 (24·9–226·4)	21·3% (1·5 to 56·7%)	21·6% (9·3–37·4%)	5·9 (1–17·6)	20·6% (-2·5 to 62·9%)
High fasting plasma glucose	Stroke	30·7% (19·8–47·9%)	289·5 (179·9–474·6)	-53·2% (-64·3 to -40·1%)	30·1% (17·3–54·3%)	16 (8·9–30·9)	-51·7% (-64·8 to -37·4%)
	Ischaemic stroke	31·4% (17·4–57·5%)	193·4 (101·1–355·5)	-47·2% (-60·3 to -32·3%)	30·1% (14·1–63·6%)	11·4 (5·2–24·9)	-45·9% (-61 to -29·6%)
	Intracerebral haemorrhage	30·4% (19·9–43·1%)	80·6 (48·8–123·8)	-64% (-73·3 to -50%)	30·2% (18·5–48·1%)	3·9 (2·3–6·6)	-63·3% (-73·3 to -49·9%)
	Subarachnoid haemorrhage	24·5% (15·4–35·7%)	15·4 (9–25·8)	-46% (-66·6 to -18·1%)	28·4% (18–42·6%)	0·6 (0·4–1·1)	-50·3% (-70·1 to -23·4%)
	Neurological disorders*	6·9% (1·4–17·8%)	94·8 (18·8–285·2)	47·2% (25·7 to 81·8%)	16·2% (3·9–34·2%)	6·3 (0·8–22)	47·1% (22·5 to 87·5%)

Table S16 Regional and national disabilities and deaths related to neurological conditions attributable to risk factors in North Africa and Middle East countries

Location<br>Risk factor (alphabetic order)	Cause	DALYs (95% UI)<br>PAF (%)<br>2019	Rate per 100,000<br>2019	Rate change (%)<br>From 1990 to 2019	Deaths (95% UI)<br>PAF (%)<br>2019	Rate per 100,000<br>2019	Rate change (%)<br>From 1990 to 2019
	Alzheimer's disease and other dementias	23·4% (6·7–44·2%)	94·8 (18·8–285·2)	47·2% (25·7 to 81·8%)	23% (6·5–43·5%)	6·3 (0·8–22)	47·1% (22·5 to 87·5%)
High LDL cholesterol	Stroke	15·5% (7·5–28·1%)	145·6 (68·1–271·2)	-45·2% (-55·6 to -34%)	14·8% (3·9–32·9%)	7·8 (2–18·6)	-43·9% (-59·2 to -30·7%)
	Ischaemic stroke	23·7% (11·6–43%)	145·6 (68·1–271·2)	-45·2% (-55·6 to -34%)	20·8% (5·5–46·5%)	7·8 (2–18·6)	-43·9% (-59·2 to -30·7%)
High systolic blood pressure	Stroke	52·1% (43·5–61·1%)	490·3 (383·5–626·8)	-52·6% (-62·5 to -40·1%)	48·6% (38·1–60·2%)	25·6 (18·5–35·4)	-49·8% (-61·7 to -34·9%)
	Ischaemic stroke	49·4% (38·5–61·2%)	302·9 (225·9–407·5)	-44% (-55·7 to -29·8%)	45·7% (33–60·8%)	17·2 (11·5–24·9)	-42·4% (-56·6 to -25%)
	Intracerebral haemorrhage	58·1% (47·8–68·2%)	153·9 (113·9–199)	-63·9% (-72·1 to -52·3%)	55·7% (42–69·1%)	7·3 (5–10)	-61·5% (-70·8 to -49%)
	Subarachnoid haemorrhage	53·4% (44·5–62·3%)	33·6 (24–48·8)	-49·1% (-65·7 to -25·9%)	55·1% (43·6–66·8%)	1·2 (0·8–1·9)	-49·8% (-68·2 to -25%)
High temperature	Stroke	3·5% (0·6–6·1%)	33·4 (5·3–64)	-50·1% (-72·5 to -33·5%)	4·3% (0·7–7·6%)	2·3 (0·4–4·5)	-41·4% (-67·7 to -21·7%)
	Ischaemic stroke	3·3% (0·6–5·8%)	20·6 (3·4–40·3)	-40·6% (-68·3 to -20·8%)	4·3% (0·7–7·6%)	1·6 (0·3–3·2)	-33·4% (-63·8 to -11·7%)
	Intracerebral haemorrhage	3·9% (0·6–6·8%)	10·4 (1·6–19·9)	-61·3% (-78·6 to -46·4%)	4·3% (0·7–7·6%)	0·6 (0·1–1·1)	-55·9% (-74·9 to -38·9%)
	Subarachnoid haemorrhage	3·8% (0·6–6·5%)	2·4 (0·4–5·2)	-55·6% (-76·8 to -31·4%)	4·3% (0·7–7·6%)	0·1 (0–0·2)	-46·4% (-73·1 to -17·1%)
Household air pollution from solid fuels	Meningitis	0% (0–0%)	<0·1 (<0·1–<0·1)	-99·6% (-99·9 to -98·8%)	0% (0–0%)	<0·1 (<0·1–<0·1)	-99·6% (-99·9 to -98·8%)
	Encephalitis	0% (0–0%)	<0·1 (<0·1–<0·1)	-98·4% (-99·6 to -94·5%)	0% (0–0%)	<0·1 (<0·1–<0·1)	-98·4% (-99·6 to -94·5%)
	Stroke	0·1% (0–0·2%)	0·9 (0·3–2·1)	-98·5% (-99·4 to -96·4%)	0·1% (0–0·2%)	<0·1 (<0·1–0·1)	-98·4% (-99·4 to -96·4%)
	Ischaemic stroke	0·1% (0–0·2%)	0·5 (0·2–1·3)	-98·3% (-99·3 to -95·9%)	0·1% (0–0·2%)	<0·1 (<0·1–0·1)	-98·2% (-99·3 to -95·8%)
	Intracerebral haemorrhage	0·1% (0–0·2%)	0·3 (0·1–0·6)	-98·9% (-99·6 to -97·3%)	0·1% (0–0·2%)	<0·1 (<0·1–<0·1)	-98·8% (-99·5 to -97·2%)
	Subarachnoid haemorrhage	0·1% (0–0·2%)	0·1 (0–0·1)	-98·4% (-99·4 to -96·2%)	0·1% (0–0·2%)	<0·1 (<0·1–<0·1)	-98·5% (-99·5 to -96·2%)
Kidney dysfunction	Stroke	11·9% (8·6–14·7%)	111·6 (78·4–152·8)	-35·6% (-49·2 to -20·8%)	10·9% (6·5–15·1%)	5·8 (3·3–8·6)	-33% (-50·7 to -14·4%)
	Ischaemic stroke	13% (8·5–17%)	80·1 (50·8–112·6)	-26·5% (-42·4 to -10·1%)	10·7% (4·6–16%)	4 (1·7–6·6)	-26·4% (-49·5 to -5·9%)
	Intracerebral haemorrhage	11·9% (9·3–14·5%)	31·6 (22·6–42·2)	-50·9% (-62·8 to -35·3%)	13·4% (9·3–17·2%)	1·8 (1·2–2·5)	-44·4% (-58·4 to -27·4%)
Lead exposure	Stroke	2·2% (0·7–3·9%)	20·3 (6·2–38·2)	-61·6% (-70·9 to -52·2%)	2·3% (0·8–4%)	1·2 (0·4–2·3)	-52·3% (-62·7 to -39·3%)
	Ischaemic stroke	2·2% (0·7–3·9%)	13·2 (4·2–24·8)	-50·6% (-62·2 to -39·5%)	2·2% (0·8–4%)	0·8 (0·3–1·6)	-41·9% (-55·6 to -26%)
	Intracerebral haemorrhage	2·3% (0·6–4·4%)	6 (1·6–12·6)	-73·7% (-82·1 to -64·7%)	2·6% (0·8–4·9%)	0·3 (0·1–0·7)	-66·3% (-75·6 to -54·8%)
	Subarachnoid haemorrhage	1·8% (0·4–3·7%)	1·1 (0·2–2·5)	-67% (-82 to -50·7%)	2·4% (0·7–4·5%)	0·1 (0–0·1)	-58·6% (-76·1 to -37%)
Low birth weight	Meningitis	1·6% (1·1–2·2%)	0·3 (0·2–0·4)	-88% (-93·4 to -78·4%)	0·8% (0·5–1·1%)	<0·1 (<0·1–<0·1)	-88% (-93·4 to -78·4%)
	Encephalitis	0·9% (0·6–1·4%)	0·1 (0·1–0·2)	-49·7% (-77·5 to 2%)	0·6% (0·4–0·9%)	<0·1 (<0·1–<0·1)	-49·7% (-77·5 to 2%)
Low birth weight and short gestation	Meningitis	1·7% (1·2–2·4%)	0·3 (0·2–0·5)	-88·1% (-93·5 to -78·8%)	0·9% (0·6–1·2%)	<0·1 (<0·1–<0·1)	-88·1% (-93·5 to -78·8%)
	Encephalitis	1% (0·6–1·6%)	0·1 (0·1–0·2)	-49·5% (-77·1 to 2%)	0·6% (0·4–1%)	<0·1 (<0·1–<0·1)	-49·5% (-77·1 to 2%)
Low physical activity	Stroke	6% (1·3–12·3%)	56·4 (12·5–117·5)	-38·3% (-49·3 to -22·7%)	7·6% (1·8–14·5%)	4 (1–8)	-36% (-49·2 to -17·9%)
	Ischaemic stroke	9·2% (2·1–18·8%)	56·4 (12·5–117·5)	-38·3% (-49·3 to -22·7%)	10·7% (2·5–20·4%)	4 (1–8)	-36% (-49·2 to -17·9%)
Low temperature	Stroke	2·4% (-1·6–5·8%)	22·3 (-14·1–56·6)		2·9% (-1·9–7·1%)	1·5 (-1–4)	
	Ischaemic stroke	2·2% (-1·5–5·5%)	13·8 (-8·8–35·3)		2·9% (-1·9–7·1%)	1·1 (-0·7–2·8)	

Table S16 Regional and national disabilities and deaths related to neurological conditions attributable to risk factors in North Africa and Middle East countries

**Location**<br>Risk factor (alphabetic order)	Cause	**DALYs (95% UI)**<br>PAF (%)<br>2019	 Rate per 100,000<br>2019	 Rate change (%)<br>From 1990 to 2019	**Deaths (95% UI)**<br>PAF (%)<br>2019	 Rate per 100,000<br>2019	 Rate change (%)<br>From 1990 to 2019
	Intracerebral haemorrhage	2·6% (-1·8–6·4%)	6·9 (-4·5–17·7)		2·9% (-1·9–7·1%)	0·4 (-0·2–1)	
	Subarachnoid haemorrhage	2·5% (-1·7–6·3%)	1·6 (-1–4·2)		2·9% (-1·9–7·1%)	0·1 (0–0·2)	
Metabolic risk factors†	Stroke	75·3% (67·7–83·1%)	709·2 (584–883·9)	-51·3% (-60·6 to -40·6%)	71·5% (60·8–83·3%)	37·8 (29·2–49·2)	-48·5% (-59·3 to -35·8%)
	Ischaemic stroke	74·9% (64·6–86·1%)	459·8 (367·6–584·1)	-43·5% (-54 to -32%)	70·6% (56·8–86·5%)	26·5 (19·9–36·1)	-41·7% (-54·1 to -27·7%)
	Intracerebral haemorrhage	77·7% (70·2–84·4%)	205·8 (163·4–260·3)	-63·1% (-71·4 to -52·4%)	74·2% (63·6–83·5%)	9·7 (7·5–12·7)	-60·9% (-70·1 to -49·1%)
	Subarachnoid haemorrhage	69·6% (62·6–76·2%)	43·7 (32·8–62·6)	-48·5% (-65·5 to -25·5%)	70% (60·1–79·2%)	1·5 (1·1–2·4)	-49·8% (-68 to -26·4%)
	Neurological disorders*	11·9% (4·2–24·9%)	163·8 (51·4–416·8)	28·5% (9·2 to 56%)	27·9% (11·4–46·8%)	10·9 (1·9–32·9)	28·2% (6·1 to 62·2%)
	Alzheimer's disease and other dementias	40·5% (20·9–61·4%)	163·8 (51·4–416·8)	28·5% (9·2 to 56%)	39·6% (20·2–60·5%)	10·9 (1·9–32·9)	28·2% (6·1 to 62·2%)
Non-optimal temperature	Stroke	5·8% (2–9·3%)	54·8 (18–95·4)	-52·8% (-69·2 to -33%)	7·1% (2·5–11·4%)	3·8 (1·2–6·6)	-44·6% (-65 to -23·2%)
	Ischaemic stroke	5·5% (1·9–8·9%)	33·9 (11–59·4)	-43·8% (-63·7 to -21·6%)	7·1% (2·5–11·4%)	2·7 (0·9–4·7)	-37·1% (-60·3 to -11·9%)
	Intracerebral haemorrhage	6·4% (2·2–10·3%)	17 (5·6–29·5)	-63·4% (-76·1 to -45·5%)	7·1% (2·5–11·4%)	0·9 (0·3–1·6)	-58·3% (-72·1 to -39·5%)
	Subarachnoid haemorrhage	6·2% (2·1–10·1%)	3·9 (1·2–7·2)	-58·1% (-75·1 to -33·9%)	7·1% (2·5–11·4%)	0·2 (0–0·3)	-49·4% (-71 to -20·3%)
Other environmental risks	Stroke	2·2% (0·7–3·9%)	20·3 (6·2–38·2)	-61·6% (-70·9 to -52·2%)	2·3% (0·8–4%)	1·2 (0·4–2·3)	-52·3% (-62·7 to -39·3%)
	Ischaemic stroke	2·2% (0·7–3·9%)	13·2 (4·2–24·8)	-50·6% (-62·2 to -39·5%)	2·2% (0·8–4%)	0·8 (0·3–1·6)	-41·9% (-55·6 to -26%)
	Intracerebral haemorrhage	2·3% (0·6–4·4%)	6 (1·6–12·6)	-73·7% (-82·1 to -64·7%)	2·6% (0·8–4·9%)	0·3 (0·1–0·7)	-66·3% (-75·6 to -54·8%)
	Subarachnoid haemorrhage	1·8% (0·4–3·7%)	1·1 (0·2–2·5)	-67% (-82 to -50·7%)	2·4% (0·7–4·5%)	0·1 (0–0·1)	-58·6% (-76·1 to -37%)
Particulate matter pollution	Meningitis	0·4% (0·2–0·7%)	0·1 (0–0·1)	-88·8% (-95·5 to -71·5%)	0·2% (0·1–0·3%)	<0·1 (<0·1–<0·1)	-88·8% (-95·5 to -71·5%)
	Encephalitis	0·2% (0·1–0·4%)	<0·1 (<0·1–<0·1)	-52·7% (-83·4 to 28·6%)	0·1% (0·1–0·3%)	<0·1 (<0·1–<0·1)	-52·8% (-83·4 to 28·6%)
	Stroke	32·4% (28–36·5%)	305·3 (242·7–382·6)	-54·8% (-63·5 to -43·6%)	27·7% (23·9–31·5%)	14·6 (11·4–19·4)	-53·1% (-63·3 to -40·9%)
	Ischaemic stroke	30·8% (26·7–34·9%)	189·2 (151·9–238·4)	-46·7% (-56·8 to -35%)	26·3% (22·6–30·1%)	9·9 (7·7–13·2)	-45·9% (-58·5 to -31·7%)
	Intracerebral haemorrhage	35·5% (30·8–39·8%)	94·1 (73·7–121·5)	-65·9% (-73·8 to -55·2%)	30·7% (26·6–34·9%)	4 (3·1–5·3)	-64·7% (-73·1 to -53·1%)
	Subarachnoid haemorrhage	35·1% (30·6–39·3%)	22 (16·3–31·6)	-51·5% (-67·1 to -30·5%)	32·5% (28·2–36·5%)	0·7 (0·5–1·1)	-53·2% (-70 to -29·4%)
Secondhand smoke	Stroke	2·9% (2·2–3·6%)	27·4 (19·4–37·8)	-60% (-68·5 to -48·1%)	2·5% (1·9–3·2%)	1·3 (0·9–1·9)	-57·6% (-67·2 to -44·5%)
	Ischaemic stroke	2·6% (1·9–3·3%)	15·8 (11·3–22·1)	-52·6% (-62·3 to -39·9%)	2·3% (1·7–3%)	0·9 (0·6–1·3)	-50·9% (-62 to -35·9%)
	Intracerebral haemorrhage	3·5% (2·7–4·4%)	9·3 (6·5–12·6)	-68·9% (-76·5 to -58·3%)	2·9% (2·2–3·7%)	0·4 (0·3–0·5)	-67·6% (-75·2 to -56·7%)
	Subarachnoid haemorrhage	3·6% (2·7–4·5%)	2·2 (1·5–3·3)	-55·9% (-70·1 to -37%)	3·2% (2·5–4·1%)	0·1 (0–0·1)	-56·9% (-72·3 to -36·6%)
Short gestation	Meningitis	1·3% (0·9–1·8%)	0·2 (0·2–0·4)	-85·9% (-92·3 to -75·1%)	0·6% (0·4–0·9%)	<0·1 (<0·1–<0·1)	-85·9% (-92·3 to -75·1%)
	Encephalitis	0·8% (0·5–1·2%)	0·1 (0·1–0·1)	-40% (-72·8 to 20·7%)	0·5% (0·3–0·8%)	<0·1 (<0·1–<0·1)	-40% (-72·8 to 20·7%)
Smoking	Stroke	11·2% (9·6–13·1%)	105 (82·9–135·7)	-66·2% (-74·3 to -55·7%)	8·1% (6·9–9·6%)	4·3 (3·3–5·9)	-66% (-74·9 to -55·1%)
	Ischaemic stroke	10·2% (8·7–12%)	62·4 (49·4–80·6)	-59·4% (-68·8 to -47·6%)	7·2% (6–8·6%)	2·7 (2·1–3·8)	-59·3% (-69·9 to -46·4%)
	Intracerebral haemorrhage	13% (11·1–15·2%)	34·4 (26·5–45·8)	-74·8% (-81·6 to -65·6%)	10·1% (8·6–12%)	1·3 (1–1·8)	-74·8% (-81·8 to -65%)
	Subarachnoid haemorrhage	13·1% (10·7–15·8%)	8·2 (5·6–12·5)	-60·5% (-75·1 to -38%)	11·8% (9·5–14·2%)	0·3 (0·2–0·4)	-62·8% (-78·2 to -40·3%)

Table S16 Regional and national disabilities and deaths related to neurological conditions attributable to risk factors in North Africa and Middle East countries

Location<br>Risk factor (alphabetic order)	Cause	DALYs (95% UI)<br>PAF (%)<br>2019	Rate per 100,000<br>2019	Rate change (%)<br>From 1990 to 2019	Deaths (95% UI)<br>PAF (%)<br>2019	Rate per 100,000<br>2019	Rate change (%)<br>From 1990 to 2019
	Neurological disorders*	4·3% (1·5–9·3%)	59·1 (19·5–147·6)	-12·4% (-30·3 to 15·9%)	8·6% (2·3–14·9%)	3·5 (0·4–10·6)	-9·7% (-32·4 to 44·4%)
	Alzheimer's disease and other dementias	16·2% (9·7–22·5%)	65 (24·5–154·3)	-16·5% (-33·8 to 6·7%)	14·3% (8·5–20·3%)	3·9 (0·8–11)	-13·3% (-34·4 to 12·4%)
	Parkinson's disease	-8·2% (-12·5–-4·4%)	-7·3 (-11·8–-3·6)	-38·3% (-57·6 to -11·6%)	-7·4% (-11·4–-3·8%)	-0·4 (-0·7–-0·2)	-34·2% (-56·4 to -4·9%)
	Multiple sclerosis	10·7% (7·7–13·9%)	1·3 (0·8–2)	-4·7% (-30 to 22·4%)	11·1% (7·6–14·6%)	<0·1 (<0·1–<0·1)	-25·6% (-60·1 to 10%)
Tobacco	Stroke	13·7% (12·1–15·6%)	128·9 (103·8–164)	-64·9% (-72·6 to -55·1%)	10·4% (9–11·9%)	5·5 (4·3–7·5)	-64·1% (-72·6 to -53·7%)
	Ischaemic stroke	12·4% (10·9–14·3%)	76·4 (61·8–97·5)	-57·9% (-67·1 to -47%)	9·3% (7·9–10·7%)	3·5 (2·7–4·8)	-57·4% (-67·9 to -44·3%)
	Intracerebral haemorrhage	16% (14·1–18·3%)	42·4 (32·7–54·9)	-73·6% (-80·2 to -64·5%)	12·7% (11·1–14·6%)	1·7 (1·3–2·2)	-73·4% (-80·1 to -63·3%)
	Subarachnoid haemorrhage	16·1% (13·7–18·8%)	10·1 (7·1–15·2)	-59·4% (-74 to -37·6%)	14·6% (12·3–17%)	0·3 (0·2–0·5)	-61·5% (-77 to -39·3%)
	Neurological disorders*	4·3% (1·5–9·3%)	59·1 (19·5–147·6)	-12·4% (-30·3 to 15·9%)	8·6% (2·3–14·9%)	3·5 (0·4–10·6)	-9·7% (-32·4 to 44·4%)
	Alzheimer's disease and other dementias	16·2% (9·7–22·5%)	65 (24·5–154·3)	-16·5% (-33·8 to 6·7%)	14·3% (8·5–20·3%)	3·9 (0·8–11)	-13·3% (-34·4 to 12·4%)
	Parkinson's disease	-8·2% (-12·5–-4·4%)	-7·3 (-11·8–-3·6)	-38·3% (-57·6 to -11·6%)	-7·4% (-11·4–-3·8%)	-0·4 (-0·7–-0·2)	-34·2% (-56·4 to -4·9%)
	Multiple sclerosis	10·7% (7·7–13·9%)	1·3 (0·8–2)	-4·7% (-30 to 22·4%)	11·1% (7·6–14·6%)	<0·1 (<0·1–<0·1)	-25·6% (-60·1 to 10%)
**Egypt**							
Air pollution	Meningitis	2·2% (1–4·1%)	1·2 (0·5–2·5)	-83·7% (-94·2 to -63·3%)	1·6% (0·7–3·1%)	<0·1 (<0·1–<0·1)	-83·7% (-94·2 to -63·3%)
	Encephalitis	1·6% (0·5–3·3%)	1 (0·2–2·4)	-29·8% (-76·3 to 74%)	1·2% (0·3–2·7%)	<0·1 (<0·1–<0·1)	-29·8% (-76·3 to 74%)
	Stroke	34·3% (29·1–38·6%)	734·1 (528·3–1009·9)	-14·4% (-34·7 to 10%)	32·5% (27·7–36·7%)	27·9 (19·3–40)	-15·7% (-38·2 to 12%)
	Ischaemic stroke	35·3% (30·1–39·5%)	487·6 (355·3–656·1)	32·7% (-2·5 to 67·6%)	31·9% (27·2–36%)	19·9 (13·8–28·2)	22·9% (-13·3 to 62·4%)
	Intracerebral haemorrhage	33·5% (28–38·9%)	215·8 (143·8–324·5)	-48·4% (-64·2 to -26·5%)	34·7% (29·8–39·1%)	7·2 (4·7–11)	-51·3% (-67 to -29%)
	Subarachnoid haemorrhage	26·9% (19·9–35%)	30·7 (20–44·9)	-57·5% (-74·9 to -23·3%)	31% (24·9–36·5%)	0·9 (0·5–1·3)	-60·6% (-78 to -22·4%)
Alcohol use	Stroke	0·1% (-0·1–0·4%)	2·4 (-2·6–8·9)	-55·1% (-397·6 to 223·5%)	0·1% (-0·1–0·3%)	0 (-0·1–0·2)	-69·3% (-538·7 to 272·6%)
	Ischaemic stroke	-0·1% (-0·4–0·1%)	-1·6 (-5·1–2)	27·1% (-456·2 to 547·2%)	-0·1% (-0·3–0·1%)	-0·1 (-0·2–0·1)	18·1% (-398·4 to 531·7%)
	Intracerebral haemorrhage	0·6% (0·1–1·3%)	4 (0·6–8·9)	-39·5% (-80 to 68·6%)	0·6% (0·1–1·1%)	0·1 (0–0·3)	-43% (-80·9 to 66·5%)
	Neurological disorders*	0·1% (0–0·2%)	1 (0·3–2·4)	-3·5% (-66·4 to 165%)	0% (0–0·1%)	<0·1 (<0·1–<0·1)	-6·6% (-52·2 to 82·5%)
	Idiopathic epilepsy	1% (0·4–1·7%)	1 (0·3–2·4)	-3·5% (-66·4 to 165%)	1·4% (0·6–2·4%)	<0·1 (<0·1–<0·1)	-6·6% (-52·2 to 82·5%)
All risk factors	Meningitis	9·5% (4·7–16·6%)	5·2 (2·1–9·9)	-83·9% (-93·8 to -65·2%)	6·9% (3·2–12·5%)	0·1 (0–0·1)	-83·9% (-93·8 to -65·2%)
	Encephalitis	6·8% (2·2–13·8%)	4·4 (0·8–10·1)	-29·6% (-73 to 62·2%)	5·1% (1·3–10·6%)	<0·1 (<0·1–0·1)	-29·6% (-73 to 62·2%)
	Stroke	82% (76·6–87%)	1755 (1315–2369·7)	-9·2% (-30·9 to 17·3%)	84·4% (79·3–89·3%)	72·4 (51·3–101·5)	-9·3% (-33·7 to 21·7%)
	Ischaemic stroke	86·4% (81·7–90·8%)	1194·4 (912·6–1589·3)	37·5% (1·1 to 75·1%)	85·4% (79·4–91·9%)	53·2 (37·7–74)	27·9% (-8·9 to 69·3%)
	Intracerebral haemorrhage	76·3% (68·7–83·9%)	491·4 (342·9–709·9)	-45·5% (-61·8 to -23·7%)	83·1% (78·4–87·8%)	17·2 (11·8–26)	-48·4% (-65·2 to -25%)
	Subarachnoid haemorrhage	60·4% (48·4–74·1%)	69·2 (46·9–98·9)	-57·6% (-74·8 to -23·6%)	72·5% (63·4–81·7%)	2 (1·3–2·9)	-58·6% (-76·6 to -19·8%)
	Neurological disorders*	11·5% (4·4–23·7%)	150·8 (55·3–362·5)	38·7% (15·7 to 70·7%)	27·7% (11·9–43·1%)	9·5 (1·7–27·8)	39·4% (9·4 to 80·6%)

Table S16 Regional and national disabilities and deaths related to neurological conditions attributable to risk factors in North Africa and Middle East countries

**Location**<br>Risk factor (alphabetic order)	Cause	**DALYs (95% UI)**<br>PAF (%)<br>2019	Rate per 100,000<br>2019	Rate change (%)<br>From 1990 to 2019	**Deaths (95% UI)**<br>PAF (%)<br>2019	Rate per 100,000<br>2019	Rate change (%)<br>From 1990 to 2019
	Alzheimer's disease and other dementias	43·8% (30·4–57·3%)	158·8 (63·2–371·8)	39% (15·6 to 70·9%)	42·6% (29·4–55·8%)	10 (2·2–28·2)	39·6% (10·5 to 80·1%)
	Parkinson's disease	-10·7% (-16·1–-5·5%)	-10·5 (-17·2–-5·1)	36·2% (0·6 to 80·7%)	-9·9% (-15–-5%)	-0·6 (-1–-0·3)	42·1% (1·1 to 92·7%)
	Idiopathic epilepsy	1% (0·4–1·7%)	1 (0·3–2·4)	-3·5% (-66·4 to 165%)	1·4% (0·6–2·4%)	<0·1 (<0·1–<0·1)	-6·6% (-52·2 to 82·5%)
	Multiple sclerosis	10·3% (3·6–17·8%)	1·5 (0·8–3·3)	30·4% (-7 to 67·5%)	12·1% (4·1–20·6%)	<0·1 (<0·1–0·1)	26·3% (-22 to 86·5%)
Ambient particulate matter pollution	Meningitis	2·2% (1–4·1%)	1·2 (0·5–2·5)	-80·5% (-93·1 to -54%)	1·6% (0·7–3·1%)	<0·1 (<0·1–<0·1)	-80·5% (-93·1 to -53·9%)
	Encephalitis	1·6% (0·5–3·3%)	1 (0·2–2·4)	-16% (-71·4 to 116·5%)	1·2% (0·3–2·7%)	<0·1 (<0·1–<0·1)	-16% (-71·4 to 116·6%)
	Stroke	34·3% (29·1–38·6%)	733·5 (527·3–1009)	-0·7% (-24·8 to 32·4%)	32·5% (27·7–36·7%)	27·9 (19·2–39·9)	-2·1% (-28·5 to 35·2%)
	Ischaemic stroke	35·2% (30–39·5%)	487·2 (354·9–655·6)	54·1% (14 to 103·2%)	31·9% (27·2–36%)	19·9 (13·8–28·1)	42·8% (1·6 to 93·8%)
	Intracerebral haemorrhage	33·5% (28–38·8%)	215·6 (143·7–324·3)	-40·2% (-58·8 to -12·4%)	34·6% (29·8–39·1%)	7·2 (4·7–11)	-43·5% (-62·3 to -15·7%)
	Subarachnoid haemorrhage	26·8% (19·9–35%)	30·7 (20–44·9)	-50·6% (-71·1 to -8·9%)	31% (24·8–36·5%)	0·9 (0·5–1·3)	-54·2% (-74·7 to -9·3%)
Behavioural risk factors‡	Meningitis	9·2% (4·5–16%)	5 (2·1–9·6)	-83·7% (-93·7 to -65·1%)	6·7% (3·1–12%)	0·1 (0–0·1)	-83·7% (-93·7 to -65%)
	Encephalitis	6·6% (2·2–13·4%)	4·3 (0·8–9·7)	-28·8% (-72·2 to 63·4%)	4·9% (1·3–10·2%)	<0·1 (<0·1–0·1)	-28·8% (-72·2 to 63·4%)
	Stroke	29·2% (24·5–35%)	626·7 (445·5–906·2)	-10·9% (-32·9 to 16·4%)	28·3% (23·2–34·2%)	24·3 (16·6–36·6)	-11·7% (-35·4 to 18·2%)
	Ischaemic stroke	32·1% (25·8–39%)	443·6 (309·6–630·1)	33·2% (-4 to 72·5%)	29·7% (23–37·3%)	18·5 (12·3–27·7)	22·8% (-14·7 to 62·7%)
	Intracerebral haemorrhage	25% (20–30·7%)	161·4 (106–249·7)	-49·4% (-65·4 to -26·8%)	25% (20·3–30·4%)	5·2 (3·3–8·3)	-52·4% (-68·7 to -30·2%)
	Subarachnoid haemorrhage	18·9% (13·9–26%)	21·7 (13·6–34)	-58·1% (-75·1 to -21·7%)	21·6% (16·9–27·5%)	0·6 (0·4–1)	-61% (-78 to -24%)
	Neurological disorders*	5·2% (1·7–11·4%)	68·6 (22·3–171·1)	35·1% (10·9 to 63·3%)	12·6% (3·3–21·2%)	4·4 (0·5–13·4)	40·5% (6·5 to 83·6%)
	Alzheimer's disease and other dementias	21·2% (13·7–27·8%)	76·6 (30·5–179·4)	36·1% (12 to 65·5%)	21·1% (13·4–28·2%)	5 (1·1–14·1)	40·8% (9·5 to 81·1%)
	Parkinson's disease	-10·7% (-16·1–-5·5%)	-10·5 (-17·2–-5·1)	36·2% (0·6 to 80·7%)	-9·9% (-15–-5%)	-0·6 (-1–-0·3)	42·1% (1·1 to 92·7%)
	Idiopathic epilepsy	1% (0·4–1·7%)	1 (0·3–2·4)	-3·5% (-66·4 to 165%)	1·4% (0·6–2·4%)	<0·1 (<0·1–<0·1)	-6·6% (-52·2 to 82·5%)
	Multiple sclerosis	10·3% (3·6–17·8%)	1·5 (0·8–3·3)	30·4% (-7 to 67·5%)	12·1% (4·1–20·6%)	<0·1 (<0·1–0·1)	26·3% (-22 to 86·5%)
Child and maternal malnutrition	Meningitis	9·2% (4·5–16%)	5 (2·1–9·6)	-83·7% (-93·7 to -65·1%)	6·7% (3·1–12%)	0·1 (0–0·1)	-83·7% (-93·7 to -65%)
	Encephalitis	6·6% (2·2–13·4%)	4·3 (0·8–9·7)	-28·8% (-72·2 to 63·4%)	4·9% (1·3–10·2%)	<0·1 (<0·1–0·1)	-28·8% (-72·2 to 63·4%)
Diet high in red meat	Stroke	4·2% (1·6–6·2%)	89·6 (32·9–150·7)	8% (-33·1 to 78·2%)	3·4% (1·3–5·1%)	2·9 (1–5·1)	4·1% (-38·3 to 67%)
	Ischaemic stroke	4% (0·7–6·1%)	55·1 (8·5–96)	80% (30 to 167·1%)	3% (0·5–4·7%)	1·9 (0·3–3·4)	64·3% (13·5 to 140·9%)
	Intracerebral haemorrhage	4·6% (0·7–7·4%)	29·9 (4·7–56·8)	-32·3% (-55·9 to 11·7%)	4·4% (0·7–7%)	0·9 (0·1–1·8)	-36·1% (-59·7 to 5·3%)
	Subarachnoid haemorrhage	4% (0·6–6·9%)	4·5 (0·7–8·4)	-44·3% (-67·3 to 17·8%)	4·1% (0·7–6·8%)	0·1 (0–0·2)	-48·6% (-71·7 to 12·6%)
Diet high in sodium	Stroke	1·8% (0·2–7·2%)	39·6 (4·7–160·3)	-12·2% (-50·7 to 56·9%)	1·7% (0·3–6·7%)	1·5 (0·2–6·1)	-13·5% (-51·7 to 53·8%)
	Ischaemic stroke	1·8% (0·2–7·4%)	25·2 (3·2–106·2)	38·6% (-26·7 to 152%)	1·6% (0·3–6·4%)	1 (0·1–4·3)	30·1% (-31·3 to 123·6%)
	Intracerebral haemorrhage	2% (0·2–7·7%)	12·7 (1·2–51·3)	-45·3% (-71·4 to 9·1%)	2% (0·2–7·9%)	0·4 (0–1·8)	-48·5% (-74·3 to -4·2%)
	Subarachnoid haemorrhage	1·5% (0·2–5·8%)	1·7 (0·2–7·1)	-53·6% (-78 to 10·9%)	1·8% (0·2–7·1%)	<0·1 (<0·1–0·2)	-57% (-80·2 to 6·9%)

Table S16 Regional and national disabilities and deaths related to neurological conditions attributable to risk factors in North Africa and Middle East countries

**Location**<br>Risk factor (alphabetic order)	Cause	**DALYs (95% UI)**<br>PAF (%)<br>2019	Rate per 100,000<br>2019	Rate change (%)<br>From 1990 to 2019	**Deaths (95% UI)**<br>PAF (%)<br>2019	Rate per 100,000<br>2019	Rate change (%)<br>From 1990 to 2019
Diet low in fibre	Stroke	0·4% (0·2–0·7%)	7·5 (3·9–15·4)	-55·6% (-70·2 to -21·1%)	0·4% (0·2–0·7%)	0·3 (0·2–0·6)	-55% (-72·1 to -18·9%)
	Ischaemic stroke	0·4% (0·2–0·7%)	5 (2·7–9·7)	-29·1% (-53·6 to 20·4%)	0·4% (0·2–0·6%)	0·2 (0·1–0·4)	-34·6% (-59·6 to 14·9%)
	Intracerebral haemorrhage	0·3% (0·2–0·7%)	2·2 (1–5·1)	-73·6% (-83·7 to -51·5%)	0·4% (0·2–0·7%)	0·1 (0–0·2)	-74·8% (-84·9 to -53·1%)
	Subarachnoid haemorrhage	0·3% (0·1–0·7%)	0·3 (0·1–0·8)	-78·5% (-87·9 to -51·8%)	0·3% (0·2–0·7%)	<0·1 (<0·1–<0·1)	-79·1% (-88·1 to -49·6%)
Diet low in fruits	Stroke	2·5% (1·2–4·1%)	54·3 (24–97)	-59·5% (-75·1 to -34·6%)	2% (0·9–3·4%)	1·8 (0·8–3·2)	-62·5% (-78·1 to -38·9%)
	Ischaemic stroke	2% (0·4–4%)	28·2 (5·6–59·1)	-33·2% (-59·3 to 13·8%)	1·6% (0·4–3·2%)	1 (0·2–2·2)	-39·9% (-65·4 to 6·2%)
	Intracerebral haemorrhage	3·5% (1·7–5·9%)	22·4 (9·8–42·8)	-71·1% (-83·2 to -51·7%)	3·2% (1·6–5·2%)	0·7 (0·3–1·3)	-74·6% (-85·5 to -55·6%)
	Subarachnoid haemorrhage	3·2% (1·5–5·7%)	3·7 (1·6–7·2)	-73·9% (-86·8 to -49·4%)	3·2% (1·6–5·4%)	0·1 (0–0·2)	-77·9% (-89·1 to -54%)
Diet low in vegetables	Stroke	0·2% (0·2–0·2%)	4 (3–5·3)	-56·1% (-80·7 to -8·4%)	0·2% (0·2–0·3%)	0·2 (0·1–0·3)	-60·6% (-81 to -15·1%)
	Ischaemic stroke	0·2% (0·2–0·2%)	2·8 (2·1–3·7)	-35·5% (-69·2 to 39·4%)	0·2% (0·2–0·3%)	0·1 (0·1–0·2)	-45·8% (-73·8 to 27·6%)
	Intracerebral haemorrhage	0·2% (0·1–0·2%)	1 (0·7–1·5)	-74·2% (-91·7 to -35%)	0·2% (0·2–0·3%)	<0·1 (<0·1–0·1)	-77·9% (-92·5 to -39%)
	Subarachnoid haemorrhage	0·1% (0·1–0·2%)	0·1 (0·1–0·2)	-79·1% (-93·9 to -32·4%)	0·2% (0·1–0·2%)	<0·1 (<0·1–<0·1)	-81·5% (-94·3 to -34·7%)
Diet low in whole grains	Stroke	3·4% (0·8–5%)	71·7 (17·5–120·5)	30·4% (-7·1 to 66·8%)	3·1% (0·8–4·7%)	2·7 (0·7–4·7)	19·7% (-15·3 to 58·6%)
	Ischaemic stroke	5·2% (1·3–7·6%)	71·7 (17·5–120·5)	30·4% (-7·1 to 66·8%)	4·3% (1·1–6·4%)	2·7 (0·7–4·7)	19·7% (-15·3 to 58·6%)
Dietary risks	Stroke	11·7% (7·4–17%)	250·3 (145·1–390·7)	-22·1% (-46·7 to 6·3%)	10·3% (6·5–15·3%)	8·9 (5–15)	-25·6% (-51·1 to 3·6%)
	Ischaemic stroke	12·7% (7·2–18·1%)	175·7 (93·6–280·9)	20·2% (-18·1 to 60·8%)	10·6% (6·1–15·5%)	6·6 (3·4–11·1)	8·5% (-27·8 to 47·7%)
	Intracerebral haemorrhage	10·1% (5·1–15·7%)	64·8 (29·8–117·3)	-56·4% (-73·3 to -33·4%)	9·7% (5–15·5%)	2 (0·9–3·7)	-60·2% (-77·2 to -37·7%)
	Subarachnoid haemorrhage	8·6% (4·3–13·8%)	9·9 (4·6–17·1)	-62·9% (-79·3 to -31·7%)	9·1% (4·7–14·3%)	0·3 (0·1–0·5)	-66·8% (-83 to -33·1%)
Environmental/occupational risk factors§	Meningitis	2·2% (1–4·1%)	1·2 (0·5–2·5)	-83·7% (-94·2 to -63·3%)	1·6% (0·7–3·1%)	<0·1 (<0·1–<0·1)	-83·7% (-94·2 to -63·3%)
	Encephalitis	1·6% (0·5–3·3%)	1 (0·2–2·4)	-29·8% (-76·3 to 74%)	1·2% (0·3–2·7%)	<0·1 (<0·1–<0·1)	-29·8% (-76·3 to 74%)
	Stroke	41% (35·7–45·5%)	878·3 (641·4–1202·4)	-18% (-37·5 to 6·1%)	40·4% (35·6–44·9%)	34·7 (24·4–49·4)	-15·6% (-37·5 to 13·5%)
	Ischaemic stroke	42·1% (36·7–46·6%)	581·6 (429·9–792·3)	30·6% (-3·9 to 65·8%)	39·8% (34·9–44·4%)	24·8 (17·4–35)	24% (-12·2 to 64·1%)
	Intracerebral haemorrhage	40·2% (34·3–45·9%)	259 (180·6–384·3)	-50·6% (-65 to -30·8%)	42·4% (37·2–46·9%)	8·8 (5·8–13·5)	-51·8% (-67·3 to -30·2%)
	Subarachnoid haemorrhage	33% (25·4–41·7%)	37·7 (25·2–54·9)	-63·1% (-77·4 to -33·2%)	38·3% (31·7–44·7%)	1·1 (0·7–1·6)	-62·4% (-78·4 to -28·5%)
High body-mass index	Stroke	38·7% (27·8–47·8%)	829 (545·9–1208·4)	6·9% (-21·9 to 44·8%)	29·1% (20–38%)	25 (14·9–38·6)	7·7% (-25·1 to 51·4%)
	Ischaemic stroke	33·9% (23·4–43·5%)	468·8 (302·7–687·4)	95·1% (39·5 to 167·3%)	24·2% (15·6–32·9%)	15 (8·8–23·6)	88·8% (27·4 to 175·1%)
	Intracerebral haemorrhage	48·6% (36·6–59·5%)	312·7 (200·2–475·2)	-30·3% (-52·5 to 5·4%)	42·7% (30·8–53·8%)	8·8 (5·4–14)	-32·5% (-55·8 to 5·5%)
	Subarachnoid haemorrhage	41·5% (29·4–53·9%)	47·5 (29·4–72·4)	-44·9% (-68·4 to 4·8%)	40·1% (28·9–51·4%)	1·1 (0·6–1·8)	-48·4% (-72·1 to 4·1%)
	Neurological disorders*	5·5% (1·6–12·4%)	71·1 (19–174)	44·7% (9·7 to 120%)	12·9% (4·1–24·5%)	4·3 (0·6–13)	42·2% (-1·8 to 135·4%)
	Alzheimer's disease and other dementias	19·6% (8·3–34·3%)	71·1 (19–174)	44·7% (9·7 to 120%)	18·3% (7·3–32·6%)	4·3 (0·6–13)	42·2% (-1·8 to 135·4%)
High fasting plasma glucose	Stroke	24·6% (15–39·5%)	526·7 (295·2–920·9)	132·5% (50·4 to 233·2%)	27·6% (16·1–47·9%)	23·8 (12·2–44·2)	107·1% (31·9 to 205·1%)
	Ischaemic stroke	28·1% (14·3–50·5%)	388·9 (186·9–733·8)	219·1% (105·1 to 359·6%)	28·9% (14·1–57·3%)	18·1 (7·8–37·5)	170% (74·2 to 313·4%)

Table S16 Regional and national disabilities and deaths related to neurological conditions attributable to risk factors in North Africa and Middle East countries

Location<br>Risk factor (alphabetic order)	Cause	DALYs (95% UI)<br>PAF (%)<br>2019	Rate per 100,000<br>2019	Rate change (%)<br>From 1990 to 2019	Deaths (95% UI)<br>PAF (%)<br>2019	Rate per 100,000<br>2019	Rate change (%)<br>From 1990 to 2019
	Intracerebral haemorrhage	19·2% (11–29·8%)	124 (62·6–227·7)	35·8% (-15·2 to 110·3%)	24·8% (14·4–38·5%)	5·2 (2·5–10)	22·4% (-26 to 91·8%)
	Subarachnoid haemorrhage	12·1% (6·7–19·2%)	13·8 (6·9–24)	3% (-46·1 to 116·4%)	19·5% (11·5–31%)	0·5 (0·3–1)	-4·5% (-51·1 to 112·2%)
	Neurological disorders*	3% (0·5–8·4%)	38·5 (6·7–124·7)	93·9% (59·1 to 165·6%)	7·3% (1·4–17·2%)	2·5 (0·3–9·4)	89·3% (45·4 to 171·5%)
	Alzheimer's disease and other dementias	10·6% (2·5–22·9%)	38·5 (6·7–124·7)	93·9% (59·1 to 165·6%)	10·4% (2·4–22·6%)	2·5 (0·3–9·4)	89·3% (45·4 to 171·5%)
High LDL cholesterol	Stroke	15·3% (9·7–23·9%)	325·4 (192·5–531·1)	46·2% (9·2 to 91%)	14·3% (6·6–27·3%)	12·2 (5·3–23·6)	32·1% (-4 to 85·1%)
	Ischaemic stroke	23·6% (15·1–37%)	325·4 (192·5–531·1)	46·2% (9·2 to 91%)	19·7% (9–37·8%)	12·2 (5·3–23·6)	32·1% (-4 to 85·1%)
High systolic blood pressure	Stroke	49·9% (42–56·6%)	1068·5 (757·4–1481·4)	3·2% (-24·4 to 37·1%)	49·2% (40–57·2%)	42·2 (28·2–61·3)	-1·6% (-32·8 to 37·4%)
	Ischaemic stroke	51% (40·9–59·5%)	704·8 (490·5–973·7)	59% (13·3 to 113·7%)	47·6% (36·4–58·2%)	29·7 (19·3–43·6)	43·4% (-2·2 to 100·7%)
	Intracerebral haemorrhage	49·7% (40·8–58·6%)	320·2 (216·6–473)	-37·1% (-57·2 to -8·3%)	54·3% (44·9–63·2%)	11·3 (7·4–17·1)	-42·2% (-62·4 to -13·4%)
	Subarachnoid haemorrhage	38% (27·4–50·2%)	43·5 (28·5–64·7)	-47·8% (-69·6 to -3·3%)	47% (36·8–57·8%)	1·3 (0·8–2)	-52·6% (-74·4 to -4·8%)
High temperature	Stroke	1·8% (0·3–4·6%)	39·7 (5·6–100·1)	-13·3% (-80·4 to 86·7%)	2·1% (0·3–5·3%)	1·8 (0·3–4·7)	10·9% (-74·5 to 142·7%)
	Ischaemic stroke	1·8% (0·2–4·4%)	24·4 (3·5–61·4)	59·1% (-65·7 to 265·1%)	2·1% (0·3–5·3%)	1·3 (0·2–3·4)	65·2% (-61·4 to 269·3%)
	Intracerebral haemorrhage	2% (0·3–5%)	13 (1·8–33·2)	-43·7% (-88·1 to 24·3%)	2·1% (0·3–5·3%)	0·4 (0·1–1·2)	-37·1% (-87·8 to 36·2%)
	Subarachnoid haemorrhage	2% (0·3–4·9%)	2·3 (0·3–5·8)	-69% (-93·4 to -17·6%)	2·1% (0·3–5·3%)	0·1 (0–0·1)	-58·6% (-90·1 to 0·5%)
Household air pollution from solid fuels	Meningitis	0% (0–0%)	<0·1 (<0·1–<0·1)	-99·9% (-100 to -99·7%)	0% (0–0%)	<0·1 (<0·1–<0·1)	-99·9% (-100 to -99·7%)
	Encephalitis	0% (0–0%)	<0·1 (<0·1–<0·1)	-99·6% (-99·9 to -98·6%)	0% (0–0%)	<0·1 (<0·1–<0·1)	-99·6% (-99·9 to -98·6%)
	Stroke	0% (0–0·1%)	0·6 (0·2–1·4)	-99·5% (-99·8 to -98·7%)	0% (0–0·1%)	<0·1 (<0·1–0·1)	-99·5% (-99·8 to -98·8%)
	Ischaemic stroke	0% (0–0·1%)	0·4 (0·1–0·9)	-99·2% (-99·7 to -98·1%)	0% (0–0·1%)	<0·1 (<0·1–<0·1)	-99·3% (-99·7 to -98·2%)
	Intracerebral haemorrhage	0% (0–0·1%)	0·2 (0·1–0·4)	-99·7% (-99·9 to -99·2%)	0% (0–0·1%)	<0·1 (<0·1–<0·1)	-99·7% (-99·9 to -99·3%)
	Subarachnoid haemorrhage	0% (0–0·1%)	<0·1 (<0·1–0·1)	-99·7% (-99·9 to -99·3%)	0% (0–0·1%)	<0·1 (<0·1–<0·1)	-99·8% (-99·9 to -99·3%)
Kidney dysfunction	Stroke	11·1% (8·9–13·4%)	237·5 (167·1–335·2)	34·9% (0·4 to 75·2%)	11·7% (8·4–14·9%)	10 (6·3–15·1)	30·1% (-8 to 75·7%)
	Ischaemic stroke	13·3% (10·2–16·4%)	184·5 (127·2–262·2)	87·6% (37·8 to 138·1%)	12·5% (8·2–16·5%)	7·8 (4·7–12)	77·9% (23·9 to 136·8%)
	Intracerebral haemorrhage	8·2% (6·4–10·3%)	53 (33·6–83)	-31·9% (-53·4 to -1·3%)	10·8% (8·3–13·3%)	2·2 (1·4–3·6)	-32·7% (-55·3 to 0·8%)
Lead exposure	Stroke	5·5% (3·5–7·7%)	118·1 (65·7–186·6)	-25·9% (-45·2 to -2·3%)	6% (3·9–8·3%)	5·2 (2·9–8·3)	-16·7% (-40·8 to 13·3%)
	Ischaemic stroke	5·8% (3·7–8·2%)	80·6 (45·7–126·7)	25·7% (-9·4 to 61·9%)	6% (3·8–8·3%)	3·7 (2–6)	31·1% (-10·2 to 74·3%)
	Intracerebral haemorrhage	5·2% (3–7·8%)	33·4 (17·2–58·7)	-59·3% (-72·9 to -41·2%)	6·4% (3·9–9·1%)	1·3 (0·7–2·3)	-55·6% (-70·3 to -34·9%)
	Subarachnoid haemorrhage	3·5% (1·8–5·9%)	4·1 (1·9–7·6)	-69·5% (-82·7 to -41·9%)	5·2% (3–7·8%)	0·1 (0·1–0·3)	-65·4% (-80·7 to -31%)
Low birth weight	Meningitis	8·9% (4·4–15·5%)	4·8 (2–9·3)	-83·7% (-93·7 to -64·6%)	6·4% (3–11·7%)	0·1 (0–0·1)	-83·7% (-93·7 to -64·6%)
	Encephalitis	6·3% (2·1–12·9%)	4·1 (0·7–9·4)	-29% (-72·3 to 64·3%)	4·7% (1·2–9·9%)	<0·1 (<0·1–0·1)	-29% (-72·3 to 64·3%)
Low birth weight and short gestation	Meningitis	9·2% (4·5–16%)	5 (2·1–9·6)	-83·7% (-93·7 to -65·1%)	6·7% (3·1–12%)	0·1 (0–0·1)	-83·7% (-93·7 to -65%)
	Encephalitis	6·6% (2·2–13·4%)	4·3 (0·8–9·7)	-28·8% (-72·2 to 63·4%)	4·9% (1·3–10·2%)	<0·1 (<0·1–0·1)	-28·8% (-72·2 to 63·4%)
Low physical activity	Stroke	4·7% (1–10·8%)	100 (17·4–238·4)	35·8% (-1·8 to 74·6%)	6·2% (1·3–13·1%)	5·3 (1·1–12·2)	22·8% (-12·6 to 63·7%)

Table S16 Regional and national disabilities and deaths related to neurological conditions attributable to risk factors in North Africa and Middle East countries

Location<br>Risk factor (alphabetic order)	Cause	DALYs (95% UI)<br>PAF (%)<br>2019	Rate per 100,000<br>2019	Rate change (%)<br>From 1990 to 2019	Deaths (95% UI)<br>PAF (%)<br>2019	Rate per 100,000<br>2019	Rate change (%)<br>From 1990 to 2019
	Ischaemic stroke	7·2% (1·5–16·7%)	100 (17·4–238·4)	35·8% (-1·8 to 74·6%)	8·6% (1·8–18%)	5·3 (1·1–12·2)	22·8% (-12·6 to 63·7%)
Low temperature	Stroke	3·5% (-0·1–7·6%)	75·6 (-1·8–172)	-38·7% (-68·7 to -7·9%)	4·1% (-0·1–8·8%)	3·5 (-0·1–8·1)	-21·5% (-61·8 to 17·9%)
	Ischaemic stroke	3·4% (-0·1–7·3%)	46·5 (-1·1–104·7)	12·6% (-47·3 to 65·6%)	4·1% (-0·1–8·8%)	2·5 (-0·1–5·9)	16·9% (-46·6 to 71%)
	Intracerebral haemorrhage	3·9% (-0·1–8·3%)	24·7 (-0·6–57·8)	-60·2% (-80·5 to -37·7%)	4·1% (-0·1–8·8%)	0·8 (0–2)	-55·5% (-78·2 to -30·5%)
	Subarachnoid haemorrhage	3·7% (-0·1–8·1%)	4·3 (-0·1–9·8)	-78·4% (-89·5 to -51%)	4·1% (-0·1–8·8%)	0·1 (0–0·3)	-71% (-85·9 to -40·6%)
Metabolic risk factors†	Stroke	70·8% (63·6–77·6%)	1516·3 (1124·2–2070·8)	5·2% (-22 to 37·8%)	71% (62·6–79·8%)	60·9 (42–86·6)	2·6% (-26·9 to 38·9%)
	Ischaemic stroke	74·6% (66·7–83·1%)	1031·9 (775·4–1392·7)	55·3% (14·6 to 100·9%)	71·3% (60·9–83·8%)	44·4 (30·4–63·9)	43·5% (1 to 93·9%)
	Intracerebral haemorrhage	66·2% (57·8–74·5%)	426·5 (293–625·4)	-36·1% (-56·1 to -8·2%)	71·5% (64·3–78·5%)	14·8 (9·7–22·6)	-40·6% (-60·6 to -12·9%)
	Subarachnoid haemorrhage	50·6% (38·1–64·2%)	58 (37·8–85·4)	-47·1% (-69·4 to -2·6%)	60·4% (50·2–70·6%)	1·7 (1–2·5)	-51·2% (-73·2 to -2·2%)
	Neurological disorders*	7·8% (2·4–16·9%)	101·9 (30·8–248·9)	54·1% (20·1 to 113·2%)	18·8% (7·1–33·6%)	6·3 (1·1–18·9)	51·8% (10·4 to 121·4%)
	Alzheimer's disease and other dementias	28·1% (13·7–44·9%)	101·9 (30·8–248·9)	54·1% (20·1 to 113·2%)	26·7% (12·6–43·3%)	6·3 (1·1–18·9)	51·8% (10·4 to 121·4%)
Non-optimal temperature	Stroke	5·3% (2·4–8·9%)	113·9 (48·2–206·8)	-32% (-56·3 to 7·9%)	6·1% (2·8–10·3%)	5·3 (2·2–9·5)	-13% (-48·2 to 39·2%)
	Ischaemic stroke	5·1% (2·2–8·6%)	70·1 (29·2–125·6)	24·8% (-27·1 to 100%)	6·1% (2·8–10·3%)	3·8 (1·6–6·8)	29·7% (-29·3 to 101·6%)
	Intracerebral haemorrhage	5·8% (2·7–9·8%)	37·3 (15·7–68·7)	-55·8% (-72·3 to -28·8%)	6·1% (2·8–10·3%)	1·3 (0·5–2·4)	-50·6% (-70·1 to -16%)
	Subarachnoid haemorrhage	5·6% (2·6–9·6%)	6·5 (2·6–12·1)	-75·9% (-86·4 to -42·5%)	6·1% (2·8–10·3%)	0·2 (0·1–0·3)	-67·7% (-81·8 to -30·1%)
Other environmental risks	Stroke	5·5% (3·5–7·7%)	118·1 (65·7–186·6)	-25·9% (-45·2 to -2·3%)	6% (3·9–8·3%)	5·2 (2·9–8·3)	-16·7% (-40·8 to 13·3%)
	Ischaemic stroke	5·8% (3·7–8·2%)	80·6 (45·7–126·7)	25·7% (-9·4 to 61·9%)	6% (3·8–8·3%)	3·7 (2–6)	31·1% (-10·2 to 74·3%)
	Intracerebral haemorrhage	5·2% (3–7·8%)	33·4 (17·2–58·7)	-59·3% (-72·9 to -41·2%)	6·4% (3·9–9·1%)	1·3 (0·7–2·3)	-55·6% (-70·3 to -34·9%)
	Subarachnoid haemorrhage	3·5% (1·8–5·9%)	4·1 (1·9–7·6)	-69·5% (-82·7 to -41·9%)	5·2% (3–7·8%)	0·1 (0·1–0·3)	-65·4% (-80·7 to -31%)
Particulate matter pollution	Meningitis	2·2% (1–4·1%)	1·2 (0·5–2·5)	-83·7% (-94·2 to -63·3%)	1·6% (0·7–3·1%)	<0·1 (<0·1–<0·1)	-83·7% (-94·2 to -63·3%)
	Encephalitis	1·6% (0·5–3·3%)	1 (0·2–2·4)	-29·8% (-76·3 to 74%)	1·2% (0·3–2·7%)	<0·1 (<0·1–<0·1)	-29·8% (-76·3 to 74%)
	Stroke	34·3% (29·1–38·6%)	734·1 (528·3–1009·9)	-14·4% (-34·7 to 10%)	32·5% (27·7–36·7%)	27·9 (19·3–40)	-15·7% (-38·2 to 12%)
	Ischaemic stroke	35·3% (30·1–39·5%)	487·6 (355·3–656·1)	32·7% (-2·5 to 67·6%)	31·9% (27·2–36%)	19·9 (13·8–28·2)	22·9% (-13·3 to 62·4%)
	Intracerebral haemorrhage	33·5% (28–38·9%)	215·8 (143·8–324·5)	-48·4% (-64·2 to -26·5%)	34·7% (29·8–39·1%)	7·2 (4·7–11)	-51·3% (-67 to -29%)
	Subarachnoid haemorrhage	26·9% (19·9–35%)	30·7 (20–44·9)	-57·5% (-74·9 to -23·3%)	31% (24·9–36·5%)	0·9 (0·5–1·3)	-60·6% (-78 to -22·4%)
Secondhand smoke	Stroke	3·9% (2·9–4·9%)	83·2 (55·2–120·8)	-26·4% (-46 to -3·5%)	3·5% (2·6–4·4%)	3 (1·9–4·4)	-28% (-48·9 to -3·7%)
	Ischaemic stroke	3·7% (2·8–4·7%)	51·5 (34–73·7)	20·5% (-13·7 to 56%)	3·2% (2·4–4·2%)	2 (1·3–3)	7·9% (-24·5 to 42·7%)
	Intracerebral haemorrhage	4·3% (3·1–5·5%)	27·4 (17·2–41·8)	-53·7% (-68·6 to -33·6%)	4·1% (3–5·3%)	0·9 (0·5–1·3)	-56·4% (-71 to -36·2%)
	Subarachnoid haemorrhage	3·7% (2·4–5·2%)	4·2 (2·5–6·5)	-61·9% (-78·9 to -28·3%)	4% (2·8–5·3%)	0·1 (0·1–0·2)	-64·7% (-81·1 to -30·1%)
Short gestation	Meningitis	6·9% (3·4–11·9%)	3·7 (1·6–7·2)	-81·4% (-92·7 to -60·9%)	5% (2·3–9%)	<0·1 (<0·1–0·1)	-81·4% (-92·7 to -60·8%)
	Encephalitis	4·9% (1·6–10·1%)	3·2 (0·6–7·3)	-17·5% (-68·3 to 86·9%)	3·7% (1–7·7%)	<0·1 (<0·1–0·1)	-17·5% (-68·3 to 86·9%)
Smoking	Stroke	13·1% (11·5–15·5%)	282·2 (202·3–403·2)	-2·4% (-28·3 to 32·1%)	11·8% (10·2–13·9%)	10·2 (6·9–15·2)	-1·7% (-30·9 to 35·3%)

Table S16 Regional and national disabilities and deaths related to neurological conditions attributable to risk factors in North Africa and Middle East countries

Location<br>Risk factor (alphabetic order)	Cause	DALYs (95% UI)<br>PAF (%)<br>2019	Rate per 100,000<br>2019	Rate change (%)<br>From 1990 to 2019	Deaths (95% UI)<br>PAF (%)<br>2019	Rate per 100,000<br>2019	Rate change (%)<br>From 1990 to 2019
	Ischaemic stroke	13·4% (11·5–15·6%)	186·4 (135·5–262·7)	53·2% (9·5 to 105·9%)	11·3% (9·6–13·3%)	7·1 (4·8–10·5)	48·9% (1·2 to 108%)
	Intracerebral haemorrhage	13·2% (10·4–16·2%)	85·7 (54·5–137·4)	-41·7% (-62 to -12%)	13·4% (10·6–15·9%)	2·8 (1·7–4·6)	-43·7% (-64·6 to -14·5%)
	Subarachnoid haemorrhage	8·9% (6·5–13·1%)	10·2 (6·5–16·6)	-50·5% (-71·9 to 2·4%)	10·8% (8·5–14%)	0·3 (0·2–0·5)	-52·6% (-73·9 to 3·3%)
	Neurological disorders*	5·1% (1·7–11·4%)	67·6 (21–170·4)	36% (12·5 to 64·8%)	12·5% (3·3–21·2%)	4·4 (0·5–13·4)	40·6% (6·5 to 83·6%)
	Alzheimer's disease and other dementias	21·2% (13·7–27·8%)	76·6 (30·5–179·4)	36·1% (12 to 65·5%)	21·1% (13·4–28·2%)	5 (1·1–14·1)	40·8% (9·5 to 81·1%)
	Parkinson's disease	-10·7% (-16·1–-5·5%)	-10·5 (-17·2–-5·1)	36·2% (0·6 to 80·7%)	-9·9% (-15–-5%)	-0·6 (-1–-0·3)	42·1% (1·1 to 92·7%)
	Multiple sclerosis	10·3% (3·6–17·8%)	1·5 (0·8–3·3)	30·4% (-7 to 67·5%)	12·1% (4·1–20·6%)	<0·1 (<0·1–0·1)	26·3% (-22 to 86·5%)
Tobacco	Stroke	16·6% (14·7–19%)	356 (258·6–499)	-8·8% (-31·7 to 21·6%)	14·9% (13·2–16·9%)	12·8 (9–18·8)	-8·9% (-34·4 to 23·4%)
	Ischaemic stroke	16·8% (14·8–19%)	232 (171–319·4)	45·1% (3·3 to 91·2%)	14·2% (12·4–16·3%)	8·9 (6·2–12·8)	37·7% (-4·8 to 86·8%)
	Intracerebral haemorrhage	17% (14·1–20%)	109·9 (71·9–171·2)	-45% (-63·2 to -19·6%)	17% (14·3–19·6%)	3·6 (2·3–5·6)	-47·1% (-65·4 to -21·5%)
	Subarachnoid haemorrhage	12·2% (9·1–17·3%)	14 (9·1–22)	-54·4% (-73·3 to -14·1%)	14·4% (11·8–17·6%)	0·4 (0·2–0·6)	-56·5% (-75·6 to -13%)
	Neurological disorders*	5·1% (1·7–11·4%)	67·6 (21–170·4)	36% (12·5 to 64·8%)	12·5% (3·3–21·2%)	4·4 (0·5–13·4)	40·6% (6·5 to 83·6%)
	Alzheimer's disease and other dementias	21·2% (13·7–27·8%)	76·6 (30·5–179·4)	36·1% (12 to 65·5%)	21·1% (13·4–28·2%)	5 (1·1–14·1)	40·8% (9·5 to 81·1%)
	Parkinson's disease	-10·7% (-16·1–-5·5%)	-10·5 (-17·2–-5·1)	36·2% (0·6 to 80·7%)	-9·9% (-15–-5%)	-0·6 (-1–-0·3)	42·1% (1·1 to 92·7%)
	Multiple sclerosis	10·3% (3·6–17·8%)	1·5 (0·8–3·3)	30·4% (-7 to 67·5%)	12·1% (4·1–20·6%)	<0·1 (<0·1–0·1)	26·3% (-22 to 86·5%)
**Iran**							
Air pollution	Meningitis	0·8% (0·6–1%)	0·3 (0·2–0·4)	-85·4% (-91·3 to -76·8%)	0·4% (0·3–0·5%)	<0·1 (<0·1–<0·1)	-85·4% (-91·3 to -76·8%)
	Encephalitis	0·6% (0·2–0·9%)	0·1 (0–0·2)	-9·6% (-51·9 to 45%)	0·4% (0·2–0·5%)	<0·1 (<0·1–<0·1)	-9·6% (-51·9 to 45%)
	Stroke	25·8% (22·8–29%)	325·8 (281·7–373·3)	-48·3% (-53·6 to -40·5%)	22% (19·3–24·9%)	14·5 (12·3–16·8)	-49·2% (-54·8 to -40%)
	Ischaemic stroke	25·2% (22·2–28·4%)	248 (213–286·4)	-45·9% (-52 to -38·1%)	21·4% (18·7–24·2%)	11·7 (9·8–13·6)	-47% (-54 to -37·7%)
	Intracerebral haemorrhage	27·8% (24·5–31·1%)	62·1 (54·1–70·3)	-54·9% (-61·8 to -46·9%)	24·6% (21·7–27·7%)	2·3 (2–2·6)	-55·5% (-62·5 to -46·3%)
	Subarachnoid haemorrhage	28·8% (25·4–32·2%)	15·7 (13·3–18·9)	-54·7% (-67·3 to -31·4%)	25·1% (22–28·5%)	0·5 (0·4–0·6)	-60·6% (-73·5 to -35·6%)
Alcohol use	Stroke	0·5% (0·3–0·7%)	6·4 (3·9–9·3)	197·8% (20 to 2297·6%)	0·3% (0·2–0·5%)	0·2 (0·1–0·3)	251·4% (-2312·6 to 2759·2%)
	Ischaemic stroke	0·3% (0·1–0·5%)	2·8 (0·9–5)	526·6% (-6564 to 4368·7%)	0·2% (0·1–0·4%)	0·1 (0–0·2)	1095% (-4274·9 to 5545·1%)
	Intracerebral haemorrhage	1·6% (1·1–2·3%)	3·6 (2·4–5·2)	110·5% (29·8 to 388·7%)	1·2% (0·8–1·8%)	0·1 (0·1–0·2)	108·8% (25·3 to 411%)
	Neurological disorders*	0·2% (0·1–0·4%)	2·5 (1·5–4)	204·6% (90·2 to 478·2%)	0·1% (0–0·2%)	<0·1 (<0·1–<0·1)	157·9% (66·6 to 398·7%)
	Idiopathic epilepsy	1·8% (1·2–2·6%)	2·5 (1·5–4)	204·6% (90·2 to 478·2%)	2·2% (1·4–3·2%)	<0·1 (<0·1–<0·1)	157·9% (66·6 to 398·7%)
All risk factors	Meningitis	4·4% (3·3–5·5%)	1·7 (1·2–2·3)	-86·4% (-91·5 to -79·4%)	2·4% (1·7–3·1%)	<0·1 (<0·1–<0·1)	-86·4% (-91·5 to -79·4%)
	Encephalitis	3·7% (1·5–5%)	0·6 (0·2–0·9)	-15·8% (-53·7 to 28·2%)	2·2% (1–3%)	<0·1 (<0·1–<0·1)	-15·8% (-53·7 to 28·2%)
	Stroke	84·9% (80·8–89·7%)	1072·1 (972·6–1165·2)	-43·9% (-49·4 to -35·4%)	82·5% (76·5–90%)	54·7 (47·4–61·1)	-43·6% (-49·3 to -33·2%)

Table S16 Regional and national disabilities and deaths related to neurological conditions attributable to risk factors in North Africa and Middle East countries

Location<br>Risk factor (alphabetic order)	Cause	DALYs (95% UI)<br>PAF (%)<br>2019	Rate per 100,000<br>2019	Rate change (%)<br>From 1990 to 2019	Deaths (95% UI)<br>PAF (%)<br>2019	Rate per 100,000<br>2019	Rate change (%)<br>From 1990 to 2019
	Ischaemic stroke	85·6% (80·7–91·4%)	842·3 (754–927·8)	-41·4% (-46·9 to -32·8%)	82·6% (75·4–91·5%)	45·3 (38·9–51·5)	-41·3% (-47·6 to -30·5%)
	Intracerebral haemorrhage	83·1% (79·4–86·6%)	185·4 (172·5–198·3)	-51·1% (-58·3 to -42·4%)	82·4% (77–87·5%)	7·7 (6·9–8·4)	-50·9% (-58·4 to -40·2%)
	Subarachnoid haemorrhage	81·2% (77·2–85·1%)	44·3 (38·8–51·3)	-53·8% (-67·2 to -29·1%)	80% (73·7–85·9%)	1·7 (1·4–2)	-59·2% (-73 to -32·7%)
	Neurological disorders*	10·1% (4·1–20%)	138·8 (51·8–339·6)	32% (14·6 to 63·3%)	24·4% (12·1–38·7%)	8·4 (1·7–25·8)	35·3% (15·7 to 89·6%)
	Alzheimer's disease and other dementias	35·8% (22·9–50·6%)	138·3 (51·8–336·3)	28·7% (11·3 to 59·1%)	34% (21·2–48·9%)	8·6 (1·9–25·9)	33% (12·9 to 84%)
	Parkinson's disease	-6·4% (-9·8–-3·2%)	-4·9 (-7·6–-2·4)	-20·8% (-40·1 to 4·9%)	-5·4% (-8·3–-2·6%)	-0·3 (-0·4–-0·1)	-15·9% (-38·2 to 15·3%)
	Idiopathic epilepsy	1·8% (1·2–2·6%)	2·5 (1·5–4)	204·6% (90·2 to 478·2%)	2·2% (1·4–3·2%)	<0·1 (<0·1–<0·1)	157·9% (66·6 to 398·7%)
	Multiple sclerosis	9·8% (7·2–13·5%)	2·9 (1·9–4·7)	-6·3% (-34·1 to 15%)	10·4% (7·4–14·7%)	<0·1 (<0·1–0·1)	0·1% (-40·7 to 40%)
Ambient particulate matter pollution	Meningitis	0·8% (0·5–1%)	0·3 (0·2–0·4)	-80·7% (-88·8 to -68·2%)	0·4% (0·3–0·5%)	<0·1 (<0·1–<0·1)	-80·7% (-88·8 to -68·2%)
	Encephalitis	0·6% (0·2–0·9%)	0·1 (0–0·2)	13·4% (-39·4 to 91·2%)	0·4% (0·2–0·5%)	<0·1 (<0·1–<0·1)	13·4% (-39·4 to 91·2%)
	Stroke	25·8% (22·8–29%)	325 (280·7–372·7)	-37·6% (-46 to -24·3%)	21·9% (19·2–24·8%)	14·5 (12·3–16·8)	-38·6% (-47 to -24·6%)
	Ischaemic stroke	25·1% (22·2–28·4%)	247·5 (212·2–285·6)	-34·4% (-42·9 to -21%)	21·3% (18·7–24·2%)	11·7 (9·8–13·6)	-35·8% (-45·2 to -21·3%)
	Intracerebral haemorrhage	27·7% (24·4–31%)	61·9 (54·1–70·2)	-45·7% (-54·8 to -32·7%)	24·6% (21·6–27·6%)	2·3 (2–2·6)	-46·4% (-55·3 to -32·1%)
	Subarachnoid haemorrhage	28·7% (25·3–32·2%)	15·7 (13·3–18·9)	-46·9% (-62·3 to -18·7%)	25·1% (22–28·5%)	0·5 (0·4–0·6)	-54·1% (-69·6 to -24·5%)
Behavioural risk factors‡	Meningitis	4·3% (3·2–5·3%)	1·6 (1·2–2·2)	-86·3% (-91·5 to -79·3%)	2·3% (1·7–2·9%)	<0·1 (<0·1–<0·1)	-86·3% (-91·5 to -79·3%)
	Encephalitis	3·5% (1·4–4·8%)	0·6 (0·2–0·8)	-15·2% (-53·5 to 28·8%)	2·1% (1–2·9%)	<0·1 (<0·1–<0·1)	-15·2% (-53·5 to 28·8%)
	Stroke	31·2% (26·4–36·7%)	394·4 (327·4–474·9)	-51·6% (-57·7 to -44·3%)	27·8% (22·5–34%)	18·4 (14·7–23)	-50·9% (-58·1 to -42·1%)
	Ischaemic stroke	32·6% (26·7–39·3%)	320·5 (259·7–395·6)	-48·7% (-55·3 to -41·5%)	28·8% (22·8–36·2%)	15·8 (12·3–20·2)	-48·3% (-55·6 to -39·1%)
	Intracerebral haemorrhage	26·9% (22·8–31·8%)	60 (50·4–71·6)	-60·8% (-67·2 to -53·2%)	22·8% (19·1–27·8%)	2·1 (1·7–2·6)	-61·6% (-68 to -53·1%)
	Subarachnoid haemorrhage	25·6% (21·3–30·9%)	14 (11·2–17·7)	-62·1% (-73·9 to -40·9%)	21·7% (17·8–26·8%)	0·5 (0·4–0·6)	-66·9% (-78·3 to -45%)
	Neurological disorders*	3·4% (1·3–6·8%)	46·4 (17·8–106·5)	-6·2% (-19·8 to 16%)	6·7% (2·5–10·9%)	2·4 (0·4–7)	-7·8% (-24·2 to 25·9%)
	Alzheimer's disease and other dementias	11·9% (7·4–16·7%)	46 (17·5–104·7)	-11·3% (-24·8 to 7·2%)	10·1% (6–14·2%)	2·5 (0·6–7·1)	-9·2% (-25·6 to 17·5%)
	Parkinson's disease	-6·4% (-9·8–-3·2%)	-4·9 (-7·6–-2·4)	-20·8% (-40·1 to 4·9%)	-5·4% (-8·3–-2·6%)	-0·3 (-0·4–-0·1)	-15·9% (-38·2 to 15·3%)
	Idiopathic epilepsy	1·8% (1·2–2·6%)	2·5 (1·5–4)	204·6% (90·2 to 478·2%)	2·2% (1·4–3·2%)	<0·1 (<0·1–<0·1)	157·9% (66·6 to 398·7%)
	Multiple sclerosis	9·8% (7·2–13·5%)	2·9 (1·9–4·7)	-6·3% (-34·1 to 15%)	10·4% (7·4–14·7%)	<0·1 (<0·1–0·1)	0·1% (-40·7 to 40%)
Child and maternal malnutrition	Meningitis	4·3% (3·2–5·3%)	1·6 (1·2–2·2)	-86·3% (-91·5 to -79·3%)	2·3% (1·7–2·9%)	<0·1 (<0·1–<0·1)	-86·3% (-91·5 to -79·3%)
	Encephalitis	3·5% (1·4–4·8%)	0·6 (0·2–0·8)	-15·2% (-53·5 to 28·8%)	2·1% (1–2·9%)	<0·1 (<0·1–<0·1)	-15·2% (-53·5 to 28·8%)
Diet high in red meat	Stroke	3·2% (1·1–4·9%)	40·9 (14·1–63·5)	-55·3% (-64·5 to -47·5%)	2·5% (0·8–3·8%)	1·6 (0·6–2·6)	-54·7% (-64 to -45·7%)
	Ischaemic stroke	3% (0·5–4·7%)	29·5 (5·1–47·4)	-52·7% (-63·7 to -44·7%)	2·3% (0·4–3·6%)	1·2 (0·2–2)	-52·1% (-63·1 to -42·2%)
	Intracerebral haemorrhage	4% (0·6–6·5%)	9 (1·4–14·7)	-60·8% (-70·5 to -53%)	3·4% (0·5–5·5%)	0·3 (0–0·5)	-60·6% (-70·5 to -51·6%)
	Subarachnoid haemorrhage	4·2% (0·7–6·8%)	2·3 (0·4–3·8)	-60·3% (-73 to -40·1%)	3·4% (0·5–5·5%)	0·1 (0–0·1)	-65·3% (-77·5 to -44%)

Table S16 Regional and national disabilities and deaths related to neurological conditions attributable to risk factors in North Africa and Middle East countries

Location<br>Risk factor (alphabetic order)	Cause	DALYs (95% UI)<br>PAF (%)<br>2019	 Rate per 100,000<br>2019	 Rate change (%)<br>From 1990 to 2019	Deaths (95% UI)<br>PAF (%)<br>2019	 Rate per 100,000<br>2019	 Rate change (%)<br>From 1990 to 2019
Diet high in sodium	Stroke	1·9% (0·3–7·3%)	23·9 (4·1–93·4)	-46·5% (-59·5 to -25·2%)	1·6% (0·3–6·1%)	1 (0·2–4·1)	-47·1% (-60·5 to -27·2%)
	Ischaemic stroke	1·8% (0·3–6·9%)	17·6 (3·2–69·3)	-43·9% (-58·3 to -22·5%)	1·5% (0·3–5·7%)	0·8 (0·2–3·2)	-44·6% (-60·6 to -24·4%)
	Intracerebral haemorrhage	2·3% (0·3–8·8%)	5·1 (0·7–19·4)	-52·4% (-63·9 to -29·7%)	2% (0·3–7·9%)	0·2 (0–0·7)	-53·2% (-64·9 to -32·7%)
	Subarachnoid haemorrhage	2·2% (0·3–8·5%)	1·2 (0·2–4·5)	-52·9% (-70·3 to -18·7%)	2% (0·3–7·6%)	<0·1 (<0·1–0·2)	-58·4% (-75·5 to -25·9%)
Diet low in fibre	Stroke	1·4% (0·4–2·9%)	18·1 (4·6–36·7)	-71% (-76·7 to -63·1%)	1·3% (0·4–2·7%)	0·9 (0·2–1·7)	-69·1% (-76·2 to -58·6%)
	Ischaemic stroke	1·4% (0·4–2·8%)	13·6 (3·5–27·3)	-69·4% (-75·3 to -60·7%)	1·3% (0·4–2·6%)	0·7 (0·2–1·4)	-67·2% (-74·9 to -56%)
	Intracerebral haemorrhage	1·6% (0·4–3·2%)	3·5 (0·8–7·2)	-74·9% (-80·3 to -67·4%)	1·5% (0·4–3%)	0·1 (0–0·3)	-74% (-79·8 to -65·3%)
	Subarachnoid haemorrhage	1·7% (0·4–3·4%)	0·9 (0·2–1·9)	-75·5% (-83·6 to -61%)	1·5% (0·4–3·1%)	<0·1 (<0·1–0·1)	-78·1% (-85·9 to -62·5%)
Diet low in fruits	Stroke	1·7% (0·8–2·8%)	21·7 (9·8–35·7)	-62·9% (-71·1 to -54%)	1·3% (0·6–2·2%)	0·9 (0·4–1·5)	-64·2% (-73·8 to -52·7%)
	Ischaemic stroke	1·4% (0·4–2·7%)	14 (3·7–25·9)	-60·2% (-69·2 to -48·3%)	1·1% (0·3–2·1%)	0·6 (0·2–1·2)	-61·4% (-72·4 to -45·8%)
	Intracerebral haemorrhage	2·7% (1·4–4·5%)	6·1 (3–10·2)	-66·9% (-74·1 to -58%)	2·2% (1·1–3·8%)	0·2 (0·1–0·3)	-68·3% (-76·1 to -58·3%)
	Subarachnoid haemorrhage	2·9% (1·4–4·8%)	1·6 (0·8–2·7)	-67·7% (-78·1 to -48·2%)	2·3% (1·1–3·8%)	<0·1 (<0·1–0·1)	-74·1% (-83·7 to -54·4%)
Diet low in vegetables	Stroke	0·6% (0·2–1·1%)	7·5 (2·9–14·1)	-85·7% (-90·8 to -75·5%)	0·7% (0·3–1·2%)	0·5 (0·2–0·8)	-80·8% (-87·6 to -69·1%)
	Ischaemic stroke	0·6% (0·2–1·2%)	5·8 (2–11·7)	-83·3% (-89·5 to -55·1%)	0·7% (0·2–1·3%)	0·4 (0·1–0·7)	-77·7% (-85·8 to -53%)
	Intracerebral haemorrhage	0·6% (0·2–1·8%)	1·4 (0·4–4)	-90·4% (-95 to -64%)	0·8% (0·2–2·1%)	0·1 (0–0·2)	-87·5% (-93·2 to -63·5%)
	Subarachnoid haemorrhage	0·6% (0·2–1·8%)	0·3 (0·1–1)	-90·9% (-95·7 to -66·1%)	0·8% (0·2–2·1%)	<0·1 (<0·1–<0·1)	-89·4% (-95·1 to -68%)
Diet low in whole grains	Stroke	7·5% (4·2–9·5%)	94·2 (53–120·6)	-42·4% (-47·9 to -34·4%)	7·1% (4·3–8·8%)	4·7 (2·9–5·9)	-42·8% (-49·8 to -32·4%)
	Ischaemic stroke	9·6% (5·4–12·2%)	94·2 (53–120·6)	-42·4% (-47·9 to -34·4%)	8·6% (5·2–10·6%)	4·7 (2·9–5·9)	-42·8% (-49·8 to -32·4%)
Dietary risks	Stroke	15·3% (11·1–20·2%)	192·9 (136·4–258·3)	-55·3% (-62·5 to -47·6%)	13·7% (10·1–17·9%)	9·1 (6·4–12·1)	-54·4% (-62·5 to -45·1%)
	Ischaemic stroke	16·6% (11·8–21·4%)	163·1 (112·7–213·7)	-51·7% (-59·5 to -43·8%)	14·6% (10·6–18·6%)	8 (5·6–10·5)	-51·2% (-60·2 to -41·7%)
	Intracerebral haemorrhage	10·7% (5·8–16·7%)	23·9 (12·9–37·2)	-67·9% (-76·3 to -58·3%)	9·5% (5·1–15·1%)	0·9 (0·5–1·4)	-68% (-76·3 to -57·8%)
	Subarachnoid haemorrhage	11% (6–17%)	6 (3·3–9·4)	-68·5% (-79·9 to -49·7%)	9·5% (5·2–15·1%)	0·2 (0·1–0·3)	-72·9% (-83·3 to -52·8%)
Environmental/occupational risk factors§	Meningitis	0·8% (0·6–1%)	0·3 (0·2–0·4)	-85·4% (-91·3 to -76·8%)	0·4% (0·3–0·5%)	<0·1 (<0·1–<0·1)	-85·4% (-91·3 to -76·8%)
	Encephalitis	0·6% (0·2–0·9%)	0·1 (0–0·2)	-9·6% (-51·9 to 45%)	0·4% (0·2–0·5%)	<0·1 (<0·1–<0·1)	-9·6% (-51·9 to 45%)
	Stroke	36·6% (33·5–40%)	461·7 (408·7–516)	-48·3% (-53·5 to -40·7%)	34·4% (31·4–37·6%)	22·8 (19·8–25·6)	-46·9% (-52·2 to -37·4%)
	Ischaemic stroke	35·9% (32·7–39·4%)	353·4 (309·2–397·3)	-45·4% (-51·1 to -37·7%)	33·8% (30·7–37·2%)	18·5 (15·9–20·9)	-44·3% (-50·4 to -34·6%)
	Intracerebral haemorrhage	38·9% (35·5–42·4%)	86·9 (77·9–96·5)	-55·7% (-62·1 to -48·3%)	37·5% (34–41·1%)	3·5 (3·1–3·9)	-54·4% (-61·4 to -45%)
	Subarachnoid haemorrhage	39·1% (35·6–42·6%)	21·4 (18·3–25·3)	-56·7% (-68·9 to -33·8%)	37·6% (33·9–41·4%)	0·8 (0·7–0·9)	-60·6% (-73·7 to -35·7%)
High body-mass index	Stroke	28% (19·9–36·2%)	353·2 (248·5–462·5)	-22·9% (-37·2 to 0·4%)	17·8% (11·4–24·4%)	11·8 (7·7–16·4)	-24·2% (-38·7 to -0·5%)
	Ischaemic stroke	23·9% (16·2–32%)	235·2 (158·4–319·6)	-16·1% (-31·9 to 8·5%)	15·2% (9·2–21·9%)	8·3 (5·1–12·1)	-17·8% (-35·3 to 7·6%)
	Intracerebral haemorrhage	42·1% (31·2–52·5%)	94 (70·1–118·7)	-33·5% (-46·7 to -11·2%)	30% (20·8–40·1%)	2·8 (1·9–3·7)	-35·1% (-48·7 to -12·5%)
	Subarachnoid haemorrhage	43·9% (33–54·5%)	24 (17·6–31·8)	-33·5% (-52·4 to -0·4%)	30·1% (20·5–40·4%)	0·6 (0·4–0·9)	-41·5% (-60·4 to -4·7%)
	Neurological disorders*	4·8% (1·4–10·7%)	65·2 (18·7–168·6)	67·3% (36·6 to 135·2%)	12·1% (4·6–22·6%)	4·2 (0·7–12·5)	70·2% (33 to 163·6%)

Table S16 Regional and national disabilities and deaths related to neurological conditions attributable to risk factors in North Africa and Middle East countries

Location<br>Risk factor (alphabetic order)	Cause	DALYs (95% UI)<br>PAF (%)<br>2019	Rate per 100,000<br>2019	Rate change (%)<br>From 1990 to 2019	Deaths (95% UI)<br>PAF (%)<br>2019	Rate per 100,000<br>2019	Rate change (%)<br>From 1990 to 2019
	Alzheimer's disease and other dementias	16·9% (7·2–29·6%)	65·2 (18·7–168·6)	67·3% (36·6 to 135·2%)	16·4% (6·8–28·7%)	4·2 (0·7–12·5)	70·2% (33 to 163·6%)
High fasting plasma glucose	Stroke	27·9% (16·5–46·7%)	352·2 (207·7–596·8)	5·7% (-13·3 to 29·2%)	28·8% (15·2–57·4%)	19·1 (10·3–38·3)	5·1% (-15·3 to 30·3%)
	Ischaemic stroke	29·1% (15–53·9%)	287 (145·3–533·2)	11·7% (-9 to 36·4%)	29·3% (13·6–64·1%)	16·1 (7·5–35·5)	9·8% (-12 to 35·6%)
	Intracerebral haemorrhage	23·5% (14·7–34·4%)	52·5 (32·9–77·7)	-11·4% (-29·4 to 11·8%)	26·5% (15·6–42·3%)	2·5 (1·4–4)	-9·1% (-28·1 to 15·6%)
	Subarachnoid haemorrhage	23·3% (14·8–33·6%)	12·7 (7·8–18·3)	-25% (-49·2 to 22·3%)	27·2% (16·1–44·2%)	0·6 (0·3–0·9)	-31·6% (-54·9 to 19·9%)
	Neurological disorders*	3·4% (0·6–9·4%)	46·9 (8·4–146·1)	70·5% (57·1 to 113·5%)	8·9% (1·9–20·1%)	3·1 (0·3–11·1)	66·2% (50·4 to 132·4%)
	Alzheimer's disease and other dementias	12·1% (2·9–25·4%)	46·9 (8·4–146·1)	70·5% (57·1 to 113·5%)	12% (2·9–25·3%)	3·1 (0·3–11·1)	66·2% (50·4 to 132·4%)
High LDL cholesterol	Stroke	18·6% (10·8–31%)	234·3 (135·1–399·7)	-44·4% (-50·7 to -37·5%)	16·6% (6·1–33·9%)	11 (4–22·8)	-44·9% (-53·4 to -35·6%)
	Ischaemic stroke	23·8% (13·9–39·8%)	234·3 (135·1–399·7)	-44·4% (-50·7 to -37·5%)	20·1% (7·4–41·1%)	11 (4–22·8)	-44·9% (-53·4 to -35·6%)
High systolic blood pressure	Stroke	48·6% (40·7–56·1%)	612·9 (506·3–718·1)	-42·7% (-48·8 to -33·6%)	44·7% (35·7–54·7%)	29·6 (23·5–36·4)	-43·6% (-50·2 to -33%)
	Ischaemic stroke	47·4% (37·9–56·5%)	466·4 (369·2–559·5)	-39·7% (-46·5 to -30·5%)	43·2% (33·2–54·7%)	23·7 (17·9–30·3)	-41% (-48·5 to -29·9%)
	Intracerebral haemorrhage	52·9% (44·3–60·9%)	118 (98·2–138)	-49·8% (-57·7 to -40·3%)	52·1% (41·1–62·4%)	4·8 (3·8–5·8)	-50·1% (-58 to -39·5%)
	Subarachnoid haemorrhage	52·3% (43·9–60·4%)	28·5 (22·9–35·4)	-53% (-66·9 to -26·9%)	51·6% (40·7–61·7%)	1·1 (0·8–1·4)	-58·9% (-72·9 to -32·1%)
High temperature	Stroke	0·6% (0·1–1·1%)	7·7 (1·6–14)	-26·1% (-404·2 to 244%)	0·7% (0·1–1·3%)	0·5 (0·1–0·8)	-25·3% (-349·5 to 245·7%)
	Ischaemic stroke	0·6% (0·1–1·1%)	6 (1·2–10·8)	-25·5% (-288·5 to 226%)	0·7% (0·2–1·3%)	0·4 (0·1–0·7)	-23·9% (-284·5 to 220·3%)
	Intracerebral haemorrhage	0·6% (0·1–1·2%)	1·4 (0·2–2·6)	-27·1% (-462·8 to 280·3%)	0·6% (0·1–1·2%)	0·1 (0–0·1)	-30·4% (-407·4 to 290·2%)
	Subarachnoid haemorrhage	0·5% (0·1–1%)	0·3 (0–0·6)	-32·8% (-437·6 to 431·9%)	0·6% (0–1·1%)	<0·1 (<0·1–<0·1)	-40·4% (-427·4 to 279·1%)
Household air pollution from solid fuels	Meningitis	0% (0–0%)	<0·1 (<0·1–<0·1)	-99·6% (-99·9 to -99·1%)	0% (0–0%)	<0·1 (<0·1–<0·1)	-99·6% (-99·9 to -99·1%)
	Encephalitis	0% (0–0%)	<0·1 (<0·1–<0·1)	-97·9% (-99·4 to -94·3%)	0% (0–0%)	<0·1 (<0·1–<0·1)	-97·9% (-99·4 to -94·3%)
	Stroke	0·1% (0–0·1%)	0·8 (0·3–1·7)	-99·3% (-99·7 to -98·3%)	0% (0–0·1%)	<0·1 (<0·1–0·1)	-99·3% (-99·7 to -98·4%)
	Ischaemic stroke	0·1% (0–0·1%)	0·6 (0·2–1·3)	-99·3% (-99·7 to -98·3%)	0% (0–0·1%)	<0·1 (<0·1–0·1)	-99·3% (-99·7 to -98·4%)
	Intracerebral haemorrhage	0·1% (0–0·2%)	0·2 (0·1–0·4)	-99·3% (-99·7 to -98·3%)	0·1% (0–0·1%)	<0·1 (<0·1–<0·1)	-99·4% (-99·7 to -98·5%)
	Subarachnoid haemorrhage	0·1% (0–0·1%)	<0·1 (<0·1–0·1)	-99·4% (-99·8 to -98·3%)	0% (0–0·1%)	<0·1 (<0·1–<0·1)	-99·5% (-99·8 to -98·6%)
Kidney dysfunction	Stroke	11·1% (8·3–13·6%)	139·5 (105·2–173·9)	-35·6% (-44·1 to -25%)	10% (5·9–13·6%)	6·6 (4–9·1)	-36·4% (-48·9 to -22·9%)
	Ischaemic stroke	12% (8·6–15·2%)	118·3 (84·3–151·7)	-33·5% (-41·9 to -22·5%)	10·2% (5·4–14·5%)	5·6 (3–8)	-35·2% (-50 to -21·5%)
	Intracerebral haemorrhage	9·5% (7·7–11·4%)	21·2 (17·1–25·7)	-45·5% (-54 to -34·3%)	11% (8·1–13·7%)	1 (0·8–1·3)	-42·3% (-51·7 to -28·3%)
Lead exposure	Stroke	6·9% (4·7–9·3%)	87·5 (57·8–117·9)	-53·2% (-60·6 to -45·7%)	7% (4·6–9·7%)	4·6 (3–6·4)	-45·8% (-54 to -35·7%)
	Ischaemic stroke	6·8% (4·5–9·4%)	67·2 (43·8–92·8)	-48·3% (-57·3 to -39·8%)	6·7% (4·3–9·8%)	3·7 (2·3–5·3)	-41·3% (-50·5 to -30·3%)
	Intracerebral haemorrhage	7·4% (4·8–10·4%)	16·5 (10·6–23·1)	-64·1% (-70·6 to -56·5%)	8·1% (5·2–11·5%)	0·8 (0·5–1·1)	-57·3% (-64·8 to -47·6%)
	Subarachnoid haemorrhage	6·8% (4·2–9·8%)	3·7 (2·2–5·5)	-66·2% (-77·3 to -47%)	7·8% (4·9–11·2%)	0·2 (0·1–0·2)	-63·1% (-75·9 to -38·9%)
Low birth weight	Meningitis	3·9% (2·9–4·8%)	1·5 (1·1–2)	-86·6% (-91·7 to -79·7%)	2·1% (1·5–2·7%)	<0·1 (<0·1–<0·1)	-86·6% (-91·7 to -79·7%)

Table S16 Regional and national disabilities and deaths related to neurological conditions attributable to risk factors in North Africa and Middle East countries

Location<br>Risk factor (alphabetic order)	Cause	DALYs (95% UI)<br>PAF (%)<br>2019	Rate per 100,000<br>2019	Rate change (%)<br>From 1990 to 2019	Deaths (95% UI)<br>PAF (%)<br>2019	Rate per 100,000<br>2019	Rate change (%)<br>From 1990 to 2019
	Encephalitis	3·2% (1·3–4·4%)	0·5 (0·2–0·8)	-16·9% (-54·1 to 26·4%)	1·9% (0·9–2·7%)	<0·1 (<0·1–<0·1)	-16·9% (-54·1 to 26·4%)
Low birth weight and short gestation	Meningitis	4·3% (3·2–5·3%)	1·6 (1·2–2·2)	-86·3% (-91·5 to -79·3%)	2·3% (1·7–2·9%)	<0·1 (<0·1–<0·1)	-86·3% (-91·5 to -79·3%)
	Encephalitis	3·5% (1·4–4·8%)	0·6 (0·2–0·8)	-15·2% (-53·5 to 28·8%)	2·1% (1–2·9%)	<0·1 (<0·1–<0·1)	-15·2% (-53·5 to 28·8%)
Low physical activity	Stroke	5% (1–12·4%)	63·4 (12–154·7)	-39·3% (-47·1 to -27·3%)	6·3% (1·3–14·1%)	4·2 (0·9–9·4)	-38·9% (-47·4 to -25·6%)
	Ischaemic stroke	6·4% (1·2–15·7%)	63·4 (12–154·7)	-39·3% (-47·1 to -27·3%)	7·6% (1·6–17%)	4·2 (0·9–9·4)	-38·9% (-47·4 to -25·6%)
Low temperature	Stroke	7·6% (5·7–9·5%)	95·6 (71·6–120·4)	-48·4% (-54·9 to -39·3%)	9% (6·9–11·2%)	6 (4·5–7·6)	-43·7% (-50·7 to -32·4%)
	Ischaemic stroke	7·4% (5·6–9·3%)	73·1 (54·8–92)	-45·5% (-53 to -35·4%)	9% (6·9–11·1%)	4·9 (3·7–6·3)	-41·1% (-48·7 to -29·6%)
	Intracerebral haemorrhage	8·2% (6·2–10·1%)	18·3 (13·8–22·9)	-55·5% (-62·4 to -47·6%)	9·1% (7–11·2%)	0·8 (0·6–1·1)	-51·7% (-59·3 to -41·1%)
	Subarachnoid haemorrhage	7·8% (6–9·8%)	4·3 (3·2–5·6)	-58·7% (-71·4 to -34·4%)	9·1% (7·1–11·2%)	0·2 (0·1–0·2)	-59·9% (-73·5 to -34·5%)
Metabolic risk factors†	Stroke	72·3% (64·4–80·5%)	912·2 (796·5–1035·6)	-39·7% (-46·2 to -30·1%)	68·3% (57·8–81·3%)	45·2 (37·1–54·9)	-39·8% (-46·9 to -27·9%)
	Ischaemic stroke	72·9% (63·7–83·4%)	717·4 (611·6–838·3)	-37·5% (-44·7 to -27·8%)	68·1% (55·7–83·7%)	37·4 (29·8–46·6)	-37·6% (-45·7 to -25·4%)
	Intracerebral haemorrhage	70·7% (63·6–77·2%)	157·9 (141–174·5)	-46·3% (-54·6 to -35·3%)	69·8% (61·4–78·1%)	6·5 (5·6–7·4)	-46·5% (-55·1 to -34%)
	Subarachnoid haemorrhage	67·6% (59·8–74·8%)	36·9 (31·2–44·3)	-49·3% (-64·1 to -21·7%)	65·9% (56·4–75·4%)	1·4 (1·1–1·7)	-55·7% (-70·7 to -27·2%)
	Neurological disorders*	7·6% (2·6–16·2%)	104·1 (33·4–264·7)	63·4% (40·8 to 114·3%)	19·5% (8·1–33·4%)	6·7 (1·2–20·6)	63·4% (37·5 to 134·8%)
	Alzheimer's disease and other dementias	26·9% (13·2–43·1%)	104·1 (33·4–264·7)	63·4% (40·8 to 114·3%)	26·4% (12·9–42·6%)	6·7 (1·2–20·6)	63·4% (37·5 to 134·8%)
Non-optimal temperature	Stroke	8·2% (6·4–10%)	103 (79·8–127·2)	-47·3% (-54·7 to -37·3%)	9·7% (7·7–11·8%)	6·4 (5–8)	-42·8% (-50·8 to -30·5%)
	Ischaemic stroke	8% (6·3–9·8%)	78·8 (60·7–97·7)	-44·4% (-53·3 to -33·5%)	9·7% (7·7–11·8%)	5·3 (4·1–6·6)	-40·2% (-49·4 to -27·4%)
	Intracerebral haemorrhage	8·8% (6·9–10·7%)	19·6 (15·3–24·1)	-54·3% (-61·7 to -45·8%)	9·7% (7·7–11·8%)	0·9 (0·7–1·1)	-50·8% (-58·5 to -39·3%)
	Subarachnoid haemorrhage	8·3% (6·6–10·2%)	4·5 (3·5–5·9)	-57·7% (-70·9 to -32·4%)	9·7% (7·6–11·7%)	0·2 (0·2–0·3)	-59·2% (-73·2 to -32·7%)
Other environmental risks	Stroke	6·9% (4·7–9·3%)	87·5 (57·8–117·9)	-53·2% (-60·6 to -45·7%)	7% (4·6–9·7%)	4·6 (3–6·4)	-45·8% (-54 to -35·7%)
	Ischaemic stroke	6·8% (4·5–9·4%)	67·2 (43·8–92·8)	-48·3% (-57·3 to -39·8%)	6·7% (4·3–9·8%)	3·7 (2·3–5·3)	-41·3% (-50·5 to -30·3%)
	Intracerebral haemorrhage	7·4% (4·8–10·4%)	16·5 (10·6–23·1)	-64·1% (-70·6 to -56·5%)	8·1% (5·2–11·5%)	0·8 (0·5–1·1)	-57·3% (-64·8 to -47·6%)
	Subarachnoid haemorrhage	6·8% (4·2–9·8%)	3·7 (2·2–5·5)	-66·2% (-77·3 to -47%)	7·8% (4·9–11·2%)	0·2 (0·1–0·2)	-63·1% (-75·9 to -38·9%)
Particulate matter pollution	Meningitis	0·8% (0·6–1%)	0·3 (0·2–0·4)	-85·4% (-91·3 to -76·8%)	0·4% (0·3–0·5%)	<0·1 (<0·1–<0·1)	-85·4% (-91·3 to -76·8%)
	Encephalitis	0·6% (0·2–0·9%)	0·1 (0–0·2)	-9·6% (-51·9 to 45%)	0·4% (0·2–0·5%)	<0·1 (<0·1–<0·1)	-9·6% (-51·9 to 45%)
	Stroke	25·8% (22·8–29%)	325·8 (281·7–373·3)	-48·3% (-53·6 to -40·5%)	22% (19·3–24·9%)	14·5 (12·3–16·8)	-49·2% (-54·8 to -40%)
	Ischaemic stroke	25·2% (22·2–28·4%)	248 (213–286·4)	-45·9% (-52 to -38·1%)	21·4% (18·7–24·2%)	11·7 (9·8–13·6)	-47% (-54 to -37·7%)
	Intracerebral haemorrhage	27·8% (24·5–31·1%)	62·1 (54·1–70·3)	-54·9% (-61·8 to -46·9%)	24·6% (21·7–27·7%)	2·3 (2–2·6)	-55·5% (-62·5 to -46·3%)
	Subarachnoid haemorrhage	28·8% (25·4–32·2%)	15·7 (13·3–18·9)	-54·7% (-67·3 to -31·4%)	25·1% (22–28·5%)	0·5 (0·4–0·6)	-60·6% (-73·5 to -35·6%)
Secondhand smoke	Stroke	3·4% (2·6–4·3%)	43·2 (32·2–53·8)	-48% (-55 to -38·8%)	2·9% (2·2–3·7%)	1·9 (1·4–2·4)	-47·6% (-55·8 to -36·9%)
	Ischaemic stroke	3·2% (2·4–4·1%)	31·9 (23·6–40·2)	-45·2% (-54·6 to -36%)	2·7% (2·1–3·5%)	1·5 (1·1–1·9)	-45% (-55·8 to -34·2%)
	Intracerebral haemorrhage	4% (3–5·1%)	9 (6·7–11·3)	-54·4% (-62·2 to -44·8%)	3·5% (2·6–4·3%)	0·3 (0·2–0·4)	-54·3% (-62·5 to -44%)

Table S16 Regional and national disabilities and deaths related to neurological conditions attributable to risk factors in North Africa and Middle East countries

Location<br>Risk factor (alphabetic order)	Cause	DALYs (95% UI)<br>PAF (%)<br>2019	Rate per 100,000<br>2019	Rate change (%)<br>From 1990 to 2019	Deaths (95% UI)<br>PAF (%)<br>2019	Rate per 100,000<br>2019	Rate change (%)<br>From 1990 to 2019
	Subarachnoid haemorrhage	4·2% (3·2–5·3%)	2·3 (1·7–3)	-54·7% (-67·2 to -31%)	3·6% (2·7–4·5%)	0·1 (0·1–0·1)	-59·7% (-72·7 to -35·2%)
Short gestation	Meningitis	2·9% (2·2–3·6%)	1·1 (0·8–1·5)	-83·5% (-89·7 to -75·2%)	1·6% (1·1–2%)	<0·1 (<0·1–<0·1)	-83·5% (-89·7 to -75·2%)
	Encephalitis	2·4% (1–3·3%)	0·4 (0·1–0·6)	1·1% (-44·1 to 53·9%)	1·5% (0·7–2%)	<0·1 (<0·1–<0·1)	1·1% (-44·1 to 53·9%)
Smoking	Stroke	11·5% (10·5–12·6%)	145·3 (129·3–161·8)	-55·2% (-62·8 to -46·8%)	7·9% (7·2–8·8%)	5·2 (4·6–5·9)	-56·8% (-64·3 to -47·5%)
	Ischaemic stroke	10·9% (9·9–11·9%)	107·1 (94·5–119·6)	-53% (-61 to -44·4%)	7·3% (6·6–8·2%)	4 (3·5–4·5)	-54·9% (-63·6 to -45·6%)
	Intracerebral haemorrhage	13·9% (12·7–15·3%)	31·1 (27·8–34·7)	-60·7% (-67·3 to -52·5%)	11·1% (10·1–12·2%)	1 (0·9–1·1)	-61·7% (-68·4 to -52·9%)
	Subarachnoid haemorrhage	13·1% (11·6–15·1%)	7·2 (6–9·3)	-58·9% (-72·5 to -35·4%)	10·6% (9·3–12·3%)	0·2 (0·2–0·3)	-63% (-76·3 to -38·6%)
	Neurological disorders*	3·2% (1·2–6·6%)	43·9 (15·3–103·2)	-9·8% (-22·8 to 9·5%)	6·6% (2·4–10·9%)	2·3 (0·3–7)	-8·3% (-25·2 to 24·4%)
	Alzheimer's disease and other dementias	11·9% (7·4–16·7%)	46 (17·5–104·7)	-11·3% (-24·8 to 7·2%)	10·1% (6–14·2%)	2·5 (0·6–7·1)	-9·2% (-25·6 to 17·5%)
	Parkinson's disease	-6·4% (-9·8–-3·2%)	-4·9 (-7·6–-2·4)	-20·8% (-40·1 to 4·9%)	-5·4% (-8·3–-2·6%)	-0·3 (-0·4–-0·1)	-15·9% (-38·2 to 15·3%)
	Multiple sclerosis	9·8% (7·2–13·5%)	2·9 (1·9–4·7)	-6·3% (-34·1 to 15%)	10·4% (7·4–14·7%)	<0·1 (<0·1–0·1)	0·1% (-40·7 to 40%)
Tobacco	Stroke	14·5% (13·3–15·8%)	182·9 (162·8–202·4)	-53·6% (-60·5 to -46%)	10·5% (9·5–11·6%)	7 (6·1–7·8)	-54·6% (-62 to -45·8%)
	Ischaemic stroke	13·7% (12·5–15%)	135 (119–150·1)	-51·3% (-59 to -43·6%)	9·8% (8·8–10·9%)	5·4 (4·7–6)	-52·5% (-61·1 to -43·9%)
	Intracerebral haemorrhage	17·4% (15·9–18·8%)	38·8 (34·9–43)	-59·3% (-65·9 to -51·5%)	14·1% (12·8–15·4%)	1·3 (1·2–1·4)	-60·1% (-67 to -51·5%)
	Subarachnoid haemorrhage	16·8% (15·1–18·9%)	9·2 (7·7–11·5)	-57·9% (-71·2 to -34·7%)	13·7% (12·2–15·5%)	0·3 (0·2–0·4)	-62·2% (-75·3 to -38·6%)
	Neurological disorders*	3·2% (1·2–6·6%)	43·9 (15·3–103·2)	-9·8% (-22·8 to 9·5%)	6·6% (2·4–10·9%)	2·3 (0·3–7)	-8·3% (-25·2 to 24·4%)
	Alzheimer's disease and other dementias	11·9% (7·4–16·7%)	46 (17·5–104·7)	-11·3% (-24·8 to 7·2%)	10·1% (6–14·2%)	2·5 (0·6–7·1)	-9·2% (-25·6 to 17·5%)
	Parkinson's disease	-6·4% (-9·8–-3·2%)	-4·9 (-7·6–-2·4)	-20·8% (-40·1 to 4·9%)	-5·4% (-8·3–-2·6%)	-0·3 (-0·4–-0·1)	-15·9% (-38·2 to 15·3%)
	Multiple sclerosis	9·8% (7·2–13·5%)	2·9 (1·9–4·7)	-6·3% (-34·1 to 15%)	10·4% (7·4–14·7%)	<0·1 (<0·1–0·1)	0·1% (-40·7 to 40%)
**Iraq**							
Air pollution	Meningitis	1·6% (0·9–2·7%)	1·1 (0·6–1·9)	-85·4% (-94 to -65·4%)	1% (0·5–1·7%)	<0·1 (<0·1–<0·1)	-85·4% (-94 to -65·4%)
	Encephalitis	0·6% (0·3–1·2%)	0·5 (0·2–0·9)	-35·3% (-76·1 to 67·1%)	0·4% (0·1–0·8%)	<0·1 (<0·1–<0·1)	-35·3% (-76·1 to 67·1%)
	Stroke	31·4% (26·2–36·4%)	917·9 (689·8–1178·5)	-27·3% (-43·3 to -7·8%)	27·4% (22·4–32%)	39·2 (29·7–49·4)	-24·1% (-40·4 to -5·4%)
	Ischaemic stroke	29·7% (24·6–34·6%)	543·9 (414·4–681·4)	-17·1% (-34·6 to 3%)	25·8% (21–30·2%)	26·1 (19·7–32·6)	-13·5% (-32·5 to 7·1%)
	Intracerebral haemorrhage	34·1% (28·7–39·3%)	349 (254·3–465·6)	-35% (-52·4 to -12·5%)	31·1% (25·8–36%)	12·4 (9·1–16·2)	-35% (-52·1 to -13·7%)
	Subarachnoid haemorrhage	34·7% (29·3–40·1%)	25·1 (17·6–35·9)	-64·2% (-77·1 to -41·8%)	31·6% (26·3–36·7%)	0·7 (0·5–1·1)	-69·3% (-81·2 to -45%)
Alcohol use	Stroke	0·4% (0–0·9%)	12 (-0·7–26·6)	-75·8% (-102·8 to -44·8%)	0·2% (-0·1–0·5%)	0·3 (-0·1–0·8)	-81·7% (-113·6 to -48·9%)
	Ischaemic stroke	-0·1% (-0·4–0·2%)	-1·9 (-7·6–4·3)	-133·9% (-745·5 to 567·5%)	-0·1% (-0·4–0·1%)	-0·1 (-0·4–0·1)	-193% (-1588·2 to 1035·1%)
	Intracerebral haemorrhage	1·4% (0·5–2·4%)	14 (4·4–27·2)	-68·2% (-87·2 to -40·8%)	1% (0·3–1·9%)	0·4 (0·1–0·8)	-69·8% (-88·3 to -43·3%)
	Neurological disorders*	0·2% (0·1–0·4%)	2·3 (0·8–5)	-64·2% (-86·1 to -18·8%)	0·1% (0–0·2%)	<0·1 (<0·1–<0·1)	-68·2% (-83·9 to -46·1%)

Table S16 Regional and national disabilities and deaths related to neurological conditions attributable to risk factors in North Africa and Middle East countries

**Location**<br>Risk factor (alphabetic order)	Cause	**DALYs (95% UI)**<br>PAF (%)<br>2019	 Rate per 100,000<br>2019	 Rate change (%)<br>From 1990 to 2019	**Deaths (95% UI)**<br>PAF (%)<br>2019	 Rate per 100,000<br>2019	 Rate change (%)<br>From 1990 to 2019
	Idiopathic epilepsy	1·8% (0·9–2·9%)	2·3 (0·8–5)	-64·2% (-86·1 to -18·8%)	2·2% (1·1–3·7%)	<0·1 (<0·1–<0·1)	-68·2% (-83·9 to -46·1%)
All risk factors	Meningitis	8·6% (5·5–12·4%)	5·8 (3·5–9·2)	-83·5% (-91·6 to -67·4%)	5·2% (3·2–7·9%)	0·1 (0–0·1)	-83·5% (-91·6 to -67·4%)
	Encephalitis	3·4% (1·6–5·9%)	2·4 (0·9–4·6)	-27·5% (-69 to 73·9%)	2·1% (0·9–3·8%)	<0·1 (<0·1–0·1)	-27·5% (-69 to 73·9%)
	Stroke	90·3% (87·5–93%)	2640·3 (2134·7–3137·5)	-16·7% (-33·9 to 3·1%)	88·6% (84·1–92·9%)	127 (104·4–149·1)	-11·4% (-29·7 to 8·2%)
	Ischaemic stroke	90·5% (86·7–94·5%)	1656·3 (1358·4–1936·1)	-5% (-24·2 to 16·5%)	88·1% (82·4–94·1%)	89 (72·6–103·8)	0·4% (-19·9 to 22·7%)
	Intracerebral haemorrhage	90·1% (87·1–92·6%)	921·6 (722·8–1159·3)	-27·2% (-46 to -4·1%)	90% (85·8–93·4%)	36 (28·7–43·9)	-26·1% (-44·1 to -4·1%)
	Subarachnoid haemorrhage	86·6% (82·7–89·9%)	62·5 (46·4–85·8)	-61·4% (-74·9 to -37·3%)	87·7% (83·3–91·4%)	2 (1·5–2·9)	-66·6% (-79·5 to -38·2%)
	Neurological disorders*	12·4% (4·7–25·1%)	163·1 (58·5–403·6)	7·4% (-8·2 to 26·4%)	28·7% (12–45·5%)	10·2 (1·8–30·8)	8·9% (-10·2 to 35·3%)
	Alzheimer's disease and other dementias	44·5% (30–60·4%)	170·2 (64·7–412·3)	9·4% (-7 to 27·8%)	42·7% (28·3–58·6%)	10·8 (2·3–31·4)	8·6% (-10 to 31·9%)
	Parkinson's disease	-13% (-19·8–-7%)	-11·1 (-17·8–-5·9)	-8·6% (-34·3 to 25·6%)	-12% (-18·2–-6·5%)	-0·7 (-1·1–-0·4)	-2·9% (-30·8 to 37·5%)
	Idiopathic epilepsy	1·8% (0·9–2·9%)	2·3 (0·8–5)	-64·2% (-86·1 to -18·8%)	2·2% (1·1–3·7%)	<0·1 (<0·1–<0·1)	-68·2% (-83·9 to -46·1%)
	Multiple sclerosis	12·1% (8·9–16·1%)	1·7 (1·1–2·6)	-8·9% (-32·6 to 16·9%)	14·6% (10·2–20·7%)	<0·1 (<0·1–<0·1)	-14·9% (-49·3 to 32%)
Ambient particulate matter pollution	Meningitis	1·6% (0·9–2·7%)	1·1 (0·6–1·9)	-78·1% (-91·2 to -46·1%)	1% (0·5–1·7%)	<0·1 (<0·1–<0·1)	-78·2% (-91·2 to -46·1%)
	Encephalitis	0·6% (0·3–1·2%)	0·5 (0·2–0·9)	-3·3% (-66·6 to 166·7%)	0·4% (0·1–0·8%)	<0·1 (<0·1–<0·1)	-3·3% (-66·6 to 166·7%)
	Stroke	31·3% (26–36·4%)	915·6 (686·5–1176)	1·8% (-25·7 to 44·6%)	27·3% (22·4–31·8%)	39·1 (29·6–49·4)	6·6% (-21·2 to 48%)
	Ischaemic stroke	29·6% (24·5–34·6%)	542·5 (413·4–680)	16·3% (-14·7 to 65·1%)	25·7% (20·9–30·1%)	26 (19·6–32·5)	21·5% (-11·3 to 70·6%)
	Intracerebral haemorrhage	34% (28·6–39·2%)	348·2 (253·7–464·6)	-9·2% (-36·3 to 34·2%)	31% (25·8–35·9%)	12·4 (9·1–16·2)	-9·1% (-35·6 to 32·8%)
	Subarachnoid haemorrhage	34·6% (29·1–39·9%)	25 (17·5–35·8)	-49·1% (-68·5 to -13·8%)	31·5% (26·2–36·6%)	0·7 (0·5–1·1)	-56·3% (-74 to -19·8%)
Behavioural risk factors‡	Meningitis	8·3% (5·3–12%)	5·6 (3·3–8·9)	-83·3% (-91·6 to -67%)	5% (3·1–7·6%)	0·1 (0–0·1)	-83·3% (-91·6 to -67%)
	Encephalitis	3·3% (1·6–5·7%)	2·3 (0·9–4·4)	-26·6% (-68·7 to 74·7%)	2·1% (0·9–3·6%)	<0·1 (<0·1–<0·1)	-26·6% (-68·7 to 74·7%)
	Stroke	38·4% (33·2–43·8%)	1123·5 (857·1–1396)	-21·5% (-39·3 to -0·3%)	35% (29·4–41%)	50·1 (38·3–62·5)	-16·3% (-34·9 to 4·6%)
	Ischaemic stroke	39% (32·5–46·6%)	714·1 (534·9–901·4)	-11·2% (-30·5 to 10%)	35·4% (28·2–43·3%)	35·8 (26·6–46·3)	-5·7% (-26·8 to 16%)
	Intracerebral haemorrhage	37·5% (32·3–43·7%)	384·1 (285·7–511·8)	-31·9% (-51·5 to -8%)	33·8% (28·9–39·7%)	13·5 (10·1–17·6)	-31·4% (-49·9 to -8·9%)
	Subarachnoid haemorrhage	34·9% (29·5–41·7%)	25·2 (17·6–36·1)	-60·3% (-75·7 to -35·2%)	32·7% (27·6–39·1%)	0·8 (0·5–1·1)	-64·9% (-79·2 to -36·3%)
	Neurological disorders*	4·9% (1·7–10·5%)	64·7 (20·8–160·1)	-16·5% (-32·9 to -0·3%)	9·7% (1·9–17%)	3·6 (0·3–11·1)	-16·4% (-47·6 to 3·9%)
	Alzheimer's disease and other dementias	18·7% (12·3–25%)	71·8 (28·4–166·4)	-11·6% (-27·2 to 4%)	16·6% (10·9–22·5%)	4·2 (1–11·9)	-13·8% (-31·2 to 4·6%)
	Parkinson's disease	-13% (-19·8–-7%)	-11·1 (-17·8–-5·9)	-8·6% (-34·3 to 25·6%)	-12% (-18·2–-6·5%)	-0·7 (-1·1–-0·4)	-2·9% (-30·8 to 37·5%)
	Idiopathic epilepsy	1·8% (0·9–2·9%)	2·3 (0·8–5)	-64·2% (-86·1 to -18·8%)	2·2% (1·1–3·7%)	<0·1 (<0·1–<0·1)	-68·2% (-83·9 to -46·1%)
	Multiple sclerosis	12·1% (8·9–16·1%)	1·7 (1·1–2·6)	-8·9% (-32·6 to 16·9%)	14·6% (10·2–20·7%)	<0·1 (<0·1–<0·1)	-14·9% (-49·3 to 32%)
Child and maternal malnutrition	Meningitis	8·3% (5·3–12%)	5·6 (3·3–8·9)	-83·3% (-91·6 to -67%)	5% (3·1–7·6%)	0·1 (0–0·1)	-83·3% (-91·6 to -67%)
	Encephalitis	3·3% (1·6–5·7%)	2·3 (0·9–4·4)	-26·6% (-68·7 to 74·7%)	2·1% (0·9–3·6%)	<0·1 (<0·1–<0·1)	-26·6% (-68·7 to 74·7%)

Table S16 Regional and national disabilities and deaths related to neurological conditions attributable to risk factors in North Africa and Middle East countries

Location<br>Risk factor (alphabetic order)	Cause	DALYs (95% UI)<br>PAF (%)<br>2019	Rate per 100,000<br>2019	Rate change (%)<br>From 1990 to 2019	Deaths (95% UI)<br>PAF (%)<br>2019	Rate per 100,000<br>2019	Rate change (%)<br>From 1990 to 2019
Diet high in red meat	Stroke	1·6% (0·5–2·8%)	48·3 (15–87·2)	-45% (-61·2 to -25·5%)	1·3% (0·4–2·2%)	1·9 (0·6–3·4)	-40·5% (-58·1 to -20·7%)
	Ischaemic stroke	1·4% (0·3–2·4%)	25·8 (4·5–48·2)	-36·1% (-55·7 to -7·2%)	1·1% (0·2–2%)	1·2 (0·2–2·1)	-29·5% (-51 to 1·1%)
	Intracerebral haemorrhage	2% (0·3–3·6%)	20·9 (2·6–42·1)	-49·9% (-66·4 to -25·8%)	1·8% (0·3–3·1%)	0·7 (0·1–1·4)	-49% (-65·2 to -26·2%)
	Subarachnoid haemorrhage	2·2% (0·2–3·9%)	1·6 (0·2–3·3)	-72·1% (-83·8 to -53·2%)	1·9% (0·3–3·4%)	<0·1 (<0·1–0·1)	-75·5% (-86·3 to -54·6%)
Diet high in sodium	Stroke	2·1% (0·3–8·3%)	61·9 (7·6–244·6)	-18·1% (-50·6 to 63%)	1·8% (0·3–7·2%)	2·6 (0·4–10·1)	-14·7% (-47·8 to 64·8%)
	Ischaemic stroke	1·9% (0·3–7·6%)	34·6 (4·7–139·3)	-7% (-44·7 to 86·1%)	1·6% (0·3–6·5%)	1·6 (0·3–6·5)	-2·5% (-40·7 to 84·9%)
	Intracerebral haemorrhage	2·5% (0·3–9·7%)	25·6 (2·5–100·3)	-25·4% (-57·7 to 43·6%)	2·3% (0·3–9%)	0·9 (0·1–3·6)	-25·6% (-58 to 41·1%)
	Subarachnoid haemorrhage	2·3% (0·2–8·9%)	1·6 (0·2–6·6)	-58·9% (-79·5 to -19%)	2·2% (0·3–8·3%)	0·1 (0–0·2)	-63·4% (-83 to -23·1%)
Diet low in fibre	Stroke	3·3% (0·7–6·4%)	97·8 (19·2–191·5)	86·4% (29·6 to 177·4%)	2·8% (0·6–5·4%)	4·1 (0·8–7·9)	78·7% (24 to 158%)
	Ischaemic stroke	3·1% (0·6–5·8%)	55·8 (11·1–109·9)	108·1% (46·4 to 198·9%)	2·6% (0·6–4·9%)	2·6 (0·5–5·1)	95% (37·5 to 175·5%)
	Intracerebral haemorrhage	3·8% (0·8–7·2%)	38·9 (7·4–78·4)	74·2% (19·5 to 176·4%)	3·4% (0·7–6·5%)	1·3 (0·3–2·7)	66·3% (13·7 to 153%)
	Subarachnoid haemorrhage	4·2% (0·8–8%)	3 (0·5–6·3)	-7·6% (-44·1 to 58·4%)	3·6% (0·7–6·9%)	0·1 (0–0·2)	-25·6% (-58 to 38·7%)
Diet low in fruits	Stroke	6·2% (2·9–10%)	180·1 (79·8–308·2)	-5·2% (-32·5 to 35·7%)	4·9% (2·2–8·1%)	7 (3–12·1)	-2·2% (-30·3 to 33·7%)
	Ischaemic stroke	4·7% (1·1–8·9%)	85·5 (18·8–166·6)	12·9% (-18·6 to 98·6%)	3·8% (0·9–7·3%)	3·8 (0·9–7·5)	17% (-14·7 to 99·4%)
	Intracerebral haemorrhage	8·6% (3·9–15·8%)	88 (37·3–171·5)	-12·5% (-39·7 to 46·8%)	7·6% (3·4–14%)	3 (1·3–5·8)	-12·8% (-39·3 to 41·5%)
	Subarachnoid haemorrhage	9·2% (4·3–16·6%)	6·6 (2·8–13)	-51·9% (-70·6 to -12·3%)	8% (3·7–14·8%)	0·2 (0·1–0·4)	-59·1% (-76·1 to -20·8%)
Diet low in vegetables	Stroke	0·9% (0·2–1·9%)	25·2 (6·8–57·1)	-32·6% (-52·6 to -0·8%)	0·9% (0·3–1·8%)	1·3 (0·4–2·7)	-24% (-47·2 to 10·4%)
	Ischaemic stroke	0·8% (0·2–1·8%)	15·4 (3·8–34)	-22% (-41·4 to 8·6%)	0·9% (0·2–1·8%)	0·9 (0·2–1·8)	-11·4% (-34·9 to 21·7%)
	Intracerebral haemorrhage	0·9% (0·2–3·3%)	9·2 (1·9–33·7)	-41·1% (-58·6 to -3·4%)	1% (0·2–3·3%)	0·4 (0·1–1·3)	-37·8% (-55·1 to -0·1%)
	Subarachnoid haemorrhage	0·9% (0·2–3·3%)	0·6 (0·1–2·4)	-69·3% (-80·8 to -41·3%)	1% (0·2–3·3%)	<0·1 (<0·1–0·1)	-72·6% (-84·3 to -40·1%)
Diet low in whole grains	Stroke	4·3% (1·7–5·9%)	125·3 (49·3–183·9)	-18% (-36·3 to 2·2%)	4·2% (1·8–5·7%)	6·1 (2·5–8·6)	-13·1% (-32·6 to 7%)
	Ischaemic stroke	6·9% (2·7–9·4%)	125·3 (49·3–183·9)	-18% (-36·3 to 2·2%)	6% (2·6–8·1%)	6·1 (2·5–8·6)	-13·1% (-32·6 to 7%)
Dietary risks	Stroke	17% (11·7–23%)	497·8 (325·6–710·4)	-10·4% (-32·7 to 14·8%)	15% (10·5–20·3%)	21·5 (14·3–30·4)	-6·5% (-28·7 to 17%)
	Ischaemic stroke	17·2% (11·3–23·8%)	315·2 (193·7–449·3)	-3·5% (-25·3 to 23·3%)	15% (10–20·7%)	15·1 (9·5–21·5)	1·3% (-20·9 to 27·3%)
	Intracerebral haemorrhage	16·6% (10·3–24·8%)	170·1 (98·6–267·2)	-15·8% (-40·5 to 22·9%)	15% (9·2–22·5%)	6 (3·5–9·3)	-15·9% (-39·7 to 19·8%)
	Subarachnoid haemorrhage	17·3% (10·9–25·5%)	12·5 (6·9–20·5)	-53·7% (-71 to -23·7%)	15·5% (9·7–22·9%)	0·4 (0·2–0·6)	-60·4% (-76·4 to -28·5%)
Environmental/occupational risk factors§	Meningitis	1·6% (0·9–2·7%)	1·1 (0·6–1·9)	-85·4% (-94 to -65·4%)	1% (0·5–1·7%)	<0·1 (<0·1–<0·1)	-85·4% (-94 to -65·4%)
	Encephalitis	0·6% (0·3–1·2%)	0·5 (0·2–0·9)	-35·3% (-76·1 to 67·1%)	0·4% (0·1–0·8%)	<0·1 (<0·1–<0·1)	-35·3% (-76·1 to 67·1%)
	Stroke	38·7% (33·3–43·8%)	1132·1 (874·6–1418·3)	-24·9% (-41·3 to -5·6%)	35·8% (30·9–40·7%)	51·3 (40·5–63·6)	-19·9% (-36·4 to -1·3%)
	Ischaemic stroke	37·1% (31·9–42·1%)	678·2 (531·6–835·8)	-13·7% (-31·8 to 7%)	34·3% (29·2–39·1%)	34·6 (27·2–42·5)	-8·3% (-27·3 to 13·4%)
	Intracerebral haemorrhage	41·4% (35·7–46·9%)	424 (314·4–553·5)	-33·6% (-51·1 to -11·5%)	39·3% (33·9–44·4%)	15·7 (12·1–20·2)	-32·6% (-49·6 to -11·3%)
	Subarachnoid haemorrhage	41·3% (35·7–46·8%)	29·8 (21·2–42)	-64·3% (-77·2 to -41·6%)	39·6% (34·2–44·9%)	0·9 (0·7–1·3)	-68·6% (-80·9 to -43%)
High body-mass index	Stroke	36·1% (25·4–46·2%)	1056·1 (692·1–1471·5)	-19·7% (-39·5 to 7·5%)	25% (16·4–33·8%)	35·9 (22·3–51)	-16·3% (-37 to 11·5%)

Table S16 Regional and national disabilities and deaths related to neurological conditions attributable to risk factors in North Africa and Middle East countries

**Location**<br>Risk factor (alphabetic order)	Cause	**DALYs (95% UI)**<br>PAF (%)<br>2019	 Rate per 100,000<br>2019	 Rate change (%)<br>From 1990 to 2019	**Deaths (95% UI)**<br>PAF (%)<br>2019	 Rate per 100,000<br>2019	 Rate change (%)<br>From 1990 to 2019
	Ischaemic stroke	27·5% (17·8–37·1%)	503·4 (319·4–715)	-5% (-26·3 to 25·4%)	19% (11·4–27·3%)	19·2 (11·2–28·6)	0·6% (-24·9 to 35·1%)
	Intracerebral haemorrhage	50·2% (36·4–62·6%)	514 (342·1–724·8)	-25·7% (-46·1 to 3·9%)	39·3% (27·4–51·6%)	15·7 (10·1–22·5)	-25·7% (-46·3 to 2·9%)
	Subarachnoid haemorrhage	53·6% (40·5–65·4%)	38·7 (25·1–57·7)	-58·3% (-73 to -33·4%)	41·6% (29·4–53·6%)	1 (0·6–1·5)	-62·7% (-77·7 to -34·3%)
	Neurological disorders*	5·8% (1·7–13·1%)	75·6 (20·6–188·6)	18·3% (-1·8 to 61·6%)	14·1% (4·9–26·4%)	4·9 (0·8–14·9)	17·2% (-7·2 to 67·8%)
	Alzheimer's disease and other dementias	19·7% (8·2–34·8%)	75·6 (20·6–188·6)	18·3% (-1·8 to 61·6%)	19·4% (8–34·1%)	4·9 (0·8–14·9)	17·2% (-7·2 to 67·8%)
High fasting plasma glucose	Stroke	30·3% (20·1–44·2%)	884·4 (563·9–1343·1)	25% (-12·1 to 74·3%)	30·8% (18·9–51·1%)	44·2 (27·5–72·3)	30% (-9·4 to 81·5%)
	Ischaemic stroke	32% (18·2–54·6%)	585·6 (327·8–1009·4)	40·7% (-0·5 to 96·4%)	31·3% (16·3–59%)	31·6 (16–58·6)	45·7% (1 to 106·1%)
	Intracerebral haemorrhage	27·6% (17·8–38·8%)	281·9 (168·6–408·6)	8·8% (-26·4 to 58·6%)	29·9% (19·7–42·3%)	12 (7·3–17·7)	9·5% (-25·3 to 59%)
	Subarachnoid haemorrhage	23·5% (15·1–32·8%)	16·9 (9·8–27)	-47·1% (-67·3 to -5·6%)	27·5% (18·3–39·4%)	0·6 (0·4–1)	-53·3% (-72 to -9·4%)
	Neurological disorders*	4·3% (0·8–12·2%)	56·2 (10·3–175·4)	54·7% (36·1 to 87·2%)	10·6% (2·3–23·9%)	3·7 (0·4–13·3)	53·5% (29·7 to 95·9%)
	Alzheimer's disease and other dementias	14·7% (3·7–30·1%)	56·2 (10·3–175·4)	54·7% (36·1 to 87·2%)	14·5% (3·6–29·9%)	3·7 (0·4–13·3)	53·5% (29·7 to 95·9%)
High LDL cholesterol	Stroke	14·2% (8·3–24·4%)	415·3 (234·4–709·9)	-4·9% (-26·6 to 19·7%)	13·6% (5·2–27·8%)	19·5 (7·4–40·3)	1·6% (-22·3 to 25·6%)
	Ischaemic stroke	22·7% (13·2–38·5%)	415·3 (234·4–709·9)	-4·9% (-26·6 to 19·7%)	19·3% (7·4–39·1%)	19·5 (7·4–40·3)	1·6% (-22·3 to 25·6%)
High systolic blood pressure	Stroke	62·3% (54·5–68·9%)	1822·7 (1421·5–2274·3)	-13·1% (-33·1 to 10%)	58·3% (49·2–67%)	83·6 (64·9–104·3)	-8·2% (-28·7 to 14·2%)
	Ischaemic stroke	59·7% (49–69%)	1091·5 (829·7–1356·3)	0·4% (-20·5 to 27%)	55·1% (42·9–66·7%)	55·7 (40·6–71·9)	5·7% (-17·7 to 34%)
	Intracerebral haemorrhage	66·9% (57·5–74·8%)	684·4 (519·1–889·2)	-23·6% (-43·4 to 2·1%)	65·9% (55·6–75·3%)	26·4 (20–33·2)	-22·7% (-41·9 to 1·7%)
	Subarachnoid haemorrhage	64·9% (55·2–73·3%)	46·8 (33·4–66·3)	-58·9% (-73·6 to -33·5%)	65·3% (55·7–74·2%)	1·5 (1·1–2·2)	-64·7% (-78·5 to -36·7%)
High temperature	Stroke	2·6% (0·6–5·2%)	76·5 (16·3–156·3)	-4% (-41·6 to 62·6%)	2·9% (0·6–5·7%)	4·2 (0·9–8·4)	4% (-35·8 to 79·3%)
	Ischaemic stroke	2·5% (0·5–5%)	46·1 (10–92·9)	12·1% (-31·3 to 94·1%)	2·9% (0·6–5·7%)	2·9 (0·6–5·9)	18·2% (-25·9 to 106·2%)
	Intracerebral haemorrhage	2·8% (0·6–5·5%)	28·5 (6–57·9)	-16·1% (-48 to 44·1%)	2·9% (0·6–5·7%)	1·2 (0·2–2·4)	-13·4% (-46·9 to 48·4%)
	Subarachnoid haemorrhage	2·6% (0·5–5·1%)	1·9 (0·4–4·1)	-59·3% (-78·5 to -19·5%)	2·9% (0·6–5·7%)	0·1 (0–0·1)	-61·6% (-79·8 to -20·2%)
Household air pollution from solid fuels	Meningitis	0% (0–0%)	<0·1 (<0·1–<0·1)	-99·9% (-100 to -99·6%)	0% (0–0%)	<0·1 (<0·1–<0·1)	-99·9% (-100 to -99·6%)
	Encephalitis	0% (0–0%)	<0·1 (<0·1–<0·1)	-99·4% (-99·8 to -98%)	0% (0–0%)	<0·1 (<0·1–<0·1)	-99·4% (-99·8 to -98%)
	Stroke	0·1% (0–0·2%)	2·3 (0·8–5·1)	-99·4% (-99·7 to -98·5%)	0·1% (0–0·1%)	0·1 (0–0·2)	-99·3% (-99·7 to -98·4%)
	Ischaemic stroke	0·1% (0–0·2%)	1·4 (0·5–3)	-99·3% (-99·7 to -98·3%)	0·1% (0–0·1%)	0·1 (0–0·1)	-99·2% (-99·7 to -98·2%)
	Intracerebral haemorrhage	0·1% (0–0·2%)	0·9 (0·3–2)	-99·4% (-99·8 to -98·7%)	0·1% (0–0·2%)	<0·1 (<0·1–0·1)	-99·4% (-99·8 to -98·7%)
	Subarachnoid haemorrhage	0·1% (0–0·2%)	0·1 (0–0·1)	-99·7% (-99·9 to -99·2%)	0·1% (0–0·2%)	<0·1 (<0·1–<0·1)	-99·7% (-99·9 to -99·3%)
Kidney dysfunction	Stroke	12·6% (10–14·9%)	367·2 (272·9–460·4)	12·7% (-11·3 to 40·4%)	12% (8·4–15·2%)	17·1 (11·6–22·6)	19·1% (-8·3 to 48·3%)
	Ischaemic stroke	14·1% (10·5–17·6%)	258·3 (184·5–337·1)	25·2% (-2·4 to 54·5%)	12·2% (7·5–16·7%)	12·3 (7·5–17·4)	30·3% (-1·4 to 64·1%)
	Intracerebral haemorrhage	10·7% (8·6–12·9%)	108·9 (79·5–143·8)	-8·7% (-32·3 to 20·6%)	12·2% (9·5–14·9%)	4·9 (3·5–6·4)	-2·2% (-25·9 to 27·2%)
Lead exposure	Stroke	3·9% (2–5·9%)	113·9 (55·8–181·6)	-28·8% (-45·5 to -9·4%)	4% (2·2–6%)	5·8 (3·1–9)	-16·5% (-34·6 to 4%)

Table S16 Regional and national disabilities and deaths related to neurological conditions attributable to risk factors in North Africa and Middle East countries

Location<br>Risk factor (alphabetic order)	Cause	DALYs (95% UI)<br>PAF (%)<br>2019	Rate per 100,000<br>2019	Rate change (%)<br>From 1990 to 2019	Deaths (95% UI)<br>PAF (%)<br>2019	Rate per 100,000<br>2019	Rate change (%)<br>From 1990 to 2019
	Ischaemic stroke	3·9% (2–6%)	71 (35·6–113·3)	-12·3% (-31·8 to 9%)	3·9% (2·1–6%)	3·9 (2·1–6·2)	0·4% (-21·4 to 26%)
	Intracerebral haemorrhage	4% (1·7–6·6%)	40·4 (16·8–70·5)	-42·8% (-59·5 to -23·5%)	4·4% (2·1–7%)	1·7 (0·8–2·9)	-34·9% (-52·3 to -15·7%)
	Subarachnoid haemorrhage	3·4% (1·2–5·9%)	2·4 (0·9–4·7)	-70·3% (-82·3 to -49·9%)	4·1% (1·9–6·6%)	0·1 (0–0·2)	-69·7% (-82·1 to -43%)
Low birth weight	Meningitis	7·7% (4·9–11·1%)	5·2 (3·1–8·1)	-83·6% (-91·7 to -67·9%)	4·6% (2·8–7·1%)	0·1 (0–0·1)	-83·6% (-91·7 to -67·9%)
	Encephalitis	3% (1·5–5·3%)	2·2 (0·8–4·1)	-27·6% (-69·3 to 72·8%)	1·9% (0·8–3·4%)	<0·1 (<0·1–<0·1)	-27·6% (-69·3 to 72·8%)
Low birth weight and short gestation	Meningitis	8·3% (5·3–12%)	5·6 (3·3–8·9)	-83·3% (-91·6 to -67%)	5% (3·1–7·6%)	0·1 (0–0·1)	-83·3% (-91·6 to -67%)
	Encephalitis	3·3% (1·6–5·7%)	2·3 (0·9–4·4)	-26·6% (-68·7 to 74·7%)	2·1% (0·9–3·6%)	<0·1 (<0·1–<0·1)	-26·6% (-68·7 to 74·7%)
Low physical activity	Stroke	5·3% (1·2–11·5%)	155·5 (34·2–351·1)	0·4% (-19·5 to 25·3%)	7% (1·7–13·8%)	10 (2·3–20·6)	4·5% (-17 to 30·5%)
	Ischaemic stroke	8·5% (1·9–18·3%)	155·5 (34·2–351·1)	0·4% (-19·5 to 25·3%)	9·9% (2·4–19·6%)	10 (2·3–20·6)	4·5% (-17 to 30·5%)
Low temperature	Stroke	4·6% (1·2–8%)	133·7 (33·9–240·9)	-14·4% (-36·6 to 13%)	5·1% (1·4–8·9%)	7·3 (1·8–13·1)	-7·1% (-29·4 to 18·9%)
	Ischaemic stroke	4·4% (1·2–7·7%)	80·7 (20·1–144)	0·1% (-26·2 to 32·1%)	5·1% (1·4–8·9%)	5·2 (1·3–9·2)	5·6% (-19·2 to 35·6%)
	Intracerebral haemorrhage	4·9% (1·3–8·5%)	49·8 (12·6–92)	-25·3% (-45·8 to 1%)	5·1% (1·4–8·9%)	2 (0·5–3·7)	-22·8% (-43 to 2·4%)
	Subarachnoid haemorrhage	4·5% (1·2–7·8%)	3·2 (0·8–6·2)	-63·9% (-78·4 to -35·9%)	5·1% (1·4–8·9%)	0·1 (0–0·2)	-65·9% (-79·6 to -34·7%)
Metabolic risk factors†	Stroke	80·4% (74·4–85·4%)	2349·4 (1872·4–2819·5)	-12·3% (-31 to 9·5%)	77·1% (69·2–85·4%)	110·5 (87·8–132)	-6·5% (-26·2 to 15·3%)
	Ischaemic stroke	80% (72·1–87·7%)	1464·3 (1166·9–1744·4)	0·1% (-20·1 to 24%)	75·8% (65·2–87·4%)	76·6 (60·1–92·8)	6·4% (-16·1 to 32·3%)
	Intracerebral haemorrhage	81·1% (74·5–86·1%)	829·7 (638·3–1055·8)	-23·1% (-43·1 to 2·2%)	80·3% (72·8–86·4%)	32·1 (25·3–39·8)	-21·9% (-40·9 to 1·9%)
	Subarachnoid haemorrhage	76·9% (69·2–82·7%)	55·5 (40·9–77·5)	-59·2% (-73·6 to -33·4%)	76·8% (68·5–83·5%)	1·8 (1·3–2·5)	-64·6% (-78·5 to -35·7%)
	Neurological disorders*	9·2% (3–20%)	120·6 (36–302·5)	28·3% (6·9 to 60·7%)	22·6% (9·1–38·6%)	7·9 (1·4–23·9)	27·2% (2·4 to 67%)
	Alzheimer's disease and other dementias	31·5% (15·5–50·1%)	120·6 (36–302·5)	28·3% (6·9 to 60·7%)	31% (15·3–49·4%)	7·9 (1·4–23·9)	27·2% (2·4 to 67%)
Non-optimal temperature	Stroke	7·1% (4–10%)	206·6 (118·4–311·7)	-11·1% (-34 to 19·2%)	7·9% (4·5–11%)	11·3 (6·4–16·7)	-3·6% (-26·4 to 26·3%)
	Ischaemic stroke	6·8% (3·9–9·7%)	124·7 (71·9–187·5)	3·8% (-22·8 to 39%)	7·9% (4·5–11%)	8 (4·5–11·7)	9·6% (-15·9 to 43·1%)
	Intracerebral haemorrhage	7·5% (4·3–10·6%)	76·9 (43·7–118·3)	-22·4% (-44·1 to 5·8%)	7·9% (4·5–11%)	3·2 (1·8–4·8)	-19·9% (-41·4 to 8·5%)
	Subarachnoid haemorrhage	6·9% (3·9–9·8%)	5 (2·7–8·3)	-62·4% (-77·3 to -33·3%)	7·9% (4·5–11%)	0·2 (0·1–0·3)	-64·5% (-78·7 to -31·8%)
Other environmental risks	Stroke	3·9% (2–5·9%)	113·9 (55·8–181·6)	-28·8% (-45·5 to -9·4%)	4% (2·2–6%)	5·8 (3·1–9)	-16·5% (-34·6 to 4%)
	Ischaemic stroke	3·9% (2–6%)	71 (35·6–113·3)	-12·3% (-31·8 to 9%)	3·9% (2·1–6%)	3·9 (2·1–6·2)	0·4% (-21·4 to 26%)
	Intracerebral haemorrhage	4% (1·7–6·6%)	40·4 (16·8–70·5)	-42·8% (-59·5 to -23·5%)	4·4% (2·1–7%)	1·7 (0·8–2·9)	-34·9% (-52·3 to -15·7%)
	Subarachnoid haemorrhage	3·4% (1·2–5·9%)	2·4 (0·9–4·7)	-70·3% (-82·3 to -49·9%)	4·1% (1·9–6·6%)	0·1 (0–0·2)	-69·7% (-82·1 to -43%)
Particulate matter pollution	Meningitis	1·6% (0·9–2·7%)	1·1 (0·6–1·9)	-85·4% (-94 to -65·4%)	1% (0·5–1·7%)	<0·1 (<0·1–<0·1)	-85·4% (-94 to -65·4%)
	Encephalitis	0·6% (0·3–1·2%)	0·5 (0·2–0·9)	-35·3% (-76·1 to 67·1%)	0·4% (0·1–0·8%)	<0·1 (<0·1–<0·1)	-35·3% (-76·1 to 67·1%)
	Stroke	31·4% (26·2–36·4%)	917·9 (689·8–1178·5)	-27·3% (-43·3 to -7·8%)	27·4% (22·4–32%)	39·2 (29·7–49·4)	-24·1% (-40·4 to -5·4%)
	Ischaemic stroke	29·7% (24·6–34·6%)	543·9 (414·4–681·4)	-17·1% (-34·6 to 3%)	25·8% (21–30·2%)	26·1 (19·7–32·6)	-13·5% (-32·5 to 7·1%)
	Intracerebral haemorrhage	34·1% (28·7–39·3%)	349 (254·3–465·6)	-35% (-52·4 to -12·5%)	31·1% (25·8–36%)	12·4 (9·1–16·2)	-35% (-52·1 to -13·7%)

Table S16 Regional and national disabilities and deaths related to neurological conditions attributable to risk factors in North Africa and Middle East countries

**Location**<br>Risk factor (alphabetic order)	Cause	**DALYs (95% UI)**<br>PAF (%)<br>2019	 Rate per 100,000<br>2019	 Rate change (%)<br>From 1990 to 2019	**Deaths (95% UI)**<br>PAF (%)<br>2019	 Rate per 100,000<br>2019	 Rate change (%)<br>From 1990 to 2019
	Subarachnoid haemorrhage	34·7% (29·3–40·1%)	25·1 (17·6–35·9)	-64·2% (-77·1 to -41·8%)	31·6% (26·3–36·7%)	0·7 (0·5–1·1)	-69·3% (-81·2 to -45%)
Secondhand smoke	Stroke	4·6% (3·5–5·8%)	134·8 (92·6–184·5)	-24·3% (-42·3 to -3%)	4% (3–5·1%)	5·8 (4–7·7)	-19·7% (-38 to 1·1%)
	Ischaemic stroke	4·2% (3·1–5·4%)	77·2 (52·8–103·3)	-11·9% (-32·3 to 12·8%)	3·7% (2·8–4·8%)	3·8 (2·6–4·9)	-7·1% (-27·8 to 15·8%)
	Intracerebral haemorrhage	5·2% (3·9–6·6%)	53·6 (35·9–75·8)	-32·5% (-51·2 to -7·4%)	4·7% (3·5–5·9%)	1·9 (1·3–2·6)	-31·7% (-50·3 to -9·2%)
	Subarachnoid haemorrhage	5·7% (4·2–7·2%)	4·1 (2·6–6·1)	-63·5% (-76·7 to -39·4%)	5·1% (3·8–6·5%)	0·1 (0·1–0·2)	-67·4% (-79·7 to -41·9%)
Short gestation	Meningitis	5·9% (3·8–8·5%)	4 (2·4–6·3)	-81·5% (-90·7 to -63·8%)	3·6% (2·2–5·4%)	<0·1 (<0·1–0·1)	-81·5% (-90·7 to -63·8%)
	Encephalitis	2·4% (1·1–4%)	1·7 (0·6–3·1)	-19·2% (-65·4 to 92·3%)	1·5% (0·6–2·6%)	<0·1 (<0·1–<0·1)	-19·2% (-65·4 to 92·3%)
Smoking	Stroke	17·9% (16·1–19·7%)	523·7 (409·6–649·5)	-31·6% (-48·8 to -9·9%)	14·3% (12·7–15·9%)	20·5 (16·1–25)	-28·7% (-46·3 to -6·7%)
	Ischaemic stroke	16·4% (14·6–18·1%)	299·4 (235–361·6)	-22·7% (-41·2 to 1%)	12·7% (11·2–14·2%)	12·8 (10·2–15·4)	-18·9% (-39·4 to 7·1%)
	Intracerebral haemorrhage	20·7% (18·6–22·8%)	211·8 (159·6–275·7)	-38·4% (-55·8 to -14·6%)	18·1% (16·2–20·2%)	7·3 (5·5–9·3)	-38% (-55 to -15·3%)
	Subarachnoid haemorrhage	17·2% (14·7–20·1%)	12·4 (8·8–18·5)	-63·8% (-79·4 to -35%)	16·5% (14–19·3%)	0·4 (0·3–0·6)	-66·8% (-81·8 to -36·3%)
	Neurological disorders*	4·7% (1·5–10·2%)	62·4 (18·6–157·6)	-12·1% (-27·6 to 4·4%)	9·6% (1·8–17%)	3·6 (0·2–11·1)	-15·7% (-43·9 to 6·3%)
	Alzheimer's disease and other dementias	18·7% (12·3–25%)	71·8 (28·4–166·4)	-11·6% (-27·2 to 4%)	16·6% (10·9–22·5%)	4·2 (1–11·9)	-13·8% (-31·2 to 4·6%)
	Parkinson's disease	-13% (-19·8–-7%)	-11·1 (-17·8–-5·9)	-8·6% (-34·3 to 25·6%)	-12% (-18·2–-6·5%)	-0·7 (-1·1–-0·4)	-2·9% (-30·8 to 37·5%)
	Multiple sclerosis	12·1% (8·9–16·1%)	1·7 (1·1–2·6)	-8·9% (-32·6 to 16·9%)	14·6% (10·2–20·7%)	<0·1 (<0·1–<0·1)	-14·9% (-49·3 to 32%)
Tobacco	Stroke	21·7% (19·5–23·7%)	633·8 (495·2–781·7)	-30% (-47·4 to -8·6%)	17·7% (15·8–19·5%)	25·3 (20–30·8)	-26·7% (-44·3 to -5·8%)
	Ischaemic stroke	19·8% (17·8–21·8%)	363·3 (288·4–438·9)	-20·5% (-39·1 to 2·3%)	15·9% (14·1–17·6%)	16 (12·9–19·2)	-16·3% (-36·2 to 8·5%)
	Intracerebral haemorrhage	24·9% (22·5–27·2%)	254·6 (191·4–330)	-37·1% (-54·7 to -14·2%)	22% (19·7–24·2%)	8·8 (6·7–11·1)	-36·7% (-54·1 to -13·6%)
	Subarachnoid haemorrhage	21·9% (19·2–25·1%)	15·9 (11·3–23·4)	-63·6% (-78·5 to -38·5%)	20·8% (18–23·6%)	0·5 (0·3–0·7)	-66·9% (-81·4 to -37·5%)
	Neurological disorders*	4·7% (1·5–10·2%)	62·4 (18·6–157·6)	-12·1% (-27·6 to 4·4%)	9·6% (1·8–17%)	3·6 (0·2–11·1)	-15·7% (-43·9 to 6·3%)
	Alzheimer's disease and other dementias	18·7% (12·3–25%)	71·8 (28·4–166·4)	-11·6% (-27·2 to 4%)	16·6% (10·9–22·5%)	4·2 (1–11·9)	-13·8% (-31·2 to 4·6%)
	Parkinson's disease	-13% (-19·8–-7%)	-11·1 (-17·8–-5·9)	-8·6% (-34·3 to 25·6%)	-12% (-18·2–-6·5%)	-0·7 (-1·1–-0·4)	-2·9% (-30·8 to 37·5%)
	Multiple sclerosis	12·1% (8·9–16·1%)	1·7 (1·1–2·6)	-8·9% (-32·6 to 16·9%)	14·6% (10·2–20·7%)	<0·1 (<0·1–<0·1)	-14·9% (-49·3 to 32%)
**Jordan**							
Air pollution	Meningitis	0·8% (0·3–1·5%)	0·4 (0·2–0·8)	-59·9% (-87·5 to 36·2%)	0·5% (0·2–1%)	<0·1 (<0·1–<0·1)	-59·9% (-87·5 to 36·2%)
	Encephalitis	0·2% (0·1–0·4%)	<0·1 (<0·1–<0·1)	-73% (-93·5 to 37·6%)	0·2% (0·1–0·3%)	<0·1 (<0·1–<0·1)	-73% (-93·5 to 37·6%)
	Stroke	22·6% (19·1–26·2%)	328 (259·2–404·4)	-49·5% (-58·7 to -39·1%)	19·2% (16–22·3%)	14·6 (11·2–18·4)	-49·8% (-60·2 to -38·1%)
	Ischaemic stroke	21·8% (18·3–25·2%)	236·1 (185·7–292·6)	-45·1% (-55·3 to -34·3%)	18·5% (15·4–21·5%)	11 (8·3–13·9)	-46·3% (-58·2 to -33·7%)
	Intracerebral haemorrhage	25% (21·2–28·6%)	81·3 (63·9–102·3)	-59·3% (-67·5 to -48·8%)	21·6% (18·2–25%)	3·3 (2·5–4·2)	-58·3% (-67·3 to -46·9%)
	Subarachnoid haemorrhage	27·3% (23·3–31·3%)	10·6 (8–13·5)	-47·2% (-61·5 to -23·8%)	22·9% (19·4–26·5%)	0·3 (0·2–0·4)	-54·5% (-69·2 to -28·3%)
Alcohol use	Stroke	0·2% (-0·1–0·6%)	3·5 (-1·6–9·2)	-21·7% (-578·9 to 398·7%)	0·1% (-0·1–0·4%)	0·1 (-0·1–0·3)	-5·7% (-672·3 to 545·7%)

Table S16 Regional and national disabilities and deaths related to neurological conditions attributable to risk factors in North Africa and Middle East countries

**Location**<br>Risk factor (alphabetic order)	Cause	**DALYs (95% UI)**<br>PAF (%)<br>2019	 Rate per 100,000<br>2019	 Rate change (%)<br>From 1990 to 2019	**Deaths (95% UI)**<br>PAF (%)<br>2019	 Rate per 100,000<br>2019	 Rate change (%)<br>From 1990 to 2019
	Ischaemic stroke	-0·1% (-0·3–0·3%)	-0·5 (-3·6–3)	-74·5% (-487·4 to 290·1%)	-0·1% (-0·3–0·1%)	-0·1 (-0·2–0·1)	-59·5% (-355·4 to 160·3%)
	Intracerebral haemorrhage	1·2% (0·4–2·2%)	4 (1·4–7·3)	-38·7% (-71·6 to 45·8%)	0·9% (0·3–1·6%)	0·1 (0–0·2)	-39·8% (-73·9 to 50·2%)
	Neurological disorders*	0·2% (0·1–0·5%)	2·4 (0·8–5·2)	6·3% (-60·5 to 176·6%)	0·1% (0–0·2%)	<0·1 (<0·1–<0·1)	-9·8% (-48·1 to 67·7%)
	Idiopathic epilepsy	2% (1–3·3%)	2·4 (0·8–5·2)	6·3% (-60·5 to 176·6%)	2·6% (1·3–4·3%)	<0·1 (<0·1–<0·1)	-9·8% (-48·1 to 67·7%)
All risk factors	Meningitis	5·9% (3·5–9·7%)	3·1 (1·7–5·6)	-61·7% (-83 to -11·6%)	3·7% (2·1–6·2%)	<0·1 (<0·1–0·1)	-61·7% (-83 to -11·6%)
	Encephalitis	1·5% (0·8–2·6%)	0·2 (0·1–0·3)	-73·7% (-90·9 to -5·6%)	1·2% (0·7–2·1%)	<0·1 (<0·1–<0·1)	-73·7% (-90·9 to -5·5%)
	Stroke	87·8% (83·9–92%)	1271·7 (1063–1477·1)	-49·7% (-58·3 to -40·2%)	85·2% (79·5–91·5%)	64·5 (52·5–76·8)	-49·2% (-59 to -38·3%)
	Ischaemic stroke	87·7% (82·9–93%)	950·6 (792·1–1107·1)	-45·8% (-55·8 to -35·6%)	85% (78·1–92·8%)	50·3 (40·7–60·5)	-46·2% (-57·5 to -34·3%)
	Intracerebral haemorrhage	88·2% (84·5–91·5%)	287·4 (236·4–341·9)	-59·5% (-67·5 to -49·8%)	85·9% (80·5–90·9%)	13·1 (10·6–15·8)	-57·9% (-66·7 to -47·2%)
	Subarachnoid haemorrhage	86·8% (82·5–90·1%)	33·8 (26·2–41·2)	-48·8% (-63·1 to -26%)	84·4% (78·8–89·5%)	1 (0·7–1·3)	-54·5% (-69·3 to -29·7%)
	Neurological disorders*	14% (5·8–27·8%)	176·8 (68·7–424·3)	7% (-9·1 to 30·7%)	32·9% (15·4–48·9%)	10·8 (2·1–31)	4·5% (-14 to 43%)
	Alzheimer's disease and other dementias	48·9% (34·6–63·6%)	181 (73·9–425·6)	3·5% (-11·6 to 22·9%)	47·2% (33·2–62·4%)	11·4 (2·6–31·3)	1·2% (-16·8 to 28·2%)
	Parkinson's disease	-13% (-19·7–-6·5%)	-9·7 (-15·4–-4·7)	-37·1% (-54·1 to -17·7%)	-11·5% (-17·9–-5·7%)	-0·6 (-0·9–-0·3)	-37·7% (-55·5 to -16·2%)
	Idiopathic epilepsy	2% (1–3·3%)	2·4 (0·8–5·2)	6·3% (-60·5 to 176·6%)	2·6% (1·3–4·3%)	<0·1 (<0·1–<0·1)	-9·8% (-48·1 to 67·7%)
	Multiple sclerosis	15·8% (12·1–19·5%)	3 (2–4·1)	-9·9% (-29·5 to 16·2%)	17·2% (12·6–21·8%)	<0·1 (<0·1–<0·1)	-16·9% (-45·6 to 18·9%)
Ambient particulate matter pollution	Meningitis	0·8% (0·3–1·5%)	0·4 (0·2–0·8)	-58·2% (-86·7 to 42·4%)	0·5% (0·2–1%)	<0·1 (<0·1–<0·1)	-58·2% (-86·7 to 42·3%)
	Encephalitis	0·2% (0·1–0·4%)	<0·1 (<0·1–<0·1)	-71·9% (-93·2 to 42·8%)	0·2% (0·1–0·3%)	<0·1 (<0·1–<0·1)	-71·9% (-93·2 to 42·8%)
	Stroke	22·6% (19·1–26·1%)	327·8 (259–404·2)	-47·7% (-57·5 to -36·8%)	19·2% (16–22·3%)	14·6 (11·2–18·4)	-48% (-58·8 to -35·8%)
	Ischaemic stroke	21·8% (18·3–25·2%)	236 (185·5–292·5)	-43·2% (-53·5 to -31·8%)	18·5% (15·4–21·5%)	11 (8·3–13·9)	-44·4% (-56·5 to -30·9%)
	Intracerebral haemorrhage	24·9% (21·2–28·6%)	81·2 (63·8–102·2)	-57·9% (-66·4 to -46·7%)	21·6% (18·2–24·9%)	3·3 (2·5–4·2)	-56·8% (-66·1 to -45·3%)
	Subarachnoid haemorrhage	27·3% (23·3–31·3%)	10·6 (8–13·5)	-45·3% (-60·3 to -20·3%)	22·9% (19·4–26·5%)	0·3 (0·2–0·4)	-52·9% (-68·4 to -25·8%)
Behavioural risk factors‡	Meningitis	5·7% (3·4–9·4%)	3 (1·7–5·5)	-61·5% (-83·1 to -10·9%)	3·6% (2·1–6%)	<0·1 (<0·1–0·1)	-61·5% (-83·1 to -10·9%)
	Encephalitis	1·5% (0·8–2·5%)	0·2 (0·1–0·3)	-73·5% (-90·7 to -6·1%)	1·2% (0·7–2%)	<0·1 (<0·1–<0·1)	-73·5% (-90·7 to -6·1%)
	Stroke	39·7% (34·6–45·1%)	575·7 (457·5–701·5)	-53·9% (-62·9 to -44·7%)	35·2% (29·7–41·1%)	26·6 (20·1–33·3)	-53·7% (-63·9 to -43·5%)
	Ischaemic stroke	40% (33·8–46·7%)	433·5 (337·9–544·6)	-50% (-59·8 to -40·4%)	35·8% (29·4–43%)	21·2 (15·6–26·9)	-50·4% (-61·7 to -39·2%)
	Intracerebral haemorrhage	38·7% (33·4–44·7%)	126·2 (97·6–157)	-63·7% (-71·5 to -54·4%)	32·9% (27·7–38·8%)	5 (3·8–6·3)	-63·4% (-71·4 to -53·7%)
	Subarachnoid haemorrhage	41% (35·4–46·9%)	15·9 (11·8–20·2)	-53·4% (-67·5 to -32·3%)	34·8% (29·4–40·8%)	0·4 (0·3–0·6)	-60% (-74·2 to -38·2%)
	Neurological disorders*	6·1% (2·3–12·8%)	77·7 (28·3–183·9)	-15·9% (-32·4 to 6·4%)	13·3% (4–21·7%)	4·5 (0·5–13·3)	-19·8% (-39·3 to 14·6%)
	Alzheimer's disease and other dementias	22·2% (14·7–29·6%)	81·9 (33·1–185·3)	-19·7% (-35·1 to -3·1%)	20·8% (13·5–28·5%)	5 (1·2–13·7)	-22·4% (-39·6 to -2·3%)
	Parkinson's disease	-13% (-19·7–-6·5%)	-9·7 (-15·4–-4·7)	-37·1% (-54·1 to -17·7%)	-11·5% (-17·9–-5·7%)	-0·6 (-0·9–-0·3)	-37·7% (-55·5 to -16·2%)
	Idiopathic epilepsy	2% (1–3·3%)	2·4 (0·8–5·2)	6·3% (-60·5 to 176·6%)	2·6% (1·3–4·3%)	<0·1 (<0·1–<0·1)	-9·8% (-48·1 to 67·7%)

Table S16 Regional and national disabilities and deaths related to neurological conditions attributable to risk factors in North Africa and Middle East countries

**Location**<br>Risk factor (alphabetic order)	Cause	**DALYs (95% UI)**<br>PAF (%)<br>2019	Rate per 100,000<br>2019	Rate change (%)<br>From 1990 to 2019	**Deaths (95% UI)**<br>PAF (%)<br>2019	Rate per 100,000<br>2019	Rate change (%)<br>From 1990 to 2019
	Multiple sclerosis	15·8% (12·1–19·5%)	3 (2–4·1)	-9·9% (-29·5 to 16·2%)	17·2% (12·6–21·8%)	<0·1 (<0·1–<0·1)	-16·9% (-45·6 to 18·9%)
Child and maternal malnutrition	Meningitis	5·7% (3·4–9·4%)	3 (1·7–5·5)	-61·5% (-83·1 to -10·9%)	3·6% (2·1–6%)	<0·1 (<0·1–0·1)	-61·5% (-83·1 to -10·9%)
	Encephalitis	1·5% (0·8–2·5%)	0·2 (0·1–0·3)	-73·5% (-90·7 to -6·1%)	1·2% (0·7–2%)	<0·1 (<0·1–<0·1)	-73·5% (-90·7 to -6·1%)
Diet high in red meat	Stroke	3·4% (1·2–5·1%)	48·8 (16·8–77·1)	-52·4% (-62·7 to -41%)	2·6% (0·9–4%)	2 (0·7–3·1)	-52·4% (-63·2 to -38·9%)
	Ischaemic stroke	3% (0·5–4·7%)	32·9 (5–54·1)	-47·4% (-57·1 to -35·7%)	2·3% (0·4–3·7%)	1·4 (0·2–2·3)	-48·3% (-59·5 to -33·6%)
	Intracerebral haemorrhage	4·3% (0·6–6·9%)	14 (2–23·7)	-61·3% (-69·2 to -51%)	3·5% (0·5–5·7%)	0·5 (0·1–0·9)	-60·1% (-68·8 to -48·6%)
	Subarachnoid haemorrhage	5·1% (0·7–8·1%)	2 (0·3–3·4)	-49·2% (-62·3 to -24%)	3·9% (0·6–6·3%)	<0·1 (<0·1–0·1)	-56·7% (-70·4 to -30·6%)
Diet high in sodium	Stroke	1·8% (0·3–7·2%)	26·3 (3·8–103·3)	-49·4% (-72·5 to -11·6%)	1·5% (0·3–6%)	1·1 (0·2–4·4)	-49·9% (-74 to -13·1%)
	Ischaemic stroke	1·7% (0·3–6·6%)	18·2 (2·8–72)	-45·3% (-70·6 to -6·1%)	1·4% (0·3–5·4%)	0·8 (0·2–3·2)	-46·5% (-72·3 to -6·7%)
	Intracerebral haemorrhage	2·2% (0·3–8·7%)	7·2 (0·8–29)	-57·8% (-78·5 to -26·1%)	1·9% (0·3–7·6%)	0·3 (0–1·2)	-57·3% (-77·5 to -23·9%)
	Subarachnoid haemorrhage	2·3% (0·3–9%)	0·9 (0·1–3·5)	-46·5% (-73·9 to 0·7%)	2% (0·3–8·1%)	<0·1 (<0·1–0·1)	-53·2% (-77·3 to -6·6%)
Diet low in fibre	Stroke	3·2% (0·7–6·2%)	46·7 (9·6–89·8)	-61·7% (-70·2 to -52·6%)	2·7% (0·6–5·3%)	2·1 (0·5–4·1)	-62·5% (-72·1 to -51·9%)
	Ischaemic stroke	3·1% (0·6–5·9%)	33·2 (7·1–64·3)	-58·4% (-67·8 to -48·9%)	2·6% (0·6–5%)	1·5 (0·3–3)	-60% (-71·1 to -47·9%)
	Intracerebral haemorrhage	3·6% (0·7–7%)	11·8 (2·3–23·5)	-68·9% (-76·7 to -60·4%)	3·2% (0·7–6·3%)	0·5 (0·1–1)	-68·3% (-76·4 to -58·9%)
	Subarachnoid haemorrhage	4·3% (0·8–8·2%)	1·7 (0·3–3·2)	-59·1% (-71·5 to -40·4%)	3·5% (0·7–6·7%)	<0·1 (<0·1–0·1)	-65·6% (-77·6 to -45·9%)
Diet low in fruits	Stroke	5% (2·1–8·4%)	72·8 (30·1–125·6)	-50·6% (-60·8 to -39·6%)	4% (1·6–6·8%)	3·1 (1·2–5·5)	-51·7% (-62·5 to -38·9%)
	Ischaemic stroke	4·2% (1–8%)	45·2 (10·4–91)	-44·8% (-55·4 to -28·9%)	3·4% (0·8–6·5%)	2 (0·4–4)	-47·5% (-59·1 to -31·3%)
	Intracerebral haemorrhage	7·4% (3·4–13·9%)	24·3 (10·8–47)	-59% (-67·4 to -47·5%)	6·4% (2·9–12·1%)	1 (0·4–1·9)	-58·3% (-67·1 to -45·8%)
	Subarachnoid haemorrhage	8·6% (4–15·9%)	3·3 (1·5–6·5)	-46·2% (-60·9 to -22·4%)	6·9% (3·2–12·9%)	0·1 (0–0·2)	-54·3% (-69·1 to -29·2%)
Diet low in vegetables	Stroke	1·9% (0·6–3·5%)	27·7 (7·5–51·2)	-58·7% (-69·2 to -47·5%)	1·7% (0·5–3%)	1·3 (0·4–2·3)	-59·2% (-70·3 to -47·8%)
	Ischaemic stroke	1·7% (0·2–3·4%)	18·8 (2·4–37·2)	-53·7% (-64·9 to -41·3%)	1·5% (0·2–2·9%)	0·9 (0·1–1·8)	-55·2% (-67·9 to -42·2%)
	Intracerebral haemorrhage	2·4% (0·2–6·6%)	7·9 (0·7–20·7)	-67·2% (-77·6 to -56·5%)	2·3% (0·2–5·9%)	0·4 (0–0·9)	-66·5% (-77·5 to -55·1%)
	Subarachnoid haemorrhage	2·6% (0·2–7·2%)	1 (0·1–2·9)	-58% (-73·7 to -39·2%)	2·4% (0·2–6·3%)	<0·1 (<0·1–0·1)	-63·8% (-78·5 to -43·2%)
Diet low in whole grains	Stroke	7% (4·1–8·8%)	101·4 (57·6–136·3)	-46·7% (-56·5 to -37%)	6·6% (4·1–8·2%)	5 (3–6·7)	-47·6% (-58·5 to -35·9%)
	Ischaemic stroke	9·4% (5·4–11·8%)	101·4 (57·6–136·3)	-46·7% (-56·5 to -37%)	8·5% (5·2–10·5%)	5 (3–6·7)	-47·6% (-58·5 to -35·9%)
Dietary risks	Stroke	20·3% (15·1–25·7%)	293·3 (210–390·7)	-52·3% (-60·9 to -42·9%)	17·8% (13·3–22·5%)	13·5 (9·5–18·1)	-52·8% (-62·3 to -42·2%)
	Ischaemic stroke	20·8% (14·8–26·5%)	225·4 (152·9–305·1)	-48·6% (-57·8 to -38·9%)	18·2% (13·4–23·1%)	10·8 (7·3–14·4)	-49·9% (-60·7 to -38·9%)
	Intracerebral haemorrhage	18·4% (11·5–26%)	59·9 (37·3–85·7)	-62·4% (-70 to -52·7%)	16·2% (10·1–23·2%)	2·5 (1·5–3·6)	-61·7% (-69·9 to -51·3%)
	Subarachnoid haemorrhage	20·7% (13·2–28·8%)	8·1 (4·9–11·7)	-51·1% (-64·7 to -28·2%)	17·3% (10·9–24·5%)	0·2 (0·1–0·3)	-58·3% (-71·9 to -34·5%)
Environmental/occupational risk factors§	Meningitis	0·8% (0·3–1·5%)	0·4 (0·2–0·8)	-59·9% (-87·5 to 36·2%)	0·5% (0·2–1%)	<0·1 (<0·1–<0·1)	-59·9% (-87·5 to 36·2%)
	Encephalitis	0·2% (0·1–0·4%)	<0·1 (<0·1–<0·1)	-73% (-93·5 to 37·6%)	0·2% (0·1–0·3%)	<0·1 (<0·1–<0·1)	-73% (-93·5 to 37·6%)
	Stroke	29% (24·8–33·3%)	419·8 (330·3–512·9)	-50·9% (-59·7 to -40·9%)	27% (22·6–31·5%)	20·4 (15·8–25·6)	-49·7% (-59·5 to -38·6%)
	Ischaemic stroke	28% (23·9–32·2%)	303·9 (239·7–371·2)	-46·5% (-56·3 to -36%)	26·3% (21·9–30·7%)	15·6 (11·9–19·7)	-46·3% (-57·4 to -34%)

Table S16 Regional and national disabilities and deaths related to neurological conditions attributable to risk factors in North Africa and Middle East countries

Location<br>Risk factor (alphabetic order)	Cause	DALYs (95% UI)<br>PAF (%)<br>2019	Rate per 100,000<br>2019	Rate change (%)<br>From 1990 to 2019	Deaths (95% UI)<br>PAF (%)<br>2019	Rate per 100,000<br>2019	Rate change (%)<br>From 1990 to 2019
	Intracerebral haemorrhage	31·7% (27·1–36·1%)	103·3 (80·3–128·5)	-60·4% (-68·3 to -50·3%)	29·4% (24·8–34·1%)	4·5 (3·4–5·6)	-58·6% (-67·2 to -47·6%)
	Subarachnoid haemorrhage	32·7% (28·2–37·1%)	12·7 (9·5–16·1)	-50·1% (-64·2 to -27·2%)	30·5% (25·8–35·1%)	0·4 (0·3–0·5)	-55% (-69·6 to -29·9%)
High body-mass index	Stroke	36% (25·5–45·1%)	522 (358·2–687·1)	-41·6% (-53·3 to -26%)	24·1% (15·9–32·5%)	18·2 (11·1–25·6)	-42·3% (-55·5 to -25·4%)
	Ischaemic stroke	31% (21·3–40%)	335·5 (221·9–454·8)	-33% (-46·5 to -15·7%)	20·5% (12·8–29·7%)	12·1 (7–17·9)	-35% (-51 to -15·2%)
	Intracerebral haemorrhage	50·2% (37·4–61·3%)	163·5 (115·3–214)	-53·9% (-63·6 to -40·3%)	36·6% (25–48·9%)	5·6 (3·6–7·8)	-53·2% (-63·8 to -39·1%)
	Subarachnoid haemorrhage	59% (46·1–69%)	23 (16·5–29·4)	-39·9% (-56·4 to -12·7%)	41·7% (29·7–53·3%)	0·5 (0·3–0·7)	-48·4% (-65 to -18·7%)
	Neurological disorders*	6·9% (2·1–15·2%)	87·5 (24·3–219·2)	38·7% (12·8 to 89·7%)	16·8% (6–29·9%)	5·4 (0·9–15·8)	35·7% (5·7 to 97·3%)
	Alzheimer's disease and other dementias	23·6% (10·4–40·2%)	87·5 (24·3–219·2)	38·7% (12·8 to 89·7%)	22·6% (9·8–38·8%)	5·4 (0·9–15·8)	35·7% (5·7 to 97·3%)
High fasting plasma glucose	Stroke	29·9% (18–47·8%)	432·5 (257·9–673·1)	-40·4% (-55·6 to -20·8%)	29·8% (16·2–53%)	22·5 (12·3–40·8)	-41·3% (-56·9 to -21·1%)
	Ischaemic stroke	30·6% (15·9–53·8%)	331·5 (170·6–568·2)	-37·3% (-53 to -17·1%)	30·1% (14·4–59·8%)	17·8 (8·4–35·4)	-38·7% (-55·2 to -18·3%)
	Intracerebral haemorrhage	28·2% (17·8–40·2%)	91·9 (54·7–139·4)	-49·8% (-63·6 to -30·7%)	29% (17·4–45%)	4·4 (2·5–7·1)	-49·7% (-64 to -29·1%)
	Subarachnoid haemorrhage	23·5% (15·4–33·3%)	9·2 (5·5–13·7)	-38·4% (-59·3 to -8·4%)	27·8% (16·9–41·5%)	0·3 (0·2–0·6)	-44·9% (-64·9 to -10·4%)
	Neurological disorders*	4·1% (0·7–11·5%)	51·9 (9·3–162·6)	27·1% (9·2 to 57·1%)	10·3% (2·3–23%)	3·3 (0·4–11·6)	25·4% (4·3 to 65·4%)
	Alzheimer's disease and other dementias	14% (3·5–28·8%)	51·9 (9·3–162·6)	27·1% (9·2 to 57·1%)	13·8% (3·5–28·5%)	3·3 (0·4–11·6)	25·4% (4·3 to 65·4%)
High LDL cholesterol	Stroke	18·3% (9·8–31·8%)	264·8 (137·4–468·4)	-43·4% (-53·7 to -32·7%)	16·6% (5·5–35%)	12·6 (4·1–26·6)	-44·1% (-56·9 to -32%)
	Ischaemic stroke	24·4% (13·2–42·5%)	264·8 (137·4–468·4)	-43·4% (-53·7 to -32·7%)	21·2% (7·1–44·3%)	12·6 (4·1–26·6)	-44·1% (-56·9 to -32%)
High systolic blood pressure	Stroke	52% (42·8–60·6%)	752·3 (585·1–924·9)	-49·4% (-60·1 to -37·2%)	48·2% (38·2–59·3%)	36·5 (27·4–47·9)	-49·3% (-61 to -35·2%)
	Ischaemic stroke	50% (39·2–60·5%)	541·9 (403·8–685·9)	-45% (-56·8 to -31·7%)	46·2% (34·6–58·9%)	27·4 (19–37·5)	-46% (-59·2 to -29·8%)
	Intracerebral haemorrhage	57·7% (47·6–67·7%)	188·1 (140·8–233·9)	-58·8% (-68·4 to -47·1%)	55·5% (43–67·8%)	8·5 (6·1–11)	-57·4% (-67·9 to -45%)
	Subarachnoid haemorrhage	57·4% (47·2–67%)	22·3 (16·5–28·5)	-47·6% (-62·8 to -23·7%)	55·7% (44·7–67·5%)	0·7 (0·5–0·9)	-53·5% (-69·4 to -27·3%)
High temperature	Stroke	0·4% (-0·3–1·4%)	6·2 (-4–19·8)	13·1% (-559·6 to 703%)	0·5% (-0·3–1·7%)	0·4 (-0·3–1·3)	24% (-609·2 to 760·5%)
	Ischaemic stroke	0·4% (-0·3–1·3%)	4·5 (-2·9–14·4)	21·8% (-599·5 to 774·1%)	0·5% (-0·3–1·7%)	0·3 (-0·2–1)	30·9% (-641·4 to 823·3%)
	Intracerebral haemorrhage	0·5% (-0·3–1·6%)	1·6 (-1–5·2)	-5·1% (-504·6 to 537·8%)	0·5% (-0·3–1·7%)	0·1 (-0·1–0·3)	3·8% (-531·2 to 583·1%)
	Subarachnoid haemorrhage	0·4% (-0·3–1·3%)	0·2 (-0·1–0·5)	3·9% (-534 to 653·2%)	0·5% (-0·3–1·7%)	<0·1 (<0·1–<0·1)	11% (-562·1 to 744·1%)
Household air pollution from solid fuels	Meningitis	0% (0–0%)	<0·1 (<0·1–<0·1)	-99·4% (-99·9 to -97·3%)	0% (0–0%)	<0·1 (<0·1–<0·1)	-99·4% (-99·9 to -97·3%)
	Encephalitis	0% (0–0%)	<0·1 (<0·1–<0·1)	-99·6% (-99·9 to -97·3%)	0% (0–0%)	<0·1 (<0·1–<0·1)	-99·6% (-99·9 to -97·3%)
	Stroke	0% (0–0%)	0·2 (0·1–0·4)	-99·3% (-99·7 to -98·1%)	0% (0–0%)	<0·1 (<0·1–<0·1)	-99·3% (-99·7 to -98·1%)
	Ischaemic stroke	0% (0–0%)	0·1 (0–0·3)	-99·2% (-99·7 to -97·9%)	0% (0–0%)	<0·1 (<0·1–<0·1)	-99·2% (-99·7 to -97·9%)
	Intracerebral haemorrhage	0% (0–0%)	<0·1 (<0·1–0·1)	-99·4% (-99·8 to -98·4%)	0% (0–0%)	<0·1 (<0·1–<0·1)	-99·4% (-99·8 to -98·4%)
	Subarachnoid haemorrhage	0% (0–0%)	<0·1 (<0·1–<0·1)	-99·2% (-99·7 to -97·9%)	0% (0–0%)	<0·1 (<0·1–<0·1)	-99·3% (-99·8 to -98·2%)
Kidney dysfunction	Stroke	12·9% (9·8–15·8%)	187·6 (135·7–240)	-33·7% (-47·2 to -20·5%)	11·7% (7·3–15·5%)	8·8 (5·3–12·3)	-34·2% (-50·6 to -18·3%)

Table S16 Regional and national disabilities and deaths related to neurological conditions attributable to risk factors in North Africa and Middle East countries

Location<br>Risk factor (alphabetic order)	Cause	DALYs (95% UI)<br>PAF (%)<br>2019	 Rate per 100,000<br>2019	 Rate change (%)<br>From 1990 to 2019	Deaths (95% UI)<br>PAF (%)<br>2019	 Rate per 100,000<br>2019	 Rate change (%)<br>From 1990 to 2019
	Ischaemic stroke	13·7% (9·7–17·3%)	148·9 (99·9–195·5)	-29·2% (-44·3 to -14·9%)	11·6% (6·1–16·2%)	6·9 (3·4–10)	-31·6% (-50·7 to -14·5%)
	Intracerebral haemorrhage	11·9% (9·4–14·5%)	38·7 (28·5–50·7)	-46·8% (-57·9 to -32·5%)	13% (9·6–16·4%)	2 (1·3–2·7)	-41·9% (-54·7 to -25%)
Lead exposure	Stroke	2·9% (1·2–4·7%)	41·4 (16·9–70)	-60·7% (-71 to -51·7%)	3% (1·4–4·8%)	2·3 (1–3·8)	-54·5% (-65·3 to -43·9%)
	Ischaemic stroke	2·8% (1·2–4·7%)	30·7 (12·3–52·1)	-55·4% (-66·6 to -45·7%)	2·9% (1·3–4·8%)	1·7 (0·8–3)	-50% (-62·7 to -37·1%)
	Intracerebral haemorrhage	3% (1–5·4%)	9·8 (3·3–17·9)	-70·9% (-79·9 to -61·9%)	3·4% (1·3–5·8%)	0·5 (0·2–0·9)	-64·7% (-74 to -54·4%)
	Subarachnoid haemorrhage	2·5% (0·7–4·8%)	1 (0·3–1·9)	-68·8% (-82·6 to -54·5%)	3·2% (1·2–5·6%)	<0·1 (<0·1–0·1)	-63·6% (-78·1 to -43·2%)
Low birth weight	Meningitis	5·4% (3·2–8·9%)	2·9 (1·6–5·2)	-61·4% (-82·9 to -9·4%)	3·4% (2–5·7%)	<0·1 (<0·1–0·1)	-61·4% (-82·9 to -9·4%)
	Encephalitis	1·4% (0·8–2·4%)	0·2 (0·1–0·3)	-73·4% (-90·7 to -6·1%)	1·1% (0·6–1·9%)	<0·1 (<0·1–<0·1)	-73·4% (-90·7 to -6·1%)
Low birth weight and short gestation	Meningitis	5·7% (3·4–9·4%)	3 (1·7–5·5)	-61·5% (-83·1 to -10·9%)	3·6% (2·1–6%)	<0·1 (<0·1–0·1)	-61·5% (-83·1 to -10·9%)
	Encephalitis	1·5% (0·8–2·5%)	0·2 (0·1–0·3)	-73·5% (-90·7 to -6·1%)	1·2% (0·7–2%)	<0·1 (<0·1–<0·1)	-73·5% (-90·7 to -6·1%)
Low physical activity	Stroke	5·2% (1·1–11·9%)	75 (15·3–171·4)	-36·9% (-51·2 to -17·5%)	6·5% (1·4–14%)	4·9 (1·1–10·6)	-38% (-52·5 to -17·1%)
	Ischaemic stroke	6·9% (1·5–15·9%)	75 (15·3–171·4)	-36·9% (-51·2 to -17·5%)	8·3% (1·8–17·9%)	4·9 (1·1–10·6)	-38% (-52·5 to -17·1%)
Low temperature	Stroke	5% (1·3–9·4%)	73 (17·3–141)	-54·4% (-64·8 to -42·3%)	6·3% (1·6–11·6%)	4·8 (1·1–9·1)	-50·1% (-61·2 to -37·7%)
	Ischaemic stroke	4·9% (1·2–9·1%)	52·7 (12·5–100·5)	-50·7% (-63·1 to -37·8%)	6·3% (1·6–11·6%)	3·7 (0·9–7)	-47·1% (-59·8 to -34·2%)
	Intracerebral haemorrhage	5·7% (1·4–10·6%)	18·5 (4·5–35·6)	-62% (-70·5 to -50·6%)	6·3% (1·6–11·6%)	1 (0·2–1·8)	-58·6% (-67·7 to -46·7%)
	Subarachnoid haemorrhage	4·5% (1·2–8·4%)	1·8 (0·4–3·4)	-58% (-72 to -33·6%)	6·3% (1·6–11·6%)	0·1 (0–0·1)	-55·1% (-69·9 to -28·8%)
Metabolic risk factors†	Stroke	76·3% (69–84·1%)	1104·2 (914–1305·6)	-47·6% (-56·9 to -37·3%)	72·1% (61·5–83·2%)	54·6 (43–66·8)	-47·4% (-57·7 to -35·1%)
	Ischaemic stroke	76·1% (66·8–86%)	824·1 (669·8–982·2)	-43·7% (-54·1 to -33·1%)	71·5% (59·2–85·6%)	42·4 (32·1–53·3)	-44·4% (-56·3 to -30·7%)
	Intracerebral haemorrhage	77·1% (69·8–83·5%)	251·1 (201·8–302·5)	-57·5% (-66 to -47·1%)	74·2% (65·2–82·5%)	11·3 (8·9–14)	-56% (-65·4 to -44·6%)
	Subarachnoid haemorrhage	74·5% (66·9–81·3%)	29 (22·4–35·8)	-45·9% (-60·9 to -21·4%)	71·1% (61·9–79·9%)	0·9 (0·6–1·1)	-52·4% (-67·9 to -26·1%)
	Neurological disorders*	10·1% (3·4–21·1%)	127·1 (40·6–314·5)	30·8% (10·5 to 64·5%)	24·7% (10·4–41·5%)	8 (1·4–23·7)	28·2% (4·9 to 72·3%)
	Alzheimer's disease and other dementias	34·3% (17·8–52·5%)	127·1 (40·6–314·5)	30·8% (10·5 to 64·5%)	33·2% (16·5–51·6%)	8 (1·4–23·7)	28·2% (4·9 to 72·3%)
Non-optimal temperature	Stroke	5·5% (2·1–9·3%)	78·9 (29·9–138·7)	-52·2% (-63·7 to -32·3%)	6·8% (2·6–11·5%)	5·1 (2–8·9)	-47·7% (-60·3 to -26·4%)
	Ischaemic stroke	5·3% (2–9·1%)	57 (21·5–99·4)	-48·4% (-61·8 to -28%)	6·8% (2·6–11·5%)	4 (1·5–7)	-44·7% (-58·5 to -23%)
	Intracerebral haemorrhage	6·1% (2·4–10·6%)	20 (7·6–35·7)	-60·2% (-69·6 to -43·2%)	6·8% (2·6–11·5%)	1 (0·4–1·8)	-56·7% (-66·8 to -37·8%)
	Subarachnoid haemorrhage	4·9% (1·9–8·4%)	1·9 (0·7–3·4)	-56% (-71·1 to -25·8%)	6·8% (2·6–11·5%)	0·1 (0–0·1)	-53% (-69·2 to -23·2%)
Other environmental risks	Stroke	2·9% (1·2–4·7%)	41·4 (16·9–70)	-60·7% (-71 to -51·7%)	3% (1·4–4·8%)	2·3 (1–3·8)	-54·5% (-65·3 to -43·9%)
	Ischaemic stroke	2·8% (1·2–4·7%)	30·7 (12·3–52·1)	-55·4% (-66·6 to -45·7%)	2·9% (1·3–4·8%)	1·7 (0·8–3)	-50% (-62·7 to -37·1%)
	Intracerebral haemorrhage	3% (1–5·4%)	9·8 (3·3–17·9)	-70·9% (-79·9 to -61·9%)	3·4% (1·3–5·8%)	0·5 (0·2–0·9)	-64·7% (-74 to -54·4%)
	Subarachnoid haemorrhage	2·5% (0·7–4·8%)	1 (0·3–1·9)	-68·8% (-82·6 to -54·5%)	3·2% (1·2–5·6%)	<0·1 (<0·1–0·1)	-63·6% (-78·1 to -43·2%)
Particulate matter pollution	Meningitis	0·8% (0·3–1·5%)	0·4 (0·2–0·8)	-59·9% (-87·5 to 36·2%)	0·5% (0·2–1%)	<0·1 (<0·1–<0·1)	-59·9% (-87·5 to 36·2%)
	Encephalitis	0·2% (0·1–0·4%)	<0·1 (<0·1–<0·1)	-73% (-93·5 to 37·6%)	0·2% (0·1–0·3%)	<0·1 (<0·1–<0·1)	-73% (-93·5 to 37·6%)

Table S16 Regional and national disabilities and deaths related to neurological conditions attributable to risk factors in North Africa and Middle East countries

**Location**<br>Risk factor (alphabetic order)	Cause	**DALYs (95% UI)**<br>PAF (%)<br>2019	Rate per 100,000<br>2019	Rate change (%)<br>From 1990 to 2019	**Deaths (95% UI)**<br>PAF (%)<br>2019	Rate per 100,000<br>2019	Rate change (%)<br>From 1990 to 2019
	Stroke	22·6% (19·1–26·2%)	328 (259·2–404·4)	-49·5% (-58·7 to -39·1%)	19·2% (16–22·3%)	14·6 (11·2–18·4)	-49·8% (-60·2 to -38·1%)
	Ischaemic stroke	21·8% (18·3–25·2%)	236·1 (185·7–292·6)	-45·1% (-55·3 to -34·3%)	18·5% (15·4–21·5%)	11 (8·3–13·9)	-46·3% (-58·2 to -33·7%)
	Intracerebral haemorrhage	25% (21·2–28·6%)	81·3 (63·9–102·3)	-59·3% (-67·5 to -48·8%)	21·6% (18·2–25%)	3·3 (2·5–4·2)	-58·3% (-67·3 to -46·9%)
	Subarachnoid haemorrhage	27·3% (23·3–31·3%)	10·6 (8–13·5)	-47·2% (-61·5 to -23·8%)	22·9% (19·4–26·5%)	0·3 (0·2–0·4)	-54·5% (-69·2 to -28·3%)
Secondhand smoke	Stroke	3·8% (2·8–4·8%)	55·1 (39–72·3)	-61·7% (-69 to -52·6%)	3·2% (2·3–4·1%)	2·4 (1·7–3·2)	-62% (-70·2 to -52·5%)
	Ischaemic stroke	3·5% (2·5–4·4%)	37·8 (26·4–50·2)	-58·3% (-67·1 to -48·8%)	3% (2·2–3·8%)	1·8 (1·2–2·4)	-59·6% (-68·7 to -49%)
	Intracerebral haemorrhage	4·7% (3·5–6%)	15·4 (10·7–20·5)	-68·1% (-75·3 to -58·9%)	3·9% (2·9–5%)	0·6 (0·4–0·8)	-67·7% (-75·2 to -58·3%)
	Subarachnoid haemorrhage	5·1% (3·8–6·5%)	2 (1·4–2·6)	-59·1% (-70·2 to -39·5%)	4·3% (3·2–5·5%)	0·1 (0–0·1)	-64% (-75·1 to -44·5%)
Short gestation	Meningitis	4·2% (2·5–6·8%)	2·2 (1·2–4)	-57·5% (-81·1 to -3·4%)	2·6% (1·5–4·3%)	<0·1 (<0·1–<0·1)	-57·5% (-81·1 to -3·4%)
	Encephalitis	1·1% (0·6–1·8%)	0·1 (0·1–0·2)	-70·9% (-89·7 to 4%)	0·8% (0·5–1·5%)	<0·1 (<0·1–<0·1)	-70·9% (-89·7 to 4%)
Smoking	Stroke	17·6% (15·8–19·5%)	255·7 (203·2–307·2)	-58·6% (-67·7 to -47·7%)	13·1% (11·5–14·8%)	9·9 (7·5–12·2)	-59·5% (-69·5 to -47·7%)
	Ischaemic stroke	16·4% (14·6–18·1%)	177·4 (140·6–214·7)	-55·3% (-65·2 to -44·5%)	12·1% (10·4–13·6%)	7·2 (5·3–8·8)	-56·7% (-67·4 to -43·8%)
	Intracerebral haemorrhage	21·3% (18·9–23·7%)	69·5 (54·9–86·3)	-65·4% (-74·2 to -54·8%)	16·8% (14·7–19%)	2·6 (2–3·2)	-65·6% (-74·4 to -54·8%)
	Subarachnoid haemorrhage	22·6% (19·4–25·3%)	8·8 (6·3–11·2)	-55·4% (-70·9 to -33·6%)	18·4% (15·3–21%)	0·2 (0·1–0·3)	-62% (-77·4 to -38·7%)
	Neurological disorders*	5·9% (2·1–12·6%)	75·3 (25·7–180·6)	-16·4% (-32·9 to 5%)	13·3% (4–21·7%)	4·5 (0·5–13·2)	-19·8% (-39·2 to 15·2%)
	Alzheimer's disease and other dementias	22·2% (14·7–29·6%)	81·9 (33·1–185·3)	-19·7% (-35·1 to -3·1%)	20·8% (13·5–28·5%)	5 (1·2–13·7)	-22·4% (-39·6 to -2·3%)
	Parkinson's disease	-13% (-19·7–-6·5%)	-9·7 (-15·4–-4·7)	-37·1% (-54·1 to -17·7%)	-11·5% (-17·9–-5·7%)	-0·6 (-0·9–-0·3)	-37·7% (-55·5 to -16·2%)
	Multiple sclerosis	15·8% (12·1–19·5%)	3 (2–4·1)	-9·9% (-29·5 to 16·2%)	17·2% (12·6–21·8%)	<0·1 (<0·1–<0·1)	-16·9% (-45·6 to 18·9%)
Tobacco	Stroke	20·7% (18·7–22·6%)	299·9 (240–359·4)	-58·8% (-67·6 to -49%)	15·8% (14–17·5%)	12 (9·2–14·6)	-59·7% (-69·4 to -48·8%)
	Ischaemic stroke	19·2% (17·3–21%)	208·2 (166·9–248·7)	-55·5% (-65·1 to -45·2%)	14·6% (12·9–16·3%)	8·7 (6·5–10·5)	-56·9% (-67·4 to -45·3%)
	Intracerebral haemorrhage	25% (22·6–27·4%)	81·4 (64·8–99·7)	-65·7% (-73·5 to -56%)	19·9% (17·8–22·1%)	3 (2·4–3·8)	-65·8% (-74 to -55·5%)
	Subarachnoid haemorrhage	26·5% (23·6–29·3%)	10·3 (7·5–12·9)	-55·7% (-70·3 to -35%)	21·8% (19–24·5%)	0·3 (0·2–0·4)	-62·1% (-76·2 to -40·3%)
	Neurological disorders*	5·9% (2·1–12·6%)	75·3 (25·7–180·6)	-16·4% (-32·9 to 5%)	13·3% (4–21·7%)	4·5 (0·5–13·2)	-19·8% (-39·2 to 15·2%)
	Alzheimer's disease and other dementias	22·2% (14·7–29·6%)	81·9 (33·1–185·3)	-19·7% (-35·1 to -3·1%)	20·8% (13·5–28·5%)	5 (1·2–13·7)	-22·4% (-39·6 to -2·3%)
	Parkinson's disease	-13% (-19·7–-6·5%)	-9·7 (-15·4–-4·7)	-37·1% (-54·1 to -17·7%)	-11·5% (-17·9–-5·7%)	-0·6 (-0·9–-0·3)	-37·7% (-55·5 to -16·2%)
	Multiple sclerosis	15·8% (12·1–19·5%)	3 (2–4·1)	-9·9% (-29·5 to 16·2%)	17·2% (12·6–21·8%)	<0·1 (<0·1–<0·1)	-16·9% (-45·6 to 18·9%)
**Kuwait**							
Air pollution	Meningitis	1·4% (0·8–2·3%)	0·3 (0·1–0·5)	-62·2% (-81·8 to -26·4%)	0·8% (0·4–1·3%)	<0·1 (<0·1–<0·1)	-62·2% (-81·8 to -26·4%)
	Encephalitis	0·5% (0·3–0·8%)	0·1 (0–0·1)	-11·4% (-61·6 to 92%)	0·4% (0·2–0·6%)	<0·1 (<0·1–<0·1)	-11·4% (-61·7 to 92%)
	Stroke	34·3% (30·5–37·8%)	321·6 (267·5–391·1)	-11·1% (-23·6 to 4·3%)	29·5% (26–32·9%)	13·7 (10·9–16·9)	-9·2% (-24·3 to 9·2%)
	Ischaemic stroke	33·1% (29·3–36·5%)	213·3 (175·1–262·4)	-12·7% (-24·7 to 1·9%)	28·4% (24·9–31·9%)	9·7 (7·6–12·1)	-10·7% (-25·7 to 8·2%)

Table S16 Regional and national disabilities and deaths related to neurological conditions attributable to risk factors in North Africa and Middle East countries

**Location**<br>Risk factor (alphabetic order)	Cause	**DALYs (95% UI)**<br>PAF (%)<br>2019	 Rate per 100,000<br>2019	 Rate change (%)<br>From 1990 to 2019	**Deaths (95% UI)**<br>PAF (%)<br>2019	 Rate per 100,000<br>2019	 Rate change (%)<br>From 1990 to 2019
	Intracerebral haemorrhage	36·9% (32·9–40·5%)	90·8 (73–113·2)	-2·2% (-21·6 to 21%)	32·4% (28·8–36%)	3·5 (2·7–4·4)	-1·3% (-23·9 to 25·3%)
	Subarachnoid haemorrhage	37·2% (33·5–40·9%)	17·5 (14·2–21·6)	-28·9% (-42·9 to -4·1%)	33·3% (29·5–36·9%)	0·5 (0·4–0·7)	-25·3% (-43·4 to 4·5%)
Alcohol use	Stroke	0% (-0·1–0·1%)	0 (-0·9–1·4)		0% (-0·1–0·1%)	<0·1 (<0·1–<0·1)	
	Ischaemic stroke	-0·1% (-0·2–0%)	-0·4 (-1·1–0·3)		-0·1% (-0·1–0%)	<0·1 (<0·1–<0·1)	
	Intracerebral haemorrhage	0·2% (0–0·6%)	0·4 (-0·1–1·4)		0·1% (0–0·4%)	<0·1 (<0·1–<0·1)	
	Neurological disorders*	0% (0–0·1%)	0·3 (0–1)		0% (0–0%)	<0·1 (<0·1–<0·1)	
	Idiopathic epilepsy	0·3% (0–0·6%)	0·3 (0–1)		0·3% (0–0·7%)	<0·1 (<0·1–<0·1)	
All risk factors	Meningitis	6·2% (3·9–8·6%)	1·2 (0·7–1·9)	-62·2% (-78·1 to -40·6%)	3·5% (2·2–5·1%)	<0·1 (<0·1–<0·1)	-62·2% (-78·1 to -40·6%)
	Encephalitis	2·3% (1·6–3·1%)	0·2 (0·2–0·3)	-12·2% (-45·8 to 46·1%)	1·6% (1·1–2·1%)	<0·1 (<0·1–<0·1)	-12·2% (-45·8 to 46·1%)
	Stroke	88·6% (85·4–91·7%)	831 (700–979)	-10·5% (-23·2 to 4·5%)	87·1% (82·2–92·2%)	40·5 (32·7–48·4)	-8·9% (-24 to 9·3%)
	Ischaemic stroke	88·9% (84·7–93·3%)	574 (476·1–674·1)	-11·8% (-24·1 to 2·7%)	87·1% (80·9–93·8%)	29·8 (23·9–36)	-10·2% (-25·8 to 7·4%)
	Intracerebral haemorrhage	88·3% (84·9–91%)	217·2 (176·9–260·5)	-2·2% (-22·3 to 21·2%)	87·4% (82·1–91·9%)	9·4 (7·4–11·5)	-2·2% (-25·2 to 24·3%)
	Subarachnoid haemorrhage	84·8% (81·4–87·8%)	39·9 (32·6–48·1)	-28·6% (-43·1 to -4%)	85·6% (80·4–89·9%)	1·3 (1·1–1·7)	-22·5% (-41·4 to 6·6%)
	Neurological disorders*	14·9% (6·1–29·3%)	189·1 (72·1–447·7)	14% (0·5 to 33·2%)	36·9% (19·5–53·9%)	11·8 (2·5–33·6)	16·5% (0·8 to 43·3%)
	Alzheimer's disease and other dementias	49·7% (33·6–65·4%)	192·7 (76·1–449·1)	11·4% (-1·6 to 27·8%)	48·1% (32·4–64·3%)	12·1 (2·8–33·9)	14·7% (-1·6 to 37·1%)
	Parkinson's disease	-10·5% (-15·4–-5·8%)	-5·9 (-9·1–-3·1)	-34·7% (-51·7 to -13·6%)	-9·9% (-14·5–-5·3%)	-0·3 (-0·5–-0·2)	-26·4% (-47 to -0·6%)
	Idiopathic epilepsy	0·3% (0–0·6%)	0·3 (0–1)		0·3% (0–0·7%)	<0·1 (<0·1–<0·1)	
	Multiple sclerosis	11·2% (7·8–14·7%)	1·9 (1·2–2·8)	3% (-15·4 to 26%)	12·8% (8·3–17·9%)	<0·1 (<0·1–<0·1)	-21·7% (-43·4 to 5·5%)
Ambient particulate matter pollution	Meningitis	1·4% (0·8–2·3%)	0·3 (0·1–0·5)	-61·4% (-81·6 to -24·7%)	0·8% (0·4–1·3%)	<0·1 (<0·1–<0·1)	-61·5% (-81·6 to -24·7%)
	Encephalitis	0·5% (0·3–0·8%)	0·1 (0–0·1)	-9·7% (-61 to 94·7%)	0·4% (0·2–0·6%)	<0·1 (<0·1–<0·1)	-9·7% (-61 to 94·8%)
	Stroke	34·3% (30·4–37·8%)	321·5 (267·4–391)	-9·7% (-22·2 to 6·4%)	29·5% (25·9–32·9%)	13·7 (10·9–16·9)	-7·7% (-22·6 to 11·2%)
	Ischaemic stroke	33% (29·2–36·5%)	213·2 (174·9–262·4)	-11·3% (-23·4 to 3·8%)	28·4% (24·9–31·9%)	9·7 (7·6–12·1)	-9·2% (-24·4 to 9·9%)
	Intracerebral haemorrhage	36·9% (32·9–40·5%)	90·8 (73–113·1)	-0·8% (-20·7 to 23%)	32·4% (28·7–35·9%)	3·5 (2·7–4·4)	0·2% (-22·7 to 27·3%)
	Subarachnoid haemorrhage	37·2% (33·5–40·9%)	17·5 (14·2–21·6)	-27·8% (-42·3 to -2·6%)	33·3% (29·5–36·9%)	0·5 (0·4–0·7)	-24·1% (-42·6 to 6·4%)
Behavioural risk factors‡	Meningitis	5·9% (3·8–8·3%)	1·2 (0·6–1·8)	-61·8% (-77·6 to -40·2%)	3·3% (2·1–4·9%)	<0·1 (<0·1–<0·1)	-61·8% (-77·6 to -40·1%)
	Encephalitis	2·2% (1·5–3%)	0·2 (0·1–0·3)	-11·2% (-44·5 to 47·3%)	1·5% (1·1–2%)	<0·1 (<0·1–<0·1)	-11·2% (-44·5 to 47·3%)
	Stroke	39·3% (33·3–45·8%)	368·8 (288·7–456)	-14·9% (-27·8 to 0·6%)	34·9% (28·3–41·8%)	16·2 (12·2–20·6)	-12·2% (-27·3 to 6·2%)
	Ischaemic stroke	39·7% (32·2–47·8%)	256 (194·4–321)	-16% (-28·1 to -0·9%)	35·6% (27·5–43·9%)	12·2 (8·9–15·8)	-12·9% (-28·2 to 5·9%)
	Intracerebral haemorrhage	38·8% (32·4–45·9%)	95·6 (72·3–121·8)	-6·8% (-26·5 to 17·3%)	33·1% (26·9–40·7%)	3·6 (2·6–4·7)	-5·6% (-28 to 21·6%)
	Subarachnoid haemorrhage	36·7% (30·2–43·5%)	17·2 (13·1–22·1)	-34·7% (-48·6 to -11·7%)	32·2% (26–39·8%)	0·5 (0·4–0·7)	-31·2% (-48·7 to -2·1%)
	Neurological disorders*	5·3% (2–11%)	67·9 (24·4–158·1)	-4·4% (-20·9 to 17·5%)	12·2% (5–19·4%)	4 (0·7–11·3)	1% (-20·2 to 34·9%)

Table S16 Regional and national disabilities and deaths related to neurological conditions attributable to risk factors in North Africa and Middle East countries

**Location**<br>Risk factor (alphabetic order)	Cause	**DALYs (95% UI)**<br>PAF (%)<br>2019	 Rate per 100,000<br>2019	 Rate change (%)<br>From 1990 to 2019	**Deaths (95% UI)**<br>PAF (%)<br>2019	 Rate per 100,000<br>2019	 Rate change (%)<br>From 1990 to 2019
	Alzheimer's disease and other dementias	18·4% (11·8–25·6%)	71·5 (28·7–161·3)	-8·6% (-24·7 to 10·3%)	17% (11–24%)	4·3 (1–11·6)	-1·9% (-22·4 to 22·7%)
	Parkinson's disease	-10·5% (-15·4–-5·8%)	-5·9 (-9·1–-3·1)	-34·7% (-51·7 to -13·6%)	-9·9% (-14·5–-5·3%)	-0·3 (-0·5–-0·2)	-26·4% (-47 to -0·6%)
	Idiopathic epilepsy	0·3% (0–0·6%)	0·3 (0–1)		0·3% (0–0·7%)	<0·1 (<0·1–<0·1)	
	Multiple sclerosis	11·2% (7·8–14·7%)	1·9 (1·2–2·8)	3% (-15·4 to 26%)	12·8% (8·3–17·9%)	<0·1 (<0·1–<0·1)	-21·7% (-43·4 to 5·5%)
Child and maternal malnutrition	Meningitis	5·9% (3·8–8·3%)	1·2 (0·6–1·8)	-61·8% (-77·6 to -40·2%)	3·3% (2·1–4·9%)	<0·1 (<0·1–<0·1)	-61·8% (-77·6 to -40·1%)
	Encephalitis	2·2% (1·5–3%)	0·2 (0·1–0·3)	-11·2% (-44·5 to 47·3%)	1·5% (1·1–2%)	<0·1 (<0·1–<0·1)	-11·2% (-44·5 to 47·3%)
Diet high in red meat	Stroke	7·8% (4·4–10·3%)	73·1 (40·2–101·2)	-16% (-29·2 to -0·4%)	5·8% (3·1–7·9%)	2·7 (1·4–3·9)	-12·2% (-29·4 to 9·5%)
	Ischaemic stroke	7% (2·6–9·6%)	44·9 (17·2–65·3)	-17·7% (-31·1 to -2·2%)	5·1% (1·8–7·2%)	1·7 (0·6–2·7)	-13·7% (-32·7 to 8·2%)
	Intracerebral haemorrhage	9·5% (3·8–13·5%)	23·5 (9·4–36)	-7·2% (-27·1 to 15·1%)	7·8% (2·9–11·2%)	0·8 (0·3–1·3)	-4·7% (-27·8 to 23·6%)
	Subarachnoid haemorrhage	10% (4–14·1%)	4·7 (1·8–7)	-34·6% (-49·6 to -11·1%)	8% (3–11·5%)	0·1 (0–0·2)	-31·8% (-50 to -2·4%)
Diet high in sodium	Stroke	3·9% (0·2–12·8%)	36·1 (2–120·8)	-3·9% (-41·8 to 45·7%)	3·3% (0·2–11·5%)	1·5 (0·1–5·4)	1·9% (-42·4 to 60·2%)
	Ischaemic stroke	3·5% (0·2–12·2%)	22·6 (1·4–78·8)	-5·2% (-39·7 to 49·4%)	3% (0·2–10·6%)	1 (0·1–3·8)	1·2% (-44·2 to 66·2%)
	Intracerebral haemorrhage	4·7% (0·2–15·4%)	11·5 (0·5–37)	4·6% (-42 to 62·8%)	4·1% (0·2–13·9%)	0·4 (0–1·5)	8·2% (-40·7 to 69·6%)
	Subarachnoid haemorrhage	4·1% (0·2–13·9%)	1·9 (0·1–6·6)	-27·9% (-59·8 to 12·2%)	3·9% (0·2–13·2%)	0·1 (0–0·2)	-21·6% (-56·3 to 23·4%)
Diet low in fibre	Stroke	2·7% (0·6–5·2%)	25 (5·3–49·1)	-38·4% (-51·7 to -24·1%)	2·2% (0·5–4·3%)	1 (0·2–2·1)	-39·1% (-54·8 to -21%)
	Ischaemic stroke	2·5% (0·5–4·9%)	16·1 (3·5–31·9)	-39·6% (-52·1 to -25·9%)	2·1% (0·5–4%)	0·7 (0·2–1·4)	-40·3% (-56·2 to -21·6%)
	Intracerebral haemorrhage	3% (0·6–5·9%)	7·4 (1·5–14·8)	-32% (-50 to -13·4%)	2·6% (0·6–5·1%)	0·3 (0·1–0·6)	-33·6% (-53·5 to -11·2%)
	Subarachnoid haemorrhage	3·1% (0·6–6·2%)	1·5 (0·3–3)	-51·3% (-64·6 to -29·9%)	2·6% (0·6–5·2%)	<0·1 (<0·1–0·1)	-49·4% (-65·3 to -26·5%)
Diet low in fruits	Stroke	4·8% (2·1–7·9%)	45·3 (19–76·4)	-4% (-20·2 to 17·1%)	3·7% (1·6–6·2%)	1·7 (0·7–3)	-4·7% (-23·9 to 21·2%)
	Ischaemic stroke	3·8% (0·8–7·5%)	24·8 (4·8–49)	-5·6% (-22·1 to 24·2%)	2·9% (0·6–5·7%)	1 (0·2–2)	-7·2% (-26·8 to 25·2%)
	Intracerebral haemorrhage	7% (3·1–13·1%)	17·1 (7·5–33)	4·6% (-17·7 to 35·3%)	5·8% (2·6–11%)	0·6 (0·3–1·2)	3·3% (-21·6 to 36·3%)
	Subarachnoid haemorrhage	7·3% (3·3–13·6%)	3·4 (1·5–6·5)	-25·5% (-41·2 to 6·3%)	6% (2·7–11·3%)	0·1 (0–0·2)	-22·5% (-42·1 to 10·2%)
Diet low in vegetables	Stroke	0·4% (0·2–0·9%)	4 (1·9–8·5)	-51·5% (-67·1 to -16%)	0·5% (0·2–0·9%)	0·2 (0·1–0·4)	-51·3% (-69·3 to -16·4%)
	Ischaemic stroke	0·4% (0·2–0·9%)	2·8 (1·2–5·9)	-51·2% (-66·7 to -10·2%)	0·5% (0·2–0·9%)	0·2 (0·1–0·3)	-50·9% (-69·7 to -7·3%)
	Intracerebral haemorrhage	0·4% (0·2–1·3%)	1·1 (0·4–3·4)	-49·5% (-68·5 to 7%)	0·5% (0·2–1·5%)	0·1 (0–0·2)	-51·5% (-71·4 to 4·5%)
	Subarachnoid haemorrhage	0·4% (0·2–1·2%)	0·2 (0·1–0·6)	-62·9% (-77·8 to -14·1%)	0·5% (0·2–1·4%)	<0·1 (<0·1–<0·1)	-58·7% (-75·2 to -7·9%)
Diet low in whole grains	Stroke	3·6% (0·9–5·4%)	34·2 (8·4–52·4)	-15·5% (-26·9 to -2·2%)	3·2% (0·8–4·6%)	1·5 (0·4–2·3)	-14·2% (-28·9 to 2·9%)
	Ischaemic stroke	5·3% (1·3–7·8%)	34·2 (8·4–52·4)	-15·5% (-26·9 to -2·2%)	4·3% (1·1–6·3%)	1·5 (0·4–2·3)	-14·2% (-28·9 to 2·9%)
Dietary risks	Stroke	20·8% (14·7–28·5%)	195 (133·6–276·9)	-15·8% (-29·1 to -0·1%)	17·2% (11·5–24·4%)	8 (5·1–11·6)	-14·5% (-30·1 to 5%)
	Ischaemic stroke	20·2% (12·9–28%)	130 (81·4–186·5)	-17·3% (-29·8 to -1%)	16·5% (10·1–23·9%)	5·6 (3·4–8·4)	-16% (-31·8 to 4·7%)
	Intracerebral haemorrhage	22·2% (13·9–31·1%)	54·6 (32·6–79·4)	-6·9% (-27·5 to 17·7%)	19·1% (11·6–28·6%)	2·1 (1·2–3·1)	-7% (-29·9 to 20·5%)
	Subarachnoid haemorrhage	22·3% (14·2–31·1%)	10·5 (6·5–15·2)	-33·8% (-47·8 to -10·8%)	19·2% (11·7–28·3%)	0·3 (0·2–0·5)	-30·5% (-48·4 to -2·5%)

Table S16 Regional and national disabilities and deaths related to neurological conditions attributable to risk factors in North Africa and Middle East countries

**Location**<br>Risk factor (alphabetic order)	Cause	**DALYs (95% UI)**<br>PAF (%)<br>2019	 Rate per 100,000<br>2019	 Rate change (%)<br>From 1990 to 2019	**Deaths (95% UI)**<br>PAF (%)<br>2019	 Rate per 100,000<br>2019	 Rate change (%)<br>From 1990 to 2019
Environmental/occupational risk factors§	Meningitis	1·4% (0·8–2·3%)	0·3 (0·1–0·5)	-62·2% (-81·8 to -26·4%)	0·8% (0·4–1·3%)	<0·1 (<0·1–<0·1)	-62·2% (-81·8 to -26·4%)
	Encephalitis	0·5% (0·3–0·8%)	0·1 (0–0·1)	-11·4% (-61·6 to 92%)	0·4% (0·2–0·6%)	<0·1 (<0·1–<0·1)	-11·4% (-61·7 to 92%)
	Stroke	40·7% (35·9–45·4%)	382·1 (311·4–464)	-11·2% (-23·9 to 5·1%)	37·8% (32·1–43·2%)	17·6 (13·3–22·1)	-8% (-23·6 to 12·3%)
	Ischaemic stroke	39·4% (34·7–44%)	254·5 (206·7–311·3)	-12·3% (-24·8 to 4·1%)	36·8% (30·9–42·3%)	12·6 (9·3–15·9)	-9·2% (-24·7 to 11·7%)
	Intracerebral haemorrhage	43·6% (38·4–48·6%)	107·4 (83·5–134·6)	-2·9% (-22·9 to 21·2%)	40·5% (34·9–46%)	4·4 (3·3–5·5)	-1·1% (-24 to 26%)
	Subarachnoid haemorrhage	43·1% (38·4–47·7%)	20·2 (16·2–25)	-30·9% (-44·6 to -5·3%)	41·2% (35·5–46·5%)	0·6 (0·5–0·8)	-24·6% (-43·1 to 5·6%)
High body-mass index	Stroke	39·3% (28·7–48·3%)	368·9 (255·9–482·7)	2·1% (-14·4 to 21·5%)	25·5% (16·7–34·2%)	11·9 (7·3–16·8)	8·1% (-13·7 to 35·6%)
	Ischaemic stroke	33% (22·8–42·2%)	212·7 (143·2–291·1)	2·8% (-12·9 to 23%)	21·2% (13–30·2%)	7·2 (4·2–10·8)	7·8% (-13·2 to 36·9%)
	Intracerebral haemorrhage	52·6% (40·3–63·1%)	129·5 (93·4–167·6)	9·2% (-13·4 to 36%)	36·9% (25·5–48·8%)	4 (2·6–5·6)	16·1% (-10·9 to 50·7%)
	Subarachnoid haemorrhage	57% (45–66·3%)	26·8 (19·8–34·2)	-25·8% (-41·1 to 4·8%)	41·6% (29·8–53%)	0·7 (0·4–0·9)	-22·4% (-42·3 to 12·6%)
	Neurological disorders*	7·6% (2·4–16·4%)	95·9 (27·3–233·5)	29% (6·1 to 67·6%)	18·8% (7·4–32·8%)	5·9 (1·1–17)	29·1% (0·6 to 79·7%)
	Alzheimer's disease and other dementias	24·7% (10·9–41·2%)	95·9 (27·3–233·5)	29% (6·1 to 67·6%)	23·6% (10·2–39·8%)	5·9 (1·1–17)	29·1% (0·6 to 79·7%)
High fasting plasma glucose	Stroke	30% (19·4–46·4%)	281·1 (178·1–446·7)	-6% (-26·1 to 22·2%)	30·2% (17·9–51·8%)	14·1 (8·3–25·4)	-9·6% (-29 to 17·5%)
	Ischaemic stroke	31·1% (17·8–54·2%)	200·8 (110·8–352·1)	-10·2% (-29 to 17·6%)	30·7% (15·7–60·5%)	10·5 (5·1–21·2)	-12·4% (-31·4 to 14·4%)
	Intracerebral haemorrhage	27·7% (18·3–39·5%)	68·3 (42·5–102·4)	9·8% (-19·4 to 49%)	29% (18·1–45·4%)	3·1 (1·8–5)	1·7% (-26 to 42·7%)
	Subarachnoid haemorrhage	25·4% (16·9–35·4%)	11·9 (7·4–17·2)	-9·6% (-32·7 to 25·1%)	29·2% (18·4–44·1%)	0·5 (0·3–0·7)	-12% (-37·8 to 28·5%)
	Neurological disorders*	5·5% (1·1–14·5%)	69·3 (13·2–210·6)	32% (17 to 58·8%)	14·3% (3·4–30%)	4·5 (0·6–15·4)	32·8% (13·7 to 67%)
	Alzheimer's disease and other dementias	17·8% (4·7–35·7%)	69·3 (13·2–210·6)	32% (17 to 58·8%)	18% (4·7–35·9%)	4·5 (0·6–15·4)	32·8% (13·7 to 67%)
High LDL cholesterol	Stroke	17·5% (10–29·5%)	164 (90·3–282·1)	-11% (-24·9 to 3·1%)	15·7% (5·4–32·1%)	7·3 (2·5–15·7)	-9·6% (-26·7 to 7·6%)
	Ischaemic stroke	25·4% (14·4–42·8%)	164 (90·3–282·1)	-11% (-24·9 to 3·1%)	21·4% (7·4–44·2%)	7·3 (2·5–15·7)	-9·6% (-26·7 to 7·6%)
High systolic blood pressure	Stroke	51% (43·1–59%)	478·8 (375·2–590·9)	-10·3% (-26·2 to 8·7%)	49·4% (39·7–60·2%)	23 (16·8–29·9)	-9·3% (-27·3 to 11·9%)
	Ischaemic stroke	49·3% (38·6–59·7%)	318·5 (236–406·5)	-12·4% (-28 to 5·6%)	47·4% (35·1–60·9%)	16·2 (11·3–22·1)	-10·9% (-28·9 to 11·3%)
	Intracerebral haemorrhage	55% (45·6–64·2%)	135·5 (100·6–173)	-1·1% (-24 to 24·2%)	55% (42·7–67%)	5·9 (4·1–7·8)	-2·2% (-26·7 to 27%)
	Subarachnoid haemorrhage	52·7% (43·7–61·7%)	24·8 (18·6–31·4)	-25·5% (-42·3 to 2·4%)	54·8% (43·6–65·9%)	0·9 (0·6–1·1)	-20·7% (-41·9 to 12·7%)
High temperature	Stroke	3·9% (1–6·5%)	36·8 (8·6–62·9)	-4·8% (-31·4 to 22·4%)	4·9% (1·2–8%)	2·3 (0·6–3·9)	-1·2% (-31 to 28·1%)
	Ischaemic stroke	3·7% (1–6·3%)	24·2 (5·8–41·9)	-5·4% (-35·4 to 24%)	4·9% (1·2–8%)	1·7 (0·4–2·9)	-2·3% (-33·8 to 26·8%)
	Intracerebral haemorrhage	4·4% (1·1–7·2%)	10·8 (2·5–18·8)	4·9% (-29·7 to 42·8%)	4·9% (1·2–8%)	0·5 (0·1–0·9)	5·4% (-29·7 to 44·7%)
	Subarachnoid haemorrhage	3·8% (1–6·3%)	1·8 (0·5–3·2)	-35·4% (-56·6 to 3·2%)	4·9% (1·2–8%)	0·1 (0–0·1)	-17·7% (-44·7 to 23·4%)
Household air pollution from solid fuels	Meningitis	0% (0–0%)	<0·1 (<0·1–<0·1)	-99% (-99·7 to -97%)	0% (0–0%)	<0·1 (<0·1–<0·1)	-99% (-99·7 to -97%)
	Encephalitis	0% (0–0%)	<0·1 (<0·1–<0·1)	-97·6% (-99·4 to -92·6%)	0% (0–0%)	<0·1 (<0·1–<0·1)	-97·6% (-99·4 to -92·6%)
	Stroke	0% (0–0%)	0·1 (0–0·4)	-97·8% (-99·2 to -94·9%)	0% (0–0%)	<0·1 (<0·1–<0·1)	-97·8% (-99·2 to -94·8%)

Table S16 Regional and national disabilities and deaths related to neurological conditions attributable to risk factors in North Africa and Middle East countries

Location<br>Risk factor (alphabetic order)	Cause	DALYs (95% UI)<br>PAF (%)<br>2019	Rate per 100,000<br>2019	Rate change (%)<br>From 1990 to 2019	Deaths (95% UI)<br>PAF (%)<br>2019	Rate per 100,000<br>2019	Rate change (%)<br>From 1990 to 2019
	Ischaemic stroke	0% (0–0%)	0·1 (0–0·2)	-97·9% (-99·2 to -95%)	0% (0–0%)	<0·1 (<0·1–<0·1)	-97·9% (-99·2 to -95%)
	Intracerebral haemorrhage	0% (0–0%)	<0·1 (<0·1–0·1)	-97·6% (-99·1 to -94·1%)	0% (0–0%)	<0·1 (<0·1–<0·1)	-97·6% (-99·1 to -94·2%)
	Subarachnoid haemorrhage	0% (0–0%)	<0·1 (<0·1–<0·1)	-98·1% (-99·3 to -95·4%)	0% (0–0%)	<0·1 (<0·1–<0·1)	-98·1% (-99·3 to -95·4%)
Kidney dysfunction	Stroke	11·1% (8·4–13·6%)	104·4 (74·3–135)	10·3% (-7·1 to 31·3%)	10·4% (6·6–14%)	4·9 (2·9–6·8)	13·9% (-8·4 to 40·4%)
	Ischaemic stroke	12·4% (8·7–15·7%)	80 (54·7–106·6)	8·1% (-9·4 to 29%)	10·6% (5·6–15·4%)	3·6 (1·9–5·4)	11·1% (-13·1 to 38·6%)
	Intracerebral haemorrhage	9·9% (8–11·9%)	24·4 (17·9–32·3)	18·5% (-6·9 to 49·2%)	11·5% (8·1–14·6%)	1·2 (0·8–1·7)	23% (-6·8 to 58·5%)
Lead exposure	Stroke	2·7% (1·1–4·6%)	25·8 (10·3–44·2)	-27·7% (-43·4 to -12·5%)	3% (1·4–4·8%)	1·4 (0·6–2·4)	-10·9% (-27·3 to 8·9%)
	Ischaemic stroke	2·8% (1·1–4·7%)	17·8 (7·4–30·3)	-24·1% (-39·7 to -8%)	2·9% (1·3–4·8%)	1 (0·4–1·7)	-8·4% (-25·3 to 13·4%)
	Intracerebral haemorrhage	2·8% (0·9–5·1%)	6·9 (2·3–12·7)	-30·2% (-52·3 to -8·2%)	3·3% (1·3–5·7%)	0·4 (0·1–0·6)	-13·1% (-35·5 to 11·2%)
	Subarachnoid haemorrhage	2·3% (0·7–4·4%)	1·1 (0·3–2·1)	-53·6% (-70·9 to -36·8%)	3% (1·1–5·3%)	<0·1 (<0·1–0·1)	-35·7% (-56·2 to -8·5%)
Low birth weight	Meningitis	5·4% (3·5–7·6%)	1·1 (0·6–1·6)	-61·8% (-77·9 to -40%)	3·1% (1·9–4·5%)	<0·1 (<0·1–<0·1)	-61·8% (-77·9 to -40%)
	Encephalitis	2% (1·4–2·7%)	0·2 (0·1–0·3)	-11·9% (-45·1 to 46·8%)	1·4% (1–1·9%)	<0·1 (<0·1–<0·1)	-11·9% (-45·1 to 46·8%)
Low birth weight and short gestation	Meningitis	5·9% (3·8–8·3%)	1·2 (0·6–1·8)	-61·8% (-77·6 to -40·2%)	3·3% (2·1–4·9%)	<0·1 (<0·1–<0·1)	-61·8% (-77·6 to -40·1%)
	Encephalitis	2·2% (1·5–3%)	0·2 (0·1–0·3)	-11·2% (-44·5 to 47·3%)	1·5% (1·1–2%)	<0·1 (<0·1–<0·1)	-11·2% (-44·5 to 47·3%)
Low physical activity	Stroke	6·8% (1·5–13·9%)	64 (14·5–129·7)	-8·2% (-22·2 to 9·2%)	8·4% (2·1–15·7%)	3·9 (1–7·5)	-9·3% (-25·2 to 10·1%)
	Ischaemic stroke	9·9% (2·2–20·1%)	64 (14·5–129·7)	-8·2% (-22·2 to 9·2%)	11·4% (2·8–21·4%)	3·9 (1–7·5)	-9·3% (-25·2 to 10·1%)
Low temperature	Stroke	3·4% (-2·9–9%)	32·1 (-26–87·7)	-5·5% (-49 to 52·8%)	4·3% (-3·6–11·1%)	2 (-1·6–5·4)	-2% (-47 to 62·2%)
	Ischaemic stroke	3·3% (-2·7–8·6%)	21·1 (-16·9–57·8)	-5·9% (-51·6 to 50·9%)	4·3% (-3·6–11·1%)	1·5 (-1·2–4)	-2·9% (-49·3 to 60·5%)
	Intracerebral haemorrhage	3·8% (-3·2–10%)	9·4 (-8–25·9)	4% (-46·8 to 65·5%)	4·3% (-3·6–11·1%)	0·5 (-0·4–1·2)	4·2% (-49·4 to 67·6%)
	Subarachnoid haemorrhage	3·3% (-2·7–8·7%)	1·6 (-1·3–4·3)	-36·2% (-64·1 to 16·7%)	4·3% (-3·6–11·1%)	0·1 (-0·1–0·2)	-18·8% (-54·6 to 36·5%)
Metabolic risk factors†	Stroke	75·8% (68·9–82·5%)	711·1 (587·5–850·4)	-8·9% (-22·2 to 7%)	72·7% (63–82·5%)	33·8 (26·2–42)	-7·9% (-23·3 to 11·9%)
	Ischaemic stroke	76·1% (67·2–85·4%)	491·4 (396·8–587·5)	-10·7% (-23·9 to 4·4%)	72·4% (59·9–86%)	24·7 (18·8–31·9)	-9·5% (-25·4 to 9·1%)
	Intracerebral haemorrhage	75·6% (68·1–81·7%)	186·1 (147·2–226·6)	0·6% (-20·7 to 25·1%)	73·9% (64·3–82·2%)	8 (6·1–10)	-0·1% (-24·2 to 27·8%)
	Subarachnoid haemorrhage	71·5% (63·8–77·7%)	33·6 (26·8–40·9)	-25·9% (-41·4 to 0·3%)	71·1% (61·5–79·2%)	1·1 (0·8–1·4)	-20·9% (-41·1 to 9·2%)
	Neurological disorders*	11·7% (4·1–24·2%)	148 (49–360·4)	26·5% (10·7 to 56·2%)	29·7% (13·2–47·7%)	9·4 (1·7–27·1)	27% (7 to 61·6%)
	Alzheimer's disease and other dementias	38·2% (19·5–57·6%)	148 (49–360·4)	26·5% (10·7 to 56·2%)	37·3% (19·1–56·7%)	9·4 (1·7–27·1)	27% (7 to 61·6%)
Non-optimal temperature	Stroke	7·2% (1·2–12·6%)	67·5 (11–121·2)	-5·3% (-40·9 to 21·7%)	9% (1·5–15·7%)	4·2 (0·7–7·6)	-1·8% (-38·6 to 25·5%)
	Ischaemic stroke	6·9% (1·1–12%)	44·4 (7·1–80·1)	-5·8% (-42·8 to 20·7%)	9% (1·5–15·7%)	3·1 (0·5–5·6)	-2·7% (-39·3 to 24·8%)
	Intracerebral haemorrhage	8% (1·4–14%)	19·8 (3·1–36·1)	4·3% (-37·1 to 37·9%)	9% (1·5–15·7%)	1 (0·1–1·8)	4·7% (-37·1 to 40·2%)
	Subarachnoid haemorrhage	7% (1·1–12·2%)	3·3 (0·5–5·9)	-35·9% (-58·2 to -1·5%)	9% (1·5–15·7%)	0·1 (0–0·3)	-18·4% (-47·5 to 19·4%)
Other environmental risks	Stroke	2·7% (1·1–4·6%)	25·8 (10·3–44·2)	-27·7% (-43·4 to -12·5%)	3% (1·4–4·8%)	1·4 (0·6–2·4)	-10·9% (-27·3 to 8·9%)
	Ischaemic stroke	2·8% (1·1–4·7%)	17·8 (7·4–30·3)	-24·1% (-39·7 to -8%)	2·9% (1·3–4·8%)	1 (0·4–1·7)	-8·4% (-25·3 to 13·4%)

Table S16 Regional and national disabilities and deaths related to neurological conditions attributable to risk factors in North Africa and Middle East countries

**Location**<br>Risk factor (alphabetic order)	Cause	**DALYs (95% UI)**<br>PAF (%)<br>2019	Rate per 100,000<br>2019	Rate change (%)<br>From 1990 to 2019	**Deaths (95% UI)**<br>PAF (%)<br>2019	Rate per 100,000<br>2019	Rate change (%)<br>From 1990 to 2019
	Intracerebral haemorrhage	2·8% (0·9–5·1%)	6·9 (2·3–12·7)	-30·2% (-52·3 to -8·2%)	3·3% (1·3–5·7%)	0·4 (0·1–0·6)	-13·1% (-35·5 to 11·2%)
	Subarachnoid haemorrhage	2·3% (0·7–4·4%)	1·1 (0·3–2·1)	-53·6% (-70·9 to -36·8%)	3% (1·1–5·3%)	<0·1 (<0·1–0·1)	-35·7% (-56·2 to -8·5%)
Particulate matter pollution	Meningitis	1·4% (0·8–2·3%)	0·3 (0·1–0·5)	-62·2% (-81·8 to -26·4%)	0·8% (0·4–1·3%)	<0·1 (<0·1–<0·1)	-62·2% (-81·8 to -26·4%)
	Encephalitis	0·5% (0·3–0·8%)	0·1 (0–0·1)	-11·4% (-61·6 to 92%)	0·4% (0·2–0·6%)	<0·1 (<0·1–<0·1)	-11·4% (-61·7 to 92%)
	Stroke	34·3% (30·5–37·8%)	321·6 (267·5–391·1)	-11·1% (-23·6 to 4·3%)	29·5% (26–32·9%)	13·7 (10·9–16·9)	-9·2% (-24·3 to 9·2%)
	Ischaemic stroke	33·1% (29·3–36·5%)	213·3 (175·1–262·4)	-12·7% (-24·7 to 1·9%)	28·4% (24·9–31·9%)	9·7 (7·6–12·1)	-10·7% (-25·7 to 8·2%)
	Intracerebral haemorrhage	36·9% (32·9–40·5%)	90·8 (73–113·2)	-2·2% (-21·6 to 21%)	32·4% (28·8–36%)	3·5 (2·7–4·4)	-1·3% (-23·9 to 25·3%)
	Subarachnoid haemorrhage	37·2% (33·5–40·9%)	17·5 (14·2–21·6)	-28·9% (-42·9 to -4·1%)	33·3% (29·5–36·9%)	0·5 (0·4–0·7)	-25·3% (-43·4 to 4·5%)
Secondhand smoke	Stroke	3·7% (2·8–4·6%)	34·8 (25·3–45·4)	-17·8% (-31·8 to -1·1%)	3·2% (2·4–4%)	1·5 (1–2)	-17·3% (-33·5 to 1%)
	Ischaemic stroke	3·4% (2·6–4·3%)	22·1 (15·8–29)	-19·7% (-32·8 to -3·4%)	3% (2·2–3·9%)	1 (0·7–1·4)	-19·2% (-34·7 to -0·4%)
	Intracerebral haemorrhage	4·3% (3·2–5·3%)	10·5 (7·5–13·8)	-8·7% (-29·3 to 15·3%)	3·6% (2·7–4·5%)	0·4 (0·3–0·5)	-8·8% (-31·8 to 17·5%)
	Subarachnoid haemorrhage	4·7% (3·6–6%)	2·2 (1·6–3)	-33·7% (-48·7 to -7·8%)	4·2% (3·2–5·2%)	0·1 (0–0·1)	-30·9% (-49·2 to -1·9%)
Short gestation	Meningitis	4·6% (3–6·4%)	0·9 (0·5–1·4)	-58% (-75·4 to -34·3%)	2·6% (1·6–3·8%)	<0·1 (<0·1–<0·1)	-58% (-75·4 to -34·3%)
	Encephalitis	1·7% (1·2–2·3%)	0·2 (0·1–0·3)	-1·4% (-38 to 63·2%)	1·2% (0·8–1·6%)	<0·1 (<0·1–<0·1)	-1·4% (-38 to 63·2%)
Smoking	Stroke	15·4% (13·9–17%)	144·9 (117·3–176·4)	-18·7% (-34·1 to 0·3%)	11·7% (10·3–13·3%)	5·4 (4·3–6·8)	-11·2% (-30·5 to 12·7%)
	Ischaemic stroke	14% (12·5–15·5%)	90·3 (72–109·9)	-20·8% (-35·5 to -1·6%)	10·5% (9·1–12·1%)	3·6 (2·8–4·5)	-11·8% (-32·2 to 13·9%)
	Intracerebral haemorrhage	19·1% (17·2–21%)	47·1 (37–59·1)	-8·9% (-30·2 to 18·1%)	15·1% (13·4–16·8%)	1·6 (1·2–2·1)	-5% (-28·8 to 27·7%)
	Subarachnoid haemorrhage	15·8% (13·8–17·8%)	7·4 (5·8–9·4)	-39·8% (-54·5 to -15·5%)	13·4% (11·4–15·4%)	0·2 (0·2–0·3)	-36·6% (-55·3 to -4·7%)
	Neurological disorders*	5·3% (1·9–11%)	67·6 (24–157·1)	-4·9% (-21·1 to 17·1%)	12·2% (5–19·4%)	4 (0·7–11·3)	0·9% (-20·3 to 34·8%)
	Alzheimer's disease and other dementias	18·4% (11·8–25·6%)	71·5 (28·7–161·3)	-8·6% (-24·7 to 10·3%)	17% (11–24%)	4·3 (1–11·6)	-1·9% (-22·4 to 22·7%)
	Parkinson's disease	-10·5% (-15·4–-5·8%)	-5·9 (-9·1–-3·1)	-34·7% (-51·7 to -13·6%)	-9·9% (-14·5–-5·3%)	-0·3 (-0·5–-0·2)	-26·4% (-47 to -0·6%)
	Multiple sclerosis	11·2% (7·8–14·7%)	1·9 (1·2–2·8)	3% (-15·4 to 26%)	12·8% (8·3–17·9%)	<0·1 (<0·1–<0·1)	-21·7% (-43·4 to 5·5%)
Tobacco	Stroke	18·5% (16·9–20·2%)	174·1 (144·2–209·6)	-18·3% (-33·4 to -0·4%)	14·4% (12·9–16·1%)	6·7 (5·4–8·3)	-12·4% (-29·8 to 10·1%)
	Ischaemic stroke	16·9% (15·3–18·6%)	109·1 (88·9–132·3)	-20·4% (-34·5 to -2·6%)	13·2% (11·6–14·8%)	4·5 (3·6–5·7)	-13·3% (-31·9 to 10%)
	Intracerebral haemorrhage	22·6% (20·6–24·6%)	55·6 (44·2–69)	-8·7% (-29·5 to 17·2%)	18·1% (16·2–20%)	1·9 (1·5–2·5)	-5·6% (-29·2 to 23·3%)
	Subarachnoid haemorrhage	19·8% (17·9–21·9%)	9·3 (7·4–11·5)	-38·3% (-52·6 to -12·9%)	16·9% (14·9–19·1%)	0·3 (0·2–0·3)	-35% (-52·6 to -5·2%)
	Neurological disorders*	5·3% (1·9–11%)	67·6 (24–157·1)	-4·9% (-21·1 to 17·1%)	12·2% (5–19·4%)	4 (0·7–11·3)	0·9% (-20·3 to 34·8%)
	Alzheimer's disease and other dementias	18·4% (11·8–25·6%)	71·5 (28·7–161·3)	-8·6% (-24·7 to 10·3%)	17% (11–24%)	4·3 (1–11·6)	-1·9% (-22·4 to 22·7%)
	Parkinson's disease	-10·5% (-15·4–-5·8%)	-5·9 (-9·1–-3·1)	-34·7% (-51·7 to -13·6%)	-9·9% (-14·5–-5·3%)	-0·3 (-0·5–-0·2)	-26·4% (-47 to -0·6%)
	Multiple sclerosis	11·2% (7·8–14·7%)	1·9 (1·2–2·8)	3% (-15·4 to 26%)	12·8% (8·3–17·9%)	<0·1 (<0·1–<0·1)	-21·7% (-43·4 to 5·5%)

Table S16 Regional and national disabilities and deaths related to neurological conditions attributable to risk factors in North Africa and Middle East countries

Location<br>Risk factor (alphabetic order)	Cause	DALYs (95% UI)<br>PAF (%)<br>2019	 Rate per 100,000<br>2019	 Rate change (%)<br>From 1990 to 2019	Deaths (95% UI)<br>PAF (%)<br>2019	 Rate per 100,000<br>2019	 Rate change (%)<br>From 1990 to 2019
**Lebanon**							
Air pollution	Meningitis	0·4% (0·1–1%)	0·1 (0–0·3)	-85·2% (-96·5 to -37·5%)	0·2% (0·1–0·5%)	<0·1 (<0·1–<0·1)	-85·2% (-96·5 to -37·6%)
	Encephalitis	0·4% (0·1–0·9%)	0·1 (0–0·2)	-23·6% (-84·9 to 286·8%)	0·2% (0·1–0·6%)	<0·1 (<0·1–<0·1)	-23·6% (-84·9 to 287·1%)
	Stroke	22·1% (17·6–26·7%)	166·1 (115·9–215·8)	-32·7% (-47·6 to -14·7%)	17·8% (14·1–21·9%)	6·3 (4–8·5)	-39·8% (-55·7 to -18·5%)
	Ischaemic stroke	21·3% (16·9–25·9%)	127·6 (89·1–165·7)	-10·9% (-30·8 to 10·6%)	17·3% (13·6–21·3%)	5·1 (3·3–6·9)	-22·9% (-45·9 to 3·1%)
	Intracerebral haemorrhage	24·5% (19·7–29·5%)	31·5 (21·1–45·4)	-63·1% (-73·9 to -48·6%)	20·6% (16·2–25·1%)	1 (0·7–1·5)	-68·3% (-78 to -53·6%)
	Subarachnoid haemorrhage	26·5% (21·3–31·8%)	7·1 (4·6–10·2)	-61% (-76·8 to -36·2%)	21·6% (17–26·3%)	0·2 (0·1–0·2)	-72·2% (-86·7 to -46%)
Alcohol use	Stroke	0·6% (0·2–1·1%)	4·9 (1·5–9·5)	-75% (-91 to -56·5%)	0·4% (0·1–0·8%)	0·2 (0–0·3)	-79% (-95 to -59·8%)
	Ischaemic stroke	0·3% (-0·1–0·7%)	1·7 (-0·8–4·7)	-65·3% (-144·1 to -18·5%)	0·2% (-0·2–0·6%)	0·1 (0–0·2)	-71·9% (-171·4 to -9·6%)
	Intracerebral haemorrhage	2·5% (1·4–3·9%)	3·3 (1·7–5·7)	-78·1% (-87·5 to -65·7%)	2% (1·1–3·2%)	0·1 (0–0·2)	-81·4% (-89·9 to -69·4%)
	Neurological disorders*	0·3% (0·1–0·7%)	4·3 (1·8–8·3)	-51·4% (-78·9 to 2·9%)	0·1% (0–0·3%)	<0·1 (<0·1–0·1)	-55·3% (-72·1 to -31·1%)
	Idiopathic epilepsy	3·3% (2–4·8%)	4·3 (1·8–8·3)	-51·4% (-78·9 to 2·9%)	4·2% (2·6–6·1%)	<0·1 (<0·1–0·1)	-55·3% (-72·1 to -31·1%)
All risk factors	Meningitis	3·3% (1·7–5·5%)	1 (0·4–1·7)	-85·5% (-93·9 to -69%)	1·7% (0·8–3%)	<0·1 (<0·1–<0·1)	-85·5% (-93·9 to -69%)
	Encephalitis	2·6% (1·3–5%)	0·4 (0·2–0·9)	-22·1% (-70·8 to 97·2%)	1·8% (0·8–3·7%)	<0·1 (<0·1–<0·1)	-22·1% (-70·8 to 97·2%)
	Stroke	87·8% (83·7–92·1%)	660·8 (511·1–818·3)	-29·8% (-45·1 to -11·7%)	84·7% (78·1–92·4%)	29·8 (20·8–38·6)	-36·2% (-54 to -15%)
	Ischaemic stroke	87·9% (83·2–93·6%)	525·8 (403·2–644·3)	-10·6% (-31·2 to 10·5%)	84·6% (76·9–93·7%)	24·9 (16·8–32·3)	-21·2% (-47·1 to 3·6%)
	Intracerebral haemorrhage	87·2% (83·3–90·7%)	112 (83·7–151·4)	-62·2% (-72·6 to -48·1%)	85·1% (79·2–90·9%)	4·3 (3–6)	-67·2% (-76·9 to -52·5%)
	Subarachnoid haemorrhage	86·1% (82–89·8%)	23 (16·1–31·9)	-60·3% (-76·6 to -35·5%)	83·2% (76·7–89·1%)	0·6 (0·3–0·9)	-70·8% (-85·9 to -43·2%)
	Neurological disorders*	15% (6·1–29·4%)	199·5 (79·5–466·2)	21·3% (3·8 to 59·8%)	34·6% (17·6–50·5%)	12·3 (2·4–35·5)	26·3% (2·1 to 95·8%)
	Alzheimer's disease and other dementias	51·1% (36·7–64·9%)	202·1 (81·7–468·2)	23·8% (6·5 to 66·9%)	48% (33·6–62·5%)	12·9 (2·9–36)	26% (2·2 to 92·8%)
	Parkinson's disease	-17% (-25·5–-9%)	-11·8 (-19·1–-6)	8·8% (-22·7 to 55·2%)	-15% (-22·6–-7·9%)	-0·7 (-1·1–-0·3)	9·6% (-24·5 to 62·9%)
	Idiopathic epilepsy	3·3% (2–4·8%)	4·3 (1·8–8·3)	-51·4% (-78·9 to 2·9%)	4·2% (2·6–6·1%)	<0·1 (<0·1–0·1)	-55·3% (-72·1 to -31·1%)
	Multiple sclerosis	21·4% (16·2–26·9%)	5 (3·4–7)	47% (13·9 to 92·5%)	22% (16·4–28·1%)	0·1 (0–0·1)	27·9% (-19·3 to 97·7%)
Ambient particulate matter pollution	Meningitis	0·4% (0·1–1%)	0·1 (0–0·3)	-80·5% (-95·8 to -17·2%)	0·2% (0·1–0·5%)	<0·1 (<0·1–<0·1)	-80·5% (-95·8 to -17·2%)
	Encephalitis	0·4% (0·1–0·9%)	0·1 (0–0·2)	0·5% (-79·3 to 422·9%)	0·2% (0·1–0·6%)	<0·1 (<0·1–<0·1)	0·5% (-79·3 to 422·8%)
	Stroke	22% (17·4–26·6%)	165·6 (115·7–215·5)	-14·7% (-35·1 to 15·8%)	17·8% (14–21·9%)	6·3 (4–8·5)	-23·7% (-46·8 to 7·8%)
	Ischaemic stroke	21·3% (16·8–25·8%)	127·2 (88·5–165·1)	12·9% (-15·8 to 50·2%)	17·2% (13·5–21·2%)	5·1 (3·2–6·9)	-2·3% (-33·5 to 35·7%)
	Intracerebral haemorrhage	24·4% (19·6–29·5%)	31·4 (21–45·2)	-53·4% (-67·4 to -31·9%)	20·5% (16·1–25%)	1 (0·7–1·5)	-59·9% (-72·9 to -39%)
	Subarachnoid haemorrhage	26·4% (21·2–31·6%)	7·1 (4·6–10·2)	-50·2% (-71 to -15·1%)	21·5% (16·9–26·2%)	0·2 (0·1–0·2)	-64·6% (-83·3 to -29·7%)
Behavioural risk factors‡	Meningitis	3·2% (1·6–5·4%)	0·9 (0·4–1·6)	-85·4% (-93·8 to -68·6%)	1·6% (0·7–2·9%)	<0·1 (<0·1–<0·1)	-85·4% (-93·8 to -68·6%)
	Encephalitis	2·6% (1·3–4·8%)	0·4 (0·2–0·9)	-21·1% (-70·3 to 98·9%)	1·7% (0·8–3·5%)	<0·1 (<0·1–<0·1)	-21·1% (-70·3 to 98·9%)
	Stroke	44·5% (39·7–49·8%)	335·1 (254·2–430·9)	-21·9% (-39 to -1·9%)	37·9% (32·4–44·1%)	13·3 (9·1–18)	-29·6% (-50·3 to -5·7%)

Table S16 Regional and national disabilities and deaths related to neurological conditions attributable to risk factors in North Africa and Middle East countries

**Location**<br>Risk factor (alphabetic order)	Cause	**DALYs (95% UI)**<br>PAF (%)<br>2019	 Rate per 100,000<br>2019	 Rate change (%)<br>From 1990 to 2019	**Deaths (95% UI)**<br>PAF (%)<br>2019	 Rate per 100,000<br>2019	 Rate change (%)<br>From 1990 to 2019
	Ischaemic stroke	45·2% (39·5–51·4%)	269·9 (200·9–343·6)	-1·5% (-24·2 to 21·4%)	38·5% (32–45·5%)	11·3 (7·2–15·5)	-14·8% (-44 to 13·9%)
	Intracerebral haemorrhage	41·8% (37·1–46·9%)	53·8 (39·1–75·6)	-58·7% (-70·8 to -42%)	35% (30·1–40·3%)	1·8 (1·2–2·5)	-63·9% (-75 to -46·6%)
	Subarachnoid haemorrhage	42·6% (37·5–47·7%)	11·4 (7·8–16·2)	-53·9% (-73 to -24·5%)	35·2% (30·1–40·6%)	0·3 (0·1–0·4)	-66·6% (-84·2 to -35%)
	Neurological disorders*	8·3% (3·3–16·9%)	110·6 (41·4–258)	15% (-7·2 to 51·9%)	16·9% (6·4–26·4%)	6·1 (0·9–17·8)	21% (-9 to 91·1%)
	Alzheimer's disease and other dementias	28·6% (18·5–37·6%)	113·1 (45–260·3)	19·3% (-3·1 to 61·1%)	24·9% (15·7–33·3%)	6·7 (1·5–18·3)	20·8% (-7·7 to 83·5%)
	Parkinson's disease	-17% (-25·5–-9%)	-11·8 (-19·1–-6)	8·8% (-22·7 to 55·2%)	-15% (-22·6–-7·9%)	-0·7 (-1·1–-0·3)	9·6% (-24·5 to 62·9%)
	Idiopathic epilepsy	3·3% (2–4·8%)	4·3 (1·8–8·3)	-51·4% (-78·9 to 2·9%)	4·2% (2·6–6·1%)	<0·1 (<0·1–0·1)	-55·3% (-72·1 to -31·1%)
	Multiple sclerosis	21·4% (16·2–26·9%)	5 (3·4–7)	47% (13·9 to 92·5%)	22% (16·4–28·1%)	0·1 (0–0·1)	27·9% (-19·3 to 97·7%)
Child and maternal malnutrition	Meningitis	3·2% (1·6–5·4%)	0·9 (0·4–1·6)	-85·4% (-93·8 to -68·6%)	1·6% (0·7–2·9%)	<0·1 (<0·1–<0·1)	-85·4% (-93·8 to -68·6%)
	Encephalitis	2·6% (1·3–4·8%)	0·4 (0·2–0·9)	-21·1% (-70·3 to 98·9%)	1·7% (0·8–3·5%)	<0·1 (<0·1–<0·1)	-21·1% (-70·3 to 98·9%)
Diet high in red meat	Stroke	5·8% (2·5–8·1%)	43·3 (17·1–65·9)	-20·9% (-45·3 to 13·4%)	3·9% (1·6–5·7%)	1·4 (0·5–2·2)	-32·9% (-58 to -3%)
	Ischaemic stroke	5·3% (1·5–7·7%)	31·6 (7·7–50)	9·8% (-16·3 to 57·6%)	3·6% (0·9–5·4%)	1·1 (0·2–1·7)	-9·3% (-40·2 to 32·6%)
	Intracerebral haemorrhage	7·3% (2·1–11·1%)	9·5 (2·5–16·5)	-55·5% (-68·6 to -30·2%)	5·7% (1·4–8·7%)	0·3 (0·1–0·5)	-62·6% (-74·7 to -40·2%)
	Subarachnoid haemorrhage	8·4% (2·4–12·6%)	2·2 (0·6–3·9)	-52·6% (-72 to -13·2%)	6·1% (1·6–9·4%)	<0·1 (<0·1–0·1)	-67·1% (-84·3 to -28%)
Diet high in sodium	Stroke	1·7% (0·3–7%)	13·2 (1·9–54·9)	-32·5% (-60·3 to 16·9%)	1·4% (0·3–5·6%)	0·5 (0·1–2·1)	-39·2% (-69·4 to 8·5%)
	Ischaemic stroke	1·6% (0·3–6·6%)	9·9 (1·5–40·9)	-9·5% (-49·2 to 53·6%)	1·3% (0·3–5·3%)	0·4 (0·1–1·7)	-19·9% (-63·5 to 38·3%)
	Intracerebral haemorrhage	2·1% (0·3–8·5%)	2·8 (0·3–12)	-62% (-78·6 to -29·6%)	1·8% (0·3–7·5%)	0·1 (0–0·4)	-67·4% (-82·4 to -41·8%)
	Subarachnoid haemorrhage	2·1% (0·2–8·2%)	0·6 (0·1–2·3)	-58·5% (-81·7 to -14%)	1·8% (0·3–7·4%)	<0·1 (<0·1–0·1)	-70·2% (-88·2 to -30·7%)
Diet low in fibre	Stroke	1% (0·3–2·3%)	7·7 (2·1–17·2)	-1·3% (-33·5 to 36·2%)	0·9% (0·3–1·9%)	0·3 (0·1–0·7)	-25·2% (-55·6 to 15·9%)
	Ischaemic stroke	1% (0·3–2·2%)	5·8 (1·6–13·3)	25·7% (-18·1 to 71·8%)	0·9% (0·3–1·8%)	0·3 (0·1–0·5)	-8·3% (-47·6 to 45·8%)
	Intracerebral haemorrhage	1·2% (0·3–2·7%)	1·5 (0·4–3·7)	-42·5% (-64·1 to -16%)	1% (0·3–2·3%)	0·1 (0–0·1)	-58% (-75·2 to -33·5%)
	Subarachnoid haemorrhage	1·3% (0·3–3%)	0·4 (0·1–0·9)	-36·8% (-66·1 to 9·7%)	1·1% (0·3–2·4%)	<0·1 (<0·1–<0·1)	-60·3% (-82·5 to -19·1%)
Diet low in fruits	Stroke	1·9% (0·8–3·2%)	14·4 (6–25·8)	57% (-9·1 to 153·1%)	1·2% (0·5–2·1%)	0·4 (0·2–0·8)	5·3% (-44·4 to 94·4%)
	Ischaemic stroke	1·5% (0·4–3%)	9·2 (2–18·7)	121·9% (5·9 to 253·6%)	1% (0·3–2%)	0·3 (0·1–0·6)	30·1% (-37·1 to 160·6%)
	Intracerebral haemorrhage	3·2% (1·5–5·4%)	4·2 (1·7–7·6)	4·6% (-34·6 to 72·8%)	2·3% (1–3·8%)	0·1 (0–0·2)	-25·3% (-57·7 to 37·1%)
	Subarachnoid haemorrhage	4% (1·8–6·9%)	1·1 (0·4–2)	1·3% (-45·4 to 92·5%)	2·6% (1·2–4·5%)	<0·1 (<0·1–<0·1)	-31·6% (-70·4 to 50%)
Diet low in vegetables	Stroke	0·2% (0·2–0·3%)	1·8 (1·3–2·5)	-30% (-51·8 to -2·5%)	0·3% (0·2–0·4%)	0·1 (0·1–0·2)	-36·3% (-62·4 to 0·8%)
	Ischaemic stroke	0·2% (0·2–0·3%)	1·4 (1–2·1)	-11·6% (-40·5 to 23·1%)	0·3% (0·2–0·4%)	0·1 (0–0·1)	-21·7% (-54·8 to 26·4%)
	Intracerebral haemorrhage	0·2% (0·2–0·3%)	0·3 (0·2–0·5)	-63·1% (-75·1 to -43·8%)	0·3% (0·2–0·5%)	<0·1 (<0·1–<0·1)	-67·9% (-80·6 to -46·4%)
	Subarachnoid haemorrhage	0·2% (0·2–0·3%)	0·1 (0–0·1)	-61·2% (-77·9 to -35·6%)	0·2% (0·2–0·5%)	<0·1 (<0·1–<0·1)	-70·5% (-86·7 to -38·9%)
Diet low in whole grains	Stroke	7·5% (4·1–9·6%)	56·5 (30·5–79·8)	-12% (-31·9 to 7·9%)	6·9% (4·2–8·7%)	2·4 (1·3–3·5)	-23·8% (-47·2 to 0·4%)
	Ischaemic stroke	9·5% (5·2–12·1%)	56·5 (30·5–79·8)	-12% (-31·9 to 7·9%)	8·3% (5–10·3%)	2·4 (1·3–3·5)	-23·8% (-47·2 to 0·4%)

Table S16 Regional and national disabilities and deaths related to neurological conditions attributable to risk factors in North Africa and Middle East countries

**Location**<br>Risk factor (alphabetic order)	Cause	**DALYs (95% UI)**<br>PAF (%)<br>2019	 Rate per 100,000<br>2019	 Rate change (%)<br>From 1990 to 2019	**Deaths (95% UI)**<br>PAF (%)<br>2019	 Rate per 100,000<br>2019	 Rate change (%)<br>From 1990 to 2019
Dietary risks	Stroke	16·8% (12·2–21·6%)	126·7 (82·7–178·7)	-15·5% (-35·7 to 6%)	13·9% (10·1–17·8%)	4·9 (2·9–7)	-27·8% (-52 to -4·2%)
	Ischaemic stroke	17·7% (12·1–22·4%)	105·5 (65·5–147·9)	-2% (-23·1 to 20·1%)	14·5% (10·3–18·5%)	4·3 (2·5–6·2)	-18·1% (-45·7 to 7·8%)
	Intracerebral haemorrhage	13·3% (7·2–19·5%)	17·1 (8·4–28·9)	-50·5% (-66 to -23·4%)	10·6% (5·5–16·3%)	0·5 (0·2–0·9)	-59·7% (-73·4 to -37%)
	Subarachnoid haemorrhage	15% (8·4–21·3%)	4 (2–6·4)	-46·8% (-68·9 to 0·2%)	11·3% (6–17%)	0·1 (0–0·1)	-63·6% (-82·3 to -22·2%)
Environmental/occupational risk factors§	Meningitis	0·4% (0·1–1%)	0·1 (0–0·3)	-85·2% (-96·5 to -37·5%)	0·2% (0·1–0·5%)	<0·1 (<0·1–<0·1)	-85·2% (-96·5 to -37·6%)
	Encephalitis	0·4% (0·1–0·9%)	0·1 (0–0·2)	-23·6% (-84·9 to 286·8%)	0·2% (0·1–0·6%)	<0·1 (<0·1–<0·1)	-23·6% (-84·9 to 287·1%)
	Stroke	29·2% (24·5–33·6%)	220·2 (158·5–284·5)	-37·1% (-50·9 to -19·8%)	27·9% (23·4–31·9%)	9·8 (6·6–13·2)	-41·4% (-57 to -20·9%)
	Ischaemic stroke	28·5% (23·9–32·7%)	170·4 (123·3–218·4)	-16·7% (-36 to 4%)	27·4% (22·9–31·4%)	8·1 (5·4–10·9)	-25·7% (-48·4 to -1·6%)
	Intracerebral haemorrhage	32% (26·8–36·7%)	41·2 (28·9–58·7)	-65·8% (-75·6 to -52·4%)	30·5% (25·7–35%)	1·5 (1–2·2)	-69·7% (-78·8 to -56·2%)
	Subarachnoid haemorrhage	32·3% (26·9–37·4%)	8·6 (5·8–12·3)	-65·3% (-79·7 to -42·3%)	31·2% (26·3–35·8%)	0·2 (0·1–0·3)	-73·3% (-87·1 to -49%)
High body-mass index	Stroke	32·7% (22·4–42·4%)	246·6 (159·2–364·3)	-18·4% (-38 to 7%)	19% (11·4–27·4%)	6·7 (3·6–11·1)	-26·8% (-49·6 to 3·7%)
	Ischaemic stroke	28·3% (18·9–37·4%)	169·1 (107·1–250·3)	22·8% (-6·7 to 58%)	16·3% (9·2–24·4%)	4·8 (2·4–8·1)	9·3% (-29 to 54·5%)
	Intracerebral haemorrhage	48·7% (36–60·1%)	62·6 (40·1–94)	-53·1% (-67·2 to -31·7%)	32·4% (21·2–44·8%)	1·6 (0·9–2·7)	-59% (-72·6 to -36·5%)
	Subarachnoid haemorrhage	55·8% (42·8–67·1%)	14·9 (9·5–21·9)	-52·1% (-71·3 to -21·3%)	36·1% (23·8–48·4%)	0·3 (0·1–0·4)	-66·1% (-83·8 to -33·3%)
	Neurological disorders*	6% (1·7–13·6%)	78·9 (21·7–196)	39·9% (14·4 to 96%)	14·7% (5·3–27·3%)	5·1 (0·9–14·8)	41·4% (9·6 to 122·7%)
	Alzheimer's disease and other dementias	19·9% (8·5–34·9%)	78·9 (21·7–196)	39·9% (14·4 to 96%)	19·2% (8·1–34%)	5·1 (0·9–14·8)	41·4% (9·6 to 122·7%)
High fasting plasma glucose	Stroke	31·1% (18·8–53%)	234·3 (122·7–400·6)	7·4% (-25·7 to 51·7%)	31·9% (16·9–63·5%)	11·2 (5–22·6)	-8·2% (-40·3 to 34·3%)
	Ischaemic stroke	32·4% (17·2–59·2%)	194 (91·2–359·7)	31·1% (-9·1 to 87%)	32·3% (14·9–70·2%)	9·5 (3·6–20·9)	10·1% (-28·8 to 64·5%)
	Intracerebral haemorrhage	26·6% (16·8–38·6%)	34·3 (19·1–57)	-42·7% (-61·6 to -14·7%)	29·8% (17·7–49·1%)	1·5 (0·8–2·8)	-51·6% (-68·1 to -24·6%)
	Subarachnoid haemorrhage	22·3% (13·9–31·9%)	6 (3·3–9·8)	-42·3% (-68·3 to 5·7%)	28·4% (17–46·4%)	0·2 (0·1–0·4)	-56·7% (-79·5 to -8·1%)
	Neurological disorders*	4·3% (0·8–11·8%)	57·1 (10·2–187·2)	51·7% (32·9 to 109·5%)	11% (2·5–24·5%)	3·9 (0·4–15)	50·8% (25·4 to 134·9%)
	Alzheimer's disease and other dementias	14·4% (3·6–29·6%)	57·1 (10·2–187·2)	51·7% (32·9 to 109·5%)	14·5% (3·6–29·6%)	3·9 (0·4–15)	50·8% (25·4 to 134·9%)
High LDL cholesterol	Stroke	21·6% (12·4–35·3%)	162·5 (86·2–271·8)	-4·6% (-25·1 to 21%)	18·8% (6·1–39·9%)	6·6 (2–14)	-18·3% (-44·9 to 13·1%)
	Ischaemic stroke	27·2% (15·4–44·2%)	162·5 (86·2–271·8)	-4·6% (-25·1 to 21%)	22·4% (7·3–47·8%)	6·6 (2–14)	-18·3% (-44·9 to 13·1%)
High systolic blood pressure	Stroke	51·4% (43·1–59·6%)	387·3 (281·4–503·4)	-28·3% (-45·9 to -6·1%)	44·9% (35·1–55·9%)	15·8 (10–22·3)	-37·5% (-57·3 to -12%)
	Ischaemic stroke	49·5% (39·6–58·8%)	295·9 (205·3–388·4)	-5·6% (-28·6 to 21·6%)	43% (32–56·1%)	12·7 (7·5–18·4)	-19·8% (-47·9 to 11·2%)
	Intracerebral haemorrhage	58·6% (49·4–67·4%)	75·3 (51·2–107·1)	-60·2% (-71·4 to -42·5%)	54·4% (42·4–65·9%)	2·7 (1·8–4)	-66·5% (-77·4 to -49·9%)
	Subarachnoid haemorrhage	60·2% (49·7–69·3%)	16·1 (10·7–23·6)	-56·7% (-74·9 to -28·3%)	54·8% (43·3–66%)	0·4 (0·2–0·6)	-69·8% (-85·6 to -40·1%)
High temperature	Stroke	0% (0–0%)	0 (-0·2–0·3)	-89·2% (-944·4 to 995·2%)	0% (0–0%)	<0·1 (<0·1–<0·1)	-89·6% (-975 to 1070·3%)
	Ischaemic stroke	0% (0–0%)	0 (-0·2–0·2)	-86·6% (-1235·7 to 1363·2%)	0% (0–0%)	<0·1 (<0·1–<0·1)	-87·7% (-1212 to 1292·2%)

Table S16 Regional and national disabilities and deaths related to neurological conditions attributable to risk factors in North Africa and Middle East countries

Location<br>Risk factor (alphabetic order)	Cause	DALYs (95% UI)<br>PAF (%)<br>2019	Rate per 100,000<br>2019	Rate change (%)<br>From 1990 to 2019	Deaths (95% UI)<br>PAF (%)<br>2019	Rate per 100,000<br>2019	Rate change (%)<br>From 1990 to 2019
	Intracerebral haemorrhage	0% (0–0%)	<0·1 (<0·1–0·1)	-92·9% (-566·3 to 560·4%)	0% (0–0%)	<0·1 (<0·1–<0·1)	-93·4% (-560·4 to 531·1%)
	Subarachnoid haemorrhage	0% (0–0%)	<0·1 (<0·1–<0·1)	-92·1% (-447·1 to 485·6%)	0% (0–0%)	<0·1 (<0·1–<0·1)	-92·7% (-458 to 507·9%)
Household air pollution from solid fuels	Meningitis	0% (0–0%)	<0·1 (<0·1–<0·1)	-99·8% (-100 to -99%)	0% (0–0%)	<0·1 (<0·1–<0·1)	-99·8% (-100 to -99%)
	Encephalitis	0% (0–0%)	<0·1 (<0·1–<0·1)	-98·8% (-99·8 to -92·1%)	0% (0–0%)	<0·1 (<0·1–<0·1)	-98·8% (-99·8 to -92·1%)
	Stroke	0·1% (0–0·2%)	0·5 (0·2–1·3)	-99% (-99·7 to -97·6%)	0·1% (0–0·1%)	<0·1 (<0·1–<0·1)	-99·1% (-99·7 to -97·8%)
	Ischaemic stroke	0·1% (0–0·2%)	0·4 (0·1–1)	-98·7% (-99·6 to -96·8%)	0·1% (0–0·1%)	<0·1 (<0·1–<0·1)	-98·9% (-99·7 to -97·1%)
	Intracerebral haemorrhage	0·1% (0–0·2%)	0·1 (0–0·2)	-99·5% (-99·8 to -98·6%)	0·1% (0–0·1%)	<0·1 (<0·1–<0·1)	-99·5% (-99·9 to -98·8%)
	Subarachnoid haemorrhage	0·1% (0–0·2%)	<0·1 (<0·1–0·1)	-99·4% (-99·8 to -98·3%)	0·1% (0–0·2%)	<0·1 (<0·1–<0·1)	-99·6% (-99·9 to -98·7%)
Kidney dysfunction	Stroke	12% (8·8–14·8%)	90·2 (60·1–122·1)	-1·5% (-25 to 25·7%)	10·5% (5·6–14·7%)	3·7 (1·7–5·7)	-13·9% (-45·1 to 18%)
	Ischaemic stroke	12·9% (8·9–16·3%)	76·9 (48·1–104·8)	21·5% (-9·3 to 51·3%)	10·4% (4·7–15·5%)	3·1 (1·2–5)	5·9% (-35·4 to 44·1%)
	Intracerebral haemorrhage	10·4% (8·3–12·5%)	13·3 (8·6–19·1)	-52·9% (-66 to -33·9%)	12·3% (8·8–15·6%)	0·6 (0·4–0·9)	-55·3% (-69·6 to -34·5%)
Lead exposure	Stroke	2·7% (1·2–4·6%)	20·6 (8·1–37)	-53·9% (-68·3 to -40·4%)	3·2% (1·6–5·1%)	1·1 (0·5–2)	-48·8% (-66·6 to -29·8%)
	Ischaemic stroke	2·8% (1·2–4·7%)	16·7 (6·7–29·7)	-36·4% (-55·9 to -17·9%)	3·1% (1·5–5·2%)	0·9 (0·4–1·7)	-32% (-54·5 to -8·9%)
	Intracerebral haemorrhage	2·6% (0·9–4·8%)	3·3 (1·1–6·8)	-78·9% (-87 to -69·8%)	3·4% (1·5–5·8%)	0·2 (0·1–0·3)	-76·3% (-84·6 to -65·8%)
	Subarachnoid haemorrhage	2% (0·6–4·2%)	0·5 (0·1–1·2)	-80·5% (-91·4 to -66·3%)	3·1% (1·2–5·3%)	<0·1 (<0·1–<0·1)	-79·4% (-90·6 to -60·2%)
Low birth weight	Meningitis	2·9% (1·5–4·9%)	0·8 (0·4–1·5)	-85·5% (-93·8 to -68·5%)	1·5% (0·7–2·6%)	<0·1 (<0·1–<0·1)	-85·5% (-93·8 to -68·5%)
	Encephalitis	2·3% (1·2–4·4%)	0·4 (0·2–0·8)	-22·3% (-69·8 to 97·3%)	1·6% (0·7–3·2%)	<0·1 (<0·1–<0·1)	-22·3% (-69·8 to 97·3%)
Low birth weight and short gestation	Meningitis	3·2% (1·6–5·4%)	0·9 (0·4–1·6)	-85·4% (-93·8 to -68·6%)	1·6% (0·7–2·9%)	<0·1 (<0·1–<0·1)	-85·4% (-93·8 to -68·6%)
	Encephalitis	2·6% (1·3–4·8%)	0·4 (0·2–0·9)	-21·1% (-70·3 to 98·9%)	1·7% (0·8–3·5%)	<0·1 (<0·1–<0·1)	-21·1% (-70·3 to 98·9%)
Low physical activity	Stroke	6·6% (1·4–14·4%)	49·6 (10·1–109·7)	-8·1% (-32·5 to 15·5%)	8·3% (2–16·5%)	2·9 (0·6–6·1)	-18·8% (-45·9 to 10·3%)
	Ischaemic stroke	8·3% (1·7–18%)	49·6 (10·1–109·7)	-8·1% (-32·5 to 15·5%)	9·9% (2·4–19·8%)	2·9 (0·6–6·1)	-18·8% (-45·9 to 10·3%)
Low temperature	Stroke	6·5% (4·5–8·7%)	49·4 (27·2–72·1)	-45·6% (-61·8 to -26·3%)	9·3% (6·6–12·1%)	3·3 (1·9–4·7)	-42·9% (-58·6 to -23·7%)
	Ischaemic stroke	6·3% (4·2–8·5%)	38·1 (21·5–55·7)	-27·1% (-48·8 to -2·6%)	9·3% (6·6–12·1%)	2·8 (1·6–4)	-29·3% (-51·2 to -6·9%)
	Intracerebral haemorrhage	7·5% (5·2–9·9%)	9·7 (5·7–15·3)	-69·8% (-78·9 to -56·3%)	9·3% (6·6–12·1%)	0·5 (0·3–0·7)	-70·8% (-79·4 to -57·5%)
	Subarachnoid haemorrhage	5·8% (3·8–8·1%)	1·6 (0·8–2·5)	-75·2% (-88 to -53·5%)	9·3% (6·6–12·1%)	0·1 (0–0·1)	-74·3% (-87·4 to -50·5%)
Metabolic risk factors†	Stroke	75·9% (68·2–84·5%)	571·6 (423–723·8)	-25·3% (-41·5 to -6%)	70·3% (58·2–84·9%)	24·7 (16·3–33·3)	-33% (-53·1 to -10%)
	Ischaemic stroke	75·8% (66·5–86·6%)	453·4 (337·9–572·5)	-5·3% (-27 to 18·9%)	69·8% (55·9–87%)	20·5 (13·1–28·1)	-17·1% (-46·4 to 10·9%)
	Intracerebral haemorrhage	76·5% (68·9–82·7%)	98·3 (71·5–135·5)	-59·2% (-70·5 to -43%)	72·9% (63·3–82·1%)	3·7 (2·5–5·2)	-65·1% (-75·7 to -48·8%)
	Subarachnoid haemorrhage	74·8% (66·6–81·6%)	20 (13·7–28·1)	-56·6% (-74·5 to -29·1%)	69·5% (59·1–79·6%)	0·5 (0·3–0·7)	-69% (-85 to -38·6%)
	Neurological disorders*	9·4% (3·2–20%)	124·6 (38–311·9)	40·3% (18·9 to 92%)	23·6% (9·5–39·8%)	8·3 (1·4–24·5)	41% (13·6 to 115·7%)
	Alzheimer's disease and other dementias	31·5% (15·7–50·1%)	124·6 (38–311·9)	40·3% (18·9 to 92%)	30·9% (14·9–49·3%)	8·3 (1·4–24·5)	41% (13·6 to 115·7%)
Non-optimal temperature	Stroke	6·5% (4·5–8·7%)	49·4 (27·2–72·2)	-45·6% (-61·8 to -26·3%)	9·3% (6·6–12·1%)	3·3 (1·9–4·7)	-42·9% (-58·6 to -23·8%)

Table S16 Regional and national disabilities and deaths related to neurological conditions attributable to risk factors in North Africa and Middle East countries

**Location**<br>Risk factor (alphabetic order)	Cause	**DALYs (95% UI)**<br>PAF (%)<br>2019	Rate per 100,000<br>2019	Rate change (%)<br>From 1990 to 2019	**Deaths (95% UI)**<br>PAF (%)<br>2019	Rate per 100,000<br>2019	Rate change (%)<br>From 1990 to 2019
	Ischaemic stroke	6·3% (4·2–8·5%)	38·2 (21·4–55·7)	-27·2% (-48·9 to -2·6%)	9·3% (6·6–12·1%)	2·8 (1·6–4)	-29·3% (-51·2 to -7·2%)
	Intracerebral haemorrhage	7·5% (5·3–9·9%)	9·7 (5·7–15·3)	-69·8% (-79 to -56·3%)	9·3% (6·6–12·1%)	0·5 (0·3–0·7)	-70·9% (-79·4 to -57·5%)
	Subarachnoid haemorrhage	5·8% (3·8–8·1%)	1·6 (0·8–2·5)	-75·2% (-88 to -53·4%)	9·3% (6·6–12·1%)	0·1 (0–0·1)	-74·3% (-87·5 to -50·4%)
Other environmental risks	Stroke	2·7% (1·2–4·6%)	20·6 (8·1–37)	-53·9% (-68·3 to -40·4%)	3·2% (1·6–5·1%)	1·1 (0·5–2)	-48·8% (-66·6 to -29·8%)
	Ischaemic stroke	2·8% (1·2–4·7%)	16·7 (6·7–29·7)	-36·4% (-55·9 to -17·9%)	3·1% (1·5–5·2%)	0·9 (0·4–1·7)	-32% (-54·5 to -8·9%)
	Intracerebral haemorrhage	2·6% (0·9–4·8%)	3·3 (1·1–6·8)	-78·9% (-87 to -69·8%)	3·4% (1·5–5·8%)	0·2 (0·1–0·3)	-76·3% (-84·6 to -65·8%)
	Subarachnoid haemorrhage	2% (0·6–4·2%)	0·5 (0·1–1·2)	-80·5% (-91·4 to -66·3%)	3·1% (1·2–5·3%)	<0·1 (<0·1–<0·1)	-79·4% (-90·6 to -60·2%)
Particulate matter pollution	Meningitis	0·4% (0·1–1%)	0·1 (0–0·3)	-85·2% (-96·5 to -37·5%)	0·2% (0·1–0·5%)	<0·1 (<0·1–<0·1)	-85·2% (-96·5 to -37·6%)
	Encephalitis	0·4% (0·1–0·9%)	0·1 (0–0·2)	-23·6% (-84·9 to 286·8%)	0·2% (0·1–0·6%)	<0·1 (<0·1–<0·1)	-23·6% (-84·9 to 287·1%)
	Stroke	22·1% (17·6–26·7%)	166·1 (115·9–215·8)	-32·7% (-47·6 to -14·7%)	17·8% (14·1–21·9%)	6·3 (4–8·5)	-39·8% (-55·7 to -18·5%)
	Ischaemic stroke	21·3% (16·9–25·9%)	127·6 (89·1–165·7)	-10·9% (-30·8 to 10·6%)	17·3% (13·6–21·3%)	5·1 (3·3–6·9)	-22·9% (-45·9 to 3·1%)
	Intracerebral haemorrhage	24·5% (19·7–29·5%)	31·5 (21·1–45·4)	-63·1% (-73·9 to -48·6%)	20·6% (16·2–25·1%)	1 (0·7–1·5)	-68·3% (-78 to -53·6%)
	Subarachnoid haemorrhage	26·5% (21·3–31·8%)	7·1 (4·6–10·2)	-61% (-76·8 to -36·2%)	21·6% (17–26·3%)	0·2 (0·1–0·2)	-72·2% (-86·7 to -46%)
Secondhand smoke	Stroke	4·2% (3·2–5·2%)	31·7 (20·8–43·2)	-30·2% (-47·2 to -9·2%)	3·6% (2·7–4·5%)	1·3 (0·8–1·8)	-34·4% (-53·8 to -11·4%)
	Ischaemic stroke	3·9% (2·9–4·9%)	23·5 (15·3–31·9)	-3·2% (-27·8 to 22·5%)	3·4% (2·6–4·4%)	1 (0·6–1·4)	-14·3% (-43·8 to 14·1%)
	Intracerebral haemorrhage	5·3% (4–6·7%)	6·8 (4·3–10·1)	-60·9% (-72·9 to -43·9%)	4·5% (3·4–5·6%)	0·2 (0·1–0·3)	-65% (-75·6 to -48·2%)
	Subarachnoid haemorrhage	5·4% (4·1–6·9%)	1·5 (0·9–2·3)	-61·9% (-78·7 to -34·5%)	4·9% (3·6–6·1%)	<0·1 (<0·1–0·1)	-69·8% (-85·6 to -41·1%)
Short gestation	Meningitis	2·3% (1·2–3·9%)	0·7 (0·3–1·2)	-82·7% (-92·6 to -63·1%)	1·2% (0·6–2·1%)	<0·1 (<0·1–<0·1)	-82·7% (-92·6 to -63·1%)
	Encephalitis	1·9% (1–3·6%)	0·3 (0·1–0·6)	-6% (-64·9 to 137·4%)	1·3% (0·6–2·6%)	<0·1 (<0·1–<0·1)	-6% (-64·9 to 137·4%)
Smoking	Stroke	25·8% (23·2–28·6%)	194 (148·6–249)	-17·8% (-38·5 to 7·3%)	18·4% (16·3–20·9%)	6·5 (4·6–8·6)	-25·8% (-49 to 3·6%)
	Ischaemic stroke	25% (22·5–27·9%)	149·6 (114·5–188·1)	11·2% (-16·8 to 43%)	17·5% (15·4–19·8%)	5·1 (3·5–6·8)	-1% (-35 to 37·8%)
	Intracerebral haemorrhage	28·5% (25·6–31·7%)	36·6 (26·6–51)	-56·9% (-70·2 to -38·4%)	23·3% (20·5–26·7%)	1·2 (0·8–1·7)	-61·4% (-74·2 to -42·6%)
	Subarachnoid haemorrhage	29·4% (25·9–33·4%)	7·9 (5·3–11·2)	-52·7% (-73 to -21·4%)	24·1% (20·8–27·7%)	0·2 (0·1–0·3)	-65·7% (-84·2 to -31·7%)
	Neurological disorders*	8% (3–16·5%)	106·3 (37·7–253·9)	21·7% (-1·5 to 64·9%)	16·7% (6·2–26·4%)	6·1 (0·9–17·8)	22·3% (-8·5 to 95·4%)
	Alzheimer's disease and other dementias	28·6% (18·5–37·6%)	113·1 (45–260·3)	19·3% (-3·1 to 61·1%)	24·9% (15·7–33·3%)	6·7 (1·5–18·3)	20·8% (-7·7 to 83·5%)
	Parkinson's disease	-17% (-25·5–-9%)	-11·8 (-19·1–-6)	8·8% (-22·7 to 55·2%)	-15% (-22·6–-7·9%)	-0·7 (-1·1–-0·3)	9·6% (-24·5 to 62·9%)
	Multiple sclerosis	21·4% (16·2–26·9%)	5 (3·4–7)	47% (13·9 to 92·5%)	22% (16·4–28·1%)	0·1 (0–0·1)	27·9% (-19·3 to 97·7%)
Tobacco	Stroke	28·7% (26·2–31·5%)	216·3 (167–275·5)	-20% (-39 to 3·5%)	21·2% (19–23·7%)	7·5 (5·1–9·8)	-27·6% (-50·1 to 0·3%)
	Ischaemic stroke	27·8% (25·4–30·5%)	166·2 (128·6–206)	8·5% (-17·5 to 37·7%)	20·1% (18–22·4%)	5·9 (4–7·8)	-4% (-36·1 to 30·4%)
	Intracerebral haemorrhage	32% (29·2–35·2%)	41·2 (30–57·1)	-57·8% (-70·6 to -40%)	26·5% (23·7–29·8%)	1·3 (0·9–1·9)	-62·2% (-74 to -43·8%)
	Subarachnoid haemorrhage	33·1% (29·6–37%)	8·9 (6·1–12·7)	-54·5% (-74 to -25·3%)	27·5% (24·1–31·3%)	0·2 (0·1–0·3)	-66·6% (-84·5 to -34·2%)
	Neurological disorders*	8% (3–16·5%)	106·3 (37·7–253·9)	21·7% (-1·5 to 64·9%)	16·7% (6·2–26·4%)	6·1 (0·9–17·8)	22·3% (-8·5 to 95·4%)

Table S16 Regional and national disabilities and deaths related to neurological conditions attributable to risk factors in North Africa and Middle East countries

**Location**<br>Risk factor (alphabetic order)	Cause	**DALYs (95% UI)**<br>PAF (%)<br>2019	 Rate per 100,000<br>2019	 Rate change (%)<br>From 1990 to 2019	**Deaths (95% UI)**<br>PAF (%)<br>2019	 Rate per 100,000<br>2019	 Rate change (%)<br>From 1990 to 2019
	Alzheimer's disease and other dementias	28·6% (18·5–37·6%)	113·1 (45–260·3)	19·3% (-3·1 to 61·1%)	24·9% (15·7–33·3%)	6·7 (1·5–18·3)	20·8% (-7·7 to 83·5%)
	Parkinson's disease	-17% (-25·5–-9%)	-11·8 (-19·1–-6)	8·8% (-22·7 to 55·2%)	-15% (-22·6–-7·9%)	-0·7 (-1·1–-0·3)	9·6% (-24·5 to 62·9%)
	Multiple sclerosis	21·4% (16·2–26·9%)	5 (3·4–7)	47% (13·9 to 92·5%)	22% (16·4–28·1%)	0·1 (0–0·1)	27·9% (-19·3 to 97·7%)
**Libya**							
Air pollution	Meningitis	0·4% (0·2–0·7%)	0·1 (0·1–0·3)	-91% (-96·9 to -75·8%)	0·2% (0·1–0·4%)	<0·1 (<0·1–<0·1)	-91% (-96·9 to -75·8%)
	Encephalitis	0·4% (0·1–0·9%)	0·1 (0–0·1)	-37·5% (-83·3 to 82·4%)	0·2% (0·1–0·5%)	<0·1 (<0·1–<0·1)	-37·5% (-83·3 to 82·5%)
	Stroke	27·1% (20·1–33·4%)	425·7 (287·9–594·2)	-18·2% (-37·4 to 8·4%)	23·8% (17·2–29·6%)	16·5 (10·8–24)	-22·3% (-41·6 to 5·6%)
	Ischaemic stroke	26·7% (19·7–33%)	299·7 (201·4–419·3)	11·5% (-14·3 to 44·7%)	23% (16·7–28·9%)	12·4 (8–18·4)	1·2% (-24·3 to 38·6%)
	Intracerebral haemorrhage	28·5% (21·5–35·2%)	107·7 (68·7–156)	-49·4% (-63·9 to -28·8%)	26·8% (19·7–33%)	3·6 (2·3–5·3)	-54% (-67·5 to -34%)
	Subarachnoid haemorrhage	25·5% (18·6–32·2%)	18·3 (11·4–28)	-52·5% (-68·1 to -25·2%)	25·5% (18·9–31·6%)	0·5 (0·3–0·9)	-57·2% (-72 to -30·1%)
Alcohol use	Stroke	0·3% (0–0·5%)	4·7 (0·1–7·9)	373·1% (-130·7 to 1564·7%)	0·2% (0–0·3%)	0·1 (0–0·2)	439·8% (-1589·2 to 3855·6%)
	Ischaemic stroke	0·2% (0–0·3%)	1·7 (-0·1–3·3)	-5465·9% (-14472·5 to 14852·3%)	0·1% (0–0·2%)	0·1 (0–0·1)	-1568% (-15250·1 to 12346%)
	Intracerebral haemorrhage	0·8% (0·1–1·2%)	3 (0·5–5·2)	194·8% (-47·9 to 669·4%)	0·7% (0·1–1%)	0·1 (0–0·2)	179·6% (-54·1 to 651·3%)
	Neurological disorders*	0·1% (0–0·2%)	1·6 (0·5–2·9)	218% (-16·4 to 732·1%)	0·1% (0–0·1%)	<0·1 (<0·1–<0·1)	249·8% (3·1 to 594·1%)
	Idiopathic epilepsy	1·1% (0·3–1·7%)	1·6 (0·5–2·9)	218% (-16·4 to 732·1%)	1·3% (0·3–1·9%)	<0·1 (<0·1–<0·1)	249·8% (3·1 to 594·1%)
All risk factors	Meningitis	2·4% (1·5–4·1%)	0·9 (0·5–1·5)	-89·9% (-95 to -80·2%)	1·2% (0·7–2·2%)	<0·1 (<0·1–<0·1)	-89·9% (-95 to -80·2%)
	Encephalitis	2·5% (1·2–4·7%)	0·4 (0·2–0·7)	-28·7% (-74·1 to 73%)	1·4% (0·7–2·7%)	<0·1 (<0·1–<0·1)	-28·7% (-74·1 to 73%)
	Stroke	86·3% (82·7–89·9%)	1355·5 (1061·2–1716·9)	-10·5% (-29·5 to 16·2%)	86·3% (81–92%)	59·9 (45·4–80·6)	-14% (-33·2 to 14·3%)
	Ischaemic stroke	88·1% (83·9–92·8%)	989 (770·4–1273·6)	18·6% (-7·2 to 51·6%)	86·5% (80·2–93·7%)	46·8 (34·9–64·6)	9% (-16·8 to 43·4%)
	Intracerebral haemorrhage	83·5% (79·1–88%)	314·5 (234·5–432·2)	-45·5% (-60 to -24·8%)	86·4% (81·8–90·7%)	11·5 (8·4–15·8)	-50·5% (-63·8 to -30·7%)
	Subarachnoid haemorrhage	72·5% (63·5–81·3%)	52 (36·8–75·5)	-50·1% (-65·4 to -22·6%)	80·2% (73·7–86·4%)	1·6 (1·1–2·4)	-53·6% (-69·2 to -25·8%)
	Neurological disorders*	12·7% (4·9–25·1%)	173·9 (59·9–434)	14·4% (-5·7 to 42·5%)	29·7% (14·3–45·7%)	11·4 (2·2–33·3)	15·8% (-8·8 to 53·2%)
	Alzheimer's disease and other dementias	42·6% (27·4–58·9%)	177 (63·3–436·3)	11·9% (-7·7 to 38·5%)	40·8% (25·5–57·7%)	11·7 (2·5–33·6)	14·2% (-10·4 to 48·9%)
	Parkinson's disease	-8·3% (-12·4–-4·4%)	-7 (-11·3–-3·5)	-17·9% (-41·6 to 19·3%)	-7·1% (-10·8–-3·7%)	-0·4 (-0·6–-0·2)	-18·9% (-44·8 to 24·8%)
	Idiopathic epilepsy	1·1% (0·3–1·7%)	1·6 (0·5–2·9)	218% (-16·4 to 732·1%)	1·3% (0·3–1·9%)	<0·1 (<0·1–<0·1)	249·8% (3·1 to 594·1%)
	Multiple sclerosis	10·8% (7·3–14·3%)	2·3 (1·5–3·2)	20·7% (-13·8 to 67%)	11·8% (6·6–16·6%)	<0·1 (<0·1–0·1)	23·1% (-32·4 to 102·5%)
Ambient particulate matter pollution	Meningitis	0·4% (0·2–0·7%)	0·1 (0·1–0·3)	-85·9% (-95·1 to -57·8%)	0·2% (0·1–0·4%)	<0·1 (<0·1–<0·1)	-86% (-95·1 to -57·8%)
	Encephalitis	0·4% (0·1–0·9%)	0·1 (0–0·1)	-2·3% (-73·3 to 192·4%)	0·2% (0·1–0·5%)	<0·1 (<0·1–<0·1)	-2·3% (-73·3 to 192·3%)
	Stroke	27% (20–33·3%)	424·7 (287·5–593·5)	19·5% (-11·7 to 73·8%)	23·7% (17·2–29·6%)	16·5 (10·7–23·9)	13·4% (-17 to 66·3%)
	Ischaemic stroke	26·6% (19·6–32·9%)	299 (201–418·1)	63% (21·5 to 130·8%)	22·9% (16·6–28·8%)	12·4 (8–18·3)	47·6% (6·4 to 115·7%)

Table S16 Regional and national disabilities and deaths related to neurological conditions attributable to risk factors in North Africa and Middle East countries

**Location**<br>Risk factor (alphabetic order)	Cause	**DALYs (95% UI)**<br>PAF (%)<br>2019	Rate per 100,000<br>2019	Rate change (%)<br>From 1990 to 2019	**Deaths (95% UI)**<br>PAF (%)<br>2019	Rate per 100,000<br>2019	Rate change (%)<br>From 1990 to 2019
	Intracerebral haemorrhage	28·5% (21·4–35·1%)	107·4 (68·7–155·4)	-26·2% (-47·9 to 13·4%)	26·7% (19·7–33%)	3·5 (2·3–5·2)	-33% (-53·3 to 4·9%)
	Subarachnoid haemorrhage	25·4% (18·6–32·1%)	18·2 (11·4–28)	-30·3% (-54·8 to 21·2%)	25·4% (18·8–31·6%)	0·5 (0·3–0·9)	-37·4% (-60·5 to 13·6%)
Behavioural risk factors‡	Meningitis	2·4% (1·4–4%)	0·9 (0·5–1·5)	-89·8% (-95 to -80·2%)	1·2% (0·7–2·1%)	<0·1 (<0·1–<0·1)	-89·8% (-95 to -80·2%)
	Encephalitis	2·5% (1·1–4·6%)	0·4 (0·2–0·7)	-27·6% (-73·3 to 77·2%)	1·4% (0·6–2·6%)	<0·1 (<0·1–<0·1)	-27·6% (-73·3 to 77·2%)
	Stroke	35·1% (29·9–41%)	552 (406·9–743·3)	-9·8% (-30·4 to 18·5%)	32·6% (26·7–38·9%)	22·7 (15·9–32·1)	-12·7% (-33·4 to 16·9%)
	Ischaemic stroke	36·9% (30·2–43·9%)	415 (296·2–568·9)	17·9% (-7·8 to 52%)	33·8% (26·6–41·2%)	18·3 (12·5–26·5)	8·2% (-17·6 to 43·9%)
	Intracerebral haemorrhage	31·2% (25·5–38%)	117·7 (80·2–173·3)	-46·8% (-62·3 to -23·8%)	28·9% (23·3–35·5%)	3·8 (2·6–5·7)	-51·2% (-66 to -30%)
	Subarachnoid haemorrhage	26·9% (21–34·1%)	19·3 (12·8–30·6)	-50·2% (-66·8 to -20·1%)	26·9% (21·2–33·9%)	0·6 (0·3–0·9)	-54·6% (-71·1 to -24%)
	Neurological disorders*	3·7% (1·4–7·6%)	50·9 (17·3–123·5)	-21·9% (-38·1 to 1·3%)	7·4% (2·4–12%)	2·9 (0·4–8·6)	-25·7% (-47·1 to 3·8%)
	Alzheimer's disease and other dementias	13% (8·3–17·5%)	54 (20–125·9)	-24·3% (-40 to -4%)	11·2% (6·9–15·3%)	3·2 (0·7–8·9)	-25·6% (-44·5 to 0·5%)
	Parkinson's disease	-8·3% (-12·4–-4·4%)	-7 (-11·3–-3·5)	-17·9% (-41·6 to 19·3%)	-7·1% (-10·8–-3·7%)	-0·4 (-0·6–-0·2)	-18·9% (-44·8 to 24·8%)
	Idiopathic epilepsy	1·1% (0·3–1·7%)	1·6 (0·5–2·9)	218% (-16·4 to 732·1%)	1·3% (0·3–1·9%)	<0·1 (<0·1–<0·1)	249·8% (3·1 to 594·1%)
	Multiple sclerosis	10·8% (7·3–14·3%)	2·3 (1·5–3·2)	20·7% (-13·8 to 67%)	11·8% (6·6–16·6%)	<0·1 (<0·1–0·1)	23·1% (-32·4 to 102·5%)
Child and maternal malnutrition	Meningitis	2·4% (1·4–4%)	0·9 (0·5–1·5)	-89·8% (-95 to -80·2%)	1·2% (0·7–2·1%)	<0·1 (<0·1–<0·1)	-89·8% (-95 to -80·2%)
	Encephalitis	2·5% (1·1–4·6%)	0·4 (0·2–0·7)	-27·6% (-73·3 to 77·2%)	1·4% (0·6–2·6%)	<0·1 (<0·1–<0·1)	-27·6% (-73·3 to 77·2%)
Diet high in red meat	Stroke	3·9% (1·4–6%)	61·3 (21·7–99·8)	-41·5% (-65·9 to -14·7%)	3% (1–4·6%)	2·1 (0·7–3·6)	-44·2% (-67·7 to -17·1%)
	Ischaemic stroke	3·6% (0·6–5·7%)	40·7 (6·4–70·3)	-15% (-54·2 to 15·3%)	2·7% (0·5–4·3%)	1·5 (0·2–2·6)	-21·9% (-54·2 to 9·7%)
	Intracerebral haemorrhage	4·7% (0·7–7·6%)	17·5 (2·5–32·9)	-63·3% (-81·5 to -44·4%)	4·1% (0·6–6·7%)	0·5 (0·1–1)	-66·2% (-82·1 to -49·2%)
	Subarachnoid haemorrhage	4·3% (0·7–7·3%)	3·1 (0·4–5·7)	-66% (-83·7 to -45%)	3·9% (0·6–6·6%)	0·1 (0–0·2)	-69·2% (-84·7 to -48·5%)
Diet high in sodium	Stroke	1·9% (0·3–7·5%)	30·1 (3·7–121·9)	-10·4% (-48 to 71%)	1·7% (0·3–6·6%)	1·2 (0·2–4·9)	-15·2% (-48·9 to 57·1%)
	Ischaemic stroke	1·8% (0·3–7·2%)	20·4 (2·7–83·4)	22·9% (-33 to 123·3%)	1·5% (0·3–6·1%)	0·8 (0·1–3·5)	12·8% (-37 to 109·4%)
	Intracerebral haemorrhage	2·2% (0·2–8·4%)	8·3 (0·8–33·9)	-42·4% (-67·7 to 13·2%)	2·1% (0·3–8%)	0·3 (0–1·2)	-48·5% (-71·6 to -2%)
	Subarachnoid haemorrhage	1·9% (0·2–7·3%)	1·4 (0·1–5·5)	-45·9% (-72·6 to 12·3%)	1·9% (0·2–7·4%)	<0·1 (<0·1–0·2)	-52% (-74·7 to 1·2%)
Diet low in fibre	Stroke	2·8% (0·6–5·7%)	44·8 (8·9–95·8)	43·6% (8 to 104·1%)	2·4% (0·5–4·8%)	1·7 (0·4–3·6)	32·6% (-3·3 to 93·6%)
	Ischaemic stroke	2·8% (0·6–5·4%)	31·2 (6·3–65·7)	99·2% (48·8 to 179·7%)	2·3% (0·5–4·5%)	1·3 (0·3–2·7)	72·7% (22·4 to 149·4%)
	Intracerebral haemorrhage	3·1% (0·6–6·2%)	11·6 (2·2–25·8)	-11·4% (-37·9 to 34·4%)	2·8% (0·6–5·6%)	0·4 (0·1–0·8)	-20·5% (-44·8 to 19·3%)
	Subarachnoid haemorrhage	2·9% (0·6–5·8%)	2·1 (0·4–4·7)	-17·5% (-45·2 to 35·1%)	2·8% (0·6–5·5%)	0·1 (0–0·1)	-25·1% (-51·5 to 26·5%)
Diet low in fruits	Stroke	4·5% (1·8–7·5%)	70·4 (27·9–127)	-5·8% (-37·7 to 33·5%)	3·6% (1·4–6·1%)	2·5 (1–4·7)	-9·6% (-41·4 to 31·1%)
	Ischaemic stroke	3·7% (0·7–7·2%)	42·2 (7·6–89·3)	47·3% (10·5 to 100·3%)	3% (0·6–5·7%)	1·6 (0·3–3·4)	34·2% (-2·6 to 88·4%)
	Intracerebral haemorrhage	6·4% (2·9–12%)	24 (10·2–48·5)	-38·1% (-56·3 to -10·7%)	5·8% (2·6–10·5%)	0·8 (0·3–1·5)	-42·8% (-59·9 to -16·9%)
	Subarachnoid haemorrhage	5·9% (2·6–11·1%)	4·2 (1·8–8·6)	-42·4% (-61·7 to -8·7%)	5·6% (2·5–10·4%)	0·1 (0–0·2)	-47% (-66·1 to -13·5%)
Diet low in vegetables	Stroke	1·9% (0·5–3·5%)	30 (7·5–59·1)	40·6% (-18 to 156·1%)	1·7% (0·5–3·1%)	1·2 (0·3–2·3)	24·3% (-25·6 to 112·5%)

Table S16 Regional and national disabilities and deaths related to neurological conditions attributable to risk factors in North Africa and Middle East countries

**Location**<br>Risk factor (alphabetic order)	Cause	**DALYs (95% UI)**<br>PAF (%)<br>2019	Rate per 100,000<br>2019	Rate change (%)<br>From 1990 to 2019	**Deaths (95% UI)**<br>PAF (%)<br>2019	Rate per 100,000<br>2019	Rate change (%)<br>From 1990 to 2019
	Ischaemic stroke	1·8% (0·2–3·6%)	20·3 (2·4–41·3)	83·7% (14·2 to 201%)	1·6% (0·2–3·1%)	0·9 (0·1–1·7)	56·3% (2·3 to 145·9%)
	Intracerebral haemorrhage	2·2% (0·2–6·2%)	8·3 (0·7–24)	-4·8% (-49·1 to 151·4%)	2·2% (0·2–6%)	0·3 (0–0·8)	-17·7% (-54·5 to 94·2%)
	Subarachnoid haemorrhage	2% (0·2–5·8%)	1·4 (0·1–4·4)	-8·9% (-53·1 to 165·8%)	2·1% (0·2–5·8%)	<0·1 (<0·1–0·1)	-20·7% (-57·8 to 112·7%)
Diet low in whole grains	Stroke	6% (2·8–8·1%)	94·7 (41–139·3)	17·2% (-8·7 to 50·9%)	5·8% (3–7·5%)	4 (1·9–6)	6% (-19·3 to 41·8%)
	Ischaemic stroke	8·4% (4–11·2%)	94·7 (41–139·3)	17·2% (-8·7 to 50·9%)	7·4% (3·8–9·6%)	4 (1·9–6)	6% (-19·3 to 41·8%)
Dietary risks	Stroke	19% (13·9–24·7%)	298·4 (201·9–422·4)	-5·7% (-28·6 to 25·7%)	16·8% (12·3–21·7%)	11·6 (7·9–17·1)	-9·8% (-32·1 to 20·9%)
	Ischaemic stroke	19·9% (13·8–25·7%)	223·7 (143·3–313·9)	22·9% (-5·5 to 58·9%)	17·1% (11·9–22·1%)	9·2 (5·8–13·5)	12·1% (-14·8 to 48·2%)
	Intracerebral haemorrhage	16·9% (10·5–24·3%)	63·7 (36·2–103)	-43·8% (-60·3 to -19·3%)	15·7% (9·8–22·8%)	2·1 (1·2–3·4)	-48·2% (-63·6 to -25·6%)
	Subarachnoid haemorrhage	15·4% (9·5–22·2%)	11 (6·1–18·1)	-47·5% (-65·4 to -16·9%)	15·1% (9·3–22·1%)	0·3 (0·2–0·5)	-51·8% (-68·9 to -19·3%)
Environmental/occupational risk factors§	Meningitis	0·4% (0·2–0·7%)	0·1 (0·1–0·3)	-91% (-96·9 to -75·8%)	0·2% (0·1–0·4%)	<0·1 (<0·1–<0·1)	-91% (-96·9 to -75·8%)
	Encephalitis	0·4% (0·1–0·9%)	0·1 (0–0·1)	-37·5% (-83·3 to 82·4%)	0·2% (0·1–0·5%)	<0·1 (<0·1–<0·1)	-37·5% (-83·3 to 82·5%)
	Stroke	32·3% (25·3–38·7%)	507·8 (354·4–695)	-20·2% (-38·7 to 5·5%)	30·2% (23·7–36·7%)	21 (14·3–29·4)	-21·6% (-41·1 to 5·8%)
	Ischaemic stroke	31·9% (24·9–38·4%)	358·6 (248·4–497)	9·9% (-14·6 to 43%)	29·5% (23·1–35·8%)	16 (10·6–22·9)	2·2% (-23·2 to 38·6%)
	Intracerebral haemorrhage	33·8% (26·3–40·3%)	127·3 (84·4–180·1)	-50·9% (-65 to -30·8%)	33% (25·9–39·7%)	4·4 (2·9–6·3)	-54·5% (-67·5 to -35·5%)
	Subarachnoid haemorrhage	30·4% (23·5–37·3%)	21·8 (14·1–33·2)	-56·9% (-70·3 to -31·8%)	31·6% (24·7–38·3%)	0·6 (0·4–1)	-58·3% (-72·4 to -32·5%)
High body-mass index	Stroke	38·5% (27·8–48%)	605·3 (392·5–858·3)	3·4% (-20·6 to 38·1%)	26·9% (18·1–35·6%)	18·7 (11·2–28·2)	1·4% (-25·3 to 40·9%)
	Ischaemic stroke	33·4% (23·1–43·1%)	375 (236·3–545·2)	60·9% (24·2 to 115·4%)	22·6% (14·3–31·3%)	12·3 (7–19·3)	52·5% (12·6 to 116·3%)
	Intracerebral haemorrhage	52·1% (39·7–62·9%)	196·3 (130·3–275·8)	-33·6% (-52·2 to -4%)	42% (30·3–53·2%)	5·6 (3·6–8·1)	-37% (-55·7 to -6·7%)
	Subarachnoid haemorrhage	47·5% (35·1–59·4%)	34·1 (21·9–50·6)	-40% (-59·5 to -5%)	40·4% (27·9–52·8%)	0·8 (0·5–1·3)	-44·9% (-64·8 to -7·9%)
	Neurological disorders*	6·4% (1·9–14·3%)	87·3 (23·8–223·1)	30·5% (6·2 to 71·5%)	15·2% (5·7–27·9%)	5·8 (1–16·8)	32·9% (2·2 to 84·4%)
	Alzheimer's disease and other dementias	21% (9·4–36·2%)	87·3 (23·8–223·1)	30·5% (6·2 to 71·5%)	20·2% (8·9–35%)	5·8 (1–16·8)	32·9% (2·2 to 84·4%)
High fasting plasma glucose	Stroke	32% (20·1–51·7%)	502·5 (284·9–866·2)	21·6% (-9·8 to 69·8%)	33·3% (18·8–61·2%)	23·1 (12·2–44·4)	6·7% (-20·7 to 49·6%)
	Ischaemic stroke	34·1% (17·9–61·4%)	382·7 (195·8–732·5)	49% (9·4 to 114·4%)	33·9% (16·3–68·4%)	18·3 (7·9–38·7)	28·7% (-5 to 83·3%)
	Intracerebral haemorrhage	27·6% (17·3–39·9%)	104 (57·9–163·5)	-23·1% (-47·2 to 16·2%)	31·5% (19·6–46·8%)	4·2 (2·3–6·9)	-35·2% (-54·4 to -2%)
	Subarachnoid haemorrhage	22% (13·5–33·4%)	15·8 (8·6–26·3)	-25·2% (-53·5 to 29·7%)	28·6% (18·1–43·5%)	0·6 (0·3–1)	-37·9% (-60·9 to 10·7%)
	Neurological disorders*	5% (0·9–13·4%)	67·7 (12·3–213·8)	60·6% (35·8 to 100·8%)	12·3% (2·8–26·9%)	4·7 (0·6–16·1)	60·1% (29·4 to 111·2%)
	Alzheimer's disease and other dementias	16·3% (4·1–33%)	67·7 (12·3–213·8)	60·6% (35·8 to 100·8%)	16·3% (4·1–33·2%)	4·7 (0·6–16·1)	60·1% (29·4 to 111·2%)
High LDL cholesterol	Stroke	17·6% (11·3–27·8%)	277·1 (162·6–451·8)	43·8% (12·1 to 94·4%)	15·7% (7–30·5%)	11 (4·5–22·2)	28·2% (-1·9 to 83·9%)
	Ischaemic stroke	24·7% (15·7–38·8%)	277·1 (162·6–451·8)	43·8% (12·1 to 94·4%)	20·2% (8·7–39·9%)	11 (4·5–22·2)	28·2% (-1·9 to 83·9%)
High systolic blood pressure	Stroke	56·9% (48–64·3%)	893·2 (668·4–1153·5)	-9·9% (-30·1 to 17·9%)	54% (43·7–63·2%)	37·5 (26·4–51·9)	-15·6% (-36·4 to 13·6%)
	Ischaemic stroke	56% (45·4–65%)	628·9 (458·2–827·5)	21% (-6 to 56·9%)	52% (40–63·4%)	28·2 (19·1–40·3)	9·3% (-18·7 to 48·2%)

Table S16 Regional and national disabilities and deaths related to neurological conditions attributable to risk factors in North Africa and Middle East countries

**Location**<br>Risk factor (alphabetic order)	Cause	**DALYs (95% UI)**<br>PAF (%)<br>2019	 Rate per 100,000<br>2019	 Rate change (%)<br>From 1990 to 2019	**Deaths (95% UI)**<br>PAF (%)<br>2019	 Rate per 100,000<br>2019	 Rate change (%)<br>From 1990 to 2019
	Intracerebral haemorrhage	60·2% (50·9–68·5%)	226·8 (163·5–313·3)	-43·6% (-59·3 to -19·9%)	61·3% (50–71·4%)	8·1 (5·8–11·5)	-49·7% (-63·7 to -28%)
	Subarachnoid haemorrhage	52·3% (42·1–63%)	37·5 (25·7–56·1)	-46·3% (-63·7 to -16·7%)	57·4% (46·5–67·4%)	1·2 (0·8–1·8)	-52·3% (-68·9 to -22·6%)
High temperature	Stroke	1·6% (-0·4–5%)	25·1 (-6·1–78·1)	-4·7% (-177·2 to 223%)	1·9% (-0·4–6%)	1·3 (-0·3–4)	5·1% (-187·6 to 261·2%)
	Ischaemic stroke	1·5% (-0·4–4·8%)	17·3 (-4·2–53·7)	39·8% (-212·2 to 366·3%)	1·9% (-0·4–6%)	1 (-0·2–3·1)	35·4% (-211·9 to 352·5%)
	Intracerebral haemorrhage	1·7% (-0·4–5·6%)	6·6 (-1·6–20·5)	-39·7% (-149·5 to 115·9%)	1·9% (-0·4–6%)	0·3 (-0·1–0·8)	-39·9% (-151·1 to 117·6%)
	Subarachnoid haemorrhage	1·6% (-0·4–5·2%)	1·2 (-0·3–3·9)	-60·4% (-127·5 to 26·2%)	1·9% (-0·4–6%)	<0·1 (<0·1–0·1)	-49·5% (-138·4 to 77·5%)
Household air pollution from solid fuels	Meningitis	0% (0–0%)	<0·1 (<0·1–<0·1)	-99·9% (-100 to -99·7%)	0% (0–0%)	<0·1 (<0·1–<0·1)	-99·9% (-100 to -99·7%)
	Encephalitis	0% (0–0%)	<0·1 (<0·1–<0·1)	-99·5% (-99·9 to -98·1%)	0% (0–0%)	<0·1 (<0·1–<0·1)	-99·5% (-99·9 to -98·1%)
	Stroke	0·1% (0–0·2%)	1 (0·3–2·6)	-99·4% (-99·8 to -98·4%)	0·1% (0–0·1%)	<0·1 (<0·1–0·1)	-99·4% (-99·8 to -98·5%)
	Ischaemic stroke	0·1% (0–0·2%)	0·7 (0·2–1·8)	-99·2% (-99·7 to -97·8%)	0·1% (0–0·1%)	<0·1 (<0·1–0·1)	-99·2% (-99·8 to -98%)
	Intracerebral haemorrhage	0·1% (0–0·2%)	0·3 (0·1–0·6)	-99·6% (-99·9 to -99%)	0·1% (0–0·2%)	<0·1 (<0·1–<0·1)	-99·7% (-99·9 to -99·1%)
	Subarachnoid haemorrhage	0·1% (0–0·2%)	<0·1 (<0·1–0·1)	-99·6% (-99·9 to -99%)	0·1% (0–0·2%)	<0·1 (<0·1–<0·1)	-99·7% (-99·9 to -99·1%)
Kidney dysfunction	Stroke	11·4% (9–13·6%)	179·3 (125–238·2)	24·2% (-4·8 to 62·4%)	11·1% (7·5–14·4%)	7·7 (4·7–11)	18·2% (-12·7 to 59·8%)
	Ischaemic stroke	12·9% (9·7–16%)	145 (98·5–195·9)	56·6% (20·5 to 103·6%)	11·5% (7·1–15·7%)	6·2 (3·5–9·4)	47% (7·2 to 100%)
	Intracerebral haemorrhage	9·1% (7·4–11%)	34·4 (24·4–48·1)	-33·7% (-51·5 to -8·5%)	11·2% (8·6–13·7%)	1·5 (1–2·1)	-35·3% (-52·4 to -10·1%)
Lead exposure	Stroke	2·2% (0·8–4%)	35·3 (11·2–68·2)	-48·3% (-67·9 to -29·9%)	2·7% (1·1–4·5%)	1·9 (0·7–3·4)	-39·2% (-59·5 to -17·7%)
	Ischaemic stroke	2·4% (0·8–4·2%)	26·8 (8·7–50·6)	-23·9% (-52·8 to 3%)	2·7% (1·1–4·6%)	1·5 (0·6–2·7)	-15·9% (-42·3 to 12·9%)
	Intracerebral haemorrhage	2% (0·5–4%)	7·4 (1·8–15·9)	-74% (-86·4 to -60·8%)	2·6% (0·9–4·8%)	0·4 (0·1–0·7)	-69·5% (-81·8 to -55·4%)
	Subarachnoid haemorrhage	1·5% (0·4–3·3%)	1·1 (0·3–2·5)	-76·9% (-88·3 to -62·2%)	2·4% (0·8–4·4%)	<0·1 (<0·1–0·1)	-71·3% (-83 to -53·6%)
Low birth weight	Meningitis	2·2% (1·3–3·7%)	0·8 (0·4–1·4)	-89·9% (-95·1 to -80·2%)	1·1% (0·6–1·9%)	<0·1 (<0·1–<0·1)	-89·9% (-95·1 to -80·2%)
	Encephalitis	2·3% (1–4·2%)	0·4 (0·2–0·7)	-28·7% (-74·3 to 76·3%)	1·3% (0·6–2·4%)	<0·1 (<0·1–<0·1)	-28·7% (-74·3 to 76·3%)
Low birth weight and short gestation	Meningitis	2·4% (1·4–4%)	0·9 (0·5–1·5)	-89·8% (-95 to -80·2%)	1·2% (0·7–2·1%)	<0·1 (<0·1–<0·1)	-89·8% (-95 to -80·2%)
	Encephalitis	2·5% (1·1–4·6%)	0·4 (0·2–0·7)	-27·6% (-73·3 to 77·2%)	1·4% (0·6–2·6%)	<0·1 (<0·1–<0·1)	-27·6% (-73·3 to 77·2%)
Low physical activity	Stroke	5·7% (1·2–12·7%)	89·1 (17·7–202·7)	19·4% (-7·3 to 53·5%)	7·3% (1·7–14·9%)	5·1 (1·2–10·9)	8·8% (-16·8 to 45%)
	Ischaemic stroke	8% (1·7–17·6%)	89·1 (17·7–202·7)	19·4% (-7·3 to 53·5%)	9·4% (2·2–19%)	5·1 (1·2–10·9)	8·8% (-16·8 to 45%)
Low temperature	Stroke	3·4% (-0·7–7·9%)	54·2 (-10–126·5)	-23·6% (-70·7 to 25·9%)	4·1% (-0·8–9·5%)	2·8 (-0·5–6·6)	-15·9% (-68·1 to 40·8%)
	Ischaemic stroke	3·3% (-0·7–7·7%)	37·4 (-7·1–87·9)	11·9% (-58·7 to 84·5%)	4·1% (-0·8–9·5%)	2·2 (-0·4–5·1)	8·3% (-59·1 to 80·2%)
	Intracerebral haemorrhage	3·8% (-0·7–8·7%)	14·2 (-2·5–33·6)	-51·5% (-81·4 to -18·8%)	4·1% (-0·8–9·5%)	0·5 (-0·1–1·3)	-51·9% (-81·9 to -18·7%)
	Subarachnoid haemorrhage	3·5% (-0·7–8·2%)	2·6 (-0·5–6·3)	-68·9% (-88·5 to -36·2%)	4·1% (-0·8–9·5%)	0·1 (0–0·2)	-60·1% (-83·6 to -21·7%)
Metabolic risk factors†	Stroke	77·2% (70·3–83·6%)	1212 (935·5–1569·1)	-5·9% (-25·8 to 22·1%)	75·1% (65·4–85·8%)	52·2 (38·8–70·6)	-10·9% (-31·4 to 17·7%)
	Ischaemic stroke	78·5% (70·3–87%)	881·1 (670·1–1145·3)	24% (-3·1 to 59·9%)	74·7% (62·8–87·8%)	40·4 (28·8–56·4)	13·1% (-14·4 to 50·8%)
	Intracerebral haemorrhage	75·6% (69·3–81·6%)	284·9 (210·4–393·2)	-42·3% (-57·6 to -19·4%)	77·4% (69·9–84·4%)	10·3 (7·5–14·4)	-48·3% (-62·4 to -26·9%)
	Subarachnoid haemorrhage	64·3% (53·9–74·4%)	46·1 (32·3–67·4)	-45·4% (-63 to -14·9%)	70·3% (61–78·6%)	1·4 (1–2·1)	-51·2% (-68 to -20·6%)

Table S16 Regional and national disabilities and deaths related to neurological conditions attributable to risk factors in North Africa and Middle East countries

Location<br>Risk factor (alphabetic order)	Cause	DALYs (95% UI)<br>PAF (%)<br>2019	Rate per 100,000<br>2019	Rate change (%)<br>From 1990 to 2019	Deaths (95% UI)<br>PAF (%)<br>2019	Rate per 100,000<br>2019	Rate change (%)<br>From 1990 to 2019
	Neurological disorders*	10·3% (3·5–21·9%)	140·7 (41·9–351·9)	37·2% (13·7 to 72·3%)	25% (10·6–41·8%)	9·5 (1·7–27·9)	38·9% (8·6 to 84·2%)
	Alzheimer's disease and other dementias	33·9% (17·2–52·7%)	140·7 (41·9–351·9)	37·2% (13·7 to 72·3%)	33·2% (16·2–52%)	9·5 (1·7–27·9)	38·9% (8·6 to 84·2%)
Non-optimal temperature	Stroke	5% (1·8–8·7%)	78·5 (27·3–143·3)	-18·7% (-49·3 to 19·7%)	5·9% (2·2–10·3%)	4·1 (1·5–7·5)	-10·4% (-42·6 to 29·3%)
	Ischaemic stroke	4·8% (1·8–8·4%)	54·2 (18·7–99·8)	19·2% (-27·3 to 71·5%)	5·9% (2·2–10·3%)	3·2 (1·1–5·9)	15·5% (-27 to 62·3%)
	Intracerebral haemorrhage	5·5% (2–9·4%)	20·6 (7–38)	-48·4% (-68·2 to -21·3%)	5·9% (2·2–10·3%)	0·8 (0·3–1·4)	-48·7% (-68·4 to -20·7%)
	Subarachnoid haemorrhage	5·1% (1·9–9%)	3·7 (1·3–7·2)	-66·7% (-81·7 to -37%)	5·9% (2·2–10·3%)	0·1 (0–0·2)	-57·3% (-75·3 to -25·3%)
Other environmental risks	Stroke	2·2% (0·8–4%)	35·3 (11·2–68·2)	-48·3% (-67·9 to -29·9%)	2·7% (1·1–4·5%)	1·9 (0·7–3·4)	-39·2% (-59·5 to -17·7%)
	Ischaemic stroke	2·4% (0·8–4·2%)	26·8 (8·7–50·6)	-23·9% (-52·8 to 3%)	2·7% (1·1–4·6%)	1·5 (0·6–2·7)	-15·9% (-42·3 to 12·9%)
	Intracerebral haemorrhage	2% (0·5–4%)	7·4 (1·8–15·9)	-74% (-86·4 to -60·8%)	2·6% (0·9–4·8%)	0·4 (0·1–0·7)	-69·5% (-81·8 to -55·4%)
	Subarachnoid haemorrhage	1·5% (0·4–3·3%)	1·1 (0·3–2·5)	-76·9% (-88·3 to -62·2%)	2·4% (0·8–4·4%)	<0·1 (<0·1–0·1)	-71·3% (-83 to -53·6%)
Particulate matter pollution	Meningitis	0·4% (0·2–0·7%)	0·1 (0·1–0·3)	-91% (-96·9 to -75·8%)	0·2% (0·1–0·4%)	<0·1 (<0·1–<0·1)	-91% (-96·9 to -75·8%)
	Encephalitis	0·4% (0·1–0·9%)	0·1 (0–0·1)	-37·5% (-83·3 to 82·4%)	0·2% (0·1–0·5%)	<0·1 (<0·1–<0·1)	-37·5% (-83·3 to 82·5%)
	Stroke	27·1% (20·1–33·4%)	425·7 (287·9–594·2)	-18·2% (-37·4 to 8·4%)	23·8% (17·2–29·6%)	16·5 (10·8–24)	-22·3% (-41·6 to 5·6%)
	Ischaemic stroke	26·7% (19·7–33%)	299·7 (201·4–419·3)	11·5% (-14·3 to 44·7%)	23% (16·7–28·9%)	12·4 (8–18·4)	1·2% (-24·3 to 38·6%)
	Intracerebral haemorrhage	28·5% (21·5–35·2%)	107·7 (68·7–156)	-49·4% (-63·9 to -28·8%)	26·8% (19·7–33%)	3·6 (2·3–5·3)	-54% (-67·5 to -34%)
	Subarachnoid haemorrhage	25·5% (18·6–32·2%)	18·3 (11·4–28)	-52·5% (-68·1 to -25·2%)	25·5% (18·9–31·6%)	0·5 (0·3–0·9)	-57·2% (-72 to -30·1%)
Secondhand smoke	Stroke	5·1% (3·8–6·3%)	79·4 (53·5–110·4)	-6·4% (-28·5 to 25·3%)	4·3% (3·3–5·3%)	3 (2–4·3)	-9·7% (-31 to 23%)
	Ischaemic stroke	4·8% (3·6–5·9%)	53·5 (36·6–74·1)	34·2% (1·8 to 75·9%)	4% (3–5%)	2·2 (1·4–3·1)	21·3% (-9·3 to 63·5%)
	Intracerebral haemorrhage	5·9% (4·4–7·5%)	22·1 (14·4–32·7)	-41·6% (-58·4 to -15·1%)	5·3% (4–6·7%)	0·7 (0·5–1)	-45·7% (-61·1 to -21·4%)
	Subarachnoid haemorrhage	5·2% (3·7–7%)	3·8 (2·3–5·7)	-46·8% (-65 to -13·7%)	5·2% (3·8–6·7%)	0·1 (0·1–0·2)	-50·5% (-67·9 to -17%)
Short gestation	Meningitis	1·7% (1–2·9%)	0·6 (0·4–1·1)	-88·1% (-94 to -77%)	0·9% (0·5–1·5%)	<0·1 (<0·1–<0·1)	-88·1% (-94 to -77%)
	Encephalitis	1·8% (0·8–3·3%)	0·3 (0·2–0·5)	-15·9% (-69·2 to 102·6%)	1% (0·5–1·9%)	<0·1 (<0·1–<0·1)	-15·9% (-69·2 to 102·6%)
Smoking	Stroke	11·2% (9·4–13%)	175·8 (124·8–246·9)	-25·4% (-45·4 to 3·6%)	8·9% (7·3–10·7%)	6·2 (4·4–8·9)	-29·2% (-50 to 0·4%)
	Ischaemic stroke	10·8% (9·2–12·6%)	121·4 (87·3–172)	3·6% (-25·5 to 42%)	8·3% (6·9–9·9%)	4·5 (3·1–6·6)	-4·4% (-34·5 to 37·8%)
	Intracerebral haemorrhage	12·4% (9·3–15·6%)	47·1 (29·5–72·6)	-53·9% (-70·4 to -30·5%)	11·1% (8·1–14·1%)	1·5 (0·9–2·3)	-58·1% (-73·3 to -35·2%)
	Subarachnoid haemorrhage	10·1% (7·6–13·7%)	7·3 (4·7–12·7)	-55·7% (-73·2 to -18·8%)	10% (7·2–13·4%)	0·2 (0·1–0·4)	-60·2% (-77·5 to -22·8%)
	Neurological disorders*	3·6% (1·2–7·5%)	49·3 (15·4–122·1)	-23·8% (-40·5 to -1·8%)	7·3% (2·3–11·9%)	2·9 (0·4–8·6)	-26% (-47·5 to 3·1%)
	Alzheimer's disease and other dementias	13% (8·3–17·5%)	54 (20–125·9)	-24·3% (-40 to -4%)	11·2% (6·9–15·3%)	3·2 (0·7–8·9)	-25·6% (-44·5 to 0·5%)
	Parkinson's disease	-8·3% (-12·4–-4·4%)	-7 (-11·3–-3·5)	-17·9% (-41·6 to 19·3%)	-7·1% (-10·8–-3·7%)	-0·4 (-0·6–-0·2)	-18·9% (-44·8 to 24·8%)
	Multiple sclerosis	10·8% (7·3–14·3%)	2·3 (1·5–3·2)	20·7% (-13·8 to 67%)	11·8% (6·6–16·6%)	<0·1 (<0·1–0·1)	23·1% (-32·4 to 102·5%)
Tobacco	Stroke	15·7% (13·7–18%)	246·5 (181–335·4)	-20·3% (-39·9 to 8·1%)	12·8% (11–14·8%)	8·9 (6·4–12·4)	-23·8% (-44 to 5·7%)

Table S16 Regional and national disabilities and deaths related to neurological conditions attributable to risk factors in North Africa and Middle East countries

Location<br>Risk factor (alphabetic order)	Cause	DALYs (95% UI)<br>PAF (%)<br>2019	Rate per 100,000<br>2019	Rate change (%)<br>From 1990 to 2019	Deaths (95% UI)<br>PAF (%)<br>2019	Rate per 100,000<br>2019	Rate change (%)<br>From 1990 to 2019
	Ischaemic stroke	15% (13·1–17·1%)	169·1 (122·8–230·4)	11·4% (-17·7 to 49·2%)	12% (10·2–13·8%)	6·5 (4·6–9·3)	2·6% (-25·8 to 42%)
	Intracerebral haemorrhage	17·7% (14·6–21%)	66·7 (45–97·8)	-50·5% (-66 to -27·1%)	15·8% (12·9–19%)	2·1 (1·4–3·2)	-54·7% (-69·2 to -33%)
	Subarachnoid haemorrhage	14·8% (12–18·5%)	10·7 (7·1–17·3)	-52·9% (-69·9 to -21·6%)	14·7% (11·9–18·4%)	0·3 (0·2–0·5)	-57·3% (-74·4 to -24·8%)
	Neurological disorders*	3·6% (1·2–7·5%)	49·3 (15·4–122·1)	-23·8% (-40·5 to -1·8%)	7·3% (2·3–11·9%)	2·9 (0·4–8·6)	-26% (-47·5 to 3·1%)
	Alzheimer's disease and other dementias	13% (8·3–17·5%)	54 (20–125·9)	-24·3% (-40 to -4%)	11·2% (6·9–15·3%)	3·2 (0·7–8·9)	-25·6% (-44·5 to 0·5%)
	Parkinson's disease	-8·3% (-12·4–-4·4%)	-7 (-11·3–-3·5)	-17·9% (-41·6 to 19·3%)	-7·1% (-10·8–-3·7%)	-0·4 (-0·6–-0·2)	-18·9% (-44·8 to 24·8%)
	Multiple sclerosis	10·8% (7·3–14·3%)	2·3 (1·5–3·2)	20·7% (-13·8 to 67%)	11·8% (6·6–16·6%)	<0·1 (<0·1–0·1)	23·1% (-32·4 to 102·5%)
**Morocco**							
Air pollution	Meningitis	1·6% (0·7–3·1%)	1·1 (0·4–2·3)	-85·6% (-95·6 to -54·2%)	0·9% (0·4–1·9%)	<0·1 (<0·1–<0·1)	-85·6% (-95·6 to -54·2%)
	Encephalitis	0·9% (0·3–2·1%)	0·2 (0·1–0·5)	-7·1% (-70·9 to 190%)	0·6% (0·1–1·2%)	<0·1 (<0·1–<0·1)	-7·1% (-70·9 to 190%)
	Stroke	26·3% (22–30·4%)	593·3 (444·8–767·1)	-17·8% (-37·4 to 4·6%)	22·6% (18·6–26·4%)	26·3 (19·7–33·9)	-14·6% (-35 to 8·1%)
	Ischaemic stroke	25·3% (21·1–29·4%)	405·3 (307·6–519·3)	20·7% (-8·7 to 51·1%)	21·7% (17·8–25·4%)	19·3 (14·4–25·1)	19·1% (-11·4 to 50·6%)
	Intracerebral haemorrhage	28·7% (24–33·1%)	159·3 (114·3–213·7)	-50·4% (-64·6 to -30·2%)	25·5% (21·2–29·6%)	6 (4·4–7·9)	-51·1% (-64·9 to -31·8%)
	Subarachnoid haemorrhage	28·5% (23·8–33·2%)	28·7 (18·9–42·7)	-55·7% (-72·1 to -22·6%)	25·3% (20·7–29·9%)	1 (0·6–1·5)	-56·7% (-72·5 to -20·3%)
Alcohol use	Stroke	0·3% (0–0·6%)	6 (0–12·9)	-73·6% (-100·1 to -49·1%)	0·1% (-0·1–0·3%)	0·2 (-0·1–0·4)	-78·2% (-119·6 to -49·5%)
	Ischaemic stroke	0% (-0·2–0·3%)	0·3 (-3·8–4·6)	-88% (-1002·1 to 790·8%)	0% (-0·2–0·2%)	0 (-0·2–0·2)	-132·3% (-1206·1 to 930·1%)
	Intracerebral haemorrhage	1% (0·3–1·8%)	5·7 (1·8–10·9)	-71·7% (-87·3 to -52·6%)	0·7% (0·2–1·3%)	0·2 (0–0·3)	-72·6% (-88·9 to -52·3%)
	Neurological disorders*	0·2% (0–0·3%)	2 (0·6–4·6)	-41·1% (-80·8 to 45·7%)	0·1% (0–0·2%)	<0·1 (<0·1–<0·1)	-49·3% (-71·2 to -20·2%)
	Idiopathic epilepsy	1·5% (0·8–2·4%)	2 (0·6–4·6)	-41·1% (-80·8 to 45·7%)	1·6% (0·7–2·7%)	<0·1 (<0·1–<0·1)	-49·3% (-71·2 to -20·2%)
All risk factors	Meningitis	10·2% (5·4–17·2%)	7 (3·3–13)	-86·8% (-94·7 to -68%)	5·8% (3–10·3%)	0·1 (0–0·1)	-86·8% (-94·7 to -68%)
	Encephalitis	5·7% (2–10·9%)	1·4 (0·6–2·5)	-19·3% (-65·8 to 82·2%)	3·4% (1·1–6·5%)	<0·1 (<0·1–<0·1)	-19·3% (-65·8 to 82·2%)
	Stroke	88·4% (84·6–92·2%)	1995·8 (1606·4–2406·6)	-14·1% (-32 to 6·4%)	86·5% (81·3–92·3%)	100·7 (79·6–122·9)	-9·6% (-27·7 to 11·8%)
	Ischaemic stroke	88·8% (84–93·8%)	1424·1 (1149·7–1720·8)	22·2% (-4·4 to 48·1%)	86·5% (79·6–94%)	76·9 (60·6–95·6)	22·2% (-5·8 to 48·8%)
	Intracerebral haemorrhage	87·9% (83·9–91·4%)	487·1 (373·9–621·4)	-49·7% (-63·3 to -30·8%)	87% (81·7–91·9%)	20·5 (15·9–25·6)	-50·1% (-63·1 to -31·7%)
	Subarachnoid haemorrhage	84·3% (79·3–88·3%)	84·6 (59·6–123·3)	-55·4% (-71·1 to -23·1%)	84·4% (78–90%)	3·3 (2·3–4·8)	-55·5% (-71·3 to -18·7%)
	Neurological disorders*	9·8% (3·7–20·3%)	131·4 (44·8–341·5)	25·7% (2·6 to 49·4%)	22·7% (9·8–37·4%)	8·2 (1·4–26)	33·3% (6·7 to 65·8%)
	Alzheimer's disease and other dementias	34·4% (20·9–49·7%)	133 (45·8–340·7)	26·7% (3·7 to 50·4%)	33·1% (19·7–48·7%)	8·4 (1·7–26·3)	32·3% (5·7 to 61·6%)
	Parkinson's disease	-5·2% (-8–-2·6%)	-5 (-7·8–-2·4)	-7·9% (-33·3 to 24·8%)	-4·6% (-7·2–-2·2%)	-0·3 (-0·4–-0·1)	-1·8% (-30·7 to 36·2%)
	Idiopathic epilepsy	1·5% (0·8–2·4%)	2 (0·6–4·6)	-41·1% (-80·8 to 45·7%)	1·6% (0·7–2·7%)	<0·1 (<0·1–<0·1)	-49·3% (-71·2 to -20·2%)
	Multiple sclerosis	6·8% (4·9–8·8%)	1·4 (0·9–1·9)	-9·9% (-36·8 to 23·1%)	7·1% (4·8–9·8%)	<0·1 (<0·1–<0·1)	-9·7% (-48·2 to 38·9%)

Table S16 Regional and national disabilities and deaths related to neurological conditions attributable to risk factors in North Africa and Middle East countries

Location<br>Risk factor (alphabetic order)	Cause	DALYs (95% UI)<br>PAF (%)<br>2019	Rate per 100,000<br>2019	Rate change (%)<br>From 1990 to 2019	Deaths (95% UI)<br>PAF (%)<br>2019	Rate per 100,000<br>2019	Rate change (%)<br>From 1990 to 2019
Ambient particulate matter pollution	Meningitis	1·5% (0·6–2·9%)	1 (0·4–2·2)	-62·4% (-88·6 to 45·9%)	0·9% (0·3–1·8%)	<0·1 (<0·1–<0·1)	-62·4% (-88·6 to 45·9%)
	Encephalitis	0·9% (0·2–1·9%)	0·2 (0·1–0·4)	143·6% (-31·5 to 922·8%)	0·5% (0·1–1·2%)	<0·1 (<0·1–<0·1)	143·6% (-31·5 to 922·8%)
	Stroke	24·7% (20·3–29%)	557·6 (417·5–724·5)	93·7% (24·3 to 264%)	21·2% (17·3–25·1%)	24·7 (18·4–32·2)	101·4% (30·5 to 271·6%)
	Ischaemic stroke	23·7% (19·4–28·1%)	380·9 (286·4–493)	185·6% (81·9 to 418·2%)	20·4% (16·6–24·2%)	18·1 (13·5–23·9)	182·5% (81·7 to 414·8%)
	Intracerebral haemorrhage	27% (22·3–31·6%)	149·8 (107·3–201·2)	16·1% (-29·5 to 125·9%)	24% (19·6–28·2%)	5·7 (4·1–7·5)	14·4% (-29·8 to 123·1%)
	Subarachnoid haemorrhage	26·8% (22–31·5%)	26·9 (17·5–40·9)	5·5% (-42·5 to 140·5%)	23·7% (19·2–28·2%)	0·9 (0·6–1·4)	2·9% (-44·5 to 138·9%)
Behavioural risk factors‡	Meningitis	9·8% (5·3–16·6%)	6·8 (3·1–12·7)	-86·8% (-94·8 to -67·4%)	5·6% (2·8–9·9%)	0·1 (0–0·1)	-86·8% (-94·8 to -67·4%)
	Encephalitis	5·4% (2–10·4%)	1·3 (0·6–2·4)	-19·3% (-65·2 to 84·1%)	3·3% (1·1–6·2%)	<0·1 (<0·1–<0·1)	-19·3% (-65·3 to 84·1%)
	Stroke	29·4% (24·1–35·1%)	662·8 (496·7–874·4)	-27·8% (-44·9 to -8%)	27% (21·5–33·2%)	31·4 (23·4–41·8)	-22·1% (-41·1 to -2·4%)
	Ischaemic stroke	31·3% (24·5–38·7%)	502·6 (369·2–674·2)	5·7% (-21·3 to 32·2%)	28·9% (21·8–36·6%)	25·7 (18·6–35·1)	7% (-20·3 to 32·7%)
	Intracerebral haemorrhage	24·7% (19·7–30·6%)	137 (96·8–189)	-63·2% (-74·6 to -47·2%)	21·2% (16·7–26·6%)	5 (3·6–7)	-64·1% (-75·2 to -49·3%)
	Subarachnoid haemorrhage	23·1% (17·9–28·7%)	23·2 (14·3–37·1)	-66·6% (-79·4 to -40·7%)	19·9% (15–25·5%)	0·8 (0·5–1·2)	-67·7% (-79·8 to -39·7%)
	Neurological disorders*	2·6% (0·9–5·5%)	34·6 (11·7–87·4)	-27·7% (-44·6 to -12·9%)	5·1% (1·3–9%)	1·9 (0·2–6·1)	-22·9% (-50·3 to -3·2%)
	Alzheimer's disease and other dementias	9·4% (5·6–13·4%)	36·2 (13·3–88·2)	-25·1% (-40·2 to -9·7%)	8·4% (4·9–12·3%)	2·1 (0·5–6·4)	-20·5% (-40·6 to 0·5%)
	Parkinson's disease	-5·2% (-8–-2·6%)	-5 (-7·8–-2·4)	-7·9% (-33·3 to 24·8%)	-4·6% (-7·2–-2·2%)	-0·3 (-0·4–-0·1)	-1·8% (-30·7 to 36·2%)
	Idiopathic epilepsy	1·5% (0·8–2·4%)	2 (0·6–4·6)	-41·1% (-80·8 to 45·7%)	1·6% (0·7–2·7%)	<0·1 (<0·1–<0·1)	-49·3% (-71·2 to -20·2%)
	Multiple sclerosis	6·8% (4·9–8·8%)	1·4 (0·9–1·9)	-9·9% (-36·8 to 23·1%)	7·1% (4·8–9·8%)	<0·1 (<0·1–<0·1)	-9·7% (-48·2 to 38·9%)
Child and maternal malnutrition	Meningitis	9·8% (5·3–16·6%)	6·8 (3·1–12·7)	-86·8% (-94·8 to -67·4%)	5·6% (2·8–9·9%)	0·1 (0–0·1)	-86·8% (-94·8 to -67·4%)
	Encephalitis	5·4% (2–10·4%)	1·3 (0·6–2·4)	-19·3% (-65·2 to 84·1%)	3·3% (1·1–6·2%)	<0·1 (<0·1–<0·1)	-19·3% (-65·3 to 84·1%)
Diet high in red meat	Stroke	4% (1·5–6%)	90·8 (33·1–143·8)	-0·1% (-35·1 to 59·6%)	3·1% (1·1–4·7%)	3·6 (1·3–5·6)	2·4% (-33·2 to 59·9%)
	Ischaemic stroke	3·6% (0·6–5·5%)	57·7 (9·5–95·8)	55·8% (15·1 to 128·3%)	2·7% (0·5–4·2%)	2·4 (0·4–4·1)	53·1% (12·7 to 119·9%)
	Intracerebral haemorrhage	5% (0·8–8%)	27·9 (4·3–49·2)	-37·4% (-56·7 to 0·9%)	4·2% (0·7–6·7%)	1 (0·2–1·7)	-38·2% (-57 to -4·1%)
	Subarachnoid haemorrhage	5·2% (0·8–8·3%)	5·2 (0·7–9·8)	-44·1% (-64·1 to 7·5%)	4·2% (0·6–6·9%)	0·2 (0–0·3)	-45·4% (-65·1 to 9·9%)
Diet high in sodium	Stroke	1·9% (0·3–7·7%)	43·8 (5·7–173·3)	-20·4% (-55·3 to 47·4%)	1·6% (0·3–6·5%)	1·9 (0·3–7·7)	-16·8% (-53·9 to 51·4%)
	Ischaemic stroke	1·8% (0·3–7%)	28·9 (4–112·5)	19·5% (-38 to 108·1%)	1·5% (0·3–6%)	1·4 (0·2–5·4)	20·1% (-39 to 111·4%)
	Intracerebral haemorrhage	2·3% (0·3–9·2%)	12·7 (1·4–52·8)	-51% (-72·2 to -1·3%)	2·1% (0·3–8·5%)	0·5 (0·1–2)	-51·3% (-74·1 to -9·8%)
	Subarachnoid haemorrhage	2·1% (0·2–8·5%)	2·2 (0·2–8·9)	-55·3% (-79·3 to -2·8%)	2% (0·3–7·9%)	0·1 (0–0·3)	-55·8% (-79·9 to 1%)
Diet low in fibre	Stroke	0·3% (0·2–0·5%)	6·4 (4·3–10·6)	-25·7% (-46·2 to -0·6%)	0·3% (0·2–0·5%)	0·3 (0·2–0·6)	-29·3% (-54·1 to -0·4%)
	Ischaemic stroke	0·3% (0·2–0·4%)	4·5 (3·1–7·3)	4·1% (-25·5 to 37·1%)	0·3% (0·2–0·5%)	0·3 (0·2–0·4)	-6·8% (-40·6 to 34·1%)
	Intracerebral haemorrhage	0·3% (0·2–0·5%)	1·6 (1–2·8)	-55·3% (-69·3 to -35·1%)	0·3% (0·2–0·5%)	0·1 (0–0·1)	-59·5% (-73·7 to -38·7%)
	Subarachnoid haemorrhage	0·3% (0·2–0·5%)	0·3 (0·2–0·6)	-59·4% (-74·2 to -25·2%)	0·3% (0·2–0·5%)	<0·1 (<0·1–<0·1)	-62·8% (-78·2 to -25·6%)
Diet low in fruits	Stroke	2·4% (1·1–4%)	54 (24·6–94·5)	-67·7% (-81·5 to -46·2%)	2% (0·9–3·3%)	2·3 (1–4)	-65·6% (-80·6 to -44·4%)

Table S16 Regional and national disabilities and deaths related to neurological conditions attributable to risk factors in North Africa and Middle East countries

**Location**<br>Risk factor (alphabetic order)	Cause	**DALYs (95% UI)**<br>PAF (%)<br>2019	Rate per 100,000<br>2019	Rate change (%)<br>From 1990 to 2019	**Deaths (95% UI)**<br>PAF (%)<br>2019	Rate per 100,000<br>2019	Rate change (%)<br>From 1990 to 2019
	Ischaemic stroke	1·9% (0·4–3·7%)	30·2 (6·4–62·2)	-49·4% (-71·7 to -7·3%)	1·6% (0·4–3·2%)	1·4 (0·3–2·9)	-46·5% (-69·4 to -4·3%)
	Intracerebral haemorrhage	3·6% (1·6–6%)	19·9 (8·9–35·9)	-77·6% (-88·2 to -59·3%)	3·1% (1·4–5·2%)	0·7 (0·3–1·3)	-78·1% (-88·2 to -61·2%)
	Subarachnoid haemorrhage	3·9% (1·8–6·6%)	3·9 (1·7–7·2)	-78·8% (-89·4 to -56·3%)	3·2% (1·5–5·4%)	0·1 (0·1–0·2)	-79·9% (-90·3 to -57·9%)
Diet low in vegetables	Stroke	1·1% (0·3–2·3%)	24·9 (6–52·8)	-73·4% (-90·2 to -38·1%)	1·1% (0·3–2·1%)	1·3 (0·3–2·6)	-68% (-86·5 to -33%)
	Ischaemic stroke	1·1% (0·2–2·3%)	17·1 (3·3–37·1)	-49·4% (-79·4 to 10·9%)	1% (0·2–2·1%)	0·9 (0·2–1·9)	-42·3% (-74·4 to 15·1%)
	Intracerebral haemorrhage	1·2% (0·2–4%)	6·6 (1–22·5)	-86·7% (-96·3 to -63·4%)	1·2% (0·2–4%)	0·3 (0–1)	-85·1% (-95·4 to -61·4%)
	Subarachnoid haemorrhage	1·2% (0·2–4%)	1·2 (0·2–4·4)	-88·2% (-97·2 to -62·3%)	1·2% (0·2–4%)	<0·1 (<0·1–0·2)	-86·7% (-96·3 to -59·8%)
Diet low in whole grains	Stroke	6·7% (3·7–8·6%)	150·7 (80·8–207·1)	17·3% (-9·1 to 42·6%)	6·5% (3·9–8·1%)	7·5 (4·3–10·4)	16·4% (-9·8 to 41·3%)
	Ischaemic stroke	9·4% (5·2–12%)	150·7 (80·8–207·1)	17·3% (-9·1 to 42·6%)	8·5% (5–10·6%)	7·5 (4·3–10·4)	16·4% (-9·8 to 41·3%)
Dietary risks	Stroke	15·4% (11–20·8%)	347·1 (234·7–492·1)	-30·6% (-49·2 to -7·1%)	13·8% (10–18·5%)	16 (11–22·9)	-26·4% (-45·5 to -2·9%)
	Ischaemic stroke	16·8% (11·3–22·2%)	269·7 (171·4–374·2)	2·6% (-24·8 to 32·6%)	14·8% (10·3–19·4%)	13·1 (8·6–18·3)	2·9% (-23·4 to 31·8%)
	Intracerebral haemorrhage	11·8% (6·1–18·2%)	65·4 (33·1–106·4)	-66·8% (-80·6 to -46·8%)	10·5% (5·5–16·2%)	2·5 (1·3–4·2)	-67·5% (-80·4 to -48·7%)
	Subarachnoid haemorrhage	12% (6·3–18%)	12 (5·8–21·3)	-69·9% (-83·5 to -41·7%)	10·5% (5·6–16%)	0·4 (0·2–0·7)	-70·8% (-84 to -41·4%)
Environmental/occupational risk factors§	Meningitis	1·6% (0·7–3·1%)	1·1 (0·4–2·3)	-85·6% (-95·6 to -54·2%)	0·9% (0·4–1·9%)	<0·1 (<0·1–<0·1)	-85·6% (-95·6 to -54·2%)
	Encephalitis	0·9% (0·3–2·1%)	0·2 (0·1–0·5)	-7·1% (-70·9 to 190%)	0·6% (0·1–1·2%)	<0·1 (<0·1–<0·1)	-7·1% (-70·9 to 190%)
	Stroke	34% (29·5–38·5%)	767·3 (587·7–965·5)	-19·1% (-37·3 to 1·3%)	31·5% (27·2–35·9%)	36·6 (28·3–46·3)	-14·4% (-33·8 to 7·5%)
	Ischaemic stroke	32·9% (28·4–37·5%)	528·5 (410·2–669·8)	19% (-9·6 to 47·8%)	30·6% (26·4–34·9%)	27·2 (20·8–34·8)	18·9% (-9·4 to 46·8%)
	Intracerebral haemorrhage	36·5% (31·8–41·3%)	202·7 (148·8–264·8)	-51·7% (-65·1 to -33·1%)	34·3% (29·7–38·9%)	8·1 (6–10·4)	-51·7% (-64·9 to -33·9%)
	Subarachnoid haemorrhage	35·9% (31·2–40·9%)	36·1 (24·2–54·2)	-57·5% (-72·8 to -26·6%)	34% (29·3–38·8%)	1·3 (0·9–2)	-57·1% (-72·1 to -22%)
High body-mass index	Stroke	30·7% (20·7–40·9%)	695·1 (432·3–999·1)	5% (-22·1 to 48·4%)	20·5% (12·9–28·9%)	23·8 (13·8–35·8)	11·4% (-18·6 to 58·4%)
	Ischaemic stroke	25·3% (16·1–34·9%)	405·5 (243·2–598·9)	82·4% (32·1 to 154·1%)	16·7% (9·9–24·9%)	14·9 (8·2–23·3)	87·5% (31·2 to 170·7%)
	Intracerebral haemorrhage	44% (31·3–56·8%)	244·3 (156·4–350·8)	-32·5% (-53·2 to 2·9%)	32·6% (21·9–44·7%)	7·7 (4·6–11·4)	-31·6% (-53·5 to 4·2%)
	Subarachnoid haemorrhage	45·1% (31·4–57·8%)	45·3 (27·1–74·1)	-41·6% (-63·9 to 7·2%)	32·6% (20·6–45·2%)	1·3 (0·7–2·1)	-42% (-64·6 to 13·7%)
	Neurological disorders*	4·8% (1·4–10·9%)	64·3 (17·2–166·4)	67·2% (32·4 to 149·3%)	11·3% (3·8–21·7%)	4 (0·6–12·7)	70·8% (27·6 to 173·7%)
	Alzheimer's disease and other dementias	16·6% (6·9–29·7%)	64·3 (17·2–166·4)	67·2% (32·4 to 149·3%)	15·8% (6·6–28·5%)	4 (0·6–12·7)	70·8% (27·6 to 173·7%)
High fasting plasma glucose	Stroke	32% (19·6–51·7%)	720·2 (429·3–1202·1)	97·7% (40·1 to 173·2%)	32·8% (17·8–60%)	38 (20·3–69·3)	98·5% (36·6 to 176%)
	Ischaemic stroke	33·5% (16·7–60·8%)	535 (270–989·6)	167·7% (89·7 to 264·6%)	33·3% (15·6–68%)	29·5 (13·4–59·6)	158% (82·1 to 257·4%)
	Intracerebral haemorrhage	28·8% (17·2–41·5%)	159·5 (90·9–244)	15% (-21·6 to 69·3%)	31·2% (18·1–47·5%)	7·4 (4–11·5)	12·4% (-23·3 to 64·4%)
	Subarachnoid haemorrhage	25·6% (15·6–38%)	25·7 (13·6–41·8)	-0·2% (-37·9 to 87·8%)	29·8% (17·6–47%)	1·2 (0·6–2·1)	-0·8% (-40·4 to 97·6%)
	Neurological disorders*	3·8% (0·7–10·7%)	50·4 (8·8–159·9)	74·8% (54·5 to 105·9%)	9·3% (2–21·1%)	3·3 (0·4–12·4)	73·9% (46·8 to 113·9%)
	Alzheimer's disease and other dementias	13% (3·1–27%)	50·4 (8·8–159·9)	74·8% (54·5 to 105·9%)	13·1% (3·2–27·2%)	3·3 (0·4–12·4)	73·9% (46·8 to 113·9%)

Table S16 Regional and national disabilities and deaths related to neurological conditions attributable to risk factors in North Africa and Middle East countries

**Location**<br>Risk factor (alphabetic order)	Cause	**DALYs (95% UI)**<br>PAF (%)<br>2019	Rate per 100,000<br>2019	Rate change (%)<br>From 1990 to 2019	**Deaths (95% UI)**<br>PAF (%)<br>2019	Rate per 100,000<br>2019	Rate change (%)<br>From 1990 to 2019
High LDL cholesterol	Stroke	16·7% (9·8–28%)	378·2 (204·9–661·9)	27·1% (-1·6 to 60·2%)	15·2% (5·9–31·4%)	17·7 (6·6–38·5)	25·7% (-3·6 to 58%)
	Ischaemic stroke	23·5% (13·7–39·3%)	378·2 (204·9–661·9)	27·1% (-1·6 to 60·2%)	19·9% (7·7–40·7%)	17·7 (6·6–38·5)	25·7% (-3·6 to 58%)
High systolic blood pressure	Stroke	60·2% (51·3–68·3%)	1360·9 (1030·4–1725·8)	-19·2% (-36·9 to 1·7%)	56·5% (46·8–66·3%)	65·8 (49–86·2)	-16·1% (-34·9 to 6·5%)
	Ischaemic stroke	58·3% (47·3–68·3%)	935·3 (694·8–1203·5)	17·5% (-9·1 to 46·3%)	54·3% (42·4–66·7%)	48·3 (34·1–65·6)	16·6% (-10·5 to 47·3%)
	Intracerebral haemorrhage	65·3% (55–74·5%)	361·8 (259·5–481·5)	-51·3% (-65 to -32·3%)	63·6% (51·4–74·9%)	15 (10·7–20)	-51·9% (-64·7 to -33·4%)
	Subarachnoid haemorrhage	63·7% (53·6–73·4%)	63·8 (43·9–94·3)	-56·5% (-72·2 to -24·1%)	63·1% (50·8–74·9%)	2·5 (1·7–3·7)	-57% (-72·4 to -20·5%)
High temperature	Stroke	0·5% (0–1·3%)	11 (0·2–28·9)	-18% (-153·2 to 123%)	0·5% (0–1·4%)	0·6 (0–1·7)	-10·4% (-158·8 to 145·5%)
	Ischaemic stroke	0·5% (0–1·2%)	7·6 (0·1–20·2)	21·6% (-177·4 to 229·6%)	0·5% (0–1·4%)	0·5 (0–1·3)	21·2% (-175·3 to 229·7%)
	Intracerebral haemorrhage	0·5% (0–1·3%)	2·9 (0·1–7·5)	-51·2% (-131·2 to 39%)	0·5% (0–1·4%)	0·1 (0–0·3)	-50·4% (-131·8 to 45·1%)
	Subarachnoid haemorrhage	0·5% (0–1·3%)	0·5 (0–1·3)	-59·9% (-124·4 to 11·6%)	0·5% (0–1·4%)	<0·1 (<0·1–0·1)	-56% (-126 to 25·3%)
Household air pollution from solid fuels	Meningitis	0·1% (0–0·3%)	0·1 (0–0·2)	-98·4% (-99·6 to -94·5%)	0·1% (0–0·2%)	<0·1 (<0·1–<0·1)	-98·4% (-99·6 to -94·5%)
	Encephalitis	0·1% (0–0·2%)	<0·1 (<0·1–<0·1)	-89·8% (-97·5 to -63·5%)	0% (0–0·1%)	<0·1 (<0·1–<0·1)	-89·8% (-97·5 to -63·5%)
	Stroke	1·6% (0·7–3·1%)	35·6 (15·6–69·3)	-91·8% (-96·2 to -84·5%)	1·3% (0·6–2·6%)	1·6 (0·7–3·1)	-91·5% (-96 to -84·1%)
	Ischaemic stroke	1·5% (0·7–3%)	24·4 (10·7–46·7)	-87·9% (-94·2 to -76·9%)	1·3% (0·6–2·5%)	1·2 (0·5–2·3)	-88·2% (-94·3 to -77·4%)
	Intracerebral haemorrhage	1·7% (0·7–3·3%)	9·5 (4–18·6)	-95·1% (-97·7 to -90·3%)	1·5% (0·7–2·9%)	0·4 (0·1–0·7)	-95·2% (-97·7 to -90·5%)
	Subarachnoid haemorrhage	1·7% (0·8–3·4%)	1·7 (0·7–3·6)	-95·5% (-98·1 to -89·6%)	1·5% (0·7–3%)	0·1 (0–0·1)	-95·7% (-98·2 to -89·4%)
Kidney dysfunction	Stroke	11·6% (9–14%)	260·8 (185–334·2)	37·1% (3·5 to 72·3%)	10·7% (7·2–14·1%)	12·5 (7·7–17)	40·9% (2·9 to 81·9%)
	Ischaemic stroke	12·7% (9·4–15·8%)	203·9 (138·2–267·2)	83·5% (39·7 to 128·5%)	11% (6·5–15·2%)	9·8 (5·4–14·1)	82·4% (30·9 to 133·4%)
	Intracerebral haemorrhage	10·3% (8·3–12·5%)	56·9 (40·6–76·2)	-28% (-48·1 to -0·2%)	11·5% (8·6–14·2%)	2·7 (1·9–3·6)	-22·8% (-43 to 7·2%)
Lead exposure	Stroke	3·8% (1·9–6%)	86 (41·7–138·9)	-32·9% (-49·7 to -14·3%)	4% (2·2–6%)	4·7 (2·4–7·4)	-20·7% (-39·9 to 1·3%)
	Ischaemic stroke	3·8% (1·9–5·9%)	60·6 (29–98·1)	4·6% (-21·6 to 30%)	3·9% (2–6%)	3·4 (1·7–5·7)	16·2% (-12·2 to 43·5%)
	Intracerebral haemorrhage	3·9% (1·7–6·4%)	21·8 (9·1–37·8)	-63·1% (-74·6 to -48·2%)	4·4% (2·1–6·9%)	1 (0·5–1·7)	-57·8% (-69·4 to -41·9%)
	Subarachnoid haemorrhage	3·5% (1·4–6%)	3·5 (1·3–6·8)	-68% (-79·7 to -46·5%)	4·2% (2–6·9%)	0·2 (0·1–0·3)	-61·9% (-74·7 to -33·3%)
Low birth weight	Meningitis	9·1% (4·9–15·4%)	6·3 (2·9–11·9)	-87% (-94·9 to -68%)	5·2% (2·6–9·2%)	0·1 (0–0·1)	-87% (-94·9 to -68%)
	Encephalitis	5% (1·8–9·6%)	1·2 (0·6–2·2)	-20·7% (-66·1 to 78·7%)	3% (1–5·8%)	<0·1 (<0·1–<0·1)	-20·7% (-66·1 to 78·7%)
Low birth weight and short gestation	Meningitis	9·8% (5·3–16·6%)	6·8 (3·1–12·7)	-86·8% (-94·8 to -67·4%)	5·6% (2·8–9·9%)	0·1 (0–0·1)	-86·8% (-94·8 to -67·4%)
	Encephalitis	5·4% (2–10·4%)	1·3 (0·6–2·4)	-19·3% (-65·2 to 84·1%)	3·3% (1·1–6·2%)	<0·1 (<0·1–<0·1)	-19·3% (-65·3 to 84·1%)
Low physical activity	Stroke	5·6% (1·2–12·4%)	126·5 (25·2–277·9)	29·5% (2 to 60·6%)	7·1% (1·6–14·4%)	8·2 (1·8–16·7)	28·1% (-0·4 to 61·5%)
	Ischaemic stroke	7·9% (1·7–17·4%)	126·5 (25·2–277·9)	29·5% (2 to 60·6%)	9·3% (2·1–18·7%)	8·2 (1·8–16·7)	28·1% (-0·4 to 61·5%)
Low temperature	Stroke	6·4% (4·2–9%)	145·2 (90·2–218·3)	-19·6% (-37·7 to 0·6%)	7·3% (4·8–10·2%)	8·5 (5·4–12·9)	-12·2% (-29·5 to 8·6%)
	Ischaemic stroke	6·3% (4·1–8·8%)	100·6 (62·7–152·5)	19·2% (-9·3 to 46·8%)	7·3% (4·8–10·2%)	6·5 (4·1–9·9)	18·7% (-7·1 to 43·2%)
	Intracerebral haemorrhage	6·9% (4·5–9·6%)	38 (23·1–57)	-52·1% (-65·3 to -34·6%)	7·3% (4·8–10·2%)	1·7 (1·1–2·6)	-51·4% (-63·8 to -33·5%)
	Subarachnoid haemorrhage	6·6% (4·3–9·2%)	6·6 (3·6–11)	-60·7% (-74·1 to -31·8%)	7·3% (4·8–10·2%)	0·3 (0·2–0·5)	-56·9% (-71·6 to -22·2%)

Table S16 Regional and national disabilities and deaths related to neurological conditions attributable to risk factors in North Africa and Middle East countries

**Location**<br>Risk factor (alphabetic order)	Cause	**DALYs (95% UI)**<br>PAF (%)<br>2019	 Rate per 100,000<br>2019	 Rate change (%)<br>From 1990 to 2019	**Deaths (95% UI)**<br>PAF (%)<br>2019	 Rate per 100,000<br>2019	 Rate change (%)<br>From 1990 to 2019
Metabolic risk factors†	Stroke	79·2% (72·4–85·7%)	1788·4 (1418·9–2171·7)	-9·9% (-28·4 to 11·7%)	76% (66·6–85·9%)	88·5 (68·7–110·9)	-5·5% (-25 to 18·2%)
	Ischaemic stroke	79·3% (70·6–88·3%)	1272·2 (1005·6–1555·8)	28·2% (-0·4 to 57·7%)	75·5% (63·7–88·8%)	67·1 (50·4–84·8)	28·4% (-2·3 to 60·9%)
	Intracerebral haemorrhage	79·5% (72·2–85·3%)	440·7 (328·7–565·3)	-47% (-61·8 to -26·8%)	78·3% (69·9–85·8%)	18·5 (14–23·3)	-47·3% (-61·3 to -26·4%)
	Subarachnoid haemorrhage	75·3% (66·6–82·2%)	75·5 (53·1–109·9)	-53·1% (-69·7 to -18·6%)	74·7% (64·7–83·8%)	2·9 (2–4·3)	-53·4% (-70 to -14·3%)
	Neurological disorders*	8% (2·6–17·4%)	106·3 (30·9–279·1)	65% (37·8 to 115·9%)	19·2% (7·4–33·8%)	6·8 (1·1–21·8)	66·7% (32·6 to 126·2%)
	Alzheimer's disease and other dementias	27·5% (13·1–44·6%)	106·3 (30·9–279·1)	65% (37·8 to 115·9%)	26·8% (12·6–43·9%)	6·8 (1·1–21·8)	66·7% (32·6 to 126·2%)
Non-optimal temperature	Stroke	6·9% (4·8–9·3%)	155·4 (101·3–229·3)	-19·5% (-38·3 to 0·8%)	7·8% (5·4–10·5%)	9·1 (6–13·3)	-12·1% (-30·6 to 9·7%)
	Ischaemic stroke	6·7% (4·6–9%)	107·7 (70·1–158·7)	19·3% (-10·7 to 47·5%)	7·8% (5·4–10·5%)	6·9 (4·5–10·3)	18·8% (-7·1 to 44%)
	Intracerebral haemorrhage	7·3% (5·1–9·9%)	40·7 (25·9–59·4)	-52·1% (-65·6 to -34·4%)	7·8% (5·4–10·5%)	1·8 (1·2–2·7)	-51·3% (-63·9 to -33·5%)
	Subarachnoid haemorrhage	7% (4·8–9·5%)	7·1 (4·1–11·8)	-60·6% (-74·5 to -31·7%)	7·8% (5·4–10·5%)	0·3 (0·2–0·5)	-56·8% (-71·8 to -22%)
Other environmental risks	Stroke	3·8% (1·9–6%)	86 (41·7–138·9)	-32·9% (-49·7 to -14·3%)	4% (2·2–6%)	4·7 (2·4–7·4)	-20·7% (-39·9 to 1·3%)
	Ischaemic stroke	3·8% (1·9–5·9%)	60·6 (29–98·1)	4·6% (-21·6 to 30%)	3·9% (2–6%)	3·4 (1·7–5·7)	16·2% (-12·2 to 43·5%)
	Intracerebral haemorrhage	3·9% (1·7–6·4%)	21·8 (9·1–37·8)	-63·1% (-74·6 to -48·2%)	4·4% (2·1–6·9%)	1 (0·5–1·7)	-57·8% (-69·4 to -41·9%)
	Subarachnoid haemorrhage	3·5% (1·4–6%)	3·5 (1·3–6·8)	-68% (-79·7 to -46·5%)	4·2% (2–6·9%)	0·2 (0·1–0·3)	-61·9% (-74·7 to -33·3%)
Particulate matter pollution	Meningitis	1·6% (0·7–3·1%)	1·1 (0·4–2·3)	-85·6% (-95·6 to -54·2%)	0·9% (0·4–1·9%)	<0·1 (<0·1–<0·1)	-85·6% (-95·6 to -54·2%)
	Encephalitis	0·9% (0·3–2·1%)	0·2 (0·1–0·5)	-7·1% (-70·9 to 190%)	0·6% (0·1–1·2%)	<0·1 (<0·1–<0·1)	-7·1% (-70·9 to 190%)
	Stroke	26·3% (22–30·4%)	593·3 (444·8–767·1)	-17·8% (-37·4 to 4·6%)	22·6% (18·6–26·4%)	26·3 (19·7–33·9)	-14·6% (-35 to 8·1%)
	Ischaemic stroke	25·3% (21·1–29·4%)	405·3 (307·6–519·3)	20·7% (-8·7 to 51·1%)	21·7% (17·8–25·4%)	19·3 (14·4–25·1)	19·1% (-11·4 to 50·6%)
	Intracerebral haemorrhage	28·7% (24–33·1%)	159·3 (114·3–213·7)	-50·4% (-64·6 to -30·2%)	25·5% (21·2–29·6%)	6 (4·4–7·9)	-51·1% (-64·9 to -31·8%)
	Subarachnoid haemorrhage	28·5% (23·8–33·2%)	28·7 (18·9–42·7)	-55·7% (-72·1 to -22·6%)	25·3% (20·7–29·9%)	1 (0·6–1·5)	-56·7% (-72·5 to -20·3%)
Secondhand smoke	Stroke	3·4% (2·5–4·3%)	76·1 (52·3–103·5)	-34·1% (-50·5 to -14·7%)	2·8% (2·1–3·6%)	3·3 (2·2–4·4)	-31·6% (-47·8 to -13·8%)
	Ischaemic stroke	3·1% (2·3–4%)	49·8 (34·2–68·4)	0·3% (-25 to 28%)	2·6% (1·9–3·4%)	2·3 (1·6–3·2)	-2·8% (-27·1 to 21·3%)
	Intracerebral haemorrhage	4% (2·9–5·2%)	22·2 (14·4–31·2)	-59·1% (-71 to -40·8%)	3·4% (2·5–4·3%)	0·8 (0·5–1·1)	-59·8% (-71·5 to -42·9%)
	Subarachnoid haemorrhage	4·1% (2·9–5·4%)	4·1 (2·4–6·6)	-64·6% (-78·4 to -37·2%)	3·4% (2·5–4·6%)	0·1 (0·1–0·2)	-65·1% (-78·2 to -36·5%)
Short gestation	Meningitis	6·9% (3·7–11·6%)	4·8 (2·2–8·8)	-85·1% (-94 to -64%)	4% (2–6·9%)	0·1 (0–0·1)	-85·1% (-94 to -64%)
	Encephalitis	3·9% (1·4–7·6%)	0·9 (0·4–1·8)	-10·5% (-62·2 to 104·6%)	2·4% (0·8–4·5%)	<0·1 (<0·1–<0·1)	-10·5% (-62·2 to 104·6%)
Smoking	Stroke	8·5% (7·3–9·8%)	192·6 (143·6–248·6)	-41% (-56·8 to -22·1%)	6·3% (5·3–7·3%)	7·3 (5·4–9·5)	-39·4% (-55·9 to -20·2%)
	Ischaemic stroke	7·7% (6·6–8·9%)	124·1 (91·7–159·3)	-10·6% (-36·3 to 19·9%)	5·6% (4·7–6·6%)	5 (3·7–6·6)	-10·9% (-37·2 to 20·9%)
	Intracerebral haemorrhage	10·6% (9–12·5%)	59·1 (40·9–80·1)	-63·2% (-74·9 to -47·4%)	8·6% (7·1–10·1%)	2 (1·4–2·7)	-63·7% (-75·1 to -48·9%)
	Subarachnoid haemorrhage	9·2% (7·2–11·7%)	9·4 (5·6–15·9)	-65·7% (-78·6 to -37·3%)	7·6% (5·5–9·9%)	0·3 (0·2–0·5)	-66·6% (-79·2 to -37%)
	Neurological disorders*	2·4% (0·8–5·3%)	32·6 (9·6–84·6)	-26·7% (-43·5 to -11·5%)	5·1% (1·2–9%)	1·9 (0·2–6·1)	-22·6% (-49·5 to -2·5%)

Table S16 Regional and national disabilities and deaths related to neurological conditions attributable to risk factors in North Africa and Middle East countries

Location<br>Risk factor (alphabetic order)	Cause	DALYs (95% UI)<br>PAF (%)<br>2019	Rate per 100,000<br>2019	Rate change (%)<br>From 1990 to 2019	Deaths (95% UI)<br>PAF (%)<br>2019	Rate per 100,000<br>2019	Rate change (%)<br>From 1990 to 2019
	Alzheimer's disease and other dementias	9·4% (5·6–13·4%)	36·2 (13·3–88·2)	-25·1% (-40·2 to -9·7%)	8·4% (4·9–12·3%)	2·1 (0·5–6·4)	-20·5% (-40·6 to 0·5%)
	Parkinson's disease	-5·2% (-8–-2·6%)	-5 (-7·8–-2·4)	-7·9% (-33·3 to 24·8%)	-4·6% (-7·2–-2·2%)	-0·3 (-0·4–-0·1)	-1·8% (-30·7 to 36·2%)
	Multiple sclerosis	6·8% (4·9–8·8%)	1·4 (0·9–1·9)	-9·9% (-36·8 to 23·1%)	7·1% (4·8–9·8%)	<0·1 (<0·1–<0·1)	-9·7% (-48·2 to 38·9%)
Tobacco	Stroke	11·6% (10·2–13·1%)	262 (197·1–333·2)	-38·8% (-54·5 to -20·8%)	8·9% (7·7–10·2%)	10·4 (7·7–13·1)	-36·9% (-53 to -19·3%)
	Ischaemic stroke	10·6% (9·3–12%)	169·9 (129·2–212·9)	-7·3% (-31·9 to 20·9%)	8·1% (6·9–9·3%)	7·2 (5·4–9·3)	-8·1% (-34·6 to 19%)
	Intracerebral haemorrhage	14·2% (12·4–16·4%)	79 (56·4–105·4)	-61·9% (-73·8 to -46·7%)	11·7% (10–13·6%)	2·8 (2–3·6)	-62·5% (-73·8 to -47·9%)
	Subarachnoid haemorrhage	12·9% (10·8–15·3%)	13·1 (8·3–20·7)	-65·2% (-77·8 to -38%)	10·8% (8·6–13·1%)	0·4 (0·3–0·7)	-65·9% (-78·4 to -37·8%)
	Neurological disorders*	2·4% (0·8–5·3%)	32·6 (9·6–84·6)	-26·7% (-43·5 to -11·5%)	5·1% (1·2–9%)	1·9 (0·2–6·1)	-22·6% (-49·5 to -2·5%)
	Alzheimer's disease and other dementias	9·4% (5·6–13·4%)	36·2 (13·3–88·2)	-25·1% (-40·2 to -9·7%)	8·4% (4·9–12·3%)	2·1 (0·5–6·4)	-20·5% (-40·6 to 0·5%)
	Parkinson's disease	-5·2% (-8–-2·6%)	-5 (-7·8–-2·4)	-7·9% (-33·3 to 24·8%)	-4·6% (-7·2–-2·2%)	-0·3 (-0·4–-0·1)	-1·8% (-30·7 to 36·2%)
	Multiple sclerosis	6·8% (4·9–8·8%)	1·4 (0·9–1·9)	-9·9% (-36·8 to 23·1%)	7·1% (4·8–9·8%)	<0·1 (<0·1–<0·1)	-9·7% (-48·2 to 38·9%)
**Palestine**							
Air pollution	Meningitis	0·6% (0·2–1·1%)	0·2 (0·1–0·5)	-88·4% (-96 to -66%)	0·3% (0·1–0·6%)	<0·1 (<0·1–<0·1)	-88·4% (-96 to -66%)
	Encephalitis	0·3% (0·1–0·6%)	<0·1 (<0·1–0·1)	-34·1% (-83·5 to 126·1%)	0·2% (0·1–0·4%)	<0·1 (<0·1–<0·1)	-34·1% (-83·5 to 126·2%)
	Stroke	22·8% (17·5–27·8%)	485·7 (356·7–612·5)	-39·6% (-54·1 to -22·2%)	19·6% (14·9–24%)	24 (17·5–30·6)	-38·2% (-52·6 to -20·4%)
	Ischaemic stroke	22% (16·8–26·8%)	350·3 (258–442·5)	-26·1% (-43·3 to -6·5%)	19% (14·5–23·3%)	18·3 (13·2–23·4)	-25·8% (-43·2 to -4·7%)
	Intracerebral haemorrhage	25% (19·4–30·2%)	122 (88–155·9)	-59·8% (-70·2 to -45·9%)	21·9% (16·7–26·8%)	5·3 (3·8–6·8)	-60·3% (-70·2 to -46·9%)
	Subarachnoid haemorrhage	26·7% (21–32·2%)	13·4 (9·8–17·3)	-49% (-64·2 to -25·8%)	23% (17·8–28%)	0·4 (0·3–0·6)	-54·3% (-69·6 to -28·8%)
Alcohol use	Stroke	1·1% (0·7–1·5%)	22·8 (14·3–32·3)	-13·5% (-48·6 to 88·6%)	0·7% (0·4–1·1%)	0·9 (0·5–1·3)	-12·9% (-51·1 to 112·3%)
	Ischaemic stroke	0·5% (0·2–0·9%)	8·7 (3·2–15·1)	42·8% (-40·3 to 532%)	0·4% (0·1–0·7%)	0·4 (0·1–0·7)	45·1% (-532·5 to 873·2%)
	Intracerebral haemorrhage	2·9% (2–4·1%)	14·1 (9·1–21·3)	-30·4% (-56·4 to 32·7%)	2·2% (1·5–3·1%)	0·5 (0·3–0·8)	-32% (-57·8 to 36·2%)
	Neurological disorders*	0·5% (0·2–1%)	6·6 (3·5–11·2)	17·3% (-39·2 to 139·2%)	0·3% (0·1–0·6%)	0·1 (0–0·1)	14·2% (-26·1 to 95·1%)
	Idiopathic epilepsy	4·1% (2·6–6%)	6·6 (3·5–11·2)	17·3% (-39·2 to 139·2%)	4·6% (2·9–6·7%)	0·1 (0–0·1)	14·2% (-26·1 to 95·1%)
All risk factors	Meningitis	4% (2·6–5·9%)	1·6 (1–2·5)	-87·7% (-93·8 to -75·9%)	2% (1·3–3%)	<0·1 (<0·1–<0·1)	-87·7% (-93·8 to -75·9%)
	Encephalitis	2% (1·1–3·3%)	0·3 (0·2–0·5)	-29·7% (-70·3 to 56·6%)	1·3% (0·7–2·1%)	<0·1 (<0·1–<0·1)	-29·7% (-70·3 to 56·6%)
	Stroke	85·8% (81·3–91%)	1825·8 (1582·8–2103·3)	-31·4% (-45·6 to -13·5%)	83·1% (76·9–90·2%)	101·7 (86·2–119·4)	-28·3% (-43·1 to -8·1%)
	Ischaemic stroke	85·9% (80·3–92·7%)	1366·3 (1171·8–1577·9)	-16·8% (-33·6 to 4·7%)	83% (75·7–92·3%)	80·1 (66·2–95·1)	-14·8% (-32·5 to 8·7%)
	Intracerebral haemorrhage	85·8% (81·4–89·8%)	418·2 (354·1–493·4)	-55·6% (-66·1 to -41·5%)	83·5% (77·7–89·2%)	20·1 (16·8–23·9)	-55·1% (-65·6 to -41·3%)
	Subarachnoid haemorrhage	82·5% (77·4–86·5%)	41·3 (34·3–49·4)	-45% (-61·1 to -20%)	81% (74·3–87%)	1·5 (1·1–1·8)	-49·2% (-65·7 to -21·9%)
	Neurological disorders*	10·8% (4·1–21·9%)	146·9 (53·9–374·1)	16·2% (-6·5 to 45·9%)	22·9% (9–38·1%)	8·7 (1·5–27·8)	15·2% (-10·5 to 60·8%)

Table S16 Regional and national disabilities and deaths related to neurological conditions attributable to risk factors in North Africa and Middle East countries

Location<br>Risk factor (alphabetic order)	Cause	DALYs (95% UI)<br>PAF (%)<br>2019	Rate per 100,000<br>2019	Rate change (%)<br>From 1990 to 2019	Deaths (95% UI)<br>PAF (%)<br>2019	Rate per 100,000<br>2019	Rate change (%)<br>From 1990 to 2019
	Alzheimer's disease and other dementias	38% (23·8–53·4%)	147·9 (55–374·5)	12·7% (-9·4 to 42%)	35·2% (21·1–51%)	9·1 (1·9–28·3)	11·9% (-13·5 to 49·3%)
	Parkinson's disease	-10·9% (-16·1–-5·7%)	-10·1 (-15·8–-4·8)	-21·6% (-45·4 to 21·8%)	-9·2% (-13·9–-4·7%)	-0·6 (-0·9–-0·3)	-22·3% (-47·5 to 25·7%)
	Idiopathic epilepsy	4·1% (2·6–6%)	6·6 (3·5–11·2)	17·3% (-39·2 to 139·2%)	4·6% (2·9–6·7%)	0·1 (0–0·1)	14·2% (-26·1 to 95·1%)
	Multiple sclerosis	13% (9·6–17·7%)	2·5 (1·6–3·8)	2·9% (-34·2 to 43%)	14·7% (10·7–21%)	<0·1 (<0·1–0·1)	-6·6% (-51 to 56%)
Ambient particulate matter pollution	Meningitis	0·6% (0·2–1·1%)	0·2 (0·1–0·4)	-72·8% (-91·2 to -1·3%)	0·3% (0·1–0·5%)	<0·1 (<0·1–<0·1)	-72·9% (-91·2 to -1·3%)
	Encephalitis	0·3% (0·1–0·6%)	<0·1 (<0·1–0·1)	55·3% (-61·5 to 602·2%)	0·2% (0·1–0·4%)	<0·1 (<0·1–<0·1)	55·3% (-61·5 to 602·5%)
	Stroke	22·5% (17·2–27·2%)	477·9 (349–603·3)	27·7% (-18·1 to 142·3%)	19·3% (14·5–23·6%)	23·6 (17·2–30·1)	30·7% (-16·6 to 145·4%)
	Ischaemic stroke	21·7% (16·6–26·5%)	344·6 (251·1–434·4)	56·7% (2·1 to 197·6%)	18·7% (14·1–22·9%)	18 (13–23·1)	57·4% (-0·9 to 195·6%)
	Intracerebral haemorrhage	24·6% (18·8–29·8%)	120·1 (86·9–154·1)	-15·6% (-47·8 to 61·1%)	21·5% (16·4–26·3%)	5·2 (3·7–6·7)	-16·5% (-47·3 to 61%)
	Subarachnoid haemorrhage	26·3% (20·5–31·7%)	13·2 (9·6–17)	10·2% (-33·1 to 120·8%)	22·6% (17·3–27·6%)	0·4 (0·3–0·5)	-1·2% (-42·8 to 107·4%)
Behavioural risk factors‡	Meningitis	3·9% (2·5–5·7%)	1·6 (1–2·4)	-87·6% (-93·8 to -75·6%)	2% (1·2–2·9%)	<0·1 (<0·1–<0·1)	-87·6% (-93·8 to -75·6%)
	Encephalitis	1·9% (1·1–3·2%)	0·3 (0·2–0·5)	-29·3% (-69·2 to 56·7%)	1·2% (0·7–2%)	<0·1 (<0·1–<0·1)	-29·3% (-69·2 to 56·7%)
	Stroke	38·1% (32·8–43·7%)	810·7 (655·4–989)	-37·4% (-51·3 to -20%)	34·5% (28·8–40·8%)	42·3 (33·2–52·5)	-34·6% (-49·1 to -17%)
	Ischaemic stroke	38·5% (32·2–45·5%)	612·3 (483·4–763·2)	-23·3% (-39·7 to -2·5%)	35·2% (28·5–42·8%)	33·9 (25·8–43·2)	-21·6% (-38·5 to 0%)
	Intracerebral haemorrhage	37% (31·3–43%)	180·7 (140·6–228·7)	-60·8% (-70·8 to -46·7%)	32·2% (26·5–37·8%)	7·8 (6–9·9)	-61·3% (-71·3 to -47·9%)
	Subarachnoid haemorrhage	35·5% (29·6–41·9%)	17·8 (13·6–22·3)	-51·5% (-66·5 to -27·7%)	30·7% (25–37%)	0·6 (0·4–0·7)	-56·3% (-71·6 to -30·8%)
	Neurological disorders*	4·4% (1·7–8·9%)	59·6 (21·8–146·8)	-12·1% (-29·7 to 14·2%)	7·7% (1·6–13·6%)	3 (0·3–9·4)	-20·2% (-44 to 15·7%)
	Alzheimer's disease and other dementias	15·6% (10·1–20·9%)	60·6 (23·4–143·4)	-16·6% (-32·9 to 7%)	13·3% (8·3–18·1%)	3·5 (0·7–9·9)	-21·2% (-40·1 to 8%)
	Parkinson's disease	-10·9% (-16·1–-5·7%)	-10·1 (-15·8–-4·8)	-21·6% (-45·4 to 21·8%)	-9·2% (-13·9–-4·7%)	-0·6 (-0·9–-0·3)	-22·3% (-47·5 to 25·7%)
	Idiopathic epilepsy	4·1% (2·6–6%)	6·6 (3·5–11·2)	17·3% (-39·2 to 139·2%)	4·6% (2·9–6·7%)	0·1 (0–0·1)	14·2% (-26·1 to 95·1%)
	Multiple sclerosis	13% (9·6–17·7%)	2·5 (1·6–3·8)	2·9% (-34·2 to 43%)	14·7% (10·7–21%)	<0·1 (<0·1–0·1)	-6·6% (-51 to 56%)
Child and maternal malnutrition	Meningitis	3·9% (2·5–5·7%)	1·6 (1–2·4)	-87·6% (-93·8 to -75·6%)	2% (1·2–2·9%)	<0·1 (<0·1–<0·1)	-87·6% (-93·8 to -75·6%)
	Encephalitis	1·9% (1·1–3·2%)	0·3 (0·2–0·5)	-29·3% (-69·2 to 56·7%)	1·2% (0·7–2%)	<0·1 (<0·1–<0·1)	-29·3% (-69·2 to 56·7%)
Diet high in red meat	Stroke	2·1% (0·7–3·4%)	44·2 (14·1–74·3)	-43·2% (-60·5 to -24%)	1·7% (0·5–2·7%)	2 (0·7–3·4)	-40·5% (-58·4 to -19·9%)
	Ischaemic stroke	1·9% (0·3–3·1%)	29·7 (4·5–51·5)	-28·4% (-44·9 to -7%)	1·5% (0·3–2·5%)	1·5 (0·3–2·5)	-25·4% (-44·1 to -3·3%)
	Intracerebral haemorrhage	2·7% (0·3–4·6%)	13 (1·5–23·6)	-61% (-71 to -47·3%)	2·3% (0·3–3·9%)	0·5 (0·1–1)	-60·7% (-70·7 to -46·9%)
	Subarachnoid haemorrhage	3% (0·3–5·2%)	1·5 (0·2–2·8)	-50·6% (-65·6 to -29%)	2·4% (0·3–4·1%)	<0·1 (<0·1–0·1)	-55·4% (-70·4 to -30·4%)
Diet high in sodium	Stroke	1·7% (0·3–6·8%)	37·1 (5·5–149·7)	-34·6% (-62 to 19·6%)	1·4% (0·3–5·7%)	1·8 (0·3–6·9)	-33·9% (-60·5 to 24·7%)
	Ischaemic stroke	1·6% (0·3–6·3%)	25·7 (4–103·2)	-18·8% (-54·2 to 51·1%)	1·3% (0·3–5·3%)	1·3 (0·2–5·1)	-19% (-52·3 to 52·9%)
	Intracerebral haemorrhage	2·1% (0·3–8·4%)	10·3 (1·2–42·9)	-55·5% (-75·7 to -17·5%)	1·8% (0·3–7·4%)	0·4 (0·1–1·8)	-56·4% (-75·9 to -17·7%)
	Subarachnoid haemorrhage	2% (0·3–8%)	1 (0·1–4)	-42·6% (-70·7 to 7·5%)	1·8% (0·3–7·1%)	<0·1 (<0·1–0·1)	-48·7% (-74·7 to -0·1%)

Table S16 Regional and national disabilities and deaths related to neurological conditions attributable to risk factors in North Africa and Middle East countries

Location<br>Risk factor (alphabetic order)	Cause	DALYs (95% UI)<br>PAF (%)<br>2019	Rate per 100,000<br>2019	Rate change (%)<br>From 1990 to 2019	Deaths (95% UI)<br>PAF (%)<br>2019	Rate per 100,000<br>2019	Rate change (%)<br>From 1990 to 2019
Diet low in fibre	Stroke	5·5% (1·6–9·2%)	118·1 (32·6–199·2)	-31·8% (-47·5 to -9·6%)	4·8% (1·4–7·9%)	5·9 (1·7–9·8)	-29·4% (-45·4 to -7·6%)
	Ischaemic stroke	5·3% (1·5–8·7%)	83·7 (23·7–141)	-16·2% (-35·8 to 9·7%)	4·6% (1·3–7·5%)	4·4 (1·3–7·3)	-14·3% (-34·1 to 12·8%)
	Intracerebral haemorrhage	6·3% (1·7–10·5%)	30·8 (8·2–52·6)	-54·1% (-66·1 to -37·5%)	5·7% (1·6–9·5%)	1·4 (0·4–2·3)	-54% (-65·8 to -37·3%)
	Subarachnoid haemorrhage	7% (2–11·6%)	3·5 (0·9–5·9)	-42% (-59·1 to -15·9%)	6% (1·7–10%)	0·1 (0–0·2)	-47·8% (-65·8 to -18·5%)
Diet low in fruits	Stroke	4·8% (2–7·8%)	101·8 (43·5–170·4)	-44·5% (-62 to -25·1%)	4% (1·6–6·6%)	4·9 (2–8·3)	-42·8% (-61·3 to -22·3%)
	Ischaemic stroke	4% (0·9–7·6%)	62·8 (15·3–124·1)	-28% (-49·8 to -6·2%)	3·3% (0·8–6·4%)	3·2 (0·8–6·2)	-26·4% (-50·4 to -1·7%)
	Intracerebral haemorrhage	7·2% (3·3–13·1%)	35 (15·9–67·8)	-60·4% (-72·4 to -45·5%)	6·3% (3–11·6%)	1·5 (0·7–3)	-60·5% (-72·2 to -45·5%)
	Subarachnoid haemorrhage	8% (3·8–14·6%)	4 (1·8–7·5)	-49·4% (-65 to -25·9%)	6·7% (3·2–12·4%)	0·1 (0·1–0·2)	-54·9% (-71·7 to -28·5%)
Diet low in vegetables	Stroke	1·8% (0·5–3·3%)	38·3 (11·1–69·8)	-78·2% (-91·2 to -58·8%)	1·9% (0·7–3·2%)	2·3 (0·8–4)	-71·7% (-86·1 to -51·7%)
	Ischaemic stroke	1·7% (0·3–3·2%)	26·5 (4·3–52·4)	-64·9% (-87·4 to -24·5%)	1·7% (0·3–3·2%)	1·7 (0·3–3·1)	-56·6% (-79·8 to -21·1%)
	Intracerebral haemorrhage	2·2% (0·3–6%)	10·6 (1·3–29·4)	-88·4% (-96·9 to -68%)	2·5% (0·4–5·9%)	0·6 (0·1–1·4)	-85% (-94·3 to -66·9%)
	Subarachnoid haemorrhage	2·2% (0·2–6·4%)	1·1 (0·1–3·2)	-86·7% (-97·2 to -58·3%)	2·4% (0·3–5·9%)	<0·1 (<0·1–0·1)	-84·8% (-95·2 to -60%)
Diet low in whole grains	Stroke	6·3% (3·4–8·1%)	134 (73·2–181·9)	-23·1% (-39·4 to -3·2%)	6·1% (3·5–7·7%)	7·4 (4·3–10·1)	-21·2% (-38·1 to 0·9%)
	Ischaemic stroke	8·4% (4·6–10·8%)	134 (73·2–181·9)	-23·1% (-39·4 to -3·2%)	7·7% (4·5–9·7%)	7·4 (4·3–10·1)	-21·2% (-38·1 to 0·9%)
Dietary risks	Stroke	20·2% (15–25·8%)	430·4 (311·8–565·6)	-42·5% (-55·2 to -25·6%)	18·4% (13·6–23·2%)	22·5 (16·1–29·6)	-39% (-52·8 to -20·7%)
	Ischaemic stroke	20·6% (14·9–26·4%)	328·5 (229·8–439)	-27·5% (-42·8 to -7·2%)	18·6% (13·5–23·6%)	18 (12·6–23·9)	-24·9% (-41·4 to -2·9%)
	Intracerebral haemorrhage	18·8% (12·2–26·2%)	91·8 (56·9–134·4)	-66·1% (-76·5 to -52·1%)	17·4% (11·3–24·2%)	4·2 (2·6–6·1)	-65·3% (-75·2 to -52%)
	Subarachnoid haemorrhage	20·2% (13·2–28%)	10·1 (6·3–14·4)	-57·2% (-71·4 to -34·5%)	17·8% (11·6–24·9%)	0·3 (0·2–0·5)	-60·9% (-75·5 to -36·9%)
Environmental/occupational risk factors§	Meningitis	0·6% (0·2–1·1%)	0·2 (0·1–0·5)	-88·4% (-96 to -66%)	0·3% (0·1–0·6%)	<0·1 (<0·1–<0·1)	-88·4% (-96 to -66%)
	Encephalitis	0·3% (0·1–0·6%)	<0·1 (<0·1–0·1)	-34·1% (-83·5 to 126·1%)	0·2% (0·1–0·4%)	<0·1 (<0·1–<0·1)	-34·1% (-83·5 to 126·2%)
	Stroke	29·7% (23·7–35·4%)	631·9 (486·1–785)	-39·5% (-53·3 to -22·3%)	27·4% (21·8–33%)	33·6 (25·5–42)	-36·7% (-50·6 to -18·7%)
	Ischaemic stroke	28·9% (23–34·5%)	459·4 (352·2–573)	-25·8% (-42·2 to -5·6%)	26·8% (21·2–32·4%)	25·8 (19·4–32·5)	-23·9% (-41 to -3%)
	Intracerebral haemorrhage	32·1% (25·7–38·1%)	156·3 (118·5–197)	-60·3% (-70 to -46·9%)	29·8% (23·8–35·9%)	7·2 (5·5–9·2)	-59·8% (-69·3 to -46·8%)
	Subarachnoid haemorrhage	32·5% (26·1–38·1%)	16·3 (12·2–21·1)	-50·4% (-65·5 to -27·1%)	30·5% (24·3–36·5%)	0·6 (0·4–0·7)	-54% (-69·2 to -28·8%)
High body-mass index	Stroke	25·5% (16·9–34·5%)	542·5 (354·3–758·9)	-21·3% (-40·5 to 7%)	16·2% (9·9–23·4%)	19·8 (11·9–29·8)	-18·9% (-40 to 14·7%)
	Ischaemic stroke	21% (13·3–29·3%)	333·6 (207·8–482·4)	4·3% (-20·7 to 42%)	13·3% (7·8–20·1%)	12·9 (7·3–20·4)	8·1% (-20·5 to 58·8%)
	Intracerebral haemorrhage	38·1% (26·5–49·7%)	185·8 (124·5–256·6)	-44·6% (-59·5 to -21·9%)	26·4% (17·3–36·9%)	6·4 (4–9·3)	-45·1% (-60 to -20·7%)
	Subarachnoid haemorrhage	46% (34–57·6%)	23 (16·1–30·7)	-31·7% (-51·4 to 2·6%)	30·9% (20·9–42·5%)	0·6 (0·4–0·8)	-38·9% (-59·3 to -1·5%)
	Neurological disorders*	3·8% (1–9·3%)	51·9 (12·9–136·8)	39·7% (8·1 to 122·5%)	8·6% (2·4–18·1%)	3·2 (0·5–9·7)	42·4% (3·7 to 159·6%)
	Alzheimer's disease and other dementias	13·4% (4·6–25·9%)	51·9 (12·9–136·8)	39·7% (8·1 to 122·5%)	12·3% (3·9–24·3%)	3·2 (0·5–9·7)	42·4% (3·7 to 159·6%)
High fasting plasma glucose	Stroke	34·4% (20·4–57·2%)	733·5 (439·8–1237·8)	-3·8% (-30·7 to 35·6%)	33·5% (18·3–63·2%)	41 (22·2–78·8)	-3·1% (-30·8 to 37·4%)
	Ischaemic stroke	35·1% (17·4–66%)	559·1 (277·8–1051·1)	13·8% (-15·5 to 59·9%)	33·7% (15·6–71·1%)	32·5 (14·7–69·3)	13·9% (-17·1 to 59·6%)

Table S16 Regional and national disabilities and deaths related to neurological conditions attributable to risk factors in North Africa and Middle East countries

Location<br>Risk factor (alphabetic order)	Cause	DALYs (95% UI)<br>PAF (%)<br>2019	Rate per 100,000<br>2019	Rate change (%)<br>From 1990 to 2019	Deaths (95% UI)<br>PAF (%)<br>2019	Rate per 100,000<br>2019	Rate change (%)<br>From 1990 to 2019
	Intracerebral haemorrhage	32·9% (19·9–47·9%)	160·7 (93·5–243·2)	-36·6% (-55·9 to -6·9%)	33·1% (19·2–52·7%)	8 (4·4–12·8)	-38·8% (-57·7 to -11·4%)
	Subarachnoid haemorrhage	27·4% (16·9–39·5%)	13·7 (8·3–20·8)	-20·7% (-48·5 to 30·4%)	31·8% (18·8–49·8%)	0·6 (0·3–0·9)	-28·2% (-54·9 to 22·4%)
	Neurological disorders*	4·4% (0·8–12·1%)	59·6 (10·7–192·1)	60% (35·4 to 100·6%)	10·3% (2·2–23·8%)	3·9 (0·4–14·6)	57·7% (28·8 to 106·7%)
	Alzheimer's disease and other dementias	15·3% (3·8–31%)	59·6 (10·7–192·1)	60% (35·4 to 100·6%)	14·8% (3·6–30·4%)	3·9 (0·4–14·6)	57·7% (28·8 to 106·7%)
High LDL cholesterol	Stroke	16·4% (8·5–30%)	350·1 (174·7–627·8)	-17·1% (-34·8 to 4·3%)	15·3% (5–32·6%)	18·7 (6–39·9)	-14·2% (-34·4 to 9·4%)
	Ischaemic stroke	22% (11·4–40%)	350·1 (174·7–627·8)	-17·1% (-34·8 to 4·3%)	19·4% (6·4–41·1%)	18·7 (6–39·9)	-14·2% (-34·4 to 9·4%)
High systolic blood pressure	Stroke	46% (36·9–54·7%)	979·3 (759·6–1210·4)	-33·3% (-48·8 to -13·4%)	42·4% (32·8–52·9%)	51·9 (38·9–67·1)	-30·4% (-48·5 to -8·6%)
	Ischaemic stroke	44·2% (33·7–54·7%)	702·6 (515·7–894)	-17·4% (-36·7 to 7·1%)	40·6% (29·5–52·9%)	39·2 (27·3–53·4)	-15·4% (-37·3 to 12·7%)
	Intracerebral haemorrhage	51·6% (41·3–61·7%)	251·4 (191·6–318)	-56% (-67·1 to -41·3%)	49·2% (37·3–61·1%)	11·9 (8·4–15·6)	-55·3% (-66·8 to -40%)
	Subarachnoid haemorrhage	50·5% (40·6–59·7%)	25·3 (18·9–32·8)	-45·5% (-62·6 to -19·8%)	49·3% (38·1–60·9%)	0·9 (0·6–1·2)	-49·4% (-66·6 to -20·2%)
High temperature	Stroke	0·4% (-0·3–2·1%)	8·7 (-5·3–44·9)	8·1% (-649·3 to 627·1%)	0·5% (-0·3–2·4%)	0·6 (-0·3–2·9)	17·7% (-700·7 to 681·5%)
	Ischaemic stroke	0·4% (-0·3–2·1%)	6·4 (-3·9–33·5)	34% (-767·3 to 805·1%)	0·5% (-0·3–2·4%)	0·4 (-0·3–2·3)	40·2% (-809 to 829·6%)
	Intracerebral haemorrhage	0·4% (-0·3–2·3%)	2·1 (-1·3–10·4)	-30·5% (-458·5 to 363·2%)	0·5% (-0·3–2·4%)	0·1 (-0·1–0·5)	-27·1% (-465·7 to 375·2%)
	Subarachnoid haemorrhage	0·4% (-0·2–1·9%)	0·2 (-0·1–1)	-17% (-495·6 to 385·2%)	0·5% (-0·3–2·4%)	<0·1 (<0·1–<0·1)	-14·3% (-506·5 to 424·6%)
Household air pollution from solid fuels	Meningitis	0% (0–0%)	<0·1 (<0·1–<0·1)	-99·6% (-99·9 to -98·7%)	0% (0–0%)	<0·1 (<0·1–<0·1)	-99·6% (-99·9 to -98·7%)
	Encephalitis	0% (0–0%)	<0·1 (<0·1–<0·1)	-97·8% (-99·5 to -90·6%)	0% (0–0%)	<0·1 (<0·1–<0·1)	-97·8% (-99·5 to -90·6%)
	Stroke	0·4% (0·2–0·7%)	7·8 (3·5–15·6)	-98·2% (-99·2 to -96·1%)	0·3% (0·1–0·6%)	0·4 (0·2–0·8)	-98·1% (-99·2 to -96%)
	Ischaemic stroke	0·4% (0·2–0·7%)	5·7 (2·6–11·5)	-97·8% (-99 to -95·3%)	0·3% (0·1–0·6%)	0·3 (0·1–0·6)	-97·7% (-99 to -95·2%)
	Intracerebral haemorrhage	0·4% (0·2–0·8%)	1·9 (0·9–3·8)	-98·8% (-99·5 to -97·3%)	0·3% (0·2–0·7%)	0·1 (0–0·2)	-98·8% (-99·5 to -97·3%)
	Subarachnoid haemorrhage	0·4% (0·2–0·9%)	0·2 (0·1–0·4)	-98·5% (-99·4 to -96·4%)	0·4% (0·2–0·8%)	<0·1 (<0·1–<0·1)	-98·6% (-99·5 to -96·6%)
Kidney dysfunction	Stroke	12·7% (9·5–15·5%)	269·8 (196–347)	-9% (-29·2 to 14·8%)	11·4% (7·1–15·2%)	14 (8·7–19·6)	-7·2% (-28·9 to 19·4%)
	Ischaemic stroke	13·4% (9·2–17%)	213·2 (143·3–282·6)	7·6% (-15·6 to 36·2%)	11·4% (6·1–16·1%)	11 (5·9–16·2)	8·4% (-18·1 to 41·5%)
	Intracerebral haemorrhage	11·6% (9·1–14·1%)	56·6 (41·8–73·4)	-42·5% (-56·7 to -23·2%)	12·6% (9·1–16%)	3 (2·1–4·1)	-38·8% (-53·4 to -19·1%)
Lead exposure	Stroke	3·9% (2·1–5·9%)	83·3 (43·5–130·2)	-47·8% (-60·9 to -32·8%)	4·1% (2·3–6·2%)	5·1 (2·9–7·9)	-39·9% (-54·7 to -22·8%)
	Ischaemic stroke	3·9% (2·1–6%)	61·9 (32·5–97·1)	-33·5% (-50·8 to -15%)	4% (2·2–6·2%)	3·9 (2·1–6·3)	-25·6% (-43·6 to -4·8%)
	Intracerebral haemorrhage	4·1% (1·9–6·5%)	19·8 (9·1–33)	-68·1% (-77·9 to -57·4%)	4·6% (2·4–7·3%)	1·1 (0·6–1·8)	-63·5% (-73·5 to -51·4%)
	Subarachnoid haemorrhage	3·2% (1·3–5·6%)	1·6 (0·6–2·9)	-63·8% (-77·7 to -46·2%)	4·1% (2–6·7%)	0·1 (0–0·1)	-59·7% (-74·6 to -36·8%)
Low birth weight	Meningitis	3·5% (2·2–5·2%)	1·4 (0·9–2·2)	-87·9% (-94 to -76·1%)	1·8% (1·1–2·7%)	<0·1 (<0·1–<0·1)	-87·9% (-94 to -76·1%)
	Encephalitis	1·7% (1–2·9%)	0·3 (0·2–0·5)	-31·1% (-70·4 to 54·2%)	1·1% (0·6–1·9%)	<0·1 (<0·1–<0·1)	-31·1% (-70·4 to 54·2%)
Low birth weight and short gestation	Meningitis	3·9% (2·5–5·7%)	1·6 (1–2·4)	-87·6% (-93·8 to -75·6%)	2% (1·2–2·9%)	<0·1 (<0·1–<0·1)	-87·6% (-93·8 to -75·6%)
	Encephalitis	1·9% (1·1–3·2%)	0·3 (0·2–0·5)	-29·3% (-69·2 to 56·7%)	1·2% (0·7–2%)	<0·1 (<0·1–<0·1)	-29·3% (-69·2 to 56·7%)
Low physical activity	Stroke	5·8% (1·3–12·7%)	122·9 (26·5–276·5)	-12·3% (-30·1 to 12·7%)	7·1% (1·6–14·5%)	8·7 (2–18·2)	-10·3% (-29·2 to 16·4%)

Table S16 Regional and national disabilities and deaths related to neurological conditions attributable to risk factors in North Africa and Middle East countries

**Location**<br>Risk factor (alphabetic order)	Cause	**DALYs (95% UI)**<br>PAF (%)<br>2019	 Rate per 100,000<br>2019	 Rate change (%)<br>From 1990 to 2019	**Deaths (95% UI)**<br>PAF (%)<br>2019	 Rate per 100,000<br>2019	 Rate change (%)<br>From 1990 to 2019
	Ischaemic stroke	7·7% (1·7–16·9%)	122·9 (26·5–276·5)	-12·3% (-30·1 to 12·7%)	9% (2·1–18·4%)	8·7 (2–18·2)	-10·3% (-29·2 to 16·4%)
Low temperature	Stroke	4·8% (0·4–9·6%)	101·3 (8·8–206·4)	-39·4% (-54·7 to -16·3%)	5·4% (0·5–10·7%)	6·6 (0·6–13·3)	-33·9% (-49·8 to -9·1%)
	Ischaemic stroke	4·7% (0·4–9·4%)	74·3 (6·5–150·9)	-25·2% (-44·7 to 3·5%)	5·4% (0·5–10·7%)	5·2 (0·5–10·5)	-21·5% (-41·3 to 5·5%)
	Intracerebral haemorrhage	5·1% (0·4–10·2%)	24·7 (2·2–51)	-60·6% (-71·5 to -44·7%)	5·4% (0·5–10·7%)	1·3 (0·1–2·6)	-58·7% (-69·3 to -42·8%)
	Subarachnoid haemorrhage	4·3% (0·4–8·8%)	2·2 (0·2–4·5)	-54·1% (-69·5 to -25·4%)	5·4% (0·5–10·7%)	0·1 (0–0·2)	-52·6% (-69·1 to -23%)
Metabolic risk factors†	Stroke	72·4% (64·4–82%)	1541·4 (1292·4–1842·5)	-27·5% (-43·2 to -8·1%)	68·5% (58–81·6%)	83·8 (67·5–104·9)	-24·2% (-40·8 to -2·2%)
	Ischaemic stroke	72·5% (62·7–85·5%)	1153·8 (948–1418·5)	-12·6% (-30·3 to 10·4%)	68·1% (55·8–84·9%)	65·7 (50·9–85)	-10·2% (-29·5 to 16·4%)
	Intracerebral haemorrhage	72·5% (64·1–79·8%)	353·5 (286·8–428·4)	-52·8% (-64·3 to -37%)	70% (59·9–79·7%)	16·9 (13·4–21·1)	-52·1% (-63·7 to -36·2%)
	Subarachnoid haemorrhage	67·9% (58·8–75·4%)	34 (26·9–41·7)	-41·5% (-58·9 to -13·4%)	66·1% (55·2–76%)	1·2 (0·9–1·5)	-46% (-64 to -16·3%)
	Neurological disorders*	7·6% (2·3–17·1%)	103·5 (29·4–273)	45·8% (19·5 to 93·1%)	17·6% (6·2–32·6%)	6·6 (1·1–20·4)	46·5% (14·1 to 107·1%)
	Alzheimer's disease and other dementias	26·6% (11·3–44·9%)	103·5 (29·4–273)	45·8% (19·5 to 93·1%)	25·3% (10·1–43·5%)	6·6 (1·1–20·4)	46·5% (14·1 to 107·1%)
Non-optimal temperature	Stroke	5·2% (1·1–9·7%)	109·6 (23·8–208·7)	-37·3% (-56·2 to -4·2%)	5·8% (1·3–11%)	7·1 (1·5–13·5)	-31·6% (-51·9 to 6·8%)
	Ischaemic stroke	5·1% (1·1–9·5%)	80·5 (17·3–153·8)	-22·6% (-46·6 to 18·2%)	5·8% (1·3–11%)	5·6 (1·2–10·6)	-18·8% (-43·1 to 26%)
	Intracerebral haemorrhage	5·5% (1·2–10·5%)	26·8 (5·8–51·3)	-59·3% (-72·1 to -35·9%)	5·8% (1·3–11%)	1·4 (0·3–2·7)	-57·2% (-70·6 to -33·2%)
	Subarachnoid haemorrhage	4·7% (1–8·9%)	2·4 (0·5–4·6)	-52·5% (-70·7 to -13·8%)	5·8% (1·3–11%)	0·1 (0–0·2)	-50·9% (-70·1 to -12·9%)
Other environmental risks	Stroke	3·9% (2·1–5·9%)	83·3 (43·5–130·2)	-47·8% (-60·9 to -32·8%)	4·1% (2·3–6·2%)	5·1 (2·9–7·9)	-39·9% (-54·7 to -22·8%)
	Ischaemic stroke	3·9% (2·1–6%)	61·9 (32·5–97·1)	-33·5% (-50·8 to -15%)	4% (2·2–6·2%)	3·9 (2·1–6·3)	-25·6% (-43·6 to -4·8%)
	Intracerebral haemorrhage	4·1% (1·9–6·5%)	19·8 (9·1–33)	-68·1% (-77·9 to -57·4%)	4·6% (2·4–7·3%)	1·1 (0·6–1·8)	-63·5% (-73·5 to -51·4%)
	Subarachnoid haemorrhage	3·2% (1·3–5·6%)	1·6 (0·6–2·9)	-63·8% (-77·7 to -46·2%)	4·1% (2–6·7%)	0·1 (0–0·1)	-59·7% (-74·6 to -36·8%)
Particulate matter pollution	Meningitis	0·6% (0·2–1·1%)	0·2 (0·1–0·5)	-88·4% (-96 to -66%)	0·3% (0·1–0·6%)	<0·1 (<0·1–<0·1)	-88·4% (-96 to -66%)
	Encephalitis	0·3% (0·1–0·6%)	<0·1 (<0·1–0·1)	-34·1% (-83·5 to 126·1%)	0·2% (0·1–0·4%)	<0·1 (<0·1–<0·1)	-34·1% (-83·5 to 126·2%)
	Stroke	22·8% (17·5–27·8%)	485·7 (356·7–612·5)	-39·6% (-54·1 to -22·2%)	19·6% (14·9–24%)	24 (17·5–30·6)	-38·2% (-52·6 to -20·4%)
	Ischaemic stroke	22% (16·8–26·8%)	350·3 (258–442·5)	-26·1% (-43·3 to -6·5%)	19% (14·5–23·3%)	18·3 (13·2–23·4)	-25·8% (-43·2 to -4·7%)
	Intracerebral haemorrhage	25% (19·4–30·2%)	122 (88–155·9)	-59·8% (-70·2 to -45·9%)	21·9% (16·7–26·8%)	5·3 (3·8–6·8)	-60·3% (-70·2 to -46·9%)
	Subarachnoid haemorrhage	26·7% (21–32·2%)	13·4 (9·8–17·3)	-49% (-64·2 to -25·8%)	23% (17·8–28%)	0·4 (0·3–0·6)	-54·3% (-69·6 to -28·8%)
Secondhand smoke	Stroke	4·1% (3·1–5·1%)	87 (62·6–114·2)	-40·4% (-54·4 to -22·2%)	3·3% (2·5–4·2%)	4 (2·9–5·4)	-38·6% (-52·7 to -19·7%)
	Ischaemic stroke	3·8% (2·8–4·8%)	60 (43·1–79·1)	-26·3% (-43·6 to -4·6%)	3·1% (2·3–4%)	3 (2·2–4)	-25·4% (-43 to -3·1%)
	Intracerebral haemorrhage	5% (3·7–6·3%)	24·1 (17–32·7)	-59·1% (-70 to -44·5%)	4% (3–5·1%)	1 (0·7–1·3)	-59·7% (-70·1 to -45·6%)
	Subarachnoid haemorrhage	5·6% (4·2–7%)	2·8 (2–3·7)	-49·6% (-65·1 to -23·5%)	4·6% (3·5–5·8%)	0·1 (0·1–0·1)	-54% (-69·6 to -27·8%)
Short gestation	Meningitis	2·7% (1·8–4%)	1·1 (0·7–1·7)	-86·4% (-93·1 to -73·4%)	1·4% (0·9–2·1%)	<0·1 (<0·1–<0·1)	-86·4% (-93·1 to -73·4%)
	Encephalitis	1·3% (0·8–2·3%)	0·2 (0·1–0·4)	-22·6% (-66·3 to 73·1%)	0·8% (0·5–1·4%)	<0·1 (<0·1–<0·1)	-22·6% (-66·3 to 73·1%)
Smoking	Stroke	13·7% (12·3–15·5%)	292·3 (244·3–347·4)	-39·2% (-54·6 to -18%)	10·2% (9–11·6%)	12·5 (10·2–14·9)	-40·5% (-55·1 to -18·6%)

Table S16 Regional and national disabilities and deaths related to neurological conditions attributable to risk factors in North Africa and Middle East countries

Location<br>Risk factor (alphabetic order)	Cause	DALYs (95% UI)<br>PAF (%)<br>2019	Rate per 100,000<br>2019	Rate change (%)<br>From 1990 to 2019	Deaths (95% UI)<br>PAF (%)<br>2019	Rate per 100,000<br>2019	Rate change (%)<br>From 1990 to 2019
	Ischaemic stroke	12·7% (11·2–14·3%)	201·6 (162·4–240·9)	-25% (-43·9 to 0·7%)	9·4% (8·1–10·6%)	9 (7·2–10·8)	-27·7% (-46·1 to -0·8%)
	Intracerebral haemorrhage	17% (15·1–19·6%)	83·2 (66·6–103·4)	-58% (-69·5 to -40·9%)	13·3% (11·6–15·5%)	3·2 (2·5–4)	-59·9% (-70·7 to -43·8%)
	Subarachnoid haemorrhage	15·1% (12·6–17·7%)	7·6 (5·6–9·6)	-44·4% (-63·1 to -12%)	12·2% (9·4–14·9%)	0·2 (0·1–0·3)	-51% (-69·6 to -17·8%)
	Neurological disorders*	3·9% (1·2–8·3%)	53 (15·4–138)	-14·8% (-31·8 to 9·4%)	7·4% (1·2–13·5%)	2·9 (0·2–9·3)	-20·8% (-46·6 to 14·4%)
	Alzheimer's disease and other dementias	15·6% (10·1–20·9%)	60·6 (23·4–143·4)	-16·6% (-32·9 to 7%)	13·3% (8·3–18·1%)	3·5 (0·7–9·9)	-21·2% (-40·1 to 8%)
	Parkinson's disease	-10·9% (-16·1–-5·7%)	-10·1 (-15·8–-4·8)	-21·6% (-45·4 to 21·8%)	-9·2% (-13·9–-4·7%)	-0·6 (-0·9–-0·3)	-22·3% (-47·5 to 25·7%)
	Multiple sclerosis	13% (9·6–17·7%)	2·5 (1·6–3·8)	2·9% (-34·2 to 43%)	14·7% (10·7–21%)	<0·1 (<0·1–0·1)	-6·6% (-51 to 56%)
Tobacco	Stroke	17·2% (15·5–19·2%)	367 (306·7–432·3)	-39·3% (-53·7 to -19·2%)	13·1% (11·7–14·7%)	16·1 (13·3–19)	-39·8% (-54·2 to -19·4%)
	Ischaemic stroke	15·9% (14·2–17·8%)	253·7 (209·3–300·1)	-25·1% (-43·1 to -1·4%)	12·2% (10·7–13·7%)	11·7 (9·4–14)	-26·9% (-44·7 to -1·7%)
	Intracerebral haemorrhage	21·2% (18·9–23·8%)	103·3 (83·5–126·7)	-58·2% (-69·2 to -42·5%)	16·8% (14·8–19%)	4 (3·3–4·9)	-59·8% (-70·1 to -44·4%)
	Subarachnoid haemorrhage	19·9% (17·3–22·5%)	10 (7·6–12·4)	-45·8% (-62·7 to -16·8%)	16·3% (13·6–19·2%)	0·3 (0·2–0·4)	-51·8% (-68·6 to -21·9%)
	Neurological disorders*	3·9% (1·2–8·3%)	53 (15·4–138)	-14·8% (-31·8 to 9·4%)	7·4% (1·2–13·5%)	2·9 (0·2–9·3)	-20·8% (-46·6 to 14·4%)
	Alzheimer's disease and other dementias	15·6% (10·1–20·9%)	60·6 (23·4–143·4)	-16·6% (-32·9 to 7%)	13·3% (8·3–18·1%)	3·5 (0·7–9·9)	-21·2% (-40·1 to 8%)
	Parkinson's disease	-10·9% (-16·1–-5·7%)	-10·1 (-15·8–-4·8)	-21·6% (-45·4 to 21·8%)	-9·2% (-13·9–-4·7%)	-0·6 (-0·9–-0·3)	-22·3% (-47·5 to 25·7%)
	Multiple sclerosis	13% (9·6–17·7%)	2·5 (1·6–3·8)	2·9% (-34·2 to 43%)	14·7% (10·7–21%)	<0·1 (<0·1–0·1)	-6·6% (-51 to 56%)
**Oman**							
Air pollution	Meningitis	0·8% (0·3–1·6%)	0·2 (0·1–0·4)	-84·3% (-94·5 to -60·3%)	0·2% (0·1–0·5%)	<0·1 (<0·1–<0·1)	-84·3% (-94·5 to -60·3%)
	Encephalitis	0·4% (0·2–0·8%)	0·5 (0·2–1)	-49·1% (-85·2 to 57%)	0·2% (0·1–0·3%)	<0·1 (<0·1–<0·1)	-49·1% (-85·2 to 57%)
	Stroke	27·5% (21·3–33%)	517·8 (390·5–652·4)	-47·3% (-59·8 to -29·8%)	24·6% (19–29·9%)	25·5 (19·1–32·5)	-40·9% (-55·5 to -18·9%)
	Ischaemic stroke	27% (20·8–32·7%)	348·1 (263·9–443·2)	-30·3% (-46·9 to -6·6%)	23·8% (18·5–29·1%)	18·2 (13·5–23·7)	-22·8% (-43·8 to 6·6%)
	Intracerebral haemorrhage	28·9% (22·8–34·8%)	146 (109·7–192·1)	-65·4% (-74·2 to -52·5%)	26·7% (20·8–32·3%)	6·4 (4·8–8·5)	-63% (-72·7 to -48·7%)
	Subarachnoid haemorrhage	26·5% (20·4–32·6%)	23·7 (14·2–39·9)	-61·6% (-77 to -26·4%)	26·7% (20·8–32·4%)	0·8 (0·4–1·4)	-62·1% (-80 to -25·2%)
Alcohol use	Stroke	0% (-0·2–0·3%)	0·4 (-4·2–5·3)	-82·9% (-661·9 to 391·3%)	0% (-0·2–0·2%)	0 (-0·2–0·2)	-280·6% (-584·6 to 980·3%)
	Ischaemic stroke	-0·2% (-0·4–0·1%)	-2·2 (-5·9–1·6)	-20·6% (-150·7 to 164%)	-0·2% (-0·4–0·1%)	-0·1 (-0·3–0)	-4·7% (-149·8 to 138·6%)
	Intracerebral haemorrhage	0·5% (0·1–1%)	2·5 (0·4–5·3)	-48·5% (-77·8 to 40·5%)	0·3% (0–0·7%)	0·1 (0–0·2)	-48% (-78 to 62·1%)
	Neurological disorders*	0·1% (0–0·3%)	1·5 (0·4–3·3)	26·3% (-57·8 to 272·4%)	0% (0–0%)	<0·1 (<0·1–<0·1)	-18·3% (-52·9 to 61·4%)
	Idiopathic epilepsy	1·4% (0·6–2·3%)	1·5 (0·4–3·3)	26·3% (-57·8 to 272·4%)	1·6% (0·7–2·7%)	<0·1 (<0·1–<0·1)	-18·3% (-52·9 to 61·4%)
All risk factors	Meningitis	4·2% (2·4–7·4%)	1 (0·5–1·9)	-83·3% (-91·7 to -64·3%)	1·2% (0·6–2·4%)	<0·1 (<0·1–<0·1)	-83·3% (-91·7 to -64·3%)
	Encephalitis	2% (1–3·7%)	2·5 (1·2–4·4)	-43·8% (-76·9 to 36·5%)	0·9% (0·4–1·6%)	<0·1 (<0·1–0·1)	-43·8% (-76·9 to 36·5%)
	Stroke	85·4% (81·1–90·1%)	1610·3 (1402·6–1845·1)	-33·1% (-48 to -12·1%)	84·6% (78·5–91·2%)	87·7 (74·8–102·8)	-23·1% (-41·6 to 4·8%)
	Ischaemic stroke	86·8% (81·3–92·9%)	1120·3 (948–1304·7)	-13% (-31·3 to 15·5%)	84·7% (77·3–93·4%)	64·9 (52·4–77·8)	-1·4% (-26·3 to 34·6%)

Table S16 Regional and national disabilities and deaths related to neurological conditions attributable to risk factors in North Africa and Middle East countries

Location<br>Risk factor (alphabetic order)	Cause	DALYs (95% UI)<br>PAF (%)<br>2019	Rate per 100,000<br>2019	Rate change (%)<br>From 1990 to 2019	Deaths (95% UI)<br>PAF (%)<br>2019	Rate per 100,000<br>2019	Rate change (%)<br>From 1990 to 2019
	Intracerebral haemorrhage	84·3% (80–88·8%)	425·1 (364–534·7)	-56·6% (-67·6 to -40·3%)	84·8% (78·8–90·5%)	20·4 (17–26·4)	-52·6% (-65 to -33·7%)
	Subarachnoid haemorrhage	72·4% (64·4–80·9%)	64·9 (41·4–103·2)	-54·1% (-72·2 to -13·7%)	79·6% (73·4–85·9%)	2·5 (1·4–4)	-52·5% (-74·9 to -8·7%)
	Neurological disorders*	11·6% (4·5–23·9%)	155·2 (52·5–394·6)	53·4% (23 to 113·9%)	23·2% (9·4–38·4%)	9·9 (1·7–30·2)	60·8% (24·3 to 160%)
	Alzheimer's disease and other dementias	37·2% (22·8–53·8%)	157·3 (55·5–397·3)	51·2% (21·2 to 108·3%)	35·2% (21–51·9%)	10·2 (2–30·4)	58·6% (21·9 to 150%)
	Parkinson's disease	-3·9% (-5·9–-2·1%)	-4·9 (-7·6–-2·3)	-9·3% (-41·2 to 40%)	-3·6% (-5·5–-1·9%)	-0·3 (-0·5–-0·1)	6·6% (-33·6 to 71·1%)
	Idiopathic epilepsy	1·4% (0·6–2·3%)	1·5 (0·4–3·3)	26·3% (-57·8 to 272·4%)	1·6% (0·7–2·7%)	<0·1 (<0·1–<0·1)	-18·3% (-52·9 to 61·4%)
	Multiple sclerosis	7·9% (5·6–10·6%)	1·3 (0·8–1·9)	-0·3% (-40 to 37·9%)	7·7% (4·8–10·9%)	<0·1 (<0·1–<0·1)	-10% (-63·3 to 52·6%)
Ambient particulate matter pollution	Meningitis	0·8% (0·3–1·6%)	0·2 (0·1–0·4)	-65·7% (-89 to 2·4%)	0·2% (0·1–0·5%)	<0·1 (<0·1–<0·1)	-65·7% (-89 to 2·3%)
	Encephalitis	0·4% (0·2–0·8%)	0·5 (0·2–1)	11·6% (-67·6 to 303·7%)	0·2% (0·1–0·3%)	<0·1 (<0·1–<0·1)	11·6% (-67·6 to 303·7%)
	Stroke	27·4% (21·2–32·9%)	515·7 (388·7–651·2)	2·1% (-30·9 to 69·8%)	24·5% (18·9–29·8%)	25·4 (19–32·5)	15·4% (-22·3 to 99%)
	Ischaemic stroke	26·8% (20·7–32·5%)	346·7 (262·4–442)	32·4% (-8·8 to 121·9%)	23·7% (18·4–29%)	18·2 (13·4–23·6)	47·9% (-0·9 to 154%)
	Intracerebral haemorrhage	28·8% (22·7–34·7%)	145·4 (108·4–191·7)	-31·4% (-54·9 to 19·9%)	26·6% (20·7–32·2%)	6·4 (4·7–8·5)	-25·8% (-51·3 to 30·3%)
	Subarachnoid haemorrhage	26·4% (20·3–32·5%)	23·6 (14·1–39·8)	-24·7% (-58·7 to 55·7%)	26·6% (20·6–32·3%)	0·8 (0·4–1·4)	-24·9% (-62·5 to 62·5%)
Behavioural risk factors‡	Meningitis	4·1% (2·2–7%)	1 (0·5–1·9)	-83% (-91·4 to -64%)	1·2% (0·6–2·2%)	<0·1 (<0·1–<0·1)	-83% (-91·4 to -64%)
	Encephalitis	1·9% (1–3·6%)	2·4 (1·1–4·3)	-42·6% (-76·3 to 39·3%)	0·8% (0·4–1·5%)	<0·1 (<0·1–<0·1)	-42·6% (-76·3 to 39·3%)
	Stroke	26·9% (21·6–32·7%)	507·3 (394·1–645·6)	-46·1% (-59 to -27·9%)	25·1% (19·3–31·8%)	26 (19·2–34·5)	-36·9% (-53·3 to -14·3%)
	Ischaemic stroke	29·2% (21·9–36·9%)	377·1 (274·5–493·2)	-29·8% (-46·2 to -6·6%)	27·1% (19·3–35·2%)	20·7 (14·5–28·4)	-19·1% (-39·4 to 10·3%)
	Intracerebral haemorrhage	22·1% (16·6–27·5%)	111·3 (80·2–154)	-68·4% (-76·7 to -55·8%)	19·5% (14·3–24·8%)	4·7 (3·3–6·6)	-66·2% (-75·4 to -53·1%)
	Subarachnoid haemorrhage	21·2% (16·3–26·9%)	19 (11·2–32·6)	-64·2% (-78·2 to -28·9%)	20·3% (15·4–26%)	0·6 (0·3–1·1)	-65% (-81·1 to -28·8%)
	Neurological disorders*	2·4% (0·8–5%)	32·4 (10–78·7)	-21·1% (-38·7 to 4·5%)	3·7% (0·4–7·2%)	1·7 (0·1–5·3)	-23% (-63·1 to 16%)
	Alzheimer's disease and other dementias	8·2% (4·9–11·8%)	34·5 (12·7–81·7)	-21·6% (-37·8 to 3·2%)	6·8% (4–10%)	2 (0·4–5·6)	-19·5% (-39·5 to 16·6%)
	Parkinson's disease	-3·9% (-5·9–-2·1%)	-4·9 (-7·6–-2·3)	-9·3% (-41·2 to 40%)	-3·6% (-5·5–-1·9%)	-0·3 (-0·5–-0·1)	6·6% (-33·6 to 71·1%)
	Idiopathic epilepsy	1·4% (0·6–2·3%)	1·5 (0·4–3·3)	26·3% (-57·8 to 272·4%)	1·6% (0·7–2·7%)	<0·1 (<0·1–<0·1)	-18·3% (-52·9 to 61·4%)
	Multiple sclerosis	7·9% (5·6–10·6%)	1·3 (0·8–1·9)	-0·3% (-40 to 37·9%)	7·7% (4·8–10·9%)	<0·1 (<0·1–<0·1)	-10% (-63·3 to 52·6%)
Child and maternal malnutrition	Meningitis	4·1% (2·2–7%)	1 (0·5–1·9)	-83% (-91·4 to -64%)	1·2% (0·6–2·2%)	<0·1 (<0·1–<0·1)	-83% (-91·4 to -64%)
	Encephalitis	1·9% (1–3·6%)	2·4 (1·1–4·3)	-42·6% (-76·3 to 39·3%)	0·8% (0·4–1·5%)	<0·1 (<0·1–<0·1)	-42·6% (-76·3 to 39·3%)
Diet high in red meat	Stroke	5·9% (3–8·2%)	112·1 (56·9–157·9)	-31% (-50·1 to 1·6%)	4·7% (2·2–6·6%)	4·9 (2·3–7·1)	-23·4% (-46 to 12·7%)
	Ischaemic stroke	5·3% (1·6–7·6%)	69 (22·1–101·2)	-6·3% (-29·9 to 43·5%)	4·1% (1·2–6%)	3·2 (0·9–4·8)	4·2% (-22·9 to 62·3%)
	Intracerebral haemorrhage	7·3% (2·4–10·6%)	36·7 (12–56·3)	-52·3% (-65·4 to -21·1%)	6·3% (2–9·3%)	1·5 (0·4–2·3)	-48·9% (-62·9 to -15·9%)
	Subarachnoid haemorrhage	7·2% (2·6–10·6%)	6·4 (2·1–11·9)	-46·4% (-67·8 to 11·5%)	6·6% (2·1–9·7%)	0·2 (0·1–0·4)	-47·4% (-72 to 11·8%)
Diet high in sodium	Stroke	1·8% (0·2–7·2%)	33·4 (4·6–136·8)	-40·9% (-63·6 to 20·5%)	1·5% (0·3–6·2%)	1·6 (0·3–6·6)	-34·2% (-60·5 to 34%)

Table S16 Regional and national disabilities and deaths related to neurological conditions attributable to risk factors in North Africa and Middle East countries

**Location**<br>Risk factor (alphabetic order)	Cause	**DALYs (95% UI)**<br>PAF (%)<br>2019	 Rate per 100,000<br>2019	 Rate change (%)<br>From 1990 to 2019	**Deaths (95% UI)**<br>PAF (%)<br>2019	 Rate per 100,000<br>2019	 Rate change (%)<br>From 1990 to 2019
	Ischaemic stroke	1·7% (0·2–7%)	22·3 (3·2–95·1)	-23% (-52·7 to 57·6%)	1·4% (0·3–5·9%)	1·1 (0·2–4·8)	-15·1% (-49·9 to 75·6%)
	Intracerebral haemorrhage	1·9% (0·2–7·5%)	9·5 (1·2–38·2)	-60·4% (-77·8 to -19·2%)	1·7% (0·3–6·9%)	0·4 (0·1–1·7)	-57·5% (-76 to -13%)
	Subarachnoid haemorrhage	1·8% (0·2–7%)	1·6 (0·1–7)	-55% (-78·3 to 1·8%)	1·8% (0·2–7·2%)	0·1 (0–0·3)	-55·7% (-80·3 to 0·2%)
Diet low in fibre	Stroke	1·4% (0·3–2·8%)	25·8 (6·2–54·7)	-74·3% (-82 to -61%)	1·3% (0·3–2·7%)	1·3 (0·3–2·7)	-69·6% (-79·4 to -53·1%)
	Ischaemic stroke	1·3% (0·3–2·7%)	17·2 (4·4–36·4)	-65·2% (-75·8 to -48·8%)	1·3% (0·3–2·6%)	1 (0·2–2)	-58·9% (-72·9 to -36·3%)
	Intracerebral haemorrhage	1·4% (0·3–3·1%)	7·2 (1·7–16·6)	-83·5% (-88·7 to -74·8%)	1·4% (0·3–2·9%)	0·3 (0·1–0·8)	-81·7% (-87·8 to -70·5%)
	Subarachnoid haemorrhage	1·5% (0·3–3·2%)	1·3 (0·2–3·2)	-81·2% (-89·3 to -63·5%)	1·4% (0·3–3·1%)	<0·1 (<0·1–0·1)	-81·4% (-90·4 to -64·1%)
Diet low in fruits	Stroke	1·9% (0·9–3%)	35·2 (17·3–57·8)	-65·4% (-76·3 to -50·7%)	1·6% (0·8–2·6%)	1·6 (0·8–2·7)	-60·6% (-73·9 to -41·6%)
	Ischaemic stroke	1·4% (0·4–2·8%)	18·7 (4·8–36·5)	-49·5% (-64 to -21·8%)	1·2% (0·3–2·4%)	0·9 (0·3–1·9)	-41·5% (-61·8 to -6%)
	Intracerebral haemorrhage	2·8% (1·3–4·5%)	13·9 (6·4–23·9)	-75·1% (-82·5 to -64·4%)	2·5% (1·2–4·1%)	0·6 (0·3–1)	-73·2% (-81·9 to -60%)
	Subarachnoid haemorrhage	2·9% (1·4–5·1%)	2·6 (1–5·2)	-70·6% (-82·2 to -45·8%)	2·6% (1·2–4·4%)	0·1 (0–0·2)	-72% (-85·4 to -47·1%)
Diet low in vegetables	Stroke	1·5% (0·4–2·9%)	28·6 (6·9–55·6)	-54% (-70·6 to -28·1%)	1·4% (0·4–2·6%)	1·4 (0·4–2·7)	-49·5% (-68·5 to -19·6%)
	Ischaemic stroke	1·4% (0·2–2·8%)	18·4 (2·7–36·9)	-35·8% (-54·7 to -11·4%)	1·3% (0·2–2·5%)	1 (0·2–2)	-29·2% (-52·9 to 1·3%)
	Intracerebral haemorrhage	1·7% (0·2–5·1%)	8·8 (1–25·4)	-70·1% (-81·1 to -56·1%)	1·7% (0·2–4·9%)	0·4 (0–1·1)	-68·7% (-80·8 to -52·1%)
	Subarachnoid haemorrhage	1·6% (0·2–4·8%)	1·4 (0·1–4·6)	-66·8% (-83·3 to -37·6%)	1·7% (0·2–4·9%)	0·1 (0–0·2)	-67·6% (-85·1 to -38·3%)
Diet low in whole grains	Stroke	3·3% (0·8–4·8%)	61·5 (15·1–93)	-30·4% (-47·7 to -7·8%)	3% (0·8–4·4%)	3·1 (0·8–4·8)	-21·5% (-42·5 to 6·5%)
	Ischaemic stroke	4·8% (1·2–7%)	61·5 (15·1–93)	-30·4% (-47·7 to -7·8%)	4·1% (1–6%)	3·1 (0·8–4·8)	-21·5% (-42·5 to 6·5%)
Dietary risks	Stroke	14·6% (10·5–19·4%)	275·4 (191·6–378·4)	-47·2% (-60·1 to -29·1%)	12·8% (9–16·9%)	13·2 (9·1–18·4)	-41% (-56·8 to -19·8%)
	Ischaemic stroke	14·9% (9·6–19·8%)	191·8 (120·4–265·7)	-31% (-47·4 to -8·7%)	12·7% (8·3–17·2%)	9·7 (6·2–13·8)	-23·4% (-43·3 to 4·9%)
	Intracerebral haemorrhage	14·1% (8·2–20·2%)	71·2 (40·5–105·9)	-66·3% (-75·5 to -52·7%)	12·8% (7·3–18·7%)	3·1 (1·7–4·7)	-64·2% (-74·5 to -49·4%)
	Subarachnoid haemorrhage	13·8% (8·4–19·9%)	12·4 (6·3–21·7)	-61·9% (-76·9 to -25·2%)	13·2% (7·7–19%)	0·4 (0·2–0·8)	-63% (-80·5 to -25·5%)
Environmental/occupational risk factors§	Meningitis	0·8% (0·3–1·6%)	0·2 (0·1–0·4)	-84·3% (-94·5 to -60·3%)	0·2% (0·1–0·5%)	<0·1 (<0·1–<0·1)	-84·3% (-94·5 to -60·3%)
	Encephalitis	0·4% (0·2–0·8%)	0·5 (0·2–1)	-49·1% (-85·2 to 57%)	0·2% (0·1–0·3%)	<0·1 (<0·1–<0·1)	-49·1% (-85·2 to 57%)
	Stroke	35·2% (27·5–42·2%)	662·8 (509·7–843·8)	-46% (-59·3 to -25·6%)	33·5% (25·2–41·3%)	34·8 (25·7–45·3)	-37·6% (-54·5 to -13·1%)
	Ischaemic stroke	34·6% (27–41·8%)	447 (336·1–565·1)	-28·1% (-45·6 to -1·1%)	32·8% (24·6–40·7%)	25·1 (17·9–32·9)	-18·4% (-41 to 14·4%)
	Intracerebral haemorrhage	36·8% (28·7–44·4%)	185·5 (138·9–244·7)	-64·6% (-73·6 to -49·8%)	35·5% (26·8–43·2%)	8·5 (6·2–11·4)	-61·4% (-71·9 to -44·9%)
	Subarachnoid haemorrhage	33·8% (26·2–42·1%)	30·2 (18·1–50·1)	-62·6% (-77·8 to -27·6%)	35·3% (26·9–43·2%)	1·1 (0·6–1·8)	-61·2% (-79·5 to -24·3%)
High body-mass index	Stroke	31·6% (22·1–40·7%)	595·5 (401·5–804·2)	-1·4% (-31·5 to 55·7%)	21·6% (14–29·8%)	22·4 (14·1–31·8)	13·1% (-25·9 to 84·3%)
	Ischaemic stroke	26% (17·6–35%)	335·2 (211·4–468·7)	59·6% (6·4 to 165·8%)	17·5% (10·4–25·8%)	13·4 (7·8–20·1)	82·2% (15·1 to 223·2%)
	Intracerebral haemorrhage	43·9% (32·3–54·6%)	221·2 (154·5–300·2)	-34·7% (-55·8 to 3%)	33% (22·2–44·5%)	7·9 (5·2–11·3)	-27·7% (-51·8 to 18·1%)
	Subarachnoid haemorrhage	43·8% (32·8–54·5%)	39·1 (22·6–65·9)	-29% (-57·8 to 37·3%)	36·1% (25·1–47·5%)	1·1 (0·5–2)	-27% (-61·4 to 48·9%)
	Neurological disorders*	6·6% (2–14·8%)	88·3 (24·5–226·5)	164·2% (82 to 416·8%)	13·7% (4·7–26·2%)	5·8 (1–17·6)	174·2% (75·1 to 501·8%)

Table S16 Regional and national disabilities and deaths related to neurological conditions attributable to risk factors in North Africa and Middle East countries

Location<br>Risk factor (alphabetic order)	Cause	DALYs (95% UI)<br>PAF (%)<br>2019	Rate per 100,000<br>2019	Rate change (%)<br>From 1990 to 2019	Deaths (95% UI)<br>PAF (%)<br>2019	Rate per 100,000<br>2019	Rate change (%)<br>From 1990 to 2019
	Alzheimer's disease and other dementias	20·9% (9·2–35·9%)	88·3 (24·5–226·5)	164·2% (82 to 416·8%)	20% (8·6–34·7%)	5·8 (1–17·6)	174·2% (75·1 to 501·8%)
High fasting plasma glucose	Stroke	31·8% (19·5–51·3%)	600·6 (370·3–978·1)	15·7% (-20·5 to 73·4%)	32% (18·1–59·6%)	33·2 (18·5–61·5)	28·5% (-16·6 to 95·7%)
	Ischaemic stroke	33·5% (17–62·5%)	433·2 (214·3–799·7)	49·8% (4·6 to 120·5%)	32·4% (15·1–69%)	24·9 (11·2–53)	64·9% (8·3 to 148·9%)
	Intracerebral haemorrhage	29·4% (18–42·9%)	148·2 (87·6–221)	-27·3% (-49·2 to 10%)	31·2% (17·7–49·1%)	7·5 (4·2–12·3)	-22·3% (-46·9 to 18·8%)
	Subarachnoid haemorrhage	21·3% (13–30·9%)	19·2 (9–33·4)	-26·7% (-59·4 to 38·4%)	28% (16·7–43·3%)	0·9 (0·4–1·6)	-23·4% (-61 to 48·3%)
	Neurological disorders*	4·3% (0·8–11·6%)	57·6 (10·3–187·2)	59·6% (36·1 to 117·3%)	9% (1·9–21·4%)	3·8 (0·4–14·3)	59·1% (29·4 to 146·2%)
	Alzheimer's disease and other dementias	13·6% (3·3–28·2%)	57·6 (10·3–187·2)	59·6% (36·1 to 117·3%)	13·1% (3·2–27·6%)	3·8 (0·4–14·3)	59·1% (29·4 to 146·2%)
High LDL cholesterol	Stroke	16% (7·8–29·3%)	302·4 (146·3–570·7)	-12% (-36·5 to 17·2%)	15·6% (4·9–32·8%)	16·1 (5–36)	1·9% (-30·5 to 40%)
	Ischaemic stroke	23·4% (11·4–42·7%)	302·4 (146·3–570·7)	-12% (-36·5 to 17·2%)	21·1% (6·6–44·6%)	16·1 (5–36)	1·9% (-30·5 to 40%)
High systolic blood pressure	Stroke	52·3% (43·2–60·6%)	986·4 (779·1–1209·1)	-18·2% (-38·9 to 14%)	49·1% (39–59·4%)	50·9 (38·8–64·5)	-6·9% (-33 to 32·5%)
	Ischaemic stroke	50·6% (39·5–60·8%)	652·8 (493–821·8)	10·2% (-19 to 52·7%)	46·5% (34·2–59·9%)	35·6 (25·4–47·6)	24·1% (-13·2 to 78·1%)
	Intracerebral haemorrhage	57·4% (46·9–66·9%)	289·4 (225·4–373·9)	-46·2% (-61 to -22·5%)	56·9% (43·6–69·1%)	13·7 (10–18·2)	-41·1% (-58·7 to -13·9%)
	Subarachnoid haemorrhage	49·2% (40·4–59·9%)	44·3 (26·3–71·3)	-41·5% (-65·4 to 12%)	54·5% (43·6–65·6%)	1·7 (0·9–2·9)	-40·1% (-68·9 to 18·5%)
High temperature	Stroke	5·1% (1·6–11·6%)	96·5 (29·4–212·3)	-39·9% (-81·8 to -13·4%)	5·9% (1·9–13·2%)	6·1 (1·9–13·3)	-26·9% (-77·1 to 2·9%)
	Ischaemic stroke	5% (1·6–11·2%)	64·1 (20·1–141·1)	-18·5% (-75·2 to 17·1%)	5·9% (1·9–13·2%)	4·5 (1·4–9·9)	-5·4% (-71·6 to 35·5%)
	Intracerebral haemorrhage	5·5% (1·8–12·4%)	27·8 (8·8–62)	-59·8% (-87·3 to -39·8%)	5·9% (1·9–13·2%)	1·4 (0·4–3·2)	-55·3% (-86·3 to -31·9%)
	Subarachnoid haemorrhage	5·3% (1·7–11·7%)	4·7 (1·3–10·8)	-63·6% (-88·8 to -19·6%)	5·9% (1·9–13·2%)	0·2 (0–0·4)	-57·2% (-86·8 to -11·2%)
Household air pollution from solid fuels	Meningitis	0% (0–0%)	<0·1 (<0·1–<0·1)	-99·9% (-100 to -99·5%)	0% (0–0%)	<0·1 (<0·1–<0·1)	-99·9% (-100 to -99·5%)
	Encephalitis	0% (0–0%)	<0·1 (<0·1–<0·1)	-99·5% (-99·9 to -98·1%)	0% (0–0%)	<0·1 (<0·1–<0·1)	-99·5% (-99·9 to -98·1%)
	Stroke	0·1% (0–0·3%)	2 (0·5–5·3)	-99·6% (-99·9 to -98·8%)	0·1% (0–0·2%)	0·1 (0–0·3)	-99·5% (-99·9 to -98·6%)
	Ischaemic stroke	0·1% (0–0·3%)	1·3 (0·3–3·4)	-99·4% (-99·8 to -98·4%)	0·1% (0–0·2%)	0·1 (0–0·2)	-99·4% (-99·8 to -98·2%)
	Intracerebral haemorrhage	0·1% (0–0·3%)	0·6 (0·2–1·5)	-99·7% (-99·9 to -99·2%)	0·1% (0–0·3%)	<0·1 (<0·1–0·1)	-99·7% (-99·9 to -99·1%)
	Subarachnoid haemorrhage	0·1% (0–0·3%)	0·1 (0–0·3)	-99·7% (-99·9 to -99%)	0·1% (0–0·3%)	<0·1 (<0·1–<0·1)	-99·7% (-99·9 to -99%)
Kidney dysfunction	Stroke	11·9% (8·9–14·4%)	224 (163·8–287·1)	-4% (-27·2 to 30·1%)	11·4% (7·5–14·9%)	11·8 (7·6–16·2)	5·5% (-23·6 to 46·4%)
	Ischaemic stroke	13% (9·1–16·5%)	167·7 (113·9–224·1)	20·7% (-9·7 to 61·9%)	11·5% (6·3–15·9%)	8·8 (4·8–12·9)	31·4% (-7·8 to 81·2%)
	Intracerebral haemorrhage	11·2% (8·7–13·7%)	56·3 (40·9–74·2)	-40·5% (-56·4 to -15·6%)	12·6% (9–16%)	3 (2–4·2)	-32·8% (-51 to -5·8%)
Lead exposure	Stroke	3·1% (1·4–4·9%)	57·7 (25·6–94)	-55·4% (-69·1 to -39·8%)	3·3% (1·6–5·2%)	3·4 (1·6–5·4)	-42·1% (-58·8 to -20·6%)
	Ischaemic stroke	3·1% (1·5–5·2%)	40·7 (18·4–67·9)	-37·3% (-55·6 to -14·2%)	3·2% (1·6–5·3%)	2·5 (1·2–4·2)	-21% (-44·5 to 9·6%)
	Intracerebral haemorrhage	3% (1·1–5·2%)	15 (5·5–27)	-73·4% (-83·3 to -61·8%)	3·4% (1·5–5·9%)	0·8 (0·3–1·5)	-66% (-77 to -52%)
	Subarachnoid haemorrhage	2·2% (0·7–4·2%)	2 (0·5–4·7)	-74·7% (-87·9 to -52·8%)	3·1% (1·2–5·4%)	0·1 (0–0·2)	-67·5% (-84·3 to -37·1%)
Low birth weight	Meningitis	3·7% (2–6·5%)	0·9 (0·5–1·7)	-83·4% (-91·8 to -64·5%)	1·1% (0·6–2·1%)	<0·1 (<0·1–<0·1)	-83·4% (-91·8 to -64·5%)

Table S16 Regional and national disabilities and deaths related to neurological conditions attributable to risk factors in North Africa and Middle East countries

Location<br>Risk factor (alphabetic order)	Cause	DALYs (95% UI)<br>PAF (%)<br>2019	Rate per 100,000<br>2019	Rate change (%)<br>From 1990 to 2019	Deaths (95% UI)<br>PAF (%)<br>2019	Rate per 100,000<br>2019	Rate change (%)<br>From 1990 to 2019
	Encephalitis	1·8% (0·9–3·3%)	2·2 (1–4)	-44% (-77·4 to 39·3%)	0·8% (0·4–1·4%)	<0·1 (<0·1–<0·1)	-44% (-77·4 to 39·3%)
Low birth weight and short gestation	Meningitis	4·1% (2·2–7%)	1 (0·5–1·9)	-83% (-91·4 to -64%)	1·2% (0·6–2·2%)	<0·1 (<0·1–<0·1)	-83% (-91·4 to -64%)
	Encephalitis	1·9% (1–3·6%)	2·4 (1·1–4·3)	-42·6% (-76·3 to 39·3%)	0·8% (0·4–1·5%)	<0·1 (<0·1–<0·1)	-42·6% (-76·3 to 39·3%)
Low physical activity	Stroke	6% (1·3–12·6%)	113·3 (24·8–235·8)	4·9% (-21·8 to 54·9%)	7·5% (1·7–14·6%)	7·7 (1·7–15·3)	15·1% (-14·7 to 71·7%)
	Ischaemic stroke	8·8% (1·9–18·2%)	113·3 (24·8–235·8)	4·9% (-21·8 to 54·9%)	10·1% (2·3–19·8%)	7·7 (1·7–15·3)	15·1% (-14·7 to 71·7%)
Low temperature	Stroke	2·8% (-4–8·7%)	52·6 (-73·8–163·7)	-41·4% (-160·3 to 426·2%)	3·2% (-4·5–10%)	3·3 (-4·7–10·5)	-28·7% (-172·9 to 518·4%)
	Ischaemic stroke	2·7% (-3·9–8·5%)	35·1 (-48·7–109·2)	-20·4% (-182·4 to 611·7%)	3·2% (-4·5–10%)	2·5 (-3·4–7·7)	-7·5% (-193·1 to 697·4%)
	Intracerebral haemorrhage	3% (-4·3–9·4%)	15 (-23–47·5)	-61·1% (-142·7 to 265·3%)	3·2% (-4·5–10%)	0·8 (-1·2–2·4)	-56·7% (-147·9 to 290·3%)
	Subarachnoid haemorrhage	2·9% (-4·1–9·1%)	2·5 (-3·8–8·9)	-64·9% (-133·4 to 200·2%)	3·2% (-4·5–10%)	0·1 (-0·1–0·4)	-58·6% (-134·3 to 249·7%)
Metabolic risk factors†	Stroke	74·9% (67·3–82·9%)	1410·9 (1186·6–1652·5)	-21·1% (-38·9 to 6·1%)	72·3% (62·2–84%)	75 (60·8–91·5)	-9·5% (-32·1 to 25·2%)
	Ischaemic stroke	75·6% (65·8–87·2%)	977 (791·7–1175·5)	0·9% (-21·6 to 35·6%)	71·8% (58·7–87·2%)	55 (41·8–69·8)	14·9% (-14·6 to 58·7%)
	Intracerebral haemorrhage	74·9% (67·9–81·2%)	378 (317·5–476·9)	-47·8% (-61·3 to -26·3%)	74·6% (65–83·3%)	17·9 (14·5–23·3)	-43·1% (-58·6 to -18·5%)
	Subarachnoid haemorrhage	62·4% (53·5–72·2%)	56 (34·3–87·8)	-42·2% (-65·6 to 6·2%)	68·3% (58·8–77·2%)	2·1 (1·2–3·4)	-41·3% (-69·1 to 13·2%)
	Neurological disorders*	10% (3·5–21·4%)	133·9 (39·3–348·1)	100·6% (55·5 to 222·5%)	20·9% (8·2–36·1%)	8·8 (1·6–27·3)	103·8% (50·6 to 268·2%)
	Alzheimer's disease and other dementias	31·6% (16·4–49·7%)	133·9 (39·3–348·1)	100·6% (55·5 to 222·5%)	30·4% (15·3–48·3%)	8·8 (1·6–27·3)	103·8% (50·6 to 268·2%)
Non-optimal temperature	Stroke	7·8% (-0·2–14·7%)	146·2 (-3·6–292·9)	-40·4% (-93·4 to 31·2%)	8·9% (-0·2–16·9%)	9·2 (-0·2–18·7)	-27·5% (-92·2 to 61·5%)
	Ischaemic stroke	7·5% (-0·2–14·2%)	97·2 (-2·3–194·2)	-19·2% (-91·4 to 74·2%)	8·9% (-0·2–16·9%)	6·8 (-0·2–13·6)	-6·1% (-90·1 to 104·1%)
	Intracerebral haemorrhage	8·3% (-0·2–15·8%)	41·9 (-1–84·3)	-60·2% (-95·7 to -17·3%)	8·9% (-0·2–16·9%)	2·1 (-0·1–4·2)	-55·8% (-95·3 to -6·4%)
	Subarachnoid haemorrhage	8% (-0·2–15·5%)	7·1 (-0·2–16·6)	-64% (-95·8 to -13·1%)	8·9% (-0·2–16·9%)	0·3 (0–0·7)	-57·7% (-95 to -2·2%)
Other environmental risks	Stroke	3·1% (1·4–4·9%)	57·7 (25·6–94)	-55·4% (-69·1 to -39·8%)	3·3% (1·6–5·2%)	3·4 (1·6–5·4)	-42·1% (-58·8 to -20·6%)
	Ischaemic stroke	3·1% (1·5–5·2%)	40·7 (18·4–67·9)	-37·3% (-55·6 to -14·2%)	3·2% (1·6–5·3%)	2·5 (1·2–4·2)	-21% (-44·5 to 9·6%)
	Intracerebral haemorrhage	3% (1·1–5·2%)	15 (5·5–27)	-73·4% (-83·3 to -61·8%)	3·4% (1·5–5·9%)	0·8 (0·3–1·5)	-66% (-77 to -52%)
	Subarachnoid haemorrhage	2·2% (0·7–4·2%)	2 (0·5–4·7)	-74·7% (-87·9 to -52·8%)	3·1% (1·2–5·4%)	0·1 (0–0·2)	-67·5% (-84·3 to -37·1%)
Particulate matter pollution	Meningitis	0·8% (0·3–1·6%)	0·2 (0·1–0·4)	-84·3% (-94·5 to -60·3%)	0·2% (0·1–0·5%)	<0·1 (<0·1–<0·1)	-84·3% (-94·5 to -60·3%)
	Encephalitis	0·4% (0·2–0·8%)	0·5 (0·2–1)	-49·1% (-85·2 to 57%)	0·2% (0·1–0·3%)	<0·1 (<0·1–<0·1)	-49·1% (-85·2 to 57%)
	Stroke	27·5% (21·3–33%)	517·8 (390·5–652·4)	-47·3% (-59·8 to -29·8%)	24·6% (19–29·9%)	25·5 (19·1–32·5)	-40·9% (-55·5 to -18·9%)
	Ischaemic stroke	27% (20·8–32·7%)	348·1 (263·9–443·2)	-30·3% (-46·9 to -6·6%)	23·8% (18·5–29·1%)	18·2 (13·5–23·7)	-22·8% (-43·8 to 6·6%)
	Intracerebral haemorrhage	28·9% (22·8–34·8%)	146 (109·7–192·1)	-65·4% (-74·2 to -52·5%)	26·7% (20·8–32·3%)	6·4 (4·8–8·5)	-63% (-72·7 to -48·7%)
	Subarachnoid haemorrhage	26·5% (20·4–32·6%)	23·7 (14·2–39·9)	-61·6% (-77 to -26·4%)	26·7% (20·8–32·4%)	0·8 (0·4–1·4)	-62·1% (-80 to -25·2%)
Secondhand smoke	Stroke	2·6% (1·9–3·3%)	48·3 (34·6–64·6)	-44·4% (-58·6 to -23·2%)	2·3% (1·7–3%)	2·4 (1·7–3·3)	-36·4% (-53 to -10·9%)
	Ischaemic stroke	2·4% (1·7–3·2%)	31·1 (21·7–42·2)	-24·3% (-44·3 to 3·4%)	2·2% (1·6–2·9%)	1·7 (1·2–2·4)	-15·3% (-38·7 to 19·5%)
	Intracerebral haemorrhage	2·9% (2·1–3·8%)	14·8 (10·6–20·3)	-62·9% (-73·2 to -47·5%)	2·6% (1·9–3·4%)	0·6 (0·4–0·9)	-60·2% (-71·4 to -43·2%)

Table S16 Regional and national disabilities and deaths related to neurological conditions attributable to risk factors in North Africa and Middle East countries

**Location**<br>Risk factor (alphabetic order)	Cause	**DALYs (95% UI)**<br>PAF (%)<br>2019	Rate per 100,000<br>2019	Rate change (%)<br>From 1990 to 2019	**Deaths (95% UI)**<br>PAF (%)<br>2019	Rate per 100,000<br>2019	Rate change (%)<br>From 1990 to 2019
	Subarachnoid haemorrhage	2·7% (1·9–3·5%)	2·4 (1·3–4·1)	-59·1% (-75·7 to -20·6%)	2·7% (2–3·5%)	0·1 (0–0·1)	-58·9% (-78·1 to -20·3%)
Short gestation	Meningitis	3·1% (1·7–5·3%)	0·8 (0·4–1·4)	-79·1% (-89·5 to -55·3%)	0·9% (0·5–1·7%)	<0·1 (<0·1–<0·1)	-79·1% (-89·5 to -55·3%)
	Encephalitis	1·4% (0·7–2·7%)	1·8 (0·8–3·2)	-28·3% (-69·8 to 68·3%)	0·6% (0·3–1·1%)	<0·1 (<0·1–<0·1)	-28·3% (-69·8 to 68·3%)
Smoking	Stroke	6·9% (6–8%)	129·7 (106·9–160·6)	-63·5% (-73·8 to -47·9%)	5·2% (4·4–6·1%)	5·4 (4·4–6·6)	-59·5% (-71·5 to -41·6%)
	Ischaemic stroke	7% (6·1–8·1%)	90·7 (74·2–110·1)	-53·7% (-67·3 to -34·2%)	5·2% (4·4–6%)	4 (3·1–4·9)	-48·8% (-64·6 to -25·1%)
	Intracerebral haemorrhage	6·5% (5·3–8%)	33 (25·5–46·3)	-76·2% (-83·6 to -63·9%)	5·1% (4·1–6·3%)	1·2 (1–1·8)	-74·7% (-82·8 to -62·3%)
	Subarachnoid haemorrhage	6·8% (5·3–8·9%)	6·1 (3·9–11·6)	-71·3% (-83·7 to -38·9%)	6·1% (4·5–8%)	0·2 (0·1–0·4)	-72·4% (-85·8 to -39·2%)
	Neurological disorders*	2·3% (0·7–5%)	30·9 (8·5–76·9)	-22·5% (-39·9 to 1·7%)	3·7% (0·4–7·2%)	1·7 (0·1–5·3)	-23% (-63·8 to 15·9%)
	Alzheimer's disease and other dementias	8·2% (4·9–11·8%)	34·5 (12·7–81·7)	-21·6% (-37·8 to 3·2%)	6·8% (4–10%)	2 (0·4–5·6)	-19·5% (-39·5 to 16·6%)
	Parkinson's disease	-3·9% (-5·9–-2·1%)	-4·9 (-7·6–-2·3)	-9·3% (-41·2 to 40%)	-3·6% (-5·5–-1·9%)	-0·3 (-0·5–-0·1)	6·6% (-33·6 to 71·1%)
	Multiple sclerosis	7·9% (5·6–10·6%)	1·3 (0·8–1·9)	-0·3% (-40 to 37·9%)	7·7% (4·8–10·9%)	<0·1 (<0·1–<0·1)	-10% (-63·3 to 52·6%)
Tobacco	Stroke	9·2% (8·1–10·5%)	174·1 (144·5–213·1)	-59·6% (-70·3 to -43·4%)	7·4% (6·4–8·5%)	7·6 (6·2–9·3)	-54·2% (-66·6 to -35·7%)
	Ischaemic stroke	9·2% (8·1–10·4%)	119·2 (97·9–143·3)	-48·4% (-62·3 to -27·8%)	7·3% (6·2–8·3%)	5·6 (4·4–6·8)	-41·7% (-58·1 to -16%)
	Intracerebral haemorrhage	9·2% (8–10·8%)	46·7 (37·5–62·9)	-73·1% (-80·9 to -61·3%)	7·6% (6·5–9%)	1·8 (1·5–2·5)	-71% (-79·4 to -58·4%)
	Subarachnoid haemorrhage	9·3% (7·7–11·6%)	8·3 (5·3–15·2)	-68·6% (-81·8 to -34·8%)	8·6% (6·9–10·6%)	0·3 (0·1–0·5)	-69·2% (-83·9 to -34·7%)
	Neurological disorders*	2·3% (0·7–5%)	30·9 (8·5–76·9)	-22·5% (-39·9 to 1·7%)	3·7% (0·4–7·2%)	1·7 (0·1–5·3)	-23% (-63·8 to 15·9%)
	Alzheimer's disease and other dementias	8·2% (4·9–11·8%)	34·5 (12·7–81·7)	-21·6% (-37·8 to 3·2%)	6·8% (4–10%)	2 (0·4–5·6)	-19·5% (-39·5 to 16·6%)
	Parkinson's disease	-3·9% (-5·9–-2·1%)	-4·9 (-7·6–-2·3)	-9·3% (-41·2 to 40%)	-3·6% (-5·5–-1·9%)	-0·3 (-0·5–-0·1)	6·6% (-33·6 to 71·1%)
	Multiple sclerosis	7·9% (5·6–10·6%)	1·3 (0·8–1·9)	-0·3% (-40 to 37·9%)	7·7% (4·8–10·9%)	<0·1 (<0·1–<0·1)	-10% (-63·3 to 52·6%)
**Qatar**							
Air pollution	Meningitis	0·8% (0·5–1·4%)	0·2 (0·1–0·4)	-85% (-93·1 to -67·7%)	0·4% (0·2–0·7%)	<0·1 (<0·1–<0·1)	-85% (-93·1 to -67·7%)
	Encephalitis	0·4% (0·2–0·7%)	0·1 (0–0·1)	-41·4% (-77 to 37%)	0·2% (0·1–0·3%)	<0·1 (<0·1–<0·1)	-41·5% (-77 to 37%)
	Stroke	34·4% (30·7–37·7%)	311·4 (248–397·1)	-47·6% (-59·5 to -32·5%)	29·2% (25·9–32·5%)	15·5 (11·9–20·7)	-41·7% (-57·7 to -21·6%)
	Ischaemic stroke	32·5% (28·7–35·9%)	187·7 (150·1–237·5)	-39·3% (-52·9 to -23·6%)	27·8% (24·4–31·2%)	10·2 (7·9–13·7)	-33·5% (-51·6 to -10·4%)
	Intracerebral haemorrhage	37% (32·9–40·3%)	90·2 (70·3–119·3)	-57·9% (-69·4 to -43%)	31·9% (28·3–35·3%)	4 (3·1–5·5)	-53·3% (-66·8 to -34·4%)
	Subarachnoid haemorrhage	40·1% (35·6–43·7%)	33·5 (24·6–44·2)	-53·2% (-72·4 to -24·7%)	34·8% (30·8–38·6%)	1·2 (0·9–1·6)	-52·8% (-73·5 to -15·6%)
Alcohol use	Stroke	0·3% (-0·1–0·7%)	2·5 (-0·8–6·3)	-74·3% (-116·5 to -43·5%)	0·1% (-0·2–0·3%)	0 (-0·1–0·2)	-83·9% (-269·6 to 11%)
	Ischaemic stroke	-0·1% (-0·5–0·2%)	-0·8 (-3–1·4)	50·8% (-379·6 to 392·6%)	-0·2% (-0·5–0·1%)	-0·1 (-0·2–0)	-21·3% (-290 to 289·2%)
	Intracerebral haemorrhage	1·4% (0·5–2·4%)	3·3 (1·1–6·1)	-67·5% (-83·7 to -46·2%)	0·8% (0·2–1·6%)	0·1 (0–0·2)	-67·2% (-85·7 to -42·9%)
	Neurological disorders*	0·2% (0·1–0·5%)	3·1 (1–6·7)	-40·8% (-79·2 to 69·9%)	0% (0–0·1%)	<0·1 (<0·1–<0·1)	-51·6% (-69·1 to -21·1%)
	Idiopathic epilepsy	2·5% (1·4–3·8%)	3·1 (1–6·7)	-40·8% (-79·2 to 69·9%)	2·5% (1·4–3·8%)	<0·1 (<0·1–<0·1)	-51·6% (-69·1 to -21·1%)

Table S16 Regional and national disabilities and deaths related to neurological conditions attributable to risk factors in North Africa and Middle East countries

**Location** Risk factor (alphabetic order)	Cause	**DALYs (95% UI)** PAF (%) 2019	Rate per 100,000 2019	Rate change (%) From 1990 to 2019	**Deaths (95% UI)** PAF (%) 2019	Rate per 100,000 2019	Rate change (%) From 1990 to 2019
All risk factors	Meningitis	3·4% (2·2–5·2%)	0·9 (0·6–1·3)	-84·9% (-92·6 to -71·1%)	1·7% (1–2·6%)	<0·1 (<0·1–<0·1)	-84·9% (-92·6 to -71·1%)
	Encephalitis	1·6% (0·8–2·5%)	0·2 (0·1–0·4)	-41·6% (-76·8 to 31·2%)	0·7% (0·4–1·3%)	<0·1 (<0·1–<0·1)	-41·6% (-76·8 to 31·2%)
	Stroke	86·5% (82·3–90·5%)	782·5 (640·1–988·2)	-44·3% (-57·4 to -28·6%)	84·3% (78·1–90·7%)	44·6 (35·3–57·7)	-37% (-54·1 to -16·6%)
	Ischaemic stroke	86·4% (80·5–92·4%)	498·8 (405·5–624·4)	-35·9% (-50·1 to -20%)	84% (76–92·4%)	31 (24·2–41)	-29·1% (-48·3 to -5·7%)
	Intracerebral haemorrhage	86·8% (82·5–90·6%)	211·6 (166·8–276·7)	-55·6% (-67·6 to -40·5%)	85% (78·3–91·1%)	10·7 (8·1–14·2)	-49·5% (-64·3 to -29·7%)
	Subarachnoid haemorrhage	86·2% (81·8–89·9%)	72·1 (53·6–94·6)	-52·2% (-71·5 to -22·9%)	84·4% (77·4–90·3%)	2·9 (2·1–4·1)	-50·8% (-72 to -12·5%)
	Neurological disorders*	15·8% (6·2–31·4%)	213·7 (75·8–551·9)	36·4% (7·6 to 77·8%)	31·2% (12·3–51·8%)	13·8 (2·4–42·6)	45·8% (5·8 to 109·5%)
	Alzheimer's disease and other dementias	53·5% (34·4–71·6%)	218 (82·1–556·1)	37% (7·5 to 77·7%)	51·6% (32·3–70·3%)	14·4 (3·2–43·5)	45·1% (6·8 to 104·5%)
	Parkinson's disease	-6·2% (-9·4–-3·2%)	-9·1 (-15·3–-4·5)	1·3% (-32·7 to 50·8%)	-5·5% (-8·5–-2·8%)	-0·6 (-1–-0·3)	25% (-19·8 to 91·6%)
	Idiopathic epilepsy	2·5% (1·4–3·8%)	3·1 (1–6·7)	-40·8% (-79·2 to 69·9%)	2·5% (1·4–3·8%)	<0·1 (<0·1–<0·1)	-51·6% (-69·1 to -21·1%)
	Multiple sclerosis	9·8% (7–12·7%)	1·6 (1–2·4)	34·6% (-2·4 to 80·1%)	9·7% (6·7–13·2%)	<0·1 (<0·1–<0·1)	-18% (-58·3 to 36·4%)
Ambient particulate matter pollution	Meningitis	0·8% (0·5–1·4%)	0·2 (0·1–0·4)	-84·9% (-93·1 to -67·5%)	0·4% (0·2–0·7%)	<0·1 (<0·1–<0·1)	-84·9% (-93·1 to -67·5%)
	Encephalitis	0·4% (0·2–0·7%)	0·1 (0–0·1)	-41·2% (-76·9 to 37·3%)	0·2% (0·1–0·3%)	<0·1 (<0·1–<0·1)	-41·2% (-76·9 to 37·3%)
	Stroke	34·4% (30·7–37·7%)	311·4 (248–397)	-47·4% (-59·4 to -32·2%)	29·2% (25·9–32·5%)	15·5 (11·9–20·7)	-41·5% (-57·6 to -21·2%)
	Ischaemic stroke	32·5% (28·7–35·9%)	187·7 (150·1–237·5)	-39% (-52·7 to -23·3%)	27·8% (24·4–31·2%)	10·2 (7·9–13·7)	-33·2% (-51·5 to -9·9%)
	Intracerebral haemorrhage	37% (32·9–40·3%)	90·2 (70·3–119·3)	-57·7% (-69·3 to -42·8%)	31·9% (28·3–35·3%)	4 (3·1–5·5)	-53·1% (-66·7 to -34·2%)
	Subarachnoid haemorrhage	40% (35·6–43·7%)	33·5 (24·6–44·2)	-53% (-72·3 to -24·4%)	34·8% (30·8–38·6%)	1·2 (0·9–1·6)	-52·6% (-73·4 to -15·3%)
Behavioural risk factors‡	Meningitis	3·3% (2·1–5%)	0·8 (0·5–1·3)	-84·8% (-92·5 to -70·8%)	1·6% (1–2·5%)	<0·1 (<0·1–<0·1)	-84·8% (-92·5 to -70·8%)
	Encephalitis	1·5% (0·8–2·4%)	0·2 (0·1–0·4)	-41·1% (-76·5 to 32·8%)	0·7% (0·3–1·3%)	<0·1 (<0·1–<0·1)	-41·1% (-76·5 to 32·8%)
	Stroke	27·4% (22·2–33·4%)	247·8 (179·5–327·6)	-47·9% (-60·7 to -33·5%)	23·8% (17·8–30·6%)	12·6 (8·5–17·7)	-40·4% (-57·6 to -20·5%)
	Ischaemic stroke	28·9% (21·4–37·3%)	167·1 (115·5–234·2)	-39·6% (-53·6 to -24·5%)	25·9% (17·7–34·6%)	9·6 (6·2–14·1)	-32·1% (-51·1 to -8·9%)
	Intracerebral haemorrhage	24·2% (19·3–29·9%)	58·9 (41·7–81·3)	-61% (-72·8 to -46·6%)	18·6% (14·1–24%)	2·3 (1·6–3·3)	-57·5% (-71 to -39·5%)
	Subarachnoid haemorrhage	26% (20·7–32·4%)	21·8 (14·6–29·9)	-54·9% (-74·6 to -26·5%)	20·2% (15·4–26·3%)	0·7 (0·5–1)	-54·8% (-75·9 to -20·1%)
	Neurological disorders*	4·2% (1·5–8·8%)	56·5 (17–141·4)	40·3% (3·3 to 85·9%)	6·6% (0·5–12·9%)	3·1 (0·1–10·3)	77·7% (-1·1 to 249·2%)
	Alzheimer's disease and other dementias	15% (9–21%)	60·9 (22·1–145·6)	42·2% (4·6 to 85·2%)	13·1% (7·8–18·9%)	3·7 (0·8–10·9)	68·1% (17·4 to 134·8%)
	Parkinson's disease	-6·2% (-9·4–-3·2%)	-9·1 (-15·3–-4·5)	1·3% (-32·7 to 50·8%)	-5·5% (-8·5–-2·8%)	-0·6 (-1–-0·3)	25% (-19·8 to 91·6%)
	Idiopathic epilepsy	2·5% (1·4–3·8%)	3·1 (1–6·7)	-40·8% (-79·2 to 69·9%)	2·5% (1·4–3·8%)	<0·1 (<0·1–<0·1)	-51·6% (-69·1 to -21·1%)
	Multiple sclerosis	9·8% (7–12·7%)	1·6 (1–2·4)	34·6% (-2·4 to 80·1%)	9·7% (6·7–13·2%)	<0·1 (<0·1–<0·1)	-18% (-58·3 to 36·4%)
Child and maternal malnutrition	Meningitis	3·3% (2·1–5%)	0·8 (0·5–1·3)	-84·8% (-92·5 to -70·8%)	1·6% (1–2·5%)	<0·1 (<0·1–<0·1)	-84·8% (-92·5 to -70·8%)
	Encephalitis	1·5% (0·8–2·4%)	0·2 (0·1–0·4)	-41·1% (-76·5 to 32·8%)	0·7% (0·3–1·3%)	<0·1 (<0·1–<0·1)	-41·1% (-76·5 to 32·8%)
Diet high in red meat	Stroke	5·4% (2·6–7·5%)	48·6 (23·2–71·8)	-47·4% (-62·3 to -29·3%)	3·9% (1·8–5·7%)	2·1 (0·9–3·3)	-42·3% (-61·4 to -17·5%)

Table S16 Regional and national disabilities and deaths related to neurological conditions attributable to risk factors in North Africa and Middle East countries

**Location**<br>Risk factor (alphabetic order)	Cause	**DALYs (95% UI)**<br>PAF (%)<br>2019	Rate per 100,000<br>2019	Rate change (%)<br>From 1990 to 2019	**Deaths (95% UI)**<br>PAF (%)<br>2019	Rate per 100,000<br>2019	Rate change (%)<br>From 1990 to 2019
	Ischaemic stroke	4·5% (1·3–6·7%)	26·1 (7·2–41·4)	-38·1% (-55 to -17·8%)	3·3% (0·9–5·1%)	1·2 (0·3–2)	-32·6% (-55·7 to -1·6%)
	Intracerebral haemorrhage	6·6% (2·1–10·1%)	16·1 (4·7–26·8)	-56·7% (-69·5 to -37%)	5·2% (1·5–8·1%)	0·7 (0·2–1·1)	-52·3% (-67·6 to -29·9%)
	Subarachnoid haemorrhage	7·6% (2·3–11·6%)	6·4 (1·8–10·5)	-51·2% (-71·8 to -17·1%)	5·9% (1·7–9·2%)	0·2 (0·1–0·3)	-51·6% (-73·4 to -12·7%)
Diet high in sodium	Stroke	1·9% (0·3–7·3%)	17 (2·2–65·9)	-46% (-66 to -4%)	1·5% (0·3–5·7%)	0·8 (0·1–3·1)	-39·3% (-65·5 to 15·8%)
	Ischaemic stroke	1·6% (0·2–6·3%)	9·5 (1·4–36·6)	-37·9% (-61·5 to 11·6%)	1·3% (0·3–5·2%)	0·5 (0·1–2)	-30% (-60·5 to 34·5%)
	Intracerebral haemorrhage	2·3% (0·3–8·8%)	5·5 (0·6–22·2)	-55·1% (-73·4 to -17·9%)	1·9% (0·3–7·5%)	0·2 (0–1)	-50·1% (-72·4 to -3·9%)
	Subarachnoid haemorrhage	2·4% (0·3–9·2%)	2 (0·2–8·2)	-49·5% (-75·7 to 5·8%)	2% (0·3–8%)	0·1 (0–0·3)	-48·9% (-76·8 to 10·7%)
Diet low in fibre	Stroke	0·3% (0·2–0·6%)	2·8 (1·7–5·2)	-61·5% (-77 to -33·4%)	0·3% (0·2–0·6%)	0·2 (0·1–0·3)	-54·2% (-74·4 to -16·1%)
	Ischaemic stroke	0·3% (0·2–0·6%)	1·8 (1·1–3·3)	-54·2% (-72 to -24·5%)	0·3% (0·2–0·6%)	0·1 (0·1–0·2)	-47·3% (-71·9 to -4·1%)
	Intracerebral haemorrhage	0·3% (0·2–0·6%)	0·8 (0·5–1·5)	-69·9% (-82·5 to -46·8%)	0·3% (0·2–0·6%)	<0·1 (<0·1–0·1)	-64% (-80·4 to -33·1%)
	Subarachnoid haemorrhage	0·3% (0·2–0·7%)	0·3 (0·1–0·6)	-68·9% (-85·1 to -33·6%)	0·3% (0·2–0·6%)	<0·1 (<0·1–<0·1)	-66·1% (-83·1 to -25·5%)
Diet low in fruits	Stroke	1·2% (0·6–2·1%)	10·8 (5·2–19·3)	-62·8% (-78·3 to -36·6%)	0·9% (0·4–1·6%)	0·5 (0·2–0·9)	-60·1% (-80·2 to -23%)
	Ischaemic stroke	0·9% (0·3–1·8%)	4·9 (1·7–10·4)	-54·6% (-73·6 to -21·1%)	0·7% (0·3–1·5%)	0·2 (0·1–0·5)	-51·6% (-77·5 to 0·9%)
	Intracerebral haemorrhage	1·7% (0·8–3%)	4·1 (1·7–7·6)	-68·9% (-83·3 to -44·9%)	1·3% (0·6–2·4%)	0·2 (0·1–0·3)	-66·8% (-83·1 to -36·3%)
	Subarachnoid haemorrhage	2·1% (0·9–3·7%)	1·7 (0·7–3·2)	-64·6% (-82·7 to -30·3%)	1·5% (0·7–2·8%)	0·1 (0–0·1)	-66·3% (-84·5 to -28·7%)
Diet low in vegetables	Stroke	0·2% (0·2–0·4%)	2·2 (1·6–3·6)	-68·2% (-83·4 to -40%)	0·3% (0·2–0·4%)	0·1 (0·1–0·2)	-63·1% (-81·6 to -28·6%)
	Ischaemic stroke	0·3% (0·2–0·4%)	1·4 (1–2·4)	-62·2% (-79 to -28·2%)	0·3% (0·2–0·4%)	0·1 (0·1–0·2)	-57·3% (-77·9 to -17·8%)
	Intracerebral haemorrhage	0·2% (0·2–0·4%)	0·6 (0·4–1·1)	-75·6% (-89·4 to -46·1%)	0·3% (0·2–0·5%)	<0·1 (<0·1–0·1)	-71·7% (-87·9 to -36·1%)
	Subarachnoid haemorrhage	0·2% (0·2–0·5%)	0·2 (0·1–0·4)	-74·8% (-90·7 to -34·2%)	0·3% (0·2–0·5%)	<0·1 (<0·1–<0·1)	-73·1% (-90·1 to -23·4%)
Diet low in whole grains	Stroke	3·5% (1·1–5%)	31·6 (9·3–47·8)	-43·8% (-58·3 to -30·2%)	3·2% (1·1–4·5%)	1·7 (0·5–2·6)	-38% (-56·9 to -17·3%)
	Ischaemic stroke	5·5% (1·7–7·7%)	31·6 (9·3–47·8)	-43·8% (-58·3 to -30·2%)	4·6% (1·5–6·5%)	1·7 (0·5–2·6)	-38% (-56·9 to -17·3%)
Dietary risks	Stroke	11·9% (7·9–17·1%)	107·2 (65·9–161·1)	-49·2% (-61·9 to -34·5%)	9·7% (6·4–13·9%)	5·1 (3·1–8)	-43·7% (-60·8 to -23·9%)
	Ischaemic stroke	12·3% (7·3–17·2%)	71·1 (40·1–103·3)	-42·6% (-56·2 to -28·1%)	10·1% (5·9–14·1%)	3·7 (2–5·6)	-37·1% (-56·3 to -14·6%)
	Intracerebral haemorrhage	10·7% (5–17·3%)	26 (11·9–44·4)	-59·7% (-72·4 to -45%)	8·7% (4–14·6%)	1·1 (0·5–1·9)	-55·7% (-70·9 to -37·3%)
	Subarachnoid haemorrhage	12·1% (5·9–19%)	10·1 (4·7–16·7)	-54·9% (-74·6 to -26·1%)	9·6% (4·5–15·9%)	0·3 (0·1–0·6)	-55·3% (-76·1 to -19·8%)
Environmental/occupational risk factors§	Meningitis	0·8% (0·5–1·4%)	0·2 (0·1–0·4)	-85% (-93·1 to -67·7%)	0·4% (0·2–0·7%)	<0·1 (<0·1–<0·1)	-85% (-93·1 to -67·7%)
	Encephalitis	0·4% (0·2–0·7%)	0·1 (0–0·1)	-41·4% (-77 to 37%)	0·2% (0·1–0·3%)	<0·1 (<0·1–<0·1)	-41·5% (-77 to 37%)
	Stroke	40·7% (34·8–45·5%)	368·3 (286·9–481·2)	-47·1% (-59·7 to -31·1%)	37·4% (30·3–43·6%)	19·8 (14·7–26·9)	-39·8% (-56·9 to -17·7%)
	Ischaemic stroke	38·7% (33·3–43·6%)	223·5 (175·4–292·3)	-38·4% (-52·5 to -21·2%)	36% (28·8–42·3%)	13·3 (9·8–18·7)	-31·3% (-50·8 to -5·1%)
	Intracerebral haemorrhage	43·6% (37·3–48·6%)	106·2 (80·2–142·6)	-57·6% (-69·5 to -41·4%)	39·9% (33·1–45·8%)	5 (3·7–6·9)	-51·8% (-66·2 to -31·5%)
	Subarachnoid haemorrhage	46·2% (40·1–51·7%)	38·6 (27·8–51·8)	-53·6% (-72·6 to -24·5%)	42·4% (35·8–48·2%)	1·5 (1–2·1)	-52% (-73·3 to -14·6%)
High body-mass index	Stroke	35·9% (26·2–44·8%)	324·2 (225·4–435·2)	-42·6% (-56·6 to -24·5%)	21·6% (13·3–30·3%)	11·4 (6·6–17·4)	-38·9% (-57·4 to -13·9%)
	Ischaemic stroke	27·9% (18·8–37·6%)	160·9 (105·2–229)	-28·6% (-46·3 to -6·7%)	16·8% (8–27%)	6·2 (2·8–10·5)	-24·1% (-52·4 to 9·6%)

Table S16 Regional and national disabilities and deaths related to neurological conditions attributable to risk factors in North Africa and Middle East countries

Location<br>Risk factor (alphabetic order)	Cause	DALYs (95% UI)<br>PAF (%)<br>2019	 Rate per 100,000<br>2019	 Rate change (%)<br>From 1990 to 2019	Deaths (95% UI)<br>PAF (%)<br>2019	 Rate per 100,000<br>2019	 Rate change (%)<br>From 1990 to 2019
	Intracerebral haemorrhage	47·9% (36–59%)	116·7 (79·4–160·3)	-53·6% (-66·4 to -35·8%)	31·2% (18·2–45·6%)	3·9 (2·2–6·1)	-50·9% (-68 to -29·3%)
	Subarachnoid haemorrhage	55·8% (43·4–66·4%)	46·7 (33–64·3)	-47·3% (-68·7 to -17%)	38·4% (25·8–51·8%)	1·3 (0·8–2·1)	-48% (-71·1 to -11%)
	Neurological disorders*	8·3% (2·4–18·2%)	112·2 (29·3–285·8)	41·8% (-13·2 to 138·1%)	17% (5–32·3%)	7·4 (1·2–23·8)	44·9% (-22·1 to 181·1%)
	Alzheimer's disease and other dementias	27·5% (11·3–45·9%)	112·2 (29·3–285·8)	41·8% (-13·2 to 138·1%)	26·6% (10·3–45·5%)	7·4 (1·2–23·8)	44·9% (-22·1 to 181·1%)
High fasting plasma glucose	Stroke	29·7% (19·5–45·8%)	269·3 (169·1–439·4)	-45·8% (-61·2 to -25·7%)	28·4% (16·4–52%)	15·1 (8·2–28·9)	-42·3% (-61·4 to -16·6%)
	Ischaemic stroke	30·1% (16·9–55·6%)	174·2 (95·9–335·9)	-41% (-57·4 to -17·7%)	28·4% (13·4–62·4%)	10·5 (4·8–23·7)	-36·5% (-56·8 to -7·5%)
	Intracerebral haemorrhage	29·3% (19·2–42·8%)	71·3 (44·1–114)	-54·6% (-68·8 to -33·4%)	28·3% (16·9–47·7%)	3·6 (2–6·4)	-52·7% (-68·9 to -29%)
	Subarachnoid haemorrhage	28·5% (18·2–40·9%)	23·8 (14·1–36·9)	-46·7% (-69·9 to -5·7%)	28·8% (18·7–44·8%)	1 (0·6–1·8)	-51·6% (-73·7 to -8·3%)
	Neurological disorders*	7·4% (1·5–19·2%)	100·4 (18·8–302·8)	56·9% (20·5 to 124·5%)	15·5% (3·4–33·9%)	6·8 (0·8–23·7)	64·2% (17·3 to 152·2%)
	Alzheimer's disease and other dementias	24·6% (6·6–46·8%)	100·4 (18·8–302·8)	56·9% (20·5 to 124·5%)	24·2% (6·4–46·4%)	6·8 (0·8–23·7)	64·2% (17·3 to 152·2%)
High LDL cholesterol	Stroke	13·2% (6–25·3%)	119·4 (51·4–239·5)	-36·5% (-53·2 to -19·2%)	12·5% (2·9–29·5%)	6·6 (1·4–16·4)	-29·6% (-55·3 to -4·3%)
	Ischaemic stroke	20·7% (9·5–39·1%)	119·4 (51·4–239·5)	-36·5% (-53·2 to -19·2%)	18% (4·2–42·2%)	6·6 (1·4–16·4)	-29·6% (-55·3 to -4·3%)
High systolic blood pressure	Stroke	51·4% (42·9–59·9%)	465 (351·9–607)	-44·5% (-58 to -27·8%)	48·2% (37·1–60%)	25·5 (18–36)	-37·8% (-55·4 to -15·9%)
	Ischaemic stroke	48·6% (37·6–60·2%)	280·6 (203·6–378·1)	-35·8% (-50·9 to -17·4%)	45·1% (31·2–61·7%)	16·6 (11–25)	-29·4% (-49·9 to -2·9%)
	Intracerebral haemorrhage	56·2% (46–66·6%)	137·1 (98·8–182·8)	-54·9% (-68·2 to -38·7%)	55·1% (41–69·4%)	6·9 (4·6–9·7)	-49% (-65·2 to -28·8%)
	Subarachnoid haemorrhage	56·5% (47–66·3%)	47·3 (32·9–65·5)	-51·3% (-71·7 to -20·1%)	56·4% (43·5–69·3%)	2 (1·3–2·9)	-49·6% (-72·3 to -7·9%)
High temperature	Stroke	5% (1·8–8·2%)	45 (15·8–81·7)	-42·4% (-72·1 to -20·9%)	6·2% (2·2–10·2%)	3·3 (1·2–6·2)	-29·6% (-65·3 to -2·1%)
	Ischaemic stroke	4·7% (1·7–7·7%)	27 (9·5–51·2)	-31·9% (-67 to -4·2%)	6·2% (2·2–10·2%)	2·3 (0·8–4·3)	-20·7% (-61·3 to 10·6%)
	Intracerebral haemorrhage	5·5% (2–9%)	13·4 (4·7–24·6)	-53·8% (-78·5 to -32·5%)	6·2% (2·2–10·2%)	0·8 (0·3–1·4)	-43·7% (-72·9 to -18·5%)
	Subarachnoid haemorrhage	5·5% (1·9–9%)	4·6 (1·5–8·7)	-51·6% (-80·4 to -18%)	6·2% (2·2–10·2%)	0·2 (0·1–0·4)	-45·2% (-76·8 to 3·2%)
Household air pollution from solid fuels	Meningitis	0% (0–0%)	<0·1 (<0·1–<0·1)	-99·7% (-99·9 to -99·1%)	0% (0–0%)	<0·1 (<0·1–<0·1)	-99·7% (-99·9 to -99·1%)
	Encephalitis	0% (0–0%)	<0·1 (<0·1–<0·1)	-98·9% (-99·8 to -95·9%)	0% (0–0%)	<0·1 (<0·1–<0·1)	-98·9% (-99·8 to -95·9%)
	Stroke	0% (0–0%)	<0·1 (<0·1–0·1)	-99·1% (-99·7 to -97·5%)	0% (0–0%)	<0·1 (<0·1–<0·1)	-99% (-99·7 to -97·3%)
	Ischaemic stroke	0% (0–0%)	<0·1 (<0·1–<0·1)	-98·9% (-99·7 to -97·1%)	0% (0–0%)	<0·1 (<0·1–<0·1)	-98·9% (-99·7 to -97%)
	Intracerebral haemorrhage	0% (0–0%)	<0·1 (<0·1–<0·1)	-99·3% (-99·8 to -98%)	0% (0–0%)	<0·1 (<0·1–<0·1)	-99·2% (-99·8 to -97·8%)
	Subarachnoid haemorrhage	0% (0–0%)	<0·1 (<0·1–<0·1)	-99·2% (-99·8 to -97·5%)	0% (0–0%)	<0·1 (<0·1–<0·1)	-99·2% (-99·8 to -97·5%)
Kidney dysfunction	Stroke	11·1% (7·8–14·1%)	100·2 (65·7–137·5)	-27·5% (-46·5 to -4·4%)	10·1% (5·6–14·4%)	5·4 (2·8–8·1)	-21·5% (-48·7 to 9·9%)
	Ischaemic stroke	12·4% (7·6–16·7%)	71·7 (41–102·6)	-20·7% (-41·7 to 3·5%)	10% (3·9–15·7%)	3·7 (1·3–6·1)	-16·5% (-50·1 to 17·9%)
	Intracerebral haemorrhage	11·7% (8·6–14·7%)	28·5 (19·3–40·2)	-40·3% (-57·9 to -16·1%)	13·3% (8·5–17·7%)	1·7 (1–2·5)	-30·6% (-53·1 to 2·1%)
Lead exposure	Stroke	1·8% (0·4–3·5%)	16·2 (3·3–33·3)	-47·6% (-61·2 to -3·7%)	1·9% (0·5–3·6%)	1 (0·3–2)	-31·8% (-50·8 to 36·5%)
	Ischaemic stroke	1·8% (0·4–3·5%)	10·2 (2·3–20·7)	-35·3% (-51·9 to 15·6%)	1·8% (0·5–3·6%)	0·7 (0·2–1·4)	-19% (-43·8 to 63·8%)

Table S16 Regional and national disabilities and deaths related to neurological conditions attributable to risk factors in North Africa and Middle East countries

**Location**<br>Risk factor (alphabetic order)	Cause	**DALYs (95% UI)**<br>PAF (%)<br>2019	Rate per 100,000<br>2019	Rate change (%)<br>From 1990 to 2019	**Deaths (95% UI)**<br>PAF (%)<br>2019	Rate per 100,000<br>2019	Rate change (%)<br>From 1990 to 2019
	Intracerebral haemorrhage	1·9% (0·3–3·9%)	4·6 (0·7–10·2)	-60·3% (-73·2 to -29·2%)	2·2% (0·5–4·4%)	0·3 (0·1–0·6)	-46·9% (-64·7 to 10·2%)
	Subarachnoid haemorrhage	1·7% (0·2–3·8%)	1·4 (0·2–3·5)	-60·8% (-79·1 to -16·6%)	2% (0·4–4·2%)	0·1 (0–0·2)	-51% (-74·3 to 16·8%)
Low birth weight	Meningitis	3·1% (2–4·7%)	0·8 (0·5–1·2)	-85% (-92·6 to -70·8%)	1·5% (0·9–2·3%)	<0·1 (<0·1–<0·1)	-85% (-92·6 to -70·8%)
	Encephalitis	1·4% (0·8–2·3%)	0·2 (0·1–0·3)	-41·7% (-76·9 to 32·2%)	0·7% (0·3–1·2%)	<0·1 (<0·1–<0·1)	-41·7% (-77 to 32·2%)
Low birth weight and short gestation	Meningitis	3·3% (2·1–5%)	0·8 (0·5–1·3)	-84·8% (-92·5 to -70·8%)	1·6% (1–2·5%)	<0·1 (<0·1–<0·1)	-84·8% (-92·5 to -70·8%)
	Encephalitis	1·5% (0·8–2·4%)	0·2 (0·1–0·4)	-41·1% (-76·5 to 32·8%)	0·7% (0·3–1·3%)	<0·1 (<0·1–<0·1)	-41·1% (-76·5 to 32·8%)
Low physical activity	Stroke	6·4% (1·5–13%)	57·8 (13·5–118·3)	-27·1% (-44·5 to -1·1%)	8% (2–15·2%)	4·2 (1·1–8·3)	-21% (-43·3 to 12%)
	Ischaemic stroke	10% (2·4–20·2%)	57·8 (13·5–118·3)	-27·1% (-44·5 to -1·1%)	11·5% (2·8–21·8%)	4·2 (1·1–8·3)	-21% (-43·3 to 12%)
Low temperature	Stroke	3% (-3·9–9%)	27·3 (-35·9–85·4)		3·8% (-4·8–11·2%)	2 (-2·6–6·2)	
	Ischaemic stroke	2·8% (-3·7–8·5%)	16·4 (-21·5–51)		3·8% (-4·8–11·2%)	1·4 (-1·8–4·3)	
	Intracerebral haemorrhage	3·3% (-4·2–9·9%)	8·1 (-10·8–26·3)		3·8% (-4·8–11·2%)	0·5 (-0·6–1·5)	
	Subarachnoid haemorrhage	3·3% (-4·2–9·9%)	2·8 (-3·5–8·8)		3·8% (-4·8–11·2%)	0·1 (-0·2–0·4)	
Metabolic risk factors†	Stroke	74·2% (66·2–81·9%)	671·1 (536·5–855·7)	-43·6% (-56·7 to -27·6%)	70% (58·6–81·6%)	37 (27·7–49·7)	-36·9% (-54·2 to -16·1%)
	Ischaemic stroke	73·4% (63–84·8%)	423·7 (331·3–545·4)	-35·5% (-50·1 to -18·8%)	68·8% (54·4–84·8%)	25·4 (18·2–35·4)	-29·2% (-48·6 to -5·7%)
	Intracerebral haemorrhage	76% (68·3–82·7%)	185·3 (143·1–244·6)	-54·4% (-67·2 to -38·7%)	73·2% (62·1–83·1%)	9·2 (6·8–12·4)	-48·7% (-63·9 to -29·4%)
	Subarachnoid haemorrhage	74·3% (66·7–81·1%)	62·1 (45·1–83·9)	-50·6% (-70·8 to -19·8%)	71·2% (59·4–81%)	2·5 (1·7–3·6)	-50% (-72·2 to -9·6%)
	Neurological disorders*	13·7% (4·8–28·4%)	184·7 (58·7–476·6)	41·8% (7·2 to 96·3%)	28·3% (10·9–49%)	12·4 (2·3–38)	46·6% (2·2 to 120·2%)
	Alzheimer's disease and other dementias	45·3% (22·3–66·2%)	184·7 (58·7–476·6)	41·8% (7·2 to 96·3%)	44·3% (22·1–65·8%)	12·4 (2·3–38)	46·6% (2·2 to 120·2%)
Non-optimal temperature	Stroke	7·8% (0·5–14·3%)	70·6 (4·5–138·3)	-46·6% (-99·5 to -0·4%)	9·8% (0·7–17·9%)	5·2 (0·3–10·2)	-34·6% (-99·4 to 20·9%)
	Ischaemic stroke	7·3% (0·5–13·6%)	42·4 (2·7–83·9)	-36·8% (-99·4 to 15·6%)	9·8% (0·7–17·9%)	3·6 (0·2–7·1)	-26·4% (-99·3 to 39·3%)
	Intracerebral haemorrhage	8·6% (0·6–15·9%)	21 (1·4–41·1)	-57·1% (-99·6 to -17%)	9·8% (0·7–17·9%)	1·2 (0·1–2·4)	-47·7% (-99·5 to -1·6%)
	Subarachnoid haemorrhage	8·6% (0·6–15·7%)	7·2 (0·4–14·6)	-55·2% (-99·6 to -8·2%)	9·8% (0·7–17·9%)	0·3 (0–0·7)	-49·3% (-99·7 to 8·6%)
Other environmental risks	Stroke	1·8% (0·4–3·5%)	16·2 (3·3–33·3)	-47·6% (-61·2 to -3·7%)	1·9% (0·5–3·6%)	1 (0·3–2)	-31·8% (-50·8 to 36·5%)
	Ischaemic stroke	1·8% (0·4–3·5%)	10·2 (2·3–20·7)	-35·3% (-51·9 to 15·6%)	1·8% (0·5–3·6%)	0·7 (0·2–1·4)	-19% (-43·8 to 63·8%)
	Intracerebral haemorrhage	1·9% (0·3–3·9%)	4·6 (0·7–10·2)	-60·3% (-73·2 to -29·2%)	2·2% (0·5–4·4%)	0·3 (0·1–0·6)	-46·9% (-64·7 to 10·2%)
	Subarachnoid haemorrhage	1·7% (0·2–3·8%)	1·4 (0·2–3·5)	-60·8% (-79·1 to -16·6%)	2% (0·4–4·2%)	0·1 (0–0·2)	-51% (-74·3 to 16·8%)
Particulate matter pollution	Meningitis	0·8% (0·5–1·4%)	0·2 (0·1–0·4)	-85% (-93·1 to -67·7%)	0·4% (0·2–0·7%)	<0·1 (<0·1–<0·1)	-85% (-93·1 to -67·7%)
	Encephalitis	0·4% (0·2–0·7%)	0·1 (0–0·1)	-41·4% (-77 to 37%)	0·2% (0·1–0·3%)	<0·1 (<0·1–<0·1)	-41·5% (-77 to 37%)
	Stroke	34·4% (30·7–37·7%)	311·4 (248–397·1)	-47·6% (-59·5 to -32·5%)	29·2% (25·9–32·5%)	15·5 (11·9–20·7)	-41·7% (-57·7 to -21·6%)
	Ischaemic stroke	32·5% (28·7–35·9%)	187·7 (150·1–237·5)	-39·3% (-52·9 to -23·6%)	27·8% (24·4–31·2%)	10·2 (7·9–13·7)	-33·5% (-51·6 to -10·4%)
	Intracerebral haemorrhage	37% (32·9–40·3%)	90·2 (70·3–119·3)	-57·9% (-69·4 to -43%)	31·9% (28·3–35·3%)	4 (3·1–5·5)	-53·3% (-66·8 to -34·4%)
	Subarachnoid haemorrhage	40·1% (35·6–43·7%)	33·5 (24·6–44·2)	-53·2% (-72·4 to -24·7%)	34·8% (30·8–38·6%)	1·2 (0·9–1·6)	-52·8% (-73·5 to -15·6%)

Table S16 Regional and national disabilities and deaths related to neurological conditions attributable to risk factors in North Africa and Middle East countries

Location<br>Risk factor (alphabetic order)	Cause	DALYs (95% UI)<br>PAF (%)<br>2019	Rate per 100,000<br>2019	Rate change (%)<br>From 1990 to 2019	Deaths (95% UI)<br>PAF (%)<br>2019	Rate per 100,000<br>2019	Rate change (%)<br>From 1990 to 2019
Secondhand smoke	Stroke	3% (2·3–3·7%)	27·2 (18·9–36·6)	-57·1% (-68·8 to -42·8%)	2·6% (1·9–3·3%)	1·4 (0·9–1·9)	-50·6% (-64·8 to -30·8%)
	Ischaemic stroke	2·6% (2–3·3%)	15·2 (10·4–20·7)	-49·3% (-62·2 to -31·6%)	2·4% (1·7–3·1%)	0·9 (0·6–1·3)	-42·7% (-59·7 to -19·2%)
	Intracerebral haemorrhage	3·5% (2·7–4·4%)	8·6 (5·8–12)	-65·4% (-75·6 to -51·6%)	3% (2·2–3·7%)	0·4 (0·3–0·5)	-60·8% (-73·1 to -44·5%)
	Subarachnoid haemorrhage	4% (3·1–5·1%)	3·4 (2·3–4·7)	-60·4% (-75·8 to -35·5%)	3·4% (2·6–4·3%)	0·1 (0·1–0·2)	-59·1% (-76·1 to -28·9%)
Short gestation	Meningitis	2·6% (1·7–4%)	0·7 (0·4–1)	-83·4% (-91·8 to -67·6%)	1·3% (0·8–2%)	<0·1 (<0·1–<0·1)	-83·4% (-91·8 to -67·6%)
	Encephalitis	1·2% (0·6–1·9%)	0·2 (0·1–0·3)	-34·8% (-73·6 to 46·8%)	0·6% (0·3–1%)	<0·1 (<0·1–<0·1)	-34·8% (-73·6 to 46·8%)
Smoking	Stroke	9·4% (8·2–10·7%)	84·9 (65·5–108·6)	-53% (-66·5 to -35·3%)	5·9% (5–6·9%)	3·1 (2·3–4·3)	-49·2% (-66·2 to -25·6%)
	Ischaemic stroke	8% (6·9–9·2%)	46 (35·1–59·4)	-45·8% (-59·7 to -24·6%)	4·9% (4–5·9%)	1·8 (1·3–2·6)	-40·2% (-60·1 to -9·2%)
	Intracerebral haemorrhage	11·5% (9·9–13·2%)	28·1 (21–37·8)	-61·3% (-74·1 to -44·7%)	7·8% (6·6–9·2%)	1 (0·7–1·3)	-59·1% (-73·5 to -40·1%)
	Subarachnoid haemorrhage	12·9% (10·8–15·2%)	10·8 (7·5–15)	-53·8% (-76·7 to -15·1%)	9·1% (7·2–11%)	0·3 (0·2–0·5)	-53·8% (-78·6 to -7·4%)
	Neurological disorders*	3·9% (1·2–8·6%)	53·4 (13·7–139·2)	52·5% (12·9 to 114·8%)	6·6% (0·5–12·9%)	3·1 (0·1–10·2)	79·8% (-14 to 283%)
	Alzheimer's disease and other dementias	15% (9–21%)	60·9 (22·1–145·6)	42·2% (4·6 to 85·2%)	13·1% (7·8–18·9%)	3·7 (0·8–10·9)	68·1% (17·4 to 134·8%)
	Parkinson's disease	-6·2% (-9·4–-3·2%)	-9·1 (-15·3–-4·5)	1·3% (-32·7 to 50·8%)	-5·5% (-8·5–-2·8%)	-0·6 (-1–-0·3)	25% (-19·8 to 91·6%)
	Multiple sclerosis	9·8% (7–12·7%)	1·6 (1–2·4)	34·6% (-2·4 to 80·1%)	9·7% (6·7–13·2%)	<0·1 (<0·1–<0·1)	-18% (-58·3 to 36·4%)
Tobacco	Stroke	12·1% (10·7–13·5%)	109·1 (85·3–138·6)	-53·7% (-66·3 to -37·6%)	8·3% (7·1–9·5%)	4·4 (3·2–5·9)	-49·2% (-64·7 to -28·3%)
	Ischaemic stroke	10·3% (9·1–11·7%)	59·8 (46·9–76·5)	-46·2% (-59·6 to -27·9%)	7·1% (6–8·3%)	2·6 (1·9–3·7)	-40·5% (-58·4 to -13·8%)
	Intracerebral haemorrhage	14·6% (12·9–16·4%)	35·6 (26·4–47·8)	-62% (-73·8 to -47%)	10·5% (9·1–12%)	1·3 (1–1·8)	-59·3% (-72·6 to -42·1%)
	Subarachnoid haemorrhage	16·4% (14·1–18·7%)	13·7 (9·6–18·8)	-55·2% (-76 to -23·2%)	12·2% (10–14·3%)	0·4 (0·3–0·6)	-55% (-77·2 to -18·6%)
	Neurological disorders*	3·9% (1·2–8·6%)	53·4 (13·7–139·2)	52·5% (12·9 to 114·8%)	6·6% (0·5–12·9%)	3·1 (0·1–10·2)	79·8% (-14 to 283%)
	Alzheimer's disease and other dementias	15% (9–21%)	60·9 (22·1–145·6)	42·2% (4·6 to 85·2%)	13·1% (7·8–18·9%)	3·7 (0·8–10·9)	68·1% (17·4 to 134·8%)
	Parkinson's disease	-6·2% (-9·4–-3·2%)	-9·1 (-15·3–-4·5)	1·3% (-32·7 to 50·8%)	-5·5% (-8·5–-2·8%)	-0·6 (-1–-0·3)	25% (-19·8 to 91·6%)
	Multiple sclerosis	9·8% (7–12·7%)	1·6 (1–2·4)	34·6% (-2·4 to 80·1%)	9·7% (6·7–13·2%)	<0·1 (<0·1–<0·1)	-18% (-58·3 to 36·4%)
**Saudi Arabia**							
Air pollution	Meningitis	0·1% (0–0·2%)	<0·1 (<0·1–0·1)	-90% (-96·4 to -74·6%)	0% (0–0·1%)	<0·1 (<0·1–<0·1)	-90·1% (-96·4 to -74·6%)
	Encephalitis	0·1% (0–0·5%)	<0·1 (<0·1–0·2)	-49·5% (-89·5 to 45·4%)	0·1% (0–0·3%)	<0·1 (<0·1–<0·1)	-49·5% (-89·6 to 45·2%)
	Stroke	35·8% (31·6–39·7%)	756·8 (587–933·1)	-27·8% (-47·1 to -2·5%)	30·5% (26·7–34·2%)	31·4 (23·9–38·6)	-32·9% (-50·5 to -7·9%)
	Ischaemic stroke	33·7% (29·6–37·6%)	438·6 (340·4–538·6)	-15·3% (-36·7 to 9·4%)	28·7% (25–32·4%)	20·2 (15·2–24·9)	-23% (-43·4 to 3·2%)
	Intracerebral haemorrhage	38·9% (34·3–43%)	297·9 (227·2–374·6)	-39·3% (-57·9 to -10·2%)	34·3% (30·1–38·2%)	10·7 (8–13·4)	-44·9% (-61·1 to -18·4%)
	Subarachnoid haemorrhage	40·9% (36–45·4%)	20·3 (14·5–27·1)	-49·4% (-70·7 to -14·8%)	36·2% (31·7–40·5%)	0·5 (0·4–0·7)	-56·3% (-76·5 to -22%)
Alcohol use	Stroke	0·2% (-0·1–0·6%)	4 (-2·6–14·2)	-76·6% (-183·4 to 70·1%)	0·1% (-0·1–0·4%)	0·1 (-0·1–0·4)	-82·2% (-223·3 to 59·1%)

Table S16 Regional and national disabilities and deaths related to neurological conditions attributable to risk factors in North Africa and Middle East countries

**Location**<br>Risk factor (alphabetic order)	Cause	**DALYs (95% UI)**<br>PAF (%)<br>2019	 Rate per 100,000<br>2019	 Rate change (%)<br>From 1990 to 2019	**Deaths (95% UI)**<br>PAF (%)<br>2019	 Rate per 100,000<br>2019	 Rate change (%)<br>From 1990 to 2019
	Ischaemic stroke	-0·1% (-0·3–0·2%)	-0·9 (-3·9–2·7)	-212·9% (-592·5 to 750·3%)	-0·1% (-0·2–0·1%)	-0·1 (-0·2–0·1)	1021·8% (-591·9 to 505·3%)
	Intracerebral haemorrhage	0·6% (0–1·6%)	4·9 (0·1–12·4)	-70·2% (-93·7 to -44·5%)	0·5% (0–1·3%)	0·2 (0–0·4)	-72·6% (-94·9 to -49·2%)
	Neurological disorders*	0·1% (0–0·4%)	2 (0·3–4·9)	-43·1% (-78·4 to 26·9%)	0·1% (0–0·2%)	<0·1 (<0·1–<0·1)	-47·3% (-75·5 to -7·5%)
	Idiopathic epilepsy	0·9% (0·2–1·9%)	2 (0·3–4·9)	-43·1% (-78·4 to 26·9%)	1·1% (0·2–2·2%)	<0·1 (<0·1–<0·1)	-47·3% (-75·5 to -7·5%)
All risk factors	Meningitis	0·5% (0·3–0·8%)	0·1 (0·1–0·2)	-90·5% (-95·6 to -80·6%)	0·2% (0·1–0·3%)	<0·1 (<0·1–<0·1)	-90·5% (-95·6 to -80·6%)
	Encephalitis	0·5% (0·1–1·8%)	0·2 (0–0·6)	-49·8% (-87·4 to 7%)	0·2% (0·1–1%)	<0·1 (<0·1–<0·1)	-49·8% (-87·4 to 7%)
	Stroke	89·6% (86·4–92·5%)	1894·5 (1500·9–2230)	-25·6% (-44 to -0·7%)	86·7% (82·2–91·3%)	89·1 (68·5–105·4)	-29·3% (-47·1 to -5%)
	Ischaemic stroke	89% (84·9–93·3%)	1158·4 (900·6–1354·2)	-13·3% (-33·9 to 12·6%)	86% (79·8–92·5%)	60·3 (44·8–72·3)	-19·1% (-39·2 to 8·2%)
	Intracerebral haemorrhage	90·5% (87·4–93·2%)	692·6 (537·9–846·2)	-38·5% (-55·9 to -9·9%)	88·3% (83·7–92·4%)	27·5 (20·9–33·5)	-43·4% (-58·5 to -17·5%)
	Subarachnoid haemorrhage	88% (84·4–91%)	43·5 (31·5–57·1)	-49% (-70·8 to -15·5%)	86·5% (81·6–90·4%)	1·3 (0·9–1·7)	-55·1% (-75·8 to -20·6%)
	Neurological disorders*	11·8% (4·7–24%)	167·4 (58·6–413)	38·4% (14·4 to 83·8%)	27% (11·4–43·8%)	10·7 (2–31·1)	43·2% (14·2 to 117·2%)
	Alzheimer's disease and other dementias	43·8% (27·4–61·3%)	170·8 (62·3–417·9)	39·2% (14·9 to 86%)	41·8% (25·4–59·8%)	11 (2·3–31·5)	41·8% (13·5 to 108·7%)
	Parkinson's disease	-5·9% (-8·8–-3·2%)	-6·5 (-10·1–-3·2)	7·7% (-29·9 to 74·6%)	-4·9% (-7·4–-2·6%)	-0·4 (-0·6–-0·2)	2·2% (-35·4 to 73·7%)
	Idiopathic epilepsy	0·9% (0·2–1·9%)	2 (0·3–4·9)	-43·1% (-78·4 to 26·9%)	1·1% (0·2–2·2%)	<0·1 (<0·1–<0·1)	-47·3% (-75·5 to -7·5%)
	Multiple sclerosis	8·9% (6·4–11·9%)	1 (0·7–1·5)	56·6% (9·9 to 129·6%)	10% (7·1–13·5%)	<0·1 (<0·1–<0·1)	61·4% (-19·4 to 275·5%)
Ambient particulate matter pollution	Meningitis	0·1% (0–0·2%)	<0·1 (<0·1–0·1)	-77·8% (-93 to -33·1%)	0% (0–0·1%)	<0·1 (<0·1–<0·1)	-77·9% (-93 to -33·2%)
	Encephalitis	0·1% (0–0·5%)	<0·1 (<0·1–0·2)	11·6% (-78·9 to 278·1%)	0·1% (0–0·3%)	<0·1 (<0·1–<0·1)	11·5% (-78·9 to 278·2%)
	Stroke	35·7% (31·5–39·6%)	755·2 (585·6–932·5)	43% (-10·3 to 165·7%)	30·5% (26·6–34·1%)	31·3 (23·8–38·5)	33·5% (-15·5 to 150·3%)
	Ischaemic stroke	33·6% (29·6–37·6%)	437·7 (339·6–537·8)	68·4% (7·4 to 203·9%)	28·7% (25–32·4%)	20·1 (15·2–24·8)	53·9% (-3·4 to 182·6%)
	Intracerebral haemorrhage	38·9% (34·3–43%)	297·3 (227–374·1)	19·7% (-28·4 to 127·3%)	34·2% (30–38·1%)	10·7 (8–13·4)	9·1% (-33·9 to 109·1%)
	Subarachnoid haemorrhage	40·8% (35·9–45·3%)	20·2 (14·5–27·1)	2·3% (-47·7 to 121·3%)	36·1% (31·7–40·4%)	0·5 (0·4–0·7)	-12% (-58·4 to 96·6%)
Behavioural risk factors‡	Meningitis	0·4% (0·2–0·7%)	0·1 (0·1–0·2)	-90·2% (-95·5 to -80·2%)	0·1% (0·1–0·3%)	<0·1 (<0·1–<0·1)	-90·2% (-95·5 to -80·2%)
	Encephalitis	0·5% (0·1–1·7%)	0·1 (0–0·6)	-48·4% (-87·3 to 10·2%)	0·2% (0·1–0·9%)	<0·1 (<0·1–<0·1)	-48·4% (-87·3 to 10·2%)
	Stroke	34·6% (28·7–40·8%)	733·4 (537·4–930·3)	-20·6% (-41·8 to 9%)	30·4% (24–36·7%)	31·3 (22–39·9)	-25·7% (-46·3 to 2·1%)
	Ischaemic stroke	36% (27·6–44·2%)	468·5 (331·8–611)	-7·8% (-30·3 to 23·7%)	31·8% (23·2–40%)	22·3 (14·7–29·5)	-16·4% (-38 to 14%)
	Intracerebral haemorrhage	32·5% (26·6–39·2%)	248·7 (175·9–329·9)	-35·4% (-55·3 to -3·9%)	27·4% (22·1–33·8%)	8·5 (5·9–11·4)	-41·1% (-58·2 to -11·6%)
	Subarachnoid haemorrhage	32·7% (26·6–39·8%)	16·2 (10·8–22·2)	-46·5% (-70·2 to -8·6%)	28·2% (22·6–34·7%)	0·4 (0·3–0·6)	-53·2% (-75·9 to -14·7%)
	Neurological disorders*	2·7% (0·9–5·7%)	37·9 (11·4–95·3)	-1·5% (-31·3 to 38·8%)	4·5% (0·6–8·5%)	1·9 (0·1–5·8)	-1·6% (-42·5 to 74·5%)
	Alzheimer's disease and other dementias	10·6% (6·3–15·5%)	41·4 (15·4–99)	2·7% (-25·9 to 43·7%)	8·3% (4·9–12·3%)	2·2 (0·5–6·2)	-0·4% (-30·4 to 52·6%)
	Parkinson's disease	-5·9% (-8·8–-3·2%)	-6·5 (-10·1–-3·2)	7·7% (-29·9 to 74·6%)	-4·9% (-7·4–-2·6%)	-0·4 (-0·6–-0·2)	2·2% (-35·4 to 73·7%)

Table S16 Regional and national disabilities and deaths related to neurological conditions attributable to risk factors in North Africa and Middle East countries

Location<br>Risk factor (alphabetic order)	Cause	DALYs (95% UI)<br>PAF (%)<br>2019	Rate per 100,000<br>2019	Rate change (%)<br>From 1990 to 2019	Deaths (95% UI)<br>PAF (%)<br>2019	Rate per 100,000<br>2019	Rate change (%)<br>From 1990 to 2019
	Idiopathic epilepsy	0·9% (0·2–1·9%)	2 (0·3–4·9)	-43·1% (-78·4 to 26·9%)	1·1% (0·2–2·2%)	<0·1 (<0·1–<0·1)	-47·3% (-75·5 to -7·5%)
	Multiple sclerosis	8·9% (6·4–11·9%)	1 (0·7–1·5)	56·6% (9·9 to 129·6%)	10% (7·1–13·5%)	<0·1 (<0·1–<0·1)	61·4% (-19·4 to 275·5%)
Child and maternal malnutrition	Meningitis	0·4% (0·2–0·7%)	0·1 (0·1–0·2)	-90·2% (-95·5 to -80·2%)	0·1% (0·1–0·3%)	<0·1 (<0·1–<0·1)	-90·2% (-95·5 to -80·2%)
	Encephalitis	0·5% (0·1–1·7%)	0·1 (0–0·6)	-48·4% (-87·3 to 10·2%)	0·2% (0·1–0·9%)	<0·1 (<0·1–<0·1)	-48·4% (-87·3 to 10·2%)
Diet high in red meat	Stroke	3·5% (1·3–5·4%)	73·2 (25·6–120·4)	-30·9% (-52·4 to -2·7%)	2·6% (1–4·1%)	2·7 (0·9–4·5)	-35% (-54·9 to -7·8%)
	Ischaemic stroke	3% (0·4–4·7%)	38·7 (5·6–66·7)	-15·6% (-38 to 12·3%)	2·2% (0·4–3·6%)	1·6 (0·2–2·7)	-22·2% (-43·5 to 5·5%)
	Intracerebral haemorrhage	4·2% (0·5–6·9%)	32·2 (4·1–58·3)	-41·7% (-59·7 to -13·5%)	3·5% (0·5–5·7%)	1·1 (0·1–2)	-46·3% (-62·5 to -20·8%)
	Subarachnoid haemorrhage	4·8% (0·6–8%)	2·4 (0·3–4·5)	-52% (-72·8 to -19·4%)	3·9% (0·5–6·5%)	0·1 (0–0·1)	-58·1% (-77·9 to -24·1%)
Diet high in sodium	Stroke	2·1% (0·3–8·4%)	44·4 (5·4–176)	-25·1% (-57·6 to 38%)	1·8% (0·3–7%)	1·8 (0·3–7·3)	-28·9% (-60 to 30·6%)
	Ischaemic stroke	1·9% (0·3–7·6%)	24·5 (3·3–96·7)	-10·6% (-52·1 to 61·8%)	1·6% (0·3–6·2%)	1·1 (0·2–4·5)	-16·6% (-54·5 to 50·5%)
	Intracerebral haemorrhage	2·5% (0·3–9·9%)	18·8 (1·9–75·7)	-36·7% (-65·1 to 21·8%)	2·2% (0·3–8·9%)	0·7 (0·1–2·9)	-41·2% (-68·4 to 10·7%)
	Subarachnoid haemorrhage	2·3% (0·2–8·9%)	1·1 (0·1–4·5)	-48·1% (-74·9 to 11·6%)	2·1% (0·3–8·6%)	<0·1 (<0·1–0·1)	-54% (-79·3 to 2·2%)
Diet low in fibre	Stroke	1·4% (0·3–3%)	29·8 (6·9–66·9)	-40·5% (-58 to -14·1%)	1·2% (0·3–2·5%)	1·2 (0·3–2·7)	-49·3% (-65·1 to -25·2%)
	Ischaemic stroke	1·3% (0·3–2·8%)	17·1 (4·1–37·3)	-30·5% (-50·8 to -4·8%)	1·1% (0·3–2·3%)	0·8 (0·2–1·7)	-42·7% (-62·1 to -14·6%)
	Intracerebral haemorrhage	1·5% (0·3–3·3%)	11·8 (2·6–27·1)	-49·4% (-65·3 to -21·9%)	1·3% (0·3–2·9%)	0·4 (0·1–0·9)	-57·3% (-70·8 to -33·9%)
	Subarachnoid haemorrhage	2% (0·4–4·2%)	1 (0·2–2·1)	-57·1% (-76·2 to -24·6%)	1·6% (0·3–3·4%)	<0·1 (<0·1–0·1)	-64·6% (-81·5 to -34·4%)
Diet low in fruits	Stroke	5·5% (2·5–9·1%)	116·7 (51·3–200·3)	-7·3% (-38·1 to 36·2%)	4·3% (2–7·1%)	4·4 (1·9–7·7)	-16·1% (-43 to 20·6%)
	Ischaemic stroke	4·2% (0·9–8·1%)	54·5 (11·2–111·4)	18·6% (-21·9 to 95%)	3·3% (0·7–6·4%)	2·3 (0·5–4·7)	2·4% (-32 to 62·8%)
	Intracerebral haemorrhage	7·6% (3·4–14%)	57·9 (24·8–113·4)	-20·9% (-48·6 to 24·1%)	6·5% (2·8–12·1%)	2 (0·9–3·9)	-28·8% (-53·2 to 11·3%)
	Subarachnoid haemorrhage	8·7% (4–16·1%)	4·3 (1·8–8·4)	-36·4% (-65·7 to 13·8%)	7·2% (3·3–13·5%)	0·1 (0–0·2)	-44·2% (-72·2 to 6·3%)
Diet low in vegetables	Stroke	2·6% (0·7–4·9%)	55·3 (15·9–106·5)	-15·9% (-41·9 to 24·6%)	2·2% (0·7–3·9%)	2·2 (0·7–4·2)	-28·5% (-49·6 to 2·1%)
	Ischaemic stroke	2·2% (0·2–4·2%)	28·2 (3·1–55·9)	-3·6% (-30·9 to 29·3%)	1·8% (0·2–3·4%)	1·3 (0·2–2·5)	-19% (-41·1 to 10·6%)
	Intracerebral haemorrhage	3·3% (0·2–7·9%)	25·4 (1·7–62·8)	-24·8% (-49·8 to 31·5%)	3% (0·2–7·1%)	0·9 (0·1–2·2)	-37·1% (-56·3 to -0·5%)
	Subarachnoid haemorrhage	3·6% (0·2–8·8%)	1·8 (0·1–4·6)	-36·2% (-64·8 to 35·6%)	3·2% (0·2–7·6%)	<0·1 (<0·1–0·1)	-48·3% (-72·7 to 1·5%)
Diet low in whole grains	Stroke	3·5% (0·9–5·1%)	73·3 (18·1–113·5)	-13·4% (-35·1 to 12·4%)	3·1% (0·8–4·5%)	3·2 (0·8–4·9)	-22% (-42·9 to 3·1%)
	Ischaemic stroke	5·6% (1·4–8·3%)	73·3 (18·1–113·5)	-13·4% (-35·1 to 12·4%)	4·6% (1·2–6·7%)	3·2 (0·8–4·9)	-22% (-42·9 to 3·1%)
Dietary risks	Stroke	17% (12·3–23·1%)	360·3 (239·3–508·8)	-21% (-42·5 to 7·4%)	14·2% (10·2–19·2%)	14·5 (9·5–20·6)	-28·3% (-48·4 to -3·2%)
	Ischaemic stroke	16·6% (10·2–22·7%)	215·8 (130·3–308·2)	-9·3% (-32·1 to 19·9%)	13·6% (8·4–18·7%)	9·5 (5·6–13·7)	-19·4% (-40·5 to 7·7%)
	Intracerebral haemorrhage	17·6% (10·7–25·6%)	134·9 (74·9–208·6)	-32·7% (-53·7 to -1%)	15·4% (9·2–22·6%)	4·8 (2·6–7·4)	-39·8% (-57·7 to -11·4%)
	Subarachnoid haemorrhage	19·5% (12·1–27·5%)	9·7 (5·4–14·8)	-44·7% (-69·4 to -3·8%)	16·7% (10·2–24·1%)	0·3 (0·1–0·4)	-52·3% (-75·2 to -11·8%)
Environmental/occupational risk factors§	Meningitis	0·1% (0–0·2%)	<0·1 (<0·1–0·1)	-90% (-96·4 to -74·6%)	0% (0–0·1%)	<0·1 (<0·1–<0·1)	-90·1% (-96·4 to -74·6%)
	Encephalitis	0·1% (0–0·5%)	<0·1 (<0·1–0·2)	-49·5% (-89·5 to 45·4%)	0·1% (0–0·3%)	<0·1 (<0·1–<0·1)	-49·5% (-89·6 to 45·2%)
	Stroke	42·3% (37·6–46·5%)	895·3 (692–1088·5)	-29·2% (-47·8 to -3·3%)	38·4% (33·8–42·5%)	39·5 (29·6–47·9)	-32·8% (-50·4 to -8%)

Table S16 Regional and national disabilities and deaths related to neurological conditions attributable to risk factors in North Africa and Middle East countries

Location<br>Risk factor (alphabetic order)	Cause	DALYs (95% UI)<br>PAF (%)<br>2019	Rate per 100,000<br>2019	Rate change (%)<br>From 1990 to 2019	Deaths (95% UI)<br>PAF (%)<br>2019	Rate per 100,000<br>2019	Rate change (%)<br>From 1990 to 2019
	Ischaemic stroke	40·3% (35·9–44·5%)	524·2 (403·5–632·7)	-16·6% (-37·7 to 9·1%)	36·7% (32·2–40·7%)	25·7 (19·4–31·2)	-22·4% (-43·1 to 3·5%)
	Intracerebral haemorrhage	45·5% (40·6–49·9%)	348·2 (267·3–438·1)	-40·9% (-58·1 to -12·8%)	42·1% (37·3–46·5%)	13·1 (9·9–16·3)	-45·5% (-60·7 to -19%)
	Subarachnoid haemorrhage	46·4% (41·5–51·1%)	23 (16·3–30·7)	-51·4% (-72·2 to -18·4%)	43·4% (38·5–48%)	0·7 (0·4–0·9)	-56·9% (-76·9 to -23·2%)
High body-mass index	Stroke	43·6% (32·3–52·9%)	921·9 (636·9–1223·3)	5·8% (-24·5 to 55·6%)	29·5% (20–38·1%)	30·3 (19·7–42)	3·8% (-27·5 to 55·8%)
	Ischaemic stroke	34·6% (24·4–43·9%)	450 (293·5–608·5)	40·8% (-0·3 to 103·7%)	22·9% (14·3–32·2%)	16·1 (9·4–23·5)	34·3% (-9·4 to 100%)
	Intracerebral haemorrhage	57·5% (44·3–68·2%)	439·8 (311·3–584·2)	-12·7% (-40 to 38·5%)	43·3% (31·2–55%)	13·5 (8·8–18·6)	-15·7% (-42·4 to 33%)
	Subarachnoid haemorrhage	64·8% (52·2–74·6%)	32·1 (22·1–44·2)	-33·3% (-60·9 to 14·9%)	49·3% (36·9–60·9%)	0·7 (0·5–1·1)	-39·1% (-66·7 to 13·5%)
	Neurological disorders*	6·7% (2·1–14·9%)	95·3 (26·6–234·5)	80·6% (39 to 174·5%)	15·9% (5·5–29·2%)	6·2 (1·1–18·2)	83·7% (34·5 to 204·7%)
	Alzheimer's disease and other dementias	24·4% (10·8–40·6%)	95·3 (26·6–234·5)	80·6% (39 to 174·5%)	23·7% (10·5–39·5%)	6·2 (1·1–18·2)	83·7% (34·5 to 204·7%)
High fasting plasma glucose	Stroke	29·9% (20·7–43·3%)	631·4 (410·5–929·6)	-5·6% (-34·8 to 38·4%)	29·6% (18·6–47·4%)	30·4 (18·7–50·8)	-16·7% (-43 to 22·1%)
	Ischaemic stroke	30·7% (17·8–51·3%)	399·1 (223·9–669·2)	4·8% (-30·8 to 55·8%)	29·6% (15·7–56·4%)	20·8 (10·6–40·5)	-8·6% (-38·9 to 39·8%)
	Intracerebral haemorrhage	28·8% (18·8–40·7%)	220·7 (135·3–329·7)	-18·5% (-46·5 to 23·6%)	29·6% (19·4–42·7%)	9·2 (5·6–13·9)	-29·3% (-53·7 to 7·7%)
	Subarachnoid haemorrhage	23·3% (15·2–33%)	11·5 (6·6–17·8)	-33·7% (-64·4 to 18·3%)	26·8% (18–38·2%)	0·4 (0·2–0·6)	-44·4% (-72 to 5·4%)
	Neurological disorders*	4·6% (0·9–12·6%)	65 (12–204·8)	46·8% (25·6 to 93·8%)	11·2% (2·5–25·7%)	4·4 (0·5–15·7)	45·5% (19·5 to 117·4%)
	Alzheimer's disease and other dementias	16·7% (4·3–33·4%)	65 (12–204·8)	46·8% (25·6 to 93·8%)	16·7% (4·4–33·7%)	4·4 (0·5–15·7)	45·5% (19·5 to 117·4%)
High LDL cholesterol	Stroke	14·7% (8·7–23·9%)	310·6 (174·2–522·4)	10·2% (-18 to 52·2%)	13·4% (5·3–27%)	13·8 (5·3–29)	-0·6% (-27·2 to 39·4%)
	Ischaemic stroke	23·9% (14·3–39·3%)	310·6 (174·2–522·4)	10·2% (-18 to 52·2%)	19·7% (7·7–39·5%)	13·8 (5·3–29)	-0·6% (-27·2 to 39·4%)
High systolic blood pressure	Stroke	53·5% (45·7–61%)	1131·9 (852·2–1403·6)	-24·5% (-44·5 to 3·1%)	50·5% (41·7–59·4%)	51·8 (37·5–65·5)	-26·8% (-46·5 to 0·2%)
	Ischaemic stroke	50·5% (40·3–60·2%)	657·2 (476·9–832·7)	-9·5% (-32·6 to 18·4%)	47·4% (36·3–59·7%)	33·2 (22·9–44·8)	-14% (-37·6 to 17·5%)
	Intracerebral haemorrhage	58·6% (48·3–68·2%)	448·2 (329·1–577·7)	-37·8% (-55·9 to -7·2%)	57·3% (45·8–68%)	17·8 (12·9–23·1)	-41·4% (-58·4 to -13·1%)
	Subarachnoid haemorrhage	53·5% (42·8–64·1%)	26·5 (18·1–36·4)	-50·4% (-72·4 to -16·5%)	54·9% (44·2–65·3%)	0·8 (0·5–1·2)	-54·7% (-76·5 to -19·3%)
High temperature	Stroke	3·5% (1·3–6·3%)	74·1 (26·3–139·7)	-15·7% (-58 to 46·5%)	4·1% (1·5–7·4%)	4·2 (1·5–7·8)	-15·3% (-56·4 to 41·1%)
	Ischaemic stroke	3·4% (1·2–6·1%)	44 (15·6–82·9)	-1·8% (-55·6 to 66%)	4·1% (1·5–7·4%)	2·9 (1–5·4)	-3·2% (-53·8 to 60·3%)
	Intracerebral haemorrhage	3·7% (1·3–6·7%)	28·4 (9·9–53·7)	-28·9% (-67·1 to 25%)	4·1% (1·5–7·4%)	1·3 (0·5–2·4)	-32·4% (-66·6 to 15·8%)
	Subarachnoid haemorrhage	3·4% (1·2–6·1%)	1·7 (0·6–3·1)	-46% (-78·6 to 2·9%)	4·1% (1·5–7·4%)	0·1 (0–0·1)	-46·6% (-78·6 to 1·2%)
Household air pollution from solid fuels	Meningitis	0% (0–0%)	<0·1 (<0·1–<0·1)	-100% (-100 to -99·8%)	0% (0–0%)	<0·1 (<0·1–<0·1)	-100% (-100 to -99·8%)
	Encephalitis	0% (0–0%)	<0·1 (<0·1–<0·1)	-99·7% (-100 to -98·9%)	0% (0–0%)	<0·1 (<0·1–<0·1)	-99·7% (-100 to -98·9%)
	Stroke	0·1% (0–0·2%)	1·6 (0·4–4·4)	-99·7% (-99·9 to -99%)	0·1% (0–0·2%)	0·1 (0–0·2)	-99·7% (-99·9 to -99·1%)
	Ischaemic stroke	0·1% (0–0·2%)	0·9 (0·2–2·5)	-99·6% (-99·9 to -98·9%)	0·1% (0–0·2%)	<0·1 (<0·1–0·1)	-99·7% (-99·9 to -99%)
	Intracerebral haemorrhage	0·1% (0–0·2%)	0·6 (0·1–1·7)	-99·7% (-99·9 to -99·1%)	0·1% (0–0·2%)	<0·1 (<0·1–0·1)	-99·8% (-99·9 to -99·2%)
	Subarachnoid haemorrhage	0·1% (0–0·2%)	<0·1 (<0·1–0·1)	-99·8% (-99·9 to -99·3%)	0·1% (0–0·2%)	<0·1 (<0·1–<0·1)	-99·8% (-100 to -99·4%)

Table S16 Regional and national disabilities and deaths related to neurological conditions attributable to risk factors in North Africa and Middle East countries

**Location**<br>Risk factor (alphabetic order)	Cause	**DALYs (95% UI)**<br>PAF (%)<br>2019	 Rate per 100,000<br>2019	 Rate change (%)<br>From 1990 to 2019	**Deaths (95% UI)**<br>PAF (%)<br>2019	 Rate per 100,000<br>2019	 Rate change (%)<br>From 1990 to 2019
Kidney dysfunction	Stroke	13·5% (10·7–16·1%)	285·8 (208–363·4)	4·8% (-21·4 to 39·7%)	12·5% (8·5–16·3%)	12·9 (8·3–17·3)	-1·7% (-29·5 to 31·7%)
	Ischaemic stroke	15% (10·8–18·7%)	194·8 (132·1–257·6)	22·1% (-8·9 to 58·3%)	12·4% (6·9–17·2%)	8·7 (4·7–12·6)	11·6% (-24 to 49·5%)
	Intracerebral haemorrhage	11·9% (9·6–14·5%)	91·1 (64·9–118·7)	-19·5% (-42·4 to 17·2%)	13·3% (10·2–16·4%)	4·1 (2·8–5·4)	-21·5% (-43·3 to 13·3%)
Lead exposure	Stroke	3·9% (1·9–6·1%)	82·9 (40–132·5)	-47·8% (-64·1 to -28·8%)	4·1% (2·2–6·2%)	4·2 (2·2–6·6)	-41·9% (-58·4 to -20·8%)
	Ischaemic stroke	3·8% (1·9–5·9%)	50 (24·7–78·4)	-32·3% (-51·5 to -12·4%)	3·9% (2·1–6·1%)	2·7 (1·4–4·5)	-27·9% (-47·7 to -4·8%)
	Intracerebral haemorrhage	4·1% (1·8–6·8%)	31·4 (13·5–54)	-60·5% (-75 to -41·4%)	4·5% (2·3–7%)	1·4 (0·7–2·3)	-56·6% (-69·8 to -37·2%)
	Subarachnoid haemorrhage	3·1% (1·2–5·6%)	1·6 (0·5–3)	-72·8% (-88·2 to -52·3%)	3·9% (1·9–6·4%)	0·1 (0–0·1)	-68·7% (-85·5 to -44%)
Low birth weight	Meningitis	0·4% (0·2–0·6%)	0·1 (0·1–0·2)	-90·7% (-95·7 to -81·1%)	0·1% (0·1–0·2%)	<0·1 (<0·1–<0·1)	-90·7% (-95·7 to -81·1%)
	Encephalitis	0·4% (0·1–1·5%)	0·1 (0–0·5)	-51% (-88 to 4·6%)	0·2% (0–0·8%)	<0·1 (<0·1–<0·1)	-51% (-88 to 4·6%)
Low birth weight and short gestation	Meningitis	0·4% (0·2–0·7%)	0·1 (0·1–0·2)	-90·2% (-95·5 to -80·2%)	0·1% (0·1–0·3%)	<0·1 (<0·1–<0·1)	-90·2% (-95·5 to -80·2%)
	Encephalitis	0·5% (0·1–1·7%)	0·1 (0–0·6)	-48·4% (-87·3 to 10·2%)	0·2% (0·1–0·9%)	<0·1 (<0·1–<0·1)	-48·4% (-87·3 to 10·2%)
Low physical activity	Stroke	7·5% (1·9–14·3%)	159·9 (40·9–298·4)	2·3% (-24·2 to 50·2%)	8·9% (2·6–16%)	9·2 (2·6–16·5)	-9·8% (-34 to 32·2%)
	Ischaemic stroke	12·3% (3·1–23%)	159·9 (40·9–298·4)	2·3% (-24·2 to 50·2%)	13·1% (3·8–23·4%)	9·2 (2·6–16·5)	-9·8% (-34 to 32·2%)
Low temperature	Stroke	3·1% (-0·2–6·5%)	66·3 (-4·6–142·4)	-37·3% (-73·4 to 7·3%)	3·6% (-0·3–7·5%)	3·7 (-0·3–8)	-37·1% (-73·4 to 2%)
	Ischaemic stroke	3% (-0·2–6·3%)	39·3 (-2·7–84·1)	-26·9% (-70·4 to 17·6%)	3·6% (-0·3–7·5%)	2·6 (-0·2–5·5)	-28% (-70·1 to 16·8%)
	Intracerebral haemorrhage	3·3% (-0·2–6·9%)	25·4 (-1·8–55·6)	-47·2% (-78·3 to -6·1%)	3·6% (-0·3–7·5%)	1·1 (-0·1–2·5)	-49·9% (-78·7 to -12·6%)
	Subarachnoid haemorrhage	3% (-0·2–6·2%)	1·5 (-0·1–3·4)	-59·8% (-85·7 to -20·3%)	3·6% (-0·3–7·5%)	0·1 (0–0·1)	-60·3% (-85·8 to -23·7%)
Metabolic risk factors†	Stroke	77·8% (71·5–83·8%)	1645·9 (1293·6–1954·6)	-20·7% (-40·7 to 6·7%)	73·5% (64·6–81·9%)	75·5 (57·1–91·8)	-24·8% (-44·6 to 1·9%)
	Ischaemic stroke	77% (68·8–85·5%)	1001·6 (772–1200·6)	-7·2% (-30·3 to 21·9%)	72·1% (61–84·8%)	50·5 (36·6–63·6)	-13·7% (-36·4 to 16·6%)
	Intracerebral haemorrhage	79·4% (72–85·2%)	607·3 (466·3–753·3)	-34·4% (-53·2 to -2·7%)	76·7% (68·4–83·8%)	23·9 (18·2–29·7)	-39·5% (-56·3 to -11%)
	Subarachnoid haemorrhage	74·8% (66·1–81·6%)	37 (26·5–50)	-45·8% (-69·2 to -9·4%)	72·8% (63·6–80·4%)	1·1 (0·7–1·5)	-52·4% (-74·7 to -14·7%)
	Neurological disorders*	10·2% (3·7–21·5%)	144·4 (44·8–358·4)	58·3% (28·7 to 123·6%)	24·5% (9·9–41·3%)	9·6 (1·7–27·9)	58·8% (24 to 147·9%)
	Alzheimer's disease and other dementias	37% (19·4–56·2%)	144·4 (44·8–358·4)	58·3% (28·7 to 123·6%)	36·5% (18·8–55·9%)	9·6 (1·7–27·9)	58·8% (24 to 147·9%)
Non-optimal temperature	Stroke	6·5% (2·9–9·9%)	137·8 (57·6–221·2)	-27·6% (-55·9 to 22·4%)	7·6% (3·4–11·4%)	7·8 (3·3–12·3)	-27·3% (-54·9 to 19·1%)
	Ischaemic stroke	6·3% (2·8–9·6%)	81·8 (33·8–130)	-15·6% (-50·2 to 40·2%)	7·6% (3·4–11·4%)	5·3 (2·3–8·4)	-16·8% (-49 to 37·6%)
	Intracerebral haemorrhage	6·9% (3·1–10·5%)	52·8 (21·7–86·3)	-39% (-62·7 to 7·1%)	7·6% (3·4–11·4%)	2·4 (1–3·8)	-42·1% (-63·6 to 0·8%)
	Subarachnoid haemorrhage	6·3% (2·7–9·6%)	3·1 (1·2–5·4)	-53·6% (-76·5 to -4·7%)	7·6% (3·4–11·4%)	0·1 (0–0·2)	-54·1% (-76·7 to -7·4%)
Other environmental risks	Stroke	3·9% (1·9–6·1%)	82·9 (40–132·5)	-47·8% (-64·1 to -28·8%)	4·1% (2·2–6·2%)	4·2 (2·2–6·6)	-41·9% (-58·4 to -20·8%)
	Ischaemic stroke	3·8% (1·9–5·9%)	50 (24·7–78·4)	-32·3% (-51·5 to -12·4%)	3·9% (2·1–6·1%)	2·7 (1·4–4·5)	-27·9% (-47·7 to -4·8%)
	Intracerebral haemorrhage	4·1% (1·8–6·8%)	31·4 (13·5–54)	-60·5% (-75 to -41·4%)	4·5% (2·3–7%)	1·4 (0·7–2·3)	-56·6% (-69·8 to -37·2%)
	Subarachnoid haemorrhage	3·1% (1·2–5·6%)	1·6 (0·5–3)	-72·8% (-88·2 to -52·3%)	3·9% (1·9–6·4%)	0·1 (0–0·1)	-68·7% (-85·5 to -44%)
Particulate matter pollution	Meningitis	0·1% (0–0·2%)	<0·1 (<0·1–0·1)	-90% (-96·4 to -74·6%)	0% (0–0·1%)	<0·1 (<0·1–<0·1)	-90·1% (-96·4 to -74·6%)

Table S16 Regional and national disabilities and deaths related to neurological conditions attributable to risk factors in North Africa and Middle East countries

Location<br>Risk factor (alphabetic order)	Cause	DALYs (95% UI)<br>PAF (%)<br>2019	Rate per 100,000<br>2019	Rate change (%)<br>From 1990 to 2019	Deaths (95% UI)<br>PAF (%)<br>2019	Rate per 100,000<br>2019	Rate change (%)<br>From 1990 to 2019
	Encephalitis	0·1% (0–0·5%)	<0·1 (<0·1–0·2)	-49·5% (-89·5 to 45·4%)	0·1% (0–0·3%)	<0·1 (<0·1–<0·1)	-49·5% (-89·6 to 45·2%)
	Stroke	35·8% (31·6–39·7%)	756·8 (587–933·1)	-27·8% (-47·1 to -2·5%)	30·5% (26·7–34·2%)	31·4 (23·9–38·6)	-32·9% (-50·5 to -7·9%)
	Ischaemic stroke	33·7% (29·6–37·6%)	438·6 (340·4–538·6)	-15·3% (-36·7 to 9·4%)	28·7% (25–32·4%)	20·2 (15·2–24·9)	-23% (-43·4 to 3·2%)
	Intracerebral haemorrhage	38·9% (34·3–43%)	297·9 (227·2–374·6)	-39·3% (-57·9 to -10·2%)	34·3% (30·1–38·2%)	10·7 (8–13·4)	-44·9% (-61·1 to -18·4%)
	Subarachnoid haemorrhage	40·9% (36–45·4%)	20·3 (14·5–27·1)	-49·4% (-70·7 to -14·8%)	36·2% (31·7–40·5%)	0·5 (0·4–0·7)	-56·3% (-76·5 to -22%)
Secondhand smoke	Stroke	4·1% (3·1–5·1%)	86·4 (59·2–115·7)	-29·8% (-49·7 to -0·8%)	3·4% (2·6–4·2%)	3·5 (2·4–4·6)	-33·4% (-52·3 to -6·9%)
	Ischaemic stroke	3·6% (2·7–4·5%)	47 (32·2–63·1)	-16·3% (-40·4 to 13·6%)	3% (2·3–3·8%)	2·1 (1·4–2·8)	-22·7% (-44·7 to 6·3%)
	Intracerebral haemorrhage	4·8% (3·6–6·1%)	36·7 (24·7–50·7)	-40·2% (-59·2 to -10·2%)	4·1% (3–5·1%)	1·3 (0·8–1·7)	-44·7% (-61·3 to -18%)
	Subarachnoid haemorrhage	5·5% (4·1–7·2%)	2·7 (1·7–4)	-50·8% (-72·9 to -14·2%)	4·8% (3·6–6%)	0·1 (0–0·1)	-56% (-76·3 to -19·6%)
Short gestation	Meningitis	0·3% (0·2–0·6%)	0·1 (0·1–0·2)	-87·2% (-94 to -73·9%)	0·1% (0·1–0·2%)	<0·1 (<0·1–<0·1)	-87·2% (-94 to -73·9%)
	Encephalitis	0·4% (0·1–1·4%)	0·1 (0–0·5)	-32·5% (-83 to 41·2%)	0·2% (0–0·8%)	<0·1 (<0·1–<0·1)	-32·5% (-83 to 41·2%)
Smoking	Stroke	11·4% (9·8–13·3%)	242·5 (174·3–305·2)	-19·7% (-44·4 to 19%)	7·9% (6·6–9·4%)	8·2 (5·8–10·3)	-24·6% (-48·4 to 11%)
	Ischaemic stroke	10% (8·5–11·6%)	129·7 (92·2–161·9)	-2·8% (-33·5 to 36·1%)	6·6% (5·5–7·9%)	4·7 (3·2–5·8)	-10·9% (-40·4 to 27·8%)
	Intracerebral haemorrhage	13·9% (11·9–16·3%)	106·9 (78·5–140·5)	-32·2% (-55 to 8·2%)	10·8% (9–12·7%)	3·4 (2·4–4·4)	-36·5% (-57·8 to 1·4%)
	Subarachnoid haemorrhage	12% (9·3–14·9%)	6 (3·9–8·4)	-45·9% (-74·1 to 2·4%)	10·1% (7·6–12·6%)	0·2 (0·1–0·2)	-51·9% (-79·1 to -0·9%)
	Neurological disorders*	2·5% (0·8–5·6%)	35·9 (9·9–94)	2·8% (-27·6 to 47·7%)	4·4% (0·6–8·5%)	1·8 (0·1–5·8)	-0·7% (-41·9 to 76·4%)
	Alzheimer's disease and other dementias	10·6% (6·3–15·5%)	41·4 (15·4–99)	2·7% (-25·9 to 43·7%)	8·3% (4·9–12·3%)	2·2 (0·5–6·2)	-0·4% (-30·4 to 52·6%)
	Parkinson's disease	-5·9% (-8·8–-3·2%)	-6·5 (-10·1–-3·2)	7·7% (-29·9 to 74·6%)	-4·9% (-7·4–-2·6%)	-0·4 (-0·6–-0·2)	2·2% (-35·4 to 73·7%)
	Multiple sclerosis	8·9% (6·4–11·9%)	1 (0·7–1·5)	56·6% (9·9 to 129·6%)	10% (7·1–13·5%)	<0·1 (<0·1–<0·1)	61·4% (-19·4 to 275·5%)
Tobacco	Stroke	15·1% (13·2–17%)	318·8 (233·6–399·3)	-22·5% (-45·1 to 10·2%)	11% (9·5–12·6%)	11·3 (8·2–14)	-27·4% (-48·9 to 3·4%)
	Ischaemic stroke	13·2% (11·5–15%)	171·6 (126–213·5)	-6·8% (-34·6 to 28·8%)	9·4% (8–10·9%)	6·6 (4·7–8·2)	-15% (-41·5 to 18·7%)
	Intracerebral haemorrhage	18·1% (15·9–20·5%)	138·8 (102–176·2)	-34·4% (-55·3 to 2·7%)	14·3% (12·5–16·5%)	4·5 (3·2–5·6)	-39% (-57·6 to -5·1%)
	Subarachnoid haemorrhage	17% (14·4–19·8%)	8·4 (5·7–11·5)	-47·4% (-72·7 to -7·2%)	14·4% (12·1–16·9%)	0·2 (0·1–0·3)	-53·2% (-77·7 to -13·2%)
	Neurological disorders*	2·5% (0·8–5·6%)	35·9 (9·9–94)	2·8% (-27·6 to 47·7%)	4·4% (0·6–8·5%)	1·8 (0·1–5·8)	-0·7% (-41·9 to 76·4%)
	Alzheimer's disease and other dementias	10·6% (6·3–15·5%)	41·4 (15·4–99)	2·7% (-25·9 to 43·7%)	8·3% (4·9–12·3%)	2·2 (0·5–6·2)	-0·4% (-30·4 to 52·6%)
	Parkinson's disease	-5·9% (-8·8–-3·2%)	-6·5 (-10·1–-3·2)	7·7% (-29·9 to 74·6%)	-4·9% (-7·4–-2·6%)	-0·4 (-0·6–-0·2)	2·2% (-35·4 to 73·7%)
	Multiple sclerosis	8·9% (6·4–11·9%)	1 (0·7–1·5)	56·6% (9·9 to 129·6%)	10% (7·1–13·5%)	<0·1 (<0·1–<0·1)	61·4% (-19·4 to 275·5%)
**Syrian Arab Republic**							
Air pollution	Meningitis	0·4% (0·1–0·7%)	0·4 (0·1–0·7)	-86·9% (-96·5 to -57·2%)	0·2% (0·1–0·5%)	<0·1 (<0·1–<0·1)	-86·9% (-96·5 to -57·2%)
	Encephalitis	0·2% (0·1–0·4%)	0·1 (0–0·1)	-57·7% (-88·5 to 38·5%)	0·1% (0·1–0·3%)	<0·1 (<0·1–<0·1)	-57·7% (-88·5 to 38·5%)
	Stroke	23·3% (19·3–27·5%)	471·7 (341·1–628·8)	-41·4% (-57·1 to -18·4%)	20·1% (16·3–24%)	20 (14·1–26·9)	-37·9% (-55 to -12%)

Table S16 Regional and national disabilities and deaths related to neurological conditions attributable to risk factors in North Africa and Middle East countries

Location<br>Risk factor (alphabetic order)	Cause	DALYs (95% UI)<br>PAF (%)<br>2019	Rate per 100,000<br>2019	Rate change (%)<br>From 1990 to 2019	Deaths (95% UI)<br>PAF (%)<br>2019	Rate per 100,000<br>2019	Rate change (%)<br>From 1990 to 2019
	Ischaemic stroke	22·5% (18·4–26·6%)	241·2 (177·7–320·5)	-28·9% (-47·7 to -3·3%)	19·1% (15·5–23%)	11·1 (7·8–15)	-27% (-48·1 to 1·7%)
	Intracerebral haemorrhage	25·3% (20·9–29·8%)	213 (149·9–290·3)	-50·6% (-64·6 to -28·5%)	21·9% (17·9–26%)	8·3 (5·8–11·4)	-47·2% (-62·2 to -24·7%)
	Subarachnoid haemorrhage	16·1% (12·6–20·1%)	17·6 (12·4–24·3)	-51% (-68·6 to -18·4%)	18·7% (15·1–22·5%)	0·5 (0·4–0·8)	-54·5% (-72·9 to -19·2%)
Alcohol use	Stroke	0·7% (0·4–1·1%)	14·7 (7·1–24·5)	-65·3% (-81 to -46%)	0·5% (0·2–0·9%)	0·5 (0·2–0·9)	-63·1% (-81·5 to -40·4%)
	Ischaemic stroke	0·3% (0–0·5%)	2·8 (0·3–5·9)	-58·7% (-91·3 to -31·3%)	0·2% (0–0·4%)	0·1 (0–0·3)	-57·9% (-99·1 to -22·7%)
	Intracerebral haemorrhage	1·4% (0·7–2·2%)	12 (6–20·3)	-66·5% (-81·1 to -47·7%)	1·1% (0·6–1·8%)	0·4 (0·2–0·7)	-64·3% (-80·8 to -44%)
	Neurological disorders*	0·1% (0–0·3%)	1·6 (0·6–2·9)	-52·2% (-79·7 to 11%)	0% (0–0·1%)	<0·1 (<0·1–<0·1)	-56·6% (-73·5 to -23·2%)
	Idiopathic epilepsy	1·5% (0·8–2·3%)	1·6 (0·6–2·9)	-52·2% (-79·7 to 11%)	2·1% (1·2–3·2%)	<0·1 (<0·1–<0·1)	-56·6% (-73·5 to -23·2%)
All risk factors	Meningitis	2·7% (1·5–4·4%)	2·6 (1·4–4·6)	-86·3% (-93·7 to -69·1%)	1·7% (0·9–2·9%)	<0·1 (<0·1–0·1)	-86·3% (-93·7 to -69·1%)
	Encephalitis	1·5% (0·9–2·2%)	0·4 (0·2–0·7)	-55·2% (-79·4 to -8·7%)	1% (0·6–1·5%)	<0·1 (<0·1–<0·1)	-55·2% (-79·4 to -8·7%)
	Stroke	84·6% (80·9–88·2%)	1708·5 (1346–2186·7)	-34·5% (-51·5 to -11%)	84% (78·9–89·6%)	83·2 (64·5–105·4)	-29·3% (-48·7 to -2·3%)
	Ischaemic stroke	86·9% (81·6–92·9%)	929·5 (736·2–1168·7)	-21·4% (-42·4 to 4%)	84·5% (77·1–93·1%)	49·3 (37·9–63·1)	-18·2% (-41·7 to 11·3%)
	Intracerebral haemorrhage	85·8% (81·8–89·4%)	719·8 (547·7–944·6)	-44·9% (-60·4 to -21·9%)	84·4% (78·6–89·9%)	32 (24·1–42)	-40·2% (-57·1 to -16·3%)
	Subarachnoid haemorrhage	54·1% (47–61·6%)	59·1 (44·6–77·5)	-50·4% (-67·7 to -20·4%)	68·8% (62–75·7%)	2 (1·4–2·7)	-51% (-70·3 to -16·8%)
	Neurological disorders*	12·2% (4·5–24·2%)	159·1 (58·7–402)	12·5% (-10 to 45·8%)	28·1% (13·7–43·3%)	10·1 (1·8–31·1)	16·9% (-10·6 to 76·3%)
	Alzheimer's disease and other dementias	42% (27·9–57%)	164·3 (63·2–409·6)	11·6% (-10·6 to 43·4%)	40·7% (26·6–55·5%)	10·6 (2·3–31·7)	15% (-12·5 to 65·3%)
	Parkinson's disease	-10·1% (-15·7–-5·2%)	-8·5 (-14–-3·9)	-22·7% (-45·7 to 10%)	-9% (-14·2–-4·5%)	-0·5 (-0·8–-0·2)	-17·2% (-43·7 to 25·2%)
	Idiopathic epilepsy	1·5% (0·8–2·3%)	1·6 (0·6–2·9)	-52·2% (-79·7 to 11%)	2·1% (1·2–3·2%)	<0·1 (<0·1–<0·1)	-56·6% (-73·5 to -23·2%)
	Multiple sclerosis	11·4% (8·4–14·4%)	1·7 (1·1–2·5)	-7·3% (-31·4 to 18·8%)	13·2% (9·7–17·4%)	<0·1 (<0·1–<0·1)	-14·4% (-50·9 to 41·3%)
Ambient particulate matter pollution	Meningitis	0·4% (0·1–0·7%)	0·4 (0·1–0·7)	-84% (-95·9 to -47·3%)	0·2% (0·1–0·5%)	<0·1 (<0·1–<0·1)	-84% (-95·9 to -47·3%)
	Encephalitis	0·2% (0·1–0·4%)	0·1 (0–0·1)	-48·6% (-86 to 76·9%)	0·1% (0·1–0·3%)	<0·1 (<0·1–<0·1)	-48·6% (-86 to 76·9%)
	Stroke	23·3% (19·2–27·4%)	470·9 (340·7–628)	-31·2% (-50·1 to -2·5%)	20·1% (16·3–24%)	19·9 (14·1–26·8)	-26·9% (-48·7 to 4·4%)
	Ischaemic stroke	22·5% (18·4–26·6%)	240·7 (177·5–320·1)	-16·3% (-39·9 to 16·3%)	19·1% (15·5–23%)	11·1 (7·8–14·9)	-14% (-39·3 to 22·4%)
	Intracerebral haemorrhage	25·3% (20·9–29·7%)	212·7 (149·5–289·8)	-42% (-59·9 to -14%)	21·8% (17·9–25·9%)	8·3 (5·8–11·3)	-38% (-57·3 to -8·2%)
	Subarachnoid haemorrhage	16% (12·6–20·1%)	17·5 (12·4–24·3)	-42·3% (-63·7 to -1·9%)	18·7% (15·1–22·4%)	0·5 (0·4–0·8)	-46·4% (-67·6 to -2·7%)
Behavioural risk factors‡	Meningitis	2·6% (1·4–4·3%)	2·6 (1·4–4·4)	-86·2% (-93·6 to -68·9%)	1·7% (0·9–2·8%)	<0·1 (<0·1–<0·1)	-86·2% (-93·6 to -68·9%)
	Encephalitis	1·5% (0·9–2·2%)	0·4 (0·2–0·7)	-55% (-79·3 to -7·4%)	0·9% (0·6–1·4%)	<0·1 (<0·1–<0·1)	-55% (-79·4 to -7·4%)
	Stroke	37·1% (32·6–42%)	749·7 (564·3–986·7)	-39·3% (-55·7 to -16%)	33·3% (28·4–38·6%)	33 (24·4–43·1)	-34·7% (-53·2 to -9·6%)
	Ischaemic stroke	39·2% (32·9–46%)	420·1 (313·1–543·4)	-26·8% (-46·6 to -2·1%)	35·1% (28·3–42·6%)	20·5 (14·7–27·4)	-23·6% (-45·7 to 4·2%)
	Intracerebral haemorrhage	36·4% (30·8–42·2%)	305·6 (217·7–415·7)	-50·2% (-64·7 to -27·8%)	31% (25·8–36·6%)	11·7 (8·3–15·8)	-46·8% (-61·9 to -23·2%)
	Subarachnoid haemorrhage	22% (17·3–27·3%)	24 (17·3–33·6)	-50% (-69·2 to -16·2%)	25·9% (20·9–31·7%)	0·7 (0·5–1·1)	-53% (-73·1 to -16·5%)
	Neurological disorders*	5·1% (1·8–10·7%)	66·9 (22·3–162·1)	-15·9% (-35·2 to 10·5%)	11·2% (3·5–18·3%)	4·1 (0·5–12·2)	-10·4% (-38·9 to 35·8%)

Table S16 Regional and national disabilities and deaths related to neurological conditions attributable to risk factors in North Africa and Middle East countries

Location<br>Risk factor (alphabetic order)	Cause	DALYs (95% UI)<br>PAF (%)<br>2019	 Rate per 100,000<br>2019	 Rate change (%)<br>From 1990 to 2019	Deaths (95% UI)<br>PAF (%)<br>2019	 Rate per 100,000<br>2019	 Rate change (%)<br>From 1990 to 2019
	Alzheimer's disease and other dementias	18·5% (11·7–25·1%)	72·1 (27·4–168·2)	-15·6% (-34·6 to 10%)	17·7% (11–24·6%)	4·6 (1–12·8)	-10·9% (-35·1 to 25·8%)
	Parkinson's disease	-10·1% (-15·7–-5·2%)	-8·5 (-14–-3·9)	-22·7% (-45·7 to 10%)	-9% (-14·2–-4·5%)	-0·5 (-0·8–-0·2)	-17·2% (-43·7 to 25·2%)
	Idiopathic epilepsy	1·5% (0·8–2·3%)	1·6 (0·6–2·9)	-52·2% (-79·7 to 11%)	2·1% (1·2–3·2%)	<0·1 (<0·1–<0·1)	-56·6% (-73·5 to -23·2%)
	Multiple sclerosis	11·4% (8·4–14·4%)	1·7 (1·1–2·5)	-7·3% (-31·4 to 18·8%)	13·2% (9·7–17·4%)	<0·1 (<0·1–<0·1)	-14·4% (-50·9 to 41·3%)
Child and maternal malnutrition	Meningitis	2·6% (1·4–4·3%)	2·6 (1·4–4·4)	-86·2% (-93·6 to -68·9%)	1·7% (0·9–2·8%)	<0·1 (<0·1–<0·1)	-86·2% (-93·6 to -68·9%)
	Encephalitis	1·5% (0·9–2·2%)	0·4 (0·2–0·7)	-55% (-79·3 to -7·4%)	0·9% (0·6–1·4%)	<0·1 (<0·1–<0·1)	-55% (-79·4 to -7·4%)
Diet high in red meat	Stroke	3·9% (1·4–5·9%)	78·1 (30·2–127·1)	-47·5% (-62·3 to -26·3%)	3% (1·1–4·6%)	3 (1·1–4·9)	-43·5% (-60·6 to -19%)
	Ischaemic stroke	3·4% (0·6–5·3%)	36·1 (5·6–61·2)	-35·1% (-53·6 to -11·9%)	2·5% (0·4–4%)	1·5 (0·2–2·6)	-31·9% (-53·6 to -4·3%)
	Intracerebral haemorrhage	4·6% (0·6–7·4%)	38·6 (5·4–70·3)	-54·9% (-68·9 to -35·4%)	3·7% (0·5–6·1%)	1·4 (0·2–2·6)	-51% (-66·1 to -29·5%)
	Subarachnoid haemorrhage	3·1% (0·4–5·1%)	3·3 (0·5–5·9)	-54·3% (-71·2 to -24·4%)	3·3% (0·5–5·4%)	0·1 (0–0·2)	-57·3% (-75·1 to -25·5%)
Diet high in sodium	Stroke	1·9% (0·3–7·4%)	39 (4·9–159·4)	-36·9% (-61·4 to 12·4%)	1·6% (0·3–6·5%)	1·6 (0·2–6·8)	-32·5% (-58·1 to 22·2%)
	Ischaemic stroke	1·7% (0·3–7%)	18·7 (2·7–76·6)	-23·2% (-55·1 to 40·6%)	1·4% (0·3–5·9%)	0·8 (0·1–3·6)	-20·1% (-52·6 to 44·3%)
	Intracerebral haemorrhage	2·2% (0·3–8·7%)	18·8 (2–76·7)	-45·7% (-68·6 to -2·8%)	1·9% (0·3–7·9%)	0·7 (0·1–3·1)	-41·6% (-66·3 to 5·2%)
	Subarachnoid haemorrhage	1·3% (0·1–5·3%)	1·5 (0·1–6)	-46·1% (-72·4 to 7·6%)	1·6% (0·2–6·5%)	<0·1 (<0·1–0·2)	-48·7% (-75 to 12·1%)
Diet low in fibre	Stroke	3·1% (0·6–6%)	63·5 (12·8–129·6)	-35·2% (-52·9 to -9%)	2·8% (0·6–5·2%)	2·7 (0·6–5·5)	-29·3% (-49·4 to 0%)
	Ischaemic stroke	3% (0·6–5·6%)	31·8 (6·5–63·8)	-20·8% (-42·1 to 10%)	2·5% (0·6–4·8%)	1·5 (0·3–2·9)	-16·5% (-42·2 to 20·3%)
	Intracerebral haemorrhage	3·5% (0·7–6·6%)	29·2 (5·7–60·5)	-45·3% (-60·9 to -20·9%)	3·1% (0·6–5·9%)	1·2 (0·2–2·4)	-39·5% (-56·2 to -12·9%)
	Subarachnoid haemorrhage	2·3% (0·5–4·5%)	2·5 (0·5–5·3)	-44·6% (-64·6 to -8·3%)	2·6% (0·6–5·1%)	0·1 (0–0·2)	-48·2% (-69 to -7·8%)
Diet low in fruits	Stroke	4·8% (2·2–7·9%)	96·4 (44·4–167·8)	-29·3% (-51 to 1·7%)	3·8% (1·8–6·3%)	3·8 (1·8–6·6)	-24·4% (-48·5 to 12·1%)
	Ischaemic stroke	3·5% (0·7–6·8%)	38 (7–77·3)	-7·2% (-35·5 to 36%)	2·8% (0·6–5·4%)	1·6 (0·3–3·3)	-5·8% (-37·1 to 44·1%)
	Intracerebral haemorrhage	6·4% (2·9–12%)	53·8 (23·5–103·3)	-38·8% (-57·9 to -9·4%)	5·4% (2·4–10·1%)	2·1 (0·9–3·9)	-33·4% (-55 to 1·1%)
	Subarachnoid haemorrhage	4·2% (1·9–8%)	4·6 (2–9·1)	-38·5% (-62 to 7·3%)	4·7% (2·2–8·8%)	0·1 (0·1–0·3)	-42·1% (-67·3 to 5·2%)
Diet low in vegetables	Stroke	2·9% (0·8–5·3%)	59·1 (15·6–110·4)	28·5% (-31·6 to 222·5%)	2·5% (0·8–4·5%)	2·5 (0·7–4·6)	20·3% (-29·3 to 135·7%)
	Ischaemic stroke	2·3% (0·3–4·4%)	25 (2·8–47·2)	30·3% (-30·8 to 194·2%)	2% (0·3–3·6%)	1·1 (0·1–2·2)	20·2% (-30·7 to 128·7%)
	Intracerebral haemorrhage	3·8% (0·3–8·3%)	31·4 (2·1–72·1)	27·2% (-44·9 to 388·1%)	3·4% (0·3–7·5%)	1·3 (0·1–2·9)	21·5% (-41·9 to 268·4%)
	Subarachnoid haemorrhage	2·4% (0·2–5·4%)	2·6 (0·2–5·9)	27·5% (-48·9 to 431·5%)	2·9% (0·2–6·4%)	0·1 (0–0·2)	5·3% (-54·2 to 264·3%)
Diet low in whole grains	Stroke	4·7% (2·5–6·2%)	95·1 (49·6–134·5)	-17·7% (-39·7 to 9·8%)	4·6% (2·7–5·9%)	4·6 (2·5–6·4)	-14·1% (-38·1 to 18·1%)
	Ischaemic stroke	8·9% (4·7–11·5%)	95·1 (49·6–134·5)	-17·7% (-39·7 to 9·8%)	7·8% (4·5–10%)	4·6 (2·5–6·4)	-14·1% (-38·1 to 18·1%)
Dietary risks	Stroke	19·3% (14·5–24·7%)	388·9 (271·7–538·6)	-29·5% (-47·9 to -3·2%)	17% (12·7–21·9%)	16·8 (11·7–23·7)	-24·5% (-44·8 to 4·3%)
	Ischaemic stroke	20·5% (14·7–25·9%)	219·6 (146·9–303)	-17·7% (-39 to 10·9%)	17·6% (12·8–22·2%)	10·3 (6·8–14·3)	-14·6% (-39·4 to 18·8%)
	Intracerebral haemorrhage	18·6% (11·8–25·9%)	156·2 (93·6–241·5)	-40·6% (-58·8 to -13%)	16·3% (10·3–22·9%)	6·2 (3·7–9·4)	-35·4% (-54·8 to -7·3%)
	Subarachnoid haemorrhage	12% (7·3–17·4%)	13·1 (7·6–20·3)	-40·7% (-62·8 to -0·9%)	13·9% (8·6–20%)	0·4 (0·2–0·6)	-44·3% (-67·3 to 0·8%)

Table S16 Regional and national disabilities and deaths related to neurological conditions attributable to risk factors in North Africa and Middle East countries

Location<br>Risk factor (alphabetic order)	Cause	DALYs (95% UI)<br>PAF (%)<br>2019	 Rate per 100,000<br>2019	 Rate change (%)<br>From 1990 to 2019	Deaths (95% UI)<br>PAF (%)<br>2019	 Rate per 100,000<br>2019	 Rate change (%)<br>From 1990 to 2019
Environmental/occupational risk factors§	Meningitis	0·4% (0·1–0·7%)	0·4 (0·1–0·7)	-86·9% (-96·5 to -57·2%)	0·2% (0·1–0·5%)	<0·1 (<0·1–<0·1)	-86·9% (-96·5 to -57·2%)
	Encephalitis	0·2% (0·1–0·4%)	0·1 (0–0·1)	-57·7% (-88·5 to 38·5%)	0·1% (0·1–0·3%)	<0·1 (<0·1–<0·1)	-57·7% (-88·5 to 38·5%)
	Stroke	32·1% (27·4–36·6%)	648·8 (480·9–855·1)	-41·6% (-57·1 to -19·5%)	30·2% (25·6–34·8%)	30 (22–40)	-35·4% (-52·7 to -10·8%)
	Ischaemic stroke	31·1% (26·4–35·6%)	333 (251·2–436·2)	-28·1% (-47·4 to -2·4%)	29·1% (24·5–33·6%)	17 (12·3–22·7)	-23·8% (-45·4 to 4%)
	Intracerebral haemorrhage	34·5% (29·5–39·3%)	289·5 (209·7–388·9)	-50·6% (-64·3 to -29·6%)	32·2% (27·2–37%)	12·2 (8·9–16·4)	-45·4% (-60·6 to -22·9%)
	Subarachnoid haemorrhage	24% (19·6–28·8%)	26·2 (18·6–35·8)	-57·6% (-71·8 to -33·6%)	28·2% (23·5–33%)	0·8 (0·6–1·1)	-55·7% (-73 to -25%)
High body-mass index	Stroke	33·7% (23·5–43·4%)	680·5 (426–986·2)	-27·1% (-46·5 to 5·9%)	22·3% (14·1–30·7%)	22·1 (12·8–33·7)	-23·9% (-45·7 to 12·4%)
	Ischaemic stroke	26·1% (17·3–35·6%)	279·5 (168·8–414·1)	-6·1% (-30·8 to 31·5%)	16·7% (9·9–24·5%)	9·7 (5·2–15·8)	-4·5% (-33·9 to 41·2%)
	Intracerebral haemorrhage	43·8% (31·3–55·5%)	367·5 (229·9–532·6)	-37·1% (-55·5 to -5·6%)	30·4% (19·7–41·5%)	11·5 (6·6–17·5)	-33·9% (-54 to -0·6%)
	Subarachnoid haemorrhage	30·6% (21·5–39·4%)	33·5 (21·5–47·5)	-35·8% (-57·8 to 6·1%)	28·8% (19·3–38·3%)	0·8 (0·5–1·3)	-40·3% (-62·8 to 3·8%)
	Neurological disorders*	5·3% (1·5–12%)	68·6 (17·6–180·4)	40·9% (9·3 to 102·4%)	12·1% (3·9–22·9%)	4·3 (0·7–13·7)	41·6% (0·4 to 134·7%)
	Alzheimer's disease and other dementias	17·5% (7·4–31·1%)	68·6 (17·6–180·4)	40·9% (9·3 to 102·4%)	16·5% (6·8–29·9%)	4·3 (0·7–13·7)	41·6% (0·4 to 134·7%)
High fasting plasma glucose	Stroke	29·2% (19·1–44·6%)	589·9 (365·6–968·3)	16·6% (-19·6 to 70·1%)	31·4% (19·1–53·4%)	31·1 (17·6–55·6)	18·6% (-19·7 to 75·8%)
	Ischaemic stroke	32·5% (16·5–58·8%)	347·9 (171·6–688·6)	34·5% (-7·6 to 93·9%)	32·8% (14·8–70·7%)	19·1 (8·4–42·7)	32·7% (-11·9 to 98·5%)
	Intracerebral haemorrhage	26·9% (17·1–39·2%)	226·8 (130·9–362·2)	-1·1% (-34·3 to 50%)	29·8% (17·3–49%)	11·3 (6·1–19·2)	2·7% (-31 to 54·9%)
	Subarachnoid haemorrhage	13·8% (8·3–20·6%)	15·1 (8·7–24·5)	-14·3% (-48·5 to 55·2%)	22·8% (13·5–35·1%)	0·7 (0·3–1·2)	-17·3% (-52·1 to 58·1%)
	Neurological disorders*	4% (0·7–11·3%)	52·3 (9·2–176·1)	55·7% (29·4 to 104·9%)	9·8% (2·1–21·9%)	3·5 (0·4–13·3)	56·5% (21·9 to 127%)
	Alzheimer's disease and other dementias	13·3% (3·3–27·9%)	52·3 (9·2–176·1)	55·7% (29·4 to 104·9%)	13·4% (3·2–28%)	3·5 (0·4–13·3)	56·5% (21·9 to 127%)
High LDL cholesterol	Stroke	12·9% (7·6–21·3%)	259·5 (141·1–442)	-22·2% (-43 to 2·5%)	12% (4·5–25·3%)	11·9 (4·3–24·9)	-18·5% (-42·6 to 9·4%)
	Ischaemic stroke	24·2% (14·4–39·7%)	259·5 (141·1–442)	-22·2% (-43 to 2·5%)	20·4% (7·7–41·8%)	11·9 (4·3–24·9)	-18·5% (-42·6 to 9·4%)
High systolic blood pressure	Stroke	50·6% (42·6–58%)	1021·4 (756·7–1342·5)	-34·7% (-52·5 to -9·3%)	48·5% (39·5–57·8%)	48 (34·8–64·6)	-30·8% (-51·2 to -2·1%)
	Ischaemic stroke	48·9% (38·1–58·5%)	523·1 (371·5–699·9)	-21·1% (-44 to 8·3%)	45·3% (33·7–58·3%)	26·4 (17·5–37·2)	-19·1% (-44·3 to 14·8%)
	Intracerebral haemorrhage	55·1% (45·2–64·7%)	462·4 (335·3–627·5)	-44·5% (-61·2 to -20·7%)	53·7% (41·7–65·9%)	20·3 (14·2–27·7)	-40·6% (-58·3 to -15·2%)
	Subarachnoid haemorrhage	32·8% (25·7–40·8%)	35·8 (25·2–49·5)	-47% (-67 to -11%)	43·6% (34·5–53·6%)	1·2 (0·8–1·8)	-50·1% (-70·2 to -11·6%)
High temperature	Stroke	0·7% (-0·3–1·9%)	13·5 (-6·3–39·9)	-3·4% (-303·6 to 263·9%)	0·8% (-0·3–2·3%)	0·7 (-0·4–2·2)	14·1% (-346 to 359·4%)
	Ischaemic stroke	0·6% (-0·3–1·8%)	6·7 (-3·2–19·7)	21·2% (-359·2 to 367·4%)	0·8% (-0·3–2·3%)	0·4 (-0·2–1·3)	33·7% (-388·4 to 429·8%)
	Intracerebral haemorrhage	0·7% (-0·3–2·1%)	6 (-2·8–17·8)	-14·6% (-280·2 to 223·3%)	0·8% (-0·3–2·3%)	0·3 (-0·1–0·8)	-2·8% (-306·4 to 286·1%)
	Subarachnoid haemorrhage	0·7% (-0·3–2%)	0·8 (-0·3–2·3)	-45·3% (-234·8 to 120·8%)	0·8% (-0·3–2·3%)	<0·1 (<0·1–0·1)	-32% (-268·7 to 175·6%)
Household air pollution from solid fuels	Meningitis	0% (0–0%)	<0·1 (<0·1–<0·1)	-99·8% (-100 to -99·3%)	0% (0–0%)	<0·1 (<0·1–<0·1)	-99·8% (-100 to -99·3%)
	Encephalitis	0% (0–0%)	<0·1 (<0·1–<0·1)	-99·5% (-99·9 to -97·8%)	0% (0–0%)	<0·1 (<0·1–<0·1)	-99·5% (-99·9 to -97·8%)
	Stroke	0% (0–0·1%)	0·8 (0·3–1·9)	-99·3% (-99·8 to -98·3%)	0% (0–0·1%)	<0·1 (<0·1–0·1)	-99·3% (-99·7 to -98·2%)

Table S16 Regional and national disabilities and deaths related to neurological conditions attributable to risk factors in North Africa and Middle East countries

Location<br>Risk factor (alphabetic order)	Cause	DALYs (95% UI)<br>PAF (%)<br>2019	 Rate per 100,000<br>2019	 Rate change (%)<br>From 1990 to 2019	Deaths (95% UI)<br>PAF (%)<br>2019	 Rate per 100,000<br>2019	 Rate change (%)<br>From 1990 to 2019
	Ischaemic stroke	0% (0–0·1%)	0·4 (0·2–1)	-99·2% (-99·7 to -97·9%)	0% (0–0·1%)	<0·1 (<0·1–<0·1)	-99·2% (-99·7 to -97·8%)
	Intracerebral haemorrhage	0% (0–0·1%)	0·4 (0·1–0·8)	-99·4% (-99·8 to -98·5%)	0% (0–0·1%)	<0·1 (<0·1–<0·1)	-99·4% (-99·8 to -98·4%)
	Subarachnoid haemorrhage	0% (0–0·1%)	<0·1 (<0·1–0·1)	-99·4% (-99·8 to -98·4%)	0% (0–0·1%)	<0·1 (<0·1–<0·1)	-99·5% (-99·8 to -98·4%)
Kidney dysfunction	Stroke	10·5% (8·2–12·5%)	212·3 (151·8–282·2)	-13·3% (-37·4 to 19·3%)	10·4% (7·2–13·6%)	10·4 (6·6–14·6)	-7·7% (-35·2 to 29·6%)
	Ischaemic stroke	12·1% (8·6–15·3%)	129·8 (85·2–179·5)	0·4% (-27·8 to 36·6%)	10·4% (5·3–14·7%)	6·1 (3–9·2)	2·2% (-31·2 to 46·4%)
	Intracerebral haemorrhage	9·8% (7·9–12%)	82·5 (55·5–112·2)	-28·7% (-50·5 to 1%)	11·3% (8·2–14·6%)	4·3 (2·8–6·1)	-18·9% (-42·7 to 15·9%)
Lead exposure	Stroke	5·1% (3–7·2%)	102·2 (57·3–157)	-47·2% (-62·5 to -27·4%)	5·4% (3·4–7·7%)	5·4 (3·1–8·3)	-34·6% (-53·1 to -10·1%)
	Ischaemic stroke	5% (3–7·3%)	54 (30·2–83·3)	-30·8% (-51·6 to -7·3%)	5·1% (3–7·7%)	3 (1·6–4·8)	-19·1% (-45·4 to 11·5%)
	Intracerebral haemorrhage	5·4% (3–8·1%)	45 (23·4–72·6)	-57·9% (-71·1 to -39·8%)	6% (3·4–9%)	2·3 (1·2–3·6)	-46·5% (-62·2 to -23·9%)
	Subarachnoid haemorrhage	2·9% (1·4–4·6%)	3·1 (1·5–5·6)	-62·9% (-79·2 to -37·6%)	4·6% (2·6–7%)	0·1 (0·1–0·2)	-56·6% (-75·3 to -23·8%)
Low birth weight	Meningitis	2·3% (1·3–3·9%)	2·3 (1·2–4)	-86·6% (-93·8 to -69·7%)	1·5% (0·8–2·6%)	<0·1 (<0·1–<0·1)	-86·6% (-93·8 to -69·7%)
	Encephalitis	1·3% (0·8–2%)	0·4 (0·2–0·6)	-56·1% (-80 to -9·4%)	0·9% (0·5–1·3%)	<0·1 (<0·1–<0·1)	-56·1% (-80 to -9·4%)
Low birth weight and short gestation	Meningitis	2·6% (1·4–4·3%)	2·6 (1·4–4·4)	-86·2% (-93·6 to -68·9%)	1·7% (0·9–2·8%)	<0·1 (<0·1–<0·1)	-86·2% (-93·6 to -68·9%)
	Encephalitis	1·5% (0·9–2·2%)	0·4 (0·2–0·7)	-55% (-79·3 to -7·4%)	0·9% (0·6–1·4%)	<0·1 (<0·1–<0·1)	-55% (-79·4 to -7·4%)
Low physical activity	Stroke	4·3% (0·9–9·4%)	86·1 (17·5–195·8)	-17·1% (-39 to 11·1%)	5·7% (1·3–11·4%)	5·6 (1·3–11·4)	-14·7% (-38·6 to 16·2%)
	Ischaemic stroke	8·1% (1·8–17·7%)	86·1 (17·5–195·8)	-17·1% (-39 to 11·1%)	9·6% (2·3–19·5%)	5·6 (1·3–11·4)	-14·7% (-38·6 to 16·2%)
Low temperature	Stroke	6·1% (2·7–9·9%)	123·7 (50·9–208·1)	-43·4% (-59 to -20·6%)	6·9% (3·1–11·3%)	6·9 (2·9–11·4)	-33·3% (-50·3 to -8·7%)
	Ischaemic stroke	5·7% (2·5–9·3%)	61·5 (25·7–103·6)	-28·8% (-49·2 to 0·2%)	6·9% (3·1–11·3%)	4 (1·7–6·8)	-21·8% (-43·2 to 8%)
	Intracerebral haemorrhage	6·6% (2·9–10·7%)	55·2 (22·9–94)	-50% (-64·7 to -29·1%)	6·9% (3·1–11·3%)	2·6 (1·1–4·4)	-43·2% (-59·3 to -20·4%)
	Subarachnoid haemorrhage	6·3% (2·8–10·3%)	6·9 (2·9–11·8)	-68% (-78·2 to -50%)	6·9% (3·1–11·3%)	0·2 (0·1–0·3)	-60·1% (-74·2 to -35·8%)
Metabolic risk factors†	Stroke	72·5% (66–78·9%)	1464·8 (1126·9–1894·9)	-29·9% (-47·9 to -2·6%)	70·8% (61·5–80·2%)	70·1 (52·4–91·9)	-25·1% (-46·1 to 5·2%)
	Ischaemic stroke	74·7% (65·6–85·7%)	800 (613·8–1033·8)	-16·9% (-38·9 to 11·9%)	70·7% (58·1–85·8%)	41·2 (29·9–55·2)	-13·7% (-39·1 to 19·9%)
	Intracerebral haemorrhage	73·6% (65·7–80·3%)	617·7 (460·7–819)	-40·9% (-58·1 to -16·2%)	72% (62·5–81·1%)	27·3 (20·1–36·1)	-36·2% (-54·5 to -8·8%)
	Subarachnoid haemorrhage	43·1% (35·5–51%)	47·2 (34·1–63·6)	-43·4% (-64·3 to -5·6%)	56·2% (47·5–65·3%)	1·6 (1·1–2·2)	-46·6% (-68·1 to -7%)
	Neurological disorders*	8·6% (2·8–18·3%)	111·7 (32·3–291·9)	43·3% (15·2 to 92%)	20·3% (8·3–35·7%)	7·2 (1·2–22·1)	44·3% (8·9 to 112·2%)
	Alzheimer's disease and other dementias	28·5% (13·7–46·2%)	111·7 (32·3–291·9)	43·3% (15·2 to 92%)	27·6% (12·6–45·4%)	7·2 (1·2–22·1)	44·3% (8·9 to 112·2%)
Non-optimal temperature	Stroke	6·7% (3·9–10%)	136·4 (73·1–215)	-41·1% (-58·5 to -16%)	7·6% (4·5–11·4%)	7·6 (4·2–11·8)	-30·6% (-50·5 to -3%)
	Ischaemic stroke	6·3% (3·7–9·4%)	67·8 (36·9–106·8)	-26% (-49·6 to 6·2%)	7·6% (4·5–11·4%)	4·5 (2·5–7)	-18·7% (-42·5 to 14·9%)
	Intracerebral haemorrhage	7·3% (4·3–10·7%)	60·9 (31·9–96·5)	-48% (-63·9 to -25·5%)	7·6% (4·5–11·4%)	2·9 (1·6–4·6)	-41% (-58·4 to -16·7%)
	Subarachnoid haemorrhage	7% (4·1–10·3%)	7·6 (4·2–12·1)	-66·8% (-77·2 to -47%)	7·6% (4·5–11·4%)	0·2 (0·1–0·3)	-58·5% (-73·3 to -31·3%)
Other environmental risks	Stroke	5·1% (3–7·2%)	102·2 (57·3–157)	-47·2% (-62·5 to -27·4%)	5·4% (3·4–7·7%)	5·4 (3·1–8·3)	-34·6% (-53·1 to -10·1%)
	Ischaemic stroke	5% (3–7·3%)	54 (30·2–83·3)	-30·8% (-51·6 to -7·3%)	5·1% (3–7·7%)	3 (1·6–4·8)	-19·1% (-45·4 to 11·5%)

Table S16 Regional and national disabilities and deaths related to neurological conditions attributable to risk factors in North Africa and Middle East countries

**Location**<br>Risk factor (alphabetic order)	Cause	**DALYs (95% UI)**<br>PAF (%)<br>2019	Rate per 100,000<br>2019	Rate change (%)<br>From 1990 to 2019	**Deaths (95% UI)**<br>PAF (%)<br>2019	Rate per 100,000<br>2019	Rate change (%)<br>From 1990 to 2019
	Intracerebral haemorrhage	5·4% (3–8·1%)	45 (23·4–72·6)	-57·9% (-71·1 to -39·8%)	6% (3·4–9%)	2·3 (1·2–3·6)	-46·5% (-62·2 to -23·9%)
	Subarachnoid haemorrhage	2·9% (1·4–4·6%)	3·1 (1·5–5·6)	-62·9% (-79·2 to -37·6%)	4·6% (2·6–7%)	0·1 (0·1–0·2)	-56·6% (-75·3 to -23·8%)
Particulate matter pollution	Meningitis	0·4% (0·1–0·7%)	0·4 (0·1–0·7)	-86·9% (-96·5 to -57·2%)	0·2% (0·1–0·5%)	<0·1 (<0·1–<0·1)	-86·9% (-96·5 to -57·2%)
	Encephalitis	0·2% (0·1–0·4%)	0·1 (0–0·1)	-57·7% (-88·5 to 38·5%)	0·1% (0·1–0·3%)	<0·1 (<0·1–<0·1)	-57·7% (-88·5 to 38·5%)
	Stroke	23·3% (19·3–27·5%)	471·7 (341·1–628·8)	-41·4% (-57·1 to -18·4%)	20·1% (16·3–24%)	20 (14·1–26·9)	-37·9% (-55 to -12%)
	Ischaemic stroke	22·5% (18·4–26·6%)	241·2 (177·7–320·5)	-28·9% (-47·7 to -3·3%)	19·1% (15·5–23%)	11·1 (7·8–15)	-27% (-48·1 to 1·7%)
	Intracerebral haemorrhage	25·3% (20·9–29·8%)	213 (149·9–290·3)	-50·6% (-64·6 to -28·5%)	21·9% (17·9–26%)	8·3 (5·8–11·4)	-47·2% (-62·2 to -24·7%)
	Subarachnoid haemorrhage	16·1% (12·6–20·1%)	17·6 (12·4–24·3)	-51% (-68·6 to -18·4%)	18·7% (15·1–22·5%)	0·5 (0·4–0·8)	-54·5% (-72·9 to -19·2%)
Secondhand smoke	Stroke	3·8% (2·9–4·7%)	76·3 (52·2–107)	-43·3% (-59·6 to -21·5%)	3·1% (2·4–3·9%)	3·1 (2·1–4·3)	-40·2% (-57·3 to -15·3%)
	Ischaemic stroke	3·4% (2·5–4·2%)	36·2 (25·2–50·3)	-31% (-50·6 to -5·4%)	2·8% (2·1–3·6%)	1·6 (1·1–2·3)	-29·4% (-49·7 to -0·9%)
	Intracerebral haemorrhage	4·4% (3·3–5·6%)	37 (24·9–53·3)	-51·1% (-65·9 to -28·1%)	3·5% (2·7–4·4%)	1·3 (0·9–1·9)	-48·6% (-63·9 to -25·3%)
	Subarachnoid haemorrhage	2·9% (2·1–3·8%)	3·2 (2·1–4·5)	-51·6% (-69 to -19·1%)	3·2% (2·4–4·1%)	0·1 (0·1–0·1)	-54·8% (-72·8 to -21·2%)
Short gestation	Meningitis	1·8% (1–3%)	1·8 (1–3·2)	-84·8% (-92·9 to -65·8%)	1·2% (0·7–2%)	<0·1 (<0·1–<0·1)	-84·8% (-92·9 to -65·8%)
	Encephalitis	1% (0·6–1·5%)	0·3 (0·2–0·5)	-50·2% (-77·1 to 3·5%)	0·7% (0·4–1%)	<0·1 (<0·1–<0·1)	-50·2% (-77·1 to 3·5%)
Smoking	Stroke	15·8% (14·2–17·3%)	319 (236·7–425·8)	-50·6% (-65·2 to -28·3%)	12% (10·7–13·4%)	11·9 (8·8–16·2)	-49% (-65·1 to -24·1%)
	Ischaemic stroke	14·4% (12·9–16%)	154·7 (116·8–200·6)	-41% (-59·4 to -16·1%)	10·5% (9·2–11·8%)	6·1 (4·5–8·3)	-40·2% (-60·7 to -10·3%)
	Intracerebral haemorrhage	18·2% (16·3–20·1%)	152·7 (110·5–208·1)	-57·1% (-70·5 to -35·7%)	14·4% (12·8–16·1%)	5·5 (4–7·5)	-55·5% (-69·2 to -32·5%)
	Subarachnoid haemorrhage	10·6% (8·4–13·6%)	11·6 (8·1–16·6)	-57·5% (-75·9 to -24·7%)	12·4% (10·1–15·1%)	0·4 (0·2–0·5)	-59·9% (-79 to -25·7%)
	Neurological disorders*	5% (1·7–10·6%)	65·3 (20·6–160·8)	-14·3% (-34·1 to 13·8%)	11·1% (3·4–18·3%)	4·1 (0·5–12·2)	-10·1% (-38·6 to 36%)
	Alzheimer's disease and other dementias	18·5% (11·7–25·1%)	72·1 (27·4–168·2)	-15·6% (-34·6 to 10%)	17·7% (11–24·6%)	4·6 (1–12·8)	-10·9% (-35·1 to 25·8%)
	Parkinson's disease	-10·1% (-15·7–-5·2%)	-8·5 (-14–-3·9)	-22·7% (-45·7 to 10%)	-9% (-14·2–-4·5%)	-0·5 (-0·8–-0·2)	-17·2% (-43·7 to 25·2%)
	Multiple sclerosis	11·4% (8·4–14·4%)	1·7 (1·1–2·5)	-7·3% (-31·4 to 18·8%)	13·2% (9·7–17·4%)	<0·1 (<0·1–<0·1)	-14·4% (-50·9 to 41·3%)
Tobacco	Stroke	18·9% (17·2–20·7%)	382·2 (284·8–508·5)	-49·1% (-63·6 to -26·9%)	14·7% (13·2–16·2%)	14·6 (10·8–19·5)	-47·1% (-63 to -22·3%)
	Ischaemic stroke	17·3% (15·6–19%)	185·1 (140·8–241·3)	-39·1% (-57·3 to -14·2%)	12·9% (11·5–14·5%)	7·6 (5·6–10·1)	-38% (-58·6 to -9·3%)
	Intracerebral haemorrhage	21·8% (19·8–23·8%)	182·9 (133·2–249·8)	-55·8% (-69·1 to -34·5%)	17·3% (15·5–19·1%)	6·6 (4·8–8·9)	-54·1% (-68·1 to -31·5%)
	Subarachnoid haemorrhage	13% (10·5–16·3%)	14·2 (10·2–20)	-56·2% (-74·2 to -23·8%)	15·1% (12·6–18·1%)	0·4 (0·3–0·6)	-58·8% (-77·5 to -25·4%)
	Neurological disorders*	5% (1·7–10·6%)	65·3 (20·6–160·8)	-14·3% (-34·1 to 13·8%)	11·1% (3·4–18·3%)	4·1 (0·5–12·2)	-10·1% (-38·6 to 36%)
	Alzheimer's disease and other dementias	18·5% (11·7–25·1%)	72·1 (27·4–168·2)	-15·6% (-34·6 to 10%)	17·7% (11–24·6%)	4·6 (1–12·8)	-10·9% (-35·1 to 25·8%)
	Parkinson's disease	-10·1% (-15·7–-5·2%)	-8·5 (-14–-3·9)	-22·7% (-45·7 to 10%)	-9% (-14·2–-4·5%)	-0·5 (-0·8–-0·2)	-17·2% (-43·7 to 25·2%)
	Multiple sclerosis	11·4% (8·4–14·4%)	1·7 (1·1–2·5)	-7·3% (-31·4 to 18·8%)	13·2% (9·7–17·4%)	<0·1 (<0·1–<0·1)	-14·4% (-50·9 to 41·3%)

Table S16 Regional and national disabilities and deaths related to neurological conditions attributable to risk factors in North Africa and Middle East countries

Location<br>Risk factor (alphabetic order)	Cause	DALYs (95% UI)<br>PAF (%)<br>2019	 Rate per 100,000<br>2019	 Rate change (%)<br>From 1990 to 2019	Deaths (95% UI)<br>PAF (%)<br>2019	 Rate per 100,000<br>2019	 Rate change (%)<br>From 1990 to 2019
**Tunisia**							
Air pollution	Meningitis	0·9% (0·3–1·9%)	0·4 (0·1–0·8)	-77·4% (-94·5 to -17·8%)	0·5% (0·2–1·1%)	<0·1 (<0·1–<0·1)	-77·4% (-94·5 to -17·8%)
	Encephalitis	0·4% (0·1–1%)	0·1 (0–0·2)	-24·2% (-81·6 to 188·9%)	0·3% (0·1–0·7%)	<0·1 (<0·1–<0·1)	-24·2% (-81·6 to 188·9%)
	Stroke	22·9% (17·4–28·5%)	337·9 (232·8–472·5)	-27·7% (-46·3 to -3·9%)	19·3% (14·3–24·3%)	15·4 (10·1–22)	-29·9% (-49·1 to -5·2%)
	Ischaemic stroke	21·9% (16·6–27·5%)	238·8 (164·3–334·5)	2·2% (-24·2 to 34·7%)	18·5% (13·7–23·5%)	11·7 (7·8–16·7)	-5·8% (-34·5 to 25·6%)
	Intracerebral haemorrhage	25·4% (19·4–31·5%)	82·1 (54·5–115·5)	-56·9% (-70·3 to -36·3%)	22% (16·7–27·6%)	3·2 (2·1–4·5)	-60·5% (-73·1 to -41·4%)
	Subarachnoid haemorrhage	26·4% (20·5–32·5%)	17 (11·1–24·6)	-60·5% (-76·2 to -32·2%)	23% (17·5–28·5%)	0·5 (0·3–0·8)	-65·3% (-80 to -36·9%)
Alcohol use	Stroke	1% (0·5–1·6%)	15 (6·7–24·8)	29·4% (-29·7 to 211·4%)	0·5% (0·1–0·9%)	0·4 (0·1–0·8)	39·4% (-225·4 to 801·6%)
	Ischaemic stroke	0·3% (-0·3–0·8%)	2·9 (-2·7–8·8)	-315·6% (-2938·7 to 3213·6%)	0·1% (-0·3–0·5%)	0·1 (-0·2–0·3)	-152·2% (-1289·1 to 2244·3%)
	Intracerebral haemorrhage	3·8% (2·3–5·4%)	12·2 (6·9–19·3)	-5·9% (-40·8 to 63·9%)	2·6% (1·5–3·8%)	0·4 (0·2–0·6)	-10·5% (-45·8 to 71·2%)
	Neurological disorders*	0·6% (0·2–1·3%)	7·6 (3·7–14)	37·5% (-33·7 to 202·8%)	0·2% (0·1–0·5%)	0·1 (0–0·1)	25·9% (-15·8 to 88·3%)
	Idiopathic epilepsy	6·5% (4·3–9%)	7·6 (3·7–14)	37·5% (-33·7 to 202·8%)	6·9% (4·7–9·6%)	0·1 (0–0·1)	25·9% (-15·8 to 88·3%)
All risk factors	Meningitis	6·2% (3·7–9·8%)	2·3 (1·3–3·9)	-78·7% (-89·9 to -55·9%)	3·5% (2–5·6%)	<0·1 (<0·1–<0·1)	-78·7% (-89·9 to -55·9%)
	Encephalitis	2·8% (1·3–5·3%)	0·5 (0·3–0·9)	-27·1% (-73·1 to 80·6%)	1·8% (0·8–3·5%)	<0·1 (<0·1–<0·1)	-27·1% (-73·1 to 80·6%)
	Stroke	85·5% (80·9–90·5%)	1263 (963–1624·7)	-21% (-41·6 to 5·3%)	82·3% (75·6–90·3%)	65·8 (48·1–85·4)	-22·7% (-43·5 to 3%)
	Ischaemic stroke	85·3% (79·6–92·1%)	931 (709·5–1185·6)	8·1% (-19·9 to 40·9%)	82% (73·9–92·1%)	51·8 (37·8–67·6)	0·9% (-28·1 to 33·3%)
	Intracerebral haemorrhage	86·3% (82–90·1%)	278·4 (204·9–370·4)	-54·2% (-68·3 to -33·4%)	83·9% (77·8–89·5%)	12·1 (8·9–16·2)	-57·8% (-70·7 to -38%)
	Subarachnoid haemorrhage	83·1% (78·3–87·1%)	53·7 (37·5–73·1)	-58·3% (-74·7 to -29·1%)	81·7% (75·3–87·5%)	1·9 (1·3–2·7)	-62·7% (-78·3 to -31·9%)
	Neurological disorders*	13·1% (5·4–25·1%)	171·4 (64·5–400·5)	16·4% (-6·5 to 49·7%)	29·6% (13·9–45·4%)	10·4 (1·9–30·1)	18·3% (-9·6 to 74·5%)
	Alzheimer's disease and other dementias	42·5% (28·3–57·8%)	170·1 (63·5–397·2)	13·8% (-8·3 to 44·9%)	40·8% (26·6–56·3%)	10·9 (2·3–30·4)	16·4% (-10·7 to 64·6%)
	Parkinson's disease	-12·1% (-18·6–-6·1%)	-8·8 (-14·4–-4·2)	-12·8% (-38·8 to 22·4%)	-11·3% (-17·2–-5·7%)	-0·5 (-0·8–-0·2)	-12·3% (-41·1 to 25·9%)
	Idiopathic epilepsy	6·5% (4·3–9%)	7·6 (3·7–14)	37·5% (-33·7 to 202·8%)	6·9% (4·7–9·6%)	0·1 (0–0·1)	25·9% (-15·8 to 88·3%)
	Multiple sclerosis	11·1% (8·1–14·1%)	2·5 (1·6–3·4)	11·5% (-15·6 to 43·6%)	12·8% (8·9–17·5%)	<0·1 (<0·1–0·1)	9·5% (-35·8 to 75%)
Ambient particulate matter pollution	Meningitis	0·9% (0·3–1·9%)	0·4 (0·1–0·8)	-68·7% (-92·3 to 14%)	0·5% (0·2–1·1%)	<0·1 (<0·1–<0·1)	-68·7% (-92·3 to 14%)
	Encephalitis	0·4% (0·1–1%)	0·1 (0–0·2)	5% (-74·9 to 318·9%)	0·3% (0·1–0·7%)	<0·1 (<0·1–<0·1)	5% (-75 to 318·9%)
	Stroke	22·8% (17·3–28·5%)	336·9 (232·4–471·7)	-4·9% (-31·2 to 34·7%)	19·2% (14·3–24·3%)	15·4 (10·1–21·9)	-7·8% (-34 to 31·8%)
	Ischaemic stroke	21·8% (16·5–27·4%)	238·1 (163·9–334)	34·4% (-1·8 to 87·8%)	18·4% (13·7–23·5%)	11·7 (7·7–16·6)	24% (-12·8 to 76·8%)
	Intracerebral haemorrhage	25·4% (19·4–31·3%)	81·8 (54·3–115·4)	-43·4% (-61·9 to -14·4%)	21·9% (16·6–27·5%)	3·2 (2·1–4·5)	-48·1% (-64·6 to -20·6%)
	Subarachnoid haemorrhage	26·4% (20·4–32·5%)	17 (11–24·6)	-47·7% (-69·4 to -6·1%)	22·9% (17·4–28·4%)	0·5 (0·3–0·8)	-54·3% (-74·8 to -9·5%)
Behavioural risk factors‡	Meningitis	5·9% (3·5–9·3%)	2·2 (1·2–3·7)	-78·6% (-90 to -55·1%)	3·3% (1·9–5·4%)	<0·1 (<0·1–<0·1)	-78·6% (-90 to -55·1%)
	Encephalitis	2·7% (1·3–5%)	0·5 (0·2–0·8)	-26·6% (-72·3 to 80·1%)	1·7% (0·8–3·3%)	<0·1 (<0·1–<0·1)	-26·6% (-72·3 to 80·1%)

Table S16 Regional and national disabilities and deaths related to neurological conditions attributable to risk factors in North Africa and Middle East countries

		DALYs (95% UI)			Deaths (95% UI)		
**Location**<br>Risk factor (alphabetic order)		PAF (%)	Rate per 100,000	Rate change (%)	PAF (%)	Rate per 100,000	Rate change (%)
	Cause	2019	2019	From 1990 to 2019	2019	2019	From 1990 to 2019
	Stroke	32·7% (28·7–37·3%)	483·5 (362·3–650·5)	-29·4% (-48 to -4·1%)	28·4% (24·2–33·4%)	22·8 (16·2–30·6)	-31% (-49·9 to -5·8%)
	Ischaemic stroke	32·5% (27·4–38%)	355 (260·5–480·3)	-2·2% (-29 to 29·7%)	28·5% (23·2–34·5%)	18 (12·6–25·1)	-8·8% (-36·5 to 21·5%)
	Intracerebral haemorrhage	33·6% (28·9–38·9%)	108·5 (76·4–149)	-59·3% (-72·4 to -39·3%)	28·5% (24·2–33·4%)	4·1 (2·9–5·6)	-63·2% (-75·4 to -45%)
	Subarachnoid haemorrhage	30·9% (25·1–36·8%)	20 (13·1–28·8)	-64·2% (-78·9 to -37·9%)	27·3% (21·7–33%)	0·6 (0·4–0·9)	-68·6% (-82·6 to -42·5%)
	Neurological disorders*	5·3% (2·2–10·3%)	70·1 (26·3–160·2)	-13·5% (-30·9 to 14·6%)	10·2% (3·4–16·4%)	3·7 (0·5–11·2)	-15·6% (-38 to 29·8%)
	Alzheimer's disease and other dementias	17·1% (11·6–22·7%)	68·8 (27·1–161·7)	-17·5% (-34·3 to 6·7%)	15·4% (10·1–20·7%)	4·1 (0·9–11·7)	-15·7% (-37 to 19·1%)
	Parkinson's disease	-12·1% (-18·6–-6·1%)	-8·8 (-14·4–-4·2)	-12·8% (-38·8 to 22·4%)	-11·3% (-17·2–-5·7%)	-0·5 (-0·8–-0·2)	-12·3% (-41·1 to 25·9%)
	Idiopathic epilepsy	6·5% (4·3–9%)	7·6 (3·7–14)	37·5% (-33·7 to 202·8%)	6·9% (4·7–9·6%)	0·1 (0–0·1)	25·9% (-15·8 to 88·3%)
	Multiple sclerosis	11·1% (8·1–14·1%)	2·5 (1·6–3·4)	11·5% (-15·6 to 43·6%)	12·8% (8·9–17·5%)	<0·1 (<0·1–0·1)	9·5% (-35·8 to 75%)
Child and maternal malnutrition	Meningitis	5·9% (3·5–9·3%)	2·2 (1·2–3·7)	-78·6% (-90 to -55·1%)	3·3% (1·9–5·4%)	<0·1 (<0·1–<0·1)	-78·6% (-90 to -55·1%)
	Encephalitis	2·7% (1·3–5%)	0·5 (0·2–0·8)	-26·6% (-72·3 to 80·1%)	1·7% (0·8–3·3%)	<0·1 (<0·1–<0·1)	-26·6% (-72·3 to 80·1%)
Diet high in red meat	Stroke	3·3% (1·1–5·1%)	49 (16·6–83·4)	-28·7% (-56·4 to 10%)	2·5% (0·8–3·9%)	2 (0·6–3·6)	-30·6% (-58·6 to 4·5%)
	Ischaemic stroke	3% (0·4–4·7%)	32·4 (4·8–57·9)	7·5% (-21·3 to 40·8%)	2·3% (0·4–3·6%)	1·4 (0·2–2·7)	0% (-31·4 to 33·4%)
	Intracerebral haemorrhage	4·2% (0·5–6·9%)	13·7 (1·7–26·1)	-56·3% (-70·3 to -34·4%)	3·5% (0·4–5·8%)	0·5 (0·1–1)	-59·4% (-72·1 to -40%)
	Subarachnoid haemorrhage	4·6% (0·6–7·4%)	3 (0·4–5·5)	-59·9% (-76·2 to -31·2%)	3·7% (0·5–6·1%)	0·1 (0–0·2)	-64·7% (-80·2 to -35·8%)
Diet high in sodium	Stroke	1·8% (0·3–7·2%)	27 (3·6–109·9)	-24·7% (-53·9 to 40·3%)	1·5% (0·3–5·9%)	1·2 (0·2–4·9)	-27% (-57 to 37·2%)
	Ischaemic stroke	1·7% (0·3–6·6%)	18·5 (2·6–74·9)	8% (-39·4 to 93·3%)	1·4% (0·3–5·4%)	0·9 (0·2–3·6)	0·9% (-43·7 to 81·2%)
	Intracerebral haemorrhage	2·2% (0·3–8·7%)	7·1 (0·8–28·8)	-53·9% (-74·9 to -10%)	1·9% (0·3–7·8%)	0·3 (0–1·1)	-57·8% (-76·1 to -17·8%)
	Subarachnoid haemorrhage	2·1% (0·2–8·2%)	1·4 (0·1–5·6)	-57·4% (-79 to 0·3%)	1·9% (0·3–7·8%)	<0·1 (<0·1–0·2)	-62·2% (-81·3 to -7·5%)
Diet low in fibre	Stroke	0·8% (0·3–1·7%)	11·4 (3·6–25·3)	-51·7% (-65·7 to -26·7%)	0·7% (0·3–1·6%)	0·6 (0·2–1·3)	-52·5% (-68·6 to -23·6%)
	Ischaemic stroke	0·7% (0·3–1·6%)	8·1 (2·6–17·9)	-31·6% (-52·8 to 0·5%)	0·7% (0·3–1·5%)	0·5 (0·2–1)	-36·2% (-60 to 1%)
	Intracerebral haemorrhage	0·8% (0·3–1·9%)	2·7 (0·8–6·2)	-71·6% (-80·6 to -54·6%)	0·8% (0·3–1·7%)	0·1 (0–0·3)	-74·2% (-82·5 to -57·3%)
	Subarachnoid haemorrhage	0·9% (0·3–2·1%)	0·6 (0·2–1·4)	-73·7% (-84·4 to -50·8%)	0·8% (0·3–1·8%)	<0·1 (<0·1–<0·1)	-76·8% (-87 to -52·3%)
Diet low in fruits	Stroke	3·5% (1·5–5·8%)	51·6 (20·9–89·7)	-48·5% (-67·7 to -23%)	2·7% (1·1–4·6%)	2·2 (0·9–4)	-51·7% (-70·4 to -26·4%)
	Ischaemic stroke	2·8% (0·6–5·3%)	30·3 (5·9–62·8)	-21% (-45·2 to 16·5%)	2·2% (0·5–4·3%)	1·4 (0·3–2·9)	-28·7% (-53·7 to 8·5%)
	Intracerebral haemorrhage	5·4% (2·5–9·1%)	17·4 (7·7–32·5)	-65·3% (-77·9 to -45·4%)	4·5% (2·1–7·6%)	0·7 (0·3–1·2)	-69·1% (-81·4 to -50·2%)
	Subarachnoid haemorrhage	6% (2·7–10·4%)	3·9 (1·7–7·2)	-66·9% (-80·8 to -40·6%)	4·9% (2·3–8·2%)	0·1 (0–0·2)	-72·3% (-84·9 to -45%)
Diet low in vegetables	Stroke	0·6% (0·2–1·3%)	9 (3·2–19·6)	-77·1% (-87·6 to -49·3%)	0·7% (0·2–1·3%)	0·5 (0·2–1·1)	-71·8% (-84·7 to -43·1%)
	Ischaemic stroke	0·6% (0·2–1·3%)	6·7 (2·1–15)	-62·7% (-80·4 to -5·1%)	0·7% (0·2–1·4%)	0·4 (0·1–0·9)	-56·2% (-76·6 to -11·1%)
	Intracerebral haemorrhage	0·6% (0·2–2%)	2 (0·5–6·6)	-88·8% (-95·3 to -58·3%)	0·7% (0·2–2·3%)	0·1 (0–0·3)	-87·3% (-94·5 to -60·3%)
	Subarachnoid haemorrhage	0·6% (0·2–2%)	0·4 (0·1–1·3)	-90·3% (-96·2 to -56·8%)	0·7% (0·2–2·2%)	<0·1 (<0·1–0·1)	-89·4% (-96 to -60·3%)
Diet low in whole grains	Stroke	5·8% (2·7–7·6%)	84·9 (40–125·7)	6·5% (-20·9 to 38·6%)	5·4% (2·6–7·1%)	4·3 (2·1–6·4)	-2·5% (-31·3 to 29·4%)

Table S16 Regional and national disabilities and deaths related to neurological conditions attributable to risk factors in North Africa and Middle East countries

Location<br>Risk factor (alphabetic order)	Cause	DALYs (95% UI)<br>PAF (%)<br>2019	Rate per 100,000<br>2019	Rate change (%)<br>From 1990 to 2019	Deaths (95% UI)<br>PAF (%)<br>2019	Rate per 100,000<br>2019	Rate change (%)<br>From 1990 to 2019
	Ischaemic stroke	7·8% (3·6–10·3%)	84·9 (40–125·7)	6·5% (-20·9 to 38·6%)	6·8% (3·4–8·9%)	4·3 (2·1–6·4)	-2·5% (-31·3 to 29·4%)
Dietary risks	Stroke	14·7% (10·3–19·8%)	217·8 (139·4–317)	-32·2% (-51·4 to -7%)	12·9% (9·1–17·1%)	10·3 (6·6–15·1)	-34·2% (-53·1 to -9·8%)
	Ischaemic stroke	15·5% (10–20·4%)	168·5 (103·9–248·1)	-6·1% (-31·2 to 24·6%)	13·4% (8·8–17·5%)	8·4 (5·2–12·7)	-13·3% (-39·6 to 14·6%)
	Intracerebral haemorrhage	12·6% (7·2–19%)	40·6 (21·8–65·7)	-64·8% (-76·8 to -45·6%)	11% (6·4–16·6%)	1·6 (0·9–2·6)	-68·2% (-79 to -50·9%)
	Subarachnoid haemorrhage	13·4% (7·7–19·6%)	8·7 (4·5–13·9)	-67·2% (-81·5 to -42·1%)	11·4% (6·4–17·2%)	0·3 (0·1–0·4)	-71·7% (-84·9 to -46·4%)
Environmental/occupational risk factors§	Meningitis	0·9% (0·3–1·9%)	0·4 (0·1–0·8)	-77·4% (-94·5 to -17·8%)	0·5% (0·2–1·1%)	<0·1 (<0·1–<0·1)	-77·4% (-94·5 to -17·8%)
	Encephalitis	0·4% (0·1–1%)	0·1 (0–0·2)	-24·2% (-81·6 to 188·9%)	0·3% (0·1–0·7%)	<0·1 (<0·1–<0·1)	-24·2% (-81·6 to 188·9%)
	Stroke	30·5% (24·6–36·3%)	450·1 (323·2–608·3)	-28·3% (-46·1 to -4·5%)	28% (22·3–33·7%)	22·4 (15·5–30·4)	-28·2% (-47·5 to -3·3%)
	Ischaemic stroke	29·5% (23·8–35·2%)	321·4 (228·5–434·1)	1·8% (-25·2 to 34·1%)	27·3% (21·6–33%)	17·2 (11·9–23·4)	-3·5% (-31·2 to 27·8%)
	Intracerebral haemorrhage	33·3% (26·9–39·5%)	107·3 (73·7–149·5)	-57·9% (-70·6 to -38·2%)	30·8% (24·8–36·9%)	4·5 (3·1–6·2)	-60·4% (-72·7 to -41·1%)
	Subarachnoid haemorrhage	33·3% (27–39·6%)	21·5 (14·3–30·4)	-62·4% (-77·2 to -35·4%)	31·5% (25·6–37·7%)	0·7 (0·5–1·1)	-65·3% (-79·9 to -37·2%)
High body-mass index	Stroke	30·4% (20·4–39·8%)	450 (280·8–668)	-6·6% (-32·8 to 31·4%)	19·4% (11·9–27·6%)	15·6 (8·8–24·7)	-7·1% (-35·8 to 34·9%)
	Ischaemic stroke	25·1% (16–34·2%)	273·4 (161·9–414·9)	55·1% (9·2 to 116·3%)	16% (9·3–24·2%)	10·1 (5·4–16·6)	48·4% (-1·9 to 119·9%)
	Intracerebral haemorrhage	44·8% (32·2–56·8%)	144·5 (90·6–213·4)	-40·8% (-60·2 to -11·1%)	31·8% (21–43·6%)	4·6 (2·7–7·2)	-43·6% (-62·9 to -12·8%)
	Subarachnoid haemorrhage	49·7% (36·9–61·4%)	32·1 (20·7–45·8)	-47·8% (-69·2 to -10·8%)	35·5% (24·4–47·4%)	0·8 (0·5–1·3)	-53·7% (-74·3 to -13·9%)
	Neurological disorders*	5·5% (1·6–12·5%)	72·2 (19·8–179·5)	48·9% (17·9 to 112·6%)	13·2% (4·8–25%)	4·6 (0·8–13·7)	50·6% (10·7 to 141·5%)
	Alzheimer's disease and other dementias	18% (7·5–32·7%)	72·2 (19·8–179·5)	48·9% (17·9 to 112·6%)	17·3% (7–31·5%)	4·6 (0·8–13·7)	50·6% (10·7 to 141·5%)
High fasting plasma glucose	Stroke	31·8% (19·1–52·3%)	470·3 (273·5–802·5)	17·2% (-18·8 to 69·6%)	32·4% (17·5–62·6%)	25·9 (13·6–48·4)	4·9% (-28·2 to 52·7%)
	Ischaemic stroke	33·1% (16·9–60·8%)	361·2 (182·8–667·7)	49·2% (4·9 to 113·5%)	32·8% (15·2–72%)	20·7 (9·3–43·3)	30·1% (-9·5 to 87·1%)
	Intracerebral haemorrhage	28·9% (18·2–42%)	93·1 (52–146·9)	-30·6% (-53·3 to 7·7%)	30·8% (18·9–48·3%)	4·5 (2·4–7·7)	-40·3% (-59·1 to -8·7%)
	Subarachnoid haemorrhage	24·8% (15·8–35·8%)	16 (9·1–26·2)	-36·1% (-63·1 to 22·2%)	29·5% (17·8–45·6%)	0·7 (0·4–1·2)	-45·2% (-69·4 to 9·3%)
	Neurological disorders*	4·6% (0·8–12·6%)	60·5 (11·2–188·6)	57·5% (32·1 to 108·1%)	11·6% (2·6–25·5%)	4 (0·4–14·8)	55% (21·8 to 122·8%)
	Alzheimer's disease and other dementias	15·1% (3·8–30·6%)	60·5 (11·2–188·6)	57·5% (32·1 to 108·1%)	15·2% (3·9–30·9%)	4 (0·4–14·8)	55% (21·8 to 122·8%)
High LDL cholesterol	Stroke	17·2% (9·5–29·9%)	254·4 (128·5–469·8)	16·1% (-12·4 to 56%)	15·7% (5·3–33·2%)	12·6 (3·9–28·5)	5·6% (-23·6 to 45·4%)
	Ischaemic stroke	23·3% (12·9–39·7%)	254·4 (128·5–469·8)	16·1% (-12·4 to 56%)	19·8% (6·8–41·1%)	12·6 (3·9–28·5)	5·6% (-23·6 to 45·4%)
High systolic blood pressure	Stroke	48·9% (40·2–57·2%)	723·1 (510·8–965·7)	-21·8% (-42·9 to 6·4%)	44·4% (34·7–54·6%)	35·5 (23·8–49·7)	-24·2% (-46·5 to 5·4%)
	Ischaemic stroke	46·7% (36·3–56·7%)	509·4 (351·5–692·7)	10·6% (-20·4 to 50·5%)	42·3% (31·1–54·4%)	26·7 (17–38·6)	2·8% (-29·8 to 43·4%)
	Intracerebral haemorrhage	55·2% (45·7–64·8%)	178·2 (124·7–248·1)	-53·4% (-68·3 to -30·3%)	52·2% (40·3–64·2%)	7·6 (5·1–10·7)	-57·2% (-70·7 to -36%)
	Subarachnoid haemorrhage	55% (45·6–64·6%)	35·5 (24·5–50·9)	-56·9% (-74·3 to -24·9%)	52·8% (41·7–64%)	1·2 (0·8–1·8)	-61·9% (-78·4 to -28·8%)
High temperature	Stroke	0·8% (-0·3–3·2%)	12·4 (-4·5–44)	-14·3% (-289·3 to 235·2%)	1% (-0·4–3·7%)	0·8 (-0·3–2·7)	-9·6% (-292·5 to 252·9%)
	Ischaemic stroke	0·8% (-0·3–3·1%)	8·9 (-3·2–31·4)	21·7% (-366 to 373·3%)	1% (-0·4–3·7%)	0·6 (-0·2–2·1)	18% (-347·1 to 361·1%)

Table S16 Regional and national disabilities and deaths related to neurological conditions attributable to risk factors in North Africa and Middle East countries

Location<br>Risk factor (alphabetic order)	Cause	DALYs (95% UI)<br>PAF (%)<br>2019	Rate per 100,000<br>2019	Rate change (%)<br>From 1990 to 2019	Deaths (95% UI)<br>PAF (%)<br>2019	Rate per 100,000<br>2019	Rate change (%)<br>From 1990 to 2019
	Intracerebral haemorrhage	0·9% (-0·3–3·5%)	2·9 (-1–10·6)	-49·4% (-213·3 to 110·9%)	1% (-0·4–3·7%)	0·1 (-0·1–0·5)	-51% (-210·2 to 102·1%)
	Subarachnoid haemorrhage	0·8% (-0·3–3·2%)	0·5 (-0·2–2·2)	-59·5% (-188·1 to 70·4%)	1% (-0·4–3·7%)	<0·1 (<0·1–0·1)	-57·1% (-195·8 to 93·6%)
Household air pollution from solid fuels	Meningitis	0% (0–0%)	<0·1 (<0·1–<0·1)	-99·7% (-99·9 to -98·8%)	0% (0–0%)	<0·1 (<0·1–<0·1)	-99·7% (-99·9 to -98·8%)
	Encephalitis	0% (0–0%)	<0·1 (<0·1–<0·1)	-99% (-99·8 to -95·5%)	0% (0–0%)	<0·1 (<0·1–<0·1)	-99% (-99·8 to -95·5%)
	Stroke	0·1% (0–0·2%)	1 (0·3–2·3)	-99·1% (-99·6 to -97·7%)	0·1% (0–0·1%)	<0·1 (<0·1–0·1)	-99·1% (-99·7 to -97·7%)
	Ischaemic stroke	0·1% (0–0·2%)	0·7 (0·2–1·6)	-98·7% (-99·5 to -96·7%)	0·1% (0–0·1%)	<0·1 (<0·1–0·1)	-98·8% (-99·5 to -96·9%)
	Intracerebral haemorrhage	0·1% (0–0·2%)	0·2 (0·1–0·6)	-99·5% (-99·8 to -98·6%)	0·1% (0–0·2%)	<0·1 (<0·1–<0·1)	-99·5% (-99·8 to -98·7%)
	Subarachnoid haemorrhage	0·1% (0–0·2%)	0·1 (0–0·1)	-99·5% (-99·8 to -98·6%)	0·1% (0–0·2%)	<0·1 (<0·1–<0·1)	-99·6% (-99·9 to -98·7%)
Kidney dysfunction	Stroke	11·5% (8·6–14·2%)	169·5 (117·6–232·5)	14·4% (-17·3 to 53·7%)	10·6% (6·5–14·3%)	8·4 (5–12·4)	8·9% (-24·4 to 48·7%)
	Ischaemic stroke	12·5% (8·6–15·9%)	136·1 (89·4–190)	49% (10·5 to 96·5%)	10·7% (5·6–15·2%)	6·7 (3·5–10·5)	39·5% (-2·9 to 90·1%)
	Intracerebral haemorrhage	10·4% (8·3–12·5%)	33·4 (23·2–46·7)	-41·2% (-59·2 to -12·6%)	11·8% (8·6–14·8%)	1·7 (1·1–2·4)	-41·7% (-59·5 to -15%)
Lead exposure	Stroke	4·1% (2·2–6·1%)	60·6 (29·7–97·9)	-40·8% (-59·1 to -20%)	4·3% (2·4–6·4%)	3·4 (1·8–5·5)	-33·2% (-53·4 to -9·6%)
	Ischaemic stroke	4·1% (2·2–6·2%)	44·6 (21·2–72·8)	-10·3% (-36 to 17·9%)	4·1% (2·3–6·3%)	2·6 (1·3–4·4)	-5% (-32·5 to 25·5%)
	Intracerebral haemorrhage	4·2% (2–6·8%)	13·7 (6–23·6)	-68·6% (-79·9 to -53·5%)	4·8% (2·5–7·5%)	0·7 (0·3–1·2)	-65·5% (-76·2 to -50·1%)
	Subarachnoid haemorrhage	3·6% (1·5–6·1%)	2·4 (0·9–4·4)	-74% (-85·9 to -54·9%)	4·5% (2·3–7·1%)	0·1 (0–0·2)	-70·7% (-83·5 to -46·5%)
Low birth weight	Meningitis	5·3% (3·1–8·4%)	2 (1·1–3·3)	-79·3% (-90·4 to -56·3%)	3% (1·7–4·8%)	<0·1 (<0·1–<0·1)	-79·3% (-90·4 to -56·3%)
	Encephalitis	2·4% (1·1–4·5%)	0·4 (0·2–0·7)	-29·8% (-74·1 to 78·4%)	1·5% (0·7–2·9%)	<0·1 (<0·1–<0·1)	-29·8% (-74·1 to 78·4%)
Low birth weight and short gestation	Meningitis	5·9% (3·5–9·3%)	2·2 (1·2–3·7)	-78·6% (-90 to -55·1%)	3·3% (1·9–5·4%)	<0·1 (<0·1–<0·1)	-78·6% (-90 to -55·1%)
	Encephalitis	2·7% (1·3–5%)	0·5 (0·2–0·8)	-26·6% (-72·3 to 80·1%)	1·7% (0·8–3·3%)	<0·1 (<0·1–<0·1)	-26·6% (-72·3 to 80·1%)
Low physical activity	Stroke	2·8% (0·4–7·6%)	40·6 (6·1–115·4)	19·1% (-13 to 59·9%)	3·5% (0·6–9·5%)	2·8 (0·5–7·7)	13·2% (-18·6 to 57·2%)
	Ischaemic stroke	3·7% (0·6–10·3%)	40·6 (6·1–115·4)	19·1% (-13 to 59·9%)	4·5% (0·7–11·9%)	2·8 (0·5–7·7)	13·2% (-18·6 to 57·2%)
Low temperature	Stroke	5·2% (1·3–9·3%)	76·3 (19–147·7)	-26·1% (-51 to 0·2%)	6% (1·5–10·8%)	4·8 (1·2–9)	-22·1% (-47·5 to 3·2%)
	Ischaemic stroke	5% (1·2–9·1%)	54·9 (13·3–105·2)	5% (-31·3 to 42·4%)	6% (1·5–10·8%)	3·8 (0·9–7·2)	1·7% (-31 to 34·6%)
	Intracerebral haemorrhage	5·6% (1·4–10·1%)	18·1 (4·6–35·6)	-56·4% (-71·7 to -37·3%)	6% (1·5–10·8%)	0·9 (0·2–1·7)	-57·9% (-72·1 to -38·4%)
	Subarachnoid haemorrhage	5·1% (1·2–9·3%)	3·3 (0·9–6·7)	-65·1% (-80·2 to -38%)	6% (1·5–10·8%)	0·1 (0–0·3)	-62·9% (-79·6 to -34·1%)
Metabolic risk factors†	Stroke	73·8% (65·7–82·3%)	1091·1 (811·6–1428)	-15·4% (-38·1 to 11·9%)	69·5% (58·8–82·8%)	55·6 (39·6–74·9)	-18% (-41·2 to 9·9%)
	Ischaemic stroke	73·8% (64·1–85·4%)	804·8 (592·3–1053·9)	14·9% (-14·8 to 50·3%)	69% (56·1–85·7%)	43·6 (30·3–60·4)	6·7% (-23·7 to 42·8%)
	Intracerebral haemorrhage	74·5% (66·6–81·4%)	240·5 (172·1–323·7)	-50·7% (-65·9 to -27·6%)	71·8% (62·5–81·3%)	10·4 (7·5–14·1)	-54·7% (-68·4 to -32·9%)
	Subarachnoid haemorrhage	71% (62·3–78·4%)	45·8 (31·9–63·9)	-54·6% (-72·4 to -21·7%)	68·6% (58·5–77·9%)	1·6 (1–2·3)	-59·9% (-76·9 to -24·8%)
	Neurological disorders*	9·3% (3·2–20%)	121·9 (36·1–303·1)	47·8% (20·1 to 99·6%)	22·8% (9·3–38·7%)	7·9 (1·4–23·5)	47·8% (12·5 to 120·7%)
	Alzheimer's disease and other dementias	30·4% (14·6–48·7%)	121·9 (36·1–303·1)	47·8% (20·1 to 99·6%)	29·9% (13·9–47·9%)	7·9 (1·4–23·5)	47·8% (12·5 to 120·7%)
Non-optimal temperature	Stroke	6% (2·8–9·5%)	88·1 (39–152·7)	-24·7% (-50·7 to 7·1%)	6·9% (3·2–11·1%)	5·5 (2·4–9·3)	-20·7% (-49·1 to 10%)

Table S16 Regional and national disabilities and deaths related to neurological conditions attributable to risk factors in North Africa and Middle East countries

**Location**<br>Risk factor (alphabetic order)	Cause	**DALYs (95% UI)**<br>PAF (%)<br>2019	Rate per 100,000<br>2019	Rate change (%)<br>From 1990 to 2019	**Deaths (95% UI)**<br>PAF (%)<br>2019	Rate per 100,000<br>2019	Rate change (%)<br>From 1990 to 2019
	Ischaemic stroke	5·8% (2·7–9·3%)	63·4 (27·8–110·1)	6·9% (-32·8 to 52·8%)	6·9% (3·2–11·1%)	4·4 (1·9–7·4)	3·6% (-34·6 to 42·7%)
	Intracerebral haemorrhage	6·5% (3–10·4%)	20·9 (9·2–36·6)	-55·6% (-71 to -33%)	6·9% (3·2–11·1%)	1 (0·4–1·7)	-57·1% (-71 to -33·8%)
	Subarachnoid haemorrhage	5·9% (2·7–9·4%)	3·8 (1·6–6·9)	-64·4% (-80·3 to -35·4%)	6·9% (3·2–11·1%)	0·2 (0·1–0·3)	-62·2% (-79·3 to -29·6%)
Other environmental risks	Stroke	4·1% (2·2–6·1%)	60·6 (29·7–97·9)	-40·8% (-59·1 to -20%)	4·3% (2·4–6·4%)	3·4 (1·8–5·5)	-33·2% (-53·4 to -9·6%)
	Ischaemic stroke	4·1% (2·2–6·2%)	44·6 (21·2–72·8)	-10·3% (-36 to 17·9%)	4·1% (2·3–6·3%)	2·6 (1·3–4·4)	-5% (-32·5 to 25·5%)
	Intracerebral haemorrhage	4·2% (2–6·8%)	13·7 (6–23·6)	-68·6% (-79·9 to -53·5%)	4·8% (2·5–7·5%)	0·7 (0·3–1·2)	-65·5% (-76·2 to -50·1%)
	Subarachnoid haemorrhage	3·6% (1·5–6·1%)	2·4 (0·9–4·4)	-74% (-85·9 to -54·9%)	4·5% (2·3–7·1%)	0·1 (0–0·2)	-70·7% (-83·5 to -46·5%)
Particulate matter pollution	Meningitis	0·9% (0·3–1·9%)	0·4 (0·1–0·8)	-77·4% (-94·5 to -17·8%)	0·5% (0·2–1·1%)	<0·1 (<0·1–<0·1)	-77·4% (-94·5 to -17·8%)
	Encephalitis	0·4% (0·1–1%)	0·1 (0–0·2)	-24·2% (-81·6 to 188·9%)	0·3% (0·1–0·7%)	<0·1 (<0·1–<0·1)	-24·2% (-81·6 to 188·9%)
	Stroke	22·9% (17·4–28·5%)	337·9 (232·8–472·5)	-27·7% (-46·3 to -3·9%)	19·3% (14·3–24·3%)	15·4 (10·1–22)	-29·9% (-49·1 to -5·2%)
	Ischaemic stroke	21·9% (16·6–27·5%)	238·8 (164·3–334·5)	2·2% (-24·2 to 34·7%)	18·5% (13·7–23·5%)	11·7 (7·8–16·7)	-5·8% (-34·5 to 25·6%)
	Intracerebral haemorrhage	25·4% (19·4–31·5%)	82·1 (54·5–115·5)	-56·9% (-70·3 to -36·3%)	22% (16·7–27·6%)	3·2 (2·1–4·5)	-60·5% (-73·1 to -41·4%)
	Subarachnoid haemorrhage	26·4% (20·5–32·5%)	17 (11·1–24·6)	-60·5% (-76·2 to -32·2%)	23% (17·5–28·5%)	0·5 (0·3–0·8)	-65·3% (-80 to -36·9%)
Secondhand smoke	Stroke	4·2% (3·2–5·3%)	62·1 (41·4–87·8)	-37·1% (-53·9 to -14·5%)	3·5% (2·6–4·4%)	2·8 (1·8–4)	-38·9% (-56 to -16·1%)
	Ischaemic stroke	3·9% (2·9–4·9%)	42·1 (28–60)	-8·6% (-34·1 to 24·9%)	3·3% (2·4–4·2%)	2·1 (1·3–3)	-17·1% (-41·6 to 14·4%)
	Intracerebral haemorrhage	5·1% (3·8–6·4%)	16·5 (10·5–24·2)	-61·3% (-74·4 to -42·8%)	4·2% (3·2–5·3%)	0·6 (0·4–0·9)	-64·5% (-76·2 to -46·8%)
	Subarachnoid haemorrhage	5·4% (4–7%)	3·5 (2·2–5·4)	-65·3% (-79·7 to -38·9%)	4·6% (3·5–5·9%)	0·1 (0·1–0·2)	-69% (-82·4 to -43·4%)
Short gestation	Meningitis	4·2% (2·5–6·7%)	1·6 (0·9–2·6)	-74·3% (-87·9 to -47·7%)	2·4% (1·3–3·9%)	<0·1 (<0·1–<0·1)	-74·3% (-87·9 to -47·7%)
	Encephalitis	1·9% (0·9–3·6%)	0·3 (0·2–0·6)	-11·7% (-66·5 to 112·4%)	1·2% (0·6–2·4%)	<0·1 (<0·1–<0·1)	-11·7% (-66·5 to 112·4%)
Smoking	Stroke	15% (13·4–16·8%)	222·5 (164·3–293·1)	-32·7% (-53 to -6·1%)	11·7% (10·2–13·3%)	9·3 (6·7–12·4)	-35·2% (-55·7 to -8·2%)
	Ischaemic stroke	14·2% (12·5–15·8%)	154·6 (113·1–205·6)	-4·5% (-33·3 to 32·4%)	10·9% (9·3–12·4%)	6·9 (4·9–9·2)	-10·8% (-41 to 26·1%)
	Intracerebral haemorrhage	17·8% (15·6–20·1%)	57·5 (40–78·9)	-59·2% (-72·8 to -38·2%)	14·7% (12·8–16·9%)	2·1 (1·5–2·9)	-62·6% (-75·4 to -42·4%)
	Subarachnoid haemorrhage	16% (11·9–19·7%)	10·4 (6·6–16·1)	-62·5% (-79 to -28·8%)	14·3% (9·9–18%)	0·3 (0·2–0·5)	-66·9% (-82·9 to -32·8%)
	Neurological disorders*	4·7% (1·6–9·7%)	62·4 (20·1–151·7)	-17·3% (-33·8 to 9·5%)	10% (3·1–16·3%)	3·6 (0·4–11·1)	-16% (-39·4 to 28·6%)
	Alzheimer's disease and other dementias	17·1% (11·6–22·7%)	68·8 (27·1–161·7)	-17·5% (-34·3 to 6·7%)	15·4% (10·1–20·7%)	4·1 (0·9–11·7)	-15·7% (-37 to 19·1%)
	Parkinson's disease	-12·1% (-18·6–-6·1%)	-8·8 (-14·4–-4·2)	-12·8% (-38·8 to 22·4%)	-11·3% (-17·2–-5·7%)	-0·5 (-0·8–-0·2)	-12·3% (-41·1 to 25·9%)
	Multiple sclerosis	11·1% (8·1–14·1%)	2·5 (1·6–3·4)	11·5% (-15·6 to 43·6%)	12·8% (8·9–17·5%)	<0·1 (<0·1–0·1)	9·5% (-35·8 to 75%)
Tobacco	Stroke	18·6% (16·8–20·5%)	275·4 (202·5–364·6)	-33·3% (-52 to -8·7%)	14·7% (13·1–16·5%)	11·8 (8·4–15·8)	-35·7% (-54·8 to -10·3%)
	Ischaemic stroke	17·5% (15·7–19·4%)	190·7 (141·5–252·2)	-5% (-32·8 to 28·6%)	13·7% (12–15·5%)	8·7 (6·2–11·7)	-12% (-39·7 to 22·2%)
	Intracerebral haemorrhage	22·1% (19·8–24·6%)	71·3 (51–97·4)	-59·4% (-72·9 to -39·8%)	18·3% (16·3–20·6%)	2·7 (1·9–3·6)	-62·8% (-75·3 to -43·5%)
	Subarachnoid haemorrhage	20·7% (16·8–24·4%)	13·4 (8·6–20)	-63% (-78·4 to -34·8%)	18·3% (14·2–21·9%)	0·4 (0·3–0·7)	-67·2% (-82·1 to -37·4%)
	Neurological disorders*	4·7% (1·6–9·7%)	62·4 (20·1–151·7)	-17·3% (-33·8 to 9·5%)	10% (3·1–16·3%)	3·6 (0·4–11·1)	-16% (-39·4 to 28·6%)

Table S16 Regional and national disabilities and deaths related to neurological conditions attributable to risk factors in North Africa and Middle East countries

Location<br>Risk factor (alphabetic order)	Cause	DALYs (95% UI)<br>PAF (%)<br>2019	Rate per 100,000<br>2019	Rate change (%)<br>From 1990 to 2019	Deaths (95% UI)<br>PAF (%)<br>2019	Rate per 100,000<br>2019	Rate change (%)<br>From 1990 to 2019
	Alzheimer's disease and other dementias	17·1% (11·6–22·7%)	68·8 (27·1–161·7)	-17·5% (-34·3 to 6·7%)	15·4% (10·1–20·7%)	4·1 (0·9–11·7)	-15·7% (-37 to 19·1%)
	Parkinson's disease	-12·1% (-18·6–-6·1%)	-8·8 (-14·4–-4·2)	-12·8% (-38·8 to 22·4%)	-11·3% (-17·2–-5·7%)	-0·5 (-0·8–-0·2)	-12·3% (-41·1 to 25·9%)
	Multiple sclerosis	11·1% (8·1–14·1%)	2·5 (1·6–3·4)	11·5% (-15·6 to 43·6%)	12·8% (8·9–17·5%)	<0·1 (<0·1–0·1)	9·5% (-35·8 to 75%)
**Türkiye**							
Air pollution	Meningitis	0·2% (0·1–0·5%)	0·1 (0–0·1)	-93·8% (-98·2 to -78·9%)	0·2% (0·1–0·3%)	<0·1 (<0·1–<0·1)	-93·8% (-98·2 to -78·9%)
	Encephalitis	0·1% (0–0·3%)	<0·1 (<0·1–0·1)	-58·4% (-91·2 to 62·5%)	0·1% (0–0·2%)	<0·1 (<0·1–<0·1)	-58·4% (-91·2 to 62·3%)
	Stroke	20·4% (17·2–23·8%)	237·9 (183·1–302·9)	-31·9% (-50·9 to -12·3%)	17·1% (14·3–20·1%)	10·4 (7·7–13·6)	-27·2% (-48·3 to -4·6%)
	Ischaemic stroke	18·9% (15·8–22·1%)	125·1 (95·5–157·4)	-18·8% (-41·8 to 3·5%)	15·8% (13·1–18·7%)	6·1 (4·5–7·9)	-14·7% (-41·6 to 15%)
	Intracerebral haemorrhage	22·1% (18·7–25·7%)	88·3 (66·8–114·8)	-42% (-59·6 to -23·1%)	19·1% (16–22·3%)	3·6 (2·6–4·6)	-38·6% (-57·5 to -17·3%)
	Subarachnoid haemorrhage	24·2% (20·6–27·9%)	24·4 (17·9–32·5)	-42·6% (-63·8 to -2·7%)	21·1% (17·9–24·4%)	0·8 (0·6–1)	-43·7% (-66·9 to 7·8%)
Alcohol use	Stroke	1·2% (0·5–1·8%)	13·8 (6–22·7)	-26·1% (-59 to 29·8%)	0·7% (0·2–1·2%)	0·4 (0·1–0·8)	-23·3% (-66·3 to 55·2%)
	Ischaemic stroke	0% (-0·5–0·6%)	0·2 (-3–4)	-186·5% (-843·5 to 727·2%)	-0·1% (-0·5–0·3%)	0 (-0·2–0·1)	-35·8% (-582·1 to 692·5%)
	Intracerebral haemorrhage	3·4% (2–4·9%)	13·6 (7·5–20·6)	-28·3% (-56·7 to 9·3%)	2·4% (1·4–3·5%)	0·5 (0·2–0·7)	-24·5% (-54·6 to 18·7%)
	Neurological disorders*	0·7% (0·3–1·4%)	9·8 (4·2–18·1)	1·5% (-52·2 to 125·2%)	0·3% (0·1–0·6%)	0·1 (0–0·1)	-5·6% (-50·9 to 56·2%)
	Idiopathic epilepsy	4·6% (2·9–6·4%)	9·8 (4·2–18·1)	1·5% (-52·2 to 125·2%)	4·7% (2·9–6·7%)	0·1 (0–0·1)	-5·6% (-50·9 to 56·2%)
All risk factors	Meningitis	2% (1·3–3·2%)	0·5 (0·3–0·9)	-93·6% (-96·9 to -86·5%)	1·2% (0·8–2%)	<0·1 (<0·1–<0·1)	-93·6% (-96·9 to -86·5%)
	Encephalitis	0·9% (0·4–2%)	0·2 (0·1–0·4)	-58·2% (-84·1 to -4·5%)	0·6% (0·3–1·4%)	<0·1 (<0·1–<0·1)	-58·2% (-84·1 to -4·5%)
	Stroke	85·2% (81·4–89·1%)	991 (816·3–1187·1)	-21·6% (-42·2 to -2·1%)	82·3% (76·7–87·9%)	49·9 (39·8–61·1)	-15·9% (-39·3 to 7·7%)
	Ischaemic stroke	84·7% (79·3–90·7%)	561·7 (464·9–674·1)	-7·5% (-33·4 to 15·5%)	81·7% (74·2–90·4%)	31·3 (24·7–38·6)	-3·3% (-32·9 to 27·3%)
	Intracerebral haemorrhage	86·1% (82–89·9%)	343·8 (276·2–421·9)	-34·1% (-53·1 to -14·9%)	83·6% (77·3–89%)	15·6 (12·3–19·4)	-29·6% (-49·3 to -7·9%)
	Subarachnoid haemorrhage	84·6% (80·8–88·2%)	85·4 (66·2–105·7)	-36·8% (-60·3 to 10·3%)	82·9% (77·1–87·9%)	3 (2·3–3·8)	-37·9% (-62·9 to 22·7%)
	Neurological disorders*	11·5% (4·9–21·8%)	168·1 (67·4–395·1)	8·2% (-9·4 to 31·7%)	25·8% (11·5–41%)	9·7 (1·9–28·3)	9·3% (-11·6 to 46·6%)
	Alzheimer's disease and other dementias	41·2% (26·8–56·1%)	163·3 (63–389·6)	4·9% (-12 to 25%)	38·5% (24·5–53·8%)	10 (2·2–28·6)	5·6% (-14·9 to 31·9%)
	Parkinson's disease	-10·9% (-16·6–-5·3%)	-8·2 (-13·7–-4)	-41·9% (-57·4 to -21·5%)	-8·8% (-13·6–-4·2%)	-0·4 (-0·7–-0·2)	-43% (-59·6 to -20·1%)
	Idiopathic epilepsy	4·6% (2·9–6·4%)	9·8 (4·2–18·1)	1·5% (-52·2 to 125·2%)	4·7% (2·9–6·7%)	0·1 (0–0·1)	-5·6% (-50·9 to 56·2%)
	Multiple sclerosis	15·5% (10·5–19·8%)	3·2 (2·3–4·4)	-24·4% (-44·7 to -3·9%)	15·4% (10·2–19·8%)	<0·1 (<0·1–0·1)	-37·5% (-63·3 to -4·9%)
Ambient particulate matter pollution	Meningitis	0·2% (0·1–0·5%)	0·1 (0–0·1)	-91·3% (-97·6 to -69·5%)	0·2% (0·1–0·3%)	<0·1 (<0·1–<0·1)	-91·3% (-97·6 to -69·5%)
	Encephalitis	0·1% (0–0·3%)	<0·1 (<0·1–0·1)	-41·9% (-87·1 to 139·6%)	0·1% (0–0·2%)	<0·1 (<0·1–<0·1)	-41·9% (-87·2 to 139·5%)
	Stroke	20·3% (17·2–23·6%)	236·5 (182–300·9)	-9·2% (-34·9 to 22·8%)	17% (14·2–20%)	10·3 (7·7–13·5)	-2·8% (-32·8 to 34·3%)
	Ischaemic stroke	18·8% (15·7–22%)	124·4 (94·8–156·4)	8·5% (-22·1 to 46·2%)	15·7% (13–18·6%)	6 (4·4–7·9)	14·2% (-23·5 to 59·5%)
	Intracerebral haemorrhage	22% (18·6–25·6%)	87·8 (66·4–114)	-23·1% (-47·5 to 7·3%)	19% (15·9–22·2%)	3·5 (2·6–4·6)	-18·2% (-42·7 to 15·5%)

Table S16 Regional and national disabilities and deaths related to neurological conditions attributable to risk factors in North Africa and Middle East countries

Location<br>Risk factor (alphabetic order)	Cause	DALYs (95% UI)<br>PAF (%)<br>2019	Rate per 100,000<br>2019	Rate change (%)<br>From 1990 to 2019	Deaths (95% UI)<br>PAF (%)<br>2019	Rate per 100,000<br>2019	Rate change (%)<br>From 1990 to 2019
	Subarachnoid haemorrhage	24% (20·4–27·7%)	24·3 (17·8–32·4)	-23·3% (-52·4 to 39·5%)	21% (17·7–24·2%)	0·8 (0·6–1)	-24·9% (-56·4 to 53·1%)
Behavioural risk factors‡	Meningitis	2% (1·3–3·2%)	0·5 (0·3–0·8)	-93·5% (-96·9 to -86·5%)	1·2% (0·7–1·9%)	<0·1 (<0·1–<0·1)	-93·5% (-96·9 to -86·5%)
	Encephalitis	0·9% (0·4–2%)	0·2 (0·1–0·3)	-57·9% (-83·7 to -4·3%)	0·6% (0·3–1·3%)	<0·1 (<0·1–<0·1)	-57·9% (-83·7 to -4·3%)
	Stroke	33·6% (29·7–38%)	390·6 (306·9–486·9)	-32·8% (-52·9 to -14·4%)	28·1% (23·8–33·1%)	17 (12·6–21·8)	-27·9% (-50·7 to -6·4%)
	Ischaemic stroke	33·8% (28·1–40·5%)	223·8 (170·6–288·9)	-18·9% (-43·4 to 1·2%)	29·1% (23·1–36·7%)	11·2 (7·9–15·1)	-14·3% (-42·6 to 12·1%)
	Intracerebral haemorrhage	32·9% (29·1–37·3%)	131·3 (102·4–165·9)	-45·6% (-63·9 to -27·7%)	25·8% (22·2–29·8%)	4·8 (3·7–6·1)	-44·2% (-62·5 to -24·9%)
	Subarachnoid haemorrhage	35·2% (31–39·5%)	35·5 (26·7–45·7)	-44·5% (-65·8 to -3·8%)	28·8% (24·8–33·1%)	1 (0·8–1·4)	-46·8% (-68·7 to 2%)
	Neurological disorders*	4·7% (2·1–8·7%)	69 (28·6–152·4)	-18·7% (-34·4 to 7·3%)	8·2% (2·6–13·6%)	3·1 (0·5–9·1)	-23·8% (-40·2 to 13·5%)
	Alzheimer's disease and other dementias	16·2% (10·5–21·7%)	64·1 (24·9–147·8)	-24·6% (-37·9 to -8·4%)	13·2% (8·4–18%)	3·4 (0·7–9·4)	-27% (-42·2 to -7·3%)
	Parkinson's disease	-10·9% (-16·6–-5·3%)	-8·2 (-13·7–-4)	-41·9% (-57·4 to -21·5%)	-8·8% (-13·6–-4·2%)	-0·4 (-0·7–-0·2)	-43% (-59·6 to -20·1%)
	Idiopathic epilepsy	4·6% (2·9–6·4%)	9·8 (4·2–18·1)	1·5% (-52·2 to 125·2%)	4·7% (2·9–6·7%)	0·1 (0–0·1)	-5·6% (-50·9 to 56·2%)
	Multiple sclerosis	15·5% (10·5–19·8%)	3·2 (2·3–4·4)	-24·4% (-44·7 to -3·9%)	15·4% (10·2–19·8%)	<0·1 (<0·1–0·1)	-37·5% (-63·3 to -4·9%)
Child and maternal malnutrition	Meningitis	2% (1·3–3·2%)	0·5 (0·3–0·8)	-93·5% (-96·9 to -86·5%)	1·2% (0·7–1·9%)	<0·1 (<0·1–<0·1)	-93·5% (-96·9 to -86·5%)
	Encephalitis	0·9% (0·4–2%)	0·2 (0·1–0·3)	-57·9% (-83·7 to -4·3%)	0·6% (0·3–1·3%)	<0·1 (<0·1–<0·1)	-57·9% (-83·7 to -4·3%)
Diet high in red meat	Stroke	3·7% (1·4–5·7%)	43·3 (16·1–68·8)	-27·6% (-47·8 to -6·2%)	2·8% (1·1–4·4%)	1·7 (0·6–2·8)	-22·8% (-45·9 to 4·7%)
	Ischaemic stroke	3·1% (0·5–4·8%)	20·3 (3·3–33·8)	-13% (-36·2 to 10·1%)	2·3% (0·4–3·6%)	0·9 (0·1–1·5)	-7·9% (-38·4 to 25·2%)
	Intracerebral haemorrhage	4·4% (0·6–7·1%)	17·7 (2·3–30·8)	-37% (-56·6 to -18·1%)	3·6% (0·5–5·9%)	0·7 (0·1–1·2)	-33·1% (-53 to -11·7%)
	Subarachnoid haemorrhage	5·2% (0·8–8·3%)	5·3 (0·7–9·3)	-36·6% (-59·9 to 8·4%)	4·2% (0·6–6·8%)	0·2 (0–0·3)	-37·8% (-62·5 to 21%)
Diet high in sodium	Stroke	1·3% (0·3–5·5%)	15·2 (3·2–65)	-25·5% (-57·5 to 36·6%)	1·1% (0·3–4·5%)	0·7 (0·2–2·8)	-21·2% (-54·8 to 42·4%)
	Ischaemic stroke	1·1% (0·3–4·6%)	7·5 (1·8–32·1)	-11·1% (-52·3 to 66·1%)	1% (0·3–3·8%)	0·4 (0·1–1·5)	-7·5% (-50·6 to 71%)
	Intracerebral haemorrhage	1·5% (0·3–6·5%)	6·1 (1·1–27·7)	-35·8% (-65·2 to 18·4%)	1·3% (0·3–5·6%)	0·2 (0·1–1·1)	-32·4% (-62·8 to 23%)
	Subarachnoid haemorrhage	1·6% (0·3–6·8%)	1·6 (0·3–7)	-34·5% (-67·7 to 46·2%)	1·4% (0·3–6·1%)	0·1 (0–0·2)	-36·5% (-69·9 to 47·5%)
Diet low in fibre	Stroke	0·5% (0·2–0·9%)	5·6 (2·5–11·5)	-22·4% (-44·4 to 7·3%)	0·5% (0·2–1%)	0·3 (0·1–0·6)	-15·5% (-43·5 to 28%)
	Ischaemic stroke	0·5% (0·2–0·9%)	3·1 (1·5–6·3)	-7·7% (-34·2 to 29·1%)	0·5% (0·2–1%)	0·2 (0·1–0·4)	-2·7% (-38·8 to 54·9%)
	Intracerebral haemorrhage	0·5% (0·2–1%)	1·9 (0·9–4)	-34·9% (-54·7 to -6·7%)	0·5% (0·2–1%)	0·1 (0–0·2)	-29·2% (-51·8 to 7·5%)
	Subarachnoid haemorrhage	0·5% (0·2–1·1%)	0·5 (0·2–1·2)	-37·5% (-61·9 to 14·2%)	0·5% (0·2–1%)	<0·1 (<0·1–<0·1)	-38·8% (-65·2 to 31·1%)
Diet low in fruits	Stroke	1·2% (0·7–1·9%)	13·8 (7·4–22·9)	-64·1% (-75·5 to -49·2%)	0·9% (0·5–1·5%)	0·6 (0·3–1)	-61% (-76·3 to -39%)
	Ischaemic stroke	0·8% (0·3–1·6%)	5·5 (1·8–10·4)	-54% (-69·3 to -24·1%)	0·7% (0·3–1·4%)	0·3 (0·1–0·5)	-49·3% (-73·3 to -6·5%)
	Intracerebral haemorrhage	1·5% (0·8–2·5%)	6·2 (2·8–10·5)	-69·3% (-80·3 to -55·6%)	1·3% (0·6–2·2%)	0·2 (0·1–0·4)	-67·3% (-80·1 to -50·2%)
	Subarachnoid haemorrhage	2·2% (1–3·5%)	2·2 (0·9–3·7)	-66·8% (-80·3 to -42·5%)	1·6% (0·7–2·5%)	0·1 (0–0·1)	-69·3% (-82·7 to -37·6%)
Diet low in vegetables	Stroke	0·2% (0·2–0·2%)	2·4 (1·9–2·8)	-25·1% (-44·8 to -4·3%)	0·2% (0·2–0·2%)	0·1 (0·1–0·2)	-23·3% (-48·4 to 4·9%)
	Ischaemic stroke	0·2% (0·2–0·2%)	1·4 (1·1–1·7)	-13·4% (-38·9 to 12%)	0·2% (0·2–0·3%)	0·1 (0·1–0·1)	-13% (-46·2 to 22%)

Table S16 Regional and national disabilities and deaths related to neurological conditions attributable to risk factors in North Africa and Middle East countries

Location<br>Risk factor (alphabetic order)	Cause	DALYs (95% UI)<br>PAF (%)<br>2019	Rate per 100,000<br>2019	Rate change (%)<br>From 1990 to 2019	Deaths (95% UI)<br>PAF (%)<br>2019	Rate per 100,000<br>2019	Rate change (%)<br>From 1990 to 2019
	Intracerebral haemorrhage	0·2% (0·2–0·2%)	0·8 (0·6–1)	-36·3% (-55·3 to -15·9%)	0·2% (0·2–0·2%)	<0·1 (<0·1–<0·1)	-35·2% (-59·1 to -10·2%)
	Subarachnoid haemorrhage	0·2% (0·2–0·2%)	0·2 (0·2–0·2)	-38·8% (-62·8 to 8·8%)	0·2% (0·2–0·2%)	<0·1 (<0·1–<0·1)	-42·7% (-67·8 to 21·7%)
Diet low in whole grains	Stroke	5·2% (3–6·5%)	60 (34·1–82·5)	-9·5% (-33·7 to 11·7%)	5·2% (3·2–6·4%)	3·1 (1·9–4·4)	-4·7% (-34 to 24·8%)
	Ischaemic stroke	9% (5·2–11·5%)	60 (34·1–82·5)	-9·5% (-33·7 to 11·7%)	8·2% (5–10·2%)	3·1 (1·9–4·4)	-4·7% (-34 to 24·8%)
Dietary risks	Stroke	11·5% (8–15·7%)	134·1 (87·3–191·2)	-27·7% (-46·7 to -9·4%)	10·3% (7·4–13·9%)	6·3 (4·2–8·9)	-21·3% (-43·9 to 3·6%)
	Ischaemic stroke	14% (9·5–17·9%)	92·9 (59·2–126·4)	-14·2% (-36·9 to 7·6%)	12·3% (8·7–15·6%)	4·7 (3·1–6·5)	-9·3% (-37·5 to 21·1%)
	Intracerebral haemorrhage	7·9% (3·6–13·2%)	31·7 (13·6–56·8)	-46·7% (-66·1 to -28·6%)	6·8% (3–11·4%)	1·3 (0·5–2·2)	-43·2% (-63·8 to -22·6%)
	Subarachnoid haemorrhage	9·3% (4·3–14·9%)	9·4 (4·2–16·1)	-46·6% (-67·6 to -8·5%)	7·7% (3·5–12·8%)	0·3 (0·1–0·5)	-47·8% (-70·1 to -0·1%)
Environmental/occupational risk factors§	Meningitis	0·2% (0·1–0·5%)	0·1 (0–0·1)	-93·8% (-98·2 to -78·9%)	0·2% (0·1–0·3%)	<0·1 (<0·1–<0·1)	-93·8% (-98·2 to -78·9%)
	Encephalitis	0·1% (0–0·3%)	<0·1 (<0·1–0·1)	-58·4% (-91·2 to 62·5%)	0·1% (0–0·2%)	<0·1 (<0·1–<0·1)	-58·4% (-91·2 to 62·3%)
	Stroke	29·2% (25·5–33%)	339·7 (267·7–424·6)	-31·5% (-50·3 to -12·7%)	27·9% (24·4–31·5%)	16·9 (12·9–21·3)	-24·8% (-46·8 to -2·8%)
	Ischaemic stroke	27·3% (23·8–31%)	181·3 (143·8–226·3)	-17·5% (-41·4 to 5·3%)	26·7% (23·2–30·2%)	10·2 (7·8–13·1)	-12·1% (-38·8 to 16%)
	Intracerebral haemorrhage	31·4% (27·4–35·3%)	125·4 (96·3–159)	-42·3% (-59·5 to -24·6%)	29·7% (26–33·7%)	5·6 (4·2–7)	-37·2% (-55·3 to -16·9%)
	Subarachnoid haemorrhage	32·8% (28·6–36·9%)	33·1 (24·7–42·3)	-43·9% (-64·9 to -3·1%)	31·3% (27·4–35·2%)	1·1 (0·8–1·5)	-43·6% (-66·7 to 10·1%)
High body-mass index	Stroke	35·5% (24·6–44·9%)	413 (275·9–564·1)	-11% (-35·2 to 14·4%)	23·3% (14·8–32%)	14·1 (8·6–20·7)	-4·3% (-33·3 to 28·6%)
	Ischaemic stroke	26·4% (17·2–35·7%)	175 (110·8–248·9)	13·2% (-18·4 to 47·4%)	16·8% (9·4–25·4%)	6·4 (3·4–10·2)	22·3% (-21·2 to 70·3%)
	Intracerebral haemorrhage	45·9% (32·1–57·8%)	183·2 (122·8–258·2)	-22·8% (-46·6 to 1·9%)	33·2% (21·9–45%)	6·2 (3·9–9·2)	-17·9% (-43·6 to 12·6%)
	Subarachnoid haemorrhage	54·3% (40·3–65·6%)	54·8 (37·2–75)	-23·9% (-51·7 to 27·3%)	41·1% (28·5–52·8%)	1·5 (1–2·2)	-23·4% (-54·2 to 41%)
	Neurological disorders*	5·8% (1·8–13%)	84·4 (23·5–206·2)	36·6% (11·8 to 80·2%)	14·4% (4·9–27·6%)	5·3 (0·9–15·7)	35·2% (5·3 to 85·2%)
	Alzheimer's disease and other dementias	21·3% (9–37·4%)	84·4 (23·5–206·2)	36·6% (11·8 to 80·2%)	20·6% (8·6–36·4%)	5·3 (0·9–15·7)	35·2% (5·3 to 85·2%)
High fasting plasma glucose	Stroke	22·2% (13·7–36%)	259 (153·1–425·8)	25·4% (-15·5 to 76·3%)	23·8% (13·3–44·4%)	14·4 (7·7–26·7)	37·4% (-10·7 to 99·2%)
	Ischaemic stroke	24·1% (12–47·1%)	159·8 (77–316·1)	47·5% (-2·2 to 108·7%)	24·7% (10·9–56%)	9·5 (4·1–21·8)	57·7% (-0·7 to 132·5%)
	Intracerebral haemorrhage	20·6% (12·7–31·1%)	82·1 (48·4–126·5)	3·3% (-32·2 to 50·2%)	22·5% (13–37·5%)	4·2 (2·3–7·1)	13·3% (-27 to 65·6%)
	Subarachnoid haemorrhage	17% (11–24·6%)	17·1 (10·4–25·8)	-8·4% (-45·5 to 75·1%)	20·6% (12·5–32·6%)	0·7 (0·4–1·3)	-4·4% (-46 to 102·7%)
	Neurological disorders*	2·9% (0·5–8·1%)	42·2 (7·2–138·5)	39% (19·8 to 68·1%)	7·4% (1·4–17·7%)	2·7 (0·3–10·3)	38·1% (12·9 to 74·1%)
	Alzheimer's disease and other dementias	10·6% (2·5–22·5%)	42·2 (7·2–138·5)	39% (19·8 to 68·1%)	10·5% (2·5–22·4%)	2·7 (0·3–10·3)	38·1% (12·9 to 74·1%)
High LDL cholesterol	Stroke	11·5% (5·9–20·8%)	133·3 (65·4–251·6)	-18·9% (-41·5 to 0·4%)	10·9% (3·2–24·3%)	6·6 (1·8–15·8)	-13·7% (-43·1 to 12·3%)
	Ischaemic stroke	20·1% (10·3–36·3%)	133·3 (65·4–251·6)	-18·9% (-41·5 to 0·4%)	17·3% (5·1–38·5%)	6·6 (1·8–15·8)	-13·7% (-43·1 to 12·3%)
High systolic blood pressure	Stroke	52·8% (44·6–60·5%)	614·5 (476·4–763·7)	-23·9% (-44·4 to -4·9%)	49·2% (39·7–58·8%)	29·8 (22–38·6)	-19·5% (-41·7 to 3·7%)
	Ischaemic stroke	49·3% (39–59·2%)	326·8 (242–422·3)	-9·9% (-35·1 to 13·2%)	45·3% (33·1–58·2%)	17·4 (11·9–24·2)	-6·5% (-35·9 to 23·6%)
	Intracerebral haemorrhage	57·6% (47·1–66·9%)	230 (169·7–294·6)	-34·9% (-54·7 to -15·5%)	55·5% (42·6–67·3%)	10·4 (7·4–13·6)	-31·4% (-51·7 to -9·9%)

Table S16 Regional and national disabilities and deaths related to neurological conditions attributable to risk factors in North Africa and Middle East countries

Location<br>Risk factor (alphabetic order)	Cause	DALYs (95% UI)<br>PAF (%)<br>2019	Rate per 100,000<br>2019	Rate change (%)<br>From 1990 to 2019	Deaths (95% UI)<br>PAF (%)<br>2019	Rate per 100,000<br>2019	Rate change (%)<br>From 1990 to 2019
	Subarachnoid haemorrhage	57·1% (47·3–66·2%)	57·7 (42·9–74)	-37% (-61 to 13%)	56·6% (45·3–66·6%)	2 (1·4–2·7)	-38·5% (-64·4 to 23·1%)
High temperature	Stroke	0% (-0·1–0·1%)	0·3 (-0·9–1·7)	34·5% (-322·8 to 234·2%)	0% (-0·1–0·2%)	0 (-0·1–0·1)	51·1% (-363·7 to 270·1%)
	Ischaemic stroke	0% (-0·1–0·1%)	0·2 (-0·5–0·9)	66·1% (-372·1 to 305·7%)	0% (-0·1–0·2%)	<0·1 (<0·1–0·1)	74·8% (-397 to 325%)
	Intracerebral haemorrhage	0% (-0·1–0·2%)	0·1 (-0·4–0·6)	13·2% (-290·7 to 168·5%)	0% (-0·1–0·2%)	<0·1 (<0·1–<0·1)	27% (-331·4 to 211·1%)
	Subarachnoid haemorrhage	0% (-0·1–0·1%)	0 (-0·1–0·1)	0·9% (-292·1 to 200·3%)	0% (-0·1–0·2%)	<0·1 (<0·1–<0·1)	4·3% (-320·5 to 210·4%)
Household air pollution from solid fuels	Meningitis	0% (0–0%)	<0·1 (<0·1–<0·1)	-99·8% (-100 to -99·4%)	0% (0–0%)	<0·1 (<0·1–<0·1)	-99·8% (-100 to -99·4%)
	Encephalitis	0% (0–0%)	<0·1 (<0·1–<0·1)	-99% (-99·8 to -95·4%)	0% (0–0%)	<0·1 (<0·1–<0·1)	-99% (-99·8 to -95·4%)
	Stroke	0·1% (0–0·3%)	1·4 (0·4–3·6)	-98·4% (-99·4 to -96·1%)	0·1% (0–0·2%)	0·1 (0–0·2)	-98·3% (-99·4 to -95·8%)
	Ischaemic stroke	0·1% (0–0·3%)	0·7 (0·2–1·9)	-98·1% (-99·3 to -95·3%)	0·1% (0–0·2%)	<0·1 (<0·1–0·1)	-98% (-99·3 to -95%)
	Intracerebral haemorrhage	0·1% (0–0·3%)	0·5 (0·2–1·3)	-98·7% (-99·5 to -96·6%)	0·1% (0–0·3%)	<0·1 (<0·1–0·1)	-98·6% (-99·5 to -96·4%)
	Subarachnoid haemorrhage	0·1% (0–0·3%)	0·1 (0–0·4)	-98·7% (-99·6 to -96·6%)	0·1% (0–0·3%)	<0·1 (<0·1–<0·1)	-98·7% (-99·6 to -96·3%)
Kidney dysfunction	Stroke	10·5% (8–12·8%)	121·6 (86·7–160·1)	18·5% (-14·6 to 52%)	10% (6·4–13·4%)	6·1 (3·7–8·7)	27·6% (-13·1 to 68·5%)
	Ischaemic stroke	11·9% (8–15·6%)	79·2 (51·6–110·4)	35·2% (-3·4 to 72·9%)	10·1% (4·9–14·9%)	3·9 (1·8–6·1)	41·3% (-9·8 to 94%)
	Intracerebral haemorrhage	10·6% (8·4–12·9%)	42·4 (30·7–56·5)	-3·6% (-31·2 to 27·6%)	11·9% (8·6–15%)	2·2 (1·5–3·1)	9·1% (-23·4 to 46·3%)
Lead exposure	Stroke	1·9% (0·5–3·7%)	22·7 (5·5–45·3)	-44·4% (-64·6 to -27·8%)	2·2% (0·7–3·9%)	1·3 (0·4–2·5)	-31·3% (-53·6 to -11·7%)
	Ischaemic stroke	2% (0·6–3·7%)	13 (3·6–25·4)	-28·5% (-54·5 to -7·9%)	2·1% (0·7–3·9%)	0·8 (0·3–1·6)	-15·1% (-45 to 12·5%)
	Intracerebral haemorrhage	2% (0·4–4·1%)	8 (1·5–17·2)	-55·8% (-75·2 to -38·3%)	2·3% (0·6–4·5%)	0·4 (0·1–0·9)	-45·4% (-65·1 to -27·6%)
	Subarachnoid haemorrhage	1·6% (0·2–3·8%)	1·7 (0·2–3·9)	-62·6% (-83·9 to -35·4%)	2·1% (0·5–4·2%)	0·1 (0–0·2)	-55·3% (-77·3 to -13·2%)
Low birth weight	Meningitis	1·8% (1·2–2·9%)	0·5 (0·3–0·8)	-93·7% (-97 to -86·9%)	1·1% (0·7–1·8%)	<0·1 (<0·1–<0·1)	-93·7% (-97 to -86·9%)
	Encephalitis	0·8% (0·4–1·8%)	0·2 (0·1–0·3)	-59·3% (-84·5 to -8·7%)	0·6% (0·3–1·2%)	<0·1 (<0·1–<0·1)	-59·3% (-84·5 to -8·7%)
Low birth weight and short gestation	Meningitis	2% (1·3–3·2%)	0·5 (0·3–0·8)	-93·5% (-96·9 to -86·5%)	1·2% (0·7–1·9%)	<0·1 (<0·1–<0·1)	-93·5% (-96·9 to -86·5%)
	Encephalitis	0·9% (0·4–2%)	0·2 (0·1–0·3)	-57·9% (-83·7 to -4·3%)	0·6% (0·3–1·3%)	<0·1 (<0·1–<0·1)	-57·9% (-83·7 to -4·3%)
Low physical activity	Stroke	4% (0·8–9·1%)	46·4 (9·1–110·3)	6·1% (-24 to 42·7%)	5·2% (1·1–11·4%)	3·2 (0·7–7)	8·4% (-23·7 to 50·9%)
	Ischaemic stroke	7% (1·4–16%)	46·4 (9·1–110·3)	6·1% (-24 to 42·7%)	8·3% (1·8–18%)	3·2 (0·7–7)	8·4% (-23·7 to 50·9%)
Low temperature	Stroke	9·2% (6·6–11·7%)	106·6 (72·6–145·2)	-30·8% (-50·9 to -11·1%)	11% (8–14·1%)	6·7 (4·6–9)	-22% (-43·7 to 0·1%)
	Ischaemic stroke	8·6% (6·1–10·9%)	56·7 (38·7–78·1)	-14·7% (-42·8 to 12·4%)	11% (8–14·1%)	4·2 (2·9–5·7)	-10% (-38 to 18·7%)
	Intracerebral haemorrhage	10% (7·2–12·8%)	40·1 (26·9–54·9)	-42·4% (-59·3 to -24·8%)	11% (8–14·1%)	2·1 (1·4–2·8)	-35·1% (-53·2 to -14·7%)
	Subarachnoid haemorrhage	9·7% (7–12·5%)	9·8 (6·5–13·7)	-45·5% (-66·6 to 0·7%)	11% (8–14·1%)	0·4 (0·3–0·6)	-43% (-66·4 to 12·1%)
Metabolic risk factors†	Stroke	72·8% (65·6–79·5%)	846·7 (678·8–1036·9)	-17·8% (-39·2 to 2·7%)	68·9% (59·5–78·5%)	41·8 (32·3–52·6)	-12·1% (-36 to 13·5%)
	Ischaemic stroke	72·1% (62·5–82·5%)	478·1 (378·6–595·3)	-3·9% (-30·1 to 20·2%)	67·5% (54·6–82·9%)	25·8 (19·2–34·3)	0·8% (-29·9 to 33·4%)
	Intracerebral haemorrhage	74·3% (66·3–80·8%)	296·4 (233·9–366·2)	-30·1% (-50·8 to -9·8%)	71·8% (62–80·3%)	13·4 (10·2–16·9)	-25·6% (-46·6 to -2·2%)
	Subarachnoid haemorrhage	71·5% (62·7–78·6%)	72·2 (55·1–90·8)	-33·2% (-57·9 to 17·7%)	69·7% (60·3–77·9%)	2·5 (1·8–3·2)	-34·6% (-61·8 to 28·8%)
	Neurological disorders*	8% (2·8–17%)	117·6 (36·1–290·4)	34·5% (13·2 to 67·1%)	20·2% (7·6–34·8%)	7·5 (1·4–22·2)	33·2% (6·8 to 73·9%)

Table S16 Regional and national disabilities and deaths related to neurological conditions attributable to risk factors in North Africa and Middle East countries

Location<br>Risk factor (alphabetic order)	Cause	DALYs (95% UI)<br>PAF (%)<br>2019	Rate per 100,000<br>2019	Rate change (%)<br>From 1990 to 2019	Deaths (95% UI)<br>PAF (%)<br>2019	Rate per 100,000<br>2019	Rate change (%)<br>From 1990 to 2019
	Alzheimer's disease and other dementias	29·6% (15·2–46·8%)	117·6 (36·1–290·4)	34·5% (13·2 to 67·1%)	28·9% (14·5–46·1%)	7·5 (1·4–22·2)	33·2% (6·8 to 73·9%)
Non-optimal temperature	Stroke	9·2% (6·6–11·7%)	106·9 (73·3–145·7)	-30·7% (-51 to -11%)	11% (8–14·1%)	6·7 (4·6–9)	-21·9% (-43·7 to 0·1%)
	Ischaemic stroke	8·6% (6·2–10·9%)	56·9 (39·2–78·1)	-14·6% (-42·9 to 12·4%)	11% (8–14·1%)	4·2 (2·9–5·7)	-9·9% (-38 to 18·7%)
	Intracerebral haemorrhage	10·1% (7·2–12·8%)	40·2 (27–54·9)	-42·4% (-59·2 to -24·7%)	11% (8–14·1%)	2·1 (1·4–2·8)	-35% (-53·2 to -14·8%)
	Subarachnoid haemorrhage	9·8% (7–12·5%)	9·9 (6·5–13·7)	-45·5% (-66·6 to 0·9%)	11% (8–14·1%)	0·4 (0·3–0·6)	-42·9% (-66·2 to 12·4%)
Other environmental risks	Stroke	1·9% (0·5–3·7%)	22·7 (5·5–45·3)	-44·4% (-64·6 to -27·8%)	2·2% (0·7–3·9%)	1·3 (0·4–2·5)	-31·3% (-53·6 to -11·7%)
	Ischaemic stroke	2% (0·6–3·7%)	13 (3·6–25·4)	-28·5% (-54·5 to -7·9%)	2·1% (0·7–3·9%)	0·8 (0·3–1·6)	-15·1% (-45 to 12·5%)
	Intracerebral haemorrhage	2% (0·4–4·1%)	8 (1·5–17·2)	-55·8% (-75·2 to -38·3%)	2·3% (0·6–4·5%)	0·4 (0·1–0·9)	-45·4% (-65·1 to -27·6%)
	Subarachnoid haemorrhage	1·6% (0·2–3·8%)	1·7 (0·2–3·9)	-62·6% (-83·9 to -35·4%)	2·1% (0·5–4·2%)	0·1 (0–0·2)	-55·3% (-77·3 to -13·2%)
Particulate matter pollution	Meningitis	0·2% (0·1–0·5%)	0·1 (0–0·1)	-93·8% (-98·2 to -78·9%)	0·2% (0·1–0·3%)	<0·1 (<0·1–<0·1)	-93·8% (-98·2 to -78·9%)
	Encephalitis	0·1% (0–0·3%)	<0·1 (<0·1–0·1)	-58·4% (-91·2 to 62·5%)	0·1% (0–0·2%)	<0·1 (<0·1–<0·1)	-58·4% (-91·2 to 62·3%)
	Stroke	20·4% (17·2–23·8%)	237·9 (183·1–302·9)	-31·9% (-50·9 to -12·3%)	17·1% (14·3–20·1%)	10·4 (7·7–13·6)	-27·2% (-48·3 to -4·6%)
	Ischaemic stroke	18·9% (15·8–22·1%)	125·1 (95·5–157·4)	-18·8% (-41·8 to 3·5%)	15·8% (13·1–18·7%)	6·1 (4·5–7·9)	-14·7% (-41·6 to 15%)
	Intracerebral haemorrhage	22·1% (18·7–25·7%)	88·3 (66·8–114·8)	-42% (-59·6 to -23·1%)	19·1% (16–22·3%)	3·6 (2·6–4·6)	-38·6% (-57·5 to -17·3%)
	Subarachnoid haemorrhage	24·2% (20·6–27·9%)	24·4 (17·9–32·5)	-42·6% (-63·8 to -2·7%)	21·1% (17·9–24·4%)	0·8 (0·6–1)	-43·7% (-66·9 to 7·8%)
Secondhand smoke	Stroke	3·5% (2·6–4·4%)	40·8 (29–54·9)	-46·4% (-60 to -30·8%)	3·1% (2·3–3·9%)	1·9 (1·3–2·5)	-43·8% (-59·5 to -26·2%)
	Ischaemic stroke	3·1% (2·3–3·9%)	20·4 (14·4–27·4)	-36·7% (-54·5 to -18·2%)	2·8% (2–3·6%)	1·1 (0·7–1·5)	-35·3% (-55·3 to -12%)
	Intracerebral haemorrhage	4% (2·9–5%)	15·9 (11·1–22)	-53·2% (-66·4 to -38·5%)	3·5% (2·6–4·4%)	0·6 (0·4–0·9)	-51·5% (-64·7 to -35·1%)
	Subarachnoid haemorrhage	4·5% (3·4–5·8%)	4·5 (3·2–6·3)	-54·4% (-70·7 to -21·8%)	4% (3–5%)	0·1 (0·1–0·2)	-55·6% (-73·2 to -15·2%)
Short gestation	Meningitis	1·5% (0·9–2·4%)	0·4 (0·2–0·6)	-92·2% (-96·3 to -83·8%)	0·9% (0·6–1·4%)	<0·1 (<0·1–<0·1)	-92·2% (-96·3 to -83·8%)
	Encephalitis	0·7% (0·3–1·4%)	0·1 (0·1–0·3)	-49·7% (-80·9 to 13·9%)	0·5% (0·2–1%)	<0·1 (<0·1–<0·1)	-49·7% (-80·9 to 13·9%)
Smoking	Stroke	18·5% (16·9–20·2%)	215·4 (175·1–260·6)	-40·3% (-59 to -21·3%)	12·3% (10·9–13·7%)	7·4 (5·9–9·2)	-39·8% (-59·9 to -18·7%)
	Ischaemic stroke	15·1% (13·6–16·6%)	99·9 (81·9–121·4)	-30·2% (-53·1 to -9·4%)	9·6% (8·4–10·9%)	3·7 (2·9–4·6)	-30·2% (-57 to -3%)
	Intracerebral haemorrhage	22·4% (20·4–24·4%)	89·4 (71·3–111·1)	-47·7% (-66·1 to -29·1%)	16·2% (14·4–18·1%)	3 (2·4–3·8)	-47% (-66 to -27·6%)
	Subarachnoid haemorrhage	25·9% (23·3–28·6%)	26·1 (19·8–33·1)	-43·6% (-66 to -0·9%)	20·2% (17·9–22·7%)	0·7 (0·5–0·9)	-46·1% (-69·1 to 3·6%)
	Neurological disorders*	4% (1·5–8·3%)	59·2 (20·2–143·6)	-21·3% (-35·1 to -0·3%)	7·9% (2·2–13·5%)	3 (0·4–9)	-24·2% (-41·7 to 12·7%)
	Alzheimer's disease and other dementias	16·2% (10·5–21·7%)	64·1 (24·9–147·8)	-24·6% (-37·9 to -8·4%)	13·2% (8·4–18%)	3·4 (0·7–9·4)	-27% (-42·2 to -7·3%)
	Parkinson's disease	-10·9% (-16·6–-5·3%)	-8·2 (-13·7–-4)	-41·9% (-57·4 to -21·5%)	-8·8% (-13·6–-4·2%)	-0·4 (-0·7–-0·2)	-43% (-59·6 to -20·1%)
	Multiple sclerosis	15·5% (10·5–19·8%)	3·2 (2·3–4·4)	-24·4% (-44·7 to -3·9%)	15·4% (10·2–19·8%)	<0·1 (<0·1–0·1)	-37·5% (-63·3 to -4·9%)
Tobacco	Stroke	21·3% (19·6–23%)	247·4 (201·1–298)	-40·6% (-58·4 to -23·1%)	14·9% (13·4–16·5%)	9 (7·1–11·2)	-40% (-58·8 to -20·2%)
	Ischaemic stroke	17·6% (16–19·2%)	116·7 (95·1–141·2)	-30·7% (-52·7 to -10·7%)	12% (10·7–13·5%)	4·6 (3·6–5·8)	-30·8% (-56·1 to -5·5%)

Table S16 Regional and national disabilities and deaths related to neurological conditions attributable to risk factors in North Africa and Middle East countries

**Location** Risk factor (alphabetic order)	Cause	**DALYs (95% UI)** PAF (%) 2019	Rate per 100,000 2019	Rate change (%) From 1990 to 2019	**Deaths (95% UI)** PAF (%) 2019	Rate per 100,000 2019	Rate change (%) From 1990 to 2019
	Intracerebral haemorrhage	25·4% (23·2–27·5%)	101·3 (81·1–125·6)	-48·1% (-65·2 to -30·6%)	19% (17·1–21%)	3·6 (2·8–4·5)	-47·3% (-65 to -29%)
	Subarachnoid haemorrhage	29·1% (26·4–31·6%)	29·4 (22·5–37·2)	-44·8% (-66 to -2·9%)	23·3% (20·8–25·7%)	0·8 (0·6–1·1)	-47·3% (-69·2 to 3%)
	Neurological disorders*	4% (1·5–8·3%)	59·2 (20·2–143·6)	-21·3% (-35·1 to -0·3%)	7·9% (2·2–13·5%)	3 (0·4–9)	-24·2% (-41·7 to 12·7%)
	Alzheimer's disease and other dementias	16·2% (10·5–21·7%)	64·1 (24·9–147·8)	-24·6% (-37·9 to -8·4%)	13·2% (8·4–18%)	3·4 (0·7–9·4)	-27% (-42·2 to -7·3%)
	Parkinson's disease	-10·9% (-16·6–-5·3%)	-8·2 (-13·7–-4)	-41·9% (-57·4 to -21·5%)	-8·8% (-13·6–-4·2%)	-0·4 (-0·7–-0·2)	-43% (-59·6 to -20·1%)
	Multiple sclerosis	15·5% (10·5–19·8%)	3·2 (2·3–4·4)	-24·4% (-44·7 to -3·9%)	15·4% (10·2–19·8%)	<0·1 (<0·1–0·1)	-37·5% (-63·3 to -4·9%)
**United Arab Emirates**							
Air pollution	Meningitis	0·3% (0·1–0·7%)	0·1 (0–0·2)	-92·7% (-97·6 to -80·6%)	0·1% (0–0·3%)	<0·1 (<0·1–<0·1)	-92·7% (-97·6 to -80·6%)
	Encephalitis	0·4% (0·1–0·9%)	0·1 (0–0·1)	-32·4% (-81·4 to 130·9%)	0·2% (0·1–0·5%)	<0·1 (<0·1–<0·1)	-32·4% (-81·4 to 130·9%)
	Stroke	30·2% (24·4–35·7%)	581·5 (421·7–780·4)	-44·3% (-58·2 to -26·9%)	25·8% (20·7–30·8%)	23·5 (16·3–32·9)	-49·9% (-62·5 to -34%)
	Ischaemic stroke	29% (23·5–34·4%)	410·8 (301·2–545·8)	-31·2% (-47·6 to -11·2%)	24·7% (19·8–29·9%)	17·7 (12·4–24·3)	-40·1% (-55·3 to -21·3%)
	Intracerebral haemorrhage	33·4% (27·3–39·7%)	150·4 (99·8–226)	-62·4% (-74·7 to -45·6%)	29·4% (23·7–35·2%)	5·3 (3·4–8·1)	-66·8% (-77·6 to -51·9%)
	Subarachnoid haemorrhage	33·4% (27·3–39·6%)	20·3 (10·5–33·7)	-58% (-74·3 to -30·2%)	30·2% (24·5–35·8%)	0·6 (0·3–1)	-63·3% (-78·2 to -38·3%)
Alcohol use	Stroke	0·6% (-0·3–1·6%)	12·1 (-4·9–31·8)	-87·6% (-108·7 to -68·6%)	0·3% (-0·4–1·1%)	0·3 (-0·4–1)	-91·6% (-119·8 to -72·4%)
	Ischaemic stroke	-0·2% (-1·1–0·8%)	-2·1 (-15·5–11·3)	-113·7% (-565·9 to 320·1%)	-0·2% (-0·9–0·6%)	-0·2 (-0·7–0·4)	-132·1% (-780·5 to 666·6%)
	Intracerebral haemorrhage	3·2% (1·1–5·5%)	14·3 (4·6–27·9)	-82·7% (-93·1 to -66·8%)	2·4% (0·8–4·4%)	0·4 (0·1–0·9)	-84·8% (-94·4 to -70·5%)
	Neurological disorders*	0·7% (0·3–1·3%)	9 (3·5–17·3)	-60·4% (-84 to -12·8%)	0·2% (0·1–0·6%)	0·1 (0–0·1)	-58% (-78 to -28·7%)
	Idiopathic epilepsy	4·2% (2·3–6·5%)	9 (3·5–17·3)	-60·4% (-84 to -12·8%)	5·4% (3–8·1%)	0·1 (0–0·1)	-58% (-78 to -28·7%)
All risk factors	Meningitis	1·4% (0·7–3·5%)	0·4 (0·2–1·1)	-92·9% (-96·5 to -85·7%)	0·6% (0·3–1·8%)	<0·1 (<0·1–<0·1)	-92·9% (-96·5 to -85·7%)
	Encephalitis	2·2% (1–4·1%)	0·4 (0·2–0·7)	-35·1% (-75·7 to 68·2%)	1·3% (0·6–2·6%)	<0·1 (<0·1–<0·1)	-35·1% (-75·7 to 68·2%)
	Stroke	89·3% (85·6–92·7%)	1719·6 (1350·3–2192·7)	-45·5% (-58·5 to -29·9%)	86·8% (80·9–92·3%)	79·2 (59·8–103·4)	-50·2% (-62·3 to -35·4%)
	Ischaemic stroke	89·3% (84·8–93·6%)	1263·3 (996·8–1560·3)	-33·7% (-48·9 to -15·3%)	86·5% (79·5–93·2%)	61·8 (46·4–78·8)	-41·3% (-55·2 to -23·6%)
	Intracerebral haemorrhage	89·8% (86·5–92·8%)	404·6 (285·2–600·7)	-63·9% (-75·5 to -48·2%)	87·9% (83–92·2%)	15·7 (10·8–23·9)	-68% (-77·9 to -54·3%)
	Subarachnoid haemorrhage	84·9% (79·3–89·7%)	51·7 (27–84·3)	-60% (-75·2 to -34·6%)	85·1% (79·8–90·2%)	1·7 (0·7–2·8)	-64·4% (-78·2 to -41·1%)
	Neurological disorders*	12·8% (5·2–25·7%)	183·1 (70·9–453)	11·9% (-10 to 39·7%)	28·7% (11–48·3%)	11·2 (2·1–33·5)	24·1% (-1·3 to 69·8%)
	Alzheimer's disease and other dementias	49·4% (32·3–66·4%)	181·3 (68·3–451·6)	22·5% (2·5 to 52·5%)	47·9% (30·7–65·1%)	11·6 (2·5–34)	24·4% (-0·3 to 73%)
	Parkinson's disease	-7·3% (-11·3–-3·7%)	-9·5 (-15·9–-4·5)	1·8% (-31·8 to 50·1%)	-6·8% (-10·7–-3·5%)	-0·5 (-0·9–-0·3)	1·5% (-33·1 to 51·8%)
	Idiopathic epilepsy	4·2% (2·3–6·5%)	9 (3·5–17·3)	-60·4% (-84 to -12·8%)	5·4% (3–8·1%)	0·1 (0–0·1)	-58% (-78 to -28·7%)
	Multiple sclerosis	11·2% (7·7–15·2%)	2·3 (1·2–4)	7·7% (-31·6 to 58·1%)	11·7% (7·2–16%)	<0·1 (<0·1–0·1)	18·7% (-38·3 to 91·2%)
Ambient particulate matter pollution	Meningitis	0·3% (0·1–0·7%)	0·1 (0–0·2)	-92·6% (-97·6 to -80·4%)	0·1% (0–0·3%)	<0·1 (<0·1–<0·1)	-92·6% (-97·6 to -80·4%)
	Encephalitis	0·4% (0·1–0·9%)	0·1 (0–0·1)	-31·9% (-81·2 to 132·5%)	0·2% (0·1–0·5%)	<0·1 (<0·1–<0·1)	-31·9% (-81·2 to 132·5%)

Table S16 Regional and national disabilities and deaths related to neurological conditions attributable to risk factors in North Africa and Middle East countries

Location<br>Risk factor (alphabetic order)	Cause	DALYs (95% UI)<br>PAF (%)<br>2019	Rate per 100,000<br>2019	Rate change (%)<br>From 1990 to 2019	Deaths (95% UI)<br>PAF (%)<br>2019	Rate per 100,000<br>2019	Rate change (%)<br>From 1990 to 2019
	Stroke	30·2% (24·4–35·7%)	581·5 (421·7–780·3)	-44% (-58 to -26·5%)	25·8% (20·7–30·8%)	23·5 (16·3–32·9)	-49·6% (-62·2 to -33·6%)
	Ischaemic stroke	29% (23·5–34·4%)	410·8 (301·1–545·8)	-30·7% (-47·3 to -10·8%)	24·7% (19·8–29·9%)	17·7 (12·4–24·3)	-39·7% (-54·8 to -20·7%)
	Intracerebral haemorrhage	33·4% (27·3–39·7%)	150·4 (99·8–226)	-62·2% (-74·5 to -45·2%)	29·4% (23·7–35·2%)	5·3 (3·4–8·1)	-66·6% (-77·5 to -51·3%)
	Subarachnoid haemorrhage	33·4% (27·3–39·6%)	20·3 (10·5–33·7)	-57·7% (-74·2 to -30%)	30·2% (24·5–35·8%)	0·6 (0·3–1)	-63·1% (-78 to -37·9%)
Behavioural risk factors‡	Meningitis	1·4% (0·7–3·4%)	0·4 (0·2–1·1)	-92·9% (-96·5 to -85·7%)	0·6% (0·3–1·7%)	<0·1 (<0·1–<0·1)	-92·9% (-96·5 to -85·7%)
	Encephalitis	2·1% (1–3·9%)	0·4 (0·2–0·7)	-34·6% (-75·4 to 68·7%)	1·3% (0·6–2·5%)	<0·1 (<0·1–<0·1)	-34·6% (-75·4 to 68·7%)
	Stroke	37·1% (31·1–43·4%)	714·2 (518·7–943·5)	-43·2% (-58·1 to -25·4%)	32·8% (26·3–39·4%)	29·9 (21·2–41·1)	-47·3% (-61·3 to -30·1%)
	Ischaemic stroke	37·4% (29·6–45·1%)	529·5 (384·7–704·1)	-30·6% (-47·9 to -8·3%)	33·3% (25·3–41·1%)	23·8 (16·5–32·8)	-38·2% (-53·8 to -17·7%)
	Intracerebral haemorrhage	36·4% (30·3–42·7%)	164 (108·5–250·1)	-63·4% (-76·6 to -44·9%)	31% (25·4–37·2%)	5·6 (3·6–8·8)	-66·9% (-78·7 to -50·4%)
	Subarachnoid haemorrhage	33·9% (27·5–40·5%)	20·7 (10·2–34·7)	-57% (-75·3 to -29·3%)	29·8% (23·7–36·1%)	0·6 (0·2–1)	-61·4% (-77·6 to -35·1%)
	Neurological disorders*	4·1% (1·6–8·6%)	58·7 (20·7–141·8)	-12·3% (-46·3 to 20·2%)	7·8% (1·4–14·9%)	3·1 (0·3–10)	14·2% (-23·6 to 66·9%)
	Alzheimer's disease and other dementias	15·5% (9·4–22·5%)	56·9 (21–135·5)	10·7% (-15·4 to 49%)	14·6% (8·8–21·4%)	3·5 (0·8–10·4)	16·2% (-12·9 to 68·6%)
	Parkinson's disease	-7·3% (-11·3–-3·7%)	-9·5 (-15·9–-4·5)	1·8% (-31·8 to 50·1%)	-6·8% (-10·7–-3·5%)	-0·5 (-0·9–-0·3)	1·5% (-33·1 to 51·8%)
	Idiopathic epilepsy	4·2% (2·3–6·5%)	9 (3·5–17·3)	-60·4% (-84 to -12·8%)	5·4% (3–8·1%)	0·1 (0–0·1)	-58% (-78 to -28·7%)
	Multiple sclerosis	11·2% (7·7–15·2%)	2·3 (1·2–4)	7·7% (-31·6 to 58·1%)	11·7% (7·2–16%)	<0·1 (<0·1–0·1)	18·7% (-38·3 to 91·2%)
Child and maternal malnutrition	Meningitis	1·4% (0·7–3·4%)	0·4 (0·2–1·1)	-92·9% (-96·5 to -85·7%)	0·6% (0·3–1·7%)	<0·1 (<0·1–<0·1)	-92·9% (-96·5 to -85·7%)
	Encephalitis	2·1% (1–3·9%)	0·4 (0·2–0·7)	-34·6% (-75·4 to 68·7%)	1·3% (0·6–2·5%)	<0·1 (<0·1–<0·1)	-34·6% (-75·4 to 68·7%)
Diet high in red meat	Stroke	5·8% (2·6–8·3%)	112 (49·7–173·2)	-68·7% (-82·3 to -54%)	4·4% (1·8–6·4%)	4·1 (1·7–6·4)	-71·2% (-83·4 to -58·2%)
	Ischaemic stroke	5·3% (1·3–7·8%)	74·3 (18·3–118·7)	-59·8% (-82·3 to -40·6%)	4% (1–5·9%)	2·8 (0·7–4·6)	-63·9% (-82·5 to -46·7%)
	Intracerebral haemorrhage	7·3% (1·8–11%)	33 (7·5–61)	-78·5% (-91·7 to -64·4%)	6·1% (1·4–9·3%)	1·1 (0·2–2)	-80·7% (-92 to -68·7%)
	Subarachnoid haemorrhage	7·7% (1·9–11·6%)	4·7 (1·1–9·3)	-76·2% (-90·8 to -57·9%)	6·4% (1·4–9·8%)	0·1 (0–0·3)	-79% (-91·7 to -62%)
Diet high in sodium	Stroke	2·2% (0·2–8·6%)	42·7 (4·5–164·6)	-44% (-68·5 to 0%)	1·8% (0·2–7·3%)	1·7 (0·2–6·6)	-48·6% (-72·3 to -5·5%)
	Ischaemic stroke	2% (0·2–8%)	28·7 (3·4–112·3)	-29·9% (-62·3 to 24·5%)	1·7% (0·2–6·7%)	1·2 (0·2–4·7)	-37·2% (-67·3 to 16·1%)
	Intracerebral haemorrhage	2·7% (0·2–10·3%)	12·4 (1–51·1)	-60·9% (-79·7 to -25·3%)	2·4% (0·2–9·5%)	0·4 (0–1·8)	-65·1% (-82·5 to -33·8%)
	Subarachnoid haemorrhage	2·5% (0·2–9·7%)	1·6 (0·1–6·5)	-56·5% (-79·6 to -8·8%)	2·3% (0·2–9·2%)	<0·1 (<0·1–0·2)	-61·4% (-82·7 to -19·8%)
Diet low in fibre	Stroke	1·6% (0·4–3·5%)	31·7 (7–75·3)	-26·6% (-48·2 to 6·6%)	1·5% (0·4–3%)	1·3 (0·3–3·1)	-35·5% (-55·4 to -4·8%)
	Ischaemic stroke	1·6% (0·4–3·3%)	22·2 (5–50·8)	-9·4% (-36·7 to 28·5%)	1·4% (0·4–2·9%)	1 (0·2–2·3)	-23·3% (-48·8 to 13·9%)
	Intracerebral haemorrhage	1·8% (0·4–3·9%)	8·3 (1·7–20·4)	-49·8% (-68·1 to -20%)	1·7% (0·4–3·5%)	0·3 (0·1–0·7)	-56·9% (-72·4 to -32·7%)
	Subarachnoid haemorrhage	2% (0·4–4·4%)	1·2 (0·2–3·2)	-44·4% (-66·9 to -0·8%)	1·8% (0·4–3·8%)	<0·1 (<0·1–0·1)	-51·6% (-71 to -15·3%)
Diet low in fruits	Stroke	3·5% (1·5–5·7%)	66·8 (27–118·5)	55·7% (-13·8 to 151·5%)	2·6% (1·1–4·4%)	2·4 (1–4·4)	33·1% (-22 to 127·9%)
	Ischaemic stroke	2·7% (0·5–5·4%)	39 (7·5–80·3)	119·5% (5·2 to 268·5%)	2·1% (0·4–4·1%)	1·5 (0·3–3·2)	64·6% (-20·2 to 200·4%)
	Intracerebral haemorrhage	5·4% (2·5–9·2%)	24·2 (10·5–47·9)	11·8% (-33·5 to 120·2%)	4·4% (2–7·4%)	0·8 (0·3–1·5)	0·5% (-42·2 to 97·8%)

Table S16 Regional and national disabilities and deaths related to neurological conditions attributable to risk factors in North Africa and Middle East countries

**Location**<br>Risk factor (alphabetic order)	Cause	**DALYs (95% UI)**<br>PAF (%)<br>2019	 Rate per 100,000<br>2019	 Rate change (%)<br>From 1990 to 2019	**Deaths (95% UI)**<br>PAF (%)<br>2019	 Rate per 100,000<br>2019	 Rate change (%)<br>From 1990 to 2019
	Subarachnoid haemorrhage	5·9% (2·7–10·3%)	3·6 (1·4–7·7)	3·2% (-44·6 to 102%)	4·8% (2·2–8·2%)	0·1 (0–0·2)	0·7% (-47·3 to 108·1%)
Diet low in vegetables	Stroke	2% (0·5–3·6%)	38·3 (9·4–72·7)	330·3% (24·2 to 650·5%)	1·7% (0·4–3%)	1·5 (0·4–2·9)	165·3% (-3·6 to 356·5%)
	Ischaemic stroke	1·8% (0·2–3·6%)	25·6 (3·2–51·4)	351·2% (-26 to 720·6%)	1·5% (0·2–2·9%)	1·1 (0·2–2·2)	171·3% (-34 to 401·6%)
	Intracerebral haemorrhage	2·5% (0·2–6·9%)	11·2 (0·9–33·3)	282·7% (-62·9 to 756·6%)	2·3% (0·2–6·1%)	0·4 (0–1·1)	146% (-66·4 to 396·1%)
	Subarachnoid haemorrhage	2·5% (0·2–7·1%)	1·5 (0·1–4·7)	396·2% (-58·5 to 1173·2%)	2·3% (0·2–6·3%)	<0·1 (<0·1–0·1)	222·5% (-64·6 to 609·5%)
Diet low in whole grains	Stroke	4·5% (1·2–6·5%)	85·7 (23·2–133)	-30·2% (-47·4 to -9·1%)	3·9% (1·1–5·6%)	3·6 (1–5·6)	-39·8% (-55·2 to -20·9%)
	Ischaemic stroke	6·1% (1·6–8·8%)	85·7 (23·2–133)	-30·2% (-47·4 to -9·1%)	5% (1·5–7·2%)	3·6 (1–5·6)	-39·8% (-55·2 to -20·9%)
Dietary risks	Stroke	17·8% (12·7–23·5%)	343·3 (223·8–504·8)	-43·6% (-60 to -22·9%)	14·8% (10·5–19·7%)	13·5 (8·7–20·1)	-48·4% (-63·1 to -29·5%)
	Ischaemic stroke	17·7% (11·2–23·5%)	250·1 (154·8–356·3)	-31·8% (-51·1 to -7·6%)	14·6% (9·4–19·5%)	10·4 (6·4–15·2)	-39·6% (-56·8 to -16·6%)
	Intracerebral haemorrhage	18·1% (10·8–25·7%)	81·7 (44–140·9)	-61·9% (-76·7 to -39·6%)	15·7% (9·3–22·8%)	2·8 (1·5–4·9)	-65·5% (-78·5 to -44·3%)
	Subarachnoid haemorrhage	18·8% (11·2–26%)	11·5 (5·2–20·1)	-58·2% (-76 to -27·6%)	16·3% (9·8–23·3%)	0·3 (0·1–0·6)	-62·4% (-78·8 to -31·5%)
Environmental/occupational risk factors§	Meningitis	0·3% (0·1–0·7%)	0·1 (0–0·2)	-92·7% (-97·6 to -80·6%)	0·1% (0–0·3%)	<0·1 (<0·1–<0·1)	-92·7% (-97·6 to -80·6%)
	Encephalitis	0·4% (0·1–0·9%)	0·1 (0–0·1)	-32·4% (-81·4 to 130·9%)	0·2% (0·1–0·5%)	<0·1 (<0·1–<0·1)	-32·4% (-81·4 to 130·9%)
	Stroke	37·5% (29–45·1%)	723·7 (511·4–1004·3)	-45·9% (-60·1 to -27·4%)	35·1% (25·6–43·7%)	32·1 (21·2–45·8)	-49·7% (-63·2 to -31·5%)
	Ischaemic stroke	36·3% (28–43·6%)	514·4 (365–692·6)	-33% (-50 to -10·3%)	34·2% (24·7–42·9%)	24·5 (16·1–34·8)	-40% (-56·3 to -17·4%)
	Intracerebral haemorrhage	41% (31·8–49%)	184·7 (120·4–289·9)	-63·5% (-75·5 to -46%)	38·4% (28·8–47·1%)	6·9 (4·3–11)	-67·2% (-78·3 to -51·8%)
	Subarachnoid haemorrhage	40·4% (31·7–48·3%)	24·6 (12·5–40·8)	-60·7% (-76·1 to -32·7%)	38·9% (29·4–47·6%)	0·8 (0·3–1·3)	-64% (-78·5 to -37·1%)
High body-mass index	Stroke	43% (32·6–51·8%)	828·5 (569·3–1144·4)	-28·8% (-48·8 to -0·7%)	29·2% (20·5–37·6%)	26·7 (16·8–39)	-33·4% (-52·5 to -5·3%)
	Ischaemic stroke	36·9% (26·8–45·7%)	521·9 (353·7–711·5)	-0·7% (-27·2 to 35·5%)	24·6% (16·4–33·9%)	17·6 (10·8–26·1)	-10·1% (-37 to 28·8%)
	Intracerebral haemorrhage	59·7% (47·7–69·8%)	269 (178·5–413·1)	-52·3% (-68·4 to -27·6%)	45·5% (33·6–57·3%)	8·1 (5–13·1)	-55·9% (-70·9 to -32·6%)
	Subarachnoid haemorrhage	61·8% (49·6–71·1%)	37·6 (19·5–61·9)	-49·4% (-69·6 to -15·3%)	48·5% (36·5–59·8%)	1 (0·4–1·7)	-54·4% (-73·2 to -19·3%)
	Neurological disorders*	6·6% (1·9–14·6%)	93·7 (25·6–237·2)	44·3% (5·6 to 112·7%)	15·4% (4·7–28·6%)	5·9 (1–18)	45·6% (-0·4 to 130·6%)
	Alzheimer's disease and other dementias	25·5% (11·1–42·2%)	93·7 (25·6–237·2)	44·3% (5·6 to 112·7%)	24·5% (10·4–40·8%)	5·9 (1–18)	45·6% (-0·4 to 130·6%)
High fasting plasma glucose	Stroke	30·5% (19·9–45·6%)	588·3 (353·8–922·6)	-45·2% (-60·5 to -26%)	29·9% (18–50·1%)	27·3 (14·6–46·7)	-51·4% (-65·1 to -33·6%)
	Ischaemic stroke	31·2% (18·2–51·6%)	442·3 (244·9–758·6)	-35·6% (-53·4 to -9·1%)	30% (15·8–54·2%)	21·4 (10·4–39·6)	-43·9% (-59·6 to -20·5%)
	Intracerebral haemorrhage	29·1% (19·3–40·8%)	131·1 (73·7–220·2)	-62·7% (-75·3 to -42·9%)	29·8% (19·4–44·4%)	5·3 (3–9·2)	-67·6% (-78·6 to -51·1%)
	Subarachnoid haemorrhage	24·5% (15·8–34·4%)	14·9 (6·6–26·9)	-57·9% (-74·5 to -28%)	27·8% (18·3–40·6%)	0·5 (0·2–1)	-64% (-78·6 to -37%)
	Neurological disorders*	5% (1–13·4%)	71·7 (13–221·6)	29·8% (9·7 to 67·1%)	12% (2·5–27·9%)	4·7 (0·5–16·4)	29·3% (5·3 to 81%)
	Alzheimer's disease and other dementias	19·5% (5·2–38·9%)	71·7 (13–221·6)	29·8% (9·7 to 67·1%)	19·2% (5–38·6%)	4·7 (0·5–16·4)	29·3% (5·3 to 81%)
High LDL cholesterol	Stroke	21·1% (12·9–34%)	403·7 (232·6–670·1)	-19·7% (-39·3 to 9·6%)	18·8% (7·4–36·5%)	17·1 (6·6–34·1)	-31·5% (-48·3 to -4·4%)
	Ischaemic stroke	28·6% (17·4–45·8%)	403·7 (232·6–670·1)	-19·7% (-39·3 to 9·6%)	24% (9·6–46·7%)	17·1 (6·6–34·1)	-31·5% (-48·3 to -4·4%)

Table S16 Regional and national disabilities and deaths related to neurological conditions attributable to risk factors in North Africa and Middle East countries

Location<br>Risk factor (alphabetic order)	Cause	DALYs (95% UI)<br>PAF (%)<br>2019	Rate per 100,000<br>2019	Rate change (%)<br>From 1990 to 2019	Deaths (95% UI)<br>PAF (%)<br>2019	Rate per 100,000<br>2019	Rate change (%)<br>From 1990 to 2019
High systolic blood pressure	Stroke	52·3% (43·6–60·1%)	1008·2 (732·4–1350·4)	-53% (-65 to -38·2%)	48·3% (38·7–58·6%)	44·1 (30·5–61·3)	-57·2% (-68·2 to -43·2%)
	Ischaemic stroke	50·2% (40·3–59·7%)	710·9 (520·3–916·7)	-42% (-56 to -25%)	46·2% (35·1–57·8%)	33 (22·5–45·7)	-48·7% (-61·8 to -32·4%)
	Intracerebral haemorrhage	58·5% (47·8–67·9%)	263·6 (174·8–410·7)	-68·1% (-78·7 to -53·9%)	56·2% (44·3–66·7%)	10·1 (6·5–16·5)	-71·6% (-80·7 to -59·2%)
	Subarachnoid haemorrhage	55·1% (45·3–65·3%)	33·7 (16·6–56·7)	-64·2% (-78·3 to -42%)	55·5% (44·6–65·7%)	1·1 (0·5–1·9)	-68·2% (-80·8 to -47·1%)
High temperature	Stroke	5·8% (1·7–11·8%)	112·3 (33–237·2)	-41·4% (-82·9 to -15·8%)	7·1% (2·1–14·4%)	6·5 (1·9–13·9)	-41·1% (-82·8 to -19·5%)
	Ischaemic stroke	5·6% (1·6–11·3%)	79·6 (23·4–172)	-27·7% (-79·9 to 3·4%)	7·1% (2·1–14·4%)	5·1 (1·5–10·9)	-30·6% (-80·3 to -4·7%)
	Intracerebral haemorrhage	6·4% (1·9–13·1%)	29·1 (7·9–61·2)	-59·6% (-88 to -33·7%)	7·1% (2·1–14·4%)	1·3 (0·4–2·7)	-62·2% (-88·4 to -40·5%)
	Subarachnoid haemorrhage	6% (1·8–12·2%)	3·7 (1–9)	-61·3% (-89·4 to -25·5%)	7·1% (2·1–14·4%)	0·1 (0–0·3)	-58·9% (-88·1 to -24·7%)
Household air pollution from solid fuels	Meningitis	0% (0–0%)	<0·1 (<0·1–<0·1)	-99·9% (-100 to -99·8%)	0% (0–0%)	<0·1 (<0·1–<0·1)	-99·9% (-100 to -99·8%)
	Encephalitis	0% (0–0%)	<0·1 (<0·1–<0·1)	-99·5% (-99·9 to -98%)	0% (0–0%)	<0·1 (<0·1–<0·1)	-99·5% (-99·9 to -98%)
	Stroke	0% (0–0%)	<0·1 (<0·1–0·1)	-99·6% (-99·9 to -98·9%)	0% (0–0%)	<0·1 (<0·1–<0·1)	-99·6% (-99·9 to -99%)
	Ischaemic stroke	0% (0–0%)	<0·1 (<0·1–0·1)	-99·5% (-99·8 to -98·7%)	0% (0–0%)	<0·1 (<0·1–<0·1)	-99·6% (-99·9 to -98·9%)
	Intracerebral haemorrhage	0% (0–0%)	<0·1 (<0·1–<0·1)	-99·7% (-99·9 to -99·3%)	0% (0–0%)	<0·1 (<0·1–<0·1)	-99·8% (-99·9 to -99·3%)
	Subarachnoid haemorrhage	0% (0–0%)	<0·1 (<0·1–<0·1)	-99·7% (-99·9 to -99·2%)	0% (0–0%)	<0·1 (<0·1–<0·1)	-99·7% (-99·9 to -99·3%)
Kidney dysfunction	Stroke	13% (10·2–15·8%)	251·4 (174·6–342·6)	-28·1% (-45·5 to -6·6%)	12·1% (7·9–15·6%)	11 (6·6–16·3)	-34·7% (-52·5 to -12·2%)
	Ischaemic stroke	14·3% (10·6–17·8%)	202·2 (136·8–278·4)	-13·9% (-34·1 to 10·8%)	12·3% (7·1–16·9%)	8·8 (4·8–13·4)	-23·2% (-43·6 to 2·1%)
	Intracerebral haemorrhage	10·9% (8·8–13·4%)	49·2 (32·1–74·7)	-57·2% (-70·1 to -38·9%)	12·5% (9·5–15·6%)	2·2 (1·4–3·7)	-59% (-71·4 to -41·4%)
Lead exposure	Stroke	1·7% (0·3–3·4%)	32·4 (5·9–69·5)	-67·9% (-84·3 to -54·1%)	1·9% (0·5–3·6%)	1·8 (0·5–3·6)	-63·6% (-77·8 to -50·3%)
	Ischaemic stroke	1·7% (0·4–3·5%)	24·4 (4·9–51·2)	-56·8% (-78·4 to -40·3%)	1·9% (0·5–3·6%)	1·4 (0·4–2·7)	-53·6% (-71·1 to -36·7%)
	Intracerebral haemorrhage	1·6% (0·2–3·7%)	7·2 (0·8–19·1)	-82% (-93·7 to -71%)	2% (0·4–4·1%)	0·4 (0·1–0·9)	-79·2% (-90·2 to -68·8%)
	Subarachnoid haemorrhage	1·2% (0·1–3·1%)	0·8 (0·1–2·2)	-82·3% (-94 to -68·4%)	1·8% (0·3–3·9%)	<0·1 (<0·1–0·1)	-78·2% (-90·2 to -62·4%)
Low birth weight	Meningitis	1·2% (0·6–3·1%)	0·4 (0·2–1)	-92·9% (-96·5 to -85·8%)	0·6% (0·3–1·6%)	<0·1 (<0·1–<0·1)	-92·9% (-96·5 to -85·8%)
	Encephalitis	1·9% (0·9–3·6%)	0·3 (0·1–0·6)	-35·4% (-75·9 to 67·6%)	1·2% (0·5–2·3%)	<0·1 (<0·1–<0·1)	-35·4% (-75·9 to 67·6%)
Low birth weight and short gestation	Meningitis	1·4% (0·7–3·4%)	0·4 (0·2–1·1)	-92·9% (-96·5 to -85·7%)	0·6% (0·3–1·7%)	<0·1 (<0·1–<0·1)	-92·9% (-96·5 to -85·7%)
	Encephalitis	2·1% (1–3·9%)	0·4 (0·2–0·7)	-34·6% (-75·4 to 68·7%)	1·3% (0·6–2·5%)	<0·1 (<0·1–<0·1)	-34·6% (-75·4 to 68·7%)
Low physical activity	Stroke	8% (1·9–16%)	153·3 (34·2–310·3)	-29·9% (-46·5 to -9·7%)	9·3% (2·4–17·4%)	8·5 (2·1–16·1)	-37·7% (-52·6 to -18·9%)
	Ischaemic stroke	10·8% (2·5–21·5%)	153·3 (34·2–310·3)	-29·9% (-46·5 to -9·7%)	11·9% (3·1–22%)	8·5 (2·1–16·1)	-37·7% (-52·6 to -18·9%)
Low temperature	Stroke	3·4% (-5·3–11·1%)	65·2 (-109·2–220·9)		4·1% (-6·5–13·1%)	3·7 (-6·3–12·7)	
	Ischaemic stroke	3·3% (-5·1–10·6%)	46·2 (-76·4–156·6)		4·1% (-6·5–13·1%)	2·9 (-4·8–10)	
	Intracerebral haemorrhage	3·7% (-5·9–12·1%)	16·9 (-28·3–59·6)		4·1% (-6·5–13·1%)	0·7 (-1·2–2·6)	
	Subarachnoid haemorrhage	3·5% (-5·7–11·5%)	2·1 (-3·7–8·2)		4·1% (-6·5–13·1%)	0·1 (-0·1–0·3)	
Metabolic risk factors†	Stroke	78% (71·2–84·5%)	1503·1 (1159·1–1956)	-45·9% (-59 to -30·1%)	73·4% (63·6–83·7%)	67 (49·6–89·4)	-51·1% (-62·7 to -36·2%)
	Ischaemic stroke	77·9% (69·8–86·2%)	1102·5 (853–1370·2)	-34% (-49 to -15·8%)	72·8% (60·9–85·4%)	52 (37·4–67·8)	-42·1% (-56 to -24·7%)

Table S16 Regional and national disabilities and deaths related to neurological conditions attributable to risk factors in North Africa and Middle East countries

**Location**<br>Risk factor (alphabetic order)	Cause	**DALYs (95% UI)**<br>PAF (%)<br>2019	Rate per 100,000<br>2019	Rate change (%)<br>From 1990 to 2019	**Deaths (95% UI)**<br>PAF (%)<br>2019	Rate per 100,000<br>2019	Rate change (%)<br>From 1990 to 2019
	Intracerebral haemorrhage	79% (72·1–84·7%)	356 (248–532·9)	-64·3% (-75·6 to -49%)	76·1% (67·6–83·3%)	13·6 (9·2–21·3)	-68·5% (-78·2 to -54·8%)
	Subarachnoid haemorrhage	73·2% (65·5–80·4%)	44·6 (22·6–72·8)	-60·1% (-75·7 to -34·9%)	72% (63·2–79·8%)	1·4 (0·6–2·3)	-65·2% (-78·8 to -41·6%)
	Neurological disorders*	10·3% (3·5–21·8%)	147 (48·8–377·7)	32·6% (8·4 to 77·1%)	24·4% (9·2–43·3%)	9·5 (1·7–28·7)	32·9% (3·5 to 91·9%)
	Alzheimer's disease and other dementias	40% (20·1–60%)	147 (48·8–377·7)	32·6% (8·4 to 77·1%)	38·9% (18·8–59%)	9·5 (1·7–28·7)	32·9% (3·5 to 91·9%)
Non-optimal temperature	Stroke	9% (-0·8–17·6%)	173 (-14·4–365·4)	-46·5% (-139·8 to 19·3%)	10·9% (-0·9–20·9%)	9·9 (-0·8–21)	-46·2% (-137·9 to 21·6%)
	Ischaemic stroke	8·6% (-0·7–17%)	122·6 (-10·2–255·2)	-34% (-150·7 to 51·6%)	10·9% (-0·9–20·9%)	7·8 (-0·7–16·1)	-36·7% (-146·3 to 41·9%)
	Intracerebral haemorrhage	9·9% (-0·8–19·1%)	44·8 (-3·6–100·6)	-63·1% (-126·4 to -15·4%)	10·9% (-0·9–20·9%)	1·9 (-0·2–4·3)	-65·5% (-123·2 to -21·6%)
	Subarachnoid haemorrhage	9·3% (-0·8–18·4%)	5·7 (-0·5–13·6)	-64·9% (-123·4 to -19·3%)	10·9% (-0·9–20·9%)	0·2 (0–0·5)	-62·6% (-126 to -16·7%)
Other environmental risks	Stroke	1·7% (0·3–3·4%)	32·4 (5·9–69·5)	-67·9% (-84·3 to -54·1%)	1·9% (0·5–3·6%)	1·8 (0·5–3·6)	-63·6% (-77·8 to -50·3%)
	Ischaemic stroke	1·7% (0·4–3·5%)	24·4 (4·9–51·2)	-56·8% (-78·4 to -40·3%)	1·9% (0·5–3·6%)	1·4 (0·4–2·7)	-53·6% (-71·1 to -36·7%)
	Intracerebral haemorrhage	1·6% (0·2–3·7%)	7·2 (0·8–19·1)	-82% (-93·7 to -71%)	2% (0·4–4·1%)	0·4 (0·1–0·9)	-79·2% (-90·2 to -68·8%)
	Subarachnoid haemorrhage	1·2% (0·1–3·1%)	0·8 (0·1–2·2)	-82·3% (-94 to -68·4%)	1·8% (0·3–3·9%)	<0·1 (<0·1–0·1)	-78·2% (-90·2 to -62·4%)
Particulate matter pollution	Meningitis	0·3% (0·1–0·7%)	0·1 (0–0·2)	-92·7% (-97·6 to -80·6%)	0·1% (0–0·3%)	<0·1 (<0·1–<0·1)	-92·7% (-97·6 to -80·6%)
	Encephalitis	0·4% (0·1–0·9%)	0·1 (0–0·1)	-32·4% (-81·4 to 130·9%)	0·2% (0·1–0·5%)	<0·1 (<0·1–<0·1)	-32·4% (-81·4 to 130·9%)
	Stroke	30·2% (24·4–35·7%)	581·5 (421·7–780·4)	-44·3% (-58·2 to -26·9%)	25·8% (20·7–30·8%)	23·5 (16·3–32·9)	-49·9% (-62·5 to -34%)
	Ischaemic stroke	29% (23·5–34·4%)	410·8 (301·2–545·8)	-31·2% (-47·6 to -11·2%)	24·7% (19·8–29·9%)	17·7 (12·4–24·3)	-40·1% (-55·3 to -21·3%)
	Intracerebral haemorrhage	33·4% (27·3–39·7%)	150·4 (99·8–226)	-62·4% (-74·7 to -45·6%)	29·4% (23·7–35·2%)	5·3 (3·4–8·1)	-66·8% (-77·6 to -51·9%)
	Subarachnoid haemorrhage	33·4% (27·3–39·6%)	20·3 (10·5–33·7)	-58% (-74·3 to -30·2%)	30·2% (24·5–35·8%)	0·6 (0·3–1)	-63·3% (-78·2 to -38·3%)
Secondhand smoke	Stroke	4·4% (3·3–5·6%)	84·4 (57·1–118·7)	-43·1% (-58·5 to -22·5%)	3·9% (3–4·9%)	3·6 (2·4–5)	-47·1% (-61·5 to -28·2%)
	Ischaemic stroke	4·1% (3·1–5·3%)	58·2 (40·5–80·4)	-28·6% (-47·4 to -3·6%)	3·7% (2·8–4·7%)	2·6 (1·8–3·7)	-36% (-53·5 to -12·7%)
	Intracerebral haemorrhage	5·1% (3·8–6·5%)	23 (14·4–36·6)	-61·3% (-74·5 to -42·7%)	4·6% (3·5–5·8%)	0·8 (0·5–1·3)	-65·1% (-76·5 to -49·2%)
	Subarachnoid haemorrhage	5·2% (3·9–6·6%)	3·1 (1·6–5·3)	-57·2% (-74·4 to -27·3%)	4·9% (3·7–6·1%)	0·1 (0–0·2)	-61·5% (-76·5 to -34·3%)
Short gestation	Meningitis	1% (0·5–2·6%)	0·3 (0·2–0·8)	-91·9% (-96 to -83·7%)	0·5% (0·2–1·3%)	<0·1 (<0·1–<0·1)	-91·9% (-96 to -83·7%)
	Encephalitis	1·6% (0·7–3%)	0·3 (0·1–0·5)	-25·5% (-71·8 to 91·9%)	1% (0·4–1·9%)	<0·1 (<0·1–<0·1)	-25·5% (-71·8 to 92%)
Smoking	Stroke	13·2% (11·4–15·1%)	255·1 (187–339·5)	-39·5% (-57·9 to -14·7%)	9·5% (8·1–11·1%)	8·7 (6·2–11·8)	-44·4% (-61·5 to -20·7%)
	Ischaemic stroke	12·1% (10·5–13·9%)	171·5 (127·5–222·4)	-22·4% (-45·2 to 9·5%)	8·6% (7·3–10%)	6·1 (4·4–8·1)	-30·6% (-52·6 to 1%)
	Intracerebral haemorrhage	16·5% (14–18·9%)	74·3 (49·4–111·5)	-58·8% (-74·5 to -36·5%)	13% (11·1–15·1%)	2·3 (1·5–3·6)	-62·5% (-76·6 to -41·5%)
	Subarachnoid haemorrhage	15·1% (11·9–18·8%)	9·3 (4·2–15·9)	-54·4% (-75·4 to -20%)	12·5% (9·7–16·1%)	0·3 (0·1–0·5)	-59·3% (-78·1 to -25·7%)
	Neurological disorders*	3·4% (1–8·2%)	49·7 (12·8–129·2)	12·4% (-15·1 to 53·1%)	7·5% (1–14·7%)	3 (0·2–10)	19·4% (-15·7 to 103·7%)
	Alzheimer's disease and other dementias	15·5% (9·4–22·5%)	56·9 (21–135·5)	10·7% (-15·4 to 49%)	14·6% (8·8–21·4%)	3·5 (0·8–10·4)	16·2% (-12·9 to 68·6%)
	Parkinson's disease	-7·3% (-11·3–-3·7%)	-9·5 (-15·9–-4·5)	1·8% (-31·8 to 50·1%)	-6·8% (-10·7–-3·5%)	-0·5 (-0·9–-0·3)	1·5% (-33·1 to 51·8%)

Table S16 Regional and national disabilities and deaths related to neurological conditions attributable to risk factors in North Africa and Middle East countries

Location<br>Risk factor (alphabetic order)	Cause	DALYs (95% UI)<br>PAF (%)<br>2019	Rate per 100,000<br>2019	Rate change (%)<br>From 1990 to 2019	Deaths (95% UI)<br>PAF (%)<br>2019	Rate per 100,000<br>2019	Rate change (%)<br>From 1990 to 2019
	Multiple sclerosis	11·2% (7·7–15·2%)	2·3 (1·2–4)	7·7% (-31·6 to 58·1%)	11·7% (7·2–16%)	<0·1 (<0·1–0·1)	18·7% (-38·3 to 91·2%)
Tobacco	Stroke	17% (15–19%)	326·9 (240·9–435·4)	-40·6% (-57·6 to -17·6%)	13% (11·4–14·7%)	11·9 (8·6–16)	-45·3% (-60·7 to -25%)
	Ischaemic stroke	15·7% (13·9–17·6%)	221·7 (167·4–284·6)	-24·3% (-45·4 to 4·5%)	11·9% (10·3–13·6%)	8·5 (6·2–11·2)	-32·5% (-51·9 to -6·3%)
	Intracerebral haemorrhage	20·7% (18·2–23·2%)	93·3 (62·8–138·8)	-59·5% (-74·5 to -38·8%)	16·9% (14·7–19·2%)	3 (2–4·6)	-63·3% (-76·4 to -44·6%)
	Subarachnoid haemorrhage	19·5% (16·1–23%)	11·9 (5·7–20·1)	-55·3% (-75 to -23·2%)	16·7% (14–20%)	0·3 (0·1–0·6)	-60·1% (-77·9 to -29·8%)
	Neurological disorders*	3·4% (1–8·2%)	49·7 (12·8–129·2)	12·4% (-15·1 to 53·1%)	7·5% (1–14·7%)	3 (0·2–10)	19·4% (-15·7 to 103·7%)
	Alzheimer's disease and other dementias	15·5% (9·4–22·5%)	56·9 (21–135·5)	10·7% (-15·4 to 49%)	14·6% (8·8–21·4%)	3·5 (0·8–10·4)	16·2% (-12·9 to 68·6%)
	Parkinson's disease	-7·3% (-11·3–-3·7%)	-9·5 (-15·9–-4·5)	1·8% (-31·8 to 50·1%)	-6·8% (-10·7–-3·5%)	-0·5 (-0·9–-0·3)	1·5% (-33·1 to 51·8%)
	Multiple sclerosis	11·2% (7·7–15·2%)	2·3 (1·2–4)	7·7% (-31·6 to 58·1%)	11·7% (7·2–16%)	<0·1 (<0·1–0·1)	18·7% (-38·3 to 91·2%)
**Yemen**							
Air pollution	Meningitis	1·1% (0·6–1·8%)	1·1 (0·6–1·8)	-79·2% (-90·3 to -57·6%)	0·6% (0·3–1·1%)	<0·1 (<0·1–<0·1)	-79·2% (-90·3 to -57·6%)
	Encephalitis	0·5% (0·2–1%)	0·1 (0·1–0·3)	-25·9% (-71 to 86·7%)	0·3% (0·1–0·7%)	<0·1 (<0·1–<0·1)	-25·9% (-71 to 86·8%)
	Stroke	33·7% (27·2–40·1%)	934 (673·2–1267·3)	-40·6% (-57·8 to -16·7%)	29·6% (23·5–35·4%)	40·2 (28·4–55·1)	-42·6% (-59·1 to -19·6%)
	Ischaemic stroke	32·3% (25·9–38·6%)	554·1 (404·3–754)	-5·7% (-33·1 to 28·9%)	28·1% (22·2–33·8%)	26·2 (18·6–36·9)	-14·1% (-40·4 to 20·8%)
	Intracerebral haemorrhage	36·3% (29·5–42·8%)	321·5 (221·6–445·2)	-62·2% (-73·9 to -44·8%)	32·8% (26·3–39·1%)	11·8 (8·1–16·5)	-65·4% (-76·1 to -50·1%)
	Subarachnoid haemorrhage	35·6% (28·8–42·3%)	58·5 (30·7–98·9)	-56·9% (-71·7 to -28·7%)	32·6% (26–39·2%)	2·1 (1–3·7)	-60·3% (-74·3 to -32·8%)
Alcohol use	Stroke	0·5% (0·2–0·7%)	12·6 (4·8–22·4)	-78·6% (-90·1 to -62·2%)	0·3% (0·1–0·5%)	0·4 (0·1–0·7)	-80·9% (-92·9 to -65·1%)
	Ischaemic stroke	0·1% (0–0·3%)	2·2 (-0·7–6)	-70·1% (-119·3 to -32·2%)	0·1% (-0·1–0·2%)	0·1 (-0·1–0·2)	-77·3% (-143·2 to -33·9%)
	Intracerebral haemorrhage	1·2% (0·6–1·9%)	10·3 (4·9–17·7)	-79·9% (-89·5 to -65·8%)	0·9% (0·4–1·5%)	0·3 (0·1–0·6)	-81·5% (-90·4 to -68·8%)
	Neurological disorders*	0·2% (0·1–0·4%)	2·2 (1–4·1)	-60·1% (-81·7 to -15·8%)	0·1% (0–0·2%)	<0·1 (<0·1–<0·1)	-60·7% (-78·2 to -32·2%)
	Idiopathic epilepsy	1·5% (0·9–2·3%)	2·2 (1–4·1)	-60·1% (-81·7 to -15·8%)	1·8% (1–2·7%)	<0·1 (<0·1–<0·1)	-60·7% (-78·2 to -32·2%)
All risk factors	Meningitis	5·3% (3–8·5%)	5·1 (2·8–8·8)	-72·4% (-86·7 to -44·7%)	2·9% (1·6–5·2%)	0·1 (0–0·1)	-72·4% (-86·7 to -44·7%)
	Encephalitis	2·3% (1·1–4·7%)	0·7 (0·3–1·3)	-3·7% (-58·3 to 134·1%)	1·6% (0·7–3%)	<0·1 (<0·1–<0·1)	-3·7% (-58·3 to 134·2%)
	Stroke	86·4% (83·1–89·9%)	2391·7 (1853·8–3075·2)	-23·1% (-40·5 to 1·9%)	84·5% (79·9–89·6%)	114·7 (86·2–148·7)	-21·7% (-38·7 to 2·7%)
	Ischaemic stroke	87% (82·8–91·6%)	1493·7 (1176·3–1906·4)	22% (-3·7 to 56%)	84·2% (78·5–91%)	78·6 (59·2–103·2)	17·1% (-8·8 to 50·4%)
	Intracerebral haemorrhage	86·1% (82·2–89·3%)	762·3 (558·6–1017·9)	-53·2% (-65·8 to -35·6%)	85·4% (80·7–89·6%)	30·8 (22·6–40·5)	-55·4% (-66·7 to -39·4%)
	Subarachnoid haemorrhage	82·4% (78–86·2%)	135·7 (70·2–225·2)	-46·8% (-63·3 to -15%)	82·9% (77·2–87·6%)	5·3 (2·6–9·1)	-48·6% (-64·2 to -17·3%)
	Neurological disorders*	8·9% (3·5–18·2%)	117 (42·4–300·1)	22% (-0·4 to 47·3%)	20% (9–31·8%)	7 (1·2–22·8)	32% (3·4 to 69·1%)
	Alzheimer's disease and other dementias	31·5% (21·1–43%)	121·1 (45·4–304·8)	25·6% (4·2 to 50·6%)	28·9% (18·8–40·5%)	7·4 (1·6–23·3)	32·1% (4·2 to 67·6%)
	Parkinson's disease	-10·8% (-15·6–-5·9%)	-8·3 (-13–-4·4)	7·2% (-23·8 to 55·5%)	-9·6% (-14·2–-5·3%)	-0·4 (-0·7–-0·2)	18% (-16·8 to 74·9%)
	Idiopathic epilepsy	1·5% (0·9–2·3%)	2·2 (1–4·1)	-60·1% (-81·7 to -15·8%)	1·8% (1–2·7%)	<0·1 (<0·1–<0·1)	-60·7% (-78·2 to -32·2%)

Table S16 Regional and national disabilities and deaths related to neurological conditions attributable to risk factors in North Africa and Middle East countries

**Location**<br>Risk factor (alphabetic order)	Cause	**DALYs (95% UI)**<br>PAF (%)<br>2019	 Rate per 100,000<br>2019	 Rate change (%)<br>From 1990 to 2019	**Deaths (95% UI)**<br>PAF (%)<br>2019	 Rate per 100,000<br>2019	 Rate change (%)<br>From 1990 to 2019
	Multiple sclerosis	14·3% (10·2–18·6%)	2 (1·2–2·9)	16·6% (-22·8 to 68%)	15·2% (10·4–20·8%)	<0·1 (<0·1–0·1)	17% (-37·2 to 102%)
Ambient particulate matter pollution	Meningitis	0·5% (0·2–1%)	0·5 (0·2–1)	14·7% (-54·5 to 355%)	0·3% (0·1–0·6%)	<0·1 (<0·1–<0·1)	14·6% (-54·5 to 355%)
	Encephalitis	0·2% (0·1–0·6%)	0·1 (0–0·2)	300·9% (27·2 to 1644%)	0·2% (0·1–0·4%)	<0·1 (<0·1–<0·1)	300·9% (27·1 to 1644·5%)
	Stroke	18·2% (9·4–27·7%)	504·6 (240·8–806·7)	204·1% (60·2 to 642·4%)	16% (8·1–24·4%)	21·7 (10·5–35·1)	201·5% (63·6 to 602·8%)
	Ischaemic stroke	17·4% (9–26·6%)	298·9 (143·9–478·8)	389% (161·3 to 1087·8%)	15·2% (7·7–23·4%)	14·2 (6·7–23·5)	359·3% (146·8 to 979·9%)
	Intracerebral haemorrhage	19·7% (10·2–29·7%)	174·4 (82·6–285·2)	92·4% (-0·6 to 400·9%)	17·8% (9·2–27·1%)	6·4 (3·1–10·6)	79·8% (-4·6 to 350·9%)
	Subarachnoid haemorrhage	19% (9·5–28·8%)	31·3 (11·8–63·9)	120·7% (8·4 to 522·8%)	17·5% (8·8–26·9%)	1·1 (0·4–2·3)	106·7% (4·6 to 463·6%)
Behavioural risk factors‡	Meningitis	5·1% (2·9–8·3%)	5 (2·7–8·6)	-71·9% (-86·4 to -43·6%)	2·9% (1·6–5·1%)	0·1 (0–0·1)	-71·9% (-86·4 to -43·6%)
	Encephalitis	2·3% (1·1–4·5%)	0·7 (0·3–1·2)	-2·1% (-57·5 to 138·7%)	1·5% (0·7–2·9%)	<0·1 (<0·1–<0·1)	-2·1% (-57·5 to 138·7%)
	Stroke	44% (38·6–49·2%)	1218·4 (918·1–1614·3)	-23·7% (-42·4 to 2·3%)	39·3% (33·7–44·9%)	53·3 (39·3–71·7)	-21·2% (-38·5 to 4·3%)
	Ischaemic stroke	43·7% (37·2–50·6%)	751 (569·5–1000·7)	23·9% (-3·3 to 60·5%)	39% (32·5–46·4%)	36·4 (26·5–50)	19·9% (-5·9 to 54·2%)
	Intracerebral haemorrhage	44·9% (38·7–51·3%)	397·1 (278·8–548·6)	-53·8% (-66·7 to -35·1%)	40·1% (34·3–46·5%)	14·5 (10·2–19·9)	-55·5% (-67·7 to -38%)
	Subarachnoid haemorrhage	42·6% (35·5–49·6%)	70·3 (36·2–120·9)	-46·6% (-63·2 to -12·6%)	38·7% (31·4–45·7%)	2·5 (1·2–4·5)	-48·3% (-64·1 to -15·8%)
	Neurological disorders*	5% (1·8–10·4%)	65·4 (22·1–163)	4·8% (-18·6 to 31·3%)	10·1% (3·4–16·9%)	3·6 (0·5–11·2)	16·5% (-17·6 to 54·2%)
	Alzheimer's disease and other dementias	18·1% (11·5–24·6%)	69·5 (26·8–167·5)	10·5% (-12·5 to 38·1%)	15·5% (9·6–21·8%)	4 (0·8–11·5)	18·1% (-11·2 to 55·7%)
	Parkinson's disease	-10·8% (-15·6–-5·9%)	-8·3 (-13–-4·4)	7·2% (-23·8 to 55·5%)	-9·6% (-14·2–-5·3%)	-0·4 (-0·7–-0·2)	18% (-16·8 to 74·9%)
	Idiopathic epilepsy	1·5% (0·9–2·3%)	2·2 (1–4·1)	-60·1% (-81·7 to -15·8%)	1·8% (1–2·7%)	<0·1 (<0·1–<0·1)	-60·7% (-78·2 to -32·2%)
	Multiple sclerosis	14·3% (10·2–18·6%)	2 (1·2–2·9)	16·6% (-22·8 to 68%)	15·2% (10·4–20·8%)	<0·1 (<0·1–0·1)	17% (-37·2 to 102%)
Child and maternal malnutrition	Meningitis	5·1% (2·9–8·3%)	5 (2·7–8·6)	-71·9% (-86·4 to -43·6%)	2·9% (1·6–5·1%)	0·1 (0–0·1)	-71·9% (-86·4 to -43·6%)
	Encephalitis	2·3% (1·1–4·5%)	0·7 (0·3–1·2)	-2·1% (-57·5 to 138·7%)	1·5% (0·7–2·9%)	<0·1 (<0·1–<0·1)	-2·1% (-57·5 to 138·7%)
Diet high in red meat	Stroke	2·6% (0·9–4·2%)	72·2 (23·1–125·3)	-21·9% (-52 to 34·6%)	2% (0·7–3·2%)	2·7 (0·9–4·8)	-23·1% (-52 to 28%)
	Ischaemic stroke	2·3% (0·3–3·8%)	38·9 (5·1–71·9)	36·9% (4·2 to 87·7%)	1·7% (0·3–2·8%)	1·6 (0·3–3)	28·1% (-2·8 to 73·9%)
	Intracerebral haemorrhage	3·2% (0·4–5·4%)	28·1 (3·1–55·2)	-49% (-64 to -24·5%)	2·7% (0·3–4·6%)	1 (0·1–1·9)	-51·7% (-65 to -31·1%)
	Subarachnoid haemorrhage	3·2% (0·3–5·5%)	5·2 (0·5–11·7)	-42·1% (-60·8 to -4·9%)	2·7% (0·3–4·7%)	0·2 (0–0·4)	-45% (-62·4 to -8·4%)
Diet high in sodium	Stroke	1·9% (0·3–7·5%)	51·8 (6·9–205·3)	-25·7% (-57·2 to 39·8%)	1·6% (0·3–6·3%)	2·2 (0·4–8·8)	-24·1% (-55·1 to 35·7%)
	Ischaemic stroke	1·7% (0·3–6·8%)	29·2 (4·3–116·9)	24·7% (-29·7 to 116·9%)	1·4% (0·3–5·6%)	1·3 (0·2–5·4)	22·7% (-31 to 108·2%)
	Intracerebral haemorrhage	2·2% (0·3–8·5%)	19·3 (2·1–79·3)	-52·1% (-74·8 to -4·9%)	2% (0·3–7·9%)	0·7 (0·1–3)	-53·8% (-75·2 to -14·1%)
	Subarachnoid haemorrhage	2% (0·2–8%)	3·3 (0·3–14·7)	-45·6% (-72·7 to 20%)	1·9% (0·3–7·5%)	0·1 (0–0·6)	-47·3% (-72·6 to 7·2%)
Diet low in fibre	Stroke	5·5% (1·3–9·4%)	152 (34·9–282·4)	-15·7% (-36·3 to 18·7%)	4·7% (1·1–8%)	6·3 (1·5–11·9)	-16·8% (-35·6 to 15·6%)
	Ischaemic stroke	5·1% (1·2–8·7%)	88·4 (20·6–164·9)	40·2% (8·4 to 89·2%)	4·3% (1·1–7·4%)	4 (1–7·5)	30·7% (0·8 to 75·9%)
	Intracerebral haemorrhage	6% (1·4–10·4%)	53·5 (11·8–99·5)	-46·9% (-62·2 to -21·5%)	5·4% (1·3–9·3%)	2 (0·4–3·6)	-50·1% (-63·3 to -28·7%)
	Subarachnoid haemorrhage	6·1% (1·4–10·4%)	10·1 (2·1–23·1)	-39·4% (-58·6 to 0·8%)	5·4% (1·3–9·3%)	0·4 (0·1–0·8)	-42·4% (-59·1 to -2·8%)

Table S16 Regional and national disabilities and deaths related to neurological conditions attributable to risk factors in North Africa and Middle East countries

Location<br>Risk factor (alphabetic order)	Cause	DALYs (95% UI)<br>PAF (%)<br>2019	Rate per 100,000<br>2019	Rate change (%)<br>From 1990 to 2019	Deaths (95% UI)<br>PAF (%)<br>2019	Rate per 100,000<br>2019	Rate change (%)<br>From 1990 to 2019
Diet low in fruits	Stroke	8% (4·1–12·2%)	220·3 (112·5–360·6)	-32·2% (-53·6 to -0·6%)	6·4% (3·2–10·1%)	8·8 (4·2–14·5)	-32·5% (-53·6 to -2·3%)
	Ischaemic stroke	6% (1·6–11%)	103·1 (25·9–205·1)	26·1% (-1·5 to 64·6%)	4·9% (1·2–9%)	4·6 (1·1–9·2)	19·9% (-6·2 to 56·8%)
	Intracerebral haemorrhage	11·2% (5·7–18·6%)	98·9 (47·5–179·8)	-52·8% (-66·2 to -32·9%)	9·9% (5–16·7%)	3·6 (1·7–6·6)	-55·1% (-67 to -38%)
	Subarachnoid haemorrhage	11·2% (5·6–18·4%)	18·3 (7·1–39·6)	-46·1% (-62·9 to -12·4%)	9·9% (4·9–16·7%)	0·6 (0·2–1·4)	-48·5% (-64·4 to -16·4%)
Diet low in vegetables	Stroke	5·5% (2·4–8·4%)	150·9 (63·4–249·5)	-30·7% (-52 to 8·6%)	4·5% (2–6·9%)	6·1 (2·6–10)	-31·5% (-52·1 to 6·6%)
	Ischaemic stroke	3·9% (0·6–7%)	66·7 (10·5–125·1)	30·6% (1·4 to 71·1%)	3·2% (0·6–5·8%)	3 (0·5–5·7)	23·5% (-3·4 to 62·3%)
	Intracerebral haemorrhage	8% (2·1–14%)	71·2 (17·5–132·6)	-50·4% (-64·5 to -26·7%)	7·3% (1·9–12·6%)	2·6 (0·7–4·8)	-53% (-65·3 to -31·9%)
	Subarachnoid haemorrhage	8% (2·1–13·7%)	13·1 (2·9–27·1)	-43·6% (-61·2 to -5·5%)	7·2% (1·9–12·4%)	0·5 (0·1–1)	-46·2% (-62·6 to -11·5%)
Diet low in whole grains	Stroke	6·1% (3·5–7·8%)	168·1 (91·8–240·2)	27·5% (0·4 to 63·4%)	6% (3·6–7·5%)	8·2 (4·7–11·9)	22·3% (-3·5 to 57·2%)
	Ischaemic stroke	9·8% (5·5–12·4%)	168·1 (91·8–240·2)	27·5% (0·4 to 63·4%)	8·8% (5·3–11%)	8·2 (4·7–11·9)	22·3% (-3·5 to 57·2%)
Dietary risks	Stroke	25·8% (19·9–31·8%)	714·6 (496·1–1006·8)	-20·7% (-40·7 to 7·3%)	22·7% (17·3–27·9%)	30·8 (20·9–44)	-20% (-38·6 to 6·1%)
	Ischaemic stroke	25·2% (18·6–31·7%)	433·1 (299–610·3)	28·7% (0·5 to 66%)	21·9% (16·2–27·6%)	20·5 (14–29·4)	22·8% (-3·6 to 59·1%)
	Intracerebral haemorrhage	26·9% (18·6–35·1%)	237·9 (145·2–359·9)	-51·1% (-64·7 to -32·2%)	24·3% (16·7–32·1%)	8·8 (5·2–13·1)	-53·6% (-65·8 to -36·7%)
	Subarachnoid haemorrhage	26·6% (18·4–34·8%)	43·7 (20·7–81)	-44·3% (-61·9 to -8·8%)	24·2% (16·5–32·1%)	1·6 (0·7–3)	-46·7% (-62·4 to -13·2%)
Environmental/occupational risk factors§	Meningitis	1·1% (0·6–1·8%)	1·1 (0·6–1·8)	-79·2% (-90·3 to -57·6%)	0·6% (0·3–1·1%)	<0·1 (<0·1–<0·1)	-79·2% (-90·3 to -57·6%)
	Encephalitis	0·5% (0·2–1%)	0·1 (0·1–0·3)	-25·9% (-71 to 86·7%)	0·3% (0·1–0·7%)	<0·1 (<0·1–<0·1)	-25·9% (-71 to 86·8%)
	Stroke	44% (37·8–49·8%)	1218 (915·9–1584·5)	-36% (-52·6 to -13·2%)	40·7% (34·9–46·4%)	55·3 (40·4–73·1)	-35·7% (-52·1 to -13·2%)
	Ischaemic stroke	42·4% (36·6–48·1%)	728·6 (552·6–981)	2·9% (-23·8 to 37·6%)	39·1% (33·4–44·9%)	36·5 (26·7–49·9)	-2·7% (-28·2 to 32·2%)
	Intracerebral haemorrhage	46·8% (40·4–52·7%)	414·4 (298·5–567·3)	-59·8% (-71·3 to -42·6%)	44·4% (38–50·5%)	16 (11·4–21·7)	-61·9% (-72·3 to -46·2%)
	Subarachnoid haemorrhage	45·5% (38·9–51·9%)	75 (38·6–124·6)	-54·5% (-69·2 to -26·9%)	43·8% (37·2–50·3%)	2·8 (1·4–4·8)	-56·3% (-70·1 to -29%)
High body-mass index	Stroke	20·4% (12–28·6%)	564 (299·1–912·1)	20·1% (-19·5 to 108·1%)	13·1% (7·4–19·3%)	17·8 (9·1–29·7)	18·6% (-19 to 105·8%)
	Ischaemic stroke	14·7% (8·3–21·6%)	252·9 (132·7–409)	131·9% (61·8 to 306·5%)	9·3% (4·9–14·4%)	8·7 (4·2–14·7)	121% (52·8 to 294·5%)
	Intracerebral haemorrhage	29·5% (17·9–40·8%)	261·5 (139·2–419)	-15·2% (-45 to 55·5%)	21·6% (12·5–31%)	7·8 (4·1–12·7)	-18·6% (-46·6 to 47·8%)
	Subarachnoid haemorrhage	30·4% (18·6–42·4%)	49·6 (21·9–93·2)	-4·6% (-41·1 to 95%)	21·9% (12·5–32%)	1·4 (0·6–2·7)	-10·2% (-44·9 to 84·9%)
	Neurological disorders*	2·1% (0·5–5·5%)	27·7 (6·2–78·6)	64·4% (16·9 to 217·1%)	4·8% (1·4–11·1%)	1·7 (0·2–5·7)	62·2% (9·2 to 262·8%)
	Alzheimer's disease and other dementias	7·2% (2·3–15·2%)	27·7 (6·2–78·6)	64·4% (16·9 to 217·1%)	6·5% (1·9–14·1%)	1·7 (0·2–5·7)	62·2% (9·2 to 262·8%)
High fasting plasma glucose	Stroke	22·7% (13·2–36·6%)	626·9 (351–1071·4)	58·1% (6·2 to 130·7%)	24·1% (13·4–40%)	32·6 (17·2–59·1)	57·4% (8 to 131·6%)
	Ischaemic stroke	24·6% (11·8–47·4%)	422·5 (193·6–847·8)	144% (69·9 to 245·1%)	24·9% (11·1–50%)	23·1 (9·9–51·6)	127·6% (57·1 to 224·2%)
	Intracerebral haemorrhage	19·7% (11·1–30·4%)	174·2 (87·2–283)	-10% (-39·8 to 37·8%)	22·5% (12·7–35·9%)	8·1 (4–13·5)	-11·9% (-41·8 to 32·7%)
	Subarachnoid haemorrhage	18·2% (10·4–28·6%)	30·2 (12·2–61·9)	0·7% (-34·6 to 70·8%)	22% (12·1–36·6%)	1·4 (0·5–3·1)	1·4% (-35·1 to 75%)
	Neurological disorders*	2·9% (0·5–8·3%)	37·6 (6·3–123·8)	57·5% (36·3 to 92·7%)	7·3% (1·5–16·6%)	2·5 (0·3–9·5)	58·7% (30·9 to 106·4%)

Table S16 Regional and national disabilities and deaths related to neurological conditions attributable to risk factors in North Africa and Middle East countries

Location<br>Risk factor (alphabetic order)	Cause	DALYs (95% UI)<br>PAF (%)<br>2019	 Rate per 100,000<br>2019	 Rate change (%)<br>From 1990 to 2019	Deaths (95% UI)<br>PAF (%)<br>2019	 Rate per 100,000<br>2019	 Rate change (%)<br>From 1990 to 2019
	Alzheimer's disease and other dementias	9·8% (2·2–20·7%)	37·6 (6·3–123·8)	57·5% (36·3 to 92·7%)	9·8% (2·2–20·9%)	2·5 (0·3–9·5)	58·7% (30·9 to 106·4%)
High LDL cholesterol	Stroke	14·4% (8·8–23·6%)	399·1 (221·3–709·5)	34·7% (4·7 to 80·1%)	13·4% (5·7–26·9%)	18·2 (7·1–40·1)	26·7% (-1·1 to 72·2%)
	Ischaemic stroke	23·2% (14–38·1%)	399·1 (221·3–709·5)	34·7% (4·7 to 80·1%)	19·5% (8·3–39·2%)	18·2 (7·1–40·1)	26·7% (-1·1 to 72·2%)
High systolic blood pressure	Stroke	48·6% (40·3–56·3%)	1344·8 (963–1816·7)	-21·4% (-42 to 7·3%)	45·3% (35·8–54·7%)	61·6 (42·7–85·1)	-21·4% (-41·3 to 8%)
	Ischaemic stroke	46% (35·7–56·1%)	790·5 (544·1–1095·6)	28·8% (-2·6 to 76·1%)	42·5% (31·4–54·2%)	39·7 (26·2–56·8)	22·2% (-8·9 to 68%)
	Intracerebral haemorrhage	53·1% (42·4–62·5%)	469·9 (320·7–655·9)	-50·4% (-64·5 to -29·3%)	51·6% (40·6–62·2%)	18·6 (12·3–26·1)	-53·2% (-66 to -33·3%)
	Subarachnoid haemorrhage	51·4% (40·5–61·1%)	84·4 (43·5–144)	-43·6% (-62·6 to -8·1%)	50·9% (40–61·8%)	3·3 (1·6–5·7)	-46·1% (-64·1 to -12·1%)
High temperature	Stroke	3·2% (1·2–6·8%)	89·3 (29·1–188·2)	-23% (-95·2 to 17%)	3·5% (1·3–7·3%)	4·8 (1·6–10·4)	-18·5% (-94·9 to 22·6%)
	Ischaemic stroke	3·1% (1·1–6·6%)	53·7 (17·7–111·9)	25·6% (-92·2 to 87·3%)	3·5% (1·3–7·3%)	3·3 (1·1–7·1)	21·4% (-92·5 to 75·4%)
	Intracerebral haemorrhage	3·4% (1·2–7·1%)	30·1 (9·7–62·4)	-52% (-97 to -26·2%)	3·5% (1·3–7·3%)	1·3 (0·4–2·6)	-53·7% (-97·1 to -28·9%)
	Subarachnoid haemorrhage	3·3% (1·2–6·8%)	5·5 (1·4–12·4)	-47·4% (-96·9 to -7·5%)	3·5% (1·3–7·3%)	0·2 (0·1–0·5)	-46·3% (-96·5 to -8·4%)
Household air pollution from solid fuels	Meningitis	0·6% (0·3–1%)	0·5 (0·2–1)	-88·3% (-94·9 to -73·6%)	0·3% (0·1–0·6%)	<0·1 (<0·1–<0·1)	-88·3% (-95 to -73·6%)
	Encephalitis	0·3% (0·1–0·6%)	0·1 (0–0·2)	-58·3% (-85 to 10%)	0·2% (0·1–0·4%)	<0·1 (<0·1–<0·1)	-58·3% (-85 to 10%)
	Stroke	15·5% (9·7–22·5%)	429·4 (244·7–650)	-69·5% (-80 to -55·2%)	13·6% (8·6–19·7%)	18·5 (10·5–28·3)	-70·6% (-80·4 to -57·2%)
	Ischaemic stroke	14·9% (9·4–21·4%)	255·2 (147·6–392·9)	-51·5% (-66·9 to -29%)	12·9% (8·1–18·7%)	12·1 (6·9–18·9)	-56% (-70·3 to -35·5%)
	Intracerebral haemorrhage	16·6% (10·4–23·7%)	147·1 (80·4–232·8)	-80·6% (-87·9 to -69·7%)	15% (9·4–21·7%)	5·4 (2·9–8·5)	-82·3% (-88·7 to -73·2%)
	Subarachnoid haemorrhage	16·6% (10–23·8%)	27·1 (11·8–49·2)	-77·7% (-86·6 to -61·5%)	15·1% (9·3–21·7%)	1 (0·4–1·8)	-79·6% (-87·6 to -63·9%)
Kidney dysfunction	Stroke	9·4% (7·5–11·4%)	260·8 (184·9–351·9)	6·2% (-18·3 to 40·9%)	9·1% (6·4–11·8%)	12·4 (7·9–17·7)	7·6% (-17·8 to 43·9%)
	Ischaemic stroke	10·7% (8·1–13·4%)	184·5 (124·8–258·6)	61·7% (26·3 to 109·8%)	9·5% (5·9–13·1%)	8·9 (5·1–13·4)	57·8% (20 to 107·1%)
	Intracerebral haemorrhage	8·6% (7–10·4%)	76·2 (53·3–104·4)	-42% (-57·6 to -19·2%)	9·6% (7·4–11·8%)	3·5 (2·3–4·9)	-40·8% (-55·4 to -18·9%)
Lead exposure	Stroke	10·9% (8·2–13·7%)	301·9 (197·4–425·5)	-27·5% (-46 to -2·3%)	10·7% (8–13·7%)	14·5 (9·4–20·9)	-18% (-37·9 to 10·2%)
	Ischaemic stroke	10·4% (7·5–13·6%)	179·5 (116·5–262)	28·5% (-0·3 to 67·1%)	10% (6·9–13·5%)	9·4 (5·9–13·9)	36·8% (6·2 to 80%)
	Intracerebral haemorrhage	11·8% (8·4–15·4%)	104·4 (66–154·6)	-56·5% (-68·4 to -39%)	12·1% (8·4–16·1%)	4·4 (2·7–6·6)	-53·7% (-65·4 to -36·7%)
	Subarachnoid haemorrhage	10·9% (7·4–14·5%)	18 (8·1–33·9)	-51·2% (-66·5 to -22·7%)	11·6% (7·8–15·8%)	0·7 (0·3–1·4)	-46·8% (-62·8 to -14%)
Low birth weight	Meningitis	5% (2·9–8%)	4·8 (2·6–8·4)	-72% (-86·5 to -43·9%)	2·8% (1·5–4·9%)	0·1 (0–0·1)	-72% (-86·5 to -43·9%)
	Encephalitis	2·2% (1·1–4·4%)	0·7 (0·3–1·2)	-2% (-57·6 to 139·7%)	1·5% (0·7–2·9%)	<0·1 (<0·1–<0·1)	-2% (-57·6 to 139·7%)
Low birth weight and short gestation	Meningitis	5·1% (2·9–8·3%)	5 (2·7–8·6)	-71·9% (-86·4 to -43·6%)	2·9% (1·6–5·1%)	0·1 (0–0·1)	-71·9% (-86·4 to -43·6%)
	Encephalitis	2·3% (1·1–4·5%)	0·7 (0·3–1·2)	-2·1% (-57·5 to 138·7%)	1·5% (0·7–2·9%)	<0·1 (<0·1–<0·1)	-2·1% (-57·5 to 138·7%)
Low physical activity	Stroke	4·3% (0·9–10%)	117·4 (23·3–274·9)	21·4% (-4·2 to 54·6%)	5·6% (1·2–12·1%)	7·6 (1·7–16·8)	18·1% (-7·9 to 52%)
	Ischaemic stroke	6·9% (1·4–16·2%)	117·4 (23·3–274·9)	21·4% (-4·2 to 54·6%)	8·2% (1·7–17·6%)	7·6 (1·7–16·8)	18·1% (-7·9 to 52%)
Low temperature	Stroke	2·2% (-0·2–4·5%)	60·9 (-6·2–127·1)	-35·9% (-71·3 to 14·8%)	2·4% (-0·2–4·9%)	3·3 (-0·3–6·8)	-32% (-68·8 to 18·8%)
	Ischaemic stroke	2·1% (-0·2–4·3%)	36·6 (-3·8–76·4)	5·1% (-53·1 to 82·5%)	2·4% (-0·2–4·9%)	2·3 (-0·2–4·7)	1·7% (-53·8 to 75·4%)

Table S16 Regional and national disabilities and deaths related to neurological conditions attributable to risk factors in North Africa and Middle East countries

Location<br>Risk factor (alphabetic order)	Cause	DALYs (95% UI)<br>PAF (%)<br>2019	Rate per 100,000<br>2019	Rate change (%)<br>From 1990 to 2019	Deaths (95% UI)<br>PAF (%)<br>2019	Rate per 100,000<br>2019	Rate change (%)<br>From 1990 to 2019
	Intracerebral haemorrhage	2·3% (-0·2–4·7%)	20·6 (-2·2–42·9)	-60·1% (-81·5 to -27·3%)	2·4% (-0·2–4·9%)	0·9 (-0·1–1·8)	-61·5% (-81·4 to -31·3%)
	Subarachnoid haemorrhage	2·3% (-0·2–4·6%)	3·8 (-0·4–9·2)	-56·5% (-82·9 to -16·1%)	2·4% (-0·2–4·9%)	0·2 (0–0·4)	-55·6% (-80·6 to -13·8%)
Metabolic risk factors†	Stroke	68·2% (60·9–75·9%)	1888·2 (1417·6–2465·6)	-13·1% (-33·4 to 16·9%)	65·5% (56·6–75·5%)	89 (63·3–116·4)	-12·1% (-32·3 to 18·3%)
	Ischaemic stroke	69·6% (60·3–79·6%)	1195·1 (902·3–1589·5)	35·8% (5·6 to 77·2%)	65·4% (53·7–79·2%)	61·1 (43·1–84·3)	30·4% (1·1 to 74·1%)
	Intracerebral haemorrhage	66·8% (58·1–74·3%)	591·3 (422·6–800·2)	-47·3% (-61·8 to -26·2%)	66·4% (56·8–74·7%)	23·9 (16·9–32·5)	-49·7% (-63·2 to -30%)
	Subarachnoid haemorrhage	62% (52·9–70·6%)	101·9 (52·5–171·8)	-39·7% (-59·6 to -2·4%)	62·1% (51·8–72%)	4 (1·9–6·9)	-41·9% (-61·3 to -5·1%)
	Neurological disorders*	4·8% (1·3–11·5%)	62·6 (16·8–171·3)	57·8% (28·2 to 116·2%)	11·7% (4–23%)	4 (0·6–13·3)	57·7% (21·2 to 127·1%)
	Alzheimer's disease and other dementias	16·3% (6·2–29·7%)	62·6 (16·8–171·3)	57·8% (28·2 to 116·2%)	15·7% (5·8–29·1%)	4 (0·6–13·3)	57·7% (21·2 to 127·1%)
Non-optimal temperature	Stroke	5·4% (2·1–8·4%)	148·1 (55·5–251·9)	-28·7% (-65·5 to 23·8%)	5·9% (2·3–9·3%)	8 (2·9–13·2)	-24·5% (-62·6 to 28·3%)
	Ischaemic stroke	5·2% (2–8·1%)	89·1 (33·2–148·4)	16·5% (-38·6 to 96·4%)	5·9% (2·3–9·3%)	5·5 (2–9·1)	12·7% (-43·3 to 87%)
	Intracerebral haemorrhage	5·6% (2·2–8·9%)	49·9 (18·8–87·6)	-55·6% (-79 to -19·9%)	5·9% (2·3–9·3%)	2·1 (0·8–3·6)	-57·1% (-79·3 to -22·9%)
	Subarachnoid haemorrhage	5·5% (2·2–8·6%)	9·1 (2·5–18·5)	-51·5% (-76·5 to 0·2%)	5·9% (2·3–9·3%)	0·4 (0·1–0·8)	-50·4% (-75·6 to 1·2%)
Other environmental risks	Stroke	10·9% (8·2–13·7%)	301·9 (197·4–425·5)	-27·5% (-46 to -2·3%)	10·7% (8–13·7%)	14·5 (9·4–20·9)	-18% (-37·9 to 10·2%)
	Ischaemic stroke	10·4% (7·5–13·6%)	179·5 (116·5–262)	28·5% (-0·3 to 67·1%)	10% (6·9–13·5%)	9·4 (5·9–13·9)	36·8% (6·2 to 80%)
	Intracerebral haemorrhage	11·8% (8·4–15·4%)	104·4 (66–154·6)	-56·5% (-68·4 to -39%)	12·1% (8·4–16·1%)	4·4 (2·7–6·6)	-53·7% (-65·4 to -36·7%)
	Subarachnoid haemorrhage	10·9% (7·4–14·5%)	18 (8·1–33·9)	-51·2% (-66·5 to -22·7%)	11·6% (7·8–15·8%)	0·7 (0·3–1·4)	-46·8% (-62·8 to -14%)
Particulate matter pollution	Meningitis	1·1% (0·6–1·8%)	1·1 (0·6–1·8)	-79·2% (-90·3 to -57·6%)	0·6% (0·3–1·1%)	<0·1 (<0·1–<0·1)	-79·2% (-90·3 to -57·6%)
	Encephalitis	0·5% (0·2–1%)	0·1 (0·1–0·3)	-25·9% (-71 to 86·7%)	0·3% (0·1–0·7%)	<0·1 (<0·1–<0·1)	-25·9% (-71 to 86·8%)
	Stroke	33·7% (27·2–40·1%)	934 (673·2–1267·3)	-40·6% (-57·8 to -16·7%)	29·6% (23·5–35·4%)	40·2 (28·4–55·1)	-42·6% (-59·1 to -19·6%)
	Ischaemic stroke	32·3% (25·9–38·6%)	554·1 (404·3–754)	-5·7% (-33·1 to 28·9%)	28·1% (22·2–33·8%)	26·2 (18·6–36·9)	-14·1% (-40·4 to 20·8%)
	Intracerebral haemorrhage	36·3% (29·5–42·8%)	321·5 (221·6–445·2)	-62·2% (-73·9 to -44·8%)	32·8% (26·3–39·1%)	11·8 (8·1–16·5)	-65·4% (-76·1 to -50·1%)
	Subarachnoid haemorrhage	35·6% (28·8–42·3%)	58·5 (30·7–98·9)	-56·9% (-71·7 to -28·7%)	32·6% (26–39·2%)	2·1 (1–3·7)	-60·3% (-74·3 to -32·8%)
Secondhand smoke	Stroke	4·5% (3·4–5·6%)	123·9 (82·1–177·5)	-23% (-43·2 to 6%)	3·8% (2·9–4·8%)	5·2 (3·4–7·3)	-22·2% (-40·6 to 4·1%)
	Ischaemic stroke	4·1% (3–5·1%)	70·2 (48·2–100·7)	30·5% (-1·4 to 73·9%)	3·5% (2·6–4·5%)	3·3 (2·2–4·8)	22·1% (-6·2 to 61·8%)
	Intracerebral haemorrhage	5·1% (3·8–6·5%)	45·3 (29–66·5)	-50·7% (-65·5 to -29%)	4·5% (3·3–5·6%)	1·6 (1–2·3)	-53·1% (-65·9 to -33·8%)
	Subarachnoid haemorrhage	5·2% (3·8–6·7%)	8·4 (4–14·6)	-44·7% (-63·3 to -9·8%)	4·5% (3·4–5·8%)	0·3 (0·1–0·5)	-47% (-63·5 to -12·8%)
Short gestation	Meningitis	3·9% (2·3–6·3%)	3·8 (2·1–6·4)	-70·5% (-85·5 to -39·9%)	2·2% (1·2–3·9%)	<0·1 (<0·1–0·1)	-70·5% (-85·5 to -39·9%)
	Encephalitis	1·7% (0·8–3·4%)	0·5 (0·3–0·9)	2·1% (-55·2 to 143·9%)	1·1% (0·5–2·3%)	<0·1 (<0·1–<0·1)	2·1% (-55·2 to 143·9%)
Smoking	Stroke	18·1% (16·2–20·1%)	500·7 (373·3–659·1)	-30·2% (-49·1 to -1·4%)	13·8% (12·1–15·5%)	18·7 (13·7–24·9)	-28·2% (-47·1 to -0·7%)
	Ischaemic stroke	16·5% (14·7–18·6%)	283·9 (217·3–379·3)	20·2% (-11·4 to 66·1%)	12·2% (10·6–13·8%)	11·3 (8·6–15·3)	19·8% (-12·2 to 67·2%)
	Intracerebral haemorrhage	20·9% (18·7–23·4%)	185·3 (131·8–254·1)	-55·7% (-69·3 to -34%)	17·5% (15·3–19·8%)	6·3 (4·5–8·6)	-56·4% (-69·3 to -36%)
	Subarachnoid haemorrhage	19% (15·4–22·5%)	31·5 (15·4–56·1)	-49·8% (-66·8 to -13·1%)	16·3% (12·9–19·9%)	1 (0·5–1·9)	-50·8% (-67·4 to -15·5%)

Table S16 Regional and national disabilities and deaths related to neurological conditions attributable to risk factors in North Africa and Middle East countries

Location<br>Risk factor (alphabetic order)	Cause	DALYs (95% UI)<br>PAF (%)<br>2019	Rate per 100,000<br>2019	Rate change (%)<br>From 1990 to 2019	Deaths (95% UI)<br>PAF (%)<br>2019	Rate per 100,000<br>2019	Rate change (%)<br>From 1990 to 2019
	Neurological disorders*	4·8% (1·7–10·2%)	63·2 (20·3–160·5)	11·1% (-12 to 37·7%)	10% (3·3–16·9%)	3·6 (0·4–11·2)	18·1% (-15·3 to 58·7%)
	Alzheimer's disease and other dementias	18·1% (11·5–24·6%)	69·5 (26·8–167·5)	10·5% (-12·5 to 38·1%)	15·5% (9·6–21·8%)	4 (0·8–11·5)	18·1% (-11·2 to 55·7%)
	Parkinson's disease	-10·8% (-15·6–-5·9%)	-8·3 (-13–-4·4)	7·2% (-23·8 to 55·5%)	-9·6% (-14·2–-5·3%)	-0·4 (-0·7–-0·2)	18% (-16·8 to 74·9%)
	Multiple sclerosis	14·3% (10·2–18·6%)	2 (1·2–2·9)	16·6% (-22·8 to 68%)	15·2% (10·4–20·8%)	<0·1 (<0·1–0·1)	17% (-37·2 to 102%)
Tobacco	Stroke	21·7% (19·5–23·9%)	599·8 (453·7–789·6)	-28·9% (-47·3 to -1%)	17% (15·2–18·8%)	23 (17·3–30·3)	-27% (-45·2 to -0·2%)
	Ischaemic stroke	19·9% (17·9–21·9%)	341·1 (265·7–450·1)	21·7% (-9 to 65·4%)	15·2% (13·4–17%)	14·2 (10·8–19)	20% (-9·4 to 64·4%)
	Intracerebral haemorrhage	24·9% (22·4–27·6%)	220·6 (158·3–303·8)	-54·9% (-68·3 to -33·9%)	21% (18·7–23·5%)	7·6 (5·5–10·4)	-55·8% (-68·7 to -36·3%)
	Subarachnoid haemorrhage	23·1% (19·7–26·4%)	38·1 (19–66·9)	-48·9% (-65·7 to -13·1%)	20% (16·7–23·5%)	1·3 (0·6–2·3)	-50·1% (-66·4 to -15·4%)
	Neurological disorders*	4·8% (1·7–10·2%)	63·2 (20·3–160·5)	11·1% (-12 to 37·7%)	10% (3·3–16·9%)	3·6 (0·4–11·2)	18·1% (-15·3 to 58·7%)
	Alzheimer's disease and other dementias	18·1% (11·5–24·6%)	69·5 (26·8–167·5)	10·5% (-12·5 to 38·1%)	15·5% (9·6–21·8%)	4 (0·8–11·5)	18·1% (-11·2 to 55·7%)
	Parkinson's disease	-10·8% (-15·6–-5·9%)	-8·3 (-13–-4·4)	7·2% (-23·8 to 55·5%)	-9·6% (-14·2–-5·3%)	-0·4 (-0·7–-0·2)	18% (-16·8 to 74·9%)
	Multiple sclerosis	14·3% (10·2–18·6%)	2 (1·2–2·9)	16·6% (-22·8 to 68%)	15·2% (10·4–20·8%)	<0·1 (<0·1–0·1)	17% (-37·2 to 102%)
**Afghanistan**							
Air pollution	Meningitis	1·6% (0·8–2·8%)	3·9 (1·8–7)	-80·3% (-91·4 to -52·2%)	1% (0·5–1·9%)	<0·1 (<0·1–0·1)	-80·3% (-91·4 to -52·2%)
	Encephalitis	0·3% (0·1–0·7%)	0·6 (0·2–1·5)	-25·9% (-70·4 to 85·9%)	0·1% (0–0·4%)	<0·1 (<0·1–<0·1)	-25·9% (-70·4 to 85·9%)
	Stroke	42% (38·1–46%)	1470·1 (1038·2–1910·8)	-32·2% (-49 to -12·5%)	37·3% (33·3–41·7%)	60·3 (41–79·3)	-32·7% (-49·3 to -13·8%)
	Ischaemic stroke	39·8% (35·8–44·1%)	742·3 (545·1–990·3)	11% (-15·9 to 44·3%)	35·2% (31·3–40%)	34·5 (24–47·2)	2·7% (-23·3 to 36·4%)
	Intracerebral haemorrhage	44·7% (40·6–48·7%)	625·2 (435·8–839·5)	-51·8% (-64·5 to -35·1%)	40·7% (36·7–44·9%)	22 (14·8–29·6)	-54·4% (-66·3 to -39·4%)
	Subarachnoid haemorrhage	43·9% (39·4–48·8%)	102·7 (33·5–177·9)	-49·5% (-65·4 to -19·4%)	40% (35·2–45·1%)	3·7 (1·1–6·8)	-51% (-65·6 to -22%)
Alcohol use	Stroke	0% (-0·1–0·1%)	0·9 (-2·1–4·5)		0% (-0·1–0·1%)	0 (-0·1–0·1)	
	Ischaemic stroke	-0·1% (-0·2–0%)	-1·4 (-3–0·3)		-0·1% (-0·1–0%)	-0·1 (-0·1–0)	
	Intracerebral haemorrhage	0·2% (0–0·4%)	2·2 (-0·1–5·3)		0·1% (0–0·3%)	0·1 (0–0·2)	
	Neurological disorders*	0% (0–0·1%)	0·7 (0·2–1·5)		0% (0–0·1%)	<0·1 (<0·1–<0·1)	
	Idiopathic epilepsy	0·3% (0·1–0·6%)	0·7 (0·2–1·5)		0·4% (0·1–0·7%)	<0·1 (<0·1–<0·1)	
All risk factors	Meningitis	6·2% (3·1–10·9%)	15 (7·3–26·9)	-75·9% (-89·3 to -45%)	4% (1·9–7·4%)	0·2 (0·1–0·3)	-75·9% (-89·3 to -45%)
	Encephalitis	1·2% (0·5–2·4%)	2·2 (0·9–5·4)	-10·7% (-63·1 to 115·8%)	0·5% (0·2–1·4%)	<0·1 (<0·1–0·1)	-10·7% (-63·1 to 115·8%)
	Stroke	89·6% (86·8–92·3%)	3136·6 (2253·2–4043·7)	-19·9% (-38·2 to -0·9%)	87·8% (84–91·9%)	141·8 (97·3–183·1)	-17·4% (-35·5 to 0·5%)
	Ischaemic stroke	89·7% (85·8–94%)	1673 (1224·9–2198·8)	30·2% (1 to 63·5%)	87·4% (82–93·8%)	85·6 (59·5–112·3)	25·5% (-1·9 to 59·5%)
	Intracerebral haemorrhage	90% (86·9–92·6%)	1257·5 (885·1–1689·8)	-44·8% (-58·8 to -27·1%)	88·8% (85–92·3%)	48 (32·7–64·2)	-46·3% (-59·1 to -31·7%)
	Subarachnoid haemorrhage	87·8% (83·8–91%)	206·1 (67·4–351·9)	-41·9% (-59 to -10·3%)	86·8% (81·7–91·2%)	8·2 (2·4–14·6)	-41·8% (-58·3 to -9·9%)
	Neurological disorders*	8·8% (3·2–19·1%)	133·2 (42·1–359·3)	46·6% (23·8 to 77·7%)	20·2% (8·2–34·5%)	9·1 (1·6–28·9)	47·2% (21·6 to 86·2%)

Table S16 Regional and national disabilities and deaths related to neurological conditions attributable to risk factors in North Africa and Middle East countries

Location<br>Risk factor (alphabetic order)	Cause	DALYs (95% UI)<br>PAF (%)<br>2019	Rate per 100,000<br>2019	Rate change (%)<br>From 1990 to 2019	Deaths (95% UI)<br>PAF (%)<br>2019	Rate per 100,000<br>2019	Rate change (%)<br>From 1990 to 2019
	Alzheimer's disease and other dementias	31·3% (18·4–46·8%)	135·9 (44·8–362·4)	44% (21·6 to 72·4%)	30·1% (17·5–45·5%)	9·3 (1·9–29·1)	45·7% (19·4 to 78·2%)
	Parkinson's disease	-4·4% (-6·9–-2·3%)	-5 (-8–-2·6)	14·4% (-18·8 to 57·9%)	-4·3% (-6·7–-2·2%)	-0·3 (-0·5–-0·1)	13·7% (-19·6 to 56·4%)
	Idiopathic epilepsy	0·3% (0·1–0·6%)	0·7 (0·2–1·5)		0·4% (0·1–0·7%)	<0·1 (<0·1–<0·1)	
	Multiple sclerosis	6·2% (3·9–9·2%)	1·7 (0·9–2·8)	81·4% (26·4 to 153·6%)	6·5% (3·6–10·3%)	<0·1 (<0·1–0·1)	60·9% (6·4 to 147·9%)
Ambient particulate matter pollution	Meningitis	0·3% (0·1–0·8%)	0·8 (0·3–1·8)	-36·6% (-76·3 to 111%)	0·2% (0·1–0·5%)	<0·1 (<0·1–<0·1)	-36·6% (-76·3 to 111%)
	Encephalitis	0·1% (0–0·2%)	0·1 (0–0·4)	137·8% (-14·5 to 803·9%)	0% (0–0·1%)	<0·1 (<0·1–<0·1)	137·8% (-14·5 to 803·9%)
	Stroke	10% (4·5–17·6%)	350·1 (150·6–677·2)	112·6% (15·3 to 436·2%)	8·9% (4–15·8%)	14·4 (6·3–28)	110·7% (13·9 to 416·7%)
	Ischaemic stroke	9·5% (4·3–16·9%)	176·8 (78·6–336·7)	245·9% (88·7 to 750·7%)	8·4% (3·8–14·9%)	8·2 (3·6–16·1)	222% (80·9 to 682·2%)
	Intracerebral haemorrhage	10·6% (4·7–18·9%)	148·9 (61·4–288·6)	51·4% (-19·2 to 285·2%)	9·7% (4·4–17·3%)	5·2 (2·2–10·2)	42·2% (-23·4 to 255·5%)
	Subarachnoid haemorrhage	10·3% (4·5–18·4%)	24·4 (6·4–58·4)	60·1% (-14·9 to 360·2%)	9·4% (4·1–16·7%)	0·9 (0·2–2·3)	54·1% (-18·2 to 340%)
Behavioural risk factors‡	Meningitis	5·9% (3–10·4%)	14·3 (7·1–25·7)	-75·4% (-89 to -43·5%)	3·8% (1·8–7·1%)	0·2 (0·1–0·3)	-75·4% (-89 to -43·5%)
	Encephalitis	1·1% (0·5–2·3%)	2·1 (0·8–5·2)	-9·4% (-62·7 to 119·3%)	0·5% (0·2–1·3%)	<0·1 (<0·1–0·1)	-9·4% (-62·7 to 119·3%)
	Stroke	40% (34–45·9%)	1397·9 (987·5–1914·4)	-16·1% (-36·2 to 6%)	35·8% (29·7–41·3%)	57·7 (38·9–80·1)	-13·6% (-32·9 to 6·9%)
	Ischaemic stroke	39·8% (32·4–47·7%)	742·2 (520–1028)	37% (4·9 to 73%)	35·8% (28·4–44%)	35·1 (23·3–50·2)	30·6% (0·9 to 65·5%)
	Intracerebral haemorrhage	40·4% (32·4–48%)	563·9 (383–790·5)	-42% (-57·2 to -22·8%)	35·9% (28·5–43·2%)	19·4 (12·9–27·4)	-43·9% (-57·8 to -27·4%)
	Subarachnoid haemorrhage	39·1% (30·7–46·9%)	91·8 (31·1–166·6)	-39·8% (-59·3 to -5·1%)	34·7% (26·8–42·2%)	3·3 (1–6·1)	-40·1% (-58 to -5·7%)
	Neurological disorders*	2·5% (0·8–5·3%)	37·2 (10·8–97·4)	24·9% (-4·9 to 62·3%)	5·1% (1·5–9%)	2·4 (0·3–7·6)	23% (-10·3 to 69·4%)
	Alzheimer's disease and other dementias	9·2% (5·6–13·3%)	39·9 (13·7–101·3)	19·9% (-8·2 to 51·3%)	8·4% (5–12·3%)	2·6 (0·5–7·8)	21·2% (-9·2 to 57%)
	Parkinson's disease	-4·4% (-6·9–-2·3%)	-5 (-8–-2·6)	14·4% (-18·8 to 57·9%)	-4·3% (-6·7–-2·2%)	-0·3 (-0·5–-0·1)	13·7% (-19·6 to 56·4%)
	Idiopathic epilepsy	0·3% (0·1–0·6%)	0·7 (0·2–1·5)		0·4% (0·1–0·7%)	<0·1 (<0·1–<0·1)	
	Multiple sclerosis	6·2% (3·9–9·2%)	1·7 (0·9–2·8)	81·4% (26·4 to 153·6%)	6·5% (3·6–10·3%)	<0·1 (<0·1–0·1)	60·9% (6·4 to 147·9%)
Child and maternal malnutrition	Meningitis	5·9% (3–10·4%)	14·3 (7·1–25·7)	-75·4% (-89 to -43·5%)	3·8% (1·8–7·1%)	0·2 (0·1–0·3)	-75·4% (-89 to -43·5%)
	Encephalitis	1·1% (0·5–2·3%)	2·1 (0·8–5·2)	-9·4% (-62·7 to 119·3%)	0·5% (0·2–1·3%)	<0·1 (<0·1–0·1)	-9·4% (-62·7 to 119·3%)
Diet high in red meat	Stroke	4·7% (1·8–7·1%)	163·1 (59·8–276·1)	-48·6% (-68·2 to -24·9%)	3·6% (1·4–5·5%)	5·8 (2·1–9·8)	-47% (-66·6 to -23·8%)
	Ischaemic stroke	4% (0·6–6·2%)	74 (11·4–133·5)	-7·1% (-47·8 to 26%)	2·9% (0·5–4·6%)	2·9 (0·5–5·3)	-8·3% (-43 to 22·3%)
	Intracerebral haemorrhage	5·5% (0·8–8·8%)	76·6 (10·9–142·2)	-62·7% (-81·6 to -46·2%)	4·6% (0·7–7·4%)	2·5 (0·4–4·6)	-63·1% (-80·4 to -48·9%)
	Subarachnoid haemorrhage	5·4% (0·9–8·9%)	12·5 (1·5–28·1)	-61·5% (-81·1 to -38%)	4·4% (0·7–7·5%)	0·4 (0–1)	-60·9% (-79·2 to -37%)
Diet high in sodium	Stroke	1·9% (0·3–7·5%)	65·6 (8–274·6)	-24·6% (-55·7 to 55%)	1·6% (0·3–6·4%)	2·6 (0·4–10·8)	-24·4% (-54 to 53·5%)
	Ischaemic stroke	1·7% (0·3–6·7%)	31·2 (4·3–127·9)	26% (-26·8 to 148%)	1·4% (0·3–5·6%)	1·4 (0·2–5·7)	21·4% (-28·5 to 143·3%)
	Intracerebral haemorrhage	2·1% (0·2–8·7%)	29·7 (3·2–124·7)	-45% (-70 to 9·9%)	1·9% (0·3–7·9%)	1 (0·1–4·4)	-47·7% (-69·7 to 6·5%)
	Subarachnoid haemorrhage	2% (0·2–8·1%)	4·7 (0·4–22·2)	-42·8% (-68·5 to 27·6%)	1·8% (0·3–7·4%)	0·2 (0–0·9)	-44·8% (-68·5 to 29%)

Table S16 Regional and national disabilities and deaths related to neurological conditions attributable to risk factors in North Africa and Middle East countries

**Location**<br>Risk factor (alphabetic order)	Cause	**DALYs (95% UI)**<br>PAF (%)<br>2019	Rate per 100,000<br>2019	Rate change (%)<br>From 1990 to 2019	**Deaths (95% UI)**<br>PAF (%)<br>2019	Rate per 100,000<br>2019	Rate change (%)<br>From 1990 to 2019
Diet low in fibre	Stroke	6·7% (1·7–11·3%)	236·5 (59–429)	17·7% (-13·6 to 76%)	5·6% (1·5–9·3%)	9 (2·2–16·5)	16·7% (-11·9 to 68·4%)
	Ischaemic stroke	6·2% (1·6–10·5%)	116·4 (29·1–206·7)	102·1% (49·4 to 195·1%)	5% (1·3–8·4%)	4·9 (1·3–8·9)	84·3% (38·7 to 162·1%)
	Intracerebral haemorrhage	7·4% (1·8–12·3%)	102·9 (24·2–185·1)	-16·6% (-40·5 to 30·2%)	6·4% (1·6–10·9%)	3·5 (0·8–6·3)	-20% (-41·9 to 21·5%)
	Subarachnoid haemorrhage	7·3% (1·8–12·1%)	17·2 (3·2–38·8)	-13·5% (-43 to 49·7%)	6·4% (1·6–10·6%)	0·6 (0·1–1·4)	-14·4% (-40·9 to 49%)
Diet low in fruits	Stroke	8·8% (4·9–13·5%)	306·1 (156·6–499·8)	-30·8% (-49·8 to -4·2%)	7% (3·8–10·7%)	11·3 (5·6–19)	-29·8% (-49·7 to -3·6%)
	Ischaemic stroke	6·3% (1·5–11·6%)	117·2 (28·5–232·5)	31·6% (0·5 to 67·4%)	5% (1·3–9·3%)	4·9 (1·2–9·8)	26·1% (-2·9 to 59·4%)
	Intracerebral haemorrhage	11·6% (5·9–19·5%)	162·1 (79–291·5)	-46·8% (-60·9 to -28·7%)	10·1% (5·2–17·2%)	5·5 (2·6–9·9)	-48·1% (-61 to -32·2%)
	Subarachnoid haemorrhage	11·5% (5·6–19·4%)	26·8 (8·2–60·5)	-44·5% (-62·1 to -13%)	10% (4·9–17·1%)	0·9 (0·3–2·2)	-44·4% (-60·4 to -12·6%)
Diet low in vegetables	Stroke	7·4% (3·3–11%)	257·1 (113·1–415·5)	-30·5% (-50 to -3·2%)	5·9% (2·7–8·9%)	9·6 (4·2–15·5)	-29·9% (-48·8 to -4%)
	Ischaemic stroke	4·9% (1–8·6%)	90·3 (19·3–167·4)	34·3% (1·8 to 68·6%)	3·9% (0·8–6·9%)	3·8 (0·8–7·1)	28·5% (-1·4 to 61·4%)
	Intracerebral haemorrhage	10·2% (3·3–16·9%)	143·2 (42·3–254·5)	-45·2% (-59·7 to -27·3%)	9·1% (2·9–15·1%)	4·9 (1·5–8·8)	-46·7% (-60·1 to -30·5%)
	Subarachnoid haemorrhage	10·1% (3·4–16·4%)	23·6 (5·2–52)	-42·8% (-60·6 to -10·6%)	8·9% (3–14·5%)	0·8 (0·2–1·9)	-42·8% (-59·3 to -10·8%)
Diet low in whole grains	Stroke	5·3% (3–7%)	186·8 (96·5–282·6)	30% (-0·4 to 63·2%)	5·4% (3·2–6·9%)	8·7 (4·7–12·7)	24·5% (-3·7 to 56·8%)
	Ischaemic stroke	10% (5·6–13%)	186·8 (96·5–282·6)	30% (-0·4 to 63·2%)	8·9% (5·3–11·2%)	8·7 (4·7–12·7)	24·5% (-3·7 to 56·8%)
Dietary risks	Stroke	29·7% (23·3–36·2%)	1038·6 (707·8–1452·7)	-22·3% (-41·5 to -1·1%)	25·6% (20–31·3%)	41·4 (27·5–59·4)	-20·1% (-38·9 to -0·5%)
	Ischaemic stroke	28·2% (21–35·3%)	526·1 (347·1–764·9)	32·5% (0·3 to 68%)	24·1% (18–30·1%)	23·6 (15–34·8)	27·2% (-2·1 to 61·7%)
	Intracerebral haemorrhage	31·5% (22·6–40·2%)	440 (275·5–646·6)	-45·8% (-59·9 to -27·4%)	28·1% (19·9–36·3%)	15·2 (9·4–22·6)	-47% (-60 to -31·5%)
	Subarachnoid haemorrhage	31% (21·7–39·7%)	72·5 (24·2–136·8)	-43·3% (-61·6 to -11·5%)	27·6% (19·1–35·7%)	2·6 (0·7–5)	-43·1% (-60·2 to -10·4%)
Environmental/occupational risk factors§	Meningitis	1·6% (0·8–2·8%)	3·9 (1·8–7)	-80·3% (-91·4 to -52·2%)	1% (0·5–1·9%)	<0·1 (<0·1–0·1)	-80·3% (-91·4 to -52·2%)
	Encephalitis	0·3% (0·1–0·7%)	0·6 (0·2–1·5)	-25·9% (-70·4 to 85·9%)	0·1% (0–0·4%)	<0·1 (<0·1–<0·1)	-25·9% (-70·4 to 85·9%)
	Stroke	53·8% (50·1–57·6%)	1883·8 (1350·2–2440·1)	-28·1% (-45·5 to -8·6%)	50·3% (46·4–54·6%)	81·2 (56·2–104·8)	-26·5% (-43·8 to -8·3%)
	Ischaemic stroke	51·5% (47·4–55·6%)	961·2 (701·7–1279·2)	18·7% (-9·6 to 51·7%)	48·1% (43·8–52·6%)	47·2 (32·8–62·7)	12·9% (-13·4 to 46·3%)
	Intracerebral haemorrhage	56·7% (52·9–60·6%)	792·1 (552·7–1058·9)	-49·3% (-62·3 to -32·2%)	53·8% (50–58·2%)	29·1 (20–39)	-51% (-63·5 to -35·7%)
	Subarachnoid haemorrhage	55·6% (51·4–60·1%)	130·5 (41·7–225·6)	-46·9% (-63·1 to -17%)	53% (48·4–57·9%)	5 (1·5–9)	-47·1% (-62·2 to -17·3%)
High body-mass index	Stroke	29·5% (19·9–39·1%)	1033 (612·1–1572·6)	7·2% (-22·8 to 59·5%)	20·6% (13·4–28·1%)	33·3 (18·7–51·1)	12·9% (-19·6 to 69·6%)
	Ischaemic stroke	21·3% (13·6–29·2%)	396·2 (228·5–604·8)	117·7% (54·2 to 231·9%)	14·4% (8·8–20·6%)	14·1 (7·6–22·4)	119·7% (52·7 to 246·6%)
	Intracerebral haemorrhage	39·2% (26·6–51·8%)	547·9 (329·8–836·8)	-18·8% (-43·6 to 26·3%)	30·6% (20·1–41·8%)	16·5 (9·5–25·5)	-17·2% (-43·1 to 29·1%)
	Subarachnoid haemorrhage	38·4% (25–52%)	88·9 (30·4–167)	-16·9% (-45·9 to 39·7%)	29·2% (17·7–42%)	2·7 (0·9–5·1)	-14·4% (-43·9 to 46·9%)
	Neurological disorders*	3·3% (0·9–7·9%)	49·9 (12·6–130·2)	67·8% (26 to 184·2%)	7·5% (2·5–15·5%)	3·3 (0·5–10·4)	69·3% (21·9 to 212·5%)
	Alzheimer's disease and other dementias	11·5% (4·5–21·7%)	49·9 (12·6–130·2)	67·8% (26 to 184·2%)	10·8% (4·1–20·7%)	3·3 (0·5–10·4)	69·3% (21·9 to 212·5%)
High fasting plasma glucose	Stroke	32·8% (22·2–47·9%)	1146·7 (695·3–1821·3)	46·5% (1·9 to 107%)	33·3% (20·7–54·2%)	53·8 (30·1–91·9)	41·7% (0·9 to 100·2%)
	Ischaemic stroke	34·6% (18·3–61·4%)	645·4 (332·7–1187·7)	118·4% (56·6 to 211·1%)	33·9% (17–67·9%)	33·2 (15·5–67·4)	103·2% (45·4 to 189·4%)

Table S16 Regional and national disabilities and deaths related to neurological conditions attributable to risk factors in North Africa and Middle East countries

Location<br>Risk factor (alphabetic order)	Cause	DALYs (95% UI)<br>PAF (%)<br>2019	Rate per 100,000<br>2019	Rate change (%)<br>From 1990 to 2019	Deaths (95% UI)<br>PAF (%)<br>2019	Rate per 100,000<br>2019	Rate change (%)<br>From 1990 to 2019
	Intracerebral haemorrhage	30·9% (18·8–44·3%)	430·9 (233·2–680·3)	2·4% (-28·5 to 50%)	32·6% (20·3–47·1%)	17·6 (9·8–28·3)	-5·7% (-33·2 to 34·6%)
	Subarachnoid haemorrhage	29·8% (17·9–43·7%)	70·4 (21–143·1)	6·6% (-30·4 to 81·1%)	31·9% (19·7–48·7%)	3 (0·8–6·3)	0·8% (-32·6 to 71·1%)
	Neurological disorders*	4·1% (0·7–11·4%)	62·4 (10·9–199·4)	62·9% (42·7 to 91·9%)	10% (2·1–22·4%)	4·4 (0·5–15·6)	62·9% (39·2 to 95·8%)
	Alzheimer's disease and other dementias	14·4% (3·6–29·7%)	62·4 (10·9–199·4)	62·9% (42·7 to 91·9%)	14·3% (3·6–29·5%)	4·4 (0·5–15·6)	62·9% (39·2 to 95·8%)
High LDL cholesterol	Stroke	13·2% (8·3–20·4%)	461·2 (265·1–763·5)	41·3% (8·1 to 83·8%)	12·2% (5·6–23·8%)	19·8 (7·8–40·4)	34·3% (4·5 to 75·6%)
	Ischaemic stroke	24·8% (15·5–38·5%)	461·2 (265·1–763·5)	41·3% (8·1 to 83·8%)	20·2% (9·1–39%)	19·8 (7·8–40·4)	34·3% (4·5 to 75·6%)
High systolic blood pressure	Stroke	50·2% (41·7–58·3%)	1759·3 (1176·5–2416·6)	-21·2% (-42·2 to 3·7%)	47·1% (38·6–55·1%)	76·2 (49·3–106·5)	-19·2% (-40·8 to 5·3%)
	Ischaemic stroke	46·7% (36·2–56·3%)	872·5 (577–1229·5)	32·6% (-2 to 71·2%)	43·5% (32·8–54·8%)	42·7 (27–61·9)	28·1% (-5·7 to 68·6%)
	Intracerebral haemorrhage	54·5% (43·3–63·8%)	761·9 (490·6–1065)	-44·1% (-60·3 to -23·8%)	52·8% (41·9–63·5%)	28·6 (17·6–40·5)	-45·7% (-60 to -27·5%)
	Subarachnoid haemorrhage	53·2% (42·2–63·7%)	124·9 (42·1–222·3)	-41·1% (-60·5 to -6·2%)	52% (40·4–63·4%)	4·9 (1·4–9)	-41% (-58·7 to -5·7%)
High temperature	Stroke	0·6% (-0·1–1·6%)	20·5 (-3·1–54·7)	-25·3% (-193·2 to 101·4%)	0·6% (-0·1–1·7%)	1 (-0·2–2·8)	-20·9% (-197·5 to 113%)
	Ischaemic stroke	0·6% (-0·1–1·5%)	10·6 (-1·6–28·8)	25·2% (-256·7 to 247·1%)	0·6% (-0·1–1·7%)	0·6 (-0·1–1·7)	20·3% (-249·2 to 217·9%)
	Intracerebral haemorrhage	0·6% (-0·1–1·6%)	8·5 (-1·2–23)	-48% (-165·8 to 39·6%)	0·6% (-0·1–1·7%)	0·3 (0–0·9)	-48·8% (-164 to 38·9%)
	Subarachnoid haemorrhage	0·6% (-0·1–1·6%)	1·4 (-0·2–4·3)	-46·4% (-167·4 to 50·1%)	0·6% (-0·1–1·7%)	0·1 (0–0·2)	-44·5% (-170·8 to 54·8%)
Household air pollution from solid fuels	Meningitis	1·3% (0·6–2·3%)	3·1 (1·4–5·7)	-83·3% (-92·9 to -59·4%)	0·8% (0·4–1·6%)	<0·1 (<0·1–0·1)	-83·3% (-92·9 to -59·4%)
	Encephalitis	0·2% (0·1–0·5%)	0·4 (0·2–1·2)	-37·3% (-74·6 to 54·3%)	0·1% (0–0·3%)	<0·1 (<0·1–<0·1)	-37·3% (-74·6 to 54·3%)
	Stroke	32% (24–39·4%)	1120·1 (755·8–1574·4)	-44·1% (-57·9 to -26·8%)	28·4% (21–35·6%)	45·9 (29·8–65·2)	-44·5% (-58·1 to -28·4%)
	Ischaemic stroke	30·3% (22·5–37·5%)	565·5 (387·8–817·9)	-8·4% (-30·4 to 18·8%)	26·8% (19·7–34%)	26·3 (17·3–38·8)	-15·4% (-35·8 to 11·6%)
	Intracerebral haemorrhage	34·1% (25·8–41·5%)	476·3 (315·8–683·1)	-60·3% (-71·3 to -46·5%)	31% (23·1–38·4%)	16·8 (10·8–24·4)	-62·4% (-72·2 to -50·1%)
	Subarachnoid haemorrhage	33·6% (25·4–41·8%)	78·3 (26–140·1)	-58·4% (-71·3 to -33·7%)	30·6% (22·6–38·7%)	2·8 (0·9–5·4)	-59·7% (-71·3 to -34·6%)
Kidney dysfunction	Stroke	9·9% (8·1–11·6%)	345·8 (241·5–470·4)	7·6% (-17·3 to 35·5%)	9·6% (7·1–12%)	15·5 (9·7–22·1)	12% (-15·3 to 42·2%)
	Ischaemic stroke	11·6% (8·9–14·3%)	216·4 (143·6–305·5)	69% (30·9 to 113·7%)	10·2% (6·5–13·8%)	10 (5·6–15·1)	66·4% (26 to 113·5%)
	Intracerebral haemorrhage	9·3% (7·6–11·2%)	129·4 (85·2–182·3)	-33·1% (-50·3 to -12·4%)	10·1% (7·9–12·2%)	5·5 (3·4–7·8)	-30·1% (-47·3 to -10·5%)
Lead exposure	Stroke	12·4% (9·4–15·4%)	434·9 (284·8–613·6)	-16·9% (-37·4 to 7%)	12% (9–14·9%)	19·3 (12·1–28)	-8·5% (-30·2 to 15·9%)
	Ischaemic stroke	11·6% (8·4–14·8%)	216·3 (137·8–313·6)	48·3% (14 to 90·9%)	11% (7·9–14·6%)	10·8 (6·5–16·5)	53·4% (18·2 to 97·5%)
	Intracerebral haemorrhage	13·5% (9·7–17·5%)	188 (116·7–272·6)	-42·6% (-57·3 to -23·8%)	13·4% (9·5–17·7%)	7·3 (4·3–10·7)	-40·4% (-54·8 to -22·6%)
	Subarachnoid haemorrhage	12·9% (9·1–17·2%)	30·5 (11·1–58·1)	-39·1% (-57·1 to -5·9%)	13% (9–17·8%)	1·2 (0·4–2·4)	-34·6% (-53·1 to 3%)
Low birth weight	Meningitis	5·7% (2·9–10%)	13·8 (6·8–24·7)	-75·6% (-89·2 to -44·2%)	3·7% (1·8–6·9%)	0·2 (0·1–0·3)	-75·6% (-89·2 to -44·2%)
	Encephalitis	1·1% (0·5–2·3%)	2 (0·8–5)	-9·5% (-63·1 to 118·9%)	0·5% (0·2–1·3%)	<0·1 (<0·1–0·1)	-9·5% (-63·1 to 118·8%)
Low birth weight and short gestation	Meningitis	5·9% (3–10·4%)	14·3 (7·1–25·7)	-75·4% (-89 to -43·5%)	3·8% (1·8–7·1%)	0·2 (0·1–0·3)	-75·4% (-89 to -43·5%)
	Encephalitis	1·1% (0·5–2·3%)	2·1 (0·8–5·2)	-9·4% (-62·7 to 119·3%)	0·5% (0·2–1·3%)	<0·1 (<0·1–0·1)	-9·4% (-62·7 to 119·3%)
Low physical activity	Stroke	3·7% (0·8–8·9%)	129·9 (25·1–306·9)	31·8% (2·6 to 64·1%)	5% (1·1–11%)	8·1 (1·7–18)	27·2% (-0·5 to 60·4%)

Table S16 Regional and national disabilities and deaths related to neurological conditions attributable to risk factors in North Africa and Middle East countries

**Location**<br>Risk factor (alphabetic order)	Cause	**DALYs (95% UI)**<br>PAF (%)<br>2019	Rate per 100,000<br>2019	Rate change (%)<br>From 1990 to 2019	**Deaths (95% UI)**<br>PAF (%)<br>2019	Rate per 100,000<br>2019	Rate change (%)<br>From 1990 to 2019
	Ischaemic stroke	7% (1·5–16·6%)	129·9 (25·1–306·9)	31·8% (2·6 to 64·1%)	8·3% (1·8–17·7%)	8·1 (1·7–18)	27·2% (-0·5 to 60·4%)
Low temperature	Stroke	8·9% (6·2–12·1%)	310 (185·3–451)	-22·6% (-41·2 to -1·5%)	9·5% (6·7–13·2%)	15·4 (9·1–22·6)	-18% (-36·1 to 1·3%)
	Ischaemic stroke	8·6% (6·1–11·7%)	159·8 (97–234·7)	29·6% (-1·9 to 67·2%)	9·5% (6·7–13·2%)	9·4 (5·7–13·8)	24·5% (-2·7 to 57%)
	Intracerebral haemorrhage	9·2% (6·4–12·6%)	128·7 (74·2–191·7)	-46·1% (-59·9 to -28·4%)	9·5% (6·7–13·2%)	5·2 (2·9–7·7)	-47% (-59·7 to -31·7%)
	Subarachnoid haemorrhage	9·1% (6·3–12·4%)	21·5 (6·5–40·5)	-44·2% (-59·9 to -14·4%)	9·5% (6·7–13·2%)	0·9 (0·3–1·8)	-42·2% (-58·3 to -11·2%)
Metabolic risk factors†	Stroke	72·9% (65·5–79·7%)	2551·6 (1806·9–3399·4)	-12·2% (-32·7 to 12·5%)	70% (61·5–78·8%)	113·2 (76·4–150·1)	-9·5% (-30·2 to 13·8%)
	Ischaemic stroke	73·9% (64·9–84·1%)	1378 (992·7–1835·5)	41·5% (9·4 to 78·5%)	69·7% (57·9–84·1%)	68·3 (45·9–94·7)	37·4% (6·3 to 74·2%)
	Intracerebral haemorrhage	72·4% (64–80%)	1012·9 (691·2–1384·7)	-39·7% (-55·2 to -19·4%)	71·4% (62·8–79·7%)	38·6 (25·9–53·1)	-41·1% (-56 to -23·1%)
	Subarachnoid haemorrhage	68·4% (58·7–76·8%)	160·7 (52·7–279·9)	-36·2% (-56·2 to -0·4%)	67·3% (57–76·8%)	6·3 (1·8–11·6)	-35·9% (-54·9 to 1·5%)
	Neurological disorders*	7% (2·1–15·7%)	105 (28·6–292·2)	60·3% (33·5 to 114·4%)	16·4% (5·7–30·5%)	7·3 (1·3–23·3)	61% (29·8 to 122·5%)
	Alzheimer's disease and other dementias	24·2% (10·5–41%)	105 (28·6–292·2)	60·3% (33·5 to 114·4%)	23·5% (10–40·2%)	7·3 (1·3–23·3)	61% (29·8 to 122·5%)
Non-optimal temperature	Stroke	9·4% (6·9–12·6%)	328·6 (204·9–469·1)	-22·8% (-41·4 to -1·6%)	10·1% (7·5–13·6%)	16·3 (10–23·6)	-18·2% (-36·2 to 1·2%)
	Ischaemic stroke	9·1% (6·7–12·1%)	169·5 (107·1–243·3)	29·3% (-1·8 to 65·8%)	10·1% (7·5–13·6%)	9·9 (6·2–14·2)	24·3% (-3 to 56·4%)
	Intracerebral haemorrhage	9·8% (7·2–13·1%)	136·4 (83·5–198·2)	-46·2% (-59·8 to -28·2%)	10·1% (7·5–13·6%)	5·5 (3·3–8)	-47·1% (-59·8 to -32%)
	Subarachnoid haemorrhage	9·6% (7–12·8%)	22·8 (6·9–42·4)	-44·3% (-60·1 to -15%)	10·1% (7·5–13·6%)	1 (0·3–1·8)	-42·3% (-58·4 to -11·5%)
Other environmental risks	Stroke	12·4% (9·4–15·4%)	434·9 (284·8–613·6)	-16·9% (-37·4 to 7%)	12% (9–14·9%)	19·3 (12·1–28)	-8·5% (-30·2 to 15·9%)
	Ischaemic stroke	11·6% (8·4–14·8%)	216·3 (137·8–313·6)	48·3% (14 to 90·9%)	11% (7·9–14·6%)	10·8 (6·5–16·5)	53·4% (18·2 to 97·5%)
	Intracerebral haemorrhage	13·5% (9·7–17·5%)	188 (116·7–272·6)	-42·6% (-57·3 to -23·8%)	13·4% (9·5–17·7%)	7·3 (4·3–10·7)	-40·4% (-54·8 to -22·6%)
	Subarachnoid haemorrhage	12·9% (9·1–17·2%)	30·5 (11·1–58·1)	-39·1% (-57·1 to -5·9%)	13% (9–17·8%)	1·2 (0·4–2·4)	-34·6% (-53·1 to 3%)
Particulate matter pollution	Meningitis	1·6% (0·8–2·8%)	3·9 (1·8–7)	-80·3% (-91·4 to -52·2%)	1% (0·5–1·9%)	<0·1 (<0·1–0·1)	-80·3% (-91·4 to -52·2%)
	Encephalitis	0·3% (0·1–0·7%)	0·6 (0·2–1·5)	-25·9% (-70·4 to 85·9%)	0·1% (0–0·4%)	<0·1 (<0·1–<0·1)	-25·9% (-70·4 to 85·9%)
	Stroke	42% (38·1–46%)	1470·1 (1038·2–1910·8)	-32·2% (-49 to -12·5%)	37·3% (33·3–41·7%)	60·3 (41–79·3)	-32·7% (-49·3 to -13·8%)
	Ischaemic stroke	39·8% (35·8–44·1%)	742·3 (545·1–990·3)	11% (-15·9 to 44·3%)	35·2% (31·3–40%)	34·5 (24–47·2)	2·7% (-23·3 to 36·4%)
	Intracerebral haemorrhage	44·7% (40·6–48·7%)	625·2 (435·8–839·5)	-51·8% (-64·5 to -35·1%)	40·7% (36·7–44·9%)	22 (14·8–29·6)	-54·4% (-66·3 to -39·4%)
	Subarachnoid haemorrhage	43·9% (39·4–48·8%)	102·7 (33·5–177·9)	-49·5% (-65·4 to -19·4%)	40% (35·2–45·1%)	3·7 (1·1–6·8)	-51% (-65·6 to -22%)
Secondhand smoke	Stroke	4·4% (3·3–5·6%)	154·5 (101–218·8)	-18·4% (-39·4 to 6·2%)	3·7% (2·8–4·7%)	6 (3·9–8·5)	-14·5% (-34·5 to 7·6%)
	Ischaemic stroke	3·9% (2·9–5%)	73 (48·6–104·1)	45·8% (8·5 to 91·5%)	3·3% (2·5–4·2%)	3·2 (2·1–4·7)	40·2% (5·9 to 83·8%)
	Intracerebral haemorrhage	5% (3·7–6·4%)	70·1 (43·8–104·8)	-41·8% (-58·1 to -21·6%)	4·3% (3·2–5·5%)	2·3 (1·4–3·4)	-42·1% (-57·5 to -23·5%)
	Subarachnoid haemorrhage	4·9% (3·5–6·5%)	11·5 (3·7–20·5)	-39·6% (-59·6 to -4%)	4·2% (3–5·7%)	0·4 (0·1–0·7)	-38·5% (-57·9 to -1·3%)
Short gestation	Meningitis	4·1% (2·1–7·3%)	10 (4·9–17·9)	-74·4% (-88·5 to -41·4%)	2·7% (1·3–4·9%)	0·1 (0·1–0·2)	-74·4% (-88·5 to -41·4%)
	Encephalitis	0·8% (0·4–1·6%)	1·5 (0·6–3·6)	-6·9% (-61·6 to 122·5%)	0·4% (0·1–0·9%)	<0·1 (<0·1–<0·1)	-6·9% (-61·6 to 122·5%)
Smoking	Stroke	7·7% (6·6–9%)	269·9 (190·2–369·4)	26·6% (-8·6 to 76·4%)	5·8% (4·9–6·9%)	9·3 (6·4–12·9)	17·7% (-13·7 to 59·4%)

Table S16 Regional and national disabilities and deaths related to neurological conditions attributable to risk factors in North Africa and Middle East countries

		DALYs (95% UI)			Deaths (95% UI)		
**Location**<br>Risk factor (alphabetic order)	Cause	PAF (%)<br>2019	Rate per 100,000<br>2019	Rate change (%)<br>From 1990 to 2019	PAF (%)<br>2019	Rate per 100,000<br>2019	Rate change (%)<br>From 1990 to 2019
	Ischaemic stroke	6·8% (5·6–8·1%)	126·2 (91·2–181)	102·9% (47·6 to 180·3%)	4·9% (4–5·9%)	4·8 (3·4–7)	81·2% (30·5 to 151·7%)
	Intracerebral haemorrhage	8·9% (7·5–10·7%)	124·7 (86·6–170·8)	-5·3% (-33·8 to 36·5%)	7·3% (6–8·9%)	3·9 (2·7–5·4)	-14·8% (-39 to 20·6%)
	Subarachnoid haemorrhage	7·9% (4·9–10·6%)	19 (6·6–38·2)	-1·7% (-34·4 to 73·9%)	6·4% (3·6–8·8%)	0·6 (0·2–1·3)	-10·8% (-37·9 to 58·6%)
	Neurological disorders*	2·4% (0·8–5·3%)	36·6 (10·2–96·7)	22·5% (-7·1 to 57·8%)	5·1% (1·5–8·9%)	2·3 (0·3–7·6)	22·6% (-10·5 to 66·5%)
	Alzheimer's disease and other dementias	9·2% (5·6–13·3%)	39·9 (13·7–101·3)	19·9% (-8·2 to 51·3%)	8·4% (5–12·3%)	2·6 (0·5–7·8)	21·2% (-9·2 to 57%)
	Parkinson's disease	-4·4% (-6·9–-2·3%)	-5 (-8–-2·6)	14·4% (-18·8 to 57·9%)	-4·3% (-6·7–-2·2%)	-0·3 (-0·5–-0·1)	13·7% (-19·6 to 56·4%)
	Multiple sclerosis	6·2% (3·9–9·2%)	1·7 (0·9–2·8)	81·4% (26·4 to 153·6%)	6·5% (3·6–10·3%)	<0·1 (<0·1–0·1)	60·9% (6·4 to 147·9%)
Tobacco	Stroke	11·8% (10·3–13·4%)	411·9 (293·6–559·7)	4·7% (-22·3 to 39·4%)	9·2% (8–10·7%)	14·9 (10·4–19·9)	2% (-22·9 to 33·4%)
	Ischaemic stroke	10·4% (8·9–11·8%)	193·8 (141·6–269)	76·2% (31·5 to 133·2%)	8% (6·8–9·3%)	7·9 (5·6–10·9)	61·2% (20·4 to 112·3%)
	Intracerebral haemorrhage	13·5% (11·7–15·6%)	188·6 (130·8–258·9)	-23·4% (-45·1 to 5·6%)	11·2% (9·6–13·1%)	6·1 (4·1–8·3)	-28·1% (-47·7 to -2·4%)
	Subarachnoid haemorrhage	12·5% (9·4–15·1%)	29·5 (10·3–55·6)	-21·1% (-47·2 to 28·7%)	10·3% (7·5–12·9%)	1 (0·3–1·9)	-24·7% (-47·3 to 23·4%)
	Neurological disorders*	2·4% (0·8–5·3%)	36·6 (10·2–96·7)	22·5% (-7·1 to 57·8%)	5·1% (1·5–8·9%)	2·3 (0·3–7·6)	22·6% (-10·5 to 66·5%)
	Alzheimer's disease and other dementias	9·2% (5·6–13·3%)	39·9 (13·7–101·3)	19·9% (-8·2 to 51·3%)	8·4% (5–12·3%)	2·6 (0·5–7·8)	21·2% (-9·2 to 57%)
	Parkinson's disease	-4·4% (-6·9–-2·3%)	-5 (-8–-2·6)	14·4% (-18·8 to 57·9%)	-4·3% (-6·7–-2·2%)	-0·3 (-0·5–-0·1)	13·7% (-19·6 to 56·4%)
	Multiple sclerosis	6·2% (3·9–9·2%)	1·7 (0·9–2·8)	81·4% (26·4 to 153·6%)	6·5% (3·6–10·3%)	<0·1 (<0·1–0·1)	60·9% (6·4 to 147·9%)
**Sudan**							
Air pollution	Meningitis	1·2% (0·6–2·2%)	1·2 (0·5–2·2)	-90·2% (-95·6 to -79·7%)	0·7% (0·3–1·4%)	<0·1 (<0·1–<0·1)	-90·2% (-95·6 to -79·7%)
	Encephalitis	0·5% (0·2–1·1%)	0·2 (0·1–0·3)	-39·4% (-77 to 76·3%)	0·3% (0·1–0·7%)	<0·1 (<0·1–<0·1)	-39·4% (-77·1 to 76·4%)
	Stroke	36·3% (31·1–40·6%)	938·9 (668–1337·9)	-44% (-57·4 to -25·9%)	32·1% (27·3–36·1%)	40·2 (28·3–59·3)	-45·2% (-59·6 to -26·4%)
	Ischaemic stroke	35·2% (30·2–39·4%)	596·3 (430·8–861·6)	-9·3% (-32·4 to 20·7%)	30·8% (26·1–34·6%)	27·8 (19·7–40·7)	-17·8% (-40·4 to 11·2%)
	Intracerebral haemorrhage	38·5% (33·2–42·9%)	296·8 (196–425·8)	-66·2% (-75·6 to -52·7%)	35·5% (30·3–39·8%)	10·8 (7·1–15·2)	-68·5% (-77·4 to -55·8%)
	Subarachnoid haemorrhage	37·3% (32–42·2%)	45·8 (25·3–77·9)	-67·1% (-79·3 to -37·3%)	35·2% (29·9–39·8%)	1·6 (0·8–2·8)	-69·7% (-80·7 to -38·3%)
Alcohol use	Stroke	0% (-0·1–0%)	-0·5 (-1·8–0·6)	-101·5% (-109 to -98·2%)	0% (-0·1–0%)	0 (-0·1–0)	-102·2% (-112·8 to -98·4%)
	Ischaemic stroke	0% (-0·1–0%)	-0·8 (-2·1–0·2)	-127·6% (-513·9 to 186·7%)	0% (-0·1–0%)	0 (-0·1–0)	-134·8% (-564·5 to 308·4%)
	Intracerebral haemorrhage	0% (0–0·1%)	0·3 (-0·2–1·1)	-99% (-100·8 to -97%)	0% (0–0·1%)	<0·1 (<0·1–<0·1)	-99·1% (-100·8 to -97·1%)
	Neurological disorders*	0% (0–0%)	0·2 (0–0·4)	-96·3% (-99 to -91·1%)	0% (0–0%)	<0·1 (<0·1–<0·1)	-96·8% (-99 to -93%)
	Idiopathic epilepsy	0·1% (0–0·2%)	0·2 (0–0·4)	-96·3% (-99 to -91·1%)	0·1% (0–0·2%)	<0·1 (<0·1–<0·1)	-96·8% (-99 to -93%)
All risk factors	Meningitis	5·3% (3–8·9%)	5·3 (2·8–9·7)	-86·8% (-93·3 to -76·2%)	3·2% (1·7–5·9%)	0·1 (0–0·1)	-86·8% (-93·3 to -76·2%)
	Encephalitis	2·2% (1·1–4·1%)	0·7 (0·3–1·1)	-22·8% (-67·6 to 98%)	1·5% (0·7–2·9%)	<0·1 (<0·1–<0·1)	-22·8% (-67·6 to 98%)
	Stroke	89·3% (86·2–92·2%)	2309·3 (1736·6–3195·8)	-29·3% (-43·2 to -11·8%)	88·8% (84·4–93·3%)	111·2 (81·4–157·5)	-27·2% (-40·6 to -8·8%)
	Ischaemic stroke	90·4% (86·6–94·3%)	1530·3 (1174·1–2133)	13·1% (-9 to 40·6%)	88·8% (83·4–94·9%)	80·2 (58·9–114·9)	8% (-12·5 to 34·6%)

Table S16 Regional and national disabilities and deaths related to neurological conditions attributable to risk factors in North Africa and Middle East countries

Location<br>Risk factor (alphabetic order)	Cause	DALYs (95% UI)<br>PAF (%)<br>2019	Rate per 100,000<br>2019	Rate change (%)<br>From 1990 to 2019	Deaths (95% UI)<br>PAF (%)<br>2019	Rate per 100,000<br>2019	Rate change (%)<br>From 1990 to 2019
	Intracerebral haemorrhage	87·8% (83·9–91%)	676·5 (461–951)	-59·1% (-69·5 to -44·9%)	89·2% (84·6–92·9%)	27·2 (18·4–37·7)	-60·3% (-69·4 to -47·1%)
	Subarachnoid haemorrhage	83·3% (76·9–87·8%)	102·4 (58·6–172·8)	-60·4% (-74·3 to -25·9%)	86·7% (81·4–91·1%)	3·9 (2·1–6·6)	-61·8% (-74·5 to -26·2%)
	Neurological disorders*	9·8% (3·7–20·4%)	131 (45·2–336·8)	33·8% (11·8 to 63·7%)	23·8% (10–39·3%)	8·2 (1·5–26·4)	39·9% (15·6 to 81·2%)
	Alzheimer's disease and other dementias	37·4% (24·3–52·1%)	137·2 (51·6–344·1)	35·1% (13·8 to 62%)	35·8% (22·8–50·5%)	8·6 (1·8–26·8)	37·4% (13·4 to 71·6%)
	Parkinson's disease	-8·8% (-13·2–-4·8%)	-7·5 (-12·2–-4)	-15% (-41·1 to 31·7%)	-8·1% (-12·5–-4·4%)	-0·4 (-0·7–-0·2)	-10·1% (-38·8 to 43·2%)
	Idiopathic epilepsy	0·1% (0–0·2%)	0·2 (0–0·4)	-96·3% (-99 to -91·1%)	0·1% (0–0·2%)	<0·1 (<0·1–<0·1)	-96·8% (-99 to -93%)
	Multiple sclerosis	8·6% (5·6–12%)	1·2 (0·7–1·9)	5·8% (-31·7 to 50·8%)	9·8% (6–14·1%)	<0·1 (<0·1–<0·1)	5·6% (-44·3 to 72·1%)
Ambient particulate matter pollution	Meningitis	0·6% (0·3–1·3%)	0·6 (0·2–1·3)	-41% (-78·6 to 95·8%)	0·4% (0·2–0·8%)	<0·1 (<0·1–<0·1)	-41% (-78·6 to 95·8%)
	Encephalitis	0·3% (0·1–0·6%)	0·1 (0–0·2)	260·8% (10·3 to 1547·2%)	0·2% (0·1–0·4%)	<0·1 (<0·1–<0·1)	260·8% (10·2 to 1547·4%)
	Stroke	21·6% (13·2–29·5%)	558·6 (317·3–894·1)	206·9% (59 to 596·3%)	19·1% (11·5–26·3%)	24 (13·4–38·7)	203% (60·9 to 572·6%)
	Ischaemic stroke	20·9% (12·6–28·8%)	354·9 (206–573)	399·8% (163·3 to 1031·7%)	18·4% (11·1–25·4%)	16·6 (9·3–27·5)	358·2% (147·2 to 899·2%)
	Intracerebral haemorrhage	22·9% (14–31·4%)	176·6 (96·2–274·8)	84·1% (-8·7 to 346·8%)	21·2% (13·1–29%)	6·4 (3·5–10·2)	72·8% (-13·1 to 306·7%)
	Subarachnoid haemorrhage	22% (13·6–30·7%)	27·1 (12·9–51)	80·2% (-15·7 to 447·9%)	20·9% (12·6–29·1%)	0·9 (0·4–1·8)	66·1% (-20·5 to 405·5%)
Behavioural risk factors‡	Meningitis	5% (2·8–8·3%)	5 (2·6–9·1)	-86·4% (-93 to -75%)	3% (1·6–5·5%)	0·1 (0–0·1)	-86·4% (-93 to -75%)
	Encephalitis	2% (1–3·9%)	0·6 (0·3–1)	-20·4% (-66·6 to 104·2%)	1·4% (0·7–2·7%)	<0·1 (<0·1–<0·1)	-20·4% (-66·6 to 104·2%)
	Stroke	38·2% (32·3–44·3%)	989·8 (705·8–1424·5)	-32·6% (-47·6 to -13·6%)	35·2% (28·9–41·5%)	44·1 (30·4–64·8)	-30·1% (-44·5 to -12%)
	Ischaemic stroke	40·7% (32·8–48·3%)	688·7 (477·7–1021)	8·8% (-13·2 to 38·2%)	37·1% (29·1–44·8%)	33·5 (22·3–50·4)	3·2% (-17·2 to 29·3%)
	Intracerebral haemorrhage	33·8% (27·2–40·6%)	261·2 (169·2–388·2)	-63·9% (-74 to -49·8%)	30·5% (24·1–37·5%)	9·3 (6–14)	-65·2% (-74·3 to -52·9%)
	Subarachnoid haemorrhage	32·3% (25·2–39·6%)	39·9 (21·1–75·2)	-64·1% (-77·1 to -31·6%)	30% (22·8–37·3%)	1·3 (0·6–2·6)	-65·9% (-77·6 to -31·9%)
	Neurological disorders*	3·6% (1·2–8%)	48·2 (14·4–122·5)	-8·6% (-30·7 to 13·5%)	7·8% (2–14%)	2·8 (0·3–8·8)	-4·2% (-30 to 30·6%)
	Alzheimer's disease and other dementias	14·8% (8·9–21·3%)	54·4 (20·4–131·1)	-3·5% (-23·1 to 20·4%)	13·2% (7·6–19·1%)	3·2 (0·6–9·1)	-3·6% (-26·7 to 24·9%)
	Parkinson's disease	-8·8% (-13·2–-4·8%)	-7·5 (-12·2–-4)	-15% (-41·1 to 31·7%)	-8·1% (-12·5–-4·4%)	-0·4 (-0·7–-0·2)	-10·1% (-38·8 to 43·2%)
	Idiopathic epilepsy	0·1% (0–0·2%)	0·2 (0–0·4)	-96·3% (-99 to -91·1%)	0·1% (0–0·2%)	<0·1 (<0·1–<0·1)	-96·8% (-99 to -93%)
	Multiple sclerosis	8·6% (5·6–12%)	1·2 (0·7–1·9)	5·8% (-31·7 to 50·8%)	9·8% (6–14·1%)	<0·1 (<0·1–<0·1)	5·6% (-44·3 to 72·1%)
Child and maternal malnutrition	Meningitis	5% (2·8–8·3%)	5 (2·6–9·1)	-86·4% (-93 to -75%)	3% (1·6–5·5%)	0·1 (0–0·1)	-86·4% (-93 to -75%)
	Encephalitis	2% (1–3·9%)	0·6 (0·3–1)	-20·4% (-66·6 to 104·2%)	1·4% (0·7–2·7%)	<0·1 (<0·1–<0·1)	-20·4% (-66·6 to 104·2%)
Diet high in red meat	Stroke	3·3% (1·1–5·2%)	85·3 (29–150·8)	-31·8% (-58·1 to 15·6%)	2·5% (0·9–4%)	3·2 (1·1–5·8)	-32·2% (-58 to 10·5%)
	Ischaemic stroke	2·9% (0·4–4·9%)	49·7 (6·8–94·3)	22·1% (-8·4 to 68·4%)	2·2% (0·4–3·6%)	2 (0·3–3·8)	13·9% (-13·8 to 57·8%)
	Intracerebral haemorrhage	4% (0·6–6·7%)	30·8 (4·3–61·7)	-57·7% (-71·1 to -36·7%)	3·4% (0·5–5·7%)	1 (0·1–2·1)	-59·3% (-71·1 to -39·8%)
	Subarachnoid haemorrhage	4% (0·6–6·8%)	4·8 (0·6–11·1)	-58·9% (-74·4 to -20·2%)	3·4% (0·5–5·8%)	0·2 (0–0·4)	-61·4% (-75·8 to -20·8%)
Diet high in sodium	Stroke	2% (0·3–7·9%)	51·6 (6·1–210·2)	-32·7% (-62 to 30·1%)	1·7% (0·3–7%)	2·2 (0·3–9·1)	-31·4% (-59·5 to 32·7%)

Table S16 Regional and national disabilities and deaths related to neurological conditions attributable to risk factors in North Africa and Middle East countries

Location<br>Risk factor (alphabetic order)	Cause	DALYs (95% UI)<br>PAF (%)<br>2019	Rate per 100,000<br>2019	Rate change (%)<br>From 1990 to 2019	Deaths (95% UI)<br>PAF (%)<br>2019	Rate per 100,000<br>2019	Rate change (%)<br>From 1990 to 2019
	Ischaemic stroke	1·9% (0·3–7·5%)	31·4 (4·1–130·4)	14·7% (-38·7 to 114·8%)	1·6% (0·3–6·3%)	1·4 (0·2–6·1)	10·4% (-42 to 103·7%)
	Intracerebral haemorrhage	2·3% (0·3–9·1%)	17·6 (1·8–73·6)	-58·8% (-77·5 to -22·2%)	2·1% (0·3–8·5%)	0·6 (0·1–2·7)	-60·3% (-77·9 to -21·9%)
	Subarachnoid haemorrhage	2·1% (0·2–8·5%)	2·6 (0·2–11·4)	-60% (-80·4 to -4·2%)	2·1% (0·3–8·3%)	0·1 (0–0·4)	-61·7% (-79·9 to -6·3%)
Diet low in fibre	Stroke	4% (0·8–7·4%)	104·6 (20·7–206)	-46·2% (-62·7 to -28·3%)	3·4% (0·8–6·3%)	4·3 (0·9–8·6)	-45·5% (-61·5 to -27·5%)
	Ischaemic stroke	3·8% (0·8–7·1%)	65·2 (12·9–128·7)	-8·1% (-34·6 to 24·7%)	3·2% (0·7–5·9%)	2·9 (0·6–5·8)	-13·3% (-39·7 to 16·3%)
	Intracerebral haemorrhage	4·4% (0·9–8%)	34 (6·6–67·3)	-68% (-78·5 to -54·6%)	4% (0·8–7·3%)	1·2 (0·2–2·4)	-69·1% (-78·4 to -56·8%)
	Subarachnoid haemorrhage	4·4% (0·9–8·2%)	5·4 (1·1–12·7)	-68·6% (-81·1 to -39·7%)	4% (0·8–7·5%)	0·2 (0–0·4)	-70·3% (-81·7 to -40·4%)
Diet low in fruits	Stroke	5·9% (2·8–9·6%)	153·1 (65·8–282·2)	-45·2% (-64·5 to -19%)	4·8% (2·2–7·8%)	6 (2·5–11·2)	-45·1% (-64·2 to -20·5%)
	Ischaemic stroke	4·7% (1·1–9%)	80·3 (17·8–175·8)	4·2% (-24·1 to 37·5%)	3·8% (0·9–7·3%)	3·5 (0·8–7·6)	-2·6% (-31·3 to 27·8%)
	Intracerebral haemorrhage	8·2% (3·8–15%)	62·9 (27·7–124·7)	-63·9% (-74·7 to -48·3%)	7·3% (3·4–13·5%)	2·2 (1–4·4)	-65·3% (-74·9 to -50·8%)
	Subarachnoid haemorrhage	8·1% (3·8–14·8%)	9·9 (3·7–21·7)	-64·8% (-78·2 to -33·6%)	7·3% (3·4–13·5%)	0·3 (0·1–0·8)	-66·7% (-78·9 to -34·8%)
Diet low in vegetables	Stroke	3·4% (1·1–5·6%)	86·7 (28·7–162·7)	-50·4% (-69·1 to -17·3%)	2·8% (1–4·7%)	3·6 (1·2–6·6)	-49·5% (-68·6 to -19%)
	Ischaemic stroke	2·7% (0·4–5%)	45·7 (6–90·2)	-0·7% (-30·9 to 33·7%)	2·3% (0·3–4·2%)	2·1 (0·3–4·1)	-5·9% (-32·8 to 24·1%)
	Intracerebral haemorrhage	4·6% (0·4–9·6%)	35·5 (2·9–77·4)	-68% (-81·9 to -54%)	4·3% (0·4–8·9%)	1·3 (0·1–2·8)	-68·8% (-81·1 to -55·8%)
	Subarachnoid haemorrhage	4·5% (0·4–9·3%)	5·5 (0·4–13·3)	-69·1% (-83·4 to -41%)	4·3% (0·4–8·7%)	0·2 (0–0·5)	-70·3% (-83·4 to -41·6%)
Diet low in whole grains	Stroke	6% (3·2–7·9%)	154·9 (78·1–238·1)	11·6% (-11·4 to 41%)	5·9% (3·4–7·6%)	7·4 (3·9–11·6)	5·4% (-16·1 to 32%)
	Ischaemic stroke	9·1% (4·9–11·9%)	154·9 (78·1–238·1)	11·6% (-11·4 to 41%)	8·1% (4·7–10·4%)	7·4 (3·9–11·6)	5·4% (-16·1 to 32%)
Dietary risks	Stroke	21·9% (16·3–27·8%)	567·2 (380·9–891·4)	-35·4% (-50·8 to -15·9%)	19·3% (14·4–24·6%)	24·3 (15·9–38·4)	-34·3% (-48·7 to -15·8%)
	Ischaemic stroke	22·4% (16–28·8%)	379·4 (241·1–592·9)	6·9% (-15·3 to 36·4%)	19·4% (14–24·9%)	17·5 (10·8–27·5)	0·8% (-19·6 to 27·5%)
	Intracerebral haemorrhage	21·1% (13·8–28·6%)	162·4 (92·2–262·9)	-64% (-73·8 to -50·1%)	19·3% (12·5–26·5%)	5·9 (3·3–9·7)	-65·3% (-74 to -52·7%)
	Subarachnoid haemorrhage	20·6% (13·4–28·2%)	25·4 (12·6–49·2)	-64·9% (-77·6 to -33·9%)	19·2% (12·4–26·6%)	0·9 (0·4–1·7)	-66·8% (-78·2 to -34·4%)
Environmental/occupational risk factors§	Meningitis	1·2% (0·6–2·2%)	1·2 (0·5–2·2)	-90·2% (-95·6 to -79·7%)	0·7% (0·3–1·4%)	<0·1 (<0·1–<0·1)	-90·2% (-95·6 to -79·7%)
	Encephalitis	0·5% (0·2–1·1%)	0·2 (0·1–0·3)	-39·4% (-77 to 76·3%)	0·3% (0·1–0·7%)	<0·1 (<0·1–<0·1)	-39·4% (-77·1 to 76·4%)
	Stroke	45·4% (39·9–49·7%)	1174·4 (841·1–1661·1)	-40·8% (-54 to -23·4%)	42·3% (37–46·8%)	53·1 (37·3–76·2)	-39·9% (-53·3 to -22·1%)
	Ischaemic stroke	44·3% (38·8–48·6%)	750·8 (551·7–1085·2)	-3·4% (-25·9 to 26·4%)	41% (35·5–45·5%)	37·1 (26·3–53·6)	-9·1% (-32·3 to 19·8%)
	Intracerebral haemorrhage	47·6% (42·1–52·4%)	367·1 (242·3–520·5)	-64·8% (-74·1 to -51·6%)	45·9% (40·2–50·7%)	14 (9·2–19·7)	-66·1% (-74·7 to -53·5%)
	Subarachnoid haemorrhage	45·9% (40·3–51%)	56·5 (31·5–97·6)	-66% (-78·2 to -36·2%)	45·3% (39·8–50·7%)	2 (1·1–3·5)	-67·4% (-78·8 to -35·9%)
High body-mass index	Stroke	30·4% (20·5–40·6%)	788·1 (470·6–1219·2)	7·6% (-24·6 to 73·4%)	20·3% (12·7–28·7%)	25·4 (14·3–40·4)	11·6% (-20·9 to 75·9%)
	Ischaemic stroke	24·1% (15·5–33·6%)	407·8 (234·4–645·8)	124·9% (64·4 to 259·2%)	15·8% (9·2–23·4%)	14·3 (7·6–24·5)	123·8% (60·3 to 255·8%)
	Intracerebral haemorrhage	42·5% (29·9–54·4%)	328·3 (190·2–509·5)	-30·5% (-54·2 to 19·7%)	32·1% (21–43·5%)	9·8 (5·4–15·6)	-31·1% (-53·2 to 17·5%)
	Subarachnoid haemorrhage	42·5% (29·5–55%)	51·9 (27·1–92·1)	-34·1% (-61 to 32·1%)	32·1% (21–44·7%)	1·4 (0·7–2·7)	-36·5% (-62 to 33%)
	Neurological disorders*	4·1% (1·1–9·4%)	54·3 (14·6–141·7)	123·6% (61·7 to 300·4%)	10% (3·4–19·8%)	3·4 (0·5–10·8)	131·1% (56·6 to 374·5%)

Table S16 Regional and national disabilities and deaths related to neurological conditions attributable to risk factors in North Africa and Middle East countries

Location<br>Risk factor (alphabetic order)	Cause	DALYs (95% UI)<br>PAF (%)<br>2019	 Rate per 100,000<br>2019	 Rate change (%)<br>From 1990 to 2019	Deaths (95% UI)<br>PAF (%)<br>2019	 Rate per 100,000<br>2019	 Rate change (%)<br>From 1990 to 2019
	Alzheimer's disease and other dementias	14·8% (6·1–26·7%)	54·3 (14·6–141·7)	123·6% (61·7 to 300·4%)	14·1% (5·7–25·5%)	3·4 (0·5–10·8)	131·1% (56·6 to 374·5%)
High fasting plasma glucose	Stroke	30·6% (19·3–48·6%)	789·9 (460·5–1335·5)	46·2% (3·8 to 110·5%)	32·3% (18–59%)	40·3 (21·4–74·9)	39·1% (-3·2 to 108·5%)
	Ischaemic stroke	32·8% (16·5–59·5%)	554·3 (271·4–1092·5)	115·5% (53·4 to 218·4%)	33·1% (15·8–68·6%)	29·8 (13·1–64·2)	92·6% (35·5 to 201·7%)
	Intracerebral haemorrhage	26·8% (16·2–38·8%)	206·2 (111·7–332·6)	-16·1% (-41·7 to 21·2%)	30·3% (18·2–47·2%)	9·2 (4·8–15·5)	-21·6% (-45·3 to 16·6%)
	Subarachnoid haemorrhage	23·9% (14·3–35·1%)	29·5 (13·3–54·8)	-21·4% (-50·9 to 56%)	29·1% (17·7–45·9%)	1·3 (0·5–2·6)	-25·7% (-53·6 to 56%)
	Neurological disorders*	3·7% (0·6–10·6%)	49·9 (9–154·8)	66·7% (50·4 to 97·2%)	9·7% (2–22·1%)	3·3 (0·4–12·2)	62·8% (42·4 to 101·4%)
	Alzheimer's disease and other dementias	13·6% (3·4–28·3%)	49·9 (9–154·8)	66·7% (50·4 to 97·2%)	13·7% (3·4–28·4%)	3·3 (0·4–12·2)	62·8% (42·4 to 101·4%)
High LDL cholesterol	Stroke	13·6% (8·2–22·3%)	351·2 (191·4–615·1)	28·8% (1·7 to 68·7%)	12·4% (5–25·5%)	15·6 (5·7–34·2)	20·2% (-4 to 58·5%)
	Ischaemic stroke	20·7% (12·2–34·5%)	351·2 (191·4–615·1)	28·8% (1·7 to 68·7%)	17·2% (6·8–34·9%)	15·6 (5·7–34·2)	20·2% (-4 to 58·5%)
High systolic blood pressure	Stroke	61·6% (54–68·4%)	1592·1 (1151·5–2261·1)	-26% (-41·5 to -4·4%)	58·6% (49·6–67·9%)	73·4 (50·8–105·5)	-23·4% (-39·3 to -1·1%)
	Ischaemic stroke	59·8% (49·2–68·9%)	1012·8 (706·9–1466·3)	24·5% (-0·8 to 57·4%)	56·1% (43·7–67·9%)	50·7 (33·7–76·8)	19·8% (-5·7 to 53·3%)
	Intracerebral haemorrhage	65·3% (55·8–73·3%)	503·1 (334·5–713·6)	-56·5% (-67·4 to -40·4%)	65·4% (54·6–75·6%)	19·9 (13·1–28·7)	-57·2% (-67·2 to -42·2%)
	Subarachnoid haemorrhage	62·1% (51·3–71%)	76·2 (43–130·2)	-57·9% (-72·9 to -20·8%)	64% (53·2–73·6%)	2·9 (1·5–4·9)	-59% (-72·8 to -20·5%)
High temperature	Stroke	3·9% (1·5–7·5%)	101 (35·3–219·9)	-25·1% (-74·3 to 11%)	4·4% (1·6–8·3%)	5·5 (1·9–12)	-17·6% (-69·7 to 23·1%)
	Ischaemic stroke	3·8% (1·4–7·3%)	64·1 (22·4–139·7)	23·8% (-57·7 to 81·3%)	4·4% (1·6–8·3%)	3·9 (1·4–8·5)	22·2% (-55·3 to 79%)
	Intracerebral haemorrhage	4·1% (1·6–8%)	31·9 (11·4–69·3)	-54·9% (-85 to -28·6%)	4·4% (1·6–8·3%)	1·3 (0·5–2·8)	-55% (-83·9 to -29·8%)
	Subarachnoid haemorrhage	4% (1·5–7·5%)	5 (1·5–11·8)	-59·2% (-85·8 to -15·2%)	4·4% (1·6–8·3%)	0·2 (0·1–0·5)	-56·9% (-83·8 to -6·9%)
Household air pollution from solid fuels	Meningitis	0·5% (0·2–1·1%)	0·5 (0·2–1·1)	-95·1% (-98·1 to -89%)	0·3% (0·1–0·7%)	<0·1 (<0·1–<0·1)	-95·1% (-98·1 to -89%)
	Encephalitis	0·2% (0·1–0·5%)	0·1 (0–0·1)	-69·4% (-89·9 to -7·4%)	0·2% (0·1–0·4%)	<0·1 (<0·1–<0·1)	-69·4% (-89·9 to -7·3%)
	Stroke	14·7% (9·1–21·2%)	380·3 (213·6–621·6)	-74·5% (-84·5 to -61·4%)	13% (8–19%)	16·2 (8·9–26·7)	-75·2% (-84·5 to -62%)
	Ischaemic stroke	14·3% (8·8–20·7%)	241·4 (135·5–392·8)	-58·8% (-74·6 to -38·5%)	12·4% (7·7–18·3%)	11·2 (6·2–18·8)	-62·8% (-76·7 to -44%)
	Intracerebral haemorrhage	15·6% (9·6–22·5%)	120·3 (63·8–200·5)	-84·6% (-91 to -75·4%)	14·3% (8·9–20·8%)	4·4 (2·4–7·3)	-85·7% (-91·5 to -77·6%)
	Subarachnoid haemorrhage	15·3% (9·4–22·3%)	18·6 (8·9–35·8)	-85% (-91·9 to -69%)	14·3% (8·8–21%)	0·6 (0·3–1·2)	-86·3% (-92·5 to -70·1%)
Kidney dysfunction	Stroke	10·1% (8–12·2%)	260·1 (182·5–369·4)	1·6% (-18·7 to 26·9%)	9·8% (6·7–12·8%)	12·2 (7·5–18·6)	3·6% (-19·7 to 32·8%)
	Ischaemic stroke	11·4% (8·4–14·2%)	192·1 (125·5–285·4)	53·5% (22·9 to 91·5%)	10·1% (6·1–14%)	9·1 (4·9–15)	48·6% (14·5 to 88·7%)
	Intracerebral haemorrhage	8·9% (7·2–10·7%)	68·1 (45·3–97)	-48·1% (-60·5 to -29·5%)	10·2% (7·8–12·6%)	3·1 (2–4·5)	-45·4% (-58·1 to -25·9%)
Lead exposure	Stroke	8·9% (6·4–11·5%)	230·7 (144·7–348·1)	-38·6% (-52·1 to -21·3%)	9% (6·4–11·8%)	11·3 (7·1–17·4)	-29·9% (-45·4 to -9·9%)
	Ischaemic stroke	8·8% (6·1–11·7%)	148·4 (90·6–227·8)	10·5% (-13·1 to 39·3%)	8·6% (5·9–11·9%)	7·8 (4·6–12·6)	16·1% (-9 to 46·1%)
	Intracerebral haemorrhage	9·3% (6·1–12·6%)	71·8 (41·9–112·1)	-65·7% (-75·2 to -53·2%)	10% (6·8–13·8%)	3·1 (1·8–4·9)	-62·6% (-71·6 to -49·3%)
	Subarachnoid haemorrhage	8·5% (5·4–11·9%)	10·6 (4·6–19·9)	-67·3% (-78·7 to -39·5%)	9·7% (6·4–13·3%)	0·4 (0·2–0·8)	-64·1% (-75·9 to -30·1%)
Low birth weight	Meningitis	4·6% (2·6–7·7%)	4·6 (2·4–8·4)	-86·7% (-93·2 to -75·9%)	2·8% (1·5–5·1%)	0·1 (0–0·1)	-86·7% (-93·2 to -75·9%)

Table S16 Regional and national disabilities and deaths related to neurological conditions attributable to risk factors in North Africa and Middle East countries

**Location**<br>Risk factor (alphabetic order)	Cause	**DALYs (95% UI)**<br>PAF (%)<br>2019	Rate per 100,000<br>2019	Rate change (%)<br>From 1990 to 2019	**Deaths (95% UI)**<br>PAF (%)<br>2019	Rate per 100,000<br>2019	Rate change (%)<br>From 1990 to 2019
	Encephalitis	1·9% (0·9–3·6%)	0·6 (0·3–1)	-21·7% (-67·4 to 102·5%)	1·3% (0·6–2·5%)	<0·1 (<0·1–<0·1)	-21·7% (-67·4 to 102·5%)
Low birth weight and short gestation	Meningitis	5% (2·8–8·3%)	5 (2·6–9·1)	-86·4% (-93 to -75%)	3% (1·6–5·5%)	0·1 (0–0·1)	-86·4% (-93 to -75%)
	Encephalitis	2% (1–3·9%)	0·6 (0·3–1)	-20·4% (-66·6 to 104·2%)	1·4% (0·7–2·7%)	<0·1 (<0·1–<0·1)	-20·4% (-66·6 to 104·2%)
Low physical activity	Stroke	7·9% (2–14·6%)	203·9 (52·2–391·6)	16·4% (-5·1 to 43·8%)	9·4% (2·7–16·5%)	11·7 (3·4–21·3)	9% (-11·5 to 35·3%)
	Ischaemic stroke	12·1% (3·1–22·5%)	203·9 (52·2–391·6)	16·4% (-5·1 to 43·8%)	13% (3·7–22·8%)	11·7 (3·4–21·3)	9% (-11·5 to 35·3%)
Low temperature	Stroke	2·3% (-1·2–5·5%)	59·2 (-32·7–151·3)	-34·1% (-118·1 to 30·3%)	2·6% (-1·3–6·1%)	3·2 (-1·7–8·1)	-27·4% (-120·4 to 41·1%)
	Ischaemic stroke	2·2% (-1·1–5·3%)	37·5 (-19·9–94·6)	9·4% (-132·3 to 121·6%)	2·6% (-1·3–6·1%)	2·3 (-1·2–5·9)	8% (-132·6 to 114·5%)
	Intracerebral haemorrhage	2·4% (-1·3–5·8%)	18·8 (-10·1–49)	-60·3% (-109·8 to -18·9%)	2·6% (-1·3–6·1%)	0·8 (-0·4–2)	-60·4% (-109·7 to -18·9%)
	Subarachnoid haemorrhage	2·4% (-1·2–5·6%)	2·9 (-1·3–8·2)	-64·4% (-108·9 to -9·4%)	2·6% (-1·3–6·1%)	0·1 (-0·1–0·3)	-62·4% (-109·7 to -7·8%)
Metabolic risk factors†	Stroke	78·3% (71·8–84·3%)	2024·1 (1510·3–2822·9)	-21·3% (-37·1 to -0·5%)	76·1% (67·7–85·2%)	95·3 (68–137·2)	-18·8% (-34·1 to 2·6%)
	Ischaemic stroke	78·8% (70·6–87·6%)	1333·9 (987·6–1858·1)	26·6% (2·1 to 59·6%)	75·4% (64·3–88·5%)	68 (47·8–101·8)	21·7% (-2·4 to 55·8%)
	Intracerebral haemorrhage	78% (70·6–83·6%)	600·9 (408·3–849·5)	-54·4% (-65·9 to -37·6%)	78·6% (70·2–85·4%)	23·9 (16·3–33·5)	-55·4% (-65·9 to -39·5%)
	Subarachnoid haemorrhage	72·6% (63·4–80·1%)	89·2 (51·2–150·1)	-55·6% (-71·1 to -17%)	74·9% (65·6–83·4%)	3·3 (1·7–5·8)	-57% (-71·5 to -16·5%)
	Neurological disorders*	7·3% (2·3–16·4%)	96·8 (29·8–247·7)	84·9% (50·9 to 163·6%)	18·4% (7·1–32·8%)	6·2 (1·1–19·9)	84·3% (46·3 to 172·4%)
	Alzheimer's disease and other dementias	26·4% (12·3–43·3%)	96·8 (29·8–247·7)	84·9% (50·9 to 163·6%)	25·9% (11·7–43·1%)	6·2 (1·1–19·9)	84·3% (46·3 to 172·4%)
Non-optimal temperature	Stroke	6·1% (2·1–9·9%)	157·8 (54·9–295·8)	-28·8% (-66·2 to 9·2%)	6·8% (2·3–11%)	8·6 (3–15·9)	-21·6% (-61·4 to 21%)
	Ischaemic stroke	5·9% (2–9·6%)	100·1 (31·6–189·8)	17·9% (-42·6 to 85·7%)	6·8% (2·3–11%)	6·2 (2–11·5)	16·4% (-42·1 to 86·3%)
	Intracerebral haemorrhage	6·5% (2·2–10·5%)	49·9 (16·7–91·2)	-57·1% (-80·3 to -29·9%)	6·8% (2·3–11%)	2·1 (0·7–3·9)	-57·2% (-78·8 to -29·6%)
	Subarachnoid haemorrhage	6·2% (2·2–10·2%)	7·8 (2·1–16·8)	-61·4% (-82·8 to -15·5%)	6·8% (2·3–11%)	0·3 (0·1–0·7)	-59·2% (-81 to -6·2%)
Other environmental risks	Stroke	8·9% (6·4–11·5%)	230·7 (144·7–348·1)	-38·6% (-52·1 to -21·3%)	9% (6·4–11·8%)	11·3 (7·1–17·4)	-29·9% (-45·4 to -9·9%)
	Ischaemic stroke	8·8% (6·1–11·7%)	148·4 (90·6–227·8)	10·5% (-13·1 to 39·3%)	8·6% (5·9–11·9%)	7·8 (4·6–12·6)	16·1% (-9 to 46·1%)
	Intracerebral haemorrhage	9·3% (6·1–12·6%)	71·8 (41·9–112·1)	-65·7% (-75·2 to -53·2%)	10% (6·8–13·8%)	3·1 (1·8–4·9)	-62·6% (-71·6 to -49·3%)
	Subarachnoid haemorrhage	8·5% (5·4–11·9%)	10·6 (4·6–19·9)	-67·3% (-78·7 to -39·5%)	9·7% (6·4–13·3%)	0·4 (0·2–0·8)	-64·1% (-75·9 to -30·1%)
Particulate matter pollution	Meningitis	1·2% (0·6–2·2%)	1·2 (0·5–2·2)	-90·2% (-95·6 to -79·7%)	0·7% (0·3–1·4%)	<0·1 (<0·1–<0·1)	-90·2% (-95·6 to -79·7%)
	Encephalitis	0·5% (0·2–1·1%)	0·2 (0·1–0·3)	-39·4% (-77 to 76·3%)	0·3% (0·1–0·7%)	<0·1 (<0·1–<0·1)	-39·4% (-77·1 to 76·4%)
	Stroke	36·3% (31·1–40·6%)	938·9 (668–1337·9)	-44% (-57·4 to -25·9%)	32·1% (27·3–36·1%)	40·2 (28·3–59·3)	-45·2% (-59·6 to -26·4%)
	Ischaemic stroke	35·2% (30·2–39·4%)	596·3 (430·8–861·6)	-9·3% (-32·4 to 20·7%)	30·8% (26·1–34·6%)	27·8 (19·7–40·7)	-17·8% (-40·4 to 11·2%)
	Intracerebral haemorrhage	38·5% (33·2–42·9%)	296·8 (196–425·8)	-66·2% (-75·6 to -52·7%)	35·5% (30·3–39·8%)	10·8 (7·1–15·2)	-68·5% (-77·4 to -55·8%)
	Subarachnoid haemorrhage	37·3% (32–42·2%)	45·8 (25·3–77·9)	-67·1% (-79·3 to -37·3%)	35·2% (29·9–39·8%)	1·6 (0·8–2·8)	-69·7% (-80·7 to -38·3%)
Secondhand smoke	Stroke	3·4% (2·6–4·3%)	89·4 (59·9–131·4)	-41·1% (-55·2 to -22·1%)	2·9% (2·2–3·6%)	3·6 (2·4–5·3)	-39·8% (-52·6 to -21·8%)
	Ischaemic stroke	3·1% (2·4–3·9%)	53·3 (35·3–79·6)	2·7% (-19·9 to 34·6%)	2·7% (2–3·4%)	2·4 (1·6–3·6)	-4·8% (-24·5 to 21·6%)
	Intracerebral haemorrhage	4% (3·1–5%)	31·2 (19·4–48)	-63·7% (-74·4 to -49·3%)	3·5% (2·6–4·3%)	1·1 (0·7–1·6)	-64·9% (-74·4 to -51·6%)

Table S16 Regional and national disabilities and deaths related to neurological conditions attributable to risk factors in North Africa and Middle East countries

**Location**<br>Risk factor (alphabetic order)	Cause	**DALYs (95% UI)**<br>PAF (%)<br>2019	Rate per 100,000<br>2019	Rate change (%)<br>From 1990 to 2019	**Deaths (95% UI)**<br>PAF (%)<br>2019	Rate per 100,000<br>2019	Rate change (%)<br>From 1990 to 2019
	Subarachnoid haemorrhage	4% (3–5·1%)	4·9 (2·6–8·6)	-65·1% (-78·4 to -33·7%)	3·5% (2·6–4·5%)	0·2 (0·1–0·3)	-66·6% (-78·9 to -34·1%)
Short gestation	Meningitis	2·8% (1·6–4·6%)	2·8 (1·5–5·1)	-83·9% (-91·6 to -71·2%)	1·7% (0·9–3·1%)	<0·1 (<0·1–0·1)	-83·9% (-91·6 to -71·2%)
	Encephalitis	1·2% (0·6–2·2%)	0·3 (0·2–0·6)	-6·6% (-61·9 to 137·7%)	0·8% (0·4–1·5%)	<0·1 (<0·1–<0·1)	-6·6% (-61·9 to 137·7%)
Smoking	Stroke	11·8% (9·7–14·1%)	306·3 (210·2–445·3)	-37·8% (-54·6 to -15·8%)	9% (7·3–10·9%)	11·4 (7·7–16·8)	-37·4% (-54·1 to -15·7%)
	Ischaemic stroke	11% (9–13·2%)	186·6 (127·8–291·9)	8·5% (-21·3 to 47·3%)	8·1% (6·5–10%)	7·4 (5–11·7)	3·9% (-26·1 to 44·4%)
	Intracerebral haemorrhage	13·5% (10·6–16·8%)	104·4 (65·4–158·9)	-62·6% (-74·4 to -44·6%)	11·4% (8·7–14·3%)	3·5 (2·1–5·3)	-63·7% (-74·7 to -47·1%)
	Subarachnoid haemorrhage	12·1% (8·4–16%)	15·2 (6·9–30·2)	-63·5% (-77·6 to -22·5%)	10·8% (7–14·6%)	0·5 (0·2–1)	-65·1% (-78·4 to -23·1%)
	Neurological disorders*	3·6% (1·2–8%)	48 (14·2–122·2)	-1·2% (-21·5 to 25·8%)	7·8% (1·9–14%)	2·8 (0·3–8·8)	-2·5% (-28·7 to 38·7%)
	Alzheimer's disease and other dementias	14·8% (8·9–21·3%)	54·4 (20·4–131·1)	-3·5% (-23·1 to 20·4%)	13·2% (7·6–19·1%)	3·2 (0·6–9·1)	-3·6% (-26·7 to 24·9%)
	Parkinson's disease	-8·8% (-13·2–-4·8%)	-7·5 (-12·2–-4)	-15% (-41·1 to 31·7%)	-8·1% (-12·5–-4·4%)	-0·4 (-0·7–-0·2)	-10·1% (-38·8 to 43·2%)
	Multiple sclerosis	8·6% (5·6–12%)	1·2 (0·7–1·9)	5·8% (-31·7 to 50·8%)	9·8% (6–14·1%)	<0·1 (<0·1–<0·1)	5·6% (-44·3 to 72·1%)
Tobacco	Stroke	14·8% (12·5–17·2%)	383·9 (269·7–549·8)	-38·3% (-54 to -18%)	11·6% (9·7–13·5%)	14·6 (9·9–21·6)	-37·7% (-53·1 to -18%)
	Ischaemic stroke	13·7% (11·6–16·1%)	233·3 (163–351·8)	7·4% (-19·8 to 44·3%)	10·5% (8·8–12·4%)	9·5 (6·6–14·6)	1·8% (-24·4 to 38%)
	Intracerebral haemorrhage	17% (14–20·2%)	131·2 (83·3–194·5)	-62·7% (-74·1 to -45·6%)	14·5% (11·7–17·3%)	4·4 (2·8–6·5)	-63·9% (-74·4 to -48·6%)
	Subarachnoid haemorrhage	15·6% (11·9–19·3%)	19·5 (9·5–36·9)	-63·8% (-77·5 to -27·7%)	13·9% (10·2–17·6%)	0·6 (0·3–1·2)	-65·4% (-78·2 to -27·6%)
	Neurological disorders*	3·6% (1·2–8%)	48 (14·2–122·2)	-1·2% (-21·5 to 25·8%)	7·8% (1·9–14%)	2·8 (0·3–8·8)	-2·5% (-28·7 to 38·7%)
	Alzheimer's disease and other dementias	14·8% (8·9–21·3%)	54·4 (20·4–131·1)	-3·5% (-23·1 to 20·4%)	13·2% (7·6–19·1%)	3·2 (0·6–9·1)	-3·6% (-26·7 to 24·9%)
	Parkinson's disease	-8·8% (-13·2–-4·8%)	-7·5 (-12·2–-4)	-15% (-41·1 to 31·7%)	-8·1% (-12·5–-4·4%)	-0·4 (-0·7–-0·2)	-10·1% (-38·8 to 43·2%)
	Multiple sclerosis	8·6% (5·6–12%)	1·2 (0·7–1·9)	5·8% (-31·7 to 50·8%)	9·8% (6–14·1%)	<0·1 (<0·1–<0·1)	5·6% (-44·3 to 72·1%)

Data in parentheses are 95% uncertainty intervals. Rate data per 100,000 and percentage data are rounded to one decimal place. Percentages of DALYs are not mutually exclusive: the sum of percentages of DALYs in the columns exceeds the totals for all risk factors combined because of overlap between various risk factors. The crude sum of population attributable fraction (PAF) of the risk factors might exceed 100% because the effects of many of these risk factors are mediated partly or fully through another risk factor or risk factors. DALY=disability-adjusted life-year. $PM_{2·5}$=particulate matter with a diameter of <25 µm. *Neurological disorders in the Global Burden of Disease Study 2019 include Alzheimer's disease and other dementias, Parkinson's disease, idiopathic epilepsy, multiple sclerosis, headache disorders (including migraine and tension-type headache), motor neuron disease, and other neurological disorders. Metabolic risk factors† cluster includes high body-mass index, high fasting plasma glucose, high LDL cholesterol, high systolic blood pressure, and kidney dysfunction. ‡Behavioural risk factors cluster includes tobacco (including smoking and second-hand smoking), dietary risks (diet high in sodium, diet low in fruits, diet low in vegetables, diet high in red meat, and diet low in whole grains), low physical activity, alcohol consumption, and child and maternal malnutrition (including low birth weight and short gestation). §Environmental risk factors cluster includes air pollution (including ambient $PM_{2·5}$ pollution and household air pollution from solid fuels), non-optimal temperature (including low ambient temperature and high ambient temperature), and other environmental risks (including lead exposure). Risk factors with insufficient data are not reported here.

Table S17 Age-specific disability and death related to neurological conditions (NCs) in North Africa and Middle East countries in 2019
(NEXT PAGE)

Table S17 Age-specific disability and death related to neurological conditions (NCs)* in North Africa and Middle East countries in 2019

		Under 5<br>Mean (95% UI)	5-14 years<br>Mean (95% UI)	15-49 years<br>Mean (95% UI)	50-69 years<br>Mean (95% UI)	70+ years<br>Mean (95% UI)
**DALYs (Disability-Adjusted Life Years)**						
Global	Number	16605633 (12900218–21704624)	11182406 (6838747–17433172)	70848156 (41725263–112830549)	82296089 (69409585–98883050)	92131170 (74791237–121617720)
	Percent (all-cause)†	3·5% (2·9–4·4%)	9·5% (5·7–14·5%)	9% (5·2–13·9%)	12·2% (10·1–14·6%)	19% (15·4–25·2%)
	Percent (proportion)‡	6·1%	4·1%	25·9%	30·1%	33·7%
North Africa and Middle East	Number	797643 (542482–1151776)	1060594 (559933–1869084)	6228226 (3408366–10432270)	4423367 (3517269–5550477)	4059720 (3240436–5508356)
	Percent (all-cause)†	2·8% (2–3·9%)	11·2% (5·9–18·6%)	9·9% (5·5–15·7%)	10·9% (8·9–13·4%)	17·9% (14·5–24·3%)
	Percent (proportion)‡	4·8%	6·4%	37·6%	26·7%	24·5%
Algeria	Number	27101 (15223–44949)	55654 (24729–107594)	378902 (193276–663212)	285076 (205640–384834)	340712 (254537–485493)
	Percent (all-cause)†	1·6% (0·9–2·6%)	10·7% (4·9–19%)	10% (5·2–16·7%)	11·6% (8·8–15·4%)	19·9% (15·5–27·8%)
	Percent (proportion)‡	2·5%	5·1%	34·8%	26·2%	31·3%
Bahrain	Number	340 (199–534)	1206 (465–2308)	13511 (6647–23530)	7028 (4797–10236)	3377 (2464–5139)
	Percent (all-cause)†	3·5% (2·1–5·4%)	12·2% (5·2–21%)	9·5% (4·8–15·4%)	7·3% (5·2–10·3%)	11·7% (9–17·6%)
	Percent (proportion)‡	1·3%	4·7%	53·1%	27·6%	13·3%
Egypt	Number	199086 (87725–360822)	246543 (106580–452385)	1042873 (533696–1790894)	790771 (546779–1154184)	481318 (335229–728958)
	Percent (all-cause)†	6·2% (2·9–11·1%)	14·7% (6·4–25·7%)	11·5% (6–18·7%)	9·2% (6·9–12·6%)	12·8% (9·4–18·9%)
	Percent (proportion)‡	7·2%	8·9%	37·8%	28·6%	17·4%
Iran	Number	38235 (23464–55577)	104897 (53514–179251)	804469 (402305–1392492)	558351 (430278–709880)	654516 (501331–914052)
	Percent (all-cause)†	2·5% (1·6–3·5%)	11·2% (5·7–18%)	9·4% (4·7–15·4%)	10·5% (8–13·5%)	18·5% (14·2–25·8%)
	Percent (proportion)‡	1·8%	4·9%	37·2%	25·8%	30·3%
Iraq	Number	50570 (28435–83370)	88491 (44666–157494)	481145 (260209–822400)	362735 (263602–476746)	278210 (218221–363373)
	Percent (all-cause)†	3·4% (2·1–5·2%)	12·1% (6·1–20·3%)	10·7% (6–17%)	15·1% (12·5–18·3%)	23·7% (19·7–30·3%)
	Percent (proportion)‡	4·0%	7·0%	38·2%	28·8%	22·1%
Jordan	Number	6975 (4105–11479)	18721 (7972–35650)	90389 (41565–165624)	53791 (39599–72764)	51367 (39003–70616)
[truncated: 543,570 more chars]
